# Evaluating large language models for pharmacotherapy simulations: a mixed-methods study

**DOI:** 10.1038/s41746-026-02626-1

**Published:** 2026-05-05

**Authors:** Ahmed N. Farrag, Amany El-Zeiny, Amani M. Ali

**Affiliations:** 1 Department of Clinical Pharmacy, Faculty of Pharmacy, Cairo University, Cairo, Egypt; 2 National Cancer Institute, Cairo University, Cairo, Egypt

**Keywords:** Cancer, Computational biology and bioinformatics, Medical research

## Abstract

Simulation-based learning is essential in clinical pharmacy education but requires substantial faculty resources that limit scalability. Large language models (LLMs) offer promise for generating scalable simulations, yet their pedagogical rigor and clinical reliability remain unclear. In a mixed-methods, counterbalanced evaluation study, PharmD students (*n* = 104) engaged with acute myeloid leukemia (AML) or chronic myeloid leukemia (CML) cases, conditions requiring complex longitudinal management yet sharing semantic similarity, generated by four LLMs using expert-guided meta-prompts. Expert panels evaluated sessions across clinical authenticity, instructional design, and clinical reasoning; students completed satisfaction surveys. Of 103 sessions, 53 (51.5%) met passing criteria across all domains. Clinical accuracy and safety emerged as the limiting domain (58.3%) compared to clinical reasoning (81.6%) and instructional design (82.5%). CML sessions outperformed AML sessions (62.3% vs 40.0%; *p* = 0.031). Platform success rates ranged from 34.5% to 62.1%. Error analysis revealed guideline misalignment, pharmacotherapeutic inaccuracies, fabricated evidence, and cross-condition therapeutic recommendations occurring exclusively in AML sessions. Students favored LLMs over traditional methods (49.8% vs 30.0%); however, we did not detect statistically significant alignment between student satisfaction and expert-assessed quality. Sessions more frequently met criteria for instructional design and clinical reasoning than for pharmacotherapeutic accuracy and guideline alignment. Expert oversight with platform-specific and disease-specific validation remains essential for safe educational deployment, and effectiveness trials assessing objective learning outcomes represent necessary subsequent work.

## Introduction

Simulation-based learning plays an important role in clinical pharmacy education because it allows students to practice clinical reasoning and therapeutic decision-making in safe, controlled settings^1–3^. Well-designed simulations provide structured scenarios and timely feedback that support cognitive development and progressive skill acquisition^4–6^. However, traditional simulation methods are difficult to scale, requiring substantial faculty time, specialized expertise, and institutional resources that limit broad curricular integration^7,8^. Large language models (LLMs) offer a promising solution by generating sophisticated, interactive clinical simulations at scale^9–11^.

Despite their potential, LLMs’ clinical accuracy and educational validity remain uncertain in specialized therapeutic areas^11,12^. Most existing evaluations focus on short, discrete knowledge questions rather than extended clinical scenarios that require sustained reasoning, longitudinal decision-making, and contextual adaptation^13–15^. This gap is critical because LLMs rely on statistical learning processes that can introduce systematic errors, which may go undetected without rigorous domain-specific testing^16^.

One notable error pattern arises from how LLMs process semantically related information. Because these models learn statistical associations from training data, conditions sharing clinical features or terminology may be inappropriately conflated in generated content^17,18^. Recent work has shown that word embeddings can misattribute symptoms between distinct diseases when conditions share semantic similarities, with errors stemming from tangential associations rather than direct clinical relevance^19,20^. In medical contexts, such conflation can inadvertently merge management strategies for conditions requiring fundamentally different therapeutic approaches, a pattern we refer to as domain entanglement^19^. These errors are particularly concerning in educational settings, as they often appear coherent and authoritative, potentially reinforcing inaccurate or unsafe knowledge among learners who lack sufficient clinical experience to recognize the inaccuracies^21,22^.

To systematically assess these vulnerabilities, rigorous evaluation requires a strategic selection of test domains that combine authentic educational applications with conditions likely to expose systematic errors^23,24^. Hematologic malignancies offer this combination, involving complex, evidence-based treatment algorithms, frequent protocol updates, and challenging clinical decisions that students must master before practice^25–28^. Within this domain, acute and chronic myeloid leukemias provide a strategically designed stress test. These conditions share myeloid cell lineage and present with overlapping clinical and laboratory features, creating semantic similarity that may challenge LLMs’ ability to maintain appropriate therapeutic boundaries. However, their management approaches differ fundamentally: AML requires time-sensitive intensive chemotherapy with consolidation decisions guided by molecular features and remission status^29^, while CML requires chronic oral tyrosine kinase inhibitor therapy with ongoing molecular monitoring and specific criteria for adjusting treatment^30^.

This pairing enables direct evaluation of whether semantic overlap causes domain entanglement. If models inappropriately recommend CML-specific tyrosine kinase inhibitors for AML patients or apply AML induction regimens to CML cases, it would demonstrate a safety-critical failure mode. Conversely, successful boundary preservation despite semantic similarity would suggest that structured prompting can mitigate this vulnerability. Additionally, the complexity gradient between these conditions (CML following relatively linear therapeutic pathways while AML requires multi-variable conditional reasoning) enables assessment of whether therapeutic complexity independently affects LLM performance, with implications for predicting performance in other therapeutic areas.

In this study, we evaluated how well LLMs generate pharmacotherapy simulations requiring accurate reasoning, safe therapeutic recommendations, and sound instructional design. Our primary aims were to (1) characterize LLM performance across instructional design quality, clinical accuracy and safety, and clinical reasoning fidelity, and (2) compare performance between AML and CML to test whether semantic similarity challenges boundary preservation while assessing whether therapeutic complexity independently affects accuracy. Our secondary aims were to (1) compare performance across four major platforms to distinguish general model capabilities from platform-specific characteristics, and (2) examine whether student satisfaction aligns with expert-rated quality to inform oversight requirements for safe educational deployment.

## Results

### Session characteristics and inter-rater reliability

A total of 103 sessions were evaluated (one student did not complete the study), comprising 50 AML and 53 CML simulations distributed across four platforms: Gemini (*n* = 29, 28.2%), GPT-4o (*n* = 29, 28.2%), DeepSeek (*n* = 23, 22.3%), and Claude (*n* = 22, 21.4%). Inter-rater reliability was excellent, with an overall Krippendorff’s alpha of 0.83 (95% CI: 0.724–0.875), exceeding the prespecified threshold of 0.80. Pairwise agreement varied across rater pairs (Supplementary D), with near-perfect concordance between two reviewers (κ = 0.955) and moderate-to-substantial concordance for pairs involving the educator reviewer (κ = 0.633–0.656)^31^. Full scoring outputs are provided in Supplementary D, and complete session transcripts are available in Supplementary E.

### Overall session success rate

**Fig. 1:**
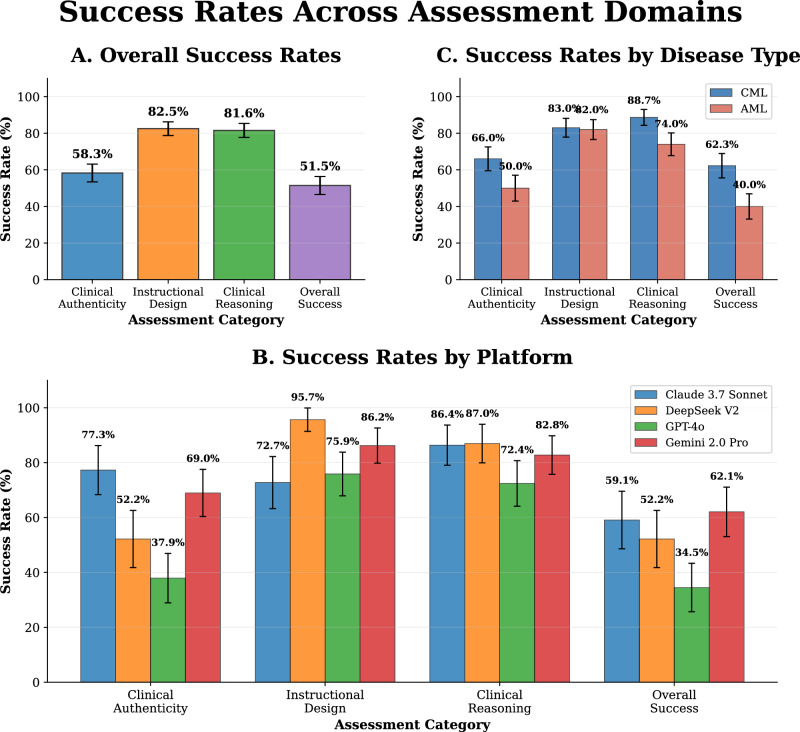
Domain-level success rates by platform and disease type. Proportion of sessions meeting domain-specific pass/fail criteria: Clinical Accuracy & Safety and Clinical Reasoning Fidelity required all subdomains ≥4.0; Instructional Design Quality required all subdomains >3.0 with mean >4.0. **A** Overall success across the three domains. **B** Comparison across LLM platforms and overall success. **C** Comparison by disease type. Error bars = standard error.

Of 103 sessions evaluated, 53 (51.5%; 95% CI: 41.7–61.2%) met passing criteria across all three domains simultaneously (Fig. 1). Domain-specific success rates were 60/103 (58.3%) for clinical accuracy and safety, 84/103 (81.6%) for clinical reasoning fidelity, and 85/103 (82.5%) for instructional design quality, with clinical accuracy and safety emerging as the limiting domain.

### Performance by disease type

CML sessions demonstrated higher overall success rates than AML sessions: 33/53 (62.3%) versus 20/50 (40.0%; OR (CML vs AML) = 2.50, 95% CI: 1.12–5.56; RR (CML/AML) = 1.56, 95% CI: 1.04–2.32; Cohen’s h = 0.449; *p* = 0.031) (Fig. 1). Domain-specific comparisons showed consistent trends favoring CML: clinical accuracy and safety CML 35/53 (66.0%) versus AML 25/50 (50.0%); clinical reasoning fidelity CML 47/53 (88.7%) versus AML 37/50 (74.0%); instructional design quality remained nearly equivalent at CML 44/53 (83.0%) versus AML 41/50 (82.0%).

**Fig. 2:**
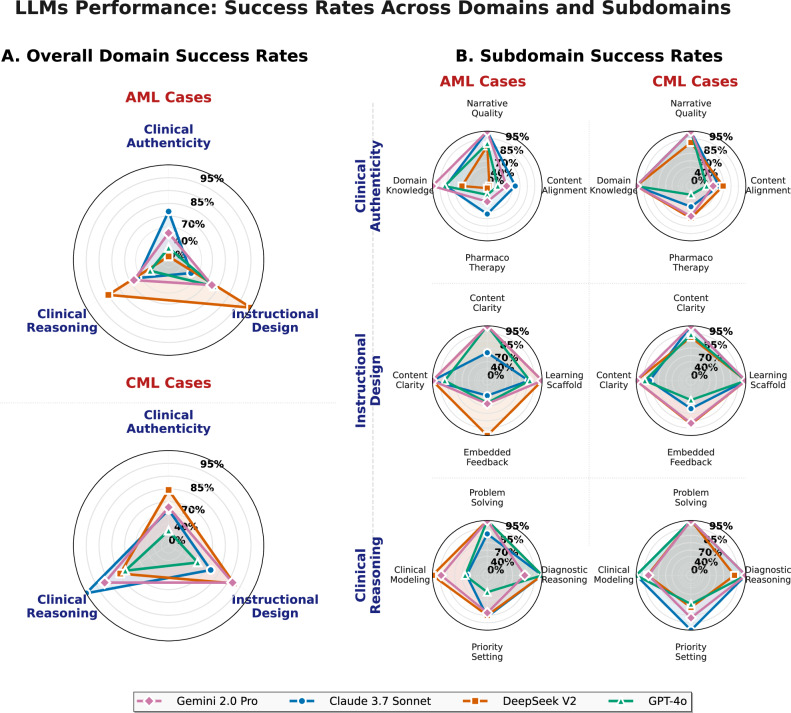
LLM performance profiles across domains and subdomains. **A** Overall success rates by domain for AML (top) and CML (bottom) cases. **B** Subdomain success rates by domain (rows) and disease type (columns: AML left, CML right). Radial axes apply a three-tier transformation to enhance separation at high-performance levels. Platforms are ranked by overall session success. AML acute myeloid leukemia, CML chronic myeloid leukemia.

At the subdomain level, the largest performance gaps occurred within clinical accuracy and safety (Fig. 2), guideline alignment: CML 40/53 (75.5%) versus AML 28/50 (56.0%), pharmacotherapeutic accuracy: CML 36/53 (67.9%) versus AML 26/50 (52.0%), domain specificity: CML 53/53 (100.0%) versus AML 46/50 (92.0%), with domain entanglement occurring exclusively in AML sessions (4/50, 8.0% vs 0/53, 0%). This relatively low event frequency limits robust mechanistic interpretation. Clinical reasoning fidelity and instructional design quality subdomains showed minimal disease-type variation.

### Performance by platform

Platform-level overall success rates ranged from 34.5% to 62.1%: Gemini 2.0 Pro 18/29 (62.1%, 95% CI: 44.0–77.3%), Claude 3.7 Sonnet 13/22 (59.1%, 95% CI: 38.7–76.7%), DeepSeek V2 12/23 (52.2%, 95% CI: 33.0–70.8%), and GPT-4o 10/29 (34.5%, 95% CI: 19.9–52.7%) (Fig. 1). Chi-square analysis revealed no significant platform differences (*p* = 0.160, Cramér’s V = 0.224). Post hoc power analysis confirmed inadequate power for all platform comparisons (5.5–39.7%).

Domain-specific analysis revealed platform strengths and weaknesses (Fig. 1). For clinical accuracy and safety, performance spanned 39.4 percentage points: Claude 17/22 (77.3%), Gemini 20/29 (69.0%), DeepSeek 12/23 (52.2%), and GPT-4o 11/29 (37.9%). For instructional design quality: DeepSeek 22/23 (95.7%), Gemini 27/29 (93.1%), GPT-4o 25/29 (86.2%), and Claude 17/22 (77.3%). For clinical reasoning fidelity: DeepSeek 20/23 (87.0%), Claude 19/22 (86.4%), Gemini 24/29 (82.8%), and GPT-4o 21/29 (72.4%).

DeepSeek demonstrated marked disease-specific performance variation: CML sessions 11/13 (84.6%) versus AML sessions 1/10 (10.0%; OR (CML vs AML) = 50.0, 95% CI: 3.85–∞; RR (CML/AML) = 8.46; *p* < 0.001).

### Subdomain-level performance patterns

Success rates varied across twelve subdomains (Fig. 2), ranging from 60.2% to 99.0%. The weakest performers were pharmacotherapeutic accuracy (62/103, 60.2%) and guideline alignment (68/103, 66.0%), both within clinical accuracy and safety. The strongest performers were problem identification (102/103, 99.0%), scaffolding quality (101/103, 98.1%), instructional framing (100/103, 97.1%), and clinical narrative plausibility (100/103, 97.1%).

**Fig. 3:**
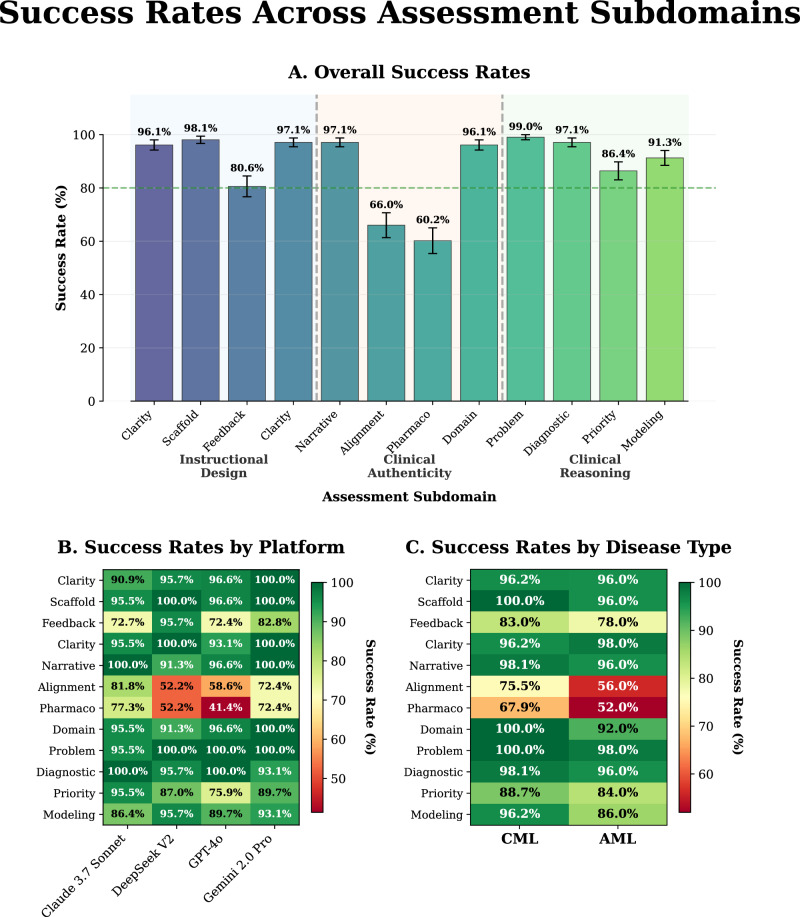
Subdomain-level success rates. **A** Overall success rates with standard error bars; pharmacotherapeutic accuracy and guideline alignment were the lowest-performing domains. **B** Platform-level heatmap showing variation in success rates, with text optimized for contrast. **C** Disease-type heatmap comparing CML and AML performance. Asterisks indicate significance: ***p < 0.001, **p < 0.01, *p < 0.05. AML acute myeloid leukemia, CML chronic myeloid leukemia.

Platform-specific patterns (Fig. 2) revealed GPT-4o with consistent weaknesses in pharmacotherapeutic accuracy (12/29, 41.4%) and guideline alignment (17/29, 58.6%). Claude achieved the highest clinical accuracy and safety performance (17/22, 77.3%) with balanced subdomain scores. DeepSeek demonstrated near-perfect instructional design quality (22/23, 95.7%) but moderate clinical accuracy and safety performance. Gemini showed consistent performance across all subdomain categories (Fig. 3).

### Clinical error analysis

Error analysis revealed three prominent failure patterns (Table 1). Domain entanglement occurred exclusively in AML sessions (4/50, 8.0%), where therapies from related hematologic conditions were inappropriately applied—including blinatumomab (a B-ALL-specific agent) recommended for AML and differentiation syndrome incorrectly attributed to standard chemotherapy^32^. Fabricated evidence emerged in 9 sessions, presenting invented clinical trials with specific statistical outcomes (e.g., “MORPHO trial^33,34^ NEJM 2023” with false gilteritinib data) and mathematically impossible risk scoring formulas. The most frequent errors involved guideline misalignment (22 AML, 13 CML) and pharmacotherapeutic inaccuracies (24 AML, 17 CML), including concurrent allopurinol with rasburicase, premature treatment-free remission attempts, and inappropriate therapy escalations at warning response milestones.

**Table 1:** Error patterns in LLM-generated hematologic pharmacotherapy simulations

Assessment domain	Subdomain	AML (*N* = 50)	CML (*N* = 53)	Error patterns and example session IDs
Clinical accuracy and safety	**Overall domain failure**	**25 (50.0%)**	**18 (34.0%)**	Lowest pass rate (60/103, 58.3%); required all 4 subdomains ≥4.0 with no exceptions permitted
	Guideline alignment	22 (44.0%)	13 (24.5%)	**AML:** FLT3-ITD+/NPM1- risk classification inconsistencies; TLS high-risk criteria misapplied (WBC >25k vs >100k); IDSA/ESMO guideline deviations (Sessions 2, 4, 5, 6, 7, 11, 18, 19, 20, 23, 24, 25, 26, 38, 39, 47). **CML:** BCR-ABL1 response thresholds misapplied (8.5% at 3 months as “warning” vs optimal); ELTS score calculation errors; TFR timing violations; mutation analysis omissions (Sessions 56, 58, 79, 84, 88, 89, 97, 98, 99, 100, 102)
	Pharmacotherapeutic accuracy	24 (48.0%)	17 (32.1%)	**AML:** Concurrent allopurinol with rasburicase; blinatumomab (B-ALL therapy) for AML; venetoclax maintenance as first-line for favorable-risk AML (HiDAC indicated); rasburicase for low-risk TLS; inappropriate consolidation for refractory disease; pegfilgrastim during induction (Sessions 1, 2, 3, 4, 5, 6, 7, 8, 9, 10, 11, 12, 13, 15, 16, 17, 20, 21, 22, 30, 40). **CML:** Premature TFR after 1 year MMR (requires ≥2 years MR4.5); dasatinib switch for nilotinib-induced pleural effusion; suboptimal imatinib for intermediate-risk patients (Sessions 58, 79, 84, 88, 89, 97, 98, 99, 100, 102)
	Domain specificity	4 (8.0%)	0 (0%)	**AML:** Session 1 (blinatumomab - CD19-targeted B-ALL therapy for NPM1-mutated AML); Session 2 (sorafenib for KIT-mutated AML based on FLT3-ITD evidence); Session 21 (differentiation syndrome for standard 7 + 3 chemotherapy); Session 35 (CML content in AML session). **CML:** No domain entanglement
	Clinical plausibility	3 (6.0%)	0 (0%)	**AML:** Session 1 (hydroxyurea “not indicated” then “WBC decreased after hydroxyurea”); Session 12 (missing blast percentage); Session 22 (severe thrombocytopenia with “normal coagulation”). **CML:** No plausibility failures
	Fabricated evidence	7 (14.0%)	2 (3.8%)	**AML:** Sessions 3, 4, 5, 8, 9, 10, 13 (“MORPHO trial NEJM 2023” with false gilteritinib outcomes - actual trial negative; “RELMAZA trial” with invented azacitidine data). **CML:** Sessions 89, 100, possibly 79 (fabricated ELTS formulas with non-existent coefficients; mathematically impossible calculations)
Clinical reasoning fidelity	**Overall domain failure**^a^	**13 (26.0%)**	**6 (11.3%)**	Required all 4 subdomains ≥4.0; pass rate 84/103 (81.6%); multiple concurrent reasoning errors per session common
	Response classification	8 (16.0%)	4 (7.5%)	**AML:** 31.5% blasts as “partial response” vs refractory disease; CR vs CRi misapplication (Sessions 14, 40, 41, 44). **CML:** BCR-ABL1 15% as “optimal” vs failure; 8.5% as “warning” vs optimal (Sessions 58, 78, 79, 98)
	Treatment sequencing	11 (22.0%)	4 (7.5%)	**AML:** Consolidation for refractory disease; Day 14 BM 5% blasts triggering second-line; venetoclax first-line vs HiDAC (Sessions 9, 11, 12, 16, 21, 28, 35, 38, 40, 42, 49). **CML:** TKI switching without mutation analysis; escalation at “warning”; premature TFR (Sessions 88, 99, 102, plus 1 additional)
	Answer-question mismatch	4 (8.0%)	1 (1.9%)	**AML:** TLS question receiving antifungal rationale; febrile neutropenia answered with consolidation (Sessions 34, 35, 39, 41). **CML:** Question-answer disconnection (Session 92)
	Question validity	1 (2.0%)	2 (3.8%)	**AML:** No correct answer among options (Session 51). **CML:** Sokal question with no valid components; unclear “none of options” (Sessions 56, 73)
Instructional design quality	**Overall domain failure**^b^	**9 (18.0%)**	**9 (17.0%)**	Highest pass rate (85/103, 82.5%); flexible criteria (subdomains >3.0, mean >4.0) permitted compensation
	Learning objectives	50 (100%)	50 (94.3%)	All AML; all CML except Sessions 78, 82, 91 lacked explicit objectives; goals implied through structure; noted as “minor weakness” in passing sessions
	Embedded feedback^b^	3 (6.0%)	8 (15.1%)	**AML:** Only “Correct!” without rationale (Sessions 14, 19, 34). **CML:** No explanatory feedback (Sessions 56, 73, 85, 94, 100)
	Scaffolding quality	2 (4.0%)	0 (0%)	Strongest subdomain (101/103, 98.1% pass). **Examples:** Sessions 24, 35 (format collapse; lost progression)
	Instructional framing	3 (6.0%)	4 (7.5%)	Second-strongest (100/103, 97.1% pass). **AML:** Sessions 4, 11, 42. **CML:** Sessions 69, 70, 76, 80 lacking proper introduction
	Absence of final scoring	48 (96.0%)	50 (94.3%)	Only 5 sessions provided summaries (7, 52, 66, 80, 84); 98 sessions (95.1%) terminated without learning closure
	Case demographic uniformity	50 (100%)	3 (5.7%)	**AML:** Ages 42–47 years; identical symptoms (early satiety, weight loss). **CML:** Sessions 57, 61, 63 with duplicates/multiple cases
	Disease stage uniformity	NA	53 (100%)	All CML chronic phase only; zero accelerated phase or blast crisis presentations limiting disease spectrum exposure
	Answer revelation	0 (0%)	2 (3.8%)	Sessions 71, 96: Complete answers on hint request; listing answers before student attempts
	Language switching	0 (0%)	1 (1.9%)	Session 103: English to Arabic mid-session without justification

*AML* acute myeloid leukemia, *CML* chronic myeloid leukemia, *CR* complete remission, *CRi* incomplete recovery, *ELN* European LeukemiaNet, *ELTS* EUTOS long-term survival, *HiDAC* high-dose cytarabine, *MR4.5* 4.5-log reduction, *MMR* major molecular response, *MRD* measurable residual disease, *N/A* not applicable, *TFR* treatment-free remission, *TKI* tyrosine kinase inhibitor, *TLS* tumor lysis syndrome, *WBC* white blood cell count.

^a^Individual sessions may exhibit multiple reasoning errors across categories.

^b^Instructional Design error frequencies reflect observed quality limitations; domain pass/fail was determined by combined performance across all subdomains (Methods - Expert evaluation procedures).

### Student satisfaction and preference-safety alignment

**Fig. 4:**
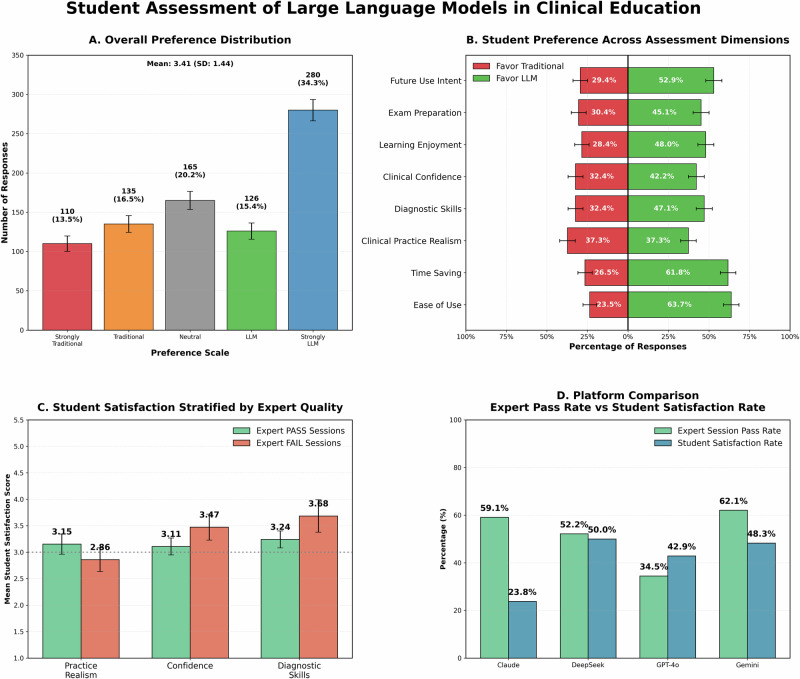
Student assessment of large language models in clinical education. **A** Overall 5-point Likert preferences (red = traditional, gray = neutral, green-blue = LLM). **B** Diverging bars for eight assessment dimensions (1–2 left, 4–5 right; neutral excluded). **C** Satisfaction by expert session quality (PASS vs FAIL; SEM; Mann–Whitney U, FDR-corrected). **D** Expert pass rates (green) vs student satisfaction (blue) across four LLM platforms. SE standard error, SEM standard error of the mean, FDR false discovery rate.

Student satisfaction data were obtained from 102 participants (one completed the simulation but not the survey) with excellent internal consistency (Cronbach’s α = 0.939; Supplementary D). Overall mean satisfaction score was 3.41 (SD = 1.44), significantly above the neutral midpoint of 3.0 (*p* < 0.001, Cohen’s d = 0.282) (Fig. 4).

Preference distribution showed 406 responses (49.8%) favoring LLMs, 165 (20.2%) neutral, and 245 (30.0%) favoring traditional methods, differing significantly from uniform expectations (*p* < 0.001, Cohen’s w = 0.368).

Students significantly favored LLMs for ease of use (65/102, 63.7%, 95% CI: 54.1–72.4%, Cohen’s h = 0.278, *p* = 0.007) and time saving (63/102, 61.8%, 95% CI: 52.1–70.6%, Cohen’s h = 0.238, *p* = 0.022) but significantly favored traditional methods for clinical practice realism (38/102, 37.3%, 95% CI: 28.5–46.9%, Cohen’s h = −0.258, *p* = 0.013) (Fig. 4). No directional preferences emerged for other dimensions including diagnostic skills, clinical confidence, learning enjoyment, exam preparation, or future use intent. Mean satisfaction scores ranged across platforms with no significant platform differences (*p* = 0.442, **ε²** ≈ **0**), with DeepSeek receiving the highest satisfaction (M = 3.68, SD = 1.14) and Claude the lowest (M = 3.11, SD = 1.03).

Stratified analyses did not detect statistically significant alignment between student satisfaction and expert-assessed content quality in this sample (Fig. 4), and these comparisons were not powered to exclude moderate associations. Effect sizes and confidence intervals for these stratified comparisons are provided in Supplementary D.

Students who experienced sessions meeting expert clinical accuracy and safety standards reported similar satisfaction on clinical practice realism compared to those who experienced failing sessions (3.15 vs 2.86, *q* = 0.626). For clinical confidence and diagnostic skills improvement, students who experienced sessions failing expert clinical reasoning fidelity criteria reported numerically higher satisfaction than those who experienced passing sessions (3.47 vs 3.11 and 3.68 vs 3.24, respectively), though differences were not statistically significant (*q* = 0.653 and *q* = 0.259). With only four platforms, rank correlation analysis is underpowered to yield interpretable results; we therefore present platform-level satisfaction and safety pass rates descriptively in Fig. 4. No confidence interval is reported for ρ = −0.80 given that bootstrap resampling is unreliable at *n* = 4 platforms. DeepSeek received the highest student preference (62.5%) but demonstrated 52.2% safety pass rate, while Claude achieved the highest safety rate (77.3%) but received only 52.4% student preference.

## Discussion

We systematically evaluated four major LLMs in generating pharmacotherapy simulations for AML and CML across instructional design quality, clinical accuracy and safety, and clinical reasoning fidelity. Overall success reached 51.5%, with notable domain heterogeneity. Models demonstrated relatively strong performance in instructional design (82.5%) and clinical reasoning fidelity (81.6%), while clinical accuracy and safety emerged as the primary limitation (58.3%). Current models appear capable of structuring learning experiences and modeling reasoning processes but demonstrate challenges with precise clinical content generation, indicating a need for targeted improvements and rigorous oversight before high-stakes educational deployment.

The clinical accuracy and safety domain showed uneven performance across its four subdomains. Clinical narrative plausibility and domain specificity both exceeded 90% success, indicating models can generate coherent scenarios and often maintain disease-specific boundaries when guided by structured prompts. However, pharmacotherapeutic accuracy and guideline alignment showed substantially lower success. Models appear capable of constructing believable clinical narratives but demonstrate difficulties translating evidence-based guidelines into accurate therapeutic recommendations. High narrative plausibility may explain why students engaged positively with content despite its pharmacotherapeutic inaccuracies, which highlights the need for expert review before educational deployment. In contrast, performance in instructional design quality and clinical reasoning fidelity remained relatively consistent across platforms and disease types. Scaffolding quality, instructional framing, learning objective clarity, problem identification, and knowledge integration all achieved relatively strong success with minimal variation. This consistency may indicate that pedagogical structure generation and reasoning process modeling represent more stable capabilities, potentially less sensitive to clinical complexity than content accuracy. The contrast between relatively stable pedagogical performance and variable clinical accuracy reinforces that content precision represents a primary technical challenge.

The disease-specific performance differences we observed provide insight into factors that influence model capabilities and suggest how findings may generalize to other conditions. Acute and chronic myeloid leukemia share myeloid cell lineage with overlapping clinical features, creating semantic similarity that prior literature suggested might challenge boundary preservation in language models^16,19,35,36^. Their management approaches differ fundamentally. CML follows relatively linear therapeutic pathways with tyrosine kinase inhibitor selection based on comorbidities and molecular monitoring^35^, while AML requires complex decision trees dependent on molecular subtype, induction response, and consolidation eligibility^36^.

This pairing allowed us to test whether semantic overlap causes domain entanglement and whether therapeutic complexity affects performance. Our findings are consistent with both concerns; however, the low absolute frequency of domain entanglement events limits mechanistic inference, and we treat entanglement as a hypothesis-generating failure mode warranting targeted experimental testing (e.g., prompt ablation studies and retrieval-augmented generation comparisons). Domain entanglement occurred exclusively in AML sessions, where models recommended blinatumomab, a B-cell acute lymphoblastic leukemia-specific agent, and incorrectly attributed differentiation syndrome to standard chemotherapy, demonstrating that semantic similarity may override explicit prompt constraints^32^. CML sessions succeeded more often, particularly in guideline alignment and pharmacotherapeutic accuracy.

These findings generate hypotheses for other domains. Conditions requiring multi-variable conditional reasoning may pose a higher risk for clinically important errors than conditions governed by more linear algorithms; however, generalizability beyond AML/CML requires direct empirical testing across additional disease states. Conceptually, conditions requiring complex multi-variable conditional reasoning (e.g., sepsis management, advanced heart failure therapy, or complex anticoagulation strategies) may present greater susceptibility to prompt override due to their non-linear decision pathways. In contrast, semantically related conditions governed by relatively structured or linear therapeutic algorithms (e.g., Crohn’s disease vs ulcerative colitis, type 1 vs type 2 diabetes, bacterial vs viral meningitis) may theoretically demonstrate higher discrimination accuracy. Establishing cross-domain robustness would require dedicated studies across additional disease pairs.

Platform patterns revealed meaningful heterogeneity. Gemini achieved the highest overall success with consistent cross-disease performance. Claude demonstrated the highest clinical accuracy with balanced disease-type performance, which may reflect conservative, guideline-adherent generation. DeepSeek demonstrated substantial disease-dependent variation, succeeding far more often with CML than AML, potentially reflecting training data imbalances or differential sensitivity to complexity. GPT-4o demonstrated lower clinical accuracy despite adequate instructional design. This variation is consistent with prior findings of domain-dependent LLM performance, indicating the need for disease-specific validation before implementation^37–39^.

Error analysis revealed patterns that may inform oversight and development priorities. Guideline misalignment reflected apparent temporal bias toward older training data, which may suggest models revert to statistically dominant training patterns when generating extended content^40^. Fabricated evidence manifested as confident citations of non-existent trials, which may indicate architectures lack robust mechanisms to verify claims or signal uncertainty. Pharmacotherapeutic inaccuracies included clinically concerning recommendations such as concurrent allopurinol-rasburicase administration, potentially reflecting failures in multi-step conditional reasoning^39^. Domain entanglement occurred despite explicit negative constraints, suggesting possible architectural limitations in maintaining categorical boundaries. These patterns indicate that while prompt engineering can elicit pedagogical structure, clinical safety requires model-level improvements, including citation verification, uncertainty quantification, temporal weighting, and enhanced boundary preservation.

Our meta-prompt framework showed promise in maintaining domain specificity and enabled systematic evaluation by standardizing content generation across platforms and disease types^41^. The framework incorporated five boundary-preservation mechanisms, including disease-specific guideline anchoring, negative constraints prohibiting cross-referencing related conditions, and consistency verification requirements. High domain specificity success (95.1%) with relatively limited entanglement (3.9% of sessions) suggests these mechanisms helped maintain therapeutic boundaries in most cases. However, the persistence of entanglement errors in complex acute myeloid leukemia cases indicates that prompt engineering alone may be insufficient to overcome architectural limitations when semantic similarity is high. Beyond enabling systematic evaluation, the template-based design allows educators to adapt the framework through variable substitution while preserving safety constraints, thereby democratizing LLM-based simulation development and offering a potential approach for research on optimal prompt architecture in medical education.

The comprehensive expert validation conducted in this study may initially appear to contradict claims of scalability for LLM-generated simulations. However, in safety-critical clinical education, the appropriate goal is augmentation of expert judgment rather than its replacement. Inaccurate pharmacotherapy recommendations embedded in training simulations risk propagating incorrect clinical reasoning patterns to learners, creating downstream patient-safety concerns. Our findings empirically support this concern: 48.5% of unmoderated sessions failed to meet minimum expert safety and accuracy criteria, yet students experiencing these sessions reported satisfaction levels statistically indistinguishable from those experiencing passing sessions, suggesting learners may not reliably detect content quality deficiencies without expert guidance. Beyond expert evaluation, established frameworks recommend incorporating learner perspectives when assessing educational technologies^42^. We included student satisfaction assessment to capture authentic end-user experience, allowing natural interaction without experimental constraints to identify real-world engagement patterns and implementation challenges^43,44^. The stratified analysis examined whether student satisfaction on specific dimensions aligned with expert evaluation of corresponding session quality. We interpret satisfaction as feasibility evidence (acceptability and usability) rather than evidence of educational effectiveness, which requires objective learning outcome measures in subsequent studies.

Stratified analyses did not detect statistically significant alignment between student satisfaction and expert-assessed content quality in this sample. Students experiencing sessions meeting expert clinical accuracy and safety standards reported similar clinical practice realism satisfaction as those experiencing failing sessions. For clinical confidence and diagnostic skills improvement, students experiencing sessions failing expert clinical reasoning fidelity criteria reported numerically higher satisfaction than those experiencing passing sessions, though differences were not statistically significant. Although these comparisons were underpowered to exclude moderate effects, the observed dissociation—positive learner experience even in sessions with expert-identified safety or guideline violations—supports the governance interpretation that student satisfaction alone is insufficient for validating clinical content quality, consistent with cognitive theory on fluency-driven illusions of competence^45–51^.

Platform-level patterns reinforced this disconnect. Gemini achieved the highest expert pass rate but moderate student satisfaction, while GPT-4o demonstrated the lowest expert pass rate with similar student satisfaction^52,53^. Combined with stratified analysis showing a lack of alignment between individual satisfaction and session quality, this may suggest that students base preferences on factors unrelated to clinical accuracy, such as conversational style or response elaboration, rather than expert-identified educational value or safety. Students significantly favored models for ease of use but significantly favored traditional methods for clinical practice realism. The ease of use advantage likely reflects immediate accessibility and conversational interaction, while preference for traditional methods regarding clinical realism suggests students recognized that model-generated scenarios might lack the authenticity of faculty-developed cases. This dimensional variation indicates students may discern certain experiential aspects but appear unable to evaluate clinical accuracy.

This potential inability to discriminate content quality has implications for unsupervised deployment. Students may find content appealing based on polished presentation, confident tone, and engaging narratives while potentially unable to identify underlying clinical inaccuracies, fabricated evidence, or guideline violations. High narrative plausibility combined with low pharmacotherapeutic accuracy creates conditions where learners may encounter content appearing authentic while containing clinically inappropriate recommendations. Advanced pharmacy students, despite substantial clinical knowledge, showed no significant ability to identify quality differences that experts readily detected, which may suggest clinical background alone does not protect learners and that structured oversight may be necessary even for experienced students^54^.

For safe implementation and future development, educators and developers face different but complementary priorities. Developers need to improve models by integrating real-time guideline updates with temporal weighting to reduce reliance on outdated patterns, implementing citation verification to prevent fabricated evidence, strengthening boundary-preservation mechanisms to limit domain entanglement, and enhancing logical consistency checks for multi-step therapeutic reasoning^55,56^. Educators must recognize current limitations and ensure that all LLM-generated content undergoes expert review, with particular attention to pharmacotherapeutic recommendations and guideline adherence in complex therapeutic areas.

The patterns of errors observed suggest several directions for research and development. Validation of the meta-prompt framework across diverse therapeutic domains is necessary to determine whether it can generalize beyond hematologic malignancies. Comparative studies of retrieval-augmented generation with real-time guideline access may reveal whether architectural modifications can reduce gaps in accuracy. Development of automated error detection targeting guideline violations, fabricated citations, domain entanglement, and reasoning failures could support scalable quality assurance. Multi-institutional studies with larger samples would help clarify whether observed platform differences and complexity-dependent performance patterns are robust, providing guidance for both model refinement and educational deployment. Future work should also include error clustering stratified by AML molecular subtype/risk category (e.g., FLT3/NPM1/IDH-driven decision branches) to determine whether failures concentrate in specific therapeutic pathways.

We applied non-compensatory thresholds for clinical accuracy and safety and clinical reasoning fidelity, and compensatory scoring for instructional design quality. This approach is consequence-based: in clinically oriented domains, compensatory averaging can mask safety-critical deficiencies, a concern reflected in patient safety–oriented standard setting and mastery learning frameworks that treat critical actions as non-compensatory requirements^57,58^. In contrast, instructional design elements may compensate for one another within a minimum-quality floor without introducing direct patient-safety risk^5,59^. These thresholds should be interpreted as conservative deployment criteria for this Phase 1 validation stage rather than as a claim that all clinical errors have equivalent severity. Accordingly, we report a structured error taxonomy (Table 1), distinguishing failure modes with potentially different consequences. Future work should develop severity-weighted scoring and explicit “never-event” catalogs through formal expert consensus (e.g., modified Delphi) and psychometric calibration (e.g., Rasch modeling or item response theory), while retaining non-compensatory gating for errors judged unacceptable for learner exposure^60^.

This study has several limitations. Sample sizes per platform were sufficient for overall characterization but offered limited power to detect small differences, making platform comparisons exploratory. Single-institution implementation and evaluation limited to two hematologic malignancies constrain external generalizability. Although differences in therapeutic complexity may conceptually influence model performance—particularly in conditions requiring multi-variable conditional reasoning—such extrapolation remains hypothetical and requires empirical validation across additional disease domains. While our design ensures a focused evaluation of each model, it precludes direct learner-based comparative analysis. Expert reviewers were not blinded to platform identity, but structured rubrics and high inter-rater reliability may have reduced bias. Conservative pass-fail thresholds prioritized safety but require further empirical evaluation. The evaluation framework and meta-prompt require formal validation, and stratified analyses had limited power, particularly for clinical reasoning domains, which may have contributed to non-significant findings despite meaningful numerical trends.

Moreover, the review intensity used in this study reflects research-grade benchmarking intended to characterize failure modes and support early validity evidence for the rubric. In this dataset, 48.5% of sessions failed to meet minimum criteria for clinical accuracy and safety, while student satisfaction ratings were statistically similar between sessions that passed and failed expert criteria. Scalability should be considered relative to the appropriate comparator workflow: LLM-assisted draft generation with structured expert verification versus traditional simulation development workflows. The study did not quantify time or cost differences. Drawing on patient-safety approaches to standard setting and critical-action gating, severity-stratified medication safety systems, and implementation governance considerations, we propose a risk-stratified quality assurance approach in which review intensity is calibrated to clinical consequence^50,58,60–63^. Operational deployment would likely require risk-stratified review.

Additionally, because model behavior may change over time in proprietary web interfaces, replication using the same prompts and rubric at future time points is required to assess the temporal stability of observed failure modes. Unfortunately, this type of version drift is now typical for continuously deployed proprietary LLMs across both web and API access routes, so transparent reporting and longitudinal re-testing are currently the most practical mitigation strategies when version-pinned snapshots are unavailable^64^. Future work should also consider benchmarking smaller locally deployable models (or institution-hosted open-weight models) that can be maintained as frozen snapshots, enabling stronger reproducibility and implementation governance than continuously updated proprietary endpoints^64^.

Additionally, a key limitation is the expert panel composition (three co-authors from one institution involved in rubric and meta-prompt development), which may introduce confirmation bias. The reported reliability reflects internal scoring consistency rather than independent external validation; stronger support for independence requires replication with external and/or blinded raters. Phase 2 work should incorporate independent external clinical experts, ideally blinded to platform identity and study hypotheses, to strengthen extrapolation and generalizability inferences^65,66^. Also, we did not perform prompt ablation experiments; therefore, we cannot attribute observed performance to any specific safeguard mechanism within the meta-prompt. DeepSeek’s marked disease-dependent variation suggests platform-specific sensitivity to therapeutic complexity or training data distribution; explaining these effects would require model-level access not available in web-interface evaluations.

Finally, because each student used only one platform, within-subject platform comparisons were not feasible in this Phase 1 content validation study, which prioritized independent expert evaluation of diverse platform outputs over learner-centered comparative usability testing. Future studies should evaluate multiple platforms per learner using counterbalanced designs with temporal spacing, disease context rotation, and burden management to strengthen evidence about individual preference patterns while controlling for carryover learning effects and fatigue. Additionally, evaluating LLM simulations across multiple professional years may clarify prerequisite knowledge thresholds and the optimal curricular timing for LLM-assisted simulation within vertically and horizontally integrated curricula.

In conclusion, using controlled meta-prompting, sessions more frequently met criteria for instructional design and clinical reasoning than for pharmacotherapeutic accuracy and guideline alignment, with performance varying by platform and disease context. Expert oversight with platform-specific and disease-specific validation remains essential for safe educational deployment, and effectiveness trials assessing objective learning outcomes represent necessary subsequent work.

## Methods

### Study design

**Fig. 5:**
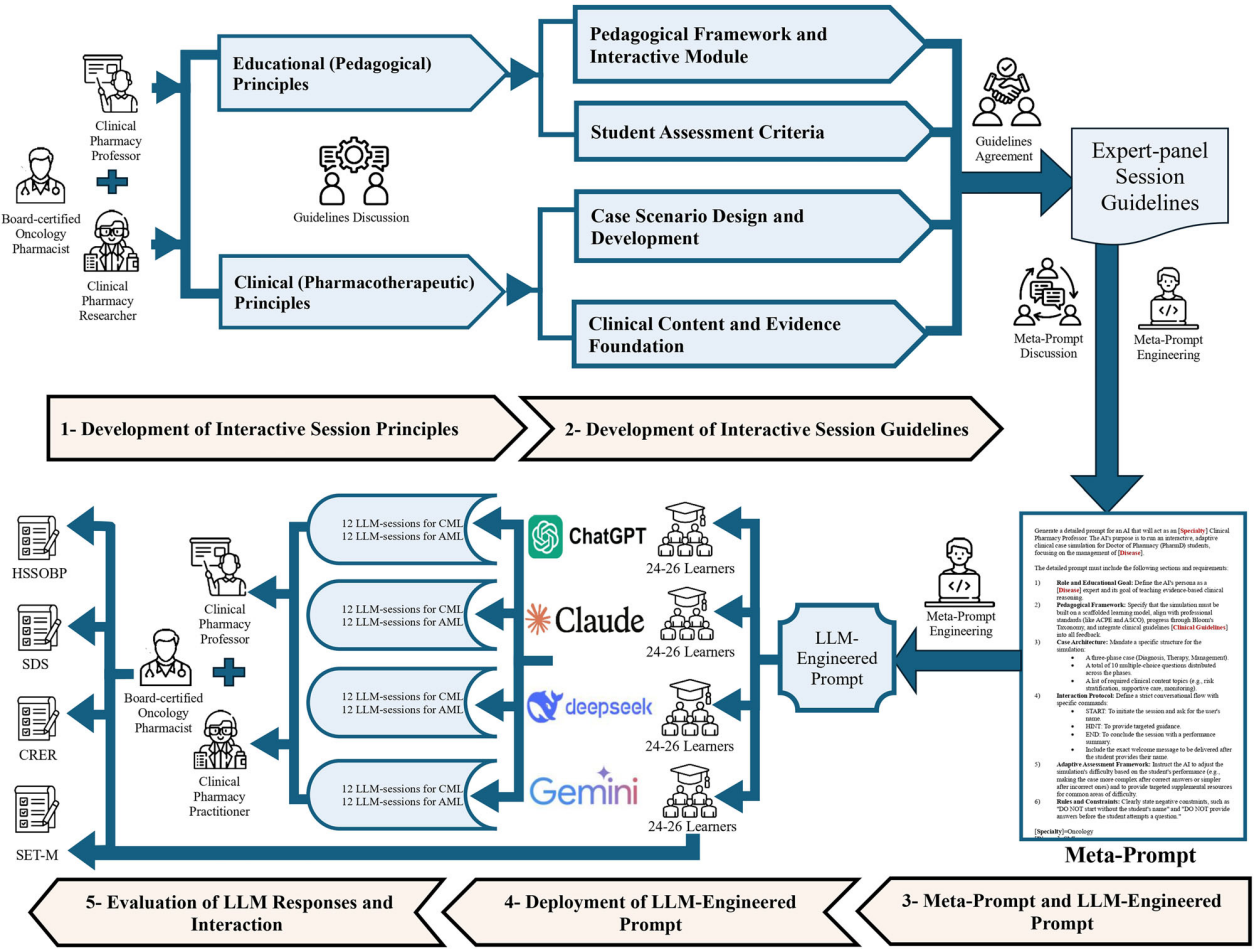
Framework for evaluating LLMs in pharmacotherapy. Five-phase systematic assessment of LLM-generated AML/CML simulations: (1–2) Expert panel established integrated pedagogical and clinical guidelines. (3) Guidelines encoded into a standardized meta-prompt with AI role, case structure, interaction, and assessment modules. (4) Deployment across four LLM platforms (ChatGPT, Claude, DeepSeek, Gemini). (5) Evaluation using HSSOBP, SDS, CRER, and SET-M. LLM large language model, AML acute myeloid leukemia, CML chronic myeloid leukemia, HSSOBP Healthcare Simulation Standards of Best Practice, SDS simulation design scale, CRER clinical reasoning evaluation rubric, SET-M simulation effectiveness tool–modified.

This was a phase 1 mixed-methods evaluation study evaluating educational materials generated during routine curricular activities from March 15 to April 10, 2025, at a single institution^67^. The study aimed to characterize LLM performance in generating pharmacotherapy simulations and to assess student perceptions of this learning modality, as outlined in the five-phase evaluation framework (Fig. 5). All clinical evaluations were benchmarked against 2022 National Comprehensive Cancer Network/European LeukemiaNet (NCCN/ELN)^36^ guidelines for AML and 2017 European Society for Medical Oncology/European LeukemiaNet (ESMO/ELN) guidelines for CML^35^, which served as the reference standards for assessing clinical accuracy and guideline adherence. This study was approved by the Research Ethics Committee of the Faculty of Pharmacy, Cairo University (Approval ID: CL 3931). All participants provided informed consent.

### Framework development and meta-prompt engineering

LLMs demonstrate substantial sensitivity to prompt design, with well-structured prompts shown to reduce hallucination and improve performance^16^. However, effective prompt engineering typically requires specialized technical expertise that may limit accessibility for educators. To address this barrier while maintaining rigor, we developed a systematic meta-prompt framework that bridges clinical domain expertise with prompt engineering principles^41^. This approach enables educators to generate high-quality pharmacotherapy simulations through structured variable substitution rather than de novo prompt development, providing a transparent and replicable method adaptable across educational settings.

Development proceeded in three stages. In the first stage, a multidisciplinary team consisting of a clinical pharmacy educator (AA), board-certified oncology pharmacist (AZ), and clinical pharmacy researcher (AF) defined foundational principles for simulation design informed by scaffolding theory and cognitive load theory (Supplementary A)^4–6,68–70^. In the second stage, these principles were translated into a universal meta-prompt template with variable placeholders for leukemia type, specialty, and guideline source (Supplementary B). The template defined the model’s role as clinical pharmacy professor, structured learner engagement through scaffolded progression aligned with Bloom’s Taxonomy^41^, and required strict guideline adherence across diagnostic, therapeutic, and follow-up scenarios. Five safeguard mechanisms were embedded to minimize domain entanglement and other clinical errors: (1) disease-specific guideline anchoring, (2) negative constraints prohibiting reference to related conditions, (3) boundary reinforcement directing limitation statements when uncertain, (4) citation requirements linking recommendations to specific guideline sections, and (5) consistency verification before each recommendation.

In the third stage, final prompts were generated using Google AI Studio (Gemini 2.0 Pro), selected for its extended context window, stable output, and academic licensing (Supplementary B)^71,72^. Subject-matter experts reviewed and standardized these prompts to remove platform-specific phrasing before deployment across all four platforms.

### Participants and randomization

We recruited 104 fourth-year Doctor of Pharmacy students after obtaining informed consent. Participants were initially randomized to one of two leukemia contexts (AML or CML) for their LLM-based simulation. To minimize sequence effects and ensure balanced academic preparedness across study arms, students were further randomized in a counterbalanced manner to receive either the LLM simulation first, followed by a traditional case-based session, or vice versa. Within each leukemia context, students assigned to the LLM condition were randomized to one of four platforms (ChatGPT GPT-4o^73^, Claude 3.7 Sonnet^74^, Gemini 2.0 Pro^71^, or DeepSeek V2^75^), yielding approximately 13 participants per platform–disease combination. Each student completed both leukemia contexts overall (one via LLM simulation and one via traditional case study); however, only one LLM transcript per student was included in the final analysis to minimize carryover learning effects.

The study was conducted within a traditional (discipline-based) curriculum where clinical therapeutics is taught as the capstone of a longitudinal science sequence. To ensure adequate academic preparedness, all participating fourth-year PharmD students had completed a prerequisite series of at least two pharmacology courses. This sequential scaffolding provides vertical integration, bridging foundational drug science with the advanced clinical reasoning required for complex hematologic oncology management.

### Deployment procedures

Students received 30-min training covering study protocol, procedures for initiating new sessions without prior conversation history to avoid context carryover, and documentation instructions. They deployed provided prompts on personal devices using publicly accessible web interfaces. ChatGPT GPT-4o was accessed via chat.openai.com^76^, Claude 3.7 Sonnet via claude.ai^77^, Gemini 2.0 Pro Experimental 0125 via aistudio.google.com^72^, and DeepSeek V2 via chat.deepseek.com^78^. All platforms used default web interface settings, including standard temperature parameters, no retrieval augmentation, and no custom instructions.

Students interacted naturally with platforms without experimental constraints, prioritizing external validity to enable identification of authentic implementation failure modes^43,44^. Participants exported sessions using native platform functions while DeepSeek users saved full webpages. All sessions were copy-pasted into structured forms with timestamped screenshots and session metadata to ensure complete documentation^79^.

Traditional case-based sessions mirrored the clinical complexity and modular design of large language model simulations. Students received unique patient cases followed by self-learning questions and complete guideline documents^35,36^. They submitted answers with highlighted screenshots of cited references documenting evidence-based reasoning. Cases covered diverse AML and CML contexts, including various patient demographics, disease subtypes, and molecular profiles. Essay-style questions guided students to interpret diagnostics, apply guideline reasoning, and propose management strategies. After expert review of all submitted materials, a reconciliation session was conducted with participants to identify and correct any clinical inaccuracies, preventing the propagation of misinformation. Acute myeloid leukemia (AML) and chronic myeloid leukemia (CML) were selected as a focused proof-of-concept pair due to their shared hematologic classification yet distinct therapeutic algorithms. This pairing was intended to examine potential domain entanglement within closely related malignancies rather than to establish generalizable conclusions across disease categories.

For transparency despite web-interface version drift, we report platform access routes, default interface settings, and full prompt syntax (Supplementary B), consistent with MI-CLEAR-LLM reporting guidance^80^. While backend updates cannot be controlled in web interfaces, this study provides benchmarking under ecologically valid access conditions and enables future longitudinal replication using identical prompts and the same rubric.

### Performance assessment framework

We adapted four established instruments for LLM evaluation contexts: the Healthcare Simulation Standards of Best Practice (HSSOBP)^81^ for simulation design quality, the Simulation Design Scale (SDS)^82^ for design element assessment, the Clinical Reasoning Evaluation Rubric (CRER)^83^ for reasoning process evaluation, and the Simulation Effectiveness Tool–Modified (SET-M)^84^ for learner-reported effectiveness. The expert panel reviewed all items for face and content validity, calculating Content Validity Indices using established methods^85^. Items achieving item-level Content Validity Index (CVI) ≥ 0.78 were retained, while those below were revised or excluded. Final instruments achieved scale-level CVI values ≥ 0.90, indicating excellent content validity. The adaptation process addressed fundamental challenges in evaluating LLM-generated content, including the absence of real-time facilitation and the need to assess algorithmic rather than human reasoning processes; specific adaptations for each instrument are detailed in Table 2.

**Table 2:** Instrument adaptation for LLM-generated medical simulation in education

Original instrument	Primary adaptation	Key rubric domains and subdomains^a^	Key changes	Rationale
**HSSOBP**^81^	Physical to narrative fidelity	**Domain:** Instructional Design & Pedagogical Quality **Subdomains:** Clarity of learning objectives; scaffolding of clinical reasoning; quality of embedded feedback; instructional clarity & framing	“Fidelity” reframed as “clinical narrative plausibility”; facilitation items excluded	Assess believability of case descriptions without physical environment
**SDS**^82^	Dynamic to static assessment	**Domain:** Clinical Authenticity & Content Fidelity **Subdomains:** Clinical narrative plausibility; alignment with clinical guidelines; pharmacotherapeutic accuracy; domain specificity (absence of entanglement)	Dynamic feedback items replaced with pre-designed element evaluation	Evaluate instructional architecture of static AI content
**CRER**^83^	Learner to simulation focus	**Domain:** Modeled Clinical Reasoning **Subdomains:** Problem identification & framing; diagnostic and therapeutic breadth; prioritization and decision-making; modeling of clinical outcomes	“Reversed” orientation from student performance to simulation modeling	Assess whether AI effectively demonstrates expert reasoning
**SET-M**^84^	Facilitator-independent evaluation	**Domain:** Learner-Centered Evaluation **Subdomains:** Ease of use; time efficiency; clinical realism; diagnostic skill development; confidence building; learning enjoyment; exam preparation; future use preference	Human facilitator references removed	Capture learner perception of LLM-generated content only

*AI* artificial intelligence, *CRER* clinical reasoning evaluation rubric, *HSSOBP* Healthcare Simulation Standards of Best Practice, *LLM* large language model, *SDS* simulation design scale, *SET-M* student evaluation of teaching in medicine, *S-CVI* scale-level content validity index.

^a^Three-domain, twelve-subdomain evaluation framework (S-CVI = 0.92).

The adapted instruments created a three-domain framework encompassing twelve subdomains: instructional design quality (learning objective clarity, instructional framing, scaffolding quality, embedded feedback quality); clinical accuracy and safety (clinical narrative plausibility, guideline alignment, pharmacotherapeutic accuracy, domain specificity); and clinical reasoning fidelity (clinical prioritization, knowledge integration, evidence-based decision-making, outcomes modeling).

Student satisfaction was assessed using eight 5-point Likert items adapted from validated instruments^84^. Students completed satisfaction questionnaires immediately after both traditional and LLM-based learning modalities, with comparative items addressing clinical confidence building, diagnostic skills improvement, time efficiency, and future use intentions. Internal consistency was evaluated using Cronbach’s alpha^86^. Complete evaluation rubrics, validation indices, and error taxonomy are provided in Supplementary C.

### Expert evaluation procedures

The three-member expert panel conducted independent evaluations of each session transcript using the adapted instruments described in the “Performance assessment framework” section. Each subdomain was rated on a 1-to-5 scale. Inter-rater reliability was assessed using Krippendorff’s alpha for ordinal ratings, with α ≥ 0.80 interpreted as strong agreement. Krippendorff’s α provides evidence of rating consistency but does not establish independence from shared systematic bias; stronger support for independence requires replication using external and/or blinded raters^31^. We therefore frame this work as Phase 1 content validation, consistent with staged approaches in instrument development in health professions education, where internal expert panels commonly define content boundaries and stabilize scoring prior to external replication^87–90^. Subdomain scores were calculated as the mean of independent ratings across the three reviewers. To minimize potential bias from non-blinding to platform identity, the rubrics emphasized objective, verifiable clinical criteria.

Given the absence of established standards for evaluating AI-generated medical education simulations, we developed domain-specific pass-fail criteria informed by our rubric’s scoring definitions and simulation assessment literature. We employed a categorical approach requiring each subdomain to meet minimum performance thresholds rather than averaging scores across subdomains, thereby preventing high performance in one area from masking critical deficiencies in another.

We applied domain-specific pass-fail criteria. Clinical Accuracy and Safety required all four subdomains to achieve mean scores ≥4.0 with no exceptions permitted (non-compensatory scoring), consistent with the Healthcare Simulation Standards of Best Practice (HSSBP)^81^. Clinical reasoning fidelity similarly required all four subdomains ≥4.0 without compensation, reflecting the expectation that simulations model expert-level reasoning processes^91,92^. In contrast, instructional design quality used compensatory scoring based on Chen et al.’s^59^ (all subdomains >3.0 with overall domain mean >4.0), allowing strengths in some instructional elements to offset modest weaknesses in others while maintaining a minimum-quality floor^59,93^. Sessions were classified as successful only when all three domains met criteria simultaneously^94^. We report a structured error taxonomy (Table 1) distinguishing among guideline misalignment, pharmacotherapeutic inaccuracies, fabricated evidence citations, and domain entanglement.

### Statistical analysis

Our sample size was 80% powered to detect at least a 30-percentage-point difference in LLM performance between leukemia types at a two-sided α = 0.05^95^. The primary endpoint was the overall success rate, defined as the proportion of sessions meeting passing criteria across all three domains, reported with exact binomial 95% confidence intervals^95^. Domain-specific success rates were estimated overall and stratified by platform and leukemia type using Wilson score confidence intervals. Error frequencies were quantified by platform and disease type to identify systematic failure patterns.

Comparisons between AML and CML used Fisher’s exact tests with risk ratios and 95% confidence intervals. Chi-square or Fisher’s exact test was applied according to standard guidelines, with Fisher’s exact test selected when expected cell counts were less than five to ensure valid inference in small-sample categorical comparisons.

Platform-level differences in overall success rates were assessed using chi-square tests, and within-platform disease differences were evaluated using Fisher’s exact tests.

Student satisfaction was analyzed by comparing mean scores on each of the eight dimensions against the neutral midpoint (μ = 3.0) using one-sample *t*-tests, consistent with established practices for Likert scale analysis in educational research^96,97^. In addition, we examined whether student satisfaction varied according to expert-assessed content quality. For three conceptually related domain-dimension pairs (clinical accuracy and safety with clinical practice realism; clinical reasoning fidelity with both clinical confidence and diagnostic skills improvement), we calculated mean student dimension scores stratified by whether their corresponding sessions passed or failed expert evaluation criteria.

Statistical significance was defined as α = 0.05. Multiple comparisons were corrected using the Benjamini–Hochberg procedure^98^. All analyses were conducted in Python 3.12 (SciPy 1.11, Statsmodels 0.14).

## Supplementary information

Supplementary Information

## Data Availability

All datasets generated and/or analyzed during the current study are available in the supplementary material.
