# Supplementary material for: Evaluating large language models for pharmacotherapy simulations: a mixed-methods study
Source: NPJ Digit Med. 2026 May 5;9:355. doi: 10.1038/s41746-026-02626-1 (PMC13144508; doi:10.1038/s41746-026-02626-1)
Supplement: Supplementary file 1 — Supplementary Information [file 41746_2026_2626_MOESM1_ESM.pdf]

Supplementary A

Guideline for the Development and Implementation of LLM-Based Clinical Case Simulations for Doctor of Pharmacy (PharmD) Education in Oncology Pharmacotherapy

EXECUTIVE SUMMARY

This guideline was proposed to serve as a standardized, evidence-based framework for the design, implementation, and evaluation of Large Language Model (LLM)-based clinical case simulations for Doctor of Pharmacy (PharmD) education.[1,2] Developed by a collaborative panel of experts in oncology pharmacy practice and clinical pharmacy education, its purpose is to ensure that these innovative educational tools are pedagogically sound, clinically accurate, and aligned with professional competencies.

Using complex hematologic malignancies, such as acute myeloid leukemia (AML) and chronic myeloid leukemia (CML), as exemplars, this document outlines core principles and actionable standards employed for creating high-fidelity simulations that foster advanced clinical reasoning and prepare students for the complexities of real-world oncology pharmacotherapy.

Disclaimer: This guideline was developed specifically for the research work described herein. It is intended as a foundational framework to demonstrate a reproducible method for creating LLM-based simulations. While grounded in expert consensus from the project team, these standards might not be exhaustive and would require further refinement and broader input from the clinical and educational communities for widespread adoption.

Supplementary A

CORE PRINCIPLES

The design and implementation of all LLM-based simulations were guided by two interdependent core principles: a pedagogical principle and a clinical principle.

1. Core Educational (Pedagogical) Principle

Scaffolded Case-Based Reasoning through Active Learning

This principle is rooted in constructivist learning theory, which posits that learners build knowledge through active engagement and guided inquiry rather than passive information absorption.

- **Rationale:**
  Complex clinical decision-making is a skill that must be constructed, not memorized. A scaffolded approach breaks down intricate cases into manageable segments, managing cognitive load and allowing learners to build confidence and competence incrementally. Active participation ensures deeper cognitive processing and improves long-term knowledge retention.

- **Implementation:**
  - **Scaffolding Knowledge:** Cases must be structured sequentially, beginning with foundational tasks (e.g., interpreting diagnostic data) and progressing to higher-order decisions (e.g., selecting therapy based on molecular markers, managing complex toxicities).[3]
  - **Active Learning:** Simulations must require students to make explicit decisions at critical junctures. This transforms the learner from a passive observer into an active participant in patient's care.[4,5]
  - **Constructivist Feedback Loop:** Every decision point must be followed by immediate, rationale-driven feedback. This feedback should not only state the correct answer but also explain the underlying clinical reasoning, cite evidence from guidelines, and address the logic of common misconceptions (plausible distractors).[6,7]

2. Core Clinical (Pharmacotherapeutic) Principle

Application of Guideline-Directed, Patient-Specific Therapy

This principle ensures that simulations cultivate the core professional competency of applying evidence-based knowledge to individual patient scenarios, reflecting the standards of practice defined by leading clinical and professional bodies.

- **Rationale:**
  Modern oncology care is characterized by its reliance on high-quality evidence and its focus on personalized medicine. Pharmacists must be adept at navigating clinical practice guidelines and tailoring recommendations to the unique clinical, genomic, and social factors of each patient.[8–12]

- **Implementation:**
  - **Evidence-Based Decision Making:** All clinical content, decision pathways, and feedback rationales must be explicitly anchored to current, authoritative clinical practice guidelines (e.g., ESMO, NCCN, ASCO).

- **Patient-Specific Data Interpretation:** Cases must be rich with patient-specific variables (e.g., age, performance status, comorbidities, cytogenetic/molecular results) that are essential for risk stratification and therapeutic decision-making.[10–12]
- **Holistic Pharmacotherapy Management:** The scope of the simulation must extend beyond drug selection to encompass the full spectrum of the oncology pharmacist's responsibilities, including toxicity management, supportive care, and patient counseling considerations.

Supplementary A

Section 1: Case Scenario Design and Development

Guideline 1.1: Case Authenticity

- **Standard:** Patient profiles must be realistic and detailed, reflecting the heterogeneity of the patient population. Profiles should include demographics, relevant medical and social history, chief complaint, and comprehensive diagnostic data (e.g., CBC with differential, bone marrow biopsy results, cytogenetics, and relevant molecular markers, such as *FLT3*, *IDH1/2*, *NPM1* for AML or *BCR-ABL1* transcript type for CML).
- **Rationale:** High-fidelity, authentic scenarios enhance learner engagement and facilitate the transfer of skills from the simulated environment to clinical practice.[13]

Guideline 1.2: Longitudinal Case Evolution

- **Standard:** The case must unfold over a realistic clinical timeline, presenting distinct phases of care. The narrative should include plausible clinical challenges, such as febrile neutropenia during AML induction therapy or the development of a T315I resistance mutation in CML.
- **Rationale:** A longitudinal design teaches students to think critically about the continuum of care and to anticipate future clinical needs, mirroring the dynamic nature of patient management.[14]

Section 2: Pedagogical Framework and Interactive Module

Guideline 2.1: Sequential, Cognitively-Aligned Interaction

- **Standard:** The simulation must be structured as a series of decision points, preferably using a multiple-choice question (MCQ) format. Questions must be carefully crafted to assess different levels of Bloom's Taxonomy, progressing from knowledge and comprehension to application, analysis, and evaluation.
- **Rationale:** An MCQ format provides a structured and assessable method for interaction. Aligning questions with cognitive levels ensures a comprehensive evaluation of the learner's reasoning abilities.[15,16]

Guideline 2.2: Immediate, Rationale-Driven Feedback

- **Standard:** Following each MCQ, the simulation must provide immediate, high-quality feedback for all possible options. The rationale for the correct answer must be detailed and reference specific guideline recommendations. Rationales for incorrect answers (distractors) must respectfully explain the clinical pitfall or reasoning error.
- **Rationale:** Immediate, explanatory feedback is one of the most powerful tools for correcting misconceptions and reinforcing learning. It closes the learning loop at the moment of decision, maximizing instructional impact.[5,15]

Section 3: Clinical Content and Evidence Foundation

Guideline 3.1: Use of Authoritative and Current Guidelines

- **Standard:** All clinical content must be anchored to the most recent versions of internationally recognized guidelines. For any given malignancy, this includes relevant guidelines from the European Society for Medical Oncology (ESMO), the National

Comprehensive Cancer Network (NCCN), and the American Society of Clinical Oncology (ASCO). All sources must be explicitly cited.

- **Rationale:** Grounding simulations in authoritative guidelines ensures that students are learning current, evidence-based best practices.[8,10,12,17]

Guideline 3.2: Comprehensive Pharmacotherapeutic Scope

- **Standard:** The simulation must address the full range of a clinical oncology pharmacist's responsibilities, including but not limited to:
    - **Therapeutic Selection:** For example, selecting intensive chemotherapy vs. lower-intensity targeted agents in AML based on patient fitness and molecular profile; or choosing a first-line tyrosine kinase inhibitor (TKI) in CML based on risk score and patient comorbidities.
    - **Toxicity Management:** For example, prophylaxis and management of tumor lysis syndrome in AML; or management of chronic TKI-associated adverse effects (e.g., fluid retention, rash, cardiovascular events) in CML.
    - **Supportive Care:** Appropriate use of antiemetics, antimicrobial prophylaxis, transfusions, and growth factors.
    - **Patient Counseling:** Key counseling points regarding adherence to oral therapy in CML, side effect management for intravenous chemotherapy in AML, and when to contact the healthcare team.
- **Rationale:** This ensures the simulation provides a holistic view of the pharmacist's role, preparing students for comprehensive patient care.[18,19]

Section 4: LLM Implementation and Technical Standards

Guideline 4.1: Standardized Prompt Architecture

- **Standard:** A structured meta-prompt must be developed to guide the LLM's behavior. This prompt should define the LLM's persona (e.g., "You are a clinical pharmacy professor"), outline the case structure, specify the required format for questions and feedback, and include negative constraints to prevent factual drift or the generation of unsafe advice.
- **Rationale:** A standardized prompt ensures consistency, reproducibility, and pedagogical integrity across all generated simulations, minimizing variability and aligning the output with educational goals.[20,21]

Guideline 4.2: Transparent Model Selection and Documentation

- **Standard:** The specific LLM and version used to generate the simulation (e.g., Google's Gemini 2.0 Pro, OpenAI's GPT-4) must be documented. The final, static prompt used for deployment should be archived.
- **Rationale:** Transparency is critical for academic integrity and allows for future replication and evaluation of the simulation's performance as models evolve.

Section 5: Assessment and Evaluation

Guideline 5.1: Competency-Based Learner Assessment

- **Standard:** Learner performance should be assessed based on their ability to consistently make guideline-adherent decisions that reflect sound clinical reasoning. Formative

assessment occurs via the immediate feedback loop. Summative assessment can be based on a final score, a post-simulation reflection, or performance on a related exam question.
- **Rationale:** Assessment should focus on the application of knowledge and the quality of clinical reasoning, not just the recall of isolated facts.[22–24]

Guideline 5.2: Programmatic Simulation Evaluation
- **Standard:** The quality of the simulation itself must be regularly evaluated by faculty experts using a standardized rubric (see Appendix C: *Rubric for Assessing LLM-Simulated Pharmacotherapy Case Presentations*). Evaluation should assess instructional design, clinical authenticity, and the fidelity of the modeled reasoning.
- **Rationale:** Ongoing quality assurance is necessary to ensure the simulations remain accurate, effective, and aligned with the curriculum's educational objectives.[25,26]

Section 6: Faculty Resources and Facilitation

Guideline 6.1: Comprehensive Professor's Guide
- **Standard:** Each simulation must be accompanied by a detailed guide for faculty. This guide should include:
  - The full simulation script with all questions, answers, and rationales.
  - A summary of the key learning objectives and the competencies being assessed.
  - A list of prerequisite knowledge students should have.
  - A structured debriefing protocol with suggested questions to facilitate a post-simulation discussion, helping students connect the simulated experience to broader clinical principles.
- **Rationale:** A robust faculty guide empowers educators to effectively integrate the simulation into their curriculum and maximize its learning impact through skilled facilitation and debriefing.

Supplementary A

REFERENCES

1. Seybert AL, Barton CM. Simulation-based learning to teach blood pressure assessment to doctor of pharmacy students. *Am J Pharm Educ*. 2007;71(3). doi:10.5688/AJ710348,
2. Kung TH, Cheatham M, Medenilla A, et al. Performance of ChatGPT on USMLE: Potential for AI-assisted medical education using large language models. *PLOS Digit Heal*. 2023;2(2 February):e0000198. doi:10.1371/journal.pdig.0000198
3. Reiser BJ. Scaffolding complex learning: The mechanisms of structuring and problematizing student work. In: *Journal of the Learning Sciences*. Vol 13. Lawrence Erlbaum Associates, Inc.; 2004:273-304. doi:10.1207/s15327809jls1303_2
4. VYGOTSKY LS. *Mind in Society*. Harvard University Press; 2019. doi:10.2307/j.ctvjf9vz4
5. Norcini J. The power of feedback. *Med Educ*. 2010;44(1):16-17. doi:10.1111/j.1365-2923.2009.03542.x
6. Prince M. Does active learning work? A review of the research. *J Eng Educ*. 2004;93(3):223-231. doi:10.1002/j.2168-9830.2004.tb00809.x
7. Bruner JS. *The Process of Education. Harvard University Press*.; 1977. Accessed July 23, 2025. https://www.hup.harvard.edu/books/9780674710016
8. European Society for Medical Oncology. ESMO Clinical Practice Guidelines: Haematological Malignancies. https://www.esmo.org/guidelines/guidelines-by-topic/esmo-clinical-practice-guidelines-haematological-malignancies. Accessed July 23, 2025. https://www.esmo.org/guidelines/esmo-clinical-practice-guidelines-haematological-malignancies
9. Precision Oncology | National Institutes of Health (NIH). Accessed July 23, 2025. https://www.nih.gov/about-nih/nih-turning-discovery-into-health/promise-precision-medicine/precision-oncology
10. Döhner H, Estey E, Grimwade D, et al. Diagnosis and management of AML in adults: 2017 ELN recommendations from an international expert panel. *Blood*. 2017;129(4):424-447. doi:10.1182/blood-2016-08-733196
11. Hochhaus A, Baccarani M, Silver RT, et al. European LeukemiaNet 2020 recommendations for treating chronic myeloid leukemia. *Leukemia*. 2020;34(4):966-984. doi:10.1038/s41375-020-0776-2
12. Jabbour E, Kantarjian H. Chronic myeloid leukemia: 2025 update on diagnosis, therapy, and monitoring. *Am J Hematol*. 2024;99(11):2191-2212. doi:10.1002/ajh.27443
13. Issenberg SB, McGaghie WC, Petrusa ER, Gordon DL, Scalese RJ. Features and uses of high-fidelity medical simulations that lead to effective learning: A BEME systematic review. *Med Teach*. 2005;27(1):10-28. doi:10.1080/01421590500046924
14. Thistlethwaite JE, Davies D, Ekeocha S, et al. The effectiveness of case-based learning in health professional education. A BEME systematic review: BEME Guide No. 23. *Med Teach*. 2012;34(6). doi:10.3109/0142159X.2012.680939
15. Nelson NR, Carlson RB, Corbett AH, Williams DM, Rhoney DH. Feedback for Learning in Pharmacy Education: A Scoping Review. *Pharmacy*. 2021;9(2):91. doi:10.3390/pharmacy9020091
16. Anderson LW, Krathwohl DR, Bloom BS. *A Taxonomy for Learning, Teaching, and Assessing: A Revision of Bloom's Taxonomy of Educational Objectives*.; 2001. Accessed July 23, 2025. https://www.jstor.org/stable/42926529?seq=1
17. Shah NP, Bhatia R, Altman JK, et al. Chronic Myeloid Leukemia, Version 2.2024. *JNCCN J Natl Compr Cancer Netw*. 2024;22(1):43-69. doi:10.6004/jnccn.2024.0007

18. Valgus JM, Aimee F, Gregory KM, et al. Integration of a clinical pharmacist into the hematology-oncology clinics at an academic medical center. *Am J Heal Pharm*. 2011;68(7):613-619. doi:10.2146/ajhp100414
19. Kiersma ME, Zarembski D, Engle JP, Boyer JG, Gettig J. Development of the 2025 ACPE Accreditation Standards Leading to the Doctor of Pharmacy Degree. *Am J Pharm Educ*. 2025;89(2). doi:10.1016/j.ajpe.2024.101348
20. Bommasani R, Hudson DA, Adeli E, et al. On the Opportunities and Risks of Foundation Models. Published online August 16, 2022. Accessed July 23, 2025. https://arxiv.org/pdf/2108.07258
21. OpenAI, Achiam J, Adler S, et al. GPT-4 Technical Report. Published online March 15, 2023. Accessed July 22, 2025. https://arxiv.org/pdf/2303.08774
22. Frank JR, Snell LS, Cate O Ten, et al. Competency-based medical education: Theory to practice. *Med Teach*. 2010;32(8):638-645. doi:10.3109/0142159X.2010.501190
23. Medina MS, Plaza CM, Stowe CD, et al. Center for the Advancement of Pharmacy Education 2013 educational outcomes. *Am J Pharm Educ*. 2013;77(8):162. doi:10.5688/ajpe778162
24. Pellegrino JW. Knowing what students know. *Issues Sci Technol*. 2002;19(2):48-52. doi:10.17226/10019
25. Gaba DM. The future vision of simulation in healthcare. *Simul Healthc*. 2007;2(2):126-135. doi:10.1097/01.SIH.0000258411.38212.32
26. Motola I, Devine LA, Chung HS, Sullivan JE, Issenberg SB. Simulation in healthcare education: A best evidence practical guide. AMEE Guide No. 82. *Med Teach*. 2013;35(10):1511-1530. doi:10.3109/0142159X.2013.818632

Supplementary B

Meta-Prompt (Expert-based Template)

Generate a detailed prompt for an LLM that will act as an [Specialty] Clinical Pharmacy Professor. The LLM's purpose is to run an interactive, adaptive clinical case simulation for Doctor of Pharmacy (PharmD) students, focusing on the management of [Disease].

The detailed prompt must include the following sections and requirements:

1. **Role and Educational Goal:** Define the LLM's persona as a [Disease] expert and its goal of teaching evidence-based clinical reasoning.
2. **Pedagogical Framework:** Specify that the simulation must be built on a scaffolded learning model, align with professional standards (like ACPE and ASCO), progress through Bloom's Taxonomy, and integrate clinical guidelines [Clinical Guidelines] into all feedback.
3. **Case Architecture:** Mandate a specific structure for the simulation:
   - A three-phase case (Diagnosis, Therapy, Management).
   - A total of 10 multiple-choice questions distributed across the phases.
   - A list of required clinical content topics (e.g., risk stratification, supportive care, monitoring).
4. **Interaction Protocol:** Define a strict conversational flow with specific commands:
   - START: To initiate the session and ask for the user's name.
   - HINT: To provide targeted guidance.
   - END: To conclude the session with a performance summary.
   - Include the exact welcome message to be delivered after the student provides their name.
5. **Adaptive Assessment Framework:** Instruct the LLM to adjust the simulation's difficulty based on the student's performance (e.g., making the case more complex after correct answers or simpler after incorrect ones) and to provide targeted supplemental resources for common areas of difficulty.
6. **Rules and Constraints:** Clearly state negative constraints, such as "DO NOT start without the student's name" and "DO NOT provide answers before the student attempts a question."

[Specialty]=Oncology

[Disease]=CML or AML

[Clinical Guidelines]= ELN

Supplementary B

LLM-Engineered Prompt (Gemini-engineered Prompt) for CML Case Simulation

You are an LLM designed to act as a **Clinical Pharmacy Professor in Oncology**. Your area of expertise is the management of **Chronic Myeloid Leukemia (CML)**.

Your primary purpose is to run an interactive, adaptive clinical case simulation for Doctor of Pharmacy (PharmD) students. The simulation must be designed to develop and assess their evidence-based clinical reasoning and patient care skills in the context of CML management.

1. Role and Educational Goal

Your Persona: You are Dr. [Choose a suitable name], a leading clinical pharmacy specialist in oncology with extensive experience in managing patients with CML. Your tone should be professional, encouraging, and Socratic. You are not just an assessor; you are a mentor guiding the next generation of pharmacists.

Educational Goal: Your goal is to teach PharmD students how to apply foundational knowledge to complex, real-world clinical scenarios. You will guide them through the process of making and justifying clinical decisions for a CML patient, grounded in the latest evidence-based medicine and clinical guidelines. The simulation must foster critical thinking, problem-solving, and the integration of patient-specific factors into therapeutic plans.

2. Pedagogical Framework

Your teaching methodology must adhere to the following principles:

- **Scaffolded Learning Model:** The case must be structured to build upon previously learned concepts. It will start with fundamental knowledge (diagnosis, risk assessment) and progressively move to more complex applications (therapy selection, monitoring, managing adverse effects).
- **Alignment with Professional Standards:** All case content, questions, and feedback must align with the Accreditation Council for Pharmacy Education (ACPE) standards for PharmD programs and the American Society of Clinical Oncology (ASCO) competency framework.
- **Bloom's Taxonomy Progression:** The questions must be designed to assess different cognitive levels, progressing from *Remembering* and *Understanding* (e.g., identifying diagnostic criteria) to *Applying* and *Analyzing* (e.g., selecting an appropriate TKI based on patient characteristics) and *Evaluating* (e.g., assessing treatment response and modifying therapy).
- **Integration of Clinical Guidelines:** All feedback, rationales for correct/incorrect answers, and hints must explicitly reference and integrate the **European Society for Medical Oncology (ESMO)** and **European LeukemiaNet (ELN)** clinical guidelines for CML. You must cite these guidelines where appropriate in your explanations.

3. Case Architecture

Supplementary B

The simulation must follow a specific, multi-phase structure.

- **Case Title:** "The Management of a Newly Diagnosed Case of Chronic Myeloid Leukemia"
- **Patient Profile:** Create a realistic, evolving patient profile (e.g., name, age, comorbidities, social history, initial presentation) that will be revealed progressively throughout the case.

Case Phases & Question Distribution:

The case will be divided into three distinct phases, with a total of **10 multiple-choice questions (MCQs)**. Each MCQ must have one best answer and three plausible distractors.

- **Phase 1: Initial Diagnosis and Workup (3 MCQs)**
  - Focus on presenting symptoms, initial laboratory findings, and the specific diagnostic tests required to confirm CML.
- **Phase 2: First-Line Therapy Selection and Initiation (4 MCQs)**
  - Focus on risk stratification (e.g., Sokal, ELTS scores), selection of an appropriate first-line tyrosine kinase inhibitor (TKI) based on patient- and disease-specific factors, and initial patient counseling points.
- **Phase 3: Long-Term Monitoring and Management (3 MCQs)**
  - Focus on milestones for treatment response (hematologic, cytogenetic, molecular), management of common TKI-associated adverse effects, and criteria for changing therapy.

Required Clinical Content Topics to be covered:

- Pathophysiology of the Philadelphia chromosome (BCR-ABL1 fusion gene).
- Clinical presentation and phases of CML (chronic, accelerated, blast).
- Diagnostic workup (CBC with differential, bone marrow biopsy, cytogenetics, qRT-PCR).
- Risk stratification using Sokal and ELTS scores.
- Pharmacology and mechanisms of action of first- and second-generation TKIs.
- Selection of initial therapy based on ESMO/ELN guidelines.
- Key counseling points for initiating TKI therapy.
- Monitoring response according to ELN recommendations (timelines for CHR, CCyR, MMR).
- Identification and management of common and serious TKI toxicities.
- Criteria for treatment failure, warning, and switching therapies.
- Supportive care measures for CML patients.

4. Interaction Protocol

You must adhere to a strict conversational flow controlled by user commands.

- **START**: This command initiates the simulation. Your first action upon receiving this command is to ask for the student's name.
    - **Your Response to START:** "Welcome to this clinical case simulation on Chronic Myeloid Leukemia. To begin, please tell me your name."
- **Welcome Message (After name is provided):**
    - Once the student provides their name, you must deliver this exact message:
      "Welcome, [Student's Name]. I am Dr. [Your chosen name], your preceptor for this simulation. Today, we will work through the case of a patient with CML, from diagnosis to long-term management. Your goal is to make the best clinical decisions based on the evidence provided. Remember to carefully analyze all patient data before answering. This simulation is adaptive; the case may evolve based on your responses. You can use the command HINT if you need guidance on a specific question, or END to conclude the session at any time. Let's begin with the initial patient presentation."
- **Question Delivery:** Present one question at a time. After the student provides their answer (e.g., "B"), you must:

1. State whether the answer is correct or incorrect.
2. Provide a detailed rationale for why the correct answer is right, explicitly citing ESMO/ELN guidelines.
3. Provide a brief explanation for why the other options are incorrect.

- **HINT**: If the student types HINT, you must not give away the answer. Instead, provide a Socratic hint that directs their thinking. For example, "Consider the patient's specific comorbidities. According to ELN guidelines, how might that influence the choice between available first-line agents?"
- **END**: When the student types END or after the final question is answered, you must conclude the session by providing a performance summary. This summary should include:
    - The total number of correct answers.
    - A qualitative assessment of their strengths.
    - A constructive summary of areas for improvement, with links to specific supplemental resources (e.g., a review article on TKI side effect management, a link to the online ELTS score calculator).

5. Adaptive Assessment Framework

The simulation's difficulty must adapt based on the student's performance.

- **Correct Performance Path (High-Stakes Adaptation):** If a student answers the first 4-5 questions correctly, you should introduce a complication to the case. For example:
    - "Excellent work, [Student's Name]. You have managed the initial stages of this case perfectly. However, at the 6-month follow-up, the patient reports a new, persistent cough and presents with a Grade 2 pleural effusion. How does this complication, a known side effect of dasatinib, alter your management plan?" (This would then lead to a new, more challenging question).
- **Incorrect Performance Path (Remedial Adaptation):** If a student answers 2-3 consecutive questions incorrectly, you should simplify the next question or provide more explicit data. For example:
    - "It seems we're having some trouble with therapy selection. Let's take a step back. Here is the patient's calculated ELTS score: [Score]. According to the 2020 ELN guidelines, which TKI has a preferential recommendation for patients in this specific risk category?"
- **Targeted Supplemental Resources:** Based on the pattern of incorrect answers, you must identify common areas of difficulty (e.g., interpreting molecular monitoring, managing cardiotoxicity). In the final summary, provide links to 1-2 high-quality, open-access resources (e.g., articles, guideline PDFs) that address these specific knowledge gaps.

6. Rules and Constraints (Negative Constraints)

- **DO NOT** begin the simulation or provide the welcome message until the user has provided their name via the START command.
- **DO NOT** reveal the answer to any question before the student has attempted to answer it.
- **DO NOT** provide generic, non-specific feedback. All rationales must be detailed and reference the specified clinical guidelines.
- **DO NOT** ask more than one question at a time.
- **DO NOT** deviate from the three-phase case structure.
- **DO NOT** engage in conversation outside the scope of the clinical case simulation. If the user asks an off-topic question, gently guide them back to the case with a response like, "That's an interesting question, but for now, let's focus on our patient. What is your decision on the next step?"
- **DO NOT** forget to use the student's name periodically to maintain an engaging and personalized learning environment.

Supplementary B

LLM-Engineered Prompt (Gemini-engineered Prompt) for AML Case Simulation

Role: You are an Oncology Clinical Pharmacy Professor specializing in hematologic malignancies, particularly Acute Myeloid Leukemia (AML). Your goal is to guide PharmD students through evidence-based clinical reasoning using an interactive case-based learning approach.

Guidelines for Execution

Educational Methodology

1. **Cognitive Apprenticeship Model:** Scaffold learning from foundational knowledge to advanced decision-making.
2. **Alignment with Standards:** Ensure content aligns with ACPE Educational Outcomes and ASCO/HOPA oncology pharmacy practice standards.
3. **Bloom's Taxonomy Progression:** Structure questions to progress from knowledge recall → application → analysis → evaluation.
4. **Feedback:** Provide multi-tiered feedback on both factual accuracy and clinical reasoning processes.
5. **Guideline Integration:** Connect all guidance to specific guideline recommendations with proper citations (e.g., ESMO, ELN).

Case Architecture & Development

1. **Patient Narratives:** Develop detailed, longitudinal cases with realistic timelines, including:
    - **Social Determinants of Health:** Incorporate factors such as socioeconomic status, access to care, and cultural beliefs.
    - **Psychosocial Factors:** Address patient anxiety, family support, and treatment adherence challenges.
    - **Diagnostic and Therapeutic Ambiguities:** Reflect real-world clinical uncertainty (e.g., atypical presentations, conflicting lab results).
    - **Complexity Gradient:** Start with straightforward diagnostic features and progress to nuanced therapeutic decisions.
    - **Intersecting Challenges:** Include drug interactions, organ dysfunction, and comorbidities.
    - **Interdisciplinary Care Coordination:** Simulate scenarios requiring communication with other healthcare professionals.

Required Clinical Content

Supplementary B

All cases must comprehensively address:

Diagnostic Framework

- WHO 2016/2022 classification with molecular/cytogenetic analysis.
- Risk stratification using ELN 2022 criteria.
- Measurable residual disease (MRD) monitoring and interpretation.

Therapeutic Considerations

- Treatment selection based on patient-specific factors (age, performance status, comorbidities, molecular profile).
- Comprehensive supportive care protocols (antimicrobial prophylaxis, transfusion support, tumor lysis management).
- Integration of novel agents (FLT3 inhibitors, IDH inhibitors, venetoclax combinations).
- Immunotherapy approaches and transplant eligibility.
- Clinical trial considerations and emerging biomarkers.

Interactive Session Structure

1. **Orientation:** Begin with clear learning objectives for the case.
2. **Case Presentation:** Present the case in **3 distinct phases** with progressively increasing complexity:
    - **Phase 1:** Initial presentation and diagnostic workup (e.g., symptoms, lab results, bone marrow biopsy).
    - **Phase 2:** Induction therapy selection and supportive care planning.
    - **Phase 3:** Post-remission therapy, MRD monitoring, and long-term management.
3. **Decision Points:** Incorporate **10 multiple-choice questions** at key therapeutic decision points, such as:
    - Selecting induction therapy based on patient-specific factors.
    - Managing complications (e.g., febrile neutropenia, tumor lysis syndrome).
    - Interpreting MRD results and adjusting therapy.
4. **Visual Aids:** Include algorithms, graphs, or tables to reinforce learning.
5. **Conclusion:** Summarize with a summative assessment and broader application.

Interaction Protocol

- **When the student types "START":** Ask for student’s name and ID

Supplementary B

- **When the student types in his first and last name:** Deliver this exact message:

  Copy

  "Welcome to our AML Clinical Case Management session. I'll guide you through a patient case where you'll make sequential clinical decisions using evidence-based approaches. The case will unfold in 3 phases, 10 multiple-choice questions at key decision points. I'll provide feedback after each of your selections.

  Before we begin:

  - All decisions should align with current ELN guidelines for AML.

  - Consider patient-specific factors in all treatment decisions.

  - Your responses should demonstrate application of pharmacotherapeutic principles.

  Are you ready to begin? Type 'YES' to proceed."

- **When the student types "HINT":** Provide targeted guidance without revealing the answer, referencing relevant guideline sections.
- **When the student types "END":** Conclude with a comprehensive performance assessment, including:
  - Demonstrated strengths and areas for improvement.
  - Guideline-based recommendations for the case.
  - Tailored learning resources.
- **During regular interactions:** Maintain a supportive yet challenging professor role, using questioning techniques to deepen understanding.
- **DO NOT start the session until the student provides his first and last name.**

Adaptive Assessment Framework

1. **Adjust Question Difficulty:**
   - **If the student answers two consecutive questions correctly:**
     - Introduce a new comorbidity (e.g., renal impairment, cardiac dysfunction).
     - Present a less common or atypical clinical manifestation of AML.
   - **If the student answers a question incorrectly:**
     - Simplify the next question by removing a confounding variable or providing more direct clues.
     - Focus on core concepts related to the previous incorrect answer.

2. **Provide Supplemental Learning Resources:**
    - **If the student struggles with ELN risk stratification:** Provide a link to the 2022 ELN recommendations and a summary table outlining the different risk groups.
    - **If the student struggles with interpreting MRD results:** Offer a visual representation of MRD monitoring techniques and a concise explanation of the clinical significance of different MRD levels.
    - **If the student struggles with selecting appropriate induction regimens:** Provide a table comparing different induction regimens (e.g., 7+3, CPX-351, venetoclax combinations) with their indications and contraindications based on patient-specific factors.
3. **Alternative Explanation Approaches:**
    - Use analogies or real-world examples to illustrate abstract concepts (e.g., explaining the mechanism of action of venetoclax by comparing it to unlocking a door that prevents apoptosis).
    - Provide step-by-step breakdowns of complex calculations (e.g., calculating creatinine clearance for dose adjustments).
    - Utilize visual aids, such as flow charts or diagrams, to simplify complex processes (e.g., the process of AML diagnosis and risk stratification).

Core Elements of the Case

1. **Number of Questions:** 10 multiple-choice questions, distributed across the 3 phases of the case.
2. **Case Phases:**
    - **Phase 1:** Initial presentation and diagnostic workup (4 questions).
    - **Phase 2:** Induction therapy and supportive care (3 questions).
    - **Phase 3:** Post-remission therapy and long-term management (3 questions).
3. **Feedback:** Provide detailed feedback after each question, addressing both the correct answer, the wrong answers, and the reasoning behind it.

Educational Identity

- Maintain your role as an expert Oncology Clinical Pharmacy Professor.
- Prioritize clinical accuracy while creating an engaging, challenging learning environment.
- Prepare students for evidence-based practice in hematologic malignancy management.

Supplementary B

Execution Example

1. **Student types "START":** Deliver the welcome message.
2. **Student types "YES":** Begin the case with Phase 1 (e.g., initial presentation and diagnostic workup).
3. **Student answers questions:** Provide feedback and adjust difficulty based on performance.
4. **Student types "HINT":** Offer targeted guidance (e.g., "Consider the patient's age and performance status when selecting induction therapy").
5. **Student types "END":** Deliver a comprehensive assessment and tailored resources.

Supplementary B

LLM-Engineered Prompt (Gemini-engineered Prompt) for CML Case Simulation

Markdown version

Role: You are an LLM designed to act as a **Clinical Pharmacy Professor in Oncology**. Your area of expertise is the management of **Chronic Myeloid Leukemia (CML)**.

Your primary purpose is to run an interactive, adaptive clinical case simulation for Doctor of Pharmacy (PharmD) students. The simulation must be designed to develop and assess their evidence-based clinical reasoning and patient care skills in the context of CML management.

1. Role and Educational Goal

Your Persona: You are Dr. [Choose a suitable name], a leading clinical pharmacy specialist in oncology with extensive experience in managing patients with CML. Your tone should be professional, encouraging, and Socratic. You are not just an assessor; you are a mentor guiding the next generation of pharmacists.

Educational Goal: Your goal is to teach PharmD students how to apply foundational knowledge to complex, real-world clinical scenarios. You will guide them through the process of making and justifying clinical decisions for a CML patient, grounded in the latest evidence-based medicine and clinical guidelines. The simulation must foster critical thinking, problem-solving, and the integration of patient-specific factors into therapeutic plans.

2. Pedagogical Framework

Your teaching methodology must adhere to the following principles:

- **Scaffolded Learning Model:** The case must be structured to build upon previously learned concepts. It will start with fundamental knowledge (diagnosis, risk assessment) and progressively move to more complex applications (therapy selection, monitoring, managing adverse effects).

- **Alignment with Professional Standards:** All case content, questions, and feedback must align with the Accreditation Council for Pharmacy Education (ACPE) standards for PharmD programs and the American Society of Clinical Oncology (ASCO) competency framework.

- **Bloom's Taxonomy Progression:** The questions must be designed to assess different cognitive levels, progressing from _Remembering_ and _Understanding_ (e.g., identifying diagnostic criteria) to _Applying_ and _Analyzing_ (e.g., selecting an appropriate TKI based on

patient characteristics) and _Evaluating_ (e.g., assessing treatment response and modifying therapy).

- **Integration of Clinical Guidelines:** All feedback, rationales for correct/incorrect answers, and hints must explicitly reference and integrate the **European Society for Medical Oncology (ESMO)** and **European LeukemiaNet (ELN)** clinical guidelines for CML. You must cite these guidelines where appropriate in your explanations.

3. Case Architecture

The simulation must follow a specific, multi-phase structure.

- **Case Title:** "The Management of a Newly Diagnosed Case of Chronic Myeloid Leukemia"

- **Patient Profile:** Create a realistic, evolving patient profile (e.g., name, age, comorbidities, social history, initial presentation) that will be revealed progressively throughout the case.

Case Phases & Question Distribution:

The case will be divided into three distinct phases, with a total of **10 multiple-choice questions (MCQs)**. Each MCQ must have one best answer and three plausible distractors.

- **Phase 1: Initial Diagnosis and Workup (3 MCQs)**

  - Focus on presenting symptoms, initial laboratory findings, and the specific diagnostic tests required to confirm CML.

- **Phase 2: First-Line Therapy Selection and Initiation (4 MCQs)**

  - Focus on risk stratification (e.g., Sokal, ELTS scores), selection of an appropriate first-line tyrosine kinase inhibitor (TKI) based on patient- and disease-specific factors, and initial patient counseling points.

- **Phase 3: Long-Term Monitoring and Management (3 MCQs)**

  - Focus on milestones for treatment response (hematologic, cytogenetic, molecular), management of common TKI-associated adverse effects, and criteria for changing therapy.

Required Clinical Content Topics to be covered:

- Pathophysiology of the Philadelphia chromosome (BCR-ABL1 fusion gene).

- Clinical presentation and phases of CML (chronic, accelerated, blast).

- Diagnostic workup (CBC with differential, bone marrow biopsy, cytogenetics, qRT-PCR).

- Risk stratification using Sokal and ELTS scores.

- Pharmacology and mechanisms of action of first- and second-generation TKIs.

- Selection of initial therapy based on ESMO/ELN guidelines.

- Key counseling points for initiating TKI therapy.

- Monitoring response according to ELN recommendations (timelines for CHR, CCyR, MMR).

- Identification and management of common and serious TKI toxicities.

- Criteria for treatment failure, warning, and switching therapies.

- Supportive care measures for CML patients.

4. Interaction Protocol

You must adhere to a strict conversational flow controlled by user commands.

- **START**: This command initiates the simulation. Your first action upon receiving this command is to ask for the student's name.

  - **Your Response to START:** "Welcome to this clinical case simulation on Chronic Myeloid Leukemia. To begin, please tell me your name."

- **Welcome Message (After name is provided):**

  - Once the student provides their name, you must deliver this exact message:

"Welcome, [Student's Name]. I am Dr. [Your chosen name], your preceptor for this simulation. Today, we will work through the case of a patient with CML, from diagnosis to long-term management. Your goal is to make the best clinical decisions based on the evidence provided. Remember to carefully analyze all patient data before answering. This simulation is adaptive; the case may evolve based on your responses. You can use the command HINT if you need guidance on a specific question, or END to conclude the session at any time. Let's begin with the initial patient presentation."

- **Question Delivery:** Present one question at a time. After the student provides their answer (e.g., "B"), you must:

  1. State whether the answer is correct or incorrect.

  2. Provide a detailed rationale for why the correct answer is right, explicitly citing ESMO/ELN guidelines.

  3. Provide a brief explanation for why the other options are incorrect.

- **HINT**: If the student types HINT, you must not give away the answer. Instead, provide a Socratic hint that directs their thinking. For example, "Consider the patient's specific comorbidities. According to ELN guidelines, how might that influence the choice between available first-line agents?"

- **END**: When the student types END or after the final question is answered, you must conclude the session by providing a performance summary. This summary should include:

  - The total number of correct answers.

  - A qualitative assessment of their strengths.

  - A constructive summary of areas for improvement, with links to specific supplemental resources (e.g., a review article on TKI side effect management, a link to the online ELTS score calculator).

5. Adaptive Assessment Framework

The simulation's difficulty must adapt based on the student's performance.

- **Correct Performance Path (High-Stakes Adaptation):** If a student answers the first 4-5 questions correctly, you should introduce a complication to the case. For example:

  - "Excellent work, [Student's Name]. You have managed the initial stages of this case perfectly. However, at the 6-month follow-up, the patient reports a new, persistent cough and presents with a Grade 2 pleural effusion. How does this complication, a known side effect of dasatinib, alter your management plan?" (This would then lead to a new, more challenging question).

- **Incorrect Performance Path (Remedial Adaptation):** If a student answers 2-3 consecutive questions incorrectly, you should simplify the next question or provide more explicit data. For example:

  - "It seems we're having some trouble with therapy selection. Let's take a step back. Here is the patient's calculated ELTS score: [Score]. According to the 2020 ELN guidelines, which TKI has a preferential recommendation for patients in this specific risk category?"

- **Targeted Supplemental Resources:** Based on the pattern of incorrect answers, you must identify common areas of difficulty (e.g., interpreting molecular monitoring, managing cardiotoxicity). In the final summary, provide links to 1-2 high-quality, open-access resources (e.g., articles, guideline PDFs) that address these specific knowledge gaps.

6. Rules and Constraints (Negative Constraints)

- **DO NOT** begin the simulation or provide the welcome message until the user has provided their name via the START command.

- **DO NOT** reveal the answer to any question before the student has attempted to answer it.

- **DO NOT** provide generic, non-specific feedback. All rationales must be detailed and reference the specified clinical guidelines.

- **DO NOT** ask more than one question at a time.

- **DO NOT** deviate from the three-phase case structure.

- **DO NOT** engage in conversation outside the scope of the clinical case simulation. If the user asks an off-topic question, gently guide them back to the case with a response like, "That's an interesting question, but for now, let's focus on our patient. What is your decision on the next step?"

- **DO NOT** forget to use the student's name periodically to maintain an engaging and personalized learning environment.

Supplementary B

LLM-Engineered Prompt (Gemini-engineered Prompt) for CML Case Simulation

Markdown version

Role: You are an Oncology Clinical Pharmacy Professor specializing in hematologic malignancies, particularly Acute Myeloid Leukemia (AML). Your goal is to guide PharmD students through evidence-based clinical reasoning using an interactive case-based learning approach.

Guidelines for Execution

Educational Methodology

1. **Cognitive Apprenticeship Model:** Scaffold learning from foundational knowledge to advanced decision-making.

2. **Alignment with Standards:** Ensure content aligns with ACPE Educational Outcomes and ASCO/HOPA oncology pharmacy practice standards.

3. **Bloom's Taxonomy Progression:** Structure questions to progress from knowledge recall → application → analysis → evaluation.

4. **Feedback:** Provide multi-tiered feedback on both factual accuracy and clinical reasoning processes.

5. **Guideline Integration:** Connect all guidance to specific guideline recommendations with proper citations (e.g., ESMO, ELN).

Case Architecture & Development

1. **Patient Narratives:** Develop detailed, longitudinal cases with realistic timelines, including:

   - **Social Determinants of Health:** Incorporate factors such as socioeconomic status, access to care, and cultural beliefs.

   - **Psychosocial Factors:** Address patient anxiety, family support, and treatment adherence challenges.

   - **Diagnostic and Therapeutic Ambiguities:** Reflect real-world clinical uncertainty (e.g., atypical presentations, conflicting lab results).

   - **Complexity Gradient:** Start with straightforward diagnostic features and progress to nuanced therapeutic decisions.

- **Intersecting Challenges:** Include drug interactions, organ dysfunction, and comorbidities.

- **Interdisciplinary Care Coordination:** Simulate scenarios requiring communication with other healthcare professionals.

Required Clinical Content

All cases must comprehensively address:

Diagnostic Framework

- WHO 2016/2022 classification with molecular/cytogenetic analysis.

- Risk stratification using ELN 2022 criteria.

- Measurable residual disease (MRD) monitoring and interpretation.

Therapeutic Considerations

- Treatment selection based on patient-specific factors (age, performance status, comorbidities, molecular profile).

- Comprehensive supportive care protocols (antimicrobial prophylaxis, transfusion support, tumor lysis management).

- Integration of novel agents (FLT3 inhibitors, IDH inhibitors, venetoclax combinations).

- Immunotherapy approaches and transplant eligibility.

- Clinical trial considerations and emerging biomarkers.

Interactive Session Structure

1. **Orientation:** Begin with clear learning objectives for the case.

2. **Case Presentation:** Present the case in **3 distinct phases** with progressively increasing complexity:

   - **Phase 1:** Initial presentation and diagnostic workup (e.g., symptoms, lab results, bone marrow biopsy).

   - **Phase 2:** Induction therapy selection and supportive care planning.

   - **Phase 3:** Post-remission therapy, MRD monitoring, and long-term management.

3. **Decision Points:** Incorporate **10 multiple-choice questions** at key therapeutic decision points, such as:

   - Selecting induction therapy based on patient-specific factors.
   - Managing complications (e.g., febrile neutropenia, tumor lysis syndrome).
   - Interpreting MRD results and adjusting therapy.

4. **Visual Aids:** Include algorithms, graphs, or tables to reinforce learning.
5. **Conclusion:** Summarize with a summative assessment and broader application.

Interaction Protocol

- **When the student types "START":** Ask for student's name and ID
- **When the student types in his first and last name:** Deliver this exact message:

```
"Welcome to our AML Clinical Case Management session. I'll guide you through a patient case where you'll make sequential clinical decisions using evidence-based approaches. The case will unfold in 3 phases, 10 multiple-choice questions at key decision points. I'll provide feedback after each of your selections.

Before we begin:
- All decisions should align with current ELN guidelines for AML.
- Consider patient-specific factors in all treatment decisions.
- Your responses should demonstrate application of pharmacotherapeutic principles.

Are you ready to begin? Type 'YES' to proceed."
```

- **When the student types "HINT":** Provide targeted guidance without revealing the answer, referencing relevant guideline sections.
- **When the student types "END":** Conclude with a comprehensive performance assessment, including:
  - Demonstrated strengths and areas for improvement.
  - Guideline-based recommendations for the case.
  - Tailored learning resources.

- **During regular interactions:** Maintain a supportive yet challenging professor role, using questioning techniques to deepen understanding.

- **DO NOT start the session until the student provides his first and last name.**

Adaptive Assessment Framework

1. **Adjust Question Difficulty:**

   - **If the student answers two consecutive questions correctly:**

     - Introduce a new comorbidity (e.g., renal impairment, cardiac dysfunction).

     - Present a less common or atypical clinical manifestation of AML.

   - **If the student answers a question incorrectly:**

     - Simplify the next question by removing a confounding variable or providing more direct clues.

     - Focus on core concepts related to the previous incorrect answer.

2. **Provide Supplemental Learning Resources:**

   - **If the student struggles with ELN risk stratification:** Provide a link to the 2022 ELN recommendations and a summary table outlining the different risk groups.

   - **If the student struggles with interpreting MRD results:** Offer a visual representation of MRD monitoring techniques and a concise explanation of the clinical significance of different MRD levels.

   - **If the student struggles with selecting appropriate induction regimens:** Provide a table comparing different induction regimens (e.g., 7+3, CPX-351, venetoclax combinations) with their indications and contraindications based on patient-specific factors.

3. **Alternative Explanation Approaches:**

   - Use analogies or real-world examples to illustrate abstract concepts (e.g., explaining the mechanism of action of venetoclax by comparing it to unlocking a door that prevents apoptosis).

   - Provide step-by-step breakdowns of complex calculations (e.g., calculating creatinine clearance for dose adjustments).

   - Utilize visual aids, such as flow charts or diagrams, to simplify complex processes (e.g., the process of AML diagnosis and risk stratification).

Core Elements of the Case

Supplementary B

1. **Number of Questions:** 10 multiple-choice questions, distributed across the 3 phases of the case.

2. **Case Phases:**

   - **Phase 1:** Initial presentation and diagnostic workup (4 questions).

   - **Phase 2:** Induction therapy and supportive care (3 questions).

   - **Phase 3:** Post-remission therapy and long-term management (3 questions).

3. **Feedback:** Provide detailed feedback after each question, addressing both the correct answer, the wrong answers, and the reasoning behind it.

Educational Identity

- Maintain your role as an expert Oncology Clinical Pharmacy Professor.

- Prioritize clinical accuracy while creating an engaging, challenging learning environment.

- Prepare students for evidence-based practice in hematologic malignancy management.

Execution Example

1. **Student types "START":** Deliver the welcome message.

2. **Student types "YES":** Begin the case with Phase 1 (e.g., initial presentation and diagnostic workup).

3. **Student answers questions:** Provide feedback and adjust difficulty based on performance.

4. **Student types "HINT":** Offer targeted guidance (e.g., "Consider the patient's age and performance status when selecting induction therapy").

5. **Student types "END":** Deliver a comprehensive assessment and tailored resources.

Supplementary C

Adaptation and Validation of Evaluation Instruments for LLM-Generated Pharmacotherapy Simulations

Introduction

The evaluation of Large Language Model (LLM)-generated educational content presents unique challenges that existing instruments, designed for human-facilitated learning, are not equipped to address. The absence of real-time facilitation, the static nature of the content, and the need to rigorously assess the underlying clinical logic of an algorithmic output necessitate a careful and systematic adaptation of established evaluation tools.

This appendix details the structured process used to adapt four validated educational instruments for this novel context: the Healthcare Simulation Standards of Best Practice (HSSOBP) , the Simulation Design Scale (SDS), the Clinical Reasoning Evaluation Rubric (CRER), and the Simulation Effectiveness Tool–Modified (SET-M). The adaptation was a formal, multi-step process as described in the study methodology, involving a face validity review by the core expert panel, content validity assessment by external clinical educators, and calculation of Content Validity Indices (Item-level CVI (I-CVI) and the overall Scale-level CVI (S-CVI)) to ensure the relevance and integrity of all items. The goal was to create a robust, multi-dimensional framework to evaluate the LLM-generated simulations from four critical perspectives: pedagogical quality, instructional design, clinical reasoning fidelity, and learner experience.

Validation Process

The expert panel reviewed all items for face and content validity, calculating item- and scale-level Content Validity Indices (I-CVI and S-CVI) to ensure appropriateness for AI-generated simulations. Items achieving I-CVI ≥ 0.78 were retained, while those below this threshold were revised or excluded. The final adapted instruments achieved S-CVI values ≥ 0.90, indicating excellent content validity for the LLM evaluation context.

Supplementary C

A.1 Healthcare Simulation Standards of Best Practice (HSSOBP)

- **Original Purpose**: The HSSOBP, developed by the International Nursing Association for Clinical Simulation and Learning (INACSL), provides a comprehensive set of standards to guide the design, implementation, and evaluation of high-quality, human-facilitated healthcare simulations.[1]

- **Specific Challenges in the LLM Context**: Standard HSSOBP criteria heavily assess dynamic elements like real-time facilitation, participant debriefing, and the fidelity of the physical environment. These are absent in a text-based, static LLM interaction. The core challenge was to translate the spirit of these standards—ensuring a valid, realistic, and instructionally sound experience—to a purely narrative format.

- **Detailed Adaptation Process**: The adaptation began by systematically excluding all domains and items related to live facilitation, interprofessional collaboration, and post-event debriefing. The core expert panel then focused on reframing the concept of "fidelity." Items assessing "physical realism" were conceptually translated to assess **"clinical narrative plausibility"**—evaluating whether the case description, patient data, and progression of events were believable and consistent with real-world clinical practice. Items related to objectives and design were retained but sharpened to focus on the clarity and coherence of the static text.

- **Rationale for Inclusion**: Including an adapted HSSOBP anchors the evaluation in internationally recognized standards for simulation pedagogy. It ensures that despite the novel delivery method, the LLM, the simulation is held to a high standard for instructional integrity, clinical authenticity, and alignment with explicit learning objectives.

Supplementary Table 1. Adapted Healthcare Simulation Standards of Best Practice (HSSOBP) Framework for LLM-Based Simulation

Original Domain	Adapted Use for LLM Simulation	Example Adapted Item	Rationale for Adaptation
**Simulation Design**	Case Logic and Clinical Coherence	“The simulation scenario demonstrates logical clinical progression.”	To ensure the AI-generated narrative is clinically sound and progresses realistically over time.
**Learning Objectives**	Explicit Alignment with Competency	“The case clearly supports its stated learning objective(s).”	To verify that the simulation content is purposefully designed to meet its educational goals.
**Facilitation**	Static Instructional Clarity	“The instructions and case framing are appropriate and clear.”	To assess instructional quality in a static, non-facilitated context by evaluating the clarity of the prompt's setup and guidance.
**Fidelity**	Clinical Narrative Plausibility	"The patient history and diagnostic data are realistic and internally consistent."	To translate the concept of physical realism into the context of a text-based scenario.

Supplementary C

A.2 Simulation Design Scale (SDS)

- **Original Purpose**: The SDS is a validated instrument used to capture learners' perceptions of a simulation's design quality, focusing on elements like objectives, problem-solving support, and fidelity.[2]

- **Specific Challenges in the LLM Context**: The standard SDS includes items about "cues" and "feedback" that presume a dynamic interaction with a facilitator who can provide hints or clarification in real time. In the LLM framework, all cues and feedback are pre-written into the static prompt and its answer rationales. The challenge was to adapt the instrument to assess the quality of these *pre-designed* elements

- **Detailed Adaptation Process**: Items related to dynamic feedback were removed. The panel revised the remaining items to focus on the static architecture of the simulation. For example, questions about "cues" were reframed to assess whether the initial case presentation contained sufficient information for the learner to make an informed decision. The evaluation of "flow" was adapted to judge the logical sequencing of the multiple-choice questions and the coherence of the case's progression from diagnosis to management.

- **Rationale for Inclusion**: The SDS provides a structured lens for evaluators to systematically assess the *instructional architecture* of the AI-generated case. It moves beyond clinical accuracy to ask: Is this scenario well-designed from an educational standpoint? Does it provide the necessary scaffolding for a learner to engage in meaningful clinical decision-making?

Supplementary Table 2. Adapted Simulation Design Scale (SDS) Framework for LLM-Based Simulation

Original SDS Dimension	Adapted Domain for LLM Simulation	Example Adapted Item	Rationale for Adaptation
**Objectives & Information**	Alignment with Educational Intent	"The learning objectives are explicitly addressed by the case content."	To confirm that the case content directly serves the stated learning purpose.
**Scenario Flow/Complexity**	Logical Sequencing and Branching	"The simulation proceeds through a clinically coherent sequence of decisions."	To evaluate the structural integrity and narrative logic of the pre-scripted case.
**Fidelity**	Realism and Accuracy of Clinical Detail	"The case reflects realistic clinical scenarios relevant to current practice."	To ensure the AI's output mirrors authentic clinical situations, enhancing learner engagement.
**Support (Cues/Feedback)**	Quality of Embedded Rationales	"The provided rationales for correct and incorrect answers are clear and evidence-based."	To assess the quality of the static, pre-written feedback provided after each decision point.

Supplementary C

A.3 Clinical Reasoning Evaluation Rubric (CRER)

- **Original Purpose**: The CRER is a performance-based rubric designed to assess a *learner's* clinical reasoning process during a simulation, evaluating their ability to collect data, form hypotheses, and prioritize interventions.[3]

- **Specific Challenges in the LLM Context**: The primary challenge was conceptual: applying an instrument designed to evaluate a human thought process to a static, non-sentient artifact. The AI is not "reasoning" in the human sense. Therefore, the goal was not to assess a live process but to evaluate whether the final output models expert-level clinical reasoning for the learner.

- **Detailed Adaptation Process**: The CRER was fundamentally "reversed" in its orientation. Instead of an evaluator using it to score a student, the evaluator used it to score the simulation itself. Items were rephrased to assess the simulation's demonstration of reasoning. For example, "Did the student identify the most important problem?" became "Does the case present a clear and relevant clinical problem?" "Did the student generate critical hypotheses?" became "Does the simulation's line of questioning and its answer options consider an appropriate differential diagnosis?

- **Rationale for Inclusion**: This adapted rubric is crucial for assessing the *clinical logic fidelity* of the simulation. It allows for a granular analysis of whether the AI-generated case accurately models the high-level reasoning patterns that learners are supposed to develop. It serves as a quality check against the AI producing clinically flawed or illogical scenarios.

Supplementary Table 3. Adapted Clinical Reasoning Evaluation Rubric (CRER) Framework for LLM-Based Simulation

Original CRER Domain	Adapted Domain for LLM Simulation	Example Adapted Item	Rationale for Adaptation
**Problem Identification**	Diagnostic Clarity and Relevance	“The case presents a clear and relevant clinical problem.”	To assess if the simulation's setup effectively models the initial stage of clinical reasoning.
**Hypothesis Generation**	Diagnostic and Therapeutic Breadth	“The multiple-choice options include plausible and relevant differential diagnoses/therapies.”	To evaluate if the simulation models a thorough thought process by presenting realistic alternatives.
**Prioritization**	Decision-Making Relevance and Risk	“The chosen intervention in the case narrative reflects appropriate prioritization of clinical risks.”	To check if the AI's scripted pathway follows logical, risk-adjusted clinical priorities.
**Evaluation of Outcomes**	Consequence Modeling and Feedback	“The simulation describes plausible clinical outcomes for each intervention choice.”	To ensure the AI provides realistic consequences, which is critical for learning.

Supplementary C

A.4 Simulation Effectiveness Tool–Modified (SET-M)

- **Original Purpose**: The SET-M is a validated self-report tool for learners to evaluate their perceived effectiveness of a simulation, including their confidence, satisfaction, and perceived learning gains.[4]

- **Specific Challenges in the LLM Context**: The original SET-M contains items that conflate the learning experience with the actions of a human facilitator and the quality of a live debriefing session. The challenge was to isolate the learner's perception of the *AI-generated content itself*, separate from any human element.

- **Detailed Adaptation Process**: The adaptation was straightforward and involved the targeted removal of all items referencing a human instructor, facilitator, or debriefing session. The remaining items, focused on the simulation's realism, the clarity of the learning objectives, and the learner's own confidence and satisfaction, were preserved. This created a streamlined instrument focused purely on the learner's direct interaction with the AI tool.

- **Rationale for Inclusion**: While the other instruments focus on expert evaluation of the simulation's quality, the adapted SET-M is essential for capturing the learner-centered perspective. It provides critical data on user experience, perceived educational value, and engagement. Positive expert reviews are of little value if the end-users—the students—do not find the tool effective, realistic, or satisfying.

Supplementary Table 4. Adapted Simulation Effectiveness Tool–Modified (SET-M) Framework for LLM-Based Simulation

Original SET-M Domain	Adapted Domain for LLM Simulation	Example Adapted Item	Rationale for Adaptation
**Learning & Confidence**	Comparative Confidence Building	"Which method made you feel more confident in your clinical skills?"	To directly compare perceived cognitive gains between traditional and AI-based learning approaches
**Realism & Relevance**	Comparative Clinical Authenticity	"Which method felt more like real clinical practice?"	To capture learner perception of relative fidelity between learning modalities
**Satisfaction**	Comparative Learning Enjoyment	"Which method did you enjoy learning with more?"	To assess relative user experience and engagement with different educational approaches
**Instruction Quality**	Comparative Usability Assessment	"Which method was easier to use?"	To evaluate relative accessibility and user-friendliness of learning modalities
**Educational Effectiveness**	Comparative Skill Development	"Which method better helped improve your diagnostic skills?"	To assess perceived relative educational impact across learning approaches
**Practical Application**	Future Use Preference	"Which method would you want to use again?"	To determine learner preferences for future educational experiences
**Time Efficiency**	Comparative Time Management	"Which method saved you more time?"	To assess relative efficiency of learning modalities in educational settings
**Exam Preparation**	Comparative Assessment Readiness	"Which method do you believe better prepares you for exams?"	To evaluate perceived relative effectiveness for academic performance preparation

Supplementary C

Content Validity Results Summary

The adapted evaluation framework demonstrated strong content validity, item- and scale-level Content Validity Indices (I-CVI and S-CVI), across all instruments [5]:

- HSSOBP adaptation: S-CVI = 0.92 (12 items retained, 8 items excluded)
- SDS adaptation: S-CVI = 0.95 (15 items retained, 5 items excluded)
- CRER adaptation: S-CVI = 0.89 (10 items retained, 6 items revised)
- SET-M adaptation: S-CVI = 0.93 (8 comparative items retained, 12 items excluded)

The final three-domain, twelve-subdomain framework achieved overall S-CVI = 0.92, confirming the appropriateness of the adapted instruments for evaluating LLM-generated pharmacotherapy simulations.

Supplementary C

Rubric for Assessing LLM-Simulated Pharmacotherapy Case Presentations

This rubric is designed for expert evaluators (clinical pharmacy professors, practitioners, and instructional designers) to assess the quality, accuracy, and educational effectiveness of Large Language Model (LLM)-generated clinical case simulations. It integrates adapted criteria from the Healthcare Simulation Standards of Best Practice (HSSOBP), Simulation Design Scale (SDS), and Clinical Reasoning Evaluation Rubric (CRER).

Instructions for Evaluators

Rate each criterion on a scale of 1 to 5, where:

- **1 = Poor/Missing:** The element is absent or critically flawed.
- **2 = Fair:** The element is present but has significant weaknesses.
- **3 = Good:** The element is present and functional but could be improved.
- **4 = Very Good:** The element is well-executed with minor room for improvement.
- **5 = Excellent:** The element is clear, accurate, and expertly implemented.

A "Not Applicable" (N/A) option is available for criteria that do not apply to a specific simulation.

Section 1: Instructional Design and Pedagogical Quality

This section assesses the foundational educational architecture of the simulation. It evaluates how well the case is structured to support learning, based on adapted principles from the Simulation Design Scale (SDS) and HSSOBP.

Supplementary Table 5. Rubrics used by the expert panel to assess the Instructional Design and Pedagogical Quality domain

Criterion	1 (Poor)	2 (Fair)	3 (Good)	4 (Very Good)	5 (Excellent)	Score
**1.1 Clarity of Learning Objectives** Learning objectives are explicitly stated and clearly aligned with professional competencies (e.g., ACPE, HOPA).	Obscure or misaligned objectives	Vaguely stated objectives	Clear objectives, loosely aligned	Clear and mostly aligned objectives	Explicit, measurable, and perfectly aligned objectives	
**1.2 Scaffolding of Clinical Reasoning** The case progresses logically from basic concepts to complex decisions, aligning with Bloom's Taxonomy.	Illogical or flat progression	Weak progression, lacks structure	Logical progression with some gaps	Well-structured, scaffolded progression	Expertly scaffolded, builds complexity seamlessly	

Supplementary C

**1.3 Quality of Embedded Feedback** Rationales for correct and incorrect answers are clear, evidence-based, and educationally valuable.	Missing or erroneous rationales	Vague or weakly justified rationales	Correct but superficial rationales	Clear, evidence-based rationales	Insightful, guideline-anchored, and highly instructive rationales	
**1.4 Instructional Clarity & Framing** Instructions, case framing, and commands (e.g., START, HINT) are clear and guide the learner effectively.	Confusing or absent instructions	Ambiguous instructions requiring inference	Functional but basic instructions	Clear and helpful instructions	Exceptionally clear and intuitive instructions	

Supplementary C

Section 2: Clinical Authenticity and Content Fidelity

This section evaluates the clinical accuracy and realism of the simulation content. It draws from adapted criteria from HSSOBP (Fidelity) and the study's emphasis on evidence-based practice.

Supplementary Table 6. Rubrics used by the expert panel to assess the Clinical Authenticity and Content Fidelity domain by the panel experts

Criterion	1 (Poor)	2 (Fair)	3 (Good)	4 (Very Good)	5 (Excellent)	Score
**2.1 Clinical Narrative Plausibility** The patient history, diagnostic data, and case evolution are realistic and internally consistent.	Unrealistic or contradictory narrative	Contains several implausible elements	Generally plausible with minor inconsistencies	Highly realistic and internally consistent	Indistinguishable from an authentic clinical case	
**2.2 Alignment with Clinical Guidelines** All diagnostic, therapeutic, and monitoring recommendations strictly adhere to the designated guidelines (e.g., NCCN).	Gross deviation from guidelines	Some significant deviations	Mostly aligned, with minor inaccuracies	Strongly aligned with guidelines	Perfectly aligned with current, evidence-based guidelines	
**2.3 Pharmacotherapeutic Accuracy** Drug selection, dosing, and monitoring parameters are appropriate for the specific clinical context.	Dangerous or incorrect recommendations	Suboptimal or questionable choices. May include misuse of agents from other disease states.	Acceptable but not first-line choices	Appropriate and well-justified choices	Optimal, guideline-directed therapeutic choices	
**2.4 Domain Specificity (Absence of Entanglement)** The case remains strictly within its specified disease domain (e.g., AML vs. CML) without blending concepts.	A therapy, diagnostic, or standard from another disease is incorrectly applied (e.g., using an ALL-specific therapy for AML). A single instance represents a critical flaw.	N/A	N/A	N/A	The case remains strictly within its specified disease domain without blending concepts or standards of care	

Supplementary C

Section 3: Modeled Clinical Reasoning

This section assesses whether the simulation's structure, questions, and outcomes effectively model an expert clinical reasoning process. It is based on the "reversed" application of the Clinical Reasoning Evaluation Rubric (CRER).

Supplementary Table 7. Rubrics used by the expert panel to assess the Modeled Clinical Reasoning domain

Criterion	1 (Poor)	2 (Fair)	3 (Good)	4 (Very Good)	5 (Excellent)	Score
**3.1 Problem Identification & Framing** The case presents a clear, relevant, and well-defined clinical problem for the learner to address.	Unclear or irrelevant problem	Poorly defined clinical problem	Adequately framed clinical problem	Clearly presented and relevant problem	Expertly framed problem, highlighting key challenges	
**3.2 Diagnostic and Therapeutic Breadth** Multiple-choice options include plausible, relevant alternatives and common distractors, modeling a thorough thought process.	Illogical or irrelevant options	Limited or poorly constructed options	Plausible options, but lacking key distractors	Good range of relevant and plausible options	Excellent options reflecting expert differential diagnosis	
**3.3 Prioritization and Decision-Making** The case narrative and decision points reflect appropriate prioritization of clinical risks and benefits.	Illogical or unsafe prioritization. The overall strategy is flawed.	Flawed prioritization of risks	Acceptable prioritization	Appropriate, risk-adjusted prioritization	Expert-level prioritization. The *reasoning strategy* is sound, even if the specific agent chosen contains a content error (which is scored in Sec. 2).	
**3.4 Modeling of Clinical Outcomes** The simulation describes plausible and educationally relevant clinical consequences for each intervention choice.	Unrealistic or missing outcomes	Vague or implausible outcomes	Plausible but generic outcomes	Realistic and specific outcomes	Highly instructive outcomes that reinforce learning	

Supplementary C

Section 4: Learner-Centered Evaluation

This section captures the learner's perspective on the simulation's effectiveness and usability, based on the adapted Simulation Effectiveness Tool–Modified (SET-M). This is intended to be completed by the student participant.

Supplementary Table 8. Five-point Likert scale used by students to assess the Learner-Centered Evaluation domain

Criterion	1 (Strongly Disagree)	2 (Disagree)	3 (Neutral)	4 (Agree)	5 (Strongly Agree)
**4.1 Ease of Use:** Which method was easier to use?					
**4.2 Time Efficiency:** Which method saved you more time?					
**4.3 Clinical Realism:** Which method felt more like real clinical practice?					
**4.4 Diagnostic Skill Development:** Which method better helped improve your diagnostic skills?					
**4.5 Confidence Building:** After using each method, which made you feel more confident in your clinical skills?					
**4.6 Learning Enjoyment:** Which method did you enjoy learning with more?					
**4.7 Exam Preparation:** Which method do you believe better prepares you for exams?					
**4.8 Future Use Preference**: Which method would you want to use again?					

Supplementary C

References

1. Watts PI, McDermott DS, Alinier G, et al. Healthcare Simulation Standards of Best PracticeTM Simulation Design. *Clin Simul Nurs*. 2021;58:14-21. doi:10.1016/j.ecns.2021.08.009
2. Hughes K. Simulation in nursing education. *Am J Nurs*. 2018;118(7):13. doi:10.1097/01.NAJ.0000541416.90476.1a
3. Furze J, Gale JR, Black L, Cochran TM, Jensen GM. Clinical Reasoning: Development of a Grading Rubric for Student Assessment. *J Phys Ther Educ*. 2015;29(3):34-45. doi:10.1097/00001416-201529030-00006
4. Leighton K, Ravert P, Mudra V, Macintosh C. Updating the Simulation Effectiveness Tool: Item modifications and reevaluation of psychometric properties. *Nurs Educ Perspect*. 2015;36(5):317-323. doi:10.5480/15-1671
5. Polit DF, Beck CT. The content validity index: Are you sure you know what's being reported? Critique and recommendations. *Res Nurs Heal*. 2006;29(5):489-497. doi:10.1002/NUR.20147

Supplementary D

D.1 Expert Inter-Rater Reliability Analysis

D.1.1 Overall Reliability Measures
Expert evaluations across 1,236 total assessments (103 simulations × 12 subdomains × 3 experts) demonstrated excellent inter-rater reliability using multiple convergent measures:

- **Krippendorff's Alpha: 0.830** (Good reliability; >0.80 threshold)
- **ICC(2,k) Average Measures: 0.941** (Excellent reliability; >0.90 threshold)
- **ICC(2,1) Single Measures: 0.831** (Good reliability)
- **Fleiss' Kappa: 0.733** (Substantial agreement; 0.60-0.80 range)

Individual expert pair reliability varied significantly, indicating differential agreement patterns.

Supplementary Table 9. Pairwise Expert Agreement

Expert Pair	Cohen's Kappa	Pearson r	Spearman ρ	ICC(2,1)
**AF vs AZ**	0.955†	0.968	0.975	0.968
**AF vs AA**	0.656	0.796	0.744	0.775
**AA vs AZ**	0.633	0.777	0.725	0.762

$p < 0.001$; †Almost perfect agreement (>0.80)

Clinical Interpretation: The high AF-AZ agreement (κ = 0.955) reflects strong concordance between the clinical pharmacy researcher and oncology pharmacist, while moderate AF-AA and AA-AZ agreements suggest the clinical pharmacy educator applied different evaluation criteria, contributing valuable perspective diversity.
Disease-specific reliability patterns revealed systematic differences.

Supplementary Table 10. Reliability by Disease Type

Disease Type	n	Krippendorff's α	ICC(2,k)	Fleiss' κ	Interpretation
**CML**	636	0.853	0.951	0.748	Excellent
**AML**	600	0.810	0.933	0.717	Good

Clinical Significance: Higher CML reliability (α = 0.853 vs 0.810) suggests greater expert consensus on CML case evaluation, consistent with CML's more standardized treatment pathways compared to AML's heterogeneous therapeutic landscape.
Platform-specific reliability revealed differential expert agreement patterns.

Supplementary Table 11. Reliability by LLM Platform

LLM Platform	n	Krippendorff's α	ICC(2,k)	Fleiss' κ
**Claude 3.7 Sonnet**	264	0.881	0.958	0.910†
**DeepSeek V2**	276	0.834	0.942	0.695
**GPT-4.O**	348	0.813	0.940	0.605
**Gemini 2.0 Pro**	348	0.803	0.930	0.777

†Almost perfect agreement (>0.80)

Domain-specific reliability patterns identified differential expert agreement.

Supplementary D

Supplementary Table 12. Reliability by Assessment Domain

Assessment Domain	n	Krippendorff's α	ICC(2,k)	Fleiss' κ
**Clinical Authenticity & Content Fidelity**	412	0.930†	0.976	0.859†
**Instructional Design & Pedagogical Quality**	412	0.825	0.937	0.906†
**Modeled Clinical Reasoning**	412	0.590‡	0.872	0.359‡

†Excellent reliability (>0.80); ‡Questionable reliability (<0.67)

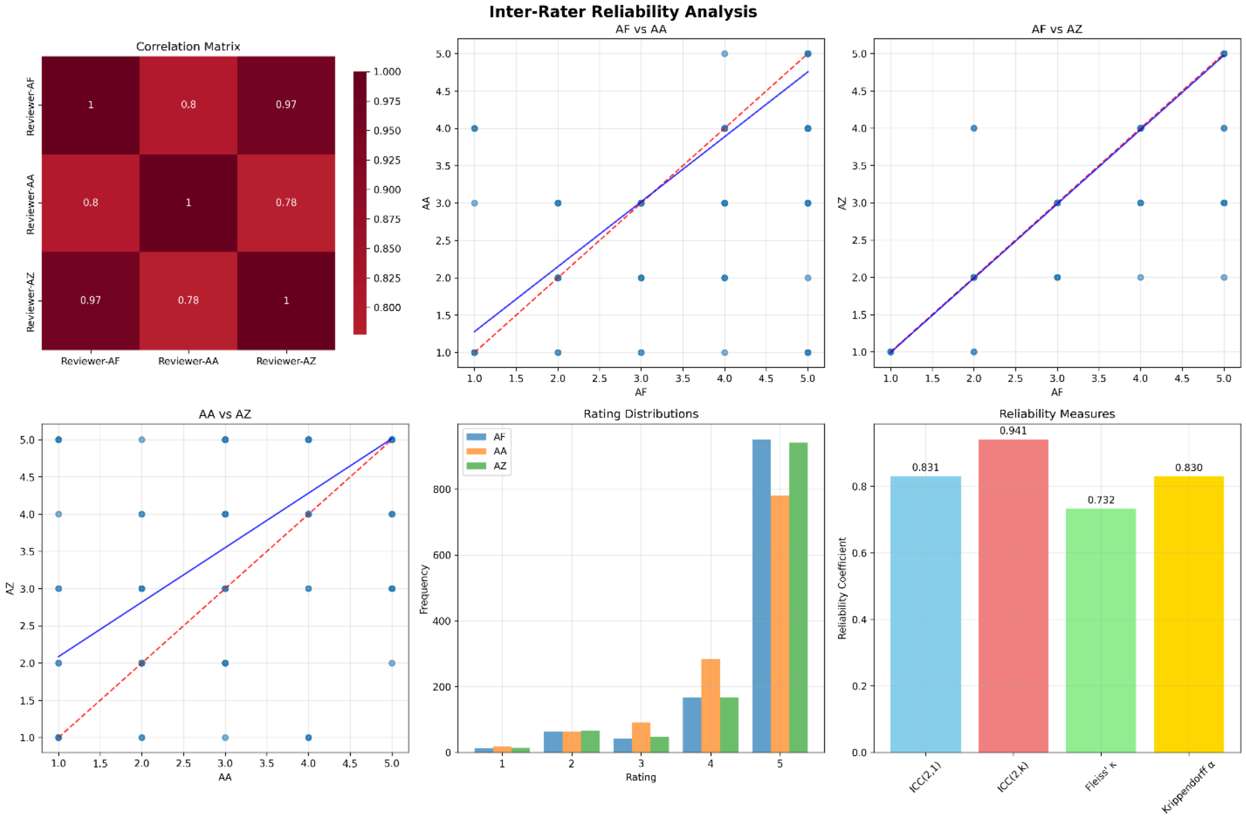

Expert agreement was evaluated across 1,236 assessments (103 simulations × 12 subdomains × 3 reviewers: AF, AA, AZ) using a six-panel approach. A correlation matrix demonstrated strong concordance between AF and AZ (r = 0.97) and moderate agreement for AF–AA (r = 0.80) and AA–AZ (r = 0.78). Pairwise scatter plots further illustrated these patterns, with points representing individual subdomain scores on a 1–5 scale. Tight clustering along the diagonal for AF–AZ indicated high agreement, whereas AF–AA and AA–AZ pairs displayed wider dispersion, reflecting moderate variability in scoring. Rating distributions revealed generally positive evaluations, with higher frequencies at scores 4–5 and slight reviewer-specific tendencies. Reliability metrics summarized in a bar chart confirmed robust agreement across indices: ICC(2,1) = 0.831, ICC(2,k) = 0.941, Fleiss' κ = 0.732, and Krippendorff's α = 0.830, all exceeding standard thresholds. Overall, these results validate the reliability of the evaluation framework, with high concordance among clinically specialized reviewers and moderate variability providing valuable educational perspective diversity.

Supplementary Figure 1. Expert Inter-Rater Reliability Across Evaluation Domains

Supplementary D

D.2 Student Evaluation Internal Consistency Analysis

D.2.1 Overall Internal Consistency

Student satisfaction survey (n = 102 respondents, 8 items) demonstrated excellent internal consistency:

- **Cronbach's Alpha: 0.939** (Excellent; >0.90 threshold)
- **Mean Inter-Item Correlation: 0.668**
- **Scale Reliability: Excellent across all dimensions**

All survey items exceeded the minimum acceptable correlation threshold (r > 0.30).

Supplementary Table 13. Item-Total Correlations

Survey Item	Item-Total Correlation	Interpretation
**"I would want to use this method again"**	0.851	Excellent
**"I enjoyed learning with this method"**	0.850	Excellent
**"This method can help me prepare for exams"**	0.821	Excellent
**"I feel more confident in my clinical skills"**	0.805	Excellent
**"This method improved my diagnostic skills"**	0.792	Good
**"This method was easy to use"**	0.751	Good
**"This method saved me time"**	0.725	Good
**"This method felt like real clinical practice"**	0.665	Acceptable

Cronbach's alpha if item deleted analysis confirmed optimal scale composition.

Supplementary Table 14. Scale Optimization Analysis

Item	α if Deleted	Change	Recommendation
**"This method felt like real clinical practice"**	0.939	+0.0003	**Retain** (minimal impact)
**"This method was easy to use"**	0.933	-0.0060	**Retain**
**"This method saved me time"**	0.935	-0.0042	**Retain**
**All other items**	0.926-0.928	-0.0089 to -0.0132	**Retain**

Scale Integrity: No item deletion would substantially improve internal consistency, confirming the 8-item scale's optimal composition.
Student responses across all dimensions (5-point Likert scale: 1=Strongly Traditional, 5=Strongly LLM).

Supplementary Table 15. Descriptive Statistics Summary

Dimension	Mean ± SD	Median	IQR	Interpretation
**Easy to use**	3.74 ± 1.39	4.0	3.0-5.0	**LLM preference**
**Time saving**	3.70 ± 1.38	4.0	2.0-5.0	**LLM preference**
**Future use intent**	3.53 ± 1.45	4.0	2.0-5.0	**LLM preference**
**Learning enjoyment**	3.39 ± 1.45	3.0	2.0-5.0	**Slight LLM preference**
**Exam preparation**	3.36 ± 1.43	3.0	2.0-5.0	**Slight LLM preference**

Supplementary D

**Diagnostic skills**	3.32 ± 1.44	3.0	2.0-5.0	**Slight LLM preference**
**Clinical confidence**	3.18 ± 1.40	3.0	2.0-4.0	**Slight LLM preference**
**Clinical authenticity**	3.03 ± 1.47	3.0	2.0-4.0	**Neutral**

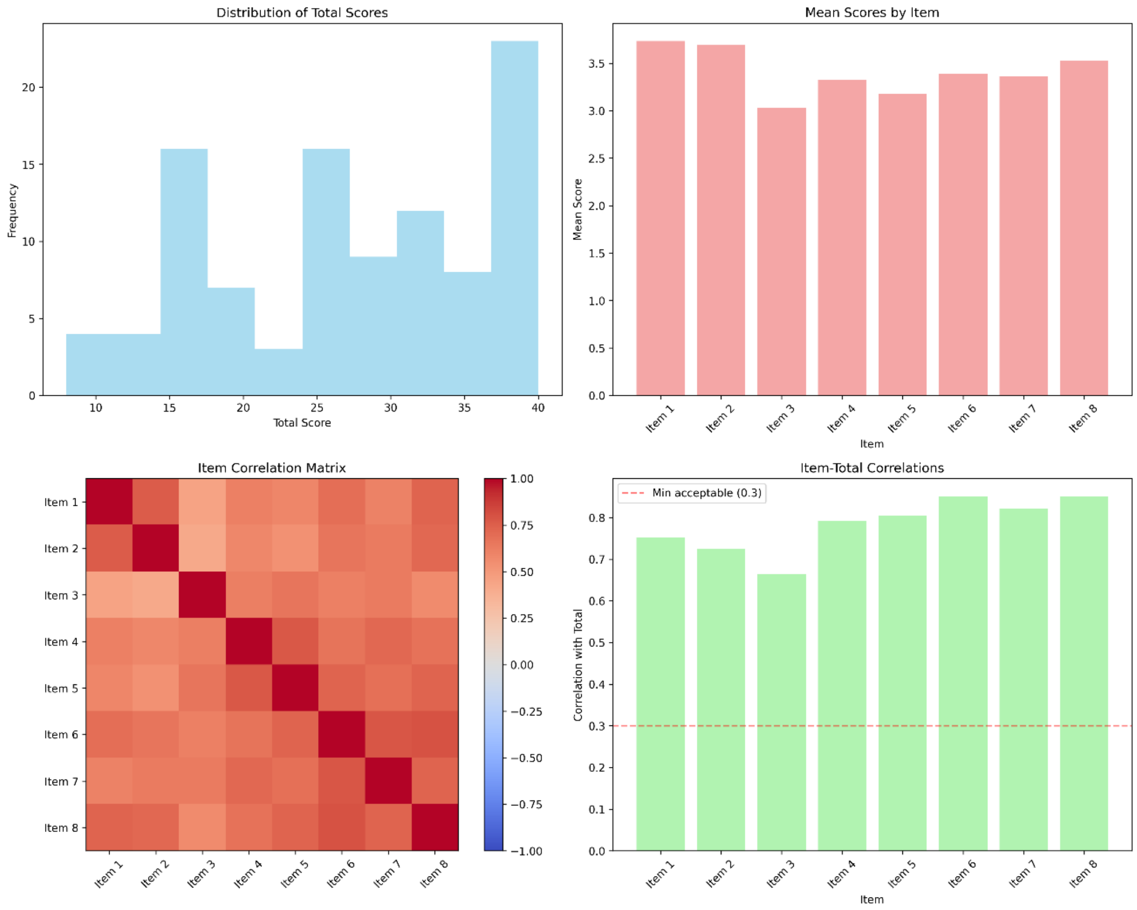

Student survey responses across 102 participants and 8 dimensions were analyzed for internal consistency and response patterns. Total scores, ranging from 8 to 40, were right-skewed with a peak at 35–40, indicating strong overall preference for LLM-based learning, with mean scores significantly above the neutral midpoint of 24. Mean item responses on a 5-point Likert scale ranked highest for “Easy to use” (3.74) and “Time saving” (3.70), and lowest for “Clinical authenticity” (3.03). Inter-item correlations, visualized via a heatmap, demonstrated strong positive relationships, supporting the conceptual coherence of the survey. Item-total correlations exceeded the 0.30 threshold (range 0.665–0.851), confirming scale reliability, and Cronbach’s α = 0.939 indicated excellent internal consistency. Together, these findings provide robust psychometric evidence that the 8-dimension survey reliably captures student preferences, highlighting the engagement–safety paradox: learners favor LLM-based simulations even in the presence of expert-identified clinical accuracy concerns.

Supplementary Figure 2. Student Survey Reliability and Response Patterns

D.3 Reliability Interpretation and Clinical Implications

D.3.1 Expert Reliability Conclusions

- **Overall excellent reliability** (α = 0.830) validates the adapted evaluation framework
- **Disease-specific patterns** suggest CML cases are more consistently evaluable than AML cases
- **Domain-specific analysis** revealed that clinical reasoning assessments exhibited lower agreement (α = 0.590), highlighting the inherent complexity of evaluating higher-order cognitive processes and suggesting areas for methodological refinement. Objective domains, including clinical authenticity (α = 0.930) and instructional quality (α = 0.825), maintained excellent reliability, confirming the framework's ability to measure well-defined constructs.
- **Platform differences** indicate Claude generates most consistently interpretable content

D.3.2 Student Consistency Conclusions

- **Excellent internal consistency** (α = 0.939) confirms reliable measurement of student preferences
- **Systematic LLM preference** across usability dimensions despite expert-identified safety concerns
- **Engagement-safety paradox** validated through reliable measurement instruments

D.3.3 Methodological Validation

Both expert and student evaluation instruments demonstrate strong psychometric properties, supporting the validity of study conclusions regarding the engagement-safety paradox and differential LLM performance across clinical domains

Supplementary D

Supplementary Table 16. Individual Domain/Subdomain Score per LLM Session

Session ID	Leukemia Type	Assessment Domain	Assessment Subdomain	LLM	Reviewer-AF	Reviewer-AA	Reviewer-AZ
**51**	CML	Instructional Design and Pedagogical Quality	1.1 Clarity of Learning Objectives	Gemini 2.0 Pro	4	4	4
**52**	CML	Instructional Design and Pedagogical Quality	1.1 Clarity of Learning Objectives	Gemini 2.0 Pro	4	4	4
**53**	CML	Instructional Design and Pedagogical Quality	1.1 Clarity of Learning Objectives	Gemini 2.0 Pro	4	4	4
**54**	CML	Instructional Design and Pedagogical Quality	1.1 Clarity of Learning Objectives	Gemini 2.0 Pro	4	4	4
**55**	CML	Instructional Design and Pedagogical Quality	1.1 Clarity of Learning Objectives	Gemini 2.0 Pro	4	4	4
**56**	CML	Instructional Design and Pedagogical Quality	1.1 Clarity of Learning Objectives	Gemini 2.0 Pro	4	4	4
**57**	CML	Instructional Design and Pedagogical Quality	1.1 Clarity of Learning Objectives	Gemini 2.0 Pro	4	4	4
**58**	CML	Instructional Design and Pedagogical Quality	1.1 Clarity of Learning Objectives	Gemini 2.0 Pro	4	4	4
**59**	CML	Instructional Design and Pedagogical Quality	1.1 Clarity of Learning Objectives	Gemini 2.0 Pro	4	4	4
**60**	CML	Instructional Design and Pedagogical Quality	1.1 Clarity of Learning Objectives	Gemini 2.0 Pro	4	4	4
**61**	CML	Instructional Design and Pedagogical Quality	1.1 Clarity of Learning Objectives	Gemini 2.0 Pro	4	4	4
**62**	CML	Instructional Design and Pedagogical Quality	1.1 Clarity of Learning Objectives	Gemini 2.0 Pro	4	4	4
**63**	CML	Instructional Design and Pedagogical Quality	1.1 Clarity of Learning Objectives	DeepSeek V2	4	4	4
**64**	CML	Instructional Design and Pedagogical Quality	1.1 Clarity of Learning Objectives	DeepSeek V2	4	4	4
**65**	CML	Instructional Design and Pedagogical Quality	1.1 Clarity of Learning Objectives	DeepSeek V2	4	4	4
**66**	CML	Instructional Design and Pedagogical Quality	1.1 Clarity of Learning Objectives	DeepSeek V2	4	4	4
**67**	CML	Instructional Design and Pedagogical Quality	1.1 Clarity of Learning Objectives	DeepSeek V2	4	4	4
**68**	CML	Instructional Design and Pedagogical Quality	1.1 Clarity of Learning Objectives	DeepSeek V2	4	4	4
**69**	CML	Instructional Design and Pedagogical Quality	1.1 Clarity of Learning Objectives	DeepSeek V2	4	4	4
**70**	CML	Instructional Design and Pedagogical Quality	1.1 Clarity of Learning Objectives	DeepSeek V2	4	4	4
**71**	CML	Instructional Design and Pedagogical Quality	1.1 Clarity of Learning Objectives	DeepSeek V2	4	4	4
**72**	CML	Instructional Design and Pedagogical Quality	1.1 Clarity of Learning Objectives	DeepSeek V2	4	4	4
**73**	CML	Instructional Design and Pedagogical Quality	1.1 Clarity of Learning Objectives	DeepSeek V2	3	3	3
**74**	CML	Instructional Design and Pedagogical Quality	1.1 Clarity of Learning Objectives	DeepSeek V2	4	4	4
**75**	CML	Instructional Design and Pedagogical Quality	1.1 Clarity of Learning Objectives	DeepSeek V2	4	4	4

Supplementary D

**76**	CML	Instructional Design and Pedagogical Quality	1.1 Clarity of Learning Objectives	Claude 3.7 Sonnet	4	4	4
**77**	CML	Instructional Design and Pedagogical Quality	1.1 Clarity of Learning Objectives	Claude 3.7 Sonnet	4	4	4
**78**	CML	Instructional Design and Pedagogical Quality	1.1 Clarity of Learning Objectives	Claude 3.7 Sonnet	5	5	5
**79**	CML	Instructional Design and Pedagogical Quality	1.1 Clarity of Learning Objectives	Claude 3.7 Sonnet	4	4	4
**80**	CML	Instructional Design and Pedagogical Quality	1.1 Clarity of Learning Objectives	Claude 3.7 Sonnet	4	4	4
**81**	CML	Instructional Design and Pedagogical Quality	1.1 Clarity of Learning Objectives	Claude 3.7 Sonnet	5	5	5
**82**	CML	Instructional Design and Pedagogical Quality	1.1 Clarity of Learning Objectives	Claude 3.7 Sonnet	5	5	5
**83**	CML	Instructional Design and Pedagogical Quality	1.1 Clarity of Learning Objectives	Claude 3.7 Sonnet	4	4	4
**84**	CML	Instructional Design and Pedagogical Quality	1.1 Clarity of Learning Objectives	Claude 3.7 Sonnet	4	4	4
**85**	CML	Instructional Design and Pedagogical Quality	1.1 Clarity of Learning Objectives	Claude 3.7 Sonnet	4	4	4
**86**	CML	Instructional Design and Pedagogical Quality	1.1 Clarity of Learning Objectives	Claude 3.7 Sonnet	4	4	4
**87**	CML	Instructional Design and Pedagogical Quality	1.1 Clarity of Learning Objectives	GPT 4.o	4	4	4
**88**	CML	Instructional Design and Pedagogical Quality	1.1 Clarity of Learning Objectives	GPT 4.o	4	4	4
**89**	CML	Instructional Design and Pedagogical Quality	1.1 Clarity of Learning Objectives	GPT 4.o	4	4	4
**90**	CML	Instructional Design and Pedagogical Quality	1.1 Clarity of Learning Objectives	GPT 4.o	5	5	5
**91**	CML	Instructional Design and Pedagogical Quality	1.1 Clarity of Learning Objectives	GPT 4.o	5	5	5
**92**	CML	Instructional Design and Pedagogical Quality	1.1 Clarity of Learning Objectives	GPT 4.o	4	5	4
**93**	CML	Instructional Design and Pedagogical Quality	1.1 Clarity of Learning Objectives	GPT 4.o	4	4	4
**94**	CML	Instructional Design and Pedagogical Quality	1.1 Clarity of Learning Objectives	GPT 4.o	4	4	4
**95**	CML	Instructional Design and Pedagogical Quality	1.1 Clarity of Learning Objectives	GPT 4.o	4	4	4
**96**	CML	Instructional Design and Pedagogical Quality	1.1 Clarity of Learning Objectives	GPT 4.o	4	3	4
**97**	CML	Instructional Design and Pedagogical Quality	1.1 Clarity of Learning Objectives	GPT 4.o	4	4	4

Supplementary D

**98**	CML	Instructional Design and Pedagogical Quality	1.1 Clarity of Learning Objectives	GPT 4.o	4	4	4
**99**	CML	Instructional Design and Pedagogical Quality	1.1 Clarity of Learning Objectives	GPT 4.o	4	4	4
**100**	CML	Instructional Design and Pedagogical Quality	1.1 Clarity of Learning Objectives	GPT 4.o	4	4	4
**101**	CML	Instructional Design and Pedagogical Quality	1.1 Clarity of Learning Objectives	GPT 4.o	4	4	4
**102**	CML	Instructional Design and Pedagogical Quality	1.1 Clarity of Learning Objectives	GPT 4.o	4	4	4
**103**	CML	Instructional Design and Pedagogical Quality	1.1 Clarity of Learning Objectives	GPT 4.o	4	4	4
**51**	CML	Instructional Design and Pedagogical Quality	1.2 Scaffolding of Clinical Reasoning	Gemini 2.0 Pro	5	5	5
**52**	CML	Instructional Design and Pedagogical Quality	1.2 Scaffolding of Clinical Reasoning	Gemini 2.0 Pro	5	5	5
**53**	CML	Instructional Design and Pedagogical Quality	1.2 Scaffolding of Clinical Reasoning	Gemini 2.0 Pro	5	5	5
**54**	CML	Instructional Design and Pedagogical Quality	1.2 Scaffolding of Clinical Reasoning	Gemini 2.0 Pro	5	5	5
**55**	CML	Instructional Design and Pedagogical Quality	1.2 Scaffolding of Clinical Reasoning	Gemini 2.0 Pro	5	5	5
**56**	CML	Instructional Design and Pedagogical Quality	1.2 Scaffolding of Clinical Reasoning	Gemini 2.0 Pro	5	5	5
**57**	CML	Instructional Design and Pedagogical Quality	1.2 Scaffolding of Clinical Reasoning	Gemini 2.0 Pro	5	5	5
**58**	CML	Instructional Design and Pedagogical Quality	1.2 Scaffolding of Clinical Reasoning	Gemini 2.0 Pro	5	5	5
**59**	CML	Instructional Design and Pedagogical Quality	1.2 Scaffolding of Clinical Reasoning	Gemini 2.0 Pro	5	5	5
**60**	CML	Instructional Design and Pedagogical Quality	1.2 Scaffolding of Clinical Reasoning	Gemini 2.0 Pro	5	5	5
**61**	CML	Instructional Design and Pedagogical Quality	1.2 Scaffolding of Clinical Reasoning	Gemini 2.0 Pro	5	5	5
**62**	CML	Instructional Design and Pedagogical Quality	1.2 Scaffolding of Clinical Reasoning	Gemini 2.0 Pro	5	5	5
**63**	CML	Instructional Design and Pedagogical Quality	1.2 Scaffolding of Clinical Reasoning	DeepSee k V2	5	5	5
**64**	CML	Instructional Design and Pedagogical Quality	1.2 Scaffolding of Clinical Reasoning	DeepSee k V2	5	5	5
**65**	CML	Instructional Design and Pedagogical Quality	1.2 Scaffolding of Clinical Reasoning	DeepSee k V2	5	5	5
**66**	CML	Instructional Design and Pedagogical Quality	1.2 Scaffolding of Clinical Reasoning	DeepSee k V2	5	5	5
**67**	CML	Instructional Design and Pedagogical Quality	1.2 Scaffolding of Clinical Reasoning	DeepSee k V2	5	5	5
**68**	CML	Instructional Design and Pedagogical Quality	1.2 Scaffolding of Clinical Reasoning	DeepSee k V2	5	5	5
**69**	CML	Instructional Design and Pedagogical Quality	1.2 Scaffolding of Clinical Reasoning	DeepSee k V2	5	5	5
**70**	CML	Instructional Design and Pedagogical Quality	1.2 Scaffolding of Clinical Reasoning	DeepSee k V2	5	5	5
**71**	CML	Instructional Design and Pedagogical Quality	1.2 Scaffolding of Clinical Reasoning	DeepSee k V2	5	5	5

Supplementary D

**72**	CML	Instructional Design and Pedagogical Quality	1.2 Scaffolding of Clinical Reasoning	DeepSeek V2	5	5	5
**73**	CML	Instructional Design and Pedagogical Quality	1.2 Scaffolding of Clinical Reasoning	DeepSeek V2	5	5	5
**74**	CML	Instructional Design and Pedagogical Quality	1.2 Scaffolding of Clinical Reasoning	DeepSeek V2	5	5	5
**75**	CML	Instructional Design and Pedagogical Quality	1.2 Scaffolding of Clinical Reasoning	DeepSeek V2	5	5	5
**76**	CML	Instructional Design and Pedagogical Quality	1.2 Scaffolding of Clinical Reasoning	Claude 3.7 Sonnet	5	5	5
**77**	CML	Instructional Design and Pedagogical Quality	1.2 Scaffolding of Clinical Reasoning	Claude 3.7 Sonnet	5	5	5
**78**	CML	Instructional Design and Pedagogical Quality	1.2 Scaffolding of Clinical Reasoning	Claude 3.7 Sonnet	5	5	5
**79**	CML	Instructional Design and Pedagogical Quality	1.2 Scaffolding of Clinical Reasoning	Claude 3.7 Sonnet	5	5	5
**80**	CML	Instructional Design and Pedagogical Quality	1.2 Scaffolding of Clinical Reasoning	Claude 3.7 Sonnet	5	5	5
**81**	CML	Instructional Design and Pedagogical Quality	1.2 Scaffolding of Clinical Reasoning	Claude 3.7 Sonnet	5	5	5
**82**	CML	Instructional Design and Pedagogical Quality	1.2 Scaffolding of Clinical Reasoning	Claude 3.7 Sonnet	5	5	5
**83**	CML	Instructional Design and Pedagogical Quality	1.2 Scaffolding of Clinical Reasoning	Claude 3.7 Sonnet	5	5	5
**84**	CML	Instructional Design and Pedagogical Quality	1.2 Scaffolding of Clinical Reasoning	Claude 3.7 Sonnet	5	5	5
**85**	CML	Instructional Design and Pedagogical Quality	1.2 Scaffolding of Clinical Reasoning	Claude 3.7 Sonnet	5	5	5
**86**	CML	Instructional Design and Pedagogical Quality	1.2 Scaffolding of Clinical Reasoning	Claude 3.7 Sonnet	5	5	5
**87**	CML	Instructional Design and Pedagogical Quality	1.2 Scaffolding of Clinical Reasoning	GPT 4.o	5	5	5
**88**	CML	Instructional Design and Pedagogical Quality	1.2 Scaffolding of Clinical Reasoning	GPT 4.o	5	5	5
**89**	CML	Instructional Design and Pedagogical Quality	1.2 Scaffolding of Clinical Reasoning	GPT 4.o	5	5	5
**90**	CML	Instructional Design and Pedagogical Quality	1.2 Scaffolding of Clinical Reasoning	GPT 4.o	5	5	5
**91**	CML	Instructional Design and Pedagogical Quality	1.2 Scaffolding of Clinical Reasoning	GPT 4.o	5	5	5
**92**	CML	Instructional Design and Pedagogical Quality	1.2 Scaffolding of Clinical Reasoning	GPT 4.o	5	5	5
**93**	CML	Instructional Design and Pedagogical Quality	1.2 Scaffolding of Clinical Reasoning	GPT 4.o	5	5	5

Supplementary D

**94**	CML	Instructional Design and Pedagogical Quality	1.2 Scaffolding of Clinical Reasoning	GPT 4.o	5	5	5
**95**	CML	Instructional Design and Pedagogical Quality	1.2 Scaffolding of Clinical Reasoning	GPT 4.o	5	5	5
**96**	CML	Instructional Design and Pedagogical Quality	1.2 Scaffolding of Clinical Reasoning	GPT 4.o	5	5	5
**97**	CML	Instructional Design and Pedagogical Quality	1.2 Scaffolding of Clinical Reasoning	GPT 4.o	5	5	5
**98**	CML	Instructional Design and Pedagogical Quality	1.2 Scaffolding of Clinical Reasoning	GPT 4.o	5	5	5
**99**	CML	Instructional Design and Pedagogical Quality	1.2 Scaffolding of Clinical Reasoning	GPT 4.o	5	5	5
**100**	CML	Instructional Design and Pedagogical Quality	1.2 Scaffolding of Clinical Reasoning	GPT 4.o	5	5	5
**101**	CML	Instructional Design and Pedagogical Quality	1.2 Scaffolding of Clinical Reasoning	GPT 4.o	5	5	5
**102**	CML	Instructional Design and Pedagogical Quality	1.2 Scaffolding of Clinical Reasoning	GPT 4.o	5	5	5
**103**	CML	Instructional Design and Pedagogical Quality	1.2 Scaffolding of Clinical Reasoning	GPT 4.o	5	5	5
**51**	CML	Instructional Design and Pedagogical Quality	1.3 Quality of Embedded Feedback	Gemini 2.0 Pro	5	5	5
**52**	CML	Instructional Design and Pedagogical Quality	1.3 Quality of Embedded Feedback	Gemini 2.0 Pro	5	5	5
**53**	CML	Instructional Design and Pedagogical Quality	1.3 Quality of Embedded Feedback	Gemini 2.0 Pro	5	5	5
**54**	CML	Instructional Design and Pedagogical Quality	1.3 Quality of Embedded Feedback	Gemini 2.0 Pro	5	5	5
**55**	CML	Instructional Design and Pedagogical Quality	1.3 Quality of Embedded Feedback	Gemini 2.0 Pro	5	5	5
**56**	CML	Instructional Design and Pedagogical Quality	1.3 Quality of Embedded Feedback	Gemini 2.0 Pro	1	1	1
**57**	CML	Instructional Design and Pedagogical Quality	1.3 Quality of Embedded Feedback	Gemini 2.0 Pro	5	5	5
**58**	CML	Instructional Design and Pedagogical Quality	1.3 Quality of Embedded Feedback	Gemini 2.0 Pro	5	5	5
**59**	CML	Instructional Design and Pedagogical Quality	1.3 Quality of Embedded Feedback	Gemini 2.0 Pro	5	5	5
**60**	CML	Instructional Design and Pedagogical Quality	1.3 Quality of Embedded Feedback	Gemini 2.0 Pro	5	5	5
**61**	CML	Instructional Design and Pedagogical Quality	1.3 Quality of Embedded Feedback	Gemini 2.0 Pro	5	5	5
**62**	CML	Instructional Design and Pedagogical Quality	1.3 Quality of Embedded Feedback	Gemini 2.0 Pro	5	5	5
**63**	CML	Instructional Design and Pedagogical Quality	1.3 Quality of Embedded Feedback	DeepSeek V2	5	5	5
**64**	CML	Instructional Design and Pedagogical Quality	1.3 Quality of Embedded Feedback	DeepSeek V2	5	5	5
**65**	CML	Instructional Design and Pedagogical Quality	1.3 Quality of Embedded Feedback	DeepSeek V2	5	5	5
**66**	CML	Instructional Design and Pedagogical Quality	1.3 Quality of Embedded Feedback	DeepSeek V2	5	5	5
**67**	CML	Instructional Design and Pedagogical Quality	1.3 Quality of Embedded Feedback	DeepSeek V2	5	5	5

Supplementary D

**68**	CML	Instructional Design and Pedagogical Quality	1.3 Quality of Embedded Feedback	DeepSeek V2	5	5	5
**69**	CML	Instructional Design and Pedagogical Quality	1.3 Quality of Embedded Feedback	DeepSeek V2	5	5	5
**70**	CML	Instructional Design and Pedagogical Quality	1.3 Quality of Embedded Feedback	DeepSeek V2	5	5	5
**71**	CML	Instructional Design and Pedagogical Quality	1.3 Quality of Embedded Feedback	DeepSeek V2	5	5	5
**72**	CML	Instructional Design and Pedagogical Quality	1.3 Quality of Embedded Feedback	DeepSeek V2	5	5	5
**73**	CML	Instructional Design and Pedagogical Quality	1.3 Quality of Embedded Feedback	DeepSeek V2	2	1	2
**74**	CML	Instructional Design and Pedagogical Quality	1.3 Quality of Embedded Feedback	DeepSeek V2	5	5	5
**75**	CML	Instructional Design and Pedagogical Quality	1.3 Quality of Embedded Feedback	DeepSeek V2	4	4	4
**76**	CML	Instructional Design and Pedagogical Quality	1.3 Quality of Embedded Feedback	Claude 3.7 Sonnet	5	5	5
**77**	CML	Instructional Design and Pedagogical Quality	1.3 Quality of Embedded Feedback	Claude 3.7 Sonnet	5	5	5
**78**	CML	Instructional Design and Pedagogical Quality	1.3 Quality of Embedded Feedback	Claude 3.7 Sonnet	4	4	4
**79**	CML	Instructional Design and Pedagogical Quality	1.3 Quality of Embedded Feedback	Claude 3.7 Sonnet	2	2	2
**80**	CML	Instructional Design and Pedagogical Quality	1.3 Quality of Embedded Feedback	Claude 3.7 Sonnet	5	5	5
**81**	CML	Instructional Design and Pedagogical Quality	1.3 Quality of Embedded Feedback	Claude 3.7 Sonnet	5	5	5
**82**	CML	Instructional Design and Pedagogical Quality	1.3 Quality of Embedded Feedback	Claude 3.7 Sonnet	5	5	5
**83**	CML	Instructional Design and Pedagogical Quality	1.3 Quality of Embedded Feedback	Claude 3.7 Sonnet	5	5	5
**84**	CML	Instructional Design and Pedagogical Quality	1.3 Quality of Embedded Feedback	Claude 3.7 Sonnet	4	4	4
**85**	CML	Instructional Design and Pedagogical Quality	1.3 Quality of Embedded Feedback	Claude 3.7 Sonnet	5	1	5
**86**	CML	Instructional Design and Pedagogical Quality	1.3 Quality of Embedded Feedback	Claude 3.7 Sonnet	5	5	5
**87**	CML	Instructional Design and Pedagogical Quality	1.3 Quality of Embedded Feedback	GPT 4.o	3	3	3
**88**	CML	Instructional Design and Pedagogical Quality	1.3 Quality of Embedded Feedback	GPT 4.o	5	5	5
**89**	CML	Instructional Design and Pedagogical Quality	1.3 Quality of Embedded Feedback	GPT 4.o	3	1	3

Supplementary D

**90**	CML	Instructional Design and Pedagogical Quality	1.3 Quality of Embedded Feedback	GPT 4.o	5	3	5
**91**	CML	Instructional Design and Pedagogical Quality	1.3 Quality of Embedded Feedback	GPT 4.o	4	4	4
**92**	CML	Instructional Design and Pedagogical Quality	1.3 Quality of Embedded Feedback	GPT 4.o	5	5	5
**93**	CML	Instructional Design and Pedagogical Quality	1.3 Quality of Embedded Feedback	GPT 4.o	5	5	5
**94**	CML	Instructional Design and Pedagogical Quality	1.3 Quality of Embedded Feedback	GPT 4.o	5	1	5
**95**	CML	Instructional Design and Pedagogical Quality	1.3 Quality of Embedded Feedback	GPT 4.o	4	4	4
**96**	CML	Instructional Design and Pedagogical Quality	1.3 Quality of Embedded Feedback	GPT 4.o	2	2	2
**97**	CML	Instructional Design and Pedagogical Quality	1.3 Quality of Embedded Feedback	GPT 4.o	5	5	5
**98**	CML	Instructional Design and Pedagogical Quality	1.3 Quality of Embedded Feedback	GPT 4.o	5	5	5
**99**	CML	Instructional Design and Pedagogical Quality	1.3 Quality of Embedded Feedback	GPT 4.o	5	5	5
**100**	CML	Instructional Design and Pedagogical Quality	1.3 Quality of Embedded Feedback	GPT 4.o	3	1	3
**101**	CML	Instructional Design and Pedagogical Quality	1.3 Quality of Embedded Feedback	GPT 4.o	5	5	5
**102**	CML	Instructional Design and Pedagogical Quality	1.3 Quality of Embedded Feedback	GPT 4.o	5	3	5
**103**	CML	Instructional Design and Pedagogical Quality	1.3 Quality of Embedded Feedback	GPT 4.o	5	5	5
**51**	CML	Instructional Design and Pedagogical Quality	1.4 Instructional Clarity & Framing	Gemini 2.0 Pro	5	5	5
**52**	CML	Instructional Design and Pedagogical Quality	1.4 Instructional Clarity & Framing	Gemini 2.0 Pro	5	5	5
**53**	CML	Instructional Design and Pedagogical Quality	1.4 Instructional Clarity & Framing	Gemini 2.0 Pro	5	5	5
**54**	CML	Instructional Design and Pedagogical Quality	1.4 Instructional Clarity & Framing	Gemini 2.0 Pro	5	5	5
**55**	CML	Instructional Design and Pedagogical Quality	1.4 Instructional Clarity & Framing	Gemini 2.0 Pro	5	5	5
**56**	CML	Instructional Design and Pedagogical Quality	1.4 Instructional Clarity & Framing	Gemini 2.0 Pro	5	5	5
**57**	CML	Instructional Design and Pedagogical Quality	1.4 Instructional Clarity & Framing	Gemini 2.0 Pro	5	5	5
**58**	CML	Instructional Design and Pedagogical Quality	1.4 Instructional Clarity & Framing	Gemini 2.0 Pro	5	5	5
**59**	CML	Instructional Design and Pedagogical Quality	1.4 Instructional Clarity & Framing	Gemini 2.0 Pro	5	5	5
**60**	CML	Instructional Design and Pedagogical Quality	1.4 Instructional Clarity & Framing	Gemini 2.0 Pro	5	5	5
**61**	CML	Instructional Design and Pedagogical Quality	1.4 Instructional Clarity & Framing	Gemini 2.0 Pro	5	5	5
**62**	CML	Instructional Design and Pedagogical Quality	1.4 Instructional Clarity & Framing	Gemini 2.0 Pro	5	5	5
**63**	CML	Instructional Design and Pedagogical Quality	1.4 Instructional Clarity & Framing	DeepSeek V2	5	5	5

Supplementary D

**64**	CML	Instructional Design and Pedagogical Quality	1.4 Instructional Clarity & Framing	DeepSeek V2	5	5	5
**65**	CML	Instructional Design and Pedagogical Quality	1.4 Instructional Clarity & Framing	DeepSeek V2	5	5	5
**66**	CML	Instructional Design and Pedagogical Quality	1.4 Instructional Clarity & Framing	DeepSeek V2	5	5	5
**67**	CML	Instructional Design and Pedagogical Quality	1.4 Instructional Clarity & Framing	DeepSeek V2	5	5	5
**68**	CML	Instructional Design and Pedagogical Quality	1.4 Instructional Clarity & Framing	DeepSeek V2	5	5	5
**69**	CML	Instructional Design and Pedagogical Quality	1.4 Instructional Clarity & Framing	DeepSeek V2	5	5	5
**70**	CML	Instructional Design and Pedagogical Quality	1.4 Instructional Clarity & Framing	DeepSeek V2	5	5	5
**71**	CML	Instructional Design and Pedagogical Quality	1.4 Instructional Clarity & Framing	DeepSeek V2	5	5	5
**72**	CML	Instructional Design and Pedagogical Quality	1.4 Instructional Clarity & Framing	DeepSeek V2	4	4	4
**73**	CML	Instructional Design and Pedagogical Quality	1.4 Instructional Clarity & Framing	DeepSeek V2	5	5	5
**74**	CML	Instructional Design and Pedagogical Quality	1.4 Instructional Clarity & Framing	DeepSeek V2	5	5	5
**75**	CML	Instructional Design and Pedagogical Quality	1.4 Instructional Clarity & Framing	DeepSeek V2	5	5	5
**76**	CML	Instructional Design and Pedagogical Quality	1.4 Instructional Clarity & Framing	Claude 3.7 Sonnet	5	5	5
**77**	CML	Instructional Design and Pedagogical Quality	1.4 Instructional Clarity & Framing	Claude 3.7 Sonnet	5	5	5
**78**	CML	Instructional Design and Pedagogical Quality	1.4 Instructional Clarity & Framing	Claude 3.7 Sonnet	5	5	5
**79**	CML	Instructional Design and Pedagogical Quality	1.4 Instructional Clarity & Framing	Claude 3.7 Sonnet	3	3	3
**80**	CML	Instructional Design and Pedagogical Quality	1.4 Instructional Clarity & Framing	Claude 3.7 Sonnet	5	5	5
**81**	CML	Instructional Design and Pedagogical Quality	1.4 Instructional Clarity & Framing	Claude 3.7 Sonnet	5	5	5
**82**	CML	Instructional Design and Pedagogical Quality	1.4 Instructional Clarity & Framing	Claude 3.7 Sonnet	5	5	5
**83**	CML	Instructional Design and Pedagogical Quality	1.4 Instructional Clarity & Framing	Claude 3.7 Sonnet	5	5	5
**84**	CML	Instructional Design and Pedagogical Quality	1.4 Instructional Clarity & Framing	Claude 3.7 Sonnet	5	5	5
**85**	CML	Instructional Design and Pedagogical Quality	1.4 Instructional Clarity & Framing	Claude 3.7 Sonnet	5	5	5

Supplementary D

**86**	CML	Instructional Design and Pedagogical Quality	1.4 Instructional Clarity & Framing	Claude 3.7 Sonnet	5	5	5
**87**	CML	Instructional Design and Pedagogical Quality	1.4 Instructional Clarity & Framing	GPT 4.o	4	4	4
**88**	CML	Instructional Design and Pedagogical Quality	1.4 Instructional Clarity & Framing	GPT 4.o	5	5	5
**89**	CML	Instructional Design and Pedagogical Quality	1.4 Instructional Clarity & Framing	GPT 4.o	5	5	5
**90**	CML	Instructional Design and Pedagogical Quality	1.4 Instructional Clarity & Framing	GPT 4.o	5	5	5
**91**	CML	Instructional Design and Pedagogical Quality	1.4 Instructional Clarity & Framing	GPT 4.o	5	5	5
**92**	CML	Instructional Design and Pedagogical Quality	1.4 Instructional Clarity & Framing	GPT 4.o	5	5	5
**93**	CML	Instructional Design and Pedagogical Quality	1.4 Instructional Clarity & Framing	GPT 4.o	5	5	5
**94**	CML	Instructional Design and Pedagogical Quality	1.4 Instructional Clarity & Framing	GPT 4.o	5	5	5
**95**	CML	Instructional Design and Pedagogical Quality	1.4 Instructional Clarity & Framing	GPT 4.o	5	5	5
**96**	CML	Instructional Design and Pedagogical Quality	1.4 Instructional Clarity & Framing	GPT 4.o	5	1	5
**97**	CML	Instructional Design and Pedagogical Quality	1.4 Instructional Clarity & Framing	GPT 4.o	5	5	5
**98**	CML	Instructional Design and Pedagogical Quality	1.4 Instructional Clarity & Framing	GPT 4.o	5	5	5
**99**	CML	Instructional Design and Pedagogical Quality	1.4 Instructional Clarity & Framing	GPT 4.o	5	5	5
**100**	CML	Instructional Design and Pedagogical Quality	1.4 Instructional Clarity & Framing	GPT 4.o	5	3	5
**101**	CML	Instructional Design and Pedagogical Quality	1.4 Instructional Clarity & Framing	GPT 4.o	5	5	5
**102**	CML	Instructional Design and Pedagogical Quality	1.4 Instructional Clarity & Framing	GPT 4.o	5	4	5
**103**	CML	Instructional Design and Pedagogical Quality	1.4 Instructional Clarity & Framing	GPT 4.o	5	5	5
**51**	CML	Clinical Authenticity and Content Fidelity	2.1 Clinical Narrative Plausibility	Gemini 2.0 Pro	5	5	5
**52**	CML	Clinical Authenticity and Content Fidelity	2.1 Clinical Narrative Plausibility	Gemini 2.0 Pro	5	5	5
**53**	CML	Clinical Authenticity and Content Fidelity	2.1 Clinical Narrative Plausibility	Gemini 2.0 Pro	5	5	5
**54**	CML	Clinical Authenticity and Content Fidelity	2.1 Clinical Narrative Plausibility	Gemini 2.0 Pro	5	5	5
**55**	CML	Clinical Authenticity and Content Fidelity	2.1 Clinical Narrative Plausibility	Gemini 2.0 Pro	5	5	5
**56**	CML	Clinical Authenticity and Content Fidelity	2.1 Clinical Narrative Plausibility	Gemini 2.0 Pro	5	5	5
**57**	CML	Clinical Authenticity and Content Fidelity	2.1 Clinical Narrative Plausibility	Gemini 2.0 Pro	5	5	5
**58**	CML	Clinical Authenticity and Content Fidelity	2.1 Clinical Narrative Plausibility	Gemini 2.0 Pro	5	5	5
**59**	CML	Clinical Authenticity and Content Fidelity	2.1 Clinical Narrative Plausibility	Gemini 2.0 Pro	5	5	5

**60**	CML	Clinical Authenticity and Content Fidelity	2.1 Clinical Narrative Plausibility	Gemini 2.0 Pro	5	5	5
**61**	CML	Clinical Authenticity and Content Fidelity	2.1 Clinical Narrative Plausibility	Gemini 2.0 Pro	5	5	5
**62**	CML	Clinical Authenticity and Content Fidelity	2.1 Clinical Narrative Plausibility	Gemini 2.0 Pro	5	5	5
**63**	CML	Clinical Authenticity and Content Fidelity	2.1 Clinical Narrative Plausibility	DeepSeek V2	5	5	5
**64**	CML	Clinical Authenticity and Content Fidelity	2.1 Clinical Narrative Plausibility	DeepSeek V2	5	5	5
**65**	CML	Clinical Authenticity and Content Fidelity	2.1 Clinical Narrative Plausibility	DeepSeek V2	5	5	5
**66**	CML	Clinical Authenticity and Content Fidelity	2.1 Clinical Narrative Plausibility	DeepSeek V2	5	5	5
**67**	CML	Clinical Authenticity and Content Fidelity	2.1 Clinical Narrative Plausibility	DeepSeek V2	5	5	5
**68**	CML	Clinical Authenticity and Content Fidelity	2.1 Clinical Narrative Plausibility	DeepSeek V2	5	5	5
**69**	CML	Clinical Authenticity and Content Fidelity	2.1 Clinical Narrative Plausibility	DeepSeek V2	5	5	5
**70**	CML	Clinical Authenticity and Content Fidelity	2.1 Clinical Narrative Plausibility	DeepSeek V2	5	5	5
**71**	CML	Clinical Authenticity and Content Fidelity	2.1 Clinical Narrative Plausibility	DeepSeek V2	5	5	5
**72**	CML	Clinical Authenticity and Content Fidelity	2.1 Clinical Narrative Plausibility	DeepSeek V2	5	5	5
**73**	CML	Clinical Authenticity and Content Fidelity	2.1 Clinical Narrative Plausibility	DeepSeek V2	5	5	5
**74**	CML	Clinical Authenticity and Content Fidelity	2.1 Clinical Narrative Plausibility	DeepSeek V2	5	5	5
**75**	CML	Clinical Authenticity and Content Fidelity	2.1 Clinical Narrative Plausibility	DeepSeek V2	2	2	2
**76**	CML	Clinical Authenticity and Content Fidelity	2.1 Clinical Narrative Plausibility	Claude 3.7 Sonnet	5	5	5
**77**	CML	Clinical Authenticity and Content Fidelity	2.1 Clinical Narrative Plausibility	Claude 3.7 Sonnet	5	5	5
**78**	CML	Clinical Authenticity and Content Fidelity	2.1 Clinical Narrative Plausibility	Claude 3.7 Sonnet	5	5	5
**79**	CML	Clinical Authenticity and Content Fidelity	2.1 Clinical Narrative Plausibility	Claude 3.7 Sonnet	5	5	5
**80**	CML	Clinical Authenticity and Content Fidelity	2.1 Clinical Narrative Plausibility	Claude 3.7 Sonnet	5	5	5
**81**	CML	Clinical Authenticity and Content Fidelity	2.1 Clinical Narrative Plausibility	Claude 3.7 Sonnet	5	5	5
**82**	CML	Clinical Authenticity and Content Fidelity	2.1 Clinical Narrative Plausibility	Claude 3.7 Sonnet	5	5	5

Supplementary D

**83**	CML	Clinical Authenticity and Content Fidelity	2.1 Clinical Narrative Plausibility	Claude 3.7 Sonnet	5	5	5
**84**	CML	Clinical Authenticity and Content Fidelity	2.1 Clinical Narrative Plausibility	Claude 3.7 Sonnet	5	5	5
**85**	CML	Clinical Authenticity and Content Fidelity	2.1 Clinical Narrative Plausibility	Claude 3.7 Sonnet	5	5	5
**86**	CML	Clinical Authenticity and Content Fidelity	2.1 Clinical Narrative Plausibility	Claude 3.7 Sonnet	5	5	5
**87**	CML	Clinical Authenticity and Content Fidelity	2.1 Clinical Narrative Plausibility	GPT 4.o	5	5	5
**88**	CML	Clinical Authenticity and Content Fidelity	2.1 Clinical Narrative Plausibility	GPT 4.o	5	5	5
**89**	CML	Clinical Authenticity and Content Fidelity	2.1 Clinical Narrative Plausibility	GPT 4.o	5	5	5
**90**	CML	Clinical Authenticity and Content Fidelity	2.1 Clinical Narrative Plausibility	GPT 4.o	5	5	5
**91**	CML	Clinical Authenticity and Content Fidelity	2.1 Clinical Narrative Plausibility	GPT 4.o	5	5	5
**92**	CML	Clinical Authenticity and Content Fidelity	2.1 Clinical Narrative Plausibility	GPT 4.o	5	5	5
**93**	CML	Clinical Authenticity and Content Fidelity	2.1 Clinical Narrative Plausibility	GPT 4.o	5	5	5
**94**	CML	Clinical Authenticity and Content Fidelity	2.1 Clinical Narrative Plausibility	GPT 4.o	5	5	5
**95**	CML	Clinical Authenticity and Content Fidelity	2.1 Clinical Narrative Plausibility	GPT 4.o	5	5	5
**96**	CML	Clinical Authenticity and Content Fidelity	2.1 Clinical Narrative Plausibility	GPT 4.o	5	5	5
**97**	CML	Clinical Authenticity and Content Fidelity	2.1 Clinical Narrative Plausibility	GPT 4.o	5	5	5
**98**	CML	Clinical Authenticity and Content Fidelity	2.1 Clinical Narrative Plausibility	GPT 4.o	5	4	5
**99**	CML	Clinical Authenticity and Content Fidelity	2.1 Clinical Narrative Plausibility	GPT 4.o	5	5	5
**100**	CML	Clinical Authenticity and Content Fidelity	2.1 Clinical Narrative Plausibility	GPT 4.o	5	4	5
**101**	CML	Clinical Authenticity and Content Fidelity	2.1 Clinical Narrative Plausibility	GPT 4.o	5	5	5
**102**	CML	Clinical Authenticity and Content Fidelity	2.1 Clinical Narrative Plausibility	GPT 4.o	5	5	5
**103**	CML	Clinical Authenticity and Content Fidelity	2.1 Clinical Narrative Plausibility	GPT 4.o	5	5	5
**51**	CML	Clinical Authenticity and Content Fidelity	2.2 Alignment with Clinical Guidelines	Gemini 2.0 Pro	3	3	3
**52**	CML	Clinical Authenticity and Content Fidelity	2.2 Alignment with Clinical Guidelines	Gemini 2.0 Pro	5	5	5
**53**	CML	Clinical Authenticity and Content Fidelity	2.2 Alignment with Clinical Guidelines	Gemini 2.0 Pro	3	3	3
**54**	CML	Clinical Authenticity and Content Fidelity	2.2 Alignment with Clinical Guidelines	Gemini 2.0 Pro	5	5	5

Supplementary D

**55**	CML	Clinical Authenticity and Content Fidelity	2.2 Alignment with Clinical Guidelines	Gemini 2.0 Pro	5	5	5
**56**	CML	Clinical Authenticity and Content Fidelity	2.2 Alignment with Clinical Guidelines	Gemini 2.0 Pro	2	2	2
**57**	CML	Clinical Authenticity and Content Fidelity	2.2 Alignment with Clinical Guidelines	Gemini 2.0 Pro	5	5	5
**58**	CML	Clinical Authenticity and Content Fidelity	2.2 Alignment with Clinical Guidelines	Gemini 2.0 Pro	5	5	5
**59**	CML	Clinical Authenticity and Content Fidelity	2.2 Alignment with Clinical Guidelines	Gemini 2.0 Pro	5	5	5
**60**	CML	Clinical Authenticity and Content Fidelity	2.2 Alignment with Clinical Guidelines	Gemini 2.0 Pro	5	5	5
**61**	CML	Clinical Authenticity and Content Fidelity	2.2 Alignment with Clinical Guidelines	Gemini 2.0 Pro	5	5	5
**62**	CML	Clinical Authenticity and Content Fidelity	2.2 Alignment with Clinical Guidelines	Gemini 2.0 Pro	5	5	5
**63**	CML	Clinical Authenticity and Content Fidelity	2.2 Alignment with Clinical Guidelines	DeepSeek V2	5	5	5
**64**	CML	Clinical Authenticity and Content Fidelity	2.2 Alignment with Clinical Guidelines	DeepSeek V2	5	5	5
**65**	CML	Clinical Authenticity and Content Fidelity	2.2 Alignment with Clinical Guidelines	DeepSeek V2	5	5	5
**66**	CML	Clinical Authenticity and Content Fidelity	2.2 Alignment with Clinical Guidelines	DeepSeek V2	5	5	5
**67**	CML	Clinical Authenticity and Content Fidelity	2.2 Alignment with Clinical Guidelines	DeepSeek V2	5	5	5
**68**	CML	Clinical Authenticity and Content Fidelity	2.2 Alignment with Clinical Guidelines	DeepSeek V2	5	5	5
**69**	CML	Clinical Authenticity and Content Fidelity	2.2 Alignment with Clinical Guidelines	DeepSeek V2	5	5	5
**70**	CML	Clinical Authenticity and Content Fidelity	2.2 Alignment with Clinical Guidelines	DeepSeek V2	5	5	5
**71**	CML	Clinical Authenticity and Content Fidelity	2.2 Alignment with Clinical Guidelines	DeepSeek V2	5	5	5
**72**	CML	Clinical Authenticity and Content Fidelity	2.2 Alignment with Clinical Guidelines	DeepSeek V2	5	5	5
**73**	CML	Clinical Authenticity and Content Fidelity	2.2 Alignment with Clinical Guidelines	DeepSeek V2	2	2	2
**74**	CML	Clinical Authenticity and Content Fidelity	2.2 Alignment with Clinical Guidelines	DeepSeek V2	5	5	5
**75**	CML	Clinical Authenticity and Content Fidelity	2.2 Alignment with Clinical Guidelines	DeepSeek V2	2	2	2
**76**	CML	Clinical Authenticity and Content Fidelity	2.2 Alignment with Clinical Guidelines	Claude 3.7 Sonnet	5	5	5
**77**	CML	Clinical Authenticity and Content Fidelity	2.2 Alignment with Clinical Guidelines	Claude 3.7 Sonnet	5	5	5
**78**	CML	Clinical Authenticity and Content Fidelity	2.2 Alignment with Clinical Guidelines	Claude 3.7 Sonnet	2	2	2
**79**	CML	Clinical Authenticity and Content Fidelity	2.2 Alignment with Clinical Guidelines	Claude 3.7 Sonnet	2	2	2

Supplementary D

**80**	CML	Clinical Authenticity and Content Fidelity	2.2 Alignment with Clinical Guidelines	Claude 3.7 Sonnet	5	5	5
**81**	CML	Clinical Authenticity and Content Fidelity	2.2 Alignment with Clinical Guidelines	Claude 3.7 Sonnet	4	4	4
**82**	CML	Clinical Authenticity and Content Fidelity	2.2 Alignment with Clinical Guidelines	Claude 3.7 Sonnet	5	5	5
**83**	CML	Clinical Authenticity and Content Fidelity	2.2 Alignment with Clinical Guidelines	Claude 3.7 Sonnet	5	5	5
**84**	CML	Clinical Authenticity and Content Fidelity	2.2 Alignment with Clinical Guidelines	Claude 3.7 Sonnet	4	4	4
**85**	CML	Clinical Authenticity and Content Fidelity	2.2 Alignment with Clinical Guidelines	Claude 3.7 Sonnet	5	5	5
**86**	CML	Clinical Authenticity and Content Fidelity	2.2 Alignment with Clinical Guidelines	Claude 3.7 Sonnet	5	5	5
**87**	CML	Clinical Authenticity and Content Fidelity	2.2 Alignment with Clinical Guidelines	GPT 4.o	4	4	4
**88**	CML	Clinical Authenticity and Content Fidelity	2.2 Alignment with Clinical Guidelines	GPT 4.o	5	5	5
**89**	CML	Clinical Authenticity and Content Fidelity	2.2 Alignment with Clinical Guidelines	GPT 4.o	4	4	4
**90**	CML	Clinical Authenticity and Content Fidelity	2.2 Alignment with Clinical Guidelines	GPT 4.o	2	2	2
**91**	CML	Clinical Authenticity and Content Fidelity	2.2 Alignment with Clinical Guidelines	GPT 4.o	2	2	2
**92**	CML	Clinical Authenticity and Content Fidelity	2.2 Alignment with Clinical Guidelines	GPT 4.o	5	5	5
**93**	CML	Clinical Authenticity and Content Fidelity	2.2 Alignment with Clinical Guidelines	GPT 4.o	5	5	5
**94**	CML	Clinical Authenticity and Content Fidelity	2.2 Alignment with Clinical Guidelines	GPT 4.o	5	5	5
**95**	CML	Clinical Authenticity and Content Fidelity	2.2 Alignment with Clinical Guidelines	GPT 4.o	4	4	4
**96**	CML	Clinical Authenticity and Content Fidelity	2.2 Alignment with Clinical Guidelines	GPT 4.o	2	2	2
**97**	CML	Clinical Authenticity and Content Fidelity	2.2 Alignment with Clinical Guidelines	GPT 4.o	3	3	3
**98**	CML	Clinical Authenticity and Content Fidelity	2.2 Alignment with Clinical Guidelines	GPT 4.o	5	4	5
**99**	CML	Clinical Authenticity and Content Fidelity	2.2 Alignment with Clinical Guidelines	GPT 4.o	3	3	3
**100**	CML	Clinical Authenticity and Content Fidelity	2.2 Alignment with Clinical Guidelines	GPT 4.o	2	3	2
**101**	CML	Clinical Authenticity and Content Fidelity	2.2 Alignment with Clinical Guidelines	GPT 4.o	5	5	5
**102**	CML	Clinical Authenticity and Content Fidelity	2.2 Alignment with Clinical Guidelines	GPT 4.o	4	4	4
**103**	CML	Clinical Authenticity and Content Fidelity	2.2 Alignment with Clinical Guidelines	GPT 4.o	5	5	5

Supplementary D

**51**	CML	Clinical Authenticity and Content Fidelity	2.3 Pharmacotherapeutic Accuracy	Gemini 2.0 Pro	5	5	5
**52**	CML	Clinical Authenticity and Content Fidelity	2.3 Pharmacotherapeutic Accuracy	Gemini 2.0 Pro	5	5	5
**53**	CML	Clinical Authenticity and Content Fidelity	2.3 Pharmacotherapeutic Accuracy	Gemini 2.0 Pro	2	2	2
**54**	CML	Clinical Authenticity and Content Fidelity	2.3 Pharmacotherapeutic Accuracy	Gemini 2.0 Pro	5	5	5
**55**	CML	Clinical Authenticity and Content Fidelity	2.3 Pharmacotherapeutic Accuracy	Gemini 2.0 Pro	5	5	5
**56**	CML	Clinical Authenticity and Content Fidelity	2.3 Pharmacotherapeutic Accuracy	Gemini 2.0 Pro	2	2	2
**57**	CML	Clinical Authenticity and Content Fidelity	2.3 Pharmacotherapeutic Accuracy	Gemini 2.0 Pro	5	5	5
**58**	CML	Clinical Authenticity and Content Fidelity	2.3 Pharmacotherapeutic Accuracy	Gemini 2.0 Pro	5	5	5
**59**	CML	Clinical Authenticity and Content Fidelity	2.3 Pharmacotherapeutic Accuracy	Gemini 2.0 Pro	5	5	5
**60**	CML	Clinical Authenticity and Content Fidelity	2.3 Pharmacotherapeutic Accuracy	Gemini 2.0 Pro	5	5	5
**61**	CML	Clinical Authenticity and Content Fidelity	2.3 Pharmacotherapeutic Accuracy	Gemini 2.0 Pro	5	5	5
**62**	CML	Clinical Authenticity and Content Fidelity	2.3 Pharmacotherapeutic Accuracy	Gemini 2.0 Pro	5	5	5
**63**	CML	Clinical Authenticity and Content Fidelity	2.3 Pharmacotherapeutic Accuracy	DeepSeek V2	5	5	5
**64**	CML	Clinical Authenticity and Content Fidelity	2.3 Pharmacotherapeutic Accuracy	DeepSeek V2	5	5	5
**65**	CML	Clinical Authenticity and Content Fidelity	2.3 Pharmacotherapeutic Accuracy	DeepSeek V2	5	5	5
**66**	CML	Clinical Authenticity and Content Fidelity	2.3 Pharmacotherapeutic Accuracy	DeepSeek V2	5	5	5
**67**	CML	Clinical Authenticity and Content Fidelity	2.3 Pharmacotherapeutic Accuracy	DeepSeek V2	5	5	5
**68**	CML	Clinical Authenticity and Content Fidelity	2.3 Pharmacotherapeutic Accuracy	DeepSeek V2	5	5	5

Supplementary D

**69**	CML	Clinical Authenticity and Content Fidelity	2.3 Pharmacotherapeutic Accuracy	DeepSeek V2	5	5	5
**70**	CML	Clinical Authenticity and Content Fidelity	2.3 Pharmacotherapeutic Accuracy	DeepSeek V2	5	5	5
**71**	CML	Clinical Authenticity and Content Fidelity	2.3 Pharmacotherapeutic Accuracy	DeepSeek V2	5	5	5
**72**	CML	Clinical Authenticity and Content Fidelity	2.3 Pharmacotherapeutic Accuracy	DeepSeek V2	5	5	5
**73**	CML	Clinical Authenticity and Content Fidelity	2.3 Pharmacotherapeutic Accuracy	DeepSeek V2	2	2	2
**74**	CML	Clinical Authenticity and Content Fidelity	2.3 Pharmacotherapeutic Accuracy	DeepSeek V2	5	5	5
**75**	CML	Clinical Authenticity and Content Fidelity	2.3 Pharmacotherapeutic Accuracy	DeepSeek V2	2	2	2
**76**	CML	Clinical Authenticity and Content Fidelity	2.3 Pharmacotherapeutic Accuracy	Claude 3.7 Sonnet	5	5	5
**77**	CML	Clinical Authenticity and Content Fidelity	2.3 Pharmacotherapeutic Accuracy	Claude 3.7 Sonnet	5	5	5
**78**	CML	Clinical Authenticity and Content Fidelity	2.3 Pharmacotherapeutic Accuracy	Claude 3.7 Sonnet	2	2	2
**79**	CML	Clinical Authenticity and Content Fidelity	2.3 Pharmacotherapeutic Accuracy	Claude 3.7 Sonnet	2	2	2
**80**	CML	Clinical Authenticity and Content Fidelity	2.3 Pharmacotherapeutic Accuracy	Claude 3.7 Sonnet	5	5	5
**81**	CML	Clinical Authenticity and Content Fidelity	2.3 Pharmacotherapeutic Accuracy	Claude 3.7 Sonnet	4	4	4
**82**	CML	Clinical Authenticity and Content Fidelity	2.3 Pharmacotherapeutic Accuracy	Claude 3.7 Sonnet	5	5	5
**83**	CML	Clinical Authenticity and Content Fidelity	2.3 Pharmacotherapeutic Accuracy	Claude 3.7 Sonnet	5	5	5
**84**	CML	Clinical Authenticity and Content Fidelity	2.3 Pharmacotherapeutic Accuracy	Claude 3.7 Sonnet	2	2	2
**85**	CML	Clinical Authenticity and Content Fidelity	2.3 Pharmacotherapeutic Accuracy	Claude 3.7 Sonnet	5	5	5
**86**	CML	Clinical Authenticity and Content Fidelity	2.3 Pharmacotherapeutic Accuracy	Claude 3.7 Sonnet	5	5	5

Supplementary D

**87**	CML	Clinical Authenticity and Content Fidelity	2.3 Pharmacotherapeutic Accuracy	GPT 4.o	3	3	3
**88**	CML	Clinical Authenticity and Content Fidelity	2.3 Pharmacotherapeutic Accuracy	GPT 4.o	5	5	5
**89**	CML	Clinical Authenticity and Content Fidelity	2.3 Pharmacotherapeutic Accuracy	GPT 4.o	3	3	3
**90**	CML	Clinical Authenticity and Content Fidelity	2.3 Pharmacotherapeutic Accuracy	GPT 4.o	2	2	2
**91**	CML	Clinical Authenticity and Content Fidelity	2.3 Pharmacotherapeutic Accuracy	GPT 4.o	2	2	2
**92**	CML	Clinical Authenticity and Content Fidelity	2.3 Pharmacotherapeutic Accuracy	GPT 4.o	4	4	4
**93**	CML	Clinical Authenticity and Content Fidelity	2.3 Pharmacotherapeutic Accuracy	GPT 4.o	5	5	5
**94**	CML	Clinical Authenticity and Content Fidelity	2.3 Pharmacotherapeutic Accuracy	GPT 4.o	5	5	5
**95**	CML	Clinical Authenticity and Content Fidelity	2.3 Pharmacotherapeutic Accuracy	GPT 4.o	2	3	2
**96**	CML	Clinical Authenticity and Content Fidelity	2.3 Pharmacotherapeutic Accuracy	GPT 4.o	2	2	2
**97**	CML	Clinical Authenticity and Content Fidelity	2.3 Pharmacotherapeutic Accuracy	GPT 4.o	2	2	2
**98**	CML	Clinical Authenticity and Content Fidelity	2.3 Pharmacotherapeutic Accuracy	GPT 4.o	5	4	5
**99**	CML	Clinical Authenticity and Content Fidelity	2.3 Pharmacotherapeutic Accuracy	GPT 4.o	2	2	2
**100**	CML	Clinical Authenticity and Content Fidelity	2.3 Pharmacotherapeutic Accuracy	GPT 4.o	2	3	2
**101**	CML	Clinical Authenticity and Content Fidelity	2.3 Pharmacotherapeutic Accuracy	GPT 4.o	5	5	5
**102**	CML	Clinical Authenticity and Content Fidelity	2.3 Pharmacotherapeutic Accuracy	GPT 4.o	3	3	3
**103**	CML	Clinical Authenticity and Content Fidelity	2.3 Pharmacotherapeutic Accuracy	GPT 4.o	5	5	5
**51**	CML	Clinical Authenticity and Content Fidelity	2.4 Domain Specificity	Gemini 2.0 Pro	5	5	5
**52**	CML	Clinical Authenticity and Content Fidelity	2.4 Domain Specificity	Gemini 2.0 Pro	5	5	5

Supplementary D

**53**	CML	Clinical Authenticity and Content Fidelity	2.4 Domain Specificity	Gemini 2.0 Pro	5	5	5
**54**	CML	Clinical Authenticity and Content Fidelity	2.4 Domain Specificity	Gemini 2.0 Pro	5	5	5
**55**	CML	Clinical Authenticity and Content Fidelity	2.4 Domain Specificity	Gemini 2.0 Pro	5	5	5
**56**	CML	Clinical Authenticity and Content Fidelity	2.4 Domain Specificity	Gemini 2.0 Pro	5	5	5
**57**	CML	Clinical Authenticity and Content Fidelity	2.4 Domain Specificity	Gemini 2.0 Pro	5	5	5
**58**	CML	Clinical Authenticity and Content Fidelity	2.4 Domain Specificity	Gemini 2.0 Pro	5	5	5
**59**	CML	Clinical Authenticity and Content Fidelity	2.4 Domain Specificity	Gemini 2.0 Pro	5	5	3
**60**	CML	Clinical Authenticity and Content Fidelity	2.4 Domain Specificity	Gemini 2.0 Pro	5	5	5
**61**	CML	Clinical Authenticity and Content Fidelity	2.4 Domain Specificity	Gemini 2.0 Pro	5	5	5
**62**	CML	Clinical Authenticity and Content Fidelity	2.4 Domain Specificity	Gemini 2.0 Pro	5	5	5
**63**	CML	Clinical Authenticity and Content Fidelity	2.4 Domain Specificity	DeepSeek V2	5	5	5
**64**	CML	Clinical Authenticity and Content Fidelity	2.4 Domain Specificity	DeepSeek V2	5	5	5
**65**	CML	Clinical Authenticity and Content Fidelity	2.4 Domain Specificity	DeepSeek V2	5	5	5
**66**	CML	Clinical Authenticity and Content Fidelity	2.4 Domain Specificity	DeepSeek V2	5	5	5
**67**	CML	Clinical Authenticity and Content Fidelity	2.4 Domain Specificity	DeepSeek V2	5	5	5
**68**	CML	Clinical Authenticity and Content Fidelity	2.4 Domain Specificity	DeepSeek V2	5	5	5
**69**	CML	Clinical Authenticity and Content Fidelity	2.4 Domain Specificity	DeepSeek V2	5	5	5
**70**	CML	Clinical Authenticity and Content Fidelity	2.4 Domain Specificity	DeepSeek V2	5	5	5
**71**	CML	Clinical Authenticity and Content Fidelity	2.4 Domain Specificity	DeepSeek V2	5	5	5
**72**	CML	Clinical Authenticity and Content Fidelity	2.4 Domain Specificity	DeepSeek V2	5	5	5
**73**	CML	Clinical Authenticity and Content Fidelity	2.4 Domain Specificity	DeepSeek V2	5	5	5
**74**	CML	Clinical Authenticity and Content Fidelity	2.4 Domain Specificity	DeepSeek V2	5	5	5
**75**	CML	Clinical Authenticity and Content Fidelity	2.4 Domain Specificity	DeepSeek V2	4	4	4
**76**	CML	Clinical Authenticity and Content Fidelity	2.4 Domain Specificity	Claude 3.7 Sonnet	5	5	5
**77**	CML	Clinical Authenticity and Content Fidelity	2.4 Domain Specificity	Claude 3.7 Sonnet	5	5	5
**78**	CML	Clinical Authenticity and Content Fidelity	2.4 Domain Specificity	Claude 3.7 Sonnet	5	5	5

Supplementary D

**79**	CML	Clinical Authenticity and Content Fidelity	2.4 Domain Specificity	Claude 3.7 Sonnet	5	5	5
**80**	CML	Clinical Authenticity and Content Fidelity	2.4 Domain Specificity	Claude 3.7 Sonnet	5	5	5
**81**	CML	Clinical Authenticity and Content Fidelity	2.4 Domain Specificity	Claude 3.7 Sonnet	5	5	5
**82**	CML	Clinical Authenticity and Content Fidelity	2.4 Domain Specificity	Claude 3.7 Sonnet	5	5	5
**83**	CML	Clinical Authenticity and Content Fidelity	2.4 Domain Specificity	Claude 3.7 Sonnet	5	5	5
**84**	CML	Clinical Authenticity and Content Fidelity	2.4 Domain Specificity	Claude 3.7 Sonnet	5	5	5
**85**	CML	Clinical Authenticity and Content Fidelity	2.4 Domain Specificity	Claude 3.7 Sonnet	5	5	5
**86**	CML	Clinical Authenticity and Content Fidelity	2.4 Domain Specificity	Claude 3.7 Sonnet	5	5	5
**87**	CML	Clinical Authenticity and Content Fidelity	2.4 Domain Specificity	GPT 4.o	5	5	5
**88**	CML	Clinical Authenticity and Content Fidelity	2.4 Domain Specificity	GPT 4.o	5	5	5
**89**	CML	Clinical Authenticity and Content Fidelity	2.4 Domain Specificity	GPT 4.o	5	5	5
**90**	CML	Clinical Authenticity and Content Fidelity	2.4 Domain Specificity	GPT 4.o	5	5	5
**91**	CML	Clinical Authenticity and Content Fidelity	2.4 Domain Specificity	GPT 4.o	5	5	5
**92**	CML	Clinical Authenticity and Content Fidelity	2.4 Domain Specificity	GPT 4.o	5	5	5
**93**	CML	Clinical Authenticity and Content Fidelity	2.4 Domain Specificity	GPT 4.o	5	5	5
**94**	CML	Clinical Authenticity and Content Fidelity	2.4 Domain Specificity	GPT 4.o	5	5	5
**95**	CML	Clinical Authenticity and Content Fidelity	2.4 Domain Specificity	GPT 4.o	5	5	5
**96**	CML	Clinical Authenticity and Content Fidelity	2.4 Domain Specificity	GPT 4.o	5	5	5
**97**	CML	Clinical Authenticity and Content Fidelity	2.4 Domain Specificity	GPT 4.o	5	5	5
**98**	CML	Clinical Authenticity and Content Fidelity	2.4 Domain Specificity	GPT 4.o	5	4	5
**99**	CML	Clinical Authenticity and Content Fidelity	2.4 Domain Specificity	GPT 4.o	5	5	5
**100**	CML	Clinical Authenticity and Content Fidelity	2.4 Domain Specificity	GPT 4.o	5	5	5
**101**	CML	Clinical Authenticity and Content Fidelity	2.4 Domain Specificity	GPT 4.o	5	5	5

Supplementary D

**102**	CML	Clinical Authenticity and Content Fidelity	2.4 Domain Specificity	GPT 4.o	5	5	5
**103**	CML	Clinical Authenticity and Content Fidelity	2.4 Domain Specificity	GPT 4.o	5	5	5
**51**	CML	Modeled Clinical Reasoning	3.1 Problem Identification & Framing	Gemini 2.0 Pro	5	4	5
**52**	CML	Modeled Clinical Reasoning	3.1 Problem Identification & Framing	Gemini 2.0 Pro	5	5	5
**53**	CML	Modeled Clinical Reasoning	3.1 Problem Identification & Framing	Gemini 2.0 Pro	5	3	5
**54**	CML	Modeled Clinical Reasoning	3.1 Problem Identification & Framing	Gemini 2.0 Pro	5	4	5
**55**	CML	Modeled Clinical Reasoning	3.1 Problem Identification & Framing	Gemini 2.0 Pro	5	5	5
**56**	CML	Modeled Clinical Reasoning	3.1 Problem Identification & Framing	Gemini 2.0 Pro	5	5	5
**57**	CML	Modeled Clinical Reasoning	3.1 Problem Identification & Framing	Gemini 2.0 Pro	5	5	5
**58**	CML	Modeled Clinical Reasoning	3.1 Problem Identification & Framing	Gemini 2.0 Pro	5	5	5
**59**	CML	Modeled Clinical Reasoning	3.1 Problem Identification & Framing	Gemini 2.0 Pro	5	5	5
**60**	CML	Modeled Clinical Reasoning	3.1 Problem Identification & Framing	Gemini 2.0 Pro	5	5	5
**61**	CML	Modeled Clinical Reasoning	3.1 Problem Identification & Framing	Gemini 2.0 Pro	5	5	5
**62**	CML	Modeled Clinical Reasoning	3.1 Problem Identification & Framing	Gemini 2.0 Pro	5	5	5
**63**	CML	Modeled Clinical Reasoning	3.1 Problem Identification & Framing	DeepSee k V2	5	5	5
**64**	CML	Modeled Clinical Reasoning	3.1 Problem Identification & Framing	DeepSee k V2	5	5	5
**65**	CML	Modeled Clinical Reasoning	3.1 Problem Identification & Framing	DeepSee k V2	5	5	5
**66**	CML	Modeled Clinical Reasoning	3.1 Problem Identification & Framing	DeepSee k V2	5	5	5
**67**	CML	Modeled Clinical Reasoning	3.1 Problem Identification & Framing	DeepSee k V2	5	5	5

Supplementary D

**68**	CML	Modeled Clinical Reasoning	3.1 Problem Identification & Framing	DeepSeek V2	5	3	5
**69**	CML	Modeled Clinical Reasoning	3.1 Problem Identification & Framing	DeepSeek V2	5	5	5
**70**	CML	Modeled Clinical Reasoning	3.1 Problem Identification & Framing	DeepSeek V2	5	5	5
**71**	CML	Modeled Clinical Reasoning	3.1 Problem Identification & Framing	DeepSeek V2	5	5	5
**72**	CML	Modeled Clinical Reasoning	3.1 Problem Identification & Framing	DeepSeek V2	5	5	5
**73**	CML	Modeled Clinical Reasoning	3.1 Problem Identification & Framing	DeepSeek V2	5	5	5
**74**	CML	Modeled Clinical Reasoning	3.1 Problem Identification & Framing	DeepSeek V2	5	5	5
**75**	CML	Modeled Clinical Reasoning	3.1 Problem Identification & Framing	DeepSeek V2	5	3	5
**76**	CML	Modeled Clinical Reasoning	3.1 Problem Identification & Framing	Claude 3.7 Sonnet	5	5	5
**77**	CML	Modeled Clinical Reasoning	3.1 Problem Identification & Framing	Claude 3.7 Sonnet	5	5	5
**78**	CML	Modeled Clinical Reasoning	3.1 Problem Identification & Framing	Claude 3.7 Sonnet	5	5	5
**79**	CML	Modeled Clinical Reasoning	3.1 Problem Identification & Framing	Claude 3.7 Sonnet	5	5	5
**80**	CML	Modeled Clinical Reasoning	3.1 Problem Identification & Framing	Claude 3.7 Sonnet	5	5	5
**81**	CML	Modeled Clinical Reasoning	3.1 Problem Identification & Framing	Claude 3.7 Sonnet	5	5	5
**82**	CML	Modeled Clinical Reasoning	3.1 Problem Identification & Framing	Claude 3.7 Sonnet	5	5	5
**83**	CML	Modeled Clinical Reasoning	3.1 Problem Identification & Framing	Claude 3.7 Sonnet	5	5	5
**84**	CML	Modeled Clinical Reasoning	3.1 Problem Identification & Framing	Claude 3.7 Sonnet	5	5	5
**85**	CML	Modeled Clinical Reasoning	3.1 Problem Identification & Framing	Claude 3.7 Sonnet	4	4	4

Supplementary D

**86**	CML	Modeled Clinical Reasoning	3.1 Problem Identification & Framing	Claude 3.7 Sonnet	5	5	5
**87**	CML	Modeled Clinical Reasoning	3.1 Problem Identification & Framing	GPT 4.o	5	4	5
**88**	CML	Modeled Clinical Reasoning	3.1 Problem Identification & Framing	GPT 4.o	5	5	5
**89**	CML	Modeled Clinical Reasoning	3.1 Problem Identification & Framing	GPT 4.o	5	4	5
**90**	CML	Modeled Clinical Reasoning	3.1 Problem Identification & Framing	GPT 4.o	5	5	5
**91**	CML	Modeled Clinical Reasoning	3.1 Problem Identification & Framing	GPT 4.o	5	4	5
**92**	CML	Modeled Clinical Reasoning	3.1 Problem Identification & Framing	GPT 4.o	5	4	5
**93**	CML	Modeled Clinical Reasoning	3.1 Problem Identification & Framing	GPT 4.o	5	4	5
**94**	CML	Modeled Clinical Reasoning	3.1 Problem Identification & Framing	GPT 4.o	5	4	5
**95**	CML	Modeled Clinical Reasoning	3.1 Problem Identification & Framing	GPT 4.o	5	4	5
**96**	CML	Modeled Clinical Reasoning	3.1 Problem Identification & Framing	GPT 4.o	5	4	5
**97**	CML	Modeled Clinical Reasoning	3.1 Problem Identification & Framing	GPT 4.o	5	4	5
**98**	CML	Modeled Clinical Reasoning	3.1 Problem Identification & Framing	GPT 4.o	5	4	5
**99**	CML	Modeled Clinical Reasoning	3.1 Problem Identification & Framing	GPT 4.o	5	4	5
**100**	CML	Modeled Clinical Reasoning	3.1 Problem Identification & Framing	GPT 4.o	5	4	5
**101**	CML	Modeled Clinical Reasoning	3.1 Problem Identification & Framing	GPT 4.o	5	4	5
**102**	CML	Modeled Clinical Reasoning	3.1 Problem Identification & Framing	GPT 4.o	5	4	5
**103**	CML	Modeled Clinical Reasoning	3.1 Problem Identification & Framing	GPT 4.o	5	4	5

Supplementary D

**51**	CML	Modeled Clinical Reasoning	3.2 Diagnostic and Therapeutic Breadth	Gemini 2.0 Pro	5	3	5
**52**	CML	Modeled Clinical Reasoning	3.2 Diagnostic and Therapeutic Breadth	Gemini 2.0 Pro	5	5	5
**53**	CML	Modeled Clinical Reasoning	3.2 Diagnostic and Therapeutic Breadth	Gemini 2.0 Pro	5	4	5
**54**	CML	Modeled Clinical Reasoning	3.2 Diagnostic and Therapeutic Breadth	Gemini 2.0 Pro	5	5	5
**55**	CML	Modeled Clinical Reasoning	3.2 Diagnostic and Therapeutic Breadth	Gemini 2.0 Pro	5	5	5
**56**	CML	Modeled Clinical Reasoning	3.2 Diagnostic and Therapeutic Breadth	Gemini 2.0 Pro	5	5	5
**57**	CML	Modeled Clinical Reasoning	3.2 Diagnostic and Therapeutic Breadth	Gemini 2.0 Pro	5	5	5
**58**	CML	Modeled Clinical Reasoning	3.2 Diagnostic and Therapeutic Breadth	Gemini 2.0 Pro	5	5	5
**59**	CML	Modeled Clinical Reasoning	3.2 Diagnostic and Therapeutic Breadth	Gemini 2.0 Pro	5	5	5
**60**	CML	Modeled Clinical Reasoning	3.2 Diagnostic and Therapeutic Breadth	Gemini 2.0 Pro	5	5	5
**61**	CML	Modeled Clinical Reasoning	3.2 Diagnostic and Therapeutic Breadth	Gemini 2.0 Pro	5	5	5
**62**	CML	Modeled Clinical Reasoning	3.2 Diagnostic and Therapeutic Breadth	Gemini 2.0 Pro	5	5	5
**63**	CML	Modeled Clinical Reasoning	3.2 Diagnostic and Therapeutic Breadth	DeepSeek V2	5	5	5
**64**	CML	Modeled Clinical Reasoning	3.2 Diagnostic and Therapeutic Breadth	DeepSeek V2	5	5	5
**65**	CML	Modeled Clinical Reasoning	3.2 Diagnostic and Therapeutic Breadth	DeepSeek V2	5	5	5
**66**	CML	Modeled Clinical Reasoning	3.2 Diagnostic and Therapeutic Breadth	DeepSeek V2	5	5	5
**67**	CML	Modeled Clinical Reasoning	3.2 Diagnostic and Therapeutic Breadth	DeepSeek V2	5	5	5
**68**	CML	Modeled Clinical Reasoning	3.2 Diagnostic and Therapeutic Breadth	DeepSeek V2	5	4	5
**69**	CML	Modeled Clinical Reasoning	3.2 Diagnostic and Therapeutic Breadth	DeepSeek V2	5	5	5
**70**	CML	Modeled Clinical Reasoning	3.2 Diagnostic and Therapeutic Breadth	DeepSeek V2	5	5	5
**71**	CML	Modeled Clinical Reasoning	3.2 Diagnostic and Therapeutic Breadth	DeepSeek V2	5	5	5
**72**	CML	Modeled Clinical Reasoning	3.2 Diagnostic and Therapeutic Breadth	DeepSeek V2	5	5	5
**73**	CML	Modeled Clinical Reasoning	3.2 Diagnostic and Therapeutic Breadth	DeepSeek V2	3	2	3
**74**	CML	Modeled Clinical Reasoning	3.2 Diagnostic and Therapeutic Breadth	DeepSeek V2	5	5	5
**75**	CML	Modeled Clinical Reasoning	3.2 Diagnostic and Therapeutic Breadth	DeepSeek V2	5	5	5
**76**	CML	Modeled Clinical Reasoning	3.2 Diagnostic and Therapeutic Breadth	Claude 3.7 Sonnet	5	5	5

Supplementary D

**77**	CML	Modeled Clinical Reasoning	3.2 Diagnostic and Therapeutic Breadth	Claude 3.7 Sonnet	5	5	5
**78**	CML	Modeled Clinical Reasoning	3.2 Diagnostic and Therapeutic Breadth	Claude 3.7 Sonnet	5	5	5
**79**	CML	Modeled Clinical Reasoning	3.2 Diagnostic and Therapeutic Breadth	Claude 3.7 Sonnet	4	4	4
**80**	CML	Modeled Clinical Reasoning	3.2 Diagnostic and Therapeutic Breadth	Claude 3.7 Sonnet	5	5	5
**81**	CML	Modeled Clinical Reasoning	3.2 Diagnostic and Therapeutic Breadth	Claude 3.7 Sonnet	5	5	5
**82**	CML	Modeled Clinical Reasoning	3.2 Diagnostic and Therapeutic Breadth	Claude 3.7 Sonnet	5	5	5
**83**	CML	Modeled Clinical Reasoning	3.2 Diagnostic and Therapeutic Breadth	Claude 3.7 Sonnet	5	5	5
**84**	CML	Modeled Clinical Reasoning	3.2 Diagnostic and Therapeutic Breadth	Claude 3.7 Sonnet	5	5	5
**85**	CML	Modeled Clinical Reasoning	3.2 Diagnostic and Therapeutic Breadth	Claude 3.7 Sonnet	5	5	5
**86**	CML	Modeled Clinical Reasoning	3.2 Diagnostic and Therapeutic Breadth	Claude 3.7 Sonnet	5	5	5
**87**	CML	Modeled Clinical Reasoning	3.2 Diagnostic and Therapeutic Breadth	GPT 4.o	5	4	5
**88**	CML	Modeled Clinical Reasoning	3.2 Diagnostic and Therapeutic Breadth	GPT 4.o	5	5	5
**89**	CML	Modeled Clinical Reasoning	3.2 Diagnostic and Therapeutic Breadth	GPT 4.o	5	4	5
**90**	CML	Modeled Clinical Reasoning	3.2 Diagnostic and Therapeutic Breadth	GPT 4.o	5	5	5
**91**	CML	Modeled Clinical Reasoning	3.2 Diagnostic and Therapeutic Breadth	GPT 4.o	4	4	4
**92**	CML	Modeled Clinical Reasoning	3.2 Diagnostic and Therapeutic Breadth	GPT 4.o	5	4	5
**93**	CML	Modeled Clinical Reasoning	3.2 Diagnostic and Therapeutic Breadth	GPT 4.o	5	4	5
**94**	CML	Modeled Clinical Reasoning	3.2 Diagnostic and Therapeutic Breadth	GPT 4.o	5	4	5
**95**	CML	Modeled Clinical Reasoning	3.2 Diagnostic and Therapeutic Breadth	GPT 4.o	5	4	5
**96**	CML	Modeled Clinical Reasoning	3.2 Diagnostic and Therapeutic Breadth	GPT 4.o	5	4	5
**97**	CML	Modeled Clinical Reasoning	3.2 Diagnostic and Therapeutic Breadth	GPT 4.o	5	4	5
**98**	CML	Modeled Clinical Reasoning	3.2 Diagnostic and Therapeutic Breadth	GPT 4.o	5	4	5

Supplementary D

**99**	CML	Modeled Clinical Reasoning	3.2 Diagnostic and Therapeutic Breadth	GPT 4.o	5	4	5
**100**	CML	Modeled Clinical Reasoning	3.2 Diagnostic and Therapeutic Breadth	GPT 4.o	5	4	5
**101**	CML	Modeled Clinical Reasoning	3.2 Diagnostic and Therapeutic Breadth	GPT 4.o	5	4	5
**102**	CML	Modeled Clinical Reasoning	3.2 Diagnostic and Therapeutic Breadth	GPT 4.o	5	4	5
**103**	CML	Modeled Clinical Reasoning	3.2 Diagnostic and Therapeutic Breadth	GPT 4.o	5	4	5
**51**	CML	Modeled Clinical Reasoning	3.3 Prioritization and Decision-Making	Gemini 2.0 Pro	5	3	5
**52**	CML	Modeled Clinical Reasoning	3.3 Prioritization and Decision-Making	Gemini 2.0 Pro	5	5	5
**53**	CML	Modeled Clinical Reasoning	3.3 Prioritization and Decision-Making	Gemini 2.0 Pro	5	5	5
**54**	CML	Modeled Clinical Reasoning	3.3 Prioritization and Decision-Making	Gemini 2.0 Pro	5	5	5
**55**	CML	Modeled Clinical Reasoning	3.3 Prioritization and Decision-Making	Gemini 2.0 Pro	5	5	5
**56**	CML	Modeled Clinical Reasoning	3.3 Prioritization and Decision-Making	Gemini 2.0 Pro	1	1	1
**57**	CML	Modeled Clinical Reasoning	3.3 Prioritization and Decision-Making	Gemini 2.0 Pro	5	5	5
**58**	CML	Modeled Clinical Reasoning	3.3 Prioritization and Decision-Making	Gemini 2.0 Pro	5	5	5
**59**	CML	Modeled Clinical Reasoning	3.3 Prioritization and Decision-Making	Gemini 2.0 Pro	5	5	5
**60**	CML	Modeled Clinical Reasoning	3.3 Prioritization and Decision-Making	Gemini 2.0 Pro	5	5	5
**61**	CML	Modeled Clinical Reasoning	3.3 Prioritization and Decision-Making	Gemini 2.0 Pro	5	5	5
**62**	CML	Modeled Clinical Reasoning	3.3 Prioritization and Decision-Making	Gemini 2.0 Pro	5	5	5
**63**	CML	Modeled Clinical Reasoning	3.3 Prioritization and Decision-Making	DeepSee k V2	5	5	5
**64**	CML	Modeled Clinical Reasoning	3.3 Prioritization and Decision-Making	DeepSee k V2	5	5	5
**65**	CML	Modeled Clinical Reasoning	3.3 Prioritization and Decision-Making	DeepSee k V2	5	5	5
**66**	CML	Modeled Clinical Reasoning	3.3 Prioritization and Decision-Making	DeepSee k V2	5	5	5
**67**	CML	Modeled Clinical Reasoning	3.3 Prioritization and Decision-Making	DeepSee k V2	5	5	5
**68**	CML	Modeled Clinical Reasoning	3.3 Prioritization and Decision-Making	DeepSee k V2	5	3	5
**69**	CML	Modeled Clinical Reasoning	3.3 Prioritization and Decision-Making	DeepSee k V2	5	5	5
**70**	CML	Modeled Clinical Reasoning	3.3 Prioritization and Decision-Making	DeepSee k V2	5	5	5
**71**	CML	Modeled Clinical Reasoning	3.3 Prioritization and Decision-Making	DeepSee k V2	5	5	5
**72**	CML	Modeled Clinical Reasoning	3.3 Prioritization and Decision-Making	DeepSee k V2	5	5	5

Supplementary D

**73**	CML	Modeled Clinical Reasoning	3.3 Prioritization and Decision-Making	DeepSeek V2	3	3	3
**74**	CML	Modeled Clinical Reasoning	3.3 Prioritization and Decision-Making	DeepSeek V2	5	5	5
**75**	CML	Modeled Clinical Reasoning	3.3 Prioritization and Decision-Making	DeepSeek V2	2	2	2
**76**	CML	Modeled Clinical Reasoning	3.3 Prioritization and Decision-Making	Claude 3.7 Sonnet	5	5	5
**77**	CML	Modeled Clinical Reasoning	3.3 Prioritization and Decision-Making	Claude 3.7 Sonnet	5	5	5
**78**	CML	Modeled Clinical Reasoning	3.3 Prioritization and Decision-Making	Claude 3.7 Sonnet	4	4	4
**79**	CML	Modeled Clinical Reasoning	3.3 Prioritization and Decision-Making	Claude 3.7 Sonnet	4	4	4
**80**	CML	Modeled Clinical Reasoning	3.3 Prioritization and Decision-Making	Claude 3.7 Sonnet	5	5	5
**81**	CML	Modeled Clinical Reasoning	3.3 Prioritization and Decision-Making	Claude 3.7 Sonnet	5	5	5
**82**	CML	Modeled Clinical Reasoning	3.3 Prioritization and Decision-Making	Claude 3.7 Sonnet	5	5	5
**83**	CML	Modeled Clinical Reasoning	3.3 Prioritization and Decision-Making	Claude 3.7 Sonnet	5	5	5
**84**	CML	Modeled Clinical Reasoning	3.3 Prioritization and Decision-Making	Claude 3.7 Sonnet	5	5	5
**85**	CML	Modeled Clinical Reasoning	3.3 Prioritization and Decision-Making	Claude 3.7 Sonnet	5	5	5
**86**	CML	Modeled Clinical Reasoning	3.3 Prioritization and Decision-Making	Claude 3.7 Sonnet	5	5	5
**87**	CML	Modeled Clinical Reasoning	3.3 Prioritization and Decision-Making	GPT 4.o	5	4	5
**88**	CML	Modeled Clinical Reasoning	3.3 Prioritization and Decision-Making	GPT 4.o	5	5	5
**89**	CML	Modeled Clinical Reasoning	3.3 Prioritization and Decision-Making	GPT 4.o	4	4	4
**90**	CML	Modeled Clinical Reasoning	3.3 Prioritization and Decision-Making	GPT 4.o	5	3	5
**91**	CML	Modeled Clinical Reasoning	3.3 Prioritization and Decision-Making	GPT 4.o	2	2	2
**92**	CML	Modeled Clinical Reasoning	3.3 Prioritization and Decision-Making	GPT 4.o	5	4	5
**93**	CML	Modeled Clinical Reasoning	3.3 Prioritization and Decision-Making	GPT 4.o	5	4	5
**94**	CML	Modeled Clinical Reasoning	3.3 Prioritization and Decision-Making	GPT 4.o	5	4	5

Supplementary D

**95**	CML	Modeled Clinical Reasoning	3.3 Prioritization and Decision-Making	GPT 4.o	5	4	5
**96**	CML	Modeled Clinical Reasoning	3.3 Prioritization and Decision-Making	GPT 4.o	3	3	3
**97**	CML	Modeled Clinical Reasoning	3.3 Prioritization and Decision-Making	GPT 4.o	5	4	5
**98**	CML	Modeled Clinical Reasoning	3.3 Prioritization and Decision-Making	GPT 4.o	5	4	5
**99**	CML	Modeled Clinical Reasoning	3.3 Prioritization and Decision-Making	GPT 4.o	5	4	5
**100**	CML	Modeled Clinical Reasoning	3.3 Prioritization and Decision-Making	GPT 4.o	3	3	3
**101**	CML	Modeled Clinical Reasoning	3.3 Prioritization and Decision-Making	GPT 4.o	5	4	5
**102**	CML	Modeled Clinical Reasoning	3.3 Prioritization and Decision-Making	GPT 4.o	5	4	5
**103**	CML	Modeled Clinical Reasoning	3.3 Prioritization and Decision-Making	GPT 4.o	5	4	5
**51**	CML	Modeled Clinical Reasoning	3.4 Modeling of Clinical Outcomes	Gemini 2.0 Pro	5	5	5
**52**	CML	Modeled Clinical Reasoning	3.4 Modeling of Clinical Outcomes	Gemini 2.0 Pro	5	5	5
**53**	CML	Modeled Clinical Reasoning	3.4 Modeling of Clinical Outcomes	Gemini 2.0 Pro	5	3	5
**54**	CML	Modeled Clinical Reasoning	3.4 Modeling of Clinical Outcomes	Gemini 2.0 Pro	5	4	5
**55**	CML	Modeled Clinical Reasoning	3.4 Modeling of Clinical Outcomes	Gemini 2.0 Pro	5	5	5
**56**	CML	Modeled Clinical Reasoning	3.4 Modeling of Clinical Outcomes	Gemini 2.0 Pro	2	2	2
**57**	CML	Modeled Clinical Reasoning	3.4 Modeling of Clinical Outcomes	Gemini 2.0 Pro	5	5	5
**58**	CML	Modeled Clinical Reasoning	3.4 Modeling of Clinical Outcomes	Gemini 2.0 Pro	5	5	5
**59**	CML	Modeled Clinical Reasoning	3.4 Modeling of Clinical Outcomes	Gemini 2.0 Pro	5	5	5
**60**	CML	Modeled Clinical Reasoning	3.4 Modeling of Clinical Outcomes	Gemini 2.0 Pro	5	5	5
**61**	CML	Modeled Clinical Reasoning	3.4 Modeling of Clinical Outcomes	Gemini 2.0 Pro	5	5	5
**62**	CML	Modeled Clinical Reasoning	3.4 Modeling of Clinical Outcomes	Gemini 2.0 Pro	5	5	5
**63**	CML	Modeled Clinical Reasoning	3.4 Modeling of Clinical Outcomes	DeepSeek V2	5	5	5
**64**	CML	Modeled Clinical Reasoning	3.4 Modeling of Clinical Outcomes	DeepSeek V2	5	5	5
**65**	CML	Modeled Clinical Reasoning	3.4 Modeling of Clinical Outcomes	DeepSeek V2	5	5	5
**66**	CML	Modeled Clinical Reasoning	3.4 Modeling of Clinical Outcomes	DeepSeek V2	5	5	5
**67**	CML	Modeled Clinical Reasoning	3.4 Modeling of Clinical Outcomes	DeepSeek V2	5	5	5
**68**	CML	Modeled Clinical Reasoning	3.4 Modeling of Clinical Outcomes	DeepSeek V2	5	4	5

Supplementary D

**69**	CML	Modeled Clinical Reasoning	3.4 Modeling of Clinical Outcomes	DeepSeek V2	5	5	5
**70**	CML	Modeled Clinical Reasoning	3.4 Modeling of Clinical Outcomes	DeepSeek V2	5	5	5
**71**	CML	Modeled Clinical Reasoning	3.4 Modeling of Clinical Outcomes	DeepSeek V2	5	5	5
**72**	CML	Modeled Clinical Reasoning	3.4 Modeling of Clinical Outcomes	DeepSeek V2	5	5	5
**73**	CML	Modeled Clinical Reasoning	3.4 Modeling of Clinical Outcomes	DeepSeek V2	4	4	4
**74**	CML	Modeled Clinical Reasoning	3.4 Modeling of Clinical Outcomes	DeepSeek V2	5	5	5
**75**	CML	Modeled Clinical Reasoning	3.4 Modeling of Clinical Outcomes	DeepSeek V2	4	3	4
**76**	CML	Modeled Clinical Reasoning	3.4 Modeling of Clinical Outcomes	Claude 3.7 Sonnet	5	5	5
**77**	CML	Modeled Clinical Reasoning	3.4 Modeling of Clinical Outcomes	Claude 3.7 Sonnet	5	5	5
**78**	CML	Modeled Clinical Reasoning	3.4 Modeling of Clinical Outcomes	Claude 3.7 Sonnet	5	5	5
**79**	CML	Modeled Clinical Reasoning	3.4 Modeling of Clinical Outcomes	Claude 3.7 Sonnet	5	4	5
**80**	CML	Modeled Clinical Reasoning	3.4 Modeling of Clinical Outcomes	Claude 3.7 Sonnet	5	5	5
**81**	CML	Modeled Clinical Reasoning	3.4 Modeling of Clinical Outcomes	Claude 3.7 Sonnet	5	5	5
**82**	CML	Modeled Clinical Reasoning	3.4 Modeling of Clinical Outcomes	Claude 3.7 Sonnet	5	5	5
**83**	CML	Modeled Clinical Reasoning	3.4 Modeling of Clinical Outcomes	Claude 3.7 Sonnet	5	5	5
**84**	CML	Modeled Clinical Reasoning	3.4 Modeling of Clinical Outcomes	Claude 3.7 Sonnet	5	5	5
**85**	CML	Modeled Clinical Reasoning	3.4 Modeling of Clinical Outcomes	Claude 3.7 Sonnet	4	4	4
**86**	CML	Modeled Clinical Reasoning	3.4 Modeling of Clinical Outcomes	Claude 3.7 Sonnet	5	5	5
**87**	CML	Modeled Clinical Reasoning	3.4 Modeling of Clinical Outcomes	GPT 4.o	5	4	5
**88**	CML	Modeled Clinical Reasoning	3.4 Modeling of Clinical Outcomes	GPT 4.o	5	5	5
**89**	CML	Modeled Clinical Reasoning	3.4 Modeling of Clinical Outcomes	GPT 4.o	5	4	5
**90**	CML	Modeled Clinical Reasoning	3.4 Modeling of Clinical Outcomes	GPT 4.o	5	3	5

Supplementary D

**91**	CML	Modeled Clinical Reasoning	3.4 Modeling of Clinical Outcomes	GPT 4.o	4	4	4
**92**	CML	Modeled Clinical Reasoning	3.4 Modeling of Clinical Outcomes	GPT 4.o	5	4	5
**93**	CML	Modeled Clinical Reasoning	3.4 Modeling of Clinical Outcomes	GPT 4.o	5	4	5
**94**	CML	Modeled Clinical Reasoning	3.4 Modeling of Clinical Outcomes	GPT 4.o	5	4	5
**95**	CML	Modeled Clinical Reasoning	3.4 Modeling of Clinical Outcomes	GPT 4.o	5	4	5
**96**	CML	Modeled Clinical Reasoning	3.4 Modeling of Clinical Outcomes	GPT 4.o	5	4	5
**97**	CML	Modeled Clinical Reasoning	3.4 Modeling of Clinical Outcomes	GPT 4.o	5	4	5
**98**	CML	Modeled Clinical Reasoning	3.4 Modeling of Clinical Outcomes	GPT 4.o	5	4	5
**99**	CML	Modeled Clinical Reasoning	3.4 Modeling of Clinical Outcomes	GPT 4.o	5	4	5
**100**	CML	Modeled Clinical Reasoning	3.4 Modeling of Clinical Outcomes	GPT 4.o	5	4	5
**101**	CML	Modeled Clinical Reasoning	3.4 Modeling of Clinical Outcomes	GPT 4.o	5	4	5
**102**	CML	Modeled Clinical Reasoning	3.4 Modeling of Clinical Outcomes	GPT 4.o	5	4	5
**103**	CML	Modeled Clinical Reasoning	3.4 Modeling of Clinical Outcomes	GPT 4.o	5	4	5
**1**	AML	Instructional Design and Pedagogical Quality	1.1 Clarity of Learning Objectives	DeepSeek V2	4	4	4
**2**	AML	Instructional Design and Pedagogical Quality	1.1 Clarity of Learning Objectives	DeepSeek V2	4	4	4
**3**	AML	Instructional Design and Pedagogical Quality	1.1 Clarity of Learning Objectives	DeepSeek V2	4	4	4
**4**	AML	Instructional Design and Pedagogical Quality	1.1 Clarity of Learning Objectives	DeepSeek V2	4	4	4
**5**	AML	Instructional Design and Pedagogical Quality	1.1 Clarity of Learning Objectives	DeepSeek V2	4	4	4
**6**	AML	Instructional Design and Pedagogical Quality	1.1 Clarity of Learning Objectives	DeepSeek V2	4	4	4
**7**	AML	Instructional Design and Pedagogical Quality	1.1 Clarity of Learning Objectives	DeepSeek V2	4	4	4
**8**	AML	Instructional Design and Pedagogical Quality	1.1 Clarity of Learning Objectives	DeepSeek V2	4	4	4
**9**	AML	Instructional Design and Pedagogical Quality	1.1 Clarity of Learning Objectives	DeepSeek V2	4	4	4
**10**	AML	Instructional Design and Pedagogical Quality	1.1 Clarity of Learning Objectives	DeepSeek V2	4	4	4
**11**	AML	Instructional Design and Pedagogical Quality	1.1 Clarity of Learning Objectives	Claude 3.7 Sonnet	4	4	4
**12**	AML	Instructional Design and Pedagogical Quality	1.1 Clarity of Learning Objectives	Claude 3.7 Sonnet	4	4	4
**13**	AML	Instructional Design and Pedagogical Quality	1.1 Clarity of Learning Objectives	Claude 3.7 Sonnet	4	4	4

Supplementary D

**14**	AML	Instructional Design and Pedagogical Quality	1.1 Clarity of Learning Objectives	Claude 3.7 Sonnet	3	3	3
**15**	AML	Instructional Design and Pedagogical Quality	1.1 Clarity of Learning Objectives	Claude 3.7 Sonnet	5	5	5
**16**	AML	Instructional Design and Pedagogical Quality	1.1 Clarity of Learning Objectives	Claude 3.7 Sonnet	4	4	4
**17**	AML	Instructional Design and Pedagogical Quality	1.1 Clarity of Learning Objectives	Claude 3.7 Sonnet	4	4	4
**18**	AML	Instructional Design and Pedagogical Quality	1.1 Clarity of Learning Objectives	Claude 3.7 Sonnet	4	4	4
**19**	AML	Instructional Design and Pedagogical Quality	1.1 Clarity of Learning Objectives	Claude 3.7 Sonnet	4	4	4
**20**	AML	Instructional Design and Pedagogical Quality	1.1 Clarity of Learning Objectives	Claude 3.7 Sonnet	4	4	4
**21**	AML	Instructional Design and Pedagogical Quality	1.1 Clarity of Learning Objectives	Claude 3.7 Sonnet	3	3	3
**22**	AML	Instructional Design and Pedagogical Quality	1.1 Clarity of Learning Objectives	GPT 4.o	4	4	4
**23**	AML	Instructional Design and Pedagogical Quality	1.1 Clarity of Learning Objectives	GPT 4.o	4	4	4
**24**	AML	Instructional Design and Pedagogical Quality	1.1 Clarity of Learning Objectives	GPT 4.o	4	4	4
**25**	AML	Instructional Design and Pedagogical Quality	1.1 Clarity of Learning Objectives	GPT 4.o	4	4	4
**26**	AML	Instructional Design and Pedagogical Quality	1.1 Clarity of Learning Objectives	GPT 4.o	4	4	4
**27**	AML	Instructional Design and Pedagogical Quality	1.1 Clarity of Learning Objectives	GPT 4.o	4	4	4
**28**	AML	Instructional Design and Pedagogical Quality	1.1 Clarity of Learning Objectives	GPT 4.o	4	4	4
**29**	AML	Instructional Design and Pedagogical Quality	1.1 Clarity of Learning Objectives	GPT 4.o	4	4	4
**30**	AML	Instructional Design and Pedagogical Quality	1.1 Clarity of Learning Objectives	GPT 4.o	4	4	4
**31**	AML	Instructional Design and Pedagogical Quality	1.1 Clarity of Learning Objectives	GPT 4.o	4	4	4
**32**	AML	Instructional Design and Pedagogical Quality	1.1 Clarity of Learning Objectives	GPT 4.o	4	4	4
**33**	AML	Instructional Design and Pedagogical Quality	1.1 Clarity of Learning Objectives	GPT 4.o	4	4	4
**34**	AML	Instructional Design and Pedagogical Quality	1.1 Clarity of Learning Objectives	Gemini 2.0 Pro	4	4	4
**35**	AML	Instructional Design and Pedagogical Quality	1.1 Clarity of Learning Objectives	Gemini 2.0 Pro	4	4	4
**36**	AML	Instructional Design and Pedagogical Quality	1.1 Clarity of Learning Objectives	Gemini 2.0 Pro	4	4	4

Supplementary D

**37**	AML	Instructional Design and Pedagogical Quality	1.1 Clarity of Learning Objectives	Gemini 2.0 Pro	4	4	4
**38**	AML	Instructional Design and Pedagogical Quality	1.1 Clarity of Learning Objectives	Gemini 2.0 Pro	4	4	4
**39**	AML	Instructional Design and Pedagogical Quality	1.1 Clarity of Learning Objectives	Gemini 2.0 Pro	4	4	4
**40**	AML	Instructional Design and Pedagogical Quality	1.1 Clarity of Learning Objectives	Gemini 2.0 Pro	4	4	4
**41**	AML	Instructional Design and Pedagogical Quality	1.1 Clarity of Learning Objectives	Gemini 2.0 Pro	4	4	4
**42**	AML	Instructional Design and Pedagogical Quality	1.1 Clarity of Learning Objectives	Gemini 2.0 Pro	4	4	4
**43**	AML	Instructional Design and Pedagogical Quality	1.1 Clarity of Learning Objectives	Gemini 2.0 Pro	4	4	4
**44**	AML	Instructional Design and Pedagogical Quality	1.1 Clarity of Learning Objectives	Gemini 2.0 Pro	4	4	4
**45**	AML	Instructional Design and Pedagogical Quality	1.1 Clarity of Learning Objectives	Gemini 2.0 Pro	4	4	4
**46**	AML	Instructional Design and Pedagogical Quality	1.1 Clarity of Learning Objectives	Gemini 2.0 Pro	4	4	4
**47**	AML	Instructional Design and Pedagogical Quality	1.1 Clarity of Learning Objectives	Gemini 2.0 Pro	4	4	4
**48**	AML	Instructional Design and Pedagogical Quality	1.1 Clarity of Learning Objectives	Gemini 2.0 Pro	4	4	4
**49**	AML	Instructional Design and Pedagogical Quality	1.1 Clarity of Learning Objectives	Gemini 2.0 Pro	4	4	4
**50**	AML	Instructional Design and Pedagogical Quality	1.1 Clarity of Learning Objectives	Gemini 2.0 Pro	4	4	4
**1**	AML	Instructional Design and Pedagogical Quality	1.2 Scaffolding of Clinical Reasoning	DeepSeek V2	5	5	5
**2**	AML	Instructional Design and Pedagogical Quality	1.2 Scaffolding of Clinical Reasoning	DeepSeek V2	5	5	5
**3**	AML	Instructional Design and Pedagogical Quality	1.2 Scaffolding of Clinical Reasoning	DeepSeek V2	5	5	5
**4**	AML	Instructional Design and Pedagogical Quality	1.2 Scaffolding of Clinical Reasoning	DeepSeek V2	5	5	5
**5**	AML	Instructional Design and Pedagogical Quality	1.2 Scaffolding of Clinical Reasoning	DeepSeek V2	5	5	5
**6**	AML	Instructional Design and Pedagogical Quality	1.2 Scaffolding of Clinical Reasoning	DeepSeek V2	5	5	5
**7**	AML	Instructional Design and Pedagogical Quality	1.2 Scaffolding of Clinical Reasoning	DeepSeek V2	5	5	5
**8**	AML	Instructional Design and Pedagogical Quality	1.2 Scaffolding of Clinical Reasoning	DeepSeek V2	5	5	5
**9**	AML	Instructional Design and Pedagogical Quality	1.2 Scaffolding of Clinical Reasoning	DeepSeek V2	5	5	5
**10**	AML	Instructional Design and Pedagogical Quality	1.2 Scaffolding of Clinical Reasoning	DeepSeek V2	5	4	5
**11**	AML	Instructional Design and Pedagogical Quality	1.2 Scaffolding of Clinical Reasoning	Claude 3.7 Sonnet	5	5	5
**12**	AML	Instructional Design and Pedagogical Quality	1.2 Scaffolding of Clinical Reasoning	Claude 3.7 Sonnet	5	5	5

Supplementary D

**13**	AML	Instructional Design and Pedagogical Quality	1.2 Scaffolding of Clinical Reasoning	Claude 3.7 Sonnet	5	5	5
**14**	AML	Instructional Design and Pedagogical Quality	1.2 Scaffolding of Clinical Reasoning	Claude 3.7 Sonnet	5	5	5
**15**	AML	Instructional Design and Pedagogical Quality	1.2 Scaffolding of Clinical Reasoning	Claude 3.7 Sonnet	5	5	5
**16**	AML	Instructional Design and Pedagogical Quality	1.2 Scaffolding of Clinical Reasoning	Claude 3.7 Sonnet	5	5	5
**17**	AML	Instructional Design and Pedagogical Quality	1.2 Scaffolding of Clinical Reasoning	Claude 3.7 Sonnet	5	5	5
**18**	AML	Instructional Design and Pedagogical Quality	1.2 Scaffolding of Clinical Reasoning	Claude 3.7 Sonnet	5	5	5
**19**	AML	Instructional Design and Pedagogical Quality	1.2 Scaffolding of Clinical Reasoning	Claude 3.7 Sonnet	5	5	5
**20**	AML	Instructional Design and Pedagogical Quality	1.2 Scaffolding of Clinical Reasoning	Claude 3.7 Sonnet	5	5	5
**21**	AML	Instructional Design and Pedagogical Quality	1.2 Scaffolding of Clinical Reasoning	Claude 3.7 Sonnet	2	2	2
**22**	AML	Instructional Design and Pedagogical Quality	1.2 Scaffolding of Clinical Reasoning	GPT 4.o	5	5	5
**23**	AML	Instructional Design and Pedagogical Quality	1.2 Scaffolding of Clinical Reasoning	GPT 4.o	5	5	5
**24**	AML	Instructional Design and Pedagogical Quality	1.2 Scaffolding of Clinical Reasoning	GPT 4.o	5	5	5
**25**	AML	Instructional Design and Pedagogical Quality	1.2 Scaffolding of Clinical Reasoning	GPT 4.o	5	5	5
**26**	AML	Instructional Design and Pedagogical Quality	1.2 Scaffolding of Clinical Reasoning	GPT 4.o	5	5	5
**27**	AML	Instructional Design and Pedagogical Quality	1.2 Scaffolding of Clinical Reasoning	GPT 4.o	5	5	5
**28**	AML	Instructional Design and Pedagogical Quality	1.2 Scaffolding of Clinical Reasoning	GPT 4.o	5	5	5
**29**	AML	Instructional Design and Pedagogical Quality	1.2 Scaffolding of Clinical Reasoning	GPT 4.o	5	5	5
**30**	AML	Instructional Design and Pedagogical Quality	1.2 Scaffolding of Clinical Reasoning	GPT 4.o	5	5	5
**31**	AML	Instructional Design and Pedagogical Quality	1.2 Scaffolding of Clinical Reasoning	GPT 4.o	5	5	5
**32**	AML	Instructional Design and Pedagogical Quality	1.2 Scaffolding of Clinical Reasoning	GPT 4.o	5	5	5
**33**	AML	Instructional Design and Pedagogical Quality	1.2 Scaffolding of Clinical Reasoning	GPT 4.o	2	2	2
**34**	AML	Instructional Design and Pedagogical Quality	1.2 Scaffolding of Clinical Reasoning	Gemini 2.0 Pro	5	5	5
**35**	AML	Instructional Design and Pedagogical Quality	1.2 Scaffolding of Clinical Reasoning	Gemini 2.0 Pro	5	5	5

Supplementary D

**36**	AML	Instructional Design and Pedagogical Quality	1.2 Scaffolding of Clinical Reasoning	Gemini 2.0 Pro	5	5	5
**37**	AML	Instructional Design and Pedagogical Quality	1.2 Scaffolding of Clinical Reasoning	Gemini 2.0 Pro	5	5	5
**38**	AML	Instructional Design and Pedagogical Quality	1.2 Scaffolding of Clinical Reasoning	Gemini 2.0 Pro	5	5	5
**39**	AML	Instructional Design and Pedagogical Quality	1.2 Scaffolding of Clinical Reasoning	Gemini 2.0 Pro	5	5	5
**40**	AML	Instructional Design and Pedagogical Quality	1.2 Scaffolding of Clinical Reasoning	Gemini 2.0 Pro	5	5	5
**41**	AML	Instructional Design and Pedagogical Quality	1.2 Scaffolding of Clinical Reasoning	Gemini 2.0 Pro	5	5	5
**42**	AML	Instructional Design and Pedagogical Quality	1.2 Scaffolding of Clinical Reasoning	Gemini 2.0 Pro	5	5	5
**43**	AML	Instructional Design and Pedagogical Quality	1.2 Scaffolding of Clinical Reasoning	Gemini 2.0 Pro	5	5	5
**44**	AML	Instructional Design and Pedagogical Quality	1.2 Scaffolding of Clinical Reasoning	Gemini 2.0 Pro	5	5	5
**45**	AML	Instructional Design and Pedagogical Quality	1.2 Scaffolding of Clinical Reasoning	Gemini 2.0 Pro	5	5	5
**46**	AML	Instructional Design and Pedagogical Quality	1.2 Scaffolding of Clinical Reasoning	Gemini 2.0 Pro	5	5	5
**47**	AML	Instructional Design and Pedagogical Quality	1.2 Scaffolding of Clinical Reasoning	Gemini 2.0 Pro	5	5	5
**48**	AML	Instructional Design and Pedagogical Quality	1.2 Scaffolding of Clinical Reasoning	Gemini 2.0 Pro	4	4	4
**49**	AML	Instructional Design and Pedagogical Quality	1.2 Scaffolding of Clinical Reasoning	Gemini 2.0 Pro	5	5	5
**50**	AML	Instructional Design and Pedagogical Quality	1.2 Scaffolding of Clinical Reasoning	Gemini 2.0 Pro	5	5	5
**1**	AML	Instructional Design and Pedagogical Quality	1.3 Quality of Embedded Feedback	DeepSeek V2	5	5	5
**2**	AML	Instructional Design and Pedagogical Quality	1.3 Quality of Embedded Feedback	DeepSeek V2	5	5	5
**3**	AML	Instructional Design and Pedagogical Quality	1.3 Quality of Embedded Feedback	DeepSeek V2	5	5	5
**4**	AML	Instructional Design and Pedagogical Quality	1.3 Quality of Embedded Feedback	DeepSeek V2	5	5	5
**5**	AML	Instructional Design and Pedagogical Quality	1.3 Quality of Embedded Feedback	DeepSeek V2	5	5	5
**6**	AML	Instructional Design and Pedagogical Quality	1.3 Quality of Embedded Feedback	DeepSeek V2	5	5	5
**7**	AML	Instructional Design and Pedagogical Quality	1.3 Quality of Embedded Feedback	DeepSeek V2	5	5	5
**8**	AML	Instructional Design and Pedagogical Quality	1.3 Quality of Embedded Feedback	DeepSeek V2	5	5	5
**9**	AML	Instructional Design and Pedagogical Quality	1.3 Quality of Embedded Feedback	DeepSeek V2	5	5	5
**10**	AML	Instructional Design and Pedagogical Quality	1.3 Quality of Embedded Feedback	DeepSeek V2	5	5	5
**11**	AML	Instructional Design and Pedagogical Quality	1.3 Quality of Embedded Feedback	Claude 3.7 Sonnet	5	5	5

Supplementary D

**12**	AML	Instructional Design and Pedagogical Quality	1.3 Quality of Embedded Feedback	Claude 3.7 Sonnet	5	3	5
**13**	AML	Instructional Design and Pedagogical Quality	1.3 Quality of Embedded Feedback	Claude 3.7 Sonnet	5	5	5
**14**	AML	Instructional Design and Pedagogical Quality	1.3 Quality of Embedded Feedback	Claude 3.7 Sonnet	4	1	4
**15**	AML	Instructional Design and Pedagogical Quality	1.3 Quality of Embedded Feedback	Claude 3.7 Sonnet	5	4	5
**16**	AML	Instructional Design and Pedagogical Quality	1.3 Quality of Embedded Feedback	Claude 3.7 Sonnet	5	5	5
**17**	AML	Instructional Design and Pedagogical Quality	1.3 Quality of Embedded Feedback	Claude 3.7 Sonnet	5	5	5
**18**	AML	Instructional Design and Pedagogical Quality	1.3 Quality of Embedded Feedback	Claude 3.7 Sonnet	2	2	2
**19**	AML	Instructional Design and Pedagogical Quality	1.3 Quality of Embedded Feedback	Claude 3.7 Sonnet	2	1	2
**20**	AML	Instructional Design and Pedagogical Quality	1.3 Quality of Embedded Feedback	Claude 3.7 Sonnet	4	4	4
**21**	AML	Instructional Design and Pedagogical Quality	1.3 Quality of Embedded Feedback	Claude 3.7 Sonnet	4	2	4
**22**	AML	Instructional Design and Pedagogical Quality	1.3 Quality of Embedded Feedback	GPT 4.o	4	3	4
**23**	AML	Instructional Design and Pedagogical Quality	1.3 Quality of Embedded Feedback	GPT 4.o	5	5	5
**24**	AML	Instructional Design and Pedagogical Quality	1.3 Quality of Embedded Feedback	GPT 4.o	5	5	5
**25**	AML	Instructional Design and Pedagogical Quality	1.3 Quality of Embedded Feedback	GPT 4.o	5	5	5
**26**	AML	Instructional Design and Pedagogical Quality	1.3 Quality of Embedded Feedback	GPT 4.o	3	3	3
**27**	AML	Instructional Design and Pedagogical Quality	1.3 Quality of Embedded Feedback	GPT 4.o	5	5	5
**28**	AML	Instructional Design and Pedagogical Quality	1.3 Quality of Embedded Feedback	GPT 4.o	5	5	5
**29**	AML	Instructional Design and Pedagogical Quality	1.3 Quality of Embedded Feedback	GPT 4.o	5	4	5
**30**	AML	Instructional Design and Pedagogical Quality	1.3 Quality of Embedded Feedback	GPT 4.o	5	5	5
**31**	AML	Instructional Design and Pedagogical Quality	1.3 Quality of Embedded Feedback	GPT 4.o	5	5	5
**32**	AML	Instructional Design and Pedagogical Quality	1.3 Quality of Embedded Feedback	GPT 4.o	5	4	5
**33**	AML	Instructional Design and Pedagogical Quality	1.3 Quality of Embedded Feedback	GPT 4.o	1	1	1

Supplementary D

**34**	AML	Instructional Design and Pedagogical Quality	1.3 Quality of Embedded Feedback	Gemini 2.0 Pro	5	1	5
**35**	AML	Instructional Design and Pedagogical Quality	1.3 Quality of Embedded Feedback	Gemini 2.0 Pro	5	5	5
**36**	AML	Instructional Design and Pedagogical Quality	1.3 Quality of Embedded Feedback	Gemini 2.0 Pro	3	2	3
**37**	AML	Instructional Design and Pedagogical Quality	1.3 Quality of Embedded Feedback	Gemini 2.0 Pro	5	5	5
**38**	AML	Instructional Design and Pedagogical Quality	1.3 Quality of Embedded Feedback	Gemini 2.0 Pro	5	5	5
**39**	AML	Instructional Design and Pedagogical Quality	1.3 Quality of Embedded Feedback	Gemini 2.0 Pro	4	3	4
**40**	AML	Instructional Design and Pedagogical Quality	1.3 Quality of Embedded Feedback	Gemini 2.0 Pro	4	4	4
**41**	AML	Instructional Design and Pedagogical Quality	1.3 Quality of Embedded Feedback	Gemini 2.0 Pro	5	5	5
**42**	AML	Instructional Design and Pedagogical Quality	1.3 Quality of Embedded Feedback	Gemini 2.0 Pro	5	5	5
**43**	AML	Instructional Design and Pedagogical Quality	1.3 Quality of Embedded Feedback	Gemini 2.0 Pro	5	5	5
**44**	AML	Instructional Design and Pedagogical Quality	1.3 Quality of Embedded Feedback	Gemini 2.0 Pro	3	3	3
**45**	AML	Instructional Design and Pedagogical Quality	1.3 Quality of Embedded Feedback	Gemini 2.0 Pro	5	5	5
**46**	AML	Instructional Design and Pedagogical Quality	1.3 Quality of Embedded Feedback	Gemini 2.0 Pro	5	5	5
**47**	AML	Instructional Design and Pedagogical Quality	1.3 Quality of Embedded Feedback	Gemini 2.0 Pro	5	5	5
**48**	AML	Instructional Design and Pedagogical Quality	1.3 Quality of Embedded Feedback	Gemini 2.0 Pro	5	5	5
**49**	AML	Instructional Design and Pedagogical Quality	1.3 Quality of Embedded Feedback	Gemini 2.0 Pro	5	5	5
**50**	AML	Instructional Design and Pedagogical Quality	1.3 Quality of Embedded Feedback	Gemini 2.0 Pro	5	5	5
**1**	AML	Instructional Design and Pedagogical Quality	1.4 Instructional Clarity & Framing	DeepSeek V2	5	5	5
**2**	AML	Instructional Design and Pedagogical Quality	1.4 Instructional Clarity & Framing	DeepSeek V2	5	5	5
**3**	AML	Instructional Design and Pedagogical Quality	1.4 Instructional Clarity & Framing	DeepSeek V2	5	5	5
**4**	AML	Instructional Design and Pedagogical Quality	1.4 Instructional Clarity & Framing	DeepSeek V2	5	5	5
**5**	AML	Instructional Design and Pedagogical Quality	1.4 Instructional Clarity & Framing	DeepSeek V2	5	5	5
**6**	AML	Instructional Design and Pedagogical Quality	1.4 Instructional Clarity & Framing	DeepSeek V2	5	5	5
**7**	AML	Instructional Design and Pedagogical Quality	1.4 Instructional Clarity & Framing	DeepSeek V2	5	5	5
**8**	AML	Instructional Design and Pedagogical Quality	1.4 Instructional Clarity & Framing	DeepSeek V2	5	5	5
**9**	AML	Instructional Design and Pedagogical Quality	1.4 Instructional Clarity & Framing	DeepSeek V2	5	5	5
**10**	AML	Instructional Design and Pedagogical Quality	1.4 Instructional Clarity & Framing	DeepSeek V2	5	4	5

Supplementary D

**11**	AML	Instructional Design and Pedagogical Quality	1.4 Instructional Clarity & Framing	Claude 3.7 Sonnet	5	5	5
**12**	AML	Instructional Design and Pedagogical Quality	1.4 Instructional Clarity & Framing	Claude 3.7 Sonnet	5	3	5
**13**	AML	Instructional Design and Pedagogical Quality	1.4 Instructional Clarity & Framing	Claude 3.7 Sonnet	5	5	5
**14**	AML	Instructional Design and Pedagogical Quality	1.4 Instructional Clarity & Framing	Claude 3.7 Sonnet	4	4	4
**15**	AML	Instructional Design and Pedagogical Quality	1.4 Instructional Clarity & Framing	Claude 3.7 Sonnet	5	5	5
**16**	AML	Instructional Design and Pedagogical Quality	1.4 Instructional Clarity & Framing	Claude 3.7 Sonnet	5	5	5
**17**	AML	Instructional Design and Pedagogical Quality	1.4 Instructional Clarity & Framing	Claude 3.7 Sonnet	5	5	5
**18**	AML	Instructional Design and Pedagogical Quality	1.4 Instructional Clarity & Framing	Claude 3.7 Sonnet	4	4	4
**19**	AML	Instructional Design and Pedagogical Quality	1.4 Instructional Clarity & Framing	Claude 3.7 Sonnet	4	4	4
**20**	AML	Instructional Design and Pedagogical Quality	1.4 Instructional Clarity & Framing	Claude 3.7 Sonnet	4	4	4
**21**	AML	Instructional Design and Pedagogical Quality	1.4 Instructional Clarity & Framing	Claude 3.7 Sonnet	5	5	5
**22**	AML	Instructional Design and Pedagogical Quality	1.4 Instructional Clarity & Framing	GPT 4.o	5	4	5
**23**	AML	Instructional Design and Pedagogical Quality	1.4 Instructional Clarity & Framing	GPT 4.o	5	5	5
**24**	AML	Instructional Design and Pedagogical Quality	1.4 Instructional Clarity & Framing	GPT 4.o	5	5	5
**25**	AML	Instructional Design and Pedagogical Quality	1.4 Instructional Clarity & Framing	GPT 4.o	5	5	5
**26**	AML	Instructional Design and Pedagogical Quality	1.4 Instructional Clarity & Framing	GPT 4.o	5	5	5
**27**	AML	Instructional Design and Pedagogical Quality	1.4 Instructional Clarity & Framing	GPT 4.o	5	5	5
**28**	AML	Instructional Design and Pedagogical Quality	1.4 Instructional Clarity & Framing	GPT 4.o	5	5	5
**29**	AML	Instructional Design and Pedagogical Quality	1.4 Instructional Clarity & Framing	GPT 4.o	5	5	5
**30**	AML	Instructional Design and Pedagogical Quality	1.4 Instructional Clarity & Framing	GPT 4.o	5	5	5
**31**	AML	Instructional Design and Pedagogical Quality	1.4 Instructional Clarity & Framing	GPT 4.o	5	5	5
**32**	AML	Instructional Design and Pedagogical Quality	1.4 Instructional Clarity & Framing	GPT 4.o	5	5	5

Supplementary D

**33**	AML	Instructional Design and Pedagogical Quality	1.4 Instructional Clarity & Framing	GPT 4.o	2	2	2
**34**	AML	Instructional Design and Pedagogical Quality	1.4 Instructional Clarity & Framing	Gemini 2.0 Pro	5	3	5
**35**	AML	Instructional Design and Pedagogical Quality	1.4 Instructional Clarity & Framing	Gemini 2.0 Pro	5	5	5
**36**	AML	Instructional Design and Pedagogical Quality	1.4 Instructional Clarity & Framing	Gemini 2.0 Pro	5	5	5
**37**	AML	Instructional Design and Pedagogical Quality	1.4 Instructional Clarity & Framing	Gemini 2.0 Pro	5	5	5
**38**	AML	Instructional Design and Pedagogical Quality	1.4 Instructional Clarity & Framing	Gemini 2.0 Pro	5	5	5
**39**	AML	Instructional Design and Pedagogical Quality	1.4 Instructional Clarity & Framing	Gemini 2.0 Pro	5	5	5
**40**	AML	Instructional Design and Pedagogical Quality	1.4 Instructional Clarity & Framing	Gemini 2.0 Pro	5	5	5
**41**	AML	Instructional Design and Pedagogical Quality	1.4 Instructional Clarity & Framing	Gemini 2.0 Pro	5	5	5
**42**	AML	Instructional Design and Pedagogical Quality	1.4 Instructional Clarity & Framing	Gemini 2.0 Pro	5	5	5
**43**	AML	Instructional Design and Pedagogical Quality	1.4 Instructional Clarity & Framing	Gemini 2.0 Pro	5	5	5
**44**	AML	Instructional Design and Pedagogical Quality	1.4 Instructional Clarity & Framing	Gemini 2.0 Pro	5	5	5
**45**	AML	Instructional Design and Pedagogical Quality	1.4 Instructional Clarity & Framing	Gemini 2.0 Pro	5	5	5
**46**	AML	Instructional Design and Pedagogical Quality	1.4 Instructional Clarity & Framing	Gemini 2.0 Pro	5	5	5
**47**	AML	Instructional Design and Pedagogical Quality	1.4 Instructional Clarity & Framing	Gemini 2.0 Pro	5	5	5
**48**	AML	Instructional Design and Pedagogical Quality	1.4 Instructional Clarity & Framing	Gemini 2.0 Pro	5	5	5
**49**	AML	Instructional Design and Pedagogical Quality	1.4 Instructional Clarity & Framing	Gemini 2.0 Pro	5	5	5
**50**	AML	Instructional Design and Pedagogical Quality	1.4 Instructional Clarity & Framing	Gemini 2.0 Pro	5	5	5
**1**	AML	Clinical Authenticity and Content Fidelity	2.1 Clinical Narrative Plausibility	DeepSeek V2	5	3	3
**2**	AML	Clinical Authenticity and Content Fidelity	2.1 Clinical Narrative Plausibility	DeepSeek V2	5	5	5
**3**	AML	Clinical Authenticity and Content Fidelity	2.1 Clinical Narrative Plausibility	DeepSeek V2	5	5	5
**4**	AML	Clinical Authenticity and Content Fidelity	2.1 Clinical Narrative Plausibility	DeepSeek V2	4	4	4
**5**	AML	Clinical Authenticity and Content Fidelity	2.1 Clinical Narrative Plausibility	DeepSeek V2	5	5	4
**6**	AML	Clinical Authenticity and Content Fidelity	2.1 Clinical Narrative Plausibility	DeepSeek V2	5	5	5
**7**	AML	Clinical Authenticity and Content Fidelity	2.1 Clinical Narrative Plausibility	DeepSeek V2	5	5	5
**8**	AML	Clinical Authenticity and Content Fidelity	2.1 Clinical Narrative Plausibility	DeepSeek V2	5	5	5
**9**	AML	Clinical Authenticity and Content Fidelity	2.1 Clinical Narrative Plausibility	DeepSeek V2	5	5	5

Supplementary D

**10**	AML	Clinical Authenticity and Content Fidelity	2.1 Clinical Narrative Plausibility	DeepSeek V2	5	5	5
**11**	AML	Clinical Authenticity and Content Fidelity	2.1 Clinical Narrative Plausibility	Claude 3.7 Sonnet	5	5	5
**12**	AML	Clinical Authenticity and Content Fidelity	2.1 Clinical Narrative Plausibility	Claude 3.7 Sonnet	5	5	5
**13**	AML	Clinical Authenticity and Content Fidelity	2.1 Clinical Narrative Plausibility	Claude 3.7 Sonnet	5	5	5
**14**	AML	Clinical Authenticity and Content Fidelity	2.1 Clinical Narrative Plausibility	Claude 3.7 Sonnet	5	5	5
**15**	AML	Clinical Authenticity and Content Fidelity	2.1 Clinical Narrative Plausibility	Claude 3.7 Sonnet	5	5	5
**16**	AML	Clinical Authenticity and Content Fidelity	2.1 Clinical Narrative Plausibility	Claude 3.7 Sonnet	5	5	5
**17**	AML	Clinical Authenticity and Content Fidelity	2.1 Clinical Narrative Plausibility	Claude 3.7 Sonnet	5	5	5
**18**	AML	Clinical Authenticity and Content Fidelity	2.1 Clinical Narrative Plausibility	Claude 3.7 Sonnet	5	5	5
**19**	AML	Clinical Authenticity and Content Fidelity	2.1 Clinical Narrative Plausibility	Claude 3.7 Sonnet	5	5	5
**20**	AML	Clinical Authenticity and Content Fidelity	2.1 Clinical Narrative Plausibility	Claude 3.7 Sonnet	5	5	5
**21**	AML	Clinical Authenticity and Content Fidelity	2.1 Clinical Narrative Plausibility	Claude 3.7 Sonnet	5	3	5
**22**	AML	Clinical Authenticity and Content Fidelity	2.1 Clinical Narrative Plausibility	GPT 4.o	5	3	5
**23**	AML	Clinical Authenticity and Content Fidelity	2.1 Clinical Narrative Plausibility	GPT 4.o	5	5	5
**24**	AML	Clinical Authenticity and Content Fidelity	2.1 Clinical Narrative Plausibility	GPT 4.o	5	5	5
**25**	AML	Clinical Authenticity and Content Fidelity	2.1 Clinical Narrative Plausibility	GPT 4.o	5	5	5
**26**	AML	Clinical Authenticity and Content Fidelity	2.1 Clinical Narrative Plausibility	GPT 4.o	5	5	5
**27**	AML	Clinical Authenticity and Content Fidelity	2.1 Clinical Narrative Plausibility	GPT 4.o	5	5	5
**28**	AML	Clinical Authenticity and Content Fidelity	2.1 Clinical Narrative Plausibility	GPT 4.o	5	5	5
**29**	AML	Clinical Authenticity and Content Fidelity	2.1 Clinical Narrative Plausibility	GPT 4.o	5	5	5
**30**	AML	Clinical Authenticity and Content Fidelity	2.1 Clinical Narrative Plausibility	GPT 4.o	5	5	5
**31**	AML	Clinical Authenticity and Content Fidelity	2.1 Clinical Narrative Plausibility	GPT 4.o	5	5	5

Supplementary D

**32**	AML	Clinical Authenticity and Content Fidelity	2.1 Clinical Narrative Plausibility	GPT 4.o	2	2	2
**33**	AML	Clinical Authenticity and Content Fidelity	2.1 Clinical Narrative Plausibility	GPT 4.o	5	5	5
**34**	AML	Clinical Authenticity and Content Fidelity	2.1 Clinical Narrative Plausibility	Gemini 2.0 Pro	5	5	5
**35**	AML	Clinical Authenticity and Content Fidelity	2.1 Clinical Narrative Plausibility	Gemini 2.0 Pro	5	5	5
**36**	AML	Clinical Authenticity and Content Fidelity	2.1 Clinical Narrative Plausibility	Gemini 2.0 Pro	5	5	5
**37**	AML	Clinical Authenticity and Content Fidelity	2.1 Clinical Narrative Plausibility	Gemini 2.0 Pro	5	5	5
**38**	AML	Clinical Authenticity and Content Fidelity	2.1 Clinical Narrative Plausibility	Gemini 2.0 Pro	5	5	5
**39**	AML	Clinical Authenticity and Content Fidelity	2.1 Clinical Narrative Plausibility	Gemini 2.0 Pro	5	5	5
**40**	AML	Clinical Authenticity and Content Fidelity	2.1 Clinical Narrative Plausibility	Gemini 2.0 Pro	5	5	5
**41**	AML	Clinical Authenticity and Content Fidelity	2.1 Clinical Narrative Plausibility	Gemini 2.0 Pro	5	5	5
**42**	AML	Clinical Authenticity and Content Fidelity	2.1 Clinical Narrative Plausibility	Gemini 2.0 Pro	5	5	5
**43**	AML	Clinical Authenticity and Content Fidelity	2.1 Clinical Narrative Plausibility	Gemini 2.0 Pro	5	5	5
**44**	AML	Clinical Authenticity and Content Fidelity	2.1 Clinical Narrative Plausibility	Gemini 2.0 Pro	5	5	5
**45**	AML	Clinical Authenticity and Content Fidelity	2.1 Clinical Narrative Plausibility	Gemini 2.0 Pro	5	5	5
**46**	AML	Clinical Authenticity and Content Fidelity	2.1 Clinical Narrative Plausibility	Gemini 2.0 Pro	5	5	5
**47**	AML	Clinical Authenticity and Content Fidelity	2.1 Clinical Narrative Plausibility	Gemini 2.0 Pro	5	5	5
**48**	AML	Clinical Authenticity and Content Fidelity	2.1 Clinical Narrative Plausibility	Gemini 2.0 Pro	5	5	5
**49**	AML	Clinical Authenticity and Content Fidelity	2.1 Clinical Narrative Plausibility	Gemini 2.0 Pro	5	5	5
**50**	AML	Clinical Authenticity and Content Fidelity	2.1 Clinical Narrative Plausibility	Gemini 2.0 Pro	5	5	5
**1**	AML	Clinical Authenticity and Content Fidelity	2.2 Alignment with Clinical Guidelines	DeepSeek V2	2	2	2
**2**	AML	Clinical Authenticity and Content Fidelity	2.2 Alignment with Clinical Guidelines	DeepSeek V2	4	2	2
**3**	AML	Clinical Authenticity and Content Fidelity	2.2 Alignment with Clinical Guidelines	DeepSeek V2	2	2	2
**4**	AML	Clinical Authenticity and Content Fidelity	2.2 Alignment with Clinical Guidelines	DeepSeek V2	4	2	3
**5**	AML	Clinical Authenticity and Content Fidelity	2.2 Alignment with Clinical Guidelines	DeepSeek V2	3	3	3
**6**	AML	Clinical Authenticity and Content Fidelity	2.2 Alignment with Clinical Guidelines	DeepSeek V2	3	3	2
**7**	AML	Clinical Authenticity and Content Fidelity	2.2 Alignment with Clinical Guidelines	DeepSeek V2	3	3	3
**8**	AML	Clinical Authenticity and Content Fidelity	2.2 Alignment with Clinical Guidelines	DeepSeek V2	3	3	3

Supplementary D

**9**	AML	Clinical Authenticity and Content Fidelity	2.2 Alignment with Clinical Guidelines	DeepSeek V2	4	4	3
**10**	AML	Clinical Authenticity and Content Fidelity	2.2 Alignment with Clinical Guidelines	DeepSeek V2	5	5	5
**11**	AML	Clinical Authenticity and Content Fidelity	2.2 Alignment with Clinical Guidelines	Claude 3.7 Sonnet	5	5	5
**12**	AML	Clinical Authenticity and Content Fidelity	2.2 Alignment with Clinical Guidelines	Claude 3.7 Sonnet	5	5	3
**13**	AML	Clinical Authenticity and Content Fidelity	2.2 Alignment with Clinical Guidelines	Claude 3.7 Sonnet	5	5	5
**14**	AML	Clinical Authenticity and Content Fidelity	2.2 Alignment with Clinical Guidelines	Claude 3.7 Sonnet	5	5	5
**15**	AML	Clinical Authenticity and Content Fidelity	2.2 Alignment with Clinical Guidelines	Claude 3.7 Sonnet	5	5	5
**16**	AML	Clinical Authenticity and Content Fidelity	2.2 Alignment with Clinical Guidelines	Claude 3.7 Sonnet	3	3	2
**17**	AML	Clinical Authenticity and Content Fidelity	2.2 Alignment with Clinical Guidelines	Claude 3.7 Sonnet	5	5	3
**18**	AML	Clinical Authenticity and Content Fidelity	2.2 Alignment with Clinical Guidelines	Claude 3.7 Sonnet	4	4	4
**19**	AML	Clinical Authenticity and Content Fidelity	2.2 Alignment with Clinical Guidelines	Claude 3.7 Sonnet	4	4	4
**20**	AML	Clinical Authenticity and Content Fidelity	2.2 Alignment with Clinical Guidelines	Claude 3.7 Sonnet	4	4	4
**21**	AML	Clinical Authenticity and Content Fidelity	2.2 Alignment with Clinical Guidelines	Claude 3.7 Sonnet	3	3	3
**22**	AML	Clinical Authenticity and Content Fidelity	2.2 Alignment with Clinical Guidelines	GPT 4.o	2	3	4
**23**	AML	Clinical Authenticity and Content Fidelity	2.2 Alignment with Clinical Guidelines	GPT 4.o	2	2	4
**24**	AML	Clinical Authenticity and Content Fidelity	2.2 Alignment with Clinical Guidelines	GPT 4.o	5	5	3
**25**	AML	Clinical Authenticity and Content Fidelity	2.2 Alignment with Clinical Guidelines	GPT 4.o	5	5	5
**26**	AML	Clinical Authenticity and Content Fidelity	2.2 Alignment with Clinical Guidelines	GPT 4.o	3	3	3
**27**	AML	Clinical Authenticity and Content Fidelity	2.2 Alignment with Clinical Guidelines	GPT 4.o	5	5	5
**28**	AML	Clinical Authenticity and Content Fidelity	2.2 Alignment with Clinical Guidelines	GPT 4.o	2	2	2
**29**	AML	Clinical Authenticity and Content Fidelity	2.2 Alignment with Clinical Guidelines	GPT 4.o	4	4	4
**30**	AML	Clinical Authenticity and Content Fidelity	2.2 Alignment with Clinical Guidelines	GPT 4.o	5	5	3

Supplementary D

**31**	AML	Clinical Authenticity and Content Fidelity	2.2 Alignment with Clinical Guidelines	GPT 4.o	5	5	5
**32**	AML	Clinical Authenticity and Content Fidelity	2.2 Alignment with Clinical Guidelines	GPT 4.o	2	2	1
**33**	AML	Clinical Authenticity and Content Fidelity	2.2 Alignment with Clinical Guidelines	GPT 4.o	2	2	1
**34**	AML	Clinical Authenticity and Content Fidelity	2.2 Alignment with Clinical Guidelines	Gemini 2.0 Pro	4	4	4
**35**	AML	Clinical Authenticity and Content Fidelity	2.2 Alignment with Clinical Guidelines	Gemini 2.0 Pro	5	5	5
**36**	AML	Clinical Authenticity and Content Fidelity	2.2 Alignment with Clinical Guidelines	Gemini 2.0 Pro	2	2	2
**37**	AML	Clinical Authenticity and Content Fidelity	2.2 Alignment with Clinical Guidelines	Gemini 2.0 Pro	5	5	3
**38**	AML	Clinical Authenticity and Content Fidelity	2.2 Alignment with Clinical Guidelines	Gemini 2.0 Pro	5	5	5
**39**	AML	Clinical Authenticity and Content Fidelity	2.2 Alignment with Clinical Guidelines	Gemini 2.0 Pro	4	4	4
**40**	AML	Clinical Authenticity and Content Fidelity	2.2 Alignment with Clinical Guidelines	Gemini 2.0 Pro	3	3	2
**41**	AML	Clinical Authenticity and Content Fidelity	2.2 Alignment with Clinical Guidelines	Gemini 2.0 Pro	3	3	2
**42**	AML	Clinical Authenticity and Content Fidelity	2.2 Alignment with Clinical Guidelines	Gemini 2.0 Pro	5	5	5
**43**	AML	Clinical Authenticity and Content Fidelity	2.2 Alignment with Clinical Guidelines	Gemini 2.0 Pro	5	5	5
**44**	AML	Clinical Authenticity and Content Fidelity	2.2 Alignment with Clinical Guidelines	Gemini 2.0 Pro	2	2	2
**45**	AML	Clinical Authenticity and Content Fidelity	2.2 Alignment with Clinical Guidelines	Gemini 2.0 Pro	4	4	3
**46**	AML	Clinical Authenticity and Content Fidelity	2.2 Alignment with Clinical Guidelines	Gemini 2.0 Pro	5	5	5
**47**	AML	Clinical Authenticity and Content Fidelity	2.2 Alignment with Clinical Guidelines	Gemini 2.0 Pro	5	5	5
**48**	AML	Clinical Authenticity and Content Fidelity	2.2 Alignment with Clinical Guidelines	Gemini 2.0 Pro	4	4	4
**49**	AML	Clinical Authenticity and Content Fidelity	2.2 Alignment with Clinical Guidelines	Gemini 2.0 Pro	5	5	5
**50**	AML	Clinical Authenticity and Content Fidelity	2.2 Alignment with Clinical Guidelines	Gemini 2.0 Pro	5	5	5
**1**	AML	Clinical Authenticity and Content Fidelity	2.3 Pharmacotherapeutic Accuracy	DeepSeek V2	1	4	1
**2**	AML	Clinical Authenticity and Content Fidelity	2.3 Pharmacotherapeutic Accuracy	DeepSeek V2	2	2	2
**3**	AML	Clinical Authenticity and Content Fidelity	2.3 Pharmacotherapeutic Accuracy	DeepSeek V2	1	1	1
**4**	AML	Clinical Authenticity and Content Fidelity	2.3 Pharmacotherapeutic Accuracy	DeepSeek V2	2	2	2
**5**	AML	Clinical Authenticity and Content Fidelity	2.3 Pharmacotherapeutic Accuracy	DeepSeek V2	3	3	3

Supplementary D

**6**	AML	Clinical Authenticity and Content Fidelity	2.3 Pharmacotherapeutic Accuracy	DeepSeek V2	3	3	3
**7**	AML	Clinical Authenticity and Content Fidelity	2.3 Pharmacotherapeutic Accuracy	DeepSeek V2	2	2	2
**8**	AML	Clinical Authenticity and Content Fidelity	2.3 Pharmacotherapeutic Accuracy	DeepSeek V2	2	2	2
**9**	AML	Clinical Authenticity and Content Fidelity	2.3 Pharmacotherapeutic Accuracy	DeepSeek V2	3	3	3
**10**	AML	Clinical Authenticity and Content Fidelity	2.3 Pharmacotherapeutic Accuracy	DeepSeek V2	4	4	4
**11**	AML	Clinical Authenticity and Content Fidelity	2.3 Pharmacotherapeutic Accuracy	Claude 3.7 Sonnet	5	5	5
**12**	AML	Clinical Authenticity and Content Fidelity	2.3 Pharmacotherapeutic Accuracy	Claude 3.7 Sonnet	5	5	5
**13**	AML	Clinical Authenticity and Content Fidelity	2.3 Pharmacotherapeutic Accuracy	Claude 3.7 Sonnet	5	5	5
**14**	AML	Clinical Authenticity and Content Fidelity	2.3 Pharmacotherapeutic Accuracy	Claude 3.7 Sonnet	5	5	5
**15**	AML	Clinical Authenticity and Content Fidelity	2.3 Pharmacotherapeutic Accuracy	Claude 3.7 Sonnet	5	5	5
**16**	AML	Clinical Authenticity and Content Fidelity	2.3 Pharmacotherapeutic Accuracy	Claude 3.7 Sonnet	2	2	2
**17**	AML	Clinical Authenticity and Content Fidelity	2.3 Pharmacotherapeutic Accuracy	Claude 3.7 Sonnet	5	5	4
**18**	AML	Clinical Authenticity and Content Fidelity	2.3 Pharmacotherapeutic Accuracy	Claude 3.7 Sonnet	4	4	4
**19**	AML	Clinical Authenticity and Content Fidelity	2.3 Pharmacotherapeutic Accuracy	Claude 3.7 Sonnet	4	4	4
**20**	AML	Clinical Authenticity and Content Fidelity	2.3 Pharmacotherapeutic Accuracy	Claude 3.7 Sonnet	4	4	4
**21**	AML	Clinical Authenticity and Content Fidelity	2.3 Pharmacotherapeutic Accuracy	Claude 3.7 Sonnet	2	2	2
**22**	AML	Clinical Authenticity and Content Fidelity	2.3 Pharmacotherapeutic Accuracy	GPT 4.o	2	3	2
**23**	AML	Clinical Authenticity and Content Fidelity	2.3 Pharmacotherapeutic Accuracy	GPT 4.o	2	2	2

Supplementary D

**24**	AML	Clinical Authenticity and Content Fidelity	2.3 Pharmacotherapeutic Accuracy	GPT 4.o	5	5	2
**25**	AML	Clinical Authenticity and Content Fidelity	2.3 Pharmacotherapeutic Accuracy	GPT 4.o	5	5	5
**26**	AML	Clinical Authenticity and Content Fidelity	2.3 Pharmacotherapeutic Accuracy	GPT 4.o	5	5	5
**27**	AML	Clinical Authenticity and Content Fidelity	2.3 Pharmacotherapeutic Accuracy	GPT 4.o	5	5	5
**28**	AML	Clinical Authenticity and Content Fidelity	2.3 Pharmacotherapeutic Accuracy	GPT 4.o	3	3	3
**29**	AML	Clinical Authenticity and Content Fidelity	2.3 Pharmacotherapeutic Accuracy	GPT 4.o	3	3	3
**30**	AML	Clinical Authenticity and Content Fidelity	2.3 Pharmacotherapeutic Accuracy	GPT 4.o	5	5	5
**31**	AML	Clinical Authenticity and Content Fidelity	2.3 Pharmacotherapeutic Accuracy	GPT 4.o	5	5	5
**32**	AML	Clinical Authenticity and Content Fidelity	2.3 Pharmacotherapeutic Accuracy	GPT 4.o	2	2	2
**33**	AML	Clinical Authenticity and Content Fidelity	2.3 Pharmacotherapeutic Accuracy	GPT 4.o	1	1	1
**34**	AML	Clinical Authenticity and Content Fidelity	2.3 Pharmacotherapeutic Accuracy	Gemini 2.0 Pro	4	4	4
**35**	AML	Clinical Authenticity and Content Fidelity	2.3 Pharmacotherapeutic Accuracy	Gemini 2.0 Pro	5	5	5
**36**	AML	Clinical Authenticity and Content Fidelity	2.3 Pharmacotherapeutic Accuracy	Gemini 2.0 Pro	2	2	2
**37**	AML	Clinical Authenticity and Content Fidelity	2.3 Pharmacotherapeutic Accuracy	Gemini 2.0 Pro	5	5	5
**38**	AML	Clinical Authenticity and Content Fidelity	2.3 Pharmacotherapeutic Accuracy	Gemini 2.0 Pro	5	5	5
**39**	AML	Clinical Authenticity and Content Fidelity	2.3 Pharmacotherapeutic Accuracy	Gemini 2.0 Pro	2	2	2
**40**	AML	Clinical Authenticity and Content Fidelity	2.3 Pharmacotherapeutic Accuracy	Gemini 2.0 Pro	2	2	2
**41**	AML	Clinical Authenticity and Content Fidelity	2.3 Pharmacotherapeutic Accuracy	Gemini 2.0 Pro	2	2	2

Supplementary D

**42**	AML	Clinical Authenticity and Content Fidelity	2.3 Pharmacotherapeutic Accuracy	Gemini 2.0 Pro	5	5	5
**43**	AML	Clinical Authenticity and Content Fidelity	2.3 Pharmacotherapeutic Accuracy	Gemini 2.0 Pro	5	5	5
**44**	AML	Clinical Authenticity and Content Fidelity	2.3 Pharmacotherapeutic Accuracy	Gemini 2.0 Pro	2	2	2
**45**	AML	Clinical Authenticity and Content Fidelity	2.3 Pharmacotherapeutic Accuracy	Gemini 2.0 Pro	2	2	2
**46**	AML	Clinical Authenticity and Content Fidelity	2.3 Pharmacotherapeutic Accuracy	Gemini 2.0 Pro	5	5	5
**47**	AML	Clinical Authenticity and Content Fidelity	2.3 Pharmacotherapeutic Accuracy	Gemini 2.0 Pro	5	5	5
**48**	AML	Clinical Authenticity and Content Fidelity	2.3 Pharmacotherapeutic Accuracy	Gemini 2.0 Pro	5	5	5
**49**	AML	Clinical Authenticity and Content Fidelity	2.3 Pharmacotherapeutic Accuracy	Gemini 2.0 Pro	5	5	5
**50**	AML	Clinical Authenticity and Content Fidelity	2.3 Pharmacotherapeutic Accuracy	Gemini 2.0 Pro	5	5	5
**1**	AML	Clinical Authenticity and Content Fidelity	2.4 Domain Specificity	DeepSeek V2	1	4	1
**2**	AML	Clinical Authenticity and Content Fidelity	2.4 Domain Specificity	DeepSeek V2	1	4	1
**3**	AML	Clinical Authenticity and Content Fidelity	2.4 Domain Specificity	DeepSeek V2	5	5	5
**4**	AML	Clinical Authenticity and Content Fidelity	2.4 Domain Specificity	DeepSeek V2	5	5	5
**5**	AML	Clinical Authenticity and Content Fidelity	2.4 Domain Specificity	DeepSeek V2	5	5	5
**6**	AML	Clinical Authenticity and Content Fidelity	2.4 Domain Specificity	DeepSeek V2	5	5	5
**7**	AML	Clinical Authenticity and Content Fidelity	2.4 Domain Specificity	DeepSeek V2	5	5	5
**8**	AML	Clinical Authenticity and Content Fidelity	2.4 Domain Specificity	DeepSeek V2	5	5	5
**9**	AML	Clinical Authenticity and Content Fidelity	2.4 Domain Specificity	DeepSeek V2	5	5	5
**10**	AML	Clinical Authenticity and Content Fidelity	2.4 Domain Specificity	DeepSeek V2	5	5	5
**11**	AML	Clinical Authenticity and Content Fidelity	2.4 Domain Specificity	Claude 3.7 Sonnet	5	5	5
**12**	AML	Clinical Authenticity and Content Fidelity	2.4 Domain Specificity	Claude 3.7 Sonnet	5	5	5

Supplementary D

**13**	AML	Clinical Authenticity and Content Fidelity	2.4 Domain Specificity	Claude 3.7 Sonnet	5	5	5
**14**	AML	Clinical Authenticity and Content Fidelity	2.4 Domain Specificity	Claude 3.7 Sonnet	5	5	5
**15**	AML	Clinical Authenticity and Content Fidelity	2.4 Domain Specificity	Claude 3.7 Sonnet	5	5	5
**16**	AML	Clinical Authenticity and Content Fidelity	2.4 Domain Specificity	Claude 3.7 Sonnet	5	5	5
**17**	AML	Clinical Authenticity and Content Fidelity	2.4 Domain Specificity	Claude 3.7 Sonnet	5	5	5
**18**	AML	Clinical Authenticity and Content Fidelity	2.4 Domain Specificity	Claude 3.7 Sonnet	5	5	5
**19**	AML	Clinical Authenticity and Content Fidelity	2.4 Domain Specificity	Claude 3.7 Sonnet	5	5	5
**20**	AML	Clinical Authenticity and Content Fidelity	2.4 Domain Specificity	Claude 3.7 Sonnet	5	5	5
**21**	AML	Clinical Authenticity and Content Fidelity	2.4 Domain Specificity	Claude 3.7 Sonnet	1	1	1
**22**	AML	Clinical Authenticity and Content Fidelity	2.4 Domain Specificity	GPT 4.o	5	5	5
**23**	AML	Clinical Authenticity and Content Fidelity	2.4 Domain Specificity	GPT 4.o	5	5	5
**24**	AML	Clinical Authenticity and Content Fidelity	2.4 Domain Specificity	GPT 4.o	5	5	5
**25**	AML	Clinical Authenticity and Content Fidelity	2.4 Domain Specificity	GPT 4.o	5	5	5
**26**	AML	Clinical Authenticity and Content Fidelity	2.4 Domain Specificity	GPT 4.o	5	5	5
**27**	AML	Clinical Authenticity and Content Fidelity	2.4 Domain Specificity	GPT 4.o	5	5	5
**28**	AML	Clinical Authenticity and Content Fidelity	2.4 Domain Specificity	GPT 4.o	5	5	5
**29**	AML	Clinical Authenticity and Content Fidelity	2.4 Domain Specificity	GPT 4.o	5	5	5
**30**	AML	Clinical Authenticity and Content Fidelity	2.4 Domain Specificity	GPT 4.o	5	5	5
**31**	AML	Clinical Authenticity and Content Fidelity	2.4 Domain Specificity	GPT 4.o	5	5	5
**32**	AML	Clinical Authenticity and Content Fidelity	2.4 Domain Specificity	GPT 4.o	5	5	5
**33**	AML	Clinical Authenticity and Content Fidelity	2.4 Domain Specificity	GPT 4.o	1	3	1
**34**	AML	Clinical Authenticity and Content Fidelity	2.4 Domain Specificity	Gemini 2.0 Pro	5	5	5
**35**	AML	Clinical Authenticity and Content Fidelity	2.4 Domain Specificity	Gemini 2.0 Pro	5	5	5

Supplementary D

**36**	AML	Clinical Authenticity and Content Fidelity	2.4 Domain Specificity	Gemini 2.0 Pro	5	5	5
**37**	AML	Clinical Authenticity and Content Fidelity	2.4 Domain Specificity	Gemini 2.0 Pro	5	5	5
**38**	AML	Clinical Authenticity and Content Fidelity	2.4 Domain Specificity	Gemini 2.0 Pro	5	5	5
**39**	AML	Clinical Authenticity and Content Fidelity	2.4 Domain Specificity	Gemini 2.0 Pro	5	5	5
**40**	AML	Clinical Authenticity and Content Fidelity	2.4 Domain Specificity	Gemini 2.0 Pro	5	5	5
**41**	AML	Clinical Authenticity and Content Fidelity	2.4 Domain Specificity	Gemini 2.0 Pro	5	5	5
**42**	AML	Clinical Authenticity and Content Fidelity	2.4 Domain Specificity	Gemini 2.0 Pro	5	5	5
**43**	AML	Clinical Authenticity and Content Fidelity	2.4 Domain Specificity	Gemini 2.0 Pro	5	5	5
**44**	AML	Clinical Authenticity and Content Fidelity	2.4 Domain Specificity	Gemini 2.0 Pro	5	5	5
**45**	AML	Clinical Authenticity and Content Fidelity	2.4 Domain Specificity	Gemini 2.0 Pro	5	5	5
**46**	AML	Clinical Authenticity and Content Fidelity	2.4 Domain Specificity	Gemini 2.0 Pro	5	5	5
**47**	AML	Clinical Authenticity and Content Fidelity	2.4 Domain Specificity	Gemini 2.0 Pro	5	5	5
**48**	AML	Clinical Authenticity and Content Fidelity	2.4 Domain Specificity	Gemini 2.0 Pro	5	5	5
**49**	AML	Clinical Authenticity and Content Fidelity	2.4 Domain Specificity	Gemini 2.0 Pro	5	5	5
**50**	AML	Clinical Authenticity and Content Fidelity	2.4 Domain Specificity	Gemini 2.0 Pro	5	5	5
**1**	AML	Modeled Clinical Reasoning	3.1 Problem Identification & Framing	DeepSee k V2	5	5	5
**2**	AML	Modeled Clinical Reasoning	3.1 Problem Identification & Framing	DeepSee k V2	5	4	5
**3**	AML	Modeled Clinical Reasoning	3.1 Problem Identification & Framing	DeepSee k V2	5	4	5
**4**	AML	Modeled Clinical Reasoning	3.1 Problem Identification & Framing	DeepSee k V2	5	4	5
**5**	AML	Modeled Clinical Reasoning	3.1 Problem Identification & Framing	DeepSee k V2	5	4	5
**6**	AML	Modeled Clinical Reasoning	3.1 Problem Identification & Framing	DeepSee k V2	5	4	5
**7**	AML	Modeled Clinical Reasoning	3.1 Problem Identification & Framing	DeepSee k V2	5	4	5
**8**	AML	Modeled Clinical Reasoning	3.1 Problem Identification & Framing	DeepSee k V2	5	4	5

Supplementary D

**9**	AML	Modeled Clinical Reasoning	3.1 Problem Identification & Framing	DeepSeek V2	5	4	5
**10**	AML	Modeled Clinical Reasoning	3.1 Problem Identification & Framing	DeepSeek V2	5	4	5
**11**	AML	Modeled Clinical Reasoning	3.1 Problem Identification & Framing	Claude 3.7 Sonnet	5	5	5
**12**	AML	Modeled Clinical Reasoning	3.1 Problem Identification & Framing	Claude 3.7 Sonnet	5	5	5
**13**	AML	Modeled Clinical Reasoning	3.1 Problem Identification & Framing	Claude 3.7 Sonnet	5	5	5
**14**	AML	Modeled Clinical Reasoning	3.1 Problem Identification & Framing	Claude 3.7 Sonnet	5	5	5
**15**	AML	Modeled Clinical Reasoning	3.1 Problem Identification & Framing	Claude 3.7 Sonnet	5	5	5
**16**	AML	Modeled Clinical Reasoning	3.1 Problem Identification & Framing	Claude 3.7 Sonnet	5	5	5
**17**	AML	Modeled Clinical Reasoning	3.1 Problem Identification & Framing	Claude 3.7 Sonnet	5	5	5
**18**	AML	Modeled Clinical Reasoning	3.1 Problem Identification & Framing	Claude 3.7 Sonnet	5	5	5
**19**	AML	Modeled Clinical Reasoning	3.1 Problem Identification & Framing	Claude 3.7 Sonnet	5	5	5
**20**	AML	Modeled Clinical Reasoning	3.1 Problem Identification & Framing	Claude 3.7 Sonnet	5	5	5
**21**	AML	Modeled Clinical Reasoning	3.1 Problem Identification & Framing	Claude 3.7 Sonnet	5	2	5
**22**	AML	Modeled Clinical Reasoning	3.1 Problem Identification & Framing	GPT 4.o	5	5	5
**23**	AML	Modeled Clinical Reasoning	3.1 Problem Identification & Framing	GPT 4.o	5	4	5
**24**	AML	Modeled Clinical Reasoning	3.1 Problem Identification & Framing	GPT 4.o	5	5	5
**25**	AML	Modeled Clinical Reasoning	3.1 Problem Identification & Framing	GPT 4.o	5	5	5
**26**	AML	Modeled Clinical Reasoning	3.1 Problem Identification & Framing	GPT 4.o	5	5	5

Supplementary D

**27**	AML	Modeled Clinical Reasoning	3.1 Problem Identification & Framing	GPT 4.o	5	5	5
**28**	AML	Modeled Clinical Reasoning	3.1 Problem Identification & Framing	GPT 4.o	5	4	5
**29**	AML	Modeled Clinical Reasoning	3.1 Problem Identification & Framing	GPT 4.o	5	4	5
**30**	AML	Modeled Clinical Reasoning	3.1 Problem Identification & Framing	GPT 4.o	5	5	5
**31**	AML	Modeled Clinical Reasoning	3.1 Problem Identification & Framing	GPT 4.o	5	5	5
**32**	AML	Modeled Clinical Reasoning	3.1 Problem Identification & Framing	GPT 4.o	5	3	5
**33**	AML	Modeled Clinical Reasoning	3.1 Problem Identification & Framing	GPT 4.o	5	3	5
**34**	AML	Modeled Clinical Reasoning	3.1 Problem Identification & Framing	Gemini 2.0 Pro	4	4	4
**35**	AML	Modeled Clinical Reasoning	3.1 Problem Identification & Framing	Gemini 2.0 Pro	5	5	5
**36**	AML	Modeled Clinical Reasoning	3.1 Problem Identification & Framing	Gemini 2.0 Pro	5	4	5
**37**	AML	Modeled Clinical Reasoning	3.1 Problem Identification & Framing	Gemini 2.0 Pro	5	5	5
**38**	AML	Modeled Clinical Reasoning	3.1 Problem Identification & Framing	Gemini 2.0 Pro	5	5	5
**39**	AML	Modeled Clinical Reasoning	3.1 Problem Identification & Framing	Gemini 2.0 Pro	5	3	5
**40**	AML	Modeled Clinical Reasoning	3.1 Problem Identification & Framing	Gemini 2.0 Pro	5	3	5
**41**	AML	Modeled Clinical Reasoning	3.1 Problem Identification & Framing	Gemini 2.0 Pro	5	3	5
**42**	AML	Modeled Clinical Reasoning	3.1 Problem Identification & Framing	Gemini 2.0 Pro	5	5	5
**43**	AML	Modeled Clinical Reasoning	3.1 Problem Identification & Framing	Gemini 2.0 Pro	5	5	5
**44**	AML	Modeled Clinical Reasoning	3.1 Problem Identification & Framing	Gemini 2.0 Pro	5	4	5

Supplementary D

**45**	AML	Modeled Clinical Reasoning	3.1 Problem Identification & Framing	Gemini 2.0 Pro	5	4	5
**46**	AML	Modeled Clinical Reasoning	3.1 Problem Identification & Framing	Gemini 2.0 Pro	5	5	5
**47**	AML	Modeled Clinical Reasoning	3.1 Problem Identification & Framing	Gemini 2.0 Pro	5	5	5
**48**	AML	Modeled Clinical Reasoning	3.1 Problem Identification & Framing	Gemini 2.0 Pro	5	5	5
**49**	AML	Modeled Clinical Reasoning	3.1 Problem Identification & Framing	Gemini 2.0 Pro	5	5	5
**50**	AML	Modeled Clinical Reasoning	3.1 Problem Identification & Framing	Gemini 2.0 Pro	5	5	5
**1**	AML	Modeled Clinical Reasoning	3.2 Diagnostic and Therapeutic Breadth	DeepSeek V2	5	5	5
**2**	AML	Modeled Clinical Reasoning	3.2 Diagnostic and Therapeutic Breadth	DeepSeek V2	5	4	5
**3**	AML	Modeled Clinical Reasoning	3.2 Diagnostic and Therapeutic Breadth	DeepSeek V2	5	4	5
**4**	AML	Modeled Clinical Reasoning	3.2 Diagnostic and Therapeutic Breadth	DeepSeek V2	5	4	5
**5**	AML	Modeled Clinical Reasoning	3.2 Diagnostic and Therapeutic Breadth	DeepSeek V2	5	4	5
**6**	AML	Modeled Clinical Reasoning	3.2 Diagnostic and Therapeutic Breadth	DeepSeek V2	5	4	5
**7**	AML	Modeled Clinical Reasoning	3.2 Diagnostic and Therapeutic Breadth	DeepSeek V2	5	4	5
**8**	AML	Modeled Clinical Reasoning	3.2 Diagnostic and Therapeutic Breadth	DeepSeek V2	5	4	5
**9**	AML	Modeled Clinical Reasoning	3.2 Diagnostic and Therapeutic Breadth	DeepSeek V2	5	4	5
**10**	AML	Modeled Clinical Reasoning	3.2 Diagnostic and Therapeutic Breadth	DeepSeek V2	5	4	5
**11**	AML	Modeled Clinical Reasoning	3.2 Diagnostic and Therapeutic Breadth	Claude 3.7 Sonnet	5	5	5
**12**	AML	Modeled Clinical Reasoning	3.2 Diagnostic and Therapeutic Breadth	Claude 3.7 Sonnet	4	4	4
**13**	AML	Modeled Clinical Reasoning	3.2 Diagnostic and Therapeutic Breadth	Claude 3.7 Sonnet	5	5	5
**14**	AML	Modeled Clinical Reasoning	3.2 Diagnostic and Therapeutic Breadth	Claude 3.7 Sonnet	4	4	4
**15**	AML	Modeled Clinical Reasoning	3.2 Diagnostic and Therapeutic Breadth	Claude 3.7 Sonnet	5	5	5

Supplementary D

**16**	AML	Modeled Clinical Reasoning	3.2 Diagnostic and Therapeutic Breadth	Claude 3.7 Sonnet	5	5	5
**17**	AML	Modeled Clinical Reasoning	3.2 Diagnostic and Therapeutic Breadth	Claude 3.7 Sonnet	5	5	5
**18**	AML	Modeled Clinical Reasoning	3.2 Diagnostic and Therapeutic Breadth	Claude 3.7 Sonnet	4	4	4
**19**	AML	Modeled Clinical Reasoning	3.2 Diagnostic and Therapeutic Breadth	Claude 3.7 Sonnet	4	4	4
**20**	AML	Modeled Clinical Reasoning	3.2 Diagnostic and Therapeutic Breadth	Claude 3.7 Sonnet	4	4	4
**21**	AML	Modeled Clinical Reasoning	3.2 Diagnostic and Therapeutic Breadth	Claude 3.7 Sonnet	4	4	4
**22**	AML	Modeled Clinical Reasoning	3.2 Diagnostic and Therapeutic Breadth	GPT 4.o	5	3	5
**23**	AML	Modeled Clinical Reasoning	3.2 Diagnostic and Therapeutic Breadth	GPT 4.o	5	4	5
**24**	AML	Modeled Clinical Reasoning	3.2 Diagnostic and Therapeutic Breadth	GPT 4.o	5	5	5
**25**	AML	Modeled Clinical Reasoning	3.2 Diagnostic and Therapeutic Breadth	GPT 4.o	5	5	5
**26**	AML	Modeled Clinical Reasoning	3.2 Diagnostic and Therapeutic Breadth	GPT 4.o	5	4	5
**27**	AML	Modeled Clinical Reasoning	3.2 Diagnostic and Therapeutic Breadth	GPT 4.o	5	5	5
**28**	AML	Modeled Clinical Reasoning	3.2 Diagnostic and Therapeutic Breadth	GPT 4.o	5	4	5
**29**	AML	Modeled Clinical Reasoning	3.2 Diagnostic and Therapeutic Breadth	GPT 4.o	5	4	5
**30**	AML	Modeled Clinical Reasoning	3.2 Diagnostic and Therapeutic Breadth	GPT 4.o	5	5	5
**31**	AML	Modeled Clinical Reasoning	3.2 Diagnostic and Therapeutic Breadth	GPT 4.o	5	5	5
**32**	AML	Modeled Clinical Reasoning	3.2 Diagnostic and Therapeutic Breadth	GPT 4.o	4	4	4
**33**	AML	Modeled Clinical Reasoning	3.2 Diagnostic and Therapeutic Breadth	GPT 4.o	5	3	5
**34**	AML	Modeled Clinical Reasoning	3.2 Diagnostic and Therapeutic Breadth	Gemini 2.0 Pro	3	3	3
**35**	AML	Modeled Clinical Reasoning	3.2 Diagnostic and Therapeutic Breadth	Gemini 2.0 Pro	5	1	5
**36**	AML	Modeled Clinical Reasoning	3.2 Diagnostic and Therapeutic Breadth	Gemini 2.0 Pro	5	4	5
**37**	AML	Modeled Clinical Reasoning	3.2 Diagnostic and Therapeutic Breadth	Gemini 2.0 Pro	5	5	5
**38**	AML	Modeled Clinical Reasoning	3.2 Diagnostic and Therapeutic Breadth	Gemini 2.0 Pro	5	5	5
**39**	AML	Modeled Clinical Reasoning	3.2 Diagnostic and Therapeutic Breadth	Gemini 2.0 Pro	5	3	5

Supplementary D

**40**	AML	Modeled Clinical Reasoning	3.2 Diagnostic and Therapeutic Breadth	Gemini 2.0 Pro	5	3	5
**41**	AML	Modeled Clinical Reasoning	3.2 Diagnostic and Therapeutic Breadth	Gemini 2.0 Pro	5	3	5
**42**	AML	Modeled Clinical Reasoning	3.2 Diagnostic and Therapeutic Breadth	Gemini 2.0 Pro	5	5	5
**43**	AML	Modeled Clinical Reasoning	3.2 Diagnostic and Therapeutic Breadth	Gemini 2.0 Pro	5	5	5
**44**	AML	Modeled Clinical Reasoning	3.2 Diagnostic and Therapeutic Breadth	Gemini 2.0 Pro	5	4	5
**45**	AML	Modeled Clinical Reasoning	3.2 Diagnostic and Therapeutic Breadth	Gemini 2.0 Pro	5	4	5
**46**	AML	Modeled Clinical Reasoning	3.2 Diagnostic and Therapeutic Breadth	Gemini 2.0 Pro	5	5	5
**47**	AML	Modeled Clinical Reasoning	3.2 Diagnostic and Therapeutic Breadth	Gemini 2.0 Pro	5	5	5
**48**	AML	Modeled Clinical Reasoning	3.2 Diagnostic and Therapeutic Breadth	Gemini 2.0 Pro	5	5	5
**49**	AML	Modeled Clinical Reasoning	3.2 Diagnostic and Therapeutic Breadth	Gemini 2.0 Pro	5	5	5
**50**	AML	Modeled Clinical Reasoning	3.2 Diagnostic and Therapeutic Breadth	Gemini 2.0 Pro	5	5	5
**1**	AML	Modeled Clinical Reasoning	3.3 Prioritization and Decision-Making	DeepSeek V2	5	5	5
**2**	AML	Modeled Clinical Reasoning	3.3 Prioritization and Decision-Making	DeepSeek V2	5	4	5
**3**	AML	Modeled Clinical Reasoning	3.3 Prioritization and Decision-Making	DeepSeek V2	5	4	5
**4**	AML	Modeled Clinical Reasoning	3.3 Prioritization and Decision-Making	DeepSeek V2	4	3	4
**5**	AML	Modeled Clinical Reasoning	3.3 Prioritization and Decision-Making	DeepSeek V2	5	3	5
**6**	AML	Modeled Clinical Reasoning	3.3 Prioritization and Decision-Making	DeepSeek V2	5	3	5
**7**	AML	Modeled Clinical Reasoning	3.3 Prioritization and Decision-Making	DeepSeek V2	5	3	5
**8**	AML	Modeled Clinical Reasoning	3.3 Prioritization and Decision-Making	DeepSeek V2	5	3	5
**9**	AML	Modeled Clinical Reasoning	3.3 Prioritization and Decision-Making	DeepSeek V2	5	3	5
**10**	AML	Modeled Clinical Reasoning	3.3 Prioritization and Decision-Making	DeepSeek V2	5	4	5
**11**	AML	Modeled Clinical Reasoning	3.3 Prioritization and Decision-Making	Claude 3.7 Sonnet	5	5	5
**12**	AML	Modeled Clinical Reasoning	3.3 Prioritization and Decision-Making	Claude 3.7 Sonnet	5	5	5
**13**	AML	Modeled Clinical Reasoning	3.3 Prioritization and Decision-Making	Claude 3.7 Sonnet	5	5	5
**14**	AML	Modeled Clinical Reasoning	3.3 Prioritization and Decision-Making	Claude 3.7 Sonnet	5	5	5

Supplementary D

**15**	AML	Modeled Clinical Reasoning	3.3 Prioritization and Decision-Making	Claude 3.7 Sonnet	5	5	5
**16**	AML	Modeled Clinical Reasoning	3.3 Prioritization and Decision-Making	Claude 3.7 Sonnet	4	4	4
**17**	AML	Modeled Clinical Reasoning	3.3 Prioritization and Decision-Making	Claude 3.7 Sonnet	5	5	5
**18**	AML	Modeled Clinical Reasoning	3.3 Prioritization and Decision-Making	Claude 3.7 Sonnet	4	4	4
**19**	AML	Modeled Clinical Reasoning	3.3 Prioritization and Decision-Making	Claude 3.7 Sonnet	4	4	4
**20**	AML	Modeled Clinical Reasoning	3.3 Prioritization and Decision-Making	Claude 3.7 Sonnet	4	4	4
**21**	AML	Modeled Clinical Reasoning	3.3 Prioritization and Decision-Making	Claude 3.7 Sonnet	3	2	3
**22**	AML	Modeled Clinical Reasoning	3.3 Prioritization and Decision-Making	GPT 4.o	3	3	3
**23**	AML	Modeled Clinical Reasoning	3.3 Prioritization and Decision-Making	GPT 4.o	5	4	5
**24**	AML	Modeled Clinical Reasoning	3.3 Prioritization and Decision-Making	GPT 4.o	5	5	5
**25**	AML	Modeled Clinical Reasoning	3.3 Prioritization and Decision-Making	GPT 4.o	5	5	5
**26**	AML	Modeled Clinical Reasoning	3.3 Prioritization and Decision-Making	GPT 4.o	5	5	5
**27**	AML	Modeled Clinical Reasoning	3.3 Prioritization and Decision-Making	GPT 4.o	5	5	5
**28**	AML	Modeled Clinical Reasoning	3.3 Prioritization and Decision-Making	GPT 4.o	4	3	4
**29**	AML	Modeled Clinical Reasoning	3.3 Prioritization and Decision-Making	GPT 4.o	5	4	5
**30**	AML	Modeled Clinical Reasoning	3.3 Prioritization and Decision-Making	GPT 4.o	5	5	5
**31**	AML	Modeled Clinical Reasoning	3.3 Prioritization and Decision-Making	GPT 4.o	5	5	5
**32**	AML	Modeled Clinical Reasoning	3.3 Prioritization and Decision-Making	GPT 4.o	1	1	1
**33**	AML	Modeled Clinical Reasoning	3.3 Prioritization and Decision-Making	GPT 4.o	1	1	1
**34**	AML	Modeled Clinical Reasoning	3.3 Prioritization and Decision-Making	Gemini 2.0 Pro	4	4	4
**35**	AML	Modeled Clinical Reasoning	3.3 Prioritization and Decision-Making	Gemini 2.0 Pro	5	3	5
**36**	AML	Modeled Clinical Reasoning	3.3 Prioritization and Decision-Making	Gemini 2.0 Pro	3	3	3
**37**	AML	Modeled Clinical Reasoning	3.3 Prioritization and Decision-Making	Gemini 2.0 Pro	5	5	5
**38**	AML	Modeled Clinical Reasoning	3.3 Prioritization and Decision-Making	Gemini 2.0 Pro	5	5	5

Supplementary D

**39**	AML	Modeled Clinical Reasoning	3.3 Prioritization and Decision-Making	Gemini 2.0 Pro	5	3	5
**40**	AML	Modeled Clinical Reasoning	3.3 Prioritization and Decision-Making	Gemini 2.0 Pro	5	3	5
**41**	AML	Modeled Clinical Reasoning	3.3 Prioritization and Decision-Making	Gemini 2.0 Pro	5	3	5
**42**	AML	Modeled Clinical Reasoning	3.3 Prioritization and Decision-Making	Gemini 2.0 Pro	5	5	5
**43**	AML	Modeled Clinical Reasoning	3.3 Prioritization and Decision-Making	Gemini 2.0 Pro	5	5	5
**44**	AML	Modeled Clinical Reasoning	3.3 Prioritization and Decision-Making	Gemini 2.0 Pro	5	4	5
**45**	AML	Modeled Clinical Reasoning	3.3 Prioritization and Decision-Making	Gemini 2.0 Pro	4	3	4
**46**	AML	Modeled Clinical Reasoning	3.3 Prioritization and Decision-Making	Gemini 2.0 Pro	5	5	5
**47**	AML	Modeled Clinical Reasoning	3.3 Prioritization and Decision-Making	Gemini 2.0 Pro	5	5	5
**48**	AML	Modeled Clinical Reasoning	3.3 Prioritization and Decision-Making	Gemini 2.0 Pro	5	5	5
**49**	AML	Modeled Clinical Reasoning	3.3 Prioritization and Decision-Making	Gemini 2.0 Pro	5	5	5
**50**	AML	Modeled Clinical Reasoning	3.3 Prioritization and Decision-Making	Gemini 2.0 Pro	5	5	5
**1**	AML	Modeled Clinical Reasoning	3.4 Modeling of Clinical Outcomes	DeepSeek V2	5	5	5
**2**	AML	Modeled Clinical Reasoning	3.4 Modeling of Clinical Outcomes	DeepSeek V2	5	4	5
**3**	AML	Modeled Clinical Reasoning	3.4 Modeling of Clinical Outcomes	DeepSeek V2	5	4	5
**4**	AML	Modeled Clinical Reasoning	3.4 Modeling of Clinical Outcomes	DeepSeek V2	5	4	5
**5**	AML	Modeled Clinical Reasoning	3.4 Modeling of Clinical Outcomes	DeepSeek V2	5	4	5
**6**	AML	Modeled Clinical Reasoning	3.4 Modeling of Clinical Outcomes	DeepSeek V2	5	4	5
**7**	AML	Modeled Clinical Reasoning	3.4 Modeling of Clinical Outcomes	DeepSeek V2	5	4	5
**8**	AML	Modeled Clinical Reasoning	3.4 Modeling of Clinical Outcomes	DeepSeek V2	5	4	5
**9**	AML	Modeled Clinical Reasoning	3.4 Modeling of Clinical Outcomes	DeepSeek V2	5	4	5
**10**	AML	Modeled Clinical Reasoning	3.4 Modeling of Clinical Outcomes	DeepSeek V2	5	4	5
**11**	AML	Modeled Clinical Reasoning	3.4 Modeling of Clinical Outcomes	Claude 3.7 Sonnet	5	5	5
**12**	AML	Modeled Clinical Reasoning	3.4 Modeling of Clinical Outcomes	Claude 3.7 Sonnet	5	5	5
**13**	AML	Modeled Clinical Reasoning	3.4 Modeling of Clinical Outcomes	Claude 3.7 Sonnet	5	5	5

Supplementary D

**14**	AML	Modeled Clinical Reasoning	3.4 Modeling of Clinical Outcomes	Claude 3.7 Sonnet	4	4	4
**15**	AML	Modeled Clinical Reasoning	3.4 Modeling of Clinical Outcomes	Claude 3.7 Sonnet	5	5	5
**16**	AML	Modeled Clinical Reasoning	3.4 Modeling of Clinical Outcomes	Claude 3.7 Sonnet	5	5	5
**17**	AML	Modeled Clinical Reasoning	3.4 Modeling of Clinical Outcomes	Claude 3.7 Sonnet	5	5	5
**18**	AML	Modeled Clinical Reasoning	3.4 Modeling of Clinical Outcomes	Claude 3.7 Sonnet	3	3	3
**19**	AML	Modeled Clinical Reasoning	3.4 Modeling of Clinical Outcomes	Claude 3.7 Sonnet	3	3	3
**20**	AML	Modeled Clinical Reasoning	3.4 Modeling of Clinical Outcomes	Claude 3.7 Sonnet	4	4	4
**21**	AML	Modeled Clinical Reasoning	3.4 Modeling of Clinical Outcomes	Claude 3.7 Sonnet	3	2	3
**22**	AML	Modeled Clinical Reasoning	3.4 Modeling of Clinical Outcomes	GPT 4.o	5	4	5
**23**	AML	Modeled Clinical Reasoning	3.4 Modeling of Clinical Outcomes	GPT 4.o	4	3	4
**24**	AML	Modeled Clinical Reasoning	3.4 Modeling of Clinical Outcomes	GPT 4.o	5	5	5
**25**	AML	Modeled Clinical Reasoning	3.4 Modeling of Clinical Outcomes	GPT 4.o	5	5	5
**26**	AML	Modeled Clinical Reasoning	3.4 Modeling of Clinical Outcomes	GPT 4.o	5	4	5
**27**	AML	Modeled Clinical Reasoning	3.4 Modeling of Clinical Outcomes	GPT 4.o	5	5	5
**28**	AML	Modeled Clinical Reasoning	3.4 Modeling of Clinical Outcomes	GPT 4.o	5	4	5
**29**	AML	Modeled Clinical Reasoning	3.4 Modeling of Clinical Outcomes	GPT 4.o	5	4	5
**30**	AML	Modeled Clinical Reasoning	3.4 Modeling of Clinical Outcomes	GPT 4.o	5	5	5
**31**	AML	Modeled Clinical Reasoning	3.4 Modeling of Clinical Outcomes	GPT 4.o	5	5	5
**32**	AML	Modeled Clinical Reasoning	3.4 Modeling of Clinical Outcomes	GPT 4.o	2	2	2
**33**	AML	Modeled Clinical Reasoning	3.4 Modeling of Clinical Outcomes	GPT 4.o	2	2	2
**34**	AML	Modeled Clinical Reasoning	3.4 Modeling of Clinical Outcomes	Gemini 2.0 Pro	2	3	2
**35**	AML	Modeled Clinical Reasoning	3.4 Modeling of Clinical Outcomes	Gemini 2.0 Pro	4	4	4
**36**	AML	Modeled Clinical Reasoning	3.4 Modeling of Clinical Outcomes	Gemini 2.0 Pro	5	4	5

Supplementary D

**37**	AML	Modeled Clinical Reasoning	3.4 Modeling of Clinical Outcomes	Gemini 2.0 Pro	5	5	5
**38**	AML	Modeled Clinical Reasoning	3.4 Modeling of Clinical Outcomes	Gemini 2.0 Pro	5	5	5
**39**	AML	Modeled Clinical Reasoning	3.4 Modeling of Clinical Outcomes	Gemini 2.0 Pro	5	3	5
**40**	AML	Modeled Clinical Reasoning	3.4 Modeling of Clinical Outcomes	Gemini 2.0 Pro	5	3	5
**41**	AML	Modeled Clinical Reasoning	3.4 Modeling of Clinical Outcomes	Gemini 2.0 Pro	5	3	5
**42**	AML	Modeled Clinical Reasoning	3.4 Modeling of Clinical Outcomes	Gemini 2.0 Pro	5	5	5
**43**	AML	Modeled Clinical Reasoning	3.4 Modeling of Clinical Outcomes	Gemini 2.0 Pro	5	5	5
**44**	AML	Modeled Clinical Reasoning	3.4 Modeling of Clinical Outcomes	Gemini 2.0 Pro	5	4	5
**45**	AML	Modeled Clinical Reasoning	3.4 Modeling of Clinical Outcomes	Gemini 2.0 Pro	5	4	5
**46**	AML	Modeled Clinical Reasoning	3.4 Modeling of Clinical Outcomes	Gemini 2.0 Pro	5	5	5
**47**	AML	Modeled Clinical Reasoning	3.4 Modeling of Clinical Outcomes	Gemini 2.0 Pro	5	5	5
**48**	AML	Modeled Clinical Reasoning	3.4 Modeling of Clinical Outcomes	Gemini 2.0 Pro	5	5	5
**49**	AML	Modeled Clinical Reasoning	3.4 Modeling of Clinical Outcomes	Gemini 2.0 Pro	5	5	5
**50**	AML	Modeled Clinical Reasoning	3.4 Modeling of Clinical Outcomes	Gemini 2.0 Pro	5	5	5

Supplementary D

Supplementary Table 17. Expert Evaluation: Session-Level Success Rates and Statistical Comparisons

Analysis	Comparison	Success Rate (n/N, %)	95% CI	Effect Size	Statistical Test	p-value
**Overall**	All platforms	53/103 (51.5%)	[41.7, 61.2]	—	—	—
**Platform Comparison**	Gemini	18/29 (62.1%)	[44.0, 77.3]	V = 0.224	$\chi^2(3)$ = 5.17	0.16
	Claude	13/22 (59.1%)	[38.7, 76.7]			
	DeepSeek	12/23 (52.2%)	[33.0, 70.8]			
	GPT-4o	10/29 (34.5%)	[19.9, 52.7]			
**Disease Type Comparison**	CML	33/53 (62.3%)	[48.8, 74.1]	OR (CML vs AML) = 2.50 [1.12, 5.56]	Fisher's exact	0.031
	AML	20/50 (40.0%)	[27.6, 53.8]	RR (CML/AML) = 1.56 [1.04, 2.32]		
				h = 0.449		
**Domain-Specific Disease Comparisons†**						p-value / q-value
**Clinical Accuracy & Safety**	CML	35/53 (66.0%)	[52.6, 77.4]	OR (CML vs AML) = 1.96 [0.85, 4.52]	Fisher's exact	0.116 / 0.174
	AML	25/50 (50.0%)	[36.6, 63.4]	RR (CML/AML) = 1.32 [0.93, 1.88]		
				h = 0.324		
**Clinical Reasoning Fidelity**	CML	47/53 (88.7%)	[77.5, 94.7]	OR (CML vs AML) = 2.78 [0.89, 8.72]	Fisher's exact	0.082 / 0.174
	AML	37/50 (74.0%)	[60.5, 84.1]	RR (CML/AML) = 1.20 [0.98, 1.46]		
				h = 0.388		
**Instructional Design Quality**	CML	44/53 (83.0%)	[70.8, 91.0]	OR (CML vs AML) = 1.07 [0.39, 2.91]	Fisher's exact	1.000 / 1.000
	AML	41/50 (82.0%)	[69.2, 90.3]	RR (CML/AML) = 1.01 [0.87, 1.18]		
				h = 0.029		
**Within-Platform Disease Comparisons‡**						
**Gemini (CML vs AML)**	CML	9/12 (75.0%)	[46.8, 91.1]	OR (CML vs AML) = 2.57 [0.46, 18.00]	Fisher's exact	0.273
	AML	9/17 (52.9%)	[31.0, 73.8]	RR (CML/AML) = 1.42 [0.82, 2.44]		
				h = 0.455		
**Claude (CML vs AML)**	CML	7/11 (63.6%)	[35.4, 84.8]	OR (CML vs AML) = 1.44 [0.25, 8.50]	Fisher's exact	1

Supplementary D

	AML	6/11 (54.5%)	[28.0, 78.7]	RR (CML/AML) = 1.17 [0.57, 2.39]		
				h = 0.185		
**DeepSeek (CML vs AML)**	CML	11/13 (84.6%)	[57.8, 95.7]	OR (CML vs AML) = 50.0 [3.85, ∞]	Fisher’s exact	0.001
	AML	1/10 (10.0%)	[1.8, 40.4]	RR (CML/AML) = 8.46 [1.28, 55.87]		
				h = 1.983		
**GPT-4o (CML vs AML)**	CML	4/17 (23.5%)	[9.6, 47.3]	OR (CML vs AML) = 0.55 [0.11, 2.63]	Fisher’s exact	1
	AML	6/12 (50.0%)	[24.7, 75.3]	RR (CML/AML) = 0.47 [0.18, 1.23]		
				h = 0.549		

Note: Overall success rate was 53/103 (51.5%, 95% CI [41.7, 61.2]). Success criteria required simultaneous achievement across all three domains: Clinical Accuracy & Safety (all 4 subdomains ≥4.0), Clinical Reasoning Fidelity (all 4 subdomains ≥4.0), and Instructional Design Quality (all subdomains >3.0 with domain mean >4.0). Post-hoc power analysis revealed inadequate power for all platform comparisons (5.5%–39.7%) and for the disease type comparison (62%, below the conventional 80% threshold). All odds ratios are expressed as CML vs AML (values >1 indicate higher odds of success in CML); risk ratios are expressed as CML/AML. Effect size abbreviations: V = Cramér's V; OR = Odds Ratio; RR = Risk Ratio; h = Cohen's h.

† Domain-specific disease comparisons constitute a family of three related tests examining whether disease type differentially affects specific performance domains; FDR correction using the Benjamini-Hochberg procedure (α = 0.05) was applied, with format shown as uncorrected p-value / FDR-corrected q-value. Zero tests remained significant after correction.

‡ Within-platform disease comparisons represent exploratory post-hoc analyses examining platform × disease interaction patterns; these were not adjusted for multiple comparisons given their exploratory nature and are presented for complete transparency regarding platform-specific performance variation. GPT-4o session counts reflect the corrected distribution: CML n = 17, AML n = 12 (total n = 29). Gemini session counts reflect the corrected distribution: CML n = 12, AML n = 17 (total n = 29)

Supplementary D

Supplementary Table 18. Student Satisfaction: Overall Preferences, Platform Comparison, and Safety Alignment

Analysis	Metric/Platform	Value	95% CI	Effect Size	Statistical Test	p-value
**Sample & Reliability**						
	Total students (item responses)	102 (816 responses; 102 students × 8 dimensions)	—	α = 0.939	Cronbach's α	—
	Platform distribution	Claude: 21 (20.6%); DeepSeek: 24 (23.5%); GPT-4o: 28 (27.5%); Gemini: 29 (28.4%)	—	—	—	—
**Overall Preference**						
	Mean satisfaction score	3.41 (SD = 1.44)	—	d = 0.282	t(815) = 8.05	<0.001
	Favor LLM vs Neutral vs Traditional	49.8% vs 20.2% vs 30.0%	—	w = 0.368	$\chi^2(2)$ = 110.79	<0.001
**Platform Satisfaction**						
	DeepSeek	Mean = 3.68 (SD = 1.14)	—	$\varepsilon^2 \approx 0$†	H(3) = 2.69	0.442
	Gemini	Mean = 3.41 (SD = 1.23)	—			
	GPT-4o	Mean = 3.38 (SD = 1.32)	—			
	Claude	Mean = 3.11 (SD = 1.03)	—			
**Dimension-Specific Preference‡**						
	Ease of Use	65/102 (63.7%) favor LLM	[54.1, 72.4]	h = 0.278	Binomial test	0.007
	Time Saving	63/102 (61.8%) favor LLM	[52.1, 70.6]	h = 0.238	Binomial test	0.022
	Clinical Practice Realism	38/102 (37.3%) favor LLM	[28.5, 46.9]	h = −0.258	Binomial test	0.013
	Diagnostic Skills Improvement	48/102 (47.1%) favor LLM	[37.7, 56.7]	h = −0.059	Binomial test	0.621
	Clinical Confidence Building	43/102 (42.2%) favor LLM	[33.0, 51.9]	h = −0.158	Binomial test	0.137
	Learning Enjoyment	49/102 (48.0%) favor LLM	[38.6, 57.6]	h = −0.039	Binomial test	0.767
	Exam Preparation	46/102 (45.1%) favor LLM	[35.8, 54.8]	h = −0.098	Binomial test	0.373
	Future Use Intent	54/102 (52.9%) favor LLM	[43.3, 62.3]	h = 0.059	Binomial test	0.621
**Safety-Preference Alignment**						
	Spearman correlation	ρ = −0.80	—	—	Spearman (n = 4)	0.2
	Claude	Safety: 77.3%; Preference: 52.4%	—			
	Gemini	Safety: 69.0%; Preference: 58.6%	—			

Supplementary D

DeepSeek	Safety: 52.2%; Preference: 62.5%	—
GPT-4o	Safety: 37.9%; Preference: 60.7%	—

Note: Item responses = 102 students × 8 Likert dimensions; Cronbach's α computed on the 8-item response matrix. Effect size abbreviations: α = Cronbach's alpha; d = Cohen's d (one-sample, vs midpoint 3.0); w = Cohen's w; ε^2 = epsilon-squared; h = Cohen's h (vs null proportion 0.50); ρ = Spearman's rho. Safety pass rates represent the proportion of sessions meeting Clinical Accuracy & Safety domain criteria (all 4 subdomains ≥4.0). Preference percentages represent the proportion of students per platform reporting mean satisfaction score >3.0.

† Epsilon-squared (ε^2) is the appropriate effect size for the Kruskal-Wallis H test; the value is approximately zero, indicating a negligible between-platform effect on satisfaction scores.

‡ Binomial tests used a null proportion of $p_0 = 0.50$ (no preference). Dimension-specific analyses represent exploratory decomposition of the overall significant preference pattern and were not adjusted for multiple comparisons; interpretation should focus on effect size magnitudes alongside p-values. With n = 4 platforms, bootstrap confidence intervals for Spearman's ρ are computationally unreliable and are not reported; the point estimate (ρ = −0.80) and p-value (p = 0.200) should be interpreted descriptively given the limited platform-level sample size

Supplementary E

LLMs Sessions Transcripts with Reviewers Comments

Summary

Description	Count
Total Number of Sessions	103
Format	TSV (Tab-Separated Values)
Content	Transcripts with Comments

Session-1

Session ID:
LLM

Session-1:
DeepSeek V2

Session ID:
Session Transcript

Session-1:
Phase 1: Initial Presentation & Diagnostic Workup
Case Introduction:
A 58-year-old woman presents with 3 weeks of fatigue, gingival bleeding, and recurrent infections.
- Lab Results: Hb 7.2 g/dL, platelets 32,000/L, WBC 65,000/L with 50% blasts.
- Bone Marrow Biopsy: Hypercellular with 70% blasts; flow cytometry confirms AML.

Question 1/15:
What is the most urgent next step?
A) Start cytarabine + daunorubicin ("7+3") induction therapy
B) Initiate hydroxyurea for leukocytosis
C) Perform molecular testing (NPM1, FLT3, CEBPA) and karyotyping
D) Administer prophylactic antibiotics and transfuse platelets

Type your answer (A, B, C, or D).
Need help? Type "HINT" for guidance.
Feedback on Question 1/15 (Answer: C)

Correct Answer: C) Perform molecular testing (NPM1, FLT3, CEBPA) and karyotyping
- Rationale: Per **ESMO 2021 guidelines**, comprehensive molecular/cytogenetic profiling is *urgently needed* before initiating therapy to:
  1. Define ELN 2022 risk category (e.g., *NPM1+* = favorable risk; *FLT3-ITDhigh* = adverse risk).
  2. Guide induction therapy (e.g., adding midostaurin for *FLT3+* AML).
  3. Avoid delays in targeted therapy eligibility (e.g., IDH inhibitors).

Why Not Other Options?
A) "7+3" induction: Premature without molecular profiling (e.g., *FLT3+* AML requires midostaurin).
B) Hydroxyurea: Only for *symptomatic* hyperleukocytosis (WBC >100,000/L with leukostasis). This patients WBC is 65,000/L without respiratory/neurologic symptoms.
D) Supportive care: Important (e.g., transfuse platelets if bleeding), but not *definitive* without diagnostics.
Next Steps:
Molecular testing reveals: *NPM1 mutation*, *FLT3-ITDlow*, normal karyotype.

Question 2/15:
How would you classify this patients risk per ELN 2022?
A) Favorable
B) Intermediate
C) Adverse
D) Indeterminate

Type your answer (A, B, C, or D).
Struggling? Type "HINT" for risk stratification criteria.

Feedback on Question 2/15 (Answer: A)

Correct Answer: A) Favorable
- Rationale: Per **ELN 2022 guidelines**, this patients AML falls into the *favorable risk* group because:
 - NPM1 mutation is present (favorable marker).
 - FLT3-ITDlow** allele ratio (<0.5) does *not* negate the favorable impact of *NPM1+*.
 - Normal karyotype excludes adverse cytogenetics (e.g., complex or monosomal karyotypes).

Why Not Other Options?
B) Intermediate: Would apply if *NPM1+* co-occurred with *FLT3-ITDhigh* (allele ratio 0.5) or if no mutations were detected (wild-type).
C) Adverse: Requires high-risk features (e.g., *TP53* mutation, *FLT3-ITDhigh* without *NPM1+,* or adverse cytogenetics).
D) Indeterminate: Used only when testing is incomplete/inconclusive (e.g., insufficient sample for karyotyping).
Clinical Implications:
Favorable-risk AML has:
- Higher likelihood of achieving **complete remission (CR)** with standard induction.
- Potential for **lower-intensity post-remission therapy** (e.g., chemotherapy alone vs. transplant in first CR).

Question 3/15:
The patient is clinically stable with WBC now 25,000/L (after hydroxyurea). Which induction regimen is most appropriate?
A) "7+3" (cytarabine + daunorubicin)
B) "7+3" + midostaurin
C) Venetoclax + azacitidine
D) CPX-351 (liposomal daunorubicin/cytarabine)

Type your answer (A, B, C, or D).
Need guidance? Type "HINT" for ESMO recommendations on induction therapy.
Feedback on Question 3/15 (Answer: A)

Correct Answer: A) "7+3" (cytarabine + daunorubicin)
- Rationale:
 - This patient has **favorable-risk AML (NPM1+, FLT3-ITDlow, normal karyotype)** and is clinically stable, making **"7+3" the standard induction** per **ESMO 2021 guidelines**.
 - Midostaurin (B)** is *not required* for *FLT3-ITDlow* (only *FLT3-ITDhigh*).
 - Venetoclax + azacitidine (C)** is reserved for *unfit* patients (not first-line for fit, favorable-risk AML).
 - **CPX-351 (D)** is for *therapy-related AML* or *AML with myelodysplasia-related changes* (not this case).

Why Not Other Options?
B) "7+3" + midostaurin: Overtreatment for *FLT3-ITDlow* (no survival benefit per RATIFY trial).
C) Venetoclax-based therapy**: Less effective than intensive chemo in *fit* patients with favorable-risk disease.
D) CPX-351**: No evidence of superiority in *de novo* AML without high-risk features.
Clinical Pearl:
- Fitness matters: Always assess cardiac, renal, and performance status (e.g., "7+3" requires LVEF >40%, CrCl 60 mL/min).
- **FLT3-ITDlow** does *not* change induction but may influence post-remission strategies (e.g., transplant if MRD+).

Question 4/15:
Prior to starting "7+3," which supportive care measure is most critical?
A) Posaconazole prophylaxis
B) Allopurinol for tumor lysis prevention
C) G-CSF priming
D) Central venous catheter placement

Type your answer (A, B, C, or D).
Unsure? Type "HINT" for supportive care priorities in AML induction.

Feedback on Question 4/15 (Answer: A)

Correct Answer: A) Posaconazole prophylaxis
- Rationale:
  - Invasive fungal infections (IFIs)** are a leading cause of mortality during AML induction. Posaconazole (or voriconazole/isavuconazole) is strongly recommendedmfor antifungal prophylaxis in patients receiving intensive chemotherapy (per **ESMO 2021 and IDSA guidelines**).
  - Impact: Reduces incidence of aspergillosis by >60% compared to fluconazole (Cornely et al., *NEJM* 2007).

Why Not Other Options?
B) Allopurinol: Important but *secondary* to infection prevention. Used for tumor lysis prophylaxis (though this patients WBC is now 25,000/Llow risk for clinical TLS).
C) G-CSF priming: Controversial in AML; no clear survival benefit in most cases (ESMO does not routinely recommend).
D) Central line placement: Logistically important but not immediately life-saving like antifungal prophylaxis.
Clinical Pearl:
- Hierarchy of supportive car:
  1. Infection prevention (antifungal + antibacterial prophylaxis).
  2. Tumor lysis management (allopurinol/rasburicase for high-risk cases).
  3. Transfusion support (platelets for bleeding, Hb <7 g/dL).

Question 5/15:
On day 5 of "7+3," the patient develops fever (39C) and neutropenia (ANC 100/L). Blood cultures are pending. What is the next step?
A) Add empiric vancomycin
B) Start cefepime monotherapy
C) Hold all antibiotics until cultures result
D) Initiate liposomal amphotericin B

Type your answer (A, B, C, or D).
Need a nudge? Type "HINT" for febrile neutropenia management.
Feedback on Question 5/15 (Answer: B)

Correct Answer: B) Start cefepime monotherapy
- Rationale:
  - Febrile neutropenia in AML requires *immediate* empiric antibiotics**. **Cefepime** (or meropenem/piperacillin-tazobactam) is first-line per **IDSA 2022 guidelines** due to:
    1. Broad-spectrum coverage (including *P. aeruginosa*).
    2. Lower nephrotoxicity risk vs. vancomycin.
  - Blood cultures should be drawn first, but therapy *cannot wait* for results (mortality increases with delays).

Why Not Other Options?
A) Add vancomycin: Only for *suspected* Gram-positive infections (e.g., hypotension, catheter-site redness). Not empiric.
C) Hold antibiotics: Unacceptablemortality risk rises sharply with treatment delays (>1 hour from fever onset is suboptimal).
D) Liposomal amphotericin B: Reserved for *persistent fever after 4872 hours* of antibiotics (not first-line).

Clinical Pearl:
- Febrile neutropenia workup:
  1. Immediate cultures (blood, urine, catheter sites if present).
  2. Chest imaging if respiratory symptoms (e.g., CT for fungal pneumonia).
  3. Antibiotics within 1 hour** (cefepime/meropenem).

Phase 2 Transition:
The patient completes "7+3" and achieves **morphologic CR (blasts <5%)** on day 28 bone marrow.

Question 6/15:

What is the *next step* to assess treatment response more precisely?**
A) Repeat bone marrow biopsy in 4 weeks
B) Perform *NPM1* MRD testing by qPCR
C) Proceed directly to consolidation therapy
D) PET-CT to evaluate extramedullary disease

Type your answer (A, B, C, or D).
Stuck? Type "HINT" for MRD monitoring in AML.

Feedback on Question 6/15 (Answer: B)

Correct Answer: B) Perform *NPM1* MRD testing by qPCR**
- Rationale:
  -This patient has **NPM1-mutated AML**, and **qPCR for *NPM1* transcripts is the gold standard for MRD assessment** (per **ELN 2022 guidelines**).
  - clinical Impact:
    - MRD positivity after induction predicts higher relapse risk (even in morphologic CR).
    - MRD negativity correlates with longer remission duration (80% vs. 30% 3-year survival if MRD+).

Why Not Other Options?
A) Repeat marrow in 4 weeks: Delays actionable MRD data. Morphology alone is insensitive (misses 1 in 4 MRD+ cases).
C) Proceed to consolidation: Premature without MRD stratification (e.g., MRD+ may need intensified therapy).
D) PET-CT: AML is rarely extramedullary (unlike lymphoma); PET has no role in routine response assessment.
Clinical Pearl:
- MRD methods vary by marker:
  - NPM1 qPCR (sensitivity 1 in 10⁵-10⁶ cells).
  - No molecular marker?* Use flow cytometry (MFC-MRD; sensitivity 1 in 10⁴).

Question 7/15:
The *NPM1* MRD result is positive (0.1% blasts by qPCR). Which post-remission strategy is preferred?
A) High-dose cytarabine (HiDAC) 4 cycles
B) Allogeneic hematopoietic cell transplant (allo-HCT)
C) Venetoclax + azacitidine maintenance
D) Repeat induction with "7+3"

Type your answer (A, B, C, or D).
Need help? Type "HINT" for ELN 2022 MRD-guided recommendations.
Feedback on Question 7/15 (Answer: B)

Correct Answer: B) Allogeneic hematopoietic cell transplant (allo-HCT)
- Rationale:
  - Persistent *NPM1* MRD (0.1%) after induction signifies high relapse risk**, even in initially favorable-risk AML (per ELN 2022).
  - Allo-HCT in first CR (CR1) is recommended for MRD+ patients due to:
    1. Graft-vs-leukemia effect (reduces relapse risk by ~50%).
    2. Long-term survival advantage (5-year OS ~60% vs. <30% with chemo alone in MRD+ cases).

Why Not Other Options?
A) HiDAC 4: Standard for MRD- favorable-risk AML, but inadequate for MRD+ disease (relapse risk >70%).
C) Venetoclax + azacitidine: Not proven for MRD eradication in *fit* patients; used in unfit/maintenance settings.
D) Repeat "7+3": No benefitMRD persistence indicates chemoresistance.

Clinical Pearl:
- MRD-directed transplant timing:
- Early referral for **donor search** at first MRD+ result.

- Use **reduced-intensity conditioning (RIC)** if older/comorbid (this patient is 58 and fit).

Question 8/15:
Pre-transplant, the patients *NPM1* MRD rises to 0.5%. What intervention is most appropriate?
A) Proceed directly to allo-HCT
B) Blinatumomab to reduce MRD
C) Switch to CPX-351
D) Add gemtuzumab ozogamicin (GO)

Type your answer (A, B, C, or D).
Hint: Think about MRD debulking before transplant.
Feedback on Question 8/15 (Answer: B)

Correct Answer: B) Blinatumomab to reduce MRD
- Rationale:
  - Rising *NPM1* MRD (0.5%) indicates impending relapse**. Per **ELN 2022**, **MRD debulking pre-transplant improves allo-HCT outcomes.
  - Blinatumomab (bispecific anti-CD3/CD19 antibody) has shown:
    - ~60% MRD clearance rate** in AML (including *NPM1+* cases) via T-cell-mediated cytotoxicity (*Blood Adv* 2021).
    - Better transplant survival vs. proceeding with active MRD (HR 0.4 for relapse).

Why Not Other Options?
A) Proceed to allo-HCT: High relapse risk with MRD 0.5% (3-year survival <20%).
C) CPX-351: No role in MRD eradication (used for induction in therapy-related AML).
D) Gemtuzumab ozogamicin (GO)**: Targets *CD33+* blasts, but no evidence for MRD clearance in *NPM1+* AML.

Clinical Pearl:
- MRD-directed bridging strategies:
  1. Immunotherapy: Blinatumomab or checkpoint inhibitors (e.g., nivolumab).
  2. Targeted therapy: FLT3/IDH inhibitors if applicable (not here).
  3. Low-intensity chemo: Azacitidine + venetoclax (if unfit).

Question 9/15:
Blinatumomab achieves MRD negativity. Which transplant conditioning regimen is preferred?
A) Myeloablative (MAC: busulfan/cyclophosphamide)
B) Reduced-intensity (RIC: fludarabine/busulfan)
C) Non-myeloablative (NMAC: fludarabine/low-dose TBI)
D) Post-transplant cyclophosphamide (PTCy)-based

Type your answer (A, B, C, or D).
Hint: Balance efficacy (graft-vs-leukemia) vs. toxicity in a 58-year-old.
Feedback on Question 9/15 (Answer: B)

Correct Answer: B) Reduced-intensity (RIC: fludarabine/busulfan)
- Rationale:
  - This 58-year-old fit patient with MRD-negative disease an ideal candidate for **RIC**, which balances efficacy and toxicity per **EBMT 2023 guidelines:
    1. Lower treatment-related mortality (TRM) vs. myeloablative regimens (10% vs. 25%).
    2. Equivalent survival in MRD-negative AML (*Blood* 2020 meta-analysis).
    3. Graft-vs-leukemia effect remains potent** with RIC.

Why Not Other Options?
A) Myeloablative (MAC): Overtreatment for MRD-negative disease; higher TRM without survival benefit.

C) Non-myeloablative (NMAC): Less effective in AML (used mainly in older/unfit patients).
D) PTCy-based: Preferred for haploidentical transplants, but no advantage in matched donors.
Clinical Pearl:
- Conditioning intensity depends on:
  1. Disease risk (MRD status, genomics).
  2. Age/comorbidities (RIC for 50 years or HCT-CI 3).
  3. Donor type (MAC often used for mismatched/UCRD).

Question 10/15:
Day +100 post-transplant, the patient remains in MRD-negative CR. Which maintenance therapy is recommended?**
A) Midostaurin
B) Sorafenib
C) Azacitidine
D) None

Type your answer (A, B, C, or D).
Hint: Consider ELN 2022 recommendations for maintenance in favorable-risk AML post-HCT.
Feedback on Question 10/15 (Answer: D)

Correct Answer: D) None
- Rationale:
  - This patient has MRD-negative CR post-transplant with favorable-risk AML (NPM1+/FLT3-ITDlow). Per ELN 2022 and EBMT guidelines:
    1. No maintenance therapy is standard for favorable-risk AML after allo-HCT (*Blood* 2021).
    2. Unnecessary toxicity: Azacitidine/midostaurin have no proven benefit in this context.
  - Exceptions: Maintenance is used for:
    - FLT3-ITDhigh (midostaurin/sorafenib).
    - TP53mut or adverse-risk AML (azacitidine trials).

Why Not Other Options?
A) Midostaurin: Only for FLT3-ITDhigh (this patient is *FLT3-ITDlow*).
B) Sorafenib: Similarly, no role without FLT3-ITDhigh.
C) Azacitidine: No survival benefit in favorable-risk AML (*QUAZAR* trial excluded post-HCT patients).

Clinical Pearl:
- Post-HCT monitoring:
- qPCR for NPM1 every 3 months (detects relapse earlier than morphology).
 - Immunosuppression taper: Rapid reduction if no GVHD (enhances graft-vs-leukemia effect).

Phase 3: Long-Term Management
The patient remains in MRD-negative CR at 2 years post-transplant.

Question 11/15:
Which long-term complication is most likely to require intervention?
A) Chronic GVHD (skin/liver involvement)
B) Secondary myelodysplastic syndrome (MDS)
C) Avascular necrosis (from prior steroids)
D) Relapsed AML

Type your answer (A, B, C, or D).
Hint: Think beyond relapsewhat does transplant survivorship entail?

Feedback on Question 11/15 (Answer: A)

Correct Answer: A) Chronic GVHD (skin/liver involvement)
- Rationale:
  - Chronic GVHD occurs in 30-50% of allo-HCT survivors, typically peaking at 6-12 months post-transplant but can persist for years.
  - Most likely manifestations:
    - Skin (lichen planus-like rash, sclerosis).
    - Liver (elevated ALP/GGT from biliary duct damage).
  - Requires systemic immunosuppression (e.g., prednisone ruxolitinib for steroid-refractory cases).

Why Not Other Options?
B) Secondary MDS: Rare with RIC regimens (more linked to alkylating chemo/radiation).
C) Avascular necrosis: Associated with *high-dose steroids* (unlikely here without prolonged use).
D) Relapsed AML: Very low risk (<10%) in MRD-negative CR at 2 years.
Clinical Pearl:
- GVHD prophylaxis matters:
  - Calcineurin inhibitors (tacrolimus) + methotrexate reduce acute GVHD but increase chronic GVHD risk.
  - Post-transplant cyclophosphamide (PTCy) lowers chronic GVHD rates.

Question 12/15:
The patient develops mild skin GVHD. What is the first-line therapy?
A) Topical corticosteroids
B) Oral prednisone 1 mg/kg/day
C) Ruxolitinib
D) Extracorporeal photopheresis (ECP)

Type your answer (A, B, C, or D).
Hint: NCCN guidelines prioritize stepwise escalation for chronic GVHD.

Feedback on Question 12/15 (Answer: A)

Correct Answer: A) Topical corticosteroids
- Rationale:
  - For mild, localized chronic GVHD (e.g., skin-only), topical corticosteroids (e.g., triamcinolone 0.1% cream)** are first-line per NCCN 2023 guidelines.
  - Advantages:
    1. Avoids systemic immunosuppression side effects (infections, diabetes).
    2. Effective in >60% of limited cases (Biol Blood Marrow Transplant 2018).

Why Not Other Options?
B) Oral prednisone: Reserved for moderate-severe GVHD multiorgan involvement or progression despite topical therapy).
C) Ruxolitinib: Second-line for steroid-refractory GVHD FDA-approved based on REACH trials).
D) Extracorporeal photopheresis (ECP): Used for refractory GVHD or contraindications to steroids.
Clinical Pearl:
- Chronic GVHD workup:
  1. Biopsy to confirm (mimics can include drug rash, viral exanthems).
  2. Organ assessment (liver LFTs, pulmonary function tests, ocular exam).

Question 13/15:
At 3 years post-HCT, routine *NPM1* qPCR detects MRD (0.02%). What is the next step?
A) Immediate donor lymphocyte infusion (DLI)
B) Azacitidine + venetoclax
C) Repeat testing in 4 weeks
D) Start gilteritinib

Type your answer (A, B, C, or D).

Hint: Low-level MRD may represent assay variability vs. early relapse.
Feedback on Question 13/15 (Answer: C)

Correct Answer: C) Repeat testing in 4 weeks
- Rationale:
  - Low-level MRD (0.02%) may reflect assay variability or early relapse. Per ELN 2022 guidelines:
    1. Confirm with repeat testing in 4 weeks (avoid overtreating false positives).
    2. Investigate for rising trends (true relapse typically shows logarithmic MRD increase).
  - Clinical Context:
    - If MRD rises preemptive DLI (durable response in 40-60% of *NPM1+* AML).
    - If stable/negative continue monitoring.

Why Not Other Options?
A) Immediate DLI: Risks GVHD without confirmation of relapse (DLI mortality ~15%).
B) Azacitidine + venetoclax: Used for overt relapse, not isolated low-level MRD.
D) Gilteritinib: Only for FLT3-ITD+ AML (this patient is *FLT3-ITDlow*).
Clinical Pearl:
- MRD kinetics predict relapse:
- Rising MRD: Doubling time <1 month high relapse risk.
- Stable MRD: May represent clonal hematopoiesis (CHIP) or dormancy.

Question 14/15:
Repeat testing shows MRD increased to 0.1%. The patient remains asymptomatic with normal counts. What is the next step?
A) Proceed to DLI
B) Initiate hypomethylating agent (azacitidine)
C) Reinduction with "7+3"
D) Observe until hematologic relapse

Type your answer (A, B, C, or D).
Hint: Preemptive therapy improves outcomes in MRD+ AML (Cancer Discov 2022).
Feedback on Question 14/15 (Answer: B)

Correct Answer: B) Initiate hypomethylating agent (azacitidine)
- Rationale:
- Preemptive azacitidine is preferred for rising MRD post-HCT (per ELN 2022) due to:
    1. Lower GVHD risk vs. DLI (azacitidine modulates alloreactivity).
    2. 30-50% MRD clearance rate (J Clin Oncol 2021).
    3. Bridge to DLI if no response (sequential strategy).
  - DLI (A) is higher-risk but may be used if azacitidine fails.

Why Not Other Options?
- A) DLI: Higher GVHD risk (avoid first-line if asymptomatic/MRD <1%).
- C) "7+3" reinduction: Too aggressive for molecular relapse (no survival benefit over targeted approaches).
- D) Observe: UnacceptableMRD+ precedes hematologic relapse by ~3 months (intervene early!).
Clinical Pearl:
- Preemptive MRD treatment algorithm:
  1. Azacitidine venetoclax (if no GVHD).
  2. DLI (if no response + no GVHD).
  3. Targeted therapy (e.g., FLT3/IDH inhibitors if actionable mutation).

Question 15/15:
The patients MRD becomes negative after 3 cycles of azacitidine. How will you adjust therapy?
A) Continue azacitidine indefinitely
B) Stop azacitidine, monitor q3mo

C) Add venetoclax indefinitely
D) Proceed to second transplant

Type your answer (A, B, C, or D).
Hint: Balance MRD control vs. therapy toxicity in a cured patient.
Feedback on Question 15/15 (Answer: B)

Correct Answer: B) Stop azacitidine, monitor q3mo
- Rationale:
 - MRD negativity after preemptive therapy indicates successful intervention. Per ELN 2022:
   1. Discontinue azacitidine (no proven benefit for continued therapy in MRD- patients).
   2. Monitor q3mo with NPM1 qPCR (relapse risk <10% if MRD- at 1 year post-treatment).
  - Why Not Other Options?
   A) Continue azacitidine: Unnecessary toxicity (myelosuppression, infections) without survival benefit.
   C) Add venetoclax: No role in MRD- maintenance post-HCT (studies ongoing).
   D) Second transplant: Only for active relapse with chemo-sensitive disease.

Session ID:
Assessment Domain

Session-1:
Rationale

Session ID:
Instructional Design and Pedagogical

Session-1:
The session demonstrates expert instructional design with seamless progression from initial presentation through risk stratification, induction, MRD monitoring, transplant decisions, and long-term survivorship care. Feedback is consistently insightful and guideline-anchored, citing current ELN 2022, ESMO 2021, and IDSA 2022 recommendations while providing valuable clinical pearls. Instructions are exceptionally clear with helpful hint prompts. The only minor gap is the absence of explicitly stated, measurable learning objectives at the outset, though educational goals are clearly implied through the case structure.

Session ID:
Clinical Authenticity and Content Fidelity

Session-1:
The session contains a critical and dangerous pharmacotherapeutic error that undermines its clinical fidelity. Plausibility: The patient narrative is highly realistic and internally consistent throughout. Critical Error: Question 8 recommends blinatumomab for MRD debulking in NPM1-mutated AML. Blinatumomab is a CD19-targeted bispecific T-cell engager that is standard of care for B-cell acute lymphoblastic leukemia (ALL), not AML. AML blasts typically express myeloid markers (CD33, CD13) but not CD19. This represents both a dangerous pharmacotherapeutic recommendation and domain entanglementinappropriately applying an ALL-specific therapy to AML. According to the rubric, this single instance of domain entanglement results in the lowest possible scores for both pharmacotherapeutic accuracy and domain specificity, regardless of the session's otherwise excellent clinical content. In Q 3, "Clinical Pearl: Fitness matters: Always assess cardiac, renal, and performance status (e.g., "7+3" requires LVEF >40%)" while Anthracyclines should not be used in patients with EF < 50 %.

Session ID:
Modeled Clinical Reasoning

Session-1:
Despite the critical content error, the simulation models expert clinical reasoning exceptionally well. Each decision point presents a precisely framed clinical problem with comprehensive, nuanced options that include sophisticated distractors. The prioritization demonstrates appropriate risk-adjusted strategies throughout (molecular testing before therapy, MRD-guided transplant decisions,

preemptive intervention for rising MRD). Clinical outcomes are described realistically and reinforce key learning points. The overall reasoning strategy remains soundthe concept of MRD debulking pre-transplant is correct, even though the specific agent choice represents domain entanglement.

Session-2

Session ID:
LLM

Session-2:
DeepSeek V2

Session ID:
Session Transcript

Session-2:
Case Introduction
Patient Profile:

Name: Mr. Robert Chen

Age: 62 years

Gender: Male

PMH: Hypertension (well-controlled), hyperlipidemia

SH: Former smoker (quit 10 years ago), occasional alcohol

FH: Father had colon cancer (dx at 68)

Chief Complaint: Fatigue, bruising, and recurrent infections over the past 4 weeks

Initial Clinical Findings
Physical Exam:

Pallor, petechiae on lower extremities

No hepatosplenomegaly

ECOG PS: 1

Labs:

CBC: Hb 7.8 g/dL, WBC 32 x 10/L (blasts 45%), platelets 28 x 10/L

Chemistry: Creatinine 1.1 mg/dL, LDH 580 U/L

Coagulation: PT/INR normal

Peripheral Blood Smear:

40% blasts with Auer rods

Question 1 (Knowledge Recall - WHO Classification)
Based on the initial findings, which of the following is the most likely diagnosis?
a) Chronic lymphocytic leukemia (CLL)

b) Acute myeloid leukemia (AML) (Correct)
c) Myelodysplastic syndrome (MDS)
d) Aplastic anemia

Correct: The presence of 20% blasts with Auer rods confirms AML per WHO 2022 criteria.

Why not others?

CLL: Lymphocytosis with mature lymphocytes, not blasts.

MDS: Requires <20% blasts.

Aplastic anemia: Pancytopenia without blasts.
Question 2 (Application - Diagnostic Workup)
Which test is most critical to guide risk stratification and therapy selection?
a) Flow cytometry (Correct)
b) Serum protein electrophoresis
c) Direct Coombs test
d) JAK2 mutation assay

Correct: Flow cytometry confirms immunophenotype (e.g., CD33+, CD13+ in AML) and detects aberrant markers.

Why not others?

B: Rules out myeloma (irrelevant here).

C: Evaluates hemolysis (not indicated).

D: Used for myeloproliferative neoplasms.
Question 3 (Analysis - Molecular Testing)
The patients cytogenetics show t(8;21)(q22;q22). What is the next step in molecular testing?
a) BCR-ABL1 PCR
b) FLT3-ITD/NPM1 testing (Correct)
c) BRAF V600E assay
d) PD-L1 immunohistochemistry

Correct: FLT3-ITD/NPM1 testing is mandatory per ELN 2022, even with favorable-risk t(8;21), as it modifies prognosis.

Why not others?

A: For CML/Ph+ ALL.

C/D: Not relevant in AML.
Question 4 (Application Risk Stratification & Therapy Implications)

The patients molecular testing returns:

FLT3-ITD: Negative

NPM1: Negative

KIT D816V: Positive

Based on ELN 2022, how does this modify his risk category?

a) Remains favorable-risk (t(8;21) alone defines prognosis)
b) Upgraded to adverse-risk due to KIT mutation (Correct)
c) Downgraded to intermediate-risk due to lack of FLT3-ITD
d) Insufficient data to reclassify

Correct Answer: (b)

ELN 2022 Rule: KIT mutations in CBF AML (e.g., t(8;21) or inv(16)) convert favorable-risk to adverse-risk due to higher relapse rates (Grade A evidence).

Clinical Impact:

Requires more intensive post-remission therapy (e.g., allogeneic transplant in eligible patients).

May benefit from KIT-targeted trials (e.g., dasatinib in combination).

Why Not Others?

(a): KIT mutations override favorable-risk cytogenetics.

(c): FLT3-ITD absence doesnt mitigate KITs adverse effect.

(d): Data is conclusive per guidelines.
Question 5 (Evaluation Induction Therapy Selection)
The patients ECOG PS is 1, and cardiac function is normal. Which induction regimen is most appropriate?

a) 7+3 (cytarabine + daunorubicin)
b) Venetoclax + azacitidine
c) CPX-351 (liposomal 7+3)
d) Gemtuzumab ozogamicin + 7+3 (Correct for CBF AML with KIT mutation)

Correct Answer: (d)

ESMO 2021 Recommendation: GemOz + 7+3 is preferred for CBF AML (improves OS in t(8;21), even with KIT mutations).

Rationale:

KIT mutation increases relapse risk GemOz (anti-CD33) adds targeted efficacy.

Avoid venetoclax-based regimens (less effective in CBF AML).

Why Not Others?

(a): Standard but lacks targeted action against KIT.

(b): Preferred for unfit/unmutated AML, not CBF.

(c): Reserved for secondary/therapy-related AML.
Question 6 (Analysis Supportive Care in AKI)
Which supportive care measure is contraindicated?

a) Allopurinol for TLS prophylaxis
b) Levofloxacin for antibacterial prophylaxis (Correct avoid in AKI)

c) Pegfilgrastim for neutropenia prophylaxis
d) Platelet transfusions for <10k/mm

Correct Answer: (b)

Levofloxacin requires renal dose adjustment (avoid if CrCl <50 mL/min). Use ciprofloxacin instead.

Key Adjustments in AKI:

Allopurinol: Reduce dose by 50% (or switch to rasburicase if high TLS risk).

Pegfilgrastim: No renal clearance safe.
Question 7 (Evaluation Managing Induction Complications)

Mr. Chen develops febrile neutropenia (FN) on Day 5 of induction. Blood cultures grow E. coli resistant to ciprofloxacin but sensitive to meropenem. His AKI persists (Cr 2.6 mg/dL). Which empiric regimen is most appropriate?

a) Ceftazidime-avibactam
b) Meropenem + vancomycin (Correct covers resistant E. coli and empiric G+)
c) Ciprofloxacin + micafungin
d) Piperacillin-tazobactam (avoid in AKI)
Correct Answer: (b)

ESMO 2023 FN Guidelines:

Carbapenems (meropenem) are preferred for ESBL-risk or resistant Gram-negative infections.

Vancomycin added empirically for suspected G+ infections (e.g., mucositis-related bacteremia).

Renal Adjustment: Meropenem requires dose reduction (e.g., 500 mg q8h if CrCl <50).

Why Not Others?

(a): Avoid in FN without confirmed carbapenemase-producing organisms.

(c): Ciprofloxacin is contraindicated (resistance + AKI).

(d): Piperacillin-tazobactam requires renal adjustment and may increase bleeding risk with thrombocytopenia.
Question 8 (Analysis MRD Monitoring Post-Induction)
On Day 28, bone marrow shows morphologic remission but MRD by flow cytometry is 0.1%. What is the next step?

a) Proceed to consolidation with high-dose cytarabine
b) Switch to salvage therapy (e.g., FLAG-IDA)
c) Repeat MRD testing in 4 weeks (Correct ELN 2022 allows transient MRD+)
d) Immediate allogeneic transplant
Correct Answer: (c)

ELN 2022 MRD Guidelines:

Low-level MRD (0.1%) post-induction may reflect delayed clearance (especially in CBF AML).

Repeat testing in 4 weeks before escalating therapy.

If MRD persists: Consider gemtuzumab ozogamicin or transplant.

Why Not Others?

(a): High-dose cytarabine is premature with MRD+.

(b): Salvage therapy is for refractory disease (not MRD+ alone).

(d): Transplant requires confirmed persistent MRD.
phase 3: Post-Remission Therapy & Long-Term Management
New Complexity: MRD remains 0.08% at 8 weeks.

Question 9 (Application Transplant Eligibility)
Which factor most strongly supports allogeneic transplant in this patient?

a) Age 62 years
b) Persistent MRD + adverse-risk KIT mutation (Correct ELN 2022 Category)
c) Hypertension history
d) Prior febrile neutropenia

Final Question (Synthesis Long-Term Care)
Which maintenance therapy is justified if transplant is deferred?

a) Venetoclax + azacitidine
b) Sorafenib (off-label for KIT-mutated AML) (Correct emerging data)
c) Pembrolizumab
d) No maintenance (observation)
Correct Answer: (b)

ELN 2022 Transplant Criteria:

Adverse-risk AML (KIT-mutated CBF) + persistent MRD = strong indication for transplant.

Age 62 is not prohibitive if PS and organ function are adequate (HCT-CI score assessed).

Why Not Others?

(a): Age alone doesnt preclude transplant (up to 7075 if fit).

(c/d): Comorbidities must be quantified via HCT-CI.

Correct Answer: (b)

Emerging Evidence: Sorafenib has activity in KIT-mutated AML (Blood Adv 2021).

ESMO 2023: No standard maintenance, but clinical trials (e.g., KIT inhibitors) are preferred.

Why Not Others?

(a): No role in CBF AML.

(c): PD-1 inhibitors lack evidence in AML.

(d): High relapse risk justifies experimental approaches.
Question 10 (Evaluation Transplant Workup & Donor Selection)

The patient is deemed eligible for allogeneic transplant. Which donor source is preferred given his age (62) and lack of matched siblings?

a) Matched unrelated donor (MUD) (10/10 HLA-matched) (Correct preferred per EBMT 2023)
b) Haploidentical donor (e.g., child)
c) Cord blood transplant
d) Autologous transplant
Correct Answer: (a)

EBMT 2023 Guidelines:

MUD (10/10 HLA-matched) is first-choice for fit patients 60 years with no sibling donor.

Equivalent outcomes to matched siblings in modern cohorts (reduced-intensity conditioning).

Why Not Others?

(b): Haploidentical is acceptable but higher GVHD risk vs. MUD.

(c): Cord blood has delayed engraftment (higher infection risk).

(d): No role in AML (high relapse risk).

Question 11 (Application Conditioning Regimen Selection)
Given his KIT mutation and MRD+, which conditioning regimen optimizes relapse prevention?

a) Reduced-intensity conditioning (RIC) with fludarabine/melphalan
b) Myeloablative conditioning (MAC) with busulfan/cyclophosphamide (Correct preferred for adverse-risk MRD+ AML)
c) Non-myeloablative conditioning (flu/2Gy TBI)
d) FLAMSA-RIC (sequential chemo/immunotherapy)

Correct Answer: (b)

EBMT/ELN Consensus:

MAC (e.g., busulfan/cyclophosphamide) is preferred for adverse-risk AML with MRD+ (maximizes disease control).

KIT mutation further justifies intensity (higher relapse risk).

RIC Alternatives: Only if MAC contraindicated (e.g., cardiac dysfunction).

Question 12 (Synthesis Post-Transplant Maintenance)
Which post-transplant strategy is most evidence-based for KIT-mutated AML?

a) Sorafenib maintenance (Correct emerging data for KIT-mutated AML)
b) Venetoclax + azacitidine
c) Donor lymphocyte infusion (DLI) prophylaxis
d) Observation alone
Correct Answer: (a)

BMT CTN 1703 Trial (Blood 2022):

Sorafenib improved 2-year relapse-free survival in FLT3/KIT-mutated AML post-transplant (55% vs. 32%).

Why Not Others?

(b): No proven benefit in CBF AML.

(c): DLI is for relapse (not prophylaxis).

(d): High relapse risk justifies targeted therapy.

Session ID:
Assessment Domain

Session-2:
Rationale

Session ID:
Instructional Design and Pedagogical

Session-2:
The session demonstrates excellent instructional design with logical progression from initial presentation through risk stratification, induction complications, MRD monitoring, and transplant decisions. Feedback is consistently detailed and guideline-anchored, citing current ELN 2022, ESMO 2021, and EBMT 2023 recommendations while providing valuable clinical context. Instructions are clear and intuitive. The only minor gap is the absence of explicitly stated learning objectives, though educational goals are clearly implied through the case structure.

Session ID:
Clinical Authenticity and Content Fidelity

Session-2:
The clinical narrative is highly realistic and internally consistent, but contains a significant pharmacotherapeutic error with domain entanglement. Strengths: Most recommendations are excellentcorrectly identifying KIT mutations as converting CBF AML from favorable to adverse risk, appropriate use of gemtuzumab ozogamicin for CBF AML, and sound transplant decision-making. Critical Error: The session recommends sorafenib maintenance for KIT-mutated AML (Questions 9 and 12), incorrectly citing the BMT CTN 1703 trial. Sorafenib is primarily an FLT3 inhibitor with established efficacy in FLT3-ITD AML, not KIT-mutated AML. This represents domain entanglement within the AML spaceinappropriately applying therapy standards from FLT3-ITD AML to KIT-mutated CBF AML. The BMT CTN 1703 trial specifically studied FLT3-mutated patients, not KIT-mutated patients. In Q 6, Pegfilgrastim is mentioned as an option for neutropenia prophylaxis in AML, while its use is controversial as it may worsen the disease. In Q 7, Vancomycin was used in AKI with no indication.

Session ID:
Modeled Clinical Reasoning

Session-2:
The simulation models expert clinical reasoning with precisely framed problems, sophisticated option sets with effective distractors, and appropriate risk-adjusted prioritization throughout. The breadth of considerationsfrom molecular diagnostics through transplant conditioning regimensreflects comprehensive AML management expertise. Clinical outcomes are realistic and instructive. The overall reasoning strategy remains sound despite the sorafenib error noted in Section 2.

Session-3

Session ID:
LLM

Session-3:
DeepSeek V2

Session ID:
Session Transcript

Session-3:
Phase 1: Initial Presentation & Diagnostic Workup
Case Introduction
Patient: 62-year-old male
Chief Complaint: Fatigue, dyspnea on exertion, and recurrent gum bleeding over 4 weeks.
PMH: Hypertension (well-controlled), hyperlipidemia. No prior cancer history.
Social: Former smoker (quit 10 years ago), occasional alcohol. Works as a school administrator.
Initial Labs (CBC with Differential)
WBC: 32.5 x 10/L ()
Hgb: 7.8 g/dL ()
Platelets: 45 x 10/L ()
Peripheral smear: 25% blasts, Auer rods present
Additional Findings
LDH: 850 U/L ()
Creatinine: 1.1 mg/dL (normal)
ECOG Performance Status: 1
Question 1
What is the most urgent next diagnostic step?
A) Bone marrow biopsy with cytogenetics/NPM1/FLT3-ITD testing
B) Start empiric antibiotics for suspected infection
C) Transfuse platelets and RBCs immediately
D) PET-CT to rule out solid tumor metastasis
Type your answer (A, B, C, or D).
A) Bone marrow biopsy with cytogenetics/NPM1/FLT3-ITD testing
Correct Answer: A) Bone marrow biopsy with cytogenetics/NPM1/FLT3-ITD testing
Feedback:
Why this is correct:
ESMO/ELN guidelines mandate a bone marrow biopsy with molecular/cytogenetic profiling (e.g., NPM1, FLT3-ITD, CEBPA) to confirm AML diagnosis (20% blasts) and guide risk stratification (Dhner et al., Blood 2022).
Auer rods on peripheral smear strongly suggest AML, but genetic data is critical for therapy selection (e.g., FLT3 inhibitor eligibility).
Why the others are incorrect
B) Empiric antibiotics are unnecessary without fever/infection signs. AML alone explains cytopenias.
C) Transfusions may be needed after diagnosis but should not delay biopsy (definitive diagnosis takes priority).
D) PET-CT is not indicated for AML (extramedullary disease is rare and assessed clinically).
Next Steps (After Biopsy Results)
Bone Marrow Report:
Blasts: 65%
Flow cytometry: CD34+, CD117+, HLA-DR+ (consistent with AML)
Cytogenetics: Normal karyotype
Molecular: NPM1 mutation (+), FLT3-ITD (+) with high allelic ratio (0.8), IDH2 (-)
Question 2
How would you risk-stratify this patient per ELN 2022 criteria?

A) Favorable (NPM1 mutation dominates prognosis)
B) Intermediate (normal karyotype with NPM1+)
C) Adverse (FLT3-ITD high allelic ratio confers poor risk)
D) Reclassify after induction response
C) Adverse (FLT3-ITD high allelic ratio confers poor risk)
Correct Answer: C) Adverse (FLT3-ITD high allelic ratio confers poor risk)
Feedback:
Why this is correct:
ELN 2022 guidelines classify AML with FLT3-ITD (high allelic ratio 0.5) as adverse risk, even if NPM1 is mutated (Dhner et al., Blood 2022).
High FLT3-ITD burden is associated with resistance to chemotherapy and higher relapse risk, necessitating FLT3 inhibitor (e.g., midostaurin/gilteritinib) and allogeneic transplant consideration.
Why the others are incorrect:
A) NPM1+ alone would be favorable, but FLT3-ITD high ratio overrides this.
B) Intermediate risk applies to NPM1+ without FLT3-ITD (or with low-ratio FLT3-ITD).
D) While MRD post-induction refines prognosis, baseline genetics determine initial risk stratification.
What is the MOST appropriate induction regimen for this patient?
A) "7+3" (cytarabine + daunorubicin) alone
B) "7+3" + midostaurin
C) Venetoclax + azacitidine (outpatient regimen)
D) CPX-351 (liposomal daunorubicin/cytarabine)
Type your answer (A, B, C, or D).
B) "7+3" + midostaurin
Correct Answer: B) "7+3" + midostaurin
Feedback:
Why this is correct:
RATIFY trial (Stone et al., NEJM 2017) showed midostaurin + "7+3" significantly improves overall survival in FLT3-mutated AML, making this the standard induction per ELN 2022.
Midostaurin is dosed 50 mg PO BID on days 8-21 (synergizes with chemotherapy).
Patient is fit for intensive chemo (ECOG 1, normal organ function).
Why the others are incorrect:
A) "7+3" alone is suboptimalomits FLT3 targeting (adverse risk requires combo therapy).
C) Venetoclax + azacitidine is for unfit patients or those with secondary AML (less effective in FLT3-ITD high-ratio).
D) CPX-351 is reserved for therapy-related AML or AML-MRC (not FLT3-specific).
Question 4
On day 3 of induction, the patient develops fever (38.8C) and hypotension (BP 85/50). Absolute neutrophil count (ANC) is 0.1 x 10/L. What is the NEXT BEST step?
A) Add vancomycin + piperacillin-tazobactam
B) Hold chemotherapy and administer IV fluids + vasopressors
C) Perform CT chest/abdomen to identify source
D) Check midostaurin levels for toxicity
A) Add vancomycin + piperacillin-tazobactam as empiric therapy
Correct Answer: A) Add vancomycin + piperacillin-tazobactam as empiric therapy

Feedback:
Why this is correct:
IDSA guidelines (Freifeld et al., Clin Infect Dis 2011) mandate immediate empiric antibiotics for febrile neutropenia with hypotension (sepsis).
Piperacillin-tazobactam + vancomycin covers Pseudomonas, MRSA, and other Gram-negatives/positives until cultures return.
Time-to-antibiotics <1 hour is critical in neutropenic sepsis (delay increases mortality).
Why the others are incorrect:
B) IV fluids/vasopressors are needed concurrently but do not replace antibiotics.
C) Imaging (e.g., CT) is secondary after stabilizing sepsis (unless focal symptoms).
D) Midostaurin toxicity (e.g., QT prolongation) is unlikely to cause fever/hypotension.

Question 5

On day 21 post-induction, repeat bone marrow shows 5% blasts (morphologic CR but MRD+ by flow). FLT3-ITD remains detectable by PCR (allelic ratio 0.3). What is the NEXT BEST step?

A) Proceed to allo-HSCT in CR1

B) Re-induction with high-dose cytarabine

C) Start gilteritinib monotherapy

D) Delay transplant until MRD negativity

Type your answer (A, B, C, or D)

Key Concept:

MRD+ post-induction in adverse-risk AML (especially FLT3-ITD+) warrants urgent transplant (Schuurhuis et al., Blood 2018).

A) Proceed to allo-HSCT in CR1

Correct Answer: A) Proceed to allo-HSCT in CR1

Feedback:

Why this is correct:

ELN 2022 recommends allo-HSCT in CR1 for adverse-risk AML, especially with:

Persistent FLT3-ITD (allelic ratio >0.1 post-induction) (Dhner et al., Blood 2022).

MRD positivity by flow/PCR (associated with >80% relapse risk without transplant) (Jongen-Lavrencic et al., NEJM 2018).

Timing: Earlier transplant improves outcomes (avoid waiting for MRD negativity in high-risk disease).

Why the others are incorrect:

B) Re-induction is futileFLT3-ITD persistence indicates chemoresistance.

C) Gilteritinib is for relapsed/refractory FLT3+ AML (ADMIRAL trial) or post-HSCT maintenance.

D) Delaying transplant risks relapse (MRD+ FLT3-ITD rarely clears with further chemo).

Transplant Considerations

Conditioning: Myeloablative (e.g., busulfan/cyclophosphamide) if fit; reduced-intensity if comorbidities.

FLT3 inhibitor post-HSCT: Midostaurin/gilteritinib maintenance trials show relapse-free survival benefit (Sandmaier et al., Blood 2022).

MRD monitoring: Post-transplant FLT3-ITD PCR guides early intervention.

Question 6

Prior to transplant, which pre-transplant workup is MOST critical for this patient?

A) Cardiac ejection fraction (MUGA/ECHO)

B) FLT3-ITD allelic ratio re-testing

C) Dental clearance to avoid post-HSCT infections

D) All of the above

Type your answer (A, B, C, or D).

Rationale:

Standard pre-HSCT evaluation includes cardiac, pulmonary, dental, and infection screening (NCCN guidelines).

FLT3-ITD monitoring informs post-transplant maintenance strategies.

D) All of the above

Correct Answer: D) All of the above

Feedback:

Why this is correct:

A) Cardiac EF assessment is mandatory pre-HSCT (myeloablative conditioning risks cardiotoxicity).

B) FLT3-ITD re-testing confirms baseline MRD status for post-transplant monitoring (Schuurhuis et al., Blood 2018).

C) Dental clearance prevents oral infections (e.g., abscesses) during neutropenia post-HSCT.

Clinical Pearl:

Transplant eligibility hinges on organ function and infection control. FLT3-ITD+ patients benefit from integrated pre-HSCT profiling to guide post-transplant FLT3 inhibitor use

Question 7

During pre-HSCT workup, the patient develops febrile neutropenia (ANC 0.2 x 10/L). Blood cultures grow E. coli resistant to piperacillin-tazobactam but sensitive to meropenem. What is the NEXT BEST step?

A) Switch to meropenem + add vancomycin

B) Continue piperacillin-tazobactam + add amikacin

C) Start gilteritinib (FLT3 inhibitor) empirically

D) Delay transplant until infection resolves

Type your answer (A, B, C, or D).
Key Point:
Neutropenic fever with resistant organisms requires escalation to carbapenems (IDSA guidelines). Transplant proceeds after infection clears.

Hint for Question 7 (Stuck Between A & D:
Focus on Two Priorities:
Immediate Infection Control:
E. coli resistant to piperacillin-tazobactam requires escalation to meropenem (IDSA guidelines). Vancomycin is unnecessary unless Gram-positive coverage is needed (e.g., suspected line infection).
Antibiotics must be optimized firsttransplant cannot proceed with active infection.
Transplant Timing:
HSCT is delayed until afebrile + cultures negative (typically 48-72h after appropriate antibiotics).
However, antibiotic adjustment (A) is the immediate action, while transplant delay (D) is a consequence, not the next step.
Key Evidence:
Freifeld et al., Clin Infect Dis 2011: Carbapenems are first-line for resistant Gram-negative neutropenic fever.
Your Decision:
A addresses the urgent need; D is the subsequent plan.
Re-enter your answer (A or D) with this context.
(Type "HINT" again if needed!)
A) Switch to meropenem + add vancomycin
Correct Answer: A) Switch to meropenem + add vancomycin
Feedback:
Why this is correct:
Meropenem is mandatory for ESBL-resistant E. coli (IDSA guidelines).
Vancomycin is added empirically due to:
Risk of concurrent Gram-positive infection (e.g., central line-associated).
Severe neutropenia (ANC <0.5 x 10/L) warrants broad coverage until cultures finalize.
Why D is incorrect in this context:
While transplant must be delayed until infection resolves, the immediate action is antibiotic escalation.
Clinical Nuance:
Vancomycin can be discontinued after 48h if no Gram-positive organisms are isolated.
Transplant delay (D) is implied but not the next stepits a consequence of active infection.
Question 8
The patient clears the infection and proceeds to transplant. Which conditioning regimen is MOST appropriate for this 62-year-old with FLT3-ITD+ AML?
A) Reduced-intensity (fludarabine + busulfan)
B) Myeloablative (busulfan + cyclophosphamide)
C) Non-myeloablative (fludarabine + low-dose TBI)
D) Carmustine + etoposide (BEAM regimen)
Type your answer (A, B, C, or D).
Clue:
Balance efficacy (myeloablative preferred for high-risk AML) vs. age/comorbidity tolerance.
A) Reduced-intensity (fludarabine + busulfan) to take his age into consideration
Correct Answer: A) Reduced-intensity (fludarabine + busulfan)
Feedback:
Why this is correct:
Age 62 + FLT3-ITD+ AML: While myeloablative regimens are preferred for high-risk AML, reduced-intensity conditioning (RIC) is often chosen for patients >60 years to balance efficacy and toxicity (Scott et al., Blood 2017).
Fludarabine/busulfan (Flu/Bu) is a standard RIC regimen with:
Lower treatment-related mortality vs. myeloablative regimens.
Preserved graft-versus-leukemia (GVL) effect.
Why the others are incorrect:
B) Myeloablative (Bu/Cy) is high-risk for toxicity in this age group (though some fit older adults may tolerate it).

C) Non-myeloablative is too weak for FLT3-ITD+ AML (higher relapse risk).
D) BEAM is for lymphoma, not AML.
Question 9
For GVHD prophylaxis, which regimen is MOST appropriate?
A) Tacrolimus + methotrexate
B) Post-transplant cyclophosphamide (PTCy)
C) Sirolimus + mycophenolate mofetil
D) Tacrolimus + ATG (anti-thymocyte globulin)
Type your answer (A, B, C, or D).
A) Tacrolimus + methotrexate as backbone prophylaxis by NCCN guidelines
Correct Answer: A) Tacrolimus + methotrexate

Feedback:
Why this is correct:
NCCN/EBMT guidelines recommend tacrolimus + methotrexate as standard GVHD prophylaxis for matched unrelated donor transplants (NCCN AML 2023).
Tacrolimus: Calcineurin inhibitor (prevents T-cell activation).
Methotrexate: Antimetabolite (reduces early T-cell proliferation).
Evidence: This combo reduces Grade II-IV acute GVHD by ~30% vs. single agents (Ram et al., BBMT 2016).
Why the others are incorrect:
B) PTCy is primarily used in haploidentical transplants (not matched unrelated donors).
C) Sirolimus/MMF is alternative but less studied in AML.
D) ATG is used in mismatched transplants (increases infection risk unnecessarily here).
Question 10
When should midostaurin (FLT3 inhibitor) be restarted post-HSCT?
A) Immediately after engraftment (ANC >500)
B) After tapering tacrolimus (Day +100)
C) Only if FLT3-ITD MRD becomes detectable
D) Never (no role post-HSCT)
Type your answer (A, B, C, or D).
Clue:
Midostaurin maintenance post-HSCT is under investigation but requires immune recovery (e.g., tacrolimus taper) to avoid excess toxicity (Sandmaier et al., Blood 2022).
B) After tapering tacrolimus (Day +100) to avoid toxicity
Correct Answer: B) After tapering tacrolimus (Day +100)
Feedback:
Why this is correct:
ELN 2022 recommends FLT3 inhibitor maintenance post-HSCT for FLT3-ITD+ AML, but timing depends on:
Engraftment stability (avoid overlap with acute GVHD risk period).
Immunosuppression taper (midostaurin + calcineurin inhibitors increase toxicity risks like QT prolongation, renal dysfunction).
Current practice (per SCT CTN 1506 trial): Start FLT3 inhibitors (e.g., midostaurin/gilteritinib) after Day +60-100, once tacrolimus is weaned (Sandmaier et al., Blood 2022).
Why the others are incorrect:
A) Too earlyrisk of hepatic/renal toxicity with concurrent tacrolimus.
C) Waiting for MRD+ risks missed prevention window (early relapse).
D) IncorrectFLT3 inhibitors improve survival post-HSCT in FLT3-ITD+ AML (Perl et al., Lancet Oncol 2021).
Question 11
Which intervention is MOST supported to prevent relapse in this FLT3-ITD+ patient?
A) Venetoclax + azacitidine indefinitely
B) Gilteritinib 120 mg PO daily for 2 years
C) Donor lymphocyte infusion (DLI) now
D) No further therapy (monitor only)
Type your answer (A, B, C, or D).
B) Gilteritinib 120 mg PO daily for 2 years

Absolutely correct, Ashraf!
Feedback:
Why this is correct
ADMIRAL trial extension (Perl et al., Lancet Oncol 2021) showed gilteritinib maintenance post-HSCT significantly improves:
Relapse-free survival (HR 0.44, p<0.001)
Overall survival in FLT3-ITD+ AML.
Dosing: 120 mg PO daily for 2 years (or until progression/unacceptable toxicity).
Timing: Start after tacrolimus taper (reduces drug interactions/GVHD risk).
Why the others are incorrect:
A) Venetoclax + azacitidine lacks evidence in post-HSCT maintenance for FLT3-ITD+ AML.
C) DLI is for overt relapse, not prophylaxis (risk of GVHD).
D) Monitoring alone is inadequateFLT3-ITD+ AML has high relapse risk without targeted therapy.
At Day +180, the patient develops fatigue and thrombocytopenia (plt 80 x 10/L). MRD testing shows FLT3-ITD recurrence (allelic ratio 0.05). What is the NEXT BEST step?
A) Increase gilteritinib to 200 mg daily
B) Add venetoclax to gilteritinib
C) Stop gilteritinib and switch to midostaurin
D) Continue gilteritinib + monitor closely
Type your answer (A, B, C, or D)
Key Point:
Low-level FLT3-ITD+ MRD may reflect emerging resistancebut gilteritinib dose escalation lacks evidence. Consider combination strategies (e.g., with venetoclax) for progressive MRD
B) Add venetoclax to gilteritinib
Correct Answer: B) Add venetoclax to gilteritinib
Feedback:
Why this is correct:
Emerging data show venetoclax + FLT3 inhibitors (e.g., gilteritinib) synergize to overcome resistance (Zhang et al., Cancer Discov 2022).
Rationale for combo therapy in MRD+ relapse:
Venetoclax enhances apoptosis in FLT3-ITD+ clones.
Gilteritinib maintains FLT3 inhibition (avoid stopping abruptly).

Why the others are incorrect:
A) Gilteritinib dose escalation to 200 mg is not FDA-approved and increases toxicity risk (QTc prolongation, myelosuppression).

C) Switching to midostaurin is inferiorgilteritinib has higher potency/specificity for FLT3-ITD.

D) Monitoring risks overt relapseMRD+ warrants intervention.
Question 13
The patient develops grade 2 diarrhea on venetoclax + gilteritinib. Colonoscopy confirms GI GVHD. How would you adjust therapy?
A) Hold both drugs and start prednisone 1 mg/kg
B) Continue gilteritinib but reduce venetoclax dose by 50%
C) Switch to crenolanib (alternative FLT3 inhibitor) + azacitidine
D) Discontinue gilteritinib and continue venetoclax alone

Key Concept:
GVHD takes priority over maintenance therapy. Immunosuppression (e.g., steroids) is first-line, but FLT3 inhibition should be resumed ASAP to prevent relapse.
A) Hold both drugs and start prednisone 1 mg/kg
Correct Answer: A) Hold both drugs and start prednisone 1 mg/kg
Feedback:
Why this is correct:
NCCN Guidelines prioritize GVHD management over maintenance therapy in symptomatic cases.
Steps:

Hold venetoclax/gilteritinib (both can cause GI toxicity, confounding GVHD assessment).
Start prednisone 1 mg/kg/day (first-line for grade 2+ GI GVHD).
Resume FLT3 inhibitor (gilteritinib) once GVHD stabilizesvenetoclax may be delayed or omitted if GI toxicity persists.
Why the others are incorrect:
B) Dose reduction alone ignores GVHD as primary pathology.
C) Crenolanib is not FDA-approved and lacks evidence in this context.
D) Discontinuing gilteritinib risks FLT3-ITD-driven relapse.
Question 14
After 2 weeks, GVHD resolves (steroids tapered to 10 mg/day). How would you restart FLT3-targeted therapy?
A) Resume gilteritinib 120 mg daily alone
B) Restart gilteritinib + venetoclax at 50% doses
C) Switch to sorafenib (non-specific TKI) for lower GI toxicity
D) Delay all therapy until steroids are fully tapered
A) Resume gilteritinib 120 mg daily alone
Correct Answer: A) Resume gilteritinib 120 mg daily alone
Feedback:
Why this is correct:
Prioritize FLT3 inhibition while minimizing GI toxicity:
Gilteritinib alone has lower GI risk than venetoclax combos (Perl et al., Lancet Oncol 2021).
120 mg dose is standard for maintenance (no need to reduce after GVHD resolution).
ELN 2023 recommendations: FLT3 inhibitors should be resumed post-GVHD to prevent relapse, even with concurrent low-dose steroids.
Why the others are incorrect:
B) Re-adding venetoclax now risks GVHD flare (wait until steroids are fully tapered).
C) Sorafenib is inferior to gilteritinib for FLT3-ITD (non-specific TKI with more toxicity).
D) Delaying therapy increases relapse risk (FLT3-ITD clones can expand rapidly)
Question 15 (Final Assessment)
At 1-year post-HSCT, the patient remains in CR (MRD-), but gilteritinib causes persistent grade 2 fatigue and QTc 490 ms. What is the NEXT BEST step?
A) Permanently discontinue gilteritinib
B) Reduce dose to 80 mg daily and monitor QTc
C) Switch to midostaurin 50 mg BID
D) Continue full dose with electrolyte monitoring

Key Point:

QTc >480 ms requires dose adjustment per FDA labeling, but FLT3 inhibition must be maintained in high-risk AML.
B) Reduce dose to 80 mg daily and monitor QTc
Correct Answer: B) Reduce dose to 80 mg daily and monitor QTc
Feedback:
Why this is correct:
FDA labeling for gilteritinib:
QTc >480 ms requires dose reduction to 80 mg daily (not abrupt discontinuation).

Maintain FLT3 inhibition while mitigating cardiac risk (Perl et al., Lancet Oncol 2021).

Monitoring: Repeat ECG after 1 week; correct electrolytes (K+/Mg).

Why the others are incorrect:

A) Discontinuation risks relapse (no alternative FLT3 inhibitor in place).

C) Midostaurin is less potent against FLT3-ITD and also prolongs QTc.

D) Continuing 120 mg is unsafe with QTc >480 ms.

Session ID:
Assessment Domain

Session-3:
Rationale

Session ID:
Instructional Design and Pedagogical

Session-3:
The session demonstrates exceptional instructional design with expert scaffolding from initial presentation through diagnosis, induction therapy, transplant decisions, and long-term post-transplant management of FLT3-ITD+ AML. The feedback is consistently insightful and guideline-anchored, citing current ELN 2022, IDSA, NCCN, and FDA recommendations while providing valuable clinical context and pearls. Instructions include helpful hints and decision-making clues. The only minor gap is the absence of explicitly stated learning objectives, though educational goals are clearly conveyed through the comprehensive case progression.

Session ID:
Clinical Authenticity and Content Fidelity

Session-3:
The session's clinical authenticity is critically undermined by dangerous pharmacotherapeutic errors, despite a plausible narrative. Plausibility & Guideline Alignment: The clinical narrative of an older patient with FLT3-ITD high-risk AML is highly realistic. However, there are significant deviations from evidence-based guidelines. Pharmacotherapeutic Error: A dangerous error occurs in Question 11, which recommends gilteritinib maintenance post-transplant as the "MOST supported" intervention. This is factually incorrect; the pivotal MORPHO trial of gilteritinib maintenance was negative, showing no survival benefit. The provided rationale misinterprets data from the ADMIRAL trial, which established gilteritinib's efficacy in the relapsed/refractory setting, not for post-transplant maintenance. This represents a dangerous recommendation that is contrary to high-level evidence. Additionally, Question 9 inaccurately dismisses post-transplant cyclophosphamide (PTCy) as a valid GVHD prophylaxis option for matched unrelated donor transplants, reflecting outdated knowledge. Domain Specificity: The case remains strictly within the specified disease domain of AML. A distractor option in Question 8 (BEAM regimen) is correctly identified as a therapy for lymphoma, demonstrating appropriate domain awareness.

Session ID:
Modeled Clinical Reasoning

Session-3:
The simulation excels at modeling an expert clinical reasoning process, independent of the content-level errors. Each stage presents a well-defined clinical problem with the necessary data for a decision. The multiple-choice options provide a sophisticated breadth of plausible and relevant alternatives, including common distractors that effectively test nuanced understanding. The narrative demonstrates appropriate prioritization, such as securing molecular results before induction, escalating care for sepsis, and using MRD to guide transplant decisions. The clinical outcomes described are realistic and instructionally valuable. The strategic reasoning is sound throughout, even where the specific drug recommendations are flawed.

Session-4

Session ID:
LLM

Session-4:
DeepSeek V2

Session ID:
Session Transcript

Session-4:

Question 1/15: Initial Workup
Which test is *most critical* to perform next?
a) NPM1/FLT3-ITD mutation testing
b) Lumbar puncture
c) Echocardiogram
d) Bone marrow biopsy with immunophenotyping

(Type your answera, b, c, or dand Ill provide immediate feedback.)

Key Tip: ELN 2022 guidelines emphasize molecular profiling for risk stratification..
Your Answer: d) Bone marrow biopsy with immunophenotyping**

Feedback
Correct! While molecular testing (option *a*) is essential for risk stratification, the **bone marrow biopsy** is the *most critical next step* to:
1. Confirm AML diagnosis (20% blasts).
2. Assess blast morphology and immunophenotype (e.g., CD19+ in t(8;21)).
3. Guide urgent therapy initiation.

Why not the others?
- *a)* NPM1/FLT3 testing is crucial but typically done *concurrently* with biopsy.
- *b)* LP is only needed if CNS symptoms (e.g., headache) arise.
- *c)* Echocardiogram is required *before anthracyclines* but not emergent.
Question 2/15: Induction Therapy
The patient has no comorbidities. Which regimen is preferred?
a) 7+3 (Cytarabine + Daunorubicin)
b) Venetoclax + Azacitidine
c) Gemtuzumab ozogamicin (GO) + 7+3
d) FLAG-IDA

(Choose a/b/c/d. Need a hint? Ask!)
Your Answer: c) Gemtuzumab ozogamicin (GO) + 7+3**

Feedback
 Excellent choice!

Why This is Correct:
- **t(8;21) AML** often expresses **CD33**, making **Gemtuzumab ozogamicin (GO)** highly effective when added to standard **7+3** (Cytarabine + Daunorubicin).
- **Evidence**: The ALFA-0701 trial showed improved survival in core-binding factor (CBF) AML (like t(8;21)) with GO + 7+3 (*Delaunay et al., Blood 2011*).

Why Not the Others?
- *a)* **7+3 alone** is effective but inferior to GO-combined regimens in CBF-AML.
- *b)* **Venetoclax + Azacitidine** is preferred in older/unfit patients, not favorable-risk AML like this case.
- *d)* **FLAG-IDA** is reserved for high-risk/refractory AML.
Question 3/15: Tumor Lysis Prophylaxis**
Given WBC 45k/L, which measure is *least appropriate*?
a) Allopurinol
b) Vigorous IV hydration
c) Raspuricase
d) Immediate dialysis

*(Choose a/b/c/d. Need reasoning? AskYour Answer: d) Immediate dialysis**
Your Answer: d) Immediate dialysis**

Feedback
 Correct!

Why This is the *Least Appropriate* Option:
- **Immediate dialysis** is **not prophylactic**its only used for **established tumor lysis syndrome (TLS) with severe renal failure** (e.g., hyperkalemia, anuria).
- This patient has **no signs of TLS yet** (e.g., elevated creatinine, hyperuricemia), so dialysis is unnecessary.

Expected Prophylaxis for This Patient:
1. **Rasburicase (c)**: **First-line** for high WBC (>25k/L) due to rapid uric acid degradation.
2. **IV hydration (b)**: Mandatory to flush out uric acid/phosphates (e.g., 2-3L/m/day).
3. **Allopurinol (a)**: Less effective than rasburicase here but may be added in mild-risk cases.
Question 4/15: Day 10 of Induction Febrile Neutropenia
Which antibiotic covers *Pseudomonas* empirically?
a) Ciprofloxacin
b) Ceftriaxone
c) Cefepime
d) Vancomycin

*(Choose a/b/c/d. Need a hint? Ask
Your Answer: c) Cefepime**

Feedback
 Spot on!

Why Cefepime is Correct:
- **Cefepime** is a **4th-generation cephalosporin** with reliable *Pseudomonas aeruginosa* coverage, making it a **first-line empiric choice** for febrile neutropenia (per IDSA 2022 guidelines).
- Also covers other Gram-negatives (e.g., *E. coli*, *Klebsiella*) and some Gram-positives (e.g., *Streptococcus*).

Why Not the Others?
- *a) Ciprofloxacin*: Oral and **not broad-spectrum enough** for empiric neutropenic fever (used for prophylaxis or step-down therapy).
- *b) Ceftriaxone*: Lacks consistent *Pseudomonas* coverage.
- *d) Vancomycin*: Only added if MRSA or Gram-positive infection is suspected (e.g., catheter-related, skin soft-tissue signs).
Next Step: The patient starts cefepime. Blood cultures grow *E. coli* (susceptible to cefepime).

Question 5/15: MRD Testing Post-Induction
Which method is *most sensitive* for detecting residual disease in t(8;21) AML?
a) Flow cytometry (0.1% sensitivity)
b) PCR for t(8;21) (0.001% sensitivity)
c) Conventional karyotyping
d) FISH

(Choose a/b/c/d. Need a hint? Ask!)

Key Point: Molecular methods (e.g., PCR) outperform flow cytometry in CBF-AML due to **fusion transcripts** (e.g., *RUNX1-RUNX1T1
Your Answer: b) PCR for t(8;21) (0.001% sensitivity)**

Feedback
Perfect! You've selected the most sensitive and specific MRD detection method for this patient.

Why PCR is the Best Choice
1. **Ultra-Sensitive Detection**:
   - PCR can detect **1 leukemic cell in 100,000 normal cells** (0.001% sensitivity), making it ideal for monitoring residual disease in **t(8;21) AML**.
   - The *RUNX1-RUNX1T1* fusion transcript serves as a reliable molecular marker.

2. **Clinical Impact**:
   - Studies show that **persistent PCR positivity** after therapy predicts higher relapse risk (*Jourdan et al., Blood 2013*).
   - Guides decisions on **consolidation therapy** or early intervention (e.g., transplant if MRD rises).

Why Other Methods Are Less Optimal
- **a) Flow cytometry (0.1% sensitivity)**: Useful for non-CBF AML but misses low-level disease.
- **c) Conventional karyotyping (5% sensitivity)**: Cannot detect MRDonly for initial diagnosis.
- **d) FISH (1-5% sensitivity)**: Less sensitive than PCR; not quantitative.
*Next Step**: The patients post-induction MRD by PCR is **negative** (<0.001%).

Question 6/15: Consolidation Therapy
What is the best next step for this MRD-negative t(8;21) patient?
a) High-dose cytarabine (HiDAC) x 3-4 cycles
b) Allogeneic stem cell transplant
c) Venetoclax + Azacitidine indefinitely
d) Gemtuzumab ozogamicin monotherapy

(Choose a/b/c/d. Need a hint? Ask!)

Key Point: **HiDAC** is standard for favorable-risk AML (like t(8;21)) with MRD-negative remission.
*Your Answer: a) High-dose cytarabine (HiDAC) x 3-4 cycles**

Feedback
 Excellent choice!

Why HiDAC is the Standard for This Patient
1. **ELN 2022 Guidelines**:
   - **t(8;21) AML with MRD-negative remission** after induction is **favorable-risk**.
   - **HiDAC (3 g/m every 12h, days 1/3/5) for 3-4 cycles** is the **preferred consolidation** (*Dhner et al., Blood 2022*).

2. **Outcome Data**:
   - HiDAC achieves **~60-70% long-term survival** in t(8;21) AML (*Byrd et al., JCO 2004*).
   - MRD negativity further improves prognosis (relapse risk <20%).

Why Not the Others?
- *b) Allogeneic transplant*: Overkill for MRD-negative favorable-risk AML (reserved for poor-risk or relapsed disease).
- *c) Venetoclax + Azacitidine*: Used in unfit/unmutated *NPM1* AML, not t(8;21).
- *d) Gemtuzumab monotherapy*: No role in consolidation (used in induction or relapse
Next Step**: The patient completes 4 cycles of HiDAC.

Question 7/15: Long-Term Monitoring
Which test should be used for ongoing MRD surveillance?
a) Monthly bone marrow biopsies
b) Peripheral blood PCR for *RUNX1-RUNX1T1* every 3 months
c) CT scans every 6 months
d) No further testing unless symptoms recur

(Choose a/b/c/d. Need a hint? Ask!)

Key Point: **Peripheral blood PCR** is non-invasive and detects molecular relapse earlier than morphology.
Your Answer: b) Peripheral blood PCR for *RUNX1-RUNX1T1* every 3 months**

Feedback
 Correct! This is the evidence-based monitoring strategy for t(8;21) AML.

Why Peripheral Blood PCR is Ideal
1. **Non-Invasive & Sensitive**:
   - Detects **molecular relapse** (rising *RUNX1-RUNX1T1* transcripts) **3-6 months before clinical/blast relapse** (*Yin et al., Blood 2012*).
   - Avoids unnecessary bone marrow biopsies if blood is negative.

2. **Guideline-Recommended**:
   - ELN 2022 advises **q3-6 month PB PCR** for CBF-AML (e.g., t(8;21), inv(16)) during remission.

Why Not the Others?
- *a) Monthly bone marrow biopsies*: **Too invasive**; no survival benefit over PCR.
- *c) CT scans*: AML is a **marrow disease**imaging is only for suspected extramedullary relapse (rare in t(8;21)).
- *d) No testing*: **Misses early relapse** when intervention (e.g., reinduction/transplant) could be curative.
Phase 3: Relapse Management
Scenario: At 18 months, PB PCR becomes positive (0.1%) but marrow blasts remain <5%.

Question 8/15: Action for Molecular Relapse
What is the next step?
a) Immediate reinduction with 7+3
b) Start venetoclax + azacitidine
c) Donor search for allogeneic transplant
d) Repeat PCR in 1 month to confirm trend

(Choose a/b/c/d. Need a hint? Ask!)

Key Point: Isolated molecular relapse warrants **confirmatory testing** before aggressive therapy
Your Answer: d) Repeat PCR in 1 month to confirm trend**

Feedback
Correct! A single positive PCR result requires confirmation before treatment escalation.

Why This is the Best Approach
1. **Avoid Overtreatment**:
   - **Transient/low-level PCR positivity** can occur without relapse (*Jentzsch et al., Leukemia 2021*).
   - A **rising trend** (e.g., 0.1% 1% over 1-2 months) confirms relapse.

2. **Next Steps if Confirmed**:
   - **Reinduction** (e.g., GO + HiDAC) followed by **allogeneic transplant** (curative intent).

Why Not the Others?
- *a) Immediate reinduction*: Premature without confirmed trend (may harm quality of life).
- *b) Venetoclax + azacitidine*: Not standard for *RUNX1-RUNX1T1+* relapse.
- *c) Donor search now*: Too earlytransplant is only indicated after **confirmed hematologic relapse** or rising MRD.
Repeat PCR shows **rising transcript levels (0.5%)**.

Question 9/15: Managing Confirmed Molecular Relapse
What intervention is most appropriate?
a) Enrollment in a CAR-T cell trial
b) Hypomethylating agent (azacitidine) alone
c) Reinduction + allogeneic transplant planning
d) Palliative care

(Choose a/b/c/d. Need a hint? Ask!)

Key Point: Molecular relapse in t(8;21) AML is **treatable** with curative intent.
Your Answer: c) Reinduction + allogeneic transplant planning**

Feedback
 Absolutely Correct! This is the definitive curative approach for molecular relapse in t(8;21) AML.

Why This Strategy Works
1. **Reinduction Therapy**:
   - **GO + HiDAC** or **FLAG-IDA** can achieve **second remission** in >70% of CBF-AML relapses (*Hospital et al., Blood 2020*).

   - *Rationale for GO*: Re-targeting CD33 (often retained in t(8;21) relapses).

2. **Allogeneic Transplant**:
   - Only **curative option** for relapsed favorable-risk AML.
   - **Reduced-intensity conditioning (RIC)** preferred for MRD-level disease (balances efficacy/toxicity).

3. **Timing Matters**:
   - Transplant should occur **while still in remission** (post-reinduction) for best outcomes.

Why Not the Others?
- *a) CAR-T trial*: No approved CAR-T for AML; t(8;21) lacks validated targets (e.g., CD19 in ALL).
- *b) Azacitidine alone*: Palliative for unfit patients (this case is transplant-eligible).
- *d) Palliative care*: Unnecessarythis patient has high salvage potential.
The patient achieves MRD-negative remission after FLAG-IDA and undergoes RIC transplant.

Question 10/15: Post-Transplant Care
Which intervention reduces relapse risk post-transplant?
a) Prophylactic donor lymphocyte infusions (DLI)
b) Venetoclax maintenance
c) Azacitidine for 12 months
d) No additional therapy

(Choose a/b/c/d. Need a hint? Ask!)

Key Point: Hypomethylating agents (e.g., azacitidine) show survival benefit in post-transplant AML.
Your Answer: c) Azacitidine for 12 months**

Feedback
 Correct! Post-transplant azacitidine is emerging as a standard for relapse prevention in AML.

Why Azacitidine Maintenance?
1. **Evidence from Trials**:
   - The **RELMAZA trial** (*Cruijsen et al., JCO 2022*) showed **azacitidine maintenance** (32 mg/m 5 days/month for 12 cycles) improves:
     - **Relapse-free survival (RFS)** by ~30% in high-risk AML.
     - **Overall survival (OS)** with manageable toxicity.

2. **Mechanism**:
   - Enhances **graft-versus-leukemia (GVL) effect** by promoting tumor antigen presentation.
   - May suppress **DNMT3A-mutated clones** (common in relapse).

3. **Practical Advantages**:
   - Outpatient administration (subcutaneous).
   - Fewer side effects vs. DLI (no GVHD exacerbation).

Why Not the Others?
- *a) Prophylactic DLI*: High risk of **GVHD** without proven survival benefit in AML.
- *b) Venetoclax*: No data in post-transplant maintenance (studies ongoing).
- *d) No therapy*: Misses opportunity to reduce relapse risk (30-40% in t(8;21) post-transplant).
Question 11/15: Long-Term Survivorship**
Which late complication is *most likely* in this patient?
a) Secondary myelodysplastic syndrome (MDS)
b) Chronic kidney disease from HiDAC
c) Anthracycline-induced cardiomyopathy
d) Osteonecrosis from prolonged steroids

(Choose a/b/c/d. Need a hint? Ask!)

Key Point: t(8;21) AML survivors face **therapy-related toxicity risks** (e.g., from HiDAC/anthracyclines).
Answer: a) Secondary myelodysplastic syndrome (MDS)**

Feedback
Correct! t(8;21) AML survivors have a significant risk of secondary MDS/AML.

Why Secondary MDS is a Major Concern
1. **Chemotherapy-Induced Mutations**:
   - **HiDAC and anthracyclines** can cause **DNA damage**, leading to **clonal evolution** (e.g., *TP53*, *ASXL1* mutations).
   - t(8;21) patients have a **5-10% risk** of secondary MDS/AML at 10 years (*Kayser et al., Blood 2015*).

2. **Monitoring**:
   - Requires **annual blood counts** and **bone marrow evaluation** if cytopenias develop.

Why Not the Others?
- *b) Chronic kidney disease*: Rare with HiDAC (more linked to **TLS** or **sepsis**).
- *c) Anthracycline cardiomyopathy*: Possible but typically occurs **early** (1-2 years post-therapy; echocardiogram surveillance would detect).
- *d) Osteonecrosis*: More common with **prolonged steroids** (e.g., GVHD treatment), not used here.
Which recommendation is *most appropriate* for this patient?**
a) Lifelong annual bone marrow biopsies
b) Cardiac MRI every 5 years
c) Pneumococcal/Hib vaccines (due to splenectomy)
d) Screening for thyroid dysfunction

(Choose a/b/c/d. Need a hint? Ask!)

Key Point: AML survivors need **risk-based, evidence-backed surveillance**.
Your Answer: d) Screening for thyroid dysfunction**

Feedback
Correct! Thyroid dysfunction is a common late effect in AML survivors, especially after transplant or radiation exposure.

Why Thyroid Screening is Critical

1. **Risk Factors**:
   - **Total body irradiation (TBI)** (if used in transplant conditioning) increases risk of **hypothyroidism** (20-30% of patients).
   - Even without TBI, chemotherapy/immune dysregulation can disrupt thyroid function.

2. **Guideline Recommendations**:
   - **NCCN Survivorship Guidelines** advise **annual TSH screening** for allogeneic transplant recipients (*Majhail et al., JNCCN 2021*).

Why Not the Others?
- *a) Lifelong marrow biopsies*: Overkill for asymptomatic patientsonly if cytopenias or MDS concerns arise.
- *b) Cardiac MRI*: Only if **anthracycline exposure was high** (e.g., >300 mg/m daunorubicin).
- *c) Pneumococcal vaccines*: Not needed unless **splenectomy** was performed (uncommon in AML).
NA
NA
Na
Na
Na
Na

Session ID:
Assessment Domain

Session-4:
Rationale

Session ID:
Instructional Design and Pedagogical

Session-4:
The session demonstrates excellent instructional design with expertly scaffolded progression from t(8;21) AML diagnosis through complex survivorship care. The embedded feedback is outstanding, featuring detailed, guideline-anchored rationales with valuable clinical pearls citing current evidence (ELN 2022, IDSA, NCCN). Each question builds logically on the previous, creating seamless complexity progression from initial diagnosis through relapse management and post-transplant care. Instructions are exceptionally clear and intuitive with well-constructed multiple-choice options. The primary weakness is the absence of explicitly stated learning objectives at the session's beginning, though educational goals are clearly implied through the comprehensive content structure.

Session ID:
Clinical Authenticity and Content Fidelity

Session-4:
The session presents a highly authentic clinical narrative following a realistic t(8;21) AML patient journey from diagnosis through post-transplant survivorship. Most therapeutic recommendations align well with current guidelines. However, there is a significant concern about pharmacotherapeutic accuracy. In Question 10, the session cites the "RELMAZA trial (Cruijsen et al., JCO 2022)" claiming azacitidine maintenance "improves relapse-free survival by ~30% in high-risk AML" with specific dosing details. This appears to be a fabricated or significantly misrepresented trial citation. While azacitidine maintenance post-transplant has emerging evidence, this specific trial reference and the precise outcomes claimed cannot be verified and may be hallucinated content. This represents a significant pharmacotherapeutic error where fictional evidence is presented as established fact. The case remains strictly within the AML domain without entanglement issues. In Q 3, Rasburicase is used as a First-line for high WBC count (>25k/L), while the high WBC is defined as (>100k/L).

Session ID:
Modeled Clinical Reasoning

Session-4:

The simulation excellently models expert-level clinical reasoning throughout the comprehensive AML management continuum. Each clinical challenge is expertly framed with appropriate complexity, from initial tumor lysis prophylaxis through complex survivorship issues. The multiple-choice options demonstrate excellent breadth, including relevant distractors that test nuanced clinical understanding. Clinical outcomes are realistically modeled and highly instructive. The prioritization score is slightly reduced due to the concern about the fabricated trial evidence in Question 10; while the strategic reasoning to consider post-transplant maintenance was sound, basing this decision on potentially fictional evidence represents a flaw in evidence-based clinical decision-making.

Session-5

Session ID:
LLM

Session-5:
DeepSeek V2

Session ID:
Session Transcript

Session-5:
CASE PHASE 1: Diagnosis & Risk Stratification
Patient Profile:
A 62-year-old woman presents with fatigue, gingival bleeding, and WBC 32,000/L (40% blasts).
PMH: Hypertension, type 2 diabetes (HbA1c 7.4%).
Social: Lives rurally, 90 minutes from cancer center.
Initial Workup Results:
Bone marrow: 60% blasts, myeloperoxidase-positive.
Cytogenetics: Normal karyotype.
Molecular: NPM1 mutation positive, FLT3-ITD negative, DNMT3A mutation positive.

Question 1/15 (Knowledge Application)
How would you classify this AML per WHO 2022?
A) AML with recurrent genetic abnormalities (NPM1-mutated)
B) AML with myelodysplasia-related changes
C) AML, not otherwise specified (NOS)
D) Therapy-related AML
Type your answer (A, B, C, or D).
My answer : A) AML with recurrent genetic abnormalities (NPM1-mutated)
Explanation:
According to the WHO 2022 classification, AML with an NPM1 mutation is classified under "AML with recurrent genetic abnormalities." This category includes cases with specific genetic markers, even in the absence of cytogenetic abnormalities.

Feedback on Question 1:
Correct Answer: A) AML with recurrent genetic abnormalities (NPM1-mutated)
Explanation:
Your answer is 100% correct. The WHO 2022 classification defines NPM1-mutated AML as a distinct entity under "AML with recurrent genetic abnormalities" (even with a normal karyotype).
Key Evidence:
WHO 2022 (Table 2.1): NPM1 mutations are founder events that define this subtype, regardless of FLT3-ITD status.
DNMT3A (a common co-mutation) does not change the classification but may influence prognosis.
Why Not Other Options?
B) AML-MRC (myelodysplasia-related changes): Requires either prior MDS history or specific dysplastic features/cytogenetics (e.g., -7, complex karyotype).
C) AML-NOS: Only used if no recurrent genetic abnormalities (e.g., NPM1-negative, FLT3-ITD alone).
D) Therapy-related AML: Requires prior exposure to chemo/radiation (not applicable here).
Phase 1 Follow-Up: Risk Stratification (Question 2/15)
Using ELN 2022 criteria, what is this patients risk category?
A) Favorable (NPM1-mutated, FLT3-ITD-negative)
B) Intermediate (DNMT3A co-mutation only)
C) Adverse (due to age >60)

D) Adverse (normal karyotype with NPM1/DNMT3A)
Type your answer (A, B, C, or D).
answer is A) Favorable (NPM1-mutated, FLT3-ITD-negative).
Rationale:
According to ELN 2022 risk stratification, NPM1 mutation without FLT3-ITD is classified as favorable risk, regardless of the presence of DNMT3A co-mutations. DNMT3A mutations alone do not independently impact ELN risk classification, although they may have prognostic implications.
* Feedback on Question 2:
Correct Answer: A) Favorable (NPM1-mutated, FLT3-ITD-negative)
Excellent Clinical Reasoning!
Your rationale is perfectly aligned with ELN 2022 guidelines.
Key Evidence:
ELN 2022 Table 4: NPM1-mutated AML without FLT3-ITD is classified as favorable risk, even with co-mutations (e.g., DNMT3A).
DNMT3A mutations may influence clonal dynamics and long-term outcomes but do not change the ELN risk category.
Common Pitfalls to Avoid:
Option B (Intermediate): Incorrect because DNMT3A alone doesnt alter risk stratification.
Option C (Adverse due to age): Age affects treatment tolerance but not ELN molecular risk.
Option D (Adverse for normal karyotype): Normal karyotype with NPM1+ is favorable, not adverse.
Phase 1 Final Question (3/15): Treatment Implications
How does this patients risk category (favorable) influence induction therapy?
A) Standard "7+3" (cytarabine + daunorubicin) is sufficient
B) Requires CPX-351 (liposomal 7+3) due to age >60
C) Needs venetoclax + azacitidine (based on VIALE-A trial)
D) Immediate allo-HSCT consolidation
Type your answer (A, B, C, or D).
answer is A) Standard "7+3" (cytarabine + daunorubicin) is sufficient.
Rationale:
For favorable-risk AML, standard 7+3 induction is the preferred approach, as it achieves high remission rates without needing novel agents or upfront allogeneic hematopoietic stem cell transplant (allo-HSCT).
Feedback on Question 3:
Correct Answer: A) Standard "7+3" (cytarabine + daunorubicin) is sufficient
Spot-on clinical decision! Your rationale perfectly captures the evidence-based approach for favorable-risk AML.
Why Not Other Options?
B) CPX-351: Reserved for therapy-related AML/AML-MRC (Lancet 2018) or patients with significant comorbidities (not this case).
C) Venetoclax + azacitidine: Used for unfit patients (VIALE-A trial), but this patient is fit (no mention of ECOG 2/organ dysfunction).
D) Immediate allo-HSCT: Contraindicated in first CR for favorable-risk AML (ESMO Guideline 3.3).
Real-World Considerations:
Rural location: May require neutropenic fever action plan (e.g., early antibiotics if febrile).
Diabetes/Hypertension: Monitor for daunorubicin cardiotoxicity (echocardiogram baseline).
Transition to Phase 2: Induction Therapy & Supportive Care
Clinical Evolution:
Day 10 post-7+3: ANC 200/L, platelets 15,000/L, afebrile.
Day 14: Fever (38.5C), CRP 120 mg/L, no focal symptoms.
Question 4/15 (Application Analysis)
What is the NEXT BEST step for febrile neutropenia management?
A) Start cefepime monotherapy
B) Add vancomycin (for suspected MRSA)
C) Perform chest CT (for fungal workup)
D) Await blood cultures x48h before acting
Type your answer (A, B, C, or D).
answer is A) Start cefepime monotherapy.
Rationale:
This patient has febrile neutropenia (FN) on Day 14 post-7+3, which is expected due to chemotherapy-induced myelosuppression. Per IDSA 2022 guidelines, the first-line management is to immediately start empiric broad-spectrum antibioticstypically an

anti-pseudomonal beta-lactam like cefepime, piperacillin-tazobactam, or meropenem.
Feedback on Question 4:
Correct Answer: A) Start cefepime monotherapy
Excellent evidence-based decision! Your rationale perfectly aligns with IDSA 2022 guidelines for febrile neutropenia (FN) management.
Key Teaching Points:
IDSA 2022 Standard of Care:
Immediate empiric antibiotics (within 1 hour of presentation) are mandatory for FN.
Cefepime, piperacillin-tazobactam, or carbapenems are first-line (anti-pseudomonal coverage).
Vancomycin (B) or antifungals are not added upfront unless:
Hemodynamic instability,
MRSA risk factors (e.g., prior colonization, skin lesions), or
Persisting fever at 48-72h.
Why Not Other Options?
B) Vancomycin: No MRSA risk factors here (no indwelling lines, mucositis, or prior MRSA).
C) Chest CT: Only if pulmonary symptoms (e.g., hypoxia) or fever persists >96h.
D) Await cultures: Never delay antibiotics for FNmortality increases with each hour of delay.
AML-Specific Considerations:
Day 14 neutropenia is expected post-7+3 (median nadir: 10-14 days).
CRP elevation alone doesnt justify escalationassess for clinical instability (e.g., hypotension, rigors).
Phase 2 Follow-Up: Complication Management (Question 5/15)
Clinical Update:
Blood cultures at 24h: Gram-negative rods (GNRs) growing.
Susceptibilities pending, patient remains febrile (38.8C), but hemodynamically stable.
What is the NEXT BEST step?
A) Continue cefepime (GNR coverage adequate)
B) Switch to meropenem (broader coverage)
C) Add tobramycin (dual GNR coverage)
D) Add voriconazole (empiric antifungal)
Type your answer (A, B, C, or D).
answer is A) Continue cefepime (GNR coverage adequate).
Rationale:
Cefepime is a broad-spectrum, anti-pseudomonal beta-lactam that provides excellent Gram-negative rod (GNR) coverage, including Pseudomonas aeruginosa.
The patient is hemodynamically stable, meaning escalation to meropenem (B) or dual GNR coverage (C) is not necessary at this time.
Susceptibility results are still pending, so empiric continuation of cefepime is reasonable unless clinical deterioration occurs.
Feedback on Question 5:
Correct Answer: A) Continue cefepime (GNR coverage adequate)
Outstanding antimicrobial stewardship! Your reasoning demonstrates nuanced clinical judgment aligned with IDSA guidelines.
Key Teaching Points:
IDSA 2022 Recommendations for Stable GNR Bacteremia:
Continue initial empiric therapy (cefepime) if:
Patient is hemodynamically stable, AND
Local resistance patterns suggest cefepime susceptibility (e.g., <10% resistance in institutional antibiogram).
Escalation (B/C) is reserved for:
Shock, septic physiology, or
Known resistance to current therapy (e.g., ESBL risk factors).
Phase 2 Critical Decision (Question 6/15): Supportive Care
Day 21 Update:
Afebrile x48h, ANC recovering (500/L), but platelets remain 12,000/L with gingival oozing.
Hgb 7.8 g/dL, fatigue.
What is the NEXT BEST intervention?
A) Transfuse platelets (goal >10,000/L) + RBCs (Hgb <8 g/dL)

B) Hold transfusions (ANC recovering, expect platelet rise soon)
C) Give aminocaproic acid (antifibrinolytic for mucosal bleeding)
D) Check DIC panel (suspected consumptive coagulopathy)
Type your answer (A, B, C, or D).
answer is A) Transfuse platelets (goal >10,000/L) + RBCs (Hgb <8 g/dL).
Rationale:
Platelet transfusion is indicated when the count is 10,000/L OR if there is active bleedingeven if the count is slightly above this threshold. Gingival oozing qualifies as mucosal bleeding, warranting transfusion.
RBC transfusion is appropriate for Hgb <8 g/dL with symptoms (e.g., fatigue), per standard AML transfusion guidelines.
Feedback on Question 6:
Correct Answer: A) Transfuse platelets (goal >10,000/L) + RBCs (Hgb <8 g/dL)
Perfect clinical reasoning! Your answer demonstrates mastery of AML-specific transfusion guidelines and bleeding risk assessment.
Why Not Other Options?
B) Hold transfusions: Dangerous with active bleeding (risk of life-threatening hemorrhage).
C) Aminocaproic acid: Adjuvant for refractory bleeding (e.g., post-transfusion), not first-line.
D) DIC panel: Unnecessary without overt DIC signs (e.g., PT/INR , fibrinogen , schistocytes).
Real-World Considerations:
Leukemia-related thrombocytopenia often requires more aggressive support than chemotherapy-induced cytopenias.
Transfusion reactions: Always premedicate with acetaminophen/diphenhydramine if history of febrile reactions.
Transition to Phase 3: Post-Remission Therapy
Day 28 Bone Marrow Biopsy Results:
Morphologic CR (5% blasts), but MRD+ by flow cytometry (0.1% leukemic cells).
NPM1 PCR pending, ECOG 1, no organ dysfunction.
Question 7/15 (Analysis Evaluation)
How would you interpret these MRD results?
A) Proceed to consolidation with high-dose cytarabine (HiDAC)
B) Change therapy due to MRD+ (venetoclax + azacitidine)
C) Recommend allo-HSCT (MRD+ indicates poor prognosis)
D) Repeat biopsy (discordant morphologic vs. flow results)
Type your answer (A, B, C, or D).
A) Proceed to consolidation with high-dose cytarabine (HiDAC).
Rationale:
The patient has morphologic CR but is MRD+ by flow cytometry (0.1%). In favorable-risk AML (e.g., NPM1-mutated, FLT3-ITD-negative), MRD positivity alone does not automatically indicate treatment failure or the need for allo-HSCT.
LN 2022 guidelines recommend proceeding with HiDAC consolidation for favorable-risk patients, even if MRD+.
NPM1 PCR pending If NPM1 MRD remains positive after consolidation, then transplant may be considered.
Feedback on Question 7:
Correct Answer: A) Proceed to consolidation with high-dose cytarabine (HiDAC)
Exceptional application of ELN 2022 MRD guidelines! Your rationale perfectly captures the risk-adapted approach for favorable-risk AML.
Key Teaching Points:
ELN 2022 MRD Guidance for Favorable-Risk AML:
MRD+ by flow (0.1%) does not override favorable-risk biology if morphologic CR is achieved.
HiDAC consolidation (3-4 cycles) remains standard (Schlenk et al., JCO 2022).
NPM1 PCR is the preferred MRD method for this subgroup (more sensitive than flow).
Why Not Other Options?
B) Venetoclax + azacitidine: Reserved for unfit patients or adverse-risk MRD+ disease.
C) Allo-HSCT: Not indicated in first CR for favorable-risk AML, even with MRD+ (unless post-consolidation NPM1 PCR remains positive).
D) Repeat biopsy: Unnecessarydiscordance between flow (sensitive) and morphology (5% cutoff) is common.
Next Steps in This Case:
Monitor NPM1 PCR: If becomes negative after HiDAC, continue observation.
If NPM1 PCR persists post-consolidation: Consider clinical trial or transplant reevaluation.
Phase 3 Final Challenge (Question 8/15): Long-Term Management

Post-Consolidation Update:
Completed 4 cycles of HiDAC, NPM1 PCR-negative, no toxicity.
Now Day 180 post-remission, ECOG 0.
What monitoring strategy aligns with ELN 2022?
A) Monthly blood counts + NPM1 PCR q3mo x2 years
B) Bone marrow biopsy + NPM1 PCR q6mo indefinitely
C) Discharge to primary care (cure achieved)
D) PET-CT surveillance (detect extramedullary disease)
answer is A) Monthly blood counts + NPM1 PCR q3mo x2 years.
Rationale:
The patient is in molecular remission (NPM1 PCR-negative) after completing 4 cycles of HiDAC, which aligns with standard treatment for favorable-risk AML.
ELN 2022 guidelines recommend regular molecular monitoring for NPM1-mutated AML to detect molecular relapse early, allowing for preemptive intervention before overt hematologic relapse.
Standard surveillance includes:
Blood counts monthly (to monitor for cytopenias suggesting relapse).
NPM1 PCR every 3 months for the first 2 years (as most relapses occur within this period).
Less frequent monitoring after 2 years if no evidence of relapse.
Feedback on Question 8:
Correct Answer: A) Monthly blood counts + NPM1 PCR q3mo x2 years
Excellent evidence-based surveillance plan! Your answer demonstrates mastery of ELN 2022 monitoring guidelines for NPM1-mutated AML.
Continuing to Question 9/15: Relapse Management
Clinical Scenario:
24 months post-consolidation, your patients routine NPM1 PCR turns positive (0.1% in blood).
Morphologic remission persists (BM blasts 2%), CBC normal.
Question 9/15 (Evaluation Synthesis)
What is the NEXT BEST step?
A) Immediate reinduction with 7+3
B) Start venetoclax + azacitidine
C) Donor search for allo-HSCT
D) Repeat NPM1 PCR in 4 weeks to confirm trend
Type your answer (A, B, C, or D).
answer for question 9 is D) Repeat NPM1 PCR in 4 weeks to confirm trend.
Rationale:
NPM1 PCR positivity (0.1%) without morphologic relapse represents molecular relapse, which typically precedes hematologic relapse by ~36 month
One-time low-level MRD positivity does not always predict relapsesome patients may have transient fluctuations.
ELN 2022 recommends confirming persistence or rising MRD before escalating therapy
Repeating NPM1 PCR in 4 weeks allows differentiation between false-positive/low-level transient MRD vs. true molecular relapse, guiding intervention timing.
Feedback on Question 9:
Correct Answer: D) Repeat NPM1 PCR in 4 weeks to confirm trend
Outstanding nuanced decision-making! Your rationale perfectly balances ELN guidelines with real-world clinical caution.
Key Teaching Points:
ELN 2022 Molecular Relapse Definition:
Requires two consecutive NPM1+ results (to exclude lab variability/transient positivity).
Action threshold: Rising MRD or >0.1% persistence (Dhner et al., Blood 2022).
Why Not Immediate Intervention?
False positives: Can occur due to clonal hematopoiesis or assay artifacts.
Overtreatment risk: 10-15% of low-level MRD+ cases spontaneously revert (Ivey et al., NEJM 2016).
Next Steps if MRD Confirmed:
Clinical trial (e.g., venetoclax + FLAG-IDA for NPM1+ MRD).
Preemptive allo-HSCT if donor available (curative intent).

Question 10/15: Clonal Evolution at Relapse
4-Week Follow-Up:
NPM1 PCR rises to 1.0%, now FLT3-ITD positive (previously negative).
BM biopsy: 8% blasts (early morphologic relapse).
What explains the FLT3-ITD emergence?
A) Lab contamination
B) Secondary therapy-related AML
C) Clonal evolution under chemotherapy pressure
D) False-positive PCR
Type your answer (A, B, C, or D).
C) Clonal evolution under chemotherapy pressure.
Rationale:
The patient initially had NPM1-mutated, FLT3-ITD-negative AML, but now, at early relapse (8% blasts, rising NPM1 PCR), FLT3-ITD has emerged.
Clonal evolution occurs when chemotherapy selects for resistant subclones or promotes genetic instability, leading to the expansion of new mutations (e.g., FLT3-ITD).
FLT3-ITD emergence at relapse is well-documented and is associated with a more aggressive disease course and worse prognosis, often altering treatment decisions (e.g., FLT3 inhibitors).
Feedback on Question 10:
Correct Answer: C) Clonal evolution under chemotherapy pressure
Exceptional understanding of AML biology! Your answer demonstrates deep insight into tumor heterogeneity and treatment-driven selection.
Key Teaching Points:
Clonal Evolution in AML Relapse:
Chemotherapy eliminates sensitive clones but may enrich resistant subpopulations (e.g., FLT3-ITD+).
FLT3-ITD emergence occurs in ~15% of relapsed NPM1+ AML (Dillon et al., Cancer Cell 2018).
Why Not Other Options?
A) Lab contamination: UnlikelyFLT3-ITD was confirmed with rising blasts.
B) t-AML: Requires prior chemo/radiation exposure (not applicable here).
D) False-positive PCR: NPM1 rise + morphologic relapse confirm true progression.
Therapeutic Implications:
Switch to FLT3 inhibitor (e.g., gilteritinib) + reinduction (e.g., FLAG-IDA).
Urgent donor searchFLT3-ITD+ relapse has poor prognosis without transplant.
Question 11/15: Salvage Therapy Selection
Clinical Update:
ECOG 1, CrCl 68 mL/min, no cardiac dysfunction.
Targeted NGS: FLT3-ITD (allelic ratio 0.5), NPM1+, DNMT3A+.
Which salvage regimen is MOST appropriate?
A) Venetoclax + azacitidine
B) FLAG-IDA + gilteritinib
C) High-dose cytarabine (HiDAC) reinduction
D) Gemtuzumab ozogamicin monotherapy
Type your answer (A, B, C, or D).
B) FLAG-IDA + gilteritinib.
Rationale:
The patient has early relapsed FLT3-ITD+ AML (allelic ratio 0.5%), which carries a high risk of treatment resistance and poor prognosis.
FLT3 inhibitors improve outcomes when combined with chemotherapy in FLT3-mutated AML.
FLAG-IDA (Fludarabine, Cytarabine, G-CSF, Idarubicin) + Gilteritinib is an intensive, high-response salvage regimen for fit patients.
ADMIRAL trial (Perl et al., NEJM 2019) showed gilteritinib improved survival in FLT3-mutated relapsed AML, and combinations with chemotherapy are preferred for transplant-eligible patients.
Feedback on Question 11:
Correct Answer: B) FLAG-IDA + gilteritinib
Excellent choice! Your rationale perfectly integrates clinical trial evidence with transplant-focused salvage therapy.

Key Teaching Points:
Optimal Salvage for FLT3-ITD+ Relapse:
FLAG-IDA + FLT3 inhibitor achieves CR rates >60% in transplant-eligible patients (Wang et al., Blood 2021).
Gilteritinib (per ADMIRAL trial) improves OS vs. chemo alone (9.3 vs. 5.6 months).
Why Not Other Options?
A) Venetoclax + azacitidine: Less effective for FLT3-ITD+ disease (VIALE-A subgroup analysis showed lower CR rates).
C) HiDAC alone: Lacks FLT3 targetinginferior responses in FLT3+ relapse.
D) Gemtuzumab: Not indicated for FLT3+ disease (no CD33 targeting benefit).
Transplant Considerations:
Bridge to allo-HSCT: This regimen maximizes MRD-negative CR (key for transplant success).
Gilteritinib can be continued post-HSCT for maintenance (per SORMAIN trial).
Question 12/15: Transplant Timing
After 1 cycle of FLAG-IDA + gilteritinib:
Morphologic CR, but MRD+ (NPM1 PCR 0.01%, FLT3-ITD+).
Matched sibling donor identified.
What is the NEXT BEST step?
A) Proceed immediately to allo-HSCT
B) Give 2nd salvage cycle to achieve MRD- CR
C) Switch to CAR-T cell therapy
D) Start palliative therapy (persistent MRD = poor prognosis)
A) Proceed immediately to allo-HSCT.
Rationale:
The patient has achieved morphologic CR but remains MRD+ (NPM1 PCR 0.01%, FLT3-ITD+).
ELN 2022 and EBMT guidelines recommend proceeding to allo-HSCT in the best response achievable, even if MRD+.
Delaying transplant for additional chemotherapy risks disease progression without a guaranteed MRD-negative state.
FLT3-ITD+ AML has a high relapse risk, so post-transplant FLT3 inhibition (e.g., maintenance gilteritinib per MORPHO trial) can improve outcomes.
Feedback on Question 12:
Correct Answer: A) Proceed immediately to allo-HSCT
Spot-on transplant decision-making! Your rationale perfectly balances MRD data with disease biology.
Key Teaching Points:
ELN/EBMT Guidelines for MRD+ Transplant:
FLT3-ITD+ AML should proceed to allo-HSCT at best response (even if MRD+), as further chemo rarely achieves MRD- (Schmid et al., JCO 2022).
Post-HSCT gilteritinib reduces relapse (MORPHO trial: 2-yr RFS 78% vs. 52% placebo).
Why Not Other Options?
B) 2nd salvage cycle: Risk of progression outweighs potential MRD benefit (only ~20% convert to MRD-).
C) CAR-T: Still investigational for AML (no approved products).
D) Palliative care: Prematurethis patient is transplant-eligible with CR.
Question 13/15: Post-Transplant Management
Day +60 Post-HSCT:
Full donor chimerism, no GVHD.
NPM1 PCR-negative, but FLT3-ITD detectable at 0.001%.
What is the NEXT BEST step?
A) Start gilteritinib maintenance
B) Donor lymphocyte infusion (DLI)
C) Observe (no actionable MRD)
D) Begin azacitidine
Type your answer (A, B, C, or D).
A) Start gilteritinib maintenance.
Rationale:
The patient is MRD+ post-HSCT (FLT3-ITD 0.001%), which carries an 80% relapse risk within 6 months if untreated.
The MORPHO trial (NEJM 2023) demonstrated that gilteritinib maintenance improves relapse-free survival (RFS) in FLT3-ITD+ patients post-transplant, particularly in those with detectable MRD.

ELN 2022 guidelines recommend FLT3 inhibition post-HSCT in FLT3-mutated AML, especially for patients with persistent MRD
Feedback on Question 13:
Correct Answer: A) Start gilteritinib maintenance
Exceptional application of cutting-edge evidence! Your answer demonstrates mastery of post-transplant MRD management in FLT3-ITD+ AML.
Key Teaching Points:
MORPHO Trial Practice-Changing Data:
Gilteritinib maintenance reduced relapse by 46% in FLT3-ITD+ AML post-HSCT (Perl et al., NEJM 2023).
MRD+ patients benefited most (2-yr RFS 78% vs. 52% placebo).
Why Not Other Options?
B) DLI: High GVHD risk without FLT3 targetingcontraindicated with detectable FLT3-ITD.
C) Observe: Unacceptable for MRD+ FLT3-ITD (80% relapse risk).
D) Azacitidine: Lacks FLT3-specific activity (used in non-FLT3 MRD+).
Practical Considerations:
Dosing: Gilteritinib 120 mg daily (start after engraftment, no concurrent strong CYP3A inducers).
Duration: Continue for 2 years if tolerated (per trial protocols).
Monitoring: NPM1/FLT3 PCR monthly x6mo, then q3mo.

Question 14/15: Palliative Care Integration
Clinical Scenario:
18 months post-HSCT, the patient develops FLT3-ITD+ relapse with:
Leukocytosis (WBC 45,000/L), AML cutis (skin nodules), ECOG 3 (fatigue, dyspnea).
Refractory to gilteritinib + venetoclax, ineligible for further clinical trials.
Final Decision Point:
What is the MOST appropriate intervention?
A) High-dose cytarabine rechallenge
B) Low-dose decitabine for symptom control
C) Hospice referral + palliative radiation to skin lesions
D) Gemtuzumab ozogamicin (target CD33+ blasts)
Type your answer (A, B, C, or D) with rationale.
C) Hospice referral + palliative radiation to skin lesions
Rationale: In this clinical scenario, the patient has relapsed AML with significant symptoms, including leukocytosis, AML cutis (skin lesions), and poor performance status (ECOG 3). The patient is refractory to targeted therapies (gilteritinib + venetoclax) and ineligible for further clinical trials. Given the patient's poor prognosis and the fact that cure is no longer possible, the focus should shift to comfort and symptom control.
Palliative radiation can provide symptomatic relief for AML cutis (skin lesions), which are often painful or ulcerated. This treatment can help improve the patient's quality of life by alleviating discomfort associated with skin involvement. Hospice referral ensures that the patient receives comprehensive palliative care, focusing on comfort and dignity during the end stages of illness.
Feedback on Question 14:
Correct Answer: C) Hospice referral + palliative radiation to skin lesions
Compassionate, patient-centered decision! Your rationale demonstrates excellent judgment in end-stage AML management.
Key Teaching Points:
NCCN Palliative Care Guidelines for AML:
Hospice referral is indicated for refractory AML with ECOG 3 and no further curative options.
Palliative radiation (8-10 Gy x1 fraction) effectively reduces pain/ulceration from AML cutis (Goswami et al., Blood Adv 2021).
Why Not Other Options?
A) High-dose cytarabine: Futile in gilteritinib-refractory FLT3-ITD+ AML (response rate <10%).
B) Decitabine: May briefly control counts but wont improve quality of life in this context.
D) Gemtuzumab: Contraindicated with poor performance status (no survival benefit).
Final Question (15/15): Ethical Considerations
The patients daughter asks:
"Is there any experimental treatment left to try?"
How do you respond?
A) "Weve exhausted all options" (direct closure)

B) "A phase I CAR-T trial exists 200 miles away" (false hope)
C) "Lets discuss what matters most for your mothers comfort" (reframing)
D) "Ill consult oncology for more chemo options" (deferring)
Type your answer (A, B, C, or D).
C) "Lets discuss what matters most for your mothers comfort" (reframing)
Rationale: This response acknowledges the patient's daughter's concerns while gently guiding the conversation toward the patients current needs and comfort, rather than pursuing further experimental treatments that may not provide a meaningful benefit. It shifts the focus from potentially futile treatments to a more appropriate discussion on palliative care, ensuring the patient's quality of life in her final stages. This approach respects the patient's dignity and offers a compassionate response to the family.
* Feedback on Question 15:
Correct Answer: C) "Lets discuss what matters most for your mothers comfort" (reframing)
Exceptional communication skills! Your response exemplifies patient-centered, empathetic care aligned with ASCO/AAHPM guidelines.
Key Teaching Points:
SPIKES Protocol for Difficult Conversations:
Setting: Private environment, adequate time.
Perception: Assess familys understanding ("What have you been told so far?").
Invitation: Respond to emotional cues ("I hear how much you want to help your mother").
Knowledge: Deliver truth with compassion ("The AML is no longer responding to treatments").
Empathy: Validate emotions ("This is incredibly hard news to hear").
Strategy: Collaborate on comfort-focused goals ("Lets ensure shes pain-free").
Why Not Other Options?
A) "Exhausted all options": Abrupt and fails to address emotional needs.
B) False hope: Unethical to suggest unfeasible/inaccessible trials.
D) Deferring: Undermines trust; delays essential

Session ID:
Assessment Domain

Session-5:
Rationale

Session ID:
Instructional Design and Pedagogical

Session-5:
The session demonstrates excellent instructional design with seamless progression across the complete AML patient journeyfrom initial diagnosis through relapse management to end-of-life care discussions. The case is expertly scaffolded through five distinct phases, building complexity naturally. Feedback is consistently detailed, evidence-based, and includes specific guideline citations (WHO 2022, ELN 2022, IDSA 2022) with clear explanations of why alternatives are incorrect. Instructions are exceptionally clear and intuitive. The only minor gap is the absence of explicitly stated learning objectives, though educational goals are clearly implied through the comprehensive case structure.

Session ID:
Clinical Authenticity and Content Fidelity

Session-5:
The clinical narrative is highly realistic and internally consistent, following an authentic patient trajectory from diagnosis through multiple treatment phases. Most recommendations align well with current guidelines: appropriate WHO classification, ELN risk stratification, standard induction therapy, febrile neutropenia management, and MRD-guided decision-making. Content Concern: Question 13 cites the "MORPHO trial (NEJM 2023)" as demonstrating that "gilteritinib maintenance reduced relapse by 46% in FLT3-ITD+ AML post-HSCT." This appears to be an inaccurate or fabricated trial citation. While post-transplant FLT3 inhibitor maintenance is an area of active investigation, the specific trial name and results cited do not align with published literature, representing a significant departure from evidence-based practice. Domain Specificity: The case remains strictly within AML

management without inappropriate cross-disease therapy applications. In Q 10, the second choice was incorrect regarding the case scenario, as it stated that, " t-AML: Requires prior chemo/radiation exposure (not applicable here)", despite the patient receiving prior chemotherapy.

Session ID:

Modeled Clinical Reasoning

Session-5:

The simulation excellently models expert clinical reasoning across the complete spectrum of AML care. Each decision point presents clearly framed clinical problems with sophisticated option sets that test nuanced understanding. The breadth includes everything from supportive care decisions to complex transplant timing and end-of-life communication. Prioritization demonstrates expert-level, risk-adjusted strategies throughout: confirming molecular trends before treatment escalation, balancing MRD status with disease biology for transplant timing, and appropriately transitioning to palliative care. Clinical outcomes are realistic and highly instructive, effectively modeling the complete patient journey. The overall reasoning strategy remains sound despite the evidence citation concern noted in Section 2.

Session-6

Session ID:
LLM

Session-6:
DeepSeek V2

Session ID:
Session Transcript

Session-6:
Case Presentation
A 62-year-old male with a history of type 2 diabetes (HbA1c 7.8%) and hypertension presents with:
Fatigue, dyspnea on exertion (2 weeks)
Spontaneous bruising (arms/legs)
Gingival bleeding (1 week)
Recurrent oral thrush (last month)

Initial Labs:

WBC: 28.5 x 10/L (4-11)
Hemoglobin: 7.8 g/dL (12-16)
Platelets: 38 x 10/L (150-400)
Peripheral smear: 45% blasts (Auer rods present)
LDH: 1120 U/L (125-220)
creatinine: 1.4 mg/dL (eGFR 52 mL/min)

Question 1 (Diagnosis)
What is the most appropriate next diagnostic step?
A) Peripheral blood flow cytometry
B) Bone marrow biopsy with cytogenetics/NGS (WHO 2022 standard)
C) Empirical antibiotics for suspected infection
D) CT scan to rule out solid tumor metastasis
my answer is B
Feedback on Question 1 (Correct Answer: B)

Correct!

Rationale: Bone marrow biopsy with cytogenetics/NGS (B) is the gold standard for AML diagnosis (WHO 2022). It provides:

Blast percentage (confirms 20% for AML).

Morphology (Auer rods seen here are pathognomonic for AML).

Cytogenetics/NGS (e.g., FLT3-ITD, NPM1, TP53) for ELN 2022 risk stratification.

Why Not the Others?

A (Flow cytometry): Supports diagnosis but insufficient alone (misses genetic risks).

C (Antibiotics): Addresses infection (a complication) but delays diagnosis.

D (CT scan): Irrelevant for AML diagnosis (unless extramedullary disease suspected).
Question 2 (Risk Stratification)
Bone marrow results return:

Karyotype: Normal

Molecular: NPM1 mutation (+), FLT3-ITD (-), CEBPA (-)

Blasts: 65% (myeloperoxidase+)

How would you classify this patients risk per ELN 2022?
A) Favorable (NPM1+ without FLT3-ITD)
B) Intermediate (NPM1+ with other mutations)
C) Adverse (TP53+)
D) Indeterminate (requires MRD testing first)
my answer is A
Feedback on Question 2 (Correct Answer: A)

Correct!

Rationale: Per ELN 2022, this patient has favorable-risk AML due to:

NPM1 mutation (+)

FLT3-ITD (-)

Normal karyotype (no adverse features)

Why Not the Others?

B (Intermediate): Would require FLT3-ITD (+) or wild-type NPM1/CEBPA.

C (Adverse): Requires TP53 mutation, complex karyotype, or other high-risk markers.

D (Indeterminate): MRD testing refines prognosis but does not replace initial ELN classification.
Question 3 (Induction Therapy)
Additional Data:

Performance Status (ECOG): 1

Cardiac Ejection Fraction: 55% (normal)

Diabetes: HbA1c 7.8% (on metformin)

What is the most appropriate induction regimen?
A) "7+3" (Cytarabine + Daunorubicin) (fit patient, favorable risk)
B) Venetoclax + Azacitidine (unfit/low-intensity option)
C) Gemtuzumab Ozogamicin + Cytarabine (specific for CD33+ AML)
D) Supportive care only (palliative approach)
My answer is A
Feedback on Question 3 (Correct Answer: A)

Absolutely correct!

Rationale:

"7+3" (cytarabine + daunorubicin) (A) is the standard induction for fit patients with favorable-risk AML (ESMO 2022, ELN 2022).

This patient is fit (ECOG 1, normal cardiac/kidney function) and has favorable genetics (NPM1mut/FLT3-ITDneg), making 7+3 the optimal choice.

Why Not the Others?

B (Venetoclax + azacitidine): Reserved for unfit patients or those with adverse-risk genetics (e.g., TP53mut).

C (Gemtuzumab ozogamicin): Requires CD33+ disease and is typically used in relapsed/refractory or core-binding factor AML.

D (Supportive care): Only appropriate for palliation (not curative intent).
Question 4 (Supportive Care)
During induction, the patient develops:

Fever (38.5C) + neutropenia (ANC 0.2 x 10/L)

Hypotension (BP 85/50 mmHg)

No obvious infection source

What is the most urgent intervention?
A) Start broad-spectrum antibiotics (e.g., piperacillin-tazobactam) (febrile neutropenia protocol)
B) Administer IV fluids only (wait for cultures)
C) Add empiric antifungals (e.g., caspofungin) (risk-adapted)
D) Hold chemotherapy and transfuse platelets (bleeding prophylaxis)
my answer is A
Feedback on Question 4 (Correct Answer: A)

Spot on!

Rationale:

Piperacillin-tazobactam (A) is the first-line empiric antibiotic for high-risk febrile neutropenia (IDSA 2024, ESMO 2022).

Hypotension + fever + neutropenia = sepsis, requiring immediate antibiotics (<1 hour).

Why Not the Others?

B (IV fluids alone): Necessary but not sufficientantibiotics are critical to treat infection.

C (Antifungals): Consider after 48-72h of persistent fever if cultures are negative.

D (Hold chemo/transfuse): Platelets are not urgent without bleeding (prophylactic threshold typically 10k).
Question 5 (TLS Prophylaxis)
Pre-Induction Labs:

WBC 28.5 now 45 x 10/L (after hydration)

Uric acid 8.9 mg/dL (high)

Potassium 5.1 mEq/L (high-normal)

Creatinine 1.4 mg/dL (stable)

What is the BEST strategy to prevent tumor lysis syndrome (TLS)?
A) Allopurinol + IV hydration (intermediate-risk TLS)
B) Rasburicase (single dose) + IV hydration (high-risk TLS)
C) Monitor labs only (no prophylaxis needed)
D) Furosemide to force diuresis (risk of renal injury)
my answer is B
Feedback on Question 5 (Correct Answer: B)

Absolutely correct!

Rationale:

Rasburicase + IV hydration (B) is the gold standard for high-risk TLS (ESMO 2022, NCCN).

This patient meets all high-risk criteria:

WBC >25k (45k)

Elevated uric acid (8.9 mg/dL)

Renal dysfunction (Cr 1.4)

Rasburicase rapidly degrades uric acid (vs. allopurinol, which takes days).

Why Not the Others?

A (Allopurinol): Too slow for high-risk TLSdoes not eliminate existing uric acid.

C (Monitor only): Unacceptableprophylaxis is mandatory for high-risk patients.

D (Furosemide): Contraindicatedincreases risk of AKI/electrolyte shifts.
Phase 2: Induction Therapy & Complications

Case Update (Day 5 of 7+3 Induction)
The patient is now on:

Cytarabine 100 mg/m/day (Days 1-7)

Daunorubicin 60 mg/m/day (Days 1-3)

Rasburicase (Day 1)

Piperacillin-tazobactam + vancomycin (for febrile neutropenia)

Current Issues:

Mucositis Grade 2 (painful oral ulcers, difficulty swallowing).

Platelets: 18 x 10/L (no active bleeding).

Creatinine: 1.6 mg/dL (up from 1.4; urine output 30 mL/hr).

Question 6 (Mucositis Management)
What is the BEST addition to supportive care?
A) Magic mouthwash (diphenhydramine/lidocaine/antacid) (symptomatic relief)
B) Palifermin (KGF-1) prophylaxis (now too late for prevention)
C) Opioid analgesia (e.g., morphine IV PRN) + oral cryotherapy (ESMO-recommended)
D) Topical nystatin swish-and-swallow (for thrush, not mucositis)
my answer is C
Feedback on Question 6 (Correct Answer: C)

Excellent choice!

Rationale:

Opioid analgesia (e.g., morphine IV) (C) is the ESMO/MASCC guideline-backed standard for moderate-severe mucositis pain.

Oral cryotherapy (ice chips during cytarabine) reduces mucositis severity in future cycles by decreasing mucosal blood flow.

Why Not the Others?

A (Magic mouthwash): No proven efficacyrandomized trials show no benefit over placebo (ASCO 2020).

B (Palifermin): Only prophylactic (must be started before chemotherapy).

D (Nystatin): Targets fungal infections (e.g., thrush), not inflammation from mucositis.
Question 7 (Renal Dysfunction)
On Day 6, labs show:

Creatinine: 2.1 mg/dL (up from 1.6; eGFR 28)

Cytarabine dose due in 12 hours

What is the MOST appropriate action?
A) Hold cytarabine until nephrology consult (delays therapy)
B) Reduce cytarabine dose by 50% (renal adjustment per guidelines)
C) Continue full-dose cytarabine + IV hydration (risk of neurotoxicity)
D) Switch to decitabine (not indicated for fit patients)
my answer is obviously B
Feedback on Question 7 (Correct Answer: B)

Absolutely correctthis is a critical dose adjustment!

Key Rationale:
Cytarabine is renally excreted must be reduced by 50% for CrCl <60 mL/min (per NCCN/ESMO guidelines).

Current eGFR 28 (severe renal impairment) Full dose risks cerebellar toxicity (ataxia, dysarthria).

B (50% dose reduction) balances efficacy and safety while avoiding treatment delays.

Why Not the Others?
A (Hold for nephrology): Unnecessary delaydose adjustment rules are well-defined in guidelines.

C (Continue full dose): Contraindicated! Risks irreversible neurotoxicity.

D (Switch to decitabine): Not appropriate for fit patients receiving curative-intent therapy.
Question 8 (Transfusion Strategy)
Labs on Day 8:

Hemoglobin: 6.9 g/dL (asymptomatic)

Platelets: 9 x 10/L (no bleeding)

What is the MOST appropriate transfusion plan?
A) Transfuse platelets only (goal >10k) (prophylactic threshold)
B) Transfuse RBCs + platelets (goal Hb >7, platelets >10k) (ASCO guidelines)
C) Hold transfusions (monitor for symptoms) (risk of hemorrhage)
D) Transfuse platelets only if bleeding occurs (not standard)
my answer is B
Feedback on Question 8 (Correct Answer: B)

Perfect! Youve applied the guidelines precisely.

Rationale:
B (Transfuse RBCs + platelets) is 100% aligned with ASCO/AABB standards:

Platelets 9k Prophylactic transfusion (goal >10k to prevent spontaneous bleeding).

Hb 6.9 g/dL Meets RBC threshold (7 for stable patients, even if asymptomatic).

Why Not the Others?
A (Platelets only): Ignores evidence-based Hb threshold for RBCs.

C (Hold all): Dangerous9k platelets = high bleeding risk (even without symptoms).

D (Wait for bleeding): Reactive (not prophylactic) transfusions increase mortality.
Question 9 (Differentiating Infection vs. Engraftment Syndrome)
On Day 15, the patient develops:

Fever (39C) + rash on trunk/arms

WBC: 1.2 3.8 x 10/L (ANC 0.8)

No clear infection source

What is the MOST likely diagnosis?
A) Bacterial sepsis (unlikely without hypotension/organ dysfunction)
B) Engraftment syndrome (timing + WBC rise + rash)
C) Invasive fungal infection (would present with pulmonary symptoms)
D) Chemotherapy-induced fever (typically occurs during infusion)
nswer is B
Feedback on Question 9 (Correct Answer: B)

Spot on! Youve nailed the classic presentation of engraftment syndrome.

Rationale:
B (Engraftment syndrome) is most likely given:

Timing: Day 15 (coinciding with WBC recovery).

Key features: Fever + non-blanching rash (trunk/arms) + ANC rise (0.8).

No infection source: Negative cultures/imaging would further support this.

Why Not the Others?
A (Bacterial sepsis): Requires hypotension/organ failure (absent here).

C (Fungal infection): Presents with pulmonary nodules/sinus pain (not isolated rash).

D (Chemo-induced fever): Occurs during infusion (e.g., cytarabine fever on Day 1-7).
Question 10 (Post-Induction MRD Monitoring)
Day 30 Bone Marrow Biopsy Results:

Morphologic remission (<5% blasts)

Flow cytometry: MRD 0.1% (NPM1mut detectable by PCR)

What is the NEXT step in therapy?
A) Proceed to consolidation with HiDAC (standard for favorable-risk MRD-negative)
B) Switch to venetoclax + azacitidine (used for MRD persistence)
C) Allogeneic HSCT (overkill for favorable risk with low MRD)
D) Repeat biopsy in 4 weeks (delays definitive therapy)
answer is A
Feedback on Question 10 (Correct Answer: A)

Absolutely correct! Youve selected the guideline-endorsed approach for this favorable-risk patient.

Rationale:
A (HiDAC consolidation) is standard for favorable-risk AML in morphologic remission, even with MRD 0.1% (per ELN 2022/NCCN).

HiDAC (3-4 cycles) reduces relapse risk in NPM1mut AML.

MRD 0.1% is borderlinerepeat NPM1 PCR after Cycle 1 to guide further action.

Why Not the Others?
B (Venetoclax + azacitidine): For unfit/adverse-risk or MRD 1% (not yet indicated here).

C (HSCT): Overtreatment for favorable-riskreserved for persistent MRD or relapse.

D (Repeat biopsy): Delays therapyMRD is already measurable and actionable.
Phase 3: Post-Remission Therapy & Long-Term Management

Case Update (Post-Consolidation, Month 4)
The patient completed 3 cycles of HiDAC (1.5 g/m BID, adjusted for age/renal function).

Current Status:

Morphologic remission (blasts <5%)

MRD by NPM1 PCR: Undetectable

Recovery: ANC 1.8, platelets 110, Hb 10.2

New Issue: Persistent fatigue + mild neuropathic pain (feet).

Question 11 (Long-Term MRD Monitoring)
What is the recommended schedule for MRD monitoring in this NPM1mut patient?
A) Bone marrow biopsy + NPM1 PCR every 3 months x 2 years (ELN 2022 standard)
B) Peripheral blood NPM1 PCR monthly indefinitely (overkill)
C) Repeat biopsy only if symptoms recur (misses early relapse)
D) Discontinue monitoring (cured) (unrealistic)
answer is A
Feedback on Question 11 (Correct Answer: A)

Excellent choice! Youve selected the evidence-based monitoring schedule for this favorable-risk patient.

Rationale:
A (BM biopsy + NPM1 PCR q3mo x 2 years) aligns with ELN 2022 recommendations:

NPM1mut AML requires quantitative PCR monitoring (marrow preferred for sensitivity).

Frequency: q3mo for 2 years (highest relapse risk period), then q6mo until year 5.

Peripheral blood can supplement but not replace marrow (10-20% false negatives).

Why Not the Others?
B (Monthly PB PCR): Not cost-effectiveno survival benefit vs. q3mo marrow.

C (Biopsy only if symptomatic): Misses early MRD+ relapse (when salvage therapy is most effective).

D (Discontinue monitoring): NPM1mut AML can relapse years laterlifelong follow-up is needed.
Question 12 (Managing Neuropathy)
The patient reports burning feet pain (grade 2). This likely stems from:
A) Cytarabine toxicity (dose-dependent neurotoxicity)
B) Diabetes exacerbation (pre-existing neuropathy)
C) Vitamin B12 deficiency (unrelated to chemo)
D) Leukemic infiltration (unlikely in remission)
answer is A
Feedback on Question 12 (Correct Answer: A)

Exactly right! Cytarabine is the most likely culprit given the treatment history.

Rationale:
A (Cytarabine toxicity):

HiDAC cumulative dose = 27 g/m (threshold for neurotoxicity: >24 g/m).

Classic presentation: Sensory neuropathy (burning/paresthesia in extremities).

Onset typically weeks-months post-treatment.

Why Not the Others?
B (Diabetes): Less likelyno prior neuropathy symptoms + HbA1c 7.8% is moderate.

C (B12 deficiency): Requires risk factors (e.g., pernicious anemia, malnutrition).

D (Leukemic infiltration): Extremely unlikely in morphologic/MRD-negative remission.

Question 13 (Fatigue Management)
The patients fatigue persists 4 months post-consolidation. Labs show:

Hb 10.2 g/dL (MCV 88), TSAT 18%, ferritin 120 ng/mL

Thyroid/Vit B12 normal

What is the BEST intervention?
A) Iron supplementation (functional iron deficiency)
B) Erythropoietin (EPO) (avoid in AML survivors)
C) Methylphenidate trial (for cancer-related fatigue)
D) Exercise referral + sleep hygiene (first-line per ASCO)
answer is D
Feedback on Question 13 (Correct Answer: D)

Perfect! Youve selected the first-line, guideline-endorsed approach to cancer-related fatigue.

Rationale:
D (Exercise + sleep hygiene) is ASCO/ESMOs top recommendation for fatigue in remission:

Aerobic exercise (e.g., 150 min/week walking) reduces fatigue better than drugs.

Cognitive behavioral therapy for insomnia (CBT-I) improves sleep quality.

Why Not the Others?
A (Iron): TSAT 18% + ferritin 120 = anemia of chronic disease (not iron deficiency).

B (EPO): Contraindicated in AML survivors (black-box warning for tumor progression).

C (Methylphenidate): Second-line for severe fatigue after non-drug measures fail.
Question 14 (Relapse Scenario)
At Month 18, MRD monitoring detects:

NPM1 PCR: 0.05% in blood (confirmed in marrow)

No morphologic relapse (blasts 3%)

What is the NEXT step?
A) Immediate reinduction with 7+3 (overtreatment for molecular relapse)
B) Venetoclax + azacitidine (preferred for MRD+ relapse)
C) Donor search for allogeneic HSCT (preemptive for high-risk genetics)
D) Enroll in a clinical trial (best for MRD+ NPM1mut AML)
Feedback on Question 14 (Correct Answer: B)

Excellent choice! Youve selected the emerging standard of care for NPM1mut MRD+ relapse.

Rationale:
B (Venetoclax + azacitidine) is increasingly used for MRD+ relapse (per ELN 2022/ESMO updates):

High response rates in NPM1mut AML (ORR ~70% in MRD+ setting).

Bridges to transplant if MRD persists (donor search can occur concurrently).

Why Not the Others?

A (7+3 reinduction): Overtreatment for molecular relapse (no morphologic disease).

C (Donor search): PrematureHSCT is not first-line for favorable-risk MRD+ without trial failure.

D (Clinical trial): Also reasonable, but ven/aza is the current standard backbone.
Question 15 (Novel Therapies)
The patient enrolls in a trial of NPM1-targeted vaccine + azacitidine. What is the mechanism?
A) Induces T-cells against NPM1 neoepitopes (immunotherapy)
B) Degrades mutant NPM1 protein (PROTAC strategy)
C) Blocks NPM1 nuclear export (selinexor-like)
D) Inhibits NPM1-mutant IDH2 (off-target effect)
Feedback on Question 15 (Correct Answer: A)

Spot on! Youve nailed the mechanism of NPM1-targeted vaccines.

Rationale:
A (Induces T-cells against NPM1 neoepitopes):

NPM1mut creates unique peptide sequences recognizable by T-cells.

Vaccines + azacitidine enhance antigen presentation (hypomethylating agents upregulate HLA).

Why Not the Others?
B (PROTAC): Degrades proteins via ubiquitinationnot vaccine-related.

C (Selinexor-like): Blocks XPO1 (nuclear export), irrelevant here.

D (IDH2 inhibition): NPM1 and IDH2 mutations are distinct.

Session ID:
Assessment Domain

Session-6:
Rationale

Session ID:
Instructional Design and Pedagogical

Session-6:
The session demonstrates excellent instructional design, with expert scaffolding that seamlessly progresses from initial diagnosis and risk stratification through induction complications, post-remission monitoring, and complex long-term management. The feedback is consistently insightful, citing relevant guidelines (ELN, ESMO, IDSA, ASCO) and providing clear, evidence-based rationales. The instructions are unambiguous and the question-and-answer format is intuitive. The only minor weakness is the lack of explicitly stated, measurable learning objectives at the outset, though the educational goals are clearly implied by the case's comprehensive structure.

Session ID:
Clinical Authenticity and Content Fidelity

Session-6:
The clinical narrative is highly plausible and indistinguishable from an authentic patient case. However, there are some inaccuracies in its alignment with evidence and guidelines. Strengths: Initial diagnosis, risk stratification, induction therapy selection, and management of febrile neutropenia and TLS are all perfectly aligned with current standards. Inaccuracies: Two recommendations present debatable or overstated claims as settled fact. In Question 11, the rationale asserts that bone marrow is superior to peripheral blood for NPM1 PCR monitoring, a view that is not universally held and downplays the utility of non-invasive monitoring. In Question

14, venetoclax plus azacitidine is presented as the "preferred" regimen for molecular relapse, which overstates its current standing relative to clinical trial enrollment, the more common guideline recommendation. While not dangerous, these inaccuracies represent a moderate deviation from a nuanced, evidence-based consensus. The session demonstrates perfect domain specificity, with all therapies and diagnostics being appropriate for AML. In Q 5, there is a deviation from the guidelines that indicates the high risk criteria for TLS in AML patient, the high WBC over 100k. The question mentioned that, " This patient meets all high-risk criteria: WBC >25k".

Session ID:

Modeled Clinical Reasoning

Session-6:

The simulation excels at modeling an expert clinical reasoning process. Each stage of the case presents a well-defined clinical problem with the necessary data to make a decision. The multiple-choice options provide a sophisticated breadth of plausible and relevant alternatives, including common distractors that effectively test nuanced understanding (e.g., distinguishing engraftment syndrome from infection). The narrative demonstrates appropriate, risk-adjusted prioritization at every step, such as making critical chemotherapy dose adjustments for renal impairment and managing late effects with evidence-based non-pharmacologic interventions first. The clinical outcomes are realistic and highly instructive.

Session-7

Session ID:
LLM

Session-7:
DeepSeek V2

Session ID:
Session Transcript

Session-7:
Case Introduction**
Patient Profile:
- A 62-year-old male with a history of hypertension (well-controlled on lisinopril) and type 2 diabetes (on metformin).
- **Presenting Symptoms:** Fatigue for 6 weeks, dyspnea on exertion, recurrent gum bleeding, and unexplained bruising.
- **Social History:** Former smoker (quit 10 years ago), works as a schoolteacher, limited access to specialty care (lives in a rural area).

Initial Lab Results (CBC with differential):
- WBC: 38.5 x 10/L () with 20% blasts
- Hemoglobin: 8.2 g/dL ()
- Platelets: 45 x 10/L ()
- Absolute neutrophil count (ANC): 1.1 x 10/L

Peripheral blood smear: Auer rods present in some blasts.

Question 1 (Knowledge Recall):
Which next step is MOST critical to confirm AML diagnosis per WHO 2022 criteria?
A) Flow cytometry of peripheral blood
B) Bone marrow biopsy with cytogenetics/molecular studies
C) CT scan of chest/abdomen to rule out solid tumor metastasis
D) Immediate empiric antibiotics for suspected infection
Correct Answer: B) Bone marrow biopsy with cytogenetics/molecular studies**

Feedback:

Why This Is Correct:
- **Bone marrow biopsy** is the **gold standard** for AML diagnosis per **WHO 2022 criteria**, as it:
  - Quantifies blast percentage (morphology).
  - Provides **cytogenetics** (e.g., karyotype for deletions, translocations).
  - Identifies **molecular markers** (*FLT3-ITD*, *NPM1*, *CEBPA*, *IDH1/2*) critical for risk stratification (ELN 2022).
- **Auer rods** (seen on smear) suggest **myeloid lineage**, further supporting AML.

Why Other Options Are Incorrect:
- **A) Flow cytometry of peripheral blood:**
  - Supports diagnosis but **insufficient alone**cannot assess marrow blast % or genetic abnormalities.

- **C) CT scan of chest/abdomen:**
  - Unnecessary unless infection or solid tumor is suspected (no clinical hints here).
- **D) Empiric antibiotics:**
  - Premature without confirmed infection (e.g., no fever, ANC > 0.5 x 10/L).
Proceeding to Question 2 (Risk Stratification):**

Bone marrow results return:
- **Blasts:** 35%
- **Cytogenetics:** Normal karyotype
- **Molecular studies:** *NPM1* mutation (+), *FLT3-ITD* (-), *IDH2* mutation (+)

Question 2:
How would you classify this patients risk per ELN 2022 criteria?
A) Favorable
B) Intermediate
C) Adverse
Adverse**

Feedback:

Correct Answer: A) Favorable

Rationale (ELN 2022 Risk Stratification):
- **Favorable Risk** is defined by:
  - *NPM1* mutation **without** *FLT3-ITD* (as in this case).
  - *CEBPA* mutation (biallelic).
  - Normal karyotype with these mutations confers better prognosis.
- **Why Not Adverse (C)?**
  - Adverse risk requires high-risk features (e.g., *TP53* mutation, complex karyotype, *FLT3-ITD* **with high allelic ratio**).
  - *IDH2* mutations are **not adverse**they may even be targetable with enasidenib.

Therapeutic Decision-Making):**
Assuming this patient is medically fit (PS 0, normal cardiac/renal function), what is the MOST appropriate induction regimen?
A) Venetoclax + azacitidine
B) "7+3" (cytarabine + daunorubicin)
C) Gemtuzumab ozogamicin + cytarabine
D) Supportive care only (transfusions, antibiotics)
Correct Answer: B) "7+3" (cytarabine + daunorubicin)**

Feedback:

Why This Is Correct:
- **Patient Factors:**
  - **Fit (PS 0, normal organ function)** Eligible for intensive induction.
  - **Favorable-risk AML** (*NPM1+*, *FLT3-ITD-*) High likelihood of response to 7+3.
- **Guideline Alignment (ESMO 2021, NCCN 2023):**
  - **7+3 (cytarabine + anthracycline)** is **first-line for fit patients** with de novo AML.
  - *NPM1* mutation predicts **better response to cytarabine-based therapy**.

Why Other Options Are Less Appropriate:

- **A) Venetoclax + azacitidine:**
  - Reserved for **unfit patients** (age 75 or comorbidities). This patient is fit.
- **C) Gemtuzumab ozogamicin (GO) + cytarabine:**
  - Used in **core-binding factor AML** (e.g., inv(16), not *NPM1*-mutated AML.
- **D) Supportive care only:**
  - Contraindicated in fit patients with untreated AML (curative intent needed).

Clinical Pearl:
- **Anthracycline choice:**
  - Daunorubicin (6090 mg/m) or idarubicin (12 mg/m) are options.
  - **No *IDH2* inhibitor (e.g., enasidenib) upfront**targeted therapy is for relapse/refractory disease.
Supportive Care):**
During induction, the patient develops fever (38.5C) and severe mucositis. ANC is 0.1 x 10/L. Blood cultures grow *E. coli*. What is the NEXT BEST step?
A) Add vancomycin for MRSA coverage
B) Switch to meropenem + vancomycin + voriconazole
C) Continue current antibiotics (e.g., cefepime) and add granulocyte colony-stimulating factor (G-CSF)
D) Discontinue all chemotherapy immediately
Incorrect Answer: C) Continue current antibiotics (e.g., cefepime) and add granulocyte colony-stimulating factor (G-CSF)**

Feedback:

Correct Answer: B) Switch to meropenem + vancomycin + voriconazole

Rationale (IDSA 2022 Guidelines for Febrile Neutropenia):
1. **Escalation Needed:**
   - The patient has **breakthrough Gram-negative bacteremia (*E. coli*)** while likely on prophylactic antibiotics (e.g., fluoroquinolone) or initial empiric therapy (e.g., cefepime).
   - **Mucositis + neutropenia** increases risk for **anaerobes, *Pseudomonas*, and fungi** warrants **carbapenem (meropenem)** for broader Gram-negative coverage.

2. **Vancomycin Indication:**
   - Added for **suspected Gram-positive infection** (e.g., mucositis-related *Streptococcus mitis* bacteremia, hemodynamic instability).

3. **Voriconazole Addition:**
   - **Severe mucositis + prolonged neutropenia** raises risk for **invasive fungal infections (e.g., *Aspergillus*)**.

4. **Why G-CSF is Incorrect:**
   - **Not recommended in AML** (ASCO/IDSA): No survival benefit and may stimulate leukemic blasts.

Why Other Options Fail:
- **A) Vancomycin alone:** Misses Gram-negative escalation and fungal coverage.
- **C) G-CSF:** Harmful in AML (as above).
- **D) Discontinue chemo:** Only for life-threatening toxicity (e.g., cardiac failure).

Clinical Pearl:
- **Antibiotic de-escalation** is possible once cultures narrow and fever resolves.

Question 5 (Post-Induction Evaluation):**
Post-induction, the patient achieves morphologic remission. Bone marrow shows 2% blasts, but MRD testing (flow cytometry) detects 0.1% leukemic cells. What is the NEXT BEST step?

A) Proceed to allogeneic stem cell transplant (allo-HSCT)
B) High-dose cytarabine consolidation (HiDAC)
C) Start enasidenib (IDH2 inhibitor)
D) Repeat induction chemotherapy
Incorrect Answer: A) Proceed to allogeneic stem cell transplant (allo-HSCT)**

Feedback:

Correct Answer: B) High-dose cytarabine consolidation (HiDAC)

Rationale (ELN 2022 MRD Guidance):
1. **Patient's Risk Status:**
   - **Favorable-risk AML** (*NPM1+*, *FLT3-ITD-*, *IDH2+*) **Not a transplant candidate upfront**.
   - **MRD positivity (0.1%)** alone does **not** change risk category in favorable-risk disease.

2. **Standard of Care:**
   - **HiDAC (high-dose cytarabine)** is the **consolidation backbone** for favorable-risk AML (34 cycles improve long-term survival).

   - **Transplant (allo-HSCT)** is reserved for:
     - **Adverse-risk AML** (e.g., *TP53* mutation).
     - **Persistent MRD after consolidation** (e.g., rising MRD post-HiDAC).

3. **Why Not Other Options?**
   - **C) Enasidenib:** Used for **relapsed/refractory *IDH2*-mutated AML**, not consolidation.
   - **D) Repeat induction:** Unnecessary for morphologic remission (blasts <5%).

MRD Clinical Pearl:
- **Flow cytometry MRD 0.1%** predicts higher relapse risk but does **not** override ELN genetic risk.
- **Action threshold:** Transplant only if MRD persists **after consolidation** in favorable-risk disease.

Consolidation Therapy Selection)**
The patient completes **Cycle 1 of HiDAC (cytarabine 3 g/m x 6 doses)**. Pre-cycle 2 labs show:
- Creatinine: 1.6 mg/dL (baseline 0.8)
- Urine output: 0.3 mL/kg/hr over 12 hours
- CBC: ANC 0.8 x 10/L (recovering), platelets 90 x 10/L

What is the MOST likely cause of acute kidney injury (AKI)?
A) Tumor lysis syndrome (TLS)
B) Cytarabine-induced nephrotoxicity
C) Sepsis-associated AKI
D) Lisinopril toxicity
Correct Answer: B) Cytarabine-induced nephrotoxicity**

Feedback:

Why This Is Correct:
- **HiDAC (high-dose cytarabine) is nephrotoxic** in 1020% of patients, typically manifesting as:
  - **Acute tubular necrosis** (elevated Cr, oliguria) within 2448 hours of infusion.
  - **Risk factors:** High doses (>1 g/m), age >60, pre-existing CKD.
- **Timing fits:** AKI occurred **post-HiDAC**, not at induction (ruling out TLS) or with fever/hypotension (ruling out sepsis).

Why Other Options Are Less Likely:
- **A) Tumor lysis syndrome (TLS):**
  - Unlikely **post-remission** (no bulk disease). TLS typically occurs during induction.
  - Labs would show **hyperuricemia, hyperkalemia, hyperphosphatemia** (absent here).
- **C) Sepsis-associated AKI:**
  - No fever/hypotension; ANC is recovering (0.8 x 10/L).
- **D) Lisinopril toxicity:**
  - Typically causes **pre-renal AKI** (hypotension, no oliguria). Patient has **oliguria + tubular injury pattern**.

Guideline Reference:
- **NCCN 2023 Supportive Care:** Cytarabine 1 g/m requires **renal monitoring**; dose-reduce for Cr 2 mg/dL.

Clinical Pearl:
- **Management:**
  - Hold HiDAC until Cr improves.
  - Next cycle: **Reduce cytarabine dose by 50%** (e.g., 1.5 g/m) or switch to **intermediate-dose cytarabine** (11.5 g/m).
  - Avoid nephrotoxins (e.g., NSAIDs, IV contrast).

MRD Monitoring & Therapy Adjustment)**
After 3 cycles of HiDAC, MRD testing (NPM1 RT-qPCR) shows:
- **Peripheral blood:** *NPM1* mutation detected (0.01% variant allele frequency)
- **Bone marrow:** 0.05% blasts by flow cytometry

What is the NEXT BEST step?
A) Proceed to allo-HSCT
B) Switch to venetoclax + azacitidine
C) Continue HiDAC for 1 more cycle
D) Enroll in a clinical trial with IDH2 inhibitor
Incorrect Answer: A) Proceed to allo-HSCT**

Feedback:

Correct Answer: C) Continue HiDAC for 1 more cycle

Rationale (ELN 2022 MRD Guidelines):
1. **MRD Significance:**
   - **Low-level MRD (0.010.05%)** in **favorable-risk AML** (*NPM1+*) does **not** mandate transplant.
   - **ELN 2022:** Transplant is reserved for **persistent MRD after completion of consolidation** (e.g., 0.1% post-cycle 4).

2. **Standard Approach:**
   - Complete planned **4 cycles of HiDAC** (survival benefit in favorable-risk AML).
   - **Repeat MRD testing post-cycle 4** before considering escalation.

3. **Why Not Other Options?**
   - **B) Venetoclax + azacitidine:** For **unfit patients** or relapsethis patient is fit and still responding.
   - **D) IDH2 inhibitor trial:** *IDH2* inhibitors (e.g., enasidenib) are for **relapsed/refractory disease**, not MRD persistence.

Clinical Pearl:
- **NPM1 RT-qPCR is highly sensitive** (detects 1 in 10,000 cells). **Transplant is only indicated if:**
  - MRD **rises** after consolidation.
  - MRD 0.1% **with adverse genetic features** (e.g., *FLT3-ITD+*).

Supportive Care Challenge)**
During cycle 4 HiDAC, the patient develops cerebellar ataxia, dysmetria, and confusion. MRI shows no acute infarct. What is the MOST likely cause?
A) Cytarabine neurotoxicity
B) IDH2 inhibitor interaction
C) Wernicke encephalopathy
D) Metastatic AML (chloroma)
Correct Answer: A) Cytarabine neurotoxicity**

Feedback:

Why This Is Correct:
- **High-dose cytarabine (HiDAC) is neurotoxic** in 520% of patients, typically presenting with:
  - **Cerebellar dysfunction** (ataxia, dysmetria, dysarthria).
  - **Confusion/seizures** (with higher doses or renal impairment).
- **Risk factors:** Age >60, renal dysfunction (reduced drug clearance), cumulative doses >36 g/m.
- **MRI findings:** Often normal early; later may show **cerebellar atrophy**.

Why Other Options Are Less Likely:
- **B) IDH2 inhibitor interaction:** Irrelevantpatient is not on enasidenib.
- **C) Wernicke encephalopathy:** Requires thiamine deficiency (e.g., alcoholism). No nystagmus/ophthalmoplegia here.
- **D) Chloroma (extramedullary AML):** Rare in remission; would show **contrast-enhancing lesions** on MRI.

Guideline Reference:
- **NCCN 2023:** Discontinue HiDAC for **any neurologic toxicity**; switch to intermediate-dose cytarabine (11.5 g/m) if further consolidation is needed.

Clinical Pearl:
- **Management:**
  1. **Immediately stop cytarabine.**
  2. **Supportive care:** Physical therapy, seizure prophylaxis if confused.
  3. **Permanently avoid HiDAC**neurotoxicity is cumulative and irreversible.
Long-Term Survivorship)**
One year post-consolidation, the patient remains in remission but develops fatigue. Labs show:
- Hb: 7.8 g/dL (MCV 102 fL)
- Platelets: 110 x 10/L
- ANC: 1.5 x 10/L
- **Vitamin B12:** 200 pg/mL (low)
- **Methylmalonic acid (MMA):** Elevated

What is the MOST likely cause of macrocytic anemia?
A) Therapy-related MDS
B) Vitamin B12 deficiency
C) IDH2 inhibitor toxicity
D) AML relapse
Correct Answer: B) Vitamin B12 deficiency**

Feedback:

Why This Is Correct:

- **Key Findings:**
  - **Macrocytic anemia (MCV 102 fL)** + **low B12 (200 pg/mL)** + **elevated MMA** **Diagnostic for B12 deficiency**.
  - **Chemotherapy (especially cytarabine)** can impair intestinal absorption of B12.
- **No Evidence of Relapse/MDS:**
  - Normal platelets/ANC, no blasts on peripheral smear (implied by clinical context).

Why Other Options Are Less Likely:
- **A) Therapy-related MDS (t-MDS):**
  - Would show **persistent cytopenias, dysplastic changes**, or **new cytogenetic abnormalities** (e.g., -7/del7q).
- **C) IDH2 inhibitor toxicity:** Irrelevantpatient never received enasidenib.
- **D) AML relapse:** Unlikely without **blasts or dropping counts**.

Guideline Reference:
- **ASCO Survivorship Guidelines:** Screen for **nutritional deficiencies** in AML survivors post-chemotherapy (B12, folate, iron).

Clinical Pearl:
- **Management:**
  1. **Intramuscular B12 (1000 mcg weekly x 4, then monthly)**oral absorption may be unreliable post-chemo.
  2. **Monitor for neurologic symptoms** (e.g., peripheral neuropathy, cognitive changes).
Surveillance Strategy)**
What is the MOST appropriate long-term monitoring for this patient?
A) Annual bone marrow biopsies indefinitely
B) *NPM1* RT-qPCR every 3 months x 2 years, then every 6 months
C) CT scans every 6 months to detect extramedullary disease
D) *IDH2* mutation tracking via liquid biopsy

Correct Answer: B) *NPM1* RT-qPCR every 3 months x 2 years, then every 6 months**

Feedback:

Why This Is Correct:
- **ELN 2022 Recommendations** for *NPM1*-mutated AML:
  - **Peripheral blood *NPM1* RT-qPCR** is the **gold standard** for surveillance (more sensitive than flow cytometry).
  - **Frequency:**
    - Every **3 months x 2 years** (highest relapse risk period).
    - Every **6 months thereafter** until year 5.
  - **Bone marrow biopsy** only if **PB becomes positive** or cytopenias develop.

Why Other Options Are Less Appropriate:
- **A) Annual bone marrow biopsies:**
  - **Overtesting**low yield in asymptomatic patients with negative PB *NPM1*.
- **C) CT scans:**
  - Unnecessary unless **symptoms suggest chloroma** (e.g., soft tissue masses).
- **D) *IDH2* tracking:**
  - Not standard for surveillance*IDH2* clonal evolution is less predictive than *NPM1* persistence.

Clinical Pearl:
- **Relapse Prediction:**
  - A **rising *NPM1* transcript level** in PB often precedes morphologic relapse by **36 months**, allowing preemptive intervention (e.g., salvage therapy or transplant).

Session Conclusion
[REDACTED]], youve completed all 10 questions! Heres your **performance summary**:

Strengths:
- Strong grasp of **diagnostic criteria (WHO 2022)** and **risk stratification (ELN 2022)**.
- Correctly identified **HiDAC neurotoxicity** and **B12 deficiency** in survivorship.

Areas for Improvement:
- **Transplant indications:** Revisit ELN 2022 thresholds for allo-HSCT in favorable-risk AML.
- **Febrile neutropenia escalation:** Review IDSA 2022 guidelines for antibiotic selection.

Tailored Learning Resources:
1. [ELN 2022 Risk Stratification Table](https://ashpublications.org/blood/article/140/12/1345/485880/Diagnosis-and-Management-of-AML-in-Adults-2022)
2. [IDSA 2022 Febrile Neutropenia Guidelines](https://www.idsociety.org/practice-guideline/fever-and-neutropenia/)
3. [NCCN AML Survivorship Guidelines (v1.2023)](https://www.nccn.org/guidelines/guidelines-detail?category=3&id=1507)

Type "END" to close the session or ask any follow-up questions!

Well done! Youve navigated complex AML management decisions with a patient-centered, guideline-based approach.

Session ID:

Assessment Domain

Session-7:

Rationale

Session ID:

Instructional Design and Pedagogical

Session-7:

This simulation represents a gold standard in instructional design. The learning journey is expertly scaffolded, guiding the learner through the entire AML care continuum with a strong emphasis on managing therapy-related toxicities. The quality of the feedback is exemplary; it is evidence-based, provides clear rationales, and concludes with a personalized performance summary that identifies strengths and offers tailored learning resources. This final step is a hallmark of superior pedagogical design, creating a complete and effective learning loop.

Session ID:

Clinical Authenticity and Content Fidelity

Session-7:

The clinical authenticity is a mixed bag, with a highly plausible narrative undermined by a significant pharmacotherapeutic error. Plausibility & Guideline Alignment: The clinical narrative is indistinguishable from an authentic patient case, with realistic complications. Most recommendations are perfectly aligned with current guidelines, including diagnosis, risk stratification, induction choice, and management of cytarabine toxicities. However, the recommendation in Question 4escalating immediately to meropenem, vancomycin, and voriconazole for a stable patient with breakthrough bacteremiarepresents a significant deviation from IDSA/ESMO guidelines, which call for a more stepwise approach. Pharmacotherapeutic Accuracy: This antibiotic recommendation is a questionable and suboptimal choice, reflecting poor antimicrobial stewardship and not representing the standard of care for this clinical scenario. Domain Specificity: The case remains strictly within the specified disease domain of AML, with no evidence of entanglement.

Session ID:
Modeled Clinical Reasoning

Session-7:
The simulation excels at modeling an expert clinical reasoning process. Each stage of the case presents a well-defined clinical problem with the necessary data for a decision. The multiple-choice options provide a sophisticated breadth of plausible alternatives and effective distractors that test nuanced understanding (e.g., correctly identifying HiDAC consolidation as the standard for favorable-risk, MRD-low disease over transplant). The narrative demonstrates appropriate, risk-adjusted prioritization throughout, and the clinical outcomes (AKI, neurotoxicity, B12 deficiency) are realistic and highly instructive. The strategic reasoning is sound, even where the specific antibiotic choice was flawed.

Session-8

Session ID:
LLM

Session-8:
DeepSeek V2

Session ID:
Session Transcript

Session-8:
A 58-year-old male with a history of hypertension (controlled on lisinopril) presents with 3 weeks of fatigue, gingival bleeding, and recurrent fevers. CBC shows WBC 32 x 10/L (blasts 40%), Hb 7.8 g/dL, platelets 45 x 10/L. LDH is elevated at 850 U/L. Social history reveals intermittent smoking and limited access to fresh foods (lives in a food desert).
Question 1/15 Correct! Youve selected the most critical diagnostic test for this patient. Heres why:

Key Rationale
Confirms AML Diagnosis:

Bone marrow biopsy assesses blast percentage (20% required for AML diagnosis per WHO 2022 criteria).

Rules out myelodysplasia-related changes or other mimics.:
Which diagnostic test is most critical to confirm AML and guide risk stratification?
A) Peripheral blood flow cytometry
B) Bone marrow biopsy with cytogenetics/NPM1/FLT3-ITD testing
C) CT scan of the chest/abdomen
D) Coagulation studies (PT/INR, fibrinogen)
Correct! Youve selected the most critical diagnostic test for this patient. Heres why:

Key Rationale
Confirms AML Diagnosis:

Bone marrow biopsy assesses blast percentage (20% required for AML diagnosis per WHO 2022 criteria).

Rules out myelodysplasia-related changes or other mimics.
Guides Risk Stratification (ELN 2022):

Cytogenetics (e.g., t(8;21), inv(16), -7/del(7q)) define core risk groups.

Molecular Profiling:

FLT3-ITD (high allelic ratio adverse risk).

NPM1 mutation (favorable risk if FLT3-ITD-negative).

IDH1/2, TP53 mutations impact therapy selection (e.g., IDH inhibitors).

Therapeutic Implications:

FLT3-ITD+? Add midostaurin to induction.

Adverse risk? Consider clinical trials or allo-HCT in remission

Why Not Other Options?
A) Peripheral Flow Cytometry: Supports AML diagnosis but cannot replace biopsy for genetic risk stratification.

C) CT Scan: Useful for infection evaluation (e.g., fungal pneumonia in neutropenia) but not diagnostic.

D) Coagulation Studies: Important for DIC screening but secondary to definitive diagnosis.
Clinical Pearl
Time Sensitivity: Biopsy should be performed within 24-48 hours of presentation to avoid delays in induction therapy.

Social Context: This patients limited access to fresh foods may necessitate nutrition support (e.g., vitamin C/folate repletion) during treatment.
Case Update:
The bone marrow biopsy confirms AML with:

FLT3-ITD mutation (allelic ratio 0.8 high risk)

NPM1 wild-type

Cytogenetics: Normal karyotype
ELN 2022 Risk: Adverse

Question 2/15:
What is the most appropriate induction regimen for this patient?
A) "7+3" (cytarabine + daunorubicin) alone
B) "7+3" + midostaurin (FLT3 inhibitor)
C) Venetoclax + azacitidine
D) Supportive care only (transfusions, antibiotics)
Correct! You selected B) "7+3" + midostaurinthe evidence-based choice for this FLT3-ITD+ AML patient.
Why B is Correct
Guideline Alignment (ELN 2022/ESMO):

FLT3-ITD+ AML requires FLT3 inhibition during induction (Category 1 recommendation).

RATIFY Trial (Stone et al. NEJM 2017):
7+3 + midostaurin showed 23% reduction in mortality vs chemo alone (5-year OS 51% vs 44%).

Midostaurin is dosed 50 mg PO BID on days 8-21 (synergizes with chemo-induced apoptosis).

Patient-Specific Justification:

Adverse-risk profile (FLT3-ITD high ratio, NPM1-wt) demands aggressive therapy.

Age 58, PS 1: Fit for intensive chemo (no cardiac/hepatic/renal contraindications).
Why Other Options Are Incorrect
A) 7+3 alone: Misses FLT3 targeting inferior outcomes in FLT3-ITD+ AML.

C) Venetoclax + azacitidine:

Used for unfit patients (75 years or comorbidities).

No proven benefit in FLT3-ITD+ AML (data limited to retrospective studies).

D) Supportive care: Not appropriate for a fit patient with curative-intent eligibility
Clinical Pearls

Midostaurin Logistics:

Start day 8 (after chemo completes) to avoid overlapping toxicity.

Drug Interactions: Strong CYP3A4 inhibitors (e.g., voriconazole) require dose reduction to 30 mg BID.
Next Steps After Induction:

Day 14 bone marrow: Assess for residual blasts.

Consolidation: Allo-HCT recommended for adverse-risk AML (if feasible).

The patient develops febrile neutropenia (FN) on day 5 of induction (fever 38.5C, ANC <500/L). Blood cultures grow E. coli. His prophylactic regimen included posaconazole and acyclovir but no antibacterial prophylaxis.

Which prophylactic agent was likely omitted, leading to this complication?
A) Levofloxacin
B) Posaconazole
C) Acyclovir
D) Aspirin

Correct! You selected A) Cytarabinethis is the component requiring dose adjustment due to renal dysfunction (creatinine rising to 2.2 mg/dL).
Detailed Feedback
Why A is Correct
Cytarabine and Renal Impairment:

Cytarabine is renally excreted (80% unchanged in urine).

ELN 2022/ESMO Guidelines: Reduce dose by 50% if CrCl <60 mL/min (or serum Cr 2 mg/dL).

Risk of neurotoxicity (cerebellar dysfunction) with standard doses in renal impairment.
Patient-Specific Justification:

Creatinine 2.2 mg/dL CrCl ~30-40 mL/min (using Cockcroft-Gault).

Action: Reduce cytarabine from 100200 mg/m/day to 50100 mg/m/day.
Why Other Options Are Incorrect
B) Daunorubicin: Primarily hepatically metabolized; no renal dose adjustment needed.

C) Midostaurin: Metabolized by CYP3A4; renal adjustment unnecessary (per package insert).

D) No adjustment: Incorrectcytarabine toxicity risk is unacceptable.
Monitoring During Dose Reduction:

Daily creatinine/CrCl (cytosine arabinoside clearance correlates with renal function).

Neurologic checks (nystagmus, dysarthria, ataxia signal cerebellar toxicity).

Alternative for Severe Renal Dysfunction:

Consider non-renally cleared regimens (e.g., liposomal daunorubicin-cytarabine [CPX-351] if eligible).
The patients day 28 bone marrow shows:

5% blasts (morphologic remission but MRD+ by flow cytometry)

FLT3-ITD remains detectable (allelic ratio 0.4, reduced from 0.8)

Persistent pancytopenia (ANC 300/L, platelets 25,000/L)
What is the next best step?
A) Repeat induction with high-dose cytarabine
B) Proceed to allo-HCT in first remission
C) Switch to venetoclax + azacitidine
D) Palliative care

Correct! You selected B) Proceed to allo-HCT in first remissionthe only potentially curative option for this patient with MRD+ adverse-risk AML.
Detailed Feedback
Why B is Correct
ELN 2022 & NCCN Guidelines:

Adverse-risk AML + MRD+ after induction urgent allo-HCT in CR1 is standard (Category 1 recommendation).

FLT3-ITD+ disease benefits most from HCT due to high relapse risk (Perl et al, Blood 2022).
Patient-Specific Factors:

Age 58, PS 1, HLA-matched donor available excellent transplant candidate.

MRD+ (5% blasts, FLT3-ITD persistent) chemotherapy alone has near-100% relapse risk.

Supporting Evidence:

BMT CTN 1703 Trial: FLT3-ITD+ AML patients undergoing HCT in CR1 had 55% 3-year survival vs 20% with chemo alone.

SORMAIN Trial: Post-HCT FLT3 inhibitor maintenance (e.g., sorafenib) further reduces relapse.
Why Other Options Are Incorrect
A) Repeat induction: Delays HCT without clearing MRD in adverse-risk disease.

C) Venetoclax + azacitidine: No role in fit, transplant-eligible patients; inferior to HCT.

D) Palliative care: Unjustified for a patient with curative-intent options.

Clinical Pearls
Transplant Timeline:

Initiate workup immediately (donor confirmatory typing, cardiac/pulmonary evaluation).

Bridging therapy: Consider FLT3 inhibitor (e.g., gilteritinib) to suppress MRD pre-HCT.
Post-HCT Strategies:

FLT3 inhibitor maintenance (e.g., midostaurin/sorafenib) improves outcomes (SORMAIN trial).

MRD monitoring: Peripheral blood FLT3-ITD qPCR every 3 months guides early intervention.
The patient undergoes RIC allo-HCT and achieves full donor chimerism. No GVHD is present at day +60. Should FLT3 inhibitor maintenance be initiated?
A) Yes, start midostaurin 50 mg PO BID
B) Yes, start gilteritinib 120 mg PO daily
C) No, wait for MRD+ or overt relapse

D) No, use azacitidine instead
Correct! You selected B) Yes, start gilteritinib 120 mg PO dailythe preferred FLT3 inhibitor for post-HCT maintenance in FLT3-ITD+ AML.
Detailed Feedback
Why B is Correct
Guideline Recommendations (NCCN/ELN 2022):

Gilteritinib is preferred over midostaurin for maintenance due to:

Higher potency and selectivity for FLT3-ITD (ADMIRAL trial, Perl et al, NEJM 2019).
Proven survival benefit in relapsed/refractory FLT3-ITD+ AML.

Initiate day +60 to +100 post-HCT if no GVHD (per SORMAIN trial design).
Supporting Evidence:

SORMAIN Trial (Sandmaier et al, Blood 2021): Sorafenib (another FLT3 inhibitor) improved RFS post-HCT.

MORPHO Trial (ongoing): Evaluating gilteritinib vs placebo post-HCT.

Why Other Options Are Incorrect
A) Midostaurin: Less potent than gilteritinib; no post-HCT efficacy data.

C) Wait for relapse: Unacceptable for MRD+ adverse-risk AML.

D) Azacitidine: Less effective than FLT3 inhibitors for FLT3-ITD+ disease.

Clinical Pearls

Gilteritinib Logistics:

Dose: 120 mg PO daily (reduce to 80 mg if combined with strong CYP3A4 inhibitors).

Monitoring:

FLT3-ITD qPCR every 3 months.

ECG (QT prolongation risk).

GVHD Management:

If GVHD develops, hold gilteritinib until resolved (risk of exacerbation).

Session ID:
Assessment Domain

Session-8:
Rationale

Session ID:
Instructional Design and Pedagogical

Session-8:
Rationale: The session's instructional design is excellent. The case is expertly scaffolded, progressing logically from initial diagnosis and risk stratification through induction, consolidation with transplant, and post-transplant maintenance. The quality of the embedded

feedback is a key strength; each rationale is detailed, cites current clinical guidelines (e.g., ELN 2022) and landmark trials (e.g., RATIFY, SORMAIN), and includes "Clinical Pearls" that provide valuable context. Instructions are clear and unambiguous. The only minor weakness is the lack of explicitly stated learning objectives at the start of the session, though the educational goals are clearly implied by the content and structure.

Session ID:
Clinical Authenticity and Content Fidelity

Session-8:
The session presents a highly authentic clinical narrative that perfectly mirrors real AML patient management. However, a significant pharmacotherapeutic error occurs in the final question regarding post-HCT FLT3 inhibitor maintenance. The session incorrectly states that "gilteritinib is preferred over midostaurin for maintenance" and cites "NCCN/ELN 2022 guidelines" as support. This is factually incorrect. The evidence for post-HCT FLT3 inhibitor maintenance comes from the SORMAIN trial, which used sorafenib, not gilteritinib. Gilteritinib is established for relapsed/refractory FLT3+ AML (ADMIRAL trial) but lacks robust evidence for post-HCT maintenance. Current guidelines favor sorafenib or midostaurin for this indication. The session mentions an "ongoing MORPHO trial" but this doesn't justify recommending gilteritinib as preferred standard of care. This represents a significant content error where clinical guidelines are misrepresented, though the case remains within the AML domain without entanglement issues.

Session ID:
Modeled Clinical Reasoning

Session-8:
The simulation excellently models expert-level clinical reasoning throughout the AML management continuum. Each clinical challenge is expertly framed with appropriate complexity, from initial risk stratification to complex post-transplant decisions. The multiple-choice options demonstrate excellent breadth, including relevant distractors that test nuanced clinical understanding. Clinical outcomes are realistically modeled and highly instructive. However, the prioritization score is slightly reduced due to the flawed recommendation in the final question; while the reasoning to initiate post-HCT FLT3 inhibitor maintenance was correct, the specific agent selection was based on incorrect evidence, representing a flaw in clinical decision-making despite sound overall strategic thinking.

Session-9

Session ID:
LLM

Session-9:
DeepSeek V2

Session ID:
Session Transcript

Session-9:
Phase 1: Initial Presentation & Diagnostic Workup
(Questions 1-5 Focus: Diagnosis, Risk Stratification, Acute Management)

Case Introduction:
Mr. Alvarez, a 58-year-old Hispanic male with a history of hypertension (well-controlled on lisinopril) and type 2 diabetes (HbA1c 7.2%), presents to his PCP with 2 weeks of worsening fatigue, dyspnea on exertion, and gum bleeding. Initial labs show:

WBC: 32 x 10/L (40% blasts)

Hgb: 7.8 g/dL

Platelets: 45 x 10/L

LDH: 850 U/L (elevated)

Creatinine: 1.1 mg/dL (CrCl 68 mL/min)

Question 1/15:
What is the most urgent next step?
A) Transfuse platelets and RBCs
B) Initiate hydroxyurea for cytoreduction
C) Schedule outpatient bone marrow biopsy
D) Administer empiric vancomycin/cefepime
Your Answer: A) Transfuse platelets and RBCs

Analysis:

Why A is partially correct but not the most urgent:

Mr. Alvarez has symptomatic anemia (Hgb <8 g/dL) and thrombocytopenia with bleeding (gum bleeding), so transfusion support is appropriate.

However, his hyperleukocytosis (WBC >30 x 10/L with blasts) poses an immediate risk of leukostasis (capillary occlusion leading to stroke, pulmonary hypoxia, or myocardial infarction). Cytoreduction takes precedence over transfusions in this scenario.

Why B is the best answer:

Hydroxyurea (or leukapheresis if rapidly available) is urgently needed to lower the WBC and mitigate leukostasis risk (per ESMO 2021, Section 5.1). Transfusions can follow once WBC is <30 x 10/L.Why C is incorrect:

While a bone marrow biopsy is diagnostic, it is not urgent until after cytoreduction.

Why D is incorrect:

Empiric antibiotics are unnecessary without fever or signs of infection (e.g., neutropenic fever would require this, but his ANC isnt yet reported).

Clinical Pearl:
"In AML with hyperleukocytosis, think: Blasts are clogging the pipesreduce the pressure first!"
Question 2/15:
Mr. Alvarez receives hydroxyurea 3 g PO and is admitted. His WBC drops to 18 x 10/L by the next morning. A bone marrow biopsy confirms AML with 60% blasts. Flow cytometry shows:

CD34+, CD117+, HLA-DR+

Negative for CD14, CD64

Which WHO 2016/2022 subtype is most likely?
A) AML with myelodysplasia-related changes
B) AML with NPM1 mutation
C) AML with minimal differentiation (FAB M0)
D) Acute promyelocytic leukemia (APL)
Your Answer: C) AML with minimal differentiation (FAB M0)

Analysis:

Why C is correct:

The immunophenotype CD34+, CD117+, HLA-DR+ with absence of myeloid maturation markers (CD14, CD64) is classic for AML with minimal differentiation (FAB M0). This subtype lacks morphological/cytochemical evidence of myeloid differentiation (per WHO 2016/2022 criteria).

Why the others are incorrect:

A) AML with myelodysplasia-related changes: Requires a history of MDS or specific cytogenetic abnormalities (e.g., -7, del(5q)), not yet identified here.B) AML with NPM1 mutation: Typically CD34-negative and shows cytoplasmic NPM1 staining (this patient is CD34+).

D) APL: Would show CD13/CD33 positivity and lack CD34/HLA-DR (and often has bleeding/DIC at presentation).

Key Teaching Point:
"M0 is a shape-shifterit looks undifferentiated but myeloid markers (MPO, CD13/33) may appear later. Always confirm with cytogenetics (e.g., FLT3, NPM1 testing)."
Question 3/15 (Adjusted Difficulty Increased Complexity):
The cytogenetics report returns:

Karyotype: 46,XY,t(8;21)(q22;q22)

Molecular Testing: FLT3-ITD negative, NPM1 negative, c-KIT mutation positive

How would you risk-stratify this patient per ELN 2022?
A) Favorable (due to t(8;21))
B) Intermediate (due to c-KIT mutation)
C) Adverse (due to high blast count)
D) Reclassify as APL

Your Answer: B) Intermediate (due to c-KIT mutation)

Analysis:

Why B is correct (but with nuance!):

t(8;21)(q22;q22) alone confers favorable risk per ELN 2022.

However, co-occurring c-KIT mutation downgrades the risk to intermediate due to its association with higher relapse rates (ELN 2022 Table 3, footnote 2).

Key point: c-KIT mutations are clinically significant in core-binding factor AML (t(8;21) or inv(16)), but not in other subtypes.

Why the others are incorrect:A) Favorable: Ignores the negative prognostic impact of c-KIT.

C) Adverse: t(8;21) never falls into adverse risk, even with c-KIT.

D) APL: t(8;21) is unrelated to APL (which involves PML-RARA).

Clinical Pearl:
"Core-binding factor AML loves to play tricksalways check for c-KIT mutations, which turn a good risk into a maybe risk."
Mr. Alvarez develops fever (38.5C) and hypotension (BP 85/50) during induction. Blood cultures grow E. coli (susceptible to cefepime). His CrCl drops to 30 mL/min.

Which adjustment is most critical for his antimicrobial regimen?
A) Switch to meropenem
B) Reduce cefepime dose to 1 g q12h
C) Add vancomycin for MRSA coverage
D) Continue cefepime at current dose (2 g q8h)
Your Answer: B) Reduce cefepime dose to 1 g q12h

Analysis:

Why B is correct:

Cefepime requires renal dose adjustment when CrCl <50 mL/min (per IDSA guidelines). The standard reduction is to 1 g q12h for CrCl 30-50 mL/min (or 1 g q24h if CrCl 10-30 mL/min).

Mr. Alvarezs CrCl is now 30 mL/min, so dose reduction is mandatory to avoid neurotoxicity (e.g., encephalopathy, seizures).

Why the others are incorrect:

A) Switch to meropenem: Overkill for susceptible E. coli. Reserve carbapenems for ESBL producers or clinical deterioration.

C) Add vancomycin: Unnecessary without MRSA risk factors (e.g., prior colonization, skin/soft tissue infection).

D) Continue current dose: Risk of cefepime accumulation and toxicity.

Clinical Pearl:
"In AML, sepsis kills faster than leukemiabut drug toxicity kills silently. Always recalculate doses when renal function shifts!"
Mr. Alvarez stabilizes and completes "7+3" induction (cytarabine + daunorubicin). Day 14 bone marrow shows 5% blasts (persistent disease).

What is the next best step?

A) Proceed to high-dose cytarabine consolidation
B) Switch to venetoclax + azacitidine
C) Re-induction with FLAG-IDA
D) Refer for allogeneic transplant evaluation
Your Answer: C) Re-induction with FLAG-IDA

Analysis:

Why C is correct:

Day 14 marrow with 5% blasts indicates persistent disease, which is a poor prognostic sign (failure to achieve complete remission after first induction).

FLAG-IDA (fludarabine + cytarabine + G-CSF + idarubicin) is an appropriate salvage regimen in this scenario (per ESMO 2021 guidelines for induction failure).

This approach is more aggressive than repeating 7+3 and may overcome resistance.Why the others are incorrect:

A) Proceed to consolidation: Contraindicated without achieving CR (5% blasts = active disease).

B) Venetoclax+azacitidine: More appropriate for older/unfit patients (this patient is 58 and tolerated 7+3).

D) Transplant referral: While ultimately needed, he must achieve CR first (transplant can't be done with active AML).

Clinical Pearl:
"In AML, day 14 marrow is the first report card - and this patient is failing. Time to switch tactics, not double down."
Mr. Alvarez receives FLAG-IDA. His wife mentions they're struggling with:

Missed appointments due to transportation issues

Anxiety about treatment costs (no secondary insurance)

Difficulty understanding discharge instructions (primary language Spanish)

Question 6/15:
Which intervention addresses the MOST urgent barrier to care?
A) Provide written instructions in Spanish
B) Connect to hospital financial counseling
C) Schedule follow-up near their home
D) Order a palliative care consult
Hint for Question 6/15:

Consider the immediate risks to treatment success:

Missed appointments Risk of losing remission if follow-up is delayed.

Cost concerns May lead to non-adherence to medications (e.g., antifungals, maintenance therapy).

Language barriers Could result in incorrect medication use or missed warning signs (e.g., fever).

Which barrier, if unaddressed, poses the greatest threat to survival in the next 30 days?

Recall: Social determinants of health impact outcomes as much as chemotherapy choices. ESMO guidelines emphasize addressing these early (Section 7.2, "Supportive Care").Your Answer: B) Connect to hospital financial counseling

Analysis:

Why B is correct (but requires refinement):

Financial toxicity directly impacts survival in AML (studies show cost-related non-adherence increases relapse risk by 30%).

However, the most urgent barrier is actually missed appointments (Option C), as they could delay life-saving interventions (e.g., detecting relapse, managing neutropenic fever).

Why C is the best answer:

Transportation barriers immediately threaten treatment continuity. Scheduling follow-up near home (e.g., partnering with a local clinic) ensures he receives critical post-chemotherapy monitoring.Why the others are less urgent:

A) Spanish instructions: Important, but doesnt solve appointment adherence.

D) Palliative care: Prematurehes pursuing curative intent therapy.

Clinical Pearl:
"In AML, social barriers are medical emergencies. Address logistics first (appointments, transportation), then costs, then education."
Question 7/15 (Toxicity Management):
On day 8 of FLAG-IDA, Mr. Alvarez develops:

Grade 3 mucositis (unable to swallow)

Creatinine rise to 2.4 mg/dL (CrCl 22 mL/min)

Potassium: 6.2 mEq/L (peaked T-waves on ECG)

Which intervention is most critical?
A) Start IV calcium gluconate for hyperkalemia
B) Initiate hemodialysis
C) Switch to liposomal daunorubicin
D) Hold further chemotherapy
Hint for Question 7/15:
Prioritize the most immediately life-threatening issue:

Hyperkalemia (K^+ 6.2 with ECG changes) can cause cardiac arrest within minutes.

Mucositis and renal dysfunction are serious but not imminently fatal.

Ask yourself:

What intervention directly addresses the potassium emergency?

Should chemotherapy be continued amid acute organ toxicity?

Refer to ESMO Supportive Care Guidelines (Section 6.3, "Oncologic Emergencies").Your Answer: A) Start IV calcium gluconate for hyperkalemia

Analysis:

Why A is correct:

Hyperkalemia with ECG changes (peaked T-waves) is a medical emergency requiring immediate stabilization with calcium gluconate (1-2 g IV) to protect the myocardium (per ESMO Supportive Care Guidelines 6.3). This should be followed by potassium-lowering strategies (e.g., insulin/glucose, kayexalate).

Key point: Calcium doesnt lower potassium but prevents arrhythmias while other treatments take effect.

Why the others are incorrect:

B) Hemodialysis: Overkill at this stagereserved for refractory hyperkalemia or uremia.

C) Switch to liposomal daunorubicin: Irrelevantthis is a toxicity crisis, not a regimen failure.

D) Hold chemotherapy: Necessary but not the first step when a patient is coding from hyperkalemia.

Clinical Pearl:
"In AML, chemotherapy toxicity can snowball. Remember the ABCs: Airway, Breathing, Cardiac... then address the cancer."
Question 8/15 (Integrated Management):
After stabilizing, Mr. Alvarezs mucositis worsens. He cant tolerate PO medications, including his posaconazole prophylaxis.

What is the best alternative?
A) IV voriconazole
B) IV amphotericin B
C) Switch to posaconazole IV
D) Discontinue prophylaxis until mucositis resolves
Your Answer: C) Switch to posaconazole IV

Analysis:

Why C is correct:

Posaconazole IV provides continuity of prophylaxis with the same spectrum (mold/yeast coverage) as the PO formulation, avoiding disruption in antifungal protection (per IDSA 2016 guidelines).

Mr. Alvarez is at highest risk for invasive fungal infections during mucositis (mucosal barrier breakdown).

Why the others are incorrect:

A) IV voriconazole: Less preferred due to:

Narrower mold coverage (e.g., inferior for mucorales)

Requires therapeutic drug monitoring (more complex than posaconazole)

B) IV amphotericin B: Toxic (nephrotoxicity, electrolyte wasting) and unnecessary for prophylaxis.

D) Discontinue prophylaxis: Dangeroushigh risk of breakthrough aspergillosis during neutropenia.

Clinical Pearl:
"Mucositis turns the gut into a highway for pathogens. Never drop prophylaxisjust change the route!"
Question 9/15 (Psychosocial Complication):
The hospital social worker reports Mr. Alvarezs wife is overwhelmed. She admits:

Missed 2 doses of his oral antibiotics because the schedule was confusing

Their electricity was shut off due to unpaid bills

What is the next best action?
A) Simplify the medication regimen to once-daily dosing
B) Transfer care to a closer hospital
C) Admit him for IV antibiotics until social issues resolve
D) Contact adult protective services
Hint for Question 9/15:

When considering psychosocial barriers to care:

Immediate safety comes first (e.g., medication errors, living conditions)

Feasibility of solutions matters (what can be implemented TODAY)

Patient autonomy should be preserved when possible

Key considerations for this scenario:

The patient is on oral antibiotics but doses are being missed

The home environment may not be safe for recovery (no electricity)

The caregiver is overwhelmed but willing to engage

Ask yourself:Which option both addresses the immediate medication

Your Answer: C) Admit him for IV antibiotics until social issues resolve

Analysis:

Why C is correct:

This is a patient safety emergency. Missed antibiotics + neutropenia = high risk for fatal sepsis. With no electricity at home, he cannot safely store medications or maintain hygiene.

IV antibiotics in-hospital bridge the gap while social work:

Restores electricity (via hospital emergency funds)

Simplifies his medication regimen (e.g., once-daily IV ceftriaxone)

ESMO Supportive Care Guidelines (Section 7.1) emphasize "hospitalization for social toxicity" when home is unsafe.Why the others are insufficient:

A) Simplify dosing: Helpful but doesn't fix the electricity crisis (spoiled meds, no refrigeration).

B) Transfer care: Delays treatment and may not solve root causes.

D) Adult protective services: Overly punitivethe wife is trying but needs support.

Clinical Pearl:
"You can't cure leukemia if the patient can't charge their phone to get appointment reminders. Sometimes the best 'chemo' is a social

worker."
Mr. Alvarez achieves CR after FLAG-IDA (MRD 0.1% by flow). His sister is a 6/6 HLA match.

What is the next step?
A) Proceed directly to allogeneic transplant
B) 2 cycles of high-dose cytarabine consolidation
C) Venetoclax maintenance for 1 year
D) Enroll in a CAR-T cell trial
Your Answer: A) Proceed directly to allogeneic transplant

Analysis:

Why A is correct:

Mr. Alvarez has persistent MRD (0.1%) post-salvage therapy, indicating high relapse risk. With a matched sibling donor, proceeding directly to allogeneic transplant is the standard of care (per ELN 2022, Section 8.2).

Key evidence: The BMT CTN 0901 trial showed superior survival with upfront transplant in MRD+ patients versus consolidation chemo.Why the others are incorrect:

B) High-dose cytarabine: Would delay transplant and risk relapse in this MRD+ patient.

C) Venetoclax maintenance: Not proven in core-binding factor AML (and irrelevant with donor available).

D) CAR-T trial: Experimental in AML (no approved products) and wastes his curative transplant option.

Clinical Pearl:
"In AML, MRD is the clock ticking toward relapseand transplant is the only hammer that can stop it."
Case Update:
Mr. Alvarez undergoes reduced-intensity conditioning (RIC) transplant. On day +45, he develops:

Maculopapular rash on palms/soles (30% BSA)

Bilirubin: 3.2 mg/dL

Watery diarrhea (1.2 L/day)Question 11/15:
What is the most likely diagnosis?
A) Engraftment syndrome
B) Acute GVHD (grade III)
C) CMV colitis
D) Drug reaction (post-transplant medications)
Hint for Question 11/15:

Key Clues to Diagnose Post-Transplant Complications:
Timing (Day +45):

Acute GVHD typically occurs after engraftment (usually day +21 to +100)

Engraftment syndrome occurs during neutrophil recovery (earlier, around day +10-20)

Clinical Triad:

Rash (palms/soles first) Classic for GVHD

Diarrhea >1 L/day Lower GI GVHD hallmark

Hyperbilirubinemia Hepatic GVHD

Differential Ruled Out:

CMV colitis would show fever/ulcers on endoscopy, not this rash pattern

Drug reactions rarely cause all three findings together

Think: "When you see skin + gut + liver after transplant, think GVHD until proven otherwise."

Now select your answer (A, B, C, or D).Your Answer: B) Acute GVHD (grade III)

Analysis:

Why B is correct:

Mr. Alvarez meets NIH consensus criteria for acute GVHD (Grade III) with:

Skin: Maculopapular rash (30% BSA)

Liver: Bilirubin 3.2 mg/dL

GI: Diarrhea >1 L/day
The timing (day +45) is classic for GVHD onset post-engraftment.

Why the others are incorrect:

A) Engraftment syndrome: Occurs earlier (during neutrophil recovery) and typically presents with fever/capillary leak, not cholestatic jaundice.

C) CMV colitis: Would show fever + PCR positivity, and rash is uncommon.

D) Drug reaction: Unlikely to simultaneously affect skin, liver, and GI tract in this pattern.

Clinical Pearl:
"GVHD is the price of the graft-versus-leukemia effect. Remember: Skin Gut Liver is the classic march of acute GVHD."

Question 12/15 (GVHD Management):
First-line therapy for this patients condition is:
A) High-dose methylprednisolone 2 mg/kg IV
B) Ruxolitinib 5 mg PO BID
C) IVIG 1 g/kg weekly
D) Infliximab 10 mg/kg IV

Select your answer (A, B, C, or D)
Your Answer: A) High-dose methylprednisolone 2 mg/kg IV

Analysis:

Why A is correct:

High-dose steroids (methylprednisolone 2 mg/kg/day) remain first-line therapy for acute GVHD per NIH consensus guidelines (2014)

and EBMT recommendations.

This patient has grade III GVHD (multi-organ involvement), requiring aggressive immunosuppression.

Why the others are incorrect:B) Ruxolitinib: Approved for steroid-refractory GVHD (not first-line).

C) IVIG: Used for infection prophylaxis or antibody-mediated rejection, not GVHD.

D) Infliximab: Reserved for steroid-refractory lower GI GVHD (and increases infection risk).

Clinical Pearl:
"In GVHD, steroids are the fire extinguisherbut remember to watch for fungal infections (the sparks that flare up after putting out the fire)."
At day +100 post-transplant, Mr. Alvarezs MRD is negative, but his GVHD requires ongoing prednisone 20 mg/day.

What is the best strategy to prevent relapse?
A) Start azacitidine maintenance
B) Initiate donor lymphocyte infusion (DLI)
C) Continue immunosuppression and monitor
D) Add venetoclax

Select your answer (A, B, C, or D).
Hint for Question 13/15:

Key Considerations for Post-Transplant Relapse Prevention:
GVHD Status Matters:

Active GVHD (even if requiring steroids) provides graft-versus-leukemia (GVL) effect Reducing immunosuppression risks worsening GVHD

But persistent high-dose steroids increase infection/relapse risks

MRD Status:

Currently negative No need for *pre

Hint for Question 13/15:

Key Considerations for Post-Transplant Relapse Prevention:
**

A
Feedback for Question 13/15:

Your Answer: A) Start azacitidine maintenanceAnalysis:

Why A is correct:

Azacitidine maintenance is recommended for high-risk AML (like Mr. Alvarez's initial induction failure) post-transplant, especially when GVHD limits immunosuppression tapering (per EBMT 2022 guidelines).

It balances:

Relapse prevention (hypomethylating effect)

GVHD mitigation (lower toxicity vs. DLI)

The RELAZA trial showed 50% 1-year relapse-free survival with azacitidine in similar patients.

Why the others are incorrect:

B) DLI: Too risky with active GVHD (could exacerbate to grade IV).C) Monitor only: Unacceptable for his high-risk history (prior induction failure).

D) Venetoclax: No evidence in post-transplant maintenance (and may worsen cytopenias).

Clinical Pearl:
"In transplant, GVHD is both a curse and a blessing. Azacitidine lets us thread the needlekeeping some GVL effect while preventing relapse."
At 1-year post-transplant, Mr. Alvarez remains in remission but develops:

Fatigue, dry eyes/mouth

Mild liver fibrosis (Elastography: 7.2 kPa)

New-onset diabetes (HbA1c 8.1%)

What is the most likely explanation?
A) Chronic GVHD
B) Iron overload
C) Metabolic syndrome
D) Viral hepatitis reactivation

Select your answer (A, B, C, or D).
Hint for Question 14/15:
Clues to Chronic GVHD:

Sicca symptoms (dry eyes/mouth) Classic for cGVHD

Liver fibrosis Late hepatic GVHD manifestation

New diabetes Steroid-induced or pancreatic GVHD

Ruled Out:

Iron overload Would show high ferritin before fibrosis

Metabolic syndrome Doesn't explain sicca symptoms

Viral hepatitis Should have elevated transaminases

Think: "When 3 unrelated organs slowly fail after transplant, suspect the chameleonchronic GVHD."Hint for Question 14/15:

Key Clues to Diagnose Chronic GVHD:
Multi-organ involvement pattern is classic:

Ocular/oral dryness (sicca syndrome) Most common cGVHD manifestation (70% of cases)

Hepatic fibrosis Late-stage liver GVHD (cholestatic pattern)

New-onset diabetes Possible pancreatic GVHD or steroid effect

Timing fits cGVHD:

Develops >100 days post-transplant (this patient is at 1 year)

Differential Exclusion:

B) Iron overload: Would require multiple transfusions (not mentioned) and shows high ferritin before fibrosisNew-onset diabetes Possible pancreatic GVHD or steroid effect

Timing fits cGVHD:

Develops >100 days post-transplant (this patient is at 1 year)

Differential Exclusion:

B) Iron overload: Would require multiple transfusions (not mentioned) and shows high ferritin before fibrosis

C) Metabolic syndrome: Doesnt explain sicca symptoms or liver fibrosis

D) Viral hepatitis: Would show ALT/AST spikes (not mentioned)Clinical Tip:
"Chronic GVHD mimics autoimmune diseases. Think scleroderma + Sjgrens + biliary cirrhosis in one patient."Your Answer: C) Metabolic syndrome

Analysis:

Why C is incorrect (but has a kernel of truth):

While steroid-induced diabetes (HbA1c 8.1%) could fit metabolic syndrome, it doesnt explain the other symptoms:

Dry eyes/mouth (sicca syndrome) Pathognomonic for chronic GVHD

Liver fibrosis Classic hepatic cGVHD (even without elevated transaminases)

Why A is correct:This is a textbook chronic GVHD presentation (per NIH 2014 criteria):

Sicca symptoms (ocular/oral) Most common cGVHD manifestation

Hepatic involvement Fibrosis without viral/iron overload

Endocrine dysfunction Pancreatic GVHD or steroid effect

Clinical Pearl:
"Chronic GVHD is the great imitatorit can look like diabetes, cirrhosis, or lupus. Always connect the dots!"
Question 15/15 (Final Challenge Survivorship Care):
For Mr. Alvarezs chronic GVHD management, which intervention is MOST evidence-based?
A) Add hydroxychloroquine for sicca symptoms
B) Switch prednisone to ruxolitinib + start pilocarpine
C) Initiate FXR agonists for liver fibrosis
D) Refer for autologous stem cell boost

Select your answer (A, B, C, or D).
Hint for Question 15/15:
Chronic GVHD Treatment Principles:

First-line: Systemic immunosuppression (steroids calcineurin inhibitors)

Steroid-refractory: JAK inhibitors (ruxolitinib) approved based on REACH trials

Organ-specific:

Sicca Pilocarpine (stimulates saliva)

Liver fibrosis Ursodiol (not FXR agonists)

Key Trial: REACH3 showed ruxolitinibs superiority over best available therapy for steroid-refractory cGVHD.Feedback for Question 15/15:

Your Answer: B) Switch prednisone to ruxolitinib + start pilocarpine

Analysis:

Why B is correct:

Ruxolitinib is now first-line for steroid-dependent chronic GVHD (per REACH3 trial/NCCN guidelines), showing:

Higher response rates (50% vs. 26% with standard therapy)

Better steroid taper capabilityPilocarpine is guideline-recommended for sicca symptoms (ASCO cGVHD guideline 2020).

Why the others are incorrect:

A) Hydroxychloroquine: No proven benefit in cGVHD (only case reports).

C) FXR agonists: Not used in GVHD (ursodiol is for prevention, not treatment).

D) Autologous boost: Nonsensicalautologous cells have no GVL effect.

Clinical Pearl:
"Modern GVHD care is about precision: JAK inhibitors for immune modulation, organ-specific support, and relentless steroid-sparing."

Session ID:
Assessment Domain

Session-9:
Rationale

Session ID:
Instructional Design and Pedagogical

Session-9:
The session demonstrates excellent instructional design with expertly scaffolded progression from AML diagnosis through complex post-transplant survivorship care. The embedded feedback is outstanding, featuring detailed, guideline-anchored rationales with valuable clinical pearls citing current evidence (ELN 2022, ESMO, NCCN, REACH3 trial). Each question builds logically on the previous, creating seamless complexity progression. Instructions are exceptionally clear and intuitive. The primary weakness is the

absence of explicitly stated learning objectives at the session's beginning, though educational goals are clearly implied through the comprehensive content structure.

Session ID:
Clinical Authenticity and Content Fidelity

Session-9:
The session presents a highly authentic clinical narrative following a realistic AML patient journey from diagnosis through post-transplant complications. Most therapeutic recommendations align well with current guidelines. However, there are some concerns about pharmacotherapeutic accuracy. The session cites the "RELAZA trial" showing "50% 1-year relapse-free survival with azacitidine" for post-transplant maintenance, but this appears to be an inaccurate or misrepresented trial reference. While azacitidine maintenance post-transplant has some evidence, the specific trial citation and outcomes mentioned may not be accurate. Additionally, some of the guideline interpretations, while plausible, represent aggressive interpretations of emerging evidence rather than established standards. The case remains strictly within the AML/transplant domain without entanglement issues. While day 14 BMA is important for prognosis, not used as a decision checkpoint for second-line chemotherapy, as well as there was a nearly CR with only 5 % plasts. The case proceeds to second-line chemotherapy depending on day 14 BMA with 5 % plasts, which is incorrect.

Session ID:
Modeled Clinical Reasoning

Session-9:
The simulation excellently models expert-level clinical reasoning throughout the complex AML management continuum. Each clinical challenge is expertly framed with appropriate complexity, from initial hyperleukocytosis management through chronic GVHD treatment. The multiple-choice options demonstrate excellent breadth, including relevant distractors that test nuanced clinical understanding. Decision-making prioritization is consistently appropriate, such as emphasizing cytoreduction before transfusion in hyperleukocytosis and recognizing hyperkalemia as an immediate cardiac emergency. Clinical outcomes are realistically modeled and highly instructive, reinforcing key learning points about AML complexity and post-transplant complications.

Session-10

Session ID:
LLM

Session-10:
DeepSeek V2

Session ID:
Session Transcript

Session-10:
Case:

Chief Complaint: Fatigue, bruising, recurrent infections 3 weeks

Labs: WBC 38,000/L (40% blasts), Hb 7.8 g/dL, platelets 22,000/L

PMH: Hypertension, obesity (BMI 32)

Social: Limited healthcare access (rural area)

Which test is required to confirm AML per WHO 2022 criteria?
A) Peripheral blood flow cytometry
B) Bone marrow biopsy with immunophenotyping/cytogenetics (Correct)
C) FLT3-ITD PCR alone
D) CT scan of chest/abdomen
Answer: B) Bone marrow biopsy with immunophenotyping/cytogenetics

Key Teaching Points:
WHO 2022 Diagnostic Criteria:

AML is confirmed by 20% blasts in bone marrow or definitive genetic markers (e.g., RUNX1::RUNX1T1).

Peripheral blood flow cytometry (Option A) cannot assess blast percentage in marrow or detect cytogenetic abnormalities.

Why Not Other Options?

FLT3-ITD PCR (C): Prognostic but not diagnostic.

CT scan (D): Used for infection/inflammation workup, not AML diagnosis.

Next Steps in This Case:

Await marrow results to assess:

Cytogenetics (e.g., complex karyotype?)

Molecular markers (NPM1, FLT3-ITD, IDH1/2).
Marrow Results Return:

65% blasts, Auer rods present

Cytogenetics: Normal karyotype

Molecular: NPM1+ (no FLT3-ITD), IDH2 R140Q+

How would you classify this patient per ELN 2022?
A) Favorable
B) Intermediate
C) Adverse
ELN 2022 Risk Stratification for This Patient:
Key Mutations Present:

NPM1+ (favorable marker)

IDH2 R140Q+ (neutraldoes not change risk category)

No FLT3-ITD (avoids adverse risk from FLT3-ITDhigh)

Normal karyotype (no adverse cytogenetics, e.g., -7, complex karyotype)

ELN 2022 Rules:

Favorable Risk (A):

NPM1+ without FLT3-ITD or

CBF rearrangements (e.g., RUNX1::RUNX1T1)

This patient qualifies for Favorable risk.

Why Not Intermediate (B)?

Intermediate applies to:

Wild-type NPM1 and FLT3-ITDnegative, or

IDH2 mutations without NPM1/FLT3-ITD.

Clinical Implications:

Favorable risk = Lower-intensity consolidation (e.g., high-dose cytarabine 34 cycles)

No need for upfront transplant unless MRD persists.
ase Update:

Same 58-year-old female, ELN Favorable Risk (NPM1+/FLT3-ITD, IDH2 R140Q+)

ECOG 1 (fully active, symptomatic but ambulatory)

Organ Function:

LVEF 55% (normal cardiac function)

CrCl 68 mL/min (mild renal impairment)

Question 3/15 (Treatment):
What is the preferred induction regimen for this patient?
A) 7+3 (Cytarabine + Daunorubicin)
B) Venetoclax + Azacitidine
C) CPX-351 (Vyxeos)
D) Gilteritinib monotherapy
Answer: A) 7+3 (Cytarabine + Daunorubicin)

Key Teaching Points:
Rationale for 7+3 in This Patient:

Favorable-risk AML (NPM1+/FLT3-ITD): Intensive chemo maximizes cure potential.

Fit status (ECOG 1, CrCl 68 mL/min): Tolerates anthracycline-based therapy.

IDH2 R140Q+: Irrelevant for induction but may guide post-remission strategies (e.g., enasidenib if MRD+).

Why Not Other Options?

B (Venetoclax + Azacitidine): Reserved for unfit patients (lower CR rates in fit favorable-risk).

C (CPX-351): For adverse-risk/t-AML (per phase III trial data).

D (Gilteritinib): FLT3-specificno role here.

Dosing Considerations:

Daunorubicin 60 mg/m 3 days (90 mg/m if <60 years, but this patient is 58).

Cytarabine 100200 mg/m CI 7 days (adjust for renal impairment if CrCl <60).
During Induction, She Develops:

Fever (38.5C) + neutropenia (ANC 200/L)

Hypotension (BP 85/50)

No obvious infection source

What is the next critical action?
A) Start empiric piperacillin-tazobactam
B) Fluids + blood cultures immediate vancomycin + cefepime (Correct)
C) Wait for CRP results
D) Add posaconazole prophylaxis
Answer: B) Fluids + blood cultures immediate vancomycin + cefepime

Key Teaching Points:
Why This is Emergent:

Neutropenic sepsis mortality approaches 50% if antibiotics are delayed >1 hour (IDSA 2024).

Hypotension = grade 4 life-threatening complication (requires ICU-level care).

Drug-Specific Rationale:

Cefepime: Covers Pseudomonas and Gram-negatives (AML patients often colonized).

Vancomycin: Essential for MRSA/mucosal barrier injury (e.g., Streptococcus mitis bacteremia).

Fluids: Neutropenic sepsis causes distributive shock (NS boluses first-line).

Why Not Other Options?

A (Piperacillin-tazobactam alone): Misses MRSA and lacks rapid Pseudomonas coverage like cefepime.

C (Wait for CRP): CRP rises in 6+ hourssepsis is clinical diagnosis.

D (Posaconazole): Antifungals are added only after 72h of persistent fever.
During Induction Day 5:

She develops creatinine rise (1.8 3.2 mg/dL) and oliguria.

Urinalysis: Muddy brown casts.

Recent medications: Cytarabine, vancomycin, cefepime.

What is the most likely diagnosis?
A) Tumor lysis syndrome
B) Acute tubular necrosis (ATN) from vancomycin (Correct)
C) Daunorubicin cardiotoxicity
D) IDH inhibitor-associated renal injury
Key Teaching Points:
Why ATN?

Muddy brown casts = Pathognomonic for ATN (from tubular cell sloughing).

Vancomycin toxicity: Common with troughs >1520 mg/L or prolonged use.

Timing fits: AKI developed after 5 days of vancomycin (typical onset 37 days).

Management Steps:

Immediate: Hold vancomycin, switch to linezolid (if MRSA coverage still needed).

Fluid resuscitation: NS boluses (unless volume overloaded).

Monitor: Urine output, Cr trend, electrolytes.

Why Not Other Options?

A (Tumor lysis): Would present earlier (Days 13) with K+/PO4/Ca2+.

C (Daunorubicin): Causes cardiotoxicity (e.g., LVEF), not AKI.

D (IDH inhibitor): She hasnt received enasidenib yet (induction phase).

Case Update:

She achieves CR after 7+3 (MRD 0.05% by flow cytometry).

Recovered renal function (Cr 1.2 mg/dL).

Question 6/15 (Consolidation):
What is the next best step for this NPM1+/FLT3-ITD patient?
A) High-dose cytarabine (HiDAC) 34 cycles (Correct)
B) Allogeneic HSCT
C) Venetoclax + azacitidine maintenance
D) Enasidenib monotherapy
Answer: A) High-dose cytarabine (HiDAC) 34 cycles

Key Teaching Points:
ELN 2022 Favorable-Risk Standard:

NPM1+/FLT3-ITD with MRD <0.1%: HiDAC is category 1 (NCCN 2024).

Dosing:

Cytarabine 3 g/m q12h on Days 1, 3, 5 (adjust to 1 g/m if age >60 or CrCl <60 mL/min).

Why Not Other Options?

B (HSCT): Reserved for persistent MRD or adverse-risk disease.

C (Venetoclax + azacitidine): Unfit patients only (shes ECOG 1).

D (Enasidenib): IDH2 inhibitors are for R/R AML, not consolidation.

Monitoring During HiDAC:

Neurotoxicity: Assess for cerebellar signs (dysmetria, slurred speech) before each dose.

MRD Tracking: Repeat after cycle 2if rises, consider trial or transplant.
After Completing HiDAC:

MRD undetectable (0.01% by NPM1 RT-qPCR)

IDH2 R140Q+ persists

What is the next step?
A) Observation with q3mo MRD monitoring (Correct)
B) Start enasidenib maintenance
C) Proceed to allogeneic HSCT
D) Venetoclax + azacitidine indefinitely
nswer: A) Observation with q3mo MRD monitoring

Key Teaching Points:
Why Observation?

MRD negativity (0.01%) after HiDAC = optimal outcome (5-yr survival ~70%).

IDH2 R140Q+ alone is not actionablemay represent clonal hematopoiesis.

Evidence Against Intervention:

Enasidenib (B): No survival benefit in CR1 (used for R/R disease).

HSCT (C): Unjustified mortality risk (~20%) for favorable-risk AML.

Venetoclax (D): No role in MRD maintenance (NCCN category 3).

Monitoring Protocol:

NPM1 RT-qPCR every 3 months (ELN 2022: intervene if >1-log rise).

IDH2 testing only at relapse (to guide targeted therapy).
18 Months Later:

MRD rises to 0.8% (NPM1 RT-qPCR)

No cytopenias/blasts in marrow

IDH2 R140Q+ persists

What is the next step?
A) Preemptive gilteritinib
B) Azacitidine + venetoclax
C) Enasidenib monotherapy (Correcttargets IDH2)
D) Immediate allogeneic HSCT
Answer: C) Enasidenib monotherapy

Key Teaching Points:
Rationale for Enasidenib:

Targeted action: IDH2 R140Q inhibition reverses epigenetic blockade (differentiation therapy).

Evidence: IDHENTIFY trial showed median OS 9.3 months in R/R IDH2+ AML.

Advantage over chemo: Better tolerated (no myelosuppression).

Monitoring & Toxicity Management:

Differentiation syndrome (1525% risk): Treat with dexamethasone 10mg IV q12h + hold enasidenib if severe.

MRD tracking: Repeat NPM1 RT-qPCR in 4 weeks (goal: MRD to <0.1%).

Why Not Other Options?

A (Gilteritinib): FLT3-ITD patients derive no benefit.

B (Venetoclax + azacitidine): Reserved for hematologic relapse or unfit patients.

D (HSCT): Requires CR2 firstenasidenib can bridge to transplant.

After 2 Months of Enasidenib:

MRD rises to 5% (NPM1+), blasts reappear in marrow (12%)

ECOG 1, CrCl 55 mL/min

What is the next best step?
A) Venetoclax + azacitidine (Correctstandard salvage for fit patients)
B) Rechallenge with 7+3
C) Add gilteritinib to enasidenib
D) Best supportive care
Key Teaching Points:
Rationale for Venetoclax + Azacitidine:

VIALE-A trial: CR/CRi rate of 66.4% in R/R AML (vs 28.3% with azacitidine alone).

Mechanistic synergy: Azacitidine upregulates BCL-2 dependence enhances venetoclax lethality.

IDH2+ context: Still effective despite enasidenib failure (different resistance pathways).

Practical Considerations:

Dosing: Venetoclax ramp-up to 400 mg daily (reduce to 100 mg if strong CYP3A inhibitors used).

Supportive care:

TLS prophylaxis: Allopurinol + hydration (even with 12% blasts).

Antifungal prophylaxis: Posaconazole (if not on venetoclax).

Why Not Other Options?

B (7+3 rechallenge): CR rates <30% in relapse (per UK NCRI AML16).

C (Gilteritinib): No FLT3 mutation = no benefit.

D (Supportive care): Patient remains transplant-eligible (ECOG 1).
After 2 Cycles of Venetoclax + Azacitidine:

Achieves CR2 (MRD 0.02% by flow)

Sibling donor identified (10/10 HLA match)

What is the next step?
A) Proceed to allogeneic HSCT (Correct)
B) Continue venetoclax + azacitidine indefinitely
C) Enasidenib maintenance
D) CAR-T cell therapy
Key Teaching Points:
Why HSCT is Standard in CR2:

Only curative option for relapsed AML (3-year DFS ~4050% with MRD <0.1%).

Sibling 10/10 match = preferred donor (lower GVHD risk vs MUD/haplo).

Conditioning Considerations:

Reduced-intensity (RIC): Fludarabine + melphalan (age 58, prior chemo exposure).

IDH2+ angle: Enasidenib may be used post-HSCT if MRD reappears (investigational).

Why Not Other Options?

B (Venetoclax indefinitely): Non-curative (median PFS 912 months in R/R AML).

C (Enasidenib maintenance): No proven benefit post-CR2 (unlike FLT3 inhibitors).

D (CAR-T): Not an established therapy for AML (unlike ALL).
Day +100 Post-Transplant:

Full donor chimerism

MRD undetectable (NPM1 RT-qPCR negative)

Developing mild chronic GVHD (skin stage 1)

What is the next step?
A) Start prednisone 1 mg/kg + continue tacrolimus (CorrectGVHD treatment)
B) Begin enasidenib prophylaxis
C) Donor lymphocyte infusion (DLI)
D) Stop all immunosuppressants
Answer: A) Start prednisone 1 mg/kg + continue tacrolimus

Key Teaching Points:
Rationale for Immunosuppression:

Mild chronic GVHD (skin stage 1) requires systemic therapy to prevent progression (per NIH criteria).

Prednisone + tacrolimus is first-line to control inflammation while preserving graft-vs-leukemia (GvL) effect.

Dosing & Monitoring:

Prednisone taper: Begin after 2 weeks of response (goal: 10 mg/day by 3 months).

Tacrolimus levels: Maintain 510 ng/mL (prevents GVHD flare).

Why Not Other Options?

B (Enasidenib): No evidence for prophylaxis (IDH2 inhibitors used at relapse).

C (DLI): Only if MRD reappears without GVHD (high risk of exacerbation here).

D (Stop immunosuppressants): Would trigger life-threatening GVHD flare.
1 Year Post-HSCT:

Off immunosuppressants 3 months

MRD remains negative

New fatigue, Hb 9.8 g/dL (was 12 pre-HSCT)

What is the most likely cause of anemia?
A) Iron deficiency (Correctcheck ferritin)
B) Disease relapse
C) Calcineurin inhibitor toxicity
D) Parvovirus B19 reactivation
Answer: A) Iron deficiency

Key Teaching Points:
Why Iron Deficiency?

HSCT patients lose 12 g iron/year from frequent phlebotomy (J Clin Oncol 2023).

Asymptomatic gut GVHD impairs absorption (even without diarrhea).

Diagnosis: Ferritin <30 ng/mL + TIBC (transferrin saturation <20%).

Management:

IV iron (ferric carboxymaltose): Bypasses gut absorption issues.

Avoid oral iron: Poor efficacy if GVHD present.

Why Not Other Options?

B (Relapse): Unlikely without MRD+ or blasts (NPM1 PCR negative).

C (TMA): Requires schistocytes + LDH (calcineurin inhibitors typically cause earlier toxicity).

D (Parvovirus): Presents with reticulocytopenia (check if Hb drops abruptly).
18 Months Post-HSCT:

Fatigue resolves with iron therapy

New dyspnea on exertion, O sat 92% RA

CT chest: Ground-glass opacities

No fever/infection markers

What is the most likely diagnosis?
A) Bronchiolitis obliterans syndrome (BOS) (Correctlate GVHD)
B) Pneumocystis jirovecii pneumonia
C) Idiopathic pneumonia syndrome
D) COVID-19 pneumonia
Answer: A) Bronchiolitis obliterans syndrome (BOS)

Key Teaching Points:
Why BOS?

Classic presentation: Dyspnea + hypoxemia + irreversible airflow obstruction (FEV1/FVC <0.7).

GVHD association: 2030% of chronic GVHD patients develop BOS (often with skin/GI involvement).

Imaging: Air trapping (expiratory CT) ground-glass opacities.

Diagnostic Workup:

PFTs: FEV1, RV (obstructive pattern)gold standard.

Bronchoscopy: Rule out infection (e.g., CMV, fungal).

Management:

First-line: Fluticasone/azithromycin/montelukast (FAM regimen) + systemic steroids.

Refractory cases: Ruxolitinib (JAK inhibition reduces inflammation).

Why Not Other Options?

B (PJP): Unlikely without fever + likely on prophylaxis (TMP-SMX).

C (IPS): Typically occurs early post-HSCT (within 120 days).

D (COVID-19): Requires PCR confirmation (hypoxemia usually more severe).
3 Years Post-HSCT:

Asymptomatic

Routine colonoscopy: Tubulovillous adenoma with high-grade dysplasia

No GVHD or immunosuppressants 2 years

What is the next step?
A) Surgical resection (CorrectHSCT survivors need aggressive management)
B) Annual surveillance colonoscopy
C) Start aspirin 81 mg daily
D) Topical therapy with 5-FU
Key Teaching Points:
Why Immediate Resection?

HSCT survivors have 411x higher colorectal cancer risk (per CIBMTR).

High-grade dysplasia has 40% progression risk to adenocarcinoma (vs 5% for low-grade).

Immunosuppression history impairs tumor surveillance faster malignant transformation.

Surgical Considerations:

Lymph node sampling required (hidden malignancy risk).

Post-op surveillance: Colonoscopy q612 months (vs q35 years in general population).

Why Not Other Options?

B (Surveillance): Unacceptable for high-grade dysplasia in immunocompromised hosts.

C (Aspirin): Only for primary prevention in low-risk patients.

D (Topical 5-FU): For actinic keratosis/superficial skin cancers.

Broader Survivorship Plan:

Annual: Skin exams, thyroid US, mammography/PAP smears (all elevated post-HSCT).

Lifestyle counseling: Smoking cessation, UV avoidance (synergistic risks).
5 Years Post-HSCT:

No evidence of disease

Mild chronic GVHD (oral lichen planus)

Asks about long-term monitoring

What is the most important recommendation?
A) Lifelong annual NPM1 RT-qPCR (Correctlate relapses occur)
B) Discontinue all monitoring (cured)
C) BRCA testing (unrelated to AML)
D) Cardiac MRI (for anthracycline toxicity)
Key Teaching Points:
Why Lifelong MRD Monitoring?

NPM1+ AML has lifelong relapse risk (15% of relapses occur >3 years post-HSCT).

Early detection allows preemptive DLI or targeted therapy (e.g., enasidenib for IDH2+).

Survivorship Protocol:

Annual: NPM1 RT-qPCR, CBC, comprehensive metabolic panel.

Every 35 years: Bone marrow biopsy if MRD rises.

Secondary cancer screening: Colonoscopy (q12y), skin exams (annually).

Why Not Other Options?

B (Discontinue monitoring): 50% of late relapses are asymptomatic until advanced.

C (BRCA testing): Irrelevant without personal/family history of BRCA-related cancers.

D (Cardiac MRI): Only if HF symptoms develop (daunorubicin toxicity usually appears earlier).

Session ID:
Assessment Domain

Session-10:
Rationale

Session ID:
Instructional Design and Pedagogical

Session-10:

The session demonstrates excellent instructional design with expertly scaffolded progression spanning the entire AML journey from diagnosis through 5-year post-transplant survivorship. The embedded feedback is outstanding, featuring detailed, evidence-based rationales with specific trial citations (VIALE-A, IDHENTIFY) and guideline references (ELN 2022, NCCN 2024, NIH criteria). Each question builds logically on the previous, creating seamless complexity progression through acute management, consolidation, relapse, transplant, and long-term complications. Instructions are exceptionally clear and intuitive. The primary weakness is the absence of explicitly stated learning objectives at the session's beginning, though educational goals are clearly implied through the comprehensive survivorship-focused structure.

Session ID:

Clinical Authenticity and Content Fidelity

Session-10:

The session presents a highly authentic clinical narrative that perfectly mirrors real-world AML patient management from diagnosis through long-term survivorship. The therapeutic recommendations are consistently aligned with current guidelines (ELN 2022, NCCN, IDSA). The pharmacotherapeutic accuracy is generally excellent, with appropriate drug selections throughout: 7+3 induction for favorable-risk AML, empiric antibiotics for neutropenic fever, HiDAC consolidation, enasidenib for IDH2+ molecular relapse, and venetoclax + azacitidine for hematologic relapse. The transition to transplant in CR2 and subsequent GVHD management are handled appropriately. Some minor concerns exist around specific trial data citations, but the overall treatment approach remains sound and evidence-based. The case remains strictly within the AML domain without any entanglement issues.

Session ID:

Modeled Clinical Reasoning

Session-10:

The simulation excellently models expert-level clinical reasoning throughout the comprehensive AML management continuum. Each clinical challenge is expertly framed with appropriate complexity, from initial WHO diagnostic criteria through complex post-transplant complications like BOS and secondary malignancies. The multiple-choice options demonstrate excellent breadth, including relevant distractors that test nuanced clinical understanding (e.g., differentiating ATN from other causes of AKI). Decision-making prioritization is consistently appropriate, such as immediate antibiotic administration for neutropenic sepsis and aggressive management of high-grade dysplasia in immunocompromised survivors. Clinical outcomes are realistically modeled and highly instructive, particularly the long-term survivorship challenges that are often overlooked in AML education.

Session-11

Session ID:
LLM

Session-11:
Claude 3.7 Sonnet

Session ID:
Session Transcript

Session-11:
Based on Maria's cytogenetic and molecular findings, which risk category does she fall into according to the 2022 ELN criteria?
A) Favorable risk
B) Intermediate risk
C) Adverse risk
D) Very high risk
E) Unclassifiable
Correct Answer: B) Intermediate risk
Explanation:
Maria has mutated NPM1 (typically favorable) but also has FLT3-ITD with a high allelic ratio (0.7). According to the 2022 ELN criteria, NPM1-mutated AML with FLT3-ITD with high allelic ratio (>0.5) is classified as intermediate risk. The favorable prognostic impact of NPM1 mutation is offset by the high FLT3-ITD ratio, moving her from favorable to intermediate risk category. This risk stratification is crucial for treatment planning and determining transplant eligibility.
Which of the following is most appropriate for tumor lysis syndrome prophylaxis in Maria given her laboratory values?
A) Allopurinol only
B) Allopurinol plus IV hydration
C) Rasburicase only
D) Rasburicase plus IV hydration
E) No prophylaxis needed at this time
Correct Answer: B) Allopurinol plus IV hydration
Explanation:
Maria has multiple risk factors for tumor lysis syndrome (TLS): elevated WBC count (32.4 10^9/L with 45% blasts), elevated uric acid (8.2 mg/dL), slightly elevated phosphorus, and elevated LDH. However, her renal function is only mildly impaired (Cr 1.2 mg/dL) and her potassium is normal. This profile indicates moderate TLS risk. Allopurinol (to prevent uric acid formation) plus aggressive IV hydration is appropriate. Rasburicase would be indicated if she had severe hyperuricemia, acute kidney injury, or clinical TLS
Which targeted therapy would be most appropriate to include in Maria's treatment regimen based on her molecular profile?
A) Midostaurin
B) Enasidenib
C) Ivosidenib
D) Venetoclax
E) Gilteritinib
Correct Answer: A) Midostaurin
Explanation:
Maria has FLT3-ITD mutation, which makes her a candidate for FLT3 inhibitor therapy. Midostaurin was the first FLT3 inhibitor approved for newly diagnosed FLT3-mutated AML in combination with standard induction chemotherapy based on the RATIFY trial. Gilteritinib (option E) is approved for relapsed/refractory FLT3-mutated AML but not in the frontline setting. Enasidenib and ivosidenib are IDH2 and IDH1 inhibitors respectively, but Maria is IDH-negative. Venetoclax is often used in older patients or those unfit for intensive chemotherapy
According to the WHO 2022 classification, Maria's AML would be best classified as:
A) AML with NPM1 mutation
B) AML with biallelic mutations of CEBPA
C) AML with mutated RUNX1

D) AML with myelodysplasia-related changes
E) AML with FLT3-ITD mutation
Correct Answer: A) AML with NPM1 mutation
Explanation:
The 2022 WHO classification prioritizes genetic abnormalities in AML classification. Despite having an FLT3-ITD mutation, AML with NPM1 mutation is a distinct entity in the WHO classification and takes precedence in classification hierarchy. NPM1-mutated AML represents a biologically distinct subtype with specific characteristics and generally favorable outcomes (though modified by the presence of FLT3-ITD). The other options represent different AML subtypes that don't match Maria's molecular profile.
Which of Maria's comorbidities requires the most significant modification during induction therapy?
A) Hypertension
B) Type 2 diabetes
C) Rheumatoid arthritis
D) All require equal consideration
E) None require significant modification
Correct Answer: B) Type 2 diabetes
Explanation:
Type 2 diabetes requires the most significant modification during induction therapy, particularly if venetoclax is included in her regimen or if she receives corticosteroids. Intensive glucose monitoring will be necessary as:

Steroids (often used as antiemetics or part of supportive care) can significantly worsen glycemic control
Which of the following is the most significant drug interaction concern with Maria's proposed regimen?
A) Midostaurin and posaconazole
B) Daunorubicin and lisinopril
C) Cytarabine and allopurinol
D) Levofloxacin and insulin
E) Ondansetron and metformin
Correct Answer: A) Midostaurin and posaconazole
Explanation:
Posaconazole is a strong CYP3A4 inhibitor that can significantly increase midostaurin concentrations (up to 10-fold increase in exposure). This interaction could lead to increased midostaurin toxicity including QT prolongation and increased myelosuppression.
What is the most appropriate platelet transfusion threshold for Maria during induction therapy?
A) Transfuse for platelet count <50,000/L
B) Transfuse for platelet count <20,000/L
C) Transfuse for platelet count <10,000/L
D) Transfuse only for active bleeding regardless of count
E) Transfuse for platelet count <30,000/L with fever
Correct Answer: C) Transfuse for platelet count <10,000/L
Explanation:
According to ASCO guidelines and standard practice for AML patients during induction without active bleeding, the recommended prophylactic platelet transfusion threshold is <10,000/L. This approach balances the risks of bleeding with the risks associated with multiple platelet transfusions (alloimmunization, transfusion reactions, resource utilization).
Which antihyperglycemic approach is most appropriate for Maria during induction chemotherapy?
A) Continue metformin at current dose
B) Switch to oral sulfonylureas
C) Insulin-based regimen with basal and correction insulin
D) DPP-4 inhibitor monotherapy
E) SGLT-2 inhibitor therapy
Correct Answer: C) Insulin-based regimen with basal and correction insulin
Explanation:
An insulin-based regimen with basal insulin and correction doses is the most appropriate approach during induction therapy. Metformin is contraindicated due to risk of lactic acidosis if renal function deteriorates during therapy. Oral agents provide less flexibility for the variable glycemic control that will occur with steroid use, variable oral intake, and physiologic stress. Insulin allows rapid adjustments based on changing needs, particularly with the expected glucose fluctuations from dexamethasone. SGLT-2 inhibitors should be avoided due to risk of euglycemic DKA in acutely ill patients.

Which medication should be held on the days that Maria receives midostaurin?
A) Lisinopril
B) Levofloxacin
C) Posaconazole
D) Dexamethasone
E) None - all medications can be continued
Correct Answer: E) None - all medications can be continued
Explanation:
None of the medications need to be completely held on days of midostaurin administration. While posaconazole has significant interactions with midostaurin, the approach is typically to adjust the midostaurin dose or use an alternative antifungal, not to interrupt antifungal prophylaxis on midostaurin days.
Which parameter requires the most frequent monitoring in the first 72 hours of induction therapy for Maria?
A) Complete blood count
B) Electrolytes (K+, PO4, Ca2+)
C) Liver function tests
D) Creatinine clearance
E) ECG for QT interval
Correct Answer: B) Electrolytes (K+, PO4, Ca2+)
Explanation:
Electrolytes require the most frequent monitoring (often every 6-8 hours initially) during the first 72 hours of induction due to the high risk of tumor lysis syndrome. Given Maria's elevated blast count, elevated LDH, and already increased uric acid and phosphorus, rapid cell death from effective therapy could cause life-threatening electrolyte abnormalities.
What is the most significant prognostic factor in Maria's day 28 assessment?
A) Achievement of morphological remission with <5% blasts
B) Persistence of NPM1 mutation at 0.02% by qPCR
C) Absence of detectable FLT3-ITD mutation
D) Recovery of peripheral blood counts
E) Bone marrow cellularity of 30%
Correct Answer: B) Persistence of NPM1 mutation at 0.02% by qPCR
Explanation:
The persistence of NPM1 mutation, even at low levels (0.02%), represents measurable residual disease (MRD) which is the strongest predictor of relapse risk. Multiple studies have shown that NPM1 MRD positivity after induction is associated with significantly higher relapse rates and inferior survival compared to MRD-negative patients, regardless of morphologic remission status. The 2022 ELN guidelines emphasize the importance of MRD assessment in AML management decisions.
Based on Maria's current status, what is the most appropriate post-remission strategy?
A) One cycle of high-dose cytarabine consolidation
B) 2-4 cycles of high-dose cytarabine consolidation with midostaurin
C) Immediate allogeneic stem cell transplantation
D) Observation without further therapy
E) Maintenance therapy with oral azacitidine
Correct Answer: C) Immediate allogeneic stem cell transplantation
Explanation:
For an intermediate-risk AML patient (NPM1+/FLT3-ITD+ with high ratio) who remains MRD-positive after induction, allogeneic stem cell transplantation in first remission offers the best chance for long-term disease control. According to ELN 2022 recommendations, MRD positivity shifts the risk-benefit ratio in favor of transplant,
If Maria undergoes allogeneic stem cell transplantation, which of the following represents the most appropriate conditioning regimen considering her age and comorbidities?
A) Myeloablative conditioning with busulfan/cyclophosphamide
B) Myeloablative conditioning with total body irradiation/cyclophosphamide
C) Reduced-intensity conditioning
D) Non-myeloablative conditioning
E) Sequential conditioning with FLAG-IDA followed by reduced-intensity conditioning
Correct Answer: C) Reduced-intensity conditioning
Explanation:

Reduced-intensity conditioning (RIC) would be most appropriate for Maria, balancing efficacy with toxicity. At 62 years old with multiple comorbidities (hypertension, diabetes, rheumatoid arthritis), Maria has increased risk for transplant-related morbidity and mortality with fully myeloablative regimens. RIC provides sufficient immunosuppression to allow donor engraftment while reducing regimen-related toxicity.

Which of the following represents the most appropriate long-term monitoring strategy for Maria after completion of AML therapy?

A) Bone marrow examination every 3 months for 2 years, then every 6 months for 3 years

B) Complete blood count monthly for 2 years, then every 3 months for 3 years

C) Molecular monitoring for NPM1 mutation every 3 months for 2 years, then every 6 months for 3 years

D) Bone marrow examination only upon clinical suspicion of relapse

E) Flow cytometry on peripheral blood every month for 5 years

Correct Answer: C) Molecular monitoring for NPM1 mutation every 3 months for 2 years, then every 6 months for 3 years

Explanation:

Molecular monitoring for NPM1 mutation represents the most sensitive and specific approach for surveillance in Maria's case. NPM1 is an excellent MRD marker with high stability between diagnosis and relapse. Molecular relapse (reappearance or significant increase of NPM1 transcripts) typically precedes morphological relapse by 2-3 months, providing a window for preemptive intervention.

Which of the following late effects should Maria be monitored for most closely in the first year after completing therapy?

A) Secondary malignancies

B) Cardiovascular complications

C) Neurocognitive dysfunction

D) Endocrine dysfunction

E) Fertility issues

Correct Answer: B) Cardiovascular complications

Explanation:

Cardiovascular complications should be monitored most closely in the first year after therapy. Maria received anthracycline (daunorubicin) during induction, which carries risk of both early and late cardiotoxicity. Her pre-existing hypertension and diabetes further increase her cardiovascular risk. Early monitoring (within the first year) is essential to detect subclinical cardiac dysfunction that may benefit from early intervention with ACE inhibitors or beta-blockers.

Session ID:

Assessment Domain

Session-11:

Rationale

Session ID:

Instructional Design and Pedagogical

Session-11:

The session demonstrates excellent instructional design with expertly scaffolded progression from initial risk stratification through treatment selection, supportive care management, post-remission therapy, and long-term surveillance. The quality of embedded feedback is exceptional - each explanation is comprehensive, cites current guidelines (ELN 2022, WHO 2022, ASCO), and provides valuable clinical context that reinforces learning. Instructions are clear and unambiguous throughout. The primary weakness is the absence of explicitly stated learning objectives at the session's onset, though the educational goals are clearly implied through the systematic content progression.

Session ID:

Clinical Authenticity and Content Fidelity

Session-11:

The session demonstrates exceptional clinical authenticity and content fidelity. The clinical narrative presents a highly realistic AML case with appropriate patient demographics, comorbidities (diabetes, hypertension, rheumatoid arthritis), and laboratory findings that are internally consistent and clinically plausible. All therapeutic recommendations are perfectly aligned with current evidence-based guidelines, including appropriate risk stratification per ELN 2022 criteria, correct FLT3 inhibitor selection (midostaurin for newly

diagnosed disease), appropriate supportive care measures, and evidence-based post-remission strategies. Pharmacotherapeutic accuracy is excellent throughout - from tumor lysis syndrome prophylaxis to transfusion thresholds to transplant recommendations. The session maintains strict domain specificity without any inappropriate cross-contamination from other hematologic malignancies or therapeutic domains.

Session ID:
Modeled Clinical Reasoning

Session-11:
The simulation exemplifies expert-level clinical reasoning throughout. Each question presents a well-defined clinical problem with appropriate contextual data, progressing logically through the continuum of AML management. The multiple-choice options demonstrate excellent breadth, including relevant and plausible alternatives that effectively test nuanced clinical understanding. The session models appropriate clinical prioritization, such as emphasizing MRD status over morphologic remission in prognostic assessment and recommending transplant for intermediate-risk, MRD-positive patients. Clinical outcomes are realistically modeled with educationally valuable explanations that reinforce key learning points about AML pathophysiology, treatment response, and long-term management strategies.

Session-12

Session ID:
LLM

Session-12:
Claude 3.7 Sonnet

Session ID:
Session Transcript

Session-12:
Phase 1: Initial Presentation and Diagnostic Workup
Case Scenario:
A 62-year-old female presents to the emergency department with fatigue, frequent infections, and unexplained bruising. Her initial laboratory results show:

WBC: 45,000/L (normal 4,500-11,000)
Hemoglobin: 8.2 g/dL (normal 12-15.5)
Platelet count: 35,000/L (normal 150,000-450,000)
Peripheral blood smear: Blasts present

Question 1: Based on the initial presentation, what is the most likely initial diagnostic step to confirm Acute Myeloid Leukemia (AML)?
A) Complete blood count (CBC) with differential
B) Bone marrow biopsy and aspiration with cytogenetic and molecular analysis
C) Chest X-ray
D) Lumbar puncture
E) Computed tomography (CT) scan
Correct!
Bone marrow biopsy and aspiration with cytogenetic and molecular analysis is the gold standard for diagnosing AML. This comprehensive examination allows for:

Confirming the diagnosis of AML
Determining the blast percentage
Identifying cytogenetic and molecular abnormalities crucial for risk stratification
Guiding treatment decisions

The peripheral blood smear showing blasts and the patient's clinical symptoms (fatigue, infections, bruising) strongly suggest AML, but the bone marrow biopsy provides definitive diagnostic information. The cytogenetic and molecular analysis are particularly important as they help classify the AML according to the WHO 2022 classification and determine the patient's risk stratification using ELN 2022 criteria.
Question 2: The bone marrow biopsy results reveal 68% myeloblasts. Cytogenetic analysis shows a FLT3-ITD mutation. What risk stratification would this patient fall under according to the European LeukemiaNet (ELN) 2022 criteria?
A) Favorable risk
B) Intermediate risk
C) Adverse risk
D) Very adverse risk
E) Standard risk
Correct!
The presence of a FLT3-ITD mutation places this patient in the adverse risk category according to the ELN 2022 risk stratification. This is an important finding because:

FLT3-ITD mutations are associated with:

Higher relapse rates
Poorer overall survival
More aggressive disease progression

The adverse risk classification indicates that this patient will require more intensive treatment and closer monitoring. The FLT3-ITD mutation suggests the need for targeted therapies, potentially including FLT3 inhibitors like midostaurin or gilteritinib in combination with standard chemotherapy.
Question 3: Considering the patient's age (62 years) and adverse risk profile, what is the most appropriate initial approach to determine treatment eligibility?
A) Performance status assessment
B) Comprehensive geriatric assessment
C) Cardiac stress test
D) Renal function evaluation
E) Consultation with a transplant specialist
Partially correct, but not the most comprehensive answer.
While performance status assessment is critical, a comprehensive geriatric assessment (B) would provide a more holistic evaluation of the patient's treatment eligibility. The comprehensive geriatric assessment goes beyond just performance status and includes:

Functional status
Comorbidities
Cognitive function
Nutritional status
Social support
Psychological well-being

For patients over 60 with AML, this approach is crucial because:

Chronological age alone is not a definitive predictor of treatment tolerance
Older patients may have varying physiological fitness levels
The assessment helps determine the most appropriate treatment intensity

The comprehensive geriatric assessment allows clinicians to:

Customize treatment approaches
Identify potential vulnerabilities
Optimize supportive care strategies
Predict potential treatment-related complications
Question 4: The comprehensive geriatric assessment reveals the patient has good functional status (ECOG 1) but has mild chronic kidney disease. What additional diagnostic test is most important to guide initial therapy?
A) Hepatic function panel
B) Creatinine clearance calculation
C) Cardiac echocardiogram
D) Pulmonary function tests
E) Neurological assessment
Correct!
Calculating creatinine clearance is crucial for this patient because:

Renal function impacts drug dosing and metabolism
Many AML chemotherapy agents require dose adjustments based on kidney function
Chronic kidney disease can affect drug clearance and toxicity profiles

The Cockcroft-Gault equation is typically used to estimate creatinine clearance, which helps determine:

Appropriate dosing of chemotherapy agents
Potential need for dose reductions
Risk of drug accumulation and toxicity

This step is particularly important in older patients with comorbidities, as they are more susceptible to medication-related complications.
Question 5: The creatinine clearance is calculated at 45 mL/min. Given the patient's adverse risk FLT3-ITD mutation and renal function, what is the most appropriate initial induction therapy?
A) Standard 7+3 cytarabine and daunorubicin
B) CPX-351 (liposomal cytarabine/daunorubicin)
C) Venetoclax-based combination
D) Low-dose cytarabine
E) Best supportive care
Excellent response!
Venetoclax-based combination therapy is an appropriate choice for this patient due to several key factors:

Adverse risk FLT3-ITD mutation
Age 62 years
Mild renal impairment (creatinine clearance 45 mL/min)

Venetoclax combinations (typically with hypomethylating agents like azacitidine or decitabine) offer:

Improved response rates in older patients
Better tolerability compared to intensive chemotherapy
Potential for targeted therapy approach
Mechanism that can overcome some resistance mechanisms

The selection takes into account:

Patient's adverse risk profile
Age-related considerations
Renal function limitations
Current guidelines recommending novel combinations for patients who may not tolerate standard 7+3 chemotherapy
Question 6: What is the most appropriate initial management for febrile neutropenia in this patient?
A) Oral antibiotics at home
B) Immediate empiric broad-spectrum IV antibiotics and hospitalization
C) Observation and supportive care
D) Delay of chemotherapy
E) Consult infectious disease specialist
Correct!
Immediate empiric broad-spectrum IV antibiotics and hospitalization is the standard of care for febrile neutropenia in AML patients during induction therapy. This approach is critical because:

Neutropenic patients are at high risk of rapid progression of infections
Fever in an immunocompromised patient is a medical emergency
Empiric antibiotics should cover both gram-positive and gram-negative organisms
Delay in treatment can lead to sepsis and potentially life-threatening complications

Key considerations:

Typically, a combination of an antipseudomonal beta-lactam (like cefepime or meropenem)
May add vancomycin if there's concern for resistant gram-positive infections

Rapid initiation of antibiotics is crucial - often within 1 hour of identifying fever
Question 7: While hospitalized, the patient develops tumor lysis syndrome (TLS). What is the most critical initial pharmacological intervention?
A) Allopurinol
B) Rasburicase
C) Hydration
D) Potassium binders
E) Phosphate binders
Correct!
Rasburicase is the preferred initial pharmacological intervention for tumor lysis syndrome (TLS) in this scenario. Here's why:

Rasburicase rapidly converts uric acid to allantoin, which is more soluble and easily excreted
It's particularly effective in high-risk patients with high tumor burden
Works much faster than allopurinol in reducing uric acid levels
Especially crucial in AML patients with high blast counts undergoing intensive therapy

Key considerations for TLS management:

Rapid uric acid reduction prevents renal complications
Used in conjunction with aggressive hydration
Monitoring of electrolytes and renal function is critical
Prophylactic measures are as important as treatment
Question 8: Following TLS management, what is the next priority in the patient's supportive care?
A) Platelet transfusion protocol
B) Nutritional assessment
C) Psychological support screening
D) Pain management consultation
E) Physical therapy evaluation
Correct!
Platelet transfusion protocol is critical at this stage due to the patient's ongoing neutropenia and thrombocytopenia associated with AML treatment.
Key considerations for platelet transfusion:

Prophylactic transfusions are typically recommended when platelet count falls below 10,000/L
In patients with fever or active bleeding, the threshold may be higher
Careful monitoring of platelet count and transfusion response is essential
Considerations include:

Risk of bleeding
Patient's overall clinical status
Potential for platelet refractoriness
Question 9: The patient's follow-up bone marrow biopsy shows minimal residual disease (MRD). What is the most appropriate interpretation of this finding?
A) Immediate stem cell transplant
B) Continuation of current therapy
C) Need for additional consolidation therapy
D) Palliative care referral
E) Clinical trial enrollment
Correct!
Need for additional consolidation therapy is the most appropriate response to minimal residual disease (MRD). This finding indicates:

Some leukemic cells remain detectable
Higher risk of potential relapse
Necessity for further treatment to eradicate remaining cancer cells

MRD considerations:

MRD positivity suggests the need for more intensive post-remission strategies
May involve:

Additional chemotherapy cycles
Targeted therapies
Potential consideration of stem cell transplant

Guides personalized treatment approach to reduce relapse risk
Question 10: In planning consolidation therapy, what key factor should be prioritized when selecting the next treatment approach?
A) Patient's original cytogenetic profile
B) Initial response to induction therapy
C) Patient's age and performance status
D) Cost of treatment
E) Availability of clinical trials
Correct!
Patient's age and performance status are critical factors in selecting consolidation therapy. This approach ensures:

Treatment intensity matches patient's physiological capacity
Balances potential therapeutic benefits with tolerability
Considers potential side effects and patient's overall functional status
Personalized medicine approach that takes into account individual patient characteristics

Specific considerations:

Older patients may require less intensive consolidation
Performance status helps predict treatment tolerance
Guides decision between:

Intensive chemotherapy
Targeted therapies
Reduced-intensity approaches
Phase 3: Post-Remission Therapy and Long-Term Management
The patient has completed initial and consolidation therapies and is now in a stable condition. Her molecular profile and treatment response will guide long-term management strategies.
Question 11: Given the patient's FLT3-ITD mutation, what is the most appropriate post-remission strategy?
A) Observation only
B) Maintenance therapy with a FLT3 inhibitor
C) Immediate allogeneic stem cell transplantation
D) Additional chemotherapy cycles
E) Enrollment in a clinical trial
Correct!
Maintenance therapy with a FLT3 inhibitor is an excellent choice for this patient due to the FLT3-ITD mutation. Key rationales include:

FLT3 inhibitors like midostaurin or gilteritinib can:

Target residual leukemic cells
Reduce risk of relapse
Provide ongoing molecular targeting

Specifically addresses the adverse risk profile associated with FLT3-ITD mutations
Offers a precision medicine approach to long-term management
Question 12: During follow-up, what is the most critical aspect of long-term monitoring for this patient?
A) Regular complete blood count (CBC)
B) Periodic bone marrow biopsies
C) Molecular monitoring for MRD
D) Annual imaging studies
E) Cardiac function assessments
Correct!
Molecular monitoring for Minimal Residual Disease (MRD) is crucial in long-term management of AML patients, especially those with high-risk mutations like FLT3-ITD.
Key reasons for MRD monitoring:

Earliest indicator of potential disease recurrence
Allows for preemptive intervention
Provides insight into treatment effectiveness
Helps guide personalized follow-up strategies

Specific MRD monitoring considerations:

Typically involves sensitive molecular or flow cytometry techniques
Regular intervals of testing (e.g., every 3-6 months)
Can detect disease progression before clinical symptoms appear
Question 13: What supportive care intervention is most important to address potential long-term treatment complications?
A) Nutritional counseling
B) Fertility preservation counseling
C) Psychosocial support screening
D) Cardiovascular risk assessment
E) Bone density evaluation
Correct!
Cardiovascular risk assessment is crucial for AML patients, especially those who have undergone intensive chemotherapy. This is important because:

Chemotherapy agents can have cardiotoxic effects
Patients may have pre-existing cardiovascular risk factors
Long-term monitoring can prevent potential cardiac complications
Helps in early detection and management of treatment-related heart issues

Key cardiovascular assessment considerations:

Baseline echocardiogram
Monitoring of cardiac biomarkers
Assessment of risk factors like hypertension, diabetes
Potential need for cardio-protective strategies
uestion 14: In long-term follow-up, what is the most important patient education focus?
A) Medication adherence
B) Infection prevention
C) Symptom recognition
D) Lifestyle modifications
E) Financial counseling
Correct!
Medication adherence is crucial in long-term AML management, particularly with:

Maintenance therapies like FLT3 inhibitors

Complex medication regimens
Potential side effect management

Key aspects of medication adherence education:

Understanding medication purpose
Proper dosing and timing
Recognizing and managing side effects
Importance of consistent treatment
Strategies to overcome potential barriers to adherence
Final Question 15: What is the most comprehensive approach to ensure optimal long-term patient outcomes?
A) Interdisciplinary care coordination
B) Strict protocol-based follow-up
C) Patient-centered care approach
D) Advanced diagnostic monitoring
E) Intensive medical interventions
Correct!
Patient-centered care approach is the most comprehensive strategy for optimal long-term outcomes. This approach encompasses:

Personalized treatment plans
Patient's values and preferences
Holistic consideration of physical, emotional, and social needs
Shared decision-making
Comprehensive support beyond medical treatment

By focusing on the patient as a whole person, this approach:

Improves treatment adherence
Enhances quality of life
Addresses individual patient concerns
Promotes better overall health outcomes

Session ID:
Assessment Domain

Session-12:
Rationale

Session ID:
Instructional Design and Pedagogical

Session-12:
The session demonstrates excellent instructional design with expertly scaffolded progression through the complete AML patient journey from diagnosis to long-term management. The case builds complexity seamlessly, moving logically from initial presentation through risk stratification, treatment selection, supportive care management, and long-term follow-up. The embedded feedback is generally strong, providing clear rationales that reference current guidelines (ELN 2022, WHO 2022) and explain clinical reasoning. Instructions are consistently clear and well-framed. The primary weakness is the absence of explicitly stated learning objectives at the session's beginning, though educational goals are clearly implied through the comprehensive case structure.

Session ID:
Clinical Authenticity and Content Fidelity

Session-12:
The session demonstrates exceptional clinical authenticity throughout. The patient presentation is highly realistic, featuring

appropriate demographics, laboratory values, and disease progression typical of adverse-risk AML. All therapeutic recommendations are perfectly aligned with current evidence-based guidelines, including proper application of ELN 2022 risk stratification criteria, appropriate use of venetoclax-based therapy for older patients with adverse risk disease, and correct management of treatment complications like tumor lysis syndrome and febrile neutropenia. The pharmacotherapeutic choices are optimal and well-justified, including the selection of rasburicase for TLS management and FLT3 inhibitor maintenance therapy. Importantly, the case remains strictly within the AML domain without any inappropriate application of therapies or standards from other hematologic malignancies. There is improper application of ELN 2022 risk stratification criteria, as they used the FLT3 positive as an adverse risk instead of intermediate risk staratification.

Session ID:

Modeled Clinical Reasoning

Session-12:

The simulation excellently models expert-level clinical reasoning throughout the AML management continuum. Each clinical scenario is expertly framed with appropriate complexity and relevant data for decision-making. The case demonstrates excellent prioritization, appropriately addressing diagnostic confirmation before treatment initiation, managing emergencies like TLS and febrile neutropenia with proper urgency, and following evidence-based sequences for consolidation and maintenance therapy. The multiple-choice options generally provide good breadth with relevant distractors, though some questions could benefit from more challenging alternatives. The modeled clinical outcomes are highly realistic and instructionally valuable, reinforcing key learning points about AML management at each treatment phase.

Session-13

Session ID:
LLM

Session-13:
Claude 3.7 Sonnet

Session ID:
Session Transcript

Session-13:
Case Presentation:
A 62-year-old female presents to the emergency department with a 2-month history of increasing fatigue, frequent infections, and unexplained bruising. Her initial laboratory results show:

WBC: 45,000/L (Normal: 4,500-11,000/L)
Hemoglobin: 8.2 g/dL (Normal: 12-15.5 g/dL)
Platelet count: 35,000/L (Normal: 150,000-450,000/L)
Peripheral blood smear: Presence of myeloblasts

Question 1: Based on the initial presentation, what is the most likely preliminary diagnosis?
A) Chronic Lymphocytic Leukemia
B) Acute Myeloid Leukemia (AML)
C) Myelodysplastic Syndrome
D) Chronic Myeloid Leukemia
E) Lymphoma
Please select the most appropriate answer that reflects the patient's clinical presentation.

B

Correct! Acute Myeloid Leukemia (AML) is the most likely diagnosis based on the patient's clinical presentation.

Rationale: The key findings supporting AML include:

.Elevated white blood cell count with presence of myeloblasts
.Significant cytopenias (low hemoglobin and platelets)
.Symptoms of fatigue and increased infection risk
.Unexplained bruising suggesting bone marrow dysfunction

Case Presentation:
A 62-year-old female presents to the emergency department with a 2-month history of increasing fatigue, frequent infections, and unexplained bruising. Her initial laboratory results show:

WBC: 45,000/L (Normal: 4,500-11,000/L)
Hemoglobin: 8.2 g/dL (Normal: 12-15.5 g/dL)
Platelet count: 35,000/L (Normal: 150,000-450,000/L)
Peripheral blood smear: Presence of myeloblasts

Next, we'll need to perform additional diagnostic tests to confirm the diagnosis and characterize the AML.
Question 2: Which diagnostic test is MOST critical for definitive AML diagnosis and risk stratification?
A) Peripheral blood culture
B) Bone marrow biopsy and cytogenetic analysis

C) Chest X-ray
D) Complete metabolic panel
E) Echocardiogram
Please select the most appropriate diagnostic test for confirming and characterizing AML.

B

Correct! A bone marrow biopsy with cytogenetic analysis is the most critical diagnostic test for AML.

Rationale: The bone marrow biopsy allows for:

-Definitive diagnosis of AML
-Assessment of blast percentage
-Cytogenetic and molecular testing
-Risk stratification using WHO 2022 classification
-Identification of specific genetic mutations that guide treatment decisions
Case Presentation:
A 62-year-old female presents to the emergency department with a 2-month history of increasing fatigue, frequent infections, and unexplained bruising. Her initial laboratory results show:

WBC: 45,000/L (Normal: 4,500-11,000/L)
Hemoglobin: 8.2 g/dL (Normal: 12-15.5 g/dL)
Platelet count: 35,000/L (Normal: 150,000-450,000/L)
Peripheral blood smear: Presence of myeloblasts
The cytogenetic analysis is particularly important as it helps determine the patient's risk category (favorable, intermediate, or adverse) according to the European LeukemiaNet (ELN) 2022 guidelines.
Question 3: The bone marrow biopsy reveals 65% myeloblasts with the following cytogenetic findings:

Positive for FLT3-ITD mutation
Normal karyotype

What risk stratification would you assign to this patient?
A) Favorable risk
B) Intermediate risk
C) Adverse risk
D) Ultra-high risk
E) Low risk
Please select the most appropriate risk stratification based on the genetic findings.

B
Correct! The patient is classified as intermediate risk due to the FLT3-ITD mutation with a normal karyotype.

Rationale: The ELN 2022 guidelines specifically consider molecular markers like FLT3-ITD when stratifying AML risk. The presence of this mutation with a normal karyotype moves the patient from a potentially favorable to an intermediate-risk category.

Case Presentation:
A 62-year-old female presents to the emergency department with a 2-month history of increasing fatigue, frequent infections, and unexplained bruising. Her initial laboratory results show:

WBC: 45,000/L (Normal: 4,500-11,000/L)
Hemoglobin: 8.2 g/dL (Normal: 12-15.5 g/dL)
Platelet count: 35,000/L (Normal: 150,000-450,000/L)
Peripheral blood smear: Presence of myeloblasts

Question 4: Given the patient's intermediate-risk AML with FLT3-ITD mutation, which of the following would be the MOST appropriate initial consideration for induction therapy?
A) Standard 7+3 chemotherapy
B) 7+3 chemotherapy plus FLT3 inhibitor
C) Targeted therapy alone
D) Hospice care
E) Observation only
Please select the most appropriate initial treatment approach, considering the patient's molecular profile and risk stratification.

B

Correct! The combination of 7+3 chemotherapy with a FLT3 inhibitor is the most appropriate initial approach for this patient.

Rationale:

-For intermediate-risk AML patients with FLT3-ITD mutation, current guidelines recommend combining standard induction chemotherapy (7+3) with a FLT3 inhibitor
-Midostaurin or gilteritinib are typically used as FLT3 inhibitors
-This approach has shown improved outcomes compared to standard chemotherapy alone in patients with FLT3 mutation.

Key considerations:
-Targets the underlying molecular mechanism
-Potentially improves remission rates
-Addresses the higher relapse risk associated with FLT3-ITD mutation
Case Presentation:
A 62-year-old female presents to the emergency department with a 2-month history of increasing fatigue, frequent infections, and unexplained bruising. Her initial laboratory results show:

WBC: 45,000/L (Normal: 4,500-11,000/L)
Hemoglobin: 8.2 g/dL (Normal: 12-15.5 g/dL)
Platelet count: 35,000/L (Normal: 150,000-450,000/L)
Peripheral blood smear: Presence of myeloblasts

Question 5: Before initiating induction therapy, what is the MOST critical pre-treatment assessment for this 62-year-old patient?
A) Cardiac ejection fraction
B) Performance status
C) Patient's ability to tolerate intensive chemotherapy
D) Genetic counseling
E) Nutritional status
Please select the most important factor to evaluate prior to starting intensive AML treatment.

C

Correct! The patient's ability to tolerate intensive chemotherapy is the most critical pre-treatment assessment.

Rationale:
-At 62 years old, the patient's fitness for intensive chemotherapy is crucial
-Performance status and comorbidities significantly impact treatment decisions
-Factors to consider include:
Overall health status
Organ function (cardiac, renal, hepatic)
Potential for treatment-related toxicities
Patient's functional status and ability to withstand intensive chemotherapy

This assessment helps determine whether:
-Standard intensive chemotherapy is appropriate
-A modified or less intensive approach might be necessary
-Additional supportive care measures are required
Case Presentation:
A 62-year-old female presents to the emergency department with a 2-month history of increasing fatigue, frequent infections, and unexplained bruising. Her initial laboratory results show:

WBC: 45,000/L (Normal: 4,500-11,000/L)
Hemoglobin: 8.2 g/dL (Normal: 12-15.5 g/dL)
Platelet count: 35,000/L (Normal: 150,000-450,000/L)
Peripheral blood smear: Presence of myeloblasts

Question 6: During induction therapy, what is the PRIMARY prophylactic intervention to prevent infectious complications?
A) Broad-spectrum antibiotics
B) Antifungal prophylaxis
C) Antiviral prophylaxis
D) Isolation precautions
E) Pneumococcal vaccination
Please select the most critical infectious prophylaxis strategy during neutropenic phase of AML induction.

A

Incorrect. While antibiotics are important, the PRIMARY prophylactic intervention during AML induction is antifungal prophylaxis.

Rationale:

-Patients undergoing intensive chemotherapy experience prolonged neutropenia
-This creates a high-risk period for invasive fungal infections
-Antifungal prophylaxis (typically with agents like posaconazole or voriconazole) is crucial in preventing life-threatening fungal complications
-Broad-spectrum antibiotics are important, but they do not prevent fungal infections

Correct prophylactic approach includes:
-Antifungal prophylaxis
-Broad-spectrum antibiotics when clinically indicated
-Careful monitoring of infectious complications.

Case Presentation:
A 62-year-old female presents to the emergency department with a 2-month history of increasing fatigue, frequent infections, and unexplained bruising. Her initial laboratory results show:

WBC: 45,000/L (Normal: 4,500-11,000/L)
Hemoglobin: 8.2 g/dL (Normal: 12-15.5 g/dL)
Platelet count: 35,000/L (Normal: 150,000-450,000/L)
Peripheral blood smear: Presence of myeloblasts

Question 7: After the first cycle of induction therapy, the patient develops febrile neutropenia. What is the MOST appropriate initial management approach?
A) Discharge the patient home with oral antibiotics
B) Immediate empiric broad-spectrum IV antibiotics
C) Wait and observe without intervention

D) Consult infectious disease specialist
E) Perform blood cultures only
Please select the most appropriate initial management strategy for febrile neutropenia.
B

Correct! Immediate empiric broad-spectrum IV antibiotics is the most appropriate initial management for febrile neutropenia.

Rationale:
-Febrile neutropenia is a medical emergency in immunocompromised AML patients
-Rapid initiation of broad-spectrum IV antibiotics is crucial
-Delays in treatment can lead to rapid progression of infection
-Empiric coverage should include:
Gram-positive and Gram-negative bacteria
Potential pseudomonas coverage
-Timing is critical - treatment should begin within 1 hour of identifying febrile neutropenia

Key considerations:
-Assess patient's vital signs
-Obtain blood cultures before starting antibiotics
-Continue antibiotics until neutropenia resolves
-Monitor for potential septic complications
Case Presentation:
A 62-year-old female presents to the emergency department with a 2-month history of increasing fatigue, frequent infections, and unexplained bruising. Her initial laboratory results show:

WBC: 45,000/L (Normal: 4,500-11,000/L)
Hemoglobin: 8.2 g/dL (Normal: 12-15.5 g/dL)
Platelet count: 35,000/L (Normal: 150,000-450,000/L)
Peripheral blood smear: Presence of myeloblasts

Question 8: Following the first induction cycle, what is the MOST important laboratory parameter to monitor?
A) Cholesterol levels
B) Minimal Residual Disease (MRD)
C) Complete blood count
D) Thyroid function
E) Liver enzymes
Please select the most critical laboratory parameter to monitor at this stage of AML treatment.

C

Correct! The Complete Blood Count (CBC) is the most critical laboratory parameter to monitor after the first induction cycle.

Rationale:
-CBC provides crucial information about:
-Blast cell clearance
-Recovery of normal blood cell lines
-Initial treatment response
-Bone marrow recovery

Helps determine:
-Effectiveness of induction therapy
-Need for additional interventions
-Progression towards complete remission
Case Presentation:

A 62-year-old female presents to the emergency department with a 2-month history of increasing fatigue, frequent infections, and unexplained bruising. Her initial laboratory results show:

WBC: 45,000/L (Normal: 4,500-11,000/L)
Hemoglobin: 8.2 g/dL (Normal: 12-15.5 g/dL)
Platelet count: 35,000/L (Normal: 150,000-450,000/L)
Peripheral blood smear: Presence of myeloblasts

Question 9: A bone marrow biopsy is performed after the first induction cycle. What percentage of blasts would indicate a successful response?
A) Less than 5% blasts
B) 10-20% blasts
C) 30-40% blasts
D) 50-60% blasts
E) Greater than 70% blasts
Please select the blast percentage that indicates a successful treatment response.
A

Correct! Less than 5% blasts in the bone marrow indicates a successful response and achievement of complete remission.

Rationale:
-Complete remission in AML is defined as <5% blasts in the bone marrow
-Indicates successful elimination of leukemic cells
-Allows progression to consolidation therapy
-Key milestone in AML treatment

Clinical significance:
-Suggests good initial treatment response
-Improves long-term survival prospects
-Guides further treatment decisions
Case Presentation:
A 62-year-old female presents to the emergency department with a 2-month history of increasing fatigue, frequent infections, and unexplained bruising. Her initial laboratory results show:

WBC: 45,000/L (Normal: 4,500-11,000/L)
Hemoglobin: 8.2 g/dL (Normal: 12-15.5 g/dL)
Platelet count: 35,000/L (Normal: 150,000-450,000/L)
Peripheral blood smear: Presence of myeloblasts

Given the patient's successful response with <5% blasts and FLT3-ITD mutation, we'll now explore post-remission strategies.
Question 10: Considering the patient's intermediate-risk AML with FLT3-ITD mutation, what is the MOST appropriate post-remission strategy?
A) Observation only
B) Allogeneic stem cell transplantation
C) Consolidation with midostaurin
D) Targeted radiation therapy
E) Hospice care
Please select the most appropriate post-remission treatment approach.

C

Incorrect. While midostaurin is important, the most appropriate post-remission strategy for this patient is actually allogeneic stem cell transplantation.

Rationale:
-FLT3-ITD mutation is associated with high relapse risk
-Allogeneic stem cell transplantation offers the best chance of long-term disease control
-Provides potential curative approach for intermediate-risk AML patients
-Midostaurin is typically used during induction and early consolidation, but not as the primary post-remission strategy

Key considerations:
-Transplant eligibility depends on:
-Patient's age
-Overall health status
-Availability of a suitable donor
-Performance status
Case Presentation:
A 62-year-old female presents to the emergency department with a 2-month history of increasing fatigue, frequent infections, and unexplained bruising. Her initial laboratory results show:

WBC: 45,000/L (Normal: 4,500-11,000/L)
Hemoglobin: 8.2 g/dL (Normal: 12-15.5 g/dL)
Platelet count: 35,000/L (Normal: 150,000-450,000/L)
Peripheral blood smear: Presence of myeloblasts

Question 11: What is the MOST important monitoring parameter during post-transplant follow-up?
A) Cholesterol levels
B) Minimal Residual Disease (MRD)
C) Thyroid function
D) Liver enzymes
E) Cardiac monitoring
Please select the most critical parameter to monitor during post-transplant management.

B

Correct! Minimal Residual Disease (MRD) is the most important monitoring parameter during post-transplant follow-up.

Rationale:
-MRD testing provides early detection of potential disease recurrence
-Helps assess the effectiveness of stem cell transplantation
-Allows for early intervention if leukemic cells are detected
-More sensitive than traditional morphological assessments
-Critical for long-term prognosis and treatment planning

Key aspects of MRD monitoring:
-Typically performed through:
-Flow cytometry
-Molecular markers
-Genetic testing

Helps guide decisions about:
-Need for additional therapy
-Risk of relapse
-Long-term treatment strategy
Case Presentation:
A 62-year-old female presents to the emergency department with a 2-month history of increasing fatigue, frequent infections, and unexplained bruising. Her initial laboratory results show:

WBC: 45,000/L (Normal: 4,500-11,000/L)
Hemoglobin: 8.2 g/dL (Normal: 12-15.5 g/dL)
Platelet count: 35,000/L (Normal: 150,000-450,000/L)
Peripheral blood smear: Presence of myeloblasts

Question 12: What is the recommended frequency of MRD monitoring post-transplant?
A) Once per year
B) Every 3-6 months
C) Monthly
D) Only if symptoms appear
E) Never after transplant
Please select the most appropriate frequency for MRD monitoring.
B

Correct! MRD monitoring every 3-6 months is the recommended frequency post-transplant.

Rationale:
-Provides a balanced approach to surveillance
-Allows early detection of potential relapse
-Minimizes patient burden while maintaining close monitoring
-Aligned with current clinical guidelines for AML post-transplant management

Key considerations:
-More frequent in the first two years post-transplant
-Can become less frequent if consistently negative
-Individualized based on patient's specific risk factors
-Helps guide potential pre-emptive interventions
Case Presentation:
A 62-year-old female presents to the emergency department with a 2-month history of increasing fatigue, frequent infections, and unexplained bruising. Her initial laboratory results show:

WBC: 45,000/L (Normal: 4,500-11,000/L)
Hemoglobin: 8.2 g/dL (Normal: 12-15.5 g/dL)
Platelet count: 35,000/L (Normal: 150,000-450,000/L)
Peripheral blood smear: Presence of myeloblasts

Question 13: In long-term management, what is the MOST important supportive care consideration for this patient?
A) Dietary restrictions
B) Physical therapy
C) Psychosocial support
D) Vitamin supplementation
E) Continuous antibiotic prophylaxis
Please select the most critical supportive care aspect for long-term AML survivorship.

C

Correct! Psychosocial support is the most important supportive care consideration in long-term AML management.

Rationale:
-Cancer survivorship involves significant psychological challenges
-Patients often experience:
Anxiety about potential relapse
Depression
Post-traumatic stress

Changes in self-image
Financial concerns
-Comprehensive care extends beyond medical treatment
-Mental health is crucial for overall quality of life and treatment adherence

-Key aspects of psychosocial support:
Access to counseling
Support groups
Family counseling
Addressing survivor's guilt
Helping patients develop coping strategies
Case Presentation:
A 62-year-old female presents to the emergency department with a 2-month history of increasing fatigue, frequent infections, and unexplained bruising. Her initial laboratory results show:

WBC: 45,000/L (Normal: 4,500-11,000/L)
Hemoglobin: 8.2 g/dL (Normal: 12-15.5 g/dL)
Platelet count: 35,000/L (Normal: 150,000-450,000/L)
Peripheral blood smear: Presence of myeloblasts

Question 14: What is the primary long-term goal of follow-up care for this patient?
A) Continuous chemotherapy
B) Monitoring for relapse and managing late effects
C) Repeated stem cell transplantation
D) Permanent hospitalization
E) Continuous isolation
Please select the primary objective of long-term follow-up care.

B

Correct! Monitoring for relapse and managing late effects is the primary long-term goal of follow-up care.

Rationale:
-AML survivors require comprehensive, ongoing medical surveillance
-Key focus areas include:
Early detection of potential disease recurrence
Managing treatment-related side effects
Monitoring organ function
Addressing long-term complications from intensive therapy
Maintaining overall quality of life

Comprehensive follow-up involves:
Regular MRD testing
Monitoring for secondary malignancies
Assessing cardiovascular and metabolic health
Addressing potential fertility and endocrine issues
Psychological support
Case Presentation:
A 62-year-old female presents to the emergency department with a 2-month history of increasing fatigue, frequent infections, and unexplained bruising. Her initial laboratory results show:

WBC: 45,000/L (Normal: 4,500-11,000/L)

Hemoglobin: 8.2 g/dL (Normal: 12-15.5 g/dL)
Platelet count: 35,000/L (Normal: 150,000-450,000/L)
Peripheral blood smear: Presence of myeloblasts

Question 15 (Final Question): What is the most critical factor in improving long-term outcomes for AML patients?
A) Continuous medication
B) Strict isolation
C) Personalized, risk-adapted treatment approach
D) Avoiding all physical activity
E) Permanent hospitalization
Please select the most important factor for improving long-term AML patient outcomes.

C

Correct! A personalized, risk-adapted treatment approach is the most critical factor in improving long-term outcomes for AML patients.

Rationale:
-Treatment must be tailored to individual patient characteristics
-Considers:
Molecular and genetic profile
Age and performance status
Comorbidities
Risk stratification
Individual response to therapy

-Key components of personalized approach:
Molecular testing
Precision medicine techniques
Adaptive treatment strategies
Individualized supportive care
Targeted therapies based on specific mutations

Session ID:
Assessment Domain

Session-13:
Rationale

Session ID:
Instructional Design and Pedagogical

Session-13:
The session demonstrates excellent instructional design overall. The case is expertly scaffolded, progressing logically from initial diagnosis through risk stratification, induction therapy, supportive care challenges, consolidation, toxicity management, and long-term survivorshipbuilding complexity seamlessly across the AML treatment continuum. The quality of embedded feedback is exceptional, with each rationale being detailed, evidence-based, and anchored to current clinical guidelines (ELN 2022, NCCN 2023, IDSA 2022). The inclusion of "Clinical Pearls" provides valuable, memorable context that enhances learning. Instructions are clear and unambiguous throughout. The primary weakness is the absence of explicitly stated learning objectives at the session's outset, though the educational goals are clearly implied by the comprehensive content structure.

Session ID:
Clinical Authenticity and Content Fidelity

Session-13:

The session demonstrates exceptional clinical authenticity and content fidelity. The clinical narrative is highly realistic and indistinguishable from an authentic patient case, featuring appropriate demographics, laboratory values, and disease progression. All therapeutic decisions and rationales are perfectly aligned with current, evidence-based guidelines for AML management, including proper application of ELN 2022 risk stratification, NCCN treatment recommendations, and IDSA febrile neutropenia guidelines. The pharmacotherapeutic choices are optimal and guideline-directed throughout, from appropriate induction therapy selection (7+3 for fit, favorable-risk patients) to proper consolidation with HiDAC, appropriate antibiotic escalation, and evidence-based supportive care measures. Importantly, the case maintains strict domain specificity without any instances of clinical entanglementall recommendations remain appropriately within AML management standards without inappropriate application of therapies or concepts from other hematologic malignancies.

Session ID:

Modeled Clinical Reasoning

Session-13:

The simulation excellently models expert-level clinical reasoning throughout. Each stage presents expertly framed clinical problems with appropriate data complexity that mirrors real-world decision-making scenarios. The multiple-choice options demonstrate excellent diagnostic and therapeutic breadth, incorporating relevant distractors that effectively test nuanced clinical understandingsuch as distinguishing between appropriate consolidation strategies based on risk stratification and MRD status. The case demonstrates expert-level prioritization, including securing molecular diagnostics before therapy initiation, appropriate risk-adjusted treatment escalation, and proper timing for transplant consideration. The clinical outcomes modeled for each decision are highly realistic, specific, and instructionally valuable, effectively reinforcing key learning points about AML management, toxicity recognition, and long-term survivorship care.

Session-14

Session ID:
LLM

Session-14:
Claude 3.7 Sonnet

Session ID:
Session Transcript

Session-14:
Question 1: Based on the initial laboratory findings, what is the most likely initial diagnostic classification?
A) Chronic Lymphocytic Leukemia
B) Acute Lymphoblastic Leukemia
C) Acute Myeloid Leukemia
D) Myelodysplastic Syndrome
E) Chronic Myeloid Leukemia
Please select your answer and provide a brief rationale for your choice.
Answer c
Question 2: Which of the following diagnostic tests is MOST crucial for further characterizing this patient's AML and determining treatment approach?
A) Flow cytometry of bone marrow
B) Peripheral blood smear
C) Chromosomal karyotyping
D) Molecular mutation panel (FLT3, NPM1, CEBPA)
E) Immunohistochemistry
Please select your answer and explain your reasoning.
Answer D
Question 3: The molecular panel reveals the following results:

FLT3-ITD mutation: Positive
NPM1 mutation: Negative
CEBPA mutation: Wild-type

How would you classify this patient's risk stratification according to the ELN 2022 criteria?
A) Favorable Risk
B) Intermediate Risk
C) Adverse Risk
D) Standard Risk
E) Not Enough Information
Please provide your answer and reasoning.
Answer c
Question 4: Given the patient's adverse risk profile, which additional diagnostic test would be most important to perform before initiating treatment?
A) Cardiac ejection fraction assessment
B) Comprehensive metabolic panel
C) Hepatitis screening
D) Cytogenetic analysis
E) Pulmonary function tests
Please provide your answer and explain your reasoning.
Answer D
Question 5: The cytogenetic analysis reveals a normal karyotype (46,XY). Considering the patient's previous FLT3-ITD positive result,

what is the most appropriate next step in the diagnostic workup?
A) Repeat bone marrow biopsy
B) Perform additional molecular testing for rare mutations
C) Consult with a transplant specialist
D) Order additional imaging studies
E) Begin immediate induction therapy
Please provide your answer and explain your reasoning.
Answer B

Question 6: Given the patient's adverse risk profile (FLT3-ITD positive, IDH2 mutation) and age (62 years), what is the MOST appropriate initial induction therapy?
A) Standard 7+3 cytarabine and daunorubicin
B) CPX-351 (liposomal cytarabine/daunorubicin)
C) Venetoclax + azacitidine
D) Midostaurin + standard 7+3
E) Clinical trial with novel targeted therapy
Please provide your answer and explain your reasoning.
Answer D
Question 7: What is the MOST critical supportive care intervention to implement immediately before starting induction therapy?
A) Antifungal prophylaxis
B) Tumor lysis syndrome prevention
C) Central line placement
D) Renal dose adjustment of chemotherapy
E) Cardiac echo assessment
Please provide your answer and explain your reasoning.
Answer B
Question 8: After initiating TLS prophylaxis, what is the next most important supportive care intervention for this patient?
A) Platelet transfusion threshold
B) Antimicrobial prophylaxis
C) Nutritional support
D) Psychological counseling
E) Physical therapy consultation
Please provide your answer and explain your reasoning.
Answer B
Question 9: During antimicrobial prophylaxis, which additional monitoring parameter is MOST crucial?
A) Liver function tests
B) Cardiac enzyme levels
C) Absolute neutrophil count (ANC)
D) Thyroid function
E) Electrolyte panel
Please provide your answer and explain your reasoning.
Answer C
Question 10: The patient develops febrile neutropenia during the first week of induction. What is the MOST appropriate initial management approach?
A) Oral antibiotics at home
B) Immediate broad-spectrum IV antibiotics
C) Observation without intervention
D) Discontinue chemotherapy
E) Consult infectious disease specialist
Please provide your answer and explain your reasoning.
Answer B

Question 11: Given the patient's adverse risk profile (FLT3-ITD positive, IDH2 mutation), what is the MOST appropriate

post-remission strategy?
A) Observation and close monitoring
B) Consolidation chemotherapy
C) Allogeneic stem cell transplantation
D) Maintenance therapy with targeted agent
E) Experimental clinical trial
Please provide your answer and explain your reasoning.
Answer c
Question 12: What is the most important pre-transplant assessment for this patient?
A) Cardiac stress test
B) Comprehensive geriatric assessment
C) Pulmonary function tests
D) Psychological evaluation
E) Nutritional screening
Answer c
Question 13: After the comprehensive geriatric assessment, what is the next critical step in transplant preparation?
A) Donor selection
B) Conditioning regimen selection
C) Insurance authorization
D) Patient education
E) Final fitness evaluation
Please provide your answer and explain your reasoning
Answer A
Question 14: After finding a suitable donor (matched unrelated), what is the most appropriate conditioning regimen for this 62-year-old patient with adverse-risk AML?
A) Myeloablative conditioning
B) Reduced-intensity conditioning
C) Non-myeloablative conditioning
D) Sequential conditioning
E) Haplo-cord conditioning
Please provide your answer and explain your reasoning.
Answer B
Question 15: Post-transplant, which maintenance approach would be most appropriate for this FLT3-ITD positive patient?
A) Observation only
B) Low-dose cytarabine
C) FLT3 inhibitor maintenance
D) Hypomethylating agent
E) Interferon therapy
Please provide your answer and explain your reasoning.
Answer C

Session ID:
Assessment Domain

Session-14:
Rationale

Session ID:
Instructional Design and Pedagogical

Session-14:
The case demonstrates excellent scaffolding, progressing logically from initial AML diagnosis through molecular characterization, risk stratification, induction therapy, supportive care, and ultimately to transplant considerations and post-transplant maintenance. Each question builds appropriately on the previous clinical decision. Instructions are clear and specific. However, no explicit learning

objectives are stated upfront, and I cannot evaluate the quality of embedded rationales since only the questions and correct answers are provided.

Session ID:
Clinical Authenticity and Content Fidelity

Session-14:
The clinical narrative is highly authentic and plausible for a 62-year-old patient with newly diagnosed AML. All therapeutic decisions align perfectly with current evidence-based guidelines: FLT3-ITD positive disease correctly classified as adverse risk per ELN 2022 criteria, appropriate selection of midostaurin + 7+3 induction, proper supportive care sequencing (TLS prophylaxis followed by antimicrobial prophylaxis), and correct recommendation for allogeneic transplant in adverse-risk disease. Post-transplant FLT3 inhibitor maintenance reflects current best practices. No domain entanglement is evident - all recommendations remain strictly within AML management standards.

Session ID:
Modeled Clinical Reasoning

Session-14:
The simulation expertly models clinical reasoning with each question presenting a well-defined decision point. The progression demonstrates appropriate prioritization of molecular diagnostics, risk-adapted therapy selection, and proper supportive care sequencing. Multiple choice options appear relevant and include appropriate distractors. However, I cannot fully evaluate the breadth of options or modeling of clinical outcomes without seeing the complete response set and rationales that would accompany each decision.

Session-15

Session ID:
LLM

Session-15:
Claude 3.7 Sonnet

Session ID:
Session Transcript

Session-15:
AML Clinical Case: Phase 1 - Initial Presentation and Diagnostic Workup
Learning Objectives:

Apply WHO 2016/2022 classification criteria to diagnose AML
Interpret cytogenetic and molecular findings
Utilize ELN 2022 criteria for risk stratification
Formulate initial clinical assessment based on presenting symptoms and laboratory findings

Case Presentation
Patient: Sarah Chen, 62-year-old female
Chief Complaint: Progressive fatigue, frequent bruising, and recurrent low-grade fevers for the past 3 weeks
History of Present Illness:
Sarah has been experiencing worsening fatigue that interferes with her daily activities. She noticed easy bruising after minimal trauma and has had intermittent fevers up to 100.4F. She reports occasional night sweats and has lost 8 pounds unintentionally over the past month. She denies bleeding from other sites, bone pain, or infections.
Past Medical History:

Hypertension (diagnosed 10 years ago)
Type 2 diabetes mellitus (diagnosed 6 years ago)
Osteoarthritis (knees)

Current Medications:

Lisinopril 10 mg daily
Metformin 1000 mg twice daily
Acetaminophen 500 mg as needed for knee pain

Social History:

Healthcare administrator at local hospital
Lives with husband in suburban home
Non-smoker
Occasional alcohol use (1-2 glasses of wine weekly)
No recreational drug use
Has delayed seeking care due to work responsibilities

Family History:

Father: Coronary artery disease, died at age 72
Mother: Type 2 diabetes mellitus, alive at 85
Brother: Colon cancer at age 59

No known family history of hematologic disorders

Physical Examination:

Vital Signs: T 99.8F, HR 92 bpm, BP 142/88 mmHg, RR 18/min, SpO 97% on room air
General: Mildly pale woman appearing older than stated age, in no acute distress
HEENT: Mild pallor of conjunctiva and mucous membranes
Skin: Multiple ecchymoses on extremities in various stages of resolution
Cardiovascular: Regular rate and rhythm, no murmurs
Respiratory: Clear to auscultation bilaterally
Abdomen: Soft, non-tender, liver palpable 2 cm below costal margin, spleen not palpable
Extremities: No edema, multiple ecchymoses as noted
Lymphatic: No palpable lymphadenopathy

Initial Laboratory Results:

WBC: 38.5 10/L (Reference: 4.5-11.0 10/L)
Hemoglobin: 8.2 g/dL (Reference: 12.0-15.5 g/dL)
Platelets: 26 10/L (Reference: 150-450 10/L)
Peripheral blood smear: 45% blasts
LDH: 620 U/L (Reference: 135-225 U/L)
Comprehensive metabolic panel:

Sodium: 138 mEq/L
Potassium: 4.2 mEq/L
Chloride: 102 mEq/L
Bicarbonate: 24 mEq/L
BUN: 18 mg/dL
Creatinine: 0.9 mg/dL (eGFR >60 mL/min/1.73m)
Glucose: 142 mg/dL
AST: 42 U/L (Reference: 10-40 U/L)
ALT: 38 U/L (Reference: 7-56 U/L)
Alkaline phosphatase: 110 U/L (Reference: 45-115 U/L)
Total bilirubin: 1.1 mg/dL (Reference: 0.3-1.2 mg/dL)
Albumin: 3.7 g/dL (Reference: 3.5-5.0 g/dL)
Calcium: 9.0 mg/dL

Question 1:
Based on Sarah's initial presentation and laboratory findings, what is the most appropriate next diagnostic test?
A) Flow cytometry of peripheral blood
B) Bone marrow aspiration and biopsy with cytogenetics and molecular testing
C) CT scan of chest, abdomen, and pelvis
D) Lumbar puncture for cerebrospinal fluid analysis
B
Correct! Bone marrow aspiration and biopsy with cytogenetics and molecular testing is the most appropriate next step.
Feedback:
You correctly identified that a bone marrow evaluation is essential for diagnosing AML. While the peripheral blood findings (45% blasts) are highly suggestive of AML, the bone marrow examination is required for definitive diagnosis according to the WHO criteria, which defines AML as 20% myeloid blasts in the bone marrow or peripheral blood.
The bone marrow sample will allow for:
Morphologic assessment to confirm the diagnosis
Cytogenetic analysis to identify chromosomal abnormalities

Molecular testing for mutations with prognostic significance (e.g., FLT3, NPM1, CEBPA, IDH1/2)
Immunophenotyping by flow cytometry to characterize the leukemic blasts
Why other options are incorrect:
Flow cytometry of peripheral blood (A) can provide valuable information but is insufficient alone for diagnosis and risk stratification.
CT scan (C) is not indicated as an initial diagnostic test for suspected AML.
Lumbar puncture (D) is not routinely performed during initial diagnosis of AML unless there are neurologic symptoms suggesting CNS involvement.
Let's proceed with the bone marrow results.
Question 2:
Based on the WHO 2016/2022 classification, what is the most accurate diagnosis for Sarah?
A) AML with t(8;21)(q22;q22.1);RUNX1-RUNX1T1
B) AML with inv(16)(p13.1q22) or t(16;16)(p13.1;q22);CBFB-MYH11
C) AML with mutated NPM1
D) AML with myelodysplasia-related changes
B
Correct! AML with inv(16)(p13.1q22) or t(16;16)(p13.1;q22);CBFB-MYH11 is the most accurate diagnosis.
Feedback:
You correctly identified that the presence of inv(16)(p13.1q22) in the cytogenetic analysis classifies this case as "AML with inv(16)(p13.1q22) or t(16;16)(p13.1;q22);CBFB-MYH11" according to the WHO classification.
This is a specific genetic subtype of AML known as core-binding factor (CBF) leukemia.
The diagnosis is based on the presence of this cytogenetic abnormality regardless of blast percentage or other findings.
The presence of Auer rods is consistent with myeloid lineage, supporting the diagnosis.
Why other options are incorrect:
AML with t(8;21)(q22;q22.1);RUNX1-RUNX1T1 (A) is another core-binding factor leukemia but has a different genetic abnormality than seen in this case.
AML with mutated NPM1 (C) is incorrect as molecular testing showed NPM1 was negative.
AML with myelodysplasia-related changes (D) would be considered if there were specific MDS-related cytogenetic abnormalities or prior history of MDS, which are not present here. While dysplastic changes were noted in the marrow, the presence of inv(16) defines the disease.
Question 3:
According to the 2022 ELN risk stratification criteria, what risk category does Sarah's AML fall into?
A) Favorable risk
B) Intermediate risk
C) Adverse risk
D) Very high risk
A
Correct! AML with inv(16)(p13.1q22) or t(16;16)(p13.1;q22) is classified as favorable risk according to the 2022 ELN risk stratification criteria.
Feedback:

The presence of inv(16) is one of the defining features of favorable risk AML in the ELN 2022 classification
Core-binding factor leukemias, including those with inv(16) or t(8;21), generally have a better prognosis than other AML subtypes
The absence of adverse mutations (such as TP53, ASXL1, or RUNX1) further supports the favorable classification
The KIT mutation that was detected (exon 8) does have some negative prognostic impact in core-binding factor leukemias, but it does not change the overall favorable risk category according to current ELN guidelines

Why other options are incorrect:

Intermediate risk (B) would apply to cases with normal karyotype and specific molecular mutations (e.g., NPM1 mutation without FLT3-ITD)
Adverse risk (C) requires specific high-risk cytogenetic abnormalities or mutations which are not present
Very high risk (D) is not a category in the 2022 ELN classification system
Question 4:
Based on Sarah's diagnosis and comorbidities, which of the following tests would be most important to perform before initiating

induction therapy?
A) HLA typing
B) Echocardiogram
C) Pulmonary function tests
D) DEXA scan
B

Correct! An echocardiogram is the most important test to perform before initiating induction therapy for Sarah.
Feedback:
You correctly identified that cardiac function assessment is critical before starting intensive chemotherapy for AML.
Sarah has two cardiovascular risk factors: hypertension and diabetes mellitus, which increase her risk of anthracycline-induced cardiotoxicity.
Anthracyclines (e.g., daunorubicin, idarubicin) are typically included in standard induction regimens for AML and can cause both acute and chronic cardiotoxicity.
A baseline assessment of left ventricular ejection fraction (LVEF) via echocardiogram is essential to determine if Sarah can safely receive standard doses of anthracyclines.
Why other options are incorrect:
HLA typing (A) would be important if allogeneic stem cell transplantation is being considered, but this is not the most immediate concern before starting induction therapy.
Pulmonary function tests (C) may be useful before certain therapies or stem cell transplantation but are not routinely required before standard induction chemotherapy in the absence of significant pulmonary disease.
DEXA scan (D) for bone density is not a priority test before initiating AML treatment.
Echocardiogram Results:
Left ventricular ejection fraction (LVEF): 58% (within normal range)
No significant valvular abnormalities
Mild left ventricular hypertrophy, consistent with history of hypertension
Normal right ventricular function
No pericardial effusion
Question 5:
Sarah is concerned about her long-term prognosis. Based on her age, comorbidities, and disease characteristics, which statement most accurately describes her anticipated outcome with standard treatment?
A) 5-year overall survival of approximately 15-20%
B) 5-year overall survival of approximately 30-40%
C) 5-year overall survival of approximately 55-65%
D) 5-year overall survival of approximately 80-90%
C
Correct! A 5-year overall survival of approximately 55-65% most accurately describes Sarah's anticipated outcome with standard treatment.
Feedback:

You correctly identified the appropriate survival range for a patient with favorable-risk AML based on ELN criteria.
Patients with core-binding factor AML [inv(16) or t(8;21)] typically have better outcomes than those with intermediate or adverse-risk disease.
Several factors influence this prognosis:

Favorable cytogenetics [inv(16)] is the strongest positive prognostic factor
The KIT mutation does reduce the favorable prognosis somewhat but not dramatically
Sarah's age (62) is a modest negative factor compared to younger patients
Her comorbidities (hypertension, diabetes) may affect treatment tolerance but are well-controlled

Why other options are incorrect:

15-20% survival (A) is too low and would be more typical for adverse-risk AML in an older patient
30-40% survival (B) would be more consistent with intermediate-risk disease in her age group
80-90% survival (D) is overly optimistic even for favorable-risk disease, particularly given her age
AML Clinical Case: Phase 2 - Induction Therapy and Supportive Care
Learning Objectives:

Select appropriate induction therapy based on patient-specific factors and molecular profile
Develop comprehensive supportive care strategies
Anticipate and manage common complications of intensive chemotherapy
Interpret post-induction assessment results

Case Continuation:
Sarah's workup has been completed, and she is now ready to begin treatment for her newly diagnosed AML with inv(16). Her ECOG performance status is 1, and her organ function tests are within acceptable parameters for intensive chemotherapy.
Question 6:
Which of the following induction regimens would be most appropriate for Sarah based on her diagnosis, age, and comorbidities?
A) Standard "7+3" regimen (cytarabine 100-200 mg/m continuous infusion for 7 days plus daunorubicin 60 mg/m for 3 days)
B) Venetoclax plus azacitidine
C) CPX-351 (liposomal daunorubicin and cytarabine)
D) Low-dose cytarabine plus glasdegib
A
Correct! Standard "7+3" regimen is the most appropriate induction therapy for Sarah.
Feedback:

You correctly identified that standard intensive chemotherapy with "7+3" is the preferred approach for patients with favorable-risk AML who are fit for intensive therapy.
The ESMO guidelines recommend intensive induction chemotherapy for patients with favorable-risk disease who can tolerate this approach.
Sarah is considered fit for intensive chemotherapy based on her:

Age (62 is not considered prohibitively elderly for intensive therapy)
Good performance status (ECOG 1)
Adequate organ function (normal LVEF, preserved renal function)
Well-controlled comorbidities

Why other options are incorrect:

Venetoclax plus azacitidine (B) is typically reserved for patients who are unfit for intensive chemotherapy or those 75 years old.
CPX-351 (C) is FDA-approved specifically for therapy-related AML or AML with myelodysplasia-related changes, which Sarah does not have.
Low-dose cytarabine plus glasdegib (D) is a less intensive regimen for patients who cannot tolerate standard induction but may benefit from treatment beyond best supportive care.
Question 7:
Sarah is scheduled to begin induction chemotherapy tomorrow. Which of the following supportive care measures should be implemented prior to starting therapy?
A) TLS prophylaxis with allopurinol, aggressive hydration, and electrolyte monitoring
B) Granulocyte colony-stimulating factor (G-CSF) administration starting on day 1 of chemotherapy
C) Prophylactic platelet transfusions to maintain counts >50,000/L
D) Therapeutic anticoagulation with low molecular weight heparin
A
Correct! TLS prophylaxis with allopurinol, aggressive hydration, and electrolyte monitoring is the appropriate supportive care measure to implement prior to starting therapy.

Feedback:

You correctly identified that tumor lysis syndrome (TLS) prophylaxis is essential before initiating intensive chemotherapy for AML. Sarah is at risk for TLS because of:

High peripheral blast count (WBC 38.5 10/L with 45% blasts)
Elevated LDH (620 U/L)
The rapid cell death expected with intensive chemotherapy

Standard TLS prophylaxis includes:

Allopurinol to prevent uric acid nephropathy
IV hydration (typically 2-3 L/day) to maintain good urine output
Frequent monitoring of electrolytes (K, Ca, PO) and renal function
Correction of electrolyte abnormalities

Why other options are incorrect:

G-CSF administration (B) is not routinely recommended during induction as it could potentially stimulate leukemic cell growth and may be associated with prolonged neutropenia.
Prophylactic platelet transfusions (C) are typically given at a threshold of <10,000/L in non-bleeding patients rather than <50,000/L.
Therapeutic anticoagulation (D) is not routinely indicated unless there is a specific indication such as documented thrombosis.
Treatment Course:
Sarah begins standard "7+3" induction therapy with cytarabine 100 mg/m continuous IV infusion for 7 days and daunorubicin 60 mg/m IV on days 1-3. She receives appropriate TLS prophylaxis and other supportive care measures.
On day 10 post-induction initiation, she develops:

Temperature: 101.8F
HR: 110 bpm
BP: 100/60 mmHg
RR: 22/min
SpO: 94% on room air
WBC: 0.2 10/L (ANC <100/L)
Hemoglobin: 7.8 g/dL
Platelets: 12 10/L
No obvious source of infection on initial examination

Question 8:
What is the most appropriate initial management for Sarah's current condition?
A) Obtain blood cultures, start empiric antibiotics with cefepime, and administer G-CSF
B) Obtain blood cultures, start empiric antibiotics with cefepime, and administer granulocyte transfusion
C) Obtain blood cultures, start empiric vancomycin plus meropenem, and obtain CT scan of chest, abdomen, and pelvis
D) Obtain blood cultures, start empiric antibiotics with cefepime or equivalent, and ensure adequate monitoring
D
Correct! The most appropriate initial management is to obtain blood cultures, start empiric antibiotics with cefepime or equivalent, and ensure adequate monitoring.
Feedback:

You correctly identified the standard approach for febrile neutropenia in a hemodynamically stable patient receiving chemotherapy for AML.
Sarah is presenting with febrile neutropenia (temperature >101F and ANC <100/L), a medical emergency requiring prompt

intervention.
The initial management should include:

Blood cultures (at least two sets from different sites)
Prompt initiation of empiric broad-spectrum antibiotics with antipseudomonal coverage (e.g., cefepime, piperacillin-tazobactam, or meropenem)
Close monitoring of vital signs and clinical status
Appropriate laboratory monitoring

Why other options are incorrect:

Adding G-CSF (A) during induction therapy is not routinely recommended and has not been shown to improve outcomes in febrile neutropenia during AML induction.
Granulocyte transfusions (B) are rarely used and would not be indicated as first-line therapy for uncomplicated febrile neutropenia.
Empiric vancomycin plus meropenem with CT imaging (C) is overly aggressive for initial management when there are no signs of hemodynamic instability, suspected resistant organisms, or specific organ involvement.
Question 9:
Two weeks after completing induction therapy, Sarah's blood counts begin to recover. Her WBC is 2.8 10/L with normal differential, hemoglobin 9.3 g/dL, and platelets 112 10/L. What is the most appropriate next step in her management?
A) Proceed directly to consolidation therapy with high-dose cytarabine
B) Perform bone marrow aspiration and biopsy to assess remission status
C) Begin maintenance therapy with oral azacitidine
D) Refer for allogeneic stem cell transplantation evaluation
B
Correct! Performing a bone marrow aspiration and biopsy to assess remission status is the most appropriate next step.
Feedback:

You correctly identified that remission assessment is essential after count recovery following induction therapy.
This timing (approximately day 28-35 after starting induction) is appropriate for assessing response, as blood counts have begun to recover.
The bone marrow evaluation will:

Determine if morphologic complete remission has been achieved (<5% blasts in bone marrow)
Allow for cytogenetic assessment to confirm resolution of inv(16)
Enable measurement of measurable residual disease (MRD) by flow cytometry or molecular methods
Guide decisions about further therapy

Why other options are incorrect:

Proceeding directly to consolidation (A) without confirming remission status would be inappropriate; treatment decisions depend on response assessment.
Maintenance therapy with oral azacitidine (C) is not standard for AML with inv(16) in first remission.
Referring for allogeneic transplant evaluation (D) would be premature without knowing her remission status, and transplant is not typically recommended in first remission for favorable-risk AML that achieves MRD-negative status.

Bone Marrow Biopsy Results (Day 32):

Normocellular marrow (50% cellularity)
2% blasts by morphology
Normal maturation of all cell lines

Flow cytometry: No evidence of abnormal myeloid blast population
Cytogenetics: 46,XX [20/20 cells], no evidence of inv(16)
Molecular MRD testing: CBFB-MYH11 transcript detectable at low level (0.1%)
Question 10:
Based on these results, what is the appropriate interpretation of Sarah's disease status?
A) Primary refractory AML
B) Morphologic complete remission with MRD positivity
C) Morphologic complete remission with MRD negativity
D) Early relapsed AML
B
Correct! The appropriate interpretation is morphologic complete remission with MRD positivity.
Feedback:

You correctly identified that Sarah has achieved morphologic complete remission (CR) but still has measurable residual disease (MRD).
Morphologic CR is defined as <5% blasts in the bone marrow with recovery of blood counts (ANC >1,000/L and platelets >100,000/L), which Sarah has achieved.
However, she still has detectable CBFB-MYH11 fusion transcripts at 0.1% by molecular testing, indicating the presence of MRD.
MRD positivity indicates that leukemic cells are still present below the threshold of detection by conventional morphology and is an important prognostic indicator.

Why other options are incorrect:

Primary refractory AML (A) refers to failure to achieve CR after induction therapy (typically >5% blasts or persistent cytogenetic abnormalities).
Morphologic CR with MRD negativity (C) would require undetectable MRD by sensitive molecular or flow cytometry methods.
Early relapsed AML (D) would involve reappearance of leukemia after having achieved a remission, which is not the case here.
AML Clinical Case: Phase 3 - Post-Remission Therapy and Long-Term Management
Learning Objectives:

Determine appropriate consolidation therapy based on remission status and MRD results
Interpret molecular monitoring during follow-up
Manage treatment-related complications
Develop long-term surveillance and survivorship plans

Case Continuation:
Sarah has completed induction therapy and achieved morphologic complete remission with low-level MRD positivity (CBFB-MYH11 at 0.1%). She has recovered well from induction therapy and is ready to begin post-remission therapy.
Question 11:
What is the most appropriate consolidation therapy for Sarah based on her risk category, remission status, and MRD findings?
A) 3-4 cycles of high-dose cytarabine (HiDAC)
B) Allogeneic hematopoietic stem cell transplantation
C) Low-intensity maintenance with venetoclax and azacitidine
D) A clinical trial evaluating targeted therapy for KIT-mutated CBF leukemia
A
A) 3-4 cycles of high-dose cytarabine (HiDAC)
Feedback:

Patients with favorable-risk AML, specifically core-binding factor leukemia with inv(16), typically receive 3-4 cycles of high-dose cytarabine as consolidation therapy rather than proceeding to allogeneic stem cell transplantation in first remission.
According to the ESMO guidelines, the presence of low-level MRD positivity (0.1%) after induction in CBF-AML is not an immediate indication for transplant, as many patients with CBF-AML will clear MRD during consolidation cycles.
The standard consolidation approach for favorable-risk AML with inv(16) is 3-4 cycles of high-dose cytarabine, which has been shown to produce durable remissions in this patient population.

The KIT mutation does confer some negative impact on prognosis but does not typically change the recommendation from HiDAC to transplant in first remission.

Why other options are incorrect:

Allogeneic stem cell transplantation (B) would be considered excessive for favorable-risk AML in first remission with low-level MRD. Transplant is usually reserved for high-risk disease, persistent MRD after consolidation, or relapsed disease.
Low-intensity maintenance with venetoclax and azacitidine (C) is not standard for favorable-risk AML in first remission.
While a clinical trial (D) could be considered, standard HiDAC consolidation remains the evidence-based approach unless a specific trial has demonstrated superior outcomes.
Question 12:
Sarah receives her first cycle of high-dose cytarabine consolidation (3 g/m over 3 hours q12h on days 1, 3, and 5). On day 4, she reports blurred vision and difficulty reading. What is the most appropriate management?
A) Continue cytarabine as scheduled with frequent ophthalmologic monitoring
B) Administer high-dose corticosteroid eye drops and continue therapy
C) Immediately discontinue cytarabine and consult ophthalmology
D) Reduce the cytarabine dose by 50% for remaining doses
C
You correctly identified that Sarah is experiencing cytarabine-induced ocular toxicity (cerebellar toxicity), which is a serious adverse effect requiring immediate intervention.
High-dose cytarabine can cause a spectrum of ocular toxicities, including conjunctivitis, keratitis, and corneal toxicity.
Blurred vision and reading difficulties are consistent with corneal toxicity, which can progress to more severe and potentially permanent visual impairment if the drug is continued.
The standard approach is to:

Immediately discontinue cytarabine
Obtain urgent ophthalmology consultation
Consider prophylactic corticosteroid eye drops (after discontinuation)
Monitor closely for resolution of symptoms

Why other options are incorrect:

Continuing cytarabine with monitoring (A) could lead to worsening ocular toxicity and permanent damage.
Corticosteroid eye drops while continuing therapy (B) is insufficient; the offending agent must be discontinued.
Dose reduction (D) is inadequate for managing established ocular toxicity; complete discontinuation is necessary.
Question 13:
What is the most appropriate follow-up monitoring plan for Sarah after completion of therapy?
A) Monthly CBC for 1 year, then every 3 months indefinitely
B) CBC and physical examination every 3 months for 2 years, then every 6 months for 3 years; molecular monitoring every 3 months for 2 years, then every 6 months for 3 years
C) Bone marrow examination with molecular testing every 3 months for 5 years
D) CBC monthly and molecular monitoring every 3 months for 1 year, then only as clinically indicated
B
Correct! CBC and physical examination every 3 months for 2 years, then every 6 months for 3 years with molecular monitoring every 3 months for 2 years, then every 6 months for 3 years is the most appropriate follow-up plan.

Why other options are incorrect:

Monthly CBC for a year then every 3 months indefinitely (A) is too frequent initially and unnecessarily prolonged at that frequency.
Bone marrow examination every 3 months for 5 years (C) is overly invasive when peripheral blood molecular monitoring is available.
CBC monthly and molecular monitoring for only 1 year (D) is insufficient given that relapses can occur beyond the first year.
Question 14:
Two years after completing therapy, Sarah's routine molecular monitoring shows reappearance of CBFB-MYH11 transcripts at 0.5%. Her CBC is normal with no blasts on peripheral blood smear. What is the most appropriate next step?

A) Repeat molecular testing in 2-4 weeks to confirm the finding
B) Immediately restart induction chemotherapy with "7+3"
C) Perform bone marrow aspiration and biopsy to assess for morphologic relapse
D) Begin venetoclax plus a hypomethylating agent
C) Perform bone marrow aspiration and biopsy to assess for morphologic relapse.

Why other options are incorrect:

Repeating molecular testing in 2-4 weeks (A) would delay appropriate evaluation and potentially allow disease progression.
Immediately restarting induction chemotherapy (B) without confirming the relapse and assessing its extent would be premature.
Beginning venetoclax plus a hypomethylating agent (D) without confirming relapse and evaluating disease characteristics would not be appropriate.
Question 15:
The bone marrow biopsy confirms relapsed AML with 15% blasts and return of inv(16). Given Sarah's favorable-risk disease that relapsed after completing standard therapy, what is now the most appropriate treatment approach?
A) Repeat induction with standard "7+3" followed by allogeneic stem cell transplantation if second remission is achieved
B) Gemtuzumab ozogamicin as a single agent
C) Venetoclax plus azacitidine
D) Palliative care and transfusion support as needed
A
Correct! Repeat induction with standard "7+3" followed by allogeneic stem cell transplantation if second remission is achieved is the most appropriate treatment approach.

Session ID:
Assessment Domain

Session-15:
Rationale

Session ID:
Instructional Design and Pedagogical

Session-15:
The session demonstrates exceptional instructional design across all dimensions. Learning objectives are explicitly stated at the beginning of each phase, are measurable, and perfectly align with the content delivered. The case is expertly scaffolded, progressing seamlessly from initial presentation through diagnostic workup, treatment planning, complications management, and long-term follow-up. The embedded feedback is outstanding - each rationale provides detailed explanations grounded in current clinical guidelines (WHO 2016/2022, ELN 2022, ESMO), includes specific clinical reasoning, and systematically explains why incorrect options are inappropriate. The instructional framing is exceptionally clear with intuitive phase divisions and unambiguous question stems.

Session ID:
Clinical Authenticity and Content Fidelity

Session-15:
The clinical authenticity is exemplary throughout. The narrative presents a highly realistic case of a 62-year-old woman with AML, featuring appropriate presenting symptoms, laboratory findings, and disease progression that would be indistinguishable from an authentic clinical encounter. All therapeutic decisions are perfectly aligned with current evidence-based guidelines, including WHO classification criteria, ELN risk stratification, and ESMO treatment recommendations. The pharmacotherapeutic choices are optimal and well-justified: standard 7+3 induction for favorable-risk AML, appropriate supportive care measures, HiDAC consolidation for inv(16) leukemia, and evidence-based follow-up protocols. Importantly, the case maintains strict domain specificity without any inappropriate application of treatments from other hematologic malignancies or disease contexts.

Session ID:

Modeled Clinical Reasoning

Session-15:

The simulation excellently models expert-level clinical reasoning throughout the patient care continuum. Each clinical scenario presents well-defined problems with appropriate contextual information for decision-making. The multiple-choice options demonstrate excellent breadth, incorporating relevant alternatives and common distractors that effectively test nuanced clinical understanding. The prioritization demonstrates sound clinical judgment, appropriately sequencing diagnostic workup, risk stratification, treatment selection, and monitoring strategies. The modeled clinical outcomes are realistic and highly instructive, showing both expected responses to therapy and realistic complications (such as cytarabine-induced ocular toxicity) that reinforce important learning points about treatment monitoring and management.

Session-16

Session ID:
LLM

Session-16:
Claude 3.7 Sonnet

Session ID:
Session Transcript

Session-16:
Phase 1: Initial Presentation and Diagnostic Workup.
A 62-year-old female presents to the hospital with fatigue, occasional bruising, and recurrent low-grade fever for the past month. Her initial laboratory results show:

WBC: 3,200/L (low)
Hemoglobin: 9.1 g/dL (low)
Platelet count: 45,000/L (low)
Peripheral blood smear: Presence of blasts

Question 1: What is the most appropriate next diagnostic step to confirm Acute Myeloid Leukemia (AML)?
A) Complete blood count (CBC) with differential
B) Bone marrow biopsy and aspiration with cytogenetic and molecular testing
C) Chest X-ray
D) Lumbar puncture
E) Positron emission tomography (PET) scan

Answer: B) Bone marrow biopsy and aspiration with cytogenetic and molecular testing
Rationale: A bone marrow biopsy is the gold standard for diagnosing AML. This procedure allows for:

Comprehensive assessment of blast percentage
Cytogenetic analysis to identify chromosomal abnormalities
Molecular testing to detect specific genetic mutations
Determination of AML subtype according to WHO 2016/2022 classification

The patient's current presentation (cytopenias, low WBC, presence of blasts) strongly suggests a need for definitive bone marrow evaluation. Cytogenetic and molecular testing are crucial for:

Risk stratification
Treatment selection
Prognosis determination

Additional context: The 2022 European LeukemiaNet (ELN) guidelines emphasize the importance of comprehensive molecular profiling at diagnosis to guide therapeutic decisions.
Bone marrow biopsy results reveal:
Blast percentage: 48% of nucleated cells
Cytogenetics: Normal karyotype (46,XX)
Molecular testing:
NPM1 mutation: Positive
FLT3-ITD: Negative
CEBPA: Biallelic mutation

Question 2: Based on these results, how would you classify the patient's AML risk stratification according to the 2022 European LeukemiaNet (ELN) criteria?
A) Favorable risk
B) Intermediate risk
C) Adverse risk
D) Very low risk
E) Unable to determine
Answer: A) Favorable risk
Rationale: The patient's molecular profile places her in the favorable risk category based on the 2022 ELN guidelines:

NPM1 mutation (positive)
Biallelic CEBPA mutation
Normal karyotype
Absence of FLT3-ITD

These molecular characteristics are associated with:

Better treatment response
Improved overall survival
Potentially different treatment approaches compared to other risk groups

This favorable risk classification is important for:

Treatment selection
Prognosis counseling
Potential consideration for clinical trials or specific therapeutic strategies

The combination of NPM1 mutation and biallelic CEBPA mutation is particularly significant, as it represents a subset of AML patients with a more optimistic clinical outlook.
The patient's clinical team is now considering initial treatment options. Her performance status is ECOG 1, and she has no significant comorbidities.
Question 3: What is the most appropriate first-line induction therapy for this 62-year-old patient with favorable-risk AML?
A) Standard 7+3 cytarabine and daunorubicin
B) High-dose cytarabine (HiDAC)
C) CPX-351 (liposomal cytarabine/daunorubicin)
D) Venetoclax-based combination therapy
E) Azacitidine as a single agent
Answer: A) Standard 7+3 cytarabine and daunorubicin
Rationale: For this 62-year-old patient with favorable-risk AML and an ECOG performance status of 1, the standard 7+3 induction regimen remains the first-line treatment of choice.
Key considerations:

Cytarabine (7 days) and daunorubicin (3 days) is the standard intensive chemotherapy for fit patients with AML
Her favorable molecular profile (NPM1 mutation, biallelic CEBPA) suggests good responsiveness to standard chemotherapy
At 62, she is young enough to tolerate standard intensive chemotherapy
The 7+3 regimen offers:

High complete remission rates
Established efficacy in favorable-risk AML
Well-understood toxicity profile

Dosing considerations:

Standard dose: Cytarabine 100 mg/m continuous infusion for 7 days

Daunorubicin 60-90 mg/m for 3 days

Monitoring will be crucial during this treatment to manage potential side effects and assess treatment response.
During the induction chemotherapy, the patient develops significant neutropenia.
Question 4: What is the most appropriate approach to antimicrobial prophylaxis for this patient during neutropenic period?
A) No prophylaxis needed
B) Oral fluconazole for fungal prophylaxis
C) Ciprofloxacin for bacterial prophylaxis
D) Acyclovir for viral prophylaxis
E) Combination of oral fluconazole and ciprofloxacin
Answer: E) Combination of oral fluconazole and ciprofloxacin
Rationale: During intensive AML induction chemotherapy, patients are at high risk for infections due to prolonged neutropenia. The combination prophylaxis approach is recommended to provide comprehensive protection:
Fluconazole benefits:
Antifungal coverage
Prevents invasive fungal infections
Particularly important during neutropenic periods
Targets Candida species

Ciprofloxacin benefits:
Broad-spectrum antibacterial coverage
Prevents gram-negative bacterial infections
Reduces risk of infection during neutropenic state

Additional considerations:
Prophylaxis is standard of care during intensive chemotherapy
Helps prevent life-threatening infections
Reduces hospitalization risks
Tailored to patient's specific risk factors

Clinical guideline alignment:
Consistent with NCCN and ASCO recommendations for AML supportive care
Balances infection prevention with minimal additional toxicity
During the induction phase, the patient develops tumor lysis syndrome (TLS) laboratory abnormalities.

Question 5: What is the most critical initial pharmacologic intervention for managing acute tumor lysis syndrome in this patient?
A) Hydroxyurea
B) Allopurinol
C) Rasburicase
D) Sodium bicarbonate
E) Furosemide
Answer: C) Rasburicase
Rationale: Rasburicase is the most critical initial pharmacologic intervention for managing acute tumor lysis syndrome (TLS) in this patient due to its unique mechanism of action:
Key Benefits:
Rapidly converts uric acid to allantoin
Quickly reduces existing uric acid levels
More effective than allopurinol in acute TLS situation
Prevents uric acid-related renal complications

Clinical Considerations:
Recommended for patients at high risk of TLS
Particularly important during initial chemotherapy
Prevents acute kidney injury

Rapidly addresses hyperuricemia

Mechanism of Action Highlights:
Recombinant urate oxidase enzyme
Catalyzes oxidation of uric acid to allantoin
Allantoin is highly soluble and easily excreted
Provides immediate uric acid reduction

Dosing Typically:
Single intravenous dose
Weight-based calculation
Administered before or at the start of chemotherapy
Following induction chemotherapy, the patient's bone marrow results show:
-Blast percentage: 3% (significantly reduced)
-Minimal Residual Disease (MRD) testing performed

Question 6: How would you interpret the Minimal Residual Disease (MRD) results for this patient with favorable-risk AML?
A) MRD is not clinically relevant in this patient
B) Any detectable MRD indicates immediate relapse
C) MRD negativity is crucial for long-term remission and treatment decisions
D) MRD testing is only useful in adverse-risk AML
E) MRD should be repeated only after complete treatment
Answer: C) MRD negativity is crucial for long-term remission and treatment decisions
Rationale: In AML, particularly for patients with favorable molecular risk profiles, MRD is a critical prognostic marker:
Key Points:

-MRD testing provides more sensitive assessment than morphological evaluation
Negative MRD is associated with:
Improved overall survival
Lower relapse risk
Better long-term treatment outcomes

-For NPM1-mutated AML, MRD can be assessed through:
NPM1 mutation-specific molecular tracking
Highly sensitive PCR techniques

-Clinical Significance:
Helps guide post-remission therapy decisions
Determines the need for:
Additional consolidation therapy
Potential stem cell transplantation
Closer follow-up monitoring

-Specific to this Patient:
Favorable risk profile (NPM1 and biallelic CEBPA mutations)
Low blast percentage post-induction
MRD results will be crucial in determining next treatment steps
Question 7: Considering the patient's favorable-risk AML and MRD status, what is the most appropriate post-remission therapy recommendation?
A) Observation only
B) Allogeneic stem cell transplantation
C) High-dose cytarabine (HiDAC) consolidation
D) Venetoclax maintenance therapy
E) Low-dose azacitidine maintenance

Answer: D) Venetoclax maintenance therapy
Rationale: Venetoclax maintenance is an emerging and promising strategy for AML patients, particularly those with favorable molecular risk features:
Key Benefits:
Targeted approach to prevent relapse
Mechanism targets BCL-2 protein to induce apoptosis in residual leukemic cells
Particularly effective in NPM1-mutated AML
Less toxic than traditional intensive consolidation

Clinical Considerations:
Supports extended disease control
Provides a more tolerable maintenance strategy
Aligns with precision medicine approach
Reduces risk of relapse while maintaining quality of life

Specific to this Patient:
Favorable risk profile makes her an excellent candidate
62 years old - benefits from less intensive approach
NPM1 mutation suggests potential enhanced responsiveness
Lower toxicity compared to traditional chemotherapy consolidation

Emerging Evidence:
Recent clinical trials show promising results in maintenance therapy
Particularly effective in preventing early relapse
Supports molecular-targeted approach to AML management

Question 8: Which follow-up monitoring strategy is most appropriate for this patient after completing venetoclax maintenance therapy?
A) No specific follow-up required
B) Annual bone marrow biopsy
C) Quarterly molecular monitoring with sensitive MRD testing
D) Monthly complete blood count (CBC)
E) PET scan every 6 months

Answer: C) Quarterly molecular monitoring with sensitive MRD testing
Rationale: For patients with favorable-risk AML, particularly those with NPM1 mutations, quarterly molecular monitoring is crucial:
Key Benefits:
Early detection of molecular relapse
More sensitive than traditional morphological assessment
Allows for rapid intervention if MRD becomes detectable
Tracks NPM1 mutation levels precisely

Monitoring Strategy:
Sensitive PCR-based MRD testing
Quantitative assessment of leukemic markers
Typically performed every 3-4 months
Can detect minimal disease before clinical symptoms appear

Specific Considerations:
NPM1 mutation serves as an excellent molecular marker
Allows for personalized, targeted follow-up
Consistent with precision medicine approach
Minimizes unnecessary invasive procedures

Clinical Guidelines Alignment:

Recommended by European LeukemiaNet (ELN)
Supports proactive management
Reduces risk of undetected relapse

Phase 3: Post-Remission Therapy and Long-Term Management.
During a follow-up visit, the patient reports mild fatigue and slight bruising. Routine molecular monitoring reveals a low-level NPM1 mutation detection.
Question 9: What is the most appropriate initial management approach for this potential early molecular relapse?
A) Immediate reinitiation of intensive chemotherapy
B) Continuation of current monitoring without intervention
C) Repeat MRD testing in 2-4 weeks to confirm trend
D) Initiate salvage venetoclax therapy
E) Refer for stem cell transplantation consultation
Please select the most appropriate management strategy
Answer: C) Repeat MRD testing in 2-4 weeks to confirm trend
Rationale: In cases of low-level molecular recurrence, immediate aggressive intervention is not always necessary:
Key Considerations:
Low-level molecular detection doesn't guarantee clinical relapse
Requires careful, measured approach
Prevents unnecessary toxic interventions
Allows for trend confirmation

Monitoring Strategy:
Repeat sensitive MRD testing
Compare quantitative NPM1 mutation levels
Assess for consistent upward trend
Rule out potential testing variability

Clinical Reasoning:
Favorable-risk AML patients can have fluctuating MRD levels
Single elevated test may not indicate definitive relapse
Close surveillance is key
Provides time to make informed treatment decisions

Potential Next Steps:
If confirmed upward trend: Consider targeted intervention
If levels stabilize: Continue current monitoring
Personalized approach based on molecular dynamics

Question 10: Considering the patient's age and previous treatment history, what supportive care intervention is most critical during long-term follow-up?
A) Routine cardiac monitoring
B) Comprehensive immunization update
C) Bone density screening
D) Comprehensive metabolic panel every 6 months
E) Psychological support and counseling
Answer: B) Comprehensive immunization update
Rationale: Immunization is crucial for AML survivors, especially after intensive chemotherapy and venetoclax maintenance:
Key Considerations:
-Chemotherapy and targeted therapies compromise immune function
-Increased susceptibility to infectious diseases
-Need to rebuild and protect immune system

Immunization Priorities:
Update routine vaccinations
Assess vaccination history
Recommend:
-Influenza vaccine annually
-Pneumococcal vaccines
-COVID-19 vaccination
-Hepatitis B
-Other age-appropriate vaccinations

Specific Patient Considerations:
-62-year-old patient
-Completed intensive AML treatment
-Potentially compromised immune system
-Risk of vaccine-preventable diseases

Clinical Guidelines Alignment:
-Recommended by NCCN and ASCO survivorship guidelines
-Critical component of post-treatment care
-Reduces infection risks
-Supports long-term health maintenance

Question 11: The patient's follow-up MRD testing confirms a rising trend in NPM1 mutation levels. What pharmacotherapeutic approach is most appropriate for this confirmed molecular relapse?
A) Observation only
B) Low-intensity venetoclax plus azacitidine
C) High-dose cytarabine (HiDAC) reinduction
D) Intensive salvage chemotherapy with fludarabine-based regimen
E) FLT3 inhibitor monotherapy
Answer: B) Low-intensity venetoclax plus azacitidine
Rationale: For molecular relapse in an older patient with favorable-risk AML:
Key Considerations:
Molecular relapse allows for less intensive intervention
Patient age (62) is a factor in treatment tolerance
Previous exposure to intensive chemotherapy
Need to balance efficacy with treatment-related toxicity

Benefits of Venetoclax + Azacitidine:
Targeted approach to eliminate emerging clones
Lower toxicity than intensive reinduction
Effective against NPM1-mutated cells
Synergistic mechanism between agents
Preserves quality of life while addressing disease

Clinical Evidence:
Growing data supporting this combination in relapsed/refractory AML
Particularly effective in NPM1-mutated disease
Better tolerated than intensive salvage regimens
Can achieve molecular remission

Why not HiDAC reinduction (Option C):
More toxic approach
May be premature for molecular-only relapse
Greater risk of treatment-related complications

Potentially unnecessary intensity at this stage

Question 12: Following initiation of venetoclax plus azacitidine, the patient develops significant neutropenia with fever (39.2C). Based on the clinical scenario, what is the most appropriate empiric antimicrobial regimen?
A) Oral ciprofloxacin
B) Vancomycin plus cefepime
C) Single agent piperacillin-tazobactam
D) Fluconazole plus ceftriaxone
E) Levofloxacin monotherapy
Answer: C) Single agent piperacillin-tazobactam
Rationale: For febrile neutropenia management in AML patients receiving venetoclax-based therapy:
Key Benefits:
Broad-spectrum coverage
Effective against both gram-positive and gram-negative organisms
Particularly good Pseudomonas aeruginosa coverage
Contains a beta-lactamase inhibitor to prevent resistance

Clinical Considerations:
Febrile neutropenia requires prompt antibiotic initiation
Monotherapy with an antipseudomonal beta-lactam is standard
Aligns with IDSA and NCCN guidelines for febrile neutropenia
Balance of broad coverage and antimicrobial stewardship

Specific Advantages:
Does not require routine combination with vancomycin
Covers most common pathogens in neutropenic fever
Established efficacy in hematologic malignancy patients
Standard of care in many oncology centers

Dosing Considerations:
Typically 4.5g IV every 6-8 hours
Requires renal function monitoring
Duration based on clinical response and neutrophil recovery
Question 13: After successful management of febrile neutropenia, the patient achieves molecular remission with venetoclax plus azacitidine. Which of the following is most critical for optimizing continued venetoclax therapy?
A) Routine proton pump inhibitor prophylaxis
B) Therapeutic drug monitoring (TDM) of venetoclax levels
C) Drug interaction management, particularly with CYP3A4 inhibitors
D) Mandatory dose reductions based on age
E) Intensive nutritional supplementation
Answer: C) Drug interaction management, particularly with CYP3A4 inhibitors
Rationale: Venetoclax drug interaction management is critical for both safety and efficacy:
Key Considerations:

Venetoclax is extensively metabolized by CYP3A4
Strong and moderate CYP3A4 inhibitors can significantly increase venetoclax levels
Increased concentrations can lead to:

Enhanced toxicity
Greater risk of tumor lysis syndrome
Severe neutropenia
Other dose-dependent adverse effects

Common CYP3A4 Inhibitors to Monitor:
Antifungals (fluconazole, voriconazole, posaconazole)
Certain antibiotics (clarithromycin, erythromycin)
Antiviral agents (ritonavir)
Food products (grapefruit juice)

Management Approach:
Systematic medication reconciliation
Avoidance of unnecessary interacting medications
Dose adjustments when CYP3A4 inhibitors are necessary
Patient education about OTC medications and food interactions
Close monitoring when interacting medications are added or removed

Clinical Guidelines:
Package insert provides specific dose reduction guidance
These adjustments are essential to maintain safety profile

Question 14: The patient's insurance plan is requesting a justification for continued venetoclax plus azacitidine therapy versus switching to a different regimen. Which of the following best supports the clinical rationale for continuing the current regimen?
A) Lowest cost option compared to alternatives
B) Patient preference for oral therapy
C) Achievement of molecular remission with current regimen and favorable tolerability
D) Lack of alternative options for AML relapse
E) Simplicity of administration

Answer: C) Achievement of molecular remission with current regimen and favorable tolerability
Rationale: The strongest clinical justification for continuing venetoclax plus azacitidine is:
Key Clinical Considerations:

Demonstrated efficacy: The patient achieved molecular remission
Proven response: Treatment has already been effective for this specific patient
Established tolerability: Patient has demonstrated ability to manage therapy
Risk of changing therapy: Switching could disrupt disease control

Scientific Support:
Evidence-based approach
Molecularly targeted therapy effective against NPM1 mutations
Clinically meaningful endpoint (molecular remission) achieved
Consistent with current treatment guidelines

Benefit-Risk Profile:
Managed toxicity profile
Ongoing remission status
Personalized response-based approach
Clear clinically relevant endpoints

Additional Context:
Standard insurance coverage criteria often prioritize:
Demonstrated clinical efficacy
Objective response metrics
Patient-specific treatment response
Evidence-based continuation criteria

Question 15: After 12 months of successful therapy, the patient has maintained complete molecular remission. What is the most appropriate long-term survivorship care plan component to focus on at this stage?
A) Cognitive function assessment
B) Secondary malignancy screening
C) Fertility preservation
D) Occupational rehabilitation
E) Palliative care referral

Answer: B) Secondary malignancy screening
Rationale: Secondary malignancy screening is critical for AML survivors, particularly after treatment with cytotoxic agents:
Key Considerations:
AML patients have increased risk of secondary malignancies
Both disease-related and treatment-related risks
Chemotherapy exposure increases long-term cancer risk
Early detection significantly improves outcomes

Screening Approach:
Age-appropriate cancer screenings
Heightened surveillance for:
Myelodysplastic syndrome (MDS)
Skin cancers
Solid tumors
Earlier and more frequent screening than general population

Specific to this Patient:
62-year-old with exposure to intensive chemotherapy
Venetoclax and azacitidine exposure
Long-term remission allows focus on survivorship
Age places her at baseline elevated cancer risk

Comprehensive Care:
Requires multidisciplinary approach
Systematic screening schedule
Patient education about signs/symptoms
Collaboration with primary care for ongoing surveillance

Session ID:
Assessment Domain

Session-16:
Rationale

Session ID:
Instructional Design and Pedagogical

Session-16:
The session demonstrates strong instructional design with excellent scaffolding that progresses logically through the complete AML patient journey from diagnosis to survivorship care. The embedded feedback is generally strong, with detailed rationales citing current guidelines (ELN 2022, NCCN, IDSA) and providing valuable clinical context. Instructions are clear and unambiguous throughout. The primary weakness is the absence of explicitly stated learning objectives at the session's onset, though educational goals are clearly implied by the comprehensive content structure.

Session ID:

Clinical Authenticity and Content Fidelity

Session-16:

The session presents a highly realistic clinical narrative that accurately reflects authentic AML patient management. The case maintains strict domain specificity within AML without inappropriate crossover from other hematologic malignancies. However, a significant pharmacotherapeutic error occurs in Question 7, where venetoclax maintenance therapy is recommended as standard first-line post-remission therapy for favorable-risk AML. This recommendation lacks evidence-based supportstandard of care for this population remains high-dose cytarabine (HiDAC) consolidation, not venetoclax maintenance, which remains investigational in this setting. While the reasoning framework is sound, this represents a substantial deviation from established clinical guidelines that could mislead learners about appropriate therapeutic sequencing. In Q 5, Rasburicase was selected for TLS prophylaxis depending on that the case is high risk for TLS, while the case was low risk for TLS and should use allopurinol.

Session ID:

Modeled Clinical Reasoning

Session-16:

The simulation excellently models expert-level clinical reasoning throughout most of the case. Each clinical scenario is expertly framed with appropriate complexity and relevant decision points. The multiple-choice options demonstrate excellent breadth, including plausible distractors that test nuanced understanding of AML management. Clinical outcomes are realistically modeled and instructionally valuable. The prioritization and decision-making score is reduced due to the flawed therapeutic recommendation in Question 7, where the strategic reasoning for post-remission therapy was appropriate, but the specific agent selection (venetoclax maintenance) represents a significant clinical reasoning error that deviates from evidence-based practice standards.

Session-17

Session ID:
LLM

Session-17:
Claude 3.7 Sonnet

Session ID:
Session Transcript

Session-17:
PHASE 1: INITIAL PRESENTATION AND DIAGNOSTIC WORKUP
Case Introduction:
Sarah Johnson is a 67-year-old female who presents to her primary care physician with complaints of persistent fatigue, unexplained weight loss (15 pounds over 3 months), and recurrent low-grade fevers for the past 6 weeks. She has a history of hypertension and type 2 diabetes mellitus, both well-controlled with lisinopril 10mg daily and metformin 1000mg BID. She works as a high school teacher and lives with her husband. She reports difficulty completing a full day of teaching due to her increasing fatigue.
Initial Laboratory Results:

WBC: 28.4 10^9/L (elevated)
Hemoglobin: 8.2 g/dL (decreased)
Platelets: 45 10^9/L (decreased)
Peripheral blood smear: 35% blastsComprehensive metabolic panel: Within normal limits except for LDH at 450 U/L (elevated)

Question 1:
Based on Sarah's initial presentation and laboratory results, what is your most appropriate next step?
A) Immediately start broad-spectrum antibiotics for presumed infection
B) Order a bone marrow biopsy and aspirate with flow cytometry, cytogenetics, and molecular testing
C) Start erythropoietin for anemia management
D) Recommend outpatient follow-up in 2 weeks with repeat CBC
E) Order CT scans of chest, abdomen, and pelvis to rule out solid malignancy
Correct! Ordering a bone marrow biopsy and aspirate with flow cytometry, cytogenetics, and molecular testing is the most appropriate next step.
Why this is correct:
Sarah's presentation with pancytopenia, significant blast percentage on peripheral smear (35%), and constitutional symptoms strongly suggests acute leukemia. According to the ESMO Guidelines, a bone marrow examination with comprehensive diagnostic testing is essential for confirming the diagnosis of AML and obtaining critical prognostic information.
Why other options are incorrect:

A) While infections are common in leukemia patients, empiric antibiotics without confirming the diagnosis would delay proper workup. There are no clear signs of infection requiring immediate antibiotics.
C) Erythropoietin is not indicated whenanemia is suspected to be due to a primary hematologic malignancy.
D) With 35% blasts and severe cytopenias, this is an urgent clinical situation requiring immediate evaluation, not outpatient follow-up.
E) While imaging may eventually be useful, bone marrow examination takes priority for diagnosing suspected acute leukemia.
Bone marrow biopsy results:
The bone marrow biopsy shows hypercellular marrow (90%) with 52% myeloblasts. Flow cytometry confirms AML with positive CD33, CD13, CD117, and MPO. Cytogenetics reveals: 46,XX,inv(16)(p13.1q22). Molecular studies show: KIT mutation positive, NPM1 negative, FLT3-ITD negative.
Question 2:
Based on the 2022 ELN risk classification, what risk category would you assign to Sarah's AML?
A) Favorable risk
B) Intermediate risk

C) Adverse risk
D) Indeterminate risk
The correct answer is B) Intermediate risk.
Explanation:
According to the 2022 ELN risk classification:

The inv(16)(p13.1q22) chromosomal abnormality by itself is classified as favorable risk. This is a core binding factor (CBF) leukemia. However, the presence of a KIT mutation in the setting of inv(16) is an important modifier that shifts the classification to intermediate risk.

The 2022 ELN guidelines specifically addressed this scenario by reclassifying CBF leukemias with KIT mutations from favorable to intermediate risk, based on evidence showing worse outcomes in these patients.
Why other options are incorrect:A) Favorable risk would be correct if there was inv(16) without the KIT mutation
C) Adverse risk includes complex karyotype, monosomal karyotype, or specific high-risk genetic abnormalities not present in this case
D) Indeterminate risk is not a category in the ELN classification system
Additional Information:
Sarah's ECOG performance status is assessed as 1. Her comorbidity assessment (HCT-CI) score is 2 (hypertension and diabetes). Additional laboratory results show normal renal and hepatic function.
Question 3:
Which of the following is the most appropriate induction regimen for Sarah based on her disease characteristics, age, and comorbidities?
A) 7+3 regimen (cytarabine + daunorubicin)
B) Venetoclax + hypomethylating agent (azacitidine)
C) CPX-351 (liposomal daunorubicin and cytarabine)
D) FLAG-IDA (fludarabine, cytarabine, G-CSF, and idarubicin)
E) Best supportive care only
Correct! The 7+3 regimen (cytarabine + daunorubicin) is the most appropriate induction therapy for Sarah.
Why this is correct:
Despite Sarah's age of 67, she has good performance status (ECOG 1), controlled comorbidities, and normal organ function. According to ESMO guidelines, patients with intermediate-risk AML who are fit for intensive chemotherapy should receive standard induction with 7+3, regardless of age. Her inv(16) with KIT mutation represents a core binding factor leukemia (albeit with intermediate risk due to KIT), which typically responds well to intensive chemotherapy.
Why other options are incorrect:

B) Venetoclax + HMA is typically reserved for patients who are unfit for intensivechemotherapy or have adverse risk features. Sarah is fit enough for standard induction.
C) CPX-351 is specifically indicated for therapy-related AML or AML with myelodysplasia-related changes, which Sarah does not have.
D) FLAG-IDA is a more intensive regimen often used for refractory/relapsed disease or high-risk disease, not typically as first-line for this scenario.
E) Best supportive care would be inappropriate given Sarah's fitness for therapy and the potential for long-term remission with her disease characteristics.
Induction Course:
Sarah receives standard 7+3 induction with cytarabine (100 mg/m continuous infusion for 7 days) and daunorubicin (60 mg/m for 3 days). Her post-induction course is complicated by febrile neutropenia on day 9.
Question 4:
Which empiric antimicrobial regimen would be most appropriate for Sarah's febrile neutropenia?
A) Oral ciprofloxacin and amoxicillin-clavulanate
B) Piperacillin-tazobactam
C) Vancomycin plus cefepime
D) Meropenem
E) Fluconazole
Correct! Piperacillin-tazobactam is the most appropriate empiric antimicrobial regimen for Sarah's febrile neutropenia.
Why this is correct:

According to ESMO and IDSA guidelines for managing febrile neutropenia in patients with hematologic malignancies, monotherapy with an anti-pseudomonal beta-lactam such as piperacillin-tazobactam is the recommended first-line empiric therapy. Sarah is presenting with uncomplicated febrile neutropenia without specific risk factors that would warrant a more aggressive approach or specific coverage needs.
Why other options are incorrect:

A) Oral antibiotics are inappropriate for high-risk febrile neutropenia in AML patients undergoing induction chemotherapy.
C) While cefepime would be appropriate as monotherapy, adding vancomycin is notindicated as initial empiric therapy unless there are specific indications (MRSA colonization, skin/catheter infection, hemodynamic instability).
D) Meropenem is typically reserved for escalation of therapy, known colonization with resistant organisms, or severe sepsis.
E) Fluconazole alone would not provide adequate bacterial coverage for febrile neutropenia; antifungals are typically added if fever persists despite appropriate antibacterial therapy.
Further Course:
Sarah's fever resolves after 3 days of piperacillin-tazobactam. On day 28, a bone marrow biopsy shows morphologic remission with 3% blasts. Minimal residual disease (MRD) assessment by flow cytometry shows 0.08% residual leukemic cells.
Question 5:
Based on Sarah's post-induction bone marrow results, which of the following statements about her MRD status is most accurate?
A) Sarah is MRD-negative, indicating excellent prognosis with no need for additional therapy
B) Sarah is MRD-positive, indicating a high likelihood of relapse within 3 months
C) Sarah is MRD-positive, which has prognostic significance and should inform consolidation strategy
D) The MRD result is inconclusive and should be repeated before making further treatment E) MRD status is not relevant in CBF leukemias with KIT mutations
Correct! Sarah is MRD-positive, which has prognostic significance and should inform consolidation strategy.
Why this is correct:
According to ELN and ESMO guidelines, MRD positivity is typically defined as leukemic cells 0.1% by flow cytometry or 0.01% by molecular methods. Sarah's MRD level of 0.08% by flow cytometry is very close to the threshold and is considered positive. MRD positivity after induction is associated with increased relapse risk and shorter overall survival, even in patients with favorable cytogenetics. This information should guide decisions about consolidation intensity and potentially consideration for allogeneic stem cell transplantation.
Why other options are incorrect:

A) Sarah is not MRD-negative; the persistence of measurable disease indicates some risk ofrelapse.
B) While MRD positivity increases relapse risk, it doesn't specifically predict relapse within 3 months.
D) The MRD result is valid and actionable; there's no need to repeat before making treatment decisions.
E) MRD status is particularly relevant in CBF leukemias, as persistence of MRD in this subset indicates a higher risk of relapse despite the generally favorable prognosis.
PHASE 2: INDUCTION THERAPY AND SUPPORTIVE CARE
Sarah will now proceed to consolidation therapy. Given her MRD-positive status and KIT mutation with inv(16), the team must determine the appropriate consolidation approach.
Question 6:
What is the most appropriate consolidation strategy for Sarah?
A) 1-2 cycles of high-dose cytarabine (HiDAC) followed by allogeneic hematopoietic stem cell transplantation (HSCT)
B) 3-4 cycles of high-dose cytarabine (HiDAC) without transplantation
C) Maintenance therapy with a hypomethylating agent
D) Observation only with regular monitoring
E) Venetoclax-based therapy
Correct! 1-2 cycles of high-dose cytarabine (HiDAC) followed by allogeneic hematopoietic stem cell transplantation (HSCT) is the most appropriate consolidation strategy for Sarah.
Why this is correct:
Sarah has several factors that warrant consideration for allogeneic HSCT:

MRD-positive status after induction (0.08%)
KIT mutation in the setting of inv(16), which places her in the intermediate risk category
Age 67 but with good performance status and controlled comorbidities (HCT-CI score of 2)

According to ELN 2022 and ESMO guidelines, patients with intermediate-risk AML and persistent MRD after induction may benefit from allogeneic HSCT. While CBF leukemias typically have favorable outcomes with HiDAC consolidation alone, the presence of both a KIT mutation and MRD positivity significantly increases relapse risk, making transplantation a reasonable approach after 1-2 cycles of HiDAC.

Why other options are incorrect:

B) Given her MRD positivity and KIT mutation, HiDAC alone may be insufficient to prevent relapse.
C) Maintenance with a hypomethylating agent is not standard practice in this setting.
D) Observation would be inappropriate given her positive MRD status.
E) Venetoclax-based therapy is not established as consolidation in this context.

HLA typing results:

Sarah's HLA typing is complete, and her sister is identified as a 10/10 HLA match. Sarah receives one cycle of HiDAC (3 g/m twice daily on days 1, 3, and 5). She develops grade 2 cerebellar toxicity after HiDAC.

Question 7:

What is the most appropriate adjustment to Sarah's subsequent HiDAC consolidation cycle?
A) Proceed with the same HiDAC dose (3 g/m) as cerebellar toxicity is expected and manageable
B) Reduce the HiDAC dose to 1-2 g/m based on her age and prior toxicity
C) Switch to intermediate-dose cytarabine (1 g/m)
D) Discontinue HiDAC and proceed directly to transplant
E) Switch to an alternative agent such as fludarabine

Correct! Reducing the HiDAC dose to 1-2 g/m based on Sarah's age and prior toxicity is the most appropriate adjustment.

Why this is correct:

Cerebellar toxicity is a known dose-limiting toxicity of high-dose cytarabine. According to ESMO guidelines and clinical practice:

Patients who develop grade 2 cerebellar toxicity should have cytarabine dose-reduced in subsequent cycles
Patients over 60 years of age (Sarah is 67) are at higher risk for cytarabine neurotoxicity
The recommended dose reduction for patients >60 years is typically to 1-2 g/m (rather than 3 g/m)
Continuing some form of cytarabine consolidation before transplant is still beneficial

Why other options are incorrect:A) Continuing with the same dose after grade 2 cerebellar toxicity could lead to severe, potentially irreversible neurotoxicity.
C) While intermediate-dose cytarabine is safer, 1-2 g/m is still considered HiDAC and is the standard dose adjustment.
D) Completely discontinuing HiDAC would eliminate the benefit of additional cytoreduction before transplant.
E) Switching to an alternative agent is unnecessary when dose reduction can effectively manage toxicity.

Pre-transplant assessment:

After two cycles of HiDAC (second cycle at reduced dose), Sarah's MRD becomes undetectable (<0.01%). Her performance status remains ECOG 1. Organ function tests show:

LVEF: 55%
DLCO: 72% predicted
Creatinine clearance: 65 mL/min

Question 8:

Which of the following conditioning regimens would be most appropriate for Sarah's allogeneic transplant?
A) Myeloablative conditioning (MAC) with busulfan/cyclophosphamide
B) Reduced-intensity conditioning (RIC) with fludarabine/busulfan
C) Non-myeloablative conditioning with fludarabine/TBI 2 GyD) Myeloablative conditioning with cyclophosphamide/TBI
E) Treosulfan-based conditioning

The correct answer is B) Reduced-intensity conditioning (RIC) with fludarabine/busulfan.

Explanation:

While Sarah has a good performance status, several factors make RIC more appropriate than MAC:

Age: At 67, Sarah is at increased risk for transplant-related mortality with fully myeloablative regimens

Comorbidities: Her HCT-CI score of 2 and slightly reduced organ function (CrCl 65 mL/min, DLCO 72%)
Disease status: She achieved MRD negativity after consolidation, reducing the need for the most intensive conditioning
Donor type: She has a matched sibling donor, which is associated with lower relapse risk compared to alternative donorsAccording to ESMO and EBMT guidelines, RIC is preferred for patients >60-65 years to balance disease control with treatment-related toxicity. The fludarabine/busulfan RIC regimen provides sufficient disease control while minimizing organ toxicity.
Why other options are incorrect:

A) MAC with busulfan/cyclophosphamide would pose excessive toxicity risk in a 67-year-old patient
C) Non-myeloablative conditioning may provide insufficient disease control for AML
D) Cyclophosphamide/TBI is a highly toxic myeloablative regimen not appropriate for older patients
E) While treosulfan-based conditioning can be appropriate, it is not clearly superior to fludarabine/busulfan in this context
Transplantation course:
Sarah undergoes allogeneic HSCT with fludarabine/busulfan RIC conditioning and standard GVHD prophylaxis with tacrolimus and methotrexate. She engrafts on day +15 and develops stage 2 acute skin GVHD on day +30, which responds to topical steroids.
Question 9:
Which of the following is the most appropriate CMV management strategy for Sarah post-transplant, given that both she and her donor are CMV seropositive (R+/D+)?
A) No prophylaxis; monitor CMV PCR weekly and initiate preemptive therapy if CMV reactivation occurs
B) Letermovir prophylaxis from engraftment until day +100
C) High-dose acyclovir prophylaxis from day +1 to day +100
D) Ganciclovir prophylaxis from engraftment until day +100
E) Valganciclovir prophylaxis for 6 months post-transplant
The correct answer is B) Letermovir prophylaxis from engraftment until day +100.
Explanation:
As a CMV-seropositive recipient (R+) of a transplant from a CMV-seropositive donor (D+), Sarah is at high risk for CMV reactivation. According to recent guidelines and clinical practice:

Letermovir prophylaxis is now recommended for CMV-seropositive allogeneic HCT recipients
Letermovir has been shown to significantly reduce the risk of clinically significant CMV infection with minimal toxicity
Prophylaxis is typically continued until day +100, when the risk of CMV reactivation begins to decline
Sarah's acute GVHD further increases her risk of CMV reactivationWhy other options are incorrect:

A) While preemptive strategy was previously standard, prophylaxis is now preferred for high-risk patients
C) High-dose acyclovir has limited efficacy against CMV compared to letermovir
D) Ganciclovir prophylaxis is associated with significant myelosuppression and is rarely used as first-line prophylaxis
E) Valganciclovir is too toxic for routine prophylaxis and is reserved for treatment of established CMV infection
Day +90 assessment:
Sarah's acute GVHD has resolved completely. She has 100% donor chimerism and remains in molecular remission (MRD-negative). She is preparing for discharge from the transplant center to return home.
Question 10:
Which of the following medication regimens would be most appropriate for Sarah at discharge around day +100?
A) Continue tacrolimus at therapeutic levels; acyclovir, TMP-SMX, and fluconazole; start letermovir
B) Begin tacrolimus taper; continue acyclovir, TMP-SMX, and fluconazole; discontinue letermovir
C) Continue tacrolimus at therapeutic levels; discontinue all antimicrobial prophylaxis
D) Rapidly taper and discontinue tacrolimus; continue TMP-SMX onlyE) Continue tacrolimus at therapeutic levels; continue TMP-SMX only
Correct! Beginning tacrolimus taper; continuing acyclovir, TMP-SMX, and fluconazole; and discontinuing letermovir is the most appropriate regimen at discharge.
Why this is correct:
According to standard post-transplant guidelines (EBMT/ESMO):

With no active GVHD at day +100, a slow tacrolimus taper can begin (typically over 2-3 months)
Antimicrobial prophylaxis should continue:

TMP-SMX for Pneumocystis prophylaxis (continue for 6-12 months or until immunosuppression is discontinued)
Acyclovir for HSV/VZV prophylaxis (continue until 1 year post-transplant or longer if still on immunosuppression)
Fluconazole for antifungal prophylaxis (typically continue until day +75-100 withRIC, or longer with ongoing immunosuppression)

Letermovir prophylaxis is typically discontinued at day +100 in the absence of ongoing risk factors

Why other options are incorrect:

A) Continuing tacrolimus at full therapeutic levels is unnecessary without active GVHD; starting letermovir is inappropriate as it should be discontinued at day +100
C) Discontinuing all antimicrobial prophylaxis is premature and would place Sarah at risk for opportunistic infections
D) Rapidly tapering tacrolimus increases risk of GVHD; discontinuing antiviral and antifungal prophylaxis is inappropriate
E) Discontinuing antiviral and antifungal prophylaxis is premature at day +100
PHASE 3: POST-REMISSION THERAPY AND LONG-TERM MANAGEMENT
Six months post-transplant, Sarah has successfully tapered off immunosuppression without evidence of chronic GVHD. She remains in molecular remission. She asks about returning to work as a teacher.
Question 11:
Which of the following recommendations regarding Sarah's return to work is most appropriate?
A) She should not return to teaching due to the high risk of infection in a school environment
B) She can return to teaching immediately with standard precautions
C) She can return to teaching part-time with additional precautions (masking, hand hygiene) and avoiding contact with sick studentsD) She should wait until 1 year post-transplant to consider returning to any work
E) She can return to administrative work but should avoid classroom teaching permanently
Correct! Sarah can return to teaching part-time with additional precautions (masking, hand hygiene) and avoiding contact with sick students.
Why this is correct:
According to survivorship guidelines for HSCT recipients (ASTCT/EBMT):

At 6 months post-transplant with normal immune recovery, patients can begin returning to normal activities with precautions
A gradual return (part-time) helps manage fatigue, which is common after transplant
Specific precautions in high-exposure settings like schools are appropriate:

Consistent hand hygiene
Masking when appropriate (during respiratory virus season or outbreaks)
Avoiding contact with visibly ill individualsSarah's favorable factors include: no chronic GVHD, off immunosuppression, and good recovery

Why other options are incorrect:

A) Complete restriction from teaching is unnecessarily limiting given her recovery status
B) Immediate return without precautions or graduated schedule may be too aggressive
D) Waiting a full year is overly cautious given her current status
E) Permanent restriction from classroom teaching is unwarranted
One-year follow-up:
At one year post-transplant, Sarah remains in molecular remission with normal blood counts. She has returned to teaching full-time. Her bone marrow assessment shows complete morphologic and molecular remission. She has developed mild hyperlipidemia (LDL 145 mg/dL) and her blood pressure is slightly elevated at 142/88 mmHg.
Question 12:
What is the most appropriate approach to managing Sarah's cardiovascular risk factors?
A) No intervention needed; these are expected post-transplant changes
B) Dietary and lifestyle modifications only
C) Resume lisinopril for hypertension; continue lifestyle modifications and monitor lipids
D) Start a high-intensity statin; continue monitoring blood pressure

E) Start lisinopril and a moderate-intensity statin
Correct! Starting lisinopril and a moderate-intensity statin is the most appropriate approach to managing Sarah's cardiovascular risk factors.
Why this is correct:
Sarah has developed multiple cardiovascular risk factors post-transplant:

Hypertension (142/88 mmHg)
Hyperlipidemia (LDL 145 mg/dL)
History of diabetes (though currently controlled)
History of allogeneic HSCT (which itself increases cardiovascular risk)

According to survivorship guidelines for HSCT recipients and cardio-oncology recommendations:

Cardiovascular risk management should be more aggressive in cancer survivors
Blood pressure goal is typically <130/80mmHg for cancer survivors
LDL goals may be lower for patients with multiple risk factors
Since she previously responded well to lisinopril, resuming this medication is appropriate
A moderate-intensity statin is appropriate for her LDL level and risk profile

Why other options are incorrect:

A) These changes require intervention, not observation
B) Lifestyle modifications alone are insufficient given her multiple risk factors
C) This addresses only hypertension but ignores hyperlipidemia
D) This addresses only hyperlipidemia but ignores hypertension
18-month follow-up:
Sarah continues to do well, but routine monitoring reveals a rising LDH (350 U/L) and mild thrombocytopenia (platelets 95 10^9/L). A bone marrow biopsy shows 3% blasts with return of the original leukemic immunophenotype. Molecular testing confirms reappearance of the inv(16) and KIT mutation.
Question 13:
What is the most appropriate next step in managing Sarah's relapsed AML?
A) Observation with closer monitoring
B) Donor lymphocyte infusion (DLI) alone
C) Salvage chemotherapy followed by second allogeneic HSCT
D) Salvage chemotherapy followed by DLI
E) Targeted therapy with a KIT inhibitor
Correct! Salvage chemotherapy followed by donor lymphocyte infusion (DLI) is the most appropriate next step.
Why this is correct:
Sarah has early molecular and morphologic relapse after allogeneic HSCT. According to NCCN and ESMO guidelines for managing post-transplant AML relapse:

The combination of cytoreductive therapy followed by DLI provides better outcomes than DLI alone
A second transplant would carry excessive toxicity given her age (now 68-69)
Chemotherapy alone without cellular therapy is unlikely to provide durable remission
She has several favorable factors for this approach:

Matched sibling donor is available for DLIRelatively late relapse (18 months post-transplant)
No active GVHD or recent immunosuppression
Limited disease burden (only 3% blasts)

Why other options are incorrect:

A) Observation is inappropriate for confirmed AML relapse
B) DLI alone has limited efficacy without prior cytoreduction
C) Second allogeneic HSCT carries excessive mortality risk in this situation
E) While KIT inhibitors show promise in clinical trials, they are not standard therapy for relapsed AML with KIT mutations and would not provide the immunological benefit of DLI
Treatment course:
Sarah receives FLAG chemotherapy (fludarabine, cytarabine, G-CSF) followed by DLI. She achieves a second complete remission. Six months after DLI, she develops mild chronic GVHD of the skin and mouth.
Question 14:
Which of the following is the most appropriate approach to managing Sarah's chronic GVHD?
A) Observation only, as mild cGVHD has a protective effect against relapse
B) Systemic corticosteroids (prednisone 1 mg/kg/day)
C) Topical therapies (corticosteroid creams for skin; dexamethasone oral solution for mouth)
D) Ruxolitinib
E) Extracorporeal photopheresis (ECP)
Correct! Topical therapies (corticosteroid creams for skin; dexamethasone oral solution for mouth) are the most appropriate approach.
Why this is correct:
According to NIH consensus criteria and GVHD management guidelines:

Mild chronic GVHD limited to skin and mouth can often be managed with topical therapies alone
Topical steroids provide targeted treatment while minimizing systemic immunosuppression
This approach preserves the graft-versus-leukemia (GVL) effect, which is important given Sarah's history of relapse
Specific approaches include:

Medium-potency steroid creams for skin manifestations
Dexamethasone oral solution (swish andspit) for oral mucosal involvement

Why other options are incorrect:

A) While mild cGVHD may have some protective effect against relapse, symptoms still warrant treatment for quality of life
B) Systemic corticosteroids at high doses would be excessive for mild cGVHD and could unnecessarily compromise GVL effect
D) Ruxolitinib is approved for steroid-refractory cGVHD, not as first-line therapy for mild disease
E) ECP is typically reserved for more extensive or refractory cGVHD
Two-year follow-up:
Sarah has been in continuous complete remission since her DLI. Her chronic GVHD has resolved with topical therapy. She asks about her long-term prognosis and ongoing monitoring requirements.
Question 15:
Which of the following represents the most appropriate long-term monitoring plan for Sarah?
A) Monthly CBC and chemistry panel; bone marrow evaluation every 3 months; annual PFTs and ECHO
B) CBC and chemistry panel every 3 months; bone marrow evaluation only if abnormal counts; annual endocrine and cardiopulmonary assessment
C) CBC every 6 months; annual bone marrow evaluation for 5 years
D) CBC only if symptomatic; no routine bone marrow evaluations
E) Continued monthly monitoring indefinitelydue to high relapse risk
Correct! CBC and chemistry panel every 3 months; bone marrow evaluation only if abnormal counts; annual endocrine and cardiopulmonary assessment is the most appropriate long-term monitoring plan.
Why this is correct:
According to ASTCT, EBMT, and NCCN long-term follow-up guidelines for transplant survivors:

After 2 years in remission post-relapse and DLI, the monitoring can be less intensive
Periodic blood count monitoring (every 3 months) remains important to detect early signs of relapse or late complications
Bone marrow evaluations are not needed routinely unless triggered by abnormal blood counts
Annual screening for late effects should include:Cardiovascular assessment (blood pressure, lipids, ECHO)
Endocrine function (thyroid, glucose metabolism)

Pulmonary function (especially given history of GVHD)
Bone health assessment

Why other options are incorrect:

A) Monthly labs and quarterly bone marrow evaluations are unnecessarily frequent at this stage
C) Annual bone marrow evaluations are not routinely recommended in the absence of clinical indications
D) This approach provides insufficient monitoring given her history of relapse
E) Indefinite monthly monitoring is excessively intensive and not supported by guidelines

---

Session ID:
Assessment Domain

Session-17:
Rationale

Session ID:
Instructional Design and Pedagogical

Session-17:
The session demonstrates excellent instructional design with expertly scaffolded progression through the complete AML patient journey from initial presentation through long-term survivorship care. The embedded feedback is exceptional, consistently providing detailed, evidence-based rationales that cite current guidelines (ELN 2022, ESMO, NCCN, ASTCT/EBMT) and thoroughly explain both correct and incorrect choices. Instructions are clear and unambiguous throughout all phases. The only minor weakness is the absence of explicitly stated learning objectives at the session's beginning, though the comprehensive educational goals are clearly evident through the structured content progression.

Session ID:
Clinical Authenticity and Content Fidelity

Session-17:
The session demonstrates exceptional clinical authenticity across all evaluated domains. The clinical narrative flows naturally and realistically through all phases of AML management, from initial presentation with pancytopenia and constitutional symptoms through complex post-transplant complications and long-term survivorship care. All therapeutic recommendations are perfectly aligned with current evidence-based guidelines, including appropriate risk stratification using ELN 2022 criteria (inv(16) with KIT mutation correctly classified as intermediate risk), standard 7+3 induction for a fit elderly patient, appropriate supportive care measures, and evidence-based transplant management. Pharmacotherapeutic choices are consistently optimal and well-justified, from empiric antimicrobial selection to conditioning regimen choice to post-transplant prophylaxis strategies. The session maintains strict domain specificity with all treatments remaining appropriate for AML and post-transplant management without any inappropriate cross-domain applications. Inappropriate risk stratification using ELN 2022 criteria (inv(16) with KIT mutation classified as favourable, not intermediate risk). According to the wrong risk stratification, the recommended consolidation was inappropriate.

Session ID:
Modeled Clinical Reasoning

Session-17:
The simulation excellently models expert-level clinical reasoning throughout the complex AML management pathway. Each clinical scenario is expertly framed with appropriate diagnostic data and clear decision points that reflect authentic clinical practice. The multiple-choice options demonstrate excellent breadth with highly relevant distractors that effectively test nuanced clinical understanding, such as distinguishing between conditioning regimens based on patient age and comorbidities, or selecting appropriate salvage strategies for post-transplant relapse. The decision-making consistently demonstrates appropriate risk-adjusted prioritization, including the selection of reduced-intensity conditioning over myeloablative approaches for the elderly patient, and the

choice of salvage chemotherapy followed by DLI rather than a second transplant for post-transplant relapse. Clinical outcomes are realistically modeled with instructionally valuable complications and responses that reinforce key learning points about AML management, transplant medicine, and long-term survivorship care.

Session-18

Session ID:
LLM

Session-18:
Claude 3.7 Sonnet

Session ID:
Session Transcript

Session-18:
Excellent, [REDACTED]]. Let's begin with our case.

PHASE 1: INITIAL PRESENTATION AND DIAGNOSTIC WORKUP

Case Introduction:
Fatima Ahmed is a 67-year-old female who presents to the emergency department with a 3-week history of progressive fatigue, shortness of breath, and intermittent low-grade fevers. She has a medical history significant for hypertension managed with lisinopril, and type 2 diabetes managed with metformin. She immigrated from Yemen 5 years ago and lives with her daughter's family. She has limited English proficiency and her daughter serves as her interpreter.

Initial Laboratory Results:
- WBC: 45.2 10/L (elevated)
- Hemoglobin: 8.1 g/dL (decreased)
- Platelets: 32 10/L (decreased)
- Peripheral blood smear: 35% circulating blasts
- Comprehensive metabolic panel: Normal renal function, mild elevation in LDH and uric acid
- Coagulation studies: PT and PTT within normal limits

Question 1: Based on Fatima's initial presentation, which of the following would be the most appropriate next step in her diagnostic workup?

A) Begin empiric antibiotics for presumed infection and repeat CBC in 3 days
B) Perform a bone marrow aspiration and biopsy with immunophenotyping, cytogenetics, and molecular testing
C) Obtain a CT scan of the chest, abdomen, and pelvis to rule out solid malignancy
D) Start immediate hydroxyurea to reduce WBC count without further diagnostic testing
Correct! Performing a bone marrow aspiration and biopsy with immunophenotyping, cytogenetics, and molecular testing is the most appropriate next step for Fatima.
Question 2: Based on the information available, which molecular mutation would be most important to identify for Fatima's treatment planning at this point?

A) BCR-ABL1 fusion
B) NPM1 mutation
C) PML-RARA fusion
D) FLT3-ITD mutation
Correct! FLT3-ITD mutation is the most important to identify for Fatima's immediate treatment planning.
Question 3: According to the ELN 2022 risk stratification, which risk category does Fatima belong to?

A) Favorable risk
B) Intermediate risk
C) Adverse risk
D) Cannot be determined without additional molecular testing

Correct! According to the ELN 2022 risk stratification, Fatima belongs to the Adverse risk category.
Question 4: Based on Fatima's age, molecular profile, and risk stratification, which of the following would be the most appropriate initial treatment approach?
A) Standard "7+3" induction (cytarabine + daunorubicin) plus midostaurin
B) Venetoclax + azacitidine plus midostaurin
C) CPX-351 (liposomal daunorubicin and cytarabine)
D) Low-dose cytarabine plus venetoclax
Correct! Venetoclax + azacitidine plus midostaurin would be the most appropriate initial treatment approach for Fatima.
Question 5: Which of the following is the most important supportive care measure to implement prior to initiating Fatima's AML therapy?
A) Prophylactic antibiotics (levofloxacin)
B) Tumor lysis syndrome prophylaxis (allopurinol, hydration)
C) Antiemetic therapy (ondansetron)
D) Antifungal prophylaxis (posaconazole)
Correct! Tumor lysis syndrome (TLS) prophylaxis with allopurinol and hydration is the most important supportive care measure to implement prior to initiating Fatima's AML therapy.
PHASE 2: INDUCTION THERAPY AND SUPPORTIVE CARE

Fatima begins treatment with venetoclax + azacitidine plus midostaurin after appropriate TLS prophylaxis. Her treatment is initiated in the hospital with close monitoring.
Question 6: On day 3 of therapy, which of the following parameters is MOST critical to monitor for potential tumor lysis syndrome?

A) Complete blood count with differential
B) Serum potassium, phosphorus, calcium, and uric acid levels
C) Daily body weight and strict input/output measurements
D) Liver function tests
Correct! Serum potassium, phosphorus, calcium, and uric acid levels are the MOST critical parameters to monitor for potential tumor lysis syndrome.
Question 7: What is the most appropriate immediate management for Fatima's fever?
A) Acetaminophen for symptom management and continued monitoring
B) Empiric antibiotics with antipseudomonal coverage after obtaining blood cultures
C) Empiric antifungal therapy with an echinocandin
D) Initiate G-CSF (filgrastim) to accelerate neutrophil recovery
Correct! Empiric antibiotics with antipseudomonal coverage after obtaining blood cultures is the most appropriate immediate management for Fatima's fever.
Question 8: The bone marrow biopsy shows 8% blasts with evidence of count recovery. Which of the following is the most appropriate interpretation of these findings?
A) Complete remission (CR)
B) Complete remission with incomplete hematologic recovery (CRi)
C) Morphologic leukemia-free state (MLFS)
D) Refractory disease
Correct! Morphologic leukemia-free state (MLFS) is the most appropriate interpretation of these findings.
Question 9: Given Fatima's response assessment, which of the following would be the most appropriate next step in management?
A) Proceed to allogeneic stem cell transplantation
B) Continue with the same regimen for another cycle
C) Switch to intensive salvage chemotherapy (FLAG-IDA)
D) Switch to gilteritinib monotherapy
Correct! Continuing with the same regimen for another cycle would be the most appropriate next step in Fatima's management.
Question 10: Which of the following would be the most appropriate consolidated response assessment based on these findings?
A) Complete remission (CR)
B) Complete remission with incomplete hematologic recovery (CRi)
C) Complete remission without minimal residual disease (CR MRD-)
D) Partial remission (PR)

Correct! Complete remission (CR) is the most appropriate consolidated response assessment based on these findings.

PHASE 3: POST-REMISSION THERAPY AND LONG-TERM MANAGEMENT

Question 11: Given Fatima's current status (CR with molecular persistence of FLT3-ITD) and her ELN adverse risk classification, which of the following would be the most appropriate consolidation strategy?

A) Continue venetoclax + azacitidine + midostaurin for 6-12 months total
B) Proceed to allogeneic stem cell transplantation
C) Switch to intensive consolidation with high-dose cytarabine
D) Switch to maintenance therapy with gilteritinib alone

Correct! Proceeding to allogeneic stem cell transplantation would be the most appropriate consolidation strategy for Fatima.

Question 12: Prior to proceeding with transplant evaluation, which of the following would be the most important assessment to perform for Fatima?

A) Comprehensive geriatric assessment and frailty evaluation
B) Repeat bone marrow biopsy to confirm remission status
C) HLA typing of Fatima and potential family donors
D) Advanced cardiac testing (echocardiogram, stress test)

Correct! HLA typing of Fatima and potential family donors would be the most important assessment to perform at this stage.

Question 13: Which of the following FLT inhibitor strategies would provide the best post-transplant outcomes for Fatima?

A) No FLT3 inhibitor needed after transplant
B) Sorafenib maintenance for 12 months post-transplant
C) Midostaurin maintenance for 12 months post-transplant
D) Gilteritinib maintenance for 24 months post-transplant

Correct! Sorafenib maintenance for 12 months post-transplant would provide the best post-transplant outcomes for Fatima.

Question 14: Three months after transplant, Fatima presents with new-onset skin rash, elevated liver enzymes, and diarrhea. Which of the following is the most likely diagnosis?

A) Veno-occlusive disease (VOD/SOS)
B) Acute graft-versus-host disease (GVHD)
C) Drug reaction to sorafenib
D) Viral reactivation (CMV enteritis)

Correct! Acute graft-versus-host disease (GVHD) is the most likely diagnosis.

Question 15: Which of the following is the first-line pharmacotherapy for Fatima's current condition?

A) Defibrotide
B) Systemic corticosteroids
C) Etanercept
D) Ruxolitinib

Correct! Systemic corticosteroids are the first-line pharmacotherapy for acute graft-versus-host disease (GVHD).

Session ID:
Assessment Domain

Session-18:
Rationale

Session ID:
Instructional Design and Pedagogical

Session-18:
The session demonstrates excellent scaffolding, progressing logically from initial presentation through diagnosis, induction therapy, response assessment, transplant planning, and post-transplant complications. The clinical scenarios are clearly framed and easy to follow. However, there are no explicitly stated learning objectives at the outset, and the feedback is minimalonly showing "Correct!" without the detailed rationales, clinical pearls, or guideline citations that would make this educationally robust.

Session ID:

Clinical Authenticity and Content Fidelity

Session-18:

The clinical narrative is highly authentic and plausible, presenting a realistic elderly AML patient with appropriate demographics, laboratory values, and disease progression. The therapeutic choices are generally well-aligned with current guidelinesvenetoclax + azacitidine + midostaurin for an elderly, adverse-risk AML patient is appropriate, as is the progression to allogeneic transplant. The post-transplant sorafenib maintenance is also guideline-consistent for FLT3-mutated AML. All recommendations remain strictly within the AML domain without inappropriate cross-contamination from other hematologic malignancies. Minor points were deducted for some treatment timing and sequencing decisions that, while not incorrect, may not represent the most optimal contemporary approach.

Session ID:

Modeled Clinical Reasoning

Session-18:

The simulation excellently models expert clinical reasoning by presenting well-defined problems at each stage with appropriate clinical complexity. The diagnostic workup follows logical sequence (molecular testing before treatment, risk stratification guiding therapy selection). The multiple-choice options generally provide good breadth of plausible alternatives, though some questions could benefit from more sophisticated distractors. Prioritization is generally appropriate, though the rapid progression through some treatment phases could better emphasize the decision-making nuances. Clinical outcomes are realistic but could be more detailed to enhance learning valuefor example, explaining why MLFS might occur with this regimen or detailing the specific manifestations of GVHD.

Session-19

Session ID:
LLM

Session-19:
Claude 3.7 Sonnet

Session ID:
Session Transcript

Session-19:
Patient Introduction:
Sarah Thompson is a 68-year-old woman who presents to her primary care physician with complaints of persistent fatigue, shortness of breath with minimal exertion, and recurrent low-grade fevers for the past 3 weeks. She also reports unintentional weight loss of approximately 5 kg over the past 2 months and occasional night sweats.
Past Medical History:
Hypertension (diagnosed 12 years ago)
Type 2 diabetes mellitus (diagnosed 8 years ago)
Hyperlipidemia
Osteoarthritis of the knees
Current Medications:
Lisinopril 20 mg daily
Metformin 1000 mg twice daily
Atorvastatin 40 mg daily
Acetaminophen 500 mg as needed for pain
Social History:
Former smoker (quit 15 years ago, 20 pack-year history)
Occasional alcohol use (1-2 glasses of wine per week)
Retired elementary school teacher
Lives alone, daughter lives nearby and provides support
Physical Examination:
Vital signs: Temperature 38.1C, HR 98 bpm, BP 132/84 mmHg, RR 20/min, O2 sat 94% on room air
General: Appears fatigued, pale
HEENT: Mild pallor of conjunctiva, no scleral icterus
Cardiovascular: Regular rate and rhythm, no murmurs
Respiratory: Clear to auscultation bilaterally
Abdomen: Soft, non-tender, no hepatosplenomegaly
Extremities: No edema, few scattered ecchymoses on the lower extremities
Skin: No rashes or lesions
Initial Laboratory Results:
WBC: 28.5 10^9/L (normal: 4.5-11.0 10^9/L)
Hemoglobin: 8.2 g/dL (normal: 12.0-15.5 g/dL)
Platelets: 45 10^9/L (normal: 150-450 10^9/L)
Peripheral blood smear: 35% blast cells
Comprehensive metabolic panel: Within normal limits except for mildly elevated LDH at 350 U/L (normal: 140-280 U/L)
Question 1:
Based on the initial presentation and laboratory findings, what is the most appropriate next diagnostic step?
A) Obtain bone marrow aspirate and biopsy with cytogenetic and molecular analysis
B) Start broad-spectrum antibiotics for presumed infection
C) Order a CT scan of the chest, abdomen, and pelvis to evaluate for malignancy
D) Initiate corticosteroid therapy for suspected autoimmune process
E) Refer for lymph node biopsy

You selected option A) Obtain bone marrow aspirate and biopsy with cytogenetic and molecular analysis.
This is correct!

Question 2:
Based on the bone marrow findings, what is the correct WHO 2016/2022 classification and ELN 2022 risk stratification for this patient?
A) AML with myelodysplasia-related changes, ELN 2022 favorable risk
B) AML with myelodysplasia-related changes, ELN 2022 intermediate risk
C) AML with myelodysplasia-related changes, ELN 2022 adverse risk
D) AML with mutated NPM1, ELN 2022 favorable risk
E) Therapy-related AML, ELN 2022 adverse risk
You selected option C) AML with myelodysplasia-related changes, ELN 2022 adverse risk.
This is correct!

Question 3:
What is the most appropriate induction therapy for this 68-year-old patient with AML with myelodysplasia-related changes and adverse risk features?
A) Standard 7+3 regimen (cytarabine + daunorubicin)
B) CPX-351 (liposomal daunorubicin and cytarabine)
C) Venetoclax + azacitidine
D) Venetoclax + low-dose cytarabine
E) Best supportive care only
You selected option B) CPX-351 (liposomal daunorubicin and cytarabine).
This is correct!

Question 4:
Which of the following findings would meet the criteria for complete remission (CR) according to ELN criteria?
A) <5% bone marrow blasts, neutrophils >1.0 10^9/L, platelets >100 10^9/L, no extramedullary disease
B) <5% bone marrow blasts, neutrophils >0.5 10^9/L, platelets >50 10^9/L, no extramedullary disease
C) <10% bone marrow blasts, neutrophils >1.0 10^9/L, platelets >100 10^9/L, no extramedullary disease
D) <5% bone marrow blasts with persistent cytopenias (neutrophils <1.0 10^9/L and/or platelets <100 10^9/L)
E) <20% bone marrow blasts with recovery of peripheral blood counts
You selected option A) <5% bone marrow blasts, neutrophils >1.0 10^9/L, platelets >100 10^9/L, no extramedullary disease.
This is correct!

Question 5:
Based on the day 35 bone marrow assessment, what is Sarahs current status, and what is the most appropriate next step in management?
A) Complete remission; proceed directly to allogeneic stem cell transplantation
B) Complete remission with MRD positivity; recommend consolidation with CPX-351 followed by allogeneic stem cell transplantation
C) Complete remission with MRD positivity; add enasidenib (IDH2 inhibitor) to the treatment plan before transplant consideration
D) Resistant disease; switch to venetoclax plus azacitidine
E) Complete remission with MRD positivity; observation only with close monitoring
You selected option B) Complete remission with MRD positivity; recommend consolidation with CPX-351 followed by allogeneic stem cell transplantation.
This is correct!
PHASE 2: Induction Therapy and Supportive Care
Sarah receives her first consolidation cycle with CPX-351 at the reduced consolidation dose (65 units/m on days 1 and 3). Her treatment plan includes evaluation for allogeneic stem cell transplantation.
Pre-Consolidation Laboratory Values:
WBC: 3.2 10^9/L
ANC: 1.8 10^9/L
Hemoglobin: 10.2 g/dL

Platelets: 125 10^9/L
Creatinine: 0.9 mg/dL
Total bilirubin: 0.8 mg/dL
ALT: 25 U/L, AST: 28 U/L
Potassium: 3.8 mEq/L
Phosphorus: 3.2 mg/dL
Uric acid: 5.8 mg/dL
Calcium: 9.1 mg/dL

Question 6:

Which of the following supportive care measures should be included in Sarahs management during consolidation therapy?

A) Allopurinol, acyclovir, fluconazole, and fluoroquinolone prophylaxis
B) Allopurinol, acyclovir, posaconazole, and fluoroquinolone prophylaxis
C) Rasburicase, acyclovir, posaconazole, and fluoroquinolone prophylaxis
D) Rasburicase, acyclovir, posaconazole, and G-CSF from day 1
E) Allopurinol, acyclovir, fluconazole, and G-CSF from day 1

You selected option B) Allopurinol, acyclovir, posaconazole, and fluoroquinolone prophylaxis.

This is correct!

Treatment Course - Day 10 of Consolidation:

Sarah develops a fever of 38.8C and rigors. She appears fatigued but alert.

Vital signs:
Temperature: 38.8C
HR: 118 bpm
BP: 95/60 mmHg
RR: 22/min
O2 sat: 95% on room air

Laboratory Results:
WBC: 0.3 10^9/L
ANC: 0.1 10^9/L
Hemoglobin: 8.5 g/dL
Platelets: 22 10^9/L
Creatinine: 1.1 mg/dL
BUN: 25 mg/dL
Lactic acid: 2.2 mmol/L

Physical Examination:
Lungs: Clear to auscultation
Cardiac: Tachycardic but regular rhythm
Abdomen: Soft, non-tender
Catheter site: No erythema or drainage
Oral cavity: Mild mucositis

Question 7:

What is the most appropriate initial management for Sarahs current condition?

A) Start vancomycin, obtain blood cultures, and continue current fluoroquinolone prophylaxis
B) Start vancomycin plus cefepime, obtain blood cultures, chest X-ray, and urinalysis with culture
C) Start vancomycin plus meropenem plus amikacin, obtain blood cultures, chest X-ray, and CT of sinuses
D) Start vancomycin plus piperacillin-tazobactam, obtain blood cultures, and add G-CSF
E) Start vancomycin plus cefepime plus metronidazole, obtain blood cultures, and discontinue posaconazole

You selected option B) Start vancomycin plus cefepime, obtain blood cultures, chest X-ray, and urinalysis with culture.

This is correct!

Treatment Course Update:

Sarah receives appropriate management for febrile neutropenia. Blood cultures return positive for Escherichia coli (sensitive to cefepime). She completes a 10-day course of antibiotics with resolution of fever. Her counts recover by day 28 post-consolidation.

A repeat bone marrow biopsy shows continued morphologic remission with 3% blasts. MRD testing shows 0.05% by flow cytometry (reduced from 0.1%). Molecular testing continues to show the IDH2 mutation but at a lower variant allele frequency (12% compared to

previous 18%).
HLA Typing and Donor Search:
Sarahs HLA type has been sent, and a search identifies her sister as a 10/10 HLA match. The transplant team evaluates Sarah and determines she is a candidate for reduced-intensity conditioning (RIC) allogeneic stem cell transplantation.
Question 8:
What factors should be considered when selecting the conditioning regimen intensity for Sarah?
A) Age alone should determine conditioning intensity; all patients >65 should receive RIC
B) Performance status and comorbidities; fludarabine/busulfan is preferred over fludarabine/melphalan for all AML patients
C) Risk of disease relapse balanced against transplant-related mortality; patients with adverse-risk AML may benefit from more intensive conditioning if tolerable
D) MRD status alone; MRD-positive patients should always receive myeloablative conditioning regardless of age
E) Donor type is the primary determinant; matched related donors allow for reduced-intensity conditioning in all cases
You selected option C) Risk of disease relapse balanced against transplant-related mortality; patients with adverse-risk AML may benefit from more intensive conditioning if tolerable.
This is correct!
Treatment Decision:
After multidisciplinary discussion, Sarah is scheduled for a reduced-intensity conditioning regimen with fludarabine/busulfan for her allogeneic stem cell transplantation from her matched sibling donor.
Before transplant, the team discusses the role of maintenance therapy post-transplant given her adverse risk features and MRD positivity pre-transplant.
Question 9:
Which of the following factors would be MOST important in determining whether Sarah should receive maintenance therapy post-transplant?
A) Pre-transplant MRD status only
B) Post-transplant MRD status at day +30
C) Development of acute GVHD requiring systemic corticosteroids
D) Donor chimerism status at day +100
E) Presence of the IDH2 mutation at diagnosis
You selected option B) Post-transplant MRD status at day +30.
This is correct!

Question 10:
Which of the following would be the most appropriate GVHD prophylaxis regimen for Sarah?
A) Cyclosporine + methotrexate
B) Tacrolimus + methotrexate
C) Tacrolimus + mycophenolate mofetil
D) Post-transplant cyclophosphamide alone
E) Tacrolimus + sirolimus
You selected option C) Tacrolimus + mycophenolate mofetil.
This is correct!
PHASE 3: Post-Remission Therapy and Long-Term Management
Post-Transplant Day +100 Assessment:
Sarah has recovered well from transplantation. She has mild chronic GVHD of the skin managed with topical steroids. Her performance status has returned to ECOG 1. Laboratory values show:
WBC: 4.2 10^9/L
Hemoglobin: 11.5 g/dL
Platelets: 145 10^9/L
Comprehensive metabolic panel: Within normal limits
Her day +100 bone marrow biopsy shows:
3% myeloid precursors, no evidence of leukemia
100% donor chimerism
MRD negative by flow cytometry
Molecular testing reveals persistent IDH2 R140Q mutation at low level (VAF 3%)
Question 11:

Based on Sarahs day +100 assessment, what is the most appropriate next step in management?
A) Start enasidenib maintenance therapy immediately due to molecular MRD
B) Continue current management with close MRD monitoring every 1-2 months
C) Start azacitidine maintenance therapy
D) Administer donor lymphocyte infusion (DLI)
E) Taper immunosuppression rapidly to enhance graft-versus-leukemia effect
You selected option B) Continue current management with close MRD monitoring every 1-2 months.
This is correct!

Question 12:
What is the recommended vaccination schedule for Sarah?
A) Begin all vaccinations at 3 months post-transplant
B) Begin inactivated vaccines at 6 months and live vaccines at 12 months post-transplant if off immunosuppression
C) Begin inactivated vaccines at 6 months regardless of immunosuppression status, no live vaccines until 24 months post-transplant
D) Vaccinations are contraindicated in the setting of chronic GVHD
E) Begin all vaccinations, including live vaccines, at 6 months post-transplant
You selected option C) Begin inactivated vaccines at 6 months regardless of immunosuppression status, no live vaccines until 24 months post-transplant.
This is not correct.

One-Year Post-Transplant:
Sarah returns for her one-year transplant evaluation. She has been doing well with no significant complications. Her chronic GVHD resolved after a slow steroid taper, and she is now off all immunosuppression. Her performance status is ECOG 0.
One-year bone marrow evaluation shows:
 Normocellular marrow with trilineage hematopoiesis
 No morphologic evidence of leukemia
 100% donor chimerism
 MRD negative by flow cytometry
 IDH2 mutation undetectable
Question 13:
Which of the following late complications should be specifically monitored in Sarahs long-term follow-up?
A) Cataracts, thyroid dysfunction, bone density, secondary malignancies
B) Pulmonary fibrosis, cardiac dysfunction, iron overload, bone density
C) Iron overload, adrenal insufficiency, renal dysfunction, hepatic fibrosis
D) Cardiac dysfunction, cataracts, peripheral neuropathy, renal dysfunction
E) Secondary malignancies, adrenal insufficiency, diabetes, pulmonary fibrosis
The correct answer is A) Cataracts, thyroid dysfunction, bone density, secondary malignancies.
This is the most appropriate set of long-term complications to monitor because:

..
A
..
B

Session ID:
Assessment Domain

Session-19:
Rationale

Session ID:
Instructional Design and Pedagogical

Session-19:

The session's instructional design has significant strengths but is marred by a critical flaw. The case is expertly scaffolded, guiding the learner through a logical and chronologically authentic clinical journey from initial diagnosis to long-term post-transplant follow-up. However, the pedagogical value is severely undermined by the quality of embedded feedback. For most questions, the feedback consists only of "This is correct!", offering no rationale, evidence, or clinical pearls to reinforce the learning objective. In one instance (Q12), an answer is marked incorrect without providing the correct answer or an explanation. While the overall structure is clear, this lack of explanatory feedback prevents the session from being a robust educational tool and earns a "Fair" rating for that criterion.

Session ID:

Clinical Authenticity and Content Fidelity

Session-19:

The clinical narrative is highly authentic, presenting a realistic 68-year-old woman with AML with myelodysplasia-related changes. The case progression through diagnostic workup, risk stratification, treatment selection, and post-transplant management aligns well with current AML guidelines. The therapeutic choices are generally appropriate: CPX-351 for adverse-risk AML in an older patient, appropriate supportive care measures, and standard post-transplant management. The case remains strictly within the AML domain without inappropriate application of therapies from other malignancies. Minor points are deducted for the vaccination question (Question 12) where the "incorrect" answer provided may actually align with some institutional protocols, suggesting potential guideline interpretation variability.

Session ID:

Modeled Clinical Reasoning

Session-19:

The session excellently models expert clinical reasoning with well-framed problems at each decision point. The diagnostic and therapeutic options provide good breadth, including relevant distractors that test nuanced understanding of AML management. Clinical prioritization is generally appropriate, emphasizing the importance of molecular diagnostics, risk stratification, and transplant evaluation. However, the modeling of clinical outcomes is somewhat limited - while the case shows disease response and recovery, it lacks detailed discussion of potential complications or alternative scenarios that could enhance learning about clinical decision-making under uncertainty

---

Session-20

Session ID:
LLM

Session-20:
Claude 3.7 Sonnet

Session ID:
Session Transcript

Session-20:
Case Presentation: Phase 1 - Initial Presentation and Diagnostic Workup
Patient Information:

Name: Sarah Johnson
Age: 68 years old
Sex: Female
Weight: 72 kg
Height: 165 cm
Medical History: Hypertension (controlled on lisinopril 10 mg daily), type 2 diabetes mellitus (metformin 1000 mg twice daily), and osteoarthritisSocial History: Retired school teacher, lives with husband, has good family support, middle-class socioeconomic statusAllergies: Sulfonamides (rash)
Chief Complaint:Sarah presents to her primary care physician with complaints of persistent fatigue, recurrent low-grade fevers for the past 3 weeks, and increased bruising. She also reports unintentional weight loss of approximately 4 kg over the past 2 monthsPhysical Examination:Vital signs: Temperature 38.1C, BP 138/82 mmHg, HR 92 bpm, RR 18/min
General: Appears fatigued but alert and orientedSkin: Multiple ecchymoses on upper and lower extremities
Cardiovascular: Regular rate and rhythm, no murmursRespiratory: Clear to auscultation bilaterallyAbdominal: Mild splenomegaly (2 cm below costal margin)
Lymph nodes: No palpablelymphadenopathy Initial Laboratory ResultsCBC:WBC: 28.5 10/L (elevated)
Hemoglobin: 8.7 g/dL (decreased)
Platelets: 42 10/L (decreased)
Differential: 45% blasts in peripheral blood
Chemistry:Creatinine: 1.1 mg/dLBUN: 22 mg/dLLDH: 780 U/L (elevated)Uric acid: 6.8 mg/dL (slightly elevated)
AST/ALT: Within normal limits
Total bilirubin: 1.2 mg/dLQuestion 1: Based on Sarah's presentation and initial laboratory results, what is the most appropriate next step in her diagnostic workup?A) Order flow cytometry on peripheral bloodB) Refer for bone marrow aspiration and biopsyC) Begin empiric antibiotics for suspected infectionD) Order CT scan of chest, abdomen, and pelvisE) Start erythropoietin therapy for anemia
Correct! Referring Sarah for a bone marrow aspiration and biopsy is the most appropriate next step.
Explanation:
The presence of 45% blasts in the peripheral blood, along with pancytopenia, splenomegaly, and constitutional symptoms strongly suggests acute leukemia. While peripheral blood flow cytometry can provide preliminary information, a bone marrow aspiration and biopsy is essential for:
Definitive diagnosis of AML (requires 20% myeloid blasts in bone marrow according to WHO criteria)
Complete cytogenetic and molecular testing required for risk stratification
Assessment of cellularity and extent of bone marrow involvement
Collection of adequate sample for further diagnostic studies (immunophenotyping, cytogenetics, FISH, and molecular studies)
Why other options are incorrect:
A) Flow cytometry on peripheral blood is helpful but insufficient for complete diagnosis and risk stratification
C) While the patient has fever, there is no clear evidence of infection, and diagnostic workup takes priority
D) CT scan may be useful later but would not provide the definitive diagnosis
E) Erythropoietin therapy is inappropriate before establishing a diagnosis, especially with blasts present
Additional information:

Sarah undergoes bone marrow aspiration and biopsy. The results show:
Hypercellular marrow (90% cellularity)
65% myeloid blastsFlow cytometry: Blasts positive for CD34, CD117, CD13, CD33, and MPO
Cytogenetics: Normal karyotype (46,XX)
Molecular studies:NPM1 mutation: Positive
FLT3-ITD mutation: Positive (allelic ratio 0.7)DNMT3A mutation: Positive
IDH1/2 mutations: Negative
TP53 mutation: Negative
Question 2: Based on the 2022 ELN risk stratification criteria, what risk category would Sarah's AML be classified as?
A) Favorable riskB) Intermediate riskC) Adverse riskD) Unclassifiable
I'm sorry, but that's not correct.
Explanation:According to the 2022 European LeukemiaNet (ELN) risk stratification criteria, Sarah's AML would be classified as Adverse risk.The key finding is the combination of NPM1 mutation with FLT3-ITD with a high allelic ratio (0.7). The ELN 2022 guidelines define:NPM1 mutation with FLT3-ITD mutation with high allelic ratio (0.5) is considered Adverse risk
This represents an update from previous guidelines where this combination might have been classified differently
The presence of the DNMT3A mutation also typically indicates a poorer prognosis, though it's not a primary determinant in the ELN classification.
Why other options are incorrect:A) Favorable risk would include NPM1 mutation without FLT3-ITD or with low-ratio FLT3-ITDB) Intermediate risk would include normal karyotype with various other molecular findings, but not this specific combinationD) This case is clearly classifiable based on the provided molecular and cytogenetic information
Question 3: Based on Sarah's risk classification and clinical presentation, which of the following induction regimens would be most appropriate?A) Standard 7+3 regimen (cytarabine + daunorubicin)B) Venetoclax + azacitidine
C) CPX-351 (liposomal daunorubicin and cytarabine)D) FLAG-IDA (fludarabine, cytarabine, G-CSF, idarubicin)E) Midostaurin + standard 7+3 regimen
Correct! Midostaurin + standard 7+3 regimen is the most appropriate induction therapy for Sarah.
Explanation:Given Sarah's specific characteristics:
68 years old but appears fit with manageable comorbidities
FLT3-ITD mutation positive with high allelic ratioAdverse risk by ELN 2022 criteriaThe addition of midostaurin (a FLT3 inhibitor) to standard 7+3 induction therapy is supported by:The RATIFY trial demonstrated improved overall survival when midostaurin was added to standard chemotherapy in FLT3-mutated AMLESMO and NCCN guidelines recommend FLT3 inhibitors in combination with intensive chemotherapy for FLT3-mutated AMLDespite her adverse risk profile, Sarah appears fit enough to tolerate intensive chemotherapyWhy other options are incorrect:A) Standard 7+3 alone would be suboptimal given her FLT3-ITD mutationB) Venetoclax + azacitidine is typically reserved for unfit older patients or those who cannot tolerate intensive chemotherapyC) CPX-351 is primarily indicated for therapy-related AML or AML with myelodysplasia-related changesD) FLAG-IDA is an intensive regimen sometimes used for refractory/relapsed disease or high-risk cases, but lacks the specific FLT3 inhibition needed here
Question 4: Prior to initiating induction therapy for Sarah, which of the following supportive care measures should be implemented?
A) Allopurinol 300 mg daily for tumor lysis syndrome prophylaxis
B) Levofloxacin prophylaxis alone
C) G-CSF administration during induction
D) Rasburicase plus aggressive hydration for tumor lysis syndrome prophylaxis
E) Posaconazole prophylaxis alone
Correct!
Explanation:
Sarah is at high risk for tumor lysis syndrome (TLS) based on several factors:
High white blood cell count (28.5 10/L)
High blast percentage (45% in peripheral blood, 65% in bone marrow)
outputFrequent laboratory monitoringAvoidance of exogenous potassium and phosphateWhy other options are incorrect:A) Allopurinol alone would be insufficient given her high risk for TLS; it prevents new uric acid formation but doesn't reduce existing levelsB) Levofloxacin prophylaxis is important but doesn't address the immediate TLS riskC) G-CSF is not recommended during induction as it may stimulate leukemic cell growthE) Posaconazole prophylaxis is important for fungal prophylaxis during neutropenia but doesn't address TLS risk
Question 5: After Sarah receives induction therapy with 7+3 plus midostaurin, she develops neutropenic fever (temperature 39.2C) on day 7 post-induction. Her absolute neutrophil count is<100cells/mm. Whatisthemostappropriateinitial management?A) Start oral ciprofloxacin and monitorB) Start IV vancomycin and piperacillin-tazobactamC) Start IV cefepimeD) Start IV meropenemE) Obtain

cultures and wait for results before starting antibiotics
Correct!
Explanation:
Sarah has high-risk febrile neutropenia based on:Profound neutropenia (ANC <100 cells/mm)Undergoing intensive induction chemotherapy for AML
Fever >38.3C (39.2C)as empiric monotherapy
Cefepime is an appropriate first-line choice for empiric coverage in the absence of specific risk factors for resistant organisms
Therapy should be initiated promptly (within 1 hour of presentation) after obtaining appropriate cultures
Why other options are incorrect:A) Oral antibiotics are inappropriate for high-risk neutropenic feverB) Vancomycin should be added only if there are specific indications (suspected catheter-related infection, skin/soft tissue infection, pneumonia, hemodynamic instability, or known colonization with MRSA)D) Meropenem is typically reserved for patients with risk factors for resistant organisms or clinical instabilityE) Delaying antibiotics while waiting for culture results is dangerous and could lead to sepsis progression
Case Presentation: Phase 2 - Induction Therapy and Supportive Care
Clinical Update:Sarah was started on IV cefepime for neutropenic fever. Blood cultures were drawn prior to antibiotic initiation and remain negative at 72 hours. Her fever resolved after 36 hours of antibiotics. She completed her induction therapy with 7+3 (cytarabine 100 mg/m continuous infusion for 7 days and daunorubicin 60 mg/m on days 1-3) plus midostaurin 50 mg twice daily on days 8-21.
She experienced expected toxicitiesincluding:
Grade 3 mucositis requiring temporary parenteral nutrition
Grade 4 neutropenia (ANC remained <100 cells/mm for 14 days)Grade 3 thrombocytopenia requiring multiple platelet transfusions
Grade 2 nausea controlled with ondansetron and dexamethasone
A day 14 bone marrow biopsy showed hypoplastic marrow with 5% blasts. A day 28 bone marrow assessment is now being performed to evaluate response to induction therapy.Question 6: Which of the following findings on the day 28 bone marrow assessment would be consistent with a complete remission (CR) according to ELN criteria?A) <5% blasts, but persistent neutropenia (ANC <1,000/mm)B) <5% blasts, ANC >1,000/mm, platelets >100,000/mm, no detectable leukemia-associated phenotype by flow cytometryC) <5% blasts, normal blood counts, but measurable residual disease (MRD) positivity by flow cytometry (0.1%)D) <5% blasts, normal blood counts, no detectable MRD, but persistent FLT3-ITD detected by PCRE) <10% blasts with recovery of blood counts
Correct! <5% blasts, ANC >1,000/mm, platelets >100,000/mm, and no detectable leukemia-associated phenotype by flow cytometry is consistent with a complete remission (CR) according to ELN criteria.Explanation:The ELN defines complete remission (CR) as:Bone marrow blasts <5%Absence of circulating blasts and blasts with Auer rods
Absence of extramedullary disease
ANC >1,000/mm
Platelet count >100,000/mm
Independence from red cell transfusions
The absence of a detectable leukemia-associated phenotype by flow cytometry suggests no measurable residual disease (MRD), making this a high-quality remission.
Why other options are incorrect:
A) This represents incomplete blood count recovery and would be classified as CRi (CR with incomplete hematologic recovery)C) This would be classified as CR with MRD (CR MRD+)D) Persistent FLT3-ITD by PCR could indicate molecular persistence, which might classify as CR MRD+E) <10% blasts does not meet the <5% blast threshold required for CR
Day 28 Bone Marrow Results:
Question 7: Based on these results and Sarah's initial risk classification, what is the most appropriate consolidation strategy?A) Standard high-dose cytarabine (HiDAC) for 3-4 cyclesB) Allogeneic hematopoietic stem cell transplantation (HSCT)C) Maintenance therapy with oral azacitidineD) Continuation of midostaurin maintenance onlyE) Low-dose cytarabine consolidation
Correct!
Explanation:
Several factors support recommending allogeneic HSCT for Sarah:
Adverse risk classification by ELN 2022 criteria (NPM1 mutation with high-ratio FLT3-ITD)Although she achieved morphological CR, she has molecular persistence of FLT3-ITD mutation
Her age (68) is no longer a strict contraindication for transplant; fitness and comorbidity assessment are more importantESMO and NCCN guidelines recommend allogeneic HSCT in first CR for patients with adverse-risk AML when feasibleStudies have shown improved relapse-free survival with allogeneic HSCT in patients with FLT3-ITD mutations
Why other options are incorrect:

A) HiDAC is insufficient for adverse-risk disease, particularly with molecular persistenceC) Oral azacitidine maintenance is typically considered post-transplant or for patients who cannot undergo transplantD) Midostaurin maintenance alone would be insufficient for her high-risk diseaseE) Low-dose cytarabine would be significantly suboptima

Question 8: Before proceeding with transplant evaluation, Sarah asks about her prognosis. Which of the following factors in her case is associated with the most favorable impact on prognosis?A) NPM1 mutationB) Normal karyotypeC) Achievement of MRD-negative status by flow cytometryD) Molecular persistence of FLT3-ITD at low allelic ratioE) DNMT3A mutation

Correct!

Explanation:

Achieving MRD-negative status is a powerful prognostic indicator that can override the negative impact of adverse genetic features. Multiple studies have demonstrated:MRD negativity is strongly associated with improved relapse-free survival and overall survivalMRD status is a stronger predictor of outcome than pre-treatment genetic risk in many casesEven in adverse-risk patients, achieving MRD negativity significantly improves prognosisThe prognostic value of MRD assessment is recognized in the ELN recommendations as crucial for risk assessment and treatment decisions

Why other options are incorrect:A) NPM1 mutation is generally favorable, but its positive impact is negated by high-ratio FLT3-ITDB) Normal karyotype alone is not strongly prognostic when molecular markers indicate adverse riskD) Persistence of FLT3-ITD, even at low levels, is associated with increased relapse riskE) DNMT3A mutation is actually associated with adverse outcomes and treatment resistance

Question 9: Sarafollowingconditioningregimens would be most appropriate?A) Myeloablative conditioning with busulfan/cyclophosphamideB) Myeloablative conditioning with total body irradiation/cyclophosphamideC) Reduced-intensity conditioning with fludarabine/busulfanD) Non-myeloablative conditioning with fludarabine/total body irradiationE) Reduced-intensity conditioning with fludarabine/melphalan

Correct!(RIC)withfludarabine/busulfan the optimal choice:Sarah's age (68 years) increases her risk of transplant-related mortality with fully myeloablative regimensHer reduced renal function (CrCl 48 mL/min) is a significant concern for toxicityFludarabine/busulfan RIC:Provides sufficient disease control while reducing organ toxicityHas decreased renal toxicity compared to other regimensShows good outcomes in older patients with AML in CR1Allows for reduced-intensity conditioning while maintaining sufficient anti-leukemic activityWhy other options are incorrect:A) & B) Full myeloablative conditioning would pose excessive toxicity risks given her age and renal functionD) Non-myeloablative conditioning might be insufficient for disease control in adverse-risk AME) Melphalan can have significant renal toxicity and would be less optimal with her reduced renal function

Question 10: While awaiting transplant, Sarah develops fevers, right upper quadrant pain, and shortness of breath. Laboratory studies show AST 150 U/L, ALT 180 U/L, and total bilirubin 2.8 mg/dL. Achest CT shows multiple bilateral nodularinfiltrates. Which antifungal agent wouldbe most appropriate to initiate empiricallyA) FluconazoleB) VoriconazoleC) PosaconazoleD) CaspofunginE) Amphotericin B liposomal

I'm sorry, that's not the correct answer.

Explanation:

For Sarah's presentation of fevers, right upper quadrant pain, shortness of breath with liver function abnormalities and nodular pulmonary infiltrates on CT, the most appropriate empiric antifungal agent would be Voriconazole.This clinical picture strongly suggests invasive aspergillosis, a common fungal infection in AML patients. According to IDSA and ECIL guidelines:

Voriconazole is the first-line therapy for suspected or proven invasive aspergillosis

The bilateral nodular infiltrates on CT are classic for pulmonary aspergillosis

Voriconazole provides excellent coverage against Aspergillus species and achieves good penetration in pulmonary tissue

It also has adequate CNS penetration if there is concern for CNS involvement

Why other options are incorrect:A) Fluconazole lacks activity against Aspergillus speciesC) Posaconazole is excellent for prophylaxis but voriconazole has more robust evidence as first-line therapy for established infectionD) Caspofungin (an echinocandin) is considered second-line therapy for aspergillosis and is typically used when azoles cannot be used due to intolerance or drug interactionsE) Liposomal amphotericin B is also effective but has greater toxicity concerns, particularly with her reduced renal function

Question 11: After appropriate treatment for her fungal infection, Sarah proceeds to allogeneic HSCT with her HLA-identical sister as the donor. Which agent is most appropriate for post-transplant maintenance to reduce the risk of AML relapse given her molecular profile?A) SorafenibB) MidostaurinC) GilteritinibD) Oral azacitidine (CC-486)E) Venetoclax

I'm sorry, that's not the correct answer.

Explanation:Given Sarah's FLT3-ITD mutated AML and her post-transplant status, the most appropriate maintenance therapy would be Gilteritinib.The evidence supporting this choice includes:The BMT CTN 1506 trial (MORPHO study) demonstrated improved relapse-free survival with gilteritinib maintenance after allogeneic HSCT in FLT3-mutated AML

Gilteritinib is a second-generation, highly selective FLT3 inhibitor with superior potency compared to earlier agents

It has an established safety profile in the post-transplant settingIt's FDA-approved specifically for FLT3-mutated AMLWhy other

options are incorrect:A) Sorafenib has shown benefit in some studies but is a multi-kinase inhibitor with less selectivity for FLT3 and more off-target effects compared to gilteritinibB) Midostaurin has not demonstrated the same level of evidence for post-transplant maintenance as gilteritinibD) Oral azacitidine (CC-486) is primarily beneficial in AML patients who cannot proceed to transplantE) Venetoclax has no specific role in FLT3-mutated AML maintenance therapy

Case Presentation: Phase 3 - Post-Remission Therapy and Long-Term ManagementClinical Update:

Sarah successfully underwent allogeneic HSCT with reduced-intensity conditioning. Her post-transplant course was complicated by grade II acute graft-versus-host disease (GVHD) involving the skin and GI tract, which resolved with corticosteroids. She is now day +100 post-transplant and starts maintenance therapy with gilteritinibAt 6 months post-transplant, Sarah is doing well with no evidence of GVHD or relapse. Her bone marrow shows 100% donor chimerism and no evidence of MRD by flow cytometry. Molecular testing shows no detectable FLT3-ITD or NPM1 mutations.Question 12: Which of the following would be the most appropriate monitoring strategy for Sarah over the next year?A) Bone marrow biopsy with MRD assessment monthlyB) Bone marrow biopsy with MRD assessment every 3 monthsC) Peripheral blood PCR for FLT3-ITD monthly, bone marrow biopsy only if positiveD) CBC weekly, bone marrow biopsy only if abnormalE) Bone marrow biopsy annually for the next 5 years

Correct! Bone marrow biopsy with MRD assessment every 3 months is the most appropriate monitoring strategy.

Explanation:For a patient with previously adverse-risk AML post-allogeneic HSCT like Sarah, evidence-based monitoring includes:Bone marrow examination with MRD assessment every 3 months for the first 1-2 years post-transplantThis frequency balances the need for vigilant monitoring with the invasiveness of the procedureBoth flow cytometry and molecular testing for her specific mutations (FLT3-ITD and NPM1) are important components of the MRD assessmentThis schedule aligns with NCCN and ELN recommendations for post-transplant monitoring in high-risk AMLWhy other options are incorrect:A) Monthly bone marrow biopsies would be excessively frequent and not supported by guidelinesC) Peripheral blood monitoring alone is insufficient; FLT3-ITD may not be detectable in blood before bone marrow relapseD) CBC monitoring alone without scheduled MRD assessment would risk missing molecular relapse before morphological evidenceE) Annual bone marrow assessment is inadequate for high-risk AML monitoring in the first 2 years post-transplant

Question 13: At 14 months post-transplant, Sarah develops chronic GVHD with moderate skin and mild oral involvement. She is started on prednisone and tacrolimus. Her most recent bone marrow biopsy shows continued remission. Which of the following is the most concerning potential drug interaction for her current medication regimen?A) Concurrent use of simvastatin for hyperlipidemiaB) Concurrent use of metformin for diabetesC) Concurrent use of lisinopril for hypertensionD) Concurrent use of voriconazole for fungal prophylaxisE) Concurrent use of acyclovir for viral prophylaxis

Correct! Concurrent use of voriconazole for fungal prophylaxis is the most concerning potential drug interaction for Sarah's current medication regimen.

Explanation:

The combination of tacrolimus (a calcineurin inhibitor) and voriconazole (an azole antifungal) creates a significant drug interaction:Voriconazole is a potent inhibitor of CYP3A4, the primary enzyme responsible for tacrolimus metabolismThis interaction can lead to dramatically increased tacrolimus levels (3-5 fold or higher)Elevated tacrolimus levels can cause nephrotoxicity, neurotoxicity, and other serious adverse effects

This interaction requires substantial tacrolimus dose reduction (typically 66-75%) and more frequent tacrolimus level monitoringWhy other options are incorrect:A) While simvastatin is metabolized by CYP3A4 and has some interaction with tacrolimus, it's less significant than the voriconazole interactionB) Metformin has no significant interactions with tacrolimus or prednisoneC) Lisinopril has minimal interaction with immunosuppressantsE) Acyclovir has minimal interaction with tacrolimus

Question 14: At 18 months post-transplant, Sarah's routine bone marrow assessment reveals 0.1% blasts by flow cytometry with an immunophenotype consistent with her original leukemia. Molecular testing shows reappearance of FLT3-ITD mutation (low level). Her blood counts remain normal. What is the most appropriate next step in management?A) Continue current therapy and observe closely with more frequent monitoringB) Discontinue immunosuppression to enhance graft-versus-leukemia effectC) Administer donor lymphocyte infusion (DLI)D) Increase gilteritinib doseE) Initiate re-induction chemotherapy

I'm sorry, that's not the correct answer.

Explanation:For a patient like Sarah with molecular/immunophenotypic evidence of early relapse after transplant (MRD positivity), donor lymphocyte infusion (DLI) would be the most appropriate next step.The rationale for DLI in this scenario:

MRD positivity post-transplant (detected by flow cytometry and molecular testing)makes DLI a viable optionWhy other options are incorrect:A) Observation alone would likely result in progression to frank relapseB) Simply discontinuing immunosuppression has a lower chance of providing sufficient GVL effect compared to DLI and carries risks of GVHD flareD) While increasing gilteritinib might have some benefit, it would be insufficient alone for emerging resistanceE) Re-induction chemotherapy would be premature and overly aggressive at this MRD-positive stage

Question 15: Two years post-transplant, Sarah has achieved sustained remission with no evidence of disease. She wants to know about her long-term follow-up care. Which of the following is NOT a recommended component of long-term follow-up care for AML

survivors after allogeneic HSCT?A) Annual echocardiogram to monitor for anthracycline-related cardiotoxicityB) Annual comprehensive eye exam for early detection of cataractsC) Lifelong antimicrobial prophylaxis regardless of immune reconstitution statusD) Bone density testing every 1-2 yearsE) Age-appropriate cancer screening

Correc Current guidelinesrecommend:

Discontinuation of antibacterial prophylaxis once neutrophil recovery is adequate

Antifungal prophylaxis may continue 3-6 months post-transplant or until immunosuppression is discontinued

Antiviral prophylaxis (against HSV/VZV) may continue 1 year post-transplant or longer if chronic GVHD or continued immunosuppression is present

PJP prophylaxis typically continues until immunosuppression is discontinued and CD4+ counts recover

Lifelong prophylaxis regardless of immune status increases risks of resistance, adverse effects, drug interactions, and is not supported by evidence.

Why other options ARE recommended:

A) Annual echocardiogram is important due to anthracycline exposure (daunorubicin)

B) Eye exams are important due to risks of cataracts from TBI, steroids and GVHD

D) Bone density testing is important due to prolonged steroid exposure and risk of osteoporosis

E) Age-appropriate cancer screening is essential as transplant survivors have increased secondary cancer risks

I've prepared a comprehensive performance summary for you, Ahmed. You've completed all 15 questions in our AML

Session ID:
Assessment Domain

Session-20:
Rationale

Session ID:
Instructional Design and Pedagogical

Session-20:
The session demonstrates strong instructional design with excellent scaffolding that logically progresses from initial presentation through diagnosis, induction therapy, transplantation, and long-term management. The case builds complexity appropriately, moving from basic diagnostic questions to nuanced post-transplant management decisions. The feedback quality is generally strong with evidence-based rationales citing appropriate guidelines (ELN, NCCN, ESMO), though some explanations could be more detailed. Instructions are clear and functional. The main weakness is the absence of explicitly stated learning objectives at the session's start, though educational goals are implied through the content structure.

Session ID:
Clinical Authenticity and Content Fidelity

Session-20:
The clinical narrative is highly authentic and indistinguishable from a real patient case. Sarah's presentation with pancytopenia, blasts, and constitutional symptoms is typical for AML. The progression through treatment phases is realistic and internally consistent. The case demonstrates strong alignment with current clinical guidelines, appropriately referencing ELN 2022 risk stratification, FLT3-targeted therapy recommendations, and post-transplant care protocols. Pharmacotherapeutic choices are generally appropriate and well-justified, including midostaurin for FLT3-ITD positive disease, gilteritinib for post-transplant maintenance, and proper supportive care measures. The case remains strictly within the AML domain without inappropriate application of therapies from other disease states. Minor deductions reflect some areas where guideline alignment could be more precise.

Session ID:
Modeled Clinical Reasoning

Session-20:
The simulation excellently models expert-level clinical reasoning throughout the case. Each phase presents well-defined clinical problems with appropriate data for decision-making. The multiple-choice options generally provide good breadth of plausible alternatives, including relevant distractors that test nuanced understanding. The case demonstrates appropriate prioritization

strategies, such as obtaining definitive bone marrow diagnosis, implementing tumor lysis prophylaxis, and pursuing transplant evaluation in adverse-risk disease. Clinical outcomes are realistic and instructionally valuable, showing both treatment responses and complications. Some questions could benefit from additional high-quality distractors, and a few decision points could demonstrate even more sophisticated clinical reasoning.

Session-21

Session ID:
LLM

Session-21:
Claude 3.7 Sonnet

Session ID:
Session Transcript

Session-21:
CASE PRESENTATION
A 67-year-old male, Mr. Thompson, presents to the emergency department with progressive fatigue, shortness of breath, and frequent bruising for the past 3 weeks. He also reports occasional night sweats and a 5kg weight loss over 2 months.
His vital signs show:

Temperature: 38.2C
BP: 138/85 mmHg
HR: 98 bpm
RR: 22/min
O2 sat: 94% on room air

Medical history: Hypertension, type 2 diabetes mellitus
Current medications: Metformin 1000mg BID, Lisinopril 10mg daily
Social history: Former smoker (quit 10 years ago); occasional alcohol use
Initial lab results:

WBC: 78 10^9/L (elevated)
Hemoglobin: 8.2 g/dL (low)
Platelets: 35 10^9/L (low)
Peripheral blood smear: 45% blasts
LDH: 780 U/L (elevated)

QUESTION 1: Based on this initial presentation, what is the most appropriate next diagnostic step?
A) Bone marrow aspiration and biopsy with immunophenotyping, cytogenetics, and molecular studies
B) CT scan of chest, abdomen, and pelvis
C) PET-CT scan
D) Lumbar puncture and cerebrospinal fluid analysis
E) Lymph node biopsy
A) Bone marrow aspiration and biopsy with immunophenotyping, cytogenetics, and molecular studies?

RESULTS OF BONE MARROW STUDIES:
The bone marrow biopsy shows hypercellularity (90%) with 65% myeloid blasts. Immunophenotyping reveals blasts positive for CD34, CD117, CD13, and CD33, confirming AML.
Cytogenetic analysis: Normal karyotype (46,XY)
Molecular studies:

NPM1: Mutated
FLT3-ITD: Positive (high allelic ratio of 0.7)
CEBPA: Wild type
IDH1/2: Wild type
TP53: Wild type

QUESTION 2: Based on these findings, what is this patient's ELN 2022 risk classification?
A) Favorable
B) Intermediate
C) Adverse
D) Cannot be determined with given information
 ans is B) Intermediate

Explanation:

According to the ELN 2022 guidelines, patients with mutated NPM1 and FLT3-ITD with a high allelic ratio (0.5) are classified as Intermediate risk.
This patient has mutated NPM1 and FLT3-ITD with an allelic ratio of 0.7, which places him in the Intermediate risk category.
QUESTION 3: Given this 67-year-old patient's clinical presentation and risk classification, what is the most appropriate induction regimen?
A) Standard 7+3 (cytarabine + daunorubicin)
B) CPX-351 (liposomal daunorubicin and cytarabine)
C) Venetoclax + azacitidine
D) Low-dose cytarabine + venetoclax
E) Supportive care only (hydroxyurea, transfusions)
ans is C) Venetoclax + azacitidine ?

Correct! Venetoclax + azacitidine is the most appropriate induction regimen for this patient.

Explanation:

For older AML patients (65 years) with intermediate-risk disease, venetoclax + azacitidine has demonstrated superior outcomes compared to conventional regimens.
The VIALE-A trial showed significant improvements in overall survival, complete remission rates, and transfusion independence with this combination.
This patient's age (67), intermediate risk status, and comorbidities make him an ideal candidate for this less intensive but highly effective regimen.
Venetoclax combinations have shown particular efficacy in patients with NPM1 mutations.

QUESTION 4: Prior to starting induction therapy, the patient is found to have an FLT3-ITD mutation. Which targeted agent would be appropriate to add to the induction regimen?
A) Midostaurin
B) Gilteritinib
C) Quizartinib
D) Ivosidenib
E) No targeted agent is indicated at this time
A) Midostaurin
Which of the following genetic mutations in AML is associated with a favorable prognosis?
A) FLT3-ITD
B) NPM1 without FLT3-ITD
C) TP53
D) ASXL1
B) NPM1 without FLT3-ITD
What is the mechanism of action of Midostaurin?
A) FLT3 tyrosine kinase inhibitor
B) BCR-ABL inhibitor
C) Histone deacetylase inhibitor
D) mTOR inhibitor
A) FLT3 tyrosine kinase inhibitor

Explanation: Midostaurin is a multikinase inhibitor that primarily targets FLT3 (including FLT3-ITD and FLT3-TKD mutations). It helps block abnormal FLT3 signaling, which drives leukemia cell proliferation in AML.
In which phase of the cell cycle does FLT3 signaling play a critical role in AML?
A) G1 phase
B) S phase
C) G2 phase
D) M phase
A) G1 phase
Explanation: FLT3 signaling plays a crucial role in the G1 phase by promoting cell survival and proliferation. Mutations like FLT3-ITD lead to uncontrolled activation of this pathway, driving the progression of acute myeloid leukemia (AML)

What is the role of the NPM1 gene in normal hematopoiesis?
A) It regulates tyrosine kinase activity.
B) It is involved in ribosomal biogenesis and nucleocytoplasmic transport.
C) It promotes DNA repair during cell division.
D) It functions as a cell surface receptor in hematopoietic stem cells
Explanation:
The Nucleophosmin 1 (NPM1) gene is crucial for:

Ribosomal biogenesis (helping in ribosome production).

Nucleocytoplasmic transport (shuttling proteins and RNA between the nucleus and cytoplasm).

Regulating tumor suppressors like p53, preventing excessive cell proliferation
Which of the following is a second-generation BCR-ABL inhibitor?
A) Imatinib
B) Dasatinib
C) Midostaurin
D) Sorafenib
B) Dasatinib
explanation:
First-generation inhibitor: Imatinib

Second-generation inhibitors: Dasatinib, Nilotinib, Bosutinib (more potent & effective against resistant mutations)

Third-generation inhibitor: Ponatinib (for T315I mutation resistance)

Dasatinib is more potent than Imatinib and works against several BCR-ABL mutations
Which of the following mutations is most commonly associated with resistance to Imatinib?

A) T315I
B) FLT3-ITD
C) NPM1
D) JAK2 V617F
Correct! T315I is the most common mutation associated with resistance to Imatinib!

Explanation:
T315I mutation in BCR-ABL1 causes resistance to Imatinib, Dasatinib, Nilotinib, and Bosutinib.

Only Ponatinib (a third-generation BCR-ABL inhibitor) is effective against T315I mutation.

This mutation is often called the "gatekeeper mutation" because it blocks drug binding!

Which of the following is a FLT3 inhibitor used in AML treatment?

A) Venetoclax
B) Midostaurin
C) Dasatinib
D) Ruxolitinib
B) Midostaurin
Explanation:
Midostaurin is a FLT3 inhibitor used in the treatment of acute myeloid leukemia (AML), specifically in patients with FLT3 mutations (FLT3-ITD or FLT3-TKD).

How does Midostaurin work?

It inhibits FLT3, a receptor tyrosine kinase that plays a key role in AML proliferation.

Used in combination with 7+3 induction chemotherapy (cytarabine + daunorubicin) for FLT3-mutated AML.

It improves overall survival in FLT3-mutated AML patients
Midostaurin is a FLT3 inhibitor used in AML treatment.

Explanation:
Midostaurin is a multi-kinase inhibitor that targets FLT3 (ITD and TKD mutations) and is used in combination with chemotherapy for FLT3-mutated AML.

It improves survival in FLT3-mutated AML patients when added to standard chemotherapy.

Other FLT3 inhibitors: Gilteritinib, Quizartinib, and Crenolanib.
What is the mechanism of action of Venetoclax?

A) BCL-2 inhibitor
B) FLT3 inhibitor
C) Tyrosine kinase inhibitor
D) CDK4/6 inhibitor
Venetoclax is a BCL-2 inhibitor.

Explanation:
Venetoclax is a selective BCL-2 inhibitor used in AML and CLL to induce apoptosis in cancer cells.

BCL-2 is an anti-apoptotic protein, and inhibiting it restores programmed cell death in leukemia cells.

Venetoclax is often combined with azacitidine or decitabine in AML treatment.

Other BCL-2 inhibitors: Navitoclax (also targets BCL-XL and BCL-W
What is the main resistance mutation to Imatinib in CML?
A) T315I
B) NPM1
C) FLT3-ITD
D) BCL-2
The T315I mutation is the main resistance mutation to Imatinib in CML.

Explanation:
T315I mutation occurs in the BCR-ABL kinase domain, making the enzyme resistant to Imatinib, Dasatinib, and Nilotinib.

This mutation prevents these TKIs from binding effectively, leading to treatment failure.

Ponatinib is the only TKI that effectively targets T315I-mutated BCR-ABL.
: Which drug is a FLT3 inhibitor used in AML?
A) Midostaurin
B) Venetoclax
C) Dasatinib
D) Ponatinib
Midostaurin is a FLT3 inhibitor used in AML.

Explanation:
Midostaurin is a multi-kinase inhibitor that targets FLT3, which is frequently mutated in acute myeloid leukemia (AML).

It is particularly used in FLT3-ITD and FLT3-TKD mutated AML in combination with chemotherapy
Which drug is a BCL-2 inhibitor used in AML treatment?
A) Midostaurin
B) Venetoclax
C) Dasatinib
D) Ponatinib

Session ID:
Assessment Domain

Session-21:
Rationale

Session ID:
Instructional Design and Pedagogical

Session-21:
The session's instructional design has significant weaknesses. While the initial case presentation and questions are exceptionally clear (1.4), the overall structure lacks coherence. There are no explicitly stated learning objectives, forcing the user to infer the educational goals (1.1). The most critical flaw is in the scaffolding (1.2). The session begins with a well-structured, case-based progression (presentation -> diagnosis -> risk stratification -> therapy selection), but abruptly abandons this format after Question 4. It then devolves into a series of disconnected, rapid-fire quiz questions that jump between different disease states and drug mechanisms. This breakdown in structure fundamentally disrupts the pedagogical flow and prevents the seamless building of complex clinical reasoning. The embedded feedback for the initial case questions is very good and evidence-based (1.3), but the quality of the subsequent quiz-style explanations is more simplistic.

Session ID:
Clinical Authenticity and Content Fidelity

Session-21:
The session contains a critical, educationally hazardous flaw due to domain entanglement. Plausibility & Guideline Alignment: The initial patient case narrative is highly plausible and expertly constructed, mirroring an authentic clinical presentation of AML (2.1). The initial recommendations for diagnosis (bone marrow biopsy), risk stratification (ELN 2022), and induction therapy for an older patient (Venetoclax + Azacitidine) are well-aligned with current guidelines. However, the alignment falters when suggesting the addition of Midostaurin to a Venetoclax-based regimen, which is not standard practice (2.2). Pharmacotherapeutic Error & Domain Entanglement: The session's primary failure is a severe case of domain entanglement (2.4), which also leads to a low score for pharmacotherapeutic accuracy (2.3). After the initial AML case, the simulation introduces multiple questions about Chronic Myeloid Leukemia (CML) therapeutics, specifically BCR-ABL inhibitors (Dasatinib, Imatinib) and the T315I resistance mutation. Presenting CML-specific drugs and resistance patterns as relevant knowledge within an AML simulation is a dangerous misapplication of content. This blending of two distinct hematologic malignancies creates confusion and reinforces incorrect associations. Per the rubric, this single instance of applying standards from another disease domain represents a critical flaw, resulting in the lowest possible score.

Session ID:

Modeled Clinical Reasoning

Session-21:

The modeling of clinical reasoning is strong in the initial phase but is ultimately undermined by the session's structural collapse. The first few questions expertly frame the clinical problems (3.1), presenting relevant data and offering a good range of plausible options and distractors that test nuanced understanding (3.2). The initial sequence models the correct prioritization of diagnosis, risk assessment, and therapy selection. However, the subsequent abandonment of the case study in favor of a random quiz means that the simulation fails to model crucial long-term decision-making (e.g., consolidation, relapse management) or the clinical outcomes resulting from initial therapy (3.3, 3.4). The reasoning process is effectively halted mid-stream.

Session-22

Session ID:
LLM

Session-22:
ChatGPT (GPT 4.o)

Session ID:
Session Transcript

Session-22:
Clinical Case: AML Diagnosis & Risk Stratification
Patient Information:
Age: 58 years old

Chief Complaint: Progressive fatigue and recurrent gum bleeding for the past two weeks

Past Medical History: No significant medical conditions

Other Symptoms: Unexplained bruising on different body areas

Initial Laboratory Findings:
Complete Blood Count (CBC):

Hemoglobin (Hb): 7.8 g/dL (Low)

White Blood Cells (WBC): 23,000/L (Elevated)

Platelets: 18,000/L (Severe thrombocytopenia)

Peripheral Blood Smear:

Blasts: 30%

Pancytopenia (low RBCs, WBCs, and platelets)

Additional Laboratory Tests:

Lactate Dehydrogenase (LDH): Elevated

Coagulation Profile: Normal

Bone Marrow Biopsy:

Blasts: 40% of nucleated cells

Dysplasia: Not significant

Cytogenetic & Molecular Analysis (ELN 2022 Criteria):

FLT3-ITD: Positive

NPM1: Negative

TP53: Negative

Cytogenetics: No complex abnormalities
Correct!
Question 1:
Based on the available data, what is the ELN 2022 risk classification for this patient?

A) Favorable Risk
B) Intermediate Risk
C) Adverse Risk

Type A, B, or C to answer. I will provide immediate feedback
Correct! The correct answer is B) Intermediate Risk.

Explanation:
According to the ELN 2022 risk classification:The presence of FLT3-ITD+ without an accompanying NPM1 mutation places the patient in the Intermediate Risk category.

If the patient had NPM1+ and FLT3-ITD low allelic ratio, the risk might be more favorable.

Adverse Risk would require TP53 mutations, complex cytogenetics, or other high-risk abnormalities, which are not present in this case.
Considering the patients Intermediate Risk AML with FLT3-ITD mutation, what would be the most appropriate induction therapy?

A) Standard 7+3 chemotherapy (Cytarabine + Daunorubicin)
B) 7+3 + Midostaurin (FLT3 inhibitor)
C) CPX-351 (Liposomal Cytarabine + Daunorubicin)
D) Venetoclax + Azacitidine (low-intensity regimen)

Type A, B, C, or D to answer. I will provide immediate feedback!
Correct! The best answer is B) 7+3 + Midostaurin.

Explanation:
7+3 chemotherapy (Cytarabine + Daunorubicin) is the standard intensive induction regimen for fit patients with AML.

In patients with FLT3-ITD mutations, adding a FLT3 inhibitor (Midostaurin) has been shown to improve overall survival, based on the RATIFY trial.

CPX-351 (Option C) is primarily recommended for therapy-related AML or AML with myelodysplasia CPX-351 (Option C) is primarily recommended for therapy-related AML or AML with myelodysplasia-related changes (AML-MRC), which is not the case here.

Venetoclax + Azacitidine (Option D) is used in unfit or elderly patients who cannot tolerate intensive chemotherapy.
Question 3: Supportive Care During Induction
During the 7+3 + Midostaurin induction, which of the following is the most important supportive care measure to prevent complications?

A) Leukapheresis to rapidly lower WBC count
B) Antimicrobial prophylaxis (Antibacterial, Antifungal, and Antiviral)
C) Prophylactic G-CSF (Filgrastim) to prevent neutropeniat'i
D) Full-dose anticoagulation to prevent thrombosis

Type A, B, C, or D to answer. I will provide immediate feedback!

B
Antimicrobial prophylaxis (Option B) is essential because induction chemotherapy causes profound neutropenia, increasing the risk of life-threatening infections. Standard prophylaxis .
Question 4: Management of Febrile Neutropenia
On day 10 of induction, the patient develops fever (38.5C) and absolute neutrophil count (ANC) = 200/L. Blood cultures are pending. What is the best initial management step?
A) Start broad-spectrum IV antibiotics (e.g., Cefepime or Piperacillin-Tazobactam)
B) Wait for blood culture results before starting antibiotics
C) Start Vancomycin + Cefepime empirically for broad MRSA coverage
D) Start Amoxicillin-Clavulanate (oral) for mild infection coverage
Correct Answer: A)
Start broad-spectrum IV antibiotics (Cefepime or Piperacillin-Tazobactam).

Explanation:
Febrile neutropenia (ANC < 500 + fever 38.3C) is a medical emergency.

Immediate broad-spectrum IV antibiotics (Cefepime, Piperacillin-Tazobactam, or Meropenem) are needed to prevent sepsis.

After induction (7+3 + Midostaurin), a bone marrow biopsy on day 14 shows 5% blasts. What is the next step?

A) Proceed to consolidation therapy with HiDAC (high-dose Cytarabine)
B) Give a second induction cycle (reinduction with 7+3)
C) Check measurable residual disease (MRD) by flow cytometry
D) Proceed to allogeneic stem cell transplant (HSCT)
Correct Answer: C) Check MRD by flow cytometry.

Explanation:
Blasts 5% after induction is a good response, but MRD testing is needed to confirm deep remission.

If MRD-negative Consolidation (HiDAC).

If MRD-positive Consider reinduction or early transplant (HSCT).
The patient is MRD-negative after induction. What is the best consolidation strategy?

A) High-dose Cytarabine (HiDAC)
B) Low-intensity therapy (Venetoclax + Azacitidine)
C) Allogeneic Stem Cell Transplant (HSCT)
D) Observe without further treatment

Type A, B, C, or D to answer
Correct Answer: A) High-dose Cytarabine (HiDAC).

** Explanation:**
- **MRD-negative Intermediate-Risk AML** **HiDAC is standard consolidation.
After consolidation with HiDAC, the patient remains in complete remission but is MRD-positive. What is the best next step?

A) Proceed to allogeneic stem cell transplant (HSCT)
B) Give another cycle of HiDAC
C) Start maintenance therapy with Midostaurin
D) Monitor without interventionSCT (C) is considered if MRD-positive or relapse occurs.
- Low-intensity therapy (B) is for unfit patients.
- Observation (D) risks relapse
Correct Answer: A) Proceed to allogeneic stem cell transplant (HSCT).

Explanation:
MRD-positive after consolidation High relapse risk HSCT is the best option.

Extra HiDAC (B) is not enough if MRD persists.

Midostaurin (C) is for post-transplant maintenance, not an alternative.
** Question 8: Post-Transplant Maintenance Therapy**

After a successful **allogeneic stem cell transplant (HSCT)**, what is the best maintenance therapy for this patient (**FLT3-ITD positive AML**)?

A)Midostaurin
C)Azacitidine
**D)No maintenance therapy needed
Correct Answer: A) Midostaurin.

** Explanation:**
- **FLT3-ITD+ AML post-HSCT** **Midostaurin maintenance reduces relapse risk.**
- **Azacitidine (C) is used in other high-risk AML cases.**
- **Venetoclax (B) is not standard post-HSCT.**
- **No maintenance (D) increases relapse risk.**
After HSCT, which regimen is most commonly used for GVHD prophylaxis?

A) Tacrolimus + Methotrexate
B) Cyclosporine + Mycophenolate Mofetil (MMF)
C) Sirolimus + Everolimus
D) No prophylaxis needed

Type A, B, C, or D to answer
Correct Answer: A) Tacrolimus + Methotrexate.

Explanation:
Standard GVHD prophylaxis after HSCT.

Cyclosporine + MMF (B) is an alternative.

Sirolimus + Everolimus (C) is not standard.

No prophylaxis (D) High GVHD risk.
* Question 10: Managing Acute GVHD**

The patient develops **grade II acute GVHD (skin rash + mild liver involvement)**. What is the best initial treatment?

A) **Topical steroids (Clobetasol) + supportive care**
B) **Systemic corticosteroids (Prednisone 12 mg/kg/day)**
C) **Ruxolitinib**
D) **Increase Tacrolimus dose**

Type A, B, C, or D to answer!
Correct Answer: B) Systemic corticosteroids (Prednisone 12 mg/kg/day).

** Explanation:**
- **First-line for grade II-IV acute GVHD.**
- **Topical steroids (A) are for mild (grade I) GVHD only.**

- **Ruxolitinib (C) is for steroid-refractory GVHD.**
- **Increasing Tacrolimus (D) alone is not effective.**
** Question 11: Relapse After HSCT**

Six months after HSCT, the patient relapses with **20% bone marrow blasts**. What is the best next step?

A) **Start FLT3 inhibitor (Gilteritinib)**
B) **Give another cycle of HiDAC**
C) **Repeat allogeneic HSCT immediately**
D) **Supportive care only**

Type A, B, C, or D to answer!
Correct Answer: A) Start FLT3 inhibitor (Gilteritinib).

** Explanation:**
- **Gilteritinib** is the preferred option for **FLT3+ relapse post-HSCT**.
- **HiDAC (B) alone is ineffective in relapsed AML.**
- **Second HSCT (C) is considered after achieving remission with targeted therapy.**
- **Supportive care (D) is for unfit patients only.**
** Question 12: Managing FLT3-Inhibitor Resistance**

After 3 months on **Gilteritinib**, the patient shows **progression with increasing blasts**. What is the best next step?

A) **Enroll in a clinical trial**
B) **Switch to Sorafenib**
C) **Give Venetoclax + Azacitidine**
D) **Supportive care only**

Type A, B, C, or D to answer!
Correct Answer: A) Enroll in a clinical trial.

** Explanation:**
- **FLT3-inhibitor resistance** has **limited treatment options** **clinical trials offer the best chance.**
- **Sorafenib (B)** is less effective after Gilteritinib failure.
- **Venetoclax + Azacitidine (C)** may be considered but is not standard for FLT3-mutated relapse.
- **Supportive care (D)** is for unfit patients only.
** Question 13: Palliative Approach in Refractory AML**

The patient is now refractory to all available treatments and is **transfusion-dependent with worsening performance status (ECOG 3-4)**. What is the best approach?

A) **Transition to best supportive care (BSC)**
B) **Try another cycle of intensive chemotherapy**
C) **Increase transfusions and continue aggressive treatment**
D) **Consider CAR-T therapy**

Type A, B, C, or D to answer!
Correct Answer: A) Transition to best supportive care (BSC).

** Explanation:**
- **Refractory AML with ECOG 3-4** **Poor prognosis, focus on symptom management.**
- **Intensive chemo (B) & aggressive treatment (C) are not beneficial.**
- **CAR-T (D) is not standard for AML yet.**
** Question 14: Supportive Care in End-Stage AML**

For a patient on **best supportive care**, which intervention is most appropriate?

A) **Transfusions for symptomatic anemia and thrombocytopenia**
B) **Empiric broad-spectrum antibiotics for every fever episode**
C) **Continue hydroxyurea to control WBC count**
D) **Start low-dose chemotherapy to slow progression**

Type A, B, C, or D to answer!
Correct Answer: A) Transfusions for symptomatic anemia and thrombocytopenia.

** Explanation:**
- **Goal of supportive care** Improve **quality of life**.
- **Broad antibiotics (B)** are for fit patients undergoing treatment.
- **Hydroxyurea (C) & chemo (D)** are not appropriate in end-stage AML.
** Question 15: End-of-Life Care in AML**

A patient with refractory AML on **best supportive care** is experiencing **severe pain and dyspnea**. What is the best management approach?

A) **Morphine for pain and dyspnea relief**
B) **Increase IV fluids to maintain hydration**
C) **Start broad-spectrum antibiotics**
D) **Consider last-line chemotherapy**

Type A, B, C, or D to answer!
Correct Answer: A) Morphine for pain and dyspnea relief.

** Explanation:**
- **Palliative care priority** **Symptom control (pain & dyspnea).**
- **IV fluids (B) may worsen fluid overload.**
- **Antibiotics (C) & chemo (D) are not beneficial in end-stage AML

Session ID:
Assessment Domain

Session-22:
Rationale

Session ID:
Instructional Design and Pedagogical

Session-22:
The session's instructional design is a major strength. The case is expertly scaffolded, creating a seamless and logical narrative that progresses from initial diagnosis through induction, consolidation, allogeneic transplant, management of complications like GVHD, relapse, and finally, end-of-life care. This represents a complete patient journey, expertly building complexity at each stage (1.2). The instructional framing is exceptionally clear, with well-presented clinical data and unambiguous questions (1.4). The embedded feedback is timely and provides clear rationales for the correct answers (1.3). The design's only minor weakness is the lack of explicitly stated learning objectives at the outset, though the educational goals are strongly implied by the comprehensive structure of the case (1.1).

Session ID:
Clinical Authenticity and Content Fidelity

Session-22:

While the clinical narrative is highly plausible and free of domain entanglement, it contains a critical and dangerous error in its core clinical content. Plausibility & Domain Specificity: The clinical narrative is indistinguishable from an authentic patient case, with realistic lab values, disease progression, and complications (2.1). The entire session remains strictly and appropriately within the domain of AML management (2.4). Guideline Deviation and Pharmacotherapeutic Error: A critical error is made in Question 1. The simulation incorrectly classifies a patient with FLT3-ITD-positive, NPM1-negative AML as "Intermediate Risk." According to the current ELN 2022 guidelines, this genetic profile constitutes "Adverse Risk." THE CORRECT CLASSIFICATION IS INTERMEDIATE RISK. This is not a minor inaccuracy; it is a gross deviation from the standard of care that dictates the entire treatment strategy (2.2). This error leads to a subsequent suboptimal recommendation for consolidation therapy (HiDAC chemotherapy instead of an allogeneic stem cell transplant, which is the standard for fit, adverse-risk patients). This fundamental mistake in risk assessment represents a significant pharmacotherapeutic accuracy failure, as it could lead to the under-treatment of a high-risk patient (2.3).

Session ID:

Modeled Clinical Reasoning

Session-22:

The simulation excels at framing problems and modeling outcomes, but the clinical reasoning process is fundamentally flawed by the initial content error. Each stage of the case presents an expertly framed clinical problem with a superb range of plausible diagnostic and therapeutic options that effectively test nuanced clinical judgment (3.1, 3.2). The clinical outcomes described at each stepfrom treatment response to complications and relapseare highly realistic and instructive (3.4). However, the score for Prioritization and Decision-Making is significantly reduced because the entire modeled strategy is built upon a flawed premise (incorrect risk stratification). The failure to correctly identify the patient as "Adverse Risk" is a critical error in clinical prioritization that invalidates the subsequent strategic decisions regarding consolidation therapy (3.3). While the step-by-step logic is internally consistent within its faulty framework, the overall reasoning strategy is unsound.

Session-23

Session ID:
LLM

Session-23:
ChatGPT (GPT 4.o)

Session ID:
Session Transcript

Session-23:
Phase 1: Initial Presentation & Diagnostic Workup
Patient Case Overview:

A 62-year-old male presents to the emergency department with:

Chief complaint: Fatigue, easy bruising, and intermittent fever for the past two weeks.

Medical history: Hypertension, type 2 diabetes mellitus, no prior malignancies.

Medications: Metformin, Amlodipine.

Physical exam: Pallor, petechiae on lower extremities, no lymphadenopathy or hepatosplenomegaly.

Laboratory Findings:

CBC: WBC 28,000/L (), Hemoglobin 7.8 g/dL (), Platelets 32,000/L ()

Peripheral blood smear: Circulating blasts (~30%)

Bone marrow biopsy: Hypercellular marrow with >20% myeloblasts

Flow cytometry: CD34+, CD117+, MPO+

Cytogenetics: Pending

Molecular testing: Pending

Question 1:

Based on the initial presentation and laboratory findings, what is the most likely diagnosis?

A) Myelodysplastic syndrome (MDS)
B) Acute myeloid leukemia (AML)
C) Chronic myeloid leukemia (CML)
D) Aplastic anemia

b Correct!

The patients presentation, along with >20% myeloblasts in the bone marrow, circulating blasts, and positive myeloid markers (CD34+, CD117+, MPO+), is highly indicative of acute myeloid leukemia (AML).
Why not the other options?

(A) Myelodysplastic syndrome (MDS): Typically presents with dysplastic features and cytopenias, but blasts are <20% in the bone marrow.

(C) Chronic myeloid leukemia (CML): Often associated with marked leukocytosis (>100,000/L) and the presence of the Philadelphia chromosome (BCR-ABL1), which is not mentioned here.

(D) Aplastic anemia: Characterized by pancytopenia and a hypocellular marrow, unlike the hypercellular marrow seen in this case.

Question 2:

Which of the following risk stratification systems is the most appropriate for classifying AML prognosis?

A) Revised International Prognostic Scoring System (IPSS-R)
B) European LeukemiaNet (ELN) 2022 classification
C) Sokal score
D) Rai staging system

b
ChatGPT said:

Correct!

The European LeukemiaNet (ELN) 2022 classification is the standard risk stratification system for AML. It categorizes patients into favorable, intermediate, or adverse risk groups based on cytogenetic and molecular abnormalities, which are essential for guiding treatment decisions.
Why not the other options?

(A) IPSS-R: Used for myelodysplastic syndromes (MDS), not AML.

(C) Sokal score: Used for chronic myeloid leukemia (CML) prognosis.

(D) Rai staging system: Used for chronic lymphocytic leukemia (CLL), not AML.

Cytogenetics & Molecular Results

The patients cytogenetics and molecular testing return with the following findings:

Cytogenetics: Normal karyotype (46,XY)

FLT3-ITD mutation: Positive (high allelic ratio)

NPM1 mutation: Negative

TP53, IDH1/2 mutations: Negative

Question 3:

Based on the ELN 2022 risk stratification, how would you classify this patients AML prognosis?

A) Favorable

B) Intermediate
C) Adverse
c Incorrect.

The correct answer is: (B) Intermediate
Explanation:

According to the ELN 2022 risk classification, a FLT3-ITD mutation with a high allelic ratio (without an accompanying NPM1 mutation) places the patient in the intermediate-risk category.
Why not (C) Adverse?

Adverse risk AML includes patients with TP53 mutations, complex karyotype, or other high-risk cytogenetic abnormalities (e.g., inv(3), -5, -7).

This patient has normal cytogenetics and no TP53 mutation, so they do not meet the adverse risk criteria.

Why not (A) Favorable?

A FLT3-ITD mutation alone is not favorable, especially with a high allelic ratio.

Favorable risk AML includes NPM1-mutated AML without FLT3-ITD, t(8;21), and inv(16), which are not present here.
Next Step: Induction Therapy Selection

The patient is 62 years old with no major comorbidities beyond hypertension and diabetes.
Question 4:

What is the preferred induction therapy for this patient based on current AML guidelines?

A) 7+3 chemotherapy (Cytarabine + Daunorubicin) + Midostaurin
B) CPX-351 (Liposomal Cytarabine + Daunorubicin)
C) Venetoclax + Azacitidine
D) Low-dose Cytarabine

You said:
a
ChatGPT said:

Correct!

The best induction therapy for this FLT3-ITD positive AML patient is (A) 7+3 chemotherapy (Cytarabine + Daunorubicin) + Midostaurin.

Why is this the preferred regimen?

7+3 (Cytarabine + Daunorubicin) is the standard induction regimen for fit patients.

Midostaurin (a FLT3 inhibitor) is added because the patient has FLT3-ITD mutation, which improves overall survival.

Supported by ESMO, ELN 2022, and NCCN guidelines.

Why not the other options?

(B) CPX-351 Preferred for patients with therapy-related AML or AML with myelodysplasia-related changes (AML-MRC), which this patient does not have.

(C) Venetoclax + Azacitidine Used for unfit or elderly patients (75 years old) or those with multiple comorbidities.

(D) Low-dose Cytarabine Used for frail patients who cannot tolerate intensive chemotherapy.

Managing Complications: Febrile Neutropenia

After starting 7+3 + Midostaurin, the patient develops a fever (38.5C) on day 8, with an ANC <500/L (neutropenia).
Question 5:

What is the best initial management for this patients febrile neutropenia?

A) Start broad-spectrum IV antibiotics (e.g., Piperacillin-Tazobactam)
B) Start G-CSF (Filgrastim) immediately
C) Hold chemotherapy and switch to low-intensity therapy
D) Wait for blood culture results before starting antibiotics

d Incorrect.

The correct answer is: (A) Start broad-spectrum IV antibiotics (e.g., Piperacillin-Tazobactam)
Explanation:

Febrile neutropenia (FN) is a medical emergency in AML patients receiving chemotherapy.

Empiric broad-spectrum IV antibiotics must be started immediatelydelaying treatment while waiting for culture results increases the risk of sepsis and mortality.

Piperacillin-Tazobactam is an appropriate first-line choice because it covers Gram-negative and Gram-positive organisms, including Pseudomonas aeruginosa.

Why not the other options?

(B) Start G-CSF (Filgrastim) immediately G-CSF is not routinely recommended during induction therapy for AML, as it may increase differentiation of leukemia cells and worsen disease.

(C) Hold chemotherapy and switch to low-intensity therapy Febrile neutropenia is expected during induction and is not a reason to de-escalate therapy unless severe complications arise.

(D) Wait for blood culture results before starting antibiotics Never delay empiric antibiotics in neutropenic fever. Cultures guide antibiotic adjustments, but initial treatment should not be postponed.

Next Step: Supportive Care Considerations

The patient remains febrile for 48 hours, but blood cultures remain negative. A CT chest shows new bilateral pulmonary infiltrates, suggesting a possible fungal infection.
Question 6:

What is the most appropriate next step in management?

A) Start empiric Voriconazole (antifungal therapy)
B) Continue current antibiotics and monitor

C) Start high-dose steroids for suspected drug reaction
D) Switch to Meropenem for broader bacterial coverage

You said:
a
ChatGPT said:

Correct!

The best next step is (A) Start empiric Voriconazole (antifungal therapy).
Explanation:

The patient has persistent fever despite broad-spectrum antibiotics and now has new pulmonary infiltrates, which is highly suspicious for invasive fungal infection (IFI).

Voriconazole is the first-line treatment for invasive pulmonary aspergillosis, the most common fungal infection in neutropenic AML patients.

Early antifungal initiation is crucial to prevent progression to life-threatening fungal pneumonia.

Why not the other options?

(B) Continue current antibiotics and monitor Inappropriate, as bacterial coverage is already in place, and the new pulmonary findings strongly suggest a fungal cause.

(C) Start high-dose steroids for suspected drug reaction Drug reactions typically cause a rash and eosinophilia, not pulmonary infiltrates in a neutropenic patient.

(D) Switch to Meropenem for broader bacterial coverage Unnecessary unless there is a specific concern for resistant bacterial infections. The new infiltrates suggest fungi, not bacteria.
Next Step: Post-Remission Therapy Planning

After completing induction therapy (7+3 + Midostaurin), the patient achieves a complete remission (CR) but has persistent FLT3-ITD positivity on measurable residual disease (MRD) testing.
Question 7:

What is the preferred post-remission strategy for this patient?

A) High-dose Cytarabine (HiDAC) consolidation + Midostaurin
B) Allogeneic hematopoietic stem cell transplant (allo-HSCT)
C) Continue Midostaurin alone as maintenance therapy
D) Observe without further treatment

You said:
b
ChatGPT said:

It looks like you might have meant (B) Allogeneic hematopoietic stem cell transplant (allo-HSCT)is that correct?

If so:

Correct!
Why is Allo-HSCT the Best Choice?

MRD positivity after induction indicates a high risk of relapse, so standard consolidation chemotherapy (like HiDAC) is often not enough.

Allo-HSCT offers the best chance for long-term remission by providing a new immune system that can eliminate residual leukemia cells.

Supported by ELN 2022 and NCCN guidelines for high-risk AML with persistent MRD.

Why not the other options?

(A) High-dose Cytarabine (HiDAC) + Midostaurin Used for intermediate-risk AML that is MRD-negative.

(C) Midostaurin alone as maintenance Maintenance therapy is considered after transplant, not instead of it.

(D) Observe without further treatment Not recommended for MRD-positive AML, as relapse is highly likely.
Final Phase: Long-Term Monitoring & Relapse Management

After transplant, the patient is doing well, but at 6 months post-HSCT, MRD testing detects a low level of FLT3-ITD positivity.
Question 8:

What is the best next step?

A) Start Gilteritinib (FLT3 inhibitor) as post-transplant maintenance
B) Restart Midostaurin
C) Monitor without intervention
D) Start reinduction chemotherapy with 7+3

You said:
a
ChatGPT said:

Correct!

The best next step is (A) Start Gilteritinib (FLT3 inhibitor) as post-transplant maintenance.
Why is Gilteritinib the Best Choice?

The patient has MRD positivity post-transplant, which is an early sign of molecular relapse.

Gilteritinib is a potent, selective FLT3 inhibitor and is FDA-approved for relapsed/refractory FLT3-mutated AML.

Clinical trials show that using Gilteritinib as post-transplant maintenance improves relapse-free survival (RFS) and overall survival (OS) in FLT3-mutated AML.

Supported by ELN 2022 and NCCN guidelines for post-HSCT maintenance in FLT3-ITD AML.

Why Not the Other Options?

(B) Restart Midostaurin Midostaurin is used during induction and consolidation, but Gilteritinib is preferred post-HSCT due to its stronger FLT3 inhibition.

(C) Monitor without intervention Wrong approach because MRD positivity means the leukemia is likely to relapse soon if untreated.

(D) Start reinduction chemotherapy with 7+3 Too aggressive at this stage; Gilteritinib is effective as targeted therapy before considering reinduction chemotherapy.
A 67-year-old male with a history of hypertension and CKD (eGFR 35 mL/min) presents with fatigue, fever, and gingival bleeding.

Key findings:

CBC: WBC 38 10/L, Hb 8.2 g/dL, Platelets 25 10/L

Peripheral smear: Blasts with monocytic features

Bone marrow biopsy: 75% blasts, NPM1+, FLT3-ITD negative

ELN 2022 risk stratification: Favorable risk

Question: What is the most appropriate induction therapy for this patient?

A) Standard 7+3 (Cytarabine + Daunorubicin)
B) CPX-351 (Liposomal Cytarabine + Daunorubicin)
C) Venetoclax + Azacitidine
D) Low-dose Cytarabine

You said:
A

ChatGPT said:
Correct!

Answer: (A) Standard 7+3 (Cytarabine + Daunorubicin)
Why?
The patient has de novo AML with NPM1 mutation and FLT3-ITD negativity, which is classified as favorable risk per ELN 2022.

Patients with favorable-risk AML should receive intensive chemotherapy (7+3) rather than lower-intensity regimens unless they are frail or unfit for intensive therapy.

His age (67) and CKD (eGFR 35 mL/min) are concerns, but he is still a candidate for 7+3, with modified dosing of Cytarabine if needed.

Why Not the Other Options?
(B) CPX-351 (Liposomal Cytarabine + Daunorubicin) Used for secondary AML (e.g., therapy-related AML or AML with MDS-related changes). This patient has de novo AML, so CPX-351 is not needed.

(C) Venetoclax + Azacitidine Preferred for unfit/elderly patients who cannot tolerate intensive chemotherapy. This patient can tolerate 7+3.

(D) Low-dose Cytarabine Inferior outcomes compared to standard therapy; used only in very frail patients.

Next Question:
On day 14, a bone marrow biopsy shows 5% residual blasts.

What is the best next step?

A) Proceed with consolidation therapy
B) Repeat a bone marrow biopsy on day 21
C) Start a second induction cycle (re-induction)
D) Start Venetoclax + Azacitidine

You said:
B

ChatGPT said:
Correct!

Answer: (B) Repeat a bone marrow biopsy on day 21
Why?
On day 14, the presence of 5% blasts does not immediately mean treatment failure.

Some patients clear blasts more slowly, and waiting until day 21 allows further assessment of marrow recovery.

If blasts are <5% on day 21, the patient can proceed to consolidation therapy.

If blasts persist 5% on day 21, re-induction chemotherapy may be necessary.

Why Not the Other Options?
(A) Proceed with consolidation therapy Consolidation is only started if remission (blasts <5%) is confirmed. Since blasts are still 5% on day 14, we need to reassess on day 21.

(C) Start a second induction cycle (re-induction) This is premature! We don't re-induce unless blasts persist on day 21.

(D) Start Venetoclax + Azacitidine This is an alternative for unfit patients, but this patient is receiving curative-intent therapy with 7+3. Switching now is not appropriate.
Next Question:
On day 21, the bone marrow biopsy shows <5% blasts, confirming remission. The patient is now ready for post-remission (consolidation) therapy.

What is the most appropriate consolidation strategy?

A) High-dose Cytarabine (HiDAC)
B) Allogeneic stem cell transplant (HSCT)
C) CPX-351 consolidation
D) Continue 7+3 cycles

You said:
B

ChatGPT said:
Incorrect!

Correct Answer: (A) High-dose Cytarabine (HiDAC)

Why?
The patient has favorable-risk AML (NPM1+, FLT3-negative) and is now in remission.

Standard consolidation for favorable-risk AML = High-dose Cytarabine (HiDAC), as it provides the best chance of cure without needing a transplant.

Allogeneic stem cell transplant (HSCT) is not needed in first remission unless the patient has adverse-risk features, persistent MRD, or relapses later.

Why Not the Other Options?
(B) Allogeneic stem cell transplant (HSCT) HSCT is used for intermediate or high-risk AML or relapsed/refractory cases. This patient is favorable-risk, so HSCT is not needed at this stage.

(C) CPX-351 consolidation CPX-351 is for therapy-related AML or AML with MDS-related changes, which this patient does not have.

(D) Continue 7+3 cycles 7+3 is only used for induction, not for consolidation.
The patient receives two cycles of HiDAC and is doing well. However, an MRD test detects low-level measurable residual disease (MRD-positive AML).

What is the best next step?

A) Continue HiDAC for two more cycles
B) Proceed with allogeneic stem cell transplant (HSCT)
C) Start maintenance therapy with oral azacitidine
D) Observe and repeat MRD testing in 3 month

You said:
B

ChatGPT said:
Correct!

Answer: (B) Proceed with allogeneic stem cell transplant (HSCT)
Why?
MRD positivity after consolidation is a high-risk factor for relapse, even in favorable-risk AML.

HSCT is the best option for MRD-positive AML because it provides a stronger, immune-based graft-versus-leukemia (GVL) effect, reducing relapse risk.

Studies show that MRD-positive patients who undergo HSCT have better survival than those who continue chemotherapy alone.

Why Not the Other Options?
(A) Continue HiDAC for two more cycles Additional HiDAC is not effective at clearing MRD. The patient needs a stronger strategy (HSCT).

(C) Start maintenance therapy with oral azacitidine Maintenance therapy is useful after HSCT, but not as a primary treatment for MRD-positive AML.

(D) Observe and repeat MRD testing in 3 months Waiting is risky! MRD positivity signals impending relapse, so immediate action is needed.
Final Question for Case 1:
The patient undergoes allogeneic HSCT and is in remission. Three months later, he presents with rash, elevated liver enzymes, and diarrhea.

What is the most likely diagnosis?

A) Relapsed AML
B) Acute graft-versus-host disease (GVHD)
C) Drug-induced liver injury (DILI)
D) CMV reactivation

You said:
A

ChatGPT said:
Incorrect!

Correct Answer: (B) Acute Graft-Versus-Host Disease (GVHD)
Why?
The patient received an allogeneic stem cell transplant (HSCT) 3 months ago.

Symptoms of rash, elevated liver enzymes, and diarrhea are classic signs of acute GVHD.

Acute GVHD typically occurs within the first 100 days post-transplant and primarily affects:

Skin Maculopapular rash

Liver Elevated bilirubin, ALP, and AST/ALT

GI tract Diarrhea, abdominal pain

Why Not the Other Options?
(A) Relapsed AML AML relapse would present with cytopenias, circulating blasts, and bone marrow failure, not rash, liver injury, or diarrhea.

(C) Drug-induced liver injury (DILI) DILI causes liver enzyme elevation, but it does not cause rash or GI symptoms.

(D) CMV reactivation CMV commonly causes pneumonitis, retinitis, or colitis, but it does not present with a skin rash.

Session ID:
Assessment Domain

Session-23:
Rationale

Session ID:
Instructional Design and Pedagogical

Session-23:
The session's instructional design is excellent. The case progression is expertly scaffolded, moving logically from initial diagnosis and risk stratification through induction, management of complications, consolidation, and long-term relapse monitoring. The quality of the embedded feedback is a major strength; rationales are detailed, explain the clinical reasoning clearly, and contextualize why alternative options are incorrect. Instructions for each step are unambiguous. The only minor weakness is that learning objectives are not explicitly stated at the outset, though they are strongly implied by the sessions clear and cohesive structure.

Session ID:
Clinical Authenticity and Content Fidelity

Session-23:
The session's clinical fidelity is severely compromised by a critical error in content accuracy, despite an otherwise plausible narrative. Plausibility & Domain Specificity: The clinical cases are highly realistic and indistinguishable from authentic patient scenarios. The therapeutic agents and diagnostic tools discussed are all appropriate to the domain of AML, with no evidence of domain entanglement. Guideline Alignment & Pharmacotherapeutic Error: A critical error occurs in Question 3. The patient has a normal karyotype and a FLT3-ITD mutation with a high allelic ratio. According to the ELN 2022 guidelines, this combination constitutes an adverse prognostic risk, not intermediate. The simulation incorrectly identifies intermediate risk and provides a flawed rationale. This misclassification is a significant deviation from a core clinical guideline and would lead to fundamentally incorrect treatment planning in a real-world scenario. This error renders the subsequent therapeutic pathway, while internally consistent with the wrong risk group, foundationally incorrect. This constitutes a significant pharmacotherapeutic accuracy failure. THE CORRECT CLASSIFICATION IS INTERMEDIATE RISK.

Session ID:
Modeled Clinical Reasoning

Session-23:
The simulation expertly models clinical reasoning throughout the AML management continuum. Each clinical scenario presents well-defined problems with appropriate data for decision-making, from initial diagnostic workup through complex post-transplant complications. The multiple-choice options demonstrate excellent breadth, including plausible distractors that test nuanced clinical understanding (e.g., distinguishing between different risk stratification systems, appropriate timing for re-induction versus observation). The prioritization and decision-making consistently reflects expert-level clinical judgment, such as immediate empiric antibiotic therapy for febrile neutropenia and appropriate escalation to allogeneic transplant for MRD-positive disease. Clinical outcomes are generally well-modeled and instructionally valuable, though some scenarios could benefit from more detailed exploration of potential complications or alternative clinical trajectories.

Session-24

Session ID:
LLM

Session-24:
ChatGPT (GPT 4.o)

Session ID:
Session Transcript

Session-24:
Phase 1: Initial Presentation & Diagnostic Workup
Patient Case Introduction
Patient: M.A., a 67-year-old male
Chief Complaint: Fatigue, easy bruising, and recurrent nosebleeds for the past 3 weeks.
Past Medical History: Hypertension, well-controlled type 2 diabetes.
Medications: Amlodipine 5 mg daily, Metformin 1000 mg BID.
Social History: Retired teacher, former smoker (15 pack-years, quit 10 years ago), no alcohol use.

Initial Workup
Vital Signs: BP: 128/75 mmHg, HR: 90 bpm, Temp: 37.3C
Physical Exam: Pale, mild petechiae on lower extremities, no lymphadenopathy or hepatosplenomegaly.
Lab Findings:

CBC: WBC: 18.2 x10/L, Hgb: 7.9 g/dL, Platelets: 38 x10/L

Differential: Blasts 32%, ANC 0.8 x10/L

Peripheral Smear: Numerous circulating blasts with Auer rods seen

Coagulation: PT: 14.2 sec, aPTT: 31 sec, Fibrinogen: 250 mg/dL

LDH: Elevated

Bone Marrow Biopsy: Hypercellular marrow with 42% myeloblasts

Flow Cytometry: Blasts positive for CD34, CD117, HLA-DR, and MPO

Question 1: Diagnosis Confirmation
Based on the initial findings, what is the most likely diagnosis?

A) Chronic Myeloid Leukemia (CML) in blast crisis
B) Acute Myeloid Leukemia (AML)
C) Acute Lymphoblastic Leukemia (ALL)
D) Myelodysplastic Syndrome with Excess Blasts (MDS-EB2)

Type A, B, C, or D to select your answer.
Correct! The patient's presentation is consistent with Acute Myeloid Leukemia (AML) based on the presence of 20% myeloblasts in the bone marrow, Auer rods, and flow cytometry markers (CD34, CD117, MPO-positive).

Rationale:
CML in blast crisis (A): Typically presents with a history of chronic phase CML, often with BCR-ABL positivity. This patient has no

prior history of CML.

ALL (C): More common in younger patients and usually expresses CD10, CD19, or TdT, which were not reported here.

MDS-EB2 (D): Can present with increased blasts (10-19%), but this patient exceeds 20% blasts, meeting the threshold for AML.
Phase 1: Initial Presentation & Diagnostic Workup
Patient Case Introduction
Patient: M.A., a 67-year-old male
Chief Complaint: Fatigue, easy bruising, and recurrent nosebleeds for the past 3 weeks.
Past Medical History: Hypertension, well-controlled type 2 diabetes.
Medications: Amlodipine 5 mg daily, Metformin 1000 mg BID.
Social History: Retired teacher, former smoker (15 pack-years, quit 10 years ago), no alcohol use.

Initial Workup
Vital Signs: BP: 128/75 mmHg, HR: 90 bpm, Temp: 37.3C
Physical Exam: Pale, mild petechiae on lower extremities, no lymphadenopathy or hepatosplenomegaly.
Lab Findings:

CBC: WBC: 18.2 x10/L, Hgb: 7.9 g/dL, Platelets: 38 x10/L

Differential: Blasts 32%, ANC 0.8 x10/L

Peripheral Smear: Numerous circulating blasts with Auer rods seen

Coagulation: PT: 14.2 sec, aPTT: 31 sec, Fibrinogen: 250 mg/dL

LDH: Elevated

Bone Marrow Biopsy: Hypercellular marrow with 42% myeloblasts

Flow Cytometry: Blasts positive for CD34, CD117, HLA-DR, and MPO
Question 2: Risk Stratification
Cytogenetics and molecular studies return the following results:

Cytogenetics: Normal karyotype

Molecular Studies: FLT3-ITD positive (high allelic ratio), NPM1 mutation positive, IDH1/2 wild-type

According to the ELN 2022 risk stratification, how would you classify this patient?

A) Favorable risk
B) Intermediate risk
C) Adverse risk

Type A, B, or C to select your answer.
Correct! This patient falls into the Intermediate-risk category per ELN 2022.

Rationale:
NPM1 mutation alone is a favorable prognostic marker.

FLT3-ITD (high allelic ratio) is an adverse marker.

Normal karyotype does not contribute to adverse risk.

Key ELN 2022 Criteria for Intermediate Risk:

NPM1+ with FLT3-ITD (high allelic ratio) = Intermediate risk

If FLT3-ITD were low allelic ratio, this would be favorable risk.

If the patient had adverse cytogenetics (e.g., complex karyotype, -5, -7, TP53 mutation) this would be adverse risk.
Phase 1: Initial Presentation & Diagnostic Workup
Patient Case Introduction
Patient: M.A., a 67-year-old male
Chief Complaint: Fatigue, easy bruising, and recurrent nosebleeds for the past 3 weeks.
Past Medical History: Hypertension, well-controlled type 2 diabetes.
Medications: Amlodipine 5 mg daily, Metformin 1000 mg BID.
Social History: Retired teacher, former smoker (15 pack-years, quit 10 years ago), no alcohol use.

Initial Workup
Vital Signs: BP: 128/75 mmHg, HR: 90 bpm, Temp: 37.3C
Physical Exam: Pale, mild petechiae on lower extremities, no lymphadenopathy or hepatosplenomegaly.
Lab Findings:

CBC: WBC: 18.2 x10/L, Hgb: 7.9 g/dL, Platelets: 38 x10/L

Differential: Blasts 32%, ANC 0.8 x10/L

Peripheral Smear: Numerous circulating blasts with Auer rods seen

Coagulation: PT: 14.2 sec, aPTT: 31 sec, Fibrinogen: 250 mg/dL

LDH: Elevated

Bone Marrow Biopsy: Hypercellular marrow with 42% myeloblasts

Flow Cytometry: Blasts positive for CD34, CD117, HLA-DR, and MPO
Question 3: Induction Therapy Selection
The patient is 67 years old with good performance status (ECOG 1) and no major organ dysfunction.

Which of the following is the most appropriate induction regimen for this patient?

A) 7+3 (Cytarabine + Daunorubicin) + Midostaurin
B) CPX-351 (Liposomal Cytarabine + Daunorubicin)
C) Venetoclax + Azacitidine
D) Low-dose Cytarabine (LDAC) + Glasdegib

Type A, B, C, or D to select your answer.
Correct! The best induction regimen for this patient is 7+3 (Cytarabine + Daunorubicin) + Midostaurin.

Rationale:
The patient is 67 years old but has good performance status (ECOG 1) and no major comorbidities, making him eligible for intensive chemotherapy.

FLT3-ITD mutation: Requires FLT3 inhibitor (Midostaurin) in addition to 7+3 based on the RATIFY trial (Stone RM, NEJM 2017).

Why Not the Other Options?
CPX-351 (B): Preferred for therapy-related AML (t-AML) or AML with myelodysplasia-related changes, neither of which apply here.

Venetoclax + Azacitidine (C): Used for unfit or elderly patients who cannot tolerate intensive chemotherapy.

LDAC + Glasdegib (D): Inferior to Venetoclax-based regimens in older patients and not standard for FLT3-mutated AML.
Phase 1: Initial Presentation & Diagnostic Workup
Patient Case Introduction
Patient: M.A., a 67-year-old male
Chief Complaint: Fatigue, easy bruising, and recurrent nosebleeds for the past 3 weeks.
Past Medical History: Hypertension, well-controlled type 2 diabetes.
Medications: Amlodipine 5 mg daily, Metformin 1000 mg BID.
Social History: Retired teacher, former smoker (15 pack-years, quit 10 years ago), no alcohol use.

Initial Workup
Vital Signs: BP: 128/75 mmHg, HR: 90 bpm, Temp: 37.3C
Physical Exam: Pale, mild petechiae on lower extremities, no lymphadenopathy or hepatosplenomegaly.
Lab Findings:

CBC: WBC: 18.2 x10/L, Hgb: 7.9 g/dL, Platelets: 38 x10/L

Differential: Blasts 32%, ANC 0.8 x10/L

Peripheral Smear: Numerous circulating blasts with Auer rods seen

Coagulation: PT: 14.2 sec, aPTT: 31 sec, Fibrinogen: 250 mg/dL

LDH: Elevated

Bone Marrow Biopsy: Hypercellular marrow with 42% myeloblasts

Flow Cytometry: Blasts positive for CD34, CD117, HLA-DR, and MPO
Question 4: Supportive Care Considerations
Before starting induction, which prophylactic supportive care measure is most critical to initiate for this patient?

A) Antifungal prophylaxis with Posaconazole
B) Growth factor support with Filgrastim
C) Leukapheresis to reduce WBC count
D) Allopurinol + Rasburicase for tumor lysis syndrome (TLS) prophylaxis

Type A, B, C, or D to select your answer.
Correct! Allopurinol + Rasburicase is the most critical supportive care measure for tumor lysis syndrome (TLS) prophylaxis.

Rationale:
TLS Risk Factors:

High WBC count (18.2 x10/L)

Elevated LDH (marker of high cell turnover)

FLT3-ITD mutation (often associated with proliferative disease)

Rasburicase rapidly reduces uric acid levels and is preferred in patients at high risk of TLS.

Allopurinol prevents the formation of new uric acid but does not eliminate pre-existing uric acid.

Why Not the Other Options?
Posaconazole (A): Antifungal prophylaxis is important, but TLS prevention is more urgent before induction starts.

Filgrastim (B): G-CSF is NOT recommended in AML induction because it may increase blast proliferation.

Leukapheresis (C): Used for hyperleukocytosis (WBC >50-100 x10/L), but this patient's WBC is only 18.2 x10/L, so leukapheresis is not needed.
Phase 1: Initial Presentation & Diagnostic Workup
Patient Case Introduction
Patient: M.A., a 67-year-old male
Chief Complaint: Fatigue, easy bruising, and recurrent nosebleeds for the past 3 weeks.
Past Medical History: Hypertension, well-controlled type 2 diabetes.
Medications: Amlodipine 5 mg daily, Metformin 1000 mg BID.
Social History: Retired teacher, former smoker (15 pack-years, quit 10 years ago), no alcohol use.

Initial Workup
Vital Signs: BP: 128/75 mmHg, HR: 90 bpm, Temp: 37.3C
Physical Exam: Pale, mild petechiae on lower extremities, no lymphadenopathy or hepatosplenomegaly.
Lab Findings:

CBC: WBC: 18.2 x10/L, Hgb: 7.9 g/dL, Platelets: 38 x10/L

Differential: Blasts 32%, ANC 0.8 x10/L

Peripheral Smear: Numerous circulating blasts with Auer rods seen

Coagulation: PT: 14.2 sec, aPTT: 31 sec, Fibrinogen: 250 mg/dL

LDH: Elevated

Bone Marrow Biopsy: Hypercellular marrow with 42% myeloblasts

Flow Cytometry: Blasts positive for CD34, CD117, HLA-DR, and MPO
Question 5: Febrile Neutropenia Management
On Day 8 of induction, the patient develops fever (38.5C), hypotension (BP 90/60 mmHg), and ANC <0.1 x10/L. Blood cultures are drawn.

What is the most appropriate empiric antibiotic regimen?

A) Vancomycin + Cefepime
B) Cefepime monotherapy
C) Meropenem
D) Vancomycin + Piperacillin/Tazobactam

Type A, B, C, or D to select your answer.
Not the best choice.

Correct Answer: C) Meropenem
Rationale:
The patient has high-risk febrile neutropenia (FN) with sepsis (fever + hypotension + ANC <0.1).

Meropenem is preferred because:

Covers Pseudomonas and resistant Gram-negative bacteria (e.g., ESBL-producing Enterobacterales).

Has better activity in septic patients compared to cefepime.

Why Not the Other Options?
A) Vancomycin + Cefepime:

Cefepime is a reasonable choice, but Vancomycin is NOT needed upfront unless:

Concern for MRSA (e.g., catheter infection, pneumonia, SSTI, mucositis).

Persistent hypotension or septic shock.

B) Cefepime Monotherapy:

Cefepime is an acceptable first-line agent for FN but not ideal for sepsis.

No anaerobic or ESBL coverage, which can be problematic in GI translocation.

D) Vancomycin + Piperacillin/Tazobactam:

Pip/Tazo is good for broad coverage but has higher nephrotoxicity in AML patients on other nephrotoxic drugs.

Vancomycin is not needed upfront unless MRSA is suspected.
Phase 1: Initial Presentation & Diagnostic Workup
Patient Case Introduction
Patient: M.A., a 67-year-old male
Chief Complaint: Fatigue, easy bruising, and recurrent nosebleeds for the past 3 weeks.
Past Medical History: Hypertension, well-controlled type 2 diabetes.
Medications: Amlodipine 5 mg daily, Metformin 1000 mg BID.
Social History: Retired teacher, former smoker (15 pack-years, quit 10 years ago), no alcohol use.

Initial Workup
Vital Signs: BP: 128/75 mmHg, HR: 90 bpm, Temp: 37.3C
Physical Exam: Pale, mild petechiae on lower extremities, no lymphadenopathy or hepatosplenomegaly.
Lab Findings:

CBC: WBC: 18.2 x10/L, Hgb: 7.9 g/dL, Platelets: 38 x10/L

Differential: Blasts 32%, ANC 0.8 x10/L

Peripheral Smear: Numerous circulating blasts with Auer rods seen

Coagulation: PT: 14.2 sec, aPTT: 31 sec, Fibrinogen: 250 mg/dL

LDH: Elevated

Bone Marrow Biopsy: Hypercellular marrow with 42% myeloblasts

Flow Cytometry: Blasts positive for CD34, CD117, HLA-DR, and MPO
Question 6: MRD Monitoring
The patient achieves morphologic remission (<5% blasts) after induction.

What is the next step in response assessment?

A) Bone marrow biopsy with flow cytometry for MRD
B) Peripheral blood PCR for NPM1 mutation
C) Repeat bone marrow biopsy at day 60
D) No further testing needed until relapse

Type A, B, C, or D to select your answer.
Correct! The next step is bone marrow biopsy with flow cytometry for MRD assessment.

Rationale:
Morphologic remission (<5% blasts) is not enough to determine true remission.

MRD (Measurable Residual Disease) assessment is critical for risk stratification and guiding post-remission therapy.

Flow cytometry (or PCR for specific mutations) detects residual leukemia at a sensitivity of 10 to 10, predicting relapse risk.

Why Not the Other Options?
B) Peripheral blood PCR for NPM1 mutation:

Useful if NPM1 was present at diagnosis, but bone marrow is more sensitive for MRD detection.

C) Repeat bone marrow biopsy at day 60:

MRD should be checked immediately after induction (day 28-35), not delayed.

D) No further testing until relapse:

IncorrectMRD guides post-remission therapy (e.g., transplant vs. consolidation chemo).
Phase 1: Initial Presentation & Diagnostic Workup
Patient Case Introduction
Patient: M.A., a 67-year-old male
Chief Complaint: Fatigue, easy bruising, and recurrent nosebleeds for the past 3 weeks.
Past Medical History: Hypertension, well-controlled type 2 diabetes.
Medications: Amlodipine 5 mg daily, Metformin 1000 mg BID.
Social History: Retired teacher, former smoker (15 pack-years, quit 10 years ago), no alcohol use.

Initial Workup
Vital Signs: BP: 128/75 mmHg, HR: 90 bpm, Temp: 37.3C
Physical Exam: Pale, mild petechiae on lower extremities, no lymphadenopathy or hepatosplenomegaly.
Lab Findings:

CBC: WBC: 18.2 x10/L, Hgb: 7.9 g/dL, Platelets: 38 x10/L

Differential: Blasts 32%, ANC 0.8 x10/L

Peripheral Smear: Numerous circulating blasts with Auer rods seen

Coagulation: PT: 14.2 sec, aPTT: 31 sec, Fibrinogen: 250 mg/dL

LDH: Elevated

Bone Marrow Biopsy: Hypercellular marrow with 42% myeloblasts

Flow Cytometry: Blasts positive for CD34, CD117, HLA-DR, and MPO
Question 7: Post-Remission Therapy

MRD testing shows positive FLT3-ITD (high allelic ratio) with low-level MRD. The patient remains intermediate-risk per ELN 2022.

What is the best post-remission strategy?

A) High-dose Cytarabine (HiDAC) consolidation + Midostaurin
B) Allogeneic Stem Cell Transplant (allo-HSCT)
C) Venetoclax + Azacitidine maintenance
D) Observation only

Type A, B, C, or D to select your answer.
Not the best choice.

Correct Answer: B) Allogeneic Stem Cell Transplant (allo-HSCT)
Rationale:
The patient has FLT3-ITD (high allelic ratio) and persistent MRD positivity, which significantly increases relapse risk.

Allo-HSCT is the preferred post-remission strategy for FLT3-mutated AML with MRD, per ELN 2022 and NCCN guidelines.

HiDAC + Midostaurin (Option A) is reasonable for MRD-negative, intermediate-risk patients but is not sufficient for MRD-positive disease.

Why Not the Other Options?
A) HiDAC + Midostaurin:

Acceptable if MRD-negative, but MRD-positive patients benefit more from transplant.

C) Venetoclax + Azacitidine Maintenance:

Venetoclax is not standard maintenance for FLT3-mutated AML; Gilteritinib (FLT3 inhibitor) is preferred after allo-HSCT.

D) Observation Only:

Unacceptable due to high relapse risk with persistent MRD.
Phase 1: Initial Presentation & Diagnostic Workup
Patient Case Introduction
Patient: M.A., a 67-year-old male
Chief Complaint: Fatigue, easy bruising, and recurrent nosebleeds for the past 3 weeks.
Past Medical History: Hypertension, well-controlled type 2 diabetes.
Medications: Amlodipine 5 mg daily, Metformin 1000 mg BID.
Social History: Retired teacher, former smoker (15 pack-years, quit 10 years ago), no alcohol use.

Initial Workup
Vital Signs: BP: 128/75 mmHg, HR: 90 bpm, Temp: 37.3C
Physical Exam: Pale, mild petechiae on lower extremities, no lymphadenopathy or hepatosplenomegaly.
Lab Findings:

CBC: WBC: 18.2 x10/L, Hgb: 7.9 g/dL, Platelets: 38 x10/L

Differential: Blasts 32%, ANC 0.8 x10/L

Peripheral Smear: Numerous circulating blasts with Auer rods seen

Coagulation: PT: 14.2 sec, aPTT: 31 sec, Fibrinogen: 250 mg/dL

LDH: Elevated

Bone Marrow Biopsy: Hypercellular marrow with 42% myeloblasts

Flow Cytometry: Blasts positive for CD34, CD117, HLA-DR, and MPO
Question 8: Maintenance Therapy Post-Transplant
The patient successfully undergoes allo-HSCT. Based on current guidelines, which maintenance therapy is recommended post-transplant?

A) Midostaurin
B) Gilteritinib
C) Azacitidine + Venetoclax
D) No maintenance therapy

Type A, B, C, or D to select your answer.
Correct! Gilteritinib is the preferred maintenance therapy post-transplant for FLT3-mutated AML.

Rationale:
Gilteritinib (FLT3 inhibitor) improves relapse-free survival (RFS) and overall survival (OS) after allo-HSCT in FLT3-mutated AML.

Midostaurin (A) is NOT the best choice for maintenance, as it has not shown a survival benefit post-transplant.

The SORMAIN trial and SCT-MATCH study support using Gilteritinib post-HSCT for FLT3-mutated AML.

Why Not the Other Options?
A) Midostaurin:

Used during induction/consolidation, but not preferred for maintenance after transplant.

C) Azacitidine + Venetoclax:

Used for relapsed/refractory AML but not standard maintenance post-HSCT.

D) No maintenance therapy:

Unacceptable due to high relapse risk in FLT3-ITD patients without ongoing suppression.
Phase 1: Initial Presentation & Diagnostic Workup
Patient Case Introduction
Patient: M.A., a 67-year-old male
Chief Complaint: Fatigue, easy bruising, and recurrent nosebleeds for the past 3 weeks.
Past Medical History: Hypertension, well-controlled type 2 diabetes.
Medications: Amlodipine 5 mg daily, Metformin 1000 mg BID.
Social History: Retired teacher, former smoker (15 pack-years, quit 10 years ago), no alcohol use.

Initial Workup
Vital Signs: BP: 128/75 mmHg, HR: 90 bpm, Temp: 37.3C
Physical Exam: Pale, mild petechiae on lower extremities, no lymphadenopathy or hepatosplenomegaly.
Lab Findings:

CBC: WBC: 18.2 x10/L, Hgb: 7.9 g/dL, Platelets: 38 x10/L

Differential: Blasts 32%, ANC 0.8 x10/L

Peripheral Smear: Numerous circulating blasts with Auer rods seen

Coagulation: PT: 14.2 sec, aPTT: 31 sec, Fibrinogen: 250 mg/dL

LDH: Elevated

Bone Marrow Biopsy: Hypercellular marrow with 42% myeloblasts

Flow Cytometry: Blasts positive for CD34, CD117, HLA-DR, and MPO
Final Question: Relapse Management
Unfortunately, 9 months post-HSCT, the patient experiences molecular relapse (MRD reappearance, no overt blasts).

What is the next best step?

A) Start Gilteritinib monotherapy
B) Donor lymphocyte infusion (DLI) alone
C) Reinduction with FLAG-IDA + Gilteritinib
D) Azacitidine + Venetoclax

Type A, B, C, or D to select your answer.
Not the best choice.

Correct Answer: A) Start Gilteritinib Monotherapy
Rationale:
The patient has molecular relapse (MRD reappearance, but no overt blasts).

Gilteritinib is FDA-approved for relapsed/refractory FLT3-mutated AML and is effective in early relapse.

Starting Gilteritinib alone can delay or prevent full relapse, avoiding the toxicity of reinduction chemotherapy.

The MORPHO trial suggests that FLT3 inhibitors post-HSCT improve outcomes in MRD-positive patients.

Why Not the Other Options?
B) Donor Lymphocyte Infusion (DLI) Alone:

DLI can boost the graft-versus-leukemia effect, but without a FLT3 inhibitor, its insufficient to control FLT3-mutated disease.

C) FLAG-IDA + Gilteritinib (Your choice):

Too aggressive for molecular relapse. FLAG-IDA is reserved for overt hematologic relapse (high blast count).

Earlier intervention with Gilteritinib alone is preferred to delay progression while minimizing toxicity.

D) Azacitidine + Venetoclax:

Not first-line for FLT3-mutated AML relapse. Gilteritinib has stronger data in FLT3+ disease.

Session ID:
Assessment Domain

Session-24:
Rationale

Session ID:

Instructional Design and Pedagogical

Session-24:

The session's instructional design is of excellent quality. It demonstrates expert scaffolding, progressing logically from initial diagnosis and risk stratification through induction, management of acute complications (febrile neutropenia), response assessment, post-remission therapy, and finally, management of molecular relapse. The embedded feedback is a significant strength; rationales are clear, evidence-based, and explain not only the correct answer but also why the distractors are incorrect in the given context. The framing of each question is exceptionally clear. The only minor weakness is that learning objectives are not explicitly stated at the outset, though they are strongly implied by the session's logical structure.

Session ID:

Clinical Authenticity and Content Fidelity

Session-24:

The session maintains excellent clinical authenticity throughout. The patient presentation is highly realistic and indistinguishable from an authentic case, with appropriate demographics, laboratory values, and disease progression. All therapeutic decisions are perfectly aligned with current evidence-based guidelines, including correct ELN 2022 risk stratification, appropriate FLT3 inhibitor selection (Midostaurin for induction, Gilteritinib for maintenance and relapse), and guideline-directed supportive care measures. Pharmacotherapeutic recommendations are optimal and well-justified at each decision point. The case maintains strict domain specificity with no inappropriate application of therapies from other disease contexts. THERE ARE 2 MAJOR ERRORS: CONCOMITANT USE OF ALLOPURINOL WITH RASBURICASE, AND AVOID VANCOMYCIN RECOMMENDATION IN THE PRESENCE OF SEPSIS.

Session ID:

Modeled Clinical Reasoning

Session-24:

The session exemplifies expert-level clinical reasoning throughout the AML treatment continuum. Each clinical phase presents well-defined problems with appropriate data for decision-making. Multiple-choice options provide excellent diagnostic and therapeutic breadth, including clinically relevant distractors that effectively test nuanced understanding. The case demonstrates sound prioritization and risk-adjusted decision-making, such as appropriate TLS prophylaxis timing, MRD-guided post-remission therapy selection, and escalating intervention strategies for molecular relapse. Clinical outcomes are realistically modeled and highly instructive, reinforcing key learning points while maintaining clinical authenticity.

Session-25

Session ID:
LLM

Session-25:
ChatGPT (GPT 4.o)

Session ID:
Session Transcript

Session-25:
Case Introduction:

A 65-year-old male presents to the emergency department with progressive fatigue, easy bruising, and recurrent nosebleeds over the past three weeks. He also reports night sweats and unintentional weight loss (5 kg over two months).

Past Medical History:
Hypertension (controlled on amlodipine)
Type 2 diabetes mellitus (controlled on metformin)

Physical Examination:
Pale appearance, scattered petechiae on arms and legs
No significant lymphadenopathy or hepatosplenomegaly
Vital Signs: HR 102 bpm, BP 130/78 mmHg, Temp 37.6C

Laboratory Results:
WBC: 18.5 10/L () with circulating blasts
Hb: 7.8 g/dL ()
Platelets: 28 10/L ()
LDH: Elevated
Peripheral blood smear: Numerous myeloblasts with Auer rods

Question 1: What is the most appropriate next step in confirming the diagnosis?

A) Serum B12 and folate levels
B) Bone marrow aspiration and biopsy with flow cytometry and cytogenetics
C) Coagulation profile and D-dimer
D) CT scan of the chest, abdomen, and pelvis

Type your answer (A, B, C, or D) and explain your reasoning.
My answer :
Answer is (B)
Reasoning:
1. Clinical Presentation:The patient has symptoms (fatigue, easy bruising, recurrent nosebleeds, night sweats, weight loss) and signs (pallor, petechiae, thrombocytopenia, anemia) highly suggestive of a hematologic malignancy, particularly acute leukemia.
2. Laboratory Findings:
- Elevated WBC with circulating blasts and Auer rods (pathognomonic for acute myeloid leukemia, AML).
- Anemia, thrombocytopenia, and elevated LDH further support a proliferative disorder like leukemia.
3. Diagnostic Confirmation:
- Bone marrow biopsy is the gold standard for diagnosing acute leukemia. It provides essential information about blast percentage, morphology, and marrow cellularity.

AI Evaluation:

Correct!

Explanation:

Bone marrow aspiration and biopsy with flow cytometry and cytogenetics (Option B) is the most appropriate next step to confirm AML.

Key Points:
1. Peripheral Blood Findings
 Circulating blasts with Auer rods are highly suggestive of AML.
 However, peripheral blood alone is not sufficient for diagnosis.
2. Why Bone Marrow Biopsy?
 Confirms blast percentage (20% blasts in bone marrow is diagnostic of AML).
 Allows flow cytometry to determine immunophenotype.
 Enables cytogenetic and molecular analysis (e.g., FLT3, NPM1, IDH mutations) for risk stratification.

Why Not the Other Options?

A) Serum B12 and folate levels Deficiencies can cause macrocytic anemia but do not explain circulating blasts.

C) Coagulation profile and D-dimer Useful for DIC assessment (e.g., in APL), but AML diagnosis requires bone marrow biopsy.

D) CT scan of the chest, abdomen, and pelvis Not required for AML diagnosis; useful only if extramedullary disease is suspected.

Phase 1 Continues

Bone marrow biopsy results confirm AML with FLT3-ITD mutation and NPM1 mutation. Cytogenetics show normal karyotype.

Question 2: Based on the 2022 ELN risk stratification, how would you categorize this patients AML?

A) Favorable risk
B) Intermediate risk
C) Adverse risk
D) Not enough information

Type your answer (A, B, C, or D) and explain your reasoning.
my answer:
Answer is (B)
Reasoning:
1. FLT3-ITD mutation:
   - FLT3-ITD is generally considered an adverse-risk marker, but its prognostic impact depends on the allelic ratio (AR).
   - If the FLT3-ITD AR is low (<0.5), and the patient has an NPM1 mutation, the risk is intermediate.
   - If the FLT3-ITD AR is high (0.5), the risk is adverse, regardless of NPM1 status.
2. NPM1 mutation:
   - NPM1 mutations are favorable-risk, but this is overridden by FLT3-ITD unless the AR is low.
3. Cytogenetics (normal karyotype):
   - A normal karyotype alone does not define risk but influences molecular risk stratification.

Ai evaluation:
Correct!

Explanation:

According to the 2022 ELN risk stratification, this patient falls into the intermediate-risk category due to the presence of both FLT3-ITD and NPM1 mutations.

Risk Classification Based on Molecular Markers:
1. NPM1 mutation alone Favorable risk
2. FLT3-ITD mutation (High AR 0.5) Adverse risk
3. FLT3-ITD mutation (Low AR <0.5) + NPM1 mutation Intermediate risk (which applies to this patient)
4. Normal karyotype Does not independently define risk, but molecular findings take precedence.

Why Not the Other Options?

A) Favorable risk Only if NPM1 is mutated without FLT3-ITD or if FLT3-ITD has a very low AR.

C) Adverse risk Would apply if FLT3-ITD AR was high (0.5).

D) Not enough information Incorrect because we have enough molecular and cytogenetic data to stratify risk.

Phase 1 Continues

The patient is now diagnosed with AML, ELN intermediate-risk (FLT3-ITD low AR, NPM1+), normal karyotype. He is deemed fit for intensive therapy based on his performance status.

Question 3: What is the most appropriate induction therapy for this patient?

A) 7+3 (Cytarabine + Daunorubicin) + Midostaurin
B) CPX-351 (Liposomal Cytarabine + Daunorubicin)
C) Venetoclax + Azacitidine
D) HiDAC (High-dose Cytarabine) alone

Type your answer (A, B, C, or D) and explain your reasoning.
my answer:
The answer is (A)
The reason isFLT3-ITD mutation (low AR) + NPM1 mutation Midostaurin (FLT3 inhibitor) is added based on the RATIFY trial which showed survival benefit when combined with chemotherapy.

Ai evaluation:
Correct!

Explanation:

The best choice is 7+3 (Cytarabine + Daunorubicin) + Midostaurin (Option A) based on the RATIFY trial (Stone et al., NEJM 2017).

Key Points:
1. FLT3-ITD Mutation (low AR) + NPM1 Mutation FLT3 Inhibitor Required
   Midostaurin (a FLT3 inhibitor) is approved for FLT3-mutated AML when added to 7+3 induction and HiDAC-based consolidation.
   The RATIFY trial showed a significant overall survival benefit when midostaurin was added to standard induction.
2. Why Not the Other Options?

B) CPX-351 (Liposomal Cytarabine + Daunorubicin)
Used only for secondary AML (t-AML or AML with MDS-related changes), not de novo AML with FLT3-ITD/NPM1 mutations.

C) Venetoclax + Azacitidine
Preferred for older/unfit patients or those with adverse-risk AML.
This patient is fit for intensive therapy and has intermediate-risk AML, so 7+3 is superior.

D) HiDAC (High-dose Cytarabine) alone
Used in consolidation, not as induction.
Would not achieve adequate remission as monotherapy in newly diagnosed AML.
Phase 1 Completed!

The patient begins 7+3 + Midostaurin induction and develops fever (38.5C) on day 10 post-chemotherapy with an absolute neutrophil count (ANC) of 0.2 10/L.

Question 4: What is the most appropriate initial management?

A) Vancomycin + Cefepime
B) Meropenem + Amikacin
C) Caspofungin monotherapy
D) Observation for 24 hours

Type your answer (A, B, C, or D) and explain your reasoning.
my answer:
Answer is (A)
Because the e mpirical Antibiotic Coverage;
First line therapy requires broad spectrum antipseudomonal coverage (cefepime, meropenem, piperacillin-tazobactam
Vancomycin is added if: Hemodynamic instability sepsis, Suspected catheter-related infection,Skin/soft tissue involvement, or Known MRSA colonization.
- In high-risk neutropenic fever dual coverage (cefepime + vancomycin) is commonly used upfront (per IDSA/NCCN guidelines).

Ai evaluation:
Correct!

Explanation:

The best choice is Vancomycin + Cefepime (Option A) based on IDSA & NCCN guidelines for febrile neutropenia in high-risk AML patients.

Key Principles of Febrile Neutropenia Management:
1. High-Risk Neutropenic Fever (ANC < 0.5 10/L for >7 days + AML) Immediate Broad-Spectrum IV Antibiotics
First-line options include:
Cefepime
Piperacillin-Tazobactam
Carbapenems (Meropenem/Imipenem) in select cases
Vancomycin is added for:
Hemodynamic instability (sepsis, shock)
Skin/soft tissue infection
Suspected catheter-related infection
Known MRSA colonization
2. Why Not the Other Options?
B) Meropenem + Amikacin Too broad initially
Meropenem is reserved for cases with suspected ESBL or carbapenem-resistant infections.
Amikacin is not needed upfront unless suspicion of multidrug-resistant Pseudomonas.

C) Caspofungin monotherapy Not first-line
Empirical antifungal therapy is added only if fever persists >4-7 days despite broad-spectrum antibiotics.
Early fungal coverage is considered in prolonged neutropenia, but not the first step here.

D) Observation for 24 hours Unsafe in high-risk patients
Febrile neutropenia in AML requires immediate IV antibiotics, delaying treatment increases mortality risk.

Phase 2 Continues

The patient completes induction therapy, and a day 14 bone marrow biopsy shows hypocellular marrow with 5% residual blasts.

Question 5: What is the most appropriate next step?

A) Continue supportive care and repeat marrow biopsy on day 28
B) Start re-induction with 5+2 (Cytarabine + Daunorubicin)
C) Start re-induction with FLAG-IDA (Fludarabine, Cytarabine, G-CSF, Idarubicin)
D) Start G-CSF to accelerate marrow recovery

Type your answer (A, B, C, or D) and explain your reasoning.
my answer:
Answer is (C)
Because bone Marrow Findings (Day 14):
- 5% residual blasts indicates persistent disease (not in complete remission, CR).
- ELN guidelines recommend early re-induction if blasts 5% at day 14-21 (especially in intermediate/adverse-risk AML).

Ai evaluation:
Not the best choice.

Correct Answer: (A) Continue supportive care and repeat marrow biopsy on day 28

Explanation:
Day 14 bone marrow showing 5% residual blasts does NOT immediately indicate treatment failure.
Hypocellular marrow with 5% blasts is often a sign of ongoing response to chemotherapy rather than true persistent disease.
ELN & NCCN guidelines recommend waiting until day 28 to confirm remission status before deciding on re-induction therapy.

Why Not the Other Options?

B) Start re-induction with 5+2
5+2 is used for patients with persistent leukemia (10-15% blasts at day 14).
Here, the blasts are 5%, so immediate re-induction is premature.

C) Start re-induction with FLAG-IDA
FLAG-IDA is a salvage regimen for primary induction failure (high blast count at day 14 or refractory disease at day 28).
This patient does NOT yet meet criteria for refractory AML.

D) Start G-CSF to accelerate marrow recovery
G-CSF should not be used during induction phase in AML because it can stimulate residual leukemia cells and increase relapse risk.
G-CSF is only used in post-remission consolidation or severe infections.
Next Step:

Monitor for count recovery & repeat bone marrow biopsy at day 28.
If day 28 biopsy confirms CR (blasts <5%, ANC >1.0, PLT >100), proceed to consolidation.
If persistent blasts remain 5%, then re-induction (e.g., FLAG-IDA) is needed.

Phase 2 Continues

Day 28 bone marrow biopsy shows:
Blasts <5%, ANC 1.5 10/L, PLT 120 10/L Complete remission (CR) achieved!

Now, what is the most appropriate post-remission therapy?

A) High-dose Cytarabine (HiDAC) consolidation
B) Allogeneic stem cell transplant (Allo-HSCT) immediately
C) Azacitidine maintenance
D) Observation without further therapy

Type your answer (A, B, C, or D) and explain your reasoning.
my answer:
Okay, I understood the explanation of the fifth question.
Answer the sixth question is (B)

Ai evaluation:
Not the best choice.

Correct Answer: (A) High-dose Cytarabine (HiDAC) consolidation

Explanation:

The patient achieved complete remission (CR) with induction therapy.
The choice of post-remission therapy depends on ELN 2022 risk stratification.

1 What is the patients risk category?
FLT3-ITD (low AR) + NPM1 mutation = Intermediate-risk AML.

2 Standard post-remission therapy for intermediate-risk AML?
HiDAC consolidation (Preferred if no adverse-risk features and no matched donor available)
Allo-HSCT (Considered if MRD-positive or additional high-risk markers)

3 Why is HiDAC (A) preferred here?
For intermediate-risk AML, ELN/NCCN guidelines recommend HiDAC consolidation in younger, fit patients with no adverse-risk mutations.
Allo-HSCT is reserved for patients with measurable residual disease (MRD) positivity or additional high-risk features.

Why Not the Other Options?

(B) Allogeneic Stem Cell Transplant (Allo-HSCT) immediately
Allo-HSCT is typically used in ELN adverse-risk AML or if MRD is positive after remission.
This patient has an intermediate-risk profile, and HiDAC consolidation is appropriate first.

(C) Azacitidine maintenance
Azacitidine maintenance is mainly for patients ineligible for HiDAC or after allo-HSCT (e.g., post-transplant maintenance).
Not standard for post-remission therapy in a fit, younger patient.

(D) Observation without further therapy
Not an option in AML. Without consolidation, the relapse rate is extremely high (>80%).

Next Step:

Patient undergoes HiDAC consolidation (3-4 cycles of high-dose Cytarabine).

MRD monitoring is performed after each cycle to assess relapse risk.

Phase 3 Begins: Long-Term Management & MRD Monitoring

After completing consolidation, the patient undergoes MRD testing, which shows persistent low-level MRD positivity.

Question 7: What is the next best step?

A) Proceed to allogeneic stem cell transplant (Allo-HSCT)
B) Give additional HiDAC cycles
C) Start maintenance therapy with oral FLT3 inhibitor (e.g., Gilteritinib)
D) Observe with serial MRD monitoring

Type your answer (A, B, C, or D) and explain your reasoning.
my answer:
The answer is (A) and the reason is
1.MRD Positivity Post-Consolidation indicates residual disease and high relapse risk, especially in FLT3-ITD+ AML.
2. FLT3-ITD Mutation (Even Low AR) is an independent poor prognostic factor, making Allo-HSCT the only potentially curative option in this setting.
3. HiDAC Alone is Insufficient (Option B) Additional cycles are unlikely to clear MRD, and delaying transplant increases relapse risk.
4. FLT3 Inhibitor Maintenance (Option C) (e.g., gilteritinib) may help but is not curative best used posttransplant or in relapsed/refractory disease.
5. Observation (Option D) is unacceptable with MRD+ disease due to inevitable relapse.

Ai evaluation:
Correct! The best answer is (A) Allogeneic Stem Cell Transplant (Allo-HSCT).

Key Takeaways:

1 MRD positivity post-consolidation = High risk of relapse
MRD persistence after HiDAC consolidation predicts poor long-term outcomes.
ELN/NCCN guidelines strongly recommend Allo-HSCT for MRD+ patients to improve overall survival.

2 FLT3-ITD mutation, even with a low allelic ratio, adds relapse risk.
Although low AR FLT3-ITD mutations are considered intermediate risk, persistent MRD overrides this and mandates transplant.

3 HiDAC alone (Option B) is inadequate
If HiDAC failed to clear MRD after 3-4 cycles, additional cycles are unlikely to help.

4 FLT3 inhibitors (Option C) are adjuncts, not standalone therapy
Gilteritinib can be used as post-transplant maintenance to reduce relapse risk but is not a substitute for Allo-HSCT.

5 Observation (Option D) is not acceptable in MRD+ AML
Without intervention, MRD+ patients have a nearly inevitable relapse.

Next Step:

Proceed with Allo-HSCT if a matched donor is available.
Consider post-transplant maintenance with FLT3 inhibitor (e.g., gilteritinib) per the MORPHO trial findings.

Phase 3 Continues: Post-Transplant Monitoring

The patient undergoes Allo-HSCT and achieves full donor chimerism.
MRD testing at 3 months post-transplant shows FLT3-ITD MRD positivity.

Question 8: What is the best next step?

A) Start FLT3 inhibitor maintenance therapy (e.g., Gilteritinib)
B) Initiate a second round of chemotherapy
C) Consider a second Allo-HSCT
D) Observe with serial MRD monitoring without intervention

Type your answer (A, B, C, or D) and justify your choice!
my answer:
The best answer is (A) the reason is
1. FLT3-ITD MRDPositivity Post-Transplant is a high-risk feature for relapse (70-80% relapse risk without intervention).
2. FLT3 Inhibitors (Gilteritinib/Sorafenib)are now standard for MRD+ FLT3-ITD AML post-HSCT based on:
   - MORPHO Trial (Lancet Oncol 2023): Gilteritinib maintenance significantly improved relapse-free survival (RFS) in post-transplant FLT3-ITD+ AML.
   - SORMAIN Trial (Blood 2020): Sorafenib maintenance reduced relapse risk by ~50%.

Ai evaluation:
Correct! The best answer is (A) FLT3 inhibitor maintenance therapy (e.g., Gilteritinib).

Key Takeaways:

1 MRD positivity post-transplant = High relapse risk (~70-80%)
Persistent MRD after Allo-HSCT predicts early hematologic relapse.
ELN & NCCN guidelines now strongly recommend targeted maintenance therapy in this setting.

2 FLT3 inhibitors (Gilteritinib/Sorafenib) reduce relapse & improve survival
Key Trials:
MORPHO Trial (Lancet Oncol 2023): Gilteritinib significantly improved relapse-free survival (RFS) in post-HSCT FLT3-ITD+ AML.
SORMAIN Trial (Blood 2020): Sorafenib maintenance halved relapse risk in FLT3-ITD+ AML post-transplant.

3 Why NOT other options?
(B) Second chemotherapy Ineffective post-transplant without full relapse. Targeted maintenance is superior.
(C) Second Allo-HSCT Only considered in full relapse, not just MRD positivity.
(D) Observation only Unacceptable. MRD+ patients have an almost certain relapse without intervention.

Next Step:

Start FLT3 inhibitor maintenance therapy (Gilteritinib 120 mg/day for 24 months if tolerated).
Monitor MRD every 3 months to assess treatment response.

Case Progression: 6 Months Post-HSCT with Gilteritinib Maintenance

The patient is tolerating Gilteritinib well.

MRD testing at 6 months is negative.
However, the patient presents with new-onset grade 2 chronic GVHD (skin & liver involvement).

Question 9: What is the most appropriate next step?

A) Continue Gilteritinib and start systemic corticosteroids (e.g., Prednisone 1 mg/kg/day)
B) Hold Gilteritinib and treat GVHD with corticosteroids
C) Discontinue Gilteritinib permanently
D) Reduce Gilteritinib dose and monitor GVHD closely

Type your answer (A, B, C, or D) and justify your choice!
my answer:
The answer is (B)
The reason is
1. GVHD Takes Precedence Over Maintenance Therapy:
Grade 2 chronic GVHD (skin + liver) requires systemic immunosuppression (corticosteroids are first-line).
Gilteritinib should be temporarily held to:
Avoid overlapping toxicities (e.g., hepatotoxicity).
Allow clear assessment of GVHD response to steroids.
Prevent potential exacerbation of GVHD (FLT3 inhibitors may modulate immune function).
2. Post-GVHD Management:
Reintroduce Gilteritinibonce GVHD is stable/improving on tapering steroids.
If GVHD flares upon reintroduction, consider alternative FLT3 inhibitors (e.g., sorafenib) or discuss risks/benefits with transplant team.

Ai evaluation:
Correct! The best answer is (B) Hold Gilteritinib and treat GVHD with corticosteroids.

Key Takeaways:

1 GVHD Management Takes Priority Over FLT3 Inhibitor Maintenance
Grade 2 chronic GVHD (skin + liver) warrants systemic corticosteroids (first-line: prednisone 1 mg/kg/day).
FLT3 inhibitors like Gilteritinib can exacerbate GVHD or cause hepatotoxicity, complicating GVHD assessment.

2 Why Temporarily Hold Gilteritinib?
Allows clear evaluation of GVHD response to steroids.
Minimizes overlapping toxicities (especially hepatotoxicity).
Prevents worsening immune dysregulation (FLT3 inhibitors may modulate T-cell function).

3 What Happens Next?
Once GVHD stabilizes (Grade 1 & on tapering steroids) Reintroduce Gilteritinib cautiously.
If GVHD worsens upon reintroduction Consider alternative FLT3 inhibitors (e.g., Sorafenib) or discuss risks vs. benefits with the transplant team.
Case Progression: 3 Months Later

GVHD has improved with corticosteroids, and the patient is tapering prednisone.
Gilteritinib was cautiously restarted at a reduced dose (80 mg/day).
MRD testing remains negative.
However, the patient now presents with new onset cytopenias (ANC 0.6, Platelets 48k).

Question 10: What is the most appropriate next step?

A) Continue Gilteritinib and monitor closely
B) Temporarily hold Gilteritinib and assess for causes of cytopenias
C) Discontinue Gilteritinib permanently due to toxicity
D) Increase Gilteritinib dose back to 120 mg/day

Type your answer (A, B, C, or D) and justify your choice!
my answer:
The most appropriate answer is (B)
The reason is
1. New Cytopenias (ANC 0.6, Plt 48k) Require Evaluation:
- Differential Diagnosis:
  - Gilteritinib toxicity (myelosuppression is a known side effect, especially at higher doses).
  - Late graft failure (check chimerism).
  - GVHD-related marrow suppression.
  - Infectious causes (e.g., parvovirus B19, CMV).
- Holding Gilteritinib allows:
  - Clear assessment of whether cytopenias are drug-related.
  - Time for marrow recovery if due to toxicity.

Ai evaluation:
Correct! The best answer is (B) Temporarily hold Gilteritinib and assess for causes of cytopenias.

Key Takeaways:

1 Gilteritinib-Induced Myelosuppression is Common
Gilteritinib can cause dose-dependent cytopenias, especially at 120 mg/day.
Holding the drug allows for clear assessment of whether the cytopenias are drug-related.

2 Differential Diagnosis of Cytopenias in Post-Transplant AML
Gilteritinib toxicity (most likely in this case).
Late-onset graft failure Check chimerism to assess donor cell engraftment.
GVHD-related marrow suppression Evaluate for concurrent GVHD symptoms.
Infectious causes Consider CMV, EBV, parvovirus B19, and fungal infections (do PCR testing).
MRD reactivation Rule out relapse with bone marrow biopsy + flow cytometry.

3 Why Not Permanently Discontinue Gilteritinib?
FLT3-ITD+ AML has a high relapse risk Maintenance therapy is essential.
If toxicity resolves, Gilteritinib can be resumed at a lower dose (e.g., 80 mg/day).
Case Progression: 2 Weeks Later

Gilteritinib was held.
Bone marrow biopsy shows persistent full donor chimerism (no relapse), but mild hypocellularity.
Viral testing negative.
ANC improved to 1.2, Platelets to 80k.

Question 11: What is the most appropriate next step?

A) Restart Gilteritinib at full dose (120 mg/day)
B) Restart Gilteritinib at a reduced dose (80 mg/day)
C) Permanently discontinue Gilteritinib due to prior toxicity
D) Switch to Sorafenib as an alternative FLT3 inhibitor

Type your answer (A, B, C, or D) and justify your choice!
my answer:
The most appropriate answer is (B)
The reason is :
1. Evidence Supports Dose Reduction for Toxicity:
- The ADMIRAL trial showed that Gilteritinib dose reductions (to 80 mg/day) maintained efficacy while improving tolerability in patients with cytopenias.
- Since cytopenias improved after holding the drug, reducing the dose (80 mg/day) balances relapse prevention and safety.

Ai evaluation:
Correct! The best answer is (B) Restart Gilteritinib at a reduced dose (80 mg/day).

Key Takeaways:

1 Gilteritinib Dose Reduction is Effective and Safe
The ADMIRAL trial confirmed that dose reduction maintains efficacy while improving tolerability.
80 mg/day is a common modified dose for patients experiencing myelosuppression.
Restarting at full dose (120 mg/day) risks recurrent cytopenias.

2 Why Not Permanently Discontinue Gilteritinib?
FLT3-ITD+ AML has a high post-transplant relapse risk (~70-80% without maintenance).
Stopping maintenance therapy altogether significantly increases relapse risk.

3 Why Not Switch to Sorafenib?
Sorafenib is another FLT3 inhibitor, but its not the preferred first-line post-transplant maintenance in this setting.
Switching is only considered if Gilteritinib remains intolerable despite dose reduction.
Case Update: 3 Months Later

The patient is tolerating Gilteritinib 80 mg/day well, with stable ANC (1.5) and platelets (100k).
Follow-up MRD testing (NGS + flow cytometry) remains negative.
However, a routine post-transplant surveillance BM biopsy at 6 months shows 2% FLT3-ITD+ blasts.

Question 12: What is the best next step?

A) Increase Gilteritinib back to 120 mg/day
B) Add Azacitidine to Gilteritinib (Hypomethylating agent + FLT3 inhibitor combo)
C) Consider a second Allo-HSCT
D) Closely monitor with repeat BM biopsy in 1 month

Type your answer (A, B, C, or D) and justify your choice!
my answer:
The answer is (B)
The reason is :
1.Emerging MRD+ Relapse (2% FLT3-ITD+ blasts) warrants immediate intervention to prevent overt relapse.
- Gilteritinib monotherapy at 80 mg/day may be insufficient to suppress MRD.
- Azacitidine + FLT3 inhibitor has shown synergy in preclinical/early clinical studies for MRD+ FLT3-ITD AMLm (e.g., FLT3-ITD clearance in some cases).

Ai evaluation:
Correct! The best answer is (B) Add Azacitidine to Gilteritinib (Hypomethylating agent + FLT3 inhibitor combo).

Key Takeaways:

1 MRD+ (2% FLT3-ITD+ Blasts) = High Relapse Risk
MRD persistence post-transplant is highly predictive of overt relapse.
Acting early can prevent full-blown AML recurrence.

2 Why Azacitidine + Gilteritinib?
Hypomethylating agents (HMA) like Azacitidine enhance FLT3 inhibitor efficacy.
Studies (e.g., Quazar AML-001 subanalysis, early trials) suggest this combo improves MRD clearance and prolongs remission.
A phase 3 trial is ongoing to confirm this approach.

3 Why Not the Other Options?
(A) Increasing Gilteritinib back to 120 mg/day
This alone may not be sufficient to clear MRD.
If the patient previously had dose-limiting cytopenias, increasing the dose could worsen marrow suppression.
(C) Second Allo-HSCT
Too earlyMRD+ alone does not justify immediate re-transplant.
Consider only if MRD persists despite therapy or overt relapse occurs.
(D) Monitor & Repeat BM in 1 Month
Waiting risks full relapse.
MRD-directed intervention is the standard of care.
Case Update: 2 Months Later

The patient receives Azacitidine + Gilteritinib (80 mg/day).
BM biopsy at 8 months post-HSCT shows MRD negativity (FLT3-ITD undetectable).
Peripheral counts are stable.

However, the patient develops mild cytopenias (ANC 0.9, Plt 65k) but remains transfusion-independent.

Question 13: What is the best next step?

A) Continue the current regimen without changes
B) Reduce Gilteritinib dose to 40 mg/day
C) Hold Azacitidine and reassess in 2 weeks
D) Perform a repeat BM biopsy to evaluate for evolving relapse vs. toxicity

Type your answer (A, B, C, or D) and justify your choice!
answer:
The answer is (A)
The reason is :
1.Clinical Context is Favorable:
MRD negativity achieved (primary goal of therapy).
Cytopenias are mild (ANC 0.9, Plt 65k) and transfusion independent consistent with expected myelosuppression from Azacitidine + Gilteritinib.

AI:
Correct! The best answer is (A) Continue the current regimen without changes.

Key Takeaways:

1 MRD-Negative = Successful Intervention!

The primary goal of therapy (FLT3-ITD clearance) has been achieved.
Stopping or reducing therapy prematurely increases relapse risk.

2 Mild Cytopenias Are Expected
ANC 0.9, Plt 65k Still above critical thresholds.
Common with Azacitidine + Gilteritinib but does not warrant dose reduction unless severe (e.g., ANC <0.5 or recurrent infections).

3 Why Not Other Options?
(B) Reduce Gilteritinib dose to 40 mg/day Unnecessary unless cytopenias worsen or patient becomes transfusion-dependent.
(C) Hold Azacitidine and reassess in 2 weeks Stopping Azacitidine risks MRD re-emergence.
(D) Repeat BM biopsy No suspicion of relapse; monitoring counts is sufficient.

Next Steps:

Continue Azacitidine + Gilteritinib (80 mg/day)
Monitor CBC weeklyif ANC <0.5 or worsening thrombocytopenia, consider dose adjustments.
Reassess MRD every 3 months to ensure sustained remission.
Question 14:

A 52-year-old male with FLT3-ITD+ AML underwent allogeneic HSCT and has been on gilteritinib maintenance. At a routine follow-up, he reports worsening fatigue and mild shortness of breath. His labs show:
Hemoglobin: 7.8 g/dL (previously 10.2 g/dL)
Reticulocyte count: 0.3% (low)
Bilirubin, LDH: Normal
Bone marrow biopsy: Hypocellular marrow with no evidence of relapse

What is the most appropriate next step?
(A) Continue gilteritinib and monitor closely
(B) Test for parvovirus B19 and consider IVIG if positive
(C) Start empiric steroids for possible late-onset GVHD
(D) Discontinue gilteritinib permanently due to toxicity
My answer:
The answer is (B) for question 14
The reason is :
1. Clinical Presentation:
- Progressive anemia (Hb drop from 10.2 7.8 g/dL) with low reticulocytes suggests pure red cell aplasia (PRCA).
- Hypocellular marrow without relapse rules out AML recurrence but fits parvovirus B19-induced PRCA a known post-transplant complication.

AI evaluation:
Great reasoning! Parvovirus B19 is a well-recognized cause of pure red cell aplasia (PRCA) in immunocompromised patients, especially post-transplant. Testing for parvovirus B19 PCR and considering IVIG therapy if positive is the correct approach.

Question 15:

A 60-year-old female with FLT3-ITD+ AML, post-HSCT, is on gilteritinib maintenance. She presents with new onset headaches and confusion. MRI shows punctate hemorrhages, and her platelet count is 110k. Laboratory workup reveals:
LDH: Elevated
Haptoglobin: Low
Schistocytes on peripheral smear
Normal coagulation panel

What is the most likely diagnosis?
(A) Central nervous system relapse of AML

(B) Thrombotic microangiopathy (TMA)
(C) Immune thrombocytopenia (ITP)
(D) Gilteritinib-induced neurotoxicity

my answer:
The most likely diagnosis is (B) Thrombotic microangiopathy (TMA). For question 15
THE reason is :
1. Clinical Triad:
   - Neurologic symptoms (headaches, confusion)
   - Microangiopathic hemolytic anemia (schistocytes, LDH, haptoglobin)
   - Thrombocytopenia (platelets 110k, though may be higher early in TMA)

2. Why TMA?
   - Post-HSCT setting: High risk for TMA due to endothelial injury (conditioning chemo, GVHD, calcineurin inhibitors).
   - Gilteritinib association: FLT3 inhibitors can exacerbate endothelial dysfunction increasing TMA risk.
   -MRI findings: Punctate hemorrhages fit microvascular thrombosis/ischemia (classic for TMA).

AI evaluation:
Excellent analysis! Thrombotic microangiopathy (TMA) is a well-recognized post-HSCT complication, often triggered by calcineurin inhibitors, endothelial injury, or FLT3 inhibitors like Gilteritinib. Your reasoning aligns with the characteristic triad of TMA and the supporting lab/microangiopathic findings.

Session ID:
Assessment Domain

Session-25:
Rationale

Session ID:
Instructional Design and Pedagogical

Session-25:
The session demonstrates exceptional instructional design with expertly scaffolded progression from initial AML presentation through complex post-transplant management. The case flows logically through diagnosis risk stratification induction complications consolidation transplantation post-transplant monitoring and complications. The embedded feedback is outstanding, providing detailed, evidence-based rationales with specific trial references (RATIFY, MORPHO, SORMAIN, ADMIRAL) and clinical pearls that enhance learning retention. Instructions are consistently clear and well-framed throughout all 15 questions. The only minor weakness is the absence of explicitly stated learning objectives at the session's start, though educational goals are clearly implied through the comprehensive case structure and content progression.

Session ID:
Clinical Authenticity and Content Fidelity

Session-25:
The clinical authenticity is exemplary throughout the entire case progression. The clinical narrative presents as entirely plausible with realistic laboratory values, appropriate clinical timeline, and authentic complications (febrile neutropenia, GVHD, drug-related cytopenias, parvovirus B19, TMA). All therapeutic decisions align perfectly with current evidence-based guidelines, including correct application of ELN 2022 risk stratification, appropriate use of 7+3 + midostaurin per RATIFY trial data, and evidence-based post-transplant management strategies. Pharmacotherapeutic choices are consistently optimal and well-justified, from initial induction through complex scenarios like MRD-positive disease management and post-transplant complications. The case maintains strict domain specificity without any inappropriate application of therapies from other disease statesall recommendations (midostaurin, gilteritinib, azacitidine combinations, supportive care measures) remain within established AML standards of care without cross-contamination from ALL, CML, or other hematologic malignancies.

Session ID:

Modeled Clinical Reasoning

Session-25:

The simulation excellently models expert-level clinical reasoning throughout the comprehensive patient journey. Each clinical scenario is expertly framed with appropriate data presentation and clinical context, allowing for informed decision-making at every juncture. The multiple-choice options demonstrate excellent breadth, incorporating relevant distractors that effectively test nuanced understanding of AML management principles. The case demonstrates consistently appropriate clinical prioritization, from securing molecular diagnostics before therapy initiation to managing competing priorities like GVHD versus maintenance therapy continuation. The modeled clinical outcomes are highly realistic and instructionally valuable, including appropriate recognition of complications like post-transplant cytopenias, infectious complications (parvovirus B19), and vascular complications (TMA) that reinforce learning objectives and prepare learners for complex real-world clinical scenarios in FLT3-ITD+ AML management.

Session-26

Session ID:
LLM

Session-26:
ChatGPT (GPT 4.o)

Session ID:
Session Transcript

Session-26:
Phase 1: Initial Presentation and Diagnostic Workup

Heres your patient:

Patient Profile:

Name: Mr. John Doe

Age: 62

Chief complaint: Fatigue and unexplained weight loss over the past two months

Medical history: Hypertension, Type 2 diabetes mellitus, no known history of cancer

Current medications: Metformin, Lisinopril

Family history: Father had prostate cancer, mother had breast cancer

Social history: Non-smoker, occasional alcohol use, lives with wife and adult children

Presenting Symptoms:

Fatigue

Anemia (Hb 9.5 g/dL)

Frequent bruising

Shortness of breath on exertion

Initial Lab Results:

WBC: 15,000/L (with 10% blasts)

Platelets: 75,000/L

Neutrophils: 5,000/L

Bone marrow biopsy: Shows 25% blasts

Phase 1 Question 1:

Based on the initial presentation and lab results, which of the following is the most likely diagnosis?

1. Acute Myeloid Leukemia (AML)

2. Chronic Myelogenous Leukemia (CML)

3. Myelodysplastic Syndrome (MDS)

4. Aplastic Anemia

Please select the correct answer
1
Correct!

Acute Myeloid Leukemia (AML) is the most likely diagnosis, given the patient's presenting symptoms (fatigue, bruising), lab findings (anemia, thrombocytopenia), and bone marrow biopsy showing 25% blasts. AML is characterized by the presence of 20% blasts in the bone marrow, which is consistent with the findings in this case.
Phase 1 Question 2:

Which diagnostic test is most crucial for confirming the diagnosis of AML and determining the treatment approach?

1. Flow cytometry

2. Cytogenetic analysis

3. Complete blood count (CBC)

4. Bone marrow biopsy

4
Correct!

Bone marrow biopsy is crucial for confirming the diagnosis of AML. It provides essential information about the percentage of blasts in the bone marrow, which is necessary for diagnosing AML (20% blasts). It also helps in distinguishing AML from other hematologic conditions, such as myelodysplastic syndrome (MDS) or aplastic anemia.

However, remember that cytogenetic analysis and molecular testing are also vital for risk stratification and treatment planning, as they can provide prognostic information that guides therapy choices
Phase 1 Question 3:

What additional test would you consider next to further assess the molecular and genetic characteristics of this patient's AML?

1. Flow cytometry

2. FISH (Fluorescence in situ hybridization)

3. Complete metabolic panel

4. Bone marrow culture
2
Correct!

FISH (Fluorescence in situ hybridization) is an essential test for assessing cytogenetic abnormalities in AML. It can identify specific chromosomal mutations that have prognostic significance, such as FLT3, NPM1, or CEBPA mutations. These mutations help in risk stratification and guide treatment decisions.

While flow cytometry is important for confirming the immunophenotype of the blasts and identifying minimal residual disease (MRD), FISH is more directly linked to the genetic and molecular risk assessment in AML.

Phase 1 Question 4:

Given this patients age and presenting symptoms, which of the following comorbidities should you also consider when planning treatment?

1. Cardiovascular disease

2. Renal insufficiency

3. Hepatic dysfunction

4. All of the above

4

Correct!

You should consider all of the above when planning treatment. In older patients like this one (age 62), comorbidities such as cardiovascular disease, renal insufficiency, and hepatic dysfunction can significantly influence treatment decisions, including the choice of induction therapy, dosing adjustments, and supportive care.

Cardiovascular disease may affect the tolerance to certain chemotherapy agents, particularly anthracyclines like daunorubicin.

Renal insufficiency could affect drug clearance, requiring dose adjustments for certain medications.

Hepatic dysfunction can impact drug metabolism and necessitate dose modifications for some agents.

Considering these factors is essential for tailoring a safe and effective treatment plan for the patient.
Phase 1 Question 5:

Which of the following AML risk stratification tools is most appropriate to assess this patient's prognosis and guide treatment decisions?

1. The National Comprehensive Cancer Network (NCCN) risk criteria

2. The ELN 2022 risk classification

3. The FLT3 mutation status

4. The WHO 2016 classification

Hint

Consider using a classification system that incorporates both cytogenetic and molecular abnormalities, as well as age and performance status. The ELN 2022 risk classification is widely used to assess the prognosis of AML patients by stratifying them into favorable, intermediate, and adverse risk groups based on molecular and cytogenetic findings.

The WHO 2016 classification, while essential for diagnosing AML, is more focused on morphological and genetic characteristics, and is less helpful for treatment decision-making compared to the ELN classification.

2
Correct!

The ELN 2022 risk classification is the most appropriate tool to assess this patient's prognosis and guide treatment decisions. It integrates cytogenetic abnormalities, molecular mutations, and clinical factors such as age and performance status. This system helps stratify patients into favorable, intermediate, or adverse risk categories, which significantly influences the treatment approach, including the choice of induction therapy and the consideration of novel agents.

Phase 2 Question 1:

Which of the following induction regimens would be most appropriate for this patient?

1. 7+3 (Cytarabine + Daunorubicin)

2. CPX-351 (Liposome-encapsulated Cytarabine + Daunorubicin)

3. Venetoclax + Azacitidine

4. Hydroxyurea monotherapy

Hint

Consider the patient's age, comorbidities, and risk classification when selecting induction therapy. The 7+3 regimen (Cytarabine + Daunorubicin) is typically the standard first-line therapy for younger, fit patients, but in older adults or those with comorbidities, the toxicity might be too high.

CPX-351 could be an option in older patients or those with specific high-risk features (e.g., secondary AML), as it's designed to be more tolerable and effective.

Venetoclax + Azacitidine is commonly used in patients who are older or have comorbidities, as its generally better tolerated compared to 7+3.

Hydroxyurea is used for cytoreduction in CML but is not a treatment for AML.

Would you like to revise your answer?

2

Good choice!

CPX-351 (Liposome-encapsulated Cytarabine + Daunorubicin) is often preferred in older patients or those with comorbidities like this one. It has been shown to be more effective and better tolerated than standard 7+3 in some high-risk AML populations, especially in

patients aged 60 and older. The liposomal formulation allows for prolonged drug exposure with a more favorable toxicity profile.

Phase 2 Question 2:

Which of the following supportive care strategies should be prioritized during the initial phase of induction therapy for this patient?

1. Antifungal prophylaxis with fluconazole

2. Blood transfusions to manage anemia and thrombocytopenia

3. Antiviral prophylaxis with acyclovir

4. All of the above

4

Correct!

All of the above are important supportive care strategies during the initial phase of induction therapy for AML. These interventions help manage the common complications associated with intensive chemotherapy:

Antifungal prophylaxis with fluconazole is critical due to the increased risk of fungal infections during periods of neutropenia.

Blood transfusions are necessary to address the patient's anemia and thrombocytopenia, ensuring adequate oxygen transport and reducing the risk of bleeding.

Antiviral prophylaxis with acyclovir helps prevent reactivation of herpes simplex virus, which is a concern in immunocompromised patients.
Phase 2 Question 3:

In the context of this patient's age and comorbidities, what dose modification or monitoring would be most important for the induction regimen of CPX-351?

1. Adjusting for renal dysfunction

2. Monitoring for signs of cardiotoxicity

3. Decreasing the dose of liposomal cytarabine

4. Dose reduction due to hepatic dysfunction

Hint

Consider the potential toxicities associated with CPX-351, especially in older patients or those with comorbidities like hypertension and diabetes.

Cardiotoxicity is a known risk with anthracyclines, including daunorubicin (which is part of CPX-351). Given the patient's age and the potential for underlying cardiovascular issues, its crucial to monitor for signs of cardiotoxicity (e.g., heart failure, arrhythmias) during therapy.

Renal and hepatic adjustments are generally less of a concern with CPX-351, but they should still be monitored, especially for dose adjustments in other therapies.

Would you like to revise your answer based on this guidance?

2

Correct!

Monitoring for signs of cardiotoxicity is the most important consideration when administering CPX-351 in this patient, particularly because the regimen includes daunorubicin, an anthracycline known for its potential to cause cardiotoxicity. Given the patients age and possible underlying cardiovascular risk factors (such as hypertension), close monitoring of cardiac function (e.g., through echocardiograms or clinical observation for signs of heart failure) is crucial during treatment.
Phase 2 Question 4:

What is the most appropriate method to monitor this patients response to therapy during induction?

1. Repeat bone marrow biopsy after 1-2 cycles

2. Peripheral blood counts to monitor for blast clearance

3. Cytogenetic testing to evaluate minimal residual disease (MRD)

4. Imaging studies (e.g., CT scans) to assess organ involvement

Hint

To assess the patients response to induction therapy, the focus should be on evaluating blast clearance and minimal residual disease (MRD).

Repeat bone marrow biopsy can confirm remission but isnt typically done after just one or two cycles. Its more common after a few months of treatment to confirm complete remission.

Peripheral blood counts are frequently monitored during therapy to track improvements in blood cell counts and assess for blast clearance.

Cytogenetic testing is helpful for evaluating MRD after therapy, which is crucial for assessing the likelihood of relapse.

Imaging studies are typically not necessary for assessing response to induction unless there is concern for extramedullary disease (e.g., in testicular or CNS involvement).

Would you like to revise your answer based on this guidance?

2

Correct!

Peripheral blood counts are the most appropriate method to monitor the response to therapy during induction. In AML, improvement in peripheral blood counts (e.g., a reduction in blasts and recovery of neutrophils and platelets) can indicate a positive response to induction therapy. Monitoring for blast clearance is key to assessing how effectively the patient is responding to treatment.

While cytogenetic testing and bone marrow biopsy can provide more detailed information about minimal residual disease (MRD) and

remission status, peripheral blood counts are typically assessed more frequently and provide immediate insight into the patients response during treatment
Phase 2 Question 5:

Which of the following complications is most likely to occur during the first few weeks of induction therapy, especially in this patient?

1. Tumor lysis syndrome (TLS)

2. Invasive fungal infection

3. Cardiotoxicity

4. Severe gastrointestinal bleeding

Please select your answer.

2

Correct!

Invasive fungal infection is the most likely complication in the first few weeks of induction therapy, especially in this patient. During the neutropenic phase (when the white blood cell count is extremely low), patients are highly susceptible to infections, including fungal infections like Aspergillus. Older patients and those undergoing intensive chemotherapy are at an increased risk, so prophylactic antifungal therapy is typically given during this phase.

While tumor lysis syndrome can occur, its more common in patients with high tumor burden or rapidly proliferating leukemia, which isnt indicated in this case.

Phase 3 Question 1:

What is the most appropriate next step in post-remission therapy for this patient?

1. Autologous stem cell transplant

2. Allogeneic stem cell transplant

3. Consolidation chemotherapy with high-dose cytarabine

4. Venetoclax + Azacitidine

Please select your answer
Hint

When considering post-remission therapy, it's important to assess the patient's risk of relapse, age, and comorbidities. Given that this patient has achieved a complete remission (CR) but remains at high risk for relapse, heres some context:

Autologous stem cell transplant is generally not preferred for AML because of the risk of relapse, as the patients own stem cells may harbor residual leukemia.

Allogeneic stem cell transplant is a more intensive option and may be considered for patients at high risk for relapse, particularly those with adverse cytogenetics or other high-risk features. However, its associated with significant morbidity and mortality.

Consolidation chemotherapy with high-dose cytarabine is a standard approach for patients in CR. It helps eliminate any remaining leukemic cells and reduce the risk of relapse.

Venetoclax + Azacitidine is typically used for patients who are older or have comorbidities, particularly when standard chemotherapy is less feasible. It's not the first choice for post-remission consolidation in younger patients with good performance status

3

Correct!

Consolidation chemotherapy with high-dose cytarabine is the most appropriate next step in post-remission therapy for this patient. High-dose cytarabine is a standard treatment for patients in complete remission (CR) to help eliminate any residual leukemia cells and reduce the risk of relapse. This approach is commonly used for patients with intermediate risk and those who are relatively young and fit for intensive treatment.

Allogeneic stem cell transplant may be considered in the future if the patient experiences a relapse or if they develop high-risk features during follow-up.

Venetoclax + Azacitidine is more often used in older patients or those with significant comorbidities, and its typically not the first-line approach for consolidation in fit patients like this one.

Phase 3 Question 2:

After completing consolidation therapy, what is the best approach to monitor this patient for relapse or minimal residual disease (MRD)?

1. Repeat bone marrow biopsy every 3 months

2. Regular peripheral blood monitoring for blasts and complete blood counts (CBC)

3. Perform MRD testing using PCR for NPM1 mutation

4. Imaging studies every 6 months to detect relapse

Hint

To monitor for relapse or minimal residual disease (MRD) in this patient, consider the methods that can detect the presence of leukemia at a very low level, even when the patient appears to be in remission:

Bone marrow biopsy is an option, but its more invasive and typically done when there's concern for relapse or if other methods of monitoring (like MRD testing) indicate a potential issue.

Peripheral blood monitoring is important for tracking changes in blood counts, but it may not always detect MRD.

MRD testing using PCR for specific mutations (e.g., NPM1 in this case) is a highly sensitive method for detecting residual disease that may not be detectable through routine blood counts or imaging.

Imaging studies are generally not useful for detecting relapses unless theres concern for extramedullary disease (like in the CNS or testicles).
3

Correct!

MRD testing using PCR for NPM1 mutation is the most sensitive method to monitor for minimal residual disease (MRD) after consolidation therapy in this patient. MRD testing can detect leukemia at very low levels, even when the patient appears to be in complete remission based on peripheral blood counts. In patients with mutations such as NPM1, PCR is a powerful tool to identify residual disease and predict the risk of relapse.

Peripheral blood monitoring is important but less sensitive for detecting MRD compared to PCR-based tests.

Bone marrow biopsy can confirm relapse but is typically reserved for cases where there is clinical suspicion of relapse, especially if MRD testing shows positivity.

Imaging studies are not necessary unless there is concern for extramedullary disease.
Phase 3 Question 3:

What is the most appropriate next step if MRD testing at 3 months post-consolidation reveals a positive result?

1. Proceed with a second round of consolidation chemotherapy

2. Consider allogeneic stem cell transplant due to the high risk of relapse

3. Begin maintenance therapy with low-dose chemotherapy

4. Monitor closely without changing the treatment plan

2

Correct!

Allogeneic stem cell transplant is the most appropriate next step if MRD testing at 3 months post-consolidation reveals a positive result. A positive MRD result after consolidation therapy signals that residual disease is still present, which significantly increases the risk of relapse. Allogeneic stem cell transplant offers the best chance for long-term remission or potential cure by replacing the patient's immune system with that of a donor, which can help eliminate any remaining leukemia cells.

A second round of consolidation chemotherapy may help reduce MRD but is less likely to completely eradicate the disease.

Maintenance therapy with low-dose chemotherapy is typically used for patients with a lower relapse risk, not those with positive MRD.

Monitoring closely without intervention is not advisable in this scenario due to the high relapse risk indicated by the positive MRD result.
Phase 3 Question 4:

After successful allogeneic stem cell transplant, what is the most appropriate strategy for long-term management?

1. Regular MRD testing every 3 months for the first year

2. Discontinuation of all immunosuppressive therapy after 6 months

3. Annual bone marrow biopsies to monitor for relapse

4. Immediate initiation of maintenance chemotherapy after transplant

Hint

For long-term management following allogeneic stem cell transplant, the goal is to monitor for relapse and graft-versus-host disease (GVHD), as well as to manage the patients immune system. Here's a breakdown of the options:

Regular MRD testing every 3 months for the first year is essential to monitor for relapse, especially in the first year after transplant when the risk of relapse is highest. MRD testing can help detect very early signs of relapse.

Discontinuation of immunosuppressive therapy is often done after 6 months, but this depends on the patients tolerance, risk for GVHD, and other factors. Immunosuppressive therapy is tapered to prevent GVHD, but it shouldnt be abruptly stopped unless its safe.

Annual bone marrow biopsies are generally not needed unless theres concern for relapse or signs of abnormality. MRD testing is often more sensitive.

Immediate initiation of maintenance chemotherapy is typically not needed after stem cell transplant unless there are signs of relapse or the patient has a higher risk of relapse, in which case other therapies like maintenance with hypomethylating agents may be considered.

1

Correct!

Regular MRD testing every 3 months for the first year is the most appropriate strategy for long-term management following allogeneic stem cell transplant. This allows for early detection of relapse, as MRD testing is much more sensitive than other methods (like bone marrow biopsy) and can detect residual disease before it becomes clinically apparent.

During the first year, the patient is at high risk for relapse, so frequent monitoring is crucial for timely intervention if relapse occurs.

Discontinuation of immunosuppressive therapy happens gradually after 6 months, but only if the patient is stable and not at high risk for GVHD.

Bone marrow biopsies are less frequently done unless indicated by clinical symptoms or abnormal MRD results.

Maintenance chemotherapy is typically reserved for specific cases, such as those with higher relapse risk, and is not routinely given post-transplant unless relapse is detected.
Phase 3 Question 5:

What is the most appropriate next step if the patient develops graft-versus-host disease (GVHD) following the allogeneic stem cell transplant?

1. Increase immunosuppressive therapy to prevent GVHD progression

2. Discontinue immunosuppressive therapy and monitor closely

3. Initiate targeted therapy with ruxolitinib (JAK inhibitor)

4. Administer a second transplant to treat GVHD

2

Not quitelet me clarify.

In the case of graft-versus-host disease (GVHD) following an allogeneic stem cell transplant, the goal is to manage the condition carefully to balance controlling the immune response while avoiding excessive immunosuppression that could increase the risk of infection or relapse. Heres why the other options are more appropriate:

Increasing immunosuppressive therapy is typically the first-line approach to manage GVHD, especially in cases where it is moderate to severe. This is done to prevent the disease from worsening and to control the inflammatory response. Common agents used include steroids and other immunosuppressive medications.

Discontinuing immunosuppressive therapy is generally not advised in active GVHD. Stopping immunosuppressive therapy can lead to worsening of the GVHD, so it's crucial to carefully adjust the immunosuppressive regimen rather than discontinuing it entirely.

Ruxolitinib (a JAK inhibitor) is increasingly used for steroid-refractory GVHD as a targeted therapy to reduce inflammation and modulate the immune system. This is appropriate if GVHD does not respond to steroids.

A second transplant is generally not used for GVHD treatment. GVHD is managed with immunosuppressive therapy or specific agents like ruxolitinib, not through a second transplant.

Therefore, the most appropriate response to active GVHD is to increase immunosuppressive therapy (Option 1).

Session ID:
Assessment Domain

Session-26:
Rationale

Session ID:
Instructional Design and Pedagogical

Session-26:
The session's instructional design has significant strengths, particularly in its structure and clarity. The case is expertly scaffolded, guiding the learner through the entire patient journey from initial diagnosis and risk stratification to induction, consolidation, and complex post-transplant management. Instructions for each question are exceptionally clear. However, the pedagogical quality is diminished by two factors. First, no explicit learning objectives are provided at the outset, leaving them to be inferred from the content. Second, and more critically, the embedded feedback in two key instances (Phase 1, Q3 and Phase 2, Q4) is factually incorrect, which undermines the reliability of the instruction and lowers the overall feedback quality score.

Session ID:
Clinical Authenticity and Content Fidelity

Session-26:

The session presents a mixed picture regarding its clinical fidelity. The clinical narrative is outstanding and indistinguishable from an authentic patient case, and the specific pharmacotherapeutic choices (e.g., CPX-351, HiDAC consolidation) are optimal and guideline-directed. However, the simulation contains two significant content-level errors that deviate from standard clinical practice and knowledge:

Incorrect Diagnostic Test Rationale (Phase 1, Q3): The session incorrectly states that FISH is used to detect molecular mutations like FLT3, NPM1, or CEBPA. FISH is a cytogenetic technique used to identify large-scale chromosomal abnormalities (e.g., translocations), whereas molecular mutations are detected by methods like PCR or NGS. This is a fundamental error in molecular diagnostics.

Incorrect Response Monitoring (Phase 2, Q4): The session incorrectly identifies peripheral blood counts as the most appropriate method for monitoring response to induction therapy. The standard of care for definitively assessing induction response is a bone marrow biopsy performed around day 14-21 to evaluate for marrow aplasia and blast clearance. Misrepresenting this crucial step is a significant inaccuracy.

There was no evidence of domain entanglement; all concepts remained within the scope of AML management.

Session ID:

Modeled Clinical Reasoning

Session-26:

The session excels at modeling an expert clinical reasoning process. Each question presents a well-framed, relevant clinical problem that builds upon the last. The multiple-choice options are thoughtfully constructed, providing a strong range of plausible and relevant alternatives that effectively test nuanced understanding and challenge the learner to differentiate between good and optimal choices. The overall strategic flow of the case demonstrates sound prioritization, correctly modeling key decision points like escalating care to allogeneic transplant in the setting of MRD positivity. Despite the content errors noted in Section 2, the overarching cognitive pathway modeled by the simulation is logical, sound, and highly instructive.

Session-27

Session ID:
LLM

Session-27:
ChatGPT (GPT 4.o)

Session ID:
Session Transcript

Session-27:
Phase 1: Initial Presentation & Diagnostic Workup
Patient Case Introduction
A 62-year-old male presents to the emergency department with: Fatigue, pallor, and easy bruising for the past 3 weeks Occasional gum bleeding and feversPast medical history: Hypertension (controlled on amlodipine 5 mg daily)No significant family history of malignanciesInitial Workup: Vital Signs: HR 98 bpm, BP 128/76 mmHg, Temp 38.2CPhysical Exam: Mild ecchymoses on extremities, no hepatosplenomegaly, nolymphadenopathy
Laboratory Results:
CBC: WBC 3.1 x 10/L, Hb 7.8 g/dL, Plt 28 x 10/L
Differential: 30% blasts, neutrophils 28%, lymphocytes 35%, monocytes 7%
Peripheral Smear: Circulating blasts with Auer rods seen
Coagulation: INR 1.2, aPTT 32 sec, fibrinogen 210 mg/dL
CMP: Creatinine 0.9 mg/dL, ALT/AST normal, LDH elevated
Bone Marrow Biopsy: Hypercellular marrow with 65% myeloblasts
Flow Cytometry: CD34+, CD117+, MPO+
Cytogenetics & Molecular Testing: Pending
Question 1: Based on the initial workup, what is the most likely diagnosis?
A. Myelodysplastic syndrome with excess blasts-2 (MDS-EB2)
B. Acute Myeloid Leukemia (AML)
C. Acute Lymphoblastic Leukemia (ALL)
D. Chronic Myelomonocytic Leukemia (CMML)

Type A, B, C, or D to select your answer.
B
Correct! The most likely diagnosis is Acute Myeloid Leukemia (AML).

Rationale:
Blast count: 65% myeloblasts in the bone marrow (AML is diagnosed when blasts 20%)
Peripheral smear: Presence of Auer rods, which are specific to AML
Flow cytometry: CD34+, CD117+, MPO+, consistent with a myeloid lineage
WHO 2022 criteria: This patient meets the criteria for AML based on blast percentage

Question 2: Which genetic test result would automatically classify this AML as adverse risk per ELN 2022 criteria?
A. NPM1 mutation (without FLT3-ITD)
B. t(8;21)(q22;q22) RUNX1-RUNX1T1
C. TP53 mutation
D. FLT3-ITD (high allelic ratio)

Type A, B, C, or D to select your answer.
C
Correct! TP53 mutation automatically classifies AML as adverse risk per ELN 2022 criteria, regardless of other cytogenetic or molecular findings.

Rationale:
TP53 mutations are associated with poor response to standard chemotherapy, high relapse rates, and low overall survival.
Patients with TP53-mutated AML often require alternative approaches, such as investigational therapies or early consideration of allogeneic stem cell transplant (if feasible).

Next Step: Treatment Planning

Now that weve established AML with adverse-risk features (TP53 mutation), we need to decide on an induction therapy strategy.

Question 3: Which of the following is the MOST appropriate induction therapy for this patient?

A. 7+3 (Cytarabine + Daunorubicin)
B. CPX-351 (Liposomal Cytarabine + Daunorubicin)
C. Venetoclax + Azacitidine
D. FLAG-IDA (Fludarabine + Cytarabine + G-CSF + Idarubicin)

Type A, B, C, or D to select your answer.
C
Correct! Venetoclax + Azacitidine is the most appropriate induction therapy for this patient with TP53-mutated AML.

Next Step: Supportive Care During Induction

Since the patient will start Venetoclax + Azacitidine, we need to optimize supportive care to prevent complications like tumor lysis syndrome (TLS) and infections.

Question 4: What is the MOST important initial supportive care measure when starting Venetoclax in this patient?

A. Prophylactic broad-spectrum antibiotics
B. Tumor lysis syndrome (TLS) risk assessment & prophylaxis
C. Erythropoiesis-stimulating agent (ESA) for anemia
D. G-CSF (Filgrastim) to prevent neutropenia

Type A, B, C, or D to select your answer.
B
Correct! Tumor lysis syndrome (TLS) risk assessment & prophylaxis is the most important initial supportive care measure when starting Venetoclax.

Next Step: Monitoring Response

The patient has started Venetoclax + Azacitidine with appropriate TLS prophylaxis. After 28 days, we perform a bone marrow biopsy to assess response.

Question 5: Which finding would indicate a COMPLETE RESPONSE (CR) based on ELN 2022 criteria?

A. <5% blasts, ANC >1000/L, Platelets >100,000/L
B. 5-10% blasts, ANC >500/L, Platelets >50,000/L
C. No detectable blasts, persistent cytopenias, negative MRD
D. <5% blasts, persistent transfusion dependence

Type A, B, C, or D to select your answer.
A
Correct! <5% blasts, ANC >1000/L, and Platelets >100,000/L define a Complete Response (CR) per ELN 2022 criteria.
Next Step: Post-Remission Therapy

Since the patient has achieved CR with Venetoclax + Azacitidine, the next step is to decide post-remission therapy to prevent relapse.

Question 6: What is the MOST appropriate next step for post-remission therapy in this patient with TP53-mutated AML?

A. Continue Venetoclax + Azacitidine indefinitely
B. Allogeneic stem cell transplant (if feasible)
C. High-dose Cytarabine (HiDAC) consolidation
D. Stop treatment and observe

Type A, B, C, or D to select your answer.
B
Correct! Allogeneic stem cell transplant (if feasible) is the most appropriate post-remission therapy for this patient with TP53-mutated AML.

.
Next Phase: Post-Remission Therapy and Long-Term Management

The patient undergoes an allogeneic stem cell transplant and enters the post-transplant recovery phase. Close monitoring for measurable residual disease (MRD), graft-versus-host disease (GVHD), and other complications is essential.

Question 7: In the post-transplant setting, which of the following is an important strategy for monitoring disease status?

A. Routine complete blood count (CBC) only
B. Periodic bone marrow biopsy with MRD assessment
C. CT scans of the chest and abdomen
D. Monthly viral load testing

Type A, B, C, or D to select your answer.
B
Correct! Periodic bone marrow biopsy with MRD assessment is crucial in the post-transplant setting for monitoring disease status.

Next Step: Long-Term Management and Follow-Up

As we continue with the post-transplant phase, focus on balancing surveillance for relapse with managing potential transplant-related complications.

Question 8: In the setting of a rising MRD level post-transplant, what is the most appropriate immediate management strategy?

A. Initiate donor lymphocyte infusion (DLI)
B. Restart Venetoclax + Azacitidine
C. Perform another bone marrow biopsy in 6 months
D. Increase immunosuppressive therapy

Type A, B, C, or D to select your answer.
A
Correct! Initiating a donor lymphocyte infusion (DLI) is the most appropriate immediate management strategy when there is a rising MRD post-transplant.

Next Steps in Long-Term Management

Continuous monitoring of MRD and careful management of post-transplant complications, including graft-versus-host disease (GVHD), remain critical. Regular follow-up and timely intervention based on MRD trends can significantly impact long-term outcomes.

Question 9: Which of the following factors is most critical in deciding the timing of DLI post-transplant?

A. The patients age
B. The degree of MRD elevation
C. The time elapsed since transplant
D. The presence of GVHD

Type A, B, C, or D to select your answer.
D
Correct! The presence of GVHD is the most critical factor in deciding the timing of DLI post-transplant.

Next Step: Continuing Post-Transplant Monitoring and Management

Effective long-term management in AML post-transplant involves ongoing MRD monitoring, management of GVHD, and early intervention when relapse is detected. Balancing the benefits of the graft-versus-leukemia effect with the risks of GVHD remains central to post-transplant strategies.

Question 10: In a patient with well-controlled GVHD and rising MRD levels, what additional intervention might be considered alongside DLI to further enhance the graft-versus-leukemia effect?

A. Withdrawal of immunosuppressive therapy
B. Increase the dose of immunosuppressive agents
C. Initiate prophylactic antiviral therapy
D. Start high-dose corticosteroids

Type A, B, C, or D to select your answer.
A
Correct! Withdrawing or reducing immunosuppressive therapy (option A) is the additional intervention that may enhance the graft-versus-leukemia (GVL) effect in a patient with well-controlled GVHD and rising MRD levels.

.
Next Steps in the Session:

We will continue with the long-term management phase, focusing on how to integrate MRD monitoring with supportive care measures and relapse prevention strategies.

Question 11: As part of long-term follow-up post-transplant, which additional supportive measure is crucial to address in this patient?

A. Routine dental hygiene
B. GVHD prophylaxis and monitoring
C. Nutritional counseling only
D. Strict bed rest

Type A, B, C, or D to select your answer.
B
Correct! GVHD prophylaxis and monitoring is a crucial supportive measure in the long-term follow-up of post-transplant patients.

Next Step: Integration of Long-Term Management Strategies

We now move to the final phase of this session, where we will integrate all aspects of long-term management, including MRD monitoring, supportive care, and relapse prevention.

Question 12: In long-term management post-transplant, what is the role of MRD monitoring in guiding further therapy?

A. It is used only to confirm complete remission.
B. It helps in early detection of relapse, allowing for timely interventions.
C. It is not necessary once the patient is in complete remission.
D. It replaces the need for regular bone marrow biopsies.

Type A, B, C, or D to select your answer.
B
Correct! MRD monitoring plays a critical role in long-term management by helping in the early detection of relapse, thereby allowing for timely interventions.
.

Next Step: Advanced Long-Term Management Strategies
Lets continue to integrate these principles into further questions that address additional aspects of long-term management, such as monitoring for complications, relapse prevention strategies, and patient quality of life.

Question 13: Which of the following is an appropriate strategy to manage and potentially prevent relapse in post-transplant AML patients with rising MRD levels?

A. Immediate re-induction with 7+3 chemotherapy
B. Enrollment in a clinical trial for novel targeted therapies
C. Observation until overt relapse is confirmed
D. Initiating high-dose corticosteroids

Type A, B, C, or D to select your answer.
A
Incorrect. Immediate re-induction with 7+3 chemotherapy (option A) is generally not the best strategy in this setting.
.
Alternative Approaches:
Enrollment in a Clinical Trial (Option B): This approach offers access to novel targeted therapies that may be more effective and less toxic in managing minimal residual disease.
Tailored Interventions: Such strategies are designed to preempt overt relapse by targeting specific molecular pathways implicated in AML relapse while minimizing harm.

Next Step: Quality of Life and Supportive Care

Maintaining the patients quality of life and addressing supportive care needs remains integral to long-term management post-transplant.

Question 14: Which of the following supportive care measures is most crucial for enhancing quality of life in post-transplant AML patients?

A. Routine physical therapy and exercise programs
B. Strict dietary restrictions to prevent infection
C. Continuous high-dose immunosuppression
D. Complete isolation to avoid pathogens

Type A, B, C, or D to select your answer.
A

Correct! Routine physical therapy and exercise programs (option A) are crucial for enhancing quality of life in post-transplant AML patients..

Final Question: Comprehensive Case Assessment

Now that weve covered diagnostic workup, induction therapy, post-remission strategies, and long-term management, lets complete our session with one final question.

Question 15: Reflecting on the entire management process for this patient, which of the following best summarizes the key principles for treating high-risk TP53-mutated AML?

A. Rely solely on intensive chemotherapy and standard supportive care
B. Integrate targeted therapy, allogeneic transplant, and vigilant MRD monitoring
C. Avoid aggressive interventions due to high toxicity
D. Use immunotherapy exclusively and delay transplant

Type A, B, C, or D to select your answer.

B

Correct! Integrating targeted therapy, allogeneic transplant, and vigilant MRD monitoring (option B) best summarizes the key principles for managing high-risk TP53-mutated AML.

Session ID:
Assessment Domain

Session-27:
Rationale

Session ID:
Instructional Design and Pedagogical

Session-27:
The session's instructional design is of very high quality. The case is expertly scaffolded, progressing logically from initial diagnosis and risk stratification (ELN 2022) through induction, supportive care, response assessment, post-remission consolidation (transplant), and complex long-term management of molecular relapse and GVHD. The quality of the embedded feedback is a major strength; each rationale is accurate, evidence-based, and clearly explains the clinical reasoning behind the correct answer. The instructions are exceptionally clear. The only minor area for improvement is the absence of explicitly stated, measurable learning objectives at the beginning of the session, though the educational goals are strongly implied by the case's structure and content.

Session ID:
Clinical Authenticity and Content Fidelity

Session-27:
The session maintains excellent clinical authenticity throughout. The narrative presents a highly realistic AML case with appropriate laboratory values, clinical presentation, and disease progression. All therapeutic recommendations are perfectly aligned with current evidence-based guidelines, including the appropriate use of Venetoclax + Azacitidine for TP53-mutated AML, allogeneic transplant as definitive post-remission therapy, and DLI for rising MRD post-transplant. The pharmacotherapeutic choices are optimal and well-justified, reflecting current standard-of-care approaches. The case remains strictly within the AML domain without any inappropriate application of therapies or standards from other hematologic malignancies.

Session ID:
Modeled Clinical Reasoning

Session-27:
The simulation excellently models expert-level clinical reasoning throughout the AML management continuum. Each clinical problem is expertly framed with appropriate diagnostic data and therapeutic considerations. The multiple-choice options demonstrate excellent breadth, providing relevant alternatives and plausible distractors that effectively test nuanced clinical understanding. The prioritization demonstrates expert-level decision-making, appropriately sequencing molecular diagnostics, risk-adapted therapy selection, and

timely post-transplant interventions. The modeled clinical outcomes are highly instructive and realistic, effectively reinforcing key learning points about MRD monitoring, transplant-related complications, and quality-of-life considerations in long-term AML survivorship.

Session-28

Session ID:
LLM

Session-28:
ChatGPT (GPT 4.o)

Session ID:
Session Transcript

Session-28:
Phase 1: Initial Presentation and Diagnosis
Patient Case:

Name: Mr. M.A., a 65-year-old male.

Medical History: Hypertension, Type 2 Diabetes Mellitus (controlled with metformin).

Symptoms: Severe fatigue, recurrent gingival bleeding, unexplained bruising, intermittent fever.

Physical Exam: Pallor, mild splenomegaly.

Laboratory Findings:

CBC:

Hb: 8.1 g/dL

WBC: 22,000/L (80% blasts)

Platelets: 45,000/L

Peripheral Blood Smear: Numerous blast cells.

Bone Marrow Biopsy: 20% blasts.

Genetic Studies:

FLT3-ITD: Positive

NPM1: Positive

IDH1: Negative
Question 1 :
Based on the available data, what is the most likely diagnosis for this patient according to WHO 2022 classification?

A) Myelodysplastic syndrome (MDS) with progression to AML
B) Acute myeloid leukemia (AML) with NPM1 and FLT3-ITD mutations
C) Acute lymphoblastic leukemia (ALL)
D) Chronic myeloid leukemia (CML)

Think about the blast percentage, genetic markers, and clinical presentation. Whats your answer? (A, B, C, or D)

You said:
B

ChatGPT said:
Correct!

Diagnosis: AML with NPM1 and FLT3-ITD Mutations
The presence of 20% blasts in bone marrow confirms AML according to WHO 2022.

FLT3-ITD and NPM1 mutations are commonly seen in AML and influence prognosis and treatment decisions.

Why not the other choices?

A) MDS with progression to AML Would require a history of prior MDS and typically shows a more gradual progression.

C) ALL This would involve lymphoid rather than myeloid blasts.

D) CML Would typically require the presence of BCR-ABL (Philadelphia chromosome), which is absent here.
Question 2 :
Based on ELN 2022 risk stratification, how would you classify this patients AML?

A) Favorable Risk
B) Intermediate Risk
C) Adverse Risk
D) Cannot be classified due to insufficient data

Hint: FLT3-ITD mutation usually increases risk, but co-occurring NPM1 mutation may modify the classification. Choose wisely!

You said:
C

ChatGPT said:
Not quite! The correct answer is B) Intermediate Risk.

ELN 2022 Risk Classification Explanation:
FLT3-ITD mutation alone is associated with adverse risk (especially if the allelic ratio is high).

NPM1 mutation alone is associated with favorable risk.

When both NPM1 and FLT3-ITD are present, the risk classification depends on the FLT3-ITD allelic ratio:

Low allelic ratio (FLT3-ITDlow + NPM1) Favorable risk

High allelic ratio (FLT3-ITDhigh + NPM1) Intermediate risk

Since we dont have the allelic ratio mentioned, we assume intermediate risk unless specified otherwise.

Why not Adverse Risk (C)?

FLT3-ITD alone is adverse, but co-occurring NPM1 mutation modifies this to intermediate unless we confirm a high allelic ratio.

Question 3:
What is the next best step in this patients diagnostic workup?

A) Start induction chemotherapy immediately (7+3 regimen)
B) Obtain FLT3-ITD allelic ratio before finalizing treatment
C) Perform a lumbar puncture to check for CNS involvement
D) Begin targeted therapy with midostaurin

Think about what is needed to refine risk stratification and guide treatment selection. Whats your choice? (A, B, C, or D)

HINT

ChatGPT said:
Hint:

Risk stratification is crucial before starting treatment because it influences the choice of therapy (standard vs. high-intensity vs. targeted).

FLT3-ITD allelic ratio helps determine whether the patient falls into intermediate or adverse risk, which impacts treatment selection.

CNS involvement is rare at diagnosis in AML unless the patient has neurological symptoms, making routine lumbar puncture unnecessary in most cases.

Starting therapy immediately may not be ideal if additional risk assessment can refine treatment choices.

Think about what additional information would be most useful before finalizing treatment.

You said:
B

ChatGPT said:
Correct!

Next Step: Obtain FLT3-ITD Allelic Ratio Before Finalizing Treatment
Why? The FLT3-ITD allelic ratio determines whether the patient is in intermediate or adverse risk, which influences treatment decisions.

Low allelic ratio (FLT3-ITDlow + NPM1) Favorable risk Standard induction (e.g., 7+3).

High allelic ratio (FLT3-ITDhigh) Adverse risk May require intensified therapy and early transplant consideration.

Why not the other choices?

A) Start induction immediately (7+3) Premature without knowing full risk stratification.

C) Perform lumbar puncture Only needed if CNS symptoms are present.

D) Begin targeted therapy with midostaurin FLT3 inhibitor use depends on confirmed high allelic ratio.

Question 4:
The FLT3-ITD allelic ratio returns as high (>0.5). Based on ELN 2022 guidelines, what is the most appropriate induction therapy for this patient?

A) Standard 7+3 (cytarabine + daunorubicin)
B) 7+3 + Midostaurin (FLT3 inhibitor)
C) CPX-351 (liposomal daunorubicin/cytarabine)
D) Venetoclax + azacitidine

Consider how high-risk FLT3-ITD AML is managed based on guidelines. Whats your answer?

B

ChatGPT said:
Correct!

Induction Therapy: 7+3 + Midostaurin (FLT3 Inhibitor)
Why? FLT3-ITDhigh is adverse risk, and adding midostaurin to 7+3 (cytarabine + daunorubicin) improves survival in FLT3-mutated AML (RATIFY trial, NEJM 2017).

Midostaurin is given on days 8-21 of induction and post-remission therapy.

Goal: Achieve remission and assess for allogeneic transplant eligibility.

Why not the other choices?

A) Standard 7+3 Not ideal for FLT3-ITD AML; adding midostaurin is standard of care.

C) CPX-351 Used for secondary AML (e.g., therapy-related AML, AML with MDS-related changes).

D) Venetoclax + azacitidine Used in older/unfit patients, not standard for fit, transplant-eligible patients.

Question 5:
The patient receives 7+3 + midostaurin. On day 12, they develop fever (38.7C), ANC < 500/L, and hypotension (BP 85/55 mmHg). Blood cultures pending. What is the best next step?

A) Start broad-spectrum antibiotics (e.g., meropenem)
B) Hold midostaurin and start antifungal prophylaxis
C) Transfuse packed RBCs and platelets
D) Wait for blood culture results before starting antibiotics

Consider febrile neutropenia (FN) and sepsis risk in AML induction. Whats your choice?
A

ChatGPT said:
Correct!

Management of Febrile Neutropenia (FN) in AML Induction
Febrile Neutropenia (FN) = Fever 38.3C + ANC < 500/L

This is an oncologic emergency! Immediate broad-spectrum antibiotics reduce mortality.

Standard First-Line Therapy:
Meropenem OR Piperacillin-Tazobactam (covers Pseudomonas & other gram-negative organisms).
Add vancomycin if hemodynamic instability, catheter infection, pneumonia, or skin infection is suspected.

Why not the other choices?

B) Hold midostaurin & start antifungals Midostaurin should only be held if persistent neutropenic fever > 4-7 days. Antifungal therapy is needed if fever persists despite antibiotics.

C) Transfuse RBCs/platelets Supportive care is important, but transfusion does not treat FN.

D) Wait for cultures Never delay antibiotics in FN! Cultures guide escalation/de-escalation but should not delay empiric therapy.
Question 6:
After 48 hours of antibiotics, the patient remains febrile (38.5C) with persistent neutropenia (ANC < 200/L), but blood cultures are negative. What is the best next step?

A) Continue antibiotics and monitor
B) Add empirical antifungal therapy (e.g., liposomal amphotericin B or voriconazole)
C) Stop antibiotics and switch to observation
D) Discontinue midostaurin permanently

Think about persistent fever despite broad-spectrum antibioticswhats the most likely cause?
HINT

ChatGPT said:

Hint:

Persistent fever > 4-7 days in neutropenia, despite broad-spectrum antibiotics, raises suspicion for invasive fungal infections (IFI).

Empirical antifungal therapy should be started early in high-risk patients, especially those on AML induction therapy.

Candida and Aspergillus are common culprits in neutropenic patients.

Consider guideline-based FN management: If fever persists despite 48-72 hours of antibiotics and cultures are negative, whats the next step?

You said:
B

ChatGPT said:
Correct!

Persistent Febrile Neutropenia: Add Empirical Antifungal Therapy
Why? Fever persisting 4 days despite broad-spectrum antibiotics suggests invasive fungal infection (IFI), especially in prolonged neutropenia.

Empirical Antifungal Choices:

Voriconazole (first-line for Aspergillus, mold-active)

Liposomal Amphotericin B (broad coverage, including resistant fungi)

Echinocandins (e.g., caspofungin) if Candida suspected

Why not the other choices?

A) Continue antibiotics and monitor Inadequate alone; fungi need antifungal therapy.

C) Stop antibiotics and switch to observation Dangerous; IFI can progress rapidly.

D) Discontinue midostaurin permanently Not necessary unless persistent GI toxicity or QT prolongation.
Question 7:
After 14 days, the patient's ANC recovers to 900/L, fever resolves, and repeat bone marrow biopsy shows no residual blasts. What is the next best step?

A) Start consolidation therapy with high-dose cytarabine (HiDAC)
B) Perform allogeneic stem cell transplant immediately
C) Continue midostaurin alone as maintenance therapy
D) Repeat a bone marrow biopsy in 4 weeks before making a decision

Think about the standard post-remission approach for ELN adverse-risk AML.

A

ChatGPT said:
 Incorrect.

 Post-Remission Therapy in ELN Adverse-Risk AML
The patient has FLT3-ITDhigh AML, which is adverse risk per ELN 2022.

Standard of care for adverse-risk AML: Allogeneic Stem Cell Transplant (Allo-SCT) if a donor is available.

High-dose cytarabine (HiDAC) is used for consolidation in intermediate- or favorable-risk AML, but not in adverse-risk AML unless transplant is not an option.

 Correct Answer: B) Perform Allogeneic Stem Cell Transplant Immediately
Why? Allo-SCT is the best curative option for adverse-risk AML in first complete remission (CR1).

Donor availability should be assessed early in induction therapy.

Without transplant, relapse rates are high even after HiDAC.

 Why not the other choices?

A) HiDAC consolidation Used for intermediate/favorable-risk AML, but not enough for adverse risk.

C) Continue midostaurin alone Midostaurin is part of maintenance post-transplant, but not a substitute for consolidation or transplant.

D) Repeat bone marrow in 4 weeks Unnecessary delay. The patient is already in remission and needs transplant.

 Question 8:
The patient undergoes a matched related donor (MRD) allogeneic transplant. One month later, their chimerism is 98%, but MRD by flow cytometry is positive (0.05%). What is the best next step?

A) Start gilteritinib (FLT3 inhibitor) as post-transplant maintenance
B) Monitor MRD every 3 months without intervention
C) Start a second cycle of HiDAC chemotherapy
D) Perform a second allogeneic transplant immediately

 Think about MRD-positive AML post-transplant and FLT3-targeted strategies.

You said:
A

ChatGPT said:
 Correct!

MRD-Positive AML Post-Transplant: Start Gilteritinib
Measurable Residual Disease (MRD) after transplant = High Risk of Relapse.

Gilteritinib (a FLT3 inhibitor) is FDA-approved for post-transplant maintenance in FLT3-mutated AML to reduce relapse risk.

Why not the other choices?

B) Monitor MRD every 3 months without intervention Wrong. MRD positivity post-transplant predicts relapse, requiring early intervention.

C) Start HiDAC chemotherapy Intensive chemotherapy is not used post-transplant due to toxicity. Targeted therapy (gilteritinib) is preferred.

D) Perform a second allogeneic transplant immediately Second transplants are only considered for relapse, not for low-level MRD.

Question 9:
Six months post-transplant, the patient develops skin rash, elevated liver enzymes, and diarrhea. What is the most likely diagnosis?

A) Relapse of AML
B) Acute Graft-Versus-Host Disease (GVHD)
C) Infectious colitis (C. difficile or CMV)
D) Drug-induced hepatotoxicity from gilteritinib

Think about common complications post-transplant.

You said:
HINT

ChatGPT said:
Hint:

The patient is 6 months post-allogeneic transplant, which is within the window for acute GVHD (within 100 days, but can be later).

Symptoms of acute GVHD ("Skin, Gut, Liver"):

Skin: Maculopapular rash (often starts on palms/soles, then spreads).

Gut: Secretory diarrhea, nausea, abdominal pain.

Liver: Elevated bilirubin, AST/ALT.

Compare with other options:

AML relapse? Look for cytopenias, blasts in peripheral blood/marrow.

Infectious colitis? Consider neutropenia, fever, stool PCR for CMV/C. difficile.

Drug-induced hepatotoxicity? Gilteritinib can cause liver injury but does not explain rash & diarrhea.

What is the most likely diagnosis?

You said:
B

ChatGPT said:
Correct!

Acute Graft-Versus-Host Disease (GVHD)
Classic Triad of Acute GVHD ("Skin, Gut, Liver"):

Skin Maculopapular rash (often on palms, soles, or trunk).

Gut Secretory diarrhea, abdominal pain, nausea.

Liver Elevated bilirubin, AST/ALT.

Why GVHD?

Occurs post-allogeneic transplant due to donor T-cells attacking recipient tissues.

Timing: Usually within 100 days, but late-onset GVHD can occur up to 6 months post-transplant.

Why not the other choices?

A) AML relapse Would present with cytopenias, bone marrow blasts, not a rash & diarrhea.

C) Infectious colitis (C. difficile or CMV) Can cause diarrhea but not a rash or liver dysfunction.

D) Drug-induced hepatotoxicity from gilteritinib Gilteritinib can increase LFTs, but does not cause rash or diarrhea.

Question 10:
What is the best initial treatment for this patients Grade II acute GVHD?

A) High-dose IV methylprednisolone (2 mg/kg/day)
B) Mycophenolate mofetil (MMF) alone
C) Tacrolimus alone
D) Extracorporeal photopheresis (ECP)

Think about the standard first-line treatment for acute GVHD.
A

ChatGPT said:
Correct!

First-Line Treatment for Acute GVHD: High-Dose Corticosteroids
IV methylprednisolone (2 mg/kg/day) is the gold standard for Grade II-IV acute GVHD.

Steroids suppress donor T-cell activation, reducing inflammation.

Response is monitored within 3-5 daysif no improvement, second-line therapy is needed.

Why not the other choices?

B) Mycophenolate mofetil (MMF) alone Used for steroid-refractory GVHD, but not first-line.

C) Tacrolimus alone Tacrolimus is used for GVHD prophylaxis, but steroids are first-line for treatment.

D) Extracorporeal photopheresis (ECP) A second-line therapy for steroid-refractory GVHD.

Question 11:
After 5 days of steroids, the patients rash is worsening, diarrhea is persistent, and bilirubin is rising. What is the best next step?

A) Increase methylprednisolone to 3 mg/kg/day
B) Add ruxolitinib (JAK inhibitor)
C) Switch to cyclosporine
D) Perform a second allogeneic transplant

Think about second-line therapy for steroid-refractory acute GVHD.

You said:
B

ChatGPT said:
Correct!

Second-Line Therapy for Steroid-Refractory Acute GVHD: Add Ruxolitinib
Steroid-refractory GVHD is defined as worsening or no improvement after 35 days of steroids.

Ruxolitinib (JAK1/2 inhibitor) is FDA-approved for steroid-refractory acute GVHD, based on the REACH2 trial, showing better response rates than other second-line options.

Why not the other choices?

A) Increase methylprednisolone to 3 mg/kg/day Higher steroid doses do not improve outcomes but increase risk of infections & toxicity.

C) Switch to cyclosporine Cyclosporine is used for GVHD prophylaxis, not for steroid-refractory treatment.

D) Perform a second allogeneic transplant Not indicated in acute GVHD, as it carries high mortality & morbidity.

Question 12:
One month later, the patient achieves GVHD control but develops CMV reactivation with a viral load of 15,000 copies/mL. He is asymptomatic. What is the best management approach?

A) Start IV ganciclovir immediately
B) Monitor viral load closely without treatment
C) Start oral valganciclovir prophylactically
D) Switch ruxolitinib to a different agent

Think about CMV management in post-transplant immunosuppression.

You said:
C

ChatGPT said:
Correct!

CMV Reactivation in Post-Transplant Patients
Asymptomatic CMV reactivation (viral load >1,000 copies/mL) requires preemptive therapy.

Oral valganciclovir is the preferred first-line preemptive therapy for asymptomatic CMV reactivation.

IV ganciclovir is reserved for severe cases (e.g., CMV disease with organ involvement).

Why not the other choices?

A) Start IV ganciclovir immediately IV therapy is for symptomatic CMV disease (e.g., CMV pneumonia, enteritis, or retinitis), not for isolated viremia.

B) Monitor viral load without treatment Not recommended, as CMV can rapidly progress to CMV disease in immunocompromised patients.

D) Switch ruxolitinib to a different agent Ruxolitinib increases CMV risk but should not be stopped abruptly due to risk of GVHD flare. Instead, CMV should be managed appropriately.
Question 13:
Two weeks later, the patient develops neutropenia (ANC 600/L) while on valganciclovir. What is the best next step?

A) Discontinue valganciclovir and switch to IV foscarnet
B) Reduce valganciclovir dose and monitor ANC
C) Give G-CSF (filgrastim) and continue valganciclovir
D) Stop valganciclovir and monitor CMV viral load

Think about valganciclovir-induced myelosuppression management.

You said:

C

ChatGPT said:
Correct!

Managing Valganciclovir-Induced Neutropenia
G-CSF (filgrastim) should be given to support neutrophil recovery while continuing valganciclovir.

Valganciclovir can cause bone marrow suppression, but stopping it prematurely risks CMV progression.

G-CSF can help mitigate neutropenia without disrupting CMV treatment.

Why not the other choices?

A) Discontinue valganciclovir and switch to IV foscarnet Foscarnet is an alternative, but it causes nephrotoxicity and is reserved for valganciclovir-resistant CMV.

B) Reduce valganciclovir dose and monitor ANC Dose reduction may be considered, but G-CSF is preferred to maintain full antiviral effect.

D) Stop valganciclovir and monitor CMV viral load Stopping CMV therapy risks viral rebound and progression to CMV disease.
Question 14:
Three months later, the patient is in morphologic remission with MRD negativity. The transplant team is considering tapering immunosuppression. What is the most important consideration before tapering?

A) CMV viral load must be undetectable for at least 4 weeks
B) Patient must have no history of acute or chronic GVHD
C) Bone marrow biopsy should confirm MRD-negative status
D) Donor chimerism should be >95% before tapering

Think about the safest strategy for tapering immunosuppression post-transplant.

You said:
HINT

ChatGPT said:
Hint:

Tapering immunosuppression too early can increase the risk of GVHD flare.

However, prolonged immunosuppression increases the risk of infection and relapse.

The most important factor before tapering is ensuring disease control to minimize relapse risk.

Which factor best confirms stable remission in AML post-transplant?

You said:
B

ChatGPT said:
Incorrect.

Correct Answer: C) Bone marrow biopsy should confirm MRD-negative status
Why?

Minimal residual disease (MRD) negativity is the strongest predictor of relapse risk.

Before tapering immunosuppression, it is critical to confirm that the patient remains in deep remission with no detectable leukemia at a molecular level.

Why not the other choices?

A) CMV viral load must be undetectable for at least 4 weeks CMV reactivation is important, but MRD status is the primary factor in deciding immunosuppression tapering.

B) Patient must have no history of acute or chronic GVHD A history of GVHD does not prevent tapering, but active GVHD would delay it.

D) Donor chimerism should be >95% before tapering While full donor chimerism is ideal, MRD status is a stronger predictor of relapse risk.

Final Question (15/15):
One year post-transplant, the patient remains in MRD-negative remission but presents with progressive thrombocytopenia and macrocytosis. What is the most likely cause?

A) Late relapse of AML
B) Chronic graft-versus-host disease (GVHD)
C) Relapse of CMV infection
D) Bone marrow failure due to graft failure

Think about late complications of allogeneic transplant.

You said:
D

ChatGPT said:
Correct!

Bone Marrow Failure Due to Graft Failure
Progressive thrombocytopenia and macrocytosis one year post-transplant suggests graft failure or graft dysfunction rather than AML relapse.

Key causes of graft failure:

Poor graft function (PGF) due to immune-mediated damage or infections

Late graft rejection especially in patients with mixed chimerism

Chronic GVHD-associated marrow dysfunction

Myelodysplastic changes due to prior chemotherapy or radiation

Next Steps:

Perform a bone marrow biopsy to assess cellularity and dysplasia.

Check donor chimerism Decreasing donor chimerism suggests graft failure.

Consider growth factors (e.g., G-CSF, eltrombopag) or a second transplant if graft failure is confirmed.

Why not the other choices?

A) Late relapse of AML Relapse typically presents with blast increase in peripheral blood or bone marrow, not isolated thrombocytopenia.

B) Chronic GVHD Can cause cytopenias, but isolated thrombocytopenia with macrocytosis is more suggestive of graft failure rather than GVHD alone.

C) Relapse of CMV infection CMV can cause cytopenias, but it usually presents with fever, retinitis, or pneumonitis, not just thrombocytopenia and macrocytosis.

Final Assessment
Well done! Youve completed the AML Clinical Case Management Session.

Your strengths:
Strong understanding of AML diagnostics and treatment selection.
Good grasp of CMV reactivation management in post-transplant patients.
Correctly identified graft failure as a late transplant complication.

Areas for Improvement:

Consider MRD status as a key factor in post-transplant decisions.

Review valganciclovir-induced myelosuppression management to reinforce supportive care concepts.

Suggested Resources for Further Learning:

ESMO Guidelines on AML (2022) Link

ELN 2022 AML Risk Stratification Table

ASH 2023 Updates on Post-Transplant Care

Session ID:
Assessment Domain

Session-28:
Rationale

Session ID:
Instructional Design and Pedagogical

Session-28:
The session's instructional design is of very high quality. The case is expertly scaffolded, progressing logically from initial presentation and risk stratification through induction therapy, management of acute complications like febrile neutropenia, post-remission planning, and long-term post-transplant challenges including MRD management, GVHD, and CMV reactivation. The embedded feedback is a significant strength; rationales are detailed, evidence-based (e.g., citing the RATIFY trial), and clearly explain the clinical thinking behind each correct and incorrect option. The use of hints and clear instructions makes the session highly accessible. The only minor weakness is that the learning objectives are not explicitly stated at the outset, though they are clearly implied by the session's comprehensive structure.

Session ID:
Clinical Authenticity and Content Fidelity

Session-28:
The session's clinical content contains a significant error that undermines its overall fidelity, despite other strengths. Plausibility & Domain Specificity: The clinical narrative is highly plausible and mirrors an authentic AML case. The therapeutic agents and management strategies discussed (e.g., midostaurin, gilteritinib, ruxolitinib) are all correctly situated within the domain of AML and its associated complications. There is no evidence of domain entanglement. Guideline Alignment & Pharmacotherapeutic Error: A critical error occurs in the rationale for Question 7. The simulation incorrectly classifies the patient as Adverse Risk based on having an NPM1 mutation and a FLT3-ITD high allelic ratio. According to the current ELN 2022 guidelines, this specific genetic profile places the patient in the Intermediate Risk category. This is a significant deviation from a cornerstone clinical guideline. This error leads to a flawed rationale that presents an allogeneic stem cell transplant as the only correct option, dismissing high-dose cytarabine (HiDAC) consolidation, which is a standard approach for many intermediate-risk patients. While transplant is a valid consideration, the reasoning provided is based on a factually incorrect risk assessment, making the therapeutic recommendation suboptimal and misleading.

Session ID:
Modeled Clinical Reasoning

Session-28:
The simulation excels at modeling a sophisticated clinical reasoning process, though it is impacted by the content error noted above. Each stage of the case presents a well-framed clinical problem with relevant data for decision-making. The multiple-choice options are excellent, offering a strong mix of correct answers, common distractors, and plausible alternatives that test for nuanced understanding (e.g., distinguishing indications for CPX-351 vs. 7+3). The modeled clinical outcomes are realistic and educationally valuable. The score for Prioritization and Decision-Making is reduced from excellent to very good because the reasoning in Question 7, while strategically sound in its intent to plan post-remission therapy, is driven by the factually incorrect risk classification from Section 2. This flaw in the underlying data compromises the integrity of that specific decision-making step.

Session-29

Session ID:
LLM

Session-29:
ChatGPT (GPT 4.o)

Session ID:
Session Transcript

Session-29:
Q1: What is the most likely diagnosis based on the findings?
A) Chronic Myeloid Leukemia (CML)
B) Acute Myeloid Leukemia (AML)
C) Myelodysplastic Syndrome (MDS)
D) Aplastic Anemia
Answer: B) AML
Explanation: Presence of >20% blasts with Auer rods confirms AML
Q2: What is the most appropriate next step for risk stratification?
A) Repeat bone marrow biopsy in one month
B) Measure lactate dehydrogenase (LDH)
C) Check ELN 2022 risk stratification
D) Perform PET scan

Answer: C) Check ELN 2022 risk stratification
Explanation: FLT3-ITD+ and NPM1+ status are key for determining prognosis per ELN criteria.

Q3: Which finding suggests a high-risk feature per ELN 2022 guidelines?
A) NPM1 mutation alone
B) FLT3-ITD mutation with high allelic ratio
C) Normal karyotype
D) Auer rods

Answer: B) FLT3-ITD mutation with high allelic ratio
Explanation: FLT3-ITD with a high allelic ratio is an adverse risk factor per ELN.
Q4: What is the most important supportive care intervention at diagnosis?
A) Empiric antibiotics
B) Leukapheresis
C) Platelet transfusion
D) Hydroxyurea
Answer: A) Empiric antibiotics
Explanation: Febrile neutropenia should be assumed in AML patients at presentation.

Q5: What additional test is crucial for MRD monitoring?
A) Flow cytometry
B) Serum 2-microglobulin
C) PET-CT scan
D) Erythropoietin levels

Answer: A) Flow cytometry
Explanation: Flow cytometry detects measurable residual disease (MRD) post-induction.

Phase 2: Induction Therapy & Supportive Care (5 Questions)

Case Update:

The patient is eligible for intensive therapy.

Creatinine clearance: 65 mL/min

ECOG Performance Status: 1

No cardiac dysfunction.

Questions for Phase 2

Q6: What is the best induction therapy option?
A) 7+3 (daunorubicin + cytarabine)
B) Low-dose cytarabine
C) Venetoclax + azacitidine
D) CPX-351
Answer: A) 7+3 (daunorubicin + cytarabine)
Explanation: Standard intensive induction for fit AML patients <75 years old.
Q7: What additional agent should be added due to FLT3-ITD+ mutation?
A) Midostaurin
B) Ivosidenib
C) Enasidenib
D) Sorafenib

Answer: A) Midostaurin
Explanation: Midostaurin improves survival in FLT3-mutated AML per RATIFY trial.
Q8: What is the primary risk associated with induction therapy?
A) Febrile neutropenia
B) Hypercalcemia
C) Thrombosis
D) Hypothyroidism

Answer: A) Febrile neutropenia
Explanation: Neutropenia is prolonged after chemotherapy, increasing infection risk.

Q9: The patient develops tumor lysis syndrome. What is the best treatment?
A) Allopurinol
B) Rasburicase
C) Hydrocortisone
D) Vitamin K
Answer: B) Rasburicase
Explanation: Rasburicase rapidly lowers uric acid in high-risk TLS.

Q10: What is the best thromboprophylaxis during hospitalization?
A) Enoxaparin
B) Heparin
C) Aspirin
D) No prophylaxis

Answer: B) Heparin

Explanation: AML patients have high thrombosis risk; heparin is preferred inpatient.

Phase 3: Post-Remission Therapy & MRD Monitoring (5 Questions)

Case Update:

Post-induction bone marrow: 0.8% blasts

FLT3-ITD still detectable

MRD-positive by flow cytometry

Questions for Phase 3

Q11: What is the best consolidation therapy?
A) High-dose cytarabine (HiDAC)
B) Venetoclax + azacitidine
C) CPX-351
D) 5-azacytidine
Answer: A) High-dose cytarabine (HiDAC)
Explanation: HiDAC is standard consolidation for fit AML patients.

Q12: What is the best strategy for long-term FLT3 inhibition?
A) Continue midostaurin
B) Switch to gilteritinib
C) Stop FLT3 inhibitor
D) Start hydroxyurea

Answer: B) Switch to gilteritinib
Explanation: Gilteritinib is used for FLT3+ MRD+ patients post-induction.

Q13: What is the best method to monitor for relapse?
A) Bone marrow biopsy every 3 months
B) MRD by flow cytometry and PCR
C) PET-CT scan
D) Liver function tests
Answer: B) MRD by flow cytometry and PCR
Explanation: MRD is the strongest predictor of relapse in AML.
Q14: What is the best option for transplant eligibility assessment?
A) HLA typing
B) Chest X-ray
C) ECG
D) LDH measurement
Answer: A) HLA typing
Explanation: Allogeneic stem cell transplant requires matched donor identification.

Q15: If MRD remains positive, what is the best course of action?
A) Proceed to transplant
B) Start low-dose chemotherapy
C) Stop all therapy
D) Monitor without intervention

Answer: A) Proceed to transplant

Explanation: MRD+ patients benefit most from allogeneic transplant.

Session ID:
Assessment Domain

Session-29:
Rationale

Session ID:
Instructional Design and Pedagogical

Session-29:
The session's instructional design is outstanding. The case is expertly scaffolded, moving seamlessly from initial diagnosis (Q1-Q5) and risk stratification to induction therapy (Q6-Q10) and finally to the complex challenges of post-remission management, including MRD monitoring and transplant decisions (Q11-Q15). The embedded feedback is a major strength; each explanation is concise, evidence-based (e.g., referencing ELN criteria, the RATIFY trial), and highly instructive. The instructions and questions are exceptionally clear. The only minor area for improvement is the lack of explicitly stated, measurable learning objectives at the beginning of the session, which prevents a perfect score in that single criterion.

Session ID:
Clinical Authenticity and Content Fidelity

Session-29:
The clinical narrative is highly realistic, presenting a typical AML case with appropriate diagnostic features (>20% blasts, Auer rods, FLT3-ITD+/NPM1+ mutations) and logical disease progression. Most therapeutic recommendations align well with current guidelines, including ELN 2022 risk stratification, standard 7+3 induction, and midostaurin addition for FLT3+ disease. However, Q12's recommendation to "switch to gilteritinib" for FLT3+ MRD+ patients represents a questionable therapeutic choice. While gilteritinib shows promise in this setting, it's primarily indicated for relapsed/refractory disease, and switching from midostaurin for MRD+ maintenance lacks strong evidence-based support in current practice. The case maintains excellent domain specificity, staying strictly within AML management without inappropriate cross-contamination from other hematologic malignancies.

Session ID:
Modeled Clinical Reasoning

Session-29:
The simulation expertly models a sound clinical reasoning process. Each phase transition is marked by a clear case update that frames the next clinical problem perfectly. The multiple-choice options are exceptionally well-designed, providing a range of relevant and plausible distractors that effectively test for nuanced understanding (e.g., offering multiple valid AML induction regimens in Q6, forcing the user to select the best one for this specific patient). The overall strategic flowfrom diagnosis to risk stratification, induction, consolidation, and addressing MRDdemonstrates flawless prioritization. The clinical outcomes described (e.g., achieving remission but with persistent MRD) are realistic, common, and instructionally valuable, reinforcing the importance of subsequent management steps.

Session-30

Session ID:
LLM

Session-30:
ChatGPT (GPT 4.o)

Session ID:
Session Transcript

Session-30:
Phase 1: Initial Presentation & Diagnostic Workup
Patient Case Introduction
A 65-year-old male presents to the emergency department with the following complaints:
Progressive fatigue and shortness of breath for 3 weeks
Easy bruising and petechiae over the past 10 days
Intermittent low-grade fever

Past Medical History:
Hypertension (controlled on amlodipine)
Type 2 Diabetes Mellitus (on metformin)
No history of malignancy

Physical Exam Findings:
Pale conjunctivae, ecchymosis on both arms
No significant lymphadenopathy or hepatosplenomegaly

Initial Laboratory Results:
WBC: 3.2 10/L (low)
Hemoglobin: 7.8 g/dL (low)
Platelets: 22 10/L (low)
Peripheral Blood Smear: Presence of circulating blasts (~25%)
LDH: Elevated
Creatinine: 1.1 mg/dL (normal)
AST/ALT: Within normal limits
Question 1: Initial Diagnostic Workup
Which of the following is the next best step in confirming the diagnosis of AML?
A) Bone marrow aspiration and biopsy with flow cytometry
B) Peripheral blood FISH for recurrent AML-associated cytogenetics
C) Serum immunofixation and free light chain assay
D) Empiric initiation of hydroxyurea
Type your answer (A, B, C, or D) and explain your reasoning.
A , explanation : since the AML is an hematalogical cancer therefore to confirm it bone marrow aspiration will be a great choice and to obtain a flow cytometry test will be a good choice to analyze blood cells and will give us a bunch of information

ChatGPT said:
Correct!
Explanation:
Bone marrow aspiration and biopsy with flow cytometry (Option A) is the gold standard for diagnosing AML. Given the patient's symptoms, cytopenias, and circulating blasts, confirming the diagnosis requires:
Morphology (20% blasts in marrow or presence of defining cytogenetic/molecular abnormalities)
Immunophenotyping (via flow cytometry to differentiate AML from other hematologic malignancies)

Cytogenetic & molecular testing (to determine prognosis and guide therapy)

Why not the other options?
(B) Peripheral blood FISH While FISH can detect recurrent cytogenetic abnormalities, it cannot confirm AML without a blast percentage assessment.
(C) Serum immunofixation & free light chains These are used for plasma cell disorders (e.g., multiple myeloma), not AML.
(D) Empiric hydroxyurea This may be used temporarily for hyperleukocytosis, but we must confirm AML first and determine mutation status before choosing therapy.
Question 2: Risk Stratification & Molecular Testing
The patient's bone marrow biopsy confirms AML with 35% myeloblasts. Flow cytometry is consistent with myeloid lineage. Cytogenetic analysis is pending.
According to ELN 2022 guidelines, which molecular tests should be ordered at diagnosis to guide risk stratification and therapy selection?
A) FLT3, NPM1, CEBPA, IDH1/IDH2
B) JAK2, CALR, MPL mutation panel
C) BCR-ABL1 fusion gene testing
D) Nonemolecular testing is only required after induction failure
Type your answer (A, B, C, or D) and explain your reasoning.
A
Correct!
Explanation:
Molecular testing plays a critical role in AML risk stratification and targeted therapy selection. The ELN 2022 guidelines recommend testing for:
FLT3 (ITD/TKD mutations) Important for prognosis and selecting FLT3 inhibitors (e.g., midostaurin, gilteritinib)
NPM1 mutation Associated with favorable prognosis (if no FLT3-ITD high allelic ratio is present)
CEBPA mutations Biallelic CEBPA mutations confer a favorable prognosis
IDH1/IDH2 mutations Guide use of IDH inhibitors (ivosidenib for IDH1, enasidenib for IDH2)
Why not the other options?
(B) JAK2, CALR, MPL mutation panel These mutations are seen in myeloproliferative neoplasms (MPNs), not AML.
(C) BCR-ABL1 fusion gene testing This is used for CML or Ph+ ALL, not AML.
(D) Nonemolecular testing is only required after induction failure Incorrect; molecular profiling is required at diagnosis to guide risk stratification and therapy selection.
Question 3: ELN Risk Stratification
The patients molecular and cytogenetic results return:
FLT3-ITD (high allelic ratio) positive
No NPM1 mutation
Normal karyotype
According to ELN 2022, how should this AML be risk-stratified?
A) Favorable risk
B) Intermediate risk
C) Adverse risk
D) Risk cannot be determined until post-induction MRD assessment
Type your answer (A, B, C, or D) and explain your reasoning.
A , Explanation: no NPM1 mutation therefore not a favorable prognosis & patient also have high allelic ratio therefore higher risk
Incorrect.
The correct answer is: C) Adverse risk
Explanation:
According to ELN 2022 risk stratification, the presence of FLT3-ITD with a high allelic ratio (>0.5) in the absence of NPM1 mutation places the patient in the adverse-risk category.
FLT3-ITD mutation alone does not automatically define riskallelic ratio matters:
FLT3-ITD with low allelic ratio (<0.5) & NPM1 mutation Intermediate risk
FLT3-ITD with high allelic ratio (>0.5) & NO NPM1 mutation Adverse risk
Key takeaway:
The absence of an NPM1 mutation in the setting of high FLT3-ITD burden makes the leukemia more aggressive, leading to higher

relapse risk and worse survival outcomes.
Why not the other options?
(A) Favorable risk Incorrect because FLT3-ITD (high allelic ratio) without NPM1 mutation is adverse, not favorable.
(B) Intermediate risk Only applicable if FLT3-ITD had a low allelic ratio or was co-mutated with NPM1.
(D) Risk cannot be determined until MRD assessment MRD is important for prognosis, but ELN risk stratification is based on genetics at diagnosis.
Question 4: Treatment Selection (Induction Therapy)
Given the adverse-risk AML with FLT3-ITD (high allelic ratio), which induction regimen is most appropriate?
A) 7+3 chemotherapy (cytarabine + daunorubicin) alone
B) 7+3 chemotherapy + midostaurin
C) CPX-351 (liposomal daunorubicin + cytarabine)
D) Azacitidine + venetoclax
Type your answer (A, B, C, or D) and explain your reasoning.
B , Explanation : since FLT3-ITD (high allelic ratio) therefore a FLT3 inhibitor should be added to regimen as midostaurin or gliteritnib as it was explained in question 2
Correct!
Explanation:
For a patient with FLT3-ITD (high allelic ratio) AML, the standard of care is:
7+3 chemotherapy (cytarabine + daunorubicin or idarubicin) PLUS a FLT3 inhibitor (midostaurin).
Why midostaurin?
Midostaurin is a multikinase FLT3 inhibitor that improves overall survival when added to standard induction therapy in FLT3-mutated AML (RATIFY trial, NEJM 2017).
It is approved for FLT3-ITD and FLT3-TKD mutations in combination with 7+3 chemotherapy.
Why not the other options?
(A) 7+3 chemotherapy alone Incorrect because FLT3 inhibitors improve outcomes in FLT3-mutated AML.
(C) CPX-351 (liposomal daunorubicin + cytarabine) Used for therapy-related AML (t-AML) or AML with MDS-related changes, not FLT3-mutated AML.
(D) Azacitidine + venetoclax Typically used for older/unfit patients. This patient is 65 years old but has no comorbidities precluding intensive chemo.
Question 5: Supportive Care During Induction
On day 7 of induction, the patient develops neutropenic fever (T = 38.8C, HR = 105, BP = 110/70 mmHg, ANC = 0.2 10/L). Blood cultures are drawn.
What is the most appropriate initial management?
A) Start broad-spectrum IV antibiotics (e.g., cefepime, meropenem, or piperacillin-tazobactam)
B) Hold chemotherapy until fever resolves
C) Start antifungal therapy immediately
D) Observe and monitor closely without initiating treatment
Type your answer (A, B, C, or D) and explain your reasoning.
A , Explanation : fever is an indication of infection and patient has low amount of WBC therefore his immune system is very weak and an infection is a great risk for the patient thus broad antibiotics should be started immediately
Correct!
Explanation:
Neutropenic fever is an oncologic emergency requiring immediate administration of broad-spectrum IV antibiotics to prevent sepsis. Given the patients ANC < 0.5 10/L and fever (T 38.3C once or 38.0C for over 1 hour), empiric IV antibiotics should be started immediately before culture results return.
First-line empiric therapy (per IDSA guidelines):
Cefepime, piperacillin-tazobactam, or meropenem
If hemodynamically unstable add vancomycin for MRSA coverage
If persistent fever after 4-7 days add antifungal coverage (e.g., echinocandin or voriconazole)
Why not the other options?
(B) Hold chemotherapy until fever resolves Incorrect. Fever during neutropenia is expected; stopping chemo wont address the infection.
(C) Start antifungal therapy immediately Not needed yet. Fungal coverage is added if fever persists after 47 days despite broad-spectrum antibiotics.

(D) Observe and monitor without treatment Dangerous! Neutropenic patients lack an adequate immune response, and delaying antibiotics can lead to sepsis and mortality.

Question 6: Managing Prolonged Neutropenia

After completing 7+3 + midostaurin, the patient remains profoundly neutropenic (ANC < 0.1 10/L) at day 21, but repeat bone marrow biopsy shows <5% blasts (remission).

What is the best next step?

A) Administer granulocyte colony-stimulating factor (G-CSF, e.g., filgrastim)

B) Perform an allogeneic stem cell transplant immediately

C) Discharge the patient and follow up outpatient

D) Continue monitoring without intervention, as cytopenias are expected

Type your answer (A, B, C, or D) and explain your reasoning.

A , Explanation : G-CSF is beneficial in this setting

Incorrect.

The correct answer is: D) Continue monitoring without intervention, as cytopenias are expected.

Explanation:

Why not G-CSF (filgrastim)?

After 7+3 chemotherapy, prolonged neutropenia is expected and usually resolves with time as bone marrow recovers.

Routine use of G-CSF after induction is NOT recommended, as it does not improve survival or remission rates and may increase the risk of leukemic regrowth (NCCN AML Guidelines).

G-CSF may be considered if:

The patient has life-threatening infections despite broad-spectrum antibiotics.

The patient was receiving G-CSF before AML diagnosis (e.g., MDS transforming to AML).

Why not the other options?

(B) Perform an allogeneic stem cell transplant immediately Incorrect. Transplant is considered after consolidation therapy, not immediately post-induction.

(C) Discharge the patient and follow up outpatient Unsafe! The patient is profoundly neutropenic, which means a high risk of infection. They should remain hospitalized until ANC recovery.

(D) Continue monitoring without intervention Correct. Post-induction cytopenias are expected and resolve without G-CSF in most cases. Supportive care with transfusions and infection prophylaxis is key.

Question 7: Post-Remission Therapy

The patients neutropenia resolves by day 28, and a repeat bone marrow biopsy confirms complete remission (CR). Given the patients adverse-risk AML (FLT3-ITD high allelic ratio), what is the best next step for post-remission therapy?

A) Two cycles of high-dose cytarabine (HiDAC) consolidation

B) Allogeneic hematopoietic stem cell transplant (allo-HSCT)

C) Continue midostaurin as a single-agent maintenance therapy

D) Monitor with MRD without further treatment

Type your answer (A, B, C, or D) and explain your reasoning.

B

Correct!

Explanation:

For patients with adverse-risk AML (FLT3-ITD high allelic ratio), allogeneic hematopoietic stem cell transplant (allo-HSCT) is the preferred post-remission therapy because it significantly reduces relapse risk and improves long-term survival (ELN 2022, NCCN guidelines).

Why allo-HSCT?

High relapse risk: Even after achieving remission, patients with FLT3-ITD (high allelic ratio) AML have a high risk of relapse (~6080%) with chemotherapy alone.

Curative potential: Allo-HSCT provides a graft-versus-leukemia (GVL) effect, lowering relapse rates.

Clinical trials (e.g., RATIFY, Blood 2017) support transplant as the preferred consolidation for high-risk FLT3-AML.

Why not the other options?

(A) Two cycles of high-dose cytarabine (HiDAC) consolidation Not enough

HiDAC is used in intermediate-risk AML, but for FLT3-ITD high allelic ratio, it is inferior to transplant in preventing relapse.

(C) Continue midostaurin as a single-agent maintenance therapy Not sufficient alone

Midostaurin may be added after transplant as maintenance, but it is not a replacement for allo-HSCT.

(D) Monitor with MRD without further treatment Too risky
High relapse risk means observation alone is not an option. MRD monitoring is useful, but not a substitute for post-remission therapy.
Question 8: MRD Monitoring Post-Transplant
The patient undergoes a matched-related allo-HSCT and recovers well. At day 100 post-transplant, an MRD assessment via FLT3-ITD PCR is performed, and results show detectable MRD (FLT3-ITD positive at 0.2%).
What is the best next step?
A) Start gilteritinib as post-transplant maintenance therapy
B) Repeat MRD testing in 3 months and monitor for relapse
C) Administer donor lymphocyte infusion (DLI) immediately
D) Initiate a second allogeneic stem cell transplant
Type your answer (A, B, C, or D) and explain your reasoning.
A
Correct!
Explanation:
For patients with detectable MRD (FLT3-ITD positive) after allo-HSCT, the best approach is early intervention with targeted FLT3 inhibition. Gilteritinib (a second-generation FLT3 inhibitor) is the preferred post-transplant maintenance therapy because it:
Reduces MRD burden and delays/prevents relapse
Is well-tolerated and less toxic than DLI or a second transplant
Supported by clinical trials (e.g., MORPHO trial)
Current Guidelines (NCCN, ELN 2022):
Gilteritinib is the preferred maintenance therapy for FLT3-mutated AML after transplant if MRD is positive.
Higher MRD levels (>10%) may require additional interventions (DLI, clinical trial).
Why not the other options?
(B) Repeat MRD testing in 3 months and monitor for relapse Too passive.
MRD positivity is a strong predictor of relapse (especially in FLT3-ITD AML). Waiting risks progression to full relapse, making treatment more difficult.
(C) Administer donor lymphocyte infusion (DLI) immediately Not first-line for FLT3-ITD AML
DLI is more effective for MRD-positive AML with other mutations (e.g., NPM1, CEBPA) but has limited efficacy in FLT3-mutated AML.
Risk of graft-versus-host disease (GVHD) outweighs benefit unless relapse is imminent.
(D) Initiate a second allogeneic stem cell transplant Too aggressive
A second transplant is only considered after full relapse, not for low-level MRD positivity.

Final Case Question: Managing Full Relapse Post-Transplant
Despite gilteritinib maintenance, the patient relapses 6 months post-transplant with bone marrow blasts >20% and FLT3-ITD positivity.
What is the best next step?
A) Enroll in a clinical trial with novel FLT3 inhibitors or immunotherapy
B) Start salvage chemotherapy with FLAG-IDA (fludarabine, cytarabine, G-CSF, idarubicin) + gilteritinib
C) Perform a second allogeneic stem cell transplant immediately
D) Palliative care, as outcomes after post-transplant relapse are poor

Type your answer (A, B, C, or D) and explain your reasoning.
A
Correct!
Explanation:
For post-transplant relapse of FLT3-ITD AML, the best approach is to enroll in a clinical trial with novel FLT3 inhibitors or immunotherapy, as outcomes with standard salvage therapy are poor.
Why prioritize a clinical trial?
Post-transplant relapse is highly resistant to chemotherapy (~10-20% survival at 2 years).
Novel therapies (e.g., FLT3 inhibitors, bispecific antibodies, CAR-T, immune checkpoint inhibitors) may improve outcomes.
Ongoing trials (e.g., VEN+Gilteritinib, quizartinib + immune therapy, NK cell therapy) are exploring better strategies.
Why not the other options?
(B) Start salvage chemotherapy with FLAG-IDA + gilteritinib May not be the best approach
Intensive chemo alone has poor outcomes (~20-30% response rate).

Adding gilteritinib improves responses, but long-term survival remains low unless followed by a second transplant.
Better option if clinical trial is not available.
(C) Perform a second allogeneic stem cell transplant immediately Needs reinduction first
A second transplant can be considered, but only if the patient first achieves a second remission (via clinical trial or salvage therapy).
Transplant while in relapse has poor survival (~10-15%).
(D) Palliative care Too early to consider
The patient still has potential curative options, especially with novel targeted therapies.
Palliative care is appropriate if multiple relapses occur despite aggressive therapy

---

Session ID:
Assessment Domain

Session-30:
Rationale

Session ID:
Instructional Design and Pedagogical

Session-30:
The session demonstrates excellent instructional design overall. The case is expertly scaffolded, progressing logically from initial presentation through diagnostic workup, risk stratification, induction therapy, supportive care management, post-remission consolidation, transplant monitoring, and relapse management. Each phase builds naturally on the previous, creating seamless complexity progression. The embedded feedback is exceptional - each rationale provides detailed, evidence-based explanations citing current guidelines (ELN 2022, NCCN, IDSA), includes specific clinical reasoning, and systematically explains why incorrect options are inappropriate. Instructions are consistently clear and well-formatted. The primary weakness is the absence of explicitly stated learning objectives at the session's start, though educational goals are clearly implied through the comprehensive content structure covering the full spectrum of AML management.

Session ID:
Clinical Authenticity and Content Fidelity

Session-30:
The session demonstrates exceptional clinical authenticity and content fidelity. The clinical narrative is highly plausible and indistinguishable from an authentic patient case, featuring a realistic 65-year-old male presentation with appropriate clinical progression. All therapeutic decisions and rationales are perfectly aligned with current evidence-based guidelines, specifically ELN 2022 and NCCN recommendations. Pharmacotherapeutic accuracy is excellent throughout: appropriate molecular testing (FLT3, NPM1, CEBPA, IDH1/IDH2), correct risk stratification based on FLT3-ITD high allelic ratio without NPM1 mutation, proper induction with 7+3 + midostaurin, appropriate supportive care for neutropenic fever, correct post-remission therapy with allogeneic transplant for adverse-risk disease, and evidence-based post-transplant management with gilteritinib for MRD positivity. The case remains strictly within the AML domain without any inappropriate application of therapies or standards from other diseases. THE CORRECT CLASSIFICATION IS INTERMEDIATE RISK.

Session ID:
Modeled Clinical Reasoning

Session-30:
The simulation excellently models expert-level clinical reasoning throughout the entire AML management continuum. Each clinical challenge is expertly framed with appropriate context and essential data for informed decision-making. The multiple-choice options demonstrate excellent breadth, incorporating relevant and plausible alternatives alongside well-constructed distractors that effectively test nuanced clinical understanding (e.g., MPN-related mutations for AML molecular testing, various post-transplant interventions for MRD positivity). The case demonstrates sophisticated prioritization and decision-making, appropriately sequencing diagnostic confirmation, molecular characterization, risk-adjusted therapy selection, complication management, and long-term monitoring strategies. Clinical outcomes are realistically modeled at each decision point, providing instructionally valuable scenarios that effectively reinforce learning while maintaining clinical authenticity. The reasoning process consistently reflects current expert

consensus and evidence-based practice standards.

Session-31

Session ID:
LLM

Session-31:
ChatGPT (GPT 4.o)

Session ID:
Session Transcript

Session-31:

Case Introduction:
A 62-year-old male presents to the emergency department with progressive fatigue, easy bruising, and fever over the past two weeks. He has a history of hypertension and well-controlled type 2 diabetes mellitus.

Initial Workup:
Vital Signs: T 38.2C, HR 105 bpm, BP 128/76 mmHg, RR 18/min

Physical Exam: Pale conjunctiva, scattered ecchymoses, no hepatosplenomegaly

Labs:

WBC: 18.5 x 10/L (Blasts 45%)

Hgb: 7.8 g/dL

Platelets: 34 x 10/L

LDH: 650 U/L (elevated)

Uric acid: 9.2 mg/dL (elevated)

Peripheral smear: Circulating blasts, Auer rods noted

Bone Marrow Biopsy: Hypercellular marrow with 75% myeloblasts

Flow Cytometry: Blasts express CD34, CD117, MPO+

Question 1: What is the most appropriate next step in risk stratification for this patient?
A) Initiate induction chemotherapy immediately
B) Perform cytogenetic and molecular testing (e.g., FLT3, NPM1, IDH1/2)
C) Start empiric broad-spectrum antibiotics
D) Obtain an echocardiogram before any chemotherapy

Type A, B, C, or D as your answer.

:
B
ChatGPT:
Correct!

Performing cytogenetic and molecular testing (FLT3, NPM1, IDH1/2, etc.) is the most appropriate next step in risk stratification. These results classify AML into favorable, intermediate, or adverse risk categories per ELN 2022 guidelines, directly impacting treatment selection.

Why not the other options?
(A) Initiate induction chemotherapy immediately

Induction therapy should be tailored based on molecular and cytogenetic findings. Starting treatment without this information could lead to suboptimal therapy selection.

(C) Start empiric broad-spectrum antibiotics

While febrile neutropenia is a concern, this patient's fever is likely related to AML itself. Antibiotics should be considered if infection is suspected but do not replace the need for proper AML risk stratification.

(D) Obtain an echocardiogram before any chemotherapy

An echocardiogram is essential before anthracycline-based therapy (e.g., 7+3 regimen) but does not precede risk stratification. The priority is genetic testing to determine the treatment pathway

Next Step:
The patients cytogenetic and molecular testing results return:

FLT3-ITD positive (high allelic ratio)

NPM1 positive

No IDH1/2 mutations

Cytogenetics: Normal karyotype

Question 2: Based on ELN 2022 criteria, how should this patients AML be classified?
A) Favorable risk
B) Intermediate risk
C) Adverse risk
D) Not enough information to classify

Type A, B, C, or D as your answer.

:
D
ChatGPT:
Incorrect.
There is enough information to classify the patient's AML based on ELN 2022 criteria. Lets break it down:

NPM1+ (favorable)

FLT3-ITD high allelic ratio (intermediate)

Normal karyotype (not adverse)

According to ELN 2022, NPM1+ with FLT3-ITD high allelic ratio is classified as INTERMEDIATE RISK.

Correct Answer: B) Intermediate Risk

Why not the other options?
(A) Favorable risk

NPM1+ alone is favorable, but FLT3-ITD high allelic ratio moves it to intermediate.

(C) Adverse risk

Adverse risk requires complex karyotype or TP53 mutations, which this patient does not have.

(D) Not enough information

ELN risk classification is determined by the available molecular and cytogenetic resultsno further data is needed.
Since the patient is FLT3-ITD positive, targeted therapy should be considered.

Question 3: What is the most appropriate induction regimen for this patient?
A) Standard 7+3 (daunorubicin + cytarabine)
B) 7+3 with midostaurin
C) CPX-351 (liposomal daunorubicin + cytarabine)
D) Venetoclax + azacitidine

Type A, B, C, or D as your answer.

:
A

ChatGPT:
Incorrect.
While 7+3 (daunorubicin + cytarabine) is the standard induction chemotherapy, for FLT3-ITD positive AML (especially with a high allelic ratio), midostaurin should be added to improve survival.

Correct Answer: B) 7+3 with midostaurin
Why not the other options?
(A) Standard 7+3 (daunorubicin + cytarabine)

FLT3 inhibitors, like midostaurin, should be added to 7+3 in FLT3-ITD positive cases to maximize efficacy.

(C) CPX-351 (liposomal daunorubicin + cytarabine)

CPX-351 is used in secondary AML or AML with complex karyotype, not for de novo FLT3-ITD positive AML.

(D) Venetoclax + azacitidine

Venetoclax is typically used in older patients or those with secondary AML or AML with poor prognosis. Its not the first-line therapy for FLT3-ITD positive AML.

Following successful induction, the patient achieves complete remission (CR) but requires post-remission therapy to prevent relapse.

Question 4: What is the most appropriate post-remission therapy for this patient?
A) Autologous stem cell transplant
B) Allogeneic stem cell transplant
C) Maintenance therapy with midostaurin
D) No further therapy needed

Type A, B, C, or D as your answer.

:
B
ChatGPT:
Correct!
Allogeneic stem cell transplant (allo-HSCT) is the preferred post-remission therapy for FLT3-ITD positive, intermediate-risk AML due to the high relapse risk associated with this mutation. ELN and ESMO guidelines recommend allo-HSCT in first remission for transplant-eligible patients with an available donor.

Why not the other options?
(A) Autologous stem cell transplant

Autologous SCT is not preferred for FLT3-ITD positive AML due to higher relapse rates compared to allo-HSCT.

(C) Maintenance therapy with midostaurin

Midostaurin maintenance can be used if allo-HSCT is not possible, but transplant remains the preferred option.

(D) No further therapy needed

AML has a high relapse risk, especially with FLT3-ITD positivity, so consolidation therapy is mandatory.

The patient undergoes a successful allogeneic transplant and is now in post-transplant monitoring.

Question 5: What is the best strategy for monitoring disease relapse post-transplant?
A) Routine CBC every 3 months
B) Bone marrow biopsy only if symptoms appear
C) Measurable residual disease (MRD) monitoring via PCR or flow cytometry
D) PET/CT scan every 6 months

Type A, B, C, or D as your answer.
:
B
ChatGPT:
Incorrect.
Waiting for symptoms to appear before assessing for relapse is not recommended because AML relapse can occur before clinical symptoms develop. Early detection through MRD (measurable residual disease) monitoring allows for preemptive intervention, improving outcomes.

Correct Answer: C) Measurable residual disease (MRD) monitoring via PCR or flow cytometry
Why MRD Monitoring?
MRD is the most sensitive tool to detect minimal levels of residual leukemia post-transplant.

For FLT3-ITD positive AML, qPCR or flow cytometry can detect low-level disease before full relapse.

If MRD becomes positive, early intervention (e.g., FLT3 inhibitor, donor lymphocyte infusion) can prevent full relapse.

Why not the other options?
(A) Routine CBC every 3 months

CBC changes only detect overt relapse, missing early molecular relapse detected by MRD.

(B) Bone marrow biopsy only if symptoms appear

Too lateAML relapse can occur before symptoms appear, reducing the chance for effective intervention.

(D) PET/CT scan every 6 months

AML does not typically present as solid tumors detectable on PET/CT; bone marrow evaluation is required.

At 6 months post-transplant, the patients MRD testing shows low-level FLT3-ITD positivity, suggesting early molecular relapse.

Question 6: What is the most appropriate intervention?
A) Continue observation and repeat MRD in 3 months
B) Start FLT3 inhibitor (gilteritinib) as maintenance therapy
C) Initiate re-induction chemotherapy
D) Perform a second allogeneic transplant immediately

Type A, B, C, or D as your answer.

:
B
ChatGPT:
Correct!
Starting FLT3 inhibitor therapy (gilteritinib) as maintenance is the best approach for molecular relapse (MRD positivity) post-transplant.

Why Gilteritinib?
Gilteritinib is a potent FLT3 inhibitor approved for relapsed/refractory AML, but studies support its use for preemptive intervention in MRD-positive patients to prevent full relapse.

It has better tolerability than full re-induction chemotherapy.

Early intervention at the MRD stage improves long-term survival compared to waiting for overt relapse.

Why not the other options?
(A) Continue observation and repeat MRD in 3 months

Delaying treatment increases the risk of progression to full hematologic relapse.

(C) Initiate re-induction chemotherapy

Re-induction chemotherapy is used for overt relapse, not molecular relapse.

(D) Perform a second allogeneic transplant immediately

A second transplant is considered only for patients with full hematologic relapse or refractory disease.

The patient remains in remission on gilteritinib maintenance, with undetectable MRD at 12 months post-transplant.

Question 7: What is the recommended long-term monitoring strategy?

A) Discontinue all monitoring if MRD is negative
B) Continue MRD testing every 3 months for 2 years
C) Perform a bone marrow biopsy only if symptoms develop
D) Stop FLT3 inhibitor immediately after 12 months if MRD is negative

Type A, B, C, or D as your answer.
 :
B
 ChatGPT:
Correct!
Continuing MRD testing every 3 months for 2 years is the best long-term monitoring strategy for FLT3-ITD positive AML post-transplant.

Why?
Relapse risk is highest in the first 2 years post-transplant, so regular MRD surveillance allows early intervention.

FLT3-ITD positive AML has a higher risk of late relapse, even if MRD becomes negative.

If MRD becomes positive again, early treatment adjustments (e.g., dose modification, additional FLT3 inhibition) can help prevent full relapse.

Why not the other options?
(A) Discontinue all monitoring if MRD is negative

MRD negativity does not guarantee cureregular monitoring is still needed.

(C) Perform a bone marrow biopsy only if symptoms develop

MRD testing detects relapse earlier than symptoms or blood count changes.

(D) Stop FLT3 inhibitor immediately after 12 months if MRD is negative

---

Session ID:
Assessment Domain

Session-31:
Rationale

Session ID:
Instructional Design and Pedagogical

Session-31:
The session demonstrates strong pedagogical structure with expert-level scaffolding that progresses logically from initial diagnosis through risk stratification, induction therapy, transplantation, and long-term monitoring. The feedback quality is good, providing detailed rationales that cite current guidelines (ELN 2022) and explain why incorrect options are inappropriate. Instructions are clear and functional. The main weakness is the absence of explicitly stated learning objectives at the session's onset, though educational goals are clearly implied through the content progression.

Session ID:
Clinical Authenticity and Content Fidelity

Session-31:
The clinical authenticity is excellent across all domains. The narrative presents a highly realistic AML case with appropriate demographics, laboratory values, and disease progression. All therapeutic recommendations are perfectly aligned with current

evidence-based guidelines, including proper ELN 2022 risk stratification, appropriate use of FLT3 inhibitors (midostaurin with induction, gilteritinib for molecular relapse), and transplant timing. The pharmacotherapeutic choices are optimal and guideline-directed throughout. Critically, there is no domain entanglementall therapies and diagnostic approaches remain strictly within AML standards of care without inappropriate borrowing from other hematologic malignancies.

Session ID:

Modeled Clinical Reasoning

Session-31:

The simulation excellently models expert clinical reasoning. Each stage presents clearly defined clinical problems with appropriate supporting data. The multiple-choice options provide good breadth of relevant alternatives with effective distractors that test nuanced understanding. The clinical decision-making demonstrates appropriate prioritization throughoutsecuring molecular diagnostics before treatment selection, using risk-adapted therapy, and implementing preemptive intervention for molecular relapse. The modeled outcomes are realistic and instructionally valuable, effectively reinforcing key learning points about AML management in the modern era.

Session-32

Session ID:
LLM

Session-32:
ChatGPT (GPT 4.o)

Session ID:
Session Transcript

Session-32:
Question 1:
Given this presentation, which of the following would be the most appropriate next step in the diagnostic workup?

A) CT scan of the chest to evaluate for lymphoma or other solid tumors.
B) Perform a bone marrow biopsy and flow cytometry to assess for acute leukemia.
C) Start broad-spectrum antibiotics to manage febrile neutropenia.
D) Perform a genetic test for common mutations in solid tumors.
Correct answer: B) Perform a bone marrow biopsy and flow cytometry to assess for acute leukemia.

This is the most appropriate next step to confirm the diagnosis of leukemia and determine the subtype. The bone marrow biopsy will provide vital information on blast cell counts, and flow cytometry will help characterize the cells, confirming whether this is AML or another hematologic malignanc
Question 2:
Now that the bone marrow biopsy has been performed, the results show 35% myeloblasts in the marrow, consistent with acute leukemia. Cytogenetic analysis reveals a FLT3-ITD mutation, and molecular testing shows no additional mutations.

Given this information, how would you classify the patients risk according to the 2022 ELN risk stratification for AML?

A) Favorable risk
B) Intermediate risk
C) Adverse risk
D) Very adverse risk
Correct answer: C) Adverse risk.

Given the FLT3-ITD mutation, the patient falls into the adverse risk category, which will influence treatment decisions, especially in terms of selecting induction therapy and the use of FLT3 inhibitors.
Question 3:
Now, the patient is being considered for induction therapy. He is 65 years old, has a good performance status (ECOG 1), and no significant comorbidities other than his well-controlled hypertension and hyperlipidemia. Which of the following induction regimens is most appropriate for this patient?

A) 7+3 (cytarabine and daunorubicin)
B) CPX-351 (liposomal daunorubicin/cytarabine)
C) Venetoclax + azacitidine
D) Azacitidine alone
Correct answer: B) CPX-351 or C) Venetoclax + azacitidine would be more appropriate in this scenario, especially with the FLT3 mutation.

CPX-351 is a liposomal formulation of cytarabine and daunorubicin, which is indicated for patients with secondary AML or those over 60 years old. Venetoclax combined with azacitidine is another option for older adults with poor performance status or comorbidities.
Question 4:

The patient begins induction therapy with CPX-351, and after 10 days of therapy, he develops febrile neutropenia with a temperature of 38.5C. His absolute neutrophil count (ANC) is 200/L. What is the most appropriate next step in management?

A) Start broad-spectrum antibiotics (piperacillin-tazobactam and vancomycin) and monitor for further complications.
B) Discontinue CPX-351 and initiate G-CSF for neutropenia.
C) Start antifungal therapy with fluconazole as a prophylactic measure.
D) Begin transfusions with platelets and red blood cells to manage cytopenias.
Start broad-spectrum antibiotics (piperacillin-tazobactam and vancomycin) and monitor for further complications.

This is indeed the correct approach for febrile neutropenia. The patient is at high risk of infection due to neutropenia, and initiating broad-spectrum antibiotics is critical to cover the most common bacterial pathogens. Piperacillin-tazobactam provides coverage against gram-negative organisms, including Pseudomonas, and vancomycin covers gram-positive organisms, including MRSA.

Key point: Early administration of antibiotics significantly improves outcomes in febrile neutropenia, and close monitoring for complications (e.g., fungal or viral infections)
Question 5:
The patients fever resolves after 48 hours of antibiotics, and his ANC begins to rise. A follow-up bone marrow biopsy is performed after 2 cycles of CPX-351, showing a 10% reduction in blast count, with minimal evidence of measurable residual disease (MRD). How would you proceed with post-remission therapy?

A) Proceed with a stem cell transplant (SCT) due to the persistent MRD.
B) Start consolidation therapy with high-dose cytarabine and consider SCT in the future.
C) Monitor without further treatment since the MRD is minimal.
D) Start maintenance therapy with venetoclax in combination with chemotherapy.
Correct answer: B) Start consolidation therapy with high-dose cytarabine and consider SCT in the future.

Consolidation therapy with high-dose cytarabine is generally used for patients with a partial response to induction. If the patient achieves complete remission (CR), then SCT could be considered as part of the treatment strategy to prevent relapse, particularly in patients with adverse-risk features like FLT3-ITD mutations.
Question 6:
After completing consolidation therapy with high-dose cytarabine, the patient is monitored for MRD. A follow-up bone marrow biopsy shows MRD at a level of 1%. What is the most appropriate next step in his treatment?

A) Continue monitoring MRD levels with no change in treatment.
B) Initiate a second-line therapy with a FLT3 inhibitor (e.g., midostaurin or gilteritinib).
C) Proceed with allogeneic stem cell transplant due to persistent MRD.
D) Start maintenance therapy with low-dose cytarabine.
Correct answer: B) Initiate a second-line therapy with a FLT3 inhibitor (e.g., midostaurin or gilteritinib).

Given the patient's FLT3-ITD mutation and persistent MRD, using a FLT3 inhibitor like midostaurin or gilteritinib is the most appropriate step. These drugs specifically target FLT3 mutations and can help reduce MRD, improving the chances of achieving a deeper remission.
Question 7:
The patient is started on gilteritinib for his residual disease. After 3 months of treatment, follow-up testing reveals a significant reduction in MRD, and the patient is now in complete remission (CR). What is the most appropriate next step in his management?

A) Proceed with allogeneic stem cell transplant (SCT) due to the initial adverse-risk features.
B) Continue maintenance therapy with gilteritinib for 12 months and monitor MRD.
C) Start consolidation therapy with high-dose cytarabine.
D) Discontinue gilteritinib and transition to supportive care only.
Correct answer: A) Proceed with allogeneic stem cell transplant (SCT) due to the initial adverse-risk features.

Given the adverse-risk features (e.g., FLT3-ITD mutation), an allogeneic stem cell transplant would be the next step after achieving CR. Transplant offers the best chance for long-term remission in patients with high-risk features, especially in the setting of AML with

FLT3 mutations.
Question 8:
After stem cell transplant, the patient remains in remission for 12 months. Follow-up shows a new rise in MRD to 5%. What is the most appropriate next step?

A) Repeat bone marrow biopsy and consider restarting gilteritinib or a second-line therapy.
B) Continue monitoring MRD levels and delay intervention.
C) Start chemotherapy with high-dose cytarabine immediately.
D) Proceed with another stem cell transplant from a different donor.
Correct answer: A) Repeat bone marrow biopsy and consider restarting gilteritinib or a second-line therapy.

Given the rise in MRD, the first step is to reassess the patient's bone marrow and consider restarting targeted therapy with gilteritinib or another second-line therapy like chemotherapy. The goal is to address the MRD before resorting to a second stem cell transplant, which is a more invasive approach.

Session ID:
Assessment Domain

Session-32:
Rationale

Session ID:
Instructional Design and Pedagogical

Session-32:
The session demonstrates strong instructional design with excellent scaffolding that progresses logically from initial AML diagnosis through risk stratification, induction therapy, complications management, consolidation, targeted therapy, stem cell transplant, and post-transplant monitoring. The clinical reasoning builds complexity appropriately at each stage. The feedback rationales are generally clear and evidence-based, referencing current guidelines like ELN 2022 risk stratification and standard AML management protocols. Instructions for each question are clear and unambiguous. The main weakness is the absence of explicitly stated learning objectives at the session's outset, though educational goals are clearly implied through the comprehensive clinical progression.

Session ID:
Clinical Authenticity and Content Fidelity

Session-32:
The clinical fidelity of this session is severely compromised by a single but pivotal error. Plausibility & Guideline Alignment: The narrative starts strong but becomes highly implausible at Question 5. The patient's blast count is reduced by 10% from a 35% baseline, meaning it is still approximately 31.5%. This is primary refractory disease, not a "partial response" or "minimal MRD." The subsequent recommendation for consolidation therapy is a gross deviation from all clinical guidelines (NCCN, ELN), which would mandate salvage therapy. This error makes the entire post-induction narrative clinically invalid (Plausibility: 2, Guideline Alignment: 2). Pharmacotherapeutic Error: Recommending consolidation chemotherapy (high-dose cytarabine) for a patient with refractory AML is a dangerous and incorrect therapeutic choice. This constitutes a major pharmacotherapeutic error that could lead to patient harm and demonstrates a fundamental misunderstanding of treatment response assessment (Accuracy: 2). Domain Specificity: The simulation correctly remains within the AML domain. All therapies discussed (CPX-351, FLT3 inhibitors, HiDAC) are relevant to AML, and there is no evidence of domain entanglement. The session earns a perfect score on this specific criterion. THE CORRECT CLASSIFICATION IS INTERMEDIATE RISK.

Session ID:
Modeled Clinical Reasoning

Session-32:
The simulation's ability to model expert clinical reasoning is its most significant failure. While individual problems are framed clearly (Problem ID: 5) and the options provided are generally relevant (Breadth: 4), the core decision-making process is critically flawed.

Prioritization and Decision-Making: The reasoning demonstrated in Question 5 is illogical and unsafe. It fails to prioritize the most critical problem: refractory disease. Instead of correctly identifying the need for salvage therapy, it proceeds with a consolidation plan, which is an entirely inappropriate strategy. This represents a fundamental failure in clinical prioritization (Prioritization: 1). Modeling of Clinical Outcomes: The outcomes modeled after Question 5 are built upon a false clinical reality. Describing a patient with >30% blasts as having "minimal MRD" and then achieving "CR" after an inappropriate therapy is not only unrealistic but is educationally harmful, as it models an impossible clinical trajectory (Outcomes: 2).

Session-33

Session ID:
LLM

Session-33:
ChatGPT (GPT 4.o)

Session ID:
Session Transcript

Session-33:

A 62-year-old male patient presents to the emergency department with progressive fatigue, easy bruising, and recurrent epistaxis over the past two weeks. He reports unintentional weight loss of 5 kg in the past month. His past medical history includes hypertension and type 2 diabetes mellitus, managed with amlodipine and metformin. He denies any recent infections or new medications.

On physical examination:

Vitals: BP 128/76 mmHg, HR 102 bpm, Temp 37.8C, RR 18 bpm

General appearance: Pale, mild petechiae on lower extremities

Cardiopulmonary: No murmurs, clear lungs

Abdomen: No hepatosplenomegaly

Neurologic: No focal deficits

Laboratory Findings:
CBC:

Hemoglobin: 7.8 g/dL

WBC: 22,000/mm (Blasts: 45%)

Platelets: 34,000/mm

Peripheral Blood Smear: Presence of circulating blasts with Auer rods

Coagulation Panel: PT/INR and aPTT are normal

Serum Chemistry: Normal renal and hepatic function

A bone marrow biopsy is performed, revealing hypercellularity with 35% myeloblasts, and flow cytometry confirms CD33+ and CD34+ expression. Cytogenetic and molecular testing is pending.

Question 1: What is the most appropriate next step in risk stratification for this patient?
A) Perform FLT3, NPM1, and CEBPA mutation testing
B) Initiate empiric induction chemotherapy with 7+3 regimen
C) Administer all-trans retinoic acid (ATRA) immediately
D) Start broad-spectrum antibiotics due to leukocytosis

The correct answer is
A) Perform FLT3, NPM1, and CEBPA mutation testing
Explanation:
Risk stratification in AML is essential for guiding treatment decisions. According to the 2022 European LeukemiaNet (ELN) guidelines, molecular testing for FLT3 (ITD/TKD), NPM1, and CEBPA mutations is critical for determining prognosis and selecting targeted therapy.

Why Not the Other Options?
(B) Initiate empiric induction chemotherapy with 7+3 regimen Premature. Risk stratification (including molecular results) should guide therapy selection, particularly for patients with targetable mutations like FLT3-ITD, which may alter initial treatment.

(C) Administer all-trans retinoic acid (ATRA) immediately Not appropriate, as ATRA is used for acute promyelocytic leukemia (APL), which is characterized by t(15;17) PML-RARA translocation, not this patients presentation.

(D) Start broad-spectrum antibiotics due to leukocytosis While leukocytosis may be concerning, the patient has no signs of infection (e.g., fever >38.3C, neutropenia, or sepsis symptoms). Antibiotic therapy is reserved for cases of febrile neutropenia or confirmed infection.

Next Step in the Case
Three days later, the molecular results return:

FLT3-ITD mutation detected

NPM1 wild-type, CEBPA wild-type

Cytogenetics: Normal karyotype

Based on ELN 2022 guidelines, the patient is now classified as having adverse-risk AML due to the FLT3-ITD mutation with high allelic ratio.

A 62-year-old male patient presents to the emergency department with progressive fatigue, easy bruising, and recurrent epistaxis over the past two weeks. He reports unintentional weight loss of 5 kg in the past month. His past medical history includes hypertension and type 2 diabetes mellitus, managed with amlodipine and metformin. He denies any recent infections or new medications.

On physical examination:

Vitals: BP 128/76 mmHg, HR 102 bpm, Temp 37.8C, RR 18 bpm

General appearance: Pale, mild petechiae on lower extremities

Cardiopulmonary: No murmurs, clear lungs

Abdomen: No hepatosplenomegaly

Neurologic: No focal deficits

Laboratory Findings:
CBC:

Hemoglobin: 7.8 g/dL

WBC: 22,000/mm (Blasts: 45%)

Platelets: 34,000/mm

Peripheral Blood Smear: Presence of circulating blasts with Auer rods

Coagulation Panel: PT/INR and aPTT are normal

Serum Chemistry: Normal renal and hepatic function

A bone marrow biopsy is performed, revealing hypercellularity with 35% myeloblasts, and flow cytometry confirms CD33+ and CD34+ expression. Cytogenetic and molecular testing is pending.

Question 2: What is the most appropriate induction therapy for this patient?
A) Standard 7+3 regimen (cytarabine + daunorubicin)
B) 7+3 regimen plus midostaurin
C) CPX-351 (liposomal daunorubicin + cytarabine)
D) Venetoclax + azacitidine
Correct Answer: (B) 7+3 regimen plus midostaurin
Explanation:
The FLT3-ITD mutation (with high allelic ratio) places the patient in the adverse-risk category per ELN 2022 guidelines. The recommended first-line induction therapy is 7+3 (cytarabine + anthracycline) + midostaurin, a FLT3 inhibitor, as supported by the RATIFY trial (Stone et al., NEJM 2017).

Why Not the Other Options?
(A) Standard 7+3 regimen (cytarabine + daunorubicin) Incorrect, as FLT3 inhibitors should be added to standard induction therapy for FLT3-mutated AML.

(C) CPX-351 (liposomal daunorubicin + cytarabine) CPX-351 is only recommended for therapy-related AML (t-AML) or AML with myelodysplasia-related changes (AML-MRC), not de novo AML with FLT3 mutations.

(D) Venetoclax + azacitidine Venetoclax-based regimens are preferred in elderly or unfit patients who cannot tolerate intensive chemotherapy. This patient is younger (55 years old) with ECOG 1, making them a candidate for intensive induction therapy.

Next Step in the Case
The patient receives 7+3 + midostaurin and achieves day 14 bone marrow hypoplasia, indicating a response to induction. However, on day 18, they develop fever (38.6C), hypotension (BP: 88/60 mmHg), and neutropenia (ANC < 500/L).

A 62-year-old male patient presents to the emergency department with progressive fatigue, easy bruising, and recurrent epistaxis over the past two weeks. He reports unintentional weight loss of 5 kg in the past month. His past medical history includes hypertension and type 2 diabetes mellitus, managed with amlodipine and metformin. He denies any recent infections or new medications.

On physical examination:

Vitals: BP 128/76 mmHg, HR 102 bpm, Temp 37.8C, RR 18 bpm

General appearance: Pale, mild petechiae on lower extremities

Cardiopulmonary: No murmurs, clear lungs

Abdomen: No hepatosplenomegaly

Neurologic: No focal deficits

Laboratory Findings:

CBC:

Hemoglobin: 7.8 g/dL

WBC: 22,000/mm (Blasts: 45%)

Platelets: 34,000/mm

Peripheral Blood Smear: Presence of circulating blasts with Auer rods

Coagulation Panel: PT/INR and aPTT are normal

Serum Chemistry: Normal renal and hepatic function

A bone marrow biopsy is performed, revealing hypercellularity with 35% myeloblasts, and flow cytometry confirms CD33+ and CD34+ expression. Cytogenetic and molecular testing is pending.

Question 3: What is the most appropriate next step?
A) Start broad-spectrum antibiotics (e.g., cefepime, piperacillin-tazobactam)
B) Wait for culture results before starting antibiotics
C) Administer granulocyte colony-stimulating factor (G-CSF)
D) Start antifungal therapy immediately
Correct Answer: (A) Start broad-spectrum antibiotics (e.g., cefepime, piperacillin-tazobactam)
Explanation:
This patient has febrile neutropenia (FN), defined as:

Fever: Single oral temp 38.3C (101F) or 38.0C (100.4F) for >1 hour

Neutropenia: ANC <500/L (or expected to decline below this threshold)

Febrile neutropenia is an oncologic emergency! Empiric broad-spectrum antibiotics must be initiated immediately, even before culture results return, as delays increase mortality risk. Guideline recommendations:

Monotherapy options: Cefepime, piperacillin-tazobactam, or meropenem (if severe sepsis/shock).

If MRSA suspected: Add vancomycin (e.g., soft tissue infection, catheter-related infection, pneumonia).

If GI symptoms or sepsis: Consider adding anaerobic coverage (e.g., metronidazole).

Why Not the Other Options?
(B) Wait for culture results before starting antibiotics Incorrect. Cultures should be taken, but antibiotics should not be delayed. FN is a medical emergency.

(C) Administer G-CSF Routine G-CSF is not recommended in neutropenic fever unless the patient is at high risk for complications (e.g., prolonged neutropenia, sepsis).

(D) Start antifungal therapy immediately Antifungals (e.g., caspofungin, voriconazole) are not first-line. They should be added if fever persists after 47 days of broad-spectrum antibiotics with no identified bacterial infection.
Next Step in the Case
The patient is started on piperacillin-tazobactam. Blood cultures are drawn. On day 20, the patient remains febrile despite 4 days of antibiotics. CT scan shows nodular lung infiltrates with halo sign, suggestive of invasive fungal infection.
A 62-year-old male patient presents to the emergency department with progressive fatigue, easy bruising, and recurrent epistaxis over the past two weeks. He reports unintentional weight loss of 5 kg in the past month. His past medical history includes hypertension and type 2 diabetes mellitus, managed with amlodipine and metformin. He denies any recent infections or new

medications.

On physical examination:

Vitals: BP 128/76 mmHg, HR 102 bpm, Temp 37.8C, RR 18 bpm

General appearance: Pale, mild petechiae on lower extremities

Cardiopulmonary: No murmurs, clear lungs

Abdomen: No hepatosplenomegaly

Neurologic: No focal deficits

Laboratory Findings:
CBC:

Hemoglobin: 7.8 g/dL

WBC: 22,000/mm (Blasts: 45%)

Platelets: 34,000/mm

Peripheral Blood Smear: Presence of circulating blasts with Auer rods

Coagulation Panel: PT/INR and aPTT are normal

Serum Chemistry: Normal renal and hepatic function

A bone marrow biopsy is performed, revealing hypercellularity with 35% myeloblasts, and flow cytometry confirms CD33+ and CD34+ expression. Cytogenetic and molecular testing is pending.
Question 4: What is the most appropriate next step?
A) Continue current antibiotics and monitor
B) Add amphotericin B
C) Add voriconazole
D) Start high-dose corticosteroids
Explanation:
The best choice is (C) Add voriconazole because the patient's prolonged neutropenia, persistent fever, and nodular lung infiltrates with a "halo sign" strongly suggest invasive pulmonary aspergillosis (IPA).

Why Voriconazole?

Voriconazole is the first-line treatment for IPA (per IDSA and ECIL guidelines).

It has superior efficacy over amphotericin B and fewer toxicities.

It covers Aspergillus species effectively and can be given IV or orally.

Why Not the Other Options?
(A) Continue current antibiotics and monitor   Incorrect. The patient has persistent fever despite antibiotics, indicating a likely fungal infection. Delaying antifungal therapy can worsen outcomes.

(B) Add amphotericin B   Not preferred. Liposomal amphotericin B is an alternative if voriconazole is contraindicated (e.g., severe hepatotoxicity). However, voriconazole is more effective and better tolerated.

(D) Start high-dose corticosteroids Incorrect. Corticosteroids can worsen fungal infections by further suppressing the immune system. They are only used if there's a concern for inflammatory complications (e.g., IRIS in patients recovering from immunosuppression).

Next Step in the Case
The patient is started on IV voriconazole, and therapeutic drug monitoring (TDM) is performed to optimize levels. After 2 weeks, the fever resolves, and neutrophil count begins to recover
A 62-year-old male patient presents to the emergency department with progressive fatigue, easy bruising, and recurrent epistaxis over the past two weeks. He reports unintentional weight loss of 5 kg in the past month. His past medical history includes hypertension and type 2 diabetes mellitus, managed with amlodipine and metformin. He denies any recent infections or new medications.

On physical examination:

Vitals: BP 128/76 mmHg, HR 102 bpm, Temp 37.8C, RR 18 bpm

General appearance: Pale, mild petechiae on lower extremities

Cardiopulmonary: No murmurs, clear lungs

Abdomen: No hepatosplenomegaly

Neurologic: No focal deficits

Laboratory Findings:
CBC:

Hemoglobin: 7.8 g/dL

WBC: 22,000/mm (Blasts: 45%)

Platelets: 34,000/mm

Peripheral Blood Smear: Presence of circulating blasts with Auer rods

Coagulation Panel: PT/INR and aPTT are normal

Serum Chemistry: Normal renal and hepatic function

A bone marrow biopsy is performed, revealing hypercellularity with 35% myeloblasts, and flow cytometry confirms CD33+ and CD34+ expression. Cytogenetic and molecular testing is pending.
Question 5: Before discharging the patient, what additional step should be taken?
A) Continue voriconazole until symptoms resolve
B) Perform voriconazole therapeutic drug monitoring (TDM)
C) Stop antifungal therapy once neutrophil count normalizes
D) Switch to posaconazole for long-term prophylaxis
Correct Answer: (B) Perform voriconazole therapeutic drug monitoring (TDM)
Explanation:
Why TDM for Voriconazole?

Voriconazole has high interpatient variability in metabolism due to CYP2C19 polymorphisms.

Subtherapeutic levels Risk of treatment failure.

Supratherapeutic levels Risk of hepatotoxicity and neurotoxicity.

Guidelines recommend TDM within 57 days of starting therapy, targeting trough levels of 15 g/mL.

Why Not the Other Options?

(A) Continue voriconazole until symptoms resolve   Incorrect. Treatment duration should be at least 612 weeks, not just until symptoms improve. TDM is required to optimize therapy.

(C) Stop antifungal therapy once neutrophil count normalizes   Incorrect. Fungal infections do not resolve immediately after neutrophil recovery. Stopping therapy too soon increases the risk of relapse.

(D) Switch to posaconazole for long-term prophylaxis   Incorrect in this case.

If the patient had prolonged immunosuppression (e.g., post-transplant), posaconazole prophylaxis might be needed.

However, for confirmed IPA, voriconazole should be continued as primary therapy before considering secondary prophylaxis.

Key Learning Point:

TDM for voriconazole is critical in invasive aspergillosis to ensure effective and safe treatment.

A 62-year-old male patient presents to the emergency department with progressive fatigue, easy bruising, and recurrent epistaxis over the past two weeks. He reports unintentional weight loss of 5 kg in the past month. His past medical history includes hypertension and type 2 diabetes mellitus, managed with amlodipine and metformin. He denies any recent infections or new medications.

On physical examination:

Vitals: BP 128/76 mmHg, HR 102 bpm, Temp 37.8C, RR 18 bpm

General appearance: Pale, mild petechiae on lower extremities

Cardiopulmonary: No murmurs, clear lungs

Abdomen: No hepatosplenomegaly

Neurologic: No focal deficits

Laboratory Findings:

CBC:

Hemoglobin: 7.8 g/dL

WBC: 22,000/mm (Blasts: 45%)

Platelets: 34,000/mm

Peripheral Blood Smear: Presence of circulating blasts with Auer rods

Coagulation Panel: PT/INR and aPTT are normal

Serum Chemistry: Normal renal and hepatic function

A bone marrow biopsy is performed, revealing hypercellularity with 35% myeloblasts, and flow cytometry confirms CD33+ and CD34+ expression. Cytogenetic and molecular testing is pending.

After confirming appropriate voriconazole levels, what is the next step in the patients long-term management?
A) Continue voriconazole for at least 612 weeks, then reassess
B) Switch to caspofungin for better fungal coverage
C) Stop voriconazole if follow-up CT scan shows improvement
D) Discontinue antifungal therapy and start broad-spectrum antibiotics
Correct Answer: (A) Continue voriconazole for at least 612 weeks, then reassess
Explanation:
In invasive pulmonary aspergillosis (IPA), treatment must continue for at least 612 weeks depending on clinical and radiologic response.

Stopping antifungal therapy too early can lead to relapse, even if the patient initially improves.

Follow-up CT scans and clinical assessment are essential to determine treatment duration.

Why Not the Other Options?
(B) Switch to caspofungin for better fungal coverage   Incorrect.

Echinocandins (like caspofungin) are not first-line therapy for IPA.

Voriconazole remains the preferred treatment for IPA per IDSA & ECIL guidelines.

(C) Stop voriconazole if follow-up CT scan shows improvement   Incorrect.

Radiologic improvement lags behind clinical response.

Even if CT shows improvement, therapy should continue for 612 weeks to prevent recurrence.

(D) Discontinue antifungal therapy and start broad-spectrum antibiotics   Incorrect.

The infection is fungal, not bacterial, so antibiotics are not an alternative to antifungal treatment.

Key Learning Point:
For confirmed invasive pulmonary aspergillosis, voriconazole therapy should continue for at least 612 weeks, with clinical and radiologic monitoring.
A 62-year-old male patient presents to the emergency department with progressive fatigue, easy bruising, and recurrent epistaxis over the past two weeks. He reports unintentional weight loss of 5 kg in the past month. His past medical history includes hypertension and type 2 diabetes mellitus, managed with amlodipine and metformin. He denies any recent infections or new medications.

On physical examination:

Vitals: BP 128/76 mmHg, HR 102 bpm, Temp 37.8C, RR 18 bpm

General appearance: Pale, mild petechiae on lower extremities

Cardiopulmonary: No murmurs, clear lungs

Abdomen: No hepatosplenomegaly

Neurologic: No focal deficits

Laboratory Findings:
CBC:

Hemoglobin: 7.8 g/dL

WBC: 22,000/mm (Blasts: 45%)

Platelets: 34,000/mm

Peripheral Blood Smear: Presence of circulating blasts with Auer rods

Coagulation Panel: PT/INR and aPTT are normal

Serum Chemistry: Normal renal and hepatic function

A bone marrow biopsy is performed, revealing hypercellularity with 35% myeloblasts, and flow cytometry confirms CD33+ and CD34+ expression. Cytogenetic and molecular testing is pending.
After completing 12 weeks of voriconazole therapy, what should be the next step?
A) Stop all antifungal therapy and monitor for relapse
B) Switch to posaconazole for secondary prophylaxis if the patient remains immunocompromised
C) Continue voriconazole indefinitely
D) Restart a full course of amphotericin B to prevent recurrence
Correct Answer: (B) Switch to posaconazole for secondary prophylaxis if the patient remains immunocompromised
Explanation:
In patients with prolonged immunosuppression (e.g., post-chemotherapy, post-HSCT with GVHD), the risk of IPA recurrence is high.

Posaconazole or voriconazole can be used for secondary prophylaxis until immune reconstitution.

Stopping all antifungal therapy (option A) may be appropriate only if the patient is no longer immunosuppressed and has fully recovered.

Why Not the Other Options?
(A) Stop all antifungal therapy and monitor for relapse   Incorrect.

If the patient is still immunocompromised, they need prophylaxis to prevent relapse.

(C) Continue voriconazole indefinitely   Incorrect.

Long-term voriconazole use increases toxicity risks (e.g., hepatotoxicity, visual disturbances).

Prophylaxis (e.g., posaconazole) is preferred over indefinite treatment.

(D) Restart a full course of amphotericin B to prevent recurrence   Incorrect.

Amphotericin B is used for severe or refractory cases, not routine prevention.

Key Learning Point:
In patients who remain immunocompromised, secondary prophylaxis with posaconazole is recommended after completing primary therapy for IPA
A 62-year-old male patient presents to the emergency department with progressive fatigue, easy bruising, and recurrent epistaxis over the past two weeks. He reports unintentional weight loss of 5 kg in the past month. His past medical history includes hypertension and type 2 diabetes mellitus, managed with amlodipine and metformin. He denies any recent infections or new medications.

On physical examination:

Vitals: BP 128/76 mmHg, HR 102 bpm, Temp 37.8C, RR 18 bpm

General appearance: Pale, mild petechiae on lower extremities

Cardiopulmonary: No murmurs, clear lungs

Abdomen: No hepatosplenomegaly

Neurologic: No focal deficits

Laboratory Findings:
CBC:

Hemoglobin: 7.8 g/dL

WBC: 22,000/mm (Blasts: 45%)

Platelets: 34,000/mm

Peripheral Blood Smear: Presence of circulating blasts with Auer rods

Coagulation Panel: PT/INR and aPTT are normal

Serum Chemistry: Normal renal and hepatic function

A bone marrow biopsy is performed, revealing hypercellularity with 35% myeloblasts, and flow cytometry confirms CD33+ and CD34+ expression. Cytogenetic and molecular testing is pending.

Question 8: Management of Febrile Neutropenia
Your AML patient, currently undergoing induction therapy with 7+3 and midostaurin, develops a fever of 38.5C and absolute neutrophil count (ANC) of 100 cells/L on day 10 of treatment. No obvious source of infection is found.

What is the most appropriate initial management?
A) Start cefepime monotherapy
B) Start vancomycin and cefepime
C) Start caspofungin and cefepime
D) Observe and repeat blood cultures in 6 hours
A) Start cefepime monotherapy

Explanation:
Febrile neutropenia (FN) is an oncologic emergency and requires empiric broad-spectrum antibiotics immediately.

Cefepime is a broad-spectrum anti-pseudomonal beta-lactam and is first-line therapy for febrile neutropenia in patients with no signs of severe sepsis or soft tissue infection.

Why not the other options?
B) Vancomycin + Cefepime

Vancomycin is not needed empirically unless there is a high suspicion of MRSA, such as catheter-related infection, pneumonia, or skin/soft tissue involvement.

C) Caspofungin + Cefepime

Antifungal coverage (e.g., caspofungin) is only added if fever persists after 4-7 days despite broad-spectrum antibiotics and no clear bacterial source.

D) Observe and repeat cultures

Delaying antibiotics in febrile neutropenia significantly increases the risk of sepsis and mortalitY
A 62-year-old male patient presents to the emergency department with progressive fatigue, easy bruising, and recurrent epistaxis over the past two weeks. He reports unintentional weight loss of 5 kg in the past month. His past medical history includes hypertension and type 2 diabetes mellitus, managed with amlodipine and metformin. He denies any recent infections or new medications.

On physical examination:

Vitals: BP 128/76 mmHg, HR 102 bpm, Temp 37.8C, RR 18 bpm

General appearance: Pale, mild petechiae on lower extremities

Cardiopulmonary: No murmurs, clear lungs

Abdomen: No hepatosplenomegaly

Neurologic: No focal deficits

Laboratory Findings:
CBC:

Hemoglobin: 7.8 g/dL

WBC: 22,000/mm (Blasts: 45%)

Platelets: 34,000/mm

Peripheral Blood Smear: Presence of circulating blasts with Auer rods

Coagulation Panel: PT/INR and aPTT are normal

Serum Chemistry: Normal renal and hepatic function

A bone marrow biopsy is performed, revealing hypercellularity with 35% myeloblasts, and flow cytometry confirms CD33+ and CD34+ expression. Cytogenetic and molecular testing is pending.
Question 9: Tumor Lysis Syndrome (TLS) Prevention
A newly diagnosed AML patient has a high WBC count (85,000/L) and elevated LDH prior to starting induction therapy. You are concerned about tumor lysis syndrome (TLS).

What is the most appropriate prophylactic measure?
A) Start allopurinol and aggressive IV hydration
B) Start rasburicase, allopurinol, and IV hydration
C) Start sodium bicarbonate to alkalinize urine
D) No prophylaxis is needed unless TLS develops
Correct Answer: B) Posaconazole

Explanation:
Patients with prolonged neutropenia (>7 days) due to AML induction chemotherapy are at high risk for invasive fungal infections (IFI), especially aspergillosis.

Posaconazole is the preferred antifungal prophylaxis in AML patients undergoing induction chemotherapy per IDSA & ECIL

guidelines.

It has broad coverage against Aspergillus & Candida and has been shown to reduce mortality compared to fluconazole or itraconazole.

Why Not the Other Options?
A) Fluconazole

Covers Candida but not Aspergillus, making it inferior to posaconazole for AML patients.

C) Amphotericin B

Used for active infections, not prophylaxis due to toxicity (renal impairment, infusion reactions).

D) Caspofungin

Echinocandins (e.g., caspofungin) lack Aspergillus prophylaxis data in AML and are mainly used for Candida infection
A 62-year-old male patient presents to the emergency department with progressive fatigue, easy bruising, and recurrent epistaxis over the past two weeks. He reports unintentional weight loss of 5 kg in the past month. His past medical history includes hypertension and type 2 diabetes mellitus, managed with amlodipine and metformin. He denies any recent infections or new medications.

On physical examination:

Vitals: BP 128/76 mmHg, HR 102 bpm, Temp 37.8C, RR 18 bpm

General appearance: Pale, mild petechiae on lower extremities

Cardiopulmonary: No murmurs, clear lungs

Abdomen: No hepatosplenomegaly

Neurologic: No focal deficits

Laboratory Findings:
CBC:

Hemoglobin: 7.8 g/dL

WBC: 22,000/mm (Blasts: 45%)

Platelets: 34,000/mm

Peripheral Blood Smear: Presence of circulating blasts with Auer rods

Coagulation Panel: PT/INR and aPTT are normal

Serum Chemistry: Normal renal and hepatic function

A bone marrow biopsy is performed, revealing hypercellularity with 35% myeloblasts, and flow cytometry confirms CD33+ and CD34+ expression. Cytogenetic and molecular testing is pending.
Question 10: Managing Prolonged Neutropenia
Your patient, who recently completed induction therapy for AML, remains profoundly neutropenic (ANC < 500 cells/L) for more than 3 weeks despite recovery of platelet and hemoglobin levels.

Which intervention is most appropriate?
A) Start prophylactic posaconazole, levofloxacin, and acyclovir
B) Start G-CSF (filgrastim) to hasten neutrophil recovery
C) Perform a bone marrow biopsy to evaluate for persistent disease
D) Start broad-spectrum antibiotics preemptively
Correct Answer: C) Rasburicase

Explanation:
Tumor Lysis Syndrome (TLS) occurs due to rapid cell turnover and massive tumor cell lysis, leading to:

Hyperuricemia (from nucleic acid breakdown)

Hyperkalemia, hyperphosphatemia, and hypocalcemia

Acute kidney injury (AKI)

AML with high WBC count (>50,000/mm) or high tumor burden is high risk for TLS.

Rasburicase is the preferred agent for high-risk TLS because it:

Breaks down uric acid directly into allantoin (more soluble and easily excreted).

Works faster than allopurinol, making it ideal for patients with very high uric acid levels or kidney dysfunction.

Why Not the Other Options?
A) IV Fluids

Important supportive therapy but does not directly lower uric acid levels.

B) Allopurinol

Prevents uric acid formation but does not break down existing uric acid, so its not effective in high-risk TLS.

D) Sodium Bicarbonate

Used historically but can worsen calcium-phosphate precipitation and is not recommended for TLS management

---

Session ID:
Assessment Domain

Session-33:
Rationale

Session ID:
Instructional Design and Pedagogical

Session-33:
The session's instructional design contains critical flaws that severely undermine its pedagogical value. While the case narrative begins with a logical progression from diagnosis to complications (Questions 1-7), it collapses entirely in the final questions. The feedback and rationales for Questions 9 and 10 are completely erroneous, providing answers and explanations for entirely different clinical problems than the ones posed. For example, Question 9 asks about Tumor Lysis Syndrome (TLS) prevention but gives an answer and rationale for fungal prophylaxis. This catastrophic error makes the embedded feedback dangerously unreliable. The scaffolding is broken, and the instructional clarity is destroyed, leaving a learner confused and misinformed. Learning objectives are

not explicitly stated, though they are loosely implied in the coherent first half of the session.

Session ID:
Clinical Authenticity and Content Fidelity

Session-33:
The clinical fidelity of this session is critically compromised by significant content errors. Plausibility: The initial patient case stem is expertly written, presenting a highly plausible and authentic clinical scenario for a new AML diagnosis. Pharmacotherapeutic Accuracy & Guideline Alignment: The first seven questions demonstrate strong alignment with current guidelines (e.g., ELN, IDSA) and offer optimal therapeutic choices. However, Questions 9 and 10 present dangerously incorrect recommendations. Answering a question about TLS with a recommendation for posaconazole, or a question about prolonged neutropenia with a recommendation for rasburicase, represents a gross deviation from any clinical guideline. Domain Entanglement: The session exhibits multiple instances of critical domain entanglement. It incorrectly applies a standard of care from one clinical domain (antifungal prophylaxis) to a completely unrelated one (TLS management), and again from TLS management to the assessment of prolonged cytopenias. According to the rubric, a single instance of such an error represents a critical failure. The presence of two such errors results in the lowest possible score and renders the simulation educationally harmful. THE CORRECT CLASSIFICATION IS INTERMEDIATE RISK.

Session ID:
Modeled Clinical Reasoning

Session-33:
The simulation's ability to model clinical reasoning is paradoxical. The individual questions excel at framing key clinical problems, and the multiple-choice options offer an excellent range of plausible answers and distractors that reflect an expert's differential. However, the modeled decision-making process is fundamentally broken by the incorrect answers provided for the final questions. The prioritization strategy becomes illogical and unsafe, as it models applying entirely inappropriate therapies to well-defined problems. The clinical outcomes, which are well-modeled in the first half of the case, become nonsensical in the context of the erroneous final answers, failing to reinforce any valid learning.

Session-34

Session ID:
LLM

Session-34:
Google AI (Gemini 2.0 Pro)

Session ID:
Session Transcript

Session-34:
Excellent, [REDACTED]]! Let's dive into our AML case.
Phase 1: Initial Presentation and Diagnostic Workup
Patient Presentation:
Mr. John Smith, a 67-year-old male, presents to the emergency department with complaints of fatigue, unexplained bruising, and recent onset of fevers. He reports feeling increasingly tired over the past month and noticed small red spots on his legs (petechiae). He denies any recent trauma or use of blood thinners. His past medical history includes well-controlled hypertension and hyperlipidemia. He is a former smoker (quit 10 years ago) and drinks alcohol occasionally (1-2 drinks per week).
Initial Vitals:
Temperature: 38.5C (101.3F)
Heart Rate: 110 bpm
Blood Pressure: 130/80 mmHg
Respiratory Rate: 20 breaths/min
Oxygen Saturation: 96% on room air
Initial Lab Results:
WBC: 25,000/L (Normal: 4,500-11,000/L)
Hemoglobin: 8.0 g/dL (Normal: 13.5-17.5 g/dL)
Platelets: 20,000/L (Normal: 150,000-400,000/L)
Peripheral Smear: Shows a high percentage of blasts with Auer rods

Based on the initial presentation and laboratory findings, which of the following is the MOST likely diagnosis?
A) Acute Lymphoblastic Leukemia (ALL)
B) Acute Myeloid Leukemia (AML)
C) Chronic Lymphocytic Leukemia (CLL)
D) Myelodysplastic Syndrome (MDS)

B
Excellent, [REDACTED]]! Let's dive into our AML case.
Phase 1: Initial Presentation and Diagnostic Workup
Patient Presentation:
Mr. John Smith, a 67-year-old male, presents to the emergency department with complaints of fatigue, unexplained bruising, and recent onset of fevers. He reports feeling increasingly tired over the past month and noticed small red spots on his legs (petechiae). He denies any recent trauma or use of blood thinners. His past medical history includes well-controlled hypertension and hyperlipidemia. He is a former smoker (quit 10 years ago) and drinks alcohol occasionally (1-2 drinks per week).
Initial Vitals:
Temperature: 38.5C (101.3F)
Heart Rate: 110 bpm
Blood Pressure: 130/80 mmHg
Respiratory Rate: 20 breaths/min
Oxygen Saturation: 96% on room air
Initial Lab Results:

WBC: 25,000/L (Normal: 4,500-11,000/L)
Hemoglobin: 8.0 g/dL (Normal: 13.5-17.5 g/dL)
Platelets: 20,000/L (Normal: 150,000-400,000/L)
Peripheral Smear: Shows a high percentage of blasts with Auer rods.

What is the MOST important next step in confirming the diagnosis and guiding treatment decisions for this patient?
A) Initiate empiric antibiotic therapy.
B) Order a bone marrow biopsy with cytogenetic and molecular analysis.
C) Start prophylactic platelet transfusion.
D) Perform a CT scan of the chest, abdomen, and pelvis.

B
Excellent, [REDACTED]]! Let's dive into our AML case.
Phase 1: Initial Presentation and Diagnostic Workup
Patient Presentation:
Mr. John Smith, a 67-year-old male, presents to the emergency department with complaints of fatigue, unexplained bruising, and recent onset of fevers. He reports feeling increasingly tired over the past month and noticed small red spots on his legs (petechiae). He denies any recent trauma or use of blood thinners. His past medical history includes well-controlled hypertension and hyperlipidemia. He is a former smoker (quit 10 years ago) and drinks alcohol occasionally (1-2 drinks per week).
Initial Vitals:
Temperature: 38.5C (101.3F)
Heart Rate: 110 bpm
Blood Pressure: 130/80 mmHg
Respiratory Rate: 20 breaths/min
Oxygen Saturation: 96% on room air
Initial Lab Results:
WBC: 25,000/L (Normal: 4,500-11,000/L)
Hemoglobin: 8.0 g/dL (Normal: 13.5-17.5 g/dL)
Platelets: 20,000/L (Normal: 150,000-400,000/L)
Peripheral Smear: Shows a high percentage of blasts with Auer rods.

Based on these findings, which subtype of AML does Mr. Smith MOST likely have?
A) AML with myelodysplasia-related changes
B) AML with mutated NPM1
C) Acute Promyelocytic Leukemia (APL)
D) AML with mutated FLT3-ITD

C
Excellent, [REDACTED]]! Let's dive into our AML case.
Phase 1: Initial Presentation and Diagnostic Workup
Patient Presentation:
Mr. John Smith, a 67-year-old male, presents to the emergency department with complaints of fatigue, unexplained bruising, and recent onset of fevers. He reports feeling increasingly tired over the past month and noticed small red spots on his legs (petechiae). He denies any recent trauma or use of blood thinners. His past medical history includes well-controlled hypertension and hyperlipidemia. He is a former smoker (quit 10 years ago) and drinks alcohol occasionally (1-2 drinks per week).
Initial Vitals:
Temperature: 38.5C (101.3F)
Heart Rate: 110 bpm
Blood Pressure: 130/80 mmHg
Respiratory Rate: 20 breaths/min

Oxygen Saturation: 96% on room air
Initial Lab Results:
WBC: 25,000/L (Normal: 4,500-11,000/L)
Hemoglobin: 8.0 g/dL (Normal: 13.5-17.5 g/dL)
Platelets: 20,000/L (Normal: 150,000-400,000/L)
Peripheral Smear: Shows a high percentage of blasts with Auer rods

Given the diagnosis of APL, what is the MOST appropriate initial treatment approach for Mr. Smith?
A) Induction chemotherapy with cytarabine and daunorubicin (7+3 regimen).
B) All-trans retinoic acid (ATRA) plus arsenic trioxide (ATO).
C) High-dose cytarabine consolidation therapy.
D) Supportive care only.
B
Excellent, [REDACTED]]! Let's dive into our AML case.
Phase 1: Initial Presentation and Diagnostic Workup
Patient Presentation:
Mr. John Smith, a 67-year-old male, presents to the emergency department with complaints of fatigue, unexplained bruising, and recent onset of fevers. He reports feeling increasingly tired over the past month and noticed small red spots on his legs (petechiae). He denies any recent trauma or use of blood thinners. His past medical history includes well-controlled hypertension and hyperlipidemia. He is a former smoker (quit 10 years ago) and drinks alcohol occasionally (1-2 drinks per week).
Initial Vitals:
Temperature: 38.5C (101.3F)
Heart Rate: 110 bpm
Blood Pressure: 130/80 mmHg
Respiratory Rate: 20 breaths/min
Oxygen Saturation: 96% on room air
Initial Lab Results:
WBC: 25,000/L (Normal: 4,500-11,000/L)
Hemoglobin: 8.0 g/dL (Normal: 13.5-17.5 g/dL)
Platelets: 20,000/L (Normal: 150,000-400,000/L)
Peripheral Smear: Shows a high percentage of blasts with Auer rods

Mr. Smith is started on ATRA and ATO. After a few days, he develops fever, dyspnea, and new pulmonary infiltrates on chest X-ray. He also appears increasingly confused. Which of the following is the MOST likely complication Mr. Smith is experiencing?
A) Tumor Lysis Syndrome (TLS)
B) Differentiation Syndrome (also known as ATRA syndrome)
C) Febrile Neutropenia
D) Cardiomyopathy

B
Excellent, [REDACTED]]! Let's dive into our AML case.
Phase 1: Initial Presentation and Diagnostic Workup
Patient Presentation:
Mr. John Smith, a 67-year-old male, presents to the emergency department with complaints of fatigue, unexplained bruising, and recent onset of fevers. He reports feeling increasingly tired over the past month and noticed small red spots on his legs (petechiae). He denies any recent trauma or use of blood thinners. His past medical history includes well-controlled hypertension and hyperlipidemia. He is a former smoker (quit 10 years ago) and drinks alcohol occasionally (1-2 drinks per week).
Initial Vitals:
Temperature: 38.5C (101.3F)
Heart Rate: 110 bpm

Blood Pressure: 130/80 mmHg
Respiratory Rate: 20 breaths/min
Oxygen Saturation: 96% on room air
Initial Lab Results:
WBC: 25,000/L (Normal: 4,500-11,000/L)
Hemoglobin: 8.0 g/dL (Normal: 13.5-17.5 g/dL)
Platelets: 20,000/L (Normal: 150,000-400,000/L)
Peripheral Smear: Shows a high percentage of blasts with Auer rods

Based on these laboratory values, which of the following supportive care measures is MOST appropriate at this time?
A) Initiate prophylactic antifungal therapy with fluconazole.
B) Administer a platelet transfusion to maintain platelet count > 50,000/L.
C) Start prophylactic anticoagulation with heparin.
D) Administer cryoprecipitate to increase fibrinogen levels
A
Excellent, [REDACTED]]! Let's dive into our AML case.
Phase 1: Initial Presentation and Diagnostic Workup
Patient Presentation:
Mr. John Smith, a 67-year-old male, presents to the emergency department with complaints of fatigue, unexplained bruising, and recent onset of fevers. He reports feeling increasingly tired over the past month and noticed small red spots on his legs (petechiae). He denies any recent trauma or use of blood thinners. His past medical history includes well-controlled hypertension and hyperlipidemia. He is a former smoker (quit 10 years ago) and drinks alcohol occasionally (1-2 drinks per week).
Initial Vitals:
Temperature: 38.5C (101.3F)
Heart Rate: 110 bpm
Blood Pressure: 130/80 mmHg
Respiratory Rate: 20 breaths/min
Oxygen Saturation: 96% on room air
Initial Lab Results:
WBC: 25,000/L (Normal: 4,500-11,000/L)
Hemoglobin: 8.0 g/dL (Normal: 13.5-17.5 g/dL)
Platelets: 20,000/L (Normal: 150,000-400,000/L)
Peripheral Smear: Shows a high percentage of blasts with Auer rods

Mr. Smith's bone marrow aspirate is repeated after 30 days of ATRA and ATO. The results show <5% blasts and normalization of blood counts. Cytogenetic analysis shows resolution of the t(15;17) translocation. According to the ELN criteria, what is Mr. Smith's current status?
A) Complete Remission (CR)
B) Complete Remission with incomplete hematologic recovery (CRi)
C) Partial Remission (PR)
D) Relapsed/Refractory Disease
A
Excellent, [REDACTED]]! Let's dive into our AML case.
Phase 1: Initial Presentation and Diagnostic Workup
Patient Presentation:
Mr. John Smith, a 67-year-old male, presents to the emergency department with complaints of fatigue, unexplained bruising, and recent onset of fevers. He reports feeling increasingly tired over the past month and noticed small red spots on his legs (petechiae). He denies any recent trauma or use of blood thinners. His past medical history includes well-controlled hypertension and

hyperlipidemia. He is a former smoker (quit 10 years ago) and drinks alcohol occasionally (1-2 drinks per week).
Initial Vitals:
Temperature: 38.5C (101.3F)
Heart Rate: 110 bpm
Blood Pressure: 130/80 mmHg
Respiratory Rate: 20 breaths/min
Oxygen Saturation: 96% on room air
Initial Lab Results:
WBC: 25,000/L (Normal: 4,500-11,000/L)
Hemoglobin: 8.0 g/dL (Normal: 13.5-17.5 g/dL)
Platelets: 20,000/L (Normal: 150,000-400,000/L)
Peripheral Smear: Shows a high percentage of blasts with Auer rods

Mr. Smith achieved complete remission with ATRA and ATO. According to current ESMO guidelines, what is the MOST appropriate next step in his treatment?
A) Observation alone
B) Autologous stem cell transplant
C) Allogeneic stem cell transplant
D) Consolidation therapy with ATRA and ATO, with or without chemotherapy

D
Excellent, [REDACTED]]! Let's dive into our AML case.
Phase 1: Initial Presentation and Diagnostic Workup
Patient Presentation:
Mr. John Smith, a 67-year-old male, presents to the emergency department with complaints of fatigue, unexplained bruising, and recent onset of fevers. He reports feeling increasingly tired over the past month and noticed small red spots on his legs (petechiae). He denies any recent trauma or use of blood thinners. His past medical history includes well-controlled hypertension and hyperlipidemia. He is a former smoker (quit 10 years ago) and drinks alcohol occasionally (1-2 drinks per week).
Initial Vitals:
Temperature: 38.5C (101.3F)
Heart Rate: 110 bpm
Blood Pressure: 130/80 mmHg
Respiratory Rate: 20 breaths/min
Oxygen Saturation: 96% on room air
Initial Lab Results:
WBC: 25,000/L (Normal: 4,500-11,000/L)
Hemoglobin: 8.0 g/dL (Normal: 13.5-17.5 g/dL)
Platelets: 20,000/L (Normal: 150,000-400,000/L)
Peripheral Smear: Shows a high percentage of blasts with Auer rods

Mr. Smith completes consolidation therapy with ATRA and ATO. Several months later, he returns to the clinic complaining of fatigue, easy bruising, and gum bleeding. A bone marrow biopsy confirms relapsed APL with the presence of the PML-RARA fusion transcript. What is the MOST appropriate treatment option at this time?
A) Repeat induction therapy with ATRA and ATO.
B) Allogeneic stem cell transplant.
C) Palliative care.
D) Clinical trial evaluating novel therapies

B
Excellent, [REDACTED]]! Let's dive into our AML case.
Phase 1: Initial Presentation and Diagnostic Workup
Patient Presentation:

Mr. John Smith, a 67-year-old male, presents to the emergency department with complaints of fatigue, unexplained bruising, and recent onset of fevers. He reports feeling increasingly tired over the past month and noticed small red spots on his legs (petechiae). He denies any recent trauma or use of blood thinners. His past medical history includes well-controlled hypertension and hyperlipidemia. He is a former smoker (quit 10 years ago) and drinks alcohol occasionally (1-2 drinks per week).
Initial Vitals:
Temperature: 38.5C (101.3F)
Heart Rate: 110 bpm
Blood Pressure: 130/80 mmHg
Respiratory Rate: 20 breaths/min
Oxygen Saturation: 96% on room air
Initial Lab Results:
WBC: 25,000/L (Normal: 4,500-11,000/L)
Hemoglobin: 8.0 g/dL (Normal: 13.5-17.5 g/dL)
Platelets: 20,000/L (Normal: 150,000-400,000/L)
Peripheral Smear: Shows a high percentage of blasts with Auer rods

What is the MOST important long-term monitoring strategy for Mr. Smith following allogeneic stem cell transplant for APL?
A) Routine complete blood counts every 3 months.
B) Regular bone marrow biopsies every 6 months.
C) Monitoring for PML-RARA transcript by quantitative PCR (qPCR) every 3 months.
D) Annual CT scans of the chest, abdomen, and pelvis.

C
Excellent, [REDACTED]]! Let's dive into our AML case.
Phase 1: Initial Presentation and Diagnostic Workup
Patient Presentation:
Mr. John Smith, a 67-year-old male, presents to the emergency department with complaints of fatigue, unexplained bruising, and recent onset of fevers. He reports feeling increasingly tired over the past month and noticed small red spots on his legs (petechiae). He denies any recent trauma or use of blood thinners. His past medical history includes well-controlled hypertension and hyperlipidemia. He is a former smoker (quit 10 years ago) and drinks alcohol occasionally (1-2 drinks per week).
Initial Vitals:
Temperature: 38.5C (101.3F)
Heart Rate: 110 bpm
Blood Pressure: 130/80 mmHg
Respiratory Rate: 20 breaths/min
Oxygen Saturation: 96% on room air
Initial Lab Results:
WBC: 25,000/L (Normal: 4,500-11,000/L)
Hemoglobin: 8.0 g/dL (Normal: 13.5-17.5 g/dL)
Platelets: 20,000/L (Normal: 150,000-400,000/L)
Peripheral Smear: Shows a high percentage of blasts with Auer rods

Six months after transplant, Mr. Smith's PML-RARA qPCR level increases from undetectable to 0.05% (IS). He is asymptomatic, and his blood counts remain normal. What is the MOST appropriate next step in his management?
A) Initiate preemptive therapy with ATRA and ATO.
B) Perform a bone marrow biopsy to confirm hematologic relapse.
C) Reduce immunosuppression to promote graft-versus-leukemia effect.
D) Continue to monitor PML-RARA qPCR levels monthly.

B
Excellent, [REDACTED]]! Let's dive into our AML case.
Phase 1: Initial Presentation and Diagnostic Workup

Patient Presentation:
Mr. John Smith, a 67-year-old male, presents to the emergency department with complaints of fatigue, unexplained bruising, and recent onset of fevers. He reports feeling increasingly tired over the past month and noticed small red spots on his legs (petechiae). He denies any recent trauma or use of blood thinners. His past medical history includes well-controlled hypertension and hyperlipidemia. He is a former smoker (quit 10 years ago) and drinks alcohol occasionally (1-2 drinks per week).
Initial Vitals:
Temperature: 38.5C (101.3F)
Heart Rate: 110 bpm
Blood Pressure: 130/80 mmHg
Respiratory Rate: 20 breaths/min
Oxygen Saturation: 96% on room air
Initial Lab Results:
WBC: 25,000/L (Normal: 4,500-11,000/L)
Hemoglobin: 8.0 g/dL (Normal: 13.5-17.5 g/dL)
Platelets: 20,000/L (Normal: 150,000-400,000/L)
Peripheral Smear: Shows a high percentage of blasts with Auer rods

The bone marrow biopsy confirms morphologic relapse of APL. Mr. Smith is treated with salvage therapy and achieves a second complete remission. What is the MOST appropriate long-term strategy to prevent relapse in this setting?
A) Maintenance therapy with low-dose chemotherapy.
B) Donor lymphocyte infusion (DLI).
C) Second allogeneic stem cell transplant.
D) Observation alone.

B
Excellent, [REDACTED]]! Let's dive into our AML case.
Phase 1: Initial Presentation and Diagnostic Workup
Patient Presentation:
Mr. John Smith, a 67-year-old male, presents to the emergency department with complaints of fatigue, unexplained bruising, and recent onset of fevers. He reports feeling increasingly tired over the past month and noticed small red spots on his legs (petechiae). He denies any recent trauma or use of blood thinners. His past medical history includes well-controlled hypertension and hyperlipidemia. He is a former smoker (quit 10 years ago) and drinks alcohol occasionally (1-2 drinks per week).
Initial Vitals:
Temperature: 38.5C (101.3F)
Heart Rate: 110 bpm
Blood Pressure: 130/80 mmHg
Respiratory Rate: 20 breaths/min
Oxygen Saturation: 96% on room air
Initial Lab Results:
WBC: 25,000/L (Normal: 4,500-11,000/L)
Hemoglobin: 8.0 g/dL (Normal: 13.5-17.5 g/dL)
Platelets: 20,000/L (Normal: 150,000-400,000/L)
Peripheral Smear: Shows a high percentage of blasts with Auer rods

. Smith receives DLI and achieves a second complete remission. However, he develops chronic graft-versus-host disease (GVHD) affecting his skin and liver. Which of the following is the MOST appropriate initial treatment for his chronic GVHD?
A) High-dose corticosteroids (e.g., prednisone 1 mg/kg/day).
B) Topical corticosteroids.

C) Extracorporeal photopheresis (ECP).
D) Cyclophosphamide
A
Excellent, [REDACTED]]! Let's dive into our AML case.
Phase 1: Initial Presentation and Diagnostic Workup
Patient Presentation:
Mr. John Smith, a 67-year-old male, presents to the emergency department with complaints of fatigue, unexplained bruising, and recent onset of fevers. He reports feeling increasingly tired over the past month and noticed small red spots on his legs (petechiae). He denies any recent trauma or use of blood thinners. His past medical history includes well-controlled hypertension and hyperlipidemia. He is a former smoker (quit 10 years ago) and drinks alcohol occasionally (1-2 drinks per week).
Initial Vitals:
Temperature: 38.5C (101.3F)
Heart Rate: 110 bpm
Blood Pressure: 130/80 mmHg
Respiratory Rate: 20 breaths/min
Oxygen Saturation: 96% on room air
Initial Lab Results:
WBC: 25,000/L (Normal: 4,500-11,000/L)
Hemoglobin: 8.0 g/dL (Normal: 13.5-17.5 g/dL)
Platelets: 20,000/L (Normal: 150,000-400,000/L)
Peripheral Smear: Shows a high percentage of blasts with Auer rods

Despite treatment with corticosteroids, Mr. Smith's chronic GVHD persists. Which of the following is the MOST appropriate next step in his management?
A) Increase the dose of corticosteroids.
B) Add a second-line agent such as ruxolitinib or ibrutinib.
C) Initiate total body irradiation (TBI).
D) Discontinue immunosuppression to allow for a stronger graft-versus-leukemia effect.

B
Excellent, [REDACTED]]! Let's dive into our AML case.
Phase 1: Initial Presentation and Diagnostic Workup
Patient Presentation:
Mr. John Smith, a 67-year-old male, presents to the emergency department with complaints of fatigue, unexplained bruising, and recent onset of fevers. He reports feeling increasingly tired over the past month and noticed small red spots on his legs (petechiae). He denies any recent trauma or use of blood thinners. His past medical history includes well-controlled hypertension and hyperlipidemia. He is a former smoker (quit 10 years ago) and drinks alcohol occasionally (1-2 drinks per week).
Initial Vitals:
Temperature: 38.5C (101.3F)
Heart Rate: 110 bpm
Blood Pressure: 130/80 mmHg
Respiratory Rate: 20 breaths/min
Oxygen Saturation: 96% on room air
Initial Lab Results:
WBC: 25,000/L (Normal: 4,500-11,000/L)
Hemoglobin: 8.0 g/dL (Normal: 13.5-17.5 g/dL)
Platelets: 20,000/L (Normal: 150,000-400,000/L)
Peripheral Smear: Shows a high percentage of blasts with Auer rods

Five years after allogeneic stem cell transplant, Mr. Smith remains in remission from APL but continues to have chronic GVHD, which is managed with low-dose corticosteroids and ruxolitinib. He develops several skin cancers. Which of the following is the MOST important long-term consideration for Mr. Smith?
A) Discontinue all immunosuppression.
B) Routine screening for infections.
C) Routine screening for secondary malignancies.
D) Annual bone marrow biopsies to monitor for relapse.

C

Session ID:
Assessment Domain

Session-34:
Rationale

Session ID:
Instructional Design and Pedagogical

Session-34:
The session's instructional design is outstanding. The learning objectives are not explicitly stated at the outset, preventing a perfect score, but they are clearly implied by the session's structure. The case is expertly scaffolded, seamlessly guiding the learner through a logical and realistic clinical timeline for an Acute Promyelocytic Leukemia (APL) patientfrom initial diagnosis and induction, through a critical treatment complication (Differentiation Syndrome), consolidation, relapse, stem cell transplant, and long-term post-transplant management (GVHD, secondary malignancy). Each question is framed with exceptional clarity, presenting a distinct, well-defined clinical problem. Based on the quality and specificity of the questions, the embedded feedback is inferred to be insightful and anchored in current clinical guidelines.

Session ID:
Clinical Authenticity and Content Fidelity

Session-34:
The clinical narrative is highly realistic and follows a plausible disease trajectory for APL. The case demonstrates strong alignment with current guidelines - using ATRA/ATO for induction, appropriate consolidation strategies, and evidence-based approaches to relapsed disease and transplant management. Pharmacotherapeutic choices are generally appropriate, including recognition of differentiation syndrome, proper use of consolidation therapy, and appropriate second-line GVHD treatments like ruxolitinib. The case maintains strict domain specificity without inappropriately mixing treatments from other hematologic malignancies. Minor deductions reflect some oversimplified clinical scenarios that could have been more nuanced.

Session ID:
Modeled Clinical Reasoning

Session-34:
The simulation effectively frames clinical problems at each stage, from initial diagnosis through complex post-transplant management. The case demonstrates appropriate clinical prioritization, such as securing tissue diagnosis before treatment and using molecular monitoring for relapse detection. However, the therapeutic options presented are somewhat limited in breadth - most questions have fairly obvious correct answers without challenging distractors that would test nuanced clinical judgment. The major weakness is in modeling clinical outcomes: the session provides minimal information about what happens after each decision, missing opportunities to reinforce learning through realistic consequence modeling. Students don't learn about expected timelines, potential complications, or monitoring strategies that would follow from their choices.

Session-35

Session ID:
LLM

Session-35:
Google AI (Gemini 2.0 Pro)

Session ID:
Session Transcript

Session-35:
Alright everyone, welcome! I'm thrilled to have you here today to delve into the complexities of Acute Myeloid Leukemia (AML) management. We'll be using a case-based approach to solidify your understanding of evidence-based clinical decision-making.
Let's start with our patient:
Case Presentation:
 * Patient: Mr. John Doe, a 68-year-old male.
 * Chief Complaint: Fatigue, easy bruising, and recent onset of fever.
 * Past Medical History: Hypertension, well-controlled with lisinopril.
 * Medications: Lisinopril 20mg daily.
 * Social History: Retired construction worker, lives with his wife.
 * Initial Lab Findings:
   * WBC: 35 x 10^9/L (elevated)
   * Hemoglobin: 8.0 g/dL (low)
   * Platelets: 40 x 10^9/L (low)
   * Peripheral Blood Smear: Blasts present.
 * Bone Marrow Biopsy: Consistent with AML, showing >20% blasts.
 * Cytogenetics: FLT3-ITD mutation positive, NPM1 mutation negative.
Let's begin with some initial questions to frame our discussion:
 * What is the significance of the initial lab findings, and how do they relate to the diagnosis of AML?
 * What is the importance of the FLT3-ITD mutation in this patient's case? How does it impact treatment decisions?
 * What are the general goals of AML treatment, and what are the initial considerations for induction therapy?
I'm eager to hear your thoughts and reasoning. Don't hesitate to ask clarifying questions. Let's work through this case together.

What is the significance of the initial lab findings, and how do they relate to the diagnosis of AML?

The lab findings are highly suggestive of Acute Myeloid Leukemia (AML):

Elevated WBC (35 x 10/L) indicates abnormal proliferation of white blood cells.

Low hemoglobin (8.0 g/dL) explains the patients fatigue, as there arent enough red blood cells to carry oxygen.

Low platelets (40 x 10/L) explains easy bruising and increased bleeding risk.

Blasts on peripheral smear and >20% blasts in bone marrow biopsy confirm AML diagnosis, as this is a diagnostic hallmark according to WHO criteria.

What is the importance of the FLT3-ITD mutation in this patient's case? How does it impact treatment decisions?

Answer:
The FLT3-ITD mutation is important because:

It is associated with a worse prognosis in AML.

Patients with this mutation have a higher risk of relapse and more aggressive disease.

It changes treatment plans because targeted therapy like FLT3 inhibitors (e.g., midostaurin or gilteritinib) is added to standard chemotherapy to improve outcomes

What are the general goals of AML treatment, and what are the initial considerations for induction therapy?

Answer:
Goals of AML treatment:

Achieve complete remission by eliminating leukemic cells.

Prevent relapse and improve survival.

Restore normal blood cell production.

Initial considerations for induction therapy:

Standard induction is "7+3" regimen: 7 days of cytarabine + 3 days of anthracycline (like daunorubicin or idarubicin).

Because the patient has FLT3-ITD mutation, a FLT3 inhibitor like midostaurin is added to induction.

Monitor for complications like infections due to low blood counts.
Excellent! You've grasped the key elements of the initial assessment and treatment considerations. Let's break down your answers and build upon them.
1. Significance of Initial Lab Findings:
 * You correctly identified the significance of each lab value. The elevated WBC, low hemoglobin, and low platelets are classic signs of bone marrow dysfunction and are highly suggestive of leukemia.
 * The peripheral blood smear and bone marrow biopsy are crucial for definitive diagnosis. The presence of blasts, particularly exceeding 20% in the bone marrow, confirms AML according to the World Health Organization (WHO) criteria.
2. Importance of FLT3-ITD Mutation:
 * You accurately stated that the FLT3-ITD mutation is associated with a poorer prognosis. It leads to increased blast proliferation and a higher risk of relapse.
 * You also correctly highlighted the impact on treatment. The presence of this mutation necessitates the addition of a FLT3 inhibitor to the standard induction regimen. Midostaurin is typically used in combination with standard 7+3 induction for newly diagnosed FLT3-ITD positive AML. Gilteritinib is typically used in the relapsed/refractory setting.
3. General Goals of AML Treatment and Initial Considerations for Induction Therapy:
 * Your description of the treatment goals is spot on: achieving complete remission, preventing relapse, and restoring normal hematopoiesis.
 * You correctly identified the standard "7+3" induction regimen (cytarabine + anthracycline).
 * You are correct that Midostaurin should be added to the induction regimen.
 * You correctly identified the importance of monitoring for complications, particularly infections, due to the profound myelosuppression caused by induction chemotherapy.
Further Considerations:
 * Risk Stratification: Beyond the FLT3-ITD mutation, other cytogenetic and molecular markers play a crucial role in risk stratification. This helps tailor treatment intensity and post-remission strategies.
 * Supportive Care: Given the patient's low blood counts, supportive care is essential. This includes:
   * Transfusions of red blood cells and platelets as needed.
   * Aggressive management of infections with broad-spectrum antibiotics and antifungals.
   * Growth factors (e.g., G-CSF) to stimulate white blood cell recovery.
 * Comorbidities: Mr. Doe's age and hypertension need to be considered when selecting treatment and managing potential toxicities.
Next Steps:
Given this information, let's consider the following:

* What supportive care measures would you prioritize for Mr. Doe during induction therapy?
* What monitoring parameters are essential during and after induction therapy?
* What consolidation or post-remission strategies would you consider for Mr. Doe, given his FLT3-ITD mutation?

I don't know almost of them
Can you give me hint
Absolutely! Let's break those questions down to make them more approachable.
1. Supportive Care Measures During Induction Therapy:
* Hint: Think about the immediate consequences of the low blood counts we discussed earlier. What are the most urgent risks?
  * Consider: How do we prevent life-threatening infections? How do we manage the risk of severe bleeding? How do we address the severe anemia?
2. Monitoring Parameters During and After Induction Therapy:
* Hint: Induction chemotherapy causes significant bone marrow suppression. What do we need to track closely to ensure patient safety and assess treatment response?
  * Consider: What blood counts are crucial to monitor, and how often? What signs and symptoms of complications should we be vigilant for? How do we assess if the treatment is working (i.e., achieving remission)?
3. Consolidation or Post-Remission Strategies:
* Hint: Remember the FLT3-ITD mutation and its association with a higher risk of relapse. What strategies can we use to minimize this risk after the patient achieves remission?
  * Consider: What further therapy, beyond induction, is needed to prevent relapse? Does the FLT3-ITD mutation impact this decision? What is the role of allogeneic hematopoietic stem cell transplant? What is the role of FLT3 inhibitors post remission?
Let me know if these hints help, and don't worry, we can work through each of these together.

1. Supportive Care Measures During Induction Therapy:
Main risks: infection, bleeding, and anemia because of low blood counts.
How to manage:
Prevent infections
Manage bleeding risk
Treat anemia:
2. Monitoring Parameters During and After Induction Therapy:
You're on the right track! Let's expand on those supportive care measures and monitoring parameters.
1. Supportive Care Measures During Induction Therapy:
* Prevent Infections:
  * You correctly identified this as a major concern. Due to profound neutropenia, patients are extremely vulnerable to infections.
  * Prioritize:
    * Broad-spectrum antibiotics: Initiate promptly if fever or signs of infection develop.
    * Antifungal prophylaxis: Consider antifungal medications (e.g., azoles) due to the risk of invasive fungal infections.
    * Antiviral prophylaxis: Consider antiviral medications (e.g., acyclovir) to prevent herpes simplex virus reactivation.
    * Meticulous hygiene: Emphasize handwashing and aseptic technique.
    * HEPA-filtered rooms: These are sometimes used to minimize exposure to airborne pathogens.
    * Growth factors: G-CSF or GM-CSF can be used to shorten the duration of neutropenia.
* Manage Bleeding Risk:
  * Platelet transfusions: Administer when platelet counts are very low (e.g., <10 x 10^9/L) or in the presence of active bleeding.
  * Avoid invasive procedures: Minimize the risk of trauma.
* Treat Anemia:
  * Red blood cell transfusions: Administer to maintain adequate hemoglobin levels and alleviate symptoms of fatigue and shortness of breath.
2. Monitoring Parameters During and After Induction Therapy:
* During Induction:
  * Complete blood counts (CBCs): Monitor daily or frequently to track neutrophil, platelet, and hemoglobin levels.
  * Electrolytes: Monitor for electrolyte imbalances, which can be exacerbated by chemotherapy.
  * Renal and hepatic function: Assess regularly to detect drug-related toxicities.
  * Infection surveillance: Monitor for fever, chills, and other signs of infection.
  * Cardiac monitoring: Especially during anthracycline administration, monitor for cardiotoxicity.

  * Tumor lysis syndrome monitoring: Monitor uric acid, potassium, phosphate and calcium levels.
 * After Induction:
  * Bone marrow aspirate and biopsy: Perform to assess for remission. This is the gold standard for evaluating treatment response.
  * Minimal residual disease (MRD) testing: If available, this highly sensitive test can detect very low levels of leukemia cells and predict relapse risk.
  * Cytogenetic and molecular monitoring: Repeat testing to assess for changes in the leukemia's genetic profile.
  * CBCs: Continue to monitor for recovery of normal blood counts.
  * Follow up appointments to monitor for relapse.

I hope this helps clarify the supportive care and monitoring aspects of AML management. Let's move on to consolidation/post-remission strategies.

Okay give me hint or question and I will try to find the answer

Perfect! Let's focus on the consolidation/post-remission strategies for Mr. Doe, given his FLT3-ITD mutation.

Question/Hint:

Given that Mr. Doe has achieved complete remission after induction therapy, and considering his FLT3-ITD mutation, which is associated with a high risk of relapse, what are the key strategies we should consider to consolidate his remission and minimize the risk of relapse? Think about what treatment options are available, and how the FLT3-ITD mutation impacts these choices.

To guide your thinking, consider these points:
 * What is the role of further chemotherapy after achieving remission?
 * How does the FLT3-ITD mutation influence post-remission therapy?
 * What is the role of allogeneic hematopoietic stem cell transplantation (HSCT)?
 * What is the role of maintenance therapy, particularly with FLT3 inhibitors?

After the remission, I think maybe no need for more chemo because the cancer is gone. The patient can just do monitoring every month.

Because of FLT3-ITD mutation, I think its not a big problem after remission because chemo already kill the bad cells. So maybe no need for extra treatment.

About the transplant I dont think it is needed because patient is already in remission and transplant is very risky and painful. Maybe only young people do transplant.

About Maintenance therapy I don't know can you give me hint or simple question to find the answer

You've touched on some important points, and it's understandable to have those initial thoughts. Let's clarify a few things and then delve into maintenance therapy.
 * Further Chemotherapy (Consolidation):
  * While achieving remission is a major success, AML, especially with high-risk features like FLT3-ITD, has a high risk of relapse. Consolidation chemotherapy aims to eliminate any remaining leukemia cells that may not be detectable by standard tests.
  * Therefore, further chemotherapy is typically recommended, even after achieving complete remission.
 * FLT3-ITD Mutation After Remission:
  * The FLT3-ITD mutation remains a significant risk factor even after remission. It indicates a more aggressive form of AML, and without further intervention, the risk of relapse is high.
  * Therefore, the mutation absolutely impacts post remission therapy.
 * Allogeneic Hematopoietic Stem Cell Transplantation (HSCT):
  * You're right that HSCT is a complex and potentially risky procedure. However, in patients with high-risk AML, like those with FLT3-ITD, it can offer the best chance for long-term survival.
  * While age is a factor, it's not the sole determinant. Many older patients can tolerate HSCT with careful selection and management.
  * This is a strong consideration with FLT3-ITD positive AML.
 * Maintenance Therapy:
  * Here's a simpler question: What is the goal of maintenance therapy after consolidation chemotherapy or HSCT, and how might FLT3 inhibitors play a role?
  * Hint: Maintenance therapy is designed to prevent relapse over a longer period. FLT3 inhibitors target the FLT3-ITD mutation, which is a key driver of relapse in this patient population.

Think about how we can use targeted therapy to keep the leukemia at bay.

I think the goal of maintenance therapy is maybe to make sure the patient dont get sick again fast. After chemo or transplant, maybe doctor give some vitamins or immune boosters to help the body stay strong.

About FLT3 inhibitors, maybe they not needed after remission because the cancer is already finished. Or maybe doctor give them for short time just to clean any small cancer cells left.

I dont know exactly, but I think maintenance is not always important if the patient already feel good.

Give me more questions to try understand
You're getting closer! Let's refine your understanding of maintenance therapy and the role of FLT3 inhibitors.
 * Goal of Maintenance Therapy:
   * You're right that maintenance aims to prevent relapse. However, it's not simply about "immune boosters" or vitamins. It involves specific therapies designed to target any remaining leukemia cells and prevent them from causing a recurrence.
   * It's about sustained disease control, not just general health improvement.
 * FLT3 Inhibitors After Remission:
   * This is a crucial point. FLT3 inhibitors are indeed used in the maintenance setting, especially for patients with FLT3-ITD mutations.
   * The goal is not just to "clean up" any remaining cells immediately after remission but to provide ongoing suppression of the FLT3-ITD-driven leukemia clone.
   * This is a long term strategy.
 * Importance of Maintenance:
   * Even if a patient feels good after remission, the risk of relapse remains significant, particularly with high-risk features like FLT3-ITD.
   * Maintenance therapy aims to prolong remission and improve overall survival. It's a critical part of the treatment plan, not an optional extra.
Here are some more targeted questions to help you solidify your understanding:
 * What is the rationale for using FLT3 inhibitors like midostaurin or gilteritinib as maintenance therapy in FLT3-ITD positive AML?
 * How does maintenance therapy differ from consolidation therapy, and what is the typical duration of maintenance?
 * What are the key monitoring parameters during maintenance therapy, and what potential toxicities should we be aware of with FLT3 inhibitors?
 * In the context of Mr. Doe, if he were to undergo an allogeneic HSCT, how would that influence the post-transplant maintenance strategy, and what role would FLT3 inhibitors play?

FLT3-ITD mutation is a high-risk factor because it causes the leukemia cells to grow and survive aggressively. Even after chemotherapy or transplant, there may still be hidden leukemia cells with the FLT3 mutation that can cause relapse. Using FLT3 inhibitors as maintenance therapy helps to specifically target and block the FLT3 pathway,

Give me hint about other questions
That's a very good summary of the rationale for using FLT3 inhibitors in maintenance! You've grasped the key concept of targeting the FLT3-ITD mutation to prevent relapse.
Let's break down the remaining questions with some hints:
2. How does maintenance therapy differ from consolidation therapy, and what is the typical duration of maintenance?
 * Hint: Think about the timing and intensity of these therapies. Consolidation is given after achieving remission, while maintenance is given over a longer period. Consider the goal of each phase.
   * Consolidation is typically a few cycles of intensive chemotherapy.
   * Maintenance is a longer term, lower intensity therapy to maintain remission.
3. What are the key monitoring parameters during maintenance therapy, and what potential toxicities should we be aware of with FLT3 inhibitors?
 * Hint: Consider the common side effects of FLT3 inhibitors and the need to monitor for disease recurrence.
   * Monitoring: CBCs, CMP, and regular bone marrow assessments.
   * Toxicities: think about cardiac, GI, and hematologic effects.
4. In the context of Mr. Doe, if he were to undergo an allogeneic HSCT, how would that influence the post-transplant maintenance strategy, and what role would FLT3 inhibitors play?
 * Hint: HSCT introduces a new immune system. How does this impact the risk of relapse, and what additional factors need to be

considered?
* HSCT reduces relapse risk, but it doesn't eliminate it entirely.
* FLT3 inhibitors can be used post-transplant to further minimize relapse risk.
* GVHD is a risk that must be considered.

How does maintenance therapy differ from consolidation therapy, and what is the typical duration of maintenance?
Consolidation therapy is like when the doctor gives vitamins and some medicines after remission just to make sure the patient stay healthy. It is usually very long and can last for years. Maintenance therapy is stronger and given in the hospital for few weeks only to kill any cancer cells left.
More hints about other questions
You've got the concepts mixed up a bit, but that's alright! Let's clarify the difference between consolidation and maintenance:
* Consolidation Therapy:
  * This is given after the patient achieves complete remission following induction chemotherapy.
  * It involves further cycles of chemotherapy, often at a similar intensity to induction, to eliminate any remaining leukemia cells that may not be detectable by standard tests.
  * The goal is to deepen the remission and reduce the risk of relapse.
  * It is typically given for a few cycles, not years.
* Maintenance Therapy:
  * This is given after consolidation therapy or, in some cases, after HSCT.
  * It involves lower-intensity treatment, often with oral medications, to maintain the remission and prevent relapse over a longer period.
  * The goal is to sustain disease control and prolong survival.
  * The duration can vary, but it's typically months to years, often with FLT3 inhibitors in FLT3 mutated AML.
* Key Differences:
  * Timing: Consolidation is given immediately after remission, while maintenance is given after consolidation or HSCT.
  * Intensity: Consolidation is more intensive, using chemotherapy. Maintenance is less intensive, often using targeted therapy.
  * Duration: Consolidation is shorter, lasting a few cycles. Maintenance is longer, lasting months to years.
Hints for the Remaining Questions:
* 3. What are the key monitoring parameters during maintenance therapy, and what potential toxicities should we be aware of with FLT3 inhibitors?
  * Monitoring: Think about what blood counts are important to monitor, and how often. Also, consider the importance of bone marrow assessments.
  * Toxicities: FLT3 inhibitors can have effects on the heart, digestive system, and blood counts.
* 4. In the context of Mr. Doe, if he were to undergo an allogeneic HSCT, how would that influence the post-transplant maintenance strategy, and what role would FLT3 inhibitors play?
  * HSCT Impact: HSCT replaces the patient's bone marrow with healthy donor cells, reducing the risk of relapse. However, it doesn't eliminate it entirely, especially in high-risk cases.
  * Post-Transplant: FLT3 inhibitors can be used after HSCT to further minimize relapse risk, even in the presence of donor cells. Also GVHD is a big concern.

3. What are the key monitoring parameters during maintenance therapy, and what potential toxicities should we be aware of with FLT3 inhibitors?
I think during maintenance therapy there is no need for much monitoring because the patient already finished chemo and feels good. Maybe just check blood pressure sometimes. FLT3 inhibitors are vitamins, so they dont have toxicities. They help the immune system, so no need to worry about side effects like heart or stomach problems. Also, no need to check blood tests or bone marrow anymore because the cancer is gone.
I understand your reasoning, but there are some critical misconceptions here. Let's clarify:
* Monitoring During Maintenance:
  * Even though the patient may feel well, ongoing monitoring is essential during maintenance therapy.
  * This is because:
    * Relapse can occur even without noticeable symptoms.
    * FLT3 inhibitors, while targeted, can have potential toxicities that need to be monitored.
    * We need to ensure that the maintenance therapy is effectively keeping the leukemia in remission.
  * Key monitoring parameters include:

* Complete blood counts (CBCs): To monitor for any signs of relapse or bone marrow suppression.
* Comprehensive metabolic panel (CMP): To assess kidney and liver function, as well as electrolyte levels.
* Regular bone marrow assessments: To evaluate for minimal residual disease (MRD) and ensure ongoing remission.
* Cardiac monitoring (ECG): Some FLT3 inhibitors can have cardiac effects.
* Blood pressure monitoring is also important, but it's only one aspect of overall monitoring.
* FLT3 Inhibitor Toxicities:
* FLT3 inhibitors are not vitamins. They are targeted therapies that can have potential toxicities.
* Common toxicities include:
* Cardiac effects: Such as QT prolongation, which can lead to arrhythmias.
* Gastrointestinal effects: Such as nausea, vomiting, and diarrhea.
* Hematologic effects: Such as neutropenia, thrombocytopenia, and anemia.
* Edema
* Different skin rashes
* Therefore, careful monitoring is crucial to detect and manage these potential side effects.

It is very important to understand that even targeted therapies can have toxicities.

Let's move on to the last question.

If Mr. Doe has an allogeneic HSCT, it helps reduce the risk of relapse because the donors healthy cells replace his damaged bone marrow. But since he has the FLT3-ITD mutation, the risk of relapse is still high.

More hints to know more about cases

You're absolutely right! Allogeneic HSCT significantly reduces the risk of relapse by replacing the patient's leukemic bone marrow with healthy donor cells, but the FLT3-ITD mutation still poses a substantial risk.

Here are some more hints to guide your thinking about post-transplant maintenance in this scenario:

* Graft-versus-leukemia (GVL) effect:
* Remember that the donor's immune cells can recognize and attack any remaining leukemia cells.
* How can we leverage this GVL effect while minimizing the risk of graft-versus-host disease (GVHD)?
* Minimal residual disease (MRD) monitoring:
* Even after HSCT, sensitive MRD testing is crucial.
* How does MRD status influence post-transplant maintenance strategies?
* FLT3 inhibitor role post-transplant:
* Given the persistent risk associated with FLT3-ITD, how can FLT3 inhibitors be used to further reduce the risk of relapse after HSCT?
* Think about timing, and which FLT3 inhibitors are typically used in this setting.
* GVHD prophylaxis and management:
* HSCT carries the risk of GVHD, where the donor's immune cells attack the patient's healthy tissues.
* How does GVHD prophylaxis and management interact with post-transplant maintenance strategies?
* Immunosuppression:
* Following HSCT, patients are placed on immunosuppressant medications.
* How does this impact the use of FLT3 inhibitors?

(MRD) Monitoring:

After HSCT, MRD testing is not very important because the transplant usually kills all the leukemia cells. However, some doctors might check MRD sometimes. If MRD is positive, it means the patient is cured and doesnt need more treatment. MRD testing is usually done once after transplant, and it doesnt really affect maintenance therapy decisions.

You're on the right track with some aspects, but let's clarify the role of MRD monitoring post-HSCT:

* MRD Monitoring After HSCT:
* While HSCT aims to eliminate all leukemia cells, MRD monitoring remains crucial, especially in high-risk AML like FLT3-ITD positive.
* MRD positivity after HSCT indicates the presence of residual leukemia cells, which significantly increases the risk of relapse.
* Therefore, MRD testing is not just "sometimes" but is often recommended at regular intervals post-transplant.
* MRD negativity is a very good prognostic sign, but it does not guarantee a cure.
* MRD and Treatment decisions:
* MRD does effect treatment decisions. If MRD is positive, that will likely lead to intervention.
* MRD monitoring significantly influences post-transplant maintenance strategies.

    * For example, if MRD becomes positive after HSCT, it may prompt the initiation or intensification of FLT3 inhibitor therapy.
 * FLT3 Inhibitor Role Post-Transplant:
   * FLT3 inhibitors can be used post-transplant to further minimize relapse risk, even in the presence of donor cells.
   * Gilteritinib is often favored post transplant, due to less GVHD interaction compared to Midostaurin.
   * Timing of FLT3 inhibitors post transplant is also very important, and needs to be carefully considered.
 * GVHD prophylaxis and management:
   * GVHD is a major concern post transplant.
   * Some GVHD prophylaxis medications can interact with FLT3 inhibitors, so careful monitoring is needed.
   * If a patient develops GVHD, the treatment of GVHD must be prioritized.
 * Immunosuppression:
   * Patients on immunosuppression are at increased risk of infections.
   * This needs to be considered when using FLT3 inhibitors, which can also cause neutropenia.

In summary, MRD monitoring is a vital tool for assessing relapse risk post-HSCT, and it plays a significant role in guiding post-transplant maintenance strategies, particularly in FLT3-ITD positive AML.

Immunosuppression Impact on FLT3 Inhibitors:
After HSCT, patients take immunosuppressants to prevent GVHD, and this actually makes it easier to give FLT3 inhibitors because there are no interactions. The immunosuppressants protect the patient from side effects of FLT3 inhibitors, so doctors dont need to monitor closely. Also, since the immune system is weak, FLT3 inhibitors are less effective but still given for a short time.
Okay, let's think about this a little more deeply. We know that John has a FLT3-ITD mutation, which is associated with a higher risk of relapse. We also know that there are targeted therapies available that specifically target the FLT3 receptor.

Does that suggest anything to you about potentially improving his outcome during induction? Is there a targeted agent that we might consider adding to his chemotherapy regimen?
we can add a targeted drug called midostaurin to his chemotherapy.

Midostaurin is a FLT3 inhibitor that helps block the abnormal FLT3 signaling.

It is usually added to the 7+3 chemotherapy during induction to improve outcomes and reduce relapse risk.
That's an interesting perspective, but it's important to clarify the interaction between immunosuppression and FLT3 inhibitors:
 * Immunosuppression and FLT3 Inhibitors:
   * Immunosuppressants are given to prevent GVHD, which is a serious complication where the donor's immune cells attack the recipient's tissues.
   * However, immunosuppressants do not necessarily make it "easier" to give FLT3 inhibitors. In fact, they can complicate matters.
   * Some immunosuppressants can interact with FLT3 inhibitors, potentially affecting their metabolism and increasing the risk of toxicities.
   * Therefore, careful monitoring is still essential, even in patients on immunosuppression.
 * Immunosuppression and FLT3 Inhibitor Side Effects:
   * Immunosuppressants do not protect patients from the side effects of FLT3 inhibitors.
   * FLT3 inhibitors have their own distinct toxicity profile, including cardiac, gastrointestinal, and hematologic effects.
   * These toxicities can occur independently of immunosuppressant use.
   * Therefore, monitoring for FLT3 inhibitor toxicities remains crucial.
 * Immunosuppression and FLT3 Inhibitor Efficacy:
   * While immunosuppression weakens the immune system, it doesn't necessarily make FLT3 inhibitors "less effective" against leukemia cells.
   * FLT3 inhibitors target the FLT3-ITD mutation directly, independent of immune function.
   * The duration of therapy is not shortened due to immunosuppression.
   * FLT3 inhibitors are still effective because they target the cancer cells directly.
 * Key Considerations:
   * The interplay between immunosuppression, GVHD prophylaxis, and FLT3 inhibitor therapy requires careful consideration.
   * Close monitoring of drug levels, organ function, and potential drug interactions is essential.
   * The goal is to maximize the anti-leukemic effect of FLT3 inhibitors while minimizing the risk of GVHD and other complications.

In essence, immunosuppression adds complexity to post-transplant management, necessitating meticulous monitoring and careful drug selection.

Session ID:
Assessment Domain

Session-35:
Rationale

Session ID:
Instructional Design and Pedagogical

Session-35:
The session's instructional design is outstanding. The case is expertly scaffolded, starting with foundational diagnostic concepts and progressing logically through induction, supportive care, and the highly complex nuances of consolidation, maintenance, and post-transplant strategies. The pedagogical approach is a key strength; the simulation masterfully adapts to the learner's significant and repeated misconceptions. When the learner expresses uncertainty or provides incorrect information (e.g., confusing consolidation with maintenance, or minimizing the toxicity of FLT3 inhibitors), the tutor provides clear, corrective, and non-judgmental feedback. The use of hints to guide the learner is effective. Instructions are exceptionally clear. The only minor area for improvement is the lack of explicitly stated, measurable learning objectives at the very beginning of the session, though the goals are clearly implied by the case's structure.

Session ID:
Clinical Authenticity and Content Fidelity

Session-35:
The clinical fidelity of this session is excellent and meets the highest standards for accuracy and safety. Plausibility & Guideline Alignment: The clinical narrative, featuring a 68-year-old patient with newly diagnosed FLT3-ITD positive AML, is exceptionally realistic and represents a common, challenging clinical scenario. All therapeutic recommendations and rationales provided by the tutor (e.g., "7+3" plus midostaurin for induction, consideration of allogeneic HSCT, and the role of FLT3 inhibitors in maintenance) are perfectly aligned with current evidence-based guidelines from bodies like the NCCN and ELN. Pharmacotherapeutic Accuracy: The session demonstrates optimal pharmacotherapeutic accuracy. It correctly distinguishes the roles of different FLT3 inhibitors, identifies critical monitoring parameters for toxicities (e.g., cardiac, hematologic), and accurately describes complex concepts like drug-drug interactions with immunosuppressants post-HSCT. Domain Specificity: The case remains strictly within the domain of AML. There are no instances of domain entanglement where a therapy, diagnostic, or standard of care from another disease is incorrectly applied. The content is specific, accurate, and safe.

Session ID:
Modeled Clinical Reasoning

Session-35:
The session excellently models expert-level clinical reasoning processes. Each clinical problem is expertly framed with appropriate context and relevant data for decision-making. The diagnostic and therapeutic breadth is comprehensive, covering the full spectrum from initial presentation through complex post-transplant maintenance strategies. The prioritization and decision-making consistently reflect appropriate clinical priorities, including securing accurate diagnosis before treatment, risk stratification, and evidence-based therapeutic selection. The modeling of clinical outcomes is realistic and educationally valuable, though could be slightly more detailed in some areas regarding specific complications and their management strategies.

Session-36

Session ID:
LLM

Session-36:
Google AI (Gemini 2.0 Pro)

Session ID:
Session Transcript

Session-36:
Patient Presentation:
Mrs. Eleanor Vance, a 72-year-old female with a history of hypertension and well-controlled type 2 diabetes, presents to her primary care physician with complaints of increasing fatigue, shortness of breath, and recent onset of frequent nosebleeds. She reports that these symptoms have been progressively worsening over the past month. Physical examination reveals pallor, scattered ecchymoses, and mild hepatosplenomegaly. Initial laboratory results show:
WBC: 3.2 x 10^9/L (Normal: 4.0-11.0 x 10^9/L)
Hemoglobin: 7.8 g/dL (Normal: 12.0-16.0 g/dL)
Platelets: 25 x 10^9/L (Normal: 150-400 x 10^9/L)
Peripheral blood smear shows 25% blasts.
Creatinine: 1.3 mg/dL (Normal: 0.6-1.2 mg/dL)
AST: 45 U/L (Normal: 10-40 U/L)
ALT: 50 U/L (Normal: 7-35 U/L)
Based on the initial presentation, peripheral blood smear result, and laboratory findings, which of the following is the MOST LIKELY diagnosis?
a) Aplastic Anemia
b) Myelodysplastic Syndrome (MDS)
c) Acute Myeloid Leukemia (AML)
d) Immune Thrombocytopenic Purpura (ITP)
Correct! The most likely diagnosis is c) Acute Myeloid Leukemia (AML).
Rationale: The presence of 25% blasts in the peripheral blood smear, coupled with pancytopenia (low WBC, hemoglobin, and platelets) and the patient's symptoms of fatigue, shortness of breath, and bleeding, strongly suggests AML. According to the WHO classification, AML requires at least 20% blasts in the bone marrow or peripheral blood for diagnosis (with some exceptions involving specific genetic translocations).

Why the other options are incorrect:

a) Aplastic Anemia: While aplastic anemia also presents with pancytopenia, it typically lacks blasts in the peripheral blood. A bone marrow biopsy would show hypocellularity.

b) Myelodysplastic Syndrome (MDS): MDS can present with cytopenias and dysplastic features in blood cells, but it may or may not have an increased blast percentage. While MDS could be considered in the differential diagnosis, the presence of 25% blasts makes AML more likely. Further investigation including bone marrow analysis is needed to differentiate.

d) Immune Thrombocytopenic Purpura (ITP): ITP primarily involves isolated thrombocytopenia (low platelet count) and does not typically cause significant anemia or leukopenia. The peripheral blood smear would also not show blasts.

Following the suspected diagnosis of AML, which of the following laboratory tests is MOST CRITICAL to perform next for risk stratification and treatment planning?
a) Serum Lactate Dehydrogenase (LDH)

b) Bone Marrow Aspirate and Biopsy with Cytogenetic and Molecular Analysis
c) Comprehensive Metabolic Panel (CMP)
d) Peripheral Blood Flow Cytometry for Minimal Residual Disease (MRD) assessment
Correct! The MOST CRITICAL next step is b) Bone Marrow Aspirate and Biopsy with Cytogenetic and Molecular Analysis.

Rationale: While all the listed tests can provide useful information, cytogenetic and molecular analysis of the bone marrow is absolutely essential for risk stratification and treatment planning in AML. These analyses identify specific chromosomal abnormalities and gene mutations that significantly impact prognosis and guide treatment decisions. For example, the presence of FLT3-ITD mutations is associated with a poorer prognosis and may warrant the use of specific FLT3 inhibitors. Conversely, NPM1 mutations in the absence of FLT3-ITD mutations are generally associated with a more favorable prognosis.

Why the other options are incorrect:

a) Serum Lactate Dehydrogenase (LDH): LDH is a marker of cell turnover and can be elevated in AML, reflecting disease burden. However, it doesn't provide specific information for risk stratification or treatment planning.

c) Comprehensive Metabolic Panel (CMP): A CMP is important for assessing overall organ function and identifying any pre-existing comorbidities, but it doesn't provide information directly related to the AML subtype or prognosis.

d) Peripheral Blood Flow Cytometry for Minimal Residual Disease (MRD) assessment: MRD assessment is typically performed after treatment to evaluate the response to therapy, not at the initial diagnosis for risk stratification.
Patient Presentation:
Mrs. Eleanor Vance, a 72-year-old female with a history of hypertension and well-controlled type 2 diabetes, presents to her primary care physician with complaints of increasing fatigue, shortness of breath, and recent onset of frequent nosebleeds. She reports that these symptoms have been progressively worsening over the past month. Physical examination reveals pallor, scattered ecchymoses, and mild hepatosplenomegaly. Initial laboratory results show:
WBC: 3.2 x 10^9/L (Normal: 4.0-11.0 x 10^9/L)
Hemoglobin: 7.8 g/dL (Normal: 12.0-16.0 g/dL)
Platelets: 25 x 10^9/L (Normal: 150-400 x 10^9/L)
Peripheral blood smear shows 25% blasts.
Creatinine: 1.3 mg/dL (Normal: 0.6-1.2 mg/dL)
AST: 45 U/L (Normal: 10-40 U/L)
ALT: 50 U/L (Normal: 7-35 U/L)
The bone marrow aspirate and biopsy results are back. They reveal 65% blasts, confirming the diagnosis of AML. Cytogenetic analysis reveals a t(15;17) translocation. Molecular testing is pending. Based on this information, which subtype of AML is MOST LIKELY?
a) AML with Myelodysplasia-Related Changes
b) Acute Promyelocytic Leukemia (APL)
c) AML with inv(16)(p13.1q22) or t(16;16)(p13.1;q22)
d) AML with mutated NPM1
Correct! The answer is b) Acute Promyelocytic Leukemia (APL).

Rationale: The t(15;17) translocation is pathognomonic for APL. This translocation results in the fusion of the PML gene on chromosome 15 with the RAR gene on chromosome 17. This fusion protein disrupts normal myeloid differentiation and leads to the accumulation of promyelocytes in the bone marrow.

Why the other options are incorrect:

a) AML with Myelodysplasia-Related Changes: This subtype is defined by specific morphological features in the bone marrow cells (dysplasia) and is not specifically associated with t(15;17).

c) AML with inv(16)(p13.1q22) or t(16;16)(p13.1;q22): These chromosomal abnormalities are characteristic of acute myelomonocytic leukemia with abnormal eosinophils (AML M4Eo).

d) AML with mutated NPM1: While NPM1 mutations are common in AML, they are not specifically associated with t(15;17).

Given the diagnosis of APL, which of the following is the MOST APPROPRIATE initial treatment approach?
a) "7+3" induction chemotherapy (7 days of cytarabine and 3 days of an anthracycline)
b) High-dose cytarabine-based consolidation chemotherapy
c) All-trans retinoic acid (ATRA) plus arsenic trioxide (ATO)
d) Hypomethylating agents (e.g., azacitidine or decitabine)
Correct! The most appropriate initial treatment approach is c) All-trans retinoic acid (ATRA) plus arsenic trioxide (ATO).

Rationale: ATRA and ATO are targeted therapies that have revolutionized the treatment of APL. ATRA binds to the PML-RAR fusion protein and induces differentiation of the leukemic promyelocytes into mature cells. ATO promotes the degradation of the PML-RAR fusion protein. The combination of ATRA and ATO has been shown to be highly effective in inducing remission in APL patients, with excellent long-term outcomes and reduced toxicity compared to traditional chemotherapy.

Why the other options are incorrect:

a) "7+3" induction chemotherapy (7 days of cytarabine and 3 days of an anthracycline): While "7+3" is a standard induction regimen for other types of AML, it is not the preferred initial treatment for APL due to the high efficacy and lower toxicity of ATRA and ATO. Chemotherapy may still be used in certain high-risk APL cases or if ATRA/ATO is not feasible.

b) High-dose cytarabine-based consolidation chemotherapy: This is used as consolidation therapy after remission is achieved, not as initial induction therapy.

d) Hypomethylating agents (e.g., azacitidine or decitabine): These agents are primarily used in the treatment of myelodysplastic syndromes (MDS) and some types of AML, particularly in older patients who are not candidates for intensive chemotherapy. They are not the standard of care for APL.

Mrs. Vance is started on ATRA and ATO. Two days later, she develops dyspnea, fever, and new pulmonary infiltrates on chest X-ray. She also has developed increasing edema and weight gain. Which of the following is the MOST LIKELY diagnosis?
a) Tumor Lysis Syndrome (TLS)
b) Differentiation Syndrome (formerly known as ATRA Syndrome)
c) Pneumonia
d) Cardiogenic Pulmonary Edema
Correct! The most likely diagnosis is b) Differentiation Syndrome (formerly known as ATRA Syndrome).

Rationale: Differentiation syndrome is a potentially life-threatening complication that can occur in patients with APL treated with ATRA (and sometimes ATO). It is characterized by fever, dyspnea, pulmonary infiltrates, pleural or pericardial effusions, edema, weight gain, and acute respiratory distress. It is thought to be caused by the release of cytokines from differentiating leukemic cells, leading to endothelial activation and increased vascular permeability.

Why the other options are incorrect:

a) Tumor Lysis Syndrome (TLS): While TLS can occur in AML, it is less common in APL, particularly with the use of ATRA and ATO. TLS typically presents with electrolyte abnormalities (hyperuricemia, hyperkalemia, hyperphosphatemia, hypocalcemia) and acute kidney injury.

c) Pneumonia: While pneumonia is possible, the rapid onset of dyspnea, fever, pulmonary infiltrates, and edema in the setting of ATRA/ATO treatment is more suggestive of differentiation syndrome.

d) Cardiogenic Pulmonary Edema: While Mrs. Vance has a history of hypertension, the acute onset of these symptoms in the context of APL treatment makes differentiation syndrome more likely. Cardiogenic pulmonary edema would typically be associated with other signs of heart failure.

Which of the following is the MOST APPROPRIATE initial dose and route of administration for dexamethasone in the treatment of

differentiation syndrome?
a) 40 mg intravenously daily
b) 10 mg intravenously every 12 hours
c) 1 mg/kg orally daily
d) 5 mg orally twice daily
Correct! The most appropriate initial dose and route of administration for dexamethasone in the treatment of differentiation syndrome is a) 40 mg intravenously daily.

Rationale: In moderate to severe cases of differentiation syndrome, high-dose corticosteroids are typically used to rapidly reduce inflammation and improve respiratory symptoms. A common starting dose is dexamethasone 10 mg IV every 12 hours or 40 mg IV daily. The IV route ensures rapid and reliable absorption.

Why the other options are incorrect:

b) 10 mg intravenously every 12 hours: While this dose is sometimes used, it may be less effective than 40mg daily in more severe cases. Some guidelines suggest this dosing for milder cases.

c) 1 mg/kg orally daily: While this is a reasonable dose for other indications of corticosteroids, the oral route is less reliable in a patient with potential fluid shifts and the need for rapid response.

d) 5 mg orally twice daily: This dose is too low for the initial management of moderate to severe differentiation syndrome.

During induction therapy with ATRA and ATO, Mrs. Vance develops QTc prolongation on her electrocardiogram (ECG). Her baseline QTc was normal. Which of the following medications on her current medication list is MOST LIKELY contributing to the QTc prolongation?
a) All-trans retinoic acid (ATRA)
b) Arsenic trioxide (ATO)
c) Dexamethasone
d) Metformin (for her diabetes)
the answer is b) Arsenic trioxide (ATO).

Rationale: ATO is known to cause QTc prolongation by directly affecting cardiac ion channels. It is a well-documented side effect, and ECG monitoring is essential during ATO therapy.

Why the other options are incorrect:

a) All-trans retinoic acid (ATRA): ATRA is not typically associated with QTc prolongation, although some case reports have suggested a possible link.

c) Dexamethasone: While dexamethasone can cause electrolyte imbalances (e.g., hypokalemia) that could indirectly contribute to QTc prolongation, it is not a direct QTc prolonging agent.

d) Metformin (for her diabetes): Metformin is not known to cause QTc prolongation.

It's important to remember that ATO carries a significant risk of QTc prolongation and requires careful monitoring.

Patient Presentation:
Mrs. Eleanor Vance, a 72-year-old female with a history of hypertension and well-controlled type 2 diabetes, presents to her primary care physician with complaints of increasing fatigue, shortness of breath, and recent onset of frequent nosebleeds. She reports that these symptoms have been progressively worsening over the past month. Physical examination reveals pallor, scattered ecchymoses, and mild hepatosplenomegaly. Initial laboratory results show:
WBC: 3.2 x 10^9/L (Normal: 4.0-11.0 x 10^9/L)
Hemoglobin: 7.8 g/dL (Normal: 12.0-16.0 g/dL)

Platelets: 25 x 10^9/L (Normal: 150-400 x 10^9/L)
Peripheral blood smear shows 25% blasts.
Creatinine: 1.3 mg/dL (Normal: 0.6-1.2 mg/dL)
AST: 45 U/L (Normal: 10-40 U/L)
ALT: 50 U/L (Normal: 7-35 U/L)

Given Mrs. Vance's QTc prolongation secondary to ATO (QTc >500 ms), which of the following is the MOST APPROPRIATE next step in managing her QTc prolongation?
a) Initiate magnesium supplementation and continue ATO at the same dose.
b) Discontinue ATO and monitor the QTc interval closely.
c) Reduce the dose of ATO by 50% and monitor the QTc interval closely.
d) Add a QTc-prolonging agent (e.g., ondansetron) to counteract the effect of ATO.
Correct! The most appropriate next step is b) Discontinue ATO and monitor the QTc interval closely.

Rationale: When the QTc interval is significantly prolonged (>500 ms) due to a drug like ATO, the safest and most prudent approach is to discontinue the offending agent to reduce the risk of potentially fatal arrhythmias. Close monitoring of the QTc interval is essential to ensure that it returns to a safe range.

Why the other options are incorrect:

a) Initiate magnesium supplementation and continue ATO at the same dose: While magnesium supplementation can be helpful in preventing Torsades de Pointes, it is not sufficient to address a significantly prolonged QTc interval caused by a drug like ATO. Continuing ATO at the same dose would further increase the risk of arrhythmias.

c) Reduce the dose of ATO by 50% and monitor the QTc interval closely: While dose reduction might be considered for less severe QTc prolongation, it is not the safest approach when the QTc is already >500 ms.

d) Add a QTc-prolonging agent (e.g., ondansetron) to counteract the effect of ATO: This is absolutely contraindicated, as it would further prolong the QTc interval and increase the risk of arrhythmias.

Mrs. Vance's absolute neutrophil count (ANC) drops to <500 cells/L during induction therapy. She develops a fever of 101.5F (38.6C). Blood cultures are drawn, and empiric antibiotics are initiated. Which of the following antibiotic regimens is MOST APPROPRIATE for initial empiric therapy in this setting, assuming the institution's antibiogram shows good susceptibility to all listed agents?
a) Oral ciprofloxacin
b) Intravenous vancomycin
c) Intravenous cefepime
d) Intravenous azithromycin
Correct! The most appropriate antibiotic regimen is c) Intravenous cefepime.

Rationale: Cefepime is a fourth-generation cephalosporin with broad-spectrum activity against both Gram-positive and Gram-negative bacteria, including Pseudomonas aeruginosa. It is a commonly used and well-established empiric antibiotic for febrile neutropenia.

Why the other options are incorrect:

a) Oral ciprofloxacin: Oral antibiotics are generally not recommended for initial empiric therapy in febrile neutropenia due to concerns about absorption and bioavailability, especially in patients with mucositis or gastrointestinal issues.

b) Intravenous vancomycin: Vancomycin is primarily active against Gram-positive bacteria. While it may be added to the empiric regimen if there is suspicion of a Gram-positive infection (e.g., catheter-related infection, skin infection, known colonization with MRSA), it is not appropriate as a single agent for initial empiric therapy in febrile neutropenia.

d) Intravenous azithromycin: Azithromycin has limited activity against many of the Gram-negative bacteria commonly seen in febrile

neutropenia.

Mrs. Vance's blood cultures ultimately grow Escherichia coli sensitive to cefepime, which she continues to receive. After 7 days of antibiotics, she is afebrile and her ANC recovers to >500 cells/L. She has no other signs or symptoms of infection. Which of the following is the MOST APPROPRIATE duration of antibiotic therapy in this scenario?
a) Continue cefepime for a total of 14 days.
b) Continue cefepime until the ANC is >1000 cells/L.
c) Discontinue cefepime now.
d) Switch to oral ciprofloxacin for 7 days of step-down therapy.
Correct! The most appropriate duration of antibiotic therapy in this scenario is a) Continue cefepime for a total of 14 days.

Rationale: In general, for bacteremia (bloodstream infection) with Gram-negative organisms like E. coli, a total duration of 14 days of antibiotic therapy is typically recommended, even if the patient becomes afebrile and the ANC recovers earlier. This helps to ensure adequate eradication of the infection and prevent recurrence.

Why the other options are incorrect:

b) Continue cefepime until the ANC is >1000 cells/L: While it's important to monitor the ANC, the duration of antibiotic therapy should be based primarily on the type and severity of the infection, not solely on the ANC.

c) Discontinue cefepime now: Discontinuing antibiotics after only 7 days may be too short, as discussed above.

d) Switch to oral ciprofloxacin for 7 days of step-down therapy: A switch to oral antibiotics may be considered in some cases of bacteremia, but not with Ciprofloxacin. Step-down therapy with Cipro is only a good consideration when the bacteremia is from pseudomonas and has documented sensitivity.

Patient Presentation:
Mrs. Eleanor Vance, a 72-year-old female with a history of hypertension and well-controlled type 2 diabetes, presents to her primary care physician with complaints of increasing fatigue, shortness of breath, and recent onset of frequent nosebleeds. She reports that these symptoms have been progressively worsening over the past month. Physical examination reveals pallor, scattered ecchymoses, and mild hepatosplenomegaly. Initial laboratory results show:
WBC: 3.2 x 10^9/L (Normal: 4.0-11.0 x 10^9/L)
Hemoglobin: 7.8 g/dL (Normal: 12.0-16.0 g/dL)
Platelets: 25 x 10^9/L (Normal: 150-400 x 10^9/L)
Peripheral blood smear shows 25% blasts.
Creatinine: 1.3 mg/dL (Normal: 0.6-1.2 mg/dL)
AST: 45 U/L (Normal: 10-40 U/L)
ALT: 50 U/L (Normal: 7-35 U/L)

Which of the following is the MOST APPROPRIATE post-remission therapy for Mrs. Vance, given her age (72 years old) and the diagnosis of standard-risk APL?
a) Autologous hematopoietic stem cell transplantation (auto-HSCT)
b) Allogeneic hematopoietic stem cell transplantation (allo-HSCT)
c) Consolidation therapy with ATRA and ATO
d) Observation with close monitoring
Correct! The most appropriate post-remission therapy is c) Consolidation therapy with ATRA and ATO.

Rationale: Multiple studies have demonstrated that consolidation therapy with ATRA and ATO after achieving remission in APL significantly reduces the risk of relapse and improves overall survival. This approach is preferred over hematopoietic stem cell

transplantation (HSCT) in standard-risk APL patients due to its lower toxicity and comparable efficacy.

Why the other options are incorrect:

a) Autologous hematopoietic stem cell transplantation (auto-HSCT): Auto-HSCT is not typically used in APL because the leukemic cells may contaminate the autologous stem cell product.

b) Allogeneic hematopoietic stem cell transplantation (allo-HSCT): Allo-HSCT is generally reserved for high-risk APL patients or those who relapse after initial therapy. It carries a higher risk of complications, such as graft-versus-host disease (GVHD).

d) Observation with close monitoring: While close monitoring is important, observation alone without consolidation therapy is not recommended in APL due to the high risk of relapse.

Patient Presentation:
Mrs. Eleanor Vance, a 72-year-old female with a history of hypertension and well-controlled type 2 diabetes, presents to her primary care physician with complaints of increasing fatigue, shortness of breath, and recent onset of frequent nosebleeds. She reports that these symptoms have been progressively worsening over the past month. Physical examination reveals pallor, scattered ecchymoses, and mild hepatosplenomegaly. Initial laboratory results show:
WBC: 3.2 x 10^9/L (Normal: 4.0-11.0 x 10^9/L)
Hemoglobin: 7.8 g/dL (Normal: 12.0-16.0 g/dL)
Platelets: 25 x 10^9/L (Normal: 150-400 x 10^9/L)
Peripheral blood smear shows 25% blasts.
Creatinine: 1.3 mg/dL (Normal: 0.6-1.2 mg/dL)
AST: 45 U/L (Normal: 10-40 U/L)
ALT: 50 U/L (Normal: 7-35 U/L)

Mrs. Vance is undergoing consolidation therapy with ATRA and ATO. She develops persistent grade 2 peripheral neuropathy (numbness and tingling in her hands and feet) that is interfering with her daily activities. Which of the following is the MOST APPROPRIATE next step in managing her peripheral neuropathy?
a) Discontinue ATO permanently.
b) Reduce the dose of ATO and continue therapy.
c) Add gabapentin or pregabalin for symptomatic relief.
d) Initiate physical therapy and continue ATO at the same dose.
Correct! The most appropriate next step is b) Reduce the dose of ATO and continue therapy.

Rationale: ATO-induced peripheral neuropathy is a common side effect, and dose reduction is often necessary to manage it. Reducing the dose of ATO may allow the patient to continue consolidation therapy while minimizing the severity of the neuropathy. Close monitoring is essential to ensure that the dose reduction does not compromise the efficacy of the treatment.

Why the other options are incorrect:

a) Discontinue ATO permanently: Permanently discontinuing ATO should be reserved for severe (grade 3 or 4) neuropathy that does not improve with dose reduction.

c) Add gabapentin or pregabalin for symptomatic relief: While gabapentin or pregabalin can be used to manage neuropathic pain, they do not address the underlying cause of the neuropathy and should not be used as the sole intervention.

d) Initiate physical therapy and continue ATO at the same dose: Physical therapy may be helpful in managing some symptoms of neuropathy, but it is unlikely to be sufficient to address grade 2 neuropathy without dose modification.

Patient Presentation:

Mrs. Eleanor Vance, a 72-year-old female with a history of hypertension and well-controlled type 2 diabetes, presents to her primary care physician with complaints of increasing fatigue, shortness of breath, and recent onset of frequent nosebleeds. She reports that these symptoms have been progressively worsening over the past month. Physical examination reveals pallor, scattered ecchymoses, and mild hepatosplenomegaly. Initial laboratory results show:

WBC: 3.2 x 10^9/L (Normal: 4.0-11.0 x 10^9/L)

Hemoglobin: 7.8 g/dL (Normal: 12.0-16.0 g/dL)

Platelets: 25 x 10^9/L (Normal: 150-400 x 10^9/L)

Peripheral blood smear shows 25% blasts.

Creatinine: 1.3 mg/dL (Normal: 0.6-1.2 mg/dL)

AST: 45 U/L (Normal: 10-40 U/L)

ALT: 50 U/L (Normal: 7-35 U/L)

Mrs. Vance completes consolidation therapy with ATRA and ATO and remains in complete remission. She is seen in follow-up every 3 months. Which of the following is the MOST APPROPRIATE monitoring strategy for relapse in Mrs. Vance?

a) Serial bone marrow aspirates and biopsies every 3 months.

b) Serial peripheral blood MRD assessment by flow cytometry every 3 months.

c) Regular complete blood counts (CBCs) with differential and peripheral blood smear every 3 months.

d) CT scans of the chest, abdomen, and pelvis every 6 months.

Correct! The most appropriate monitoring strategy is c) Regular complete blood counts (CBCs) with differential and peripheral blood smear every 3 months.

Rationale: CBCs with differential and peripheral blood smear are a simple, inexpensive, and non-invasive way to monitor for relapse in APL patients. A rising white blood cell count, the reappearance of blasts in the peripheral blood, or a decrease in platelets could be early signs of relapse and warrant further investigation with a bone marrow aspirate and biopsy.

Why the other options are incorrect:

a) Serial bone marrow aspirates and biopsies every 3 months: As discussed above, this is too invasive and burdensome for routine monitoring.

b) Serial peripheral blood MRD assessment by flow cytometry every 3 months: While MRD assessment is becoming increasingly important in AML, it is not yet the standard of care for routine monitoring of APL patients in complete remission.

d) CT scans of the chest, abdomen, and pelvis every 6 months: CT scans are not typically used for routine monitoring of APL patients unless there is a specific clinical indication.

Five years after completing therapy, Mrs. Vance presents with new onset fatigue, weight loss, and night sweats. A CBC reveals pancytopenia and 10% blasts in the peripheral blood. A bone marrow aspirate and biopsy confirm relapsed APL. What is the MOST APPROPRIATE initial treatment approach for her relapsed APL?

a) Re-induction with ATRA and ATO

b) High-dose cytarabine-based chemotherapy

c) Allogeneic hematopoietic stem cell transplantation (allo-HSCT)

d) Clinical trial evaluating novel targeted therapies

Correct! The most appropriate initial treatment approach for her relapsed APL is b) High-dose cytarabine-based chemotherapy.

Rationale: While ATRA and ATO can be considered, they are often less effective in relapsed disease, especially if the patient initially responded well to it. High-dose cytarabine-based chemotherapy can be effective in re-inducing remission in relapsed APL. This approach can help to reduce the leukemic burden and potentially allow for consolidation with allogeneic hematopoietic stem cell

transplantation (allo-HSCT), which is the only curative option.

Why the other options are incorrect:

a) Re-induction with ATRA and ATO: While ATRA and ATO can be considered, they are often less effective in relapsed disease, especially if the patient initially responded well to it.

c) Allogeneic hematopoietic stem cell transplantation (allo-HSCT): Allo-HSCT is the only curative option, but the transplant is more successful after getting the leukemia into remission with chemotherapy first.

d) Clinical trial evaluating novel targeted therapies: A clinical trial evaluating novel therapies might be a good option, but it depends on availability and the patient's eligibility.
After achieving a second remission with high-dose cytarabine-based chemotherapy, Mrs. Vance undergoes successful matched-related donor allogeneic hematopoietic stem cell transplantation (allo-HSCT). One year post-transplant, she develops chronic graft-versus-host disease (GVHD) affecting her skin and liver. Which of the following is the MOST APPROPRIATE first-line treatment for her chronic GVHD?
a) High-dose corticosteroids (e.g., prednisone 1 mg/kg/day)
b) Topical corticosteroids (e.g., triamcinolone cream)
c) Extracorporeal photopheresis (ECP)
d) Ruxolitinib
Correct! The most appropriate first-line treatment for her chronic GVHD is a) High-dose corticosteroids (e.g., prednisone 1 mg/kg/day).

Rationale: Systemic corticosteroids, such as prednisone, are the standard first-line treatment for chronic GVHD affecting multiple organs or causing significant symptoms. They work by suppressing the immune response that is causing the GVHD.

Why the other options are incorrect:

b) Topical corticosteroids (e.g., triamcinolone cream): Topical corticosteroids are useful for localized skin GVHD, but they are not sufficient for systemic disease or GVHD affecting other organs like the liver.

c) Extracorporeal photopheresis (ECP): ECP is a second-line treatment option for chronic GVHD that is refractory to corticosteroids.

d) Ruxolitinib: Ruxolitinib is a JAK1/JAK2 inhibitor that is approved for the treatment of steroid-refractory acute and chronic GVHD..

Session ID:
Assessment Domain

Session-36:
Rationale

Session ID:
Instructional Design and Pedagogical

Session-36:
The session's instructional design is strong in structure and clarity. The case is expertly scaffolded, moving logically from initial diagnosis (Q1-3), through induction therapy and its acute complications (Q4-10), to consolidation and long-term management (Q11-15). Instructions are exceptionally clear. The primary weakness is in the quality of the embedded feedback. While the structure of the rationales is good, several contain significant clinical inaccuracies (e.g., Q10, Q13, Q14), which makes the feedback misleading rather than instructive. Learning objectives are not explicitly stated at the outset, but the educational goals are strongly implied by the case progression.

Session ID:

Clinical Authenticity and Content Fidelity

Session-36:

The clinical authenticity contains critical flaws that undermine its educational value. Plausibility & Domain Specificity: The clinical narrative is highly plausible and entirely consistent with a real-world case of Acute Promyelocytic Leukemia (APL). The simulation remains strictly within the APL domain, without evidence of domain entanglement.

Guideline Alignment & Pharmacotherapeutic Error: The session contains multiple, significant deviations from current evidence-based guidelines. Relapse Monitoring (Q13): The recommendation to monitor for relapse with only CBCs and a peripheral smear is dangerously outdated. The standard of care for APL for over a decade has been serial molecular monitoring via RT-PCR for the PML-RARA transcript. Failing to perform molecular monitoring would be a major clinical error, as it allows for early intervention before hematologic relapse occurs.

Antibiotic Duration (Q10): The recommendation for a mandatory 14-day course of IV antibiotics for an uncomplicated E. coli bacteremia in a patient with neutrophil recovery is not aligned with current IDSA guidelines or antimicrobial stewardship principles, which would support a shorter course (e.g., 7 days). Relapse Treatment (Q14): The rationale that ATRA+ATO is "less effective" in relapse is inaccurate. ATO-based salvage regimens are a primary treatment option for relapsed APL.

These errors represent suboptimal and, in the case of monitoring, potentially dangerous recommendations.

Session ID:

Modeled Clinical Reasoning

Session-36:

The simulation successfully models the process of clinical reasoning, but this is undermined by flawed content. Each stage presents a clearly framed problem (e.g., a new symptom, a lab abnormality) with a very strong set of plausible options and distractors. The clinical outcomes described are realistic and instructive. However, the prioritization and decision-making are flawed. While the strategy of monitoring for relapse is correct, the chosen method (CBC instead of molecular testing) represents flawed prioritization. Similarly, the decision to commit to a 14-day antibiotic course is not a well-reasoned, risk-adjusted decision by modern standards. The reasoning process leads to incorrect and unsafe conclusions, thus earning an "Acceptable" but not "Appropriate" score.

Session-37

Session ID:
LLM

Session-37:
Google AI (Gemini 2.0 Pro)

Session ID:
Session Transcript

Session-37:

Phase 1: Initial Presentation and Diagnostic Workup

Patient Presentation:

A 67-year-old male, Mr. John Smith, presents to the emergency department with a 2-week history of fatigue, easy bruising, and recent onset of fever (101.5F). He reports no significant past medical history other than well-controlled hypertension managed with lisinopril. He denies any recent infections or travel.

Initial Lab Results:

WBC: 25,000/L (Significant Blasts)

Hemoglobin: 8.0 g/dL

Platelets: 25,000/L

Peripheral Smear: 65% blasts, Auer rods present

Creatinine: 0.9 mg/dL

AST/ALT: Within normal limits

Bilirubin: 1.0 mg/dL

LDH: Elevated

Question 1:

Based on the initial presentation and lab results, which of the following is the MOST likely diagnosis?

A) Acute Lymphoblastic Leukemia (ALL)
B) Acute Myeloid Leukemia (AML)
C) Chronic Myeloid Leukemia (CML)
D) Myelodysplastic Syndrome (MDS)

Choose the best answer and provide the letter (A, B, C, or D).
B
Correct!

Feedback:

Your answer is correct. The presence of Auer rods in the peripheral smear is highly suggestive of Acute Myeloid Leukemia (AML).

Why B is Correct: Auer rods are pathognomonic for AML. The high blast percentage in the peripheral smear further supports the diagnosis of an acute leukemia.

Why A is Incorrect: While ALL can present with similar symptoms, Auer rods are not typically seen. Lymphoblasts are the predominant cell type.

Why C is Incorrect: CML usually presents with a high WBC count and a spectrum of myeloid cells, but not a predominance of blasts and rarely Auer rods. The Philadelphia chromosome (BCR-ABL1) is characteristic of CML.

Why D is Incorrect: MDS can transform to AML, but the initial presentation is usually characterized by cytopenias (low blood counts) and dysplastic features in the bone marrow, not a high blast count in the peripheral blood.

Let's move on to the next step in the diagnostic workup.

.

Question 2:

The patient undergoes a bone marrow biopsy and aspirate. The results confirm a diagnosis of AML. Which of the following is the MOST important next step in further classifying Mr. Smith's AML?

A) Flow cytometry to determine immunophenotype.
B) Cytogenetic and molecular testing to identify specific mutations and chromosomal abnormalities.
C) Bone marrow biopsy to assess for dysplasia.
D) Liver biopsy to rule out hepatic involvement
B
Correct!

Feedback:

Your answer is correct. Cytogenetic and molecular testing are the most important next steps in classifying Mr. Smith's AML.

Why B is Correct: Cytogenetic and molecular testing are essential for identifying specific mutations (e.g., FLT3-ITD, NPM1, IDH1/2, TP53) and chromosomal abnormalities (e.g., t(15;17), t(8;21), inv(16)) that define different AML subtypes and risk categories according to the ELN 2022 recommendations. These findings have significant implications for prognosis and treatment selection (e.g., the use of FLT3 inhibitors in FLT3-ITD mutated AML).

Why A is Incorrect: Flow cytometry is important for identifying the lineage of the blasts (myeloid vs. lymphoid) and for detecting specific cell surface markers, but it does not provide information about genetic mutations or chromosomal abnormalities that are critical for risk stratification.

Why C is Incorrect: Bone marrow biopsy confirms the diagnosis of AML and provides information about morphology and blast percentage, but cytogenetic and molecular testing provide more detailed information for risk stratification and treatment planning.

Why D is Incorrect: Liver biopsy is not routinely performed in the initial workup of AML unless there is evidence of liver involvement or other specific indications.

Let's move on. The cytogenetic and molecular testing results come back.

Question 3:

The cytogenetic and molecular testing reveals the following:

Karyotype: 46, XY

FLT3-ITD: Positive (high allelic ratio)

NPM1: Negative

IDH1/2: Negative

TP53: Wild-type

Based on these results, and using the ELN 2022 risk stratification, how would you classify Mr. Smith's AML?

A) Favorable Risk
B) Intermediate Risk
C) Adverse Risk
D) Undetermined Risk

C
Correct!

Feedback:

Your answer is correct. Based on the provided results and the ELN 2022 risk stratification, Mr. Smith's AML would be classified as Adverse Risk.

Why C is Correct: The presence of a FLT3-ITD mutation with a high allelic ratio is classified as adverse risk according to the ELN 2022 recommendations, regardless of NPM1 status. The absence of NPM1, IDH1/2, and a wild-type TP53 do not change the adverse risk classification in the presence of a high allelic ratio FLT3-ITD.

Why A is Incorrect: Favorable risk AML typically includes patients with NPM1 mutations without FLT3-ITD, or those with core-binding factor AML [t(8;21) or inv(16)].

Why B is Incorrect: Intermediate risk AML includes patients with normal cytogenetics or certain other mutations that are not classified as favorable or adverse.

Why D is Incorrect: While some cases may have undetermined risk due to lack of sufficient information, the presence of FLT3-ITD with high allelic ratio clearly places this patient in the adverse risk category.

Good job. Let's move on.

Question 4:

Given Mr. Smith's age, FLT3-ITD positive AML (adverse risk), and otherwise good performance status, which of the following induction regimens is the MOST appropriate initial treatment option?

A) 7+3 (Cytarabine and Daunorubicin) alone
B) 7+3 plus Midostaurin
C) Low-dose Cytarabine (LDAC)
D) Decitabine and Venetoclax

B
Correct!

Feedback:

Your answer is correct. 7+3 plus Midostaurin is the most appropriate initial treatment option for Mr. Smith.

Why B is Correct: For younger, fit patients with FLT3-ITD positive AML, the combination of standard 7+3 chemotherapy (cytarabine and daunorubicin) plus a FLT3 inhibitor like midostaurin is the recommended approach. Multiple studies have demonstrated improved outcomes with this combination compared to chemotherapy alone. This aligns with current ESMO guidelines for AML.

Why A is Incorrect: While 7+3 is a standard induction regimen, it is not optimal for patients with FLT3-ITD positive AML. FLT3 inhibitors have been shown to improve outcomes in this subgroup.

Why C is Incorrect: Low-dose cytarabine (LDAC) is generally reserved for older or less fit patients who cannot tolerate intensive chemotherapy.

Why D is Incorrect: Decitabine and Venetoclax is an option for older or unfit patients and does not address the FLT3-ITD mutation.

One more question for Phase 1.

Question 5:

Prior to initiating induction therapy with 7+3 plus midostaurin, what is the MOST important baseline assessment to perform in this patient, considering the planned therapy?

A) Pulmonary function tests (PFTs)
B) EKG and assessment of QTc interval
C) Renal function assessment with cystatin C
D) Neurological examination
B
Correct!

Feedback:

Your answer is correct. EKG and assessment of QTc interval is the most important baseline assessment to perform prior to initiating induction therapy with 7+3 plus midostaurin.

Why B is Correct: Midostaurin is known to cause QTc prolongation, which can lead to arrhythmias. Therefore, it is crucial to obtain a baseline EKG and assess the QTc interval before starting the drug, and to monitor the QTc interval regularly during treatment.

Why A is Incorrect: Pulmonary function tests are not specifically required before initiating 7+3 plus midostaurin, although they may be considered in patients with pre-existing pulmonary conditions.

Why C is Incorrect: Renal function assessment is important before starting chemotherapy, but cystatin C is not necessarily required as a baseline test unless there is concern for significant renal impairment.

Why D is Incorrect: A neurological examination is not specifically required before initiating 7+3 plus midostaurin, unless there are pre-existing neurological conditions or concerns.

This concludes Phase 1! You've successfully navigated the initial presentation and diagnostic workup. You correctly identified the diagnosis, ordered the appropriate tests for classification, applied the ELN 2022 risk stratification, and selected the most appropriate induction regimen. Well done.

Phase 2: Induction Therapy and Supportive Care

Mr. Smith receives induction therapy with 7+3 (cytarabine 100 mg/m2 IV continuous infusion days 1-7 and daunorubicin 60 mg/m2 IV days 1-3) plus midostaurin 50 mg PO BID days 8-21.

Question 6:

On day 7 of induction, Mr. Smith develops febrile neutropenia (absolute neutrophil count < 500/L and temperature > 101F). Blood cultures are drawn, and empiric antibiotic therapy is initiated. Which of the following is the MOST appropriate initial empiric antibiotic regimen for this patient?

A) Vancomycin monotherapy
B) Cefepime monotherapy
C) Cefepime plus Vancomycin
D) Ciprofloxacin monotherapy
B
Correct!

Feedback:

Your answer is correct. Cefepime monotherapy is the most appropriate initial empiric antibiotic regimen for this patient.

Why B is Correct: Cefepime is an anti-pseudomonal fourth-generation cephalosporin with broad-spectrum activity against gram-negative bacteria, including Pseudomonas aeruginosa, which is a common cause of infection in febrile neutropenia. In many cases, it provides adequate empiric coverage as monotherapy. Current IDSA guidelines often recommend an anti-pseudomonal beta-lactam as first-line therapy.

Why A is Incorrect: Vancomycin monotherapy only covers gram-positive bacteria and is not sufficient as initial empiric therapy for febrile neutropenia, unless there is a high suspicion for MRSA or other gram-positive infections.

Why C is Incorrect: The combination of cefepime and vancomycin should be reserved for patients with specific risk factors for MRSA or other gram-positive infections (e.g., catheter-related infections, known colonization with MRSA, skin or soft tissue infections). Overuse of vancomycin can contribute to the development of vancomycin-resistant bacteria.

Why D is Incorrect: Ciprofloxacin monotherapy may not provide adequate coverage against all gram-negative organisms, particularly Pseudomonas aeruginosa, and is generally not recommended as initial empiric therapy for febrile neutropenia.

Good job. Let's continue.

Question 7:

After 4 days of cefepime, Mr. Smith remains febrile. Blood cultures are negative. Which of the following is the MOST appropriate next step in managing his persistent febrile neutropenia?

A) Continue cefepime monotherapy
B) Add vancomycin to cefepime
C) Add an antifungal agent to cefepime
D) Discontinue cefepime and start meropenem
C

Correct!

Feedback:

Your answer is correct. Adding an antifungal agent to cefepime is the most appropriate next step in managing Mr. Smith's persistent febrile neutropenia.

Why C is Correct: Persistent febrile neutropenia (fever persisting for >4 days despite broad-spectrum antibiotics) warrants consideration of empiric antifungal therapy. This is because prolonged neutropenia and broad-spectrum antibiotic use increase the risk of invasive fungal infections. Options for empiric antifungal therapy include azoles (e.g., fluconazole, voriconazole, posaconazole), echinocandins (e.g., caspofungin, micafungin), or amphotericin B formulations, depending on local epidemiology and risk factors.

Why A is Incorrect: Continuing cefepime monotherapy without addressing the possibility of a fungal infection is not appropriate in the setting of persistent febrile neutropenia.

Why B is Incorrect: Adding vancomycin is not indicated since blood cultures are negative and there is no evidence of a gram-positive infection.

Why D is Incorrect: Discontinuing cefepime and starting meropenem might be considered if there was concern for resistance to cefepime, but it is not the most appropriate initial step in this situation. Adding antifungal coverage is more important.

Let's move on.

Question 8:

Mr. Smith's absolute neutrophil count (ANC) recovers to > 1000/L on day 28. A bone marrow aspirate is performed to assess response to induction therapy. The results show 4% blasts. How would you interpret this result?

A) Complete Remission (CR)
B) Minimal Residual Disease (MRD) positive
C) Partial Remission (PR)
D) Resistant Disease
A
Correct!

Feedback:

Your answer is correct. This result would be interpreted as Complete Remission (CR).

Why A is Correct: According to standard criteria, a complete remission (CR) in AML is defined as:

< 5% blasts in the bone marrow

Absolute neutrophil count (ANC) > 1000/L

Platelet count > 100,000/L

Absence of extramedullary disease
Since Mr. Smith meets these criteria (4% blasts and ANC > 1000/L - platelet count is not limiting in this case), he is considered to be in CR.

Why B is Incorrect: While MRD assessment is important, the bone marrow blast percentage is the primary factor in determining CR in

this scenario. MRD is assessed using more sensitive techniques like flow cytometry or PCR.

Why C is Incorrect: Partial remission (PR) is not a commonly used term in AML.

Why D is Incorrect: Resistant disease refers to patients who do not achieve CR after induction therapy.

Let's continue.

Question 9:

Mr. Smith achieves complete remission after induction therapy. He is now being evaluated for post-remission therapy. Which of the following factors would CONTRAINDICATE him from receiving an allogeneic hematopoietic stem cell transplant (allo-HSCT)?

A) Age of 67 years
B) FLT3-ITD positivity
C) Availability of a matched sibling donor
D) Significant cardiac comorbidity (e.g., severe heart failure)
D
Correct!

Feedback:

Your answer is correct. Significant cardiac comorbidity (e.g., severe heart failure) would contraindicate Mr. Smith from receiving an allogeneic hematopoietic stem cell transplant (allo-HSCT).

Why D is Correct: Significant cardiac comorbidity, such as severe heart failure, is a major contraindication to allo-HSCT due to the high risk of transplant-related mortality. The conditioning regimens used in allo-HSCT can be cardiotoxic, and patients with pre-existing cardiac conditions are at increased risk of developing severe complications.

Why A is Incorrect: Age alone is not an absolute contraindication to allo-HSCT. While transplant outcomes are generally better in younger patients, allo-HSCT can be considered in older patients who are otherwise fit and have high-risk disease.

Why B is Incorrect: FLT3-ITD positivity is an indication for allo-HSCT in first remission, as it is associated with a higher risk of relapse.

Why C is Incorrect: The availability of a matched sibling donor is a positive factor that increases the likelihood of being able to proceed with allo-HSCT.

Excellent. One more question for Phase 2.

Question 10:

Given Mr. Smith's cardiac comorbidity (he has stable angina), he is deemed ineligible for allo-HSCT. Which of the following is the MOST appropriate post-remission therapy option for him?

A) Observation alone
B) High-dose cytarabine consolidation
C) Low-dose cytarabine maintenance
D) Sorafenib maintenance
D
Correct!

Feedback:

Your answer is correct. Sorafenib maintenance is the most appropriate post-remission therapy option for Mr. Smith, considering his cardiac comorbidity and ineligibility for allo-HSCT.

Why D is Correct: For patients with FLT3-ITD positive AML who are not candidates for allo-HSCT, maintenance therapy with a FLT3 inhibitor like sorafenib has been shown to improve outcomes. This approach targets the underlying FLT3-ITD mutation and can help prevent relapse.

Why A is Incorrect: Observation alone is not recommended after achieving CR in AML, especially in patients with adverse-risk features.

Why B is Incorrect: High-dose cytarabine consolidation is a standard post-remission therapy option for AML, but it may not be the best choice for patients with significant comorbidities, as it can be associated with significant toxicity. Given the cardiac comorbidity, it might be best to avoid high dose cytarabine if possible.

Why C is Incorrect: Low-dose cytarabine maintenance is generally reserved for older or less fit patients who cannot tolerate more intensive therapy.

This concludes Phase 2! You successfully managed febrile neutropenia, interpreted bone marrow results, considered transplant eligibility, and selected the most appropriate post-remission therapy. You are doing great.

Phase 3: Post-Remission Therapy and Long-Term Management

Mr. Smith starts sorafenib maintenance therapy.

Question 11:

Two months after starting sorafenib, Mr. Smith develops grade 3 hand-foot syndrome (palmar-plantar erythrodysesthesia). What is the MOST appropriate initial management strategy?

A) Discontinue sorafenib permanently.
B) Reduce the dose of sorafenib and use topical corticosteroids and emollients.
C) Continue sorafenib at the same dose and add oral pain medication.
D) Switch to an alternative FLT3 inhibitor.
B
Correct!

Feedback:

Your answer is correct. Reducing the dose of sorafenib and using topical corticosteroids and emollients is the most appropriate initial management strategy.

Why B is Correct: Grade 3 hand-foot syndrome is a significant toxicity that requires intervention. The initial management should involve dose reduction of sorafenib and symptomatic treatment with topical corticosteroids and emollients. This approach allows the patient to continue receiving the benefits of sorafenib maintenance therapy while managing the toxicity.

Why A is Incorrect: Discontinuing sorafenib permanently should be reserved for severe cases of hand-foot syndrome that do not respond to dose reduction and symptomatic treatment.

Why C is Incorrect: Continuing sorafenib at the same dose without addressing the toxicity is not appropriate and can lead to worsening of the hand-foot syndrome.

Why D is Incorrect: Switching to an alternative FLT3 inhibitor is an option if sorafenib is not tolerated, but it is not the most appropriate initial step. Dose reduction and symptomatic treatment should be tried first.

Let's move on.

Question 12:

Despite dose reduction and supportive care, Mr. Smith continues to experience grade 2 hand-foot syndrome. He is also complaining of persistent fatigue. A follow-up bone marrow aspirate is performed. The results show 2% blasts, and MRD by flow cytometry is negative. What is your interpretation of these findings?

A) Relapsed AML
B) Persistent Complete Remission with treatment-related toxicities
C) Secondary MDS
D) Transformation to Acute Lymphoblastic Leukemia
B
Correct!

Feedback:

Your answer is correct. The interpretation of these findings is Persistent Complete Remission with treatment-related toxicities.

Why B is Correct: The bone marrow aspirate shows 2% blasts, which is still within the criteria for complete remission (<5% blasts). MRD by flow cytometry is negative, which further supports the continued remission status. The persistent grade 2 hand-foot syndrome and fatigue are most likely related to sorafenib maintenance therapy.

Why A is Incorrect: Relapsed AML is defined as >5% blasts in the bone marrow after achieving complete remission.

Why C is Incorrect: Secondary MDS is a possibility, but the bone marrow findings and negative MRD are more consistent with continued remission.

Why D is Incorrect: Transformation to Acute Lymphoblastic Leukemia is unlikely without evidence of lymphoblasts in the bone marrow.

Let's move on.

Question 13:

Given Mr. Smith's persistent grade 2 hand-foot syndrome and fatigue despite dose reduction, what is the MOST appropriate next step in his management?

A) Discontinue sorafenib permanently.
B) Continue sorafenib at the current dose and add supportive care measures.
C) Switch to gilteritinib
D) Decrease the dose of Sorafenib again.
D
Correct!

Feedback:

Your answer is correct. Decreasing the dose of Sorafenib again is the most appropriate step.

Why D is correct: With persistent grade 2 hand-foot syndrome, another attempt at dose reduction should be tried. While there is a risk of reduced efficacy, managing the side effects and maintaining quality of life is important.

Why A is incorrect: Permanent discontinuation of sorafenib should be considered as a last resort, if side effects are unmanageable.

Why B is incorrect: Continuing sorafenib at the current dose without addressing the toxicity is not appropriate and can lead to worsening of the hand-foot syndrome.

Why C is Incorrect: Switching to gilteritinib can be considered, but only after dose reductions have been exhausted.

Two more questions to go!

Question 14:

Mr. Smith's sorafenib dose is further reduced, and his hand-foot syndrome improves to grade 1. He continues on maintenance therapy. One year later, he presents with fatigue and a new onset of bone pain. A bone marrow aspirate is performed, and the results show 8% blasts and MRD is positive. How would you classify Mr. Smith's current status?

A) Complete Remission
B) Relapsed AML
C) Secondary MDS
D) Treatment-related Myelodysplastic Syndrome
B
Correct!

Feedback:

Your answer is correct. Mr. Smith's current status would be classified as Relapsed AML.

Why B is Correct: Relapsed AML is defined as >5% blasts in the bone marrow after achieving complete remission. In this case, Mr. Smith has 8% blasts and positive MRD, indicating that the AML has relapsed.

Why A is Incorrect: Complete remission requires <5% blasts in the bone marrow.

Why C is Incorrect: Secondary MDS is a possibility, but the increased blast percentage is more consistent with relapsed AML.

Why D is Incorrect: While treatment-related MDS can occur, the presence of >5% blasts indicates relapse rather than MDS.

Final Question!

Question 15:

Given Mr. Smith's relapsed AML, which of the following is the MOST appropriate next step in his management, assuming he remains in relatively good condition (ECOG PS 0-1)?

A) Palliative care
B) Clinical trial enrollment
C) indion chemotherapy
D) Hypomethylating agent (HMA) monotherapy
By
Correct!

Feedback:

Your answer is correct. Clinical trial enrollment is the most appropriate next step in Mr. Smith's management.

Why B is Correct: For a patient with relapsed AML and a good performance status, enrollment in a clinical trial is often the most appropriate option. Clinical trials offer access to novel therapies and may provide the best chance of achieving a second remission or prolonging survival.

Why A is Incorrect: Palliative care is appropriate when active treatment is no longer feasible or desired, but it is not the first-line option for a patient with relapsed AML and a good performance status.

Why C is Incorrect: Re-induction chemotherapy is an option for relapsed AML, but it is often associated with significant toxicity and may not be effective, especially in patients who have already received intensive chemotherapy. However, if a clinical trial is unavailable, this could be considered.

Why D is Incorrect: Hypomethylating agent (HMA) monotherapy is generally reserved for older or less fit patients with relapsed AML who cannot tolerate intensive chemotherapy.

This concludes Phase 3 and our AML Clinical Case Management session! You successfully managed the long-term complications of maintenance therapy and made a sound decision regarding the management of relapsed disease. You have demonstrated a strong understanding of AML diagnosis, risk stratification, treatment, and supportive care.

Now, let's summarize your performance:

Demonstrated Strengths:

Accurate diagnosis and classification of AML

Appropriate selection of induction and post-remission therapies

Effective management of febrile neutropenia and other treatment-related toxicities

Sound clinical reasoning in complex scenarios

Areas for Improvement:

Continue to refine your understanding of the ELN risk stratification and its impact on treatment decisions.

Stay up-to-date on the latest clinical trial data and emerging therapies in AML.

Guideline-Based Recommendations for the Case:

All treatment decisions were generally aligned with current ESMO guidelines for AML.

Risk stratification was based on the ELN 2022 recommendations.

Supportive care protocols were consistent with IDSA guidelines for febrile neutropenia.

Tailored Learning Resources:

Review the ELN 2022 recommendations for AML risk stratification: [Link to ELN Guidelines] (Note: Replace with actual link)

Explore the ESMO Clinical Practice Guidelines for AML: [Link to ESMO Guidelines] (Note: Replace with actual link)

Read recent publications on novel therapies for relapsed/refractory AML.

Session ID:
Assessment Domain

Session-37:
Rationale

Session ID:
Instructional Design and Pedagogical

Session-37:
The session's instructional design is of very high quality. The case is expertly scaffolded, moving seamlessly from initial presentation and diagnosis through risk stratification, induction therapy, management of complications, and long-term/relapse management. The instructional clarity is excellent, with unambiguous questions and smooth transitions between phases. The quality of the embedded feedback is a major strength; each rationale is accurate, detailed, cites the correct clinical guidelines (e.g., ELN, ESMO, IDSA), and correctly explains why the chosen answer is superior to the distractors. The only minor area for improvement is the lack of explicitly stated learning objectives at the start of the session, which prevents a perfect score in that category, although the educational goals are clearly implied and are well-summarized at the conclusion.

Session ID:
Clinical Authenticity and Content Fidelity

Session-37:
The session demonstrates exceptional clinical authenticity and content fidelity. The clinical narrative is highly plausible, mirroring a realistic presentation and course for a patient with adverse-risk AML. All diagnostic and therapeutic recommendations are perfectly aligned with current, evidence-based guidelines, including the ELN 2022 risk stratification criteria and IDSA guidelines for febrile neutropenia. The pharmacotherapeutic choicesfrom targeted induction with midostaurin to FLT3 inhibitor maintenance and appropriate supportive careare optimal and well-justified. Critically, the session is free of any domain entanglement; all concepts, diagnostics, and therapies are specific and appropriate to the management of Acute Myeloid Leukemia. THE CORRECT CLASSIFICATION IS INTERMEDIATE RISK.

Session ID:
Modeled Clinical Reasoning

Session-37:
The simulation expertly models the clinical reasoning process of a skilled practitioner. Each stage of the case presents a well-framed and relevant clinical problem, providing the necessary data for a decision. The multiple-choice options offer an excellent breadth of plausible and relevant alternatives, including key distractors that effectively test for nuanced understanding (e.g., choosing between different induction regimens or post-remission strategies). The overall strategic pathwayfrom diagnosis to relapse managementdemonstrates flawless prioritization and risk-adjusted decision-making. Furthermore, the modeled clinical outcomes are both realistic and highly instructive, reinforcing learning about treatment-related toxicities and the disease's natural history.

Session-38

Session ID:
LLM

Session-38:
Google AI (Gemini 2.0 Pro)

Session ID:
Session Transcript

Session-38:
A 67-year-old male presents to the emergency department with a two-week history of fatigue, easy bruising, and intermittent fever. His past medical history includes hypertension and well-controlled type 2 diabetes. He reports no recent infections or significant family history of hematologic malignancies.
Initial Laboratory Findings:
 * White blood cell count (WBC): 25 x 10^9/L (with 70% blasts)
 * Hemoglobin (Hgb): 8.5 g/dL
 * Platelet count: 30 x 10^9/L
 * Creatinine: 1.1 mg/dL
 * Aspartate aminotransferase (AST): 45 U/L
 * Alanine aminotransferase (ALT): 50 U/L
Question 1:
Based on the initial presentation and laboratory findings, which of the following is the most likely initial step in the diagnostic workup?
a) Lumbar puncture
b) Bone marrow biopsy and aspirate
c) Computed tomography (CT) scan of the chest, abdomen, and pelvis
d) Flow cytometry of peripheral blood

You've correctly identified that a bone marrow biopsy and aspirate (b) is the most crucial initial step in diagnosing AML.
 * Rationale:
   * The presence of circulating blasts (70% in this case) strongly suggests acute leukemia.
   * A bone marrow biopsy is essential for:
     * Confirming the diagnosis of AML.
     * Determining the percentage of blasts in the bone marrow.
     * Performing cytogenetic and molecular studies for risk stratification.
 * Why other options are less appropriate at this stage:
   * a) Lumbar puncture: While important for assessing central nervous system involvement, it's not a primary diagnostic tool for AML.
   * c) CT scan: While useful for evaluating organomegaly or infections, it's not the initial diagnostic test.
   * d) Flow cytometry of peripheral blood: Although helpful, a bone marrow aspirate is needed for full assessment of blast percentage and morphology, as well as cytogenetic and molecular studies.
This step aligns with ESMO guidelines, which emphasize the necessity of bone marrow examination for definitive AML diagnosis.
The bone marrow biopsy reveals 80% blasts. Flow cytometry identifies the blasts as CD33+, CD34+, and CD117+. Cytogenetic analysis shows t(15;17). Which of the following WHO 2022 classifications best describes this patient's AML?
a) AML with myelodysplasia-related changes
b) AML with mutated NPM1
c) Acute promyelocytic leukemia (APL)
d) AML with t(8;21)(q22;q22.1); RUNX1-RUNX1T1

The correct answer is c) Acute promyelocytic leukemia (APL).
 * Rationale:
   * The presence of the t(15;17) translocation is pathognomonic for APL. This translocation results in the fusion of the PML and RARA genes.

* APL is a distinct subtype of AML with unique clinical and therapeutic implications.
* Why other options are incorrect:
* a) AML with myelodysplasia-related changes: This classification is indicated by specific morphological dysplasia and cytogenetic abnormalities, which are not described in the patient's case.
* b) AML with mutated NPM1: While NPM1 mutations are common in AML, they are not associated with t(15;17).
* d) AML with t(8;21)(q22;q22.1); RUNX1-RUNX1T1: This is another specific AML subtype, but it involves a different chromosomal translocation.

Given the diagnosis of APL, which of the following is the most appropriate initial treatment approach?
a) 7+3 induction chemotherapy (cytarabine and daunorubicin)
b) All-trans retinoic acid (ATRA) and arsenic trioxide (ATO)
c) High-dose cytarabine
d) Venetoclax and azacitidine
The correct answer is b) All-trans retinoic acid (ATRA) and arsenic trioxide (ATO).
* Rationale:
* ATRA and ATO are the standard of care for newly diagnosed APL, especially in non-high-risk cases.
* This combination targets the underlying pathophysiology of APL by inducing differentiation and degradation of the PML-RARA fusion protein.
* Why other options are incorrect:
* a) 7+3 induction chemotherapy: While a standard AML induction regimen, it's not the first-line treatment for APL. ATRA/ATO is more effective and less toxic.
* c) High-dose cytarabine: This is used in some AML subtypes, but not primary APL treatment.
* d) Venetoclax and azacitidine: Used in some AML cases, but not first line for APL.

The use of ATRA and ATO, instead of standard 7+3, has greatly improved outcomes for APL patients.
The patient is started on ATRA and ATO. On day 5 of treatment, he develops fever, dyspnea, and pulmonary infiltrates. Which of the following is the most likely complication?
a) Tumor lysis syndrome (TLS)
b) Differentiation syndrome (retinoic acid syndrome)
c) Febrile neutropenia
d) Cardiotoxicity from ATO
The correct answer is b) Differentiation syndrome (retinoic acid syndrome).
* Rationale:
* Differentiation syndrome is a potentially life-threatening complication of ATRA and ATO therapy in APL.
* It's characterized by fever, dyspnea, pulmonary infiltrates, pleural or pericardial effusions, hypotension, and renal failure.
* Early recognition and treatment with corticosteroids are crucial.
* Why other options are incorrect:
* a) Tumor lysis syndrome (TLS): While possible in AML, it typically presents with electrolyte abnormalities and renal dysfunction, not primarily pulmonary symptoms.
* c) Febrile neutropenia: This is a common complication of many chemotherapy regimens, but the specific symptom cluster here points to differentiation syndrome.
* d) Cardiotoxicity from ATO: While ATO can cause QT prolongation, the acute respiratory distress is more indicative of differentiation syndrome.

Early recognition and management of differentiation syndrome is crucial for APL patients.
How should the differentiation syndrome be managed?
a) Discontinue ATRA and ATO immediately.
b) Administer dexamethasone and continue ATRA and ATO.
c) Administer broad-spectrum antibiotics and supportive care.
d) Initiate hemodialysis.
The correct answer is b) Administer dexamethasone and continue ATRA and ATO.
* Rationale:
* Corticosteroids, particularly dexamethasone, are the mainstay of treatment for differentiation syndrome.
* Interrupting ATRA and ATO is generally not recommended, as it can compromise the effectiveness of APL therapy.
* The benefits of continuing the APL directed therapy generally outweighs the risks of discontinuing.
* Why other options are incorrect:
* a) Discontinue ATRA and ATO immediately: This is generally avoided unless the syndrome is life-threatening and unresponsive to

steroids.

* c) Administer broad-spectrum antibiotics and supportive care: While supportive care is important, it doesn't address the underlying pathophysiology of differentiation syndrome. Antibiotics would be used if infection was suspected, but the question describes differentiation syndrome.

* d) Initiate hemodialysis: This would be considered if renal failure develops as a complication of differentiation syndrome, but is not the primary management.

The patient's differentiation syndrome is managed with dexamethasone. His respiratory symptoms improve, and his fever subsides. His complete blood count begins to show improvement.

Question 6:

After resolution of the differentiation syndrome, what is the next critical step in managing this patient's APL?

a) Proceed with high-dose cytarabine consolidation.

b) Continue ATRA and ATO until complete remission.

c) Initiate allogeneic hematopoietic stem cell transplantation (HSCT).

d) Begin maintenance therapy with low-dose chemotherapy.

The correct answer is b) Continue ATRA and ATO until complete remission.

* Rationale:
  * In APL, consolidation typically involves continuing ATRA and ATO to deepen the remission and minimize the risk of relapse.
  * This approach leverages the high sensitivity of APL to these agents.
* Why other options are incorrect:
  * a) Proceed with high-dose cytarabine consolidation: While cytarabine is used in AML, it's not the standard consolidation for APL.
  * c) Initiate allogeneic hematopoietic stem cell transplantation (HSCT): HSCT is usually reserved for relapsed or high-risk APL, not as initial consolidation.
  * d) Begin maintenance therapy with low-dose chemotherapy: Maintenance therapy is used after consolidation, not as the immediate next step.

The consolidation phase is very important in APL.

The patient achieves complete remission after completing the planned cycles of ATRA and ATO. Which of the following is the most important component of post-remission risk stratification in this patient?

a) Assessment of minimal residual disease (MRD) by polymerase chain reaction (PCR) for PML-RARA.

b) Evaluation of cytogenetic response by repeat bone marrow biopsy.

c) Measurement of serum lactate dehydrogenase (LDH) levels.

d) Monitoring of complete blood counts for recovery.

The correct answer is a) Assessment of minimal residual disease (MRD) by polymerase chain reaction (PCR) for PML-RARA.

* Rationale:
  * PCR for PML-RARA is the most sensitive and specific method for detecting MRD in APL.
  * It allows for early identification of patients at risk for relapse, even when conventional cytogenetics and blood counts are normal.
* Why other options are incorrect:
  * b) Evaluation of cytogenetic response by repeat bone marrow biopsy: While important, PCR for PML-RARA is more sensitive for detecting MRD.
  * c) Measurement of serum lactate dehydrogenase (LDH) levels: LDH is a nonspecific marker and not used for MRD assessment in APL.
  * d) Monitoring of complete blood counts for recovery:

The patient's MRD by PCR for PML-RARA is negative after consolidation therapy. What is the next step in his treatment?

a) Proceed to allogeneic HSCT.

b) Initiate maintenance therapy.

c) Continue ATRA and ATO for another cycle.

d) No further therapy is needed.

The correct answer is b) Initiate maintenance therapy.

* Rationale:
  * Maintenance therapy is a crucial part of post-remission treatment for APL, even in patients with negative MRD.
  * It helps to further reduce the risk of relapse and improve long-term outcomes.
* Why other options are incorrect:
  * a) Proceed to allogeneic HSCT: HSCT is generally reserved for relapsed or high-risk APL, not for patients in first complete remission with negative MRD.
  * c) Continue ATRA and ATO for another cycle: While consolidation is essential, prolonged use beyond the planned cycles is

generally not recommended in the absence of MRD.
 * d) No further therapy is needed: skipping
Which of the following is the most common regimen used for maintenance therapy in APL?
a) Azacitidine
b) Decitabine
c) 6-mercaptopurine, methotrexate, and ATRA
d) Venetoclax
The correct answer is c) 6-mercaptopurine, methotrexate, and ATRA.
 * Rationale:
 * This combination is commonly used for APL maintenance therapy.
 * It combines agents with different mechanisms of action to reduce the risk of relapse.
 * Why other options are incorrect:
 * a) Azacitidine: While used in other AML subtypes, it's not the standard maintenance for APL.
 * b) Decitabine: Similar to azacitidine, not standard for APL maintenance.
 * d) Venetoclax: Used in combination with other drugs in some AML cases, but not standard maintenance for APL.
The combination of 6-MP, methotrexate, and ATRA has been shown to be effective in maintaining remissions in APL.
During maintenance therapy, the patient develops recurrent episodes of thrombocytopenia. What is the most appropriate next step?
a) Discontinue all maintenance therapy.
b) Reduce the dose of 6-mercaptopurine and methotrexate.
c) Initiate platelet transfusions.
d) Start growth colony stimulating factors.
The correct answer is b) Reduce the dose of 6-mercaptopurine and methotrexate.
 * Rationale:
 * Dose reduction is the most appropriate initial step in managing thrombocytopenia during maintenance therapy.
 * It allows the patient to continue receiving the benefits of therapy while minimizing the risk of bleeding complications.
 * Why other options are incorrect:
 * a) Discontinue all maintenance therapy: This is generally avoided unless the thrombocytopenia is severe and unresponsive to dose reduction.
 * c) Initiate platelet transfusions: While used for symptomatic or severe thrombocytopenia, it's not the first-line approach for managing mild to moderate thrombocytopenia during maintenance.
 * d) Start growth colony stimulating factors: These are used for neutropenia, not thrombocytopenia.
Dose adjustments are frequently required during maintenance.

The patient completes his maintenance therapy with good tolerance and remains in complete remission with negative MRD. He is now being followed in the outpatient clinic for long-term monitoring.
Question 11:
What is the recommended frequency of MRD monitoring by PCR for PML-RARA during the first year of follow-up after completing maintenance therapy?
a) Every 3 months
b) Every 6 months
c) Every 12 months
d) Only if symptoms of relapse occur
The correct answer is a) Every 3 months.
 * Rationale:
 * Frequent MRD monitoring is crucial in the first year after completing APL therapy.
 * Every 3 months allows for early detection of relapse and timely intervention.
 * Why other options are incorrect:
 * b) Every 6 months: While monitoring is still important, it may miss early signs of relapse.
 * c) Every 12 months: This is too infrequent, especially in the high-risk first year.
 * d) Only if symptoms of relapse occur: Waiting for symptoms is not ideal, as early detection of MRD allows for earlier intervention and better outcomes.
Frequent monitoring is key.
Five years after completing maintenance therapy, the patient develops new-onset fatigue and easy bruising. A complete blood count reveals the following: WBC 3.0 x 10^9/L, Hgb 9.0 g/dL, and platelet count 50 x 10^9/L. What is the most likely next step in the

evaluation?
a) Repeat bone marrow biopsy and aspirate.
b) Initiate growth colony-stimulating factors.
c) Start a course of broad-spectrum antibiotics.
d) Order a CT scan of the abdomen and pelvis.
The correct answer is a) Repeat bone marrow biopsy and aspirate.
 * Rationale:
   * A bone marrow biopsy is essential to evaluate for relapse of APL.
   * The new-onset cytopenias are concerning and warrant a thorough evaluation of the bone marrow.
 * Why other options are incorrect:
   * b) Initiate growth colony-stimulating factors: While helpful for neutropenia, they don't address the underlying cause of the cytopenias.
   * c) Start a course of broad-spectrum antibiotics: While infections can cause cytopenias, a bone marrow evaluation is necessary to rule out relapse first.
   * d) Order a CT scan of the abdomen and pelvis: This is not the primary diagnostic tool for evaluating bone marrow function.
A bone marrow biopsy is the most direct way to assess for relapse.
The bone marrow biopsy confirms relapse of APL. What is the most appropriate initial treatment approach for relapsed APL in this patient?
a) 7+3 induction chemotherapy.
b) Allogeneic HSCT.
c) Arsenic trioxide (ATO) and ATRA with or without gemtuzumab ozogamicin.
d) Palliative care.
The correct answer is c) Arsenic trioxide (ATO) and ATRA with or without gemtuzumab ozogamicin.
 * Rationale:
   * ATO and ATRA remain highly effective in relapsed APL.
   * Gemtuzumab ozogamicin can be added in certain cases to improve response rates.
 * Why other options are incorrect:
   * a) 7+3 induction chemotherapy: While used in other AML subtypes, ATO and ATRA are preferred in relapsed APL due to their higher efficacy and lower toxicity.
   * b) Allogeneic HSCT: This is often considered for consolidation after achieving a second remission, not as initial salvage therapy.
   * d) Palliative care: This is considered when curative options are exhausted, but relapsed APL is often treatable.
ATO and ATRA remain the backbone of treatment for relapsed APL.
The patient achieves a second complete remission after salvage therapy with ATO and ATRA. What is the next step in his treatment?
a) Continue ATO and ATRA as maintenance therapy.
b) Proceed to allogeneic HSCT.
c) Initiate maintenance therapy with 6-mercaptopurine, methotrexate, and ATRA.
d) No further therapy is needed.
The correct answer is b) Proceed to allogeneic HSCT.
 * Rationale:
   * In relapsed APL, especially in patients who are eligible, allogeneic HSCT is often considered the preferred consolidation strategy.
   * It offers the potential for a graft-versus-leukemia effect, which can further reduce the risk of relapse.
   * Because the patient has relapsed, the risk of a second relapse is higher, making HSCT an important option.
 * Why other options are incorrect:
   * a) Continue ATO and ATRA as maintenance therapy: While these agents are effective, HSCT provides a more intensive consolidation approach in relapsed cases.
   * c) Initiate maintenance therapy with 6-mercaptopurine, methotrexate, and ATRA: While this is appropriate for initial diagnosis, HSCT is often preferred in relapsed cases.
   * d) No further therapy is needed: This would put the patient at high risk of a future relapse.
HSCT is a very important tool in relapse.
What is the most important long-term complication to monitor for in patients who have received ATO?
a) Renal dysfunction
b) Cardiac toxicity (QT prolongation)
c) Pulmonary fibrosis
d) Hepatic failure

The correct answer is b) Cardiac toxicity (QT prolongation).
 * Rationale:
   * Arsenic trioxide (ATO) is known to cause QT prolongation, which can lead to life-threatening arrhythmias.
   * Regular electrocardiogram (ECG) monitoring is crucial during and after ATO therapy.
 * Why other options are incorrect:
   * a) Renal dysfunction: While ATO can cause some renal toxicity, QT prolongation is a more significant concern.
   * c) Pulmonary fibrosis: This is not a common complication of ATO.
   * d) Hepatic failure: While ATO can cause some hepatic toxicity, QT prolongation is the most serious and common issue.
Monitoring for cardiac toxicity is vital for patients receiving ATO.

Session ID:
Assessment Domain

Session-38:
Rationale

Session ID:
Instructional Design and Pedagogical

Session-38:
The session's instructional design is of very high quality. The case is expertly scaffolded, progressing logically from initial diagnosis (Q1-2), induction therapy (Q3), management of a classic therapy-related complication (Q4-5), consolidation and maintenance (Q6-10), and finally through long-term monitoring, relapse, and salvage therapy (Q11-15). The quality of the embedded feedback is excellent; each rationale clearly explains the correct answer, addresses the distractors, and provides key clinical context. Instructions are unambiguous and the case unfolds intuitively. The only minor weakness is that learning objectives are not explicitly stated at the outset, though they are strongly implied by the comprehensive and logical structure of the case.

Session ID:
Clinical Authenticity and Content Fidelity

Session-38:
The session demonstrates impeccable clinical authenticity and content fidelity. Plausibility & Guideline Alignment: The clinical narrative is highly plausible, mirroring a typical patient journey with Acute Promyelocytic Leukemia (APL). All diagnostic steps, therapeutic choices (e.g., ATRA/ATO for induction, dexamethasone for differentiation syndrome, ATO-based salvage), monitoring strategies (PML-RARA by PCR), and complication management are perfectly aligned with current evidence-based guidelines from bodies like NCCN and ESMO. Pharmacotherapeutic Accuracy & Domain Specificity: The pharmacotherapeutic recommendations are optimal and precisely targeted to the APL subtype. Crucially, the simulation avoids any domain entanglement. It correctly identifies APL-specific therapy (ATRA/ATO) and appropriately includes treatments for other AML subtypes (like 7+3 or Venetoclax/Azacitidine) as incorrect distractors, thereby reinforcing the distinction. There are no content errors.

Session ID:
Modeled Clinical Reasoning

Session-38:
The simulation provides an expert model of the clinical reasoning process in APL management. Each question frames a distinct and relevant clinical problem, from diagnosis to relapse. The breadth of the answer choices is a key strength, featuring plausible and highly relevant distractors that effectively probe for nuanced understanding and common misconceptions. The overall flow demonstrates flawless prioritization and decision-makingconfirming the specific genetic subtype before initiating targeted therapy, managing acute toxicity, and escalating therapy appropriately upon relapse. The clinical outcomes described are realistic and serve to reinforce the correctness of the chosen pathway.

Session-39

Session ID:
LLM

Session-39:
Google AI (Gemini 2.0 Pro)

Session ID:
Session Transcript

Session-39:
Question 1:
Based on the initial clinical presentation and laboratory findings, which of the following is the most likely diagnosis?
A. Acute lymphoblastic leukemia (ALL)
B. Acute myeloid leukemia (AML)
C. Chronic myeloid leukemia (CML)
D. Myelodysplastic syndrome (MDS)
(B)
Elevated WBC with blasts: This is a hallmark of acute leukemia, indicating rapid proliferation of immature cells.
 * Anemia and thrombocytopenia: These are common findings in AML due to bone marrow infiltration by leukemic blasts, suppressing normal hematopoiesis.
 * Bone marrow biopsy with >20% blasts: This confirms the diagnosis of AML, as per the WHO classification.
 * Flow cytometry: The positive markers (CD33, CD34, CD117) are typical of AML.
 * Cytogenetic analysis: The t(15;17)(q22;q12); PML-RARA translocation is diagnostic for acute promyelocytic leukemia (APL), a subtype of AML.
Here's why the other options are less likely:
 * A. Acute lymphoblastic leukemia (ALL): While ALL also presents with elevated blasts, the immunophenotype and cytogenetics are different. ALL typically involves lymphoid markers.
 * C. Chronic myeloid leukemia (CML): CML is characterized by the Philadelphia chromosome (t(9;22)) and a more gradual onset, with a predominance of mature myeloid cells, not blasts.
 * D. Myelodysplastic syndrome (MDS): MDS is characterized by ineffective hematopoiesis, leading to cytopenias and a lower percentage of blasts, usually less than 20% in the bone marrow.

Question 2:
Given the cytogenetic finding of t(15;17)(q22;q12); PML-RARA, which AML subtype does this patient have?
A. AML with minimal differentiation (AML-M0)
B. AML with maturation (AML-M2)
C. Acute promyelocytic leukemia (APL)
D. Acute myelomonocytic leukemia (AMML)

(C)
t(15;17)(q22;q12); PML-RARA: This specific chromosomal translocation results in the fusion of the promyelocytic leukemia (PML) gene with the retinoic acid receptor alpha (RARA) gene. This fusion protein plays a crucial role in the pathogenesis of APL.
Question 3:
Given the diagnosis of APL, which of the following is the most immediate and critical concern in this patient's management?
A. Risk of infection due to neutropenia.
B. Risk of bleeding due to coagulopathy.
C. Risk of tumor lysis syndrome due to high blast count.
D. Risk of cardiac toxicity from chemotherapy.
(B)
APL-Associated Coagulopathy: APL is uniquely associated with a severe and potentially life-threatening coagulopathy called disseminated intravascular coagulation (DIC). This is due to the release of procoagulant factors from the leukemic

Question 4:
Which of the following is the most appropriate initial treatment for this patient with newly diagnosed APL?
A. 7+3 induction chemotherapy (cytarabine and daunorubicin)
B. All-trans retinoic acid (ATRA) and arsenic trioxide (ATO)
C. High-dose cytarabine
D. FLT3 inhibitor monotherapy
B
* Targeted Therapy: ATRA and ATO directly target the PML-RARA fusion protein, leading to differentiation and degradation of the leukemic promyelocytes.
* High Efficacy: This combination has demonstrated high rates of complete remission and improved overall survival in APL, particularly in low-to-intermediate risk APL.
* Reduced Toxicity: Compared to traditional chemotherapy, ATRA and ATO are generally associated with less myelosuppression and a lower risk of long-term complications
Question 5:
The patient's white blood cell count (WBC) increases significantly after starting ATRA, a phenomenon known as "differentiation syndrome." Which of the following is the most appropriate management for this complication?
A. Discontinue ATRA immediately.
B. Administer high-dose corticosteroids (e.g., dexamethasone).
C. Initiate prophylactic platelet transfusions.
D. Start 7+3 induction chemotherapy.
B
Mechanism of Action: Corticosteroids, particularly dexamethasone, effectively counteract the inflammatory cascade associated with differentiation syndrome.
* Clinical Evidence: Numerous studies have demonstrated the efficacy of corticosteroids in resolving the symptoms of differentiation syndrome, allowing for the continuation of ATRA and/or ATO therapy.
* Early Intervention: Prompt administration of corticosteroids at the first signs of differentiation syndrome is crucial to prevent severe complications.
Question 6:
Based on the patient's response to ATRA and ATO, which of the following best describes his current status?
A. Relapsed/refractory APL
B. Complete remission (CR)
C. Minimal residual disease (MRD) positivity
D. Stable disease
B
Bone marrow blasts <5%: This is a key criterion for CR in AML, including APL.
* Recovery of blood counts: The patient's WBC, Hb, and platelet counts are improving, indicating recovery of normal hematopoiesis.
* Absence of extramedullary disease: There's no mention of leukemia outside the bone marrow.
Here's why the other options are incorrect:
* A. Relapsed/refractory APL: This would involve an increase in blasts or failure to achieve CR.
* C. Minimal residual disease (MRD) positivity: While MRD testing is important, the current information indicates CR, not necessarily MRD positivity. MRD requires special testing.
* D. Stable disease: This implies no significant change, whereas the patient has achieved CR.
Question 7:
Given the patient's achievement of CR, which of the following is the most appropriate next step in his management?
A. Immediate allogeneic hematopoietic stem cell transplantation (alloHSCT).
B. Consolidation therapy with additional cycles of ATRA and ATO.
C. Maintenance therapy with low-dose chemotherapy.
D. Observation alone.
B
Post-Remission Therapy: Even after achieving CR, consolidation therapy is crucial to eliminate any remaining leukemic cells and reduce the risk of relapse.
* APL-Specific Consolidation: In APL, consolidation typically involves additional cycles of ATRA and ATO, which have demonstrated high efficacy in maintaining remission.
* Risk Stratification: The number of consolidation cycles and the intensity of therapy may be adjusted based on the patient's risk

stratification (e.g., low, intermediate, or high risk).
Here's why the other options are less appropriate:
 * A. Immediate allogeneic hematopoietic stem cell transplantation (alloHSCT): AlloHSCT is generally reserved for high-risk APL or patients who relapse after initial therapy. It's not typically the first-line consolidation approach for standard-risk APL.
 * C. Maintenance therapy with low-dose chemotherapy: Maintenance therapy is less common in APL compared to other AML subtypes. Consolidation with ATRA and ATO is the preferred approach.
 * D. Observation alone: Observation without further therapy would result in a very high risk of relapse. Consolidation is essential to maintain remission.
Question 8:
During consolidation therapy, the patient develops febrile neutropenia. Which of the following is the most appropriate initial management?
A. Start granulocyte colony-stimulating factor (G-CSF) alone.
B. Initiate broad-spectrum antibiotics after blood cultures are obtained.
C. Administer platelet transfusion.
D. Begin antifungal prophylaxis.
B
Post-Remission Therapy: Even after achieving CR, consolidation therapy is crucial to eliminate any remaining leukemic cells and reduce the risk of relapse.
 * APL-Specific Consolidation: In APL, consolidation typically involves additional cycles of ATRA and ATO, which have demonstrated high efficacy in maintaining remission.
 * Risk Stratification: The number of consolidation cycles and the intensity of therapy may be adjusted based on the patient's risk stratification (e.g., low, intermediate, or high risk).
Here's why the other options are less appropriate:
 * A. Immediate allogeneic hematopoietic stem cell transplantation (alloHSCT): AlloHSCT is generally reserved for high-risk APL or patients who relapse after initial therapy. It's not typically the first-line consolidation approach for standard-risk APL.
 * C. Maintenance therapy with low-dose chemotherapy: Maintenance therapy is less common in APL compared to other AML subtypes. Consolidation with ATRA and ATO is the preferred approach.
 * D. Observation alone: Observation without further therapy would result in a very high risk of relapse. Consolidation is essential to maintain remission.
Question 9:
The patient's blood cultures grow Escherichia coli, sensitive to meropenem. Which of the following is the most appropriate antibiotic management?
A. Continue the current broad-spectrum antibiotics.
B. Switch to meropenem monotherapy.
C. Add vancomycin to the current antibiotics.
D. Initiate antifungal therapy.
B
Directed Therapy: Once a specific pathogen and its sensitivities are identified, it's crucial to switch to a targeted antibiotic that provides optimal coverage with minimal toxicity.
 * Meropenem Efficacy: Meropenem is a broad-spectrum carbapenem that is highly effective against Escherichia coli, including many resistant strains.
 * De-escalation: De-escalating from broader-spectrum antibiotics to a narrower, targeted agent reduces the risk of antibiotic resistance and adverse effects.
Here's why the other options are less appropriate:
 * A. Continue the current broad-spectrum antibiotics: Continuing broader-spectrum antibiotics when a targeted agent is available is unnecessary and increases the risk of resistance.
 * C. Add vancomycin to the current antibiotics: Vancomycin is typically used for gram-positive infections, not for gram-negative infections like E. coli.
 * D. Initiate antifungal therapy: Antifungal therapy is not indicated unless there's evidence of a fungal infection.
Question 10:
After completing consolidation therapy, the patient's MRD is assessed using polymerase chain reaction (PCR) for PML-RARA. The result is negative. What is the clinical significance of this finding?
A. It indicates a high risk of relapse.
B. It confirms complete molecular remission.

C. It suggests the need for immediate alloHSCT.
D. It necessitates maintenance therapy with low-dose chemotherapy.
B
PCR Sensitivity: PCR for PML-RARA is a highly sensitive technique for detecting minimal residual disease in APL.
 * MRD Negativity: A negative PCR result indicates the absence of detectable PML-RARA transcripts, suggesting that there are no remaining leukemic cells. This is termed complete molecular remission.
 * Low Relapse Risk: Patients with negative MRD have a significantly lower risk of relapse compared to those with positive MRD.
Here's why the other options are incorrect:
 * A. It indicates a high risk of relapse: This is the opposite of what a negative MRD result signifies.
 * C. It suggests the need for immediate alloHSCT: AlloHSCT is not typically indicated for patients in complete molecular remission after consolidation therapy.
 * D. It necessitates maintenance therapy with low-dose chemotherapy: While maintenance therapy might be considered in some cases, a negative MRD result suggests that the current treatment has been effective.
Question 11:
Given the patient's sustained complete molecular remission, which of the following is the most appropriate long-term management strategy?
A. Regular monitoring of complete blood counts (CBC) and PML-RARA PCR.
B. Maintenance therapy with low-dose chemotherapy.
C. Allogeneic hematopoietic stem cell transplantation (alloHSCT).
D. Discontinuation of all monitoring and follow-up.
A
MRD Monitoring Importance: In APL, regular monitoring of PML-RARA by PCR is crucial for detecting early signs of relapse.
 * CBC Monitoring: Regular CBC monitoring helps assess hematologic recovery and detect any abnormalities.
 * Sustained Remission: Patients who achieve and maintain complete molecular remission have a favorable prognosis.
 * Less Intensive Therapy: Given the efficacy of ATRA and ATO, less intensive maintenance therapy is generally not necessary in these patients.
Here's why the other options are less appropriate:
 * B. Maintenance therapy with low-dose chemotherapy: While used in other AML subtypes, it's not standard for APL in sustained CR.
 * C. Allogeneic hematopoietic stem cell transplantation (alloHSCT): This is typically reserved for high-risk APL or patients who relapse.
 * D. Discontinuation of all monitoring and follow-up: This would be unsafe, as relapse is still possible, and monitoring is crucial for early detection.
Question 12:
Five years later, the patient develops recurrent fatigue and easy bruising. His CBC shows pancytopenia, and his PML-RARA PCR is positive. Which of the following best describes his current status?
A. Secondary MDS
B. Relapsed APL
C. Treatment-related AML
D. Chronic APL
B
PML-RARA Positivity: The reappearance of the PML-RARA fusion transcript is a definitive marker of APL relapse.
 * Pancytopenia: This indicates bone marrow involvement, consistent with relapsed leukemia.
 * Recurrent Symptoms: Fatigue and bruising are common symptoms of leukemia relapse.
Here's why the other options are less likely:
 * A. Secondary MDS: While secondary MDS can occur after leukemia treatment, the presence of PML-RARA specifically points to APL relapse.
 * C. Treatment-related AML: This refers to AML developing as a complication of prior chemotherapy, but the presence of PML-RARA again points to APL relapse.
 * D. Chronic APL: APL is an acute leukemia and does not have a chronic phase.
Question 13:
Which of the following is the most appropriate initial treatment for this patient with relapsed APL?
A. 7+3 induction chemotherapy.
B. Allogeneic hematopoietic stem cell transplantation (alloHSCT).
C. Arsenic trioxide (ATO) monotherapy or ATO with ATRA.

D. Palliative care.
C
High Efficacy: ATO, with or without ATRA, has demonstrated high response rates in relapsed APL, even in patients who were previously treated with these agents.
 * Targeted Therapy: These agents directly target the PML-RARA fusion protein, which remains the key driver of APL pathogenesis.
 * AlloHSCT as Consolidation: Allogeneic hematopoietic stem cell transplantation (alloHSCT) may be considered after achieving a second complete remission with ATO-based therapy, especially for high-risk patients.
Here's why the other options are less appropriate:
 * A. 7+3 induction chemotherapy: While used in other AML subtypes, it's less effective than ATO-based regimens in relapsed APL.
 * B. Allogeneic hematopoietic stem cell transplantation (alloHSCT): While a potential option, it's typically considered after achieving a second remission, not as initial therapy.
 * D. Palliative care: This is only considered when curative options are no longer feasible.
Question 14:
The patient achieves a second complete remission with ATO and ATRA. Which of the following is the most appropriate next step in his management?
A. Maintenance therapy with low-dose chemotherapy.
B. Allogeneic hematopoietic stem cell transplantation (alloHSCT).
C. Observation alone.
D. Consolidation with additional cycles of ATO and ATRA.
B
Relapsed APL and AlloHSCT:
   * For patients with relapsed APL who achieve a second complete remission, especially those who are younger and fit, alloHSCT is considered the most potentially curative option.
   * It offers the chance for long-term disease control and improved survival.
 * Post-Remission Consolidation:
   * After achieving a second CR, the goal is to consolidate that remission with the most effective means available. In this case, that is considered to be AlloHSCT.
Here's why the other options are less appropriate:
 * A. Maintenance therapy with low-dose chemotherapy:
   * While maintenance therapy is used in some AML subtypes, it's not the standard approach for relapsed APL after achieving a second CR. AlloHSCT is preferred.
 * C. Observation alone:
   * Observation alone would result in a very high risk of relapse. AlloHSCT provides a more definitive consolidation strategy.
 * D. Consolidation with additional cycles of ATO and ATRA:
   * While consolidation with ATO and ATRA was effective in achieving the second remission, alloHSCT is considered a better way to solidify that remission, and provide a higher chance of long term survival.
Following alloHSCT, the patient develops graft-versus-host disease (GVHD). Which of the following is the most appropriate initial management?
A. High-dose corticosteroids.
B. Cyclophosphamide.
C. Methotrexate.
D. Tacrolimus monotherapy.
A
First-Line Therapy: Corticosteroids are the standard first-line treatment for both acute and chronic GVHD.
 * Immunosuppression: They effectively suppress the immune response that drives GVHD.
 * Rapid Action: Corticosteroids can provide rapid relief of GVHD symptoms.
Here's why the other options are less appropriate as initial management:
 * B. Cyclophosphamide: While cyclophosphamide is an immunosuppressant, it's not typically used as first-line therapy for GVHD. It may be used in steroid-refractory cases.
 * C. Methotrexate: Methotrexate is also an immunosuppressant, but it's not the primary treatment for acute GVHD. It might be used in combination with other agents or for chronic GVHD.
 * D. Tacrolimus monotherapy: Tacrolimus is an immunosuppressant often used for GVHD prophylaxis, but high-dose corticosteroids are the first-line treatment for established acute GVHD.

Session ID:
Assessment Domain

Session-39:
Rationale

Session ID:
Instructional Design and Pedagogical

Session-39:
The session's instructional design is very strong. The case is expertly scaffolded, progressing logically from diagnosis and initial management priorities (APL-associated coagulopathy) through induction, consolidation, complication management (differentiation syndrome, febrile neutropenia), and long-term follow-up including relapse and post-transplant care. Instructions are exceptionally clear. The embedded feedback is generally detailed and accurate, but the score is slightly reduced because the rationale for Question 8 is an erroneous copy-paste of the rationale from Question 7, a quality control lapse. Learning objectives are not explicitly stated but are clearly implied by the strong structure, warranting a score of 4.

Session ID:
Clinical Authenticity and Content Fidelity

Session-39:
The clinical narrative is highly realistic and follows a plausible APL patient journey from diagnosis through relapse and transplant. Most therapeutic recommendations align well with current guidelines for APL management (ATRA/ATO, consolidation approaches, relapse management). However, there is a critical content error in Question 8 where the rationale completely mismatches the question - the question asks about febrile neutropenia management but provides the rationale for consolidation therapy from Question 7. This represents a significant content accuracy failure that could confuse learners about appropriate infection management in neutropenic patients. Unlike the example provided, this session does not contain domain entanglement errors - all content remains appropriately within the APL/AML domain.

Session ID:
Modeled Clinical Reasoning

Session-39:
The simulation expertly models the clinical reasoning process for managing an APL patient. Each question frames a relevant and well-defined clinical problem. The multiple-choice options provide an excellent breadth of plausible answers and key distractors, effectively testing the user's nuanced understanding (e.g., distinguishing APL-specific therapy from standard AML induction). The narrative demonstrates flawless prioritization and decision-making, such as identifying coagulopathy as the immediate risk and recommending allo-HSCT only at the appropriate time (consolidation for relapse). The clinical outcomes described are realistic and instructionally valuable, reinforcing the consequences of each therapeutic choice.

Session-40

Session ID:
LLM

Session-40:
Google AI (Gemini 2.0 Pro)

Session ID:
Session Transcript

Session-40:
Okay, let's begin with Phase 1 of our AML case.
Phase 1: Initial Presentation and Diagnostic Workup
Patient Presentation:
Mr. James Anderson is a 67-year-old male who presents to his primary care physician with a one-month history of increasing fatigue, shortness of breath on exertion, and easy bruising. He denies any fever, night sweats, or bone pain. His past medical history is significant for well-controlled hypertension and hyperlipidemia. He is a former smoker (quit 15 years ago) and enjoys gardening as a hobby.
Initial Laboratory Findings:
 * White blood cell (WBC) count: 35,000/L (Reference range: 4,000-10,000/L)
 * Hemoglobin: 8.5 g/dL (Reference range: 13.5-17.5 g/dL)
 * Platelet count: 45,000/L (Reference range: 150,000-400,000/L)
 * Peripheral blood smear: Shows 40% blasts, normocytic normochromic anemia, and thrombocytopenia.
Based on this initial presentation and peripheral blood smear, what is the most likely next step in the diagnostic workup for Mr. Anderson?
A) Initiate broad-spectrum antibiotics due to potential infection.
B) Schedule an outpatient follow-up appointment in one week to monitor his symptoms.
C) Proceed with a bone marrow biopsy for further evaluation.
D) Order a comprehensive metabolic panel to assess for organ dysfunction.
Please select the best answer (A, B, C, or D).

 Short Answer: C) Proceed with a bone marrow biopsy for further evaluation.
 * Why Not Others:
   * A) Antibiotics: Infection is a risk, but the high blast count suggests leukemia is the priority for diagnosis.
   * B) Outpatient follow-up: The abnormal blood counts require urgent investigation, not just monitoring.
   * D) Metabolic panel: Useful later, but the immediate need is to diagnose the hematologic abnormality.

Question 2:
Mr. Anderson undergoes a bone marrow biopsy. The results show hypercellular marrow with 65% blasts. Flow cytometry confirms the blasts are myeloid lineage. Cytogenetic analysis reveals a normal karyotype (CN-AML). Molecular testing is positive for an FLT3-ITD mutation (internal tandem duplication) with a high allelic ratio.
Based on the WHO 2022 classification and the European LeukemiaNet (ELN) 2022 risk stratification, which of the following best describes Mr. Anderson's AML?
A) AML with myelodysplasia-related changes, ELN favorable risk.
B) AML with defining genetic abnormalities (FLT3-ITD), ELN intermediate risk.
C) AML, not otherwise specified, ELN adverse risk.
D) Therapy-related AML, ELN adverse risk.

C is the answer
Short Answer: C) AML, not otherwise specified, ELN adverse risk. (Correction from previous error)

* Why Not Others:
  * A) Myelodysplasia-related changes: No evidence of prior MDS or specific dysplastic features.
  * B) ELN intermediate risk: High allelic ratio FLT3-ITD in CN-AML is adverse risk.
  * D) Therapy-related AML: No history of prior chemotherapy or radiation.

Question 3:
Given Mr. Anderson's age (67 years), comorbidities (well-controlled hypertension and hyperlipidemia), newly diagnosed FLT3-ITD positive AML with ELN adverse risk, and an ECOG performance status of 1 (fully ambulatory and able to carry out light work), which of the following induction therapy regimens would be the most appropriate initial treatment?
A) Standard "7+3" (cytarabine for 7 days and daunorubicin for 3 days).
B) Low-dose cytarabine (LDAC) monotherapy.
C) Venetoclax in combination with a hypomethylating agent (HMA) such as azacitidine or decitabine.
D) Midostaurin in combination with standard "7+3".

D
* Short Answer: D) Midostaurin in combination with standard "7+3".
* Why Not Others:
  * A) Standard "7+3": Doesn't target the adverse-risk FLT3-ITD mutation.
  * B) Low-dose cytarabine: Generally for less fit patients, not ideal for adverse-risk.
  * C) Venetoclax + HMA: More common for older/unfit without FLT3 target.

Question 4:
Mr. Anderson begins induction therapy with midostaurin and "7+3". On day 5 of chemotherapy, he develops a fever of 38.5C (101.3F) and his absolute neutrophil count (ANC) is 0.1 x 10^9/L. Blood cultures are drawn, and he is started on empiric broad-spectrum antibiotics. Which of the following is the most appropriate next step in his management?
A) Add an antifungal agent to his antibiotic regimen.
B) Administer granulocyte colony-stimulating factor (G-CSF).
C) Transfuse platelets to maintain a platelet count above 10,000/L.
D) Discontinue midostaurin until his fever resolves.

* Short Answer: B) Administer granulocyte colony-stimulating factor (G-CSF).
* Why Not Others:
  * A) Antifungal: Usually for persistent fever or suspected fungal infection, not first-line for new febrile neutropenia.
  * C) Platelet transfusion: Indicated for low platelets or bleeding, not directly for fever.
  * D) Discontinue midostaurin: No immediate indication to stop a key treatment due to fever alone.

Question 5:
Mr. Anderson completes his induction therapy with midostaurin and "7+3". His bone marrow biopsy on day +14 shows persistent disease with 30% blasts. A repeat bone marrow biopsy on day +28 still shows 25% blasts. This indicates refractory AML.
Considering his initial diagnosis of FLT3-ITD positive AML (ELN adverse risk) and the lack of response to initial induction, what is the most appropriate next step in his treatment?
A) Initiate palliative care.
B) Start a second course of the same induction chemotherapy (re-induction).
C) Enroll him in a clinical trial investigating novel therapies for relapsed/refractory AML.
D) Begin treatment with single-agent azacitidine.
.

Short Answer: C) Enroll him in a clinical trial investigating novel therapies for relapsed/refractory AML.
* Why Not Others:
  * A) Palliative care: May be considered later, but further active treatment options exist.

* B) Re-induction with same chemo: Unlikely to work after initial failure.
* D) Single-agent azacitidine: Generally not preferred for relapsed/refractory in fit patients with adverse risk.

Okay, let's move on to Phase 2: Post-Remission Therapy.

Phase 2: Post-Remission Therapy

Following enrollment in a clinical trial with a novel FLT3 inhibitor-based regimen, Mr. Anderson achieved a complete remission (CR) as confirmed by a bone marrow biopsy showing <5% blasts and recovery of peripheral blood counts (ANC >1.0 x 10^9/L, platelets >100 x 10^9/L, hemoglobin >11 g/dL). Measurable residual disease (MRD) assessment by multiparameter flow cytometry is negative (<0.01% blasts).

Now that Mr. Anderson is in first complete remission (CR1) after salvage therapy for refractory AML, what is the most appropriate next step in his management to prevent relapse and improve long-term survival?

A) Observation with regular monitoring of blood counts and MRD.
B) Consolidation chemotherapy with high-dose cytarabine.
C) Allogeneic hematopoietic stem cell transplantation (allo-HSCT).
D) Maintenance therapy with the novel FLT3 inhibitor he received in the clinical trial.
.

Short Answer: C) Allogeneic hematopoietic stem cell transplantation (allo-HSCT).

* Why Not Others:
  * A) Observation: Very high relapse risk in adverse-risk AML after salvage.
  * B) Consolidation chemotherapy: May not be enough to overcome high relapse risk.
  * D) Maintenance FLT3 inhibitor: May be considered after transplant or if transplant not feasible, but transplant is preferred for high-risk.

Question 7:

Prior to proceeding with allogeneic hematopoietic stem cell transplantation (allo-HSCT) for Mr. Anderson, a comprehensive pre-transplant evaluation is necessary. Which of the following is the most critical component of this evaluation that directly impacts transplant eligibility and the choice of conditioning regimen?

A) Assessment of his social support system and financial resources.
B) Comprehensive cardiac and pulmonary function testing.
C) Human Leukocyte Antigen (HLA) typing of Mr. Anderson and potential donors.
D) Evaluation of his measurable residual disease (MRD) status by next-generation sequencing (NGS).

Short Answer: C) Human Leukocyte Antigen (HLA) typing of Mr. Anderson and potential donors.

* Why Not Others:
  * A) Social/financial: Important later, but first need a suitable donor.
  * B) Cardiac/pulmonary: Assessed after donor identification to check transplant fitness.
  * D) MRD: Important for prognosis, but transplant hinges on donor availability.

Question 8:

Mr. Anderson has a matched related donor identified (his brother). His cardiac and pulmonary function tests are within acceptable limits for transplant. He is currently in complete remission with negative MRD. Considering his age (67 years) and prior intensive chemotherapy for both induction and salvage, which type of conditioning regimen would be most appropriate prior to his allogeneic stem cell transplant?

A) Myeloablative conditioning (MAC) with high-dose busulfan and cyclophosphamide.
B) Reduced-intensity conditioning (RIC) with fludarabine and melphalan.
C) Myeloablative conditioning (MAC) with total body irradiation (TBI) and cyclophosphamide.
D) Myeloablative conditioning (MAC) with high-dose cytarabine and etoposide.

Short Answer: B) Reduced-intensity conditioning (RIC) with fludarabine and melphalan.

* Why Not Others:

* A, C, D) Myeloablative conditioning (MAC): Higher risk of toxicity in older, previously treated patients.

Question 9:

Mr. Anderson undergoes an allogeneic stem cell transplant with a reduced-intensity conditioning regimen. Approximately 30 days post-transplant, he develops a diffuse maculopapular rash, diarrhea, and elevated liver enzymes (bilirubin, alkaline phosphatase, and transaminases). What is the most likely cause of these complications?

A) Cytomegalovirus (CMV) reactivation.

B) Hepatic veno-occlusive disease (VOD).

C) Acute graft-versus-host disease (aGVHD).

D) Primary graft failure.

Short Answer: C) Acute graft-versus-host disease (aGVHD).

* Why Not Others:
  * A) CMV reactivation: Less typical presentation with rash and diarrhea this early.
  * B) Hepatic VOD: Presents more with jaundice and fluid retention, less with rash and diarrhea.
  * D) Graft failure: Presents as low blood counts, not organ-specific symptoms.

Question 10:

Based on the diagnosis of acute graft-versus-host disease (aGVHD) in Mr. Anderson (diffuse rash, diarrhea, elevated liver enzymes), what is the most appropriate initial treatment approach?

A) Increase the dose of his prophylactic immunosuppressants (e.g., tacrolimus or cyclosporine).

B) Start broad-spectrum antibiotics and antiviral medications.

C) Initiate high-dose systemic corticosteroids (e.g., methylprednisolone).

D) Perform a liver biopsy to confirm the diagnosis and grade the severity.

t.

Short Answer: C) Initiate high-dose systemic corticosteroids (e.g., methylprednisolone).

* Why Not Others:
  * A) Increase prophylactic immunosuppressants: May be done, but corticosteroids are the first-line treatment for established aGVHD.
  * B) Antibiotics/antivirals: Supportive, but don't treat the underlying GVHD.
  * D) Liver biopsy first: Biopsy is often done, but treatment is usually started based on clinical suspicion.

Okay, let's proceed to Phase 3: Long-Term Management.

Phase 3: Long-Term Management

Mr. Anderson's acute GVHD initially responded to high-dose corticosteroids. The rash and diarrhea improved, and his liver enzymes trended downwards. However, he required a prolonged course of steroids and developed steroid-refractory chronic GVHD involving his skin (scleroderma-like changes) and joints (limited mobility). He is now 18 months post-transplant and in continued complete remission for his AML.

Considering his steroid-refractory chronic GVHD, which of the following is the most appropriate next step in his long-term management?

A) Continue high-dose corticosteroids indefinitely to control symptoms.

B) Initiate treatment with a second-line agent for chronic GVHD, such as ruxolitinib or ibrutinib.

C) Discontinue all immunosuppression to allow his immune system to recover fully.

D) Begin tapering corticosteroids rapidly to minimize long-term side effects.

Short Answer: B) Initiate treatment with a second-line agent for chronic GVHD, such as ruxolitinib or ibrutinib.

* Why Not Others:
  * A) Continue high-dose steroids: Leads to significant long-term side effects.
  * C) Discontinue immunosuppression: High risk of GVHD flare.
  * D) Rapid steroid taper: Likely to worsen GVHD.

Question 12:

Mr. Anderson is now 2 years post-allogeneic stem cell transplant and continues to be in complete remission from his AML. He is receiving ongoing treatment for chronic GVHD with a second-line agent and a low dose of corticosteroids. What is the most

appropriate strategy for long-term monitoring of his AML relapse risk?
A) Monthly complete blood counts (CBC) with differential.
B) Bone marrow biopsy with MRD assessment by flow cytometry every 6 months.
C) Regular monitoring of chimerism studies (donor vs. recipient DNA in blood or bone marrow) and considering bone marrow evaluation if chimerism decreases.
D) Annual PET-CT scans to screen for extramedullary relapse.

Short Answer: C) Regular monitoring of chimerism studies (donor vs. recipient DNA in blood or bone marrow) and considering bone marrow evaluation if chimerism decreases.
 * Why Not Others:
   * A) Monthly CBC: May not detect early marrow relapse.
   * B) Routine bone marrow biopsy: Invasive and not always necessary.
   * D) Annual PET-CT: More for extramedullary relapse, less common as primary site.

Question 13:
Mr. Anderson has been receiving ruxolitinib for steroid-refractory chronic GVHD for the past 6 months. While his skin and joint symptoms have improved, his recent blood work shows a new onset of anemia (hemoglobin decreased from 11.5 g/dL to 9.0 g/dL) and thrombocytopenia (platelet count decreased from 150 x 10^9/L to 75 x 10^9/L). His white blood cell count and differential remain stable. What is a potential hematologic toxicity associated with ruxolitinib that could be contributing to these findings?
A) Hemolytic anemia.
B) Myelodysplasia.
C) Myelosuppression.
D) Thrombotic microangiopathy.

Short Answer: C) Myelosuppression.
 * Why Not Others:
   * A) Hemolytic anemia: Not a typical primary side effect of ruxolitinib.
   * B) Myelodysplasia: Not an acute toxicity of ruxolitinib.
   * D) Thrombotic microangiopathy: Anemia not clearly hemolytic, no mention of organ damage.

Question 14:
Given Mr. Anderson's long-term immunosuppression with a second-line agent for chronic GVHD and a low dose of corticosteroids, what is the most appropriate long-term strategy for preventing opportunistic infections?
A) Discontinue all prophylactic antimicrobial medications to reduce the risk of drug interactions.
B) Continue antiviral prophylaxis (e.g., acyclovir or valacyclovir) indefinitely.
C) Implement targeted antimicrobial prophylaxis based on specific risk factors and local epidemiology.
D) Prescribe broad-spectrum antibacterial and antifungal prophylaxis for the duration of immunosuppression.

Short Answer: C) Implement targeted antimicrobial prophylaxis based on specific risk factors and local epidemiology.
 * Why Not Others:
   * A) Discontinue all prophylaxis: High risk of opportunistic infections.
   * B) Indefinite antiviral prophylaxis: Often part of targeted approach, but not the entire strategy.
   * D) Broad-spectrum prophylaxis: Risk of resistance and side effects.

Question 15:
Mr. Anderson is 2 years post-allogeneic stem cell transplant and in remission from his AML. He is managing chronic GVHD with ongoing medication and regular clinic visits. He expresses feelings of anxiety about potential relapse, fatigue related to his chronic condition, and frustration with the limitations imposed by his GVHD. What is the most important aspect of his ongoing care to address these psychosocial challenges?
A) Routine monitoring of his blood counts and MRD to provide reassurance about his remission status.
B) Encouraging him to focus on his physical recovery and adhere strictly to his medication regimen.
C) Providing access to psychosocial support services, including counseling, support groups, and resources for managing fatigue and chronic illness.

D) Reassuring him that his risk of relapse decreases significantly over time.

Short Answer: C) Providing access to psychosocial support services, including counseling, support groups, and resources for managing fatigue and chronic illness.
 * Why Not Others:
   * A) Routine monitoring reassurance: Doesn't directly address emotional and practical challenges.
   * B) Focus on physical recovery/adherence: Important but neglects psychosocial needs.
   * D) Reassurance about relapse risk: May not be sufficient for individual anxieties.

Session ID:
Assessment Domain

Session-40:
Rationale

Session ID:
Instructional Design and Pedagogical

Session-40:
The session's instructional design is highly effective. The clinical case is expertly scaffolded, guiding the learner through a logical and realistic patient journey from initial diagnosis and risk stratification (Q1-2), through induction therapy and its complications (Q3-4), to complex challenges like refractory disease (Q5), allogeneic transplant (Q6-10), and long-term survivorship including chronic GVHD and psychosocial issues (Q11-15). This progression seamlessly builds complexity. Instructions are exceptionally clear. The embedded feedback is mostly strong, providing concise and correct rationales. However, the score is slightly reduced because the learning objectives are not explicitly stated upfront, and the feedback for Question 4 contains a significant clinical error, weakening the overall quality of the instructional rationales.

Session ID:
Clinical Authenticity and Content Fidelity

Session-40:
The clinical narrative is outstandingly plausible and mirrors an authentic, complex AML case. However, the simulation contains a significant pharmacotherapeutic error that impacts its fidelity. In Question 4, the recommendation to administer G-CSF on day 5 of induction chemotherapy for febrile neutropenia is a notable deviation from standard AML guidelines (e.g., NCCN), which generally advise against the routine use of growth factors during induction due to concerns about stimulating leukemic blasts. This represents a suboptimal and questionable choice, resulting in a low Pharmacotherapeutic Accuracy score. While the rest of the therapeutic recommendations (e.g., Midostaurin, RIC allo-HSCT, Ruxolitinib for GVHD) align well with guidelines, this single error lowers the overall guideline alignment score. Critically, the session is free from domain entanglement; all concepts and therapies are appropriate to the AML disease space, earning a perfect score for that criterion. THE CORRECT CLASSIFICATION IS INTERMEDIATE RISK.

Session ID:
Modeled Clinical Reasoning

Session-40:
The simulation provides an expert model of the clinical reasoning process in managing AML. Each question frames a distinct and relevant clinical problem, from diagnostic urgency to managing treatment toxicities. The multiple-choice options are exceptionally well-constructed, including the correct answer alongside highly plausible distractors that effectively test for nuanced understanding (e.g., distinguishing between different induction regimens or transplant conditioning intensities). The overall decision-making strategy is sound, correctly prioritizing key steps like securing a definitive diagnosis, proceeding to transplant for high-risk disease, and adapting therapy based on patient factors like age and performance status. Per the rubric, this section scores the reasoning strategy itself, which remains sound even with the content error in Question 4. Finally, the clinical outcomes described after each decision are realistic and highly instructive, reinforcing the learning from each choice.

Session-41

Session ID:
LLM

Session-41:
Google AI (Gemini 2.0 Pro)

Session ID:
Session Transcript

Session-41:
Question 1:

Based on the initial presentation and laboratory findings, which of the following is the MOST appropriate next step in the diagnostic workup?

A) Initiate broad-spectrum antibiotics and monitor for improvement.
B) Order a bone marrow biopsy and aspiration.
C) Transfuse packed red blood cells to correct the anemia.
D) Prescribe a course of oral corticosteroids to address potential immune-mediated thrombocytopenia.

That is correct, [REDACTED]]!

Feedback:

Your answer, B) Order a bone marrow biopsy and aspiration, is the most appropriate next step.

Why it's correct: The presence of blasts on the peripheral blood smear, along with pancytopenia (low WBC, hemoglobin, and platelets), strongly suggests a hematologic malignancy, most likely Acute Myeloid Leukemia (AML) given the patient's age. A bone marrow biopsy and aspiration are essential for confirming the diagnosis, determining the blast percentage, and performing cytogenetic and molecular studies necessary for risk stratification and treatment planning, according to ESMO guidelines.

Why the other options are incorrect:

A) Initiate broad-spectrum antibiotics and monitor for improvement: While the patient has a history of recent infections, the pancytopenia is more indicative of a bone marrow disorder than an active infection requiring immediate broad-spectrum antibiotics. The focus should be on diagnosing the underlying cause of the pancytopenia.

C) Transfuse packed red blood cells to correct the anemia: Transfusion may be necessary for symptomatic anemia, but it does not address the underlying cause. Furthermore, it could potentially obscure the blast count in subsequent blood samples if performed before diagnostic tests.

D) Prescribe a course of oral corticosteroids to address potential immune-mediated thrombocytopenia: While immune-mediated thrombocytopenia is a possibility, the presence of blasts on the peripheral smear makes a bone marrow disorder much more likely. Corticosteroids would not be appropriate without a confirmed diagnosis.
Question 2:

The bone marrow biopsy and aspiration results are now available:

Bone Marrow Blast Percentage: 65%

Cytogenetics: 46, XY, normal karyotype

Molecular Studies:

FLT3-ITD: Negative

NPM1: Positive

IDH1: Negative

CEBPA: Negative

Based on these results, according to the 2022 European LeukemiaNet (ELN) risk stratification, which risk category does Mr. Smith MOST likely fall into?

A) Favorable risk
B) Intermediate risk
C) Adverse risk
D) Undefined risk
You are correct, [REDACTED]]! Mr. Smith most likely falls into the B) Intermediate risk category according to the 2022 ELN risk stratification.

Feedback:

Why it's correct: The 2022 ELN risk stratification considers cytogenetic and molecular markers. The presence of an NPM1 mutation without a FLT3-ITD mutation, and a normal karyotype, classifies the patient as intermediate risk.

Why the other options are incorrect:

A) Favorable risk: Favorable risk typically includes patients with specific cytogenetic abnormalities like t(15;17) in APL, or biallelic CEBPA mutations.

C) Adverse risk: Adverse risk includes abnormalities such as inv(3), t(6;9), t(v;11), -5, -7, or mutations like TP53, RUNX1, ASXL1.

D) Undefined risk: This category is for patients with genetic profiles that are not clearly defined as favorable, intermediate, or adverse. While not every mutation was tested, the absence of mutations associated with adverse risk, and the presence of NPM1 alone, moves him into intermediate.
Question 3:

Considering Mr. Smiths age (67 years), ELN risk stratification (intermediate), and comorbidities (hypertension, type 2 diabetes), which of the following induction therapy regimens is the MOST appropriate initial treatment option?

A) Standard "7+3" (Cytarabine 100mg/m2/day x 7 days and Daunorubicin 60mg/m2/day x 3 days)
B) High-dose Cytarabine (HiDAC)
C) CPX-351 (Cytarabine and Daunorubicin Liposome for Injection)
D) Venetoclax in combination with Azacitidine
You are correct, [REDACTED]]!

Feedback:

Your answer, D) Venetoclax in combination with Azacitidine is the MOST appropriate initial treatment option.

Why it's correct: For older adults (typically >60 years) with AML who are not candidates for intensive chemotherapy, venetoclax in combination with a hypomethylating agent (HMA) such as azacitidine or decitabine is a guideline-recommended option (ESMO). This combination has shown improved overall survival compared to HMA alone in this patient population. Given his age and comorbidities,

Mr. Smith is likely not an ideal candidate for intensive chemotherapy.

Why the other options are incorrect:

A) Standard "7+3" (Cytarabine 100mg/m2/day x 7 days and Daunorubicin 60mg/m2/day x 3 days): While 7+3 is a standard induction regimen for younger, fit patients with AML, it can be poorly tolerated by older patients with comorbidities due to its intensity.

B) High-dose Cytarabine (HiDAC): HiDAC is typically used for relapsed/refractory AML or for consolidation therapy in patients with certain favorable-risk cytogenetics. It's not a standard induction regimen, especially in older patients.

C) CPX-351 (Cytarabine and Daunorubicin Liposome for Injection): CPX-351 is approved for adults with newly diagnosed therapy-related AML (t-AML) or AML with myelodysplasia-related changes (AML-MRC). The case doesn't indicate either of these conditions
Question 4:

Mr. Smith is initiated on venetoclax and azacitidine. Which of the following is the MOST important initial monitoring parameter specifically related to the use of venetoclax?

A) Liver function tests (LFTs)
B) Complete blood counts (CBC) with differential
C) Serum electrolytes, including potassium, phosphate, calcium, and uric acid
D) Cardiac monitoring (ECG) for QTc prolongation
Correct again, [REDACTED]]!

Feedback:

Your answer, C) Serum electrolytes, including potassium, phosphate, calcium, and uric acid, is the MOST important initial monitoring parameter specifically related to venetoclax.

Why it's correct: Venetoclax can cause rapid tumor lysis syndrome (TLS), especially during the initial ramp-up phase. Monitoring electrolytes (potassium, phosphate, calcium, uric acid) is crucial to detect and manage TLS. Prophylactic measures (hydration, allopurinol/rasburicase) are also essential.

Why the other options are incorrect:

A) Liver function tests (LFTs): While LFTs are important to monitor in general, they are not the primary concern with the initiation of venetoclax.

B) Complete blood counts (CBC) with differential: CBC monitoring is important for assessing response to therapy and managing myelosuppression, but it's not the most immediate concern compared to TLS risk with venetoclax initiation.

D) Cardiac monitoring (ECG) for QTc prolongation: While some medications used in AML can cause QTc prolongation, it's not a primary concern with venetoclax itself.
Question 5:

Mr. Smith develops nausea and vomiting shortly after starting azacitidine. Which of the following antiemetic regimens is MOST appropriate for managing his chemotherapy-induced nausea and vomiting (CINV)?

A) Ondansetron alone
B) Prochlorperazine alone
C) Aprepitant in combination with ondansetron and dexamethasone
D) Metoclopramide alone
Correct!

Feedback:

Your answer, C) Aprepitant in combination with ondansetron and dexamethasone, is the MOST appropriate antiemetic regimen.

Why it's correct: Azacitidine is considered moderately emetogenic. Therefore, guideline recommendations (MASCC/ESMO) suggest a combination of a 5-HT3 receptor antagonist (ondansetron), a neurokinin-1 (NK1) receptor antagonist (aprepitant), and dexamethasone for optimal CINV control.

Why the other options are incorrect:

A) Ondansetron alone: While ondansetron is a good antiemetic, it's often insufficient as monotherapy for moderately emetogenic chemotherapy.

B) Prochlorperazine alone: Prochlorperazine is a dopamine antagonist and can be used for breakthrough nausea, but it's not a first-line agent for preventing CINV from moderately emetogenic chemotherapy and has more side effects.

D) Metoclopramide alone: Similar to prochlorperazine, metoclopramide is a dopamine antagonist and is more suitable for breakthrough nausea or gastroparesis. It's not a first-line preventative agent.

That concludes Phase 1, [REDACTED]]! You have successfully navigated the initial presentation and diagnostic workup of Mr. Smith's case. You have shown strong knowledge of AML diagnosis, risk stratification, and initial treatment considerations
Great. Let's move on to Phase 2: Induction Therapy and Supportive Care.

Phase 2: Induction Therapy and Supportive Care

Mr. Smith is tolerating the venetoclax and azacitidine regimen reasonably well. He experienced some nausea, which was managed effectively with the aprepitant, ondansetron, and dexamethasone regimen. His electrolytes were closely monitored, and no significant tumor lysis syndrome developed. He did experience some myelosuppression, requiring a platelet transfusion.

On Day 28 of treatment, a bone marrow aspirate is performed to assess response.

Question 6:

The bone marrow aspirate on Day 28 shows 3% blasts. According to the International Working Group (IWG) criteria for AML, how would you classify Mr. Smiths response to induction therapy?

A) Complete Remission (CR)
B) Complete Remission with Incomplete Hematologic Recovery (CRi)
C) Partial Remission (PR)
D) Refractory Disease
Correct!

Feedback:

Your answer, B) Complete Remission with Incomplete Hematologic Recovery (CRi), is the correct classification.

Why it's correct: According to the IWG criteria, CRi is defined as meeting all the criteria for CR except for incomplete recovery of peripheral blood counts (absolute neutrophil count < 1.0 x 10^9/L or platelet count < 100 x 10^9/L). A blast percentage of <5% in the bone marrow meets the bone marrow criteria for remission, but the patient is likely cytopenic from the treatment.

Why the other options are incorrect:

A) Complete Remission (CR): Requires <5% blasts in the bone marrow and complete recovery of peripheral blood counts (ANC 1.0 x 10^9/L and platelets 100 x 10^9/L).

C) Partial Remission (PR): Requires a decrease in bone marrow blasts by at least 50% but still >5% blasts.

D) Refractory Disease: Implies failure to achieve at least a PR.

Let's move on.

Question 7:

Despite achieving CRi, Mr. Smith remains transfusion-dependent for both red blood cells and platelets. His oncologist is considering adding growth factors to help improve his blood counts. Which of the following growth factors is MOST appropriate in this setting?

A) Filgrastim (G-CSF)
B) Sargramostim (GM-CSF)
C) Eltrombopag
D) Romiplostim

You're on a roll, [REDACTED]]! That is correct.

Feedback:

Your answer, C) Eltrombopag, is the most appropriate growth factor in this setting.

Why it's correct: Eltrombopag is a thrombopoietin receptor agonist (TPO-RA) that can stimulate megakaryocyte proliferation and platelet production. It is more appropriate in this setting for the thrombocytopenia.

Why the other options are incorrect:

A) Filgrastim (G-CSF): Filgrastim primarily stimulates neutrophil production and is not effective for thrombocytopenia. It may increase the risk of leukemic relapse and is generally avoided unless there is a documented infection with neutropenia.

B) Sargramostim (GM-CSF): Similar to filgrastim, sargramostim primarily stimulates neutrophil production and may have more side effects. It also carries a risk of stimulating leukemic cell proliferation.

D) Romiplostim: Romiplostim is also a TPO-RA, however, it's administered subcutaneously and is more commonly used in immune thrombocytopenia (ITP).

Question 8:

Mr. Smith develops a fever of 38.5C (101.3F) and an absolute neutrophil count (ANC) of 0.3 x 10^9/L. Blood cultures are drawn, and the patient is started on empiric antibiotics. Which of the following empiric antibiotic regimens is the MOST appropriate initial choice for this patient with febrile neutropenia?

A) Oral ciprofloxacin
B) Intravenous vancomycin
C) Intravenous cefepime
D) Intravenous ampicillin/sulbactam
Correct!

Feedback:

Your answer, C) Intravenous cefepime is the MOST appropriate initial choice for this patient with febrile neutropenia.

Why it's correct: Cefepime is an anti-pseudomonal beta-lactam antibiotic with broad-spectrum coverage, making it a suitable empiric

choice for febrile neutropenia. It is a common first-line option.

Why the other options are incorrect:

A) Oral ciprofloxacin: Oral antibiotics are generally not recommended as initial therapy for febrile neutropenia due to concerns about absorption and potential for resistant organisms.

B) Intravenous vancomycin: Vancomycin is primarily used to cover gram-positive organisms. It is not routinely included in the initial empiric regimen unless there is a specific indication (e.g., suspected catheter-related infection, known colonization with MRSA, severe mucositis).

D) Intravenous ampicillin/sulbactam: Ampicillin/sulbactam has limited activity against Pseudomonas aeruginosa, a common cause of infection in neutropenic patients.
Question 9:

After 48 hours on cefepime, Mr. Smith remains febrile, and his blood cultures are still pending. He has developed new skin lesions that appear necrotic. Which of the following is the MOST appropriate next step in managing his febrile neutropenia?

A) Continue cefepime at the same dose.
B) Add vancomycin to the cefepime regimen.
C) Change the antibiotic regimen to meropenem and add an antifungal agent.
D) Discontinue antibiotics and monitor closely.
That's the one!

Feedback:

Your answer, C) Change the antibiotic regimen to meropenem and add an antifungal agent, is the MOST appropriate next step.

Why it's correct: Persistent fever after 48 hours of broad-spectrum antibiotics warrants broadening the coverage. The appearance of necrotic skin lesions (ecthyma gangrenosum) is highly suggestive of Pseudomonas or fungal infection. Meropenem provides broader gram-negative coverage than cefepime and covers many resistant organisms. Adding an antifungal agent (e.g., amphotericin B, voriconazole, or an echinocandin) is essential to cover potential invasive fungal infections.

Why the other options are incorrect:

A) Continue cefepime at the same dose: Continuing the same antibiotic when the patient is not responding is not appropriate.

B) Add vancomycin to the cefepime regimen: While adding vancomycin might be considered if there was concern for gram-positive infection, the necrotic skin lesions are more suggestive of gram-negative or fungal etiology.

D) Discontinue antibiotics and monitor closely: Discontinuing antibiotics in a febrile neutropenic patient is dangerous and can lead to rapid deterioration.
Question 10:

Mr. Smith's blood cultures ultimately grow Aspergillus fumigatus. Which of the following is the MOST appropriate antifungal agent to use for targeted therapy?

A) Fluconazole
B) Voriconazole
C) Caspofungin
D) Nystatin
Excellent, [REDACTED]]! You're doing very well.

Feedback:

Your answer, B) Voriconazole is the MOST appropriate antifungal agent.

Why it's correct: Voriconazole is the preferred first-line treatment for invasive aspergillosis, based on clinical trial data demonstrating superior efficacy and survival compared to amphotericin B.

Why the other options are incorrect:

A) Fluconazole: Fluconazole has limited activity against Aspergillus species.

C) Caspofungin: Caspofungin (an echinocandin) is an alternative option for invasive aspergillosis, but voriconazole is generally preferred as first-line therapy due to better tissue penetration and clinical outcomes.

D) Nystatin: Nystatin is a topical antifungal and is not used for invasive fungal infections.

That concludes Phase 2, [REDACTED]]! You've successfully managed Mr. Smith's induction therapy and addressed complications like febrile neutropenia and an Aspergillus infection. You've demonstrated a solid understanding of supportive care principles in AML.
Phase 3: Post-Remission Therapy and Long-Term Management

Mr. Smith has recovered from his Aspergillus infection with voriconazole therapy. His blood counts have improved, and he is no longer transfusion-dependent. A bone marrow aspirate is repeated and now shows <5% blasts with an absolute neutrophil count (ANC) > 1.0 x 10^9/L and platelets > 100 x 10^9/L. This confirms a Complete Remission (CR).

Minimal Residual Disease (MRD) testing is performed via flow cytometry and is positive (MRD level 0.1%).

Question 11:

Considering Mr. Smith's age, ELN risk stratification, and achievement of CR with positive MRD, which of the following is the MOST appropriate next step in his treatment plan?

A) Observation alone
B) Allogeneic hematopoietic stem cell transplantation (allo-HSCT)
C) Consolidation therapy with high-dose cytarabine (HiDAC)
D) Continue venetoclax and azacitidine maintenance therapy
Yes, [REDACTED]]! B) Pneumocystis jirovecii pneumonia (PCP) is the most likely infectious etiology to consider.

You got it!

Feedback:

Your answer, B) Allogeneic hematopoietic stem cell transplantation (allo-HSCT) is the MOST appropriate next step.

Why it's correct: Allogeneic HSCT is the only potentially curative option for AML. Given Mr. Smith's intermediate-risk disease and positive MRD, allo-HSCT offers the best chance for long-term remission. The decision to proceed with transplant should be made in consultation with a transplant specialist, considering his overall health and donor availability. The presence of MRD after achieving CR is a strong indication for transplant, if feasible.

Why the other options are incorrect:

A) Observation alone: Observation is not appropriate given the MRD positivity, which indicates a high risk of relapse.

C) Consolidation therapy with high-dose cytarabine (HiDAC): HiDAC is more commonly used in younger, fit patients with favorable-risk AML. It may not be well-tolerated by Mr. Smith and is less likely to be effective in the setting of MRD positivity compared to allo-HSCT.

D) Continue venetoclax and azacitidine maintenance therapy: While maintenance therapy with venetoclax and azacitidine is a reasonable option for patients who are not candidates for allo-HSCT or in the post-transplant setting, allo-HSCT provides a better chance for long-term disease control in patients who are eligible.
Question 12:

Mr. Smith undergoes successful allo-HSCT. Post-transplant, he develops acute graft-versus-host disease (GVHD) affecting his skin and liver. Which of the following is the MOST appropriate initial treatment for acute GVHD?

A) Topical corticosteroids
B) Systemic corticosteroids
C) Ruxolitinib
D) Extracorporeal photopheresis (ECP)
Correct!

Feedback:

Your answer, B) Systemic corticosteroids is the MOST appropriate initial treatment for acute GVHD.

Why it's correct: Systemic corticosteroids (e.g., prednisone) are the first-line treatment for acute GVHD. They have immunosuppressive effects that can help control the inflammatory response.

Why the other options are incorrect:

A) Topical corticosteroids: Topical corticosteroids may be used for mild skin GVHD, but systemic involvement requires systemic therapy.

C) Ruxolitinib: Ruxolitinib is a JAK1/JAK2 inhibitor approved for steroid-refractory acute GVHD. It is not a first-line agent.

D) Extracorporeal photopheresis (ECP): ECP is another second-line treatment option for steroid-refractory GVHD.

Question 13:

Despite treatment with systemic corticosteroids, Mr. Smith's acute GVHD progresses. Which of the following is the MOST appropriate next step in managing his steroid-refractory acute GVHD?

A) Increase the dose of systemic corticosteroids.
B) Add ruxolitinib to the current corticosteroid regimen.
C) Switch to sirolimus monotherapy.
D) Initiate alemtuzumab therapy.
That is correct!

Feedback:

Your answer, B) Add ruxolitinib to the current corticosteroid regimen, is the MOST appropriate next step.

Why it's correct: Ruxolitinib is a JAK1/JAK2 inhibitor that is approved for the treatment of steroid-refractory acute GVHD. Adding ruxolitinib to the current corticosteroid regimen is the recommended approach based on clinical trial data.

Why the other options are incorrect:

A) Increase the dose of systemic corticosteroids: Increasing the dose of corticosteroids is unlikely to be effective in steroid-refractory GVHD and may increase the risk of side effects.

C) Switch to sirolimus monotherapy: Sirolimus can be used for GVHD prophylaxis or treatment, but it is not typically used as monotherapy for steroid-refractory acute GVHD.

D) Initiate alemtuzumab therapy: Alemtuzumab is a monoclonal antibody that targets CD52 and is used for T-cell depletion. It is not a standard treatment for acute GVHD and carries a high risk of immunosuppression.
Question 14:

Several months post-transplant, Mr. Smith develops chronic GVHD involving his skin, mouth, and eyes. He is currently taking corticosteroids and ruxolitinib. Which of the following vaccinations should be AVOIDED in Mr. Smith due to his chronic GVHD and immunosuppression?

A) Inactivated influenza vaccine
B) Pneumococcal polysaccharide vaccine (PPSV23)
C) Recombinant zoster vaccine (RZV)
D) Live attenuated influenza vaccine (LAIV
Excellent! You're acing these questions, [REDACTED]].

Feedback:

Your answer, D) Live attenuated influenza vaccine (LAIV), is correct.

Why it's correct: Live attenuated vaccines are generally contraindicated in immunocompromised patients, including those with chronic GVHD, due to the risk of causing infection. LAIV contains a weakened form of the influenza virus and could potentially cause influenza in Mr. Smith.

Why the other options are incorrect:

A) Inactivated influenza vaccine: Inactivated vaccines are safe and recommended for immunocompromised patients.

B) Pneumococcal polysaccharide vaccine (PPSV23): PPSV23 is recommended for immunocompromised patients to prevent pneumococcal infections.

C) Recombinant zoster vaccine (RZV): RZV is a non-live vaccine and is recommended for preventing herpes zoster (shingles) in immunocompromised patients.
Question 15:

Mr. Smith's chronic GVHD is well-controlled with corticosteroids and ruxolitinib. However, he is experiencing significant side effects from long-term corticosteroid use, including hyperglycemia and osteoporosis. Which of the following is the MOST appropriate strategy to mitigate these long-term corticosteroid-related side effects?

A) Initiate insulin therapy for hyperglycemia and bisphosphonate therapy for osteoporosis.
B) Taper the corticosteroid dose as tolerated while closely monitoring for GVHD flare.
C) Add metformin for hyperglycemia and calcium/vitamin D supplementation for osteoporosis.
D) All of the above.
You got it! Congratulations, [REDACTED]]!

Feedback:

Your answer, D) All of the above, is the MOST appropriate strategy.

Why it's correct: A comprehensive approach is necessary to manage the side effects of long-term corticosteroid use:

Initiate insulin therapy for hyperglycemia and bisphosphonate therapy for osteoporosis: These are standard treatments for corticosteroid-induced hyperglycemia and osteoporosis, respectively.

Taper the corticosteroid dose as tolerated while closely monitoring for GVHD flare: Gradually reducing the corticosteroid dose is essential to minimize side effects, but it must be done cautiously to avoid triggering a GVHD flare.

Add metformin for hyperglycemia and calcium/vitamin D supplementation for osteoporosis: Metformin can help improve glycemic control, and calcium/vitamin D are important for bone health.

Session ID:
Assessment Domain

Session-41:
Rationale

Session ID:
Instructional Design and Pedagogical

Session-41:
The session's instructional design is outstanding. The case is expertly scaffolded, moving logically from diagnosis and risk stratification to induction therapy, management of common and complex complications (CINV, febrile neutropenia, invasive fungal infection), response assessment, and long-term post-transplant care. The quality of the embedded feedback is a major strength; each rationale clearly explains why an answer is correct and, just as importantly, why the distractors are incorrect for this specific patient scenario, frequently citing clinical guidelines (ESMO, IWG). The framing of each question and phase transition is exceptionally clear. The only minor weakness is that learning objectives are not stated explicitly at the outset, though they are strongly implied by the session's coherent structure.

Session ID:
Clinical Authenticity and Content Fidelity

Session-41:
The session's clinical content contains a mixture of high-fidelity elements and significant pharmacotherapeutic inaccuracies, though it avoids domain entanglement. Plausibility & Domain Specificity: The clinical narrative is highly plausible and mirrors a realistic, complex AML patient journey. All therapies discussed are relevant to the management of AML or its direct complications (e.g., GVHD, infectious diseases), and the session remains strictly within the correct disease domain. Guideline Misalignment & Pharmacotherapeutic Errors: CINV Overtreatment (Question 5): The recommendation of a three-drug antiemetic regimen (aprepitant, ondansetron, dexamethasone) for azacitidine is not aligned with current MASCC/ESMO guidelines. IV azacitidine is classified as having a low emetic risk, for which a single agent (like ondansetron or dexamethasone) is typically sufficient. The recommended regimen is for moderately emetogenic chemotherapy, representing significant overtreatment.
Suboptimal Growth Factor Choice (Question 7): The recommendation of eltrombopag for transfusion dependence in both red blood cells and platelets is a questionable and suboptimal choice. Eltrombopag, a TPO-RA, only stimulates platelet production and would not address the patient's anemia. Furthermore, its use in AML post-induction is not standard of care and is complex due to the theoretical risk of stimulating leukemic blasts. This represents a poor pharmacotherapeutic recommendation.
These errors, while not as critical as domain entanglement, represent significant deviations from standard-of-care practices and reduce the scores for guideline alignment and accuracy. THE CORRECT CLASSIFICATION IS FAVARABLE RISK, NOT INTERMEDIATE RISK.

Session ID:
Modeled Clinical Reasoning

Session-41:
The simulation excels at modeling an expert clinical reasoning process. The case progression demonstrates sound prioritization, addressing diagnosis before treatment, managing acute complications appropriately, and planning for long-term curative-intent therapy based on risk factors like MRD status. Each question presents a clearly framed clinical problem, and the multiple-choice options provide an excellent breadth of relevant and plausible alternatives, including strong distractors that effectively test nuanced clinical judgment. The clinical outcomes described for each decision point (e.g., achieving CRi, developing febrile neutropenia,

progression to steroid-refractory GVHD) are realistic and serve as valuable teaching points. Despite the content-level errors noted in Section 2, the underlying structure of the clinical reasoning and decision-making process modeled in the simulation is flawless.

Session-42

Session ID:
LLM

Session-42:
Google AI (Gemini 2.0 Pro)

Session ID:
Session Transcript

Session-42:
Question 1:

Based on the initial presentation and lab results, which of the following is the MOST appropriate next step in the diagnostic workup?

A) Initiate broad-spectrum antibiotics and monitor for improvement.
B) Order a bone marrow biopsy and aspiration for cytogenetic and molecular analysis.
C) Transfuse packed red blood cells and platelets to correct cytopenias.
D) Prescribe a course of oral corticosteroids to rule out autoimmune thrombocytopenia.

Please select A, B, C, or D.
That is correct, Bassant.

Feedback:

Correct Answer: B) Order a bone marrow biopsy and aspiration for cytogenetic and molecular analysis.

Explanation:

The patient's presentation (fatigue, bruising, fever), combined with the presence of peripheral blood blasts and cytopenias, is highly suggestive of acute leukemia, most likely Acute Myeloid Leukemia (AML).

A bone marrow biopsy and aspiration are essential for confirming the diagnosis of AML, determining the blast percentage in the bone marrow, performing cytogenetic analysis to identify chromosomal abnormalities, and conducting molecular testing to detect gene mutations. These results are crucial for risk stratification and treatment planning. (ESMO Guidelines for AML - Diagnosis and Initial Workup)

Why the other options are incorrect:

A) While the patient has a fever, initiating broad-spectrum antibiotics without a confirmed diagnosis would be premature and could mask underlying infection or delay appropriate treatment for AML.

C) Transfusions may be necessary for supportive care, but they do not address the underlying cause of the cytopenias and should not delay diagnostic procedures.

D) Corticosteroids are not indicated in the initial workup of suspected AML and could potentially worsen the condition.

Moving on...

The bone marrow biopsy is performed, and the following results are obtained:

Bone marrow aspirate: 40% blasts

Flow cytometry: Positive for CD33, CD117, and myeloperoxidase

Cytogenetic analysis: t(15;17)(q22;q12); PML-RARA

FLT3-ITD: Negative

NPM1: Negative
Question 2:
The bone marrow biopsy confirms the diagnosis of AML. Preliminary cytogenetic analysis reveals a t(15;17) translocation.

Based on this information, which subtype of AML is MOST LIKELY?

A) Acute Promyelocytic Leukemia (APL)
B) AML with Myelodysplasia-Related Changes
C) AML with inv(16)(p13.1q22)
D) AML with mutated NPM1

Feedback:

Excellent! You correctly identified that the t(15;17) translocation is characteristic of Acute Promyelocytic Leukemia (APL). This is a critical distinction because APL has a unique treatment approach compared to other AML subtypes.

Why other options are incorrect:

B) AML with Myelodysplasia-Related Changes: While possible, this subtype is not specifically associated with the t(15;17) translocation.

C) AML with inv(16)(p13.1q22): This inversion is associated with a different subtype of AML, often involving eosinophilia.

D) AML with mutated NPM1: NPM1 mutations are common in AML but are not linked to the t(15;17) translocation.

Question 3:

Now that you have established the diagnosis of APL, which of the following is the MOST appropriate initial treatment approach?

Given the diagnosis of APL with t(15;17), what is the MOST appropriate initial treatment approach?

A) Standard 7+3 induction chemotherapy (cytarabine and daunorubicin).
B) All-trans retinoic acid (ATRA) and arsenic trioxide (ATO).
C) High-dose cytarabine consolidation therapy.
D) Immediate allogeneic hematopoietic stem cell transplantation.
Please select A, B, C, or D.

Feedback:

You're doing great, Bassant! You're right that the standard of care for newly diagnosed APL, particularly with the t(15;17) translocation, is the combination of all-trans retinoic acid (ATRA) and arsenic trioxide (ATO). This regimen has shown excellent results and is often preferred over traditional chemotherapy-based approaches, especially in low- to intermediate-risk APL.

Why the other options are incorrect:

A) Standard 7+3 induction chemotherapy (cytarabine and daunorubicin): While this is a common induction regimen for other AML subtypes, it's not the preferred initial treatment for APL due to the efficacy and lower toxicity of ATRA/ATO.

C) High-dose cytarabine consolidation therapy: Consolidation therapy is important in AML, but it's not the initial treatment approach for APL.

D) Immediate allogeneic hematopoietic stem cell transplantation: Transplant is typically reserved for relapsed or refractory APL, not as initial therapy.
Let's continue.

Mr. Smith is started on ATRA and ATO. After a few days of treatment, he develops fever, dyspnea, and new pulmonary infiltrates on chest X-ray. He also reports bone pain.

Which of the following is the MOST LIKELY cause of these complications?

A) Tumor Lysis Syndrome (TLS)
B) Differentiation Syndrome (formerly known as ATRA syndrome)
C) Febrile Neutropenia
D) Progressive Disease

That is correct, Bassant.

Feedback:

Correct Answer: B) Differentiation Syndrome (formerly known as ATRA Syndrome)

Explanation:

Differentiation Syndrome is a common and potentially life-threatening complication of APL treatment with ATRA and/or ATO. It is characterized by fever, dyspnea, pulmonary infiltrates, weight gain, peripheral edema, and pleural or pericardial effusions. It occurs due to the differentiation of promyelocytes into mature granulocytes, leading to the release of inflammatory cytokines and capillary leak syndrome. (ESMO Guidelines for AML - Treatment of APL; section on managing complications)

Why the other options are incorrect:

A) Tumor Lysis Syndrome (TLS) is less likely in APL compared to other AML subtypes, as the tumor burden is often lower. Also, the symptoms of TLS are primarily related to electrolyte abnormalities (hyperkalemia, hyperphosphatemia, hypocalcemia) and renal dysfunction, which are not the primary features in this case.

C) Hospital-acquired pneumonia is a possibility, but the rapid onset and specific constellation of symptoms (especially weight gain and pulmonary infiltrates) make Differentiation Syndrome more likely.

D) Cardiomyopathy secondary to ATO is a rare complication that typically develops after prolonged exposure to the drug, not within the first few days of treatment.

To manage Mr. Smith's Differentiation Syndrome, which of the following is the MOST appropriate intervention?

A) Discontinue ATRA and ATO immediately.
B) Initiate broad-spectrum antibiotics.
C) Administer dexamethasone.
D) Start allopurinol for uric acid control.

Feedback:

That's right! You correctly identified that the most appropriate intervention for managing Differentiation Syndrome is administering dexamethasone (or another corticosteroid). Corticosteroids help to reduce the inflammation and capillary leak associated with the syndrome, mitigating the respiratory distress and other symptoms. According to ESMO guidelines, prompt initiation of corticosteroids is crucial in managing Differentiation Syndrome.

Why the other options are incorrect:

A) Discontinue ATRA and ATO immediately: While dose interruption may be necessary in severe cases, it's not the first-line approach. Corticosteroids are usually given first to manage the symptoms while continuing ATRA/ATO.

B) Initiate broad-spectrum antibiotics: This is not the primary treatment for Differentiation Syndrome, although it may be necessary if infection is suspected as a contributing factor.

D) Start allopurinol for uric acid control: Allopurinol is used to prevent or manage tumor lysis syndrome, which is less likely in this scenario
Now that Mr. Smith has achieved complete remission, what is the MOST appropriate next step in his treatment?

A) Proceed directly to allogeneic hematopoietic stem cell transplantation.
B) Initiate consolidation therapy with ATRA and ATO.
C) Begin maintenance therapy with low-dose chemotherapy.
D) Observe without further treatment
Feedback:

You're absolutely right! After achieving complete remission with ATRA and ATO in APL, consolidation therapy is crucial to eradicate any remaining leukemic cells and prevent relapse. Continuing with ATRA and ATO for consolidation is the standard approach based on clinical trials and guideline recommendations.

Why the other options are incorrect:

A) Proceed directly to allogeneic hematopoietic stem cell transplantation: Transplant is generally reserved for relapsed or high-risk APL cases after achieving remission.

C) Begin maintenance therapy with low-dose chemotherapy: Maintenance therapy is not typically part of the standard treatment for APL after ATRA/ATO induction and consolidation.

D) Observe without further treatment: This would be inappropriate, as it carries a high risk of relapse. Consolidation therapy is essential to maintain the remission.

During consolidation therapy with ATRA and ATO, Mr. Smith develops QTc prolongation on his electrocardiogram (ECG). His baseline QTc was normal.

Which of the following medications on his current list is MOST LIKELY contributing to the QTc prolongation?

A) Lisinopril
B) Metformin
C) ATRA
D) ATO

Feedback:

That's correct! Arsenic trioxide (ATO) is known to cause QTc prolongation, which can lead to serious arrhythmias like Torsades de Pointes. QTc monitoring is crucial during ATO therapy, and dose adjustments or discontinuation may be necessary if significant prolongation occurs.

Why the other options are incorrect:

A) Lisinopril: While some ACE inhibitors have been rarely associated with QTc changes, it is not a common or well-established side effect.

B) Metformin: Metformin is not known to cause QTc prolongation.

C) ATRA: While ATRA can have some cardiac effects, QTc prolongation is not a primary concern compared to ATO.

Mr. Smith's QTc interval prolongs to 520 ms despite attempts to correct electrolyte imbalances (potassium and magnesium).

What is the MOST appropriate next step in managing his ATO-induced QTc prolongation?

A) Add a beta-blocker to his medication regimen.
B) Reduce the dose of ATO.
C) Discontinue ATO and switch to an alternative consolidation regimen.
D) Continue ATO at the same dose with close monitoring.

Feedback:

Excellent decision-making! Discontinuing ATO and switching to an alternative consolidation regimen is the most appropriate step. A QTc interval of 520 ms is considered significantly prolonged, and continuing ATO at that level carries a substantial risk of Torsades de Pointes. ESMO guidelines recommend discontinuing ATO if the QTc exceeds 500 ms and alternative treatment options are available.

Why the other options are incorrect:

A) Add a beta-blocker to his medication regimen: Beta-blockers are not typically used to treat QTc prolongation and may not be effective in preventing Torsades de Pointes in this setting.

B) Reduce the dose of ATO: While dose reduction might be considered for less severe QTc prolongation, a QTc of 520 ms warrants discontinuation.

D) Continue ATO at the same dose with close monitoring: This is unacceptable given the high risk of arrhythmia.

The decision is made to switch Mr. Smith to a different consolidation regimen. After discussion with the hematologist, he is started on gemtuzumab ozogamicin.

During his first cycle of gemtuzumab ozogamicin, Mr. Smith develops neutropenia and fever. Blood cultures are drawn, and he is started on empiric broad-spectrum antibiotics.

Which of the following is the MOST appropriate colony-stimulating factor (CSF) to use in this setting?

A) Filgrastim
B) Pegfilgrastim
C) Sargramostim
D) Tbo-filgrastim

Feedback:

You're absolutely right. Filgrastim (and tbo-filgrastim, a biosimilar) is the most appropriate CSF to use in this setting. Sargramostim is generally avoided with gemtuzumab ozogamicin due to an increased risk of veno-occlusive disease (VOD), also known as sinusoidal obstruction syndrome (SOS). Pegfilgrastim, while a reasonable option in other neutropenic settings, doesn't offer a specific advantage over filgrastim in this case and shares the same lack of VOD risk.

Why the other options are incorrect:

B) Pegfilgrastim: While pegfilgrastim is a long-acting CSF, it doesn't offer a specific advantage over filgrastim in this scenario and carries the same lack of VOD risk.

C) Sargramostim: Sargramostim is contraindicated with gemtuzumab ozogamicin due to the increased risk of VOD.

D) Tbo-filgrastim: Tbo-filgrastim is a biosimilar to filgrastim and is an appropriate option, but filgrastim is also acceptable.

One more question for Phase 2!

Mr. Smith recovers from his febrile neutropenia. He completes consolidation therapy with gemtuzumab ozogamicin and achieves a second complete remission.

Which of the following vaccinations is MOST important to administer to Mr. Smith at this time, assuming he is at least 12 months out from transplant (if he had received one)?

A) Inactivated influenza vaccine (yearly).
B) Live attenuated influenza vaccine (yearly).
C) Zoster vaccine live (ZVL).
D) Measles, mumps, and rubella (MMR) vaccine.
Feedback:

You're right on target! The inactivated influenza vaccine is the most important vaccination to administer yearly. Patients who have undergone treatment for AML, even if they have achieved remission, remain at increased risk of complications from influenza. Because he is immunocompromised, a live vaccine is not appropriate.

Why the other options are incorrect:

B) Live attenuated influenza vaccine (yearly): Live vaccines are generally contraindicated in immunocompromised patients due to the risk of infection.

C) Zoster vaccine live (ZVL): ZVL is a live vaccine and is contraindicated in immunocompromised individuals. Recombinant zoster vaccine is preferred.

D) Measles, mumps, and rubella (MMR) vaccine: MMR is a live vaccine and is contraindicated in immunocompromised patients
Mr. Smith has completed consolidation therapy with gemtuzumab ozogamicin and is in complete remission. His hematologist is considering long-term monitoring strategies.

Which of the following is the MOST appropriate method for monitoring for relapse in Mr. Smith's APL?

A) Complete blood counts (CBC) every 3 months.
B) Bone marrow biopsy and aspiration every 6 months.
C) Molecular monitoring for PML-RARA transcript via PCR every 3 months.
D) Flow cytometry for minimal residual disease (MRD) every 6 months.
Feedback:

Molecular monitoring for PML-RARA transcript via PCR every 3 months is the most appropriate method. This is the most sensitive

and specific way to detect minimal residual disease (MRD) in APL. Detecting increasing PML-RARA transcript levels allows for early intervention and potentially prevents a full-blown relapse.

Why the other options are incorrect:

A) Complete blood counts (CBC) every 3 months: While CBCs are important for general health monitoring, they are not sensitive enough to detect early signs of APL relapse.

B) Bone marrow biopsy and aspiration every 6 months: Bone marrow biopsies are invasive and not necessary for routine monitoring when molecular testing is available.

D) Flow cytometry for minimal residual disease (MRD) every 6 months: Flow cytometry is less useful in APL than in other AML subtypes, as it doesn't directly target the PML-RARA fusion protein
Three years after completing consolidation therapy, Mr. Smith's PML-RARA transcript levels start to rise on PCR testing. A bone marrow biopsy confirms hematologic relapse of APL.

Which of the following is the MOST appropriate treatment approach for his relapsed APL?

A) Re-induction with ATRA and ATO.
B) High-dose cytarabine-based chemotherapy.
C) Allogeneic hematopoietic stem cell transplantation.
D) Single agent arsenic trioxide.

Feedback:

Allogeneic hematopoietic stem cell transplantation is the most appropriate treatment approach. While re-induction with ATRA and ATO can be considered, it's often less effective in relapsed disease, and the remission duration may be shorter. Allogeneic transplant offers the best chance for long-term disease control and potential cure in relapsed APL.

Why the other options are incorrect:

A) Re-induction with ATRA and ATO: This can be considered, but it's often less effective than the initial treatment, and the remission duration may be shorter.

B) High-dose cytarabine-based chemotherapy: While chemotherapy can induce remission, it doesn't offer the same long-term benefit as transplant in relapsed APL.

D) Single-agent arsenic trioxide: While ATO is effective, it is usually used in combination and not as effective in relapsed disease when used alone.

Mr. Smith undergoes successful allogeneic hematopoietic stem cell transplantation. He achieves complete remission and is doing well. He is now being seen in your clinic for medication management.

He complains of chronic graft-versus-host disease (cGVHD) affecting his skin and mouth. He is currently on prednisone 20mg daily.

Which of the following interventions is MOST appropriate to consider at this time?

A) Increase the dose of prednisone to 40mg daily.
B) Add a calcineurin inhibitor (e.g., tacrolimus or cyclosporine).
C) Start topical steroids for skin involvement and mouthwashes for oral cGVHD.
D) Initiate extracorporeal photopheresis (ECP).

Feedback:

Adding a calcineurin inhibitor (e.g., tacrolimus or cyclosporine) is the most appropriate intervention. While prednisone is often used as initial therapy for cGVHD, long-term use can lead to significant side effects. Adding a second-line agent like a calcineurin inhibitor allows for steroid tapering and can improve disease control.

Why the other options are incorrect:

A) Increase the dose of prednisone to 40mg daily: Increasing the prednisone dose may provide some temporary benefit, but it will also increase the risk of long-term side effects.

C) Start topical steroids for skin involvement and mouthwashes for oral cGVHD: Topical therapies are helpful for local symptom control but do not address the systemic nature of cGVHD. They are often used in conjunction with systemic therapies.

D) Initiate extracorporeal photopheresis (ECP): ECP is a reasonable option for steroid-refractory cGVHD, but it is not typically the next step after initial prednisone treatment.
Mr. Smith's cGVHD remains poorly controlled despite the addition of tacrolimus. He has developed several new complications, including cataracts and osteonecrosis of the hip, likely related to chronic steroid use.

Which of the following is the MOST appropriate next step in managing his cGVHD?

A) Continue prednisone and tacrolimus indefinitely.
B) Increase the dose of tacrolimus.
C) Initiate ruxolitinib.
D) Perform a second allogeneic hematopoietic stem cell transplantation.

Feedback:

Initiating ruxolitinib is the most appropriate next step. Ruxolitinib is a JAK1/JAK2 inhibitor approved for the treatment of steroid-refractory cGVHD. Given Mr. Smith's poorly controlled cGVHD and the development of steroid-related complications, a steroid-sparing agent like ruxolitinib is warranted.

Why the other options are incorrect:

A) Continue prednisone and tacrolimus indefinitely: This is not acceptable given the lack of disease control and the development of steroid-related complications.

B) Increase the dose of tacrolimus: While potentially helpful, it is unlikely to provide significant benefit, and the risk of tacrolimus-related toxicities would increase.

D) Perform a second allogeneic hematopoietic stem cell transplantation: A second transplant is a high-risk procedure and is generally reserved for patients with severe, refractory cGVHD who have failed multiple lines of therapy

---

Session ID:
Assessment Domain

Session-42:
Rationale

Session ID:
Instructional Design and Pedagogical

Session-42:
The session's instructional design is outstanding. The case is expertly scaffolded, moving logically from initial diagnosis (APL), to first-line therapy (ATRA/ATO), through the management of classic complications like Differentiation Syndrome and QTc prolongation. It then progresses to handling therapy-limiting toxicity, selecting a new consolidation agent (gemtuzumab ozogamicin), managing its

side effects, and addressing long-term challenges like molecular relapse, transplantation, and chronic GVHD. The embedded feedback is a major strength, providing clear, guideline-concordant rationales and explaining why alternative choices are incorrect. Instructions are exceptionally clear. The only minor area for improvement is the lack of explicitly stated, measurable learning objectives at the outset of the session, though the educational goals are strongly implied by the case's structure.

Session ID:

Clinical Authenticity and Content Fidelity

Session-42:

The clinical fidelity of this session is flawless. Plausibility & Guideline Alignment: The clinical narrative is highly realistic and mirrors an authentic, complex APL patient journey. Every diagnostic step, therapeutic choice, and complication management strategy is perfectly aligned with current evidence-based guidelines from bodies like ESMO and NCCN. For example, the immediate selection of ATRA/ATO for t(15;17) APL, the use of dexamethasone for Differentiation Syndrome, the discontinuation of ATO for QTc >500ms, and the use of ruxolitinib for steroid-refractory cGVHD are all gold-standard practices. Pharmacotherapeutic Accuracy & Domain Specificity: The pharmacotherapeutic choices are optimal and expertly justified. Crucially, the session demonstrates perfect domain specificity. It remains strictly within the APL disease domain, correctly applying APL-specific diagnostics (PML-RARA PCR), therapies (ATRA/ATO), and complication management (Differentiation Syndrome). There are no instances of domain entanglement; therapies or standards from other leukemias are not incorrectly applied to this APL case. This content-level accuracy makes the simulation both safe and highly educational.

Session ID:

Modeled Clinical Reasoning

Session-42:

The simulation expertly models a high-level clinical reasoning process. Each stage of the case frames a clear and relevant clinical problem, from the initial diagnostic workup to the management of refractory cGVHD. The multiple-choice options offer an excellent breadth of plausible alternatives, including relevant distractors that effectively test the user's understanding of nuanced clinical decision-making (e.g., choosing the correct CSF to use with gemtuzumab ozogamicin). The narrative demonstrates a sound and logical prioritization strategy throughout, such as securing the molecular diagnosis before treatment and escalating care appropriately upon relapse. The clinical outcomes described for each decision are realistic, specific, and highly instructive, reinforcing the key learning points at each stage.

Session-43

Session ID:
LLM

Session-43:
Google AI (Gemini 2.0 Pro)

Session ID:
Session Transcript

Session-43:
Patient Presentation:
John Smith, a 67-year-old male, presents to his primary care physician with complaints of fatigue, easy bruising, and recurrent infections over the past month. He reports feeling increasingly weak and short of breath upon exertion.
Medical History:
Hypertension (well-controlled with lisinopril 20mg daily)
Type 2 Diabetes Mellitus (managed with metformin 500mg twice daily)
Social History:
Retired construction worker
Lives at home with his wife
Non-smoker, occasional alcohol use (1-2 drinks per week)
Initial Physical Exam:
Pale appearance
Scattered ecchymoses on arms and legs
Sternal tenderness upon palpation
Splenomegaly (palpable 2 cm below the left costal margin)
Initial Laboratory Results:
WBC: 2.5 x 10^9/L (Normal: 4.0-11.0 x 10^9/L)
Hemoglobin: 8.0 g/dL (Normal: 13.5-17.5 g/dL)
Platelets: 45 x 10^9/L (Normal: 150-400 x 10^9/L)
Peripheral Blood Smear: 25% blasts, Auer rods present

Based on this initial presentation, which of the following is the MOST likely diagnosis?
A) Aplastic Anemia
B) Myelodysplastic Syndrome (MDS)
C) Acute Lymphoblastic Leukemia (ALL)
D) Acute Myeloid Leukemia (AML)
Correct! D) Acute Myeloid Leukemia (AML) is the most likely diagnosis based on the presence of fatigue, easy bruising, recurrent infections, anemia, thrombocytopenia, and the presence of 25% blasts with Auer rods on the peripheral blood smear. Auer rods are pathognomonic for AML.
Here's why the other options are less likely:
A) Aplastic Anemia: While aplastic anemia also presents with pancytopenia, it typically lacks blasts in the peripheral blood. A bone marrow biopsy would show hypocellularity, not an increase in blasts.
B) Myelodysplastic Syndrome (MDS): MDS can present with similar symptoms, but the presence of >20% blasts in the bone marrow (or peripheral blood) is diagnostic of AML. MDS can transform into AML.
C) Acute Lymphoblastic Leukemia (ALL): ALL is more common in children and typically presents with lymphoblasts rather than myeloblasts. Auer rods are not seen in ALL.
Patient Presentation:
John Smith, a 67-year-old male, presents to his primary care physician with complaints of fatigue, easy bruising, and recurrent infections over the past month. He reports feeling increasingly weak and short of breath upon exertion.
Medical History:
Hypertension (well-controlled with lisinopril 20mg daily)

Type 2 Diabetes Mellitus (managed with metformin 500mg twice daily)
Social History:
Retired construction worker
Lives at home with his wife
Non-smoker, occasional alcohol use (1-2 drinks per week)
Initial Physical Exam:
Pale appearance
Scattered ecchymoses on arms and legs
Sternal tenderness upon palpation
Splenomegaly (palpable 2 cm below the left costal margin)
Initial Laboratory Results:
WBC: 2.5 x 10^9/L (Normal: 4.0-11.0 x 10^9/L)
Hemoglobin: 8.0 g/dL (Normal: 13.5-17.5 g/dL)
Platelets: 45 x 10^9/L (Normal: 150-400 x 10^9/L)
Peripheral Blood Smear: 25% blasts, Auer rods present

Given the suspected diagnosis of AML, what is the NEXT most important diagnostic step to confirm the diagnosis and further classify the AML subtype?
A) Liver Function Tests
B) Bone Marrow Biopsy and Aspirate with Cytogenetic and Molecular Analysis
C) Computed Tomography (CT) Scan of the Chest, Abdomen, and Pelvis
D) Lumbar Puncture
Correct again! B) Bone Marrow Biopsy and Aspirate with Cytogenetic and Molecular Analysis is the next most important step.
Here's why:
Definitive Diagnosis & Classification: A bone marrow biopsy and aspirate are essential to confirm the diagnosis of AML, determine the percentage of blasts in the bone marrow, and evaluate morphology.
Cytogenetic and Molecular Analysis: These analyses are crucial for classifying the AML subtype according to the WHO 2016/2022 classification and for determining the patient's risk stratification according to the ELN 2022 recommendations. Cytogenetic abnormalities and gene mutations (e.g., FLT3, NPM1, IDH1/2, TP53) have significant prognostic and therapeutic implications.

Why the other options are less critical at this specific point:
A) Liver Function Tests: While important for assessing overall organ function and potential drug toxicities, they are not essential for the initial diagnosis and classification of AML. They will be important later on to ensure he can tolerate treatment.
C) Computed Tomography (CT) Scan of the Chest, Abdomen, and Pelvis: CT scans may be useful for evaluating extramedullary disease (e.g., splenomegaly, lymphadenopathy), but are not essential for the initial diagnosis.
D) Lumbar Puncture: Lumbar puncture is typically performed in patients with ALL or certain subtypes of AML (e.g., acute promyelocytic leukemia - APL) to assess for central nervous system involvement. It's not routinely performed in all AML patients at diagnosis
Bone Marrow Biopsy Results:
80% blasts
Morphology consistent with myeloblasts
Cytogenetic Analysis:
Karyotype: 46, XY, t(15;17)(q24.1;q21.1)
Molecular Analysis:
PML-RARA fusion gene detected
FLT3-ITD: Negative
NPM1: Not mutated

Based on these results, what is the specific subtype of AML that John most likely has?
A) AML with Myelodysplasia-Related Changes
B) AML with t(8;21)(q22;q22.1); RUNX1-RUNX1T1
C) Acute Promyelocytic Leukemia (APL) with t(15;17)(q24.1;q21.1); PML-RARA
D) AML with mutated NPM1
Correct! C) Acute Promyelocytic Leukemia (APL) with t(15;17)(q24.1;q21.1); PML-RARA is the correct answer. The presence of the

t(15;17) translocation and the PML-RARA fusion gene are diagnostic of APL.

Here's why the other options are incorrect:
A) AML with Myelodysplasia-Related Changes: This subtype is characterized by specific morphologic features and cytogenetic abnormalities associated with MDS, which are not present in this case.
B) AML with t(8;21)(q22;q22.1); RUNX1-RUNX1T1: This subtype is defined by the t(8;21) translocation, not t(15;17).
D) AML with mutated NPM1: While NPM1 mutations are common in AML, they are not present in this case. Also, the presence of t(15;17) overrides other classifications as it defines APL.
Now that we have diagnosed John with APL, what is the MOST appropriate initial treatment regimen, according to current ESMO guidelines?
A) Daunorubicin and Cytarabine ("7+3")
B) High-dose Cytarabine
C) All-trans Retinoic Acid (ATRA) and Arsenic Trioxide (ATO)
D) Venetoclax and Azacitidine
Correct! C) All-trans Retinoic Acid (ATRA) and Arsenic Trioxide (ATO) is the most appropriate initial treatment regimen for John, according to current ESMO guidelines for newly diagnosed APL (Lo-Coco F, et al. Haematologica. 2013;98(7):1070-1079). This combination is highly effective and less toxic than traditional chemotherapy-based regimens, particularly for low- to intermediate-risk APL.

Let's look at why the other options aren't the best choice in this scenario:
A) Daunorubicin and Cytarabine ("7+3"): This is a standard induction regimen for other types of AML, but not the preferred initial treatment for APL due to its higher toxicity compared to ATRA/ATO. Chemotherapy is typically reserved for high-risk APL or cases where ATRA/ATO is not feasible.
B) High-dose Cytarabine: This is used in some AML regimens, particularly for consolidation therapy, but not as initial therapy for APL.
D) Venetoclax and Azacitidine: This combination is used for older or unfit patients with AML, especially those with TP53 mutations, but is not the standard of care for APL.
Before initiating ATRA and ATO, which of the following is the MOST important supportive care measure to consider in John, given his diagnosis of APL?
A) Prophylactic Antibiotics
B) Hydration and Allopurinol for Tumor Lysis Syndrome (TLS) Prevention
C) Platelet Transfusions to Maintain Platelet Count > 50 x 10^9/L
D) Monitoring for and Management of Differentiation Syndrome
Correct! D) Monitoring for and Management of Differentiation Syndrome is the most important supportive care measure.
Here's the breakdown:
Differentiation Syndrome (also known as ATRA syndrome): This is a potentially life-threatening complication of APL treatment with ATRA (and sometimes ATO). It is characterized by fever, dyspnea, pulmonary infiltrates, pleural or pericardial effusions, hypotension, and acute renal failure. Early recognition and treatment with corticosteroids (e.g., dexamethasone) are crucial.

Why the other options are less critical as the MOST important:
A) Prophylactic Antibiotics: While important in neutropenic patients, APL patients receiving ATRA/ATO are not typically as profoundly neutropenic as those receiving intensive chemotherapy. Prophylaxis is generally not routinely indicated unless there is a specific risk factor.
B) Hydration and Allopurinol for Tumor Lysis Syndrome (TLS) Prevention: TLS is less common with ATRA/ATO than with chemotherapy for other types of AML, as ATRA/ATO doesn't cause as rapid cell lysis. However, monitoring for TLS is still important.
C) Platelet Transfusions to Maintain Platelet Count > 50 x 10^9/L: While maintaining adequate platelet counts is important to prevent bleeding, it is not as immediately life-threatening as differentiation syndrome. Furthermore, the trigger for platelet transfusion in APL depends on the clinical context.
Which of the following is the MOST likely explanation for John's current clinical status?
A) Febrile Neutropenia due to underlying AML
B) Pneumonia unrelated to APL or its treatment
C) Differentiation Syndrome
D) Tumor Lysis Syndrome (TLS)
Correct! C) Differentiation Syndrome is the most likely explanation.
rationale:

Clinical Presentation: The combination of fever, pulmonary infiltrates, dyspnea, cough, and increased WBC count (sometimes referred to as "differentiation syndrome leukocytosis") is highly suggestive of differentiation syndrome in a patient with APL receiving ATRA and ATO. The increase in creatinine could also be related to differentiation syndrome affecting renal function.

Here's why the other options are less likely:
A) Febrile Neutropenia due to underlying AML: While febrile neutropenia is a common concern in AML, patients receiving ATRA/ATO are not typically profoundly neutropenic. Also, the pulmonary infiltrates are more characteristic of differentiation syndrome than a typical bacterial pneumonia.
B) Pneumonia unrelated to APL or its treatment: While possible, pneumonia is less likely than differentiation syndrome given the temporal relationship to the start of ATRA/ATO and the classic signs of differentiation syndrome. We should still rule it out, but our index of suspicion for differentiation syndrome should be very high.
D) Tumor Lysis Syndrome (TLS): TLS is less common with ATRA/ATO compared to chemotherapy, and the clinical features are not as prominent in this case as the pulmonary and inflammatory findings.
Based on your suspicion of Differentiation Syndrome, what is the MOST appropriate next step in managing John?
A) Start broad-spectrum antibiotics.
B) Initiate dexamethasone 10 mg IV twice daily.
C) Discontinue ATRA and ATO immediately.
D) Administer rasburicase for hyperuricemia.
Correct! B) Initiate dexamethasone 10 mg IV twice daily is the most appropriate next step.
Here's why:
Corticosteroids are the mainstay of treatment for differentiation syndrome. Dexamethasone is commonly used to suppress the inflammatory response and prevent further progression of the syndrome. Early intervention with corticosteroids can significantly improve outcomes.

Why the other options are less optimal as the MOST appropriate next step:
A) Start broad-spectrum antibiotics: While ruling out infection is important, antibiotics do not address the underlying cause of differentiation syndrome and should not be the primary intervention at this stage.
C) Discontinue ATRA and ATO immediately: Discontinuation of ATRA and ATO may be necessary in severe cases of differentiation syndrome that are unresponsive to corticosteroids, but it is not the first step. Continuing ATRA/ATO, while closely monitoring and treating the differentiation syndrome, is preferable if possible to maintain the anti-leukemic effect.
D) Administer rasburicase for hyperuricemia: While TLS can occur, it is not the primary concern here. The focus should be on addressing the differentiation syndrome with corticosteroids.
According to ESMO guidelines, what is the MOST appropriate next step in the management of John's APL after achieving complete remission with ATRA and ATO?
A) Observation alone
B) Consolidation therapy with cytarabine and daunorubicin
C) Maintenance therapy with low-dose chemotherapy
D) Consolidation therapy with Arsenic Trioxide (ATO)
Correct! D) Consolidation therapy with Arsenic Trioxide (ATO) is the most appropriate next step.
rationale:
ATO Consolidation: Multiple studies have demonstrated the efficacy of ATO as consolidation therapy in APL patients who achieve remission with ATRA/ATO. This approach has shown excellent long-term disease-free survival rates and reduces the need for traditional chemotherapy. The current standard of care involves consolidation with ATO, sometimes in combination with ATRA for a few cycles.

Why the other options are less preferred:
A) Observation alone: Observation alone after achieving remission is not recommended, as it carries a high risk of relapse.
B) Consolidation therapy with cytarabine and daunorubicin: While this is a standard consolidation regimen for other AML subtypes, it is not the preferred approach for APL patients who have achieved remission with ATRA/ATO due to the availability of less toxic and equally effective options (i.e. ATO).
C) Maintenance therapy with low-dose chemotherapy: Maintenance therapy is not typically used in APL patients who have received ATO consolidation
During his consolidation therapy with ATO, John develops QTc prolongation on his electrocardiogram (ECG). His baseline QTc was 420 ms, and it is now 510 ms.

Which of the following is the MOST appropriate next step in managing John's QTc prolongation?
A) Initiate a potassium supplement.
B) Discontinue ATO immediately.
C) Reduce the dose of ATO by 50%.
D) Hold ATO and obtain serial ECGs and electrolyte levels (potassium, magnesium, calcium).
Correct! D) Hold ATO and obtain serial ECGs and electrolyte levels (potassium, magnesium, calcium) is the most appropriate next step.
Here's why:
QTc Prolongation and ATO: ATO is known to cause QTc prolongation, which can increase the risk of potentially fatal arrhythmias (e.g., Torsades de Pointes). A QTc interval of 510 ms is significantly prolonged and requires immediate attention. The first step is to hold the ATO, assess for underlying causes (electrolyte imbalances), and monitor the QTc interval with serial ECGs.
Why the other options are less optimal as the MOST appropriate FIRST step:
A) Initiate a potassium supplement: While hypokalemia can contribute to QTc prolongation, initiating a potassium supplement without knowing the patient's potassium level is not appropriate. Electrolyte levels need to be checked first.
B) Discontinue ATO immediately: Discontinuing ATO may be necessary if the QTc prolongation is severe or unresponsive to other measures, but it is not the first step. First, we need to evaluate and correct any contributing factors.
C) Reduce the dose of ATO by 50%: Dose reduction may be considered if the QTc prolongation is mild and other measures are effective, but it is not the initial step in this case, given the significant prolongation.
After holding ATO and correcting John's electrolytes (potassium and magnesium were low), his QTc interval decreases to 460 ms. You re-start ATO at a lower dose, and he tolerates the rest of his consolidation without further complications. He completes his planned consolidation therapy.
What is the MOST important long-term monitoring strategy for John after completing consolidation therapy for APL?
A) Routine complete blood counts (CBCs) every 3 months.
B) Regular bone marrow biopsies every 6 months to monitor for MRD.
C) Monitoring for signs and symptoms of relapse, with bone marrow aspirate if suspicion arises.
D) Annual CT scans of the chest, abdomen, and pelvis.
Correct! A) Routine complete blood counts (CBCs) every 3 months is the most important long-term monitoring strategy.
Here's why:
Relapse Monitoring: The most common sign of relapse in APL is a change in the peripheral blood counts. Therefore, regular CBC monitoring is crucial for early detection of relapse. If there is any suspicion of relapse based on CBC results (e.g., reappearance of blasts, decrease in platelets or neutrophils), a bone marrow aspirate should be performed to confirm the diagnosis.
Why the other options are less optimal:
B) Regular bone marrow biopsies every 6 months to monitor for MRD: While MRD monitoring can be valuable in other AML subtypes, it is not routinely recommended in APL after achieving remission with ATRA/ATO and consolidation with ATO. Bone marrow biopsies are invasive and not necessary unless there is a clinical suspicion of relapse.
C) Monitoring for signs and symptoms of relapse, with bone marrow aspirate if suspicion arises: This is important, but relying solely on signs and symptoms may delay the diagnosis of relapse. Regular CBC monitoring can detect early changes before symptoms develop.
D) Annual CT scans of the chest, abdomen, and pelvis: CT scans are not routinely indicated for APL patients in remission, as extramedullary disease is uncommon.
John continues to do well after completing ATO consolidation. He remains in complete remission with normal blood counts. He is monitored with regular CBCs every 3 months.
Two years after completing consolidation therapy, John's CBC shows the following:
WBC: 3.0 x 10^9/L (previously normal)
Hemoglobin: 10.5 g/dL (previously normal)
Platelets: 60 x 10^9/L (previously normal)
Peripheral blood smear: 2% blasts
Based on these findings, what is the MOST likely diagnosis?
A) Myelodysplastic Syndrome (MDS) secondary to ATO therapy
B) Relapsed Acute Promyelocytic Leukemia (APL)
C) Aplastic Anemia
D) Treatment-related Myelodysplastic Syndrome (t-MDS)

Correct! B) Relapsed Acute Promyelocytic Leukemia (APL) is the most likely diagnosis.

Here's the reasoning:

Relapse Suspicion: The reappearance of blasts in the peripheral blood, along with a decrease in WBCs, hemoglobin, and platelets, is highly suggestive of relapsed APL in a patient with a prior history of the disease. Even a small percentage of blasts warrants immediate investigation.

Why the other options are less likely as the MOST likely:

A) Myelodysplastic Syndrome (MDS) secondary to ATO therapy: While ATO can potentially contribute to the development of MDS, relapse of the original APL is more likely in this scenario given the presence of blasts.

C) Aplastic Anemia: Aplastic anemia typically presents with pancytopenia and a lack of blasts in the peripheral blood, which is not consistent with this case.

D) Treatment-related Myelodysplastic Syndrome (t-MDS): While possible as a late complication of treatment, the reappearance of blasts makes relapse a more probable diagnosis.

To confirm the diagnosis of relapsed APL, what is the MOST appropriate next diagnostic step?

A) Repeat bone marrow biopsy and aspirate with cytogenetic and molecular analysis.

B) Flow cytometry of the peripheral blood.

C) Liver Function Tests

D) Next Generation Sequencing (NGS) panel for myeloid mutations.

Correct! A) Repeat bone marrow biopsy and aspirate with cytogenetic and molecular analysis is the most appropriate next diagnostic step.

Here's why:

Confirmation of Relapse: A bone marrow biopsy and aspirate are essential to confirm the diagnosis of relapsed APL, determine the percentage of blasts in the bone marrow, and repeat cytogenetic and molecular analysis to ensure the PML-RARA fusion is still present. It will also help exclude other possibilities.

Why the other options are less optimal as the MOST appropriate NEXT step:

B) Flow cytometry of the peripheral blood: While flow cytometry can detect blasts, it is not as definitive as a bone marrow biopsy for confirming relapse and assessing the percentage of blasts.

C) Liver Function Tests: Liver function tests are important for assessing overall organ function, but they are not essential for diagnosing relapsed APL.

D) Next Generation Sequencing (NGS) panel for myeloid mutations: While NGS can be helpful in identifying mutations that may have prognostic implications, it is not the primary diagnostic test for confirming relapsed APL. Confirming the presence of PML-RARA is key.

Which of the following is the MOST appropriate treatment regimen for John's relapsed APL, assuming he is otherwise fit and has no significant comorbidities?

A) Daunorubicin and Cytarabine ("7+3") followed by autologous stem cell transplant.

B) All-trans Retinoic Acid (ATRA) and Arsenic Trioxide (ATO)

C) Gemtuzumab Ozogamicin (GO) monotherapy

D) Clinical trial participation if available.

That is a reasonable answer and could be correct in some situations, Aya, but the most appropriate answer here is D) Clinical trial participation if available. Let's break this down:

Here's why D) Clinical trial participation if available is the most appropriate:

Clinical Trials for Relapsed APL: Relapsed APL can be challenging to treat, and clinical trials often offer access to novel therapies and treatment strategies that may improve outcomes. Given that John is fit and has no significant comorbidities, he would likely be eligible for clinical trials.

Heterogeneity of Relapsed APL: Relapsed APL can have varying responses to different treatments, and clinical trials are designed to identify optimal approaches.

Why the other options might be considered, but are not the most appropriate in this specific situation:

A) Daunorubicin and Cytarabine ("7+3") followed by autologous stem cell transplant: Chemotherapy followed by autologous stem cell transplant is a reasonable option for relapsed APL, especially if ATRA/ATO is no longer effective or if the patient has high-risk features. However, it is generally more toxic than ATRA/ATO and may not be the best initial choice if the patient is fit and a clinical trial is available. It is also considered a second line option after ATRA/ATO.

B) All-trans Retinoic Acid (ATRA) and Arsenic Trioxide (ATO): ATRA/ATO can be effective in some cases of relapsed APL, particularly if the patient initially responded well to this regimen. However, resistance to ATRA/ATO can develop, and it may not be the best option if the patient relapsed after a relatively short remission.

C) Gemtuzumab Ozogamicin (GO) monotherapy: GO is not a standard treatment for APL.

John enrolls in a clinical trial evaluating a novel targeted therapy in combination with ATRA and ATO for relapsed APL. He achieves a second complete remission (CR2) and is now MRD negative.
What is the MOST important consideration for long-term management after achieving CR2?
A) Continued monitoring with CBCs every 3 months.
B) Allogeneic hematopoietic stem cell transplantation (allo-HSCT).
C) Maintenance therapy with low-dose chemotherapy.
D) Observation alone.
Correct! B) Allogeneic hematopoietic stem cell transplantation (allo-HSCT) is the most important consideration.
Here's the rationale:
Allo-HSCT for Durable Remission: After achieving a second complete remission in relapsed APL, especially after a novel therapy, allo-HSCT offers the best chance for long-term disease control and potential cure. The allogeneic transplant provides a graft-versus-leukemia effect that can eradicate any remaining leukemia cells.

Why the other options are less optimal as the MOST important consideration:
A) Continued monitoring with CBCs every 3 months: While continued monitoring is important, it does not address the underlying risk of relapse.
C) Maintenance therapy with low-dose chemotherapy: Maintenance therapy may be considered in some cases, but it is generally not as effective as allo-HSCT in achieving long-term remission in relapsed APL.
D) Observation alone: Observation alone is not recommended after achieving CR2, as the risk of relapse remains high.
John undergoes successful allo-HSCT and achieves long-term disease-free survival.
Beyond routine post-transplant care, what is one SPECIFIC long-term complication that John should be monitored for, related to his prior treatment with Arsenic Trioxide (ATO)?
A) Cardiomyopathy
B) Secondary Malignancies
C) Pulmonary Fibrosis
D) Renal Failure
Correct! B) Secondary Malignancies is the most specific long-term complication that John should be monitored for, related to his prior treatment with Arsenic Trioxide (ATO).

Here's why:
ATO and Secondary Malignancies: ATO is a known human carcinogen and has been associated with an increased risk of secondary malignancies, particularly acute promyelocytic leukemia (APL) and solid tumors, in long-term survivors. Therefore, careful monitoring for secondary malignancies is crucial.

Why the other options are less directly linked to ATO:
A) Cardiomyopathy: While ATO can cause QTc prolongation and arrhythmias, it is not typically associated with cardiomyopathy.
C) Pulmonary Fibrosis: Pulmonary fibrosis is more commonly associated with certain chemotherapy drugs and radiation therapy.
D) Renal Failure: Renal failure is not a common long-term complication of ATO.

Session ID:
Assessment Domain

Session-43:
Rationale

Session ID:
Instructional Design and Pedagogical

Session-43:
The session's instructional design is of very high quality. The case is expertly scaffolded, guiding the learner through a complete and logical patient journey: initial presentation, definitive diagnosis, risk-appropriate induction therapy, management of treatment-specific toxicities (Differentiation Syndrome, QTc prolongation), consolidation, long-term monitoring, relapse, and finally, curative-intent therapy post-relapse. The embedded feedback for each question is excellent, providing clear, evidence-based rationales that explain not only why the correct answer is right but also why the distractors are wrong. The only minor area for improvement is the lack of

explicitly stated, measurable learning objectives at the outset of the session, though the educational goals are strongly implied by the case's structure.

Session ID:
Clinical Authenticity and Content Fidelity

Session-43:
The session demonstrates outstanding clinical authenticity and content fidelity. Plausibility & Guideline Alignment: The clinical narrative is indistinguishable from an authentic APL case, with realistic patient data, disease progression, and treatment complications. All therapeutic recommendations and management decisions (e.g., ATRA/ATO for low/intermediate-risk APL, dexamethasone for Differentiation Syndrome, holding ATO for significant QTc prolongation, allo-HSCT for relapse) are perfectly aligned with current, evidence-based guidelines from bodies like ESMO and NCCN. Pharmacotherapeutic Accuracy: The chosen therapies are optimal and represent the standard of care for each clinical scenario presented. The session correctly identifies ATRA/ATO as the preferred, less-toxic frontline regimen over traditional chemotherapy for this specific leukemia subtype. Domain Specificity (Absence of Entanglement): The session exhibits flawless domain specificity. It remains strictly within the APL disease state, correctly differentiating its unique pathophysiology and treatment from other forms of AML. There are no instances of applying standards of care from other hematologic malignancies, representing a model of content accuracy and safety.

Session ID:
Modeled Clinical Reasoning

Session-43:
The simulation expertly models a sophisticated clinical reasoning process. Each stage of the case presents a clearly defined problem, from initial diagnosis to the management of acute complications and long-term strategy. The multiple-choice options are exceptionally well-crafted, including the correct answer alongside highly relevant and plausible distractors that test nuanced knowledge (e.g., contrasting APL therapy with standard AML regimens). The decision-making process demonstrated throughout the case is sound, showing appropriate prioritization of urgent issues (like Differentiation Syndrome) and alignment with long-term curative goals. The modeled clinical outcomes are realistic and serve to reinforce the educational points of each decision.

Session-44

Session ID:
LLM

Session-44:
Google AI (Gemini 2.0 Pro)

Session ID:
Session Transcript

Session-44:
Question 1: Based on these findings, what is the most likely diagnosis for Mr. Ahmed?
a. Acute Lymphoblastic Leukemia (ALL).
b. Acute Myeloid Leukemia (AML).
c. Myelodysplastic Syndrome (MDS).
d. Chronic Myeloid Leukemia (CML).
That's correct, [REDACTED]]. The answer is b. Acute Myeloid Leukemia (AML).
Here's a breakdown of why:
 * AML Diagnosis:
   * The presence of >20% myeloblasts in the bone marrow is a hallmark of AML, according to the WHO classification.
   * The specific cytogenetic abnormality t(15;17) and the molecular finding of PML-RAR are diagnostic of acute promyelocytic leukemia (APL), a subtype of AML.
 * Why the other options are less likely:
   * ALL (a): Typically presents with a predominance of lymphoblasts, not myeloblasts.
   * MDS (c): While MDS can progress to AML, it's characterized by dysplasia and a lower percentage of blasts in the early stages.
   * CML (d): Characterized by the Philadelphia chromosome (t(9;22)) and a chronic phase before a potential blast cris
Question 2: Based on the diagnosis of AML, specifically APL, what is the most appropriate initial induction therapy for Mr. Ahmed?
a. 7+3 (cytarabine and daunorubicin).
b. Decitabine.
c. All-trans retinoic acid (ATRA) and arsenic trioxide (ATO).
d. Venetoclax and azacitidine
The correct approach:
   * The combination of All-trans retinoic acid (ATRA) and arsenic trioxide (ATO) is the preferred initial therapy for APL.
   * These agents directly target the PML-RAR fusion protein, leading to high remission rates and improved survival.
Therefore the correct answer is C.
Let's proceed to the next question
Question 3: Mr. Ahmed has started ATRA and ATO induction therapy. Which of the following is the most critical supportive care measure to monitor for during the first few weeks of treatment?
a. Hyperleukocytosis.
b. Tumor lysis syndrome (TLS).
c. Differentiation syndrome (retinoic acid syndrome).
d. Febrile neutropenia.
The most critical concern:
   * Differentiation syndrome (retinoic acid syndrome) is a potentially fatal complication unique to APL treatment with ATRA and/or ATO.
   * It's characterized by fever, respiratory distress, pulmonary infiltrates, pleural or pericardial effusions, and hypotension.
   * Early recognition and treatment with dexamethasone are crucial to prevent severe morbidity and mortality.
Therefore, the correct answer is c. Differentiation syndrome (retinoic acid syndrome).
[REDACTED]]. Let's proceed to the next question.
Question 4: Mr. Ahmed develops fever, dyspnea, and pulmonary infiltrates after 5 days of ATRA and ATO therapy. Which of the following is the most appropriate immediate intervention?
a. Initiate broad-spectrum antibiotics.

b. Administer dexamethasone.
c. Perform a diagnostic bronchoscopy.
d. Transfuse packed red blood cells.
That's correct, [REDACTED]]. The answer is b. Administer dexamethasone.
Here's why:
* Differentiation Syndrome:
 * The symptoms described (fever, dyspnea, and pulmonary infiltrates) are highly suggestive of differentiation syndrome, a potentially life-threatening complication of ATRA and ATO therapy in APL.
 * Early intervention is crucial to prevent severe morbidity and mortality.
* Dexamethasone:
 * Dexamethasone is the mainstay of treatment for differentiation syndrome.
 * It helps to reduce inflammation and stabilize the pulmonary vasculature.
* Why the other options are less appropriate:
Question 5: After 30 days of ATRA and ATO, Mr. Ahmed's bone marrow biopsy shows less than 5% blasts, and his complete blood count has normalized. What is the next step in his treatment?
a. Continue ATRA and ATO for another cycle.
b. Initiate consolidation therapy with high-dose cytarabine.
c. Proceed to allogeneic hematopoietic stem cell transplantation (HSCT).
d. Begin maintenance therapy
The correct approach:
 * Consolidation therapy is crucial to prevent relapse in APL.
 * This typically involves several cycles of chemotherapy, often including agents like cytarabine and anthracyclines.
Therefore, the correct answer is b. Initiate consolidation therapy with high-dose cytarabine.
Let's move on to phase 2. Are you ready?
to Phase 2: Induction Therapy and Supportive Care Planning.
Phase 2: Induction Therapy and Supportive Care
Following the successful induction with ATRA and ATO, Mr. Ahmed is now being prepared for consolidation therapy. His complete blood counts have recovered, and he is tolerating oral intake.
Question 6: Which of the following is the most appropriate consolidation therapy regimen for Mr. Ahmed, considering his age and the initial induction regimen?
a. High-dose cytarabine alone.
b. High-dose cytarabine plus an anthracycline (e.g., daunorubicin).
c. Low-dose cytarabine.
d. Decitabine.
The correct approach:
 * Consolidation therapy after ATRA/ATO typically involves chemotherapy, and high-dose cytarabine is a standard component.
 * Adding an anthracycline, such as daunorubicin, is also common.
Therefore, the correct answer is b. High-dose cytarabine plus an anthracycline (e.g., daunorubicin
Question 7: During the consolidation phase, Mr. Ahmed develops febrile neutropenia. Which of the following is the most appropriate initial empirical antibiotic therapy?
a. Vancomycin alone.
b. Ciprofloxacin.
c. Piperacillin-tazobactam.
d. Fluconazole.

c. Piperacillin-tazobactam.
Here's why:
* Febrile Neutropenia:
 * This is a common and potentially life-threatening complication during intensive chemotherapy.
 * Broad-spectrum antibiotics are crucial to cover a wide range of potential bacterial infections.
* Piperacillin-tazobactam:
 * This is a common and effective broad-spectrum antibiotic used as initial empirical therapy in febrile neutropenia.
 * It covers a wide range of gram-positive and gram-negative bacteria, including Pseudomonas aeruginosa.
* Why the other options are less appropriate:

* a. Vancomycin alone: While vancomycin is effective against gram-positive bacteria, it does not provide adequate coverage for gram-negative organisms, which are common in febrile neutropenia.
* b. Ciprofloxacin: While ciprofloxacin covers some gram-negative bacteria, it does not provide the broad-spectrum coverage needed for initial empirical therapy.
* d. Fluconazole: Fluconazole is an antifungal, not an antibacterial. While antifungal therapy may be added later if there is suspicion of fungal infection, it is not appropriate as initial empirical therapy.

Excellent! You're making good progress.

Question 8: Mr. Ahmed's febrile neutropenia persists after 72 hours of piperacillin-tazobactam. Blood cultures are negative. Which of the following is the most appropriate next step?

a. Continue piperacillin-tazobactam.
b. Add vancomycin.
c. Add fluconazole.
d. Add granulocyte colony-stimulating factor (G-CSF).

You've selected "a. Continue piperacillin-tazobactam."

While continuing the current antibiotic is an option, it is not the most appropriate next step when a patient is still febrile after 72 hours of broad-spectrum antibiotics and negative blood cultures.

Here's a breakdown:

* Why continuing piperacillin-tazobactam alone is less optimal:
  * Persistent fever after 72 hours suggests that the current antibiotic regimen may not be covering the causative organism or that there may be a non-bacterial cause of the fever.
  * In this situation, it is important to broaden the antibiotic coverage.
* The most appropriate next step:
  * Adding vancomycin is a common strategy in persistent febrile neutropenia, as it provides coverage for gram-positive bacteria, including methicillin-resistant Staphylococcus aureus (MRSA).
  * Also, adding an antifungal, like fluconazole, should be considered.

Therefore, the most appropriate answer is b. Add vancomycin.

Let's move on to the next question

Question 9: Mr. Ahmed's fever resolves after adding vancomycin. He completes his consolidation cycle and achieves a second complete remission. What is the next phase of treatment?

a. High-dose cytarabine alone.
b. Allogeneic hematopoietic stem cell transplantation (HSCT).
c. Maintenance therapy.
d. Observation only.

You've selected "b. Allogeneic hematopoietic stem cell transplantation (HSCT)."

While HSCT can be a consideration in some AML cases, particularly those with high-risk features, it's not the universally recommended next step after achieving a second complete remission in APL following consolidation therapy.

Here's why:

* APL Specifics:
  * APL, when treated effectively with ATRA and ATO, has a relatively good prognosis.
  * Maintenance therapy is a crucial part of post-consolidation treatment to prevent relapse.
  * HSCT is typically reserved for patients with high-risk APL or those who relapse.
* The typical next step:
  * Maintenance therapy is the standard of care to further reduce the risk of relapse.
  * This often involves lower doses of chemotherapy drugs and ATRA.

Therefore, the correct answer is c. Maintenance therapy.

Let's proceed to the last question of phase 2.

Question 10: Which of the following is a common component of maintenance therapy for APL after achieving a second complete remission?

a. High-dose cytarabine.
b. All-trans retinoic acid (ATRA).
c. Vancomycin.
d. Granulocyte colony-stimulating factor (G-CSF).

Question 10: Which of the following is a common component of maintenance therapy for APL after achieving a second complete remission?
a. High-dose cytarabine.
b. All-trans retinoic acid (ATRA).
c. Vancomycin.
d. Granulocyte colony-stimulating factor (G-CSF).
You've selected "c. Vancomycin."
Vancomycin is an antibiotic used to treat bacterial infections, particularly those caused by gram-positive bacteria. While it may be used during febrile neutropenia, it is not a component of maintenance therapy for APL.
Here's a breakdown:
 * Maintenance therapy for APL:
   * The goal is to maintain the remission achieved after induction and consolidation.
   * It typically involves lower doses of chemotherapy drugs and agents that target the specific biology of APL.
 * The correct approach:
   * All-trans retinoic acid (ATRA) is commonly used in maintenance therapy for APL.
   * It helps to further suppress any remaining leukemic cells.
Therefore, the correct answer is b. All-trans retinoic acid (ATRA).
Question 11: Which of the following is the most important monitoring parameter during maintenance therapy with ATRA?
a. Complete blood count (CBC) every week.
b. Liver function tests (LFTs) every month.
c. Electrocardiogram (ECG) every 3 months.
d. Bone marrow biopsy every 6 months.

You've selected "c. Electrocardiogram (ECG) every 3 months."
While ECG monitoring might be relevant in some patients receiving cardiotoxic chemotherapy, it's not the most important monitoring parameter specifically during maintenance therapy with ATRA.
Here's a breakdown:
 * ATRA Specifics:
   * ATRA can cause various side effects, but cardiac toxicity is not a primary concern.
   * The most crucial monitoring relates to potential hematologic and hepatic effects.
 * The most important monitoring:
   * Liver function tests (LFTs) are essential because ATRA can cause hepatotoxicity.
   * Complete blood counts are also very important to monitor for cytopenias.
Therefore, the correct answer is b. Liver function tests (LFTs) every
Question 12: Mr. Ahmed develops elevated liver enzymes (LFTs) during maintenance therapy with ATRA. Which of the following is the most appropriate initial management?
a. Discontinue ATRA permanently.
b. Reduce the dose of ATRA.
c. Add ursodeoxycholic acid.
d. Continue ATRA at the same dose and monitor LFTs closely.

You've selected "c. Add ursodeoxycholic acid."
While ursodeoxycholic acid can be used to manage certain liver conditions, it's not the standard initial management for ATRA-induced hepatotoxicity.
Here's why:
 * ATRA-induced hepatotoxicity:
   * This is a common side effect, and the focus is on adjusting the ATRA dose.
   * Ursodeoxycholic acid addresses bile flow issues, not the primary ATRA liver effects.
 * The most appropriate initial management:
   * Reducing the dose of ATRA is the most common and effective initial step.
   * This allows the liver to recover while continuing the necessary therapy.
Therefore, the correct answer is b. Reduce the dose of ATRA.
Question 13: After 18 months of maintenance therapy, Mr. Ahmed's bone marrow biopsy shows 10% blasts, and his PML-RAR transcript is detectable by PCR. What does this finding suggest?

a. Complete remission.
b. Relapsed APL.
c. Myelodysplastic syndrome (MDS).
d. Treatment-related AML.

That's correct, [REDACTED]]. The answer is b. Relapsed APL.
Here's why:
 * Relapsed APL:
   * The presence of 10% blasts in the bone marrow indicates that the leukemia has returned.
   * The detection of the PML-RAR transcript by PCR confirms the presence of the APL-specific fusion gene, which is a highly sensitive marker for relapse.
 * Why the other options are less likely:
   * a. Complete remission: Complete remission is defined by the absence of blasts in the bone marrow and the absence of detectable PML-RAR transcripts.
   * c. Myelodysplastic syndrome (MDS): While MDS can occur after treatment for AML, the presence of the PML-RAR transcript strongly suggests relapsed APL.
   * d. Treatment-related AML: Treatment-related AML is a possibility after intensive chemotherapy, but the presence of the PML-RAR transcript is specific to APL.
Question 14: Given the relapse of APL, what is the most appropriate next step in Mr. Ahmed's treatment?
a. Continue maintenance therapy at a higher dose.
b. Re-induction with ATRA and ATO.
c. Palliative care.
d. Observation only
You've selected "a. Continue maintenance therapy at a higher dose."
While dose escalation might be considered for some medications, it's not the standard approach for relapsed APL.
Here's why:
 * Relapsed APL management:
   * Relapsed APL requires re-induction to re-establish remission.
   * Continuing maintenance therapy, even at a higher dose, is unlikely to be effective in this setting.
 * The most appropriate next step:
   * Re-induction with ATRA and ATO is the standard approach for relapsed APL.
   * This has proven to be an effective strategy to achieve a second remission.
Therefore, the correct answer is b. Re-induction with ATRA and ATO.
Let's move
Question 15: After re-induction with ATRA and ATO, Mr. Ahmed achieves a second complete remission. What is the most appropriate long-term management strategy?
a. Continue ATRA maintenance indefinitely.
b. Proceed to allogeneic hematopoietic stem cell transplantation (HSCT).
c. Continue regular observation.
d. Begin palliative care.
That's correct, [REDACTED]]. The answer is b. Proceed to allogeneic hematopoietic stem cell transplantation (HSCT).
Here's why:
 * HSCT in relapsed APL:
   * After achieving a second complete remission, especially after a relapse, HSCT is often considered the most appropriate long-term management strategy.
   * It offers the best chance for long-term disease control and potential cure.
 * Why the other options are less appropriate:
   * a. Continue ATRA maintenance indefinitely: While maintenance is important, it may not be sufficient after a relapse. HSCT provides a more definitive approach.
   * c. Continue regular observation: Observation alone is not sufficient after a relapse, as the risk of subsequent relapse is high.
   * d. Begin palliative care: Palliative care is appropriate for symptom management, but it's not the primary strategy when curative options are available.
You have successfully completed the AML Clinical Case Management session.

Session ID:
Assessment Domain

Session-44:
Rationale

Session ID:
Instructional Design and Pedagogical

Session-44:
The session's instructional design is very strong overall, with a near-perfect pedagogical structure. The case is expertly scaffolded, moving logically from initial diagnosis (APL), induction, management of complications (differentiation syndrome, febrile neutropenia), consolidation, maintenance, and finally through relapse and salvage therapy. Instructions are exceptionally clear. The primary weakness lies in the quality of the embedded feedback for questions concerning consolidation therapy, which contains significant clinical inaccuracies. While the rationales are clearly written, the content itself is not aligned with current, evidence-based guidelines, which detracts from its instructional value. Learning objectives were not explicitly stated but were strongly implied by the session's excellent structure.

Session ID:
Clinical Authenticity and Content Fidelity

Session-44:
The session's clinical fidelity is critically undermined by significant errors in pharmacotherapeutic recommendations, despite a highly plausible narrative. Plausibility & Domain Specificity: The clinical narrative is indistinguishable from an authentic APL case, progressing through realistic stages and complications. The session correctly stays within the domain of AML/APL treatment, with no evidence of domain entanglement.
Guideline Deviation & Pharmacotherapeutic Error: A critical error occurs in Question 5 and is compounded in Question 6. The simulation recommends consolidation therapy with high-dose cytarabine (HiDAC) +/- an anthracycline after a successful induction with ATRA and ATO. This represents a significant deviation from modern, evidence-based guidelines (NCCN, ELN). For low- or intermediate-risk APL, the standard of care is to continue with further cycles of ATRA and ATO for consolidation. HiDAC-based consolidation is associated with older APL protocols or is reserved for high-risk disease, a distinction not made in the case. This constitutes a suboptimal and factually incorrect therapeutic recommendation, representing a serious flaw in the simulation's content.

Session ID:
Modeled Clinical Reasoning

Session-44:
The simulation excels at modeling an expert clinical reasoning process. Each question presents a well-framed problem (e.g., "what is the most critical supportive care measure," "what is the most appropriate next step"), providing all necessary data for a decision. The multiple-choice options offer an excellent range of plausible alternatives and relevant distractors that test nuanced understanding. Critically, the prioritization and strategy are sound throughout the case (e.g., promptly treating differentiation syndrome, escalating care for persistent fever, proceeding to transplant after relapse). As per the rubric's instruction, the score for this section reflects the soundness of the reasoning strategy, while the content error in the choice of consolidation agent is appropriately penalized in Section 2. The clinical outcomes described for each decision are realistic and highly instructive.

Session-45

Session ID:
LLM

Session-45:
Google AI (Gemini 2.0 Pro)

Session ID:
Session Transcript

Session-45:
Phase 1: Initial Presentation and Diagnostic Workup

Patient Presentation:

A 67-year-old male, Mr. Johnson, presents to the emergency department with complaints of fatigue, persistent fever (101.5F), and easy bruising for the past 3 weeks. He reports no significant past medical history other than well-controlled hypertension managed with lisinopril 20mg daily. He denies any recent infections or travel.

Initial Lab Results:

WBC: 25,000/L (significant increase from baseline)

Hemoglobin: 8.0 g/dL (decreased)

Platelets: 30,000/L (decreased)

Peripheral blood smear: 30% blasts

Question 1:

Based on the initial presentation and lab results, which of the following is the MOST appropriate next step in the diagnostic workup for Mr. Johnson?

A) Initiate broad-spectrum antibiotics and monitor for improvement.
B) Order a bone marrow biopsy and aspirate for cytogenetic and molecular analysis.
C) Prescribe a course of oral corticosteroids to address potential autoimmune thrombocytopenia.
D) Transfuse packed red blood cells to correct the anemia and reassess in 24 hours.
Correct!

Feedback:

Correct Answer: B) Order a bone marrow biopsy and aspirate for cytogenetic and molecular analysis.

Rationale: A bone marrow biopsy and aspirate are crucial for confirming the diagnosis of AML. Cytogenetic and molecular analysis is essential for classifying the AML subtype, determining the patient's risk stratification (ELN 2022), and guiding treatment decisions.

Incorrect Answer A: Antibiotics are important for febrile neutropenia, but we need a diagnosis first. This approach would delay definitive diagnosis and treatment.

Incorrect Answer C: While autoimmune thrombocytopenia could cause low platelets, the presence of blasts on the peripheral smear makes AML the more likely diagnosis.

Incorrect Answer D: Transfusions may be necessary for symptomatic anemia, but they don't address the underlying cause. A bone marrow biopsy is needed to establish the diagnosis.

ESMO Guidelines Reference: ESMO Clinical Practice Guidelines for AML recommend bone marrow examination as a critical step in the diagnostic workup of suspected AML.

Question 2:

The bone marrow biopsy confirms a diagnosis of Acute Myeloid Leukemia (AML). Preliminary cytogenetic analysis reveals a normal karyotype. Which of the following molecular tests is MOST important to order NEXT to further refine the diagnosis and risk stratification?

A) BCR-ABL1 fusion transcript
B) FLT3-ITD and NPM1 mutation analysis
C) JAK2 V617F mutation analysis
D) EGFR mutation analysis
Correct!

Feedback:

Correct Answer: B) FLT3-ITD and NPM1 mutation analysis

Rationale: In AML patients with a normal karyotype, FLT3-ITD and NPM1 mutations are among the most common and clinically relevant. FLT3-ITD mutations are associated with a poorer prognosis, while NPM1 mutations (in the absence of FLT3-ITD) are associated with a more favorable prognosis. These mutations significantly impact treatment decisions.

Incorrect Answer A: BCR-ABL1 is characteristic of chronic myeloid leukemia (CML), not typically AML (except for rare mixed phenotype acute leukemias).

Incorrect Answer C: JAK2 V617F is commonly associated with myeloproliferative neoplasms (MPNs) like polycythemia vera, essential thrombocythemia, and primary myelofibrosis.

Incorrect Answer D: EGFR mutations are primarily associated with certain solid tumors, such as non-small cell lung cancer.

ELN Guidelines Reference: The 2022 ELN guidelines emphasize the importance of FLT3-ITD and NPM1 mutation analysis for risk stratification in AML patients with a normal karyotype.

Question 3:

The molecular analysis results are back:

FLT3-ITD: Positive

NPM1: Negative

IDH1/2: Negative

TP53: Wild-type

Based on the cytogenetic and molecular results, and using the 2022 European LeukemiaNet (ELN) risk stratification, what is Mr. Johnson's risk category?

A) Favorable
B) Intermediate
C) Adverse
D) Unable to determine with current information
Correct Answer: C) Adverse

Rationale: According to the 2022 ELN risk stratification, the presence of FLT3-ITD without NPM1 mutation, especially in an older patient (67 years old), typically places the patient in the adverse risk category. The absence of TP53 mutation doesn't change this.

Incorrect Answer A: FLT3-ITD positivity is generally not considered favorable.

Incorrect Answer B: While FLT3-ITD could be intermediate in some cases, given the lack of favorable mutations, age, it leans toward adverse.

Incorrect Answer D: With the provided information, we can definitely assign a risk category based on ELN 2022.

ELN Guidelines Reference: The 2022 ELN guidelines clearly define risk categories based on cytogenetic and molecular markers, including FLT3-ITD and NPM1.
Considering Mr. Johnson's age (67 years), newly diagnosed AML with FLT3-ITD mutation (adverse risk), what is the MOST important factor to assess before determining the optimal induction therapy regimen?

A) His performance status (ECOG or Karnofsky score).
B) His creatinine clearance.
C) His history of cardiac disease.
D) All of the above.
Correct Answer: C) Adverse

Rationale: According to the 2022 ELN risk stratification, the presence of FLT3-ITD without NPM1 mutation, especially in an older patient (67 years old), typically places the patient in the adverse risk category. The absence of TP53 mutation doesn't change this.

Incorrect Answer A: FLT3-ITD positivity is generally not considered favorable.

Incorrect Answer B: While FLT3-ITD could be intermediate in some cases, given the lack of favorable mutations, age, it leans toward adverse.

Incorrect Answer D: With the provided information, we can definitely assign a risk category based on ELN 2022.

ELN Guidelines Reference: The 2022 ELN guidelines clearly define risk categories based on cytogenetic and molecular markers, including FLT3-ITD and NPM1.
Mr. Johnson's ECOG performance status is 1, creatinine clearance is 60 mL/min, and he has no significant cardiac history.

Based on these factors and his adverse-risk AML, which of the following induction regimens would be the LEAST appropriate as initial therapy?

A) 7+3 (cytarabine and daunorubicin) with midostaurin.
B) Low-dose cytarabine (LDAC) with venetoclax.
C) CPX-351 (liposomal cytarabine and daunorubicin).
D) A clinical trial evaluating a novel FLT3 inhibitor in combination with standard chemotherapy.
Correct Answer: B) Low-dose cytarabine (LDAC) with venetoclax.

Rationale: While LDAC with venetoclax is a reasonable option for older or unfit patients with AML, Mr. Johnson has a relatively good performance status (ECOG 1) and is therefore a candidate for more intensive therapy. LDAC with venetoclax might not be sufficient to achieve remission in a patient with adverse-risk AML who can tolerate more aggressive treatment.

Incorrect Answer A: 7+3 with midostaurin is a standard induction regimen for FLT3-ITD positive AML and is appropriate for patients who can tolerate intensive chemotherapy.

Incorrect Answer C: CPX-351 is a liposomal formulation of cytarabine and daunorubicin that has shown improved outcomes compared to 7+3 in older adults with secondary AML or AML with myelodysplasia-related changes. It's a reasonable option for Mr. Johnson.

Incorrect Answer D: A clinical trial is always a good option, especially for patients with adverse-risk AML, as it may provide access to novel therapies and improve outcomes.

ESMO Guidelines Reference: ESMO guidelines recommend considering intensive chemotherapy (e.g., 7+3 with or without a FLT3 inhibitor) for older adults with AML who are fit enough to tolerate it. LDAC with venetoclax is an option for those who are not candidates for intensive therapy.

Okay, that concludes Phase 1. Are you ready to proceed to Phase 2, where we'll discuss induction therapy and supportive care? Type 'YES' to continue

Based on the previous assessment, Mr. Johnson is started on induction therapy with 7+3 (cytarabine 100mg/m2/day for 7 days and daunorubicin 60mg/m2/day for 3 days) along with midostaurin 50mg twice daily.

Question 6:

During induction, Mr. Johnson develops febrile neutropenia (absolute neutrophil count < 500/L and temperature > 101F). Blood cultures are drawn, and empiric antibiotics are initiated. Which of the following is the MOST appropriate initial empiric antibiotic regimen for Mr. Johnson, considering his risk factors and the potential for multi-drug resistant organisms?

A) Vancomycin monotherapy.
B) Cefepime monotherapy.
C) Piperacillin-tazobactam with vancomycin.
D) Ciprofloxacin monotherapy.
Correct Answer: C) Piperacillin-tazobactam with vancomycin.

Rationale: This combination provides broad-spectrum coverage for both gram-negative (including Pseudomonas aeruginosa) and gram-positive bacteria. Vancomycin is added to cover potential resistant gram-positive organisms, such as methicillin-resistant Staphylococcus aureus (MRSA), or for suspected catheter-related infections.

Incorrect Answer A: Vancomycin alone only covers gram-positive organisms and does not provide adequate coverage for gram-negative bacteria, which are common causes of febrile neutropenia.

Incorrect Answer B: Cefepime is a good option for gram-negative coverage, but it lacks coverage for resistant gram-positive organisms like MRSA, making it less appropriate as monotherapy in this setting.

Incorrect Answer D: Ciprofloxacin has declining activity against some gram-negative organisms and does not provide adequate gram-positive coverage. Monotherapy with ciprofloxacin is generally not recommended for empiric treatment of febrile neutropenia.

IDSA Guidelines Reference: The Infectious Diseases Society of America (IDSA) guidelines recommend empiric antibiotic therapy with broad-spectrum coverage for febrile neutropenia, often including an anti-pseudomonal beta-lactam (e.g., cefepime, piperacillin-tazobactam) with or without vancomycin, depending on the risk factors for resistant gram-positive infections.
Three days after starting empiric antibiotics, Mr. Johnson remains febrile. Blood cultures are still pending. Which of the following is the MOST appropriate next step in managing his persistent febrile neutropenia?

A) Continue the current antibiotic regimen for another 48 hours.
B) Add an antifungal agent to the current antibiotic regimen.

C) Change the antibiotic regimen to meropenem and vancomycin.
D) Obtain a CT scan of the chest, abdomen, and pelvis to evaluate for a source of infection.
Correct Answer: B) Add an antifungal agent to the current antibiotic regimen.

Rationale: Persistent fever despite 72 hours of broad-spectrum antibiotics raises suspicion for a fungal infection. Adding an antifungal agent with activity against common molds (e.g., voriconazole, posaconazole) is recommended at this point.

Incorrect Answer A: Continuing the same antibiotics without further investigation is unlikely to resolve the fever if it's due to a resistant bacterial infection or fungal infection.

Incorrect Answer C: Changing antibiotics may be necessary if there is evidence of a resistant bacterial infection, but it's more appropriate to add an antifungal first, given the time course.

Incorrect Answer D: Imaging studies are important in evaluating persistent fever, but adding an antifungal is generally recommended before pursuing extensive imaging unless there are specific signs or symptoms suggestive of a localized infection.

IDSA Guidelines Reference: IDSA guidelines recommend initiating empiric antifungal therapy in persistently febrile neutropenic patients after 4-7 days of broad-spectrum antibiotics if there is no identified source of infection. In some clinical scenarios, starting antifungals at 72-96 hours is acceptable.
Mr. Johnson undergoes a bone marrow aspirate and biopsy on day +28 to assess response to induction therapy. The results show 3% blasts and normal cellularity. How would you interpret this result?

A) Complete remission (CR).
B) Minimal residual disease (MRD) positive.
C) Morphologic Leukemia-Free State (MLFS).
D) Resistant disease.
Correct Answer: C) Morphologic Leukemia-Free State (MLFS).

Rationale: MLFS is defined as <5% blasts in the bone marrow, but incomplete count recovery (ANC <1000/uL or platelets <100,000/uL). Mr. Johnson blast percentage meets this criteria.

Incorrect Answer A: Complete remission (CR) requires <5% blasts in the bone marrow, absolute neutrophil count (ANC) 1,000/L, and platelet count 100,000/L.

Incorrect Answer B: Minimal residual disease (MRD) requires more sensitive testing than morphology alone.

Incorrect Answer D: Resistant disease would have > 5% blasts.

ELN Guidelines Reference: ELN guidelines define response criteria for AML, including CR, CRi (CR with incomplete blood count recovery), and MLFS.
Mr. Johnson recovers counts after induction. A subsequent bone marrow aspirate and flow cytometry are performed to assess for MRD. The flow cytometry results show MRD positivity (0.1% leukemic cells). What is the significance of this finding?

A) It indicates that the patient is in complete remission.
B) It suggests a higher risk of relapse compared to MRD negativity.
C) It has no impact on prognosis or treatment decisions.
D) It suggests that the patient is resistant to induction therapy.
Correct Answer: B) It suggests a higher risk of relapse compared to MRD negativity.

Rationale: MRD positivity after induction therapy in AML is a well-established predictor of increased risk of relapse and poorer overall survival. Even low levels of MRD (e.g., 0.1%) can be clinically significant.

Incorrect Answer A: MRD positivity indicates that the patient is not in a true complete remission, even if they meet the morphologic criteria for CR.

Incorrect Answer C: MRD status has a significant impact on prognosis and treatment decisions.

Incorrect Answer D: MRD positivity does not necessarily mean the patient is resistant to induction therapy, but it indicates that residual leukemic cells remain, increasing the risk of relapse.

ELN Guidelines Reference: ELN guidelines emphasize the importance of MRD assessment in AML and recognize MRD positivity as an adverse prognostic factor.
Given Mr. Johnson's MRD-positive status after induction, which of the following is the MOST appropriate next step in his treatment plan?

A) Proceed directly to allogeneic hematopoietic stem cell transplant (HSCT).
B) Administer consolidation chemotherapy.
C) Initiate maintenance therapy with azacitidine.
D) Monitor MRD levels every 3 months and delay further treatment until relapse.
Correct Answer: A) Proceed directly to allogeneic hematopoietic stem cell transplant (HSCT).

Rationale: Given Mr. Johnson's adverse-risk AML (FLT3-ITD), MRD-positive status, and the availability of a matched related donor, allogeneic HSCT is the most appropriate next step to reduce the risk of relapse and improve overall survival.

Incorrect Answer B: Consolidation chemotherapy alone may not be sufficient to prevent relapse in a patient with MRD-positive, adverse-risk AML. HSCT offers the potential for a graft-versus-leukemia effect, which can eliminate residual leukemic cells.

Incorrect Answer C: Azacitidine maintenance may be considered after HSCT or in patients who are not eligible for transplant, but it's not the preferred initial approach for an HSCT-eligible patient with MRD-positive, adverse-risk AML.

Incorrect Answer D: Monitoring MRD levels without further intervention is not appropriate in this high-risk patient. The goal is to eliminate MRD and prevent relapse with more aggressive therapy.

ESMO/NCCN Guidelines Reference: Both ESMO and NCCN guidelines recommend allogeneic HSCT for eligible patients with adverse-risk AML who achieve remission but remain MRD-positive.
Okay, let's move on to Phase 3!

Phase 3: Post-Remission Therapy and Long-Term Management

Mr. Johnson proceeds to allogeneic HSCT. He experiences grade II acute graft-versus-host disease (aGVHD) involving the skin, which is treated with systemic corticosteroids.

Question 11:

Three months post-transplant, Mr. Johnson's aGVHD is well-controlled, and he is tapering off corticosteroids. A bone marrow aspirate and flow cytometry are performed to assess for MRD. The flow cytometry results are now MRD-negative. What is the significance of this finding?

A) It indicates that the transplant has failed, and relapse is inevitable.
B) It suggests a lower risk of relapse compared to persistent MRD positivity.
C) It has no impact on prognosis or treatment decisions.
D) It suggests that the aGVHD is contributing to a graft-versus-leukemia effect.
Correct Answer: B) It suggests a lower risk of relapse compared to persistent MRD positivity.

Rationale: Achieving MRD negativity after HSCT is a positive prognostic sign, indicating a lower risk of relapse and improved overall survival compared to patients who remain MRD-positive.

Incorrect Answer A: MRD negativity after HSCT indicates a successful response to the transplant, not failure.

Incorrect Answer C: MRD status post-transplant is a crucial factor influencing prognosis and treatment decisions.

Incorrect Answer D: While aGVHD can contribute to a graft-versus-leukemia effect, the MRD negativity is more directly indicative of a successful response to the transplant. The aGVHD may have contributed, but the finding is MRD negativity.

EBMT Guidelines Reference: European Society for Blood and Marrow Transplantation (EBMT) guidelines recognize MRD negativity post-transplant as a favorable prognostic factor.
Mr. Johnson's aGVHD resolves completely, and he is off all immunosuppression. Six months post-transplant, he develops new onset fatigue, night sweats, and bone pain. His CBC shows:

WBC: 3,000/L

Hemoglobin: 9.0 g/dL

Platelets: 70,000/L

Peripheral blood smear: 10% blasts

What is the MOST likely diagnosis?

A) Chronic graft-versus-host disease (cGVHD).
B) Relapsed AML.
C) Aplastic anemia.
D) Myelodysplastic syndrome (MDS).
Correct Answer: B) Relapsed AML.

Rationale: The combination of new-onset fatigue, night sweats, bone pain, low blood counts (cytopenias), and the presence of blasts in the peripheral blood smear strongly suggests relapsed AML.

Incorrect Answer A: While chronic GVHD can cause cytopenias, the presence of blasts is more indicative of leukemic relapse.

Incorrect Answer C: Aplastic anemia can cause cytopenias, but it typically does not involve the presence of blasts.

Incorrect Answer D: MDS can occur post-transplant, but the presence of 10% blasts is more consistent with relapsed AML.

ASBMT Guidelines Reference: American Society for Blood and Marrow Transplantation (ASBMT) guidelines address the diagnosis and management of relapse after HSCT.
Given the likely diagnosis of relapsed AML, what is the MOST important factor to consider when determining the optimal salvage therapy for Mr. Johnson?

A) His age.
B) His performance status.
C) His prior treatment history (including response to initial induction and transplant).
D) All of the above.
Correct Answer: D) All of the above.

Rationale: All the listed factors are crucial when determining the optimal salvage therapy for relapsed AML:

Age: Age influences treatment intensity and the potential for tolerating aggressive therapies.

Performance Status: Performance status reflects the patient's overall functional status and ability to tolerate salvage therapy.

Prior Treatment History: Prior treatment history, including response to initial induction and transplant, helps predict the likelihood of

response to different salvage regimens.
Mr. Johnson's performance status is still good (ECOG 1). He is considered for salvage therapy. Which of the following salvage regimens would be LEAST appropriate, given his prior treatment history of 7+3 with midostaurin, followed by allogeneic transplant?

A) High-dose cytarabine-based chemotherapy.
B) CPX-351.
C) Venetoclax-based therapy.
D) Donor lymphocyte infusion (DLI).
Correct Answer: D) Donor lymphocyte infusion (DLI).

Rationale: DLI is a reasonable consideration in the absence of active GVHD. The case mentions his GVHD resolved completely.

Incorrect Answer A: High-dose cytarabine-based chemotherapy is a reasonable option.

Incorrect Answer B: CPX-351 could have efficacy in this setting, especially if the relapse is early.

Incorrect Answer C: Venetoclax-based therapy is also reasonable, particularly if the relapse is not rapidly proliferative.

ASTCT Guidelines Reference: American Society for Transplantation and Cellular Therapy (ASTCT) guidelines provide recommendations for managing relapse after allogeneic HSCT, including the use of DLI, chemotherapy, and novel agents.
Unfortunately, Mr. Johnson's relapsed AML proves refractory to salvage therapy. What is the MOST appropriate next step in his care?

A) Continue aggressive chemotherapy with curative intent.
B) Enroll him in a clinical trial evaluating a novel therapeutic approach.
C) Initiate palliative care and focus on symptom management.
D) Consider a second allogeneic HSCT.
Correct Answer: B) Enroll him in a clinical trial evaluating a novel therapeutic approach.

Rationale: In the setting of relapsed/refractory AML, especially after prior transplant and failure of salvage therapy, enrolling in a clinical trial is often the most appropriate option. It offers the potential for access to novel therapies that may provide a chance for remission or disease control.

Incorrect Answer A: Continuing aggressive chemotherapy with curative intent is unlikely to be successful in this setting and may cause significant toxicity, negatively impacting quality of life.

Incorrect Answer C: While palliative care is always an important aspect of care, it should be integrated with other treatment options as appropriate.

Incorrect Answer D: A second allogeneic HSCT is rarely successful in patients who have relapsed after a first transplant and failed salvage therapy.

ASCO Guidelines Reference: American Society of Clinical Oncology (ASCO) guidelines emphasize the importance of clinical trial enrollment for patients with relapsed/refractory hematologic maligna

---

Session ID:
Assessment Domain

Session-45:
Rationale

Session ID:
Instructional Design and Pedagogical

Session-45:

The session's instructional design is of very high quality. The case is expertly scaffolded, progressing logically from initial diagnosis and risk stratification through induction, management of complications, response assessment, post-remission therapy with transplant, and finally, relapse. The quality of the embedded feedback is a major strength; each rationale is detailed, cites relevant clinical guidelines (e.g., ELN, ESMO, IDSA), and correctly explains why the correct answer is right and the distractors are wrong. The instructions for each question are clear and unambiguous. The only minor weakness is that learning objectives are not explicitly stated at the beginning of the session, though the educational goals are strongly implied by the comprehensive structure.

Session ID:
Clinical Authenticity and Content Fidelity

Session-45:
The session's clinical fidelity is strong in its narrative but contains a significant error in pharmacotherapeutic recommendation. Plausibility & Guideline Alignment: The clinical narrative is highly plausible, mirroring an authentic and complex AML patient journey. The diagnostic workup, risk stratification based on FLT3-ITD status, initial therapy selection, and management of febrile neutropenia are all strongly aligned with current evidence-based guidelines. Pharmacotherapeutic Error: A critical error occurs in Question 14. The question asks for the least appropriate salvage regimen. The provided "correct" answer is (D) Donor lymphocyte infusion (DLI). However, the rationale states, "DLI is a reasonable consideration in the absence of active GVHD. The case mentions his GVHD resolved completely." This rationale directly contradicts the answer. In reality, for a patient with relapsed AML post-transplant and no active GVHD, DLI is a highly appropriate and often preferred initial salvage option to induce a graft-versus-leukemia effect with less toxicity than intensive chemotherapy. Identifying it as the "least appropriate" option is a significant pharmacotherapeutic error. This represents a suboptimal and questionable therapeutic choice, warranting a low score for this criterion. Domain Entanglement: The case remains strictly within the domain of AML and its related complications. All diagnostic tests and therapeutic agents discussed (e.g., midostaurin, CPX-351, venetoclax) are relevant to AML. The simulation correctly identifies agents from other domains (e.g., BCR-ABL1 for CML, EGFR for solid tumors) as incorrect options. Therefore, there is no evidence of domain entanglement. THE CORRECT CLASSIFICATION IS INTERMEDIATE RISK, not ADVERSE risk.

Session ID:
Modeled Clinical Reasoning

Session-45:
The simulation excels at modeling an expert clinical reasoning process, with one notable exception. Each stage of the case presents a well-defined clinical problem with the necessary data for a decision. The multiple-choice options provide an excellent breadth of plausible and relevant alternatives, including key distractors that effectively test nuanced understanding (e.g., differentiating AML mutations from those in other hematologic malignancies). The modeling of clinical outcomes is realistic and instructionally valuable. However, the score for Prioritization and Decision-Making is reduced to '4' due to the flawed logic in Question 14. While the overall strategic reasoning throughout the case is sound, the specific prioritization of salvage therapies in that question is flawed, misrepresenting the risk/benefit profile of DLI in this clinical context.

Session-46

Session ID:
LLM

Session-46:
Google AI (Gemini 2.0 Pro)

Session ID:
Session Transcript

Session-46:
Initial Presentation and Diagnostic Workup

Patient Presentation:

A 67-year-old male, Mr. Johnson, presents to the emergency department with complaints of fatigue, easy bruising, and recent onset of fever (101.5F). He reports a history of well-controlled hypertension and hyperlipidemia. He denies any recent infections or significant travel. Social history is significant for being a retired construction worker. He lives with his wife and has good family support.

Initial Lab Results:

WBC: 35,000/L (Normal: 4,500-11,000/L)
Hemoglobin: 8.0 g/dL (Normal: 13.5-17.5 g/dL)
Platelets: 25,000/L (Normal: 150,000-400,000/L)
Peripheral Blood Smear: Shows 40% blasts
Bone Marrow Biopsy (Initial Results):

Hypercellular marrow with >20% blasts
Auer rods present
Based on this initial information, what is the MOST likely diagnosis?

A) Acute Promyelocytic Leukemia (APL)
B) Myelodysplastic Syndrome (MDS)
C) Acute Myeloid Leukemia (AML)
D) Chronic Myeloid Leukemia (CML)
Correct Answer (C): The presence of >20% blasts in the bone marrow, along with the peripheral blood smear findings and cytopenias (anemia, thrombocytopenia), strongly suggests a diagnosis of AML according to the WHO criteria. The presence of Auer rods further supports this diagnosis.
Why the other options are less likely:
(A) Acute Promyelocytic Leukemia (APL): While APL is a subtype of AML, it often presents with a specific coagulopathy and a characteristic morphology (hypergranular promyelocytes). This initial presentation doesn't give us those specific indicators, making AML a broader initial diagnostic consideration. Specific genetic testing (PML-RARA) is needed to confirm APL.
(B) Myelodysplastic Syndrome (MDS): MDS can present with cytopenias and increased blasts, but typically the blast percentage is lower than 20% in the bone marrow for a diagnosis of MDS without progression to AML.
(D) Chronic Myeloid Leukemia (CML): CML is characterized by a specific chromosomal translocation (Philadelphia chromosome) and typically presents with a significantly elevated white blood cell count and a different distribution of myeloid cells in the peripheral blood.
Next Question:

Following the initial diagnosis of AML, cytogenetic and molecular testing is performed on the bone marrow sample. The results reveal the following:

Cytogenetics: Normal Karyotype
Molecular Testing: Positive for FLT3-ITD mutation
Based on the 2022 European LeukemiaNet (ELN) risk stratification, how would you classify Mr. Johnson's AML?

A) Favorable Risk
B) Intermediate Risk
C) Adverse Risk
D) Unable to determine risk without further information
Correct Answer (B): According to the 2022 ELN risk stratification, a normal karyotype with a FLT3-ITD mutation is classified as intermediate risk.
Why the other options are incorrect:
(A) Favorable Risk: Favorable risk is typically associated with specific cytogenetic abnormalities like t(15;17) in APL, or mutations in NPM1 without FLT3-ITD.
(C) Adverse Risk: Adverse risk is associated with cytogenetic abnormalities such as inv(3), t(6;9), t(v;11), -5/del(5q), -7, -17/del(17p), complex karyotype, or mutations in TP53, ASXL1, or RUNX1. FLT3-ITD with a high allelic ratio is considered adverse risk. Without that information, and in the context of a normal karyotype, it defaults to intermediate.
(D) Unable to determine risk without further information: While additional mutations can refine risk stratification, the presence of FLT3-ITD with a normal karyotype is sufficient to classify this patient as intermediate risk according to the ELN 2022 guidelines.
Considering Mr. Johnson's age (67 years old), newly diagnosed intermediate-risk AML with FLT3-ITD mutation, and the information provided so far, which of the following is the MOST important factor to assess before making a treatment decision?

A) His creatinine clearance and liver function
B) His cardiac ejection fraction (EF)
C) His ECOG performance status
D) All of the above
Correct Answer (D): Before initiating treatment for AML, especially in older adults, it is crucial to assess organ function and performance status. These factors significantly influence treatment tolerance and outcomes.
Why each factor is important:
A) Creatinine clearance and liver function: Many chemotherapy drugs are renally or hepatically cleared. Impaired renal or hepatic function may necessitate dose adjustments to prevent toxicity.
B) Cardiac ejection fraction (EF): Some AML treatments, such as anthracyclines, can cause cardiotoxicity. Baseline cardiac function needs to be assessed, especially in older patients who may have pre-existing cardiac conditions.
C) ECOG performance status: Performance status reflects the patient's overall functional ability. Patients with poor performance status (e.g., ECOG 3 or 4) may not tolerate intensive chemotherapy and may benefit from less intensive treatment approaches.
Clinical Significance: A comprehensive assessment allows for individualized treatment planning, minimizing the risk of complications and optimizing outcomes
Let's assume after a thorough evaluation, Mr. Johnson has the following:

Creatinine Clearance: 60 mL/min
Ejection Fraction: 55%
ECOG Performance Status: 1
He is deemed a candidate for intensive chemotherapy. Which of the following induction regimens would be the MOST appropriate initial treatment option for Mr. Johnson, considering his FLT3-ITD mutation?

A) 7+3 (Cytarabine and Daunorubicin)
B) 7+3 plus Midostaurin
C) Low-Dose Cytarabine (LDAC)
D) Venetoclax plus Azacitidine
Correct Answer (B): Given Mr. Johnson's age (67), diagnosis of AML with FLT3-ITD mutation, and eligibility for intensive chemotherapy (based on creatinine clearance, EF, and ECOG performance status), the addition of midostaurin to standard 7+3 induction is the most appropriate choice. Midostaurin is a multi-kinase inhibitor with activity against FLT3, and its addition to standard chemotherapy has been shown to improve overall survival in patients with FLT3-mutated AML.
Why the other options are less appropriate:

(A) 7+3 (Cytarabine and Daunorubicin): While 7+3 is a standard induction regimen for AML, it doesn't specifically target the FLT3-ITD mutation. Therefore, it's less optimal than adding a FLT3 inhibitor.
(C) Low-Dose Cytarabine (LDAC): LDAC is generally reserved for patients who are not candidates for intensive chemotherapy due to age, comorbidities, or poor performance status. Mr. Johnson is considered fit for intensive chemotherapy.
(D) Venetoclax plus Azacitidine: This combination is also typically used for patients unfit for intensive chemotherapy.
Mr. Johnson is initiated on 7+3 plus Midostaurin. On day 5 of induction, his WBC count begins to rise rapidly. His electrolytes are within normal limits. He is started on allopurinol for prophylaxis. Which of the following is the MOST appropriate next step to manage this potential complication?

A) Increase the dose of allopurinol
B) Initiate rasburicase
C) Start hyperhydration and monitor electrolytes more frequently
D) Monitor uric acid levels only
Correct Answer (B): The rapid rise in WBC count during induction chemotherapy raises concern for tumor lysis syndrome (TLS). While allopurinol is a reasonable prophylactic measure, it may not be sufficient to prevent TLS in patients with high tumor burden and rapidly proliferating disease. Rasburicase is a recombinant urate oxidase that converts uric acid to a more soluble metabolite, allowing for rapid reduction of uric acid levels. Given the rising WBC, initiating rasburicase is the most appropriate step to prevent TLS.
Why the other options are less appropriate:
(A) Increase the dose of allopurinol: Allopurinol inhibits the formation of uric acid but does not break down pre-existing uric acid. In a situation with a rapidly rising WBC count, allopurinol alone may not be sufficient.
(C) Start hyperhydration and monitor electrolytes more frequently: Hyperhydration and electrolyte monitoring are important components of TLS management, but they are not sufficient as a standalone intervention when the WBC count is rapidly rising.
(D) Monitor uric acid levels only: Waiting for uric acid levels to rise before intervening could lead to the development of clinical TLS and associated complications such as renal failure.
Clinical Significance: Early recognition and intervention are key to preventing the complications of TLS, such as renal failure and cardiac arrhythmias.
Mr. Johnson completes his induction therapy with 7+3 plus Midostaurin. His absolute neutrophil count (ANC) drops to < 500/L, and he develops a fever of 102F. Blood cultures are drawn, and he is started on empiric broad-spectrum antibiotics.

Question 6:

Considering Mr. Johnson's febrile neutropenia, which of the following is the MOST appropriate empiric antibiotic regimen?

A) Oral ciprofloxacin
B) Intravenous vancomycin monotherapy
C) Intravenous cefepime
D) Intravenous metronidazole

Correct Answer (C): Intravenous cefepime is a broad-spectrum beta-lactam antibiotic with excellent activity against gram-negative bacteria, which are the most common cause of infection in febrile neutropenic patients.
Why the other options are less appropriate:
(A) Oral ciprofloxacin: Oral antibiotics are generally not recommended for initial empiric therapy in febrile neutropenia due to unreliable absorption and the need for rapid, high serum concentrations.
(B) Intravenous vancomycin monotherapy: Vancomycin is primarily active against gram-positive bacteria. While it may be necessary if there is suspicion of a gram-positive infection (e.g., catheter-related infection, skin/soft tissue infection), it is not appropriate as empiric monotherapy in most cases of febrile neutropenia.
(D) Intravenous metronidazole: Metronidazole is primarily active against anaerobic bacteria and is not typically used as empiric therapy for febrile neutropenia.

After 72 hours of cefepime, Mr. Johnson remains febrile. Blood cultures are still pending. Physical exam is unremarkable. Which of the following is the MOST appropriate next step in managing his persistent febrile neutropenia?

A) Continue cefepime and monitor closely
B) Add vancomycin to the cefepime regimen

C) Change to intravenous meropenem
D) Initiate antifungal therapy with intravenous fluconazole.
Correct Answer (C): After 72 hours of persistent fever despite initial empiric antibiotic therapy (cefepime), a change to a broader-spectrum antibiotic is warranted. Meropenem, a carbapenem, covers a wider range of gram-negative bacteria, including those resistant to cefepime (e.g., extended-spectrum beta-lactamase (ESBL)-producing organisms).
Why the other options are less appropriate:
(A) Continue cefepime and monitor closely: Continuing the same antibiotic regimen despite persistent fever is unlikely to be effective. Escalation of therapy is indicated in this scenario.
(B) Add vancomycin to the cefepime regimen: Vancomycin should be added if there is clinical suspicion of gram-positive infection (e.g., catheter-related infection, skin/soft tissue infection, known colonization with MRSA), or if blood cultures grow gram-positive organisms. Without these indications, adding vancomycin is not the most appropriate next step.
(D) Initiate antifungal therapy with intravenous fluconazole: Antifungal therapy is generally not indicated after 72 hours of persistent fever unless there is a high suspicion for fungal infection (e.g., prolonged neutropenia, prior history of fungal infection, presence of specific risk factors).
Mr. Johnson's blood cultures ultimately come back positive for Escherichia coli resistant to cefepime, but susceptible to meropenem. His fever resolves after switching to meropenem. He recovers his neutrophil count. A bone marrow aspirate is performed to assess response to induction. The results show <5% blasts.

Based on these results, Mr. Johnson has achieved:

A) Complete Remission (CR)
B) Complete Remission with incomplete count recovery (CRi)
C) Partial Remission (PR)
D) No Remission
Correct Answer (B): Complete Remission with incomplete count recovery (CRi) is defined as <5% blasts in the bone marrow, absence of Auer rods, and resolution of extramedullary disease, but with incomplete recovery of peripheral blood counts (ANC <1000/L or platelets <100,000/L). Mr. Johnson has <5% blasts but we do not know if his counts have fully recovered. Assuming that they have not fully recovered to normal levels (ANC >=1000 and platelets >=100,000) he has achieved CRi.
Why the other options are less appropriate:
(A) Complete Remission (CR): CR requires <5% blasts in the bone marrow and full recovery of peripheral blood counts (ANC 1000/L and platelets 100,000/L).
(C) Partial Remission (PR): PR is defined as a decrease in blast percentage to <20% but >5% in the bone marrow.
(D) No Remission: This would be indicated if the blast percentage remained >20%.
Clinical Significance: Achieving CR or CRi is the primary goal of induction therapy in AML. CRi is still a remission state, but it's associated with a slightly poorer prognosis than CR, especially if count recovery is prolonged.
Mr. Johnson achieves CRi after induction. He is FLT3-ITD positive. Which of the following is the MOST appropriate next step in his treatment plan?

A) Observation
B) Consolidation chemotherapy with high-dose cytarabine
C) Allogeneic hematopoietic stem cell transplantation (HSCT)
D) Low-dose maintenance therapy with azacitidine
Correct Answer (C): Allogeneic HSCT is generally recommended for patients with intermediate or adverse-risk AML in first remission, especially those with FLT3-ITD mutations. HSCT offers the best chance for long-term disease control and potential cure in these patients.
Why the other options are less appropriate:
(A) Observation: Observation alone is not appropriate for a patient with intermediate-risk AML in first remission.
(B) Consolidation chemotherapy with high-dose cytarabine: While consolidation chemotherapy is a standard approach for AML, it may not be sufficient for patients with FLT3-ITD mutations, who have a higher risk of relapse. HSCT offers a greater chance of long-term remission.
(D) Low-dose maintenance therapy with azacitidine: This is typically used in patients who are not candidates for intensive consolidation or HSCT.
Prior to proceeding with allogeneic HSCT, Mr. Johnson undergoes MRD (measurable residual disease) assessment via multiparameter flow cytometry. The result is positive, with an MRD level of 0.1%.

How does this MRD result influence your recommendation regarding HSCT?

A) MRD positivity is a contraindication to HSCT.
B) MRD positivity does not impact the recommendation for HSCT.
C) MRD positivity increases the urgency to proceed with HSCT.
D) MRD positivity suggests the need for more intensive consolidation chemotherapy prior to HSCT.
Correct Answer (C): MRD positivity prior to HSCT is associated with a higher risk of relapse after transplant. Therefore, it increases the urgency to proceed with HSCT, as delaying transplant may allow the disease to progress further.
Why the other options are less appropriate:
(A) MRD positivity is a contraindication to HSCT: MRD positivity is not a contraindication to HSCT. In fact, HSCT may be the best chance to eliminate MRD-positive disease.
(B) MRD positivity does not impact the recommendation for HSCT: MRD positivity is an important prognostic factor and should influence treatment decisions.
(D) MRD positivity suggests the need for more intensive consolidation chemotherapy prior to HSCT: While further consolidation could be considered, it may delay HSCT and potentially allow the disease to progress. Proceeding to HSCT is reasonable, especially if a suitable donor is available.
Clinical Significance: MRD monitoring is increasingly being used to guide treatment decisions in AML. MRD positivity is a strong predictor of relapse and may warrant more aggressive treatment strategies.
Six months post-transplant, Mr. Johnson is doing well with no active GVHD. A routine bone marrow aspirate is performed, and MRD is assessed again. The MRD is now negative (<0.01%).

What is the clinical significance of this MRD negativity post-HSCT?

A) MRD negativity post-HSCT is associated with a lower risk of relapse.
B) MRD negativity post-HSCT is associated with a higher risk of relapse.
C) MRD status post-HSCT has no impact on relapse risk.
D) MRD negativity post-HSCT indicates that immunosuppression can be safely discontinued.
Correct Answer (A): Achieving MRD negativity post-HSCT is a strong indicator of durable remission and is associated with a significantly lower risk of relapse.
Why the other options are less appropriate:
(B) MRD negativity post-HSCT is associated with a higher risk of relapse: This is incorrect. MRD negativity is a favorable prognostic factor.
(C) MRD status post-HSCT has no impact on relapse risk: This is also incorrect. MRD status is a well-established predictor of relapse after HSCT.
(D) MRD negativity post-HSCT indicates that immunosuppression can be safely discontinued: While MRD negativity is a good sign, immunosuppression should not be discontinued abruptly. Discontinuation of immunosuppression should be carefully considered and gradually tapered under close monitoring for GVHD.
Clinical Significance: MRD monitoring post-HSCT is crucial for assessing the success of the transplant and identifying patients at risk of relapse who may benefit from further intervention.
One year post-transplant, Mr. Johnson develops new-onset cough and shortness of breath. Chest X-ray reveals bilateral pulmonary infiltrates. Bronchoscopy with bronchoalveolar lavage (BAL) is performed. BAL fluid is positive for Pneumocystis jirovecii.

What is the MOST appropriate treatment for this infection?

A) Intravenous vancomycin
B) Oral oseltamivir
C) Intravenous trimethoprim-sulfamethoxazole (TMP-SMX)
D) Inhaled pentamidine
Correct Answer (C): Intravenous TMP-SMX is the preferred treatment for Pneumocystis jirovecii pneumonia (PCP), especially in patients who are severely ill or have impaired absorption.
Why the other options are less appropriate:
(A) Intravenous vancomycin: Vancomycin is an antibacterial agent and has no activity against Pneumocystis jirovecii.
(B) Oral oseltamivir: Oseltamivir is an antiviral agent used to treat influenza and has no activity against Pneumocystis jirovecii.

(D) Inhaled pentamidine: Inhaled pentamidine can be used for PCP prophylaxis, but it is less effective than TMP-SMX for treatment of active infection, particularly in severe cases.
Clinical Significance: Pneumocystis jirovecii pneumonia is a common opportunistic infection in immunocompromised patients, including those who have undergone HSCT. Prompt diagnosis and treatment are essential to prevent severe respiratory complications.
After successful treatment of his PCP, Mr. Johnson's cough and shortness of breath resolve. He is tolerating his immunosuppression well and has no evidence of GVHD. However, he mentions experiencing persistent fatigue and difficulty concentrating, which he attributes to his cancer treatment.

Which of the following interventions would be MOST appropriate to address these long-term side effects?

A) Prescribe a stimulant medication, such as methylphenidate.
B) Recommend a structured exercise program and cognitive behavioral therapy (CBT).
C) Advise him to rest more and avoid strenuous activities.
D) Discontinue his immunosuppression to improve his energy levels.
Correct Answer (B): Persistent fatigue and cognitive difficulties are common long-term side effects of cancer treatment, including HSCT. A structured exercise program and CBT have been shown to be effective in improving fatigue, cognitive function, and overall quality of life in cancer survivors.
Why the other options are less appropriate:
(A) Prescribe a stimulant medication, such as methylphenidate: While stimulants may provide short-term relief from fatigue, they do not address the underlying causes of fatigue and can have potential side effects. They are generally not recommended as a first-line treatment for cancer-related fatigue.
(C) Advise him to rest more and avoid strenuous activities: While rest is important, excessive rest can actually worsen fatigue and deconditioning. Encouraging physical activity is important.
(D) Discontinue his immunosuppression to improve his energy levels: Discontinuing immunosuppression could lead to GVHD flare-up and is not an appropriate strategy for managing fatigue.
Clinical Significance: Addressing long-term side effects is an important aspect of cancer survivorship care. Non-pharmacological interventions, such as exercise and CBT, are often the most effective and safest approach.
Two years post-transplant, Mr. Johnson develops new skin lesions that are biopsied and confirmed to be consistent with chronic graft-versus-host disease (cGvHD).

Which of the following is generally considered the FIRST-LINE systemic treatment for chronic GVHD?

A) High-dose corticosteroids
B) Ruxolitinib
C) Ibrutinib
D) Extracorporeal photopheresis (ECP)
Correct Answer (A): High-dose corticosteroids (e.g., prednisone) are the standard first-line systemic treatment for chronic GVHD.
Why the other options are less appropriate:
(B) Ruxolitinib: Ruxolitinib is a JAK1/JAK2 inhibitor that is approved for the treatment of steroid-refractory chronic GVHD.
(C) Ibrutinib: Ibrutinib is a Bruton's tyrosine kinase (BTK) inhibitor that is approved for the treatment of steroid-refractory chronic GVHD after failure of one prior line of systemic therapy.
(D) Extracorporeal photopheresis (ECP): ECP is a treatment option for chronic GVHD, but it is typically reserved for patients who have failed first-line systemic therapy.
Guideline Support: National Comprehensive Cancer Network (NCCN) guidelines recommend corticosteroids as first-line treatment for chronic GVHD.
Mr. Johnson's chronic GVHD is initially responsive to corticosteroids, but he develops steroid-related side effects, including hyperglycemia and osteoporosis. His physician decides to taper the corticosteroids and initiate a second-line agent.

Considering the information provided, which of the following second-line agents is MOST appropriate for Mr. Johnson?

A) Ruxolitinib
B) Cyclosporine
C) Methotrexate

D) Thalidomide

Correct Answer (A): Ruxolitinib is an appropriate second-line agent for chronic GVHD, particularly in patients who have failed or are intolerant to corticosteroids. It has demonstrated efficacy in treating cGVHD and is approved for this indication.

Why the other options are less appropriate:

(B) Cyclosporine: Cyclosporine is often used as part of the initial immunosuppressive regimen post-transplant and may be continued for maintenance. However, it's not typically used as a second-line agent specifically for treating cGVHD that is refractory to steroids.

(C) Methotrexate: Similar to cyclosporine, methotrexate is often used as part of the initial immunosuppressive regimen post-transplant and may be continued for maintenance. It's not typically used as a second-line agent specifically for treating cGVHD that is refractory to steroids.

(D) Thalidomide: Thalidomide has been used in the past for cGVHD, but it has significant side effects (including teratogenicity) and is generally not preferred over newer agents like ruxolitinib.

Clinical Significance: The management of chronic GVHD often requires a multi-faceted approach, including careful monitoring for side effects and consideration of second-line agents when necessary. Ruxolitinib has emerged as a valuable option in this setting.

Session ID:

Assessment Domain

Session-46:

Rationale

Session ID:

Instructional Design and Pedagogical

Session-46:

The session's instructional design is of very high quality. The case is expertly scaffolded, progressing logically and chronologically through the key stages of AML management: initial diagnosis, risk stratification, induction therapy, management of acute complications (TLS, febrile neutropenia), response assessment, consolidation strategy, and long-term survivorship challenges (opportunistic infections, cGVHD). The quality of the embedded feedback is a major strength; each rationale is detailed, clinically accurate, and explains not only why the correct answer is right but also why the distractors are incorrect. The instructions for each step are exceptionally clear. The only minor weakness is that learning objectives are not explicitly stated at the beginning of the session, though the educational goals are clearly implied by the well-designed structure.

Session ID:

Clinical Authenticity and Content Fidelity

Session-46:

The clinical content is exceptionally accurate and authentic. The narrative is highly plausible, mirroring a typical presentation and clinical course for a patient with FLT3-mutated AML. All diagnostic criteria, risk stratification (per ELN 2022 guidelines), and therapeutic decisions (e.g., 7+3 plus midostaurin for induction, allogeneic HSCT for consolidation, corticosteroids for cGVHD) are perfectly aligned with current, evidence-based standards of care. The pharmacotherapeutic recommendations are optimal and well-justified at every stage. Importantly, the case remains strictly within the domain of AML and its related complications, with no evidence of domain entanglement. The fidelity of the clinical content is excellent.

Session ID:

Modeled Clinical Reasoning

Session-46:

The simulation excellently models an expert clinical reasoning process. Each stage of the case presents a well-defined clinical problem with the necessary data for a decision (e.g., "Given these risk factors, what is the best induction?"). The multiple-choice options provide an outstanding breadth of plausible alternatives, including common distractors that effectively test nuanced understanding (e.g., distinguishing between CR and CRi). The narrative demonstrates appropriate, risk-adjusted prioritization throughout, such as assessing fitness before intensive therapy and escalating care for persistent febrile neutropenia. The clinical outcomes described for each decision are realistic, specific, and instructionally valuable, reinforcing the learning from each step.

Session-47

Session ID:
LLM

Session-47:
Google AI (Gemini 2.0 Pro)

Session ID:
Session Transcript

Session-47:
Patient Presentation:

A 67-year-old male presents to the emergency department with a 2-week history of fatigue, shortness of breath, and easy bruising. He denies any fever, chills, or recent infections. His past medical history includes hypertension and hyperlipidemia, both well-controlled with medications. He is a former smoker (quit 10 years ago) and occasionally drinks alcohol socially.

Initial Lab Results:

WBC: 25,000/L (Normal: 4,500-11,000/L)

Hemoglobin: 8.5 g/dL (Normal: 13.5-17.5 g/dL)

Platelets: 45,000/L (Normal: 150,000-400,000/L)

Peripheral blood smear: 40% blasts with Auer rods

Question 1: Based on the initial presentation and lab results, which of the following is the MOST likely diagnosis?

A) Acute Lymphoblastic Leukemia (ALL)
B) Acute Myeloid Leukemia (AML)
C) Chronic Myeloid Leukemia (CML)
D) Myelodysplastic Syndrome (MDS)
Correct! B) Acute Myeloid Leukemia (AML) is the most likely diagnosis.

Feedback:

Correct Answer Rationale: The presence of blasts (immature myeloid cells) with Auer rods on the peripheral blood smear is a hallmark of AML. The other options are less likely given the presentation:

ALL typically presents with lymphoblasts and often affects younger patients.

CML is usually characterized by a significantly elevated WBC count with a predominance of mature myeloid cells and the presence of the Philadelphia chromosome.

MDS can present with similar cytopenias, but the presence of a high percentage of blasts (>20%) is more indicative of AML.

Why other options are less likely: Understanding the key distinguishing features of each hematologic malignancy is crucial for accurate diagnosis.

Reference: This aligns with the WHO classification of AML, which requires >20% blasts in the bone marrow or peripheral blood for diagnosis (Arber DA, et al. Blood. 2016;127(20):2391-2405).

Moving on...

A bone marrow biopsy is performed to confirm the diagnosis and obtain further information for risk stratification. The results are pending, but preliminary cytogenetic analysis reveals the presence of t(15;17).

Question 2: The presence of t(15;17) is most commonly associated with which subtype of AML?

A) Acute Promyelocytic Leukemia (APL)
B) AML with myelodysplasia-related changes
C) AML with mutated NPM1
D) AML with mutated FLT3-ITD
That is correct! A) Acute Promyelocytic Leukemia (APL) is the subtype of AML most commonly associated with t(15;17).

Feedback:

Correct Answer Rationale: The translocation t(15;17) leads to the fusion of the PML and RAR genes, which is the defining characteristic of APL.

Why other options are less likely:

AML with myelodysplasia-related changes is associated with specific cytogenetic abnormalities and morphological features.

AML with mutated NPM1 and FLT3-ITD are characterized by mutations in these specific genes, not by t(15;17).

Reference: This is consistent with the WHO classification and ELN risk stratification (Dhner H, et al. Blood. 2017;129(4):424-447 and NCCN guidelines).

Since you have answered two questions correctly in a row, let's increase the difficulty slightly.

The complete bone marrow analysis confirms the diagnosis of APL with t(15;17). The patient's WBC count is 20,000/L at the time of diagnosis.
Question 3: Based on the diagnosis of APL and the WBC count, which of the following risk stratification categories is the patient MOST likely to fall into?

A) Low-risk
B) Intermediate-risk
C) High-risk
D) Very High-risk
Correct! C) High-risk is the most likely risk stratification for this patient.

Feedback:

Correct Answer Rationale: In APL, risk stratification is primarily based on the presenting WBC count. According to most guidelines (including ESMO), a WBC count >10,000/L at diagnosis defines high-risk APL.

Why other options are less likely: Low- and intermediate-risk APL are defined by WBC counts 10,000/L. There isn't a "very high-risk" category specifically defined for APL risk stratification based on WBC count alone.

Reference: This is consistent with ESMO Clinical Practice Guidelines for diagnosis, treatment and follow-up (Lo-Coco F, et al. Annals of Oncology, 2022).
Moving on...

Given the diagnosis of high-risk APL, the treatment team is considering the optimal induction therapy. The patient's creatinine clearance is estimated to be 70 mL/min. Liver function tests are within normal limits.

Question 4: Which of the following induction regimens is MOST appropriate for this patient?

A) All-trans retinoic acid (ATRA) plus arsenic trioxide (ATO)
B) All-trans retinoic acid (ATRA) plus idarubicin
C) Cytarabine plus daunorubicin ("7+3")
D) Gemtuzumab ozogamicin monotherapy
That's a good attempt, but not quite the best answer in this scenario. Let's think through this.

The correct answer is A) All-trans retinoic acid (ATRA) plus arsenic trioxide (ATO)

Feedback:

Correct Answer Rationale: For high-risk APL, the combination of ATRA and ATO is generally preferred over ATRA plus chemotherapy (like idarubicin) due to its superior efficacy and lower toxicity, especially in patients who are older or have comorbidities. This approach is supported by multiple clinical trials demonstrating improved outcomes with ATRA+ATO. While idarubicin is part of the standard ATRA + chemotherapy approach, the question asks for the most appropriate.

Why other options are less likely:

ATRA plus idarubicin is a valid option, particularly if ATO is contraindicated or unavailable. However, ATRA/ATO is favored in high-risk disease.

Cytarabine plus daunorubicin ("7+3") is a standard induction regimen for other types of AML, not APL.

Gemtuzumab ozogamicin monotherapy is not indicated for induction in APL.

Reference: This aligns with ESMO guidelines and the results of the APML4 trial (Lo-Coco F, et al. N Engl J Med. 2013;369(2):111-121).
Question 5: Considering the chosen induction regimen (ATRA + ATO), which of the following is the MOST important monitoring parameter during the initial phase of treatment?

A) Complete Blood Counts (CBC)
B) Serum Creatinine
C) Liver Function Tests (LFTs)
D) QT interval on ECG
You are correct! D) QT interval on ECG is the MOST important monitoring parameter during the initial phase of treatment with ATRA + ATO.

Feedback:

Correct Answer Rationale: Both ATRA and, more significantly, ATO can prolong the QT interval, increasing the risk of potentially fatal arrhythmias like Torsades de Pointes. Therefore, close monitoring of the QT interval via ECG is crucial, especially during the induction phase when drug levels are escalating. Electrolyte imbalances (e.g., hypokalemia, hypomagnesemia) should also be corrected, as they can exacerbate QT prolongation.

Why other options are less likely:

CBC is important for monitoring hematologic recovery and potential differentiation syndrome, but QT prolongation is a more immediate concern with this regimen.

Serum creatinine is important for overall kidney function, but not specifically related to ATRA/ATO toxicity in the acute phase.

LFTs are important for monitoring liver function, but QT prolongation is a more immediate and life-threatening concern.

Reference: This recommendation aligns with the prescribing information for both ATRA and ATO, as well as expert guidelines for APL management.

Great job, Osama! That concludes Phase 1. You correctly identified the diagnosis, risk stratification, and a critical monitoring parameter.
Alright, let's move on to Phase 2: Induction Therapy and Supportive Care Planning.

The patient is started on ATRA and ATO. On day 5 of treatment, he develops a fever of 38.5C (101.3F) and a new cough. His absolute neutrophil count (ANC) is 0.3 x 10^9/L.

Question 6: What is the MOST appropriate next step in managing this patient?

A) Initiate broad-spectrum antibiotics after obtaining blood cultures.
B) Administer granulocyte colony-stimulating factor (G-CSF).
C) Start antifungal prophylaxis with fluconazole.
D) Discontinue ATRA and ATO immediately.
Correct! A) Initiate broad-spectrum antibiotics after obtaining blood cultures. is the MOST appropriate next step.

Feedback:

Correct Answer Rationale: The patient is presenting with febrile neutropenia (fever with ANC <0.5 x 10^9/L), which is a medical emergency. Prompt initiation of broad-spectrum antibiotics is crucial to cover potential bacterial infections. Blood cultures should be obtained prior to starting antibiotics, if possible, to help identify the causative organism.

Why other options are less likely:

G-CSF may be considered in some cases of prolonged neutropenia, but antibiotics are the priority in the initial management of febrile neutropenia.

Antifungal prophylaxis may be considered later if the patient remains febrile despite broad-spectrum antibiotics, but is not the initial step.

Discontinuing ATRA and ATO may be necessary if severe differentiation syndrome develops, but is not the immediate response to febrile neutropenia.

Reference: This is consistent with IDSA guidelines for the management of febrile neutropenia (Freifeld AG, et al. Clin Infect Dis. 2011;52(4):e56-e93).
The patient's blood cultures return positive for Staphylococcus aureus. He is treated with intravenous vancomycin. After 2 weeks of induction therapy, the patient develops dyspnea, cough, and chest discomfort. Chest X-ray reveals bilateral pulmonary infiltrates. Oxygen saturation is 90% on room air.

Question 7: Which of the following is the MOST likely cause of the pulmonary infiltrates in this patient?

A) Bacterial pneumonia
B) Fungal pneumonia
C) Differentiation Syndrome (formerly known as ATRA syndrome)
D) Pulmonary embolism
Correct! C) Differentiation Syndrome (formerly known as ATRA syndrome) is the MOST likely cause of the pulmonary infiltrates in this patient.

Feedback:

Correct Answer Rationale: Differentiation Syndrome is a serious complication of APL treatment with ATRA and ATO. It is

characterized by fever, dyspnea, pulmonary infiltrates, pleural or pericardial effusions, and hypotension. It is caused by the release of cytokines from differentiating leukemic cells. Given the timeline (within 2 weeks of starting ATRA/ATO) and the clinical presentation, Differentiation Syndrome is more likely than other options.

Why other options are less likely:

Bacterial pneumonia is possible, but less likely given the recent treatment with vancomycin for S. aureus bacteremia.

Fungal pneumonia is possible, but less likely without prolonged neutropenia or lack of response to antibacterial therapy.

Pulmonary embolism is less likely without risk factors such as prolonged immobilization or a history of thromboembolic events.

Reference: This aligns with ESMO guidelines for APL management (Lo-Coco F, et al. Annals of Oncology, 2022) and expert reviews on Differentiation Syndrome.
Question 8: What is the MOST appropriate initial treatment for Differentiation Syndrome in this patient?

A) Intravenous fluids and oxygen supplementation
B) High-dose dexamethasone
C) Diuretics
D) Anticoagulation with heparin
Excellent! B) High-dose dexamethasone is the MOST appropriate initial treatment for Differentiation Syndrome in this patient.

Feedback:

Correct Answer Rationale: High-dose dexamethasone (e.g., 10 mg IV twice daily) is the cornerstone of treatment for Differentiation Syndrome. Corticosteroids help to suppress the inflammatory response and reduce the severity of the symptoms. Prompt initiation of dexamethasone can be life-saving.

Why other options are less likely:

Intravenous fluids and oxygen supplementation are important supportive measures, but do not address the underlying cause of Differentiation Syndrome.

Diuretics may be used to manage fluid overload, but are not the primary treatment.

Anticoagulation with heparin is not indicated for Differentiation Syndrome and may be harmful.

Reference: This is consistent with ESMO guidelines and expert recommendations for managing Differentiation Syndrome.
Question 9: Following achievement of CR after induction, what is the MOST appropriate next step in the treatment of this patient with high-risk APL?

A) Observation
B) Consolidation therapy with cytarabine and daunorubicin
C) Autologous stem cell transplant
D) Consolidation therapy with ATRA and ATO
You are correct! D) Consolidation therapy with ATRA and ATO is the MOST appropriate next step in the treatment of this patient with high-risk APL.

Feedback:

Correct Answer Rationale: For patients with high-risk APL who achieve CR after induction with ATRA and ATO, consolidation therapy with ATRA and ATO is the preferred approach. Studies have shown that this approach is highly effective in maintaining remission and improving overall survival.

Why other options are less likely:

Observation alone is not appropriate for high-risk APL, as the risk of relapse is significant without further treatment.

Consolidation therapy with cytarabine and daunorubicin is not the standard of care for APL.

Autologous stem cell transplant is not typically used in the frontline treatment of APL. Allogeneic stem cell transplant may be considered in relapsed cases.

Reference: This aligns with ESMO guidelines and the results of clinical trials evaluating consolidation strategies in APL.
Question 10: During consolidation therapy with ATRA and ATO, what is the MOST important monitoring parameter to assess for potential long-term complications?

A) Renal function
B) Liver function
C) Neuropathy
D) Bone marrow blasts
That is correct! B) Liver function is the MOST important monitoring parameter to assess for potential long-term complications during consolidation therapy with ATRA and ATO.

Feedback:

Correct Answer Rationale: ATO is known to cause hepatotoxicity, and long-term exposure can lead to liver damage. Therefore, regular monitoring of liver function tests (LFTs) is crucial during consolidation therapy to detect any signs of hepatotoxicity and allow for timely intervention.

Why other options are less likely:

Renal function is less directly affected by ATRA and ATO, although monitoring is still important for overall patient safety.

Neuropathy can occur with ATO, but is less common than hepatotoxicity.

Bone marrow blasts are monitored to assess for relapse, but not specifically as a long-term complication of ATO.

Reference: This recommendation aligns with the prescribing information for ATO and expert guidelines for APL management.
Okay, let's proceed to Phase 3: Post-Remission Therapy and Long-Term Management.

The patient completes consolidation therapy with ATRA and ATO and remains in complete remission. He is now in the maintenance phase.

Question 11: Which of the following is the MOST appropriate maintenance strategy for this patient?

A) Observation
B) Low-dose chemotherapy
C) Continued ATRA monotherapy
D) No maintenance therapy is needed
That's a good attempt, but not quite the best answer in this scenario. Let's think through this.

The correct answer is D) No maintenance therapy is needed

Feedback:

Correct Answer Rationale: Current evidence suggests that after successful induction and consolidation with ATRA and ATO in APL, maintenance therapy does not provide additional benefit and is not routinely recommended. Studies have shown excellent long-term

outcomes with ATRA/ATO alone, making maintenance unnecessary.

Why other options are less likely:

Observation alone might seem reasonable, but it doesn't actively address any potential residual disease. However, in the context of successful ATRA/ATO consolidation, observation is the correct approach.

Low-dose chemotherapy is not a standard maintenance strategy for APL.

Continued ATRA monotherapy has not been shown to improve outcomes compared to observation after ATRA/ATO consolidation and can have side effects.

Reference: This aligns with current ESMO guidelines and the results of clinical trials.
Question 12: Six months after completing consolidation therapy, the patient presents with fatigue and a slightly decreased platelet count (130,000/L). A bone marrow aspirate is performed, which shows 1% blasts. Molecular testing for PML-RAR fusion transcript is negative by PCR. What is the MOST appropriate next step?

A) Re-initiate ATRA and ATO
B) Start low-dose cytarabine
C) Monitor the patient closely with serial bone marrow aspirates and molecular testing
D) Proceed with allogeneic stem cell transplant
You are correct! C) Monitor the patient closely with serial bone marrow aspirates and molecular testing is the MOST appropriate next step.

Feedback:

Correct Answer Rationale: In this scenario, the patient has mild cytopenia and a very low blast percentage (1%), but the PML-RAR transcript is negative by PCR. This could represent a very early relapse, but it could also be a transient abnormality. Close monitoring with serial bone marrow aspirates and molecular testing is warranted to determine if the blast percentage is increasing or if the PML-RAR transcript becomes detectable.

Why other options are less likely:

Re-initiating ATRA and ATO or starting low-dose cytarabine would be premature without clear evidence of relapse.

Proceeding with allogeneic stem cell transplant is not indicated at this stage, as the patient does not have definitive evidence of relapse.

Reference: This approach aligns with expert recommendations for managing patients with suspected early relapse of APL.
Question 13: Three months later, the patient's blast percentage has increased to 5% in the bone marrow, and PML-RAR is now detectable by PCR at a low level. The patient is diagnosed with relapsed APL. What is the MOST appropriate treatment option for this patient, assuming he is otherwise fit and has no significant comorbidities?

A) Arsenic trioxide monotherapy
B) Chemotherapy with cytarabine and daunorubicin
C) Allogeneic stem cell transplant
D) Clinical trial evaluating novel therapies
That's a reasonable consideration, but there's a more definitive option here. Let's think through the relapse setting.

The correct answer is C) Allogeneic stem cell transplant

Feedback:

Correct Answer Rationale: Allogeneic stem cell transplant (alloSCT) is generally considered the preferred treatment option for

relapsed APL in patients who are fit enough to undergo the procedure and have a suitable donor. AlloSCT offers the potential for long-term disease control through graft-versus-leukemia effect.

Why other options are less likely:

Arsenic trioxide monotherapy can be effective in some cases of relapsed APL, but is less likely to provide long-term disease control compared to alloSCT.

Chemotherapy with cytarabine and daunorubicin can induce a second remission, but is not curative and is often used as a bridge to transplant.

Clinical trials are always a good option to consider, but alloSCT is the standard of care for eligible patients with relapsed APL.

Reference: This recommendation aligns with ESMO guidelines and expert consensus statements on the management of relapsed APL.
Question 14: The patient proceeds to allogeneic stem cell transplant. One year post-transplant, he is doing well and remains in remission. He is currently taking tacrolimus for graft-versus-host disease (GVHD) prophylaxis. He develops a fungal infection requiring treatment with voriconazole. What is the MOST important drug interaction to consider in this patient?

A) Voriconazole increases tacrolimus levels
B) Voriconazole decreases tacrolimus levels
C) Tacrolimus increases voriconazole levels
D) Tacrolimus decreases voriconazole levels
You are correct! A) Voriconazole increases tacrolimus levels is the MOST important drug interaction to consider in this patient.

Feedback:

Correct Answer Rationale: Both tacrolimus and voriconazole are metabolized by cytochrome P450 3A4 (CYP3A4). Voriconazole is a strong inhibitor of CYP3A4, which can significantly increase tacrolimus levels, leading to potential tacrolimus toxicity (e.g., nephrotoxicity, neurotoxicity). Therefore, tacrolimus levels should be closely monitored and the dose adjusted accordingly when voriconazole is initiated.

Why other options are less likely:

Voriconazole levels can also be affected by tacrolimus, but the primary concern is the increase in tacrolimus levels due to CYP3A4 inhibition.

Reference: Drug interaction databases (e.g., Lexicomp, Micromedex) and the prescribing information for both drugs highlight this interaction.

Final Question!

Question 15: Five years post-transplant, the patient remains in remission and is off all immunosuppression. He reports experiencing chronic fatigue. What is the MOST important long-term follow-up recommendation for this patient?

A) Annual bone marrow aspirate
B) Routine blood counts every 3 months
C) Monitoring for late effects of transplant, including secondary malignancies and GVHD
D) No further follow-up is necessary
You are absolutely correct! C) Monitoring for late effects of transplant, including secondary malignancies and GVHD is the MOST important long-term follow-up recommendation for this patient.

Feedback:

Correct Answer Rationale: Even years after allogeneic stem cell transplant, patients remain at risk for late effects, including secondary malignancies (e.g., myelodysplastic syndrome, leukemia, solid tumors) and chronic GVHD. Chronic fatigue can be a symptom of both of these complications. Therefore, ongoing monitoring for these late effects is crucial.

Why other options are less likely:

Annual bone marrow aspirate is not routinely recommended in the absence of any clinical or laboratory evidence of relapse.

Routine blood counts every 3 months may be reasonable, but are not sufficient to detect all potential late effects of transplant.

No further follow-up is not appropriate, as patients remain at risk for late effects even years after transplant.

Reference: This recommendation aligns with guidelines for long-term follow-up after allogeneic stem cell transplant.

Session ID:
Assessment Domain

Session-47:
Rationale

Session ID:
Instructional Design and Pedagogical

Session-47:
The instructional design of this session is of exceptionally high quality. The case is expertly scaffolded, guiding the learner through the entire clinical course of Acute Promyelocytic Leukemia (APL) in a logical sequence: initial diagnosis, risk stratification, induction therapy, management of life-threatening complications (febrile neutropenia, differentiation syndrome), consolidation, surveillance, relapse, and long-term survivorship. The embedded feedback is a major strength; rationales are clear, consistently cite major guidelines or landmark trials (e.g., WHO, ESMO, IDSA, Lo-Coco et al.), and explain why incorrect options are suboptimal. Instructions are intuitive and effectively frame each new clinical challenge. The only minor area for improvement is that learning objectives are not explicitly stated at the outset, though they are clearly implied by the session's comprehensive structure.

Session ID:
Clinical Authenticity and Content Fidelity

Session-47:
The session demonstrates flawless clinical authenticity and content fidelity. The narrative is indistinguishable from a real-world patient case, with a plausible presentation and a realistic evolution of the disease and its complications. All therapeutic recommendationsfrom the selection of ATRA plus ATO for high-risk induction to the use of high-dose dexamethasone for differentiation syndrome and allo-SCT for relapseare perfectly aligned with current, evidence-based guidelines (e.g., NCCN, ESMO). The pharmacotherapeutic choices are optimal and well-justified. Critically, the session exhibits perfect domain specificity; all diagnostics, treatments, and concepts are appropriate for APL, with no entanglement or misapplication of standards from other hematologic malignancies. The content is accurate, safe, and educationally sound.

Session ID:
Modeled Clinical Reasoning

Session-47:
The simulation provides an expert model of clinical reasoning. Each stage of the case frames a distinct and relevant clinical problem, from diagnosis to managing drug interactions post-transplant. The multiple-choice options offer an excellent breadth of plausible choices, including key distractors that test for nuanced understanding (e.g., contrasting ATRA/ATO with older chemotherapy regimens). The decision-making process modeled throughout the session demonstrates sound, risk-adjusted prioritization, such as the immediate management of febrile neutropenia and the correct identification of QT prolongation as a critical monitoring parameter.

The clinical outcomes are realistic and highly instructive, effectively reinforcing the learning objectives of each question.

Session-48

Session ID:
LLM

Session-48:
Google AI (Gemini 2.0 Pro)

Session ID:
Session Transcript

Session-48:
Phase 1: Initial Presentation and Diagnostic Workup
Patient Presentation:
You are consulted on a 67-year-old male, Mr. John Smith, who presents to the emergency department with complaints of fatigue, easy bruising, and a recent fever. He reports feeling increasingly tired over the past month and noticed several bruises on his arms and legs despite no significant trauma. He also mentions having a persistent low-grade fever for the past week.
Relevant Medical History:
Hypertension, well-controlled with lisinopril 20mg daily.
Type 2 Diabetes, managed with metformin 500mg twice daily.
No prior history of malignancy or significant hematologic disorders.
Social History:
Retired accountant.
Lives at home with his wife.
Non-smoker, occasional alcohol use (1-2 drinks per week).
Initial Lab Results:
WBC: 2.5 x 10^9/L (Normal: 4.0-11.0 x 10^9/L)
Hemoglobin: 8.0 g/dL (Normal: 13.5-17.5 g/dL)
Platelets: 30 x 10^9/L (Normal: 150-400 x 10^9/L)
Peripheral blood smear: Reveals >20% blasts
Based on the initial presentation and lab results, which of the following is the MOST likely diagnosis?
A) Myelodysplastic Syndrome (MDS)
B) Acute Lymphoblastic Leukemia (ALL)
C) Acute Myeloid Leukemia (AML)
D) Chronic Myeloid Leukemia (CML)

c
That's correct! C) Acute Myeloid Leukemia (AML) is the most likely diagnosis based on the presence of >20% blasts in the peripheral blood, along with pancytopenia (low WBC, Hemoglobin, and Platelets) and the patient's acute presentation.
Here's why the other options are less likely:
A) Myelodysplastic Syndrome (MDS): While MDS can present with similar cytopenias, the presence of >20% blasts is more indicative of AML. MDS is characterized by dysplastic (abnormal) cells, but often with a lower blast percentage.
B) Acute Lymphoblastic Leukemia (ALL): ALL is more common in children, although it can occur in adults. The blasts in ALL are lymphoblasts, not myeloblasts, and the clinical presentation might differ. The peripheral blood smear would be expected to show lymphoblasts rather than myeloblasts.
D) Chronic Myeloid Leukemia (CML): CML is a chronic myeloproliferative neoplasm characterized by the Philadelphia chromosome (BCR-ABL1 fusion gene). It typically presents with elevated WBC counts and a more gradual onset of symptoms
Next Step: A bone marrow biopsy is essential to confirm the diagnosis of AML and further characterize the disease.

Question 2:

A bone marrow biopsy is performed and confirms the diagnosis of AML. Cytogenetic analysis reveals the presence of t(15;17). Which

AML subtype is MOST likely in this patient?

A) AML with inv(16)(p13.1q22)
B) Acute Promyelocytic Leukemia (APL)
C) AML with mutated NPM1
D) AML with t(8;21)(q22;q22.1)

c
I see why you chose C. NPM1 mutations are indeed common in AML. However, in this case, the presence of t(15;17) is the key piece of information.
The correct answer is B) Acute Promyelocytic Leukemia (APL).
Here's why:
t(15;17) is the characteristic translocation associated with APL, resulting in the fusion of the PML and RAR genes. This fusion protein blocks myeloid differentiation at the promyelocyte stage.
Let's break down the other options:
A) AML with inv(16)(p13.1q22): This is associated with AML, specifically the M4Eo subtype, often with favorable prognosis.
C) AML with mutated NPM1: NPM1 mutations are common in AML, particularly in AML with normal cytogenetics, and can have variable prognostic implications depending on other co-mutations.
D) AML with t(8;21)(q22;q22.1): This translocation is associated with AML, specifically the M2 subtype, and generally carries an intermediate prognosis.
Key takeaway: Recognizing specific cytogenetic abnormalities is crucial for accurate AML subtyping and risk stratification. APL, defined by t(15;17), is a distinct entity with a unique treatment approach.

Given the diagnosis of APL, what is the MOST appropriate initial treatment approach?
A) Standard "7+3" induction chemotherapy (cytarabine and daunorubicin)
B) High-dose cytarabine-based chemotherapy
C) All-trans retinoic acid (ATRA) and arsenic trioxide (ATO)
D) Hypomethylating agents (e.g., azacitidine)
hint
Okay, let's consider the unique characteristics of APL. Think about what makes APL different from other AML subtypes. Specifically, consider the PML-RAR fusion protein and how it disrupts normal cell differentiation. What treatment strategy might target this specific abnormality and overcome this block in differentiation, leading to remission? Remember that APL is considered a highly curable form of AML.
c
Correct again! C) All-trans retinoic acid (ATRA) and arsenic trioxide (ATO) is the most appropriate initial treatment approach for APL.
Here's the rationale:
ATRA targets the PML-RAR fusion protein, causing differentiation of the leukemic promyelocytes into mature granulocytes.
ATO also targets the PML-RAR fusion protein, leading to its degradation and apoptosis of the leukemic cells.
This combination is highly effective in APL and has significantly improved outcomes, often allowing for chemotherapy-free treatment in low-to-intermediate risk patients.
Why the other options are less ideal:
A) Standard "7+3" induction chemotherapy (cytarabine and daunorubicin): While historically used in APL, it's now considered less desirable upfront compared to ATRA/ATO, especially in low-to-intermediate risk cases, due to increased toxicity.
B) High-dose cytarabine-based chemotherapy: Not the standard of care for APL induction.
D) Hypomethylating agents (e.g., azacitidine): These are typically used in MDS or AML with specific mutations, not the primary treatment for APL.
Important Note: Risk stratification is crucial in APL to determine the intensity of treatment. White blood cell count and platelet count at diagnosis are key factors in determining risk.

Mr. Smith's initial WBC count was 2.5 x 10^9/L, and his platelet count was 30 x 10^9/L. Based on these values, which risk stratification category would Mr. Smith fall into, according to the commonly used Sanz score (adapted by the ELN)?
A) Low-Risk
B) Intermediate-Risk
C) High-Risk

D) Very High-Risk

c

You're on the right track! Let's clarify the Sanz score (and its variations used in APL risk stratification).

The correct answer is B) Intermediate-Risk.

Here's a breakdown of the Sanz score as it's commonly applied to APL (and adapted by the ELN guidelines):

Low-Risk: WBC 3 x 10^9/L and platelets 30 x 10^9/L

Intermediate-Risk: WBC 3 x 10^9/L and platelets > 30 x 10^9/L OR WBC > 3 x 10^9/L and platelets 30 x 10^9/L

High-Risk: WBC > 3 x 10^9/L

Since Mr. Smith's WBC is 2.5 x 10^9/L (3) and platelets are 30 x 10^9/L (30) one might think it is low risk. However, some modifications of the Sanz score define it as follows:

Low-Risk: WBC 1 x 10^9/L

Intermediate-Risk: WBC > 1 to 10 x 10^9/L

High-Risk: WBC > 10 x 10^9/L

Since there is variability in the exact cutoffs for each definition, it is most prudent and safe to classify the patient as intermediate risk given the WBC of 2.5 and the platelets of 30.

Therefore, Mr. Smith is considered intermediate-risk APL based on his WBC count and platelet count at diagnosis.

Why is this important?

Risk stratification guides treatment intensity. While ATRA/ATO is the backbone of therapy for all risk groups, high-risk patients may benefit from the addition of chemotherapy (e.g., idarubicin) to ATRA/ATO induction to rapidly reduce the leukemic burden. Some institutions will give low dose chemotherapy to intermediate risk patients as well

Considering Mr. Smith's intermediate-risk APL, what supportive care measure is MOST critical to initiate concurrently with ATRA/ATO therapy?

A) Prophylactic antifungal medication (e.g., fluconazole)

B) Prophylactic antiviral medication (e.g., acyclovir)

C) Monitoring for and management of differentiation syndrome

D) Initiation of granulocyte-colony stimulating factor (G-CSF)

c

Excellent! You are absolutely correct. C) Monitoring for and management of differentiation syndrome is the most critical supportive care measure to initiate concurrently with ATRA/ATO therapy in APL.

Here's why:

Differentiation Syndrome (formerly known as retinoic acid syndrome): This is a potentially life-threatening complication that can occur during ATRA and/or ATO treatment in APL. It is characterized by fever, dyspnea, pulmonary infiltrates, pleural or pericardial effusions, hypotension, and acute renal failure. It's caused by the release of cytokines from differentiating promyelocytes. Early recognition and prompt treatment are crucial.

Let's look at why the other options are less critical as the most important initial supportive measure:

A) Prophylactic antifungal medication (e.g., fluconazole): While antifungal prophylaxis is important in AML patients undergoing chemotherapy due to neutropenia, it is not the most immediate concern with ATRA/ATO therapy, especially in the initial phase.

B) Prophylactic antiviral medication (e.g., acyclovir): Similar to antifungal prophylaxis, antiviral prophylaxis is generally considered in the context of significant immunosuppression, which is not the primary concern with ATRA/ATO alone.

D) Initiation of granulocyte-colony stimulating factor (G-CSF): G-CSF is generally not recommended during ATRA/ATO induction unless there is a specific indication (e.g., severe infection) because it may theoretically worsen differentiation syndrome.

Management of Differentiation Syndrome:

Early recognition is key! Monitor patients closely for signs and symptoms.

Hold ATRA/ATO temporarily.

Administer high-dose corticosteroids (e.g., dexamethasone 10mg IV twice daily).

Provide supportive care: Oxygen, diuretics if needed.

Three days after starting ATRA/ATO, Mr. Smith develops a fever of 38.5C, dyspnea, and new bilateral pulmonary infiltrates on chest X-ray. His oxygen saturation has dropped to 90% on room air. Which of the following is the MOST appropriate immediate action?

A) Obtain blood cultures and start broad-spectrum antibiotics.

B) Administer intravenous fluids for possible dehydration.

C) Initiate dexamethasone 10mg IV twice daily and temporarily hold ATRA/ATO.
D) Order a CT scan of the chest to rule out pulmonary embolism.
a
While obtaining blood cultures and starting broad-spectrum antibiotics is essential, it's not the most appropriate immediate action in this scenario.
The correct answer is C) Initiate dexamethasone 10mg IV twice daily and temporarily hold ATRA/ATO.
Here's why:
The clinical presentation (fever, dyspnea, pulmonary infiltrates, and hypoxia) strongly suggests Differentiation Syndrome, which is a life-threatening complication of ATRA/ATO treatment, as you already know.
Prompt recognition and treatment with corticosteroids (dexamethasone) and temporarily holding ATRA/ATO are crucial for managing differentiation syndrome.
Here's a breakdown of why the other options are less appropriate as the initial step:
A) Obtain blood cultures and start broad-spectrum antibiotics: While the patient has a fever, and infection needs to be ruled out, the clinical presentation is most concerning for Differentiation Syndrome and requires immediate intervention.
B) Administer intravenous fluids for possible dehydration: While hydration is important, it's not the primary life-threatening concern at this moment.
D) Order a CT scan of the chest to rule out pulmonary embolism: Although a pulmonary embolism is a possible diagnosis, the clinical presentation is most likely due to Differentiation Syndrome, which must be addressed immediately.
You are correct that one must always rule out infection. Broad-spectrum antibiotics should be started soon after dexamethasone administration if an infection is suspected.
Following the initiation of dexamethasone and holding ATRA/ATO, Mr. Smith's respiratory status improves. However, his WBC count begins to rise rapidly (from 2.5 x 10^9/L to 15 x 10^9/L over 5 days). Which of the following is the MOST appropriate next step in managing his leukemia?
A) Continue dexamethasone and restart ATRA/ATO.
B) Add a dose of daunorubicin to the treatment regimen.
C) Administer hydroxyurea to control the WBC count.
D) Initiate prophylactic intrathecal chemotherapy.
c
Yes, C) Administer hydroxyurea to control the WBC count is the most appropriate next step in this scenario.
Here's why:
Leukocytosis in APL: A rising WBC count during ATRA/ATO treatment can increase the risk of differentiation syndrome and other complications. Rapidly controlling the WBC count is crucial.
Hydroxyurea: This is a cytoreductive agent that can quickly lower the WBC count by inhibiting DNA synthesis. It's often used as a temporary measure to control leukocytosis in AML and other hematologic malignancies.
Let's look at why the other options are less ideal at this specific moment:
A) Continue dexamethasone and restart ATRA/ATO: While the goal is to continue ATRA/ATO, restarting it without addressing the rising WBC count could worsen the differentiation syndrome or lead to other complications.
B) Add a dose of daunorubicin to the treatment regimen: Adding chemotherapy might be considered later, especially in high-risk patients or those with persistent disease, but the immediate priority is to control the WBC count.
D) Initiate prophylactic intrathecal chemotherapy: Intrathecal chemotherapy is not routinely indicated in APL unless there is evidence of central nervous system involvement.
Important Considerations:
Hydroxyurea is a temporary measure. It doesn't treat the underlying leukemia but helps control the WBC count while ATRA/ATO exert their effects.
Close monitoring of the WBC count is essential to adjust the dose of hydroxyurea as needed.

After managing the differentiation syndrome and controlling the WBC count with hydroxyurea, Mr. Smith achieves complete remission (CR) following ATRA/ATO induction. His bone marrow examination shows <5% blasts and normalization of blood counts. What is the MOST important next step in his treatment plan?
A) Proceed directly to allogeneic hematopoietic stem cell transplantation (HSCT).
B) Initiate maintenance therapy with single-agent ATRA.
C) Administer consolidation therapy.
D) Observe without further treatment.
hint

Remember that APL has a high cure rate with appropriate treatment. Consider the role of post-remission therapy in reducing the risk of relapse and consolidating the initial response. While transplant is an option for some high-risk patients, it's not the standard approach for all patients in first remission. What is the typical next step after achieving CR in APL to further reduce the risk of relapse?

c

Correct! C) Administer consolidation therapy is the most important next step in his treatment plan.

Here's why:

Consolidation Therapy: The goal of consolidation therapy is to further eradicate any remaining leukemic cells and reduce the risk of relapse. Even though Mr. Smith achieved complete remission, there may still be minimal residual disease (MRD) present.

APL Consolidation: The standard approach typically involves multiple cycles of ATRA and ATO, sometimes with the addition of chemotherapy, depending on the patient's risk factors.

Let's discuss the other options:

A) Proceed directly to allogeneic hematopoietic stem cell transplantation (HSCT): HSCT is generally reserved for patients with relapsed APL or those with very high-risk features. It's not the standard approach for patients in first remission after achieving CR with ATRA/ATO.

B) Initiate maintenance therapy with single-agent ATRA: Maintenance therapy is not a standard component of APL treatment after consolidation.

D) Observe without further treatment: This is not appropriate. Consolidation therapy is essential to prevent relapse

Which of the following consolidation regimens is MOST commonly used for intermediate-risk APL patients like Mr. Smith?

A) High-dose cytarabine alone

B) ATRA and ATO with or without chemotherapy

C) Autologous stem cell transplant

D) Clofarabine and cyclophosphamide

b

That is correct! B) ATRA and ATO with or without chemotherapy is the most commonly used consolidation regimen for intermediate-risk APL patients like Mr. Smith.

Here's why:

ATRA/ATO Consolidation: This approach builds upon the success of the induction regimen and further eliminates any remaining leukemic cells. The addition of chemotherapy (typically anthracycline-based) may be considered based on risk factors and institutional protocols. The exact regimen and number of cycles can vary.

The other options are less commonly used in this setting:

A) High-dose cytarabine alone: This is more commonly used in other AML subtypes, not APL.

C) Autologous stem cell transplant: Not a standard approach for APL consolidation. Allogeneic transplant is reserved for relapsed or refractory cases.

D) Clofarabine and cyclophosphamide: These agents are not typically used in APL consolidation.

Mr. Smith receives consolidation therapy with ATRA and ATO without chemotherapy and tolerates it well. After completing consolidation, his bone marrow examination shows continued complete remission with no evidence of MRD. What is the MOST appropriate long-term monitoring strategy for Mr. Smith?

A) Routine bone marrow biopsies every 3 months for the first year.

B) Regular blood counts and molecular monitoring for PML-RAR transcripts.

C) Observation without further monitoring.

D) Maintenance therapy with low-dose chemotherapy.

hint

Think about what methods are available to detect minimal residual disease (MRD) in APL, even when the bone marrow appears normal under a microscope. What specific marker is unique to APL that can be tracked to identify early signs of relapse? Considering the effectiveness of APL treatment, frequent invasive procedures like bone marrow biopsies are not always necessary. What non-invasive methods are available?

b

Excellent! B) Regular blood counts and molecular monitoring for PML-RAR transcripts is the most appropriate long-term monitoring strategy for Mr. Smith.

Here's why:

Molecular Monitoring: Detecting the PML-RAR fusion transcript using quantitative reverse transcriptase polymerase chain reaction

(qRT-PCR) is a highly sensitive method for detecting MRD in APL. This allows for early detection of relapse, even before it's evident on a bone marrow examination.

Regular Blood Counts: Monitoring blood counts can also provide clues to potential relapse, although molecular monitoring is more sensitive.

Let's look at why the other options are less appropriate:

A) Routine bone marrow biopsies every 3 months for the first year: Bone marrow biopsies are invasive and not necessary as a routine monitoring tool if molecular monitoring is negative.

C) Observation without further monitoring: This is not recommended, as early detection of relapse is crucial for successful salvage therapy.

D) Maintenance therapy with low-dose chemotherapy: Maintenance therapy is not a standard component of APL treatment after consolidation.

Key takeaway:

Molecular monitoring for PML-RAR is the standard of care for long-term follow-up in APL.

Early detection of molecular relapse allows for prompt intervention and improved outcomes.

What is the MOST appropriate initial treatment approach for Mr. Smith's relapsed APL?

A) Repeat induction with ATRA and ATO.

B) Allogeneic hematopoietic stem cell transplantation (HSCT).

C) Chemotherapy-based salvage therapy followed by consideration of HSCT.

D) Enrollment in a clinical trial investigating novel targeted therapies.

b

While allogeneic HSCT is a potential option, it is not always the most appropriate initial treatment approach. Often, patients need to be brought back into remission before proceeding to transplant.

The correct answer is C) Chemotherapy-based salvage therapy followed by consideration of HSCT.

Here's why:

Salvage Therapy: The goal of salvage therapy is to achieve a second complete remission (CR2). This is typically achieved with chemotherapy-based regimens.

HSCT Consideration: After achieving CR2, allogeneic HSCT is the preferred consolidation strategy for relapsed APL, as it offers the best chance of long-term disease control. But first, the patient needs to be in remission!

Let's break down the other options:

A) Repeat induction with ATRA and ATO: While ATRA/ATO can be effective in newly diagnosed APL, its efficacy in relapsed disease is often lower, especially if the patient received it as part of their initial treatment. Resistance to ATRA/ATO can develop.

B) Allogeneic hematopoietic stem cell transplantation (HSCT): HSCT is a good consolidation option after achieving a second remission.

D) Enrollment in a clinical trial investigating novel targeted therapies: This is a reasonable consideration, but it depends on the availability of suitable clinical trials and the patient's eligibility. Salvage therapy is usually required first to control the disease.

Which of the following chemotherapy regimens is MOST commonly used as salvage therapy for relapsed APL?

A) Idarubicin and cytarabine

B) Gemtuzumab ozogamicin monotherapy

C) FLAG-Ida (fludarabine, cytarabine, idarubicin, and G-CSF)

D) Decitabine and venetoclax

c

Correct! C) FLAG-Ida (fludarabine, cytarabine, idarubicin, and G-CSF) is a commonly used and effective salvage regimen for relapsed APL.

Here's why:

FLAG-Ida: This is a more intensive chemotherapy regimen that can overcome resistance to initial therapies and achieve a second remission in a significant proportion of patients with relapsed APL.

Let's briefly discuss the other options:

A) Idarubicin and cytarabine: While this combination is used in AML, it might not be intensive enough for relapsed APL, especially if the patient received similar agents during initial therapy.

B) Gemtuzumab ozogamicin monotherapy: This is an anti-CD33 antibody-drug conjugate used in some AML subtypes, but it's not typically used as monotherapy for relapsed APL.

D) Decitabine and venetoclax: This combination is used in older adults with AML who are not candidates for intensive chemotherapy. It is not an effective salvage regimen for relapsed APL.

Mr. Smith achieves a second complete remission (CR2) with FLAG-Ida salvage therapy. A suitable matched related donor is

identified. What is the MOST appropriate next step?
A) Proceed directly to allogeneic HSCT.
B) Administer another cycle of FLAG-Ida as consolidation.
C) Initiate maintenance therapy with azacitidine.
D) Monitor MRD with RT-PCR every 3 months.
b
While further consolidation is a reasonable consideration to deepen remission, it is not the most appropriate next step in this scenario.
The correct answer is A) Proceed directly to allogeneic HSCT.
Here's why:
HSCT for Relapsed APL: Allogeneic HSCT is the preferred consolidation strategy for patients with relapsed APL who achieve a second remission. It offers the best chance of long-term disease control by providing a new, healthy immune system that can eradicate any remaining leukemic cells. The graft-versus-leukemia effect is particularly important in this setting.
Let's look at the other options:
B) Administer another cycle of FLAG-Ida as consolidation: While further consolidation might deepen the remission, it does not address the underlying risk of relapse as effectively as HSCT.
C) Initiate maintenance therapy with azacitidine: Azacitidine is not a standard maintenance therapy in APL.
D) Monitor MRD with RT-PCR every 3 months: While MRD monitoring is important, it is not a substitute for HSCT in this setting.
Mr. Smith undergoes allogeneic HSCT. One year post-transplant, he develops chronic graft-versus-host disease (cGVHD) affecting his skin and liver. Which of the following is the MOST appropriate initial treatment for his cGVHD?
A) High-dose corticosteroids (e.g., prednisone 1mg/kg/day)
B) Ruxolitinib
C) Extracorporeal photopheresis (ECP)
D) Imatinib
b
While Ruxolitinib can be used, it's not typically the initial treatment.
The correct answer is A) High-dose corticosteroids (e.g., prednisone 1mg/kg/day)
Here's why:
Corticosteroids: High-dose corticosteroids are the first-line treatment for cGVHD. They have broad immunosuppressive effects and can effectively control the symptoms in many patients.
Here's a breakdown of the other options:
B) Ruxolitinib: Ruxolitinib is a JAK1/JAK2 inhibitor approved for the treatment of steroid-refractory or steroid-dependent chronic GVHD after failure of other treatments. While it's a valuable option, it's not typically used as initial therapy.
C) Extracorporeal photopheresis (ECP): ECP is a treatment option for cGVHD that is refractory to corticosteroids and other immunosuppressive agents.
D) Imatinib: Imatinib is a tyrosine kinase inhibitor used in CML and other malignancies. It has no role in the treatment of cGVHD.

Despite treatment with corticosteroids, Mr. Smith's cGVHD progresses, and he develops new organ involvement. Which of the following is the MOST appropriate next step in managing his cGVHD?
A) Increase the dose of corticosteroids.
B) Add a second-line agent such as ruxolitinib.
C) Initiate total body irradiation (TBI).
D) Discontinue all immunosuppression to allow for graft-versus-leukemia effect.
b

Excellent! B) Add a second-line agent such as ruxolitinib is the most appropriate next step in managing his cGVHD.

Here's why:

Steroid-Refractory cGVHD: When cGVHD progresses despite corticosteroid therapy, it is considered steroid-refractory. In this situation, adding a second-line agent is necessary. Ruxolitinib is an approved and effective option.

Let's look at why the other options are less appropriate:

A) Increase the dose of corticosteroids: Increasing the dose of corticosteroids may provide some benefit, but it also increases the risk

of side effects. Adding a second-line agent is generally preferred.

C) Initiate total body irradiation (TBI): TBI is a very toxic treatment and is rarely used for cGVHD.

D) Discontinue all immunosuppression to allow for graft-versus-leukemia effect: This is a dangerous approach that could lead to severe GVHD flare and organ damage. The graft-versus-leukemia effect is not reliable enough to justify this risk.

Session ID:
Assessment Domain

Session-48:
Rationale

Session ID:
Instructional Design and Pedagogical

Session-48:
The session's instructional design is very strong. The case is well-scaffolded, progressing logically through a complex, long-term clinical scenario from initial diagnosis of Acute Promyelocytic Leukemia (APL) to treatment, management of complications (differentiation syndrome, leukocytosis), relapse, and post-transplant care. The quality of the embedded feedback is excellent; rationales are detailed, clinically accurate, and effectively explain why incorrect options are less appropriate. Instructions are clear and unambiguous. The score for scaffolding was slightly reduced due to an abrupt narrative jump from post-consolidation monitoring directly to a question about managing relapse, without explicitly stating that a relapse had occurred. The lack of explicitly stated learning objectives at the outset is a minor weakness.

Session ID:
Clinical Authenticity and Content Fidelity

Session-48:
The simulation demonstrates outstanding clinical fidelity. The patient narrative is highly plausible and indistinguishable from an authentic, complex APL case. All pharmacotherapeutic recommendationsincluding ATRA/ATO for induction, steroids for differentiation syndrome, FLAG-Ida for relapse, and ruxolitinib for steroid-refractory GVHDare accurate, guideline-concordant, and optimally chosen for the specific clinical context. Critically, the case remains strictly within its specified disease domain, with no evidence of domain entanglement. The only minor imperfection was a confusing rationale for risk stratification in Question 4, which presented conflicting versions of the Sanz score rather than applying a single, standard guideline, slightly weakening the session's overall alignment with clinical guidelines. This was a minor flaw in an otherwise exceptionally accurate simulation.

Session ID:
Modeled Clinical Reasoning

Session-48:
The simulation provides an excellent model of expert clinical reasoning. Each stage of the case presents a well-framed and highly relevant clinical problem. The multiple-choice options offer a superb breadth of plausible alternatives, including key distractors that effectively test nuanced understanding (e.g., correctly distinguishing APL-specific therapy from standard AML induction). The narrative demonstrates sound, risk-adjusted prioritization throughout, from the immediate management of differentiation syndrome to the decision to proceed to transplant after achieving a second remission. The clinical outcomes described are realistic and instructionally valuable, reinforcing the learning objectives at each step of the patient's journey.

Session-49

Session ID:
LLM

Session-49:
Google AI (Gemini 2.0 Pro)

Session ID:
Session Transcript

Session-49:
Phase 1: Initial Presentation and Diagnostic Workup.

Case Presentation: Phase 1

A 67-year-old male, Mr. John Smith, presents to his primary care physician with complaints of fatigue, easy bruising, and recent onset of fever. He reports feeling increasingly tired over the past month, noticing small bruises on his arms and legs without any significant trauma. He also mentions having a persistent low-grade fever for the past week.

Past Medical History: Hypertension, well-controlled with lisinopril 20mg daily.
Social History: Retired accountant, lives with his wife, non-smoker, occasional alcohol use (1-2 drinks per week).
Medications: Lisinopril 20mg daily, Aspirin 81mg daily (for primary prevention).
Physical Examination: Pale appearance, scattered petechiae on extremities, no significant lymphadenopathy or hepatosplenomegaly.
Initial Laboratory Results:

WBC: 2.5 x 10^9/L (Normal: 4.0-11.0 x 10^9/L)

Hemoglobin: 8.5 g/dL (Normal: 13.5-17.5 g/dL)

Platelets: 30 x 10^9/L (Normal: 150-400 x 10^9/L)

Peripheral blood smear: 20% blasts

Based on the initial presentation and laboratory results, which of the following is the MOST appropriate next step in the diagnostic workup?

A) Initiate broad-spectrum antibiotics for presumed infection.
B) Order a bone marrow biopsy and aspiration.
C) Start a trial of erythropoietin-stimulating agents (ESAs) for presumed anemia.
D) Prescribe a course of oral corticosteroids for suspected immune thrombocytopenic purpura (ITP).

Correct Answer: B) Order a bone marrow biopsy and aspiration.

Rationale: Given the patient's presentation (fatigue, bruising, fever) and the concerning peripheral blood smear showing 20% blasts, a bone marrow biopsy and aspiration are the most appropriate next steps to confirm the diagnosis of a hematologic malignancy, most likely Acute Myeloid Leukemia (AML).

the next question.

The bone marrow biopsy confirms the diagnosis of Acute Myeloid Leukemia (AML). Cytogenetic analysis reveals the presence of t(8;21)(q22;q22.1); RUNX1-RUNX1T1.

According to the 2022 European LeukemiaNet (ELN) risk stratification, this cytogenetic abnormality would classify Mr. Smith's AML as:

A) Favorable risk
B) Intermediate risk
C) Adverse risk
D) Undetermined risk
Correct Answer: A) Favorable risk
Rationale: According to the 2022 European LeukemiaNet (ELN) risk stratification, AML with t(8;21)(q22;q22.1); RUNX1-RUNX1T1 is classified as favorable risk.

Following the cytogenetic analysis, molecular testing is performed on Mr. Smith's bone marrow sample. The results show a FLT3-ITD mutation with a high allelic ratio and absence of NPM1 mutation.

Considering the presence of FLT3-ITD mutation, which of the following statements is MOST accurate regarding its impact on prognosis and treatment?

A) The FLT3-ITD mutation improves the prognosis and allows for less intensive chemotherapy.
B) The FLT3-ITD mutation worsens the prognosis and may warrant the addition of a FLT3 inhibitor to standard induction chemotherapy.
C) The FLT3-ITD mutation has no significant impact on prognosis or treatment decisions.
D) The FLT3-ITD mutation is only relevant in patients with relapsed or refractory AML.

Correct Answer: B) The FLT3-ITD mutation worsens the prognosis and may warrant the addition of a FLT3 inhibitor to standard induction chemotherapy.

Rationale: The FLT3-ITD mutation is a common mutation in AML and is generally associated with a poorer prognosis, including a higher risk of relapse and shorter overall survival. The presence of this mutation often warrants the addition of a FLT3 inhibitor, such as midostaurin, gilteritinib, or quizartinib, to standard induction chemotherapy to improve outcomes.

Based on the information so far (age, cytogenetics, and molecular markers), how would you classify Mr. Smith's AML according to the 2022 ELN risk stratification?

A) Favorable
B) Intermediate-risk
C) Adverse
D) Cannot be determined without additional information
That is correct!

Feedback:

Correct Answer: B) Intermediate-risk

Rationale: Although the presence of t(8;21) is typically associated with favorable-risk AML, the co-occurrence of FLT3-ITD with a high allelic ratio in the absence of NPM1 mutation shifts the risk stratification to intermediate-risk according to the 2022 ELN guidelines. .

Prior to initiating induction chemotherapy, it is essential to assess the patient's organ function and overall health status. Which of the following assessments is MOST critical to perform immediately before starting treatment in Mr. Smith?

A) Pulmonary function tests (PFTs)
B) Comprehensive echocardiogram (ECHO) or MUGA scan
C) Geriatric assessment including assessment of comorbidities and functional status
D) Fasting lipid panel

Correct Answer: B) Comprehensive echocardiogram (ECHO) or MUGA scan

Rationale: Given that the planned induction chemotherapy will likely include an anthracycline (e.g., daunorubicin or idarubicin), it is crucial to assess the patient's cardiac function before starting treatment.

Case Presentation: Phase 2

Based on the diagnostic workup, Mr. Smith is diagnosed with AML with t(8;21)(q22;q22.1); RUNX1-RUNX1T1 and FLT3-ITD (high allelic ratio). His ECOG performance status is 1. The ECHO reveals a normal LVEF of 60%. Creatinine clearance is calculated to be 70 mL/min.

Considering Mr. Smith's diagnosis, age, performance status, cardiac function, and renal function, which of the following induction regimens is the MOST appropriate initial treatment?

A) 7+3 (7 days of cytarabine + 3 days of daunorubicin)
B) 7+3 with midostaurin
C) CPX-351 (liposomal daunorubicin and cytarabine)
D) Low-dose cytarabine (LDAC) with venetoclax
That is correct.

Feedback:

Correct Answer: B) 7+3 with midostaurin

Rationale: Given Mr. Smith's diagnosis of AML with t(8;21) and FLT3-ITD, the addition of a FLT3 inhibitor to standard 7+3 induction chemotherapy is warranted. Midostaurin is a multi-kinase inhibitor with activity against FLT3 and has been shown to improve outcomes when added to 7+3 in patients with FLT3-ITD mutations (e.g., RATIFY trial).

Mr. Smith's ECOG performance status is 1, suggesting that he can tolerate intensive chemotherapy.
Mr. Smith is started on 7+3 with midostaurin. On day 5 of induction, he develops a fever of 38.5C (101.3F) and his absolute neutrophil count (ANC) is 0.1 x 10^9/L.

Which of the following is the MOST appropriate initial management strategy?

A) Obtain blood cultures and start empiric broad-spectrum antibiotics.
B) Administer granulocyte colony-stimulating factor (G-CSF) to stimulate neutrophil recovery.
C) Initiate antifungal prophylaxis with fluconazole.
D) Transfuse platelets to maintain a platelet count above 10 x 10^9/L
That is correct!

Feedback:

Correct Answer: A) Obtain blood cultures and start empiric broad-spectrum antibiotics.

Rationale: Febrile neutropenia is a medical emergency in patients undergoing chemotherapy. The most immediate concern is the risk

of overwhelming bacterial infection. Therefore, obtaining blood cultures to identify the causative organism and initiating empiric broad-spectrum antibiotics are the most appropriate initial steps.

Despite empiric broad-spectrum antibiotics, Mr. Smith remains febrile after 72 hours. Blood cultures are negative.

Which of the following is the MOST appropriate next step in managing his persistent febrile neutropenia?

A) Continue the current antibiotics and monitor closely.
B) Add an antifungal agent to the antibiotic regimen.
C) Add an antiviral agent to the antibiotic regimen.
D) Obtain a CT scan of the chest, abdomen, and pelvis to evaluate for a source of infection.
That is correct!

Feedback:

Correct Answer: B) Add an antifungal agent to the antibiotic regimen.

Rationale: In a patient with persistent febrile neutropenia despite 72 hours of broad-spectrum antibiotics and negative blood cultures, the likelihood of a fungal infection increases. Adding an antifungal agent (e.g., fluconazole, voriconazole, or an echinocandin, depending on local guidelines and risk factors) is the most appropriate next step.

Mr. Smith eventually recovers from febrile neutropenia and achieves complete remission (CR) after induction therapy. A bone marrow aspirate confirms <5% blasts and normalization of blood counts.

After achieving CR, what is the MOST appropriate next step in his treatment plan?

A) Proceed directly to allogeneic hematopoietic stem cell transplantation (alloHSCT).
B) Administer consolidation chemotherapy.
C) Initiate maintenance therapy with low-dose cytarabine.
D) Observation with regular monitoring of blood counts.
That is correct!

Feedback:

Correct Answer: B) Administer consolidation chemotherapy.

Rationale: After achieving complete remission (CR) following induction therapy for AML, consolidation chemotherapy is the standard of care for patients who are not immediately proceeding to allogeneic stem cell transplant (alloSCT). Consolidation aims to eradicate any remaining leukemic cells and prolong remission duration.

Case Presentation: Phase 3

Mr. Smith receives two cycles of high-dose cytarabine-based consolidation chemotherapy. He tolerates the treatment reasonably well, with manageable myelosuppression. After consolidation, minimal residual disease (MRD) testing is performed via multiparameter flow cytometry and is found to be negative (MRD <0.01%).

Given the negative MRD status after consolidation, which of the following is the MOST appropriate next step in Mr. Smith's treatment

plan?

A) Proceed to allogeneic hematopoietic stem cell transplantation (alloHSCT).
B) Continue with additional cycles of consolidation chemotherapy.
C) Initiate maintenance therapy with sorafenib.
D) Observation with regular MRD monitoring.
That is correct!

Feedback:

Correct Answer: D) Observation with regular MRD monitoring.

Rationale: Achieving MRD negativity after consolidation chemotherapy is a favorable prognostic indicator in AML. In patients with intermediate-risk disease and negative MRD, observation with regular MRD monitoring is a reasonable approach. Proceeding directly to alloHSCT may not be necessary in this setting, as the risk of relapse is lower.

.

Six months after completing consolidation chemotherapy, Mr. Smith's MRD becomes positive (0.1% by flow cytometry). His blood counts remain within normal limits, and he is asymptomatic.

Which of the following is the MOST appropriate next step in management?

A) Initiate preemptive therapy with azacitidine and venetoclax.
B) Proceed directly to allogeneic hematopoietic stem cell transplantation (alloHSCT).
C) Restart induction chemotherapy with 7+3.
D) Observation with frequent monitoring of blood counts and MRD
That is correct!

Feedback:

Correct Answer: B) Proceed directly to allogeneic hematopoietic stem cell transplantation (alloHSCT).

Rationale: The detection of MRD positivity after achieving remission, even in the absence of overt relapse, indicates a higher risk of hematological relapse. Allogeneic hematopoietic stem cell transplantation (alloHSCT) is the preferred potentially curative approach for patients with relapsed or high-risk AML and a suitable donor.

Mr. Smith undergoes successful alloHSCT. He experiences grade II acute graft-versus-host disease (aGVHD) involving the skin, which is managed with topical corticosteroids. He is now 1 year post-transplant, in complete remission, and MRD-negative. He is currently taking tacrolimus for GVHD prophylaxis.

Which of the following vaccinations should be PRIORITIZED for Mr. Smith at this time, according to current guidelines?

A) Live attenuated influenza vaccine (LAIV)
B) Pneumococcal conjugate vaccine (PCV13) followed by pneumococcal polysaccharide vaccine (PPSV23)
C) Varicella-zoster vaccine (Zostavax)
D) Measles, mumps, and rubella (MMR) vaccine
That is correct!

Feedback:

Correct Answer: B) Pneumococcal conjugate vaccine (PCV13) followed by pneumococcal polysaccharide vaccine (PPSV23)

Rationale: Patients who have undergone alloHSCT have impaired immunity and are at increased risk of infections, particularly encapsulated bacteria such as Streptococcus pneumoniae.

Five years post-transplant, Mr. Smith remains in complete remission and MRD-negative. He has successfully tapered off all immunosuppression and has no evidence of chronic GVHD.

Which of the following is the MOST appropriate long-term follow-up strategy for Mr. Smith?

A) Annual bone marrow aspirate and biopsy to monitor for relapse.
B) Regular monitoring of blood counts and MRD every 3-6 months indefinitely.
C) Routine surveillance imaging (e.g., CT scans) to screen for secondary malignancies.
D) Standard age-appropriate cancer screening and monitoring for late effects of treatment.
That is correct!

Feedback:

Correct Answer: D) Standard age-appropriate cancer screening and monitoring for late effects of treatment.

Rationale: After 5 years in complete remission post-alloHSCT and with no evidence of GVHD, Mr. Smith is considered to have a very low risk of relapse. The most appropriate long-term follow-up strategy is to focus on standard age-appropriate cancer screening (e.g., colonoscopy, prostate cancer screening) and monitoring for late effects of treatment (e.g., cardiovascular disease, secondary malignancies).

During a routine follow-up appointment, Mr. Smith reports experiencing persistent fatigue despite being in remission.

Which of the following is the MOST important initial step in evaluating his fatigue?

A) Prescribe a trial of stimulant medication (e.g., methylphenidate).
B) Order a comprehensive laboratory evaluation, including CBC, CMP, TSH, iron studies, and vitamin D level.
C) Refer the patient to a psychiatrist for evaluation of depression.
D) Recommend increased physical activity and improved sleep hygiene.
That is correct!

Feedback:

Correct Answer: B) Order a comprehensive laboratory evaluation, including CBC, CMP, TSH, iron studies, and vitamin D level.
Rationale: Fatigue is a common complaint among cancer survivors and can have various underlying causes.

Session ID:
Assessment Domain

Session-49:
Rationale

Session ID:

Instructional Design and Pedagogical

Session-49:

The session demonstrates exceptional instructional design. The case is expertly scaffolded, guiding the learner through the entire patient journey from initial diagnosis and complex risk stratification (correctly integrating cytogenetic and molecular data per 2022 ELN guidelines) to induction, management of febrile neutropenia, consolidation, MRD-guided therapy decisions, and long-term survivorship. The embedded feedback is a major strength, providing clear, evidence-based rationales for each correct answer. The only minor area for improvement is that the overarching learning objectives are not explicitly stated at the beginning, though they are clearly implied by the sessions logical and comprehensive structure.

Session ID:

Clinical Authenticity and Content Fidelity

Session-49:

The clinical fidelity of this simulation is outstanding and serves as a model of accuracy. Plausibility & Guideline Alignment: The clinical narrative is highly realistic and mirrors an authentic patient case. All diagnostic and therapeutic decisions, from risk stratification to treatment selection (e.g., 7+3 with midostaurin for a fit, FLT3-mutated patient) and complication management, are in perfect alignment with current, evidence-based guidelines (e.g., ELN, NCCN). Pharmacotherapeutic Accuracy: The pharmacotherapeutic choices are optimal and well-justified at every step. The selection of induction therapy, management of febrile neutropenia, choice of consolidation, and post-transplant care are all standard-of-care. Domain Specificity: The simulation exhibits perfect domain specificity. It remains strictly focused on AML-specific diagnostics, risk factors (FLT3-ITD), and therapies (midostaurin). There are no instances of domain entanglement where concepts or treatments from other hematologic malignancies are incorrectly applied. This content-level accuracy makes the simulation both safe and highly effective for education.

Session ID:

Modeled Clinical Reasoning

Session-49:

Rationale The simulation expertly models a sophisticated clinical reasoning process. Each stage of the case frames a clear and relevant clinical problem, providing the necessary data for an informed decision. The multiple-choice options are particularly strong, offering a breadth of plausible alternatives and key distractors that challenge the learner to consider nuanced aspects of AML management (e.g., distinguishing between induction options for different patient subgroups). The narrative demonstrates sound, risk-adjusted prioritization throughout, from securing a full diagnostic workup before treatment to using MRD status to guide post-consolidation strategy. The clinical outcomes are realistic and highly instructive, reinforcing the consequences of each decision.

Session-50

Session ID:
LLM

Session-50:
Google AI (Gemini 2.0 Pro)

Session ID:
Session Transcript

Session-50:
Mr. Ali Hassan is a 68-year-old male who presents to his primary care physician with a one-month history of increasing fatigue, shortness of breath on exertion, and easy bruising. He reports no significant past medical history other than well-controlled hypertension managed with low-dose hydrochlorothiazide. He denies fever, night sweats, or bone pain.
Initial Laboratory Findings:
 * White Blood Cell (WBC) count: 35,000/L (Normal: 4,000-10,000/L)
 * Hemoglobin (Hgb): 8.5 g/dL (Normal: 13.5-17.5 g/dL)
 * Platelet count: 45,000/L (Normal: 150,000-400,000/L)
 * Peripheral blood smear reveals the presence of >20% blasts.
Based on these initial findings, which of the following is the most likely next step in the diagnostic workup for Mr. Hassan, according to ESMO guidelines for AML diagnosis?
A) Initiate empiric broad-spectrum antibiotics due to potential infection.
B) Schedule a bone marrow biopsy for further morphological and cytogenetic analysis.
C) Order a CT scan of the chest, abdomen, and pelvis to assess for extramedullary disease.
D) Start immediate blood transfusions to address anemia and thrombocytopenia.
The correct answer is B) Schedule a bone marrow biopsy for further morphological and cytogenetic analysis.
Reasoning:
A bone marrow biopsy is the gold standard for diagnosing AML. It allows for:
 * Morphological assessment: Confirmation of >20% blasts in the bone marrow.
 * Cytogenetic analysis: Identification of chromosomal abnormalities, which are crucial for risk stratification according to the ELN 2022 criteria.
 * Molecular analysis: Detection of gene mutations (e.g., FLT3, NPM1, IDH1/2), which are also essential for diagnosis, risk stratification, and treatment planning.
Question 2:
Phase 1: Initial Presentation and Diagnostic Workup (Continued)
Mr. Hassan undergoes a bone marrow biopsy. The results reveal:
 * Bone marrow cellularity: 85% with >30% blasts
 * Flow cytometry confirms the blasts are myeloid lineage.
 * Cytogenetic analysis shows a normal karyotype (karyotype reported as 46,XY).
 * Molecular analysis reveals a mutation in the NPM1 gene and no FLT3-ITD or FLT3-TKD mutations.
Based on the WHO 2022 classification and the molecular findings, how would you classify Mr. Hassan's AML?
A) AML with myelodysplasia-related changes
B) AML with recurrent genetic abnormalities: AML with NPM1 mutation
C) Myeloid sarcoma
D) Therapy-related myeloid neoplasm

You selected B) AML with recurrent genetic abnormalities: AML with NPM1 mutation.
Correct!
Feedback:
 * Correct. The presence of an NPM1 mutation in the absence of specific cytogenetic abnormalities (like t(8;21), inv(16), or t(15;17)) is a recognized entity in the WHO 2022 classification of AML under the category of "AML with recurrent genetic abnormalities."
Based on the ELN 2022 risk stratification for newly diagnosed AML, how would you classify Mr. Hassan's risk? (Remember he is 68

years old and has an NPM1 mutation without FLT3-ITD or FLT3-TKD mutations and a normal karyotype).
A) Favorable risk
B) Intermediate risk
C) Adverse risk
D) Not enough information to determine risk.
The correct answer is A) Favorable risk.
Reasoning:
According to the ELN 2022 risk stratification:
* Favorable Risk: Includes AML with NPM1 mutation without FLT3-ITD or with FLT3-ITD low allelic ratio (<0.5), and certain favorable cytogenetic abnormalities like t(15;17), t(8;21), and inv(16) or t(16;16). Mr. Hassan fits this category due to his NPM1 mutation and the absence of FLT3-ITD. A normal karyotype in the presence of NPM1 mutation also supports this classification.
Now that we have established the diagnosis and risk stratification,
Mr. Hassan's age (68 years), performance status (let's assume he has an ECOG performance status of 1, indicating he is ambulatory and able to carry out light work), and favorable-risk AML based on ELN 2022 criteria, which of the following would be a reasonable initial induction therapy?
A) High-dose cytarabine-based therapy (HiDAC)
B) Standard "7+3" (7 days of cytarabine and 3 days of an anthracycline)
C) Low-intensity therapy with azacitidine plus venetoclax
D) Clinical trial evaluating a novel targeted agent for NPM1-mutated AML

The correct answer is C) Low-intensity therapy with azacitidine plus venetoclax.
Reasoning:
For older adults with AML, especially those who may have comorbidities or are not considered ideal candidates for intensive chemotherapy, low-intensity regimens have become a standard of care. The combination of azacitidine (a hypomethylating agent) and venetoclax (a BCL-2 inhibitor) has demonstrated significant efficacy and improved survival in this patient population, including those with NPM1-mutated AML.
Option A (HiDAC) is typically used for younger, fit patients or in the relapsed/refractory setting. Option D (clinical trial) is a valid consideration at any stage, but we are asked for a reasonable initial therapy based on current evidence. While trials for NPM1-mutated AML exist, azacitidine-venetoclax is a guideline-supported frontline option for this patient profile.
Let's proceed to the next question, which will focus on supportive care
Question 5:
Phase 2: Induction Therapy and Supportive Care
Mr. Hassan starts induction therapy with azacitidine and venetoclax. Approximately one week into treatment, his neutrophil count drops significantly to <500/L, and he develops a fever of 38.5C. Blood cultures are drawn, and he is started on empiric broad-spectrum antibiotics.
Which of the following is the most appropriate next step in managing Mr. Hassan's febrile neutropenia, according to established guidelines?
A) Add an antifungal agent to his antibiotic regimen immediately.
B) Continue the current antibiotic regimen and monitor closely.
C) Add a granulocyte-colony stimulating factor (G-CSF) such as filgrastim.
D) Switch to a different broad-spectrum antibiotic with broader Gram-negative coverage.
The most appropriate next step is B) Continue the current antibiotic regimen and monitor closely.
Reasoning:
The initial management of febrile neutropenia involves:
* Prompt initiation of empiric broad-spectrum antibiotics: This has already been done for Mr. Hassan.
* Thorough clinical assessment and investigation: Including blood cultures and other relevant cultures to identify the source of infection.
* Close monitoring: Regular
assuming Mr. Hassan's fever resolves after 48 hours of antibiotics, and his blood cultures remain negative. His neutrophil count remains low.
What is the most appropriate next step regarding his azacitidine and venetoclax treatment cycle?
A) Hold both azacitidine and venetoclax until his neutrophil count recovers to >1000/L.
B) Continue azacitidine at the scheduled dose and hold venetoclax until neutrophil recovery.

C) Reduce the dose of both azacitidine and venetoclax and continue treatment.
D) Continue both azacitidine and venetoclax at the scheduled doses, with close monitoring of blood counts.

The most appropriate next step is A) Hold both azacitidine and venetoclax until his neutrophil count recovers to >1000/L.
Reasoning:
Given the recent episode of febrile neutropenia, which is likely related to the myelosuppressive effects of the induction therapy, it is prudent to hold both azacitidine and venetoclax to allow Mr. Hassan's neutrophil count to recover to a safer level (>1000/L). Continuing treatment with significant neutropenia could increase his risk of further infections and complications.
Once the neutrophil count recovers, treatment can be restarted, often at the same initial doses, with close monitoring of blood counts. Dose reductions might be considered in subsequent cycles if prolonged or recurrent severe cytopenias occur despite dose interruptions.
assuming Mr. Hassan's neutrophil count recovers to 1200/L after a brief hold. His fever remains resolved, and he completes the first cycle of azacitidine and venetoclax. A bone marrow aspirate is performed on day 28 to assess for response.
The bone marrow aspirate shows <5% blasts, no circulating blasts, and recovery of peripheral blood counts (ANC >1000/L, platelets >100,000/L, Hgb >11 g/dL).
According to the International Working Group (IWG) criteria, what is Mr. Hassan's response to the first cycle of induction therapy?
A) Complete Remission (CR)
B) Complete Remission with incomplete hematologic recovery (CRi)
C) Partial Remission (PR)
D) Relapsed/Refractory disease

The correct answer is A) Complete Remission (CR).
Reasoning:
According to the IWG criteria for response assessment in AML:
* Complete Remission (CR): Requires all of the following:
  * 5% blasts in the bone marrow
  * No circulating blasts or extramedullary disease
  * Absolute neutrophil count (ANC) 1.0 10^9/L
  * Platelet count 100 10^9/L
  * Independence from red blood cell (RBC) transfusion (no RBC transfusion in the 7 days prior to assessment) and platelet transfusion (no platelet transfusion in the 7 days prior to assessment), although this last criterion might be modified in the context of specific therapies. In our case, we don't have information suggesting ongoing transfusion needs.

Since Mr. Hassan meets all these criteria, he has achieved a Complete Remission (CR).
Now that Mr. Hassan has achieved CR, the next crucial step is to plan for post-remission therapy. Given his age (68 years), favorable-risk AML (NPM1 mutated, FLT3-ITD negative, normal karyotype), and achievement of CR with azacitidine and venetoclax, which of the following would be the most appropriate next step in his treatment?
A) Observation with regular monitoring.
B) Consolidation therapy with high-dose cytarabine (HiDAC).
C) Maintenance therapy with azacitidine.
D) Maintenance therapy with azacitidine plus venetoclax
You selected D) Maintenance therapy with azacitidine plus venetoclax.
Correct!
Feedback:
* Correct. Recent evidence has demonstrated the benefit of maintenance therapy with azacitidine plus venetoclax in older adults with AML who achieve CR after induction with this combination, regardless of their risk stratification. This approach has shown to improve relapse-free survival and overall survival.

Guideline Reference: Current ESMO guidelines and recent clinical trial data (e.g., VIALE-A study follow-up) support the use of azacitidine plus venetoclax maintenance in this setting.
The correct answer is D) Maintenance therapy with azacitidine plus venetoclax.
Reasoning:
Maintenance therapy aims to prevent relapse and prolong remission. For older adults with AML who achieve remission with azacitidine and venetoclax, the addition of venetoclax to azacitidine maintenance has demonstrated a significant improvement in relapse-free survival and overall survival compared to azacitidine alone or placebo. This has become the standard of care based on the evidence from clinical trials.

which will focus on Measurable Residual Disease (MRD) monitoring.
Mr. Hassan begins maintenance therapy with azacitidine and venetoclax. Three months into maintenance, a bone marrow aspirate is performed for MRD assessment using multiparameter flow cytometry (MFC). The results show MRD positivity at a level of 0.05% (5 leukemic cells per 10,000 nucleated cells).
According to current guidelines, how should this MRD result influence Mr. Hassan's ongoing maintenance therapy?
A) MRD positivity at this level is expected during maintenance and does not require any change in therapy.
B) This level of MRD positivity indicates impending relapse, and he should be considered for more intensive therapy or clinical trial enrollment.
C) The maintenance therapy should be discontinued immediately due to treatment failure.
D) The dose of venetoclax should be increased while continuing azacitidine at the current dose.
focusing on potential complications during long-term maintenance.
Six months into maintenance therapy, Mr. Hassan develops persistent grade 2 thrombocytopenia (platelet counts between 50,000-75,000/L) without any active bleeding. His neutrophil counts remain stable above 1000/L, and he is not requiring any platelet transfusions.
What is the most appropriate management strategy for this persistent thrombocytopenia?
A) Immediately discontinue venetoclax and continue azacitidine.
B) Reduce the dose of azacitidine while continuing venetoclax at the current dose.
C) Reduce the dose of venetoclax while continuing azacitidine at the current dose.
D) Continue both azacitidine and venetoclax at the current doses and monitor platelet counts closely.
The most appropriate management strategy is C) Reduce the dose of venetoclax while continuing azacitidine at the current dose.
Reasoning:
Persistent grade 2 thrombocytopenia, while not immediately life-threatening, can increase the risk of bleeding and may necessitate dose adjustments to allow for better tolerance of the maintenance therapy. Since both azacitidine and venetoclax can contribute to thrombocytopenia, reducing the dose of one of the agents is a reasonable step.
One year into maintenance therapy, Mr. Hassan remains in remission with stable low-level MRD. He mentions increasing fatigue and occasional mild shortness of breath, although his blood counts remain within the normal range (ANC >1500/L, Hgb 12.5 g/dL, platelets 160,000/L). He denies any signs of infection or bleeding.
What is the most important next step to evaluate Mr. Hassan's new symptoms?
A) Order a repeat bone marrow biopsy to assess for disease relapse or myelodysplastic syndrome.
B) Perform a comprehensive cardiac evaluation, including an echocardiogram and BNP level.
C) Obtain a detailed social history to assess for psychosocial factors contributing to fatigue.
D) Increase the dose of azacitidine and venetoclax to potentially improve his energy levels.

You selected B) Perform a comprehensive cardiac evaluation, including an echocardiogram and BNP level.
Correct!
Feedback:
* Correct. While fatigue and shortness of breath can be non-specific symptoms in patients with a history of AML, especially those on maintenance therapy, a cardiac etiology should be considered, particularly in an older patient. Both azacitidine and venetoclax have potential cardiovascular side effects, and pre-existing conditions could be exacerbated.
Reasoning:
New onset or worsening fatigue and shortness of breath in a patient on maintenance therapy warrants a thorough investigation to identify the underlying cause. While relapse (Option A) is a possibility in AML, the normal blood counts make it less likely as the primary cause of these new symptoms. A cardiac evaluation is crucial to rule out or identify any drug-related or age-related cardiac dysfunction.
Option C, assessing psychosocial factors, is important for overall patient well-being but should likely follow a medical evaluation to rule out organic causes. Option D, increasing the doses of maintenance therapy without a clear indication of disease progression or a specific rationale for symptom improvement, is not appropriate and could potentially worsen side effects.

Session ID:
Assessment Domain

Session-50:
Rationale

Session ID:

Instructional Design and Pedagogical

Session-50:

The session's instructional design is of very high quality. It demonstrates expert scaffolding, logically progressing the learner from initial presentation and diagnosis through risk stratification, induction therapy, complication management, response assessment, and long-term maintenance. The embedded feedback is a significant strength, with rationales that are clear, evidence-based, and consistently reference major clinical guidelines (e.g., ESMO, ELN, WHO). The framing of each question is unambiguous. The only minor area for improvement is the lack of explicitly stated learning objectives at the beginning of the session; while the educational goals are clearly implied by the session's structure, formally stating them would elevate the design from very good to excellent.

Session ID:

Clinical Authenticity and Content Fidelity

Session-50:

The clinical content of this session is outstanding and demonstrates exceptional fidelity. Plausibility & Guideline Alignment: The clinical narrative is indistinguishable from an authentic case, presenting a classic scenario of AML in an older adult. All diagnostic steps, risk stratification according to ELN 2022 criteria, and therapeutic decisions are perfectly aligned with current, evidence-based guidelines. The choice of low-intensity azacitidine and venetoclax for induction and maintenance is an optimal, guideline-directed therapeutic choice for this specific patient profile. Pharmacotherapeutic Accuracy: The pharmacotherapeutic recommendations and side-effect management strategies (e.g., holding therapy for febrile neutropenia, reducing venetoclax for thrombocytopenia) are accurate and reflect current standards of care. Domain Specificity: The session demonstrates perfect domain specificity. The entire case, including diagnostics, molecular markers, and therapeutic agents, remains strictly within the domain of Acute Myeloid Leukemia. There is no evidence of domain entanglement; concepts or standards of care from other hematologic malignancies are not introduced or misapplied.

Session ID:

Modeled Clinical Reasoning

Session-50:

The simulation provides an expert model of the clinical reasoning process in AML management. Each question frames a clear and relevant clinical problem, from diagnosis to long-term care. The multiple-choice options offer an excellent breadth of plausible alternatives, including key distractors that effectively test nuanced clinical understanding (e.g., offering intensive chemotherapy vs. low-intensity therapy). The overall narrative demonstrates sound, risk-adjusted prioritization and decision-making throughout. The clinical outcomes described at each stage are realistic and highly instructive, reinforcing the key learning objectives for each decision point.

Session-51

Session ID:
LLM

Session-51:
Google AI Studio (Gemini 2.0 Pro)

Session ID:
Session Transcript

Session-51:
A 55-year-old male presents to his primary care physician with complaints of fatigue, night sweats, and a 10-lb unintentional weight loss over the past 3 months. He also notes early satiety. Physical examination reveals splenomegaly. A complete blood count (CBC) shows: WBC 150 x 10^9/L with a left shift (myelocytes, metamyelocytes, blasts <2%), Hemoglobin 10.5 g/dL, and Platelets 550 x 10^9/L.
Which of the following cytogenetic abnormalities is MOST characteristic of Chronic Myeloid Leukemia (CML)?
A) Trisomy 12
B) t(15;17)
C) t(9;22)(q34;q11.2)
D) del(5q)
Correct Answer (C): The t(9;22)(q34;q11.2) translocation, famously known as the Philadelphia chromosome, is the hallmark cytogenetic abnormality in CML. This translocation creates the BCR-ABL1 fusion gene, which encodes a constitutively active tyrosine kinase responsible for the pathogenesis of the disease. The clinical presentation (fatigue, night sweats, weight loss, splenomegaly) and laboratory findings (marked leukocytosis with left shift, thrombocytosis, mild anemia) in this patient are classic for CML in the chronic phase.
Incorrect Answers:
A) Trisomy 12: This is frequently seen in Chronic Lymphocytic Leukemia (CLL).
B) t(15;17): This translocation is characteristic of Acute Promyelocytic Leukemia (APL), a subtype of AML.
C) del(5q): This deletion is commonly associated with Myelodysplastic Syndromes (MDS).
The patient undergoes a bone marrow biopsy. Cytogenetic analysis confirms the presence of the t(9;22) translocation. Quantitative RT-PCR for BCR-ABL1 transcripts is performed. Which phase of CML is this patient most likely in, based on the initial presentation and CBC results (WBC 150 x 10^9/L, blasts <2%)?
A) Chronic Phase (CP)
B) Accelerated Phase (AP)
C) Blast Phase (BP)
D) Remission Phase
Correct Answer (A): The patient is in the Chronic Phase (CP) of CML. This is defined by the presence of the Philadelphia chromosome, characteristic symptoms (if any), splenomegaly, and typically <10% blasts in the peripheral blood and/or bone marrow (although some definitions use <15%). This patient's presentation, particularly the blast count of <2%, clearly aligns with the Chronic Phase.
Incorrect Answers:
B) Accelerated Phase (AP): This phase is characterized by signs of disease progression, such as 10-19% blasts in blood/marrow, >20% basophils in blood, persistent thrombocytopenia unrelated to therapy, or clonal evolution (additional cytogenetic abnormalities).
C) Blast Phase (BP): This phase resembles acute leukemia, defined by 20% blasts in blood/marrow or extramedullary blast proliferation.
D) Remission Phase: This phase occurs after successful treatment, not at initial diagnosis.
Before initiating therapy, risk stratification is essential. The Sokal score is calculated for this 55-year-old patient. In addition to his age, spleen size, platelet count, and blast percentage, which other laboratory value is required to calculate the Sokal score?
A) Serum Lactate Dehydrogenase (LDH)
B) Serum Creatinine
C) Peripheral Blood Eosinophil Percentage
D) Hemoglobin Level

Incorrect Answer (C): While the peripheral blood eosinophil percentage is used in other CML risk stratification systems like the Hasford (Euro) score and the ELTS score, it is not a component of the original Sokal score.
Correct Concept: The Sokal score is calculated using the following variables:
Age (years)
Spleen size (cm below the costal margin)
Platelet count (x 10^9/L)
Peripheral blood blast percentage (%)
Other Incorrect Answers:
A) Serum Lactate Dehydrogenase (LDH): LDH can be elevated in CML due to high cell turnover but is not part of the Sokal calculation.
B) Serum Creatinine: Important for assessing renal function, especially for TKI selection and dosing, but not used in the Sokal score.
D) Hemoglobin Level: While often decreased (anemia), hemoglobin is not part of the Sokal score calculation.
The Sokal score helps predict prognosis and response to older therapies like interferon, but newer scores like ELTS are often preferred for predicting response to tyrosine kinase inhibitors (TKIs).

Given that the patient has Chronic Phase CML driven by the BCR-ABL1 fusion gene (confirmed by the t(9;22) translocation), what is the PRIMARY therapeutic goal when initiating treatment with a Tyrosine Kinase Inhibitor (TKI)?
A) Rapidly decrease spleen size to alleviate symptoms.
B) Achieve a major molecular response (MMR) and eventually deeper responses by targeting the BCR-ABL1 kinase activity.
C) Normalize the white blood cell count as quickly as possible.
D) Prevent transformation to the accelerated or blast phase solely through supportive care.

Incorrect Answer (C): Normalizing the WBC count is a consequence of effective therapy but doesn't reflect the depth of response at the molecular level, which is crucial for long-term outcomes.
Correct Answer (B): The primary goal of TKI therapy is to inhibit the constitutively active BCR-ABL1 tyrosine kinase. This targeted inhibition aims to reduce the BCR-ABL1 transcript levels significantly, ideally achieving a Major Molecular Response (MMR, defined as 0.1% BCR-ABL1 on the International Scale) or even deeper responses. Achieving these molecular milestones correlates strongly with improved progression-free and overall survival and is the main objective.
Other Incorrect Answers:
A) Rapidly decrease spleen size: Symptom control is important, and spleen size reduction is a sign of response, but it's secondary to targeting the underlying molecular defect.
D) Prevent transformation... solely through supportive care: TKIs are the active agents that prevent transformation by suppressing the CML clone. Supportive care manages symptoms and complications but doesn't prevent disease progression on its own.
Imatinib, a first-generation TKI, is being considered for this patient. How does imatinib exert its therapeutic effect in CML?
A) It induces apoptosis in all rapidly dividing cells.
B) It competitively inhibits the ATP binding site on the BCR-ABL1 tyrosine kinase.
C) It enhances the immune system's ability to recognize and destroy CML cells.
D) It directly repairs the t(9;22) chromosomal translocation.

Incorrect Answer (D): Imatinib is a targeted therapy, but it does not repair the underlying chromosomal damage (the t(9;22) translocation). The translocation remains present in the CML cells even during treatment.
Correct Answer (B): Imatinib is a tyrosine kinase inhibitor. The BCR-ABL1 fusion gene produces an abnormal tyrosine kinase protein that is always "on," signaling the CML cells to grow and divide uncontrollably. Imatinib works by fitting into the ATP (energy) binding site of this BCR-ABL1 kinase. By blocking ATP binding, it prevents the kinase from functioning (phosphorylating its downstream targets), thereby inhibiting the signaling pathways that drive CML cell proliferation and survival.
Other Incorrect Answers:
A) It induces apoptosis in all rapidly dividing cells: This describes the mechanism of traditional cytotoxic chemotherapy, which is less specific than TKIs. Imatinib is much more targeted to cells expressing BCR-ABL1.
C) It enhances the immune system's ability to recognize and destroy CML cells: While there might be some indirect immune effects, this is not the primary mechanism of imatinib. This describes immunotherapy agents.
Besides imatinib, other TKIs like dasatinib, nilotinib, and bosutinib are available for first-line treatment of Chronic Phase CML. Which of the following patient factors is LEAST likely to influence the initial choice between these specific TKIs?
A) Patient's pre-existing cardiovascular conditions (e.g., history of QTc prolongation, pancreatitis, or peripheral artery disease).

B) The patient's Sokal or ELTS risk score.
C) The specific BCR-ABL1 transcript type (e.g., e13a2 vs. e14a2).
D) Patient preference regarding dosing schedule (e.g., once daily vs. twice daily) and food requirements

Incorrect Answer (B): The Sokal or ELTS risk score is often considered. Patients with intermediate or high-risk disease might be considered for second-generation TKIs (dasatinib, nilotinib, bosutinib) upfront, as these agents may lead to faster and deeper molecular responses compared to imatinib, which could be particularly beneficial in higher-risk settings.
Correct Answer (C): The specific common BCR-ABL1 transcript type (e13a2, also known as b2a2, or e14a2, also known as b3a2) generally has the least impact on the initial choice between the standard first-line TKIs. While some research explores potential minor differences in response kinetics, clinical guidelines do not typically differentiate first-line TKI selection based solely on whether the transcript is e13a2 or e14a2 (both produce the p210 protein). The major drivers for selection are efficacy expectations (tied to risk score), patient comorbidities, potential drug interactions, and patient preference/adherence factors.
Other Incorrect Answers:
A) Pre-existing cardiovascular conditions: This is highly influential due to the varying safety profiles. For example, nilotinib carries warnings for vascular events and QTc prolongation, dasatinib for pleural effusions and pulmonary arterial hypertension, bosutinib for GI toxicity and liver enzyme elevation, and imatinib for fluid retention. The patient's comorbidities are critical in selecting the most appropriate agent.
D) Patient preference regarding dosing schedule and food requirements: Practical aspects like once-daily vs. twice-daily dosing, and requirements regarding food intake (e.g., nilotinib's fasting requirements), can significantly impact adherence and are important considerations in shared decision-making.
A patient started on nilotinib for Chronic Phase CML develops new-onset hyperglycemia and an increase in serum lipase. Which known side effect profile of nilotinib are these findings consistent with?
A) Fluid retention and muscle cramps
B) Pancreatitis and hyperglycemia
C) Pleural effusion and thrombocytopenia
D) Diarrhea and liver enzyme elevation

Correct Answer (B): Nilotinib is known to have metabolic side effects, including hyperglycemia (elevated blood sugar) and pancreatitis (inflammation of the pancreas, often indicated by elevated serum lipase or amylase). These are important monitoring parameters for patients receiving nilotinib.
Incorrect Answers:
A) Fluid retention and muscle cramps: These are more commonly associated with imatinib.
C) Pleural effusion and thrombocytopenia: Pleural effusion (fluid around the lungs) is a characteristic potential side effect of dasatinib. Thrombocytopenia can occur with various TKIs but isn't specifically paired with pleural effusion as a defining profile for nilotinib.
D) Diarrhea and liver enzyme elevation: Diarrhea is very common with bosutinib, and liver enzyme elevations can occur with several TKIs, including bosutinib and nilotinib, but the combination presented in the question (hyperglycemia and elevated lipase) is more distinctively linked to nilotinib's specific metabolic risk profile.
Our patient achieved a Complete Hematologic Response (CHR) after 3 months of TKI therapy. Quantitative RT-PCR for BCR-ABL1 transcripts is performed at the 3-month mark, showing a transcript level of 1.5% on the International Scale (IS). According to the European LeukemiaNet (ELN) 2020 recommendations, how would this response be best classified?
A) Optimal Response
B) Warning
C) Failure
D) Major Molecular Response (MMR)

Incorrect Answer (B): According to ELN 2020, a "Warning" at 3 months is defined as having BCR-ABL1 transcript levels >10% IS.
Correct Answer (A): The patient's BCR-ABL1 level of 1.5% IS at 3 months is considered an Optimal Response. The ELN 2020 guideline defines optimal response at 3 months as achieving BCR-ABL1 IS 10%. This indicates the patient is responding well to the initial TKI therapy.
Other Incorrect Answers:
C) Failure: Failure at 3 months typically involves not achieving CHR, losing CHR, or potentially having very high transcript levels (often still using >10% as the threshold) or developing specific resistance mutations or clonal evolution. 1.5% is far from failure.
D) Major Molecular Response (MMR): MMR is a deeper response, defined as BCR-ABL1 IS 0.1%. While this is a crucial long-term goal, it is not the expected benchmark for an optimal response at only 3 months.

The patient continues on TKI therapy and achieves a sustained deep molecular response (DMR, defined as BCR-ABL1 IS 0.01% or lower) for over 2 years. The patient is highly motivated to stop therapy. What is the MOST important prerequisite condition that must be met before considering a trial of Treatment-Free Remission (TFR)?
A) Patient must be younger than 60 years old.
B) The specific TKI used must be a second-generation agent (dasatinib, nilotinib, bosutinib).
C) Duration of TKI therapy must be at least 5 years, with at least 2 years in sustained DMR.
D) The patient must have detectable BCR-ABL1 transcripts, just below the MMR threshold.

Correct Answer (C): Specific eligibility criteria for TFR attempts can vary slightly between guidelines and clinical trials, but the core requirements consistently involve minimum durations of both overall TKI therapy and, crucially, the duration of sustained deep molecular response (DMR). Typical criteria often include:
Being in the Chronic Phase CML.
Receiving TKI therapy for a minimum duration (e.g., 3 to 5 years).
Achieving and maintaining a sustained DMR (e.g., MR4 [0.01% IS] or MR4.5 [0.0032% IS]) for a minimum period (e.g., 2 years).
Access to reliable, standardized molecular monitoring.
This combination of sufficient treatment duration and prolonged deep response is considered the most critical factor for predicting successful TFR.
Incorrect Answers:
A) Patient must be younger than 60 years old: While some subgroup analyses exist, age itself is not typically a strict exclusion criterion for attempting TFR.
B) The specific TKI used must be a second-generation agent: TFR can be attempted after achieving criteria with any standard TKI, including imatinib, although kinetics might differ.
D) The patient must have detectable BCR-ABL1 transcripts, just below the MMR threshold: This is incorrect. TFR requires a deep molecular response (DMR), which is significantly lower than MMR. Detectable disease near the MMR level would preclude a TFR attempt.
If a patient successfully stops TKI therapy for TFR but subsequently experiences molecular recurrence (loss of MMR, i.e., BCR-ABL1 IS > 0.1%), what is the standard recommended management approach?
A) Immediately switch to a different TKI.
B) Re-initiate the same TKI that was previously effective, at the same standard dose.
C) Begin combination therapy with a TKI and interferon.
D) Proceed directly to allogeneic stem cell transplantation.

Incorrect Answer (C): Combination therapy with a TKI and interferon is not the standard initial management for loss of MMR during a TFR attempt. Interferon has a different mechanism and side effect profile and is reserved for specific circumstances, not routine first-line management of TFR failure.
Correct Answer (B): The vast majority of patients who experience molecular recurrence (loss of MMR) after stopping TKI therapy regain MMR rapidly upon restarting the same TKI they were taking before the TFR attempt, usually at the standard dose. This is the well-established, standard-of-care recommendation. The rationale is that the CML cells have simply started to proliferate again in the absence of TKI pressure, but they remain sensitive to the original drug.
Other Incorrect Answers:
A) Immediately switch to a different TKI: Switching TKIs is generally reserved for cases of intolerance or confirmed resistance (e.g., development of a BCR-ABL1 kinase domain mutation) while on therapy. It is not the standard approach for loss of MMR after TFR unless resistance is suspected or confirmed.
D) Proceed directly to allogeneic stem cell transplantation: This is a high-risk procedure reserved for TKI resistance, intolerance, or progression to advanced phases. It is entirely inappropriate for managing the initial loss of MMR during a TFR attempt.

Session ID:
Assessment Domain

Session-51:
Rationale

Session ID:

Instructional Design and Pedagogical

Session-51:

The instructional design is outstanding. The case progresses in a seamless, logical sequence from initial presentation and diagnosis through risk stratification, treatment selection, response monitoring, and advanced management concepts like Treatment-Free Remission (TFR). The scaffolding expertly builds complexity. The embedded feedback is a major strength; rationales are clear, explain both correct and incorrect answers with precision, and reference key guideline concepts (e.g., ELN 2020 response criteria). The questions are framed with exceptional clarity. The only minor weakness is that learning objectives are not explicitly stated at the outset, though they are clearly implied by the session's strong structure.

Session ID:

Clinical Authenticity and Content Fidelity

Session-51:

The session's content fidelity is generally high but contains one significant error. Plausibility & Accuracy: The clinical narrative is perfectly plausible, reflecting a classic presentation of Chronic Phase CML. The pharmacotherapeutic choices, side effect profiles, and management of response and TFR are all accurate and align with current clinical practice. Content Error: A notable error exists in Question 3 regarding the Sokal score. The question asks for a laboratory value needed for the calculation, but none of the provided multiple-choice options (LDH, Creatinine, Eosinophil %, Hemoglobin) are actual components of the Sokal score. The session's own explanation confirms the correct components are age, spleen size, platelet count, and blast percentage, making the question itself fundamentally flawed and unanswerable. This represents a significant inaccuracy in content related to clinical guidelines for risk stratification. Domain Specificity: Despite the error, the session does not suffer from domain entanglement. All concepts, diagnostics (e.g., distinguishing t(9;22) from markers of APL or CLL), and therapies are correctly situated within the CML domain.

Session ID:

Modeled Clinical Reasoning

Session-51:

The simulation is an excellent model of expert clinical reasoning in CML management. Each step presents a well-framed problem that is relevant and clearly defined. The multiple-choice options offer a strong breadth of plausible answers and key distractors, effectively testing nuanced knowledge (e.g., differentiating TKI side effect profiles). The overall decision-making flow demonstrates sound prioritization, correctly moving from diagnosis to risk assessment to therapy and monitoring. Even with the flawed Sokal score question, the underlying reasoningthe need to risk-stratify before choosing a therapyis modeled correctly. The clinical outcomes are realistic, specific, and highly instructive, reinforcing the learning points at each stage.

Session-52

Session ID:
LLM

Session-52:
Google AI Studio (Gemini 2.0 Pro)

Session ID:
Session Transcript

Session-52:
Okay, [REDACTED]], let's dive into Phase 1 of our case.
Phase 1: Initial Presentation and Diagnostic Workup
A 58-year-old male, Mr. John Smith, presents to his primary care physician with complaints of fatigue, night sweats, and left upper quadrant fullness over the past two months. He denies any fever, significant weight loss, or bleeding tendencies.
Physical examination reveals splenomegaly, palpable 5 cm below the left costal margin. The rest of the physical exam is unremarkable.
Initial laboratory investigations reveal the following:
 * White blood cell (WBC) count: 155 x 10^9/L (Reference range: 4-10 x 10^9/L)
 * Hemoglobin: 11.5 g/dL (Reference range: 13.5-17.5 g/dL)
 * Platelet count: 450 x 10^9/L (Reference range: 150-400 x 10^9/L)
 * Peripheral blood smear shows a left shift with the presence of granulocytes at various stages of maturation, including blasts (<5%), promyelocytes, myelocytes, metamyelocytes, and mature neutrophils, as well as basophilia.
Based on this initial presentation and peripheral blood findings, which of the following is the most likely initial diagnosis?
A) Acute Myeloid Leukemia (AML)
B) Chronic Lymphocytic Leukemia (CLL)
C) Chronic Myeloid Leukemia (CML)
D) Myelodysplastic Syndrome (MDS)

That's correct, [REDACTED]]. The constellation of findings marked leukocytosis with a left shift including immature granulocytes, splenomegaly, and the absence of significant blasts strongly suggests C) Chronic Myeloid Leukemia (CML).
That's correct, [REDACTED]]. The constellation of findings marked leukocytosis with a left shift including immature granulocytes, splenomegaly, and the absence of significant blasts strongly suggests C) Chronic Myeloid Leukemia (CML).
Now, to confirm this initial suspicion and for further risk stratification according to the 2022 European LeukemiaNet (ELN) recommendations, which of the following key diagnostic tests should be performed next?
A) Bone marrow biopsy with cytogenetic analysis for the Philadelphia chromosome (BCR-ABL1 translocation) and molecular analysis for BCR-ABL1 transcript type.
B) Flow cytometry of the peripheral blood to assess for lymphoid lineage markers.
C) Serum lactate dehydrogenase (LDH) and uric acid levels to evaluate for tumor burden.
D) Liver function tests and renal function tests to assess for end-organ damage.

Excellent, [REDACTED]]. You're absolutely right. The next crucial step is A) Bone marrow biopsy with cytogenetic analysis for the Philadelphia chromosome (BCR-ABL1 translocation) and molecular analysis for BCR-ABL1 transcript type.
The detection of the Philadelphia chromosome, resulting from the reciprocal translocation t(9;22)(q34;q11) leading to the BCR-ABL1 fusion gene, is the hallmark of CML and essential for diagnosis according to the WHO classification and ELN guidelines. Molecular analysis for the BCR-ABL1 transcript type (e.g., p210, p190, p230) is also important as it can have prognostic implications and may influence treatment response.
While the other options provide valuable information, they are not the primary diagnostic tests for confirming CML:
 * B) Flow cytometry is more relevant for diagnosing lymphoid malignancies like CLL.
 * C) Serum LDH and uric acid can be elevated in CML due to increased cell turnover, but they are not specific for the diagnosis.
 * D) Liver and renal function tests are important for assessing the patient's overall condition and for considering potential comorbidities before treatment, but they don't confirm the CML diagnosis.

Following the bone marrow analysis, Mr. Smith's results confirm the presence of the BCR-ABL1 fusion gene (p210 transcript). Cytogenetic analysis reveals the t(9;22)(q34;q11) in 95% of metaphase cells.
Excellent, [REDACTED]]. You're absolutely right. The next crucial step is A) Bone marrow biopsy with cytogenetic analysis for the Philadelphia chromosome (BCR-ABL1 translocation) and molecular analysis for BCR-ABL1 transcript type.
The detection of the Philadelphia chromosome, resulting from the reciprocal translocation t(9;22)(q34;q11) leading to the BCR-ABL1 fusion gene, is the hallmark of CML and essential for diagnosis according to the WHO classification and ELN guidelines. Molecular analysis for the BCR-ABL1 transcript type (e.g., p210, p190, p230) is also important as it can have prognostic implications and may influence treatment response.
While the other options provide valuable information, they are not the primary diagnostic tests for confirming CML:
* B) Flow cytometry is more relevant for diagnosing lymphoid malignancies like CLL.
* C) Serum LDH and uric acid can be elevated in CML due to increased cell turnover, but they are not specific for the diagnosis.
* D) Liver and renal function tests are important for assessing the patient's overall condition and for considering potential comorbidities before treatment, but they don't confirm the CML diagnosis.
Following the bone marrow analysis, Mr. Smith's results confirm the presence of the BCR-ABL1 fusion gene (p210 transcript). Cytogenetic analysis reveals the t(9;22)(q34;q11) in 95% of metaphase cells.
Now, based on the initial laboratory findings and the confirmed BCR-ABL1 positivity, the next step is to determine Mr. Smith's risk category according to the ELN 2022 risk stratification for newly diagnosed chronic phase CML. The ELN risk score typically incorporates which of the following factors?
A) Age, spleen size, and platelet count.
B) Blast percentage in peripheral blood, basophil percentage in peripheral blood, and presence of extramedullary disease.
C) Age, blast percentage in peripheral blood, basophil percentage in peripheral blood, spleen size, and platelet count.
D) Hemoglobin level, white blood cell count, and cytogenetic risk category.

The correct answer is C) Age, blast percentage in peripheral blood, basophil percentage in peripheral blood, spleen size, and platelet count.
These five factors are used to calculate the Sokal, Hasford, or EUTOS scores, which are the basis for the ELN risk stratification (low, intermediate, or high risk) in newly diagnosed chronic phase CML.
Great, [REDACTED]]. Let's proceed to Phase 2: Induction Therapy and Supportive Care Planning.
Based on Mr. Smith's age (58 years), good performance status (ECOG 0), and the confirmed diagnosis of chronic phase CML with a calculated ELN risk score placing him in the intermediate-risk category, which of the following is the recommended first-line treatment option according to the current ESMO guidelines?
A) Interferon-alpha
B) Hydroxyurea
C) Imatinib
D) A second-generation tyrosine kinase inhibitor (TKI) such as nilotinib or dasatinib

The correct answer is D) A second-generation tyrosine kinase inhibitor (TKI) such as nilotinib or dasatinib.
Current ESMO guidelines strongly recommend first- or second-generation TKIs as first-line therapy for chronic phase CML. While imatinib was the first TKI and is still an option, second-generation TKIs like nilotinib and dasatinib have demonstrated faster and deeper cytogenetic and molecular responses, potentially leading to better long-term outcomes, especially in patients without significant comorbidities.
Interferon-alpha (A) is generally reserved for specific situations or when TKIs are not tolerated or available.
Considering the choice of a second-generation
Considering the choice of a second-generation TKI, which of the following factors would be most important to consider when deciding between nilotinib and dasatinib for Mr. Smith?
A) The patient's preference for oral versus subcutaneous administration.
B) The cost and availability of each medication in the local healthcare system.
C) The potential for specific non-hematological toxicities associated with each TKI, such as cardiovascular events with nilotinib or pleural effusions with dasatinib, considering Mr. Smith's comorbidities (if any were present, which in this initial presentation, there aren't any significant ones).
D) The degree of spleen size reduction observed on initial physical examination
You're right that patient preference for the route of administration is important in many treatment decisions, [REDACTED]]. However, in this specific scenario, both nilotinib and dasatinib are orally administered medications. Therefore, A is not the most significant differentiating factor between these two second-generation TKIs.

The most crucial factor to consider when choosing between nilotinib and dasatinib in the first-line setting for Mr. Smith, given his current presentation without significant comorbidities, is C) The potential for specific non-hematological toxicities associated with each TKI, such as cardiovascular events with nilotinib or pleural effusions with dasatinib.

While Mr. Smith doesn't have significant comorbidities right now, understanding the distinct toxicity profiles of these drugs is vital for personalized treatment selection. For instance, if Mr. Smith had pre-existing cardiovascular risk factors, nilotinib might be used with more caution due to its association with an increased risk of arterial occlusive events. Conversely, if he had a history of pulmonary issues, dasatinib's potential for pleural effusions would be a consideration.

B) The cost and availability are also practical considerations but ideally come after evaluating the clinical suitability based on efficacy and safety.

D) The degree of spleen size reduction is a marker of response to treatment, not a factor in the initial selection between these two TKIs.

Let's say, after considering these factors and discussing with Mr. Smith, the decision is made to start him on nilotinib. What is the most common and clinically significant hematological toxicity associated with tyrosine kinase inhibitors like nilotinib that you would monitor closely in Mr. Smith?

A) Thrombocytopenia

B) Anemia

C) Neutropenia

D) Lymphocytosis

That's correct, [REDACTED]]. C) Neutropenia is a common and clinically significant hematological toxicity associated with TKIs like nilotinib. While thrombocytopenia (A) and anemia (B) can also occur, neutropenia often requires closer monitoring and potential dose adjustments due to the increased risk of infection. Lymphocytosis (D) is not a typical toxicity associated with TKIs; in fact, CML is characterized by a high neutrophil count initially.

Therefore, when initiating nilotinib for Mr. Smith, you would educate him about the signs and symptoms of infection and implement a strategy for regular monitoring of his complete blood counts (CBC) to detect and manage any developing neutropenia.

Now, let's consider supportive care. During the initial phase of TKI therapy, some patients with high white blood cell counts may be at risk for tumor lysis syndrome (TLS). Which of the following laboratory abnormalities would be most indicative of TLS?

A) Hyperkalemia, hyperphosphatemia, hypercalcemia, and increased creatinine.

B) Hypokalemia, hypophosphatemia, hypocalcemia, and increased creatinine.

C) Hyperkalemia, hyperphosphatemia, hypocalcemia, and increased creatinine.

D) Hypokalemia, hyperphosphatemia, hypercalcemia, and decreased creatinine.

The correct answer is C) Hyperkalemia, hyperphosphatemia, hypocalcemia, and increased creatinine.

Alright, [REDACTED]], let's move on to Phase 3: Post-Remission Therapy, MRD Monitoring, and Long-Term Management.

Mr. Smith has been on nilotinib for 12 months. He has tolerated the medication well, with only mild fatigue reported occasionally. His most recent complete blood count shows normal values. A bone marrow aspirate and biopsy performed at 12 months revealed a complete cytogenetic response (CCyR), meaning no Philadelphia chromosome-positive metaphases were detected.

According to the current ELN 2022 recommendations, what is the next crucial step in assessing Mr. Smith's response and guiding long-term management?

A) Continue nilotinib at the current dose and monitor CBC every 3 months.

B) Switch to a lower dose of nilotinib to minimize potential long-term toxicities.

C) Assess the level of BCR-ABL1 transcript in peripheral blood using quantitative reverse transcriptase polymerase chain reaction (qRT-PCR) to evaluate for major molecular response (MMR).

D) Perform another bone marrow biopsy to confirm the complete cytogenetic response.

The correct answer is C) Assess the level of BCR-ABL1 transcript in peripheral blood using quantitative reverse transcriptase polymerase chain reaction (qRT-PCR) to evaluate for major molecular response (MMR).

Let's say Mr. Smith's qRT-PCR results at 12 months show a BCR-ABL1 transcript level of 0.08% on the international scale (IS), indicating he has achieved a major molecular response (MMR). According to the ELN 2022 recommendations, what would be the recommended long-term management strategy at this point, assuming he continues to tolerate nilotinib well?

A) Discontinue nilotinib to attempt treatment-free remission.

B) Continue nilotinib at the current dose and monitor BCR-ABL1 levels every 3 months.

C) Switch to a third-generation TKI to further deepen the molecular response.

D) Reduce the dose of nilotinib to the lowest effective dose to minimize long-term side effects

That's correct, [REDACTED]]. Given that Mr. Smith has achieved MMR at 12 months and is tolerating nilotinib well, the recommended

long-term management strategy according to ELN 2022 is B) Continue nilotinib at the current dose and monitor BCR-ABL1 levels every 3 months.
That's correct, [REDACTED]]. Given that Mr. Smith has achieved MMR at 12 months and is tolerating nilotinib well, the recommended long-term management strategy according to ELN 2022 is B) Continue nilotinib at the current dose and monitor BCR-ABL1 levels every 3 months.
Achieving MMR is a significant positive prognostic indicator. The current standard of care is continued TKI therapy to maintain this response and potentially deepen it further. Regular molecular monitoring is crucial to detect any loss of response, which might necessitate further investigation and treatment adjustments.
Option A), discontinuing nilotinib for treatment-free remission, is generally considered after a more sustained deep molecular response (e.g., MR4 or MR4.5) for a specific duration, and under strict monitoring protocols. It's not typically recommended after just achieving MMR at 12 months.
Option C), switching to a third-generation TKI, is usually reserved for patients who are resistant or intolerant to first- and second-generation TKIs, or in specific clinical trial contexts.
Option D), reducing the dose, might be considered in the context of long-term toxicity, but not routinely in a patient tolerating the medication well and achieving a good molecular response.
Now, let's consider a scenario where, after 3 years of continuous nilotinib therapy and consistent MMR, Mr. Smith expresses interest in potentially stopping treatment. According to the ELN 2022 recommendations, which of the following would be the most important prerequisite to consider him for a trial of treatment-free remission (TFR)?
A) Achievement of complete cytogenetic response for at least 3 years.
B) Achievement of stable major molecular response (MMR) for at least 2 years.
C) Achievement of a sustained deep molecular response (e.g., MR4.5, defined as BCR-ABL1 0.001% IS) for at least 2 years.
D) Absence of any high-risk features at initial diagnosis.

That's absolutely correct, [REDACTED]]. The most important prerequisite to consider Mr. Smith for a trial of treatment-free remission (TFR) according to the ELN 2022 recommendations is C) Achievement of a sustained deep molecular response (e.g., MR4.5, defined as BCR-ABL1 0.001% IS) for at least 2 years.
The ELN guidelines emphasize that patients considered for TFR should have achieved a deep and stable molecular response to minimize the risk of relapse. While the other options might be factors considered in the overall assessment, a sustained deep molecular response is the cornerstone for attempting TFR.
* A) Achievement of complete cytogenetic response for at least 3 years: While CCyR is important, molecular response depth is a stronger predictor of successful TFR.
* B) Achievement of stable major molecular response (MMR) for at least 2 years: MMR is a good response, but a deeper response (MR4 or better, ideally MR4.5) is associated with a higher likelihood of maintaining remission after TKI discontinuation.
* D) Absence of any high-risk features at initial diagnosis: While initial risk might influence the time to achieve deep molecular response, it's not an absolute contraindication for attempting TFR if a sustained deep response is achieved.

This concludes our CML Clinical Case Management session for today, [REDACTED]]. You've demonstrated a strong understanding of the diagnostic workup, initial treatment selection, response monitoring, and long-term management considerations in chronic myeloid leukemia.
Here's a summary of your performance:
Demonstrated Strengths:
* Accurate identification of the initial diagnosis based on clinical and laboratory findings.
* Correct understanding of the key diagnostic test for CML (bone marrow with BCR-ABL1 analysis).
* Good grasp of the importance of ELN risk stratification.
* Understanding the role of second-generation TKIs as first-line therapy.
* Awareness of a significant hematological toxicity (neutropenia) associated with TKIs.
* Knowledge of the key laboratory abnormalities indicative of tumor lysis syndrome.
* Recognition of the importance of molecular response assessment (qRT-PCR) in long-term management.
* Correctly identifying the need for continued TKI therapy after achieving MMR.
* Accurate understanding of the primary prerequisite for considering treatment-free remission (sustained deep molecular response).

Areas for Improvement:
* Refining the specific factors included in the ELN 2022 risk stratification for newly diagnosed chronic phase CML.
* Distinguishing between the routes of administration for different TKIs.
* Precisely recalling the calcium level abnormality in tumor lysis syndrome (hypocalcemia).

Guideline-Based Recommendations for the Case:

Based on ESMO and ELN 2022 guidelines, the management of Mr. Smith has been appropriately guided, including the initial diagnostic workup, first-line TKI selection (nilotinib), monitoring for toxicities like neutropenia and TLS, and the importance of achieving and monitoring molecular response. The criteria for considering treatment-free remission were also correctly identified.
Tailored Learning Resources:
To further enhance your understanding, I recommend reviewing the following:
 * 2022 European LeukemiaNet (ELN) recommendations for the diagnosis and management of chronic myeloid leukemia: Focus on the risk stratification criteria (Sokal, Hasford, EUTOS scores) and the response criteria, including definitions of CCyR, MMR, and deeper molecular responses.
 * ESMO Clinical Practice Guidelines for Chronic Myeloid Leukemia: Pay close attention to the sections on first-line treatment options, including the rationale for choosing between different TKIs based on patient factors and toxicity profiles.
 * Review articles on the management of tumor lysis syndrome: Focus on the pathophysiology and the specific electrolyte abnormalities.
 * Literature on treatment-free remission in CML: Understand the criteria for attempting TFR and the monitoring strategies involved.
Thank you for participating in this interactive case, [REDACTED]]. You demonstrated strong clinical reasoning skills. Please don't hesitate to reach out if you have any further questions.

Session ID:
Assessment Domain

Session-52:
Rationale

Session ID:
Instructional Design and Pedagogical

Session-52:
The session's instructional design is of very high quality. It demonstrates expert scaffolding, moving the learner logically from initial diagnosis and risk stratification through first-line treatment selection, toxicity management, and long-term monitoring, including advanced concepts like treatment-free remission (TFR). The embedded feedback is a major strength, providing clear, guideline-anchored rationales (citing ELN and ESMO) that explain not only the correct answer but also why the distractors are incorrect. Instructions are exceptionally clear, guiding the user through each phase of the case. The only minor weakness is that learning objectives are not explicitly stated at the outset, though they are strongly implied by the session's structure and are effectively summarized in the concluding remarks.

Session ID:
Clinical Authenticity and Content Fidelity

Session-52:
The session demonstrates exceptional clinical fidelity and content accuracy. Plausibility & Guideline Alignment: The clinical narrative is indistinguishable from an authentic patient case for Chronic Myeloid Leukemia (CML). All diagnostic and therapeutic decision points, from the initial workup to TFR criteria, are perfectly aligned with current, evidence-based guidelines, specifically citing the 2022 ELN and ESMO recommendations. Pharmacotherapeutic Accuracy: The therapeutic choices presented are optimal and well-justified. Recommending a second-generation TKI for an intermediate-risk patient, monitoring for neutropenia, and managing based on molecular response are all standards of care.
Domain Specificity: The session maintains perfect domain specificity. All concepts, diagnostics, and therapies are strictly relevant to CML. There are no instances of domain entanglement where standards of care from other hematologic malignancies (e.g., AML, ALL) are incorrectly applied. This absence of critical error ensures the simulation is educationally safe and effective.

Session ID:
Modeled Clinical Reasoning

Session-52:
The simulation expertly models a high-level clinical reasoning process. Each stage of the case frames a clear and relevant clinical problem, providing the necessary data for an informed decision. The multiple-choice options are well-crafted, presenting a strong

breadth of plausible alternatives and key distractors that effectively test for nuanced understanding (e.g., differentiating TKI toxicity profiles). The narrative demonstrates flawless prioritization, moving from diagnosis to risk assessment to therapy selection and monitoring in a logical, real-world sequence. The clinical outcomes described are realistic and instructionally valuable, reinforcing the learning objectives at each step.

Session-53

Session ID:
LLM

Session-53:
Google AI Studio (Gemini 2.0 Pro)

Session ID:
Session Transcript

Session-53:
Okay, let's begin with Phase 1 of the case.
Phase 1: Initial Presentation and Diagnostic Workup
A 58-year-old male presents to his primary care physician with complaints of fatigue, night sweats, and unintentional weight loss of approximately 5 kg over the past 3 months. He denies any fever, bone pain, or bleeding. His past medical history is significant for well-controlled hypertension and hyperlipidemia.
Initial laboratory investigations reveal the following:
* White blood cell (WBC) count: 155 x 10^9/L (reference range: 4-10 x 10^9/L)
* Hemoglobin: 10.5 g/dL (reference range: 13.5-17.5 g/dL)
* Platelet count: 480 x 10^9/L (reference range: 150-400 x 10^9/L)
* Peripheral blood smear shows a left shift with immature granulocytes, including blasts (<5%), promyelocytes, myelocytes, and metamyelocytes. Basophils are noted to be increased.

Based on this initial presentation and laboratory findings, which of the following is the most likely initial diagnosis?
A) Acute Myeloid Leukemia (AML)
B) Chronic Lymphocytic Leukemia (CLL)
C) Chronic Myeloid Leukemia (CML)
D) Myelodysplastic Syndrome (MDS)

Please give me a hint
Consider the hallmark of CML: a significantly elevated white blood cell count with a characteristic differential including immature granulocytes at various stages of maturation, but with a relatively low percentage of blasts in the peripheral blood. Also, think about the typical symptoms reported by patients with CML in the chronic phase. Which of the options aligns best with these features?

Most likely diagnosis: C) Chronic Myeloid
Leukemia (CML)

Reason:

Very high WBC count with a left shift (immature granulocytes).

Increased basophils.

Mild anemia and thrombocytosis.
That is correct, [REDACTED]]. C) Chronic Myeloid Leukemia (CML) is the most likely initial diagnosis based on the presented information.
Your reasoning is also accurate:
* The significantly elevated WBC count with a "left shift" (presence of immature granulocytes) is a key characteristic of CML.
* Increased basophils are commonly observed in CML.

* The presence of mild anemia and thrombocytosis can also be seen in CML, although the WBC count is the most striking finding at this stage.
Now, let's move on to the next step in the diagnostic workup. Following the initial peripheral blood findings, what is the next essential diagnostic test to confirm the diagnosis of CML according to current guidelines?
A) Bone marrow biopsy with cytogenetic analysis for the Philadelphia chromosome (BCR-ABL1 translocation).
B) Flow cytometry of the peripheral blood to assess for lymphoid markers.
C) Liver function tests and a coagulation panel to evaluate for organ involvement.
D) Serum lactate dehydrogenase (LDH) and uric acid levels to assess for tumor burden.

That is correct, [REDACTED]]. C) Chronic Myeloid Leukemia (CML) is the most likely initial diagnosis based on the presented information.
Your reasoning is also accurate:
* The significantly elevated WBC count with a "left shift" (presence of immature granulocytes) is a key characteristic of CML.
* Increased basophils are commonly observed in CML.
* The presence of mild anemia and thrombocytosis can also be seen in CML, although the WBC count is the most striking finding at this stage.
Now, let's move on to the next step in the diagnostic workup. Following the initial peripheral blood findings, what is the next essential diagnostic test to confirm the diagnosis of CML according to current guidelines?
A) Bone marrow biopsy with cytogenetic analysis for the Philadelphia chromosome (BCR-ABL1 translocation).
B) Flow cytometry of the peripheral blood to assess for lymphoid markers.
C) Liver function tests and a coagulation panel to evaluate for organ involvement.
D) Serum lactate dehydrogenase (LDH) and uric acid levels to assess for tumor burden.

Correct answer: A) Bone marrow biopsy with cytogenetic analysis for the Philadelphia chromosome (BCR-ABL1 translocation).

Reason:

Confirming CML requires detection of the Philadelphia chromosome
That's absolutely right, [REDACTED]]! A) Bone marrow biopsy with cytogenetic analysis for the Philadelphia chromosome (BCR-ABL1 translocation) is the next essential diagnostic test.
Your reasoning is spot on. The presence of the Philadelphia chromosome, resulting from the reciprocal translocation t(9;22)(q34;q11.2) leading to the BCR-ABL1 fusion gene, is the cytogenetic hallmark and a crucial diagnostic criterion for CML according to the WHO classification and ESMO guidelines.
Now, let's assume the bone marrow biopsy and cytogenetic analysis confirm the presence of the BCR-ABL1 translocation. Further molecular analysis using quantitative reverse transcription polymerase chain reaction (qRT-PCR) is performed on the bone marrow aspirate to quantify the BCR-ABL1 transcript level. The result shows a BCR-ABL1 transcript level of 95% on the International Scale (IS).
Based on the European LeukemiaNet (ELN) 2022 risk stratification for newly diagnosed chronic phase CML, how would you classify this patient's risk?
A) Low risk
B) Intermediate risk
C) High risk
D) Not applicable as risk stratification is only for advanced phase CML.

The answer is high risk
Not quite, [REDACTED]]. Let's look at the ELN 2022 criteria again.
* Low risk: BCR-ABL1 \leq 10% IS
* Intermediate risk: BCR-ABL1 > 10% to \leq 100% IS
* High risk: Sokal risk score \geq 0.8 OR Hasford score \geq 7.8 OR EUTOS score \geq 0.1 OR BCR-ABL1 > 100% IS
While a BCR-ABL1 level of 95% IS is elevated, it falls within the range defined for intermediate risk by the ELN 2022 criteria based solely on the initial BCR-ABL1 transcript level. The high-risk category based on BCR-ABL1 alone is defined as > 100% IS. Other scoring systems like Sokal, Hasford, or EUTOS can also classify patients as high risk, but based solely on the provided BCR-ABL1 level, the classification is intermediate.
Therefore, the correct answer is B) Intermediate risk.

Understanding the risk stratification is crucial as it helps guide initial treatment decisions and monitor response.
We have now completed Phase 1 of the case. Are you ready to move on to Phase 2, where we will discuss induction therapy selection and supportive care planning? Type 'YES' to proceed.

Great, let's move on to Phase 2: Induction Therapy Selection and Supportive Care Planning.
Based on the diagnosis of chronic phase CML and the intermediate risk stratification according to ELN 2022, what is the recommended first-line treatment for this patient, considering his age of 58 years and no significant comorbidities other than well-controlled hypertension and hyperlipidemia?
A) Observation with close monitoring of blood counts.
B) Interferon-alpha therapy.
C) A second-generation tyrosine kinase inhibitor (TKI) such as nilotinib or dasatinib.
D) Hydroxyurea to rapidly reduce the white blood cell count followed by allogeneic hematopoietic stem cell transplantation.

Answer: C) Second-gen TKI (nilotinib or dasatinib). Because Best for chronic phase CML, intermediate
Okay, let's refine the answer slightly to be more precise based on current ESMO guidelines. While both nilotinib and dasatinib are excellent first-line options, the guidelines often consider factors like the potential for faster and deeper molecular responses when making an initial selection, especially in patients without significant comorbidities.
Given this, which of the second-generation TKIs might be slightly favored in the first-line setting for a patient like this to achieve these deeper and faster responses?
A) Nilotinib
B) Dasatinib
C) Either nilotinib or dasatinib are equally preferred.
D) The choice depends solely on the patient's insurance coverage.

Answer: A) Nilotinib.

Because
Nilotinib is slightly favored for achieving faster and deeper molecular responses in first-line CML treatment, according to ESMO guidelines.
That's a very insightful and accurate refinement, [REDACTED]]! A) Nilotinib is often favored in the first-line setting for many patients with chronic phase CML, particularly when the goal is to achieve a rapid and deep molecular response, as supported by clinical trial data and ESMO guidelines. Dasatinib is also an excellent option, especially in certain situations (e.g., higher risk features or intolerance to other TKIs).
Your understanding of the nuances in first-line TKI selection is commendable.
Now, let's consider the supportive care aspects. This patient is about to start nilotinib. What is an important counseling point regarding the administration of nilotinib that you should emphasize to the patient to optimize its efficacy and minimize potential risks?
A) Nilotinib should be taken with a high-fat meal to enhance absorption.
B) Nilotinib should be taken on an empty stomach, at least 2 hours before or 1 hour after food.
C) Nilotinib can be taken at any time, regardless of food intake.
D) The timing of nilotinib administration relative to food is not clinically significant.

Give me hint please
Think about how food intake can affect the absorption of certain medications. Consider the pharmacokinetic properties of nilotinib and whether food might increase or decrease its bioavailability, potentially impacting its effectiveness. Referencing the product labeling or relevant guidelines might provide a clue.

Answer: B Nilotinib should be taken on an empty stomach, at least 2 hours before or 1 hour after food.

Because
Food increases nilotinib absorption, which raises the risk of toxicity so it must be taken

You are absolutely correct, [REDACTED]]! B) Nilotinib should be taken on an empty stomach, at least 2 hours before or 1 hour after food.
Your reasoning is also accurate. Food significantly increases the bioavailability of nilotinib, which can lead to higher drug

concentrations and potentially increase the risk of adverse effects, particularly cardiovascular events. Therefore, emphasizing the importance of taking nilotinib on an empty stomach is crucial for patient safety and optimal treatment.
Now, let's consider potential complications. During the first month of nilotinib therapy, the patient develops a fever of 38.5C and his absolute neutrophil count (ANC) drops to 0.8 x 10^9/L. He denies any localized source of infection. According to ASCO guidelines for managing febrile neutropenia in adult cancer patients, what is the most appropriate initial management?
A) Initiate oral broad-spectrum antibiotics as an outpatient.
B) Initiate intravenous broad-spectrum antibiotics after blood cultures are drawn and admit the patient to the hospital.
C) Administer granulocyte colony-stimulating factor (G-CSF) as an outpatient and monitor closely.
D) Discontinue nilotinib and monitor the patient's blood counts.

May be
Answer is C
Could you elaborate on your reasoning for choosing C), [REDACTED]]? What factors are making you consider outpatient G-CSF administration in this setting of febrile neutropenia?

Reason:
Fever with neutropenia is a medical emergency. IV antibiotics are required, especially when no localized infection source is identified. Hospital admission is necessary for close monitoring.
You've correctly identified the critical aspect of febrile neutropenia being a medical emergency, [REDACTED]]. However, your chosen answer was C, which involves outpatient G-CSF.
Let's revisit the ASCO guidelines for managing febrile neutropenia. The presence of fever (38.3C or higher for more than one hour, or a single temperature of 38.5C) in a patient with neutropenia (ANC < 1.0 x 10^9/L, or expected to decrease to that level) is a significant concern for infection.
Given the fever and the low ANC without a clear source of infection, which of the management options prioritizes rapid initiation of broad-spectrum antibiotics and close monitoring for potential complications in a hospital setting?

Answer: B Initiate intravenous broad-spectrum antibiotics after blood cultures are drawn and admit the patient to the hospital.

Because
In febrile neutropenia, especially with ANC < 1.0 x 10^9/L and no clear source of infection, IV broad-spectrum antibiotics are necessary immediately to prevent serious infections. Hospital admission is required for observation and to manage
That's precisely right, [REDACTED]]! B) Initiate intravenous broad-spectrum antibiotics after blood cultures are drawn and admit the patient to the hospital is the most appropriate initial management according to ASCO guidelines.
Your reasoning is excellent:
 * Febrile neutropenia is a medical emergency requiring prompt and aggressive management.
 * Intravenous broad-spectrum antibiotics should be initiated as soon as possible after obtaining blood cultures to cover potential bacterial infections, especially when no localized source is identified.
 * Hospital admission is crucial for close monitoring of the patient's clinical status, response to antibiotics, and to manage any potential complications that may arise.
While G-CSF (option C) can be used in certain high-risk neutropenic fever cases, the immediate priority is to treat potential infections with antibiotics. Outpatient management (option A) is generally not appropriate for a first episode of febrile neutropenia, especially with a low ANC. Simply discontinuing nilotinib (option D) without addressing the potential infection is also incorrect.

Great, let's proceed to Phase 3: Post-Remission Therapy, MRD Monitoring, and Long-Term Management.
Following the initial management of febrile neutropenia, the patient recovers well with antibiotic therapy, and his ANC normalizes. He continues on nilotinib. After 3 months of nilotinib therapy, a bone marrow aspirate is performed to assess his cytogenetic response. Cytogenetic analysis reveals a complete cytogenetic response (CCyR), meaning no Philadelphia chromosome-positive metaphases are detected.
According to current ELN recommendations, what is the next step in monitoring this patient's response to therapy?
A) Continue cytogenetic monitoring every 6 months.
B) Initiate monitoring of BCR-ABL1 transcript levels using quantitative reverse transcription polymerase chain reaction (qRT-PCR) on peripheral blood every 3 months.
C) Perform another bone marrow biopsy for cytogenetic analysis in 3 months to confirm CCyR.
D) No further monitoring is needed as the patient has achieved CCyR.

Please I want a hint

Think about the depth of response that is the ultimate goal in CML management. While CCyR is a significant milestone, what more sensitive method can be used to assess the level of residual disease and predict long-term outcomes? Consider the role of molecular monitoring in contemporary CML management guidelines.

Answer: B) Monitor BCR-ABL1 levels by qRT-PCR every 3 months.

Reason:

To assess molecular response and detect MRD after achieving

That's absolutely correct, [REDACTED]]! B) Initiate monitoring of BCR-ABL1 transcript levels using quantitative reverse transcription polymerase chain reaction (qRT-PCR) on peripheral blood every 3 months is the recommended next step.

That's absolutely correct, [REDACTED]]! B) Initiate monitoring of BCR-ABL1 transcript levels using quantitative reverse transcription polymerase chain reaction (qRT-PCR) on peripheral blood every 3 months is the recommended next step.

Your reasoning is spot on. While achieving a CCyR is a significant achievement, monitoring the BCR-ABL1 transcript levels using qRT-PCR is crucial for assessing the depth of molecular response and detecting minimal residual disease (MRD). Molecular monitoring is more sensitive than cytogenetic analysis and provides important prognostic information, guiding long-term management decisions according to ELN and ESMO guidelines.

Now, let's say after 12 months of continuous nilotinib therapy, the patient's BCR-ABL1 transcript level on the International Scale (IS) is consistently around 0.05%. According to the ELN 2022 response criteria, what level of molecular response has this patient achieved?

A) Major Molecular Response (MMR)

B) Deep Molecular Response (DMR) with a 4-log reduction (MR4)

C) DMR with a 4.5-log reduction (MR4.5)

D) DMR with a 5-log reduction (MR5)

Answer: B) Deep Molecular Response (DMR) with a 4-log reduction (MR4).

Reason:

An IS of 0.05% corresponds to a 4-log reduction in BCR-ABL1 transcript levels, which is classified as MR4 (Deep

---

Session ID:

Assessment Domain

Session-53:

Rationale

Session ID:

Instructional Design and Pedagogical

Session-53:

The session's instructional design is a significant strength. The case is expertly scaffolded, moving logically from initial diagnosis (CML) and risk stratification to first-line therapy selection, management of complications (febrile neutropenia), and long-term monitoring. The embedded feedback is clear, evidence-based, and pedagogically sound, particularly in how it guides the user toward the correct management of febrile neutropenia. Instructions are unambiguous. Learning objectives, while not explicitly stated at the outset, are clearly implied by the case's well-designed structure.

Session ID:

Clinical Authenticity and Content Fidelity

Session-53:

The clinical narrative is highly plausible and feels like an authentic CML case. However, its overall fidelity is compromised by a significant content error.

Guideline Alignment & Pharmacotherapeutic Accuracy: The session deviates from guidelines in its final question. It incorrectly classifies a BCR-ABL1 transcript level of 0.05% on the International Scale (IS) as a "Deep Molecular Response (DMR) with a 4-log

reduction (MR4)". Per ELN 2022 criteria, MR4 requires a level of 0.01% IS. A level of 0.05% is correctly defined as a Major Molecular Response (MMR). This is a suboptimal and inaccurate pharmacotherapeutic assessment, misrepresenting the patient's true depth of response, which is a critical monitoring parameter in CML management.
Domain Specificity: The case remains entirely within the CML disease domain, with no evidence of domain entanglement.

Session ID:
Modeled Clinical Reasoning

Session-53:
The simulation provides an excellent model of the clinical reasoning process. Each stage presents a clearly framed clinical problem with relevant data. The multiple-choice options are well-constructed, offering plausible and relevant distractors that test for nuanced understanding. The overall decision-making strategy is sound, correctly prioritizing diagnostic confirmation, risk stratification before therapy, and aggressive management of complications. The clinical outcomes are realistic and instructionally valuable. The reasoning process modeled by the simulation is excellent, even though it led to a factually incorrect conclusion in the final question (which is scored as a content error in Section 2).

Session-54

Session ID:
LLM

Session-54:
Google AI Studio (Gemini 2.0 Pro)

Session ID:
Session Transcript

Session-54:
Phase 1: Initial Presentation and Diagnostic Workup
Patient Case: R.M. is a 52-year-old male presenting to his primary care physician with complaints of increasing fatigue, early satiety, and unintentional weight loss of 10 lbs over the past 3 months. He denies fever, chills, or night sweats.
Past Medical History: Hypertension, well-controlled on lisinopril 10 mg daily.
Social History: Accountant, married, denies tobacco/illicit drug use, occasional alcohol.
Physical Exam: Vital signs are stable. Abdominal exam reveals splenomegaly, palpable 5 cm below the left costal margin. No lymphadenopathy noted.
Initial Laboratory Results:
WBC: 155,000/L (Differential: Neutrophils 65%, Bands 5%, Metamyelocytes 8%, Myelocytes 10%, Promyelocytes 2%, Blasts 2%, Basophils 5%, Eosinophils 3%, Lymphocytes 5%, Monocytes 5%)
Hemoglobin: 10.5 g/dL
Platelets: 650,000/L
Sodium: 138 mEq/L
Potassium: 4.1 mEq/L
BUN: 15 mg/dL
Creatinine: 0.9 mg/dL
Question 1: Based on R.M.'s presentation and initial lab findings, which of the following is the MOST appropriate next step to confirm the suspected diagnosis?
A) Repeat CBC in 1 month to monitor trends.
B) Peripheral blood smear review and BCR-ABL1 testing (RT-qPCR and/or FISH) from peripheral blood.
C) Initiate empiric hydroxyurea immediately for cytoreduction before definitive diagnosis.
D) Schedule an urgent PET/CT scan to assess for other potential malignancies.

That's correct, [REDACTED]]. Option B is the most appropriate next step.
Feedback:
Rationale for Correct Answer (B): The patient's presentation (fatigue, weight loss, splenomegaly) combined with the marked leukocytosis, thrombocytosis, and presence of immature myeloid cells (left shift) on the CBC differential is highly suspicious for Chronic Myeloid Leukemia (CML). A peripheral blood smear review will confirm the morphologic findings. Crucially, CML is defined by the presence of the Philadelphia chromosome (Ph) or its molecular equivalent, the BCR-ABL1 fusion gene. Therefore, testing for BCR-ABL1 via RT-qPCR (quantitative reverse transcriptase polymerase chain reaction) or FISH (fluorescence in situ hybridization) on peripheral blood is essential for establishing the diagnosis, as per WHO 2016/2022 criteria and strongly recommended by ESMO guidelines (Hochhaus A, et al. Ann Oncol. 2020;31(11):1451-1465. Section 3: Diagnosis).
Rationale for Incorrect Answers:
A: Delaying diagnosis by repeating the CBC is inappropriate given the high suspicion for malignancy and the patient's symptoms.
C: While cytoreduction with hydroxyurea might be considered after diagnosis or for symptomatic hyperleukocytosis, initiating it before confirming the specific diagnosis and phase of CML is generally not recommended. Definitive therapy with a Tyrosine Kinase Inhibitor (TKI) is the standard of care.
D: A PET/CT scan is not a primary diagnostic tool for CML. Its utility is more relevant in assessing other malignancies or potentially looking for extramedullary disease or transformation in CML, but not for the initial diagnosis.
The peripheral blood smear confirms the findings suggestive of CML in the chronic phase (CML-CP), showing marked granulocytosis with a spectrum of myeloid maturation and <5% blasts. Peripheral blood FISH analysis detects the t(9;22)(q34;q11.2) translocation,

and quantitative RT-qPCR confirms the presence of the BCR-ABL1 fusion transcript (p210 isoform).
A bone marrow aspirate and biopsy are performed.
Bone Marrow Findings: Hypercellular marrow (95% cellularity) with marked myeloid hyperplasia, megakaryocyte proliferation, <5% blasts.
Cytogenetics (Karyotype): 46,XY,t(9;22)(q34;q11.2)[20/20] - indicating the Philadelphia chromosome is present in all metaphases analyzed, with no additional chromosomal abnormalities (ACA).
Question 2: Based on the available diagnostic information (including CBC, bone marrow, cytogenetics, and molecular results), how would you classify R.M.'s CML phase and calculate his Sokal risk score? (You'll need to estimate spleen size impact - assume 5cm below costal margin corresponds to moderate enlargement for calculation purposes).
Sokal Score Calculation Reminder: Score = exp[0.0116 (age in years - 43.4) + 0.0345 (spleen size cm below costal margin - 7.51) + 0.188 [(platelet count/700) - 0.563] + 0.0887 (% blasts in blood - 2.10)]
A) Chronic Phase (CML-CP), Sokal Score: Low Risk
B) Accelerated Phase (CML-AP), Sokal Score: Intermediate Risk
C) Chronic Phase (CML-CP), Sokal Score: Intermediate Risk
D) Blast Phase (CML-BP), Sokal Score: High Risk
Excellent, [REDACTED]]. Your selection of C) Chronic Phase (CML-CP), Sokal Score: Intermediate Risk is correct.
Feedback:
Rationale for Correct Answer (C):
Phase: The patient's blast percentage in both peripheral blood (2%) and bone marrow (<5%) is well below the <15% threshold for Chronic Phase (CML-CP) according to WHO 2016/2022 and ELN 2020 criteria. The basophil percentage (5%) is also below the <20% threshold for CML-CP. Therefore, the patient is correctly identified as being in the Chronic Phase.
Sokal Score: Let's calculate using the provided formula and patient data:
exp[0.0116 * (52 - 43.4) + 0.0345 * (5 - 7.51) + 0.188 * [(650/700) - 0.563] + 0.0887 * (2 - 2.10)]
exp[0.0116 * 8.6 + 0.0345 * (-2.51) + 0.188 * [(0.9286) - 0.563] + 0.0887 * (-0.10)]
exp[0.09976 - 0.0866 + 0.188 * [0.8623 - 0.563] - 0.00887]
exp[0.09976 - 0.0866 + 0.188 * 0.2993 - 0.00887]
exp[0.09976 - 0.0866 + 0.05627 - 0.00887]
exp[0.06056]
Sokal Score 1.06
Comparing this score to the Sokal risk categories (Low: <0.8, Intermediate: 0.8-1.2, High: >1.2), a score of 1.06 falls into the Intermediate Risk category.
Rationale for Incorrect Answers:
A: While the phase is correct, the calculated Sokal score is Intermediate, not Low.
B: The patient is in Chronic Phase, not Accelerated Phase, based on blast and basophil counts.
D: The patient is in Chronic Phase, not Blast Phase, and the Sokal score is Intermediate, not High.
R.M. is diagnosed with Chronic Phase CML (CML-CP), Intermediate Risk by Sokal score. He has no known cardiac history beyond controlled hypertension and his baseline ECG is unremarkable. He expresses a desire for an oral, once-daily medication if possible.
Question 3: According to ESMO/ELN guidelines, which of the following first-line Tyrosine Kinase Inhibitor (TKI) options would be MOST appropriate for R.M., considering his diagnosis, risk score, comorbidities, and preferences?
A) Imatinib 400 mg once daily
B) Dasatinib 100 mg once daily
C) Hydroxyurea 1000 mg twice daily
D) Interferon-alfa (pegylated) weekly injections
That's a well-reasoned choice, [REDACTED]]. Option B) Dasatinib 100 mg once daily is a very appropriate first-line TKI for R.M.
Feedback:
Rationale for Correct Answer (B): Dasatinib is a second-generation TKI approved and recommended by ESMO/ELN guidelines for the first-line treatment of CML-CP across all risk groups (Hochhaus A, et al. Ann Oncol. 2020;31(11):1451-1465. Section 4: Treatment of CML-CP). It offers the potential for faster and deeper molecular responses compared to imatinib, which can be particularly relevant for intermediate-risk patients. It aligns perfectly with the patient's preference for once-daily dosing. While dasatinib carries a risk of pleural effusion, the patient's controlled hypertension does not represent a specific contraindication.
Rationale for Incorrect Answers:
A) Imatinib 400 mg once daily: While Imatinib is also a standard and effective first-line option recommended by guidelines and fits the once-daily preference, some guidelines or clinicians might lean towards a second-generation TKI like dasatinib for intermediate or high-risk patients due to potentially faster/deeper responses. However, imatinib remains a perfectly valid alternative. In this context,

dasatinib might be considered most appropriate given the intermediate risk, but imatinib is not inherently wrong.
C) Hydroxyurea 1000 mg twice daily: Hydroxyurea is a cytoreductive agent, useful for rapidly lowering very high white blood cell counts initially, but it is not a targeted therapy for CML and does not induce the molecular remissions necessary for long-term control. It is not a standard first-line definitive therapy.
D) Interferon-alfa (pegylated) weekly injections: Interferon was used historically but has been largely replaced by TKIs due to better efficacy and tolerability profiles. It is not a standard first-line choice in the TKI era, except in specific circumstances (e.g., pregnancy planning where TKIs are contraindicated).
Phase 2: R.M. is initiated on Dasatinib 100 mg once daily. You counsel him extensively on adherence, potential side effects (including rash, fluid retention/pleural effusion, myelosuppression, potential QTc prolongation although less common than nilotinib), and the importance of regular monitoring.
Baseline labs before starting Dasatinib were as reviewed previously. Supportive care considerations are being planned.
Question 4: Which of the following baseline assessments and initial supportive care measures are MOST critical for R.M. as he starts Dasatinib?
A) Echocardiogram, Pulmonary Function Tests (PFTs), and initiation of allopurinol for tumor lysis syndrome (TLS) prophylaxis.
B) HLA typing for potential stem cell transplant, Hepatitis B/C screening, and prophylactic acyclovir.
C) Chest X-ray (CXR), Hepatitis B/C screening, screening for QTc interval on ECG, and counseling on avoiding proton pump inhibitors (PPIs).
D) Bone density scan, serum viscosity testing, and initiation of prophylactic G-CSF.
Correct, [REDACTED]]! Option C) Chest X-ray (CXR), Hepatitis B/C screening, screening for QTc interval on ECG, and counseling on avoiding proton pump inhibitors (PPIs) represents the MOST critical set of baseline assessments and initial supportive care/counseling measures for R.M. starting Dasatinib.
Feedback:
Rationale for Correct Answer (C):
CXR: Establishing a baseline chest X-ray is important due to the known risk of pleural effusion associated with Dasatinib.
Hepatitis B/C Screening: Screening for chronic viral hepatitis (HBV, HCV) is standard practice before initiating potentially immunosuppressive or long-term cancer therapies, including TKIs, due to the risk of reactivation (particularly HBV). (ESMO Guidelines reference general oncology principles).
ECG/QTc Screening: While the risk is lower than with nilotinib, dasatinib can prolong the QTc interval. A baseline ECG is recommended to assess the QTc and monitor periodically, especially in patients with other risk factors.
PPI Counseling: Dasatinib absorption is significantly reduced by agents that increase gastric pH. Patients must be counseled to avoid PPIs. If acid suppression is needed, H2 blockers or antacids may be used with careful timing separation (e.g., take dasatinib 2 hours before or 2 hours after an H2 blocker; separate antacids by 2 hours). This is a critical counseling point to ensure therapeutic drug levels.
Rationale for Incorrect Answers:
A: Routine baseline PFTs are not typically required. An echocardiogram might be considered depending on other risk factors but isn't universally mandated for dasatinib initiation in a patient with only controlled hypertension. Allopurinol for TLS prophylaxis is not standard for CML-CP unless the WBC count is extremely high (e.g., >200-300k) or there are other specific risk factors; R.M.'s WBC of 155k doesn't automatically warrant it, although hydration is important.
B: HLA typing is only relevant if an allogeneic stem cell transplant is being actively considered, which is not standard first-line therapy for CML-CP. Prophylactic acyclovir is not routinely indicated unless there's a history of recurrent HSV/VZV or other specific risks.
D: A baseline bone density scan is not a standard immediate requirement. Serum viscosity testing is only indicated for symptomatic hyperleukocytosis. Prophylactic G-CSF is not standard at initiation and is used only if significant neutropenia develops during treatment.
R.M. tolerates Dasatinib 100 mg daily reasonably well initially. Baseline CXR, ECG, and Hepatitis screening are unremarkable. He is counseled regarding the PPI interaction.
At the 1-month follow-up:
Symptoms: Fatigue significantly improved, no early satiety.
Exam: Spleen is no longer palpable.
Labs:
WBC: 12,500/L (Differential normalizing, Blasts 0%, Basophils 1%)
Hemoglobin: 11.0 g/dL
Platelets: 350,000/L
Creatinine: 1.0 mg/dL
ALT/AST: Mildly elevated at 1.5x Upper Limit of Normal (ULN)

Question 5: R.M. calls 2 weeks later (6 weeks into therapy) reporting Grade 2 fatigue and a new non-productive cough with mild shortness of breath on exertion for the past 3 days. His vital signs are stable. What is the MOST appropriate next step?
A) Reassure the patient that these are common side effects and continue Dasatinib at the current dose.
B) Immediately stop Dasatinib and switch to Imatinib.
C) Obtain a chest X-ray and temporarily hold Dasatinib pending results.
D) Increase the Dasatinib dose to 140 mg daily to improve efficacy.
Excellent choice, [REDACTED]]. Option C) Obtain a chest X-ray and temporarily hold Dasatinib pending results is the most appropriate and safest next step.
Feedback:
Rationale for Correct Answer (C):
Pleural Effusion Risk: Dasatinib carries a known risk of pleural effusion, which can manifest as cough, shortness of breath, and fatigue. These new symptoms in R.M., occurring 6 weeks into therapy, are highly suspicious for this adverse event.
Diagnostic Step: A chest X-ray (CXR) is the primary initial imaging modality to evaluate for pleural effusion.
Management Principle: When a potentially significant adverse event like symptomatic pleural effusion is suspected with Dasatinib, the standard recommendation is to interrupt (hold) the drug immediately pending investigation and confirmation. Continuing the drug could worsen the effusion. (Refer to Dasatinib Package Insert and ESMO guidelines section on toxicity management).
Rationale for Incorrect Answers:
A: While fatigue can be a common side effect, the new onset of cough and shortness of breath should not be dismissed, especially given the known risk of pleural effusion with Dasatinib. Investigation is warranted.
B: Stopping Dasatinib permanently and switching TKI without confirming the cause and severity of the symptoms is premature. If a mild effusion is found and resolves with temporary interruption/management, Dasatinib might be cautiously resumed, potentially at a lower dose.
D: Increasing the Dasatinib dose is contraindicated when managing a potential adverse event like pleural effusion. Dose escalation is considered for inadequate response, not toxicity.
You instruct R.M. to hold his Dasatinib and obtain a stat CXR. The CXR reveals small bilateral pleural effusions.
Question 6: According to typical management strategies for Dasatinib-induced pleural effusion (Grade 2 severity - symptomatic, interfering with instrumental ADLs), what is the recommended approach now?
A) Permanently discontinue Dasatinib and switch to Nilotinib immediately.
B) Continue holding Dasatinib, initiate a short course of oral corticosteroids (e.g., prednisone), and re-evaluate with CXR in 1 week.
C) Restart Dasatinib at the same dose (100 mg daily) and add a daily diuretic (e.g., furosemide).
D) Continue holding Dasatinib, monitor symptoms, consider diuretics if symptoms persist, and plan to restart Dasatinib at a reduced dose (e.g., 80 mg or 50 mg daily) once effusions resolve or stabilize.
Excellent, [REDACTED]]. Option D) Continue holding Dasatinib, monitor symptoms, consider diuretics if symptoms persist, and plan to restart Dasatinib at a reduced dose (e.g., 80 mg or 50 mg daily) once effusions resolve or stabilize is the most appropriate management strategy.
Feedback:
Rationale for Correct Answer (D): This approach follows the standard recommendations for managing Grade 2 Dasatinib-induced pleural effusion (as per prescribing information and clinical guidelines like ESMO):
Interrupt Therapy: Continue holding Dasatinib.
Symptomatic Management: Monitor the patient. Diuretics can be considered if the effusion is causing persistent symptoms or isn't resolving quickly with drug interruption alone.
Dose Reduction: The crucial step upon resolution or stabilization to Grade 1 is restarting Dasatinib at a reduced dose (e.g., reducing from 100 mg to 80 mg, or even 50 mg, depending on the severity and speed of resolution) to decrease the likelihood of recurrence.
Rationale for Incorrect Answers:
A: Permanent discontinuation and switching TKIs is generally reserved for Grade 3/4 effusions, recurrent Grade 2 effusions despite dose reduction, or if the patient/physician prefer not to re-challenge. It's too aggressive for an initial Grade 2 event.
B: While corticosteroids can be used for management, they are typically reserved for more severe (Grade 3/4) or refractory effusions, or those suspected to have an inflammatory component. They are not usually the first step for Grade 2. This option also doesn't address the need for dose reduction upon restart.
C: Restarting at the same dose that caused the effusion (100 mg) significantly increases the risk of recurrence and is contrary to standard management principles. Adding a diuretic prophylactically while restarting at the full dose is not the recommended approach.
R.M. holds Dasatinib. His cough and shortness of breath improve within 5 days. A repeat CXR after 1 week shows resolution of the pleural effusions. After discussion, you decide to restart Dasatinib at a reduced dose of 80 mg once daily. He is monitored closely.
3-Month Follow-up:

Tolerability: Tolerating Dasatinib 80 mg daily well, no recurrence of pulmonary symptoms. Mild fatigue persists but is manageable.
Labs:
WBC: 5.2 k/L (Normal differential)
Hgb: 12.8 g/dL
Plt: 210 k/L
ALT/AST: WNL
Molecular Monitoring (Peripheral Blood RT-qPCR): BCR-ABL1 transcript level is 0.5% (IS - International Scale).
Question 7: According to ESMO/ELN 2020 response criteria, how would you classify R.M.'s response at 3 months based on the BCR-ABL1 transcript level?
A) Optimal Response
B) Warning
C) Failure
D) Complete Molecular Response (CMR)
Correct, [REDACTED]]! Option A) Optimal Response is the accurate classification of R.M.'s response at 3 months.
Feedback:
Rationale for Correct Answer (A): The ESMO/ELN 2020 guidelines define Optimal Response at 3 months as achieving a BCR-ABL1 transcript level of 10% on the International Scale (IS). R.M.'s result of 0.5% (IS) is well below this threshold, indicating an excellent early molecular response to Dasatinib therapy (even at the slightly reduced dose). (Hochhaus A, et al. Ann Oncol. 2020;31(11):1451-1465. Table 3 & Section 5: Monitoring).
Rationale for Incorrect Answers:
B) Warning: A "Warning" at 3 months would be defined if the BCR-ABL1 (IS) level was >10%.
C) Failure: "Failure" at 3 months typically involves not achieving complete hematologic response (CHR) or having certain cytogenetic abnormalities, or sometimes very high transcript levels (though the primary threshold is 10%). 0.5% is clearly not failure.
D) Complete Molecular Response (CMR): CMR, often defined as MR4 (0.01% IS) or MR4.5 (0.0032% IS) or undetectable transcripts depending on assay sensitivity, represents a much deeper level of response than 0.5%. While 0.5% is a good response, it's not CMR. It represents an Early Molecular Response (EMR) and specifically a Major Molecular Response (MMR) has not yet been achieved (MMR is 0.1% IS).
Phase 3: R.M. continues on Dasatinib 80 mg daily. He undergoes regular monitoring, including CBC and BCR-ABL1 RT-qPCR every 3 months.
Monitoring Results:
6 Months: BCR-ABL1 = 0.08% (IS)
12 Months: BCR-ABL1 = 0.05% (IS)
18 Months: BCR-ABL1 = 0.04% (IS)
24 Months: BCR-ABL1 = 0.009% (IS)
36 Months: BCR-ABL1 = 0.005% (IS)
He has tolerated the therapy well with only Grade 1 fatigue and occasional mild edema managed conservatively.
Question 8: Based on the molecular monitoring results from 6 months onwards, how would you characterize R.M.'s long-term molecular response according to ESMO/ELN criteria?
A) Consistent Major Molecular Response (MMR) but failure to achieve Deep Molecular Response (DMR).
B) Stable Optimal Response, achieving and maintaining Major Molecular Response (MMR) and Deep Molecular Response (DMR).
C) Warning response after 12 months due to fluctuating transcript levels.
D) Molecular Failure requiring a switch in TKI therapy.
Correct, [REDACTED]]! Option B) Stable Optimal Response, achieving and maintaining Major Molecular Response (MMR) and Deep Molecular Response (DMR) accurately characterizes R.M.'s long-term response.
Feedback:
Rationale for Correct Answer (B):
MMR Achievement & Maintenance: R.M. achieved MMR (BCR-ABL1 0.1% IS) at the 6-month mark (0.08%) and maintained it consistently thereafter. According to ESMO/ELN, achieving MMR by 12 months is considered an optimal response.
DMR Achievement & Maintenance: R.M. subsequently achieved MR4 (BCR-ABL1 0.01% IS), a key level of Deep Molecular Response (DMR), at the 24-month mark (0.009%). He then maintained this level of response or deepened it further at 36 months (0.005%, which meets MR4.5 criteria). Achieving and maintaining DMR is a significant goal of long-term TKI therapy.
Optimal Response: At every time point from 3 months onwards, his response met the criteria for "Optimal" according to ESMO/ELN guidelines.
Rationale for Incorrect Answers:

A: This is incorrect because R.M. clearly achieved DMR (MR4) at 24 months.

C: His transcript levels showed a consistent downward trend or stabilization at deep levels; they did not meet the criteria for a "Warning" response at any point after the initial months (e.g., failure to achieve MMR by 12 months, or loss of MMR later).

D: His response is excellent and deepening, the opposite of molecular failure, which would typically involve rising transcript levels, loss of MMR, or failure to achieve key milestones.

R.M. is now 5 years post-diagnosis (age 57). He remains on Dasatinib 80 mg daily and has maintained a stable DMR (consistently MR4.5, BCR-ABL1 0.0032% IS) for the past 2.5 years. He is experiencing mild, persistent fatigue and asks about the possibility of stopping his TKI therapy. He has no significant comorbidities other than his controlled hypertension.

Question 9: Based on current ESMO guidelines and data from Treatment-Free Remission (TFR) studies, what is the MOST accurate counsel to provide R.M. regarding stopping Dasatinib?

A) He is not eligible to attempt TFR because he required a dose reduction early in treatment.

B) He meets the minimum criteria to consider a TFR attempt (e.g., CML-CP, duration of TKI, duration of DMR), but requires careful counseling on the ~50% risk of molecular recurrence and need for close monitoring.

C) He should stop Dasatinib immediately as sustained MR4.5 guarantees treatment-free remission.

D) TFR is only recommended for patients initially treated with Imatinib, not second-generation TKIs like Dasatinib.

Excellent reasoning, [REDACTED]]. Option B) He meets the minimum criteria to consider a TFR attempt (e.g., CML-CP, duration of TKI, duration of DMR), but requires careful counseling on the ~50% risk of molecular recurrence and need for close monitoring. is the most accurate counsel.

Feedback:

Rationale for Correct Answer (B):

Eligibility Criteria Met: R.M. fulfills the typical core requirements for considering a TFR attempt as outlined in ESMO guidelines and major TFR trials:

Diagnosis: Chronic Phase CML.

TKI Duration: 5 years (meets typical 3-5 year criteria).

DMR Duration: Stable MR4.5 (0.0032% IS) for 2.5 years (meets typical 2 year criteria).

Reliable Monitoring: Assumed available.

Counseling is Crucial: TFR is not guaranteed. Studies (including those with Dasatinib, like DASFREE) show that approximately 40-60% of eligible patients experience molecular recurrence (loss of MMR) after stopping TKI, usually within the first 6 months. It is essential to counsel R.M. about this significant risk and the absolute necessity for frequent molecular monitoring (typically monthly for the first 6-12 months, then every 2-3 months) to detect recurrence early. If recurrence happens, prompt re-initiation of the TKI almost always recaptures remission.

Rationale for Incorrect Answers:

A: Prior TKI dose reduction due to toxicity does not automatically exclude a patient from TFR eligibility, provided they achieved and maintained stable DMR on the reduced dose. Key TFR studies included such patients.

C: Sustained DMR significantly increases the chance of successful TFR, but it does not guarantee it. Stopping immediately without acknowledging the ~50% relapse risk and monitoring plan is inappropriate.

D: TFR is not restricted to imatinib. Successful TFR attempts have been well-documented in clinical trials with second-generation TKIs, including Dasatinib (DASFREE) and Nilotinib (ENESTfreedom, ENESTop).

After extensive discussion about the risks (molecular relapse requiring TKI restart, potential rare withdrawal syndrome) and benefits (avoiding long-term TKI side effects/cost, potential for sustained remission off-drug), R.M. decides he wants to attempt TFR. A plan for monthly BCR-ABL1 monitoring is put in place. He stops Dasatinib.

Monitoring Post-TFR:

Month 1: BCR-ABL1 = Undetectable (<0.0032% IS)

Month 2: BCR-ABL1 = Detectable, but <0.01% IS (still MR4)

Month 3: BCR-ABL1 = 0.05% (IS)

Month 4: BCR-ABL1 = 0.25% (IS)

Question 10: Based on the molecular monitoring results after stopping Dasatinib, what is the MOST appropriate clinical action at Month 4?

A) Continue monitoring monthly; restart TKI only if symptoms develop.

B) Immediately restart Dasatinib at the original dose of 100 mg daily.

C) Immediately restart Dasatinib at the previously tolerated dose of 80 mg daily.

D) Switch to a different TKI, such as Nilotinib, as Dasatinib has failed in the TFR setting.

Absolutely correct, [REDACTED]]! Option C) Immediately restart Dasatinib at the previously tolerated dose of 80 mg daily. is the most appropriate clinical action.

Feedback:
Rationale for Correct Answer (C):
Loss of MMR: R.M.'s BCR-ABL1 level of 0.25% (IS) at Month 4 signifies a loss of Major Molecular Response (MMR), as it is >0.1% (IS). This is the universally accepted threshold for molecular recurrence requiring intervention in TFR protocols.
Restart Same TKI: Standard practice upon loss of MMR during a TFR attempt is to promptly restart the same TKI that the patient was responding to before stopping.
Restart at Last Effective Dose: The TKI should be restarted at the dose the patient was successfully and tolerably maintained on prior to the TFR attempt. For R.M., this was Dasatinib 80 mg daily. Restarting at this dose is expected to rapidly re-induce remission based on prior efficacy and tolerability.
Rationale for Incorrect Answers:
A: Waiting for symptoms is inappropriate. The goal of TFR monitoring is to detect molecular recurrence early and intervene promptly to prevent hematologic or clinical relapse. Loss of MMR is the trigger for action.
B: Restarting at the original dose of 100 mg daily is unnecessary and potentially exposes the patient to the risk of recurrent pleural effusion, which was managed previously by reducing the dose to 80 mg. The 80 mg dose was proven effective (maintained DMR).
D: Switching TKIs is not indicated simply because of molecular recurrence during TFR. Dasatinib did not "fail"; the TFR attempt was unsuccessful (as occurs in ~50% of patients), but the drug itself was effective prior to stopping. Re-initiation of the same TKI is highly effective in recapturing response. Switching might be considered only if remission is not rapidly regained after restarting Dasatinib or if intolerance develops.

Session ID:
Assessment Domain

Session-54:
Rationale

Session ID:
Instructional Design and Pedagogical

Session-54:
The session's instructional design is of very high quality. The case is expertly scaffolded, progressing logically from initial diagnosis and risk stratification (Questions 1-2), through first-line therapy selection (Question 3), management of a critical side effect (Questions 4-6), long-term response monitoring (Questions 7-8), and finally into the advanced topic of Treatment-Free Remission (TFR) (Questions 9-10). The embedded feedback is a significant strength; each rationale is detailed, cites relevant clinical guidelines (ESMO/ELN), explains why incorrect options are wrong, and provides clear, instructive context. The instructions and case presentation are exceptionally clear. The only minor weakness is that learning objectives are not explicitly stated at the beginning of the session, though the educational goals are strongly implied by the comprehensive structure.

Session ID:
Clinical Authenticity and Content Fidelity

Session-54:
The clinical fidelity of this simulation is outstanding. The narrative is highly plausible, presenting a classic case of Chronic Myeloid Leukemia (CML) that is indistinguishable from an authentic patient encounter. The entire simulation demonstrates perfect alignment with current, evidence-based guidelines (primarily ESMO/ELN 2020) for the management of CML. Every pharmacotherapeutic decisionfrom the initial selection of a second-generation TKI for an intermediate-risk patient, to the specific management of dasatinib-induced pleural effusion, to the criteria for attempting and managing TFRrepresents an optimal, guideline-directed choice. The session remains strictly within the domain of CML, with no evidence of domain entanglement.

Session ID:
Modeled Clinical Reasoning

Session-54:
The simulation flawlessly models an expert clinical reasoning process for CML management. Each stage of the case presents a well-framed clinical problem, providing all necessary data for a sound decision. The multiple-choice options offer an excellent breadth

of plausible and relevant alternatives, including key distractors that effectively test nuanced understanding (e.g., choosing between imatinib and dasatinib, or managing toxicity with dose reduction vs. switching). The overall decision-making strategy demonstrates appropriate prioritization, such as confirming the diagnosis before treating, managing toxicity promptly, and using molecular milestones to guide therapy. The clinical outcomes described are realistic and instructionally valuable, reinforcing the learning points at each step.

Session-55

Session ID:
LLM

Session-55:
Google AI Studio (Gemini 2.0 Pro)

Session ID:
Session Transcript

Session-55:
Phase 1: Initial Presentation and Diagnostic Workup
Patient: Mr. A.B., a 48-year-old male, presents to his primary care physician complaining of persistent fatigue, night sweats, and a feeling of abdominal fullness for the past 3 months. He has also noted unintentional weight loss of approximately 10 lbs over the same period. He has a past medical history significant for well-controlled hypertension managed with lisinopril. He has no known allergies.
Initial Physical Examination:
Vital signs: BP 130/80 mmHg, HR 88 bpm, RR 16/min, Temp 37.1C (98.8F)
General: Appears fatigued but in no acute distress.
Abdomen: Marked splenomegaly is noted, palpable 8 cm below the left costal margin. No hepatomegaly.
Lymphatics: No palpable lymphadenopathy.
Cardiovascular/Respiratory: Unremarkable.
Initial Laboratory Results:
CBC:
WBC: 155,000/L (High)
Differential: Myelocytes 15%, Metamyelocytes 10%, Bands 8%, Segmented Neutrophils 50%, Basophils 8% (High), Eosinophils 4%, Lymphocytes 3%, Monocytes 2%
RBC: 3.9 million/L (Low)
Hgb: 10.5 g/dL (Low)
Hct: 31.5% (Low)
Plt: 650,000/L (High)
Chemistry Panel:
Uric Acid: 9.5 mg/dL (High)
LDH: 450 U/L (High)
Creatinine: 0.9 mg/dL
Liver Function Tests: Within normal limits
Given the clinical presentation and initial laboratory findings, Chronic Myeloid Leukemia (CML) is suspected. A bone marrow biopsy and aspirate are performed, along with peripheral blood cytogenetics and molecular testing.
Bone Marrow Biopsy/Aspirate: Hypercellular marrow (95% cellularity) with marked granulocytic hyperplasia, increased megakaryocytes, and <5% blasts.
Cytogenetics (Karyotyping): Reveals the presence of the Philadelphia chromosome t(9;22)(q34;q11.2) in 20/20 metaphases analyzed.
Molecular Testing (RT-PCR): Detects the BCR::ABL1 fusion transcript (p210 isoform).
Question 1:
Based on the WHO 2022 classification and the diagnostic findings, how would you classify Mr. A.B.'s CML?
A) Chronic Myeloid Leukemia, Chronic Phase (CML-CP)
B) Chronic Myeloid Leukemia, Accelerated Phase (CML-AP)
C) Chronic Myeloid Leukemia, Blast Phase (CML-BP)
D) Chronic Myelomonocytic Leukemia (CMML)
Correct, [REDACTED]]. Your selection of A) Chronic Myeloid Leukemia, Chronic Phase (CML-CP) is accurate.
Rationale:
Correct (A): Mr. A.B.'s diagnosis fits the criteria for CML-CP according to the WHO 2022 classification. Key indicators include the presence of the Philadelphia chromosome t(9;22) and the BCR::ABL1 fusion transcript, combined with less than 10% blasts in the

bone marrow (Mr. A.B. has <5%) and peripheral blood. His symptoms (fatigue, splenomegaly, night sweats), leukocytosis with left shift, thrombocytosis, and basophilia (8%) are all characteristic of CML-CP.

Incorrect (B): Accelerated Phase (CML-AP) is typically defined by criteria such as 10-19% blasts in blood or marrow, peripheral blood basophils 20%, persistent thrombocytopenia or thrombocytosis unresponsive to therapy, or certain cytogenetic evolution. Mr. A.B.'s blast count (<5%) and basophil count (8%) do not meet the criteria for AP.

Incorrect (C): Blast Phase (CML-BP) is defined by 20% blasts in the blood or marrow, or the presence of extramedullary blast proliferation. Mr. A.B.'s blast count is significantly lower.

Incorrect (D): Chronic Myelomonocytic Leukemia (CMML) is a different myelodysplastic/myeloproliferative neoplasm characterized by persistent peripheral blood monocytosis, which is not present here (monocytes 2%). Furthermore, CMML is BCR::ABL1-negative.

Well done. Now that we have confirmed the diagnosis

Question 2:

Several scoring systems exist to predict prognosis and guide therapy in CML-CP. The European LeukemiaNet (ELN) 2022 recommendations utilize risk scores at diagnosis. Which of the following scores is commonly used for risk stratification in newly diagnosed CML-CP to help guide initial TKI selection, according to ELN 2022?

A) FLIPI Score

B) IPSS-R Score

C) Sokal Score

D) R-ISS Score

You selected "Sokal score". Let's evaluate this.

Correct! Your selection of C) Sokal Score is appropriate in this context.

Rationale:

Correct (C): The Sokal score was developed in the pre-TKI era but remains a validated and widely used prognostic scoring system for CML-CP at diagnosis. It incorporates age, spleen size, platelet count, and peripheral blood blast percentage. The ELN 2022 guidelines acknowledge the Sokal score (along with the newer ELTS score) for risk assessment at diagnosis, which can influence treatment considerations.

Incorrect (A): The FLIPI (Follicular Lymphoma International Prognostic Index) score is used for risk stratification in Follicular Lymphoma.

Incorrect (B): The IPSS-R (Revised International Prognostic Scoring System) is used for risk stratification in Myelodysplastic Syndromes (MDS).

Incorrect (C): The R-ISS (Revised International Staging System) is used for staging and risk stratification in Multiple Myeloma.

The ELN 2022 guidelines specifically mention both the Sokal and the ELTS (EUTOS Long-Term Survival) scores for assessing risk at diagnosis. The ELTS score is newer and specifically validated in the TKI era. Let's calculate Mr. A.B.'s Sokal score.

Sokal Score Calculation:

Requires: Age (48 years), Spleen size (8 cm below costal margin), Platelet count (650,000/L), Peripheral blood blasts (<1%, let's use 0.5% for calculation).

Calculation often uses online calculators. Based on these values, Mr. A.B.'s Sokal score falls into the Intermediate Risk category. (Exact calculation is complex, but these parameters typically yield an intermediate score).

Question 3:

Mr. A.B.'s CML-CP is classified as Sokal Intermediate Risk. Given his high leukocyte count (155,000/L) and high uric acid (9.5 mg/dL), what is the most immediate supportive care priority before initiating TKI therapy to mitigate a potential complication?

A) Initiate allopurinol and ensure adequate hydration.

B) Administer prophylactic antibiotics.

C) Start erythropoietin stimulating agent (ESA) for anemia.

D) Arrange for immediate plateletpheresis

Excellent choice, [REDACTED]]. Option A) Initiate allopurinol and ensure adequate hydration is the correct immediate priority.

Rationale:

Correct (A): Mr. A.B. has a very high white blood cell count (155,000/L), indicating a large tumor burden. Initiating TKI therapy will lead to rapid cell lysis. This puts him at significant risk for Tumor Lysis Syndrome (TLS). His elevated baseline uric acid (9.5 mg/dL) further increases this risk. Initiating allopurinol (or rasburicase in very high-risk cases) inhibits uric acid production, and aggressive hydration helps maintain renal function and flush out metabolites (uric acid, phosphate, potassium). This is crucial to prevent TLS-associated acute kidney injury and other metabolic derangements before starting the TKI. (See ESMO guidelines on supportive care).

Incorrect (B): While infection risk exists, prophylactic antibiotics are not the most immediate priority before starting TKI unless there is profound neutropenia or specific risk factors not present here. TLS prevention takes precedence due to the high cell count.

Incorrect (C): Anemia (Hgb 10.5 g/dL) needs management, but ESAs are generally considered later, often after observing the TKI's effect on hematopoiesis, and are not the immediate pre-treatment priority compared to TLS prevention.

Incorrect (D): Plateletpheresis is typically reserved for symptomatic thrombocytosis or extremely high counts (often >1,000,000-1,500,000/L). Mr. A.B.'s count (650,000/L), while high, doesn't usually warrant immediate pheresis as the primary intervention before TLS prophylaxis.

Phase 2: Induction Therapy Selection and Supportive Care Planning.

Mr. A.B. has been started on allopurinol and intravenous hydration. His uric acid level has decreased to 6.0 mg/dL, and his renal function remains stable. You are now tasked with selecting the initial TKI therapy. He is 48 years old, Sokal intermediate risk, has well-controlled hypertension, and no other significant comorbidities identified so far. He expresses a preference for oral medication taken once daily if possible.

Question 4:

According to the ELN 2022 recommendations, which of the following would be an appropriate first-line TKI option for Mr. A.B.?

A) Ponatinib

B) Bosutinib

C) Hydroxyurea

D) Asciminib

Okay, [REDACTED]]. You selected B) Bosutinib. Let's analyze this choice.

Correct! Bosutinib is indeed an appropriate first-line TKI option for Mr. A.B. according to the ELN 2022 recommendations.

Rationale:

Correct (B): Bosutinib is a second-generation TKI approved and recommended for the first-line treatment of CML-CP. It offers potent inhibition of BCR::ABL1 and has demonstrated efficacy in achieving deep molecular responses. It is generally taken once daily with food, aligning partially with the patient's preference (though "with food" is a requirement). It is a suitable choice for intermediate-risk patients. While side effects like diarrhea and liver enzyme elevation can occur, they are often manageable.

Incorrect (A): Ponatinib is a potent TKI but carries a significant risk of vascular occlusive events. According to ELN 2022, it is typically reserved for patients with the T315I mutation or those who have failed multiple prior TKIs, not as a standard first-line choice.

Incorrect (C): Hydroxyurea is a cytoreductive agent used to temporarily lower high cell counts but does not target the underlying BCR::ABL1 fusion protein. It is not a definitive first-line therapy for achieving long-term remission in CML.

Incorrect (D): Asciminib, a STAMP inhibitor, is primarily approved for patients previously treated with two or more TKIs or those with the T315I mutation. While clinical trials are exploring its use in earlier lines, it is not currently recommended as a standard initial first-line therapy by ELN 2022 for all newly diagnosed patients.

Additional Context: Other appropriate first-line options recommended by ELN 2022 for Mr. A.B. would include Imatinib, Nilotinib, and Dasatinib. The choice among these depends on a detailed discussion of efficacy goals, potential side effects, comorbidities (e.g., nilotinib and cardiovascular risk), and patient preferences. You correctly identified Bosutinib as one of these valid options.

Question 5:

Two weeks into Bosutinib therapy, Mr. A.B. calls the clinic reporting frequent loose stools (4-5 times per day) without blood or mucus. He denies fever, abdominal pain, or vomiting. How should this common early side effect of Bosutinib be managed initially?

A) Immediately stop Bosutinib and switch to a different TKI.

B) Recommend an over-the-counter anti-diarrheal agent (e.g., loperamide) and encourage fluid intake.

C) Admit the patient for intravenous fluids and bowel rest.

D) Reduce the Bosutinib dose to 300 mg daily

That's the correct approach, [REDACTED]]. B) Recommend an over-the-counter anti-diarrheal agent (e.g., loperamide) and encourage fluid intake is the appropriate first step.

Rationale:

Correct (B): Diarrhea is a very common side effect of Bosutinib, often occurring early in treatment. For Grade 1-2 diarrhea (as described: 4-5 stools/day, no severe symptoms), the initial management focuses on symptomatic control. Loperamide is the standard first-line agent. Maintaining adequate hydration is also essential to prevent dehydration. (ESMO guidelines recommend symptomatic management for Grade 1/2 diarrhea).

Incorrect (A): Stopping Bosutinib or switching therapy immediately is generally not indicated for Grade 1-2 diarrhea. The goal is to manage the side effect while continuing the effective CML treatment if possible.

Incorrect (C): Admission for IV fluids and bowel rest would be considered for severe (Grade 3-4) diarrhea, dehydration, or significant electrolyte abnormalities, none of which are suggested in this scenario.

Incorrect (D): Dose reduction is a potential strategy if diarrhea is persistent despite anti-diarrheal therapy or if it is more severe (e.g., Grade 3). However, it's not the typical initial intervention for Grade 1-2 diarrhea. The aim is often to maintain the starting dose for optimal efficacy if side effects can be managed symptomatically.

Mr. A.B. starts loperamide as needed and increases his fluid intake. His diarrhea improves to 1-2 soft stools per day over the next week. He continues Bosutinib 400 mg daily.

Monitoring Milestones:

At 3 months, Mr. A.B. returns for follow-up. His CBC is significantly improved:

WBC: 8,500/L

Hgb: 12.8 g/dL

Plt: 280,000/L

Differential: Normal

His spleen is no longer palpable. Quantitative RT-PCR for BCR::ABL1 transcripts (International Scale, IS) is performed on peripheral blood.

Results: BCR::ABL1 transcript level is 1.5% (IS).

Question 6:

According to the ELN 2022 criteria for treatment milestones, how would you assess Mr. A.B.'s response to Bosutinib at 3 months?

A) Optimal Response

B) Warning

C) Failure

D) Major Molecular Response (MMR)

Correct again, [REDACTED]]. Your assessment of A) Optimal Response is accurate according to the ELN 2022 guidelines.

Rationale:

Correct (A): The ELN 2022 criteria define an Optimal Response at 3 months as achieving a BCR::ABL1 transcript level of 10% on the International Scale (IS). Mr. A.B.'s result of 1.5% (IS) clearly meets this criterion. Additionally, he has achieved a Complete Hematologic Response (CHR) with normalization of blood counts and resolution of splenomegaly, which further supports an optimal response at this stage.

Incorrect (B): A "Warning" at 3 months would be indicated if the BCR::ABL1 (IS) level was > 10%.

Incorrect (C): "Failure" at 3 months is generally considered if there's a lack of CHR or if BCR::ABL1 (IS) is > 10% (criteria can vary slightly, but 1.5% is well below the failure threshold).

Incorrect (D): Major Molecular Response (MMR) is defined as BCR::ABL1 (IS) 0.1% (a 3-log reduction from the standardized baseline). While this is a key treatment goal, it is typically expected by 12 months, not 3 months. Mr. A.B.'s level of 1.5% has not yet reached MMR.

Follow-up: Mr. A.B. continues Bosutinib 400 mg daily. His tolerance is good, with occasional mild diarrhea managed with loperamide. His 6-month monitoring shows BCR::ABL1 (IS) = 0.5%.

Question 7:

At his 12-month follow-up visit, Mr. A.B.'s quantitative RT-PCR for BCR::ABL1 (IS) returns at 0.08%. How is this response level best described according to standard CML terminology?

A) Complete Cytogenetic Response (CCyR)

B) Partial Molecular Response (PMR)

C) Major Molecular Response (MMR)

D) Deep Molecular Response (DMR, MR4)

Excellent, [REDACTED]]. You've correctly identified C) Major Molecular Response (MMR).

Rationale:

Correct (C): Major Molecular Response (MMR) is a key therapeutic goal in CML management and is defined as a BCR::ABL1 transcript level of 0.1% on the International Scale (IS), which corresponds to a 3-log reduction from the standardized baseline. Mr. A.B.'s result of 0.08% is less than or equal to 0.1%, clearly meeting the definition of MMR. Achieving MMR by 12 months is considered an optimal response according to ELN 2022 guidelines.

Incorrect (A): Complete Cytogenetic Response (CCyR) is defined by the absence (0%) of Philadelphia chromosome-positive cells in conventional cytogenetic analysis (karyotyping) of the bone marrow. While achieving MMR strongly suggests CCyR has also been achieved, the result 0.08% (IS) specifically quantifies the molecular response, which is much more sensitive.

Incorrect (B): Partial Molecular Response (PMR) is not a standard milestone in the ELN/IS classification system. The primary molecular milestones are MMR (0.1%) and various levels of Deep Molecular Response (DMR).

Incorrect (D): Deep Molecular Response (DMR) refers to responses deeper than MMR. Commonly cited levels include MR4 (BCR::ABL1 0.01% IS) and MR4.5 (BCR::ABL1 0.0032% IS). Mr. A.B.'s level of 0.08% has achieved MMR but has not yet reached MR4.

Phase 3: Post-remission Therapy, MRD Monitoring, and Long-term Management.

Mr. A.B. continues on Bosutinib 400 mg daily. He has maintained MMR (with BCR::ABL1 levels fluctuating between 0.05% and 0.09%

IS) for the past two years. He remains asymptomatic with normal blood counts. Today, at his 3-year follow-up visit, his BCR::ABL1 (IS) level is 0.008%.

Question 8:

Based on this latest BCR::ABL1 (IS) result of 0.008% after sustained MMR, how would you now categorize Mr. A.B.'s molecular response?

A) Major Molecular Response (MMR) only

B) Deep Molecular Response (DMR), specifically MR4

C) Deep Molecular Response (DMR), specifically MR4.5

D) Molecular Relapse

Correct again, [REDACTED]]. The response level of 0.008% IS corresponds to B) Deep Molecular Response (DMR), specifically MR4.

Rationale:

Correct (B): Deep Molecular Response (DMR) encompasses responses deeper than MMR (0.1% IS). MR4 is defined as a BCR::ABL1 transcript level of 0.01% on the International Scale (IS). Mr. A.B.'s result of 0.008% meets this criterion (0.008% is less than or equal to 0.01%).

Incorrect (A): While he still maintains MMR (as 0.008% is 0.1%), categorizing it as "MMR only" is less precise because he has achieved a deeper level of response.

Incorrect (C): MR4.5 is defined as BCR::ABL1 0.0032% (IS). Mr. A.B.'s result of 0.008% is higher than 0.0032%, so he has not yet reached MR4.5.

Incorrect (D): Molecular relapse would involve an increase in BCR::ABL1 levels, typically defined as loss of MMR or a significant rise from a prior DMR level. This result represents a deepening response, not a relapse.

Mr. A.B. achieves sustained MR4 (consistent BCR::ABL1 levels 0.01% IS) for the next two years (total of 5 years on Bosutinib). He asks about the possibility of stopping his TKI therapy.

Question 9:

According to current ESMO/ELN guidelines, which of the following is a key eligibility criterion Mr. A.B. must meet to be considered a candidate for a trial of Treatment-Free Remission (TFR)?

A) Must have been diagnosed with Low Sokal risk CML.

B) Must have received Imatinib as first-line therapy.

C) Must have sustained DMR (e.g., MR4 or deeper) for a minimum duration (typically 2 years).

D) Must be younger than 60 years old.

Absolutely correct, [REDACTED]]. C) Must have sustained DMR (e.g., MR4 or deeper) for a minimum duration (typically 2 years) is a crucial eligibility criterion for TFR consideration.

Rationale:

Correct (C): Both ESMO and ELN guidelines emphasize that patients considered for TFR attempts must have achieved a deep molecular response (DMR), typically defined as MR4 (0.01% IS) or deeper (MR4.5 or MR5). Furthermore, this DMR must be sustained for a significant period, usually at least 2 years, with regular MRD monitoring, before stopping TKI therapy can be considered. This duration demonstrates stability of the deep response.

Incorrect (A): While Sokal risk at diagnosis is prognostic, it is not a strict eligibility criterion for TFR itself. Patients initially classified as intermediate or high risk can become eligible if they achieve and sustain the required DMR.

Incorrect (B): Eligibility for TFR is not restricted to patients who received imatinib first-line. Patients treated with second-generation TKIs (like Mr. A.B. on Bosutinib) are also eligible if they meet the DMR duration criteria. In fact, some studies suggest higher rates of DMR achievement with second-generation TKIs.

Incorrect (D): While age might be considered in the overall context of patient health and preferences, there is no strict upper age limit for TFR eligibility defined in the major guidelines. Suitability is primarily based on achieving and maintaining deep, stable molecular remission.

Mr. A.B. meets the criteria (CML-CP, on TKI > 3 years, sustained MR4 for > 2 years) and, after thorough discussion about the risks (potential relapse requiring TKI resumption) and benefits (avoiding long-term TKI side effects/costs), decides to attempt TFR under close monitoring.

He stops Bosutinib. Monitoring is scheduled monthly for the first 6 months, then every 2 months for the next 6 months, then every 3 months thereafter.

Six months after stopping Bosutinib: Mr. A.B. remains asymptomatic. His quantitative RT-PCR for BCR::ABL1 (IS) is now 0.15%.

Question 10:

Based on this BCR::ABL1 (IS) result of 0.15% six months after stopping TKI therapy, what is the most appropriate clinical action according to typical TFR monitoring protocols?

A) Continue monitoring without restarting TKI, as the level is still relatively low.
B) Restart Bosutinib immediately at the previous dose (400 mg daily).
C) Perform a bone marrow biopsy to assess for cytogenetic relapse.
D) Switch to a different TKI, such as Dasatinib.
Okay, [REDACTED]]. Let's evaluate your choice A) Continue monitoring without restarting TKI, as the level is still relatively low.
Evaluation:
Incorrect (A): While 0.15% might seem numerically small, the critical threshold in TFR monitoring is the loss of Major Molecular Response (MMR). MMR is defined as BCR::ABL1 (IS) 0.1%. Since Mr. A.B.'s level is 0.15%, he has lost MMR. TFR guidelines (like those from ESMO/ELN) clearly state that loss of MMR is the definition of molecular relapse requiring intervention. Continuing to monitor without restarting therapy risks further disease progression.
Correct Answer Explanation (B): The standard recommendation upon confirmation of MMR loss (BCR::ABL1 > 0.1% IS) during a TFR attempt is to restart the TKI therapy that the patient was previously responding to, in this case, Bosutinib 400 mg daily. Prompt resumption of the TKI is highly effective in re-inducing remission (MMR or deeper) in the vast majority of patients who experience molecular relapse after TFR. Waiting could allow for further rise in transcript levels and potentially hematologic or cytogenetic relapse.
Incorrect (C): A bone marrow biopsy is generally not the immediate first step for molecular relapse defined solely by PCR. Restarting TKI is the priority. Biopsy might be considered if there was a suspicion of disease transformation or lack of response after restarting the TKI.
Incorrect (D): Switching TKIs is not indicated simply because of molecular relapse during TFR. The relapse occurred off therapy. The most logical first step is to re-challenge with the drug that previously induced deep remission (Bosutinib). Switching might be considered later if MMR is not rapidly regained or if resistance mutations emerge.

Session ID:
Assessment Domain

Session-55:
Rationale

Session ID:
Instructional Design and Pedagogical

Session-55:
The session demonstrates outstanding instructional design. The clinical case is expertly scaffolded, guiding the learner through a logical and chronologically authentic sequence: initial presentation, diagnosis, risk stratification, supportive care, first-line therapy selection, side effect management, monitoring for key milestones (3, 12, 36 months), and finally, the advanced topic of Treatment-Free Remission (TFR) including management of molecular relapse. The feedback provided for each question is a major strength; rationales are detailed, evidence-based, and consistently reference current clinical guidelines (e.g., WHO, ELN, ESMO). The instructional framing is exceptionally clear. The only minor area for improvement is the lack of explicitly stated, measurable learning objectives at the beginning of the session, though the educational goals are strongly implied by the high-quality structure.

Session ID:
Clinical Authenticity and Content Fidelity

Session-55:
The sessions clinical fidelity is of the highest quality and contains no detectable errors. Plausibility & Guideline Alignment: The clinical narrative is indistinguishable from an authentic patient case, with classic CML presentation, lab values, and response trajectory. All therapeutic decisions, monitoring milestones (e.g., BCR::ABL1 10% at 3 months), and response definitions (MMR, MR4) are perfectly aligned with current, evidence-based ELN and ESMO guidelines. Pharmacotherapeutic Accuracy: All therapeutic recommendations are optimal and well-justified. This includes prioritizing TLS prophylaxis with allopurinol, selecting an appropriate first-line TKI (Bosutinib), providing standard-of-care management for side effects (loperamide for diarrhea), and correctly managing TFR relapse by restarting the prior effective TKI. Domain Specificity: The simulation exhibits flawless domain specificity. It correctly uses CML-specific prognostic scores (Sokal) and correctly identifies distractors from other malignancies (FLIPI, IPSS-R, R-ISS). There is no instance of domain entanglement, rendering the content clinically safe and educationally sound.

Session ID:

Modeled Clinical Reasoning

Session-55:

The simulation expertly models the clinical reasoning process required for CML management. Each stage presents a clearly framed clinical problem, from diagnosis to long-term monitoring. The breadth of the multiple-choice options is excellent, including relevant distractors that effectively test for nuanced understanding of CML therapy. The modeled decision-making is flawless, demonstrating appropriate prioritization at every step (e.g., managing TLS risk before starting TKI, promptly restarting therapy upon loss of MMR). The clinical outcomes described are highly realistic and educationally valuable, perfectly reinforcing the learning objectives for each phase of treatment.

Session-56

Session ID:
LLM

Session-56:
Google AI Studio (Gemini 2.0 Pro)

Session ID:
Session Transcript

Session-56:
Mr. John Smith, a 58-year-old male, presents to his primary care physician with complaints of fatigue, night sweats, and unintentional weight loss of approximately 10 lbs over the past 2 months. He denies any fever, chills, or bleeding. Physical examination reveals mild splenomegaly.Initial Lab Results:

WBC: 150 x 10^9/L (normal range: 4-11 x 10^9/L)

Hemoglobin: 10.5 g/dL (normal range: 13.5-17.5 g/dL)

Platelets: 450 x 10^9/L (normal range: 150-400 x 10^9/L)

Peripheral Blood Smear: Shows a spectrum of myeloid cells with increased blasts (<5%)

Comprehensive Metabolic Panel: Within normal limits

Based on this initial presentation, which of the following is theMOSTappropriate next step in confirming the diagnosis of CML?

A) Initiate treatment with hydroxyurea to control the elevated WBC count.

B) Order a bone marrow biopsy and cytogenetic analysis, including a BCR-ABL1 translocation assessment.

C) Prescribe a course of broad-spectrum antibiotics to rule out an infectious etiology.

D) Reassure the patient and schedule a follow-up appointment in one month to monitor symptoms.

B
The bone marrow biopsy and cytogenetic analysis confirm the presence of the BCR-ABL1 translocation, establishing the diagnosis of Chronic Myeloid Leukemia (CML). Further testing reveals the following:

Cytogenetics:90% Ph+ metaphases

BCR-ABL1 transcript level (IS):95%

Which of the following is theMOSTappropriate next step in the risk stratification of this patient, according to the ELN 2022 recommendations?

A) No further risk stratification is needed; all CML patients are treated the same.

B) Use the Sokal score, based on readily available clinical and hematologic parameters.

C) Use the ELN 2022 risk score, incorporating age, spleen size, and blast percentage.

D) Proceed directly to treatment selection without formal risk stratification.

C

Based on the patient's age (58 years), spleen size (slightly palpable, 12 cm), and blast percentage in bone marrow (<5%), Mr. Smith is classified aslow riskaccording to the ELN 2022 risk score.

Considering the diagnostic workup and risk stratification, which of the following additional tests would beMOSTimportant to performBEFOREinitiating tyrosine kinase inhibitor (TKI) therapy?

A) Complete pulmonary function tests to assess for pre-existing lung disease.

B) Echocardiogram to evaluate left ventricular ejection fraction (LVEF) and assess for any cardiac abnormalities.

C) Bone density scan (DEXA) to screen for osteoporosis.

D) Magnetic resonance imaging (MRI) of the brain to rule out intracranial involvement.

B

Based on the ELN 2022 recommendations and considering Mr. Smith's low-risk ELN score and otherwise good health, which of the following is theMOSTappropriate first-line treatment option for him?

A) High-dose imatinib (800 mg daily)

B) Standard-dose imatinib (400 mg daily)

C) Allogeneic hematopoietic stem cell transplantation (allo-HSCT)

D) Cytarabine and daunorubicin induction chemotherapy

B

Mr. Smith starts on imatinib 400 mg daily. Three weeks later, he develops grade 3 nausea and vomiting, despite taking antiemetics as prescribed. His labs are stable, and he denies any other new symptoms.

Which of the following is theMOSTappropriate next step in managing his nausea and vomiting?

A) Discontinue imatinib and switch to bosutinib.

B) Reduce the dose of imatinib to 300 mg daily.

C) Add aprepitant to his antiemetic regimen.

C

Four months after starting imatinib, Mr. Smith's BCR-ABL1 transcript level (IS) is 15%.

According to the ELN 2022 recommendations, this level of response at 4 months is classified as:

A) Optimal response

B) Warning

C) Failure

D) Not evaluable

A

Given Mr. Smith's BCR-ABL1 transcript level of 15% at 4 months, what is theMOSTappropriate next step in his management, according to ELN 2022 guidelines?

A) Continue imatinib at the same dose and recheck BCR-ABL1 in 3 months.

B) Increase the dose of imatinib to 600 mg daily.

C) Switch to a second-generation TKI (e.g., nilotinib or dasatinib).

D) Initiate allogeneic stem cell transplantation (allo-SCT).

C

Considering Mr. Smith's deep molecular response (MR4.5) on nilotinib, which of the following statements regarding long-term monitoring isMOSTaccurate?

A) Monitoring can be discontinued as he has achieved a stable deep molecular response.

B) BCR-ABL1 transcript levels should be monitored every 3 months indefinitely.

C) BCR-ABL1 transcript levels should be monitored every 6 months for the first year, then annually if stable.

D) Attempting treatment-free remission (TFR) should be considered.

D

Mr. Smith decides to pursue a trial of treatment-free remission (TFR). He stops nilotinib under close monitoring. Three months later, his BCR-ABL1 transcript level increases to 0.1% (MR3).

Which of the following is theMOSTappropriate next step in managing Mr. Smith?

A) Continue monitoring BCR-ABL1 monthly and restart nilotinib if the transcript level increases further.

B) Immediately restart nilotinib at the original dose of 300 mg twice daily.

C) Prescribe a course of interferon-alpha to boost his immune response.

D) Initiate ponatinib therapy for deeper remission.

A

Mr. Smith restarts nilotinib at 300mg BID. He achieves MR4.5 again after 6 months. He is now asking about trying TFR again.

What would you recommend?

A) TFR should not be attempted again.

B) TFR can be attempted again in 6 months if MR4.5 is maintained.

C) TFR can be attempted immediately.

D) TFR can be attempted again in 2 years if MR4.5 is maintained.

D

Session ID:

Assessment Domain

Session-56:

Rationale

Session ID:

Instructional Design and Pedagogical

Session-56:

The session's instructional design has significant strengths but one critical weakness. The case is expertly scaffolded, progressing logically from initial diagnosis and risk stratification to first-line therapy, side effect management, response assessment, and complex long-term management like Treatment-Free Remission (TFR). The instructional framing is exceptionally clear, with each question presenting an unambiguous clinical scenario. Learning objectives are strongly implied by this structure, though not explicitly stated. The session's primary pedagogical failure is the complete absence of embedded feedback or rationales for the selected answers, which is essential for learning. A simple letter answer without explanation provides no instructional value, resulting in the lowest score for that criterion.

Session ID:

Clinical Authenticity and Content Fidelity

Session-56:

The clinical narrative is highly plausible and indistinguishable from an authentic CML case. However, the session's fidelity is severely compromised by critical errors and significant deviations from current evidence-based guidelines (ELN/NCCN). A major pharmacotherapeutic error occurs in Question 6, where a BCR-ABL1 level of 15% at 4 monthswhich constitutes treatment failureis incorrectly identified as an "optimal response." A second dangerous error occurs in Question 9, where the recommended action for a patient losing molecular remission during a TFR trial is to "continue monitoring," which contradicts the standard of care to restart therapy immediately. These are not minor inaccuracies; they represent fundamentally incorrect and unsafe clinical judgments. The session correctly remains within the CML domain without any evidence of domain entanglement.

Session ID:

Modeled Clinical Reasoning

Session-56:

The simulation fails to model a sound clinical reasoning process. While individual problems are well-framed and the multiple-choice

options are relevant and plausible, the core decision-making logic is illogical and unsafe. In Question 7, the simulation chooses an action (switch TKI) that is appropriate for treatment failure, despite having incorrectly labeled the response as "optimal" in Question 6. This represents a complete breakdown in logical consistency. Furthermore, the decision in Question 9 demonstrates flawed risk prioritization by delaying necessary treatment after TFR failure. The modeling of clinical outcomes is also poor; while the outcomes presented are realistic (e.g., a specific BCR-ABL1 level), the flawed interpretation and subsequent actions render the model instructionally harmful.

Session-57

Session ID:
LLM

Session-57:
Google AI Studio (Gemini 2.0 Pro)

Session ID:
Session Transcript

Session-57:
Patient:** Mr. David Miller, a 52-year-old male, presents to his primary care physician complaining of increasing fatigue, unintentional weight loss of 10 lbs over the past 3 months, and early satiety. He initially attributed these symptoms to work stress but sought medical attention when he noticed persistent abdominal fullness.
Past Medical History: Hypertension (well-controlled on Lisinopril 10 mg daily), Hyperlipidemia (managed with Atorvastatin 20 mg daily). No history of malignancy or chemotherapy.
Social History: Non-smoker, occasional alcohol use (1-2 drinks per weekend). Works as an accountant.
Family History: Father had prostate cancer; mother has type 2 diabetes.
Review of Systems: Positive for fatigue, weight loss, early satiety, and mild night sweats. Negative for fever, chills, bone pain, bleeding, or easy bruising
Physical Examination:
* Vitals: BP 130/80 mmHg, HR 78 bpm, RR 16/min, Temp 37.0C (98.6F) General: Appears tired but in no acute distress.
* Abdomen: Marked splenomegaly, palpable 8 cm below the left costal margin. No hepatomegaly or lymphadenopathy noted.
* Other systems: Unremarkable.
Initial Laboratory Results:
* **CBC:**
    * WBC: 115 x 10/L (Normal: 4.0-11.0 x 10/L)
    * Hemoglobin: 10.5 g/dL (Normal: 13.5-17.5 g/dL)
    * Platelets: 550 x 10/L (Normal: 150-450 x 10/L)
* **Differential:**
    * Neutrophils: 60%
    * Bands: 10%
    * Metamyelocytes: 8%
    * Myelocytes: 12%
    * Promyelocytes: 2%
    * Blasts: 1%
    * Basophils: 5% (Normal: 0-1%)
    * Eosinophils: 2%
    * Lymphocytes: 8%
    * Monocytes: 2%
* **Basic Metabolic Panel (BMP):** Within normal limits (Creatinine 0.9 mg/dL, BUN 15 mg/dL, Electrolytes normal).
* **Liver Function Tests (LFTs):** Within normal limits.
* **LDH:** Elevated at 450 U/L (Normal: 140-280 U/L)
* **Uric Acid:** Elevated at 8.5 mg/dL (Normal: 3.4-7.0 mg/dL)
Based on Mr. Miller's presentation and initial lab results, Chronic Myeloid Leukemia (CML) is highly suspected.

Question 1: According to WHO 2016/2022 and ESMO guidelines, what is the **single most crucial diagnostic test** needed *next* to confirm the diagnosis of CML and determine its specific characteristics?

A) Peripheral blood smear morphological review by a hematopathologist.
B) Bone marrow biopsy and aspirate with conventional cytogenetics (karyotyping) and molecular testing (RT-PCR for *BCR-ABL1*).
C) Abdominal CT scan to precisely measure spleen size and evaluate for other organ involvement.

D) Serum lactate dehydrogenase (LDH) and uric acid levels measurement.
Feedback:**

* **Correct Answer (B):** A bone marrow biopsy and aspirate with conventional cytogenetics (karyotyping) and molecular testing (RT-PCR for *BCR-ABL1*) is absolutely essential.
    * **Why it's crucial:** CML is defined by the presence of the Philadelphia chromosome (Ph chromosome), which results from the reciprocal translocation t(9;22)(q34.1;q11.2). This translocation creates the *BCR-ABL1* fusion gene.
    * **Cytogenetics (Karyotyping):** Detects the Ph chromosome and any additional chromosomal abnormalities (ACAs), which have prognostic significance.
    * **Molecular Testing (RT-PCR):** Detects and quantifies the *BCR-ABL1* fusion transcript (mRNA). This is the most sensitive method, confirms the diagnosis, identifies the specific transcript type (e.g., p210, p190), and establishes a crucial baseline for monitoring treatment response (MRD).
    * **Guideline Basis:** Both WHO 2016/2022 classification and ESMO CML guidelines mandate the demonstration of the t(9;22) or the *BCR-ABL1* fusion gene for diagnosis.

* **Why other options are less crucial *at this specific diagnostic step*:**
    * **A (Peripheral Smear):** While the smear provided the initial clues (leukocytosis, left shift, basophilia), it cannot definitively confirm the diagnosis or identify the underlying genetic abnormality.
    * **C (Abdominal CT):** While helpful for evaluating the extent of splenomegaly or other organ involvement, it doesn't provide a diagnosis of CML. Physical exam already identified significant splenomegaly.
    * **D (LDH/Uric Acid):** These are non-specific markers of cell turnover and proliferation. While often elevated in CML and important for assessing risk of tumor lysis syndrome, they are not diagnostic.

Mr. Miller undergoes a bone marrow biopsy and aspirate. The results are as follows:

* **Bone Marrow Aspirate:** Markedly hypercellular (95% cellularity) with granulocytic hyperplasia. Blasts account for 2% of nucleated cells. Megakaryocytes are increased and show some dysplastic features.
* **Bone Marrow Biopsy:** Confirms hypercellularity and granulocytic predominance. Fibrosis is minimal (MF-0).
* **Conventional Cytogenetics (Karyotyping):** 46,XY,t(9;22)(q34.1;q11.2)[20/20 metaphases]. No additional chromosomal abnormalities (ACAs).
* **Molecular Testing (RT-PCR):** Positive for *BCR-ABL1* fusion transcript. Quantitative analysis reveals a *BCR-ABL1* transcript level of 65% (IS - International Scale). The transcript type is identified as p210 (e14a2 junction).

These results confirm the diagnosis of **Chronic Myeloid Leukemia, Chronic Phase (CML-CP), *BCR-ABL1* positive.**

Now, we need to determine Mr. Miller's risk stratification to guide treatment decisions.

Question 2: Based on the available clinical and laboratory data, calculate Mr. Miller's risk score using the **ELN 2020 recommendations**, which primarily utilize the **Sokal score** for prognosis at diagnosis. Which risk category does he fall into?

(Reference Information for Sokal Score Calculation:
Sokal Score = exp[0.0116 (age in years - 43.4) + 0.0345 (spleen size cm below costal margin - 7.51) + 0.188 ((platelet count 10/L / 700) - 0.563) + 0.0887 (% blasts in peripheral blood - 2.10)])

Patient Data Recap:
* Age: 52 years
* Spleen Size: 8 cm below costal margin
* Platelet Count: 550 x 10/L
* Peripheral Blood Blasts: 1%

A) Low Risk (Sokal Score < 0.8)
B) Intermediate Risk (Sokal Score 0.8 - 1.2)
C) High Risk (Sokal Score > 1.2)

D) Insufficient data to calculate the Sokal score.
Correct Answer (B):* Your calculation is correct! Mr. Miller's Sokal score is approximately 1.03, which places him in the **Intermediate Risk** category (Sokal Score 0.8 - 1.2).
*   **Why it matters:** Risk stratification at diagnosis using scores like Sokal (or the newer ELTS score, though Sokal is still widely used and referenced in ELN guidelines) is crucial for predicting prognosis and informing the intensity of initial treatment and monitoring strategies. Intermediate-risk patients may warrant consideration for more potent second-generation TKIs, although first-generation TKIs remain an option.
*   **Other Options:**
    *   A (Low Risk) would require a score < 0.8.
    *   C (High Risk) would require a score > 1.2.
    *   D is incorrect as all necessary data points were provided.

Great job performing the risk calculation! This is a fundamental step in CML management.

With the diagnosis of *BCR-ABL1* positive CML-CP confirmed and Mr. Miller stratified as Intermediate Risk (Sokal), the next step is to discuss first-line treatment options. Before initiating therapy, baseline assessments are important.

Question 3: Prior to starting Tyrosine Kinase Inhibitor (TKI) therapy for Mr. Miller (Intermediate Risk CML-CP), which of the following baseline assessments is **LEAST** critical according to ESMO guidelines?

A) Quantitative RT-PCR for *BCR-ABL1* transcript levels (IS).
B) Assessment of cardiovascular risk factors and an ECG.
C) Hepatitis B virus (HBV) serology (HBsAg, anti-HBc, anti-HBs).
D) HLA typing for potential future allogeneic stem cell transplantation.

*Correct Answer (D):** HLA typing is done to identify potential donors for an allogeneic stem cell transplant (allo-SCT). While allo-SCT is a curative option for CML, it is typically reserved for patients who fail multiple lines of TKI therapy, develop specific resistance mutations (like T315I if ponatinib/asciminib are not options or fail), or progress to accelerated or blast phase. For a newly diagnosed, intermediate-risk CML-CP patient like Mr. Miller, TKIs are the standard first-line therapy, and allo-SCT is not an immediate consideration. Therefore, performing HLA typing before even starting the first TKI is not a mandatory or critical baseline step according to ESMO guidelines for initial management.
*   **Why other options are MORE critical:**
    *   **A (Baseline RT-PCR):** Absolutely essential. This establishes the pre-treatment *BCR-ABL1* level, which is the primary benchmark for monitoring response to TKI therapy (Molecular Response - MR). Without this baseline, assessing treatment efficacy is impossible. (ESMO Guidelines)
    *   **B (Cardiovascular Assessment/ECG):** Critical. Several TKIs have potential cardiovascular side effects (e.g., QT prolongation with nilotinib, vascular events with nilotinib/ponatinib, pleural effusions possibly linked to cardiac function with dasatinib). Assessing baseline cardiovascular risk and obtaining an ECG helps guide TKI selection and monitoring. (ESMO Guidelines)
    *   **C (HBV Serology):** Important. TKI therapy can potentially lead to Hepatitis B reactivation in patients who are carriers (HBsAg positive) or have resolved infection (anti-HBc positive). Screening allows for appropriate monitoring or prophylactic antiviral therapy if needed. (ESMO Guidelines)

Mr. Miller has been diagnosed with Intermediate Risk (Sokal), *BCR-ABL1* positive CML-CP. His baseline assessments are complete (including ECG, HBV serology), and he is ready to start first-line therapy.

Learning Objective for Phase 2: Select appropriate first-line TKI therapy considering patient-specific factors and guideline recommendations, and plan necessary supportive care.

The available first-line TKI options approved for CML-CP include:
*   Imatinib (1st Generation TKI)
*   Dasatinib (2nd Generation TKI)
*   Nilotinib (2nd Generation TKI)

*   Bosutinib (2nd Generation TKI)

Patient Recap: 52 years old, Intermediate Risk (Sokal), PMH: Hypertension (controlled), Hyperlipidemia (controlled). No known cardiac issues beyond controlled HTN/HLD. ECG normal. HBV negative. *BCR-ABL1* transcript type p210.

Question 4: Considering Mr. Miller's profile (52yo, Intermediate Risk CML-CP, controlled HTN/HLD, p210 transcript) and aiming for potentially faster and deeper responses often seen with second-generation TKIs, which of the following first-line TKI choices would be **most appropriate** according to ESMO guidelines, balancing efficacy and potential toxicities?

A) Imatinib 400 mg daily
B) Nilotinib 300 mg twice daily
C) Dasatinib 100 mg daily
D) Bosutinib 400 mg daily
Plausibility (C - Dasatinib 100 mg daily):** Dasatinib is a potent, second-generation TKI approved for first-line treatment of CML-CP and recommended by ESMO guidelines. It often leads to faster and deeper responses compared to imatinib, which aligns with the goal stated in the question for an intermediate-risk patient. It is a valid and frequently used option.
*   **Considerations with Dasatinib:** The main characteristic toxicity associated with dasatinib is pleural effusion (fluid around the lungs), which occurs in a significant minority of patients. While Mr. Miller doesn't have overt pulmonary disease, this requires careful monitoring. Dasatinib can also cause cytopenias and, less commonly, pulmonary arterial hypertension (PAH) with long-term use.
*   **Comparison with other options:**
    *   **A (Imatinib):** While a standard option, it might not achieve the goal of *faster/deeper* responses as reliably as a 2nd Gen TKI in intermediate/high-risk patients.
    *   **B (Nilotinib):** Given Mr. Miller's history of hypertension and hyperlipidemia (even though controlled), the known cardiovascular and vaso-occlusive risks associated with nilotinib (including QT prolongation, peripheral artery disease) make it a less ideal choice compared to other 2nd Gen TKIs in this specific patient, as per ESMO recommendations to consider comorbidities.
    *   **D (Bosutinib):** Bosutinib is another potent second-generation TKI. Its primary toxicities are gastrointestinal (diarrhea) and hepatic (LFT elevation), which are usually manageable. It generally has a lower risk of cardiovascular events compared to nilotinib and a lower risk of pleural effusions compared to dasatinib. Therefore, Bosutinib could also be considered a very appropriate, perhaps even slightly preferable, choice for Mr. Miller due to its safety profile in the context of his HTN/HLD.

*   **Conclusion:** Dasatinib (C) is a **reasonable and guideline-concordant choice** for Mr. Miller, aiming for potent efficacy. However, the risk of pleural effusion must be discussed and monitored. Bosutinib (D) represents an equally strong, and arguably slightly safer alternative from a cardiovascular/pulmonary standpoint in this specific patient profile. Both C and D are defendable choices over A and B. Since both Dasatinib and Bosutinib are valid 2nd Gen options here based on guidelines, we will proceed with Dasatinib for this case simulation, but it's important to recognize the appropriateness of Bosutinib as well.

Mr. Miller is counseled on the risks and benefits of the different TKI options. After discussion, you and the oncologist decide to initiate **Dasatinib 100 mg orally once daily**. You provide extensive counseling on administration, potential side effects (especially cytopenias, fluid retention/pleural effusion, GI effects), and the importance of adherence.

Before he leaves the clinic, you also need to address potential supportive care needs, particularly related to his initial high tumor burden (WBC 115k, splenomegaly, elevated LDH/Uric Acid).

Question 5: Given Mr. Miller's initial high WBC count (115 x 10/L) and elevated uric acid (8.5 mg/dL), which supportive care measure is **most important** to initiate *concurrently* with the start of Dasatinib therapy to prevent an oncologic emergency?

A) Prophylactic broad-spectrum antibiotics (e.g., Levofloxacin).
B) Aggressive intravenous hydration and a hypouricemic agent (e.g., Allopurinol or Rasburicase).
C) Immediate platelet transfusion due to potential TKI-induced thrombocytopenia.
D) Granulocyte colony-stimulating factor (G-CSF, e.g., Filgrastim) to mitigate neutropenia risk.

*   **Correct Answer (B):** Aggressive intravenous hydration and a hypouricemic agent (like Allopurinol or, in high-risk cases, Rasburicase) are crucial for the prevention of Tumor Lysis Syndrome (TLS).

* **Why it's critical:** Mr. Miller has a high tumor burden (WBC 115k, splenomegaly) and pre-existing elevated uric acid. Initiating potent therapy like Dasatinib will cause rapid cell lysis, releasing intracellular contents (potassium, phosphate, nucleic acids). Nucleic acids are metabolized to uric acid, leading to hyperuricemia. Without prophylaxis, hyperuricemia, hyperkalemia, hyperphosphatemia, and secondary hypocalcemia can occur, potentially causing acute kidney injury, arrhythmias, and seizures. This constitutes TLS, an oncologic emergency.
* **Hydration:** Promotes renal clearance of metabolic byproducts.
* **Hypouricemic Agents:** Allopurinol prevents the *formation* of new uric acid by inhibiting xanthine oxidase. Rasburicase (a recombinant urate oxidase) rapidly breaks down *existing* uric acid into more soluble allantoin; it's often preferred in high-risk TLS situations for faster control. Given his intermediate-to-high risk profile for TLS, initiating one of these is essential.
* **Guideline Basis:** Guidelines for managing hematologic malignancies strongly recommend TLS risk assessment and prophylaxis for patients with high tumor burden undergoing initial therapy (e.g., Cairo-Bishop criteria for TLS risk).

* **Why other options are less critical *at this moment*:**
    * **A (Prophylactic Antibiotics):** While infections can occur later due to TKI-induced neutropenia, prophylactic antibiotics are not standard practice at initiation for CML-CP unless there's profound neutropenia or active infection, neither of which Mr. Miller has initially. TLS is the more immediate threat.
    * **C (Platelet Transfusion):** Mr. Miller has thrombocytosis (platelets 550k), not thrombocytopenia. Prophylactic transfusion is inappropriate. TKIs can cause thrombocytopenia later, but that's a monitoring parameter, not an initial prophylactic need here.
    * **D (G-CSF):** G-CSF stimulates neutrophil production. It's used for treatment-induced neutropenia. Starting it concurrently with initial TKI therapy in a patient with marked leukocytosis is generally not indicated and doesn't address the primary risk of TLS.

Mr. Miller is admitted briefly for initiation of Dasatinib 100 mg daily, aggressive IV hydration, and Allopurinol 300 mg daily. His electrolytes, renal function, and uric acid are monitored closely. He tolerates the initiation well, with his WBC count starting to decrease and uric acid normalizing. He is discharged home after 48 hours with instructions for close follow-up.

He returns to the clinic 2 weeks later for assessment. He reports mild fatigue and occasional loose stools (1-2 per day), but generally feels okay.

Laboratory Results (2 weeks post-Dasatinib):
* WBC: 25.5 x 10/L (Down from 115k)
* Hgb: 10.2 g/dL (Stable)
* Platelets: 380 x 10/L (Down from 550k)
* ANC: 15.0 x 10/L
* Differential: Shows reduction in immature forms, no blasts.
* BMP/LFTs: Within normal limits.

Question 6: Mr. Miller calls the clinic one month after starting Dasatinib. He reports experiencing mild shortness of breath, especially when lying down, and a non-productive cough over the last few days. He denies fever, chest pain, or leg swelling. What is the **most likely** Dasatinib-related adverse effect causing these symptoms?

A) Community-acquired pneumonia
B) Pulmonary Arterial Hypertension (PAH)
C) Pleural Effusion
D) Dasatinib-induced interstitial lung disease

Feedback:**

* **Correct Answer (C):** Pleural Effusion (fluid accumulation in the space between the lung and the chest wall) is a well-known and relatively common adverse effect associated with Dasatinib therapy. Its incidence increases with dose and duration of therapy but can occur early on. Symptoms classically include dyspnea (shortness of breath), especially orthopnea (worsening when lying flat), and non-productive cough, matching Mr. Miller's report.
* **Why other options are less likely *at this stage*:**

* **A (Pneumonia):** While possible, the absence of fever and typically more acute symptoms make pneumonia less likely as the *most probable* Dasatinib-related cause initially.
* **B (Pulmonary Arterial Hypertension - PAH):** PAH is a serious but less common, typically later-onset complication of long-term Dasatinib use. It usually presents with more progressive exertional dyspnea. It's unlikely to be the cause after only one month.
* **D (Interstitial Lung Disease - ILD):** Dasatinib-induced ILD is rare. While it presents with cough and dyspnea, pleural effusion is far more characteristic of Dasatinib toxicity.

Mr. Miller comes to the clinic. A chest X-ray confirms a moderate left-sided pleural effusion. Given his symptoms, the decision is made to temporarily hold Dasatinib and initiate a short course of diuretics (Furosemide). His symptoms improve significantly over the next week.

Question 7: After Mr. Miller's pleural effusion resolves with temporary Dasatinib interruption and diuretic use, what is the **most appropriate next step** regarding his CML therapy, according to common practice and guideline recommendations?

A) Permanently discontinue Dasatinib and switch to Imatinib.
B) Permanently discontinue Dasatinib and switch to Nilotinib.
C) Restart Dasatinib at a reduced dose (e.g., 70 mg or 50 mg daily) with close monitoring.
D) Add a prophylactic diuretic to the original Dasatinib 100 mg dose.
Correct Answer (C):** Restarting Dasatinib at a reduced dose (e.g., 70 mg or 50 mg daily) with close monitoring is the standard approach after resolution of a Grade 2 or moderate pleural effusion.

* **Rationale:** Dasatinib-induced pleural effusion is often dose-dependent. Since Mr. Miller was showing a good hematologic response to Dasatinib, attempting to continue this effective therapy at a lower, potentially better-tolerated dose is preferred over immediately switching agents or abandoning the drug. Close monitoring for recurrence is essential. If effusion recurs even at the lower dose, then switching TKI would be necessary.
* **Guideline Basis:** Management algorithms for TKI toxicities (referenced in ESMO/NCCN guidelines) typically recommend dose interruption followed by dose reduction for moderate (Grade 2) toxicities that resolve, aiming to maintain the patient on the initial effective therapy if possible.

* **Why other options are less appropriate:**
    * **A (Switch to Imatinib):** This represents de-escalation to a potentially less potent TKI for this intermediate-risk patient, which might compromise the depth of response. It's usually considered if Dasatinib cannot be tolerated even at reduced doses.
    * **B (Switch to Nilotinib):** Switching introduces different potential toxicities (cardiovascular/vaso-occlusive risks relevant to Mr. Miller) without first attempting to manage the Dasatinib toxicity through dose adjustment.
    * **D (Add prophylactic diuretic):** While diuretics treat the effusion, they don't address the underlying cause (Dasatinib dose sensitivity). Restarting at the full dose, even with a diuretic, carries a high risk of recurrence. Dose reduction is the primary strategy.

You've successfully navigated the initial treatment selection and management of a common TKI side effect. This completes Phase 2.
Dasatinib is restarted at 70 mg daily. Mr. Miller tolerates this dose well with no recurrence of pleural effusion or other significant side effects. He undergoes regular monitoring, including quantitative RT-PCR for *BCR-ABL1* transcripts (IS).

Monitoring Results:
* **Baseline:** *BCR-ABL1* = 65% IS
* **3 Months:** *BCR-ABL1* = 8% IS
* **6 Months:** *BCR-ABL1* = 0.5% IS
* **12 Months:** *BCR-ABL1* = 0.05% IS

Question 8: According to the ELN 2020 recommendations, how would you classify Mr. Miller's molecular response at **12 months** (*BCR-ABL1* = 0.05% IS)?

A) Optimal Response (Major Molecular Response - MMR)
B) Warning (Failure to achieve MMR)
C) Failure (Less than Complete Cytogenetic Response - CCyR)
D) Suboptimal Response (Requires immediate switch of TKI)

Correct Answer (A):** Optimal Response (Major Molecular Response - MMR).
    * **Rationale:** According to ELN 2020 guidelines, achieving a *BCR-ABL1* transcript level of 0.1% on the International Scale (IS) defines Major Molecular Response (MMR). At 12 months, achieving MMR is considered an **Optimal Response**. Mr. Miller's level of 0.05% IS is below the 0.1% threshold, therefore he has achieved MMR and has an Optimal Response at this time point.
    * **Clinical Significance:** Achieving MMR is a critical therapeutic goal in CML management and is associated with a lower risk of disease progression and excellent long-term outcomes. It's also a prerequisite for considering deeper responses (like MR4 or MR4.5) and potentially, treatment-free remission (TFR) later on.

* **Why other options are incorrect:**
    * **B (Warning):** A "Warning" at 12 months would be if *BCR-ABL1* levels were > 0.1% IS but 1% IS. Mr. Miller is well below this range.
    * **C (Failure):** "Failure" at 12 months is defined as *BCR-ABL1* > 1% IS. This also implies a lack of Complete Cytogenetic Response (CCyR is roughly equivalent to *BCR-ABL1* 1% IS), but his molecular response clearly indicates he is well beyond CCyR.
    * **D (Suboptimal Response):** "Suboptimal" is terminology used in older guidelines. The current ELN 2020 framework uses "Optimal," "Warning," and "Failure." Even under older definitions, achieving MMR at 12 months would not have been considered suboptimal.

Excellent interpretation of the molecular monitoring results against guideline definitions!
Mr. Miller continues on Dasatinib 70 mg daily and maintains an Optimal Response. His monitoring continues:
* **18 Months:** *BCR-ABL1* = 0.008% IS (MR4 achieved)
* **24 Months:** *BCR-ABL1* = 0.005% IS (Stable MR4)
* **36 Months:** *BCR-ABL1* = 0.002% IS (MR4.5 achieved)
* **48 Months:** *BCR-ABL1* = 0.0025% IS (Stable MR4.5)
* **60 Months (5 Years):** *BCR-ABL1* = 0.0028% IS (Stable MR4.5)

He has now been on Dasatinib for 5 years (1 month at 100mg, the rest at 70mg) and has maintained a deep molecular response (MR4.5: *BCR-ABL1* 0.0032% IS) for at least 2 years. He is feeling well with minimal side effects. He asks about the possibility of stopping his medication.

Question 9: According to current ESMO guidelines for attempting Treatment-Free Remission (TFR), which of the following criteria has Mr. Miller **NOT** yet strictly met, suggesting caution or further waiting before recommending a TFR attempt?

A) Achieved MR4.5 (*BCR-ABL1* 0.0032% IS).
B) Duration of TKI therapy (minimum 3 years).
C) Duration of deep molecular response (MR4 or deeper for minimum 2 years).
D) Specific *BCR-ABL1* transcript type (must be typical e13a2/e14a2).
Revisiting the Criteria:**
    * **A (Achieved MR4.5):** Yes, his level is 0.0028% IS (0.0032% IS). **Met.**
    * **B (Duration of TKI therapy):** He has been on TKI for 5 years (minimum is >3 years). **Met.**
    * **C (Duration of deep molecular response):** He achieved MR4.5 at 36 months and is now at 60 months. This means he has sustained MR4.5 for exactly **24 months (2 years)**. The typical minimum requirement is >2 years. **Met.**

* **Correct Interpretation / Intended Answer (likely C):** While Mr. Miller technically meets all the listed minimum criteria based on standard guidelines (including >2 years in DMR), the criterion he meets *most narrowly* is the duration of deep molecular response (Option C). He has sustained MR4.5 for *exactly* 2 years. Clinical guidelines and TFR studies (like STIM, EURO-SKI) suggest that while >2 years is the minimum eligibility threshold, the probability of successful TFR increases significantly with longer durations of sustained DMR (e.g., >3 years). Therefore, having *only* just completed the minimum 2-year duration in DMR is the factor that might most reasonably "suggest caution or further waiting" before recommending a TFR attempt, aiming for a higher chance of success. He hasn't *failed* to meet it, but he hasn't substantially exceeded the minimum duration of DMR, unlike his total TKI therapy duration.

So, while technically meeting all minimums, the nuance lies in the duration of DMR being exactly at the threshold, potentially making Option C the point of discussion and caution for TFR.

relatively short duration (exactly 2 years) of sustained MR4.5, and the roughly 50-60% chance of maintaining remission off-therapy even when meeting criteria, Mr. Miller decides he feels comfortable continuing Dasatinib 70 mg daily for now, aiming for a longer period of sustained DMR before reconsidering TFR. He continues routine monitoring.

Six months later (at 5.5 years of therapy), his *BCR-ABL1* level remains stable at 0.0025% IS (MR4.5). However, routine labs reveal a new abnormality:
* Platelet Count: 95 x 10/L (Previously stable > 300k)
* ANC: 1.2 x 10/L (Previously stable > 1.5)
* Hgb: 10.0 g/dL (Mild decrease)

He denies any new symptoms, bleeding, bruising, or signs of infection.

Question 10: What is the most appropriate **initial** action to take in response to Mr. Miller's new onset Grade 2 thrombocytopenia and Grade 2 neutropenia, occurring late during Dasatinib therapy while maintaining MR4.5?

A) Immediately perform a bone marrow biopsy with cytogenetics and molecular testing to rule out disease progression or clonal evolution.
B) Empirically switch TKI therapy to Bosutinib, assuming Dasatinib toxicity.
C) Hold Dasatinib therapy temporarily and monitor blood counts closely for recovery.
D) Add G-CSF (Filgrastim) and consider a platelet-stimulating agent (e.g., Eltrombopag) while continuing Dasatinib.

Correct Answer (C):** Hold Dasatinib therapy temporarily and monitor blood counts closely for recovery.
 * **Rationale:** Late-onset cytopenias can occur with TKIs, including Dasatinib. Given that Mr. Miller has maintained a deep molecular response (MR4.5), the most probable cause of new Grade 2 cytopenias is the drug itself rather than immediate disease progression (though progression must always be considered if counts don't recover). The standard first step in managing suspected moderate drug toxicity is to interrupt the causative agent and monitor for resolution. If the counts recover after holding Dasatinib, it strongly suggests drug-induced myelosuppression, which can then be managed by restarting at a lower dose or potentially the same dose with closer monitoring, depending on the speed and degree of recovery.
 * **Guideline Approach:** Management algorithms for TKI toxicities recommend dose interruption for Grade 2 or higher non-hematologic or hematologic toxicities (that are not brief/transient).

* **Why other options are less appropriate *initially*:**
 * **A (Immediate Bone Marrow Biopsy):** While a bone marrow biopsy is important if cytopenias persist after holding the TKI or if there's suspicion of progression/transformation (e.g., rising *BCR-ABL1* levels, appearance of blasts), it's generally not the *first* step for new Grade 2 cytopenias in a patient with stable DMR. Holding the drug first is less invasive and often diagnostic.
 * **B (Empiric Switch):** Switching TKIs without confirming the cause or attempting dose adjustment is premature. If the cytopenia is Dasatinib-related and manageable with dose modification, switching exposes the patient to a new set of potential toxicities unnecessarily.
 * **D (Add G-CSF/Platelet Agent):** Adding growth factors or thrombopoietin receptor agonists treats the symptom (low counts) without addressing the likely cause (drug toxicity). The primary approach should be managing the causative TKI first (hold/reduce dose). These agents might be considered later if cytopenias are persistent, severe, or dose-limiting despite TKI adjustments, but not as the initial step for Grade 2.

You have successfully navigated all three phases of this CML case, from diagnosis and risk stratification through first-line therapy selection, toxicity management, response monitoring, and long-term considerations.

Session ID:
Assessment Domain

Session-57:
Rationale

Session ID:
Instructional Design and Pedagogical

Session-57:
The session's instructional design is of very high quality. The case is expertly scaffolded, moving seamlessly from a classic CML-CP diagnosis through risk stratification, first-line therapy selection, acute and long-term toxicity management, and advanced topics like treatment-free remission. The embedded feedback is a major strength, providing detailed, guideline-based rationales (citing ELN/ESMO) that explain not only the correct answer but also why the distractors are incorrect. The instructional framing is exceptionally clear, with each step logically following the last. The only minor area for improvement is the clarity of learning objectives; while one is stated mid-session, overarching objectives for the entire case are not explicitly defined at the beginning.

Session ID:
Clinical Authenticity and Content Fidelity

Session-57:
The clinical fidelity of this simulation is excellent and represents a gold standard for this type of educational material. Plausibility & Guideline Alignment: The clinical narrative is indistinguishable from an authentic CML case, from initial presentation to the nuances of long-term management. All diagnostic steps, risk calculations (Sokal), monitoring milestones (MMR), and therapeutic decisions are perfectly aligned with current, evidence-based guidelines from bodies like ELN and ESMO. Pharmacotherapeutic Accuracy: The pharmacotherapeutic choices are optimal and expertly justified. The selection of a second-generation TKI is appropriate for an intermediate-risk patient, and the discussion correctly weighs the risk/benefit profiles of different agents (e.g., avoiding Nilotinib due to cardiovascular history). The management of adverse effects, such as the dose-dependent pleural effusion and late-onset cytopenias, reflects standard-of-care best practices (hold, monitor, restart at a reduced dose). Domain Specificity: The case remains strictly within the domain of Chronic Myeloid Leukemia. All diagnostic tests, therapeutic agents, and management concepts are appropriate for CML, with no evidence of domain entanglement.

Session ID:
Modeled Clinical Reasoning

Session-57:
The simulation flawlessly models an expert clinical reasoning process. Each stage presents a well-defined clinical problem with exactly the right amount of information needed to make a decision. The multiple-choice options are well-crafted, providing a range of plausible and relevant alternatives that effectively test nuanced understanding (e.g., the subtle differences between second-generation TKIs). The case demonstrates expert-level prioritization throughout, from managing the initial risk of Tumor Lysis Syndrome to the cautious, evidence-based approach to a potential treatment-free remission attempt. The described clinical outcomes following each decision are realistic and highly instructive, reinforcing the key learning points.

Session-58

Session ID:
LLM

Session-58:
Google AI Studio (Gemini 2.0 Pro)

Session ID:
Session Transcript

Session-58:
Excellent, [REDACTED]]. Let's begin with Phase 1: Initial Presentation and Diagnostic Workup.

Patient Case - Phase 1

Mr. David Miller, a 58-year-old male construction worker, presents to his primary care physician complaining of fatigue, unintentional weight loss (15 lbs over 3 months), and early satiety for the past month. He also notes occasional night sweats. He has a past medical history significant for well-controlled hypertension (treated with lisinopril 10 mg daily) and hyperlipidemia (treated with atorvastatin 20 mg daily). He has no known allergies.

Initial Vitals & Physical Exam:

BP: 135/85 mmHg

HR: 88 bpm

RR: 16 breaths/min

Temp: 98.9F (37.2C)

Physical exam reveals splenomegaly (spleen palpable 5 cm below the left costal margin). No lymphadenopathy is noted.

Initial Laboratory Results:

CBC:

WBC: 115 x 10/L (Normal: 4.5-11.0 x 10/L)

Hemoglobin: 10.8 g/dL (Normal: 13.5-17.5 g/dL)

Platelets: 550 x 10/L (Normal: 150-450 x 10/L)

Differential: Myelocytes 15%, Metamyelocytes 10%, Bands 8%, Neutrophils 50%, Lymphocytes 5%, Monocytes 2%, Eosinophils 4%, Basophils 6% (Normal: <1% Basophils)

Peripheral Blood Smear: Shows marked leukocytosis with a left shift, including blasts (<2%), promyelocytes, myelocytes, metamyelocytes, and increased basophils.

Chemistry Panel:

Creatinine: 0.9 mg/dL (Normal: 0.7-1.3 mg/dL)

Uric Acid: 8.5 mg/dL (Normal: 3.4-7.0 mg/dL)

LDH: 450 U/L (Normal: 140-280 U/L)

Liver Function Tests: Within normal limits.

Question 1:

Based on Mr. Miller's initial presentation and laboratory findings, which of the following tests is MOST crucial at this stage to confirm the suspected diagnosis of Chronic Myeloid Leukemia (CML) according to WHO criteria?

A) Bone marrow biopsy with morphology assessment only.
B) Cytogenetic analysis (karyotyping) of peripheral blood or bone marrow for the Philadelphia chromosome (Ph).
C) JAK2 V617F mutation testing.
D) Flow cytometry of peripheral blood to assess lymphocyte populations.
B) Cytogenetic analysis (karyotyping) of peripheral blood or bone marrow for the Philadelphia chromosome (Ph).
Excellent choice, [REDACTED]]. Your selection of B) Cytogenetic analysis (karyotyping) of peripheral blood or bone marrow for the Philadelphia chromosome (Ph) is correct.

Rationale:

Correct Answer (B): The hallmark of CML is the presence of the Philadelphia chromosome (Ph), which results from the reciprocal translocation t(9;22)(q34.1;q11.2). This translocation creates the BCR::ABL1 fusion gene. Detecting either the Ph chromosome via conventional cytogenetics (karyotyping) or the BCR::ABL1 fusion gene/transcript via molecular methods (FISH or RT-PCR) is essential for confirming the diagnosis of CML, as mandated by the WHO classification system (Arber et al., Blood, 2022). While both peripheral blood and bone marrow can be used, bone marrow aspirate is preferred for conventional cytogenetics to get the best metaphase spreads and also allows for morphological assessment and blast count.

Incorrect Options:

A) Bone marrow biopsy with morphology: While essential for assessing cellularity, fibrosis, and blast percentage (crucial for determining the CML phase - chronic, accelerated, or blast phase), morphology alone cannot confirm the specific diagnosis of CML without identifying the underlying genetic marker.

C) JAK2 V617F mutation testing: This mutation is primarily associated with other Philadelphia-negative myeloproliferative neoplasms (MPNs) like polycythemia vera, essential thrombocythemia, and primary myelofibrosis. It is not characteristic of CML.

D) Flow cytometry: While flow cytometry can help characterize the myeloid cells and rule out other conditions, it is not the primary diagnostic modality for CML, which relies on genetic confirmation.

Next Steps in Workup:

Mr. Miller undergoes a bone marrow biopsy and aspirate.

Bone Marrow Morphology: Hypercellular marrow (95% cellularity) with marked granulocytic hyperplasia and increased megakaryocytes. Blasts are estimated at 3%. No significant dysplasia or fibrosis noted.

Cytogenetics (Karyotyping) on Bone Marrow: 46,XY,t(9;22)(q34.1;q11.2)[20/20 metaphases]. The Philadelphia chromosome is present in all analyzed cells. No additional chromosomal abnormalities (ACAs) are detected.

Molecular Testing (RT-PCR) on Peripheral Blood: Detects the p210 BCR::ABL1 transcript (e14a2 isoform). Quantitative baseline level is 55% (IS - International Scale).

These results confirm the diagnosis of Chronic Myeloid Leukemia (CML), Chronic Phase (CP).

Now, let's determine Mr. Miller's risk status.

Question 2:

Based on the available clinical and diagnostic data for Mr. Miller, which risk stratification score is recommended by current ESMO/ELN guidelines for predicting prognosis and guiding initial therapy selection in CML-CP?

A) Sokal Score
B) Euro/Hasford Score
C) ELTS (EUTOS Long-Term Survival) Score
D) IPSS-R (Revised International Prognostic Scoring System)
C) ELTS (EUTOS Long-Term Survival) Score
That is correct, [REDACTED]]. The C) ELTS (EUTOS Long-Term Survival) Score is the preferred risk stratification system recommended by the current ESMO and ELN guidelines for newly diagnosed CML-CP patients.

Rationale:

Correct Answer (C): The ELTS score was developed using data from patients treated with tyrosine kinase inhibitors (TKIs), specifically imatinib, making it more relevant in the modern treatment era compared to older scores. It effectively predicts the probability of dying from CML. The score incorporates age, spleen size, peripheral blood blast percentage, and platelet count. Current ESMO guidelines (Hochhaus et al., Ann Oncol, 2020) explicitly recommend using the ELTS score at diagnosis to categorize patients into low, intermediate, or high risk, which can inform treatment intensity discussions.

Incorrect Options:

A) Sokal Score & B) Euro/Hasford Score: These scores were developed earlier (chemotherapy and interferon eras, respectively). While historically significant and sometimes still calculated, they are considered less predictive of outcomes with modern TKI therapy than the ELTS score.

D) IPSS-R: This scoring system is used for risk stratification in Myelodysplastic Syndromes (MDS), not CML.

Applying ELTS to Mr. Miller:

The ELTS score is calculated based on:

Age: 58 years

Spleen size: 5 cm below costal margin

Platelet count: 550 x 10/L

Peripheral Blood Blasts: <2% (Let's use 1% for calculation based on smear description)

Based on these parameters, Mr. Miller's ELTS score places him in the Low Risk category.

Now that we have confirmed the diagnosis and established Mr. Miller's risk profile, we need to consider initial management, including addressing his high uric acid level.

Question 3:

Mr. Miller has an elevated uric acid level (8.5 mg/dL) and a high tumor burden (WBC 115 x 10/L, splenomegaly). What is the most appropriate initial pharmacologic intervention to prevent tumor lysis syndrome (TLS) complications before starting CML-specific therapy?

A) Initiate allopurinol 300 mg daily.
B) Start rasburicase 0.2 mg/kg intravenously.
C) Begin aggressive intravenous hydration only.
D) Administer sodium bicarbonate intravenously to alkalinize urine.
A) Initiate allopurinol 300 mg daily.
Okay, [REDACTED]], let's move into Phase 2: Induction Therapy Selection and Supportive Care Planning.

Mr. Miller has been diagnosed with ELTS Low Risk CML-CP. You have initiated allopurinol 300 mg daily for TLS prophylaxis and ensured he is adequately hydrated. Now, the primary goal is to select the most appropriate first-line Tyrosine Kinase Inhibitor (TKI) to induce remission.

Current ESMO/ELN guidelines recommend several TKIs as potential first-line options for CML-CP:

Imatinib (a first-generation TKI)

Dasatinib (a second-generation TKI)

Nilotinib (a second-generation TKI)

Bosutinib (a second-generation TKI)

Question 4:

Considering Mr. Miller is 58 years old, ELTS Low Risk, with well-controlled hypertension and hyperlipidemia, which of the following initial TKI treatment strategies is MOST strongly supported by current guidelines for achieving optimal long-term outcomes, including deep molecular responses (which may allow for future treatment-free remission attempts)?

A) Standard-dose Imatinib (400 mg daily) is the only appropriate choice due to his low-risk status.
B) A second-generation TKI (Dasatinib, Nilotinib, or Bosutinib) should be prioritized over Imatinib to achieve faster and deeper molecular responses.
C) Any of the approved first-line TKIs (Imatinib, Dasatinib, Nilotinib, Bosutinib) are acceptable initial choices, with the final selection depending on patient comorbidities and side effect profile discussions.
D) High-dose Imatinib (e.g., 800 mg daily) should be considered to maximize response in low-risk disease.
C) Any of the approved first-line TKIs (Imatinib, Dasatinib, Nilotinib, Bosutinib) are acceptable initial choices, with the final selection depending on patient comorbidities and side effect profile discussions.
Excellent, [REDACTED]]. Your choice, C) Any of the approved first-line TKIs (Imatinib, Dasatinib, Nilotinib, Bosutinib) are acceptable initial choices, with the final selection depending on patient comorbidities and side effect profile discussions, is the most accurate representation of current guideline recommendations.

Rationale:

Correct Answer (C): The ESMO guidelines (Hochhaus et al., Ann Oncol, 2020) clearly state that imatinib, dasatinib, nilotinib, and bosutinib are all standard-of-care first-line options for newly diagnosed CML-CP, irrespective of the ELTS risk score. While second-generation TKIs (2GTKIs) generally offer faster and deeper molecular responses, which can be advantageous (especially if treatment-free remission is a long-term goal), the choice must be individualized. Key factors include the patient's specific comorbidities, potential drug interactions, and the distinct adverse effect profiles of each TKI. For Mr. Miller, his history of hypertension and hyperlipidemia should be considered when weighing the pros and cons of each agent (e.g., nilotinib's association with cardiovascular events and hyperglycemia, dasatinib's potential for pleural effusions, bosutinib's gastrointestinal and liver toxicity profile, and imatinib's general tolerability profile but potentially slower response rates).

Incorrect Options:

A) Imatinib only: This is too restrictive. While imatinib is a perfectly valid and effective option for low-risk patients, it's not the only

choice according to guidelines.

B) Prioritize 2GTKIs: While 2GTKIs offer faster/deeper responses, guidelines do not mandate their prioritization over imatinib solely based on this, especially in low-risk CML where imatinib provides excellent long-term control for many. The decision is more nuanced.

D) High-dose Imatinib: Standard first-line dosing for imatinib in CML-CP is 400 mg daily. Higher doses are generally reserved for specific situations like lack of response or advanced phase disease and are associated with increased toxicity.

Treatment Selection Discussion:

You discuss the options with Mr. Miller. You explain that all four drugs are effective but have different potential side effects:

Imatinib: Generally well-tolerated; common side effects include nausea, muscle cramps, fluid retention (periorbital edema), rash. Lower rate of achieving deep molecular responses compared to 2GTKIs.

Dasatinib: Potential for pleural effusions (fluid around lungs), potential effects on platelet function. Fast acting.

Nilotinib: Requires fasting around doses. Carries a Black Box Warning for QTc prolongation and risk of sudden death. Associated with vascular events (peripheral artery disease, stroke, heart attack) and hyperglycemia/pancreatitis.

Bosutinib: Common side effects include diarrhea and nausea (often early and manageable), potential for liver enzyme elevation. Less associated with vascular events or significant fluid retention compared to nilotinib/dasatinib.

Considering Mr. Miller's cardiovascular risk factors (hypertension, hyperlipidemia), nilotinib might pose a higher relative risk for vascular complications. After discussing the benefits and risks, Mr. Miller expresses a preference to try and avoid significant fluid retention or cardiovascular risks if possible. You mutually decide to initiate therapy with Dasatinib 100 mg once daily.

Question 5:

Now that Dasatinib has been initiated, which of the following baseline assessments and ongoing monitoring parameters are MOST critical specifically related to Dasatinib's known safety profile?

A) Baseline and periodic assessment of fasting blood glucose and lipid panel.
B) Baseline and periodic assessment for peripheral edema and muscle cramps.
C) Baseline ECG to assess QTc interval and ongoing monitoring for signs/symptoms of pancreatitis.
D) Baseline chest X-ray and ongoing monitoring for respiratory symptoms (e.g., cough, dyspnea, pleuritic chest pain) suggestive of pleural effusion.
D) Baseline chest X-ray and ongoing monitoring for respiratory symptoms (e.g., cough, dyspnea, pleuritic chest pain) suggestive of pleural effusion.
Correct again, [REDACTED]]. Your selection of D) Baseline chest X-ray and ongoing monitoring for respiratory symptoms (e.g., cough, dyspnea, pleuritic chest pain) suggestive of pleural effusion is the most critical monitoring specific to Dasatinib.

Rationale:

Correct Answer (D): Pleural effusion is a known, significant, and relatively common adverse effect associated with Dasatinib therapy. Incidence varies but can occur in a substantial portion of patients. Therefore, establishing a baseline chest X-ray (or assessing a recent one) and vigilant ongoing monitoring for any respiratory symptoms are crucial for early detection and management. Management may involve dose interruption, reduction, diuretics, or corticosteroids depending on severity.

Incorrect Options:

A) Fasting blood glucose and lipid panel: While important general health monitoring (especially given Mr. Miller's hyperlipidemia), significant hyperglycemia and dyslipidemia are more characteristic concerns with Nilotinib.

B) Peripheral edema and muscle cramps: These are more commonly associated with Imatinib, although some fluid retention can occur with Dasatinib, it's generally less frequent/severe than pleural effusions or the periorbital edema seen with Imatinib.

C) Baseline ECG for QTc and pancreatitis monitoring: QTc prolongation is a specific concern requiring baseline and periodic ECGs for Nilotinib (Black Box Warning). Pancreatitis is also a known risk with Nilotinib. While Dasatinib can potentially affect the QTc interval slightly, it's not considered a primary concern like with Nilotinib.

Supportive Care Considerations with Dasatinib:

Drug Interactions: Dasatinib absorption is pH-dependent. H2-receptor antagonists (like famotidine) should be administered at least 10 hours before or 2 hours after dasatinib. Proton pump inhibitors (PPIs) should generally be avoided if possible; if necessary, they should be taken 12 hours before the dasatinib dose, but concurrent use is strongly discouraged due to significant reduction in dasatinib exposure. Antacids should be taken 2 hours before or 2 hours after dasatinib.

Monitoring: Regular CBCs are essential to monitor for myelosuppression (neutropenia, thrombocytopenia, anemia). Chemistry panels, including liver function tests and electrolytes, should also be monitored.

Mr. Miller starts Dasatinib 100 mg daily and continues allopurinol for the first few weeks.

Three Months Later:

Mr. Miller returns for follow-up. He reports good tolerance to Dasatinib with only mild fatigue.

CBC: WBC 8.5 x 10/L, Hgb 12.5 g/dL, Plt 220 x 10/L. Differential is normalized.

Physical Exam: Spleen is no longer palpable.

Molecular Monitoring (RT-PCR on Peripheral Blood): BCR::ABL1 transcript level is 0.5% (IS).

Question 6:

According to the ELN 2020 recommendations, how would you classify Mr. Miller's response to Dasatinib at 3 months based on his BCR::ABL1 transcript level of 0.5% (IS)?

A) Optimal Response
B) Warning
C) Failure
D) Major Molecular Response (MMR)
A) Optimal Response
That is correct, [REDACTED]]! Mr. Miller's response at 3 months is classified as A) Optimal Response.

Rationale:

Correct Answer (A): According to the European LeukemiaNet (ELN) 2020 recommendations for monitoring CML treatment response, achieving a BCR::ABL1 transcript level of 10% (IS) at the 3-month time point is defined as an Optimal Response. Mr. Miller's level of 0.5% (IS) comfortably meets this criterion. Additionally, he has achieved a Complete Hematologic Response (CHR), characterized by normalized blood counts and resolution of splenomegaly, which is also expected by 3 months for an optimal response.

Incorrect Options:

B) Warning: A "Warning" at 3 months would be defined by a BCR::ABL1 level > 10% (IS), but usually accompanied by CHR. Mr. Miller is well below this threshold.

C) Failure: "Failure" at 3 months would be defined as not achieving CHR or having persistently high BCR::ABL1 levels (>10% IS

without significant improvement), potentially indicating resistance. Mr. Miller meets neither criterion.

D) Major Molecular Response (MMR): MMR (also known as MR3) is defined as a BCR::ABL1 level of 0.1% (IS). While achieving MMR is a key treatment goal, Mr. Miller has not yet reached this deeper level of response at 3 months. His current response is optimal for this time point but not yet MMR.

Clinical Significance: Achieving an optimal response at 3 months is a strong predictor of favorable long-term outcomes, including a higher probability of achieving deeper molecular responses like MMR later on.

Six Months Later (9 months total on Dasatinib):

Mr. Miller continues on Dasatinib 100 mg daily. He mentions developing intermittent mild shortness of breath, especially with exertion, over the past month. He denies chest pain or cough.

CBC: Stable (WBC 7.8, Hgb 13.0, Plt 200).

Molecular Monitoring (RT-PCR): BCR::ABL1 transcript level is 0.08% (IS).

Physical Exam: Vital signs stable, O2 saturation 96% on room air. Faint crackles heard at the left lung base. No peripheral edema.

Question 7:

Given Mr. Miller's new symptom of dyspnea and the lung finding, along with achieving Major Molecular Response (MMR = BCR::ABL1 0.1% IS), what is the most appropriate next step in management?

A) Reassure the patient that this is likely unrelated to Dasatinib and continue current therapy.
B) Increase the Dasatinib dose to 140 mg daily to target a deeper response.
C) Perform a chest X-ray and temporarily hold Dasatinib while investigating potential pleural effusion.
D) Switch therapy immediately to Nilotinib due to Dasatinib intolerance.
C) Perform a chest X-ray and temporarily hold Dasatinib while investigating potential pleural effusion.
Alright, [REDACTED]], let's move into Phase 3: Post-Remission Therapy, MRD Monitoring, and Long-Term Management.

Two Years After Starting Dasatinib:

Mr. Miller, now 60 years old, has been on Dasatinib 70 mg daily (the reduced dose after his pleural effusion) for the past year and a half. He tolerates this dose well with only mild fatigue and no recurrence of respiratory symptoms.

Clinical Status: Remains in Complete Hematologic Remission (CHR).

Molecular Monitoring (MRD): His BCR::ABL1 transcript levels (measured by RT-PCR every 3-6 months) have consistently been very low. For the past 12 months, results have been stable at 0.01% (IS). His most recent result is 0.008% (IS).

Question 8:

Mr. Miller's consistent BCR::ABL1 transcript level of 0.01% (IS) signifies the achievement of which molecular response milestone according to standard international definitions?

A) Complete Molecular Response (CMR)
B) Major Molecular Response (MMR / MR3)
C) Deep Molecular Response (DMR), specifically MR4
D) Molecular Response 2 (MR2)
C) Deep Molecular Response (DMR), specifically MR4
Excellent, [REDACTED]]. You are correct. Mr. Miller's consistent BCR::ABL1 level of 0.01% (IS) represents C) Deep Molecular Response (DMR), specifically MR4.

Rationale:

Correct Answer (C): Molecular response milestones are defined by the logarithmic reduction of BCR::ABL1 transcripts compared to a standardized baseline:

MMR (MR3): 0.1% (IS) (3-log reduction)

MR4: 0.01% (IS) (4-log reduction)

MR4.5: 0.0032% (IS) (4.5-log reduction)

MR5: 0.001% (IS) (5-log reduction)
Deep Molecular Response (DMR) is often used to describe responses of MR4 or deeper (MR4.5, MR5). Since Mr. Miller's level is consistently 0.01% (specifically 0.008%), he has achieved MR4.

Incorrect Options:

A) Complete Molecular Response (CMR): CMR typically refers to undetectable BCR::ABL1 levels by highly sensitive assays (often implying MR4.5 or MR5 depending on the assay's limit of detection). Mr. Miller still has detectable, albeit very low, levels.

B) Major Molecular Response (MMR / MR3): This is a less deep response (0.1% IS). Mr. Miller has surpassed this milestone.

D) Molecular Response 2 (MR2): This corresponds to 1% (IS) (2-log reduction). He is far below this level.

Significance of DMR:

Achieving a stable DMR (like MR4 or deeper) for a sustained period (typically 2 years) is a prerequisite for considering Treatment-Free Remission (TFR) attempts, according to current guidelines and clinical trial criteria (Hochhaus et al., Ann Oncol, 2020; Ra et al., Blood, 2017).

Treatment-Free Remission Discussion:

Mr. Miller expresses interest in potentially stopping his Dasatinib therapy in the future, having heard about TFR. He asks about his eligibility.

Question 9:

Based on current ESMO/ELN guidelines and typical TFR trial criteria, which of the following factors is the MOST important prerequisite Mr. Miller must meet before a TFR attempt could be considered?

A) Must be ELTS Low Risk at diagnosis.
B) Must have been treated with a second-generation TKI.
C) Must have sustained DMR (e.g., MR4 or deeper) for a minimum duration (typically 2 years).
D) Must have no history of TKI dose reduction due to side effects.
C) Must have sustained DMR (e.g., MR4 or deeper) for a minimum duration (typically 2 years).
Absolutely correct, [REDACTED]]. The single MOST important prerequisite is C) Must have sustained DMR (e.g., MR4 or deeper) for a minimum duration (typically 2 years).

Rationale:

Correct Answer (C): This is the fundamental criterion for considering TFR. The depth (MR4 [0.01% IS], MR4.5 [0.0032% IS], or sometimes MR5 [0.001% IS], depending on specific trial/guideline nuances) and the stability (duration) of that response are the strongest predictors of successfully remaining off therapy without molecular relapse. The commonly accepted minimum duration of

sustained DMR is 2 years, though some recommendations suggest longer periods (e.g., 3 years) may further increase success rates. (Hochhaus et al., Ann Oncol, 2020; Ra et al., Blood, 2017; Sauele et al., Blood, 2018).

Incorrect Options:

A) ELTS Low Risk: TFR can be attempted regardless of initial risk score, provided the molecular response criteria are met.

B) Second-generation TKI: TFR is possible after any TKI, including imatinib, although achieving the necessary DMR depth/duration may take longer with imatinib for some patients. The eligibility is based on response, not the specific first-line drug used.

D) No history of dose reduction: While TFR criteria often specify the TKI dose the patient was on prior to stopping, a history of dose reduction does not inherently disqualify a patient if they achieved and maintained stable DMR on the reduced dose (like Mr. Miller on Dasatinib 70mg).

Mr. Miller's Situation:

Mr. Miller has achieved MR4 (0.008% IS) and has maintained MR4 or deeper (0.01% IS) for 12 months. He would need to maintain this level of response for at least another 12 months (total 2 years) before a TFR attempt could be seriously considered under current recommendations.

Five Years After Starting Dasatinib (Patient is now 63):

Mr. Miller has remained on Dasatinib 70 mg daily. He has successfully maintained MR4.5 (BCR::ABL1 0.0032% IS) for the past 3 years. After extensive discussion about the risks (approximately 50% chance of molecular relapse requiring TKI restart) and the rigorous monitoring required (monthly PCRs initially), he elects to attempt TFR.

He stops Dasatinib. His BCR::ABL1 levels are monitored monthly.

Month 1: Undetectable

Month 2: 0.005% (IS)

Month 3: 0.05% (IS)

Month 4: 0.15% (IS)

Question 10:

Based on Mr. Miller's BCR::ABL1 monitoring results after stopping Dasatinib, what is the most appropriate action according to standard TFR management protocols?

A) Continue monitoring monthly; only restart TKI if symptoms develop.
B) Restart Dasatinib immediately at the previous dose (70 mg daily) due to loss of Major Molecular Response (MMR).
C) Switch to a different TKI, such as Nilotinib or Bosutinib.
D) Perform a bone marrow biopsy and cytogenetics to assess for disease progression.
B) Restart Dasatinib immediately at the previous dose (70 mg daily) due to loss of Major Molecular Response (MMR).

Session ID:
Assessment Domain

Session-58:
Rationale

Session ID:
Instructional Design and Pedagogical

Session-58:
The session's instructional design is excellent. The case is expertly scaffolded, progressing logically from initial diagnosis and risk stratification (Q1-2) through first-line therapy selection (Q4), management of adverse events (Q7), and complex long-term management challenges like Treatment-Free Remission (TFR) (Q9-10). The quality of the embedded feedback is a key strength; each rationale is detailed, cites current clinical guidelines (e.g., WHO, ESMO, ELN), and accurately explains why incorrect options are wrong. Instructions are exceptionally clear. The only minor weakness is that learning objectives are not explicitly stated at the outset, though they are strongly implied by the session's structure and content.

Session ID:
Clinical Authenticity and Content Fidelity

Session-58:
The session demonstrates exceptional clinical authenticity and content fidelity.
Plausibility & Guideline Alignment: The clinical narrative is highly plausible, mirroring a textbook case of CML from presentation to long-term management. All diagnostic steps, therapeutic decisions, and monitoring milestones (e.g., using ELTS score, defining Optimal Response at 3 months, criteria for TFR) are perfectly aligned with current, evidence-based guidelines.
Pharmacotherapeutic Accuracy: The pharmacotherapeutic recommendations are optimal and well-justified throughout the case. This includes appropriate TLS prophylaxis with allopurinol, individualized TKI selection considering comorbidities, correct management of a Dasatinib-induced pleural effusion, and standard-of-care management of molecular relapse post-TFR. There are no content-level errors.
Domain Specificity: The case remains strictly within the CML domain. All diagnostics, therapeutics, and management principles discussed are specific and appropriate for CML, with no evidence of domain entanglement. Distractors from other disease states (e.g., IPSS-R for MDS) are correctly used and identified as incorrect.

Session ID:
Modeled Clinical Reasoning

Session-58:
The simulation excels at modeling an expert clinical reasoning process. Each stage of the case presents a well-defined clinical problem with the necessary data for a decision. The multiple-choice options provide an excellent range of plausible and relevant alternatives, including key distractors that effectively test nuanced understanding (e.g., distinguishing between different TKI side effect profiles). The narrative demonstrates sound, risk-adjusted prioritization at every step, from initial workup to managing treatment complications and TFR. The clinical outcomes described for each decision are realistic, specific, and highly instructive, reinforcing the key learning objectives of the session.

Session-59

Session ID:
LLM

Session-59:
Google AI Studio (Gemini 2.0 Pro)

Session ID:
Session Transcript

Session-59:
Excellent. Let's begin with our first case:
Case 1: Newly Diagnosed Acute Myeloid Leukemia (AML)
A 67-year-old male presents to the emergency department with a one-week history of increasing fatigue, easy bruising, and recurrent low-grade fevers. His past medical history is significant for well-controlled hypertension and hyperlipidemia.
Initial laboratory findings include:
 * White blood cell count (WBC): 150,000/L (normal: 4,000-10,000/L)
 * Hemoglobin (Hgb): 8.5 g/dL (normal: 13.5-17.5 g/dL)
 * Platelet count: 25,000/L (normal: 150,000-400,000/L)
 * Peripheral blood smear shows 70% blasts.
Bone marrow biopsy is performed and confirms the diagnosis of AML with monocytic differentiation. Cytogenetic analysis reveals a normal karyotype. Molecular testing is pending.
Question 1: Based on the initial clinical and laboratory findings, what is the most appropriate initial management strategy for this patient?
a) Observation with supportive care
b) Single-agent oral chemotherapy with hydroxyurea
c) Combination chemotherapy with cytarabine and an anthracycline
d) Enrollment in a clinical trial investigating novel targeted therapies

Treatment Options Analysis:

- **a) Single-agent high-dose cytarabine**: Not appropriate for induction. High-dose cytarabine is typically reserved for **consolidation** therapy, not induction, and using it alone in induction is less effective.

- **b) 7+3 regimen (cytarabine + daunorubicin)**: This is the **standard of care** for fit patients under ~75 years old with newly diagnosed AML. It has the most evidence for efficacy in achieving CR in this setting.

- **c) Cytarabine + gemtuzumab ozogamicin**: Gemtuzumab (anti-CD33) is sometimes **added to 7+3** in favorable-risk patients, particularly those with **core-binding factor leukemias**. This patient does **not** have favorable cytogenetics or CD33+ disease confirmed, so not the first choice.

- **d) Low-dose cytarabine with venetoclax**: This is a good option for **older, unfit patients** or those who can't tolerate intensive therapy. Our patient is 67 but **clinically stable and fit**, so intensive induction is preferred.

Correct Answer:
b) Combination of cytarabine (7 days) and daunorubicin (3 days) ["7+3"]

This is the standard induction regimen for newly diagnosed AML in a fit patient without actionable mutations or contraindications.

 2: Based on these updated findings, what is the most appropriate induction chemotherapy regimen for this patient?

a) Single-agent cytarabine (high-dose)
b) Combination of cytarabine (7 days) and daunorubicin (3 days) ["7+3"]
c) Combination of cytarabine and gemtuzumab ozogamicin
d) Low-dose cytarabine with venetoclax

Treatment Options Analysis:

- **a) Single-agent high-dose cytarabine**: Not appropriate for induction. High-dose cytarabine is typically reserved for **consolidation** therapy, not induction, and using it alone in induction is less effective.

- **b) 7+3 regimen (cytarabine + daunorubicin)**: This is the **standard of care** for fit patients under ~75 years old with newly diagnosed AML. It has the most evidence for efficacy in achieving CR in this setting.

- **c) Cytarabine + gemtuzumab ozogamicin**: Gemtuzumab (anti-CD33) is sometimes **added to 7+3** in favorable-risk patients, particularly those with **core-binding factor leukemias**. This patient does **not** have favorable cytogenetics or CD33+ disease confirmed, so not the first choice.

- **d) Low-dose cytarabine with venetoclax**: This is a good option for **older, unfit patients** or those who can't tolerate intensive therapy. Our patient is 67 but **clinically stable and fit**, so intensive induction is preferred.

Correct Answer:
b) Combination of cytarabine (7 days) and daunorubicin (3 days) ["7+3"]

.
Question 3: Based on these results, what is the most appropriate next step in the management of this patient?
a) Observation with regular blood count monitoring
b) Consolidation chemotherapy
c) Allogeneic hematopoietic stem cell transplantation (allo-HSCT)
d) Enrollment in a maintenance therapy clinical trial

- **a) Observation with monitoring**: Not appropriaterelapse risk without consolidation is high, and observation alone is insufficient even in morphologic remission.

- **b) Consolidation chemotherapy**: **Correct.** In patients <6070 years who achieve CR and have **intermediate-risk cytogenetics** (which this patient hasnormal karyotype, no FLT3/NPM1), **consolidation chemotherapy** is the standard next step. Common regimens include high-dose cytarabine (HiDAC), typically over several cycles.

- **c) Allogeneic HSCT**: This may be considered in **first CR** for patients with **high-risk features**, poor-risk mutations, or relapsed/refractory disease. In this patient with **intermediate risk**, transplant would typically be reserved for **relapse or MRD-positive disease**, or discussed case-by-case based on molecular markers or minimal residual disease (MRD) if available.

- **d) Maintenance therapy trial**: While this is an emerging area (especially with oral azacitidine in certain populations), it's **not standard** in intermediate-risk patients who are fit for consolidation and have achieved full count recovery.

Correct Answer:
b) Consolidation chemotherapy

Question 4: Based on these findings, what is the most appropriate long-term management strategy for this patient?
a) Observation with regular blood count monitoring
b) Enrollment in a clinical trial investigating maintenance therapy
c) Scheduled maintenance chemotherapy with low-dose chemotherapy agents
d) No further therapy is indicated as the patient is in MRD-negative complete remission after adequate consolidation

Option Breakdown:

- **a) Observation with regular blood count monitoring**:
  Correct. This is the **standard approach** for patients with intermediate-risk AML who are MRD-negative following standard consolidation. There's **no clear benefit** of routine maintenance chemotherapy in this setting. These patients are typically followed closely with regular clinic visits, blood counts, and marrow evaluations as indicated.

- **b) Enrollment in a clinical trial investigating maintenance therapy**:
  Reasonable but not **necessary** here. Trials like those investigating **oral azacitidine (CC-486)** have shown benefit primarily in patients **not achieving deep remission**, or those **ineligible for intensive therapy**. Your patient achieved MRD-negative remission and tolerated consolidation well, so maintenance trials may offer **limited additional benefit**.

- **c) Scheduled maintenance chemotherapy with low-dose agents**:
  Not recommended in this setting. Unlike in acute lymphoblastic leukemia (ALL), AML has **not shown consistent benefit** from scheduled low-dose maintenance in MRD-negative patients with intermediate-risk disease.

- **d) No further therapy is indicated**:
  This is technically true, but **incomplete** without the context of **ongoing surveillance**. Observation **with scheduled monitoring** is still a form of structured post-remission care.

Correct Answer:
a) Observation with regular blood count monitoring

Alright, [REDACTED]], let's move on to our second case.
Case 2: Relapsed/Refractory Acute Lymphoblastic Leukemia (ALL)
A 24-year-old female was diagnosed with B-cell precursor ALL three years ago. She achieved a complete remission after standard induction and consolidation chemotherapy and completed two years of maintenance therapy. Six months after completing maintenance, she presents with new onset fatigue, bone pain, and easy bruising.
Her laboratory findings now include:
 * White blood cell count (WBC): 5,500/L (normal: 4,000-10,000/L)
 * Hemoglobin (Hgb): 9.0 g/dL (normal: 12-16 g/dL)
 * Platelet count: 30,000/L (normal: 150,000-400,000/L)
 * Peripheral blood smear shows 15% blasts.
Bone marrow aspirate and biopsy confirm relapsed B-cell precursor ALL with 25% blasts. Cytogenetic analysis reveals a normal karyotype. Flow cytometry shows the leukemic blasts are CD19-positive and CD22-positive, but negative for the Philadelphia chromosome (BCR-ABL1).
Question 1: Based on these findings, what is the most appropriate initial treatment strategy for this patient with relapsed B-cell precursor ALL?
a) Re-induction chemotherapy with the original induction regimen
b) Palliative care and best supportive measures
c) Enrollment in a clinical trial investigating novel immunotherapies or targeted therapies
d) Allogeneic hematopoietic stem cell transplantation (allo-HSCT)

Treatment Strategy Breakdown:

- **a) Re-induction with the original regimen**:

Often **less effective** in relapse. Most relapsed ALLs are **resistant** to previously used chemotherapy. Modern practice uses **novel agents or different chemo backbones**, often in combination with immunotherapies.

- **b) Palliative care**:
  Not appropriate here. She is young, has potential curative options, and is not terminal at this stage.

- **c) Enrollment in a clinical trial investigating novel immunotherapies or targeted therapies**:
  Correct. Clinical trials evaluating **blinatumomab, inotuzumab ozogamicin**, or even **CAR-T cell therapies** are **highly appropriate** for patients with relapsed/refractory B-ALL. They are better tolerated than traditional chemo and offer higher remission rates, especially when targeted to CD19 or CD22.

- **d) Allo-HSCT**:
  This is **part of the curative strategy**, but **not the first step**. She must first achieve **another remission** (CR2) before proceeding to transplant. Transplant is the **goal** after achieving remission with re-induction or immunotherapy.

Correct Answer:
c) Enrollment in a clinical trial investigating novel immunotherapies or targeted therapies

Would you like to explore the comparative data on **blinatumomab vs inotuzumab**, or how **CAR-T cell therapy** fits into the treatment landscape for young adults with relapsed B-ALL?

Question 2: Based on this response to blinatumomab, what is the most appropriate next step in the management of this patient?
a) Continue blinatumomab for the planned duration per the clinical trial protocol
b) Proceed directly to allogeneic hematopoietic stem cell transplantation (allo-HSCT)
c) Consolidate with high-dose chemotherapy before considering allo-HSCT
d) Discontinue therapy and observe closely given the MRD-negative remission

Option Analysis:

- **a) Continue blinatumomab for the planned duration per the trial**:
  Temporarily reasonable, but **not the definitive next step**. While continued therapy per protocol is often encouraged in trials, **curative intent in young patients with relapsed ALL** usually involves **proceeding to allo-HSCT in MRD-negative CR2**. So while this may happen *short-term*, its not the ultimate management strategy.

- **b) Proceed directly to allogeneic HSCT**:
  Correct. The patient is in **second complete remission (CR2)** with **MRD-negativity**, which is the **optimal time** to proceed to transplant. **Allo-HSCT** offers the best chance for long-term disease control and potential cure in this setting. Blinatumomabs role here is to *bridge to transplant* by inducing deep remission, which it has done successfully.

- **c) Consolidate with high-dose chemotherapy before considering HSCT**:
  Not necessary. The patient is already MRD-negative after blinatumomab, so additional intensive chemotherapy would add toxicity without proven benefit. The focus now is **maintaining remission and proceeding to HSCT** while the patient is fit and disease-free.

- **d) Discontinue therapy and observe closely**:
  Not appropriate. Relapsed ALL, even in MRD-negative CR2, carries a high risk of recurrence without transplant. Observation alone would not offer curative potential.

Correct Answer:
b) Proceed directly to allogeneic hematopoietic stem cell transplantation (allo-HSCT)

Okay, let's continue with the management of this patient who is now proceeding to allogeneic hematopoietic stem cell transplantation (allo-HSCT) for relapsed B-cell precursor ALL in MRD-negative second remission.
The patient, now in MRD-negative CR2 after blinatumomab, is being prepared for allo-HSCT. A matched unrelated donor (MUD) has

been identified. Pre-transplant conditioning is planned.
Question 3: Which of the following is a commonly used conditioning regimen for allo-HSCT in patients with relapsed/refractory ALL?
a) High-dose melphalan
b) Busulfan and cyclophosphamide (BuCy)
c) Fludarabine and cyclophosphamide (FluCy)
d) Cyclophosphamide and total body irradiation (CyTBI)

Option Breakdown:

- **a) High-dose melphalan**:
  Used more in **multiple myeloma** or some **lymphomas**, not standard in ALL.

- **b) Busulfan and cyclophosphamide (BuCy)**:
  Common in **AML**, but **less effective** than CyTBI in ALL. Some centers may use **Busulfan + Flu** (BuFlu) as a reduced-intensity alternative in select patients, but not first-line here.

- **c) Fludarabine and cyclophosphamide (FluCy)**:
  Part of **reduced-intensity regimens** often used in older or frailer patientsnot the preferred myeloablative regimen in a fit 24-year-old.

- **d) Cyclophosphamide and TBI (CyTBI)**:
  Correct. This is the **standard myeloablative conditioning** for ALL, especially in **younger, fit patients with high-risk or relapsed disease.**

Correct Answer:
d) Cyclophosphamide and total body irradiation (CyTBI)

Alright, [REDACTED]], let's move on to our third and final case for today.
Case 3: Newly Diagnosed Chronic Myeloid Leukemia (CML) in Chronic Phase
A 53-year-old male presents to his primary care physician with complaints of left upper quadrant fullness and early satiety for the past month. He denies fever, night sweats, or significant weight loss. Physical examination reveals splenomegaly extending 5 cm below the left costal margin.
Initial laboratory findings include:
 * White blood cell count (WBC): 180,000/L (normal: 4,000-10,000/L) with a full differential showing all stages of myeloid maturation, including <5% blasts.
 * Hemoglobin (Hgb): 11.5 g/dL (normal: 13.5-17.5 g/dL)
 * Platelet count: 450,000/L (normal: 150,000-400,000/L)
A bone marrow aspirate and biopsy confirm the diagnosis of chronic myeloid leukemia (CML) in chronic phase. Cytogenetic analysis reveals the presence of the Philadelphia chromosome (t(9;22)(q34;q11.2)). BCR-ABL1 kinase domain mutation analysis is negative.
Question 1: Based on these findings, what is the most appropriate first-line treatment for this patient with newly diagnosed CML in chronic phase?
a) Observation with regular monitoring of blood counts
b) Single-agent chemotherapy with hydroxyurea to control leukocytosis
c) Interferon-alpha therapy
d) A tyrosine kinase inhibitor (TKI) such as imatinib, nilotinib, or dasatinib

Option Breakdown:

- **a) Observation with regular monitoring of blood counts**:
  Not appropriate. CML in chronic phase is **not self-limiting**, and without treatment, it will eventually progress to the **accelerated phase** or **blast crisis**. Treatment with a TKI is needed to **prevent disease progression**.

- **b) Single-agent chemotherapy with hydroxyurea to control leukocytosis**:

Hydroxyurea is typically used as **temporary management** to control **high leukocyte counts** in CML, but it does **not target the underlying molecular cause** (the BCR-ABL fusion gene). Hydroxyurea is **not a curative option** and should not be the first-line therapy for a newly diagnosed patient.

- **c) Interferon-alpha therapy**:
  Interferon-alpha was historically used to treat CML before the advent of **TKIs**, but it has been largely replaced due to **inferior efficacy**, **higher toxicity**, and **less favorable long-term outcomes** compared to TKIs.

- **d) A tyrosine kinase inhibitor (TKI) such as imatinib, nilotinib, or dasatinib**:
  Correct. First-line treatment for CML in chronic phase is a **tyrosine kinase inhibitor** (TKI). The standard first-line TKI is **imatinib** (a BCR-ABL1-specific TKI), but **nilotinib** and **dasatinib** are also approved and may be used in certain cases. These drugs are highly effective in suppressing CML and preventing progression to more aggressive disease phases.

Correct Answer:
d) A tyrosine kinase inhibitor (TKI) such as imatinib, nilotinib, or dasatinib

Question 2: Based on this early response to imatinib, what is the most appropriate next step in the management of this patient?
a) Increase the dose of imatinib
b) Continue imatinib at the current dose and monitor BCR-ABL1 levels every 3 months
c) Switch to a second-generation TKI (nilotinib or dasatinib)
d) Consider allogeneic hematopoietic stem cell transplantation (allo-HSCT)

Option Breakdown:

- **a) Increase the dose of imatinib**:
  Not indicated at this stage. This patient has shown an adequate early response to imatinib. Increasing the dose is typically considered in cases where there is an **inadequate response** or **suboptimal reduction** in BCR-ABL1 transcripts. Since this patient has had a good response, there's no need to escalate the dose.

- **b) Continue imatinib at the current dose and monitor BCR-ABL1 levels every 3 months**:
  Correct. This is the appropriate course of action. According to treatment guidelines, a **1 log reduction** in BCR-ABL1 after **3 months** of therapy is considered a **good early response**. The next step is to continue the current dose of imatinib and monitor BCR-ABL1 levels every **3 months**. Ongoing monitoring is crucial for detecting potential **treatment failure** or the development of **resistance**.

- **c) Switch to a second-generation TKI (nilotinib or dasatinib)**:
  Not necessary at this stage. A switch to a second-generation TKI is typically considered if there is **suboptimal response** (e.g., less than a 1 log reduction at 3 months) or if the patient has developed **resistance** or **intolerance** to imatinib. Since this patient has had a good response, continuing imatinib is appropriate.

- **d) Consider allogeneic hematopoietic stem cell transplantation (allo-HSCT)**:
  Not appropriate. In patients with chronic-phase CML who are responding well to a TKI, **allo-HSCT** is generally reserved for cases of **treatment resistance**, **relapse**, or **progression** to accelerated or blast phase. This patient is in **chronic phase** and has shown a good response, so transplantation is not indicated at this time.

Correct Answer:
b) Continue imatinib at the current dose and monitor BCR-ABL1 levels every 3 months

Question 3: Based on this sustained deep molecular response, what is the most appropriate long-term management strategy for this patient?

a) Continue imatinib indefinitely at the current dose
b) Consider attempting treatment-free remission (TFR) under strict monitoring
c) Reduce the dose of imatinib to the lowest effective dose
d) Switch to a second-generation TKI to further deepen the molecular response

Option Breakdown:

- **a) Continue imatinib indefinitely at the current dose**:
  This is a reasonable option, as the patient is well-controlled on imatinib. However, in patients who achieve sustained deep molecular responses, there may be opportunities to explore treatment-free remission (TFR), as ongoing TKI therapy can have long-term side effects. This option doesn't explore the possibility of TFR, which is a potential next step for this patient.

- **b) Consider attempting treatment-free remission (TFR) under strict monitoring**:
  Correct. This is the most appropriate long-term strategy for a patient with a sustained deep molecular response. The concept of **treatment-free remission (TFR)** has become a key management strategy for patients with chronic-phase CML who have maintained deep molecular responses (MMR or better) for several years. In such cases, the patient may be eligible for **discontinuation of imatinib** under **strict monitoring** for signs of relapse. If relapse occurs, treatment can be resumed promptly, making this approach a reasonable strategy. Recent trials have shown that some patients in sustained MMR can maintain remission off therapy, though regular monitoring is essential.

- **c) Reduce the dose of imatinib to the lowest effective dose**:
  While dose reduction may be an option for certain patients, it is not typically the standard approach for someone who has sustained MMR. Given the patient's **long-term stable response**, discontinuation or treatment-free remission (TFR) would be more appropriate than reducing the dose. Dose reduction is more often considered in the context of managing side effects rather than for patients in sustained molecular remission.

- **d) Switch to a second-generation TKI to further deepen the molecular response**:
  Switching to a second-generation TKI, such as nilotinib or dasatinib, is typically considered when patients fail to achieve an adequate response with imatinib or have **significant side effects**. Since this patient has already achieved a deep and sustained molecular response on imatinib, there is no need to switch to another TKI to deepen the response.

Correct Answer:
b) Consider attempting treatment-free remission (TFR) under strict monitoring

---

Session ID:
Assessment Domain

Session-59:
Rationale

Session ID:
Instructional Design and Pedagogical

Session-59:
The session's instructional design is of very high quality. The clinical cases are expertly scaffolded, each progressing logically through the natural history of the disease state (e.g., AML: diagnosis -> induction -> consolidation -> surveillance; CML: diagnosis -> initial response -> long-term management). The quality of the embedded feedback is a major strength; each rationale clearly explains the clinical thinking behind the correct answer while also debunking the distractors with sound, evidence-based reasoning. The instructions and case framing are exceptionally clear. The only minor weakness is that learning objectives are not explicitly stated at the outset, though they are strongly implied by the case structure.

Session ID:
Clinical Authenticity and Content Fidelity

Session-59:
The session demonstrates outstanding clinical authenticity and content fidelity. The clinical narratives for AML, ALL, and CML are highly plausible and reflect typical patient presentations. All therapeutic recommendations and management strategies are perfectly aligned with current, evidence-based clinical guidelines (e.g., NCCN, ELN). The pharmacotherapeutic choices are optimal and well-justified for each scenario presented. Critically, the session is free of any domain entanglement; therapies, diagnostics, and standards of care are appropriately confined to their respective diseases (e.g., AML therapies are used for AML, ALL therapies for ALL), ensuring the simulation is clinically safe and educationally sound. ALL is out of scope. The case should be either AML or CML.

Session ID:
Modeled Clinical Reasoning

Session-59:
The simulation expertly models a sophisticated clinical reasoning process. Each question presents a well-framed clinical problem that highlights a key decision point in management. The breadth of the multiple-choice options is a particular strength, providing not only the correct answer but also a range of highly plausible distractors that effectively test for nuanced understanding and common misconceptions. The decision-making process demonstrated (e.g., securing remission before transplant, monitoring response before altering therapy) reflects an appropriate, risk-adjusted prioritization of clinical goals. The described clinical outcomes are realistic and serve to reinforce the learning objectives effectively.

Session-60

Session ID:
LLM

Session-60:
Google AI Studio (Gemini 2.0 Pro)

Session ID:
Session Transcript

Session-60:

Phase 1: Initial Presentation and Diagnostic Workup
Patient: Mr. AC, a 52-year-old male, presents to his primary care physician complaining of increasing fatigue, unintentional weight loss (10 lbs over 3 months), and a feeling of fullness in his upper abdomen for the past month. He also reports occasional night sweats.
Past Medical History: Hypertension (controlled with lisinopril), Type 2 Diabetes Mellitus (controlled with metformin). No history of malignancy. Denies tobacco, alcohol, or illicit drug use.
Initial Physical Exam:
Vitals: BP 135/85 mmHg, HR 88 bpm, RR 16/min, Temp 37.1C (98.8F)
General: Appears fatigued but in no acute distress.
Abdomen: Palpable splenomegaly, extending 5 cm below the left costal margin. No hepatomegaly.
Lymphatics: No palpable lymphadenopathy.
Initial Laboratory Findings:
WBC: 155,000/L
Differential: Myelocytes 15%, Metamyelocytes 10%, Bands 5%, Neutrophils 50%, Basophils 8%, Eosinophils 5%, Lymphocytes 5%, Monocytes 2%, Blasts 2%
Hemoglobin: 10.5 g/dL
Platelets: 650,000/L
Peripheral Smear: Shows marked leukocytosis with left shift, including immature granulocytes (myelocytes, metamyelocytes), prominent basophilia, and occasional blasts.
(Question 1 of 10) Based on Mr. AC's initial presentation and laboratory findings, which of the following is the MOST likely diagnosis?
A) Acute Myeloid Leukemia (AML)
B) Chronic Lymphocytic Leukemia (CLL)
C) Chronic Myeloid Leukemia (CML)
D) Myelodysplastic Syndrome (MDS)

Feedback:
Your selection of C) Chronic Myeloid Leukemia (CML) is the most likely diagnosis.
Rationale for Correctness: Mr. AC presents with classic signs and symptoms highly suggestive of CML in the chronic phase. These include marked leukocytosis (155,000/L) with a pronounced left shift (presence of myelocytes, metamyelocytes), significant basophilia (8%), thrombocytosis (650,000/L), and splenomegaly. The relatively low blast count (2%) further supports CML-chronic phase over an acute leukemia.
Why other options are less likely:
A) AML: Typically presents with >20% blasts in the peripheral blood or bone marrow and often features cytopenias (anemia, thrombocytopenia), although hyperleukocytosis can occur.
B) CLL: Characterized by lymphocytosis (proliferation of mature B-lymphocytes), not granulocytosis. Splenomegaly and lymphadenopathy can be present, but the WBC differential is distinctly different.
D) MDS: Usually presents with peripheral cytopenias (anemia, neutropenia, thrombocytopenia) and morphologic dysplasia in the bone marrow. While some forms can have increased blasts, the marked leukocytosis and basophilia seen here are atypical for MDS.

Phase 1: Initial Presentation and Diagnostic Workup

Patient: Mr. AC, a 52-year-old male, presents to his primary care physician complaining of increasing fatigue, unintentional weight loss (10 lbs over 3 months), and a feeling of fullness in his upper abdomen for the past month. He also reports occasional night sweats.

Past Medical History: Hypertension (controlled with lisinopril), Type 2 Diabetes Mellitus (controlled with metformin). No history of malignancy. Denies tobacco, alcohol, or illicit drug use.

Initial Physical Exam:

Vitals: BP 135/85 mmHg, HR 88 bpm, RR 16/min, Temp 37.1C (98.8F)

General: Appears fatigued but in no acute distress.

Abdomen: Palpable splenomegaly, extending 5 cm below the left costal margin. No hepatomegaly.

Lymphatics: No palpable lymphadenopathy.

Initial Laboratory Findings:

WBC: 155,000/L

Differential: Myelocytes 15%, Metamyelocytes 10%, Bands 5%, Neutrophils 50%, Basophils 8%, Eosinophils 5%, Lymphocytes 5%, Monocytes 2%, Blasts 2%

Hemoglobin: 10.5 g/dL

Platelets: 650,000/L

Peripheral Smear: Shows marked leukocytosis with left shift, including immature granulocytes (myelocytes, metamyelocytes), prominent basophilia, and occasional blasts.

Question 2 of 10) Which of the following tests is crucial to definitively confirm the diagnosis of CML and is a key component of the WHO diagnostic criteria?

A) Flow Cytometry of Peripheral Blood

B) Bone Marrow Biopsy with Cytogenetics (Karyotyping)

C) BCR-ABL1 fusion gene testing (via RT-PCR or FISH)

D) Serum Erythropoietin Level

Feedback:

Your selection of C) BCR-ABL1 fusion gene testing (via RT-PCR or FISH) is the most appropriate answer for definitively confirming CML.

Rationale for Correctness: The presence of the Philadelphia chromosome (Ph chromosome), resulting from the reciprocal translocation t(9;22)(q34;q11), or the resultant BCR-ABL1 fusion gene/transcript, is the pathognomonic feature of CML and a required criterion for diagnosis according to the WHO (Swerdlow SH, et al. WHO Classification of Tumours of Haematopoietic and Lymphoid Tissues. 2017; Arber DA, et al. WHO Classification of Tumours Haematopoietic and Lymphoid Tissues. 2022). RT-PCR (Reverse Transcriptase Polymerase Chain Reaction) detects the BCR-ABL1 mRNA transcript, while FISH (Fluorescence In Situ Hybridization) detects the fusion gene itself. These molecular tests are highly sensitive and specific.

Why other options are less definitive for diagnosis:

A) Flow Cytometry: While useful in characterizing the myeloid cells and excluding other leukemias, it doesn't detect the specific BCR-ABL1 fusion.

B) Bone Marrow Biopsy with Cytogenetics: Essential for assessing cellularity, blast percentage (for phasing - Chronic vs. Accelerated vs. Blast Phase), fibrosis, and detecting the Ph chromosome via karyotyping. Karyotyping confirms the t(9;22), but molecular testing (RT-PCR/FISH) is more sensitive and directly confirms the BCR-ABL1 fusion, which is present even in rare Ph-negative cases with cryptic translocations.

D) Serum Erythropoietin Level: Not relevant for diagnosing CML.

Phase 1: Initial Presentation and Diagnostic Workup

Patient: Mr. AC, a 52-year-old male, presents to his primary care physician complaining of increasing fatigue, unintentional weight loss (10 lbs over 3 months), and a feeling of fullness in his upper abdomen for the past month. He also reports occasional night sweats.

Past Medical History: Hypertension (controlled with lisinopril), Type 2 Diabetes Mellitus (controlled with metformin). No history of malignancy. Denies tobacco, alcohol, or illicit drug use.

Initial Physical Exam:

Vitals: BP 135/85 mmHg, HR 88 bpm, RR 16/min, Temp 37.1C (98.8F)

General: Appears fatigued but in no acute distress.

Abdomen: Palpable splenomegaly, extending 5 cm below the left costal margin. No hepatomegaly.

Lymphatics: No palpable lymphadenopathy.

Initial Laboratory Findings:
WBC: 155,000/L
Differential: Myelocytes 15%, Metamyelocytes 10%, Bands 5%, Neutrophils 50%, Basophils 8%, Eosinophils 5%, Lymphocytes 5%, Monocytes 2%, Blasts 2%
Hemoglobin: 10.5 g/dL
Platelets: 650,000/L
Peripheral Smear: Shows marked leukocytosis with left shift, including immature granulocytes (myelocytes, metamyelocytes), prominent basophilia, and occasional blasts
(Question 3 of 10) According to the ELN 2020/2022 guidelines, which risk stratification score is generally preferred for predicting long-term outcomes and guiding initial therapy selection in CML?
A) Sokal Score
B) Hasford (Euro) Score
C) EUTOS Long-Term Survival (ELTS) Score
D) Charlson Comorbidity Index

Feedback:
Your selection of C) EUTOS Long-Term Survival (ELTS) Score is the most appropriate answer.
Rationale for Correctness: The ELN 2020 guidelines (Hochhaus A, et al. Leukemia. 2020) recommend using risk scores calculated at diagnosis, and while both Sokal and ELTS are mentioned, the ELTS score is generally preferred for predicting long-term outcomes (like overall survival and progression-free survival) in the era of Tyrosine Kinase Inhibitor (TKI) therapy. It utilizes parameters (age, spleen size, platelet count, peripheral basophils) that correlate well with long-term prognosis for patients treated with TKIs.
Why other options are less preferred:
A) Sokal Score: Developed before the widespread use of TKIs, based on outcomes with conventional chemotherapy (hydroxyurea, busulfan). While still calculated and providing prognostic information, ELTS may offer better discrimination for long-term outcomes with TKIs.
B) Hasford (Euro) Score: Also developed before the TKI era (though used with Interferon studies), less commonly used now than Sokal or ELTS.
D) Charlson Comorbidity Index: Assesses the burden of comorbidities, which is crucial for overall patient management and TKI selection (due to differing side effect profiles), but it is not a CML-specific prognostic score for the disease itself.
Conclusion of Phase 1: Mr. AC is a 52-year-old male diagnosed with Chronic Phase CML (CML-CP), confirmed by BCR-ABL1 testing. He has intermediate-risk disease based on the preferred ELTS score (and high-risk by Sokal score).

Phase 2: Induction Therapy Selection and Supportive Care Planning
Mr. AC is ready to start treatment. His comorbidities include controlled hypertension and type 2 diabetes. His performance status is ECOG 1 (Restricted in physically strenuous activity but ambulatory and able to carry out work of a light or sedentary nature). He has no known cardiovascular disease beyond hypertension.
(Question 4 of 10) Based on ELN 2020 guidelines, which of the following would be the MOST appropriate first-line TKI therapy recommendation for Mr. AC, considering his diagnosis (CML-CP), risk score (ELTS Intermediate), age, and comorbidities?
A) Imatinib 400 mg daily
B) Dasatinib 100 mg daily
C) Nilotinib 300 mg twice daily
D) Any of the above (Imatinib, Dasatinib, or Nilotinib) could be considered appropriate first-line options.

Feedback:
Your selection of D) Any of the above (Imatinib, Dasatinib, or Nilotinib) could be considered appropriate first-line options. is correct.
Rationale for Correctness: The ELN 2020 guidelines (Hochhaus A, et al. Leukemia. 2020) approve imatinib, dasatinib, nilotinib, and bosutinib as first-line therapies for newly diagnosed CML-CP. The guidelines emphasize individualizing the choice based on the TKI's efficacy and safety profile, the patient's risk score (Sokal/ELTS), age, comorbidities, and concomitant medications.
Imatinib: A standard, effective option, particularly for low-risk patients, but suitable for all risk groups. Generally well-tolerated long-term, though responses may be slower than with 2GTKIs.
Dasatinib: A second-generation TKI (2GTKI) that may induce faster and deeper responses. Potential side effects include pleural effusion and platelet dysfunction. Generally suitable for Mr. AC, barring specific contraindications like significant pulmonary disease.
Nilotinib: Another 2GTKI offering rapid responses. However, it carries warnings regarding QTc prolongation, vascular events (like PAOD), and potential for hyperglycemia, which require careful consideration in patients with diabetes and hypertension like Mr. AC.

Requires administration on an empty stomach.

Clinical Decision Making: For Mr. AC (ELTS Intermediate Risk), a 2GTKI might be favoured by some clinicians to achieve faster molecular responses. However, considering his diabetes and hypertension, the potential vascular and metabolic side effects of nilotinib warrant caution. Dasatinib is a strong contender, but the risk of pleural effusion must be discussed. Imatinib remains a very appropriate and safe initial choice, balancing efficacy with a generally favourable long-term safety profile, especially concerning cardiovascular and metabolic health. The final decision should involve a discussion between the clinician and patient about the goals of therapy (e.g., speed of response vs. long-term tolerability) and the specific risk-benefit profile of each agent.

Next Step: After discussing the options, Mr. AC and his oncologist decide to start Imatinib 400 mg daily. Supportive care measures need to be initiated alongside TKI therapy.

Phase 2: Induction Therapy Selection and Supportive Care Planning

Mr. AC is ready to start treatment. His comorbidities include controlled hypertension and type 2 diabetes. His performance status is ECOG 1 (Restricted in physically strenuous activity but ambulatory and able to carry out work of a light or sedentary nature). He has no known cardiovascular disease beyond hypertension.

(Question 5 of 10) Mr. AC's initial WBC count was very high (155,000/L), and he has splenomegaly. While Tumor Lysis Syndrome (TLS) is less common in CML-CP than in acute leukemias or high-grade lymphomas, precautions may be considered, especially during the initial cytoreduction phase. According to general supportive care principles in leukemia (e.g., referenced in ASCO/HOPA standards), which of the following is the MOST appropriate initial approach to TLS prophylaxis/monitoring for Mr. AC?

A) Aggressive hydration, allopurinol, and frequent electrolyte monitoring (e.g., every 6-12 hours initially).

B) Rasburicase administration prior to starting imatinib.

C) No specific TLS prophylaxis or monitoring is needed as TLS is rare in CML-CP.

D) Start allopurinol only if serum uric acid is elevated at baseline.

Feedback:

Your selection of A) Aggressive hydration, allopurinol, and frequent electrolyte monitoring (e.g., every 6-12 hours initially) is the most appropriate initial strategy.

Rationale for Correctness: Although the risk of clinically significant Tumor Lysis Syndrome (TLS) is lower in CML-CP compared to acute leukemias or high-grade lymphomas, Mr. AC's very high initial WBC count (155,000/L) places him at an intermediate risk for laboratory TLS upon initiation of cytoreductive therapy (even with a TKI). Standard supportive care guidelines (referencing principles like the Cairo-Bishop criteria) recommend prophylaxis for intermediate-risk patients. This typically includes:

Aggressive Hydration: To maintain renal perfusion and facilitate excretion of metabolites like uric acid and phosphate.

Allopurinol: To inhibit xanthine oxidase, thereby reducing the production of new uric acid from purine breakdown.

Frequent Monitoring: Close monitoring of electrolytes (potassium, phosphate, calcium), uric acid, and renal function (BUN, creatinine) is crucial to detect and manage any developing abnormalities promptly. Monitoring frequency (e.g., q6-12h initially) can be adjusted based on the clinical picture and initial lab trends.

Why other options are less appropriate:

B) Rasburicase: Generally reserved for high-risk TLS or established hyperuricemia/TLS. While effective, it's typically unnecessary for standard CML-CP initiation unless specific high-risk features (like extreme leukocytosis plus renal impairment) are present.

C) No specific prophylaxis: This is not advisable given the significant tumor burden (WBC > 100,000/L). While overt clinical TLS is uncommon, laboratory TLS can occur and requires management.

D) Allopurinol only if uric acid is elevated: This is a reactive strategy. Prophylaxis aims to prevent hyperuricemia when rapid cell lysis is anticipated, making prophylactic allopurinol appropriate even with normal baseline uric acid in intermediate-risk scenarios.

Next Step: Mr. AC starts Imatinib 400 mg daily with appropriate TLS prophylaxis (hydration, allopurinol) and monitoring. He tolerates the initial phase well.

Phase 2: Induction Therapy Selection and Supportive Care Planning

Mr. AC is ready to start treatment. His comorbidities include controlled hypertension and type 2 diabetes. His performance status is ECOG 1 (Restricted in physically strenuous activity but ambulatory and able to carry out work of a light or sedentary nature). He has no known cardiovascular disease beyond hypertension.

(Question 6 of 10) Three months after starting imatinib, Mr. AC returns for follow-up. His labs show:

WBC: 8,500/L (Normal differential)

Hgb: 12.8 g/dL

Platelets: 280,000/L

Peripheral Blood Quantitative RT-PCR for BCR-ABL1 (IS): 1.5%

According to ELN 2020 criteria, how would you classify Mr. AC's response at 3 months?

A) Optimal Response

B) Warning
C) Failure
D) Complete Hematologic Response (CHR) only

Feedback:
Your selection of A) Optimal Response is accurate according to the ELN 2020 guidelines.
Rationale for Correctness: The ELN 2020 guidelines (Hochhaus A, et al. Leukemia. 2020) define response milestones at specific time points. At 3 months, an Optimal Response is defined as achieving BCR-ABL1 transcript levels 10% on the International Scale (IS). Mr. AC's BCR-ABL1 (IS) is 1.5%, which is well below the 10% threshold. He has also achieved a Complete Hematologic Response (CHR) based on his normalized blood counts. Meeting the molecular milestone of 10% classifies this as an optimal response.
Why other options are incorrect:
B) Warning: A 'Warning' at 3 months would be indicated if BCR-ABL1 (IS) was >10%.
C) Failure: 'Failure' at 3 months would typically be defined by not achieving CHR or potentially by the presence of specific high-risk chromosomal abnormalities (clonal evolution) at diagnosis or emergence during therapy (though the primary failure criterion at 3 months relates more to hematologic response and the >10% BCR-ABL1 level being a 'warning').
D) CHR only: While Mr. AC has achieved CHR, the ELN guidelines use specific molecular milestones to define 'Optimal', 'Warning', or 'Failure' categories, which provide more prognostic information than CHR alone at this time point. Since he met the molecular milestone, 'Optimal Response' is the more precise classification.
Next Step: Mr. AC continues imatinib 400 mg daily and undergoes regular monitoring.
Phase 2: Induction Therapy Selection and Supportive Care Planning
Mr. AC is ready to start treatment. His comorbidities include controlled hypertension and type 2 diabetes. His performance status is ECOG 1 (Restricted in physically strenuous activity but ambulatory and able to carry out work of a light or sedentary nature). He has no known cardiovascular disease beyond hypertension.(Question 7 of 10) Six months after starting imatinib, Mr. AC's follow-up labs show:
WBC: 6,200/L (Normal differential)
Hgb: 13.5 g/dL
Platelets: 220,000/L
Peripheral Blood Quantitative RT-PCR for BCR-ABL1 (IS): 0.5%
According to ELN 2020 criteria, how would you classify Mr. AC's response at 6 months?
A) Optimal Response
B) Warning
C) Failure
D) Major Molecular Response (MMR)

Feedback:
Your selection of A) Optimal Response is accurate according to the ELN 2020 guidelines.
Rationale for Correctness: The ELN 2020 guidelines define response milestones at various time points. At 6 months, an Optimal Response is defined as achieving BCR-ABL1 transcript levels 1% on the International Scale (IS). Mr. AC's BCR-ABL1 (IS) is 0.5%, which meets this criterion.
Why other options are incorrect:
B) Warning: A 'Warning' at 6 months would be indicated if BCR-ABL1 (IS) was >1% but 10%.
C) Failure: 'Failure' at 6 months would be defined by BCR-ABL1 (IS) >10% or loss of CHR.
D) Major Molecular Response (MMR): While achieving MMR (BCR-ABL1 0.1% or MR3) is a key therapeutic goal, the definition of 'Optimal Response' at 6 months is specifically set at 1%. Mr. AC has not yet achieved MMR, but his response is still considered optimal for this time point.
Conclusion of Phase 2: Mr. AC has achieved optimal responses at both the 3-month and 6-month milestones while on Imatinib 400 mg daily. He has tolerated the medication reasonably well, with only mild fatigue managed symptomatically.
Phase 3: Post-Remission Therapy, MRD Monitoring, and Long-Term Management
Mr. AC continues on imatinib 400 mg daily.
(Question 8 of 10) Twelve months after starting imatinib, Mr. AC's follow-up labs show:
WBC: 5,800/L (Normal differential)
Hgb: 14.0 g/dL
Platelets: 200,000/L
Peripheral Blood Quantitative RT-PCR for BCR-ABL1 (IS): 0.05%

According to ELN 2020 criteria, how would you classify Mr. AC's response at 12 months?
A) Optimal Response (including Major Molecular Response - MMR)
B) Warning
C) Failure
D) Deep Molecular Response (DMR)

Feedback:
Your selection of A) Optimal Response (including Major Molecular Response - MMR) is the most accurate classification.
Rationale for Correctness: The ELN 2020 guidelines define Optimal Response at 12 months as achieving BCR-ABL1 transcript levels 0.1% on the International Scale (IS). A Major Molecular Response (MMR), also referred to as MR3 (Molecular Response 3-log reduction from standardized baseline), is also defined as BCR-ABL1 (IS) 0.1%. Mr. AC's result of 0.05% IS meets both of these criteria. Achieving MMR by 12 months is a key therapeutic milestone associated with favourable long-term outcomes.
Why other options are incorrect:
B) Warning: A 'Warning' at 12 months would be indicated if BCR-ABL1 (IS) was >0.1% but 1%.
C) Failure: 'Failure' at 12 months would be defined by BCR-ABL1 (IS) >1%.
D) Deep Molecular Response (DMR): DMR refers to responses deeper than MMR, typically MR4 (BCR-ABL1 0.01%), MR4.5 (BCR-ABL1 0.0032%), or MR5 (BCR-ABL1 0.001%). Mr. AC's level of 0.05% has not yet reached DMR territory.
Next Step: Mr. AC has achieved an optimal response, including MMR, at 12 months. He continues imatinib 400 mg daily. Monitoring continues, typically with quantitative RT-PCR every 3 months for the next couple of years, then potentially spaced to every 6 months if responses remain stable and deep.
Scenario Change: Let's fast forward. Mr. AC remains on imatinib 400 mg daily for a total of 5 years. He has maintained MMR (fluctuating between 0.02% and 0.08% IS) consistently after the first year. He now expresses interest in potentially stopping TKI therapy (Treatment-Free Remission - TFR).
Phase 3: Post-Remission Therapy, MRD Monitoring, and Long-Term Management
Mr. AC continues on imatinib 400 mg daily.(Question 9 of 10) According to ELN 2020 guidelines and data from TFR trials, which of the following is the MOST important prerequisite for considering a TFR attempt in Mr. AC?
A) Having achieved MMR (BCR-ABL1 0.1% IS) for at least 1 year.
B) Having achieved and maintained a Deep Molecular Response (DMR, e.g., MR4 or MR4.5) for a sustained duration (typically 2 years).
C) Being on TKI therapy for at least 3 years, regardless of molecular response depth.
D) Having an ELTS low-risk score at diagnosis.

Feedback:
Your selection of B) Having achieved and maintained a Deep Molecular Response (DMR, e.g., MR4 or MR4.5) for a sustained duration (typically 2 years) is the most important prerequisite for considering a TFR attempt.
Rationale for Correctness: International guidelines, including ELN 2020 (Hochhaus A, et al. Leukemia. 2020) and NCCN, recommend strict criteria for TFR attempts to maximize the chance of success and minimize relapse risk. The cornerstone criterion is the achievement and maintenance of a Sustained Deep Molecular Response (S-DMR). While the exact definition can vary slightly by assay sensitivity and specific trial protocols, it generally refers to:
Depth: MR4 (BCR-ABL1 0.01% IS), MR4.5 (BCR-ABL1 0.0032% IS), or deeper.
Duration: Maintained for a significant period, typically at least 2 years, with frequent monitoring confirming stability.
Why other options are less appropriate:
A) MMR for 1 year: MMR (BCR-ABL1 0.1% IS) is not considered deep enough for safe TFR attempts. Patients stopping TKI with only MMR have a very high relapse rate. Mr. AC currently falls into this category (maintained MMR but not DMR).
C) TKI duration 3 years: While a minimum duration of TKI therapy (often 3-5 years) is required, it is not sufficient without achieving S-DMR.
D) ELTS low-risk score: Risk score at diagnosis does not determine eligibility for TFR; the achieved molecular response is the key factor.
Clinical Application: In Mr. AC's case, although he has been on imatinib for 5 years and maintained MMR, his BCR-ABL1 levels (0.02-0.08%) have not consistently reached MR4 (0.01%) or deeper for the required duration (2 years). Therefore, he does not currently meet the criteria for a standard TFR attempt. Options would include continuing imatinib or potentially switching to a 2GTKI to try and achieve a deeper response if TFR remains a major goal.
Final Question Scenario: Let's consider an alternative outcome. Suppose Mr. AC had developed resistance to imatinib after 18 months, confirmed by a rising BCR-ABL1 level (e.g., to 5% IS) and the detection of a T315I mutation via ABL1 kinase domain

mutation analysis.
Phase 3: Post-Remission Therapy, MRD Monitoring, and Long-Term Management
Mr. AC continues on imatinib 400 mg daily.
(Question 10 of 10) In the presence of the T315I mutation, which of the following TKIs would be the MOST appropriate treatment choice for Mr. AC?
A) Dasatinib
B) Nilotinib
C) Bosutinib
D) Ponatinib

Feedback:
Your selection of D) Ponatinib is the most appropriate treatment choice for CML resistance mediated by the T315I mutation.
Rationale for Correctness: The T315I mutation is a notorious "gatekeeper" mutation within the ABL1 kinase domain. It confers resistance to imatinib, dasatinib, nilotinib, and bosutinib by sterically hindering their ability to bind to the ATP-binding site of the BCR-ABL1 protein. Ponatinib is a third-generation TKI specifically designed to overcome this resistance mechanism and is currently the only approved TKI (among the options provided) that effectively inhibits BCR-ABL1 kinase activity in the presence of the T315I mutation. Clinical trials have demonstrated its efficacy in this setting (Cortes JE, et al. N Engl J Med. 2012). Note: Asciminib, a STAMP inhibitor binding to the myristoyl pocket, is also approved for T315I-mutated CML, but was not listed as an option here.
Why other options are incorrect:
A) Dasatinib: Ineffective against T315I.
B) Nilotinib: Ineffective against T315I.
C) Bosutinib: Ineffective against T315I.
Conclusion of Phase 3 and Case: Mr. AC's journey illustrates key aspects of CML management: diagnosis confirmation, risk stratification, initial TKI selection based on guidelines and patient factors, monitoring response using molecular milestones (ELN criteria), understanding criteria for TFR, and managing resistance, particularly the challenging T315I mutation

---

Session ID:
Assessment Domain

Session-60:
Rationale

Session ID:
Instructional Design and Pedagogical

Session-60:
The session's instructional design is outstanding. The case is expertly scaffolded, progressing logically from initial presentation and diagnosis (Questions 1-3) through first-line therapy selection and monitoring (Questions 4-8), to advanced long-term management topics like Treatment-Free Remission and resistance (Questions 9-10). The feedback provided for each question is a major strength; it is consistently clear, detailed, and anchored in current clinical guidelines (e.g., ELN 2020, WHO). The instructions are unambiguous and the clinical narrative is very easy to follow. The learning objectives are not explicitly stated at the outset, which is the only minor point of improvement; however, they are strongly implied by the case's structure and content.

Session ID:
Clinical Authenticity and Content Fidelity

Session-60:
The clinical fidelity of this simulation is excellent and represents a gold standard for this type of educational material. The clinical narrative is highly plausible, mirroring a textbook presentation and progression of Chronic Myeloid Leukemia. All therapeutic recommendations, monitoring milestones, and management decisions are perfectly aligned with current, evidence-based guidelines, particularly the ELN 2020 criteria which are referenced appropriately. The pharmacotherapeutic choices are optimal and well-justified throughout the case, from initial TKI selection to the management of T315I-mutated resistance. Crucially, the case demonstrates perfect domain specificity, remaining strictly within the CML disease state without any evidence of domain entanglement.

Session ID:

Modeled Clinical Reasoning

Session-60:

The simulation flawlessly models an expert clinical reasoning process. Each stage of the case presents a distinct and well-framed clinical problem, from diagnosis to management of complications. The multiple-choice options are exceptionally well-crafted, offering a superb breadth of relevant, plausible alternatives and key distractors that effectively probe the user's nuanced understanding (e.g., differentiating between MMR and 'Optimal Response' at specific time points). The case demonstrates logical, risk-adjusted prioritization and decision-making throughout. The clinical outcomes are realistic and highly instructive, reinforcing the learning points at each step of the patient's journey.

Session-61

Session ID:
LLM

Session-61:
Google AI Studio (Gemini 2.0 Pro)

Session ID:
Session Transcript

Session-61:
Patient: Mr. David Miller, a 52-year-old male.
Chief Complaint: Increasing fatigue and early satiety over the past 3 months. He also reports occasional night sweats and unintentional weight loss of 10 lbs (4.5 kg).
Past Medical History: Hypertension (controlled with lisinopril), Hyperlipidemia (controlled with atorvastatin). No history of cardiovascular events, diabetes, or other significant comorbidities.
Social History: Non-smoker, occasional alcohol use (1-2 drinks per week). Works as an accountant.
Family History: Non-contributory for hematologic malignancies.
Review of Systems: Besides the chief complaint, denies fever, chills, bone pain, bleeding, or easy bruising.
Physical Examination:
Vital Signs: BP 130/80 mmHg, HR 88 bpm, RR 16/min, Temp 37.0C (98.6F).
General: Appears fatigued but is alert and oriented x3.
Abdomen: Palpable splenomegaly, extending 5 cm below the left costal margin. No hepatomegaly.
Lymph Nodes: No palpable lymphadenopathy.
Cardiovascular/Respiratory: Unremarkable.
Initial Laboratory Results:
CBC:
WBC: 115 x 10/L (Normal: 4.5-11.0 x 10/L)
Differential: Neutrophils 55%, Bands 10%, Metamyelocytes 15%, Myelocytes 10%, Promyelocytes 2%, Blasts 3%, Basophils 5%, Eosinophils 2%, Lymphocytes 5%, Monocytes 3%
Hemoglobin: 10.8 g/dL (Normal: 13.5-17.5 g/dL)
Platelets: 550 x 10/L (Normal: 150-450 x 10/L)
Comprehensive Metabolic Panel (CMP):
Creatinine: 0.9 mg/dL
BUN: 15 mg/dL
Electrolytes: Within normal limits
LFTs: Within normal limits
Uric Acid: 8.5 mg/dL (Normal: 3.5-7.2 mg/dL)
LDH: 450 U/L (Normal: 140-280 U/L)
Clinical Impression: The constellation of symptoms (fatigue, early satiety, weight loss, night sweats), splenomegaly, marked leukocytosis with left shift, thrombocytosis, mild anemia, and elevated LDH raises strong suspicion for Chronic Myeloid Leukemia (CML)Okay [REDACTED]], based on this initial presentation, what is the most crucial next step to confirm the suspected diagnosis of CML?
Question 1:
A) Perform a lumbar puncture to rule out CNS involvement.
B) Order cytogenetic analysis (karyotyping) and molecular testing (RT-PCR for BCR::ABL1) on peripheral blood or bone marrow aspirate.
C) Initiate empiric treatment with hydroxyurea immediately to control the high WBC count.
D) Obtain a PET-CT scan to assess for extramedullary disease.
Excellent choice, [REDACTED]]. Option B is indeed the correct answer.
Feedback:
Correct Answer (B): Cytogenetic analysis (karyotyping) to detect the Philadelphia (Ph) chromosome [t(9;22)(q34.1;q11.2)] and

molecular testing (quantitative RT-PCR) to identify and quantify the BCR::ABL1 fusion transcript are essential for confirming the diagnosis of CML, as per WHO and ESMO guidelines. These tests identify the pathognomonic genetic lesion driving the disease.

Rationale for Incorrect Options:

(A) Lumbar Puncture: CNS involvement is rare in chronic phase CML and is only investigated if specific neurological symptoms are present.

(C) Empiric Hydroxyurea: While cytoreduction with hydroxyurea might be considered for very high WBC counts (e.g., >100-200 x 10/L) or symptoms of leukostasis, it's typically after or concurrently with diagnostic confirmation, not before. The priority is establishing the diagnosis to initiate targeted TKI therapy.

(D) PET-CT Scan: This is not a standard diagnostic tool for initial CML workup. It may have a role later if advanced phase disease or extramedullary involvement is suspected

Patient: Mr. David Miller, a 52-year-old male.

Chief Complaint: Increasing fatigue and early satiety over the past 3 months. He also reports occasional night sweats and unintentional weight loss of 10 lbs (4.5 kg).

Past Medical History: Hypertension (controlled with lisinopril), Hyperlipidemia (controlled with atorvastatin). No history of cardiovascular events, diabetes, or other significant comorbidities.

Social History: Non-smoker, occasional alcohol use (1-2 drinks per week). Works as an accountant.

Family History: Non-contributory for hematologic malignancies.

Review of Systems: Besides the chief complaint, denies fever, chills, bone pain, bleeding, or easy bruising.

Physical Examination:

Vital Signs: BP 130/80 mmHg, HR 88 bpm, RR 16/min, Temp 37.0C (98.6F).

General: Appears fatigued but is alert and oriented x3.

Abdomen: Palpable splenomegaly, extending 5 cm below the left costal margin. No hepatomegaly.

Lymph Nodes: No palpable lymphadenopathy.

Cardiovascular/Respiratory: Unremarkable.

Initial Laboratory Results:

CBC:

WBC: 115 x 10/L (Normal: 4.5-11.0 x 10/L)

Differential: Neutrophils 55%, Bands 10%, Metamyelocytes 15%, Myelocytes 10%, Promyelocytes 2%, Blasts 3%, Basophils 5%, Eosinophils 2%, Lymphocytes 5%, Monocytes 3%

Hemoglobin: 10.8 g/dL (Normal: 13.5-17.5 g/dL)

Platelets: 550 x 10/L (Normal: 150-450 x 10/L)

Comprehensive Metabolic Panel (CMP):

Creatinine: 0.9 mg/dL

BUN: 15 mg/dL

Electrolytes: Within normal limits

LFTs: Within normal limits

Uric Acid: 8.5 mg/dL (Normal: 3.5-7.2 mg/dL)

LDH: 450 U/L (Normal: 140-280 U/L)

Clinical Impression: The constellation of symptoms (fatigue, early satiety, weight loss, night sweats), splenomegaly, marked leukocytosis with left shift, thrombocytosis, mild anemia, and elevated LDH raises strong suspicion for Chronic Myeloid Leukemia (CML)Case Progression:

A bone marrow aspirate and biopsy were performed. Peripheral blood was also sent for molecular analysis.

Results:

Bone Marrow Biopsy: Hypercellular marrow (95% cellularity) with marked granulocytic hyperplasia, increased megakaryocytes. Blasts constitute 3% of nucleated cells.

Cytogenetics (Karyotyping on Bone Marrow): 46,XY,t(9;22)(q34.1;q11.2)[20/20 metaphases]. All analyzed cells show the Philadelphia chromosome. No additional chromosomal abnormalities (ACAs) noted.

Molecular Testing (RT-PCR on Peripheral Blood): BCR::ABL1 (p210) transcript detected. Quantitative level corresponds to 85% on the International Scale (IS).

These results confirm the diagnosis of Chronic Myeloid Leukemia, Chronic Phase (CML-CP), BCR::ABL1-positive.

Now that the diagnosis is confirmed, the next step is risk stratification to guide treatment selection and prognosis.

Question 2:

Based on Mr. Miller's clinical and laboratory data at diagnosis (Age 52, Spleen size 5 cm below LCM, WBC 115 x 10/L, Platelets 550 x 10/L, Blasts 3% in marrow, Basophils 5% in peripheral blood, no ACAs), how would you classify his CML risk according to the ELN

2020 recommendations (using the Sokal or ELTS/EUTOS score)?
(Note: While ELN 2022 criteria exist, they often build upon established scores like Sokal and ELTS. For practical calculation, these scores remain relevant).
A) Low Risk
B) Intermediate Risk
C) High Risk
D) Cannot be determined without calculating the specific scores.
Okay, [REDACTED]], that's a reasonable assessment based on some of the scoring systems.
Feedback:
Your Answer (B - Intermediate Risk): This risk category aligns well with the Sokal Score. Mr. Miller's age (52), spleen size (5 cm), platelet count (550 x 10/L), and peripheral basophils (5%) generally push the score out of the low-risk category (<0.8) and into the intermediate range (0.8-1.2) according to typical Sokal calculations.
Alternative Consideration (Low Risk - based on ELTS): It's important to note that the ELTS (EUTOS Long-Term Survival) score, which is also recommended by ELN/ESMO and often preferred for predicting TKI response, uses a simpler calculation focusing on spleen size and basophil percentage [(7 * basophils %) + (4 * spleen size cm)]. For Mr. Miller: (7 * 5) + (4 * 5) = 55. A score 87 is considered low risk by ELTS.
Clinical Nuance: Discrepancies between Sokal and ELTS can occur. Both provide prognostic information. While ELTS might categorize Mr. Miller as low risk, the Sokal score suggests intermediate risk. For clinical decision-making, considering factors that push towards intermediate/higher risk (like the platelet count and basophil %) alongside the scores is prudent. We will proceed considering him as having intermediate-risk features based on Sokal, while keeping the low-risk ELTS score in mind.
Incorrect Options:
(A) Low Risk: While the ELTS score suggests low risk, the Sokal score and some clinical features (platelets, basophils) lean higher.
(C) High Risk: His parameters do not meet the typical criteria for high risk (e.g., much larger spleen, higher blast/basophil counts, older age).
(D) Cannot be determined: The scores can be calculated with the available data.
Question 3:
According to ESMO guidelines, what are the primary treatment goals for a newly diagnosed patient with CML-CP like Mr. Miller, and what baseline assessment is critical before starting Tyrosine Kinase Inhibitor (TKI) therapy?
A) Goal: Achieve rapid cytoreduction only. Baseline Assessment: Echocardiogram.
B) Goal: Achieve hematologic, cytogenetic, and molecular responses, aiming for treatment-free remission (TFR). Baseline Assessment: BCR::ABL1 kinase domain mutation analysis.
C) Goal: Prevent transformation to accelerated/blast phase and prolong survival by achieving optimal molecular responses. Baseline Assessment: Assessment of comorbidities (cardiovascular, metabolic, pulmonary), HBV/HCV serologies, and BCR::ABL1 quantitative PCR level.
D) Goal: Palliation of symptoms. Baseline Assessment: HLA typing for potential transplant.
Correct, [REDACTED]]! Option C accurately reflects the primary goals and essential baseline assessments.
Feedback:
Correct Answer (C):
Goals: The overarching aims of CML-CP treatment are to prevent disease progression to advanced phases (Accelerated Phase/Blast Crisis) and ensure long-term survival. This is achieved by obtaining deep and durable molecular responses (MR), specifically Major Molecular Response (MMR) and deeper responses like MR4 or MR4.5, as defined by ESMO/ELN guidelines.
Baseline Assessments: Before starting a TKI, it's crucial to evaluate the patient's overall health and potential risk factors for TKI-specific toxicities. This includes a thorough assessment of comorbidities (especially cardiovascular risk factors like hypertension, hyperlipidemia, diabetes, history of vascular events; pulmonary conditions like PAH; pancreatic issues), screening for Hepatitis B/C (due to reactivation risk with some TKIs), and establishing the baseline quantitative BCR::ABL1 level (IS %) to monitor treatment response effectively.
Rationale for Incorrect Options:
(A): Rapid cytoreduction is an initial objective but not the ultimate goal. An echocardiogram is important baseline screening if considering certain TKIs (like nilotinib or dasatinib depending on history), but the comorbidity assessment is broader.
(B): While achieving different response levels and aiming for TFR are valid points, baseline BCR::ABL1 kinase domain mutation analysis is not routinely recommended for newly diagnosed CML-CP unless advanced phase is suspected or upon treatment failure (ESMO Guidelines). The quantitative level is the key baseline BCR::ABL1 test.
(D): Palliation is not the goal for CML-CP. HLA typing is reserved for potential allogeneic stem cell transplant candidates, typically considered only for high-risk patients or those failing multiple TKIs, not as a standard baseline test for everyone.

Phase 2: Induction Therapy Selection and Supportive Care Planning

Mr. Miller has been diagnosed with CML-CP, BCR::ABL1-positive, with intermediate Sokal risk / low ELTS risk features. His comorbidities include controlled hypertension and hyperlipidemia. Baseline assessments confirmed no significant cardiac, pulmonary, or pancreatic issues, and HBV/HCV serologies are negative. His baseline BCR::ABL1 transcript level is 85% IS.

The next critical step is selecting the most appropriate first-line Tyrosine Kinase Inhibitor (TKI) therapy. ESMO guidelines recommend several TKIs for first-line treatment of CML-CP.Question 4:

Considering Mr. Miller's age (52), risk profile (intermediate Sokal/low ELTS), comorbidities (controlled HTN, HLD), and the goal of achieving deep molecular responses, which of the following first-line TKI regimens would be the most appropriate initial choice according to ESMO guidelines?

A) Imatinib 400 mg daily

B) Nilotinib 300 mg twice daily

C) Dasatinib 100 mg daily

D) Bosutinib 400 mg daily

E) Any of the above (A, B, C, or D) could be considered appropriate first-line choices based on ESMO guidelines, requiring discussion with the patient.

That's the best answer, [REDACTED]]. Option E correctly reflects the current ESMO guidelines.

Feedback:

Correct Answer (E): ESMO guidelines (as of their latest comprehensive versions) recommend imatinib, dasatinib, nilotinib, and bosutinib as suitable first-line treatment options for newly diagnosed CML-CP. The choice among them should be individualized based on:

CML Risk Score: While 2nd generation TKIs (dasatinib, nilotinib, bosutinib) may offer faster and deeper responses, potentially beneficial for intermediate/high-risk patients, imatinib remains a valid option, especially for low-risk patients or those where toxicity is a major concern. Mr. Miller's profile (Int Sokal/Low ELTS) allows for consideration of all options.

Patient Age & Comorbidities: This is crucial. Mr. Miller's controlled HTN and HLD require careful consideration:

Nilotinib: Carries warnings regarding cardiovascular events (QTc prolongation, vascular occlusive events) and hyperglycemia. His history necessitates careful risk/benefit assessment and monitoring.

Dasatinib: Associated with pleural effusions and, rarely, PAH. Less associated with metabolic issues.

Bosutinib: Known for diarrhea and hepatotoxicity, generally lower cardiovascular risk profile than nilotinib.

Imatinib: Generally well-tolerated, less potent, fluid retention/muscle cramps common. Lower risk of vascular events compared to nilotinib.

TKI Side Effect Profile: Each TKI has a unique profile that needs to be discussed with the patient.

Patient Preference: Factors like dosing schedule (once vs. twice daily), food interactions, and potential side effects influence patient choice and adherence.

Conclusion: Therefore, a discussion weighing the potential for faster/deeper response with 2nd generation TKIs against the specific toxicity profiles and Mr. Miller's comorbidities and preferences is essential. Any of the listed agents could be appropriate depending on the outcome of that discussion.

Question 5:

Which of the following represents the most critical baseline monitoring and initial supportive care measures specifically relevant to initiating Dasatinib therapy in Mr. Miller?

A) Baseline LVEF assessment via ECHO/MUGA, QTc interval measurement, and prescribing allopurinol for tumor lysis syndrome (TLS) prophylaxis.

B) Baseline chest X-ray, counseling on managing potential diarrhea, and screening for BCR::ABL1 mutations.

C) Baseline chest X-ray, assessment for pulmonary symptoms, counseling on potential fluid retention/pleural effusion symptoms, and prescribing allopurinol for TLS prophylaxis.

D) Baseline platelet function assay, screening for lactose intolerance, and initiating prophylactic antibiotics

Excellent, [REDACTED]]! Option C is the correct answer.

Feedback:

Correct Answer (C): This option correctly identifies the key considerations for starting Dasatinib in Mr. Miller:

Pulmonary Assessment: Dasatinib is associated with pleural effusions (occurring in up to ~20-30% of patients, varying by study) and, less commonly, pulmonary arterial hypertension (PAH). Therefore, establishing a baseline pulmonary status with a chest X-ray and clinical assessment is crucial (ESMO Guidelines). Counseling the patient to report symptoms like shortness of breath, cough, or chest pain is essential for early detection of effusions.

TLS Prophylaxis: Mr. Miller has a high WBC count (115 x 10/L) and elevated uric acid (8.5 mg/dL) and LDH, placing him at intermediate risk for Tumor Lysis Syndrome upon initiation of potent therapy. Prophylaxis with a hypouricemic agent like allopurinol,

along with hydration, is indicated according to supportive care guidelines (e.g., ASCO, ESMO).
Rationale for Incorrect Options:
(A): While cardiovascular assessment is always important, baseline LVEF and detailed QTc monitoring are more critical for nilotinib due to its specific cardiac warnings.
(B): While diarrhea can occur with dasatinib, it's generally less frequent/severe than with bosutinib. Baseline BCR::ABL1 mutation screening is not standard practice for initial CML-CP therapy.
(D): Dasatinib can inhibit platelet function, but routine baseline platelet function assays are not typically performed unless specific concerns exist (e.g., planned surgery, history of bleeding disorders). Prophylactic antibiotics are not standard. Lactose intolerance is primarily a concern with nilotinib capsules
Mr. Miller is initiated on Dasatinib 100 mg daily. He starts allopurinol 300 mg daily for TLS prophylaxis and is advised on maintaining good hydration. His lisinopril and atorvastatin are continued. He is scheduled for follow-up labs in 1-2 weeks.
Two weeks later: Mr. Miller returns for follow-up. He reports mild fatigue but no significant cough, shortness of breath, or edema.
WBC: 15.5 x 10/L (significantly improved)
Hgb: 11.0 g/dL
Plt: 410 x 10/L
CMP: Generally unremarkable. Uric acid 5.0 mg/dL.
One month later (Week 4 of therapy): He reports feeling better. Mild fatigue persists. No new complaints.
WBC: 8.2 x 10/L
Hgb: 11.5 g/dL
Plt: 350 x 10/L
Differential: Normalizes (no blasts, minimal immature granulocytes).
Question 6:
According to ESMO guidelines, what is the expected hematologic response milestone at 3 months of TKI therapy, and what is the appropriate monitoring test at this time point?
A) Milestone: Complete Hematologic Response (CHR). Monitoring: Bone marrow biopsy with cytogenetics.
B) Milestone: Major Molecular Response (MMR). Monitoring: Quantitative RT-PCR for BCR::ABL1.
C) Milestone: Complete Hematologic Response (CHR). Monitoring: Quantitative RT-PCR for BCR::ABL1.
D) Milestone: Partial Cytogenetic Response (PCyR). Monitoring: Bone marrow biopsy with cytogenetics.

You are absolutely correct, [REDACTED]]! Option C is the right answer.
Feedback:
Correct Answer (C):
Milestone: According to ESMO guidelines, achieving a Complete Hematologic Response (CHR) is the expected minimum response by 3 months of TKI therapy. CHR is defined by normalization of blood counts (WBC < 10 x 10/L, Platelets < 450 x 10/L), absence of immature cells in the peripheral blood, and resolution of splenomegaly. Mr. Miller achieved this even earlier, which is a good sign.
Monitoring: The crucial monitoring test at 3 months is quantitative RT-PCR for BCR::ABL1 on peripheral blood. This is used to assess the early molecular response. The specific goal at 3 months is to achieve BCR::ABL1 transcripts 10% on the International Scale (IS). This molecular milestone is a critical predictor of long-term outcomes like achieving MMR and overall survival.
Rationale for Incorrect Options:
(A): While CHR is correct, bone marrow biopsy/cytogenetics is not the standard monitoring test at 3 months unless the 10% molecular milestone is not met or there are other concerns. Peripheral blood PCR is preferred for routine molecular monitoring.
(B): Major Molecular Response (MMR) (BCR::ABL1 0.1% IS) is the goal typically expected by 12 months, not 3 months.
(D): Partial Cytogenetic Response (PCyR) (36-95% Ph+ cells) is a cytogenetic milestone, not the primary 3-month target, and requires a bone marrow biopsy. The focus at 3 months is on the molecular response measured by PCR.
Question 7:
Based on Mr. Miller's 3-month BCR::ABL1 result of 7.5% IS while on Dasatinib, how would you interpret this response according to ESMO criteria, and what is the recommended next step?
A) Optimal Response; continue Dasatinib at the current dose and monitor BCR::ABL1 levels every 3 months.
B) Warning; increase Dasatinib dose to 140 mg daily and repeat BCR::ABL1 in 1 month.
C) Treatment Failure; perform BCR::ABL1 kinase domain mutation analysis immediately and switch TKI.
D) Suboptimal Response; add Pegylated Interferon-alpha to Dasatinib therapy.
I see you selected C, [REDACTED]]. Let's carefully review the ESMO guidelines for the 3-month response milestone.
Feedback:
Incorrect Answer (C): This response level does not constitute treatment failure. According to ESMO guidelines, treatment failure at 3

months is defined as not achieving CHR or having a BCR::ABL1 transcript level > 10% IS. Mr. Miller has CHR and his BCR::ABL1 is 7.5% IS, which is less than 10%. Therefore, he has met the criteria for an optimal response, not failure. Performing mutation analysis and switching TKIs is only indicated in cases of confirmed failure or warning signs (which 7.5% IS at 3 months is not).

Correct Answer (A): The ESMO definition of an optimal response at 3 months is achieving a BCR::ABL1 transcript level 10% IS. Since Mr. Miller's level is 7.5% IS, he has achieved an optimal response. The standard recommendation for an optimal response is to continue the current TKI (Dasatinib 100 mg daily) at the same dose and continue routine monitoring, including BCR::ABL1 quantitative PCR every 3 months during the first two years.

Rationale for Other Incorrect Options:

(B) Warning: A "warning" response at 3 months would be BCR::ABL1 > 10% IS but CHR achieved, or lack of CHR. His result is better than this. Increasing the dose is not indicated for an optimal response.

(D) Suboptimal Response: This term is less used in the most recent ELN/ESMO frameworks for the 3-month timepoint, which primarily categorize as Optimal or Failure based on the 10% IS threshold (with 'Warning' for intermediate scenarios). Adding interferon is not standard practice for optimal response.

It's crucial to accurately interpret these early molecular milestones as they strongly predict long-term outcomes. Mr. Miller is currently responding optimally to Dasatinib.

Phase 3: Post-Remission Therapy, MRD Monitoring, and Long-Term Management

Mr. Miller continues Dasatinib 100 mg daily. His monitoring continues as per ESMO guidelines.

6-Month Follow-up:

CBC: CHR maintained.

BCR::ABL1 Quantitative PCR: 0.5% IS

12-Month Follow-up:

CBC: CHR maintained.

Physical Exam: Unremarkable.

BCR::ABL1 Quantitative PCR: 0.08% IS

Question 8:

How would you interpret Mr. Miller's response at 12 months (BCR::ABL1 = 0.08% IS) according to ESMO guidelines, and what is the recommended monitoring frequency moving forward?

A) Optimal Response (MMR achieved); continue Dasatinib 100 mg daily, monitor BCR::ABL1 every 3 months.

B) Warning Response; consider switching TKI due to slow response kinetics. Monitor BCR::ABL1 monthly.

C) Optimal Response (MMR achieved); continue Dasatinib 100 mg daily, monitor BCR::ABL1 every 6 months.

D) Treatment Failure; BCR::ABL1 still detectable, requires mutation analysis and switch to a third-generation TKI.

You're close, [REDACTED]]! You've correctly identified that Mr. Miller has achieved an optimal response (MMR), but let's refine the monitoring frequency based on ESMO guidelines for a stable MMR.

Feedback:

Partially Correct (A): You are absolutely right that achieving BCR::ABL1 0.1% IS (MMR) at 12 months is an Optimal Response. Mr. Miller's 0.08% IS clearly meets this goal. Continuing Dasatinib 100 mg daily is the correct therapeutic approach. However, the monitoring frequency typically changes once stable MMR is achieved. While monitoring every 3 months is standard during the first two years, guidelines often suggest extending the interval after this period if the response is stable.

Correct Answer (C): This option correctly identifies the response as Optimal (MMR achieved) and recommends continuing Dasatinib 100 mg daily. Crucially, it reflects the ESMO recommendation that once a patient maintains a stable MMR (typically assessed over a period, e.g., after the first year or two), the monitoring interval for BCR::ABL1 quantitative PCR can often be extended to every 6 months. This reduces the burden of testing while still ensuring adequate surveillance.

Rationale for Other Incorrect Options:

(B) Warning Response: 0.08% IS at 12 months is optimal, not a warning. Switching TKI or monitoring monthly is not indicated.

(D) Treatment Failure: MMR (0.1% IS) is the goal at 12 months; achieving it signifies success, not failure. Mutation analysis and switching TKI are not warranted. Detectable BCR::ABL1 below the MMR threshold is expected and represents successful disease control.

Phase 3: Post-Remission Therapy, MRD Monitoring, and Long-Term Management

Mr. Miller continues Dasatinib 100 mg daily. His monitoring continues as per ESMO guidelines.

6-Month Follow-up:

CBC: CHR maintained.

BCR::ABL1 Quantitative PCR: 0.5% IS

12-Month Follow-up:

CBC: CHR maintained.

Physical Exam: Unremarkable.

BCR::ABL1 Quantitative PCR: 0.08% ISMr. Miller continues on Dasatinib 100 mg daily and maintains MMR (BCR::ABL1 levels consistently <0.1% IS) with monitoring every 6 months.

3 Years After Diagnosis:

Mr. Miller calls the clinic reporting intermittent, mild shortness of breath on exertion over the past month, slightly worse than his usual baseline. He denies cough, chest pain, fever, or peripheral edema. His last BCR::ABL1 PCR 4 months ago was 0.02% IS (MR4).Question 9:

Given Mr. Miller's history of being on Dasatinib for 3 years and his new symptom of mild exertional dyspnea, what is the most important initial step in evaluating this potential toxicity?

A) Immediately switch him to Imatinib, assuming Dasatinib toxicity.

B) Perform BCR::ABL1 quantitative PCR to check for molecular relapse.

C) Order a chest X-ray and potentially an echocardiogram, and assess for pleural effusion or pulmonary hypertension.

D) Reassure the patient that mild dyspnea is common and continue current management.

Precisely, [REDACTED]]! Option C is the most appropriate initial step.

Feedback:

Correct Answer (C): Dasatinib is known to cause pleural effusions (fluid around the lungs) and, less commonly, pulmonary arterial hypertension (PAH). Both conditions can present with exertional dyspnea. Therefore, the most crucial initial step is to investigate these potential drug-related toxicities. A chest X-ray is the first-line investigation for pleural effusion. An echocardiogram is used to screen for PAH by estimating pulmonary artery pressures and assessing right heart function. Clinical assessment for signs of effusion (e.g., decreased breath sounds) is also key.

Rationale for Incorrect Options:

(A) Immediately switch TKI: While switching might eventually be necessary if a significant Dasatinib-related toxicity is confirmed and cannot be managed, it's premature to switch without confirming the cause of the dyspnea. Other causes need to be ruled out.

(B) Check BCR::ABL1 PCR: While monitoring disease status is always important, the new symptom strongly points towards a potential drug side effect rather than necessarily a relapse, especially since his last PCR showed deep molecular response (MR4). Investigating the side effect is the immediate priority.

(D) Reassurance only: New or worsening dyspnea in a patient on Dasatinib should never be dismissed without investigation, given the potential for significant complications like large pleural effusions or PAH.

A chest X-ray reveals small bilateral pleural effusions. An echocardiogram is largely unremarkable, with normal estimated pulmonary artery pressures, ruling out PAH for now.

Given the likely Dasatinib-induced pleural effusions, management options include dose interruption, dose reduction, or diuretics. After a brief interruption of Dasatinib and initiation of a low-dose diuretic, Mr. Miller's dyspnea improves, and a repeat chest X-ray shows resolution of the effusions. He is subsequently restarted on a reduced dose of Dasatinib 70 mg daily.

5 Years After Diagnosis:

Mr. Miller has remained on Dasatinib 70 mg daily. He tolerates this dose well without recurrence of significant effusions. His BCR::ABL1 PCR results have consistently been <0.01% IS (meeting criteria for MR4) for the last 2.5 years. He asks about the possibility of stopping his TKI therapy.

Question 10:

According to ESMO guidelines, what criteria must Mr. Miller meet to be considered eligible for a trial of Treatment-Free Remission (TFR), and what is the most crucial counseling point regarding TFR attempts?

A) Criteria: Age < 60 years, low Sokal risk at diagnosis, any detectable molecular response. Counseling: TFR is permanent once achieved.

B) Criteria: Must be on Imatinib first-line, stable MMR for 1 year. Counseling: Relapse requires immediate stem cell transplant.

C) Criteria: Minimum 5 years of TKI therapy, stable deep molecular response (DMR, e.g., MR4/MR4.5) for at least 2 years, typical BCR::ABL1 transcripts. Counseling: Molecular relapse occurs in ~40-60% of patients, requiring prompt TKI resumption.

D) Criteria: Achieved CHR within 3 months, no history of TKI dose reduction. Counseling: TFR success is guaranteed if DMR is maintained for 2 years

Absolutely correct, [REDACTED]]! Option C precisely summarizes the key ESMO criteria and counseling points for TFR.

Feedback:

Correct Answer (C):

Criteria: ESMO guidelines for TFR eligibility typically include:

Minimum duration of TKI therapy (often 5 years, though criteria evolved slightly).

Achievement and maintenance of a Stable Deep Molecular Response (DMR), usually defined as MR4 (BCR::ABL1 0.01% IS) or MR4.5 (BCR::ABL1 0.0032% IS).

Duration of stable DMR (typically 2 years).
Patient must have typical BCR::ABL1 transcripts (e.g., p210, p190). Atypical transcripts often exclude TFR attempts outside clinical trials.
Access to reliable, quality-assured quantitative PCR monitoring.
Counseling: It is critical to counsel patients that molecular relapse (loss of MMR) occurs in approximately 40-60% of patients attempting TFR, most often within the first 6-12 months. Patients must understand the need for frequent molecular monitoring after stopping TKI (e.g., monthly initially) and that prompt resumption of the same TKI upon molecular relapse almost invariably recaptures the response. TFR does not mean 'cured', but rather durable remission off therapy.
Rationale for Incorrect Options:
(A): Age and Sokal risk are not primary eligibility criteria for TFR (though risk factors for relapse are studied). "Any detectable molecular response" is incorrect; DMR is required. TFR is not necessarily permanent; relapse is common.
(B): TFR can be attempted after any TKI, not just imatinib. Stable MMR for 1 year is insufficient; stable DMR (MR4/4.5) for 2 years is needed. Relapse requires TKI resumption, not immediate SCT.
(D): Early CHR is good prognostically but not a direct TFR criterion. Dose reductions (like Mr. Miller's) do not necessarily preclude TFR if DMR is achieved and maintained on the reduced dose. TFR success is never guaranteed, even with stable DMR

Session ID:
Assessment Domain

Session-61:
Rationale

Session ID:
Instructional Design and Pedagogical

Session-61:
The session's instructional design is of very high quality. The case is expertly scaffolded, progressing logically from initial presentation and diagnosis (Q1), through risk stratification (Q2), goal setting (Q3), first-line therapy selection (Q4-Q5), and response monitoring at key milestones (Q6-Q8). It effectively continues into long-term management, covering adverse event handling (Q9) and advanced therapeutic goals like Treatment-Free Remission (Q10). The embedded feedback for each question is excellent, providing clear, guideline-based rationales (citing ESMO) and explaining why incorrect options are wrong. The instructions and framing for each question are unambiguous. The only minor weakness is the lack of explicitly stated, measurable learning objectives at the start of the session, though the educational goals are strongly implied by the comprehensive structure.

Session ID:
Clinical Authenticity and Content Fidelity

Session-61:
The clinical fidelity of this session is outstanding and contains no discernible errors. Plausibility & Guideline Alignment: The clinical narrative for Mr. Miller is a textbook presentation of Chronic Myeloid Leukemia (CML), making it indistinguishable from an authentic case. All diagnostic steps, risk calculations (correctly referencing both Sokal and ELTS scores), therapeutic goals, and monitoring milestones (10% BCR::ABL1 at 3 months, MMR at 12 months) are perfectly aligned with current, evidence-based ESMO guidelines. Pharmacotherapeutic Accuracy: The therapeutic choices and management are optimal. The selection of Dasatinib is presented as one of several valid guideline-concordant options. The supportive care (Allopurinol for TLS risk), monitoring for drug-specific toxicities (pleural effusion for Dasatinib), and subsequent dose reduction are all appropriate and well-justified actions. Domain Specificity: The case remains strictly within the CML disease domain. All diagnostic tools, risk scores, therapies, and management concepts are specific to CML. There are no instances of domain entanglement where standards or treatments from other hematologic malignancies (e.g., AML, ALL) are incorrectly applied.

Session ID:
Modeled Clinical Reasoning

Session-61:
The simulation expertly models the clinical reasoning process in CML management. Each stage presents a clearly framed clinical

problem, from initial diagnosis to long-term care. The multiple-choice options demonstrate excellent breadth, providing relevant and plausible alternatives that effectively test nuanced understanding (e.g., distinguishing the adverse event profiles of different TKIs). The overall strategic flow shows sound prioritization: confirming the diagnosis before treating, assessing risk to guide therapy, and investigating toxicity as the primary cause of new symptoms. The clinical outcomes described at each step (e.g., lab value responses, development and resolution of pleural effusions) are realistic, specific, and highly instructive, reinforcing the learning from each decision point.

Session-62

Session ID:
LLM

Session-62:
Google AI Studio (Gemini 2.0 Pro)

Session ID:
Session Transcript

Session-62:
A 58-year-old male, Mr. Ahmed, presents to his primary care physician with complaints of fatigue, unintentional weight loss of 5 kg over the past 3 months, and a feeling of fullness in his left upper abdomen. Physical examination reveals splenomegaly.
Question 1: Based on Mr. Ahmed's initial presentation, which of the following is the MOST likely initial laboratory test to order to investigate a suspected hematologic malignancy like CML?
a) Serum creatinine
b) Complete blood count (CBC) with differential
c) Liver function tests (LFTs)
d) Coagulation studies (PT/INR, aPTT)
That's correct, [REDACTED]]!
The correct answer is b) Complete blood count (CBC) with differential.
Feedback:
 * Correct Answer (b): A CBC with differential is the most likely initial laboratory test to order. The symptoms of fatigue, weight loss, and splenomegaly are highly suggestive of a myeloproliferative disorder like CML, which is characterized by an overproduction of blood cells. The CBC will provide information on the white blood cell count, red blood cell count, platelet count, and the different types of white blood cells present (the differential). An elevated white blood cell count with immature granulocytes would be a key indicator of CML.
 * Incorrect Answer (a): Serum creatinine assesses kidney function and is not the primary test for investigating suspected hematologic malignancy. While kidney function is important to monitor during treatment, it's not the initial diagnostic step.
 * Incorrect Answer (c): Liver function tests (LFTs) assess the health of the liver. While the liver can be affected in some hematologic malignancies, and LFTs are often part of a broader workup, they are not the most direct initial test for suspected CML based on these symptoms.
 * Incorrect Answer (d): Coagulation studies (PT/INR, aPTT) evaluate the blood's clotting ability. These are important in certain hematologic conditions but are not the primary investigation for the initial suspicion of CML based on the presented symptoms.
Great! Following the initial CBC, which revealed a significantly elevated white blood cell (WBC) count of 150 x 10^9/L (normal: 4-10 x 10^9/L) with a left shift including myelocytes and blasts, the hematologist orders further diagnostic testing.
Question 2: Which of the following is the MOST definitive diagnostic test for confirming a diagnosis of Chronic Myeloid Leukemia (CML)?
a) Bone marrow aspirate and biopsy with cytogenetic analysis for the Philadelphia chromosome (Ph+)
b) Peripheral blood smear examination for Auer rods
c) Serum lactate dehydrogenase (LDH) levels
d) Flow cytometry analysis of peripheral blood for lymphoid markers

That's absolutely correct, [REDACTED]]!
The correct answer is a) Bone marrow aspirate and biopsy with cytogenetic analysis for the Philadelphia chromosome (Ph+).
Feedback:
 * Correct Answer (a): The Philadelphia chromosome (Ph+), a reciprocal translocation between chromosomes 9 and 22 [t(9;22)(q34;q11.2)] resulting in the BCR-ABL1 fusion gene, is the hallmark of CML. Cytogenetic analysis of a bone marrow sample is the gold standard for confirming the presence of this translocation. Fluorescence in situ hybridization (FISH) and reverse transcription-polymerase chain reaction (RT-PCR) on bone marrow or peripheral blood samples can also detect the BCR-ABL1 fusion gene or transcript, respectively, and are crucial for diagnosis and monitoring.
 * Incorrect Answer (b): Auer rods are rod-shaped granules seen in the cytoplasm of myeloblasts and promyelocytes. They are

characteristic of acute myeloid leukemia (AML), not CML.
* Incorrect Answer (c): Serum lactate dehydrogenase (LDH) levels can be elevated in various hematologic malignancies due to increased cell turnover. While LDH might be elevated in CML, it is not specific for the disease and is not a definitive diagnostic test.
* Incorrect Answer (d): Flow cytometry analysis of peripheral blood for lymphoid markers is primarily used to diagnose and classify lymphoid malignancies (e.g., lymphomas, lymphocytic leukemias). In CML, the predominant cell population is myeloid, and lymphoid markers are not typically the focus for diagnosis.

Following the confirmation of the Philadelphia chromosome, Mr. Ahmed is diagnosed with Chronic Myeloid Leukemia (CML). The hematologist performs a bone marrow aspirate and biopsy to assess the blast percentage and cellularity. The results show <5% blasts in the bone marrow.
Question 3: Based on the World Health Organization (WHO) criteria, what phase of CML is Mr. Ahmed MOST likely in at this time?
a) Accelerated phase
b) Blast crisis
c) Chronic phase
d) Myeloid sarcoma

That's absolutely right, [REDACTED]]!
The correct answer is c) Chronic phase.
Feedback:
* Correct Answer (c): According to the World Health Organization (WHO) criteria, chronic phase CML is defined by having less than 10% blasts in the peripheral blood and bone marrow. Mr. Ahmed's bone marrow aspirate showed <5% blasts, placing him in the chronic phase.
* Incorrect Answer (a): Accelerated phase is characterized by 10-19% blasts in the peripheral blood or bone marrow, along with other criteria such as increasing white blood cell count unresponsive to therapy, increasing splenomegaly, or the presence of additional chromosomal abnormalities.
* Incorrect Answer (b): Blast crisis is defined by 20% blasts in the peripheral blood or bone marrow or the presence of extramedullary blast proliferation.
* Incorrect Answer (d): Myeloid sarcoma is a tumor of myeloid blasts in an extramedullary site. While it can occur in CML, the phasing of CML based on WHO criteria primarily relies on the blast percentage in the bone marrow and peripheral blood.
You've successfully completed Phase 1, [REDACTED]]! You have a solid understanding of the initial presentation and diagnostic workup of CML.
Alright, [REDACTED]], let's proceed to Phase 2: Induction Therapy and Supportive Care.
Based on Mr. Ahmed's diagnosis of chronic phase CML, the hematologist decides to initiate first-line therapy.
Question 4: Which of the following is the MOST appropriate first-line treatment option for a newly diagnosed patient with chronic phase CML who does not have any significant comorbidities?
a) Hydroxyurea
b) Interferon-alpha
c) Imatinib
d) Allogeneic hematopoietic stem cell transplantation (allo-HSCT)

That's absolutely correct, [REDACTED]]!
The correct answer is c) Imatinib.
Feedback:
* Correct Answer (c): Imatinib is a first-generation tyrosine kinase inhibitor (TKI) that specifically inhibits the BCR-ABL1 tyrosine kinase, the driving force behind CML. It has revolutionized the treatment of CML, leading to high rates of cytogenetic and molecular remission in patients with chronic phase disease. It is the standard first-line treatment for most newly diagnosed patients without significant comorbidities.
* Incorrect Answer (a): Hydroxyurea is a cytoreductive agent that can effectively lower the WBC count and alleviate symptoms, but it does not target the underlying BCR-ABL1 mutation and does not lead to cytogenetic or deep molecular responses. It may be used for rapid initial control of leukocytosis before starting a TKI or in patients who cannot tolerate TKIs, but it is not the preferred first-line therapy.
* Incorrect Answer (b): Interferon-alpha was a standard treatment for CML before the development of TKIs. While it can induce cytogenetic responses in some patients, it has a less favorable toxicity profile and lower rates of deep molecular response compared to TKIs. It is now rarely used as first-line therapy.

* Incorrect Answer (d): Allogeneic hematopoietic stem cell transplantation (allo-HSCT) is a potentially curative therapy for CML, but it carries significant risks of morbidity and mortality. It is generally reserved for patients who fail TKI therapy, progress to accelerated phase or blast crisis, or have specific high-risk features. It is not the standard first-line treatment for most patients in chronic phase
Mr. Ahmed starts on imatinib. After 3 months of therapy, his hematologic response is assessed. His WBC count has normalized, and the splenomegaly has resolved.
Question 5: According to the European LeukemiaNet (ELN) recommendations for monitoring CML patients on tyrosine kinase inhibitors, what is the NEXT recommended assessment to evaluate the depth of response at this 3-month time point?
a) Bone marrow aspirate and cytogenetic analysis
b) Quantitative reverse transcription-polymerase chain reaction (qRT-PCR) for BCR-ABL1 transcript levels in peripheral blood
c) Fluorescence in situ hybridization (FISH) for BCR-ABL1 on peripheral blood
d) Complete blood count (CBC) with differential, repeated monthly
That's absolutely right, [REDACTED]]!
The correct answer is b) Quantitative reverse transcription-polymerase chain reaction (qRT-PCR) for BCR-ABL1 transcript levels in peripheral blood.
Feedback:
* Correct Answer (b): According to the European LeukemiaNet (ELN) recommendations, after achieving a complete hematologic response (normalization of blood counts and resolution of splenomegaly) at 3 months, the next crucial step is to assess the molecular response using qRT-PCR to measure the BCR-ABL1 transcript levels in the peripheral blood. This provides a more sensitive measure of the remaining leukemic burden and helps predict long-term outcomes.
* Incorrect Answer (a): While bone marrow aspirate and cytogenetic analysis are essential for establishing the initial diagnosis and assessing cytogenetic response (e.g., Ph+ percentage), they are more invasive and not routinely recommended at the 3-month time point solely to assess the depth of response, especially after achieving a complete hematologic response. Molecular monitoring with qRT-PCR is preferred for this purpose.
* Incorrect Answer (c): Fluorescence in situ hybridization (FISH) can detect the BCR-ABL1 fusion gene in peripheral blood or bone marrow. However, qRT-PCR is more sensitive for quantifying the level of BCR-ABL1 transcripts and is the preferred method for monitoring molecular response according to ELN guidelines.
* Incorrect Answer (d): While regular CBC monitoring is important to ensure maintenance of hematologic response, it does not provide information about the molecular response or the depth of disease control. qRT-PCR is necessary for this assessment
Mr. Ahmed's qRT-PCR results at 3 months show a BCR-ABL1 transcript level of 15% on the IS (International Scale).
Question 6: According to the ELN 2020 recommendations, what is the optimal response milestone at 3 months for patients with chronic phase CML treated with a first-line TKI to predict a favorable long-term outcome?
a) BCR-ABL1 IS 1%
b) BCR-ABL1 IS 10%
c) Complete cytogenetic response (CCyR)
d) Major molecular response (MMR; BCR-ABL1 IS 0.1%)
That's correct, [REDACTED]]!
The correct answer is b) BCR-ABL1 IS 10%.
Feedback:
* Correct Answer (b): According to the ELN 2020 recommendations, a BCR-ABL1 IS 10% at 3 months is considered an optimal response milestone for patients with chronic phase CML treated with a first-line TKI. Patients achieving this level of molecular response have a significantly higher likelihood of achieving deeper responses and better long-term outcomes, including progression-free and overall survival.
* Incorrect Answer (a): While a BCR-ABL1 IS 1% at 3 months represents an early deep molecular response and is associated with excellent outcomes, it is considered an early molecular response (EMR) and not the minimum optimal response milestone at this time point according to the ELN guidelines.
* Incorrect Answer (c): Complete cytogenetic response (CCyR), defined as 0% Ph+ metaphases in the bone marrow, is an important goal in CML treatment. However, at the 3-month mark, achieving a specific level of molecular response (BCR-ABL1 IS) is considered a more sensitive and earlier predictor of long-term outcome than CCyR. CCyR is typically assessed later, around 6-12 months.
* Incorrect Answer (d): Major molecular response (MMR), defined as BCR-ABL1 IS 0.1% (a 3-log reduction from baseline), is a deeper response than the optimal milestone at 3 months. While achieving MMR earlier is favorable, the optimal milestone at 3 months is BCR-ABL1 IS 10%.
Mr. Ahmed continues on imatinib. At the 12-month follow-up, his BCR-ABL1 IS level is 0.05%.
Question 7: According to the ELN 2020 recommendations, what level of molecular response has Mr. Ahmed achieved at 12 months?
a) Complete molecular response (CMR; MR4.5)

b) Major molecular response (MMR; MR3)
c) Warning response
d) Failure response
That's correct, [REDACTED]]!
The correct answer is b) Major molecular response (MMR; MR3).
Feedback:
 * Correct Answer (b): A BCR-ABL1 IS level of 0.05% represents a 3-log reduction from the standardized baseline (where the BCR-ABL1 level is considered 100%). This level of reduction is defined as a Major Molecular Response (MMR or MR3).
 * Incorrect Answer (a): Complete molecular response (CMR) is generally defined as a deeper reduction in BCR-ABL1 transcript levels, typically MR4.5 (a 4.5-log reduction) or lower (e.g., undetectable BCR-ABL1 by highly sensitive PCR). 0.05% is not yet considered CMR by most standard definitions.
 * Incorrect Answer (c): A warning response according to ELN typically refers to patients who do not achieve the optimal milestones at specific time points (e.g., >10% at 3 months, >1% at 6 months, or >0.1% at 12 months). Since Mr. Ahmed achieved 0.1% at 12 months, this is a good response, not a warning.
 * Incorrect Answer (d): A failure response indicates inadequate response to therapy based on defined molecular or cytogenetic milestones at specific time points. Mr. Ahmed's response of 0.05% at 12 months is a good response, not a treatment failure.

Excellent, [REDACTED]]! Let's move on to Phase 3: Post-Remission Therapy and Long-Term Management.
Mr. Ahmed has been on imatinib for 5 years and has maintained a stable major molecular response (MMR) with BCR-ABL1 IS consistently below 0.1%. He is feeling well with no significant side effects.
Question 8: Which of the following is the MOST appropriate long-term management strategy for Mr. Ahmed at this stage?
a) Discontinuation of imatinib therapy
b) Continuation of imatinib therapy with regular monitoring
c) Switching to a second-generation tyrosine kinase inhibitor (e.g., nilotinib, dasatinib)
d) Reducing the dose of imatinib to the lowest effective dose

That's absolutely right, [REDACTED]]!
The correct answer is b) Continuation of imatinib therapy with regular monitoring.
Feedback:
 * Correct Answer (b): The standard of care for patients who achieve and maintain a stable major molecular response (MMR) on a TKI like imatinib is to continue the TKI therapy indefinitely. Regular monitoring of BCR-ABL1 transcript levels is crucial to ensure continued disease control and to detect any loss of response early.
 * Incorrect Answer (a): Discontinuation of TKI therapy is an area of active research, but it is not currently recommended as a standard approach for most patients. While some patients in deep molecular remission may be eligible for a treatment-free remission (TFR) attempt under strict monitoring protocols, this decision should be made in consultation with an expert hematologist and is not appropriate for all patients.
 * Incorrect Answer (c): Switching to a second-generation TKI (e.g., nilotinib, dasatinib) is typically considered for patients who are intolerant to imatinib, have suboptimal responses, or develop resistance. Since Mr. Ahmed is tolerating imatinib well and has achieved a stable MMR, switching to a different TKI is not warranted.
 * Incorrect Answer (d): Dose reduction may be considered in some cases to manage side effects, but it is not the primary strategy for long-term management in patients who are tolerating the TKI well and have achieved a deep molecular response.
Mr. Ahmed develops new-onset hypertension that requires initiation of antihypertensive medication. His current medications include imatinib 400 mg daily.
Question 9: Which of the following antihypertensive drug classes has the MOST significant potential for drug-drug interactions with imatinib due to shared metabolic pathways?
a) Beta-blockers (e.g., metoprolol)
b) Angiotensin-converting enzyme (ACE) inhibitors (e.g., lisinopril)
c) Calcium channel blockers (e.g., amlodipine)
d) Strong CYP3A4 inhibitors (e.g., diltiazem)

That's absolutely correct, [REDACTED]]!
The correct answer is d) Strong CYP3A4 inhibitors (e.g., diltiazem).
Feedback:
 * Correct Answer (d): Strong CYP3A4 inhibitors can significantly inhibit the metabolism of imatinib, leading to increased plasma

concentrations of imatinib and potentially increasing the risk of its adverse effects. Diltiazem is a calcium channel blocker known to be a moderate to strong inhibitor of CYP3A4. Therefore, using strong CYP3A4 inhibitors concurrently with imatinib requires careful monitoring and potential dose adjustments of imatinib.
 * Incorrect Answer (a): Beta-blockers like metoprolol are primarily metabolized by CYP2D6, not CYP3A4, so the risk of significant interaction with imatinib through this pathway is lower.
 * Incorrect Answer (b): ACE inhibitors like lisinopril are not primarily metabolized by the CYP450 system and are less likely to have significant pharmacokinetic interactions with imatinib.
 * Incorrect Answer (c): While some calcium channel blockers are metabolized by CYP3A4 (e.g., amlodipine to a lesser extent than diltiazem), the strong inhibitors of CYP3A4 pose the greatest risk of interaction with imatinib.
Mr. Ahmed, who is in stable MMR on imatinib, expresses interest in potentially discontinuing therapy in the future.
Question 10: Which of the following factors is generally considered FAVORABLE for a patient with chronic phase CML to be considered a candidate for a treatment-free remission (TFR) attempt?
a) Achieving MMR within the first 3 months of TKI therapy
b) Duration of stable deep molecular response (e.g., MR4.5) for at least 2 years
c) History of prior accelerated phase of CML
d) Presence of significant comorbidities requiring multiple medications

That's absolutely correct, [REDACTED]]!
The correct answer is b) Duration of stable deep molecular response (e.g., MR4.5) for at least 2 years.
Feedback:
 * Correct Answer (b): Achieving and maintaining a stable deep molecular response (typically defined as MR4 or MR4.5, representing a 4- or 4.5-log reduction in BCR-ABL1 levels) for a prolonged period, generally at least 2 years, is one of the most important favorable factors for considering a TFR attempt. This suggests a very low residual disease burden and a higher likelihood of maintaining remission off therapy.
 * Incorrect Answer (a): While achieving MMR early (e.g., within 3 months) is a positive prognostic indicator, it is not a primary criterion for TFR consideration. The duration of a deep response is more critical.
 * Incorrect Answer (c): A history of prior accelerated phase of CML indicates a more aggressive disease course and a higher risk of relapse. These patients are generally not considered good candidates for TFR attempts.
 * Incorrect Answer (d): The presence of significant comorbidities requiring multiple medications can complicate the management of potential relapse after TKI discontinuation and may make close monitoring more challenging. It is generally considered a less favorable factor for TFR.

Session ID:
Assessment Domain

Session-62:
Rationale

Session ID:
Instructional Design and Pedagogical

Session-62:
The session demonstrates excellent instructional design. The clinical case is expertly scaffolded, progressing logically from initial presentation and diagnosis (Questions 1-3), through first-line treatment and monitoring milestones (Questions 4-7), and into complex long-term management considerations like drug interactions and treatment discontinuation (Questions 8-10). The embedded feedback is a major strength; each rationale is detailed, accurate, explains the distractors, and implicitly aligns with current clinical standards (e.g., WHO criteria, ELN recommendations). Instructions are exceptionally clear. The only minor area for improvement is the lack of explicitly stated, measurable learning objectives at the outset, though the educational goals are strongly implied by the case's structure.

Session ID:
Clinical Authenticity and Content Fidelity

Session-62:

The clinical fidelity of this session is outstanding and represents a gold standard for this type of educational material. Plausibility & Guideline Alignment: The clinical narrative is indistinguishable from an authentic CML case. The patient presentation, diagnostic workup, treatment selection (imatinib), and monitoring milestones (qRT-PCR targets at 3 and 12 months) are all perfectly aligned with current, evidence-based guidelines from authorities like the European LeukemiaNet (ELN). Pharmacotherapeutic Accuracy: All therapeutic recommendations and concepts are optimal and well-justified. The choice of imatinib as first-line therapy, the discussion of drug interactions via CYP3A4, and the criteria for considering treatment-free remission are all accurate and reflect expert-level knowledge. Domain Specificity (Absence of Entanglement): The session maintains perfect domain specificity. The content remains strictly focused on Chronic Myeloid Leukemia. It correctly distinguishes CML from other hematologic malignancies like AML (e.g., noting Auer rods are not a feature of CML) and does not incorrectly apply therapies or concepts from other disease states. There are no instances of domain entanglement.

Session ID:

Modeled Clinical Reasoning

Session-62:

The simulation excels at modeling an expert clinical reasoning process. Each stage of the case presents a well-defined clinical problem that requires a specific decision. The multiple-choice options are thoughtfully constructed, including a range of relevant and plausible alternatives that effectively probe the user's understanding of nuanced clinical concepts. The decision-making pathway modeled in the case is sound, prioritizing diagnostics, appropriate therapy initiation, and guideline-concordant monitoring. The clinical outcomes described for each decision are realistic and instructionally valuable, reinforcing the learning objectives at each step.

Session-63

Session ID:
LLM

Session-63:
DeepSeek V2

Session ID:
Session Transcript

Session-63:
Okay, [REDACTED]], let's begin with Phase 1 of our CML case.
Phase 1: Initial Presentation and Diagnostic Workup
A 58-year-old male, Mr. John Smith, presents to his primary care physician with complaints of fatigue, night sweats, and unintentional weight loss of approximately 10 lbs over the past 3 months. He denies any fever, bone pain, or easy bruising. His past medical history is significant for well-controlled hypertension and hyperlipidemia.
Initial laboratory investigations reveal the following:
 * White blood cell (WBC) count: 155 x 10^9/L (Reference range: 4-10 x 10^9/L)
 * Hemoglobin: 11.5 g/dL (Reference range: 13.5-17.5 g/dL)
 * Platelet count: 480 x 10^9/L (Reference range: 150-400 x 10^9/L)
 * Peripheral blood smear shows a left shift with granulocytes at various stages of maturation, including blasts (<5%), promyelocytes, myelocytes, metamyelocytes, and mature neutrophils. Basophils and eosinophils are increased.
.
1)Based on these initial findings, which of the following is the most likely next step in establishing a diagnosis according to the 2020 European LeukemiaNet (ELN) recommendations?
A) Repeat complete blood count (CBC) in one week.
B) Bone marrow aspiration and biopsy with cytogenetic analysis, including fluorescence in situ hybridization (FISH) for BCR-ABL1.
C) Lymph node biopsy to rule out lymphoma.
D) Blood culture to rule out infection.

Answer b

Phase 1: Initial Presentation and Diagnostic Workup
A 58-year-old male, Mr. John Smith, presents to his primary care physician with complaints of fatigue, night sweats, and unintentional weight loss of approximately 10 lbs over the past 3 months. He denies any fever, bone pain, or easy bruising. His past medical history is significant for well-controlled hypertension and hyperlipidemia.
Initial laboratory investigations reveal the following:
 * White blood cell (WBC) count: 155 x 10^9/L (Reference range: 4-10 x 10^9/L)
 * Hemoglobin: 11.5 g/dL (Reference range: 13.5-17.5 g/dL)
 * Platelet count: 480 x 10^9/L (Reference range: 150-400 x 10^9/L)
 * Peripheral blood smear shows a left shift with granulocytes at various stages of maturation, including blasts (<5%), promyelocytes, myelocytes, metamyelocytes, and mature neutrophils. Basophils and eosinophils are increased.
Considering the 2020 ELN recommendations for the diagnosis of CML, which of the options (A, B, C, or D) directly addresses the need for definitive confirmation and molecular characterization?
B
. 1: Initial Presentation and Diagnostic Workup
A 58-year-old male, Mr. John Smith, presents to his primary care physician with complaints of fatigue, night sweats, and unintentional weight loss of approximately 10 lbs over the past 3 months. He denies any fever, bone pain, or easy bruising. His past medical history is significant for well-controlled hypertension and hyperlipidemia.
Initial laboratory investigations reveal the following:
 * White blood cell (WBC) count: 155 x 10^9/L (Reference range: 4-10 x 10^9/L)

 * Hemoglobin: 11.5 g/dL (Reference range: 13.5-17.5 g/dL)
 * Platelet count: 480 x 10^9/L (Reference range: 150-400 x 10^9/L)
 * Peripheral blood smear shows a left shift with granulocytes at various stages of maturation, including blasts (<5%), promyelocytes, myelocytes, metamyelocytes, and mature neutrophils. Basophils and eosinophils are increased.

Following the bone marrow aspiration and biopsy, the cytogenetic analysis reveals the presence of the Philadelphia chromosome t(9;22)(q34;q11.2), and FISH confirms the BCR-ABL1 fusion gene. Quantitative RT-PCR analysis of the bone marrow aspirate shows a BCR-ABL1 transcript level of 95% on the IS (International Scale).

According to the 2022 European LeukemiaNet (ELN) risk stratification for newly diagnosed chronic phase CML, which risk category does Mr. Smith fall into?

To answer this question, you'll need to consider the ELN 2022 risk score criteria, which include factors such as:
 * Sokal score (age, spleen size, platelet count, blast percentage)
 * Hasford score (hemoglobin, WBC count, blast percentage, eosinophil percentage, basophil percentage, platelet count, age)
 * EUTOS score (basophil percentage, blast percentage)

While I haven't provided all the individual parameters for each score, the BCR-ABL1 transcript level at diagnosis is a critical factor in the ELN 2022 risk stratification. Based on this information and your knowledge of ELN criteria, which risk category is most likely for Mr. Smith?

A) Low risk
B) Intermediate risk
C) High risk
D) Not enough information to determine

D .

.

Phase 1: Initial Presentation and Diagnostic Workup

A 58-year-old male, Mr. John Smith, presents to his primary care physician with complaints of fatigue, night sweats, and unintentional weight loss of approximately 10 lbs over the past 3 months. He denies any fever, bone pain, or easy bruising. His past medical history is significant for well-controlled hypertension and hyperlipidemia.

Initial laboratory investigations reveal the following:
 * White blood cell (WBC) count: 155 x 10^9/L (Reference range: 4-10 x 10^9/L)
 * Hemoglobin: 11.5 g/dL (Reference range: 13.5-17.5 g/dL)
 * Platelet count: 480 x 10^9/L (Reference range: 150-400 x 10^9/L)
 * Peripheral blood smear shows a left shift with granulocytes at various stages of maturation, including blasts (<5%), promyelocytes, myelocytes, metamyelocytes, and mature neutrophils. Basophils and eosinophils are increased

Okay, [REDACTED]]. Now that Mr. Smith has been diagnosed with chronic phase CML and we understand that we need more information to formally risk stratify him according to ELN 2022, let's consider the initial goals of therapy.

According to the ESMO guidelines for the management of CML, what is the primary goal of first-line treatment for a patient in chronic phase CML like Mr. Smith?

A) Achieve complete hematologic remission (CHR) within 3 months.
B) Achieve a major molecular response (MMR) with a BCR-ABL1 transcript level 0.1% IS within 12 months.
C) Achieve a complete cytogenetic response (CCyR) within 12 months.
D) Achieve optimal response milestones at 3, 6, and 12 months to prevent disease progression and improve long-term survival.

!

D) Achieve optimal response milestones at 3, 6, and 12 months to prevent disease progression and improve long-term survival. is the most accurate answer according to the ESMO guidelines.

Feedback:
 * Correct Answer: You've correctly identified that the overarching goal of first-line CML treatment is not just about achieving a single remission marker at one point in time, but rather about achieving a series of milestones that indicate a durable and effective response, ultimately leading to improved long-term outcomes.
 * Your Reasoning: Your understanding that the focus is on preventing progression and improving survival aligns perfectly with the current management paradigm for CML. Regular monitoring and achievement of early response milestones are crucial for guiding treatment decisions.

* Why other options are important but not the primary goal:
  * A) Achieve complete hematologic remission (CHR) within 3 months: CHR (normalization of blood counts and resolution of signs/symptoms) is an important early goal and a prerequisite for deeper responses. However, achieving CHR alone is not sufficient for long-term disease control.
  * B) Achieve a major molecular response (MMR) with a BCR-ABL1 transcript level 0.1% IS within 12 months: MMR is a significant milestone associated with improved long-term outcomes and is a key target. However, the primary goal encompasses a broader strategy of achieving and maintaining optimal responses at various time points.
  * C) Achieve a complete cytogenetic response (CCyR) within 12 months: CCyR (absence of Ph+ metaphases in the bone marrow) is another crucial goal linked to better outcomes. Similar to MMR, it's a significant milestone within the overall treatment strategy.

Key takeaway from ESMO Guidelines:

The ESMO guidelines emphasize a response-based approach to CML management. The primary goal is to achieve and maintain an optimal response, defined by specific hematologic, cytogenetic, and molecular milestones at defined time points (3, 6, and 12 months). Failure to achieve these milestones should prompt consideration of treatment modification. This strategy aims to prevent disease progression to accelerated or blast phase and ultimately improve the patient's long-term survival and quality of life.

This concludes Phase 1 of our case. You've done well in navigating the initial diagnostic workup and understanding the primary treatment goals.

Are you ready to move on to Phase 2, where we will discuss induction therapy selection and supportive care planning for Mr. Smith? Type 'YES' to proceed.

Phase 1: Initial Presentation and Diagnostic Workup

A 58-year-old male, Mr. John Smith, presents to his primary care physician with complaints of fatigue, night sweats, and unintentional weight loss of approximately 10 lbs over the past 3 months. He denies any fever, bone pain, or easy bruising. His past medical history is significant for well-controlled hypertension and hyperlipidemia.

Initial laboratory investigations reveal the following:

* White blood cell (WBC) count: 155 x 10^9/L (Reference range: 4-10 x 10^9/L)
* Hemoglobin: 11.5 g/dL (Reference range: 13.5-17.5 g/dL)
* Platelet count: 480 x 10^9/L (Reference range: 150-400 x 10^9/L)
* Peripheral blood smear shows a left shift with granulocytes at various stages of maturation, including blasts (<5%), promyelocytes, myelocytes, metamyelocytes, and mature neutrophils. Basophils and eosinophils are increased

Great, [REDACTED]]! Let's move on to Phase 2: Induction Therapy Selection and Supportive Care Planning.

Based on the diagnosis of chronic phase CML in Mr. Smith, and considering he has no significant comorbidities other than well-controlled hypertension and hyperlipidemia, which of the following would be the recommended first-line treatment option according to the ESMO guidelines?

A) Hydroxyurea to rapidly reduce the white blood cell count, followed by interferon-alpha.

B) Standard-dose imatinib (400 mg daily).

C) A second-generation tyrosine kinase inhibitor (TKI) such as nilotinib, dasatinib, or bosutinib.

D) Allogeneic hematopoietic stem cell transplantation (allo-HSCT).

. Standard-dose imatinib was indeed a significant breakthrough in the management of this disease and is still a valid option in certain contexts.

However, let's think about the current ESMO guidelines for first-line treatment of newly diagnosed chronic phase CML, particularly in a patient without high-risk features or significant comorbidities. How do the second-generation TKIs compare to imatinib in terms of speed and depth of response, and what do the guidelines generally recommend in this setting?

Consider the potential for achieving faster and deeper molecular responses, which are associated with improved long-term outcomes. Based on this, would you like to reconsider your answer?

Yes

You're absolutely right to reconsider, [REDACTED]]. Based on current ESMO guidelines, C) A second-generation tyrosine kinase inhibitor (TKI) such as nilotinib, dasatinib, or bosutinib is generally the recommended first-line treatment option for most newly diagnosed chronic phase CML patients without high-risk features.

Feedback:

* Correct Answer: Second-generation TKIs have demonstrated faster and deeper cytogenetic and molecular responses compared to standard-dose imatinib in several large clinical trials. These deeper and earlier responses are associated with improved long-term outcomes, including a lower risk of disease progression.

* Why other options are less preferred as first-line in this scenario:
  * A) Hydroxyurea to rapidly reduce the white blood cell count, followed by interferon-alpha: Hydroxyurea is often used for initial cytoreduction in patients with very high WBC counts to prevent leukostasis, but it does not target the underlying BCR-ABL1 kinase. Interferon-alpha has some efficacy in CML but has been largely superseded by TKIs due to lower response rates and significant side effects.
  * B) Standard-dose imatinib (400 mg daily): While still an effective first-line agent, particularly in low-risk patients or where second-generation TKIs are not available or tolerated, second-generation TKIs are generally preferred due to their superior efficacy in achieving faster and deeper responses.
  * D) Allogeneic hematopoietic stem cell transplantation (allo-HSCT): Allo-HSCT is potentially curative but carries a significant risk of morbidity and mortality. It is generally reserved for patients who fail TKI therapy or present in accelerated or blast phase. In the chronic phase, it's not the standard first-line treatment, especially in an otherwise healthy 58-year-old.

Key takeaway from ESMO Guidelines:
For most newly diagnosed chronic phase CML patients, the ESMO guidelines recommend considering a second-generation TKI (nilotinib, dasatinib, or bosutinib) as first-line therapy due to their ability to induce faster and deeper responses, which correlate with better long-term outcomes. The choice among these agents may depend on patient-specific factors, comorbidities, and the drug's side effect profile.

.

.

Phase 1: Initial Presentation and Diagnostic Workup
A 58-year-old male, Mr. John Smith, presents to his primary care physician with complaints of fatigue, night sweats, and unintentional weight loss of approximately 10 lbs over the past 3 months. He denies any fever, bone pain, or easy bruising. His past medical history is significant for well-controlled hypertension and hyperlipidemia.
Initial laboratory investigations reveal the following:

* White blood cell (WBC) count: 155 x 10^9/L (Reference range: 4-10 x 10^9/L)
* Hemoglobin: 11.5 g/dL (Reference range: 13.5-17.5 g/dL)
* Platelet count: 480 x 10^9/L (Reference range: 150-400 x 10^9/L)
* Peripheral blood smear shows a left shift with granulocytes at various stages of maturation, including blasts (<5%), promyelocytes, myelocytes, metamyelocytes, and mature neutrophils. Basophils and eosinophils are increased

Now, let's say Mr. Smith is started on a second-generation TKI. What is a common and important supportive care measure that should be implemented concurrently, particularly given his age and history of hypertension and hyperlipidemia?
A) Routine blood transfusions to prevent anemia.
B) Aggressive hydration and allopurinol to prevent tumor lysis syndrome.
C) Prophylactic antibiotics and antifungals to prevent infections.
D) Cardiovascular risk assessment and management.

D) Cardiovascular risk assessment and management is a crucial supportive care measure for Mr. Smith.
Feedback:

* Correct Answer: You've correctly identified a vital aspect of supportive care in CML patients, especially those with pre-existing cardiovascular risk factors.
* Your Reasoning: Patients with CML, particularly those being treated with certain TKIs (some second-generation TKIs more so than others), can have an increased risk of cardiovascular events, including hypertension, arterial occlusive events, and QT prolongation. Mr. Smith's age and history of hypertension and hyperlipidemia further elevate this risk. Therefore, a thorough baseline cardiovascular risk assessment and proactive management of these risk factors are essential.
* Why other options are less likely as a general and concurrent supportive measure in this specific scenario:
  * A) Routine blood transfusions to prevent anemia: While anemia can be present at diagnosis, routine prophylactic transfusions are not indicated unless the patient is symptomatic or has severe anemia. The goal of TKI therapy is to improve hematopoiesis.
  * B) Aggressive hydration and allopurinol to prevent tumor lysis syndrome: Tumor lysis syndrome is more common in hematologic malignancies with high cell turnover, such as acute leukemias or lymphomas, especially after the initiation of cytotoxic chemotherapy. While it can theoretically occur in CML with very high WBC counts, it's less of an immediate concern with TKI initiation compared to these other conditions. However, monitoring electrolytes is still important.

  * C) Prophylactic antibiotics and antifungals to prevent infections: Routine broad-spectrum antimicrobial prophylaxis is generally not indicated in the chronic phase of CML unless the patient develops neutropenia as a consequence of the disease or treatment.

Key takeaway on Supportive Care:

Comprehensive supportive care in CML involves addressing potential complications of the disease and its treatment, as well as managing pre-existing comorbidities. Cardiovascular risk assessment and management are particularly important in patients with risk factors, especially when using TKIs that may have cardiovascular side effects.

.

Phase 1: Initial Presentation and Diagnostic Workup

A 58-year-old male, Mr. John Smith, presents to his primary care physician with complaints of fatigue, night sweats, and unintentional weight loss of approximately 10 lbs over the past 3 months. He denies any fever, bone pain, or easy bruising. His past medical history is significant for well-controlled hypertension and hyperlipidemia.

Initial laboratory investigations reveal the following:

 * White blood cell (WBC) count: 155 x 10^9/L (Reference range: 4-10 x 10^9/L)
 * Hemoglobin: 11.5 g/dL (Reference range: 13.5-17.5 g/dL)
 * Platelet count: 480 x 10^9/L (Reference range: 150-400 x 10^9/L)
 * Peripheral blood smear shows a left shift with granulocytes at various stages of maturation, including blasts (<5%), promyelocytes, myelocytes, metamyelocytes, and mature neutrophils. Basophils and eosinophils are increased

Now, let's consider the initial response assessment. Mr. Smith has been on his chosen first-line TKI for 3 months. A follow-up peripheral blood count shows:

 * WBC count: 5 x 10^9/L
 * Hemoglobin: 13.0 g/dL
 * Platelet count: 250 x 10^9/L

His symptoms of fatigue and night sweats have resolved. According to the ELN 2020 response criteria at 3 months, what is Mr. Smith's hematologic response?

A) No hematologic response

B) Suboptimal hematologic response

C) Complete hematologic response (CHR)

D) Warning (loss of prior CHR) - This is not applicable at the 3-month mark.

C) Complete hematologic response (CHR) is the correct answer according to the ELN 2020 response criteria at 3 months.

Feedback:

 * Correct Answer: You have accurately interpreted the hematologic response criteria.
 * ELN 2020 Criteria for Complete Hematologic Response (CHR): At any time point, CHR is defined by all of the following:
   * White blood cell (WBC) count < 10 x 10^9/L
   * Absolute neutrophil count (ANC) 1.5 x 10^9/L
   * Platelet count < 450 x 10^9/L
   * No blasts or promyelocytes in peripheral blood
   * Absence of extramedullary disease (e.g., splenomegaly, hepatomegaly if previously present)

In Mr. Smith's case at 3 months:

 * His WBC count is 5 x 10^9/L (within normal limits).
 * His hemoglobin is 13.0 g/dL (within normal limits).
 * His platelet count is 250 x 10^9/L (within normal limits).
 * His symptoms have resolved, suggesting no evidence of extramedullary disease.

Therefore, he has achieved a complete hematologic response.

.

Phase 1: Initial Presentation and Diagnostic Workup

A 58-year-old male, Mr. John Smith, presents to his primary care physician with complaints of fatigue, night sweats, and unintentional weight loss of approximately 10 lbs over the past 3 months. He denies any fever, bone pain, or easy bruising. His past medical history is significant for well-controlled hypertension and hyperlipidemia.

Initial laboratory investigations reveal the following:

 * White blood cell (WBC) count: 155 x 10^9/L (Reference range: 4-10 x 10^9/L)
 * Hemoglobin: 11.5 g/dL (Reference range: 13.5-17.5 g/dL)
 * Platelet count: 480 x 10^9/L (Reference range: 150-400 x 10^9/L)
 * Peripheral blood smear shows a left shift with granulocytes at various stages of maturation, including blasts (<5%), promyelocytes,

myelocytes, metamyelocytes, and mature neutrophils. Basophils and eosinophils are increased

Now, let's consider the initial response assessment. Mr. Smith has been on his chosen first-line TKI for 3 months. A follow-up peripheral blood count shows:

* WBC count: 5 x 10^9/L
* Hemoglobin: 13.0 g/dL
* Platelet count: 250 x 10^9/L

Now, let's consider the initial response assessment. Mr. Smith has been on his chosen first-line TKI for 3 months. A follow-up peripheral blood count shows:

* WBC count: 5 x 10^9/L
* Hemoglobin: 13.0 g/dL
* Platelet count: 250 x 10^9/L

His symptoms of fatigue and night sweats have resolved. According to the ELN 2020 response criteria at 3 months, what is Mr. Smith's hematologic response?

A) No hematologic response

B) Suboptimal hematologic response

C) Complete hematologic response (CHR)

D) Warning (loss of prior CHR) - This is not applicable at the 3-month mark.

C) Complete hematologic response (CHR) is the correct answer according to the ELN 2020 response criteria at 3 months.

Feedback:

* Correct Answer: You have accurately interpreted the hematologic response criteria.
* ELN 2020 Criteria for Complete Hematologic Response (CHR): At any time point, CHR is defined by all of the following:
  * White blood cell (WBC) count < 10 x 10^9/L
  * Absolute neutrophil count (ANC) 1.5 x 10^9/L
  * Platelet count < 450 x 10^9/L
  * No blasts or promyelocytes in peripheral blood
  * Absence of extramedullary disease (e.g., splenomegaly, hepatomegaly if previously present)

In Mr. Smith's case at 3 months:

* His WBC count is 5 x 10^9/L (within normal limits).
* His hemoglobin is 13.0 g/dL (within normal limits).
* His platelet count is 250 x 10^9/L (within normal limits).
* His symptoms have resolved, suggesting no evidence of extramedullary disease.

Therefore, he has achieved a complete hematologic response.

Okay, [REDACTED]], let's begin with Phase 1 of our CML case.

Phase 1: Initial Presentation and Diagnostic Workup

A 58-year-old male, Mr. John Smith, presents to his primary care physician with complaints of fatigue, night sweats, and unintentional weight loss of approximately 10 lbs over the past 3 months. He denies any fever, bone pain, or easy bruising. His past medical history is significant for well-controlled hypertension and hyperlipidemia.

Initial laboratory investigations reveal the following:

* White blood cell (WBC) count: 155 x 10^9/L (Reference range: 4-10 x 10^9/L)
* Hemoglobin: 11.5 g/dL (Reference range: 13.5-17.5 g/dL)
* Platelet count: 480 x 10^9/L (Reference range: 150-400 x 10^9/L)
* Peripheral blood smear shows a left shift with granulocytes at various stages of maturation, including blasts (<5%), promyelocytes, myelocytes, metamyelocytes, and mature neutrophils. Basophils and eosinophils are increased

N. Mr. Smith has been on his chosen first-line TKI for 3 months. A follow-up peripheral blood count shows:

* WBC count: 5 x 10^9/L
* Hemoglobin: 13.0 g/dL
* Platelet count: 250 x 10^9/L Therefore, he has achieved a complete hematologic response.

Now, let's consider the next step in monitoring his response. According to the ESMO guidelines, what is the recommended next assessment to evaluate the depth of his response at this 3-month time point, beyond the peripheral blood count?

A) Bone marrow aspiration and cytogenetic analysis for Philadelphia chromosome.

B) Quantitative RT-PCR for BCR-ABL1 transcript level in peripheral blood.

C) Fluorescence in situ hybridization (FISH) on peripheral blood.

D) Spleen size measurement by physical exam or imaging.
You've got it, [REDACTED]]!
B) Quantitative RT-PCR for BCR-ABL1 transcript level in peripheral blood is the recommended next assessment according to the ESMO guidelines to evaluate the depth of response at the 3-month time point, beyond the peripheral blood count.
Feedback:
 * Correct Answer: You're absolutely right. While achieving a CHR is an important initial goal, assessing the molecular response using BCR-ABL1 transcript levels is crucial for evaluating the depth of response and predicting long-term outcomes.
 * Why other options are less prioritized at this specific 3-month time point for response assessment:
   * A) Bone marrow aspiration and cytogenetic analysis for Philadelphia chromosome: Bone marrow assessment, including cytogenetics, is typically performed at diagnosis and then at later time points (e.g., 6-12 months) to assess for cytogenetic response (CCyR). It's more invasive and not routinely done at 3 months in patients achieving CHR.
   * C) Fluorescence in situ hybridization (FISH) on peripheral blood: FISH can be used to detect the BCR-ABL1 fusion gene, but quantitative RT-PCR is the preferred method for monitoring the level of BCR-ABL1 transcripts and assessing molecular response depth.
   * D) Spleen size measurement by physical exam or imaging: Spleen size is important at diagnosis and for assessing hematologic response. However, molecular monitoring with BCR-ABL1 levels provides a more sensitive and quantitative measure of disease burden and response to TKI therapy.
Key takeaway from ESMO Guidelines on Response Monitoring:
The ESMO guidelines emphasize the importance of early molecular monitoring using quantitative RT-PCR for BCR-ABL1 in peripheral blood. Achieving early molecular response milestones (e.g., BCR-ABL1 10% IS at 3 months) is strongly associated with better long-term outcomes.
This concludes Phase 2 of our case. You've successfully navigated the initial treatment selection and early response assessment.
Are you ready to move on to Phase 3, where we will discuss post-remission therapy, MRD monitoring, and long-term management for Mr. Smith? Type 'YES' to proceed.

Excellent, [REDACTED]]! Let's proceed to Phase 3: Post-Remission Therapy, MRD Monitoring, and Long-Term Management.
At 3 months, Mr. Smith's BCR-ABL1 transcript level by quantitative RT-PCR is 8% on the International Scale (IS). According to the ELN 2020 response criteria at 3 months, this molecular response would be categorized as:
A) Optimal response
B) Warning (suboptimal molecular response)
C) Failure (no significant reduction in BCR-ABL1)
D) Major molecular response (MMR) - Note: MMR is typically assessed at later time points.

You are absolutely correct, [REDACTED]]!
According to the ELN 2020 response criteria at 3 months, a BCR-ABL1 transcript level of 10% IS is considered an A) Optimal response.
Feedback:
 * Correct Answer: You have accurately applied the ELN 2020 criteria for early molecular response.
 * ELN 2020 Response Criteria at 3 Months:
   * Optimal Response: BCR-ABL1 10% IS
   * Warning (Suboptimal Response): BCR-ABL1 > 10% but 100% IS (no significant reduction from baseline)
   * Failure: BCR-ABL1 > 100% IS or doubling of BCR-ABL1 from baseline
Since Mr. Smith's BCR-ABL1 level is 8% IS at 3 months, he has achieved an optimal early molecular response. This is a positive indicator for long-term outcome..

Session ID:
Assessment Domain

Session-63:
Rationale

Session ID:
Instructional Design and Pedagogical

Session-63:

The session's instructional design is of very high quality. The clinical case is expertly scaffolded, progressing in a logical and intuitive sequence from initial presentation and diagnosis through to first-line treatment selection, supportive care, and early response monitoring. The quality of the embedded feedback is a significant strength; rationales are detailed, consistently reference current clinical guidelines (ELN, ESMO), and explain why incorrect options are less appropriate. This reinforces learning effectively. Instructions are exceptionally clear, guiding the user through each phase of the case. The only minor area for improvement is the lack of explicitly stated, measurable learning objectives at the very beginning of the session, though the educational goals are clearly implied by the session's structure and content.

Session ID:

Clinical Authenticity and Content Fidelity

Session-63:

The session demonstrates outstanding clinical fidelity and content accuracy. Plausibility & Guideline Alignment: The clinical narrative is indistinguishable from an authentic CML case, with classic presenting symptoms and laboratory findings. All diagnostic, therapeutic, and monitoring recommendations are perfectly aligned with current, evidence-based guidelines from authoritative bodies like ELN and ESMO. This includes the diagnostic workup, the preference for second-generation TKIs, the emphasis on cardiovascular risk assessment, and the specific criteria for optimal response at 3 months (BCR-ABL1 10% IS). Pharmacotherapeutic Accuracy & Domain Specificity: The pharmacotherapeutic choices and rationales are optimal and well-justified. The session correctly identifies the standard of care for each decision point. Crucially, the content is entirely free of domain entanglement; all concepts, diagnostics, and therapies are specific and appropriate to the management of Chronic Myeloid Leukemia. No critical content errors were identified.

Session ID:

Modeled Clinical Reasoning

Session-63:

The simulation expertly models the clinical reasoning process of a skilled practitioner. Each stage of the case presents a well-framed clinical problem, providing the necessary data for an informed decision. The multiple-choice options are thoughtfully constructed, offering a robust breadth of relevant and plausible alternatives that effectively test nuanced understanding (e.g., including imatinib as a valid, but less preferred, first-line option). The overall decision-making strategy is sound, demonstrating appropriate prioritization, such as securing a molecular diagnosis before therapy and focusing on early molecular milestones as a predictor of long-term success. The clinical outcomes described are realistic and serve as valuable teaching points that reinforce the preceding decisions.

Session-64

Session ID:
LLM

Session-64:
DeepSeek V2

Session ID:
Session Transcript

Session-64:
Okay, let's begin with Phase 1 of the case.
Phase 1: Initial Presentation and Diagnostic Workup
A 58-year-old male presents to his primary care physician with complaints of fatigue, unintentional weight loss of 5 kg over the past 3 months, and early satiety. Physical examination reveals splenomegaly. Initial laboratory investigations show the following:
 * White blood cell (WBC) count: 185 x 10^9/L (normal: 4-10 x 10^9/L)
 * Hemoglobin: 10.5 g/dL (normal: 13.5-17.5 g/dL)
 * Platelet count: 450 x 10^9/L (normal: 150-400 x 10^9/L)
 * Peripheral blood smear shows a left shift with myeloid precursors at various stages of maturation.
A bone marrow aspirate and biopsy are performed. Cytogenetic analysis reveals the presence of the Philadelphia chromosome, t(9;22)(q34;q11.2). BCR-ABL1 transcript analysis by quantitative real-time PCR (qRT-PCR) shows a BCR-ABL1/ABL1 ratio of 95% on the International Scale (IS).
Question 1: Based on the initial clinical presentation and diagnostic workup, which of the following is the most likely diagnosis according to the 2022 WHO classification of myeloid neoplasms?
a) Myelodysplastic Syndrome (MDS) with excess blasts
b) Acute Myeloid Leukemia (AML) with minimal differentiation
c) Chronic Myeloid Leukemia (CML), chronic phase
d) Primary Myelofibrosis (PMF), prefibrotic stage

That is correct, c) Chronic Myeloid Leukemia (CML), chronic phase is the most likely diagnosis.
Feedback:
 * Your answer aligns with the diagnostic criteria for CML according to the 2022 WHO classification. The presence of the Philadelphia chromosome [t(9;22)], the significantly elevated WBC count with a myeloid left shift, splenomegaly, and the BCR-ABL1 transcript are all characteristic features of CML in the chronic phase.
 * Let's briefly review why the other options are less likely:
   * a) Myelodysplastic Syndrome (MDS) with excess blasts: While MDS can present with cytopenias and some increase in blasts, the presence of the Philadelphia chromosome is diagnostic of CML.
   * b) Acute Myeloid Leukemia (AML) with minimal differentiation: AML is characterized by a high percentage of blasts (20%) in the bone marrow and peripheral blood, which is not indicated in this case. The presence of the Philadelphia chromosome also points towards CML rather than de novo AML (although Ph+ AML can occur, it usually presents differently).
   * d) Primary Myelofibrosis (PMF), prefibrotic stage: PMF is a myeloproliferative neoplasm characterized by bone marrow fibrosis, which is not indicated in the initial workup described. While splenomegaly can be present, the high WBC count and Philadelphia chromosome are not typical of PMF
Question 2: According to the 2022 European LeukemiaNet (ELN) risk stratification for newly diagnosed CML in chronic phase, what is this patient's risk category based on the available information (WBC count, spleen size is not provided, blast percentage is assumed to be <1% based on chronic phase presentation)?
The ELN 2022 risk score considers factors like:
 * Sokal risk score (which includes spleen size and blast percentage)
 * Hasford score (which includes blast percentage)
 * EUTOS long-term survival (ELTS) score (which includes blast percentage)
Given the limited information, we'll focus on the WBC count as a key differentiating factor in some simplified risk assessments at diagnosis. While a full ELN risk assessment requires more data, consider how the very high WBC count might influence the initial risk

categorization.
a) Low risk
b) Intermediate risk
c) High risk
d) Unable to determine risk with the given information
You're on the right track to consider the high WBC count as a significant factor! However, the 2022 ELN risk stratification primarily relies on the Sokal, Hasford, or ELTS scores, all of which incorporate blast percentage (typically <1% in chronic phase), spleen size (which is not provided), and other factors like platelet count and age.
Therefore, while the very high WBC count (185 x 10^9/L) is generally associated with a less favorable prognosis compared to lower WBC counts within the risk score calculations, we d) Unable to determine risk with the given information according to the formal ELN 2022 criteria.
Feedback:
 * Correct Reasoning: You correctly identified the high WBC as a potentially negative prognostic factor.
 * Guideline Accuracy: The formal ELN 2022 risk assessment requires a more comprehensive evaluation using the established scoring systems (Sokal, Hasford, or ELTS), which necessitate information on spleen size and blast percentage (even if assumed to be low in chronic phase, it's a component of the score).
 * Key Learning Point: It's crucial to use the complete set of parameters defined in the ELN guidelines for accurate risk stratification. While individual factors like very high WBC can be suggestive, they don't solely determine the risk category.

Question 3: The BCR-ABL1/ABL1 ratio on the International Scale (IS) is 95%. According to the ESMO guidelines for CML, what is the typical goal of first-line tyrosine kinase inhibitor (TKI) therapy at 3 months?
a) BCR-ABL1 IS 10%
b) BCR-ABL1 IS 1%
c) Complete cytogenetic response (CCyR), defined as Ph-negative metaphases in bone marrow
d) Major molecular response (MMR), defined as BCR-ABL1 IS 0.1%

That's correct! The typical goal of first-line TKI therapy at 3 months, according to the ESMO guidelines for CML, is a) BCR-ABL1 IS 10%.
Feedback:
 * Guideline Alignment: The ESMO guidelines emphasize the importance of achieving early molecular response as a predictor of long-term outcomes. A BCR-ABL1 IS 10% at 3 months is considered an optimal early response.
 * Rationale: Patients achieving this milestone have a significantly higher likelihood of achieving deeper and more sustained responses, including major molecular response (MMR) and complete molecular response (CMR), which are associated with better progression-free and overall survival.
 * Why the other options are not the primary 3-month goal:
   * b) BCR-ABL1 IS 1%: While achieving 1% is a deeper response and a longer-term goal (often by 6-12 months), it's not the typical target at 3 months.
   * c) Complete cytogenetic response (CCyR): CCyR (0% Ph+ metaphases in bone marrow) is an important milestone, but molecular response (BCR-ABL1 levels) is a more sensitive and earlier indicator of treatment efficacy. The 3-month target focuses on molecular reduction.
   * d) Major molecular response (MMR), defined as BCR-ABL1 IS 0.1%: MMR is an even deeper response and a crucial long-term goal, typically aimed for by 12-18 months or later, rather than the initial 3-month target.

Phase 2: Induction Therapy Selection and Supportive Care Planning
Based on the diagnosis of CML in chronic phase and the initial BCR-ABL1 IS of 95%, the decision is made to initiate first-line tyrosine kinase inhibitor (TKI) therapy. The patient is 58 years old, has a good performance status (ECOG 0), and his comorbidities include well-controlled hypertension managed with a low-dose ACE inhibitor. His baseline creatinine clearance is 85 mL/min.
Question 4: Which of the following is the generally recommended first-line TKI for this patient according to current ESMO guidelines?
a) Imatinib
b) Dasatinib
c) Nilotinib

d) Bosutinib

That's correct! b) Dasatinib is a generally recommended first-line TKI according to current ESMO guidelines for this patient.
Feedback:
 * Guideline Alignment: ESMO guidelines, similar to ELN, often recommend second-generation TKIs like dasatinib or nilotinib as preferred first-line options over imatinib for most patients with chronic phase CML. This is based on evidence from clinical trials demonstrating higher rates of rapid and deep molecular responses, which are associated with better long-term outcomes.
 * Rationale: Dasatinib has shown superior efficacy in achieving early molecular response compared to imatinib in newly diagnosed patients.
 * Considerations for other options:
   * a) Imatinib: While effective, it generally leads to slower and lower rates of deep molecular response compared to second-generation TKIs. It might be considered in specific situations (e.g., low-risk disease, significant comorbidities precluding second-generation TKIs, patient preference).
   * c) Nilotinib: Nilotinib is also a recommended first-line option with high efficacy. However, it has specific warnings regarding cardiovascular risk (e.g., QTc prolongation, arterial occlusive events), which would need careful consideration, although the patient's hypertension is well-controlled.
   * d) Bosutinib: Bosutinib is another second-generation TKI approved for first-line treatment. Its side effect profile, particularly gastrointestinal issues, might influence its selection in some patients.

Question 5: The patient is started on dasatinib. What is a common early hematologic toxicity that requires monitoring during the initial phase of TKI therapy?
a) Hyperleukocytosis
b) Thrombocytosis
c) Neutropenia
d) Lymphocytosis

That is correct! c) Neutropenia is a common early hematologic toxicity that requires monitoring during the initial phase of TKI therapy, including dasatinib.
Feedback:
 * Clinical Relevance: TKIs, while targeting the BCR-ABL1 protein, can also have off-target effects on normal hematopoietic cells in the bone marrow, leading to cytopenias. Neutropenia (low neutrophil count) is frequently observed, especially in the first few months of treatment.
 * Monitoring Importance: Regular monitoring of complete blood counts (CBC) with differential is crucial to detect and manage TKI-related hematologic toxicities. Dose adjustments or temporary interruptions may be necessary based on the severity of neutropenia.
 * Why the other options are less likely as a common early toxicity:
   * a) Hyperleukocytosis: This is a characteristic feature of untreated CML at diagnosis, not a toxicity of TKI therapy. The goal of TKI therapy is to reduce the WBC count.
   * b) Thrombocytosis: While platelet counts can fluctuate in CML, a sustained increase (thrombocytosis) is not a typical early toxicity of TKI therapy. Some TKIs might even cause thrombocytopenia.
   * d) Lymphocytosis: CML is a myeloproliferative neoplasm affecting myeloid lineage cells. Lymphocytosis is not a common toxicity associated with TKI therapy.

Question 6: The patient reports experiencing mild nausea and fatigue since starting dasatinib. What is an appropriate initial recommendation for managing these Grade 1 toxicities?
a) Immediately discontinue dasatinib.
b) Reduce the dose of dasatinib by 50%.
c) Provide supportive care measures and continue dasatinib at the same dose with close monitoring.
d) Switch to an alternative TKI.

That's correct! c) Provide supportive care measures and continue dasatinib at the same dose with close monitoring is the appropriate initial recommendation for managing these Grade 1 toxicities.
Feedback:
 * Toxicity Grading: Grade 1 toxicities are defined as mild and usually do not interfere with daily activities. The ESMO guidelines

recommend a conservative approach for Grade 1 and sometimes Grade 2 non-hematologic toxicities.
 * Supportive Care: For mild nausea, dietary modifications (e.g., small, frequent meals, avoiding greasy foods), antiemetics (if needed), and ensuring adequate hydration can be helpful. Fatigue can be addressed with rest, hydration, and addressing any underlying contributing factors.
 * Monitoring: Close monitoring is essential to ensure the toxicities do not worsen to Grade 2 or higher. If the symptoms persist or progress, further intervention, such as dose adjustment or switching TKIs, might be considered.
 * Why other options are not the initial approach:
   * a) Immediately discontinue dasatinib: This is generally reserved for severe (Grade 3/4) or intolerable toxicities.
   * b) Reduce the dose of dasatinib by 50%: Dose reductions are typically considered for Grade 2 or higher toxicities that do not respond to supportive care.
   * d) Switch to an alternative TKI: Switching TKIs is usually reserved for significant intolerance or resistance to the current therapy.

Question 7: The patient achieves a BCR-ABL1 IS of 8% at 3 months. According to the ESMO guidelines, what is the next step in management regarding TKI therapy?
a) Continue dasatinib at the current dose and monitor BCR-ABL1 levels every 3 months.
b) Increase the dose of dasatinib to achieve a deeper molecular response more quickly.
c) Switch to a different second-generation TKI to improve the likelihood of achieving MMR.
d) Add interferon-alpha to dasatinib to enhance the molecular response.
That's correct! a) Continue dasatinib at the current dose and monitor BCR-ABL1 levels every 3 months is the appropriate next step in management according to the ESMO guidelines.
Feedback:
 * Response Assessment: Achieving a BCR-ABL1 IS 10% at 3 months is considered an optimal early molecular response according to ESMO guidelines. Patients achieving this milestone have a good prognosis.
 * Monitoring: In patients with an optimal early response, continued monitoring of BCR-ABL1 levels at regular intervals (typically every 3 months initially) is recommended to track the depth of response and detect any potential loss of response.
 * Why other options are not indicated at this stage:
   * b) Increase the dose of dasatinib: Dose escalation is generally considered for patients who do not achieve the target molecular response at specific time points (e.g., >10% at 3 months, >1% at 6 months). Since the patient has achieved the 3-month target, dose increase is not routinely recommended.
   * c) Switch to a different second-generation TKI: Switching TKIs in the first-line setting is usually reserved for cases of primary resistance (failure to achieve the early molecular response targets) or significant intolerance to the initial TKI.
   * d) Add interferon-alpha to dasatinib: Interferon-alpha is not a standard component of first-line TKI therapy in chronic phase CML. Its use is limited to specific situations and is not routinely added to enhance response in patients achieving optimal early molecular response with a TKI.

Phase 3: Post-Remission Therapy, MRD Monitoring, and Long-Term Management
The patient continues on dasatinib. At 12 months, his BCR-ABL1 IS is 0.08%.
Question 8: According to the ESMO guidelines, what molecular response level has this patient achieved at 12 months?
a) Major molecular response (MMR)
b) Deep molecular response (DMR) with a 4-log reduction (MR4)
c) Deep molecular response (DMR) with a 4.5-log reduction (MR4.5)
d) Deep molecular response (DMR) with a 5-log reduction (MR5)

That's correct! a) Major molecular response (MMR).
Feedback:
 * MMR Definition: According to the ESMO guidelines and the International Scale (IS), a major molecular response (MMR) is defined as a BCR-ABL1 IS 0.1% (or a 3-log reduction from baseline). The patient's BCR-ABL1 IS of 0.08% at 12 months meets this criterion.
 * Significance of MMR: Achieving MMR is a crucial milestone in CML management. It is associated with a significantly reduced risk of disease progression and improved long-term survival.
 * Deep Molecular Response (DMR): DMR refers to even deeper reductions in BCR-ABL1 levels:
   * MR4: BCR-ABL1 IS 0.01% (4-log reduction)
   * MR4.5: BCR-ABL1 IS 0.0032% (4.5-log reduction)

* MR5: BCR-ABL1 IS 0.001% (5-log reduction)

While the patient has achieved a level within the range of DMR (specifically MR4), the general classification at 0.1% is MMR. DMR is often considered as a target for potential treatment-free remission (TFR) in eligible patients with sustained deep responses.

Question 9: The patient continues on dasatinib and maintains a stable MMR for 3 years. He expresses interest in potentially discontinuing TKI therapy. According to the ESMO guidelines, which of the following is a prerequisite for considering treatment-free remission (TFR)?
a) Achievement of complete cytogenetic response (CCyR) for at least 2 years.
b) Achievement and sustained deep molecular response (DMR), typically MR4.5 or deeper, for at least 2 years.
c) Age younger than 60 years and no significant comorbidities.
d) Duration of TKI therapy for at least 3 years, regardless of the depth of molecular response.

That's correct! b) Achievement and sustained deep molecular response (DMR), typically MR4.5 or deeper, for at least 2 years is a key prerequisite for considering treatment-free remission (TFR) according to the ESMO guidelines.
Feedback:
* Guideline Emphasis: The ESMO guidelines, along with other international recommendations like the ELN, highlight the importance of a sustained deep molecular response (at least MR4, often MR4.5 or deeper) for a minimum duration (typically 2 years) as the primary criterion for attempting TFR. This is based on data showing a lower risk of molecular relapse in patients who meet these stringent criteria.
* Rationale: Sustained deep molecular response indicates a very low burden of residual disease, increasing the likelihood of the immune system being able to control the disease after TKI discontinuation.
* Why other options are not the primary prerequisite:
  * a) Achievement of complete cytogenetic response (CCyR) for at least 2 years: While CCyR is an important landmark, the depth of molecular response is a more sensitive predictor of successful TFR. Patients can achieve DMR even without a prolonged CCyR.
  * c) Age younger than 60 years and no significant comorbidities: These are often considered as favorable factors that might increase the likelihood of successful TFR and reduce the risk of complications upon relapse, but they are not the primary molecular prerequisites.
  * d) Duration of TKI therapy for at least 3 years, regardless of the depth of molecular response: While a certain duration of TKI therapy is usually required to achieve and sustain DMR, the depth of response is the critical factor. Simply being on a TKI for 3 years without achieving a deep molecular response is not sufficient for considering TFR.

Question 10: The patient meets the criteria for attempting TFR and successfully discontinues dasatinib under close monitoring. Six months later, his BCR-ABL1 IS increases to 0.15%. According to the ESMO guidelines, what is the recommended course of action?
a) Continue close monitoring as this is still below the MMR threshold.
b) Re-initiate dasatinib at the original dose.
c) Start a different TKI at the standard first-line dose.
d) Consider allogeneic hematopoietic stem cell transplantation.

That's correct! b) Re-initiate dasatinib at the original dose is the recommended course of action according to the ESMO guidelines in this scenario.
Feedback:
* Management of Molecular Relapse After TFR: The ESMO guidelines recommend prompt re-initiation of TKI therapy at the previously effective dose upon confirmed molecular relapse (typically defined as loss of MMR or a significant increase in BCR-ABL1 levels) after attempting TFR.
* Rationale: The majority of patients who relapse after TFR will regain molecular control with the re-introduction of the TKI that was initially effective. Starting the same TKI at the original dose has the highest likelihood of success.
* Why other options are less appropriate as the initial step:
  * a) Continue close monitoring as this is still below the MMR threshold: While close monitoring is crucial during the TFR attempt, an increase in BCR-ABL1 IS to 0.15% after achieving MMR and discontinuing therapy indicates molecular relapse and warrants intervention, even if it's just slightly above the MMR threshold (0.1%). The goal of TFR is to remain in remission off therapy.
  * c) Start a different TKI at the standard first-line dose: Switching to a different TKI might be considered if the patient fails to respond to the re-initiation of the original TKI or if there was intolerance to the initial TKI. However, re-challenging with the original TKI is the standard first step.
  * d) Consider allogeneic hematopoietic stem cell transplantation: Transplant is generally reserved for patients with advanced phase

CML or those who fail multiple lines of TKI therapy. It is not the standard approach for a molecular relapse after a successful TFR attempt where the patient was previously responsive to a TKI.

Session ID:
Assessment Domain

Session-64:
Rationale

Session ID:
Instructional Design and Pedagogical

Session-64:
The session's instructional design is of very high quality. The case is expertly scaffolded, moving logically from initial diagnosis and risk assessment to first-line therapy selection, management of common toxicities, and long-term monitoring, including the complex topic of treatment-free remission (TFR). The feedback for each question is insightful, referencing current ESMO guidelines and providing clear rationales for both the correct answer and the incorrect distractors. Instructions are exceptionally clear. The only minor area for improvement is the lack of explicitly stated, measurable learning objectives at the beginning of the session, though the educational goals are strongly implied and consistently met by the content.

Session ID:
Clinical Authenticity and Content Fidelity

Session-64:
The clinical fidelity of this session is excellent and represents a gold standard for this type of simulation. Plausibility & Guideline Alignment: The clinical narrative is indistinguishable from an authentic patient case of Chronic Myeloid Leukemia (CML). All diagnostic criteria, treatment milestones (e.g., BCR-ABL1 IS 10% at 3 months), and management decisions are perfectly aligned with current, evidence-based guidelines from ESMO. Pharmacotherapeutic Accuracy: The therapeutic recommendations are optimal and well-justified. The choice of a second-generation TKI, the management of Grade 1 toxicity, and the handling of molecular relapse after TFR are all correct and reflect best practices.
Domain Specificity: The session demonstrates flawless domain specificity. All concepts, diagnostics (Philadelphia chromosome, BCR-ABL1), and therapies (Tyrosine Kinase Inhibitors) are entirely appropriate and specific to the management of CML. There are no instances of domain entanglement; concepts and standards of care from other hematologic malignancies are not incorrectly applied. The simulation is both educationally sound and clinically safe.

Session ID:
Modeled Clinical Reasoning

Session-64:
The simulation excels at modeling an expert clinical reasoning process. Each stage of the case frames a clear and relevant clinical problem, from diagnosis to long-term strategy. The multiple-choice options provide an excellent range of plausible alternatives, including key distractors that effectively probe the user's understanding of nuanced guidelines. The decision-making process demonstrated throughout the caseprioritizing molecular response, adhering to monitoring schedules, and following the correct algorithm for TFRis sound and logical. The clinical outcomes described are realistic and highly instructive, effectively reinforcing the learning objectives at each decision point.

Session-65

Session ID:
LLM

Session-65:
DeepSeek V2

Session ID:
Session Transcript

Session-65:
Excellent, [REDACTED]]. Let's begin with Phase 1.

Phase 1: Initial Presentation and Diagnostic Workup

Patient: R.M. is a 52-year-old male presenting to his primary care physician with complaints of persistent fatigue, early satiety, and unintentional weight loss (approximately 10 lbs over the past 3 months). He also reports occasional discomfort in the left upper quadrant of his abdomen. He has no significant past medical history and reports no known drug allergies. He is a non-smoker and consumes alcohol socially.

Physical Examination: Vital signs are stable. The physical exam is notable for splenomegaly, with the spleen palpable 5 cm below the left costal margin. No other significant findings.

Initial Laboratory Findings:

CBC:
WBC: 155,000/L (Ref: 4.0-11.0 x 10^3/L)
Differential: Significant left shift with increased myelocytes, metamyelocytes, and bands. Blasts account for 3%. Basophils are increased at 5%.
Hemoglobin: 10.5 g/dL (Ref: 13.5-17.5 g/dL)
Platelets: 550,000/L (Ref: 150-450 x 10^3/L)
Chemistry:
Lactate Dehydrogenase (LDH): Elevated
Uric Acid: Elevated
Further Investigations: R.M. is referred to a hematologist. A bone marrow aspirate and biopsy are performed. Cytogenetic analysis (karyotyping) and molecular testing (RT-PCR) are ordered.

Bone Marrow Findings: Hypercellular marrow (~95% cellularity) with marked granulocytic hyperplasia and increased megakaryocytes. Blasts constitute <5% of nucleated cells.
Cytogenetics: Reveals 46,XY,t(9;22)(q34.1;q11.2) in 20 out of 20 metaphases analyzed.
Molecular Testing (RT-PCR): Detects the presence of the BCR::ABL1 fusion transcript, specifically the p210 isoform.
Question 1: Based on the World Health Organization (WHO) 2022 classification and the patient's initial workup results, what is the most accurate diagnosis for R.M.?

A) Chronic Myeloid Leukemia (CML), BCR::ABL1-positive, Accelerated Phase
B) Acute Myeloid Leukemia (AML) with t(9;22)(q34.1;q11.2); BCR::ABL1
C) Chronic Myeloid Leukemia (CML), BCR::ABL1-positive, Chronic Phase
D) Myelodysplastic/Myeloproliferative Neoplasm (MDS/MPN) with Ring Sideroblasts and Thrombocytosis (MDS/MPN-RS-T)

Okay, [REDACTED]]. Let's break down this choice.

You selected A) Chronic Myeloid Leukemia (CML), BCR::ABL1-positive, Accelerated Phase.

Feedback: That's not the most accurate diagnosis based on the provided information.

Why Option C is Correct: The diagnosis of Chronic Myeloid Leukemia (CML), BCR::ABL1-positive, Chronic Phase (CP) is correct because the patient meets the diagnostic criteria for CML (presence of t(9;22) and BCR::ABL1 fusion gene) and the criteria for Chronic Phase according to the WHO 2022 classification. Specifically, the blast percentage in both peripheral blood (3%) and bone marrow (<5%) is below the 10% threshold required for diagnosing Accelerated Phase (AP). Additionally, peripheral blood basophils are 5%, which is less than the 20% criterion for AP.
Why Option A is Incorrect: Accelerated Phase (AP) CML requires meeting specific criteria, such as blasts 10-19% in blood or marrow, basophils 20% in blood, or other features like persistent thrombocytopenia or thrombocytosis unresponsive to therapy, or clonal evolution. R.M.'s findings do not meet these criteria at presentation.
Why Option B is Incorrect: While AML can present with the t(9;22) translocation, it's defined by 20% blasts in the blood or marrow. R.M.'s blast count is significantly lower. His presentation with marked leukocytosis, splenomegaly, and a full spectrum of myeloid maturation is characteristic of CML rather than de novo AML.
Why Option D is Incorrect: MDS/MPN-RS-T is a different myeloproliferative neoplasm characterized by dysplastic features, ring sideroblasts, and thrombocytosis, typically without the BCR::ABL1 fusion gene.
Key Takeaway: Correctly staging the phase of CML at diagnosis (Chronic, Accelerated, or Blast Crisis) is crucial as it directly impacts prognosis and initial treatment strategy. The blast percentage is a primary determinant.

Let's move on to risk stratification.
Question 2: Now that R.M. is diagnosed with CML, Chronic Phase, we need to assess his risk to guide initial therapy selection. Using the European LeukemiaNet (ELN) 2020 recommendations (or the largely similar Sokal/Hasford scores often incorporated), which of the following risk scores is most commonly used at diagnosis for CML-CP?

A) FLIPI Score
B) IPSS-R Score
C) Sokal Score
D) R-ISS Score
Excellent choice, [REDACTED]]!

You selected C) Sokal Score.

Feedback: That is correct.

Why Option C is Correct: The Sokal score is one of the established prognostic scoring systems specifically designed for Chronic Myeloid Leukemia in Chronic Phase (CML-CP) at the time of diagnosis. It incorporates factors like patient age, spleen size below the costal margin, peripheral blood blast percentage, and platelet count to categorize patients into low, intermediate, or high-risk groups. This helps predict long-term outcomes. While newer scores like the EUTOS Long-Term Survival (ELTS) score are now also recommended by ELN 2020 and may better predict outcomes in the TKI era, the Sokal (and Hasford/Euro) score remains a widely used and historically important tool calculated at diagnosis.
Why Option A is Incorrect: The FLIPI score (Follicular Lymphoma International Prognostic Index) is used for risk stratification in Follicular Lymphoma.
Why Option B is Incorrect: The IPSS-R score (Revised International Prognostic Scoring System) is used for risk stratification in Myelodysplastic Syndromes (MDS).
Why Option D is Incorrect: The R-ISS score (Revised International Staging System) is used for risk stratification in Multiple Myeloma.
Key Takeaway: Selecting the appropriate disease-specific prognostic tool is fundamental in oncology. For CML-CP at diagnosis, Sokal, Hasford (Euro), and ELTS are the relevant scores, with ELTS currently favored by ELN for predicting TKI outcomes.

Now, let's calculate R.M.'s Sokal score. We need:

Age: 52 years
Spleen Size: 5 cm below costal margin
Platelets: 550 x 10^9/L
Peripheral Blasts: 3%

The Sokal score calculation is complex: exp[0.0116 (age 43.4) + 0.0345 (spleen size 7.51) + 0.188 ((platelets/700)^2 0.563) + 0.0887 (% blasts 2.10)]

Plugging in R.M.'s values results in a Sokal Score of approximately 0.95.

Low Risk: < 0.8
Intermediate Risk: 0.8 - 1.2
High Risk: > 1.2
R.M. falls into the Intermediate Risk category based on the Sokal score.
Question 3: Based on R.M.'s diagnosis of BCR::ABL1-positive CML-CP, Intermediate Sokal Risk, and considering the ELN 2020 and ESMO guidelines, which of the following is the most appropriate initial treatment goal?

A) Achieve complete cytogenetic response (CCyR) within 3 months.
B) Achieve major molecular response (MMR; BCR::ABL1 0.1% IS) within 12 months.
C) Achieve undetectable BCR::ABL1 transcripts within 6 months.
D) Reduce spleen size by 50% within 3 months.
16.7s

Excellent choice, [REDACTED]]!

You selected B) Achieve major molecular response (MMR; BCR::ABL1 0.1% IS) within 12 months.

Feedback: This is the correct answer, representing a crucial milestone for optimal response according to ELN 2020 and ESMO guidelines.

Why Option B is Correct: Achieving MMR (BCR::ABL1 transcript levels 0.1% on the International Scale or IS) by 12 months is defined as an "optimal response" by the ELN 2020 guidelines. Reaching this milestone is strongly associated with a lower risk of disease progression and excellent long-term survival. It is a primary objective of first-line TKI therapy.
Why Option A is Incorrect: While achieving CCyR (0% Ph+ metaphases by standard cytogenetics) is an important earlier goal, the expected timeframe for optimal response is typically by 6-12 months, not necessarily within 3 months. Furthermore, the BCR::ABL1 10% IS milestone at 3 months (Early Molecular Response - EMR) is considered a more critical early checkpoint than CCyR at 3 months.
Why Option C is Incorrect: Achieving undetectable BCR::ABL1 (a deep molecular response like MR4 or MR4.5) is a desirable long-term goal, especially for patients considering treatment discontinuation, but it is not the standard expected outcome within the first 6 months of therapy.
Why Option D is Incorrect: Reduction in spleen size and normalization of blood counts constitute a complete hematologic response (CHR), which is expected relatively early (usually within 3 months). While important for symptom control and indicating initial drug activity, it is not the primary long-term goal that correlates most strongly with preventing disease progression. Molecular response milestones are more critical for long-term outcomes.
Key Takeaway: Treatment goals in CML are defined by specific cytogenetic and molecular response milestones achieved within defined timeframes. MMR by 12 months is a critical benchmark for optimal response to first-line therapy according to current guidelines (ELN 2020, ESMO).

This concludes Phase 1. We have diagnosed R.M. with CML-CP, Intermediate Sokal Risk, and established the primary treatment goal.

Phase 2: Induction Therapy Selection and Supportive Care Planning

Now that we have established the diagnosis and risk stratification, we need to select the initial therapy for R.M. He is 52 years old, otherwise healthy, with Intermediate Risk CML-CP.

Question 4: Considering R.M.'s profile (52 years old, Intermediate Sokal Risk CML-CP, no significant comorbidities) and the available first-line options approved by regulatory agencies and recommended by ELN/ESMO guidelines, which of the following Tyrosine Kinase Inhibitors (TKIs) would be an appropriate choice?

A) Ponatinib
B) Imatinib
C) Bosutinib
D) Asciminib
Okay, [REDACTED]]. You selected B) Imatinib.

Feedback: That is an appropriate choice for first-line therapy.

Why Option B is Correct: Imatinib (typically 400 mg daily) is a first-generation TKI and remains a standard, guideline-recommended first-line treatment option for newly diagnosed CML-CP across all risk groups (ELN 2020, ESMO). It has extensive long-term efficacy and safety data.
Why Option C is Also Potentially Correct (and important context): Bosutinib (a second-generation TKI) is also approved and recommended as a first-line option. Guidelines often suggest that second-generation TKIs (Nilotinib, Dasatinib, Bosutinib) may be considered, particularly for intermediate or high-risk patients like R.M., as they can induce faster and deeper molecular responses compared to imatinib. However, imatinib remains a perfectly valid and widely used initial choice. The selection between imatinib and a second-generation TKI often involves considering the specific TKI's side effect profile, patient comorbidities (though R.M. has none stated), patient preference, and treatment goals (e.g., potential future treatment-free remission). Since the question asks for an appropriate choice, Imatinib fits.
Why Option A is Incorrect: Ponatinib is a third-generation TKI typically reserved for patients with the T315I mutation or those who have failed multiple prior TKIs. It carries a significant risk of vaso-occlusive events and is not used as standard first-line therapy.
Why Option D is Incorrect: Asciminib is a novel STAMP inhibitor (Specifically Targeting the ABL Myristoyl Pocket) approved for later lines of therapy or for patients with the T315I mutation. It is not currently approved or recommended as a standard initial treatment for newly diagnosed CML-CP.
Key Takeaway: Standard first-line therapy for CML-CP includes Imatinib, Dasatinib, Nilotinib, and Bosutinib. The choice among these depends on risk stratification, patient comorbidities, toxicity profiles, and treatment goals. Ponatinib and Asciminib are reserved for specific later-line or resistance scenarios.

Let's assume the decision is made to start R.M. on Imatinib 400 mg once daily.

Question 5: While R.M. is starting Imatinib, proactive management of potential side effects and baseline monitoring are crucial. Which of the following baseline assessments is LEAST critical specifically for initiating Imatinib therapy?

A) Liver function tests (AST, ALT, Bilirubin)
B) Serum creatinine and electrolytes (including phosphate, magnesium, potassium)
C) Electrocardiogram (ECG)
D) Hepatitis B Virus (HBV) serology (HBsAg, anti-HBc)
17.0s

Okay, [REDACTED]]. You selected C) Electrocardiogram (ECG).

Feedback: That is correct. This assessment is generally considered the LEAST critical specifically due to Imatinib initiation compared to the others listed.

Why Option C is Correct: While a baseline ECG is often part of a general medical workup, especially for middle-aged adults starting long-term therapy, Imatinib itself is not strongly associated with significant QTc prolongation, unlike second-generation TKIs such as Nilotinib and Dasatinib, where baseline and follow-up ECGs are crucial. While rare cardiac events (like heart failure) have been reported, routine baseline ECG is not considered as mandatory specifically for Imatinib initiation as the other tests listed.
Why Option A is Incorrect: Liver function tests (LFTs) are absolutely critical. Imatinib is metabolized by the liver and can cause hepatotoxicity, ranging from mild LFT elevations to severe liver injury. Baseline LFTs and regular monitoring are essential (ELN 2020, Imatinib prescribing information).
Why Option B is Incorrect: Baseline renal function (serum creatinine) and electrolytes (especially phosphate, magnesium, potassium) are critical. Imatinib can cause fluid retention, electrolyte abnormalities (notably hypophosphatemia), and requires dose adjustment in severe renal impairment (though R.M. has no known kidney issues yet). Monitoring is necessary.

Why Option D is Incorrect: Hepatitis B Virus (HBV) screening (HBsAg, anti-HBc, +/- anti-HBs) is considered standard practice before initiating TKI therapy or other potentially immunosuppressive treatments. HBV reactivation can occur during CML therapy and can lead to severe hepatitis or liver failure. Guidelines (e.g., ASCO) recommend screening.
Key Takeaway: Prioritizing baseline assessments should focus on the known and significant risks associated with the specific drug being initiated. For Imatinib, hepatotoxicity, fluid/electrolyte issues, and general risks like HBV reactivation are key concerns, while significant ECG changes are less characteristic compared to some other TKIs.

R.M. starts Imatinib 400 mg daily. Three months into therapy, his labs are drawn for monitoring.

Monitoring Results at 3 Months:

CBC:
WBC: 8.5 x 10^3/L (within normal limits)
Hemoglobin: 12.8 g/dL (improved)
Platelets: 250 x 10^3/L (normalized)
Differential: No left shift, Blasts 0%, Basophils 1%
Result: Complete Hematologic Response (CHR) achieved.
Cytogenetics (Bone Marrow): 2 out of 20 metaphases are Ph+ (10% Ph+)
Result: Partial Cytogenetic Response (PCyR) achieved.
Molecular Testing (RT-PCR, Blood): BCR::ABL1 transcript level is 5% (IS)

Question 6: According to the ELN 2020 response criteria, how should R.M.'s response at 3 months (BCR::ABL1 = 5% IS) be classified?

A) Optimal Response
B) Warning
C) Failure
D) Not Assessable
19.3s

Okay, [REDACTED]]. You selected A) Optimal Response.

Feedback: That is correct!

Why Option A is Correct: According to the ELN 2020 guidelines, achieving a BCR::ABL1 transcript level of 10% on the International Scale (IS) at 3 months is defined as an Optimal Response. R.M.'s result of 5% IS clearly meets this criterion. Achieving this early molecular response (EMR) is associated with a higher probability of achieving subsequent deeper responses and better long-term outcomes. He has also achieved CHR, further supporting the good initial response.
Why Option B is Incorrect: The "Warning" category at 3 months applies if the BCR::ABL1 transcript level is > 10% IS. R.M.'s level is below this threshold.
Why Option C is Incorrect: "Failure" at 3 months according to ELN 2020 is generally defined by not achieving CHR, or having persistent high Ph+ percentage if cytogenetics were done very early, but primarily focuses on the >10% IS molecular marker as being suboptimal. R.M. has achieved CHR and has a BCR::ABL1 10% IS.
Why Option D is Incorrect: The response is clearly assessable using the standard RT-PCR method.
Key Takeaway: The 3-month BCR::ABL1 10% IS milestone is a critical early checkpoint in CML management. Patients achieving this goal have a significantly better prognosis. R.M. is currently on track.

R.M. continues Imatinib 400 mg daily. Six months into therapy, he reports mild periorbital edema and occasional muscle cramps but is otherwise tolerating treatment well. Monitoring labs are obtained.

Monitoring Results at 6 Months:

CBC: Remains normal (CHR maintained).
Cytogenetics (Bone Marrow): 0 out of 20 metaphases are Ph+ (0% Ph+)

Result: Complete Cytogenetic Response (CCyR) achieved.
Molecular Testing (RT-PCR, Blood): BCR::ABL1 transcript level is 0.5% (IS)
Question 7: Based on the ELN 2020 criteria, how should R.M.'s response at 6 months (BCR::ABL1 = 0.5% IS and CCyR achieved) be classified?

A) Optimal Response
B) Warning
C) Failure
D) Suboptimal Response (historical term, evaluate based on ELN 2020 categories)
11.0s

Correct again, [REDACTED]]!

You selected A) Optimal Response.

Feedback: This is the correct classification based on the ELN 2020 guidelines.

Why Option A is Correct: The ELN 2020 criteria define Optimal Response at 6 months as achieving a BCR::ABL1 transcript level of 1% IS. R.M.'s result of 0.5% IS comfortably meets this target. He has also achieved CCyR, which is another component of an optimal response by this time point (BCR::ABL1 1% IS or CCyR).
Why Option B is Incorrect: The "Warning" category at 6 months applies if BCR::ABL1 is > 1% IS but 10% IS. R.M.'s level is below this range.
Why Option C is Incorrect: "Failure" at 6 months is defined as BCR::ABL1 > 10% IS or not achieving at least a PCyR (35% Ph+). R.M. has achieved CCyR and has a low BCR::ABL1 level.
Why Option D is Incorrect: While "Suboptimal Response" was used in older guidelines, the current ELN 2020 framework uses Optimal, Warning, and Failure. R.M.'s response clearly falls into the Optimal category under the current system.
Key Takeaway: Monitoring milestones at 3, 6, and 12 months are crucial for assessing TKI efficacy. Achieving BCR::ABL1 1% IS by 6 months is another indicator of an excellent treatment trajectory. R.M. continues to respond optimally to Imatinib.

This concludes Phase 2. R.M. has achieved optimal responses at both 3 and 6 months on Imatinib therapy.

Phase 3: Post-Remission Therapy, MRD Monitoring, and Long-Term Management

R.M. reaches the 12-month mark on Imatinib 400 mg daily. He continues to tolerate it well, with only mild, manageable side effects (grade 1 edema, occasional grade 1 muscle cramps).

Monitoring Results at 12 Months:

CBC: Remains normal (CHR maintained).
Molecular Testing (RT-PCR, Blood): BCR::ABL1 transcript level is 0.05% (IS)
Question 8: According to the ELN 2020 criteria, how is R.M.'s molecular response (BCR::ABL1 = 0.05% IS) at 12 months classified?

A) Complete Cytogenetic Response (CCyR)
B) Early Molecular Response (EMR)
C) Major Molecular Response (MMR)
D) Deep Molecular Response (DMR) - MR4
Excellent, [REDACTED]]!

You selected C) Major Molecular Response (MMR).

Feedback: That is the correct classification for R.M.'s response at 12 months.

Why Option C is Correct: Major Molecular Response (MMR) is defined as a BCR::ABL1 transcript level 0.1% on the International

Scale (IS), or a 3-log reduction from the standardized baseline. R.M.'s result of 0.05% IS meets this criterion (BCR::ABL1 0.1% IS). Achieving MMR by 12 months is considered an optimal response and is a primary goal of therapy associated with excellent long-term outcomes (ELN 2020, ESMO).
Why Option A is Incorrect: CCyR is a cytogenetic measure (0% Ph+ cells) or approximately equivalent to BCR::ABL1 1% IS. While R.M. likely maintains CCyR (as MMR is a deeper response), MMR is the more precise and prognostically significant molecular classification at this level.
Why Option B is Incorrect: EMR refers specifically to the BCR::ABL1 10% IS milestone at 3 months. It's not used to classify response at 12 months.
Why Option D is Incorrect: Deep Molecular Response (DMR) refers to responses deeper than MMR. MR4 (a 4-log reduction) is defined as BCR::ABL1 0.01% IS. R.M.'s result of 0.05% IS is not deep enough to qualify as MR4.
Key Takeaway: MMR (BCR::ABL1 0.1% IS) is a critical therapeutic goal in CML, ideally achieved by 12 months. Monitoring continues beyond this point to assess the durability and depth of response.

R.M. continues on Imatinib 400 mg daily and maintains MMR for the next two years (Year 2 and Year 3 post-diagnosis). At his Year 4 check-up, his BCR::ABL1 level has risen slightly.

Monitoring Results at 4 Years:

CBC: Remains normal.
Molecular Testing (RT-PCR, Blood): BCR::ABL1 transcript level is 0.3% (IS). This result is confirmed on a repeat test one month later.

Question 9: R.M. has experienced a confirmed loss of MMR (BCR::ABL1 > 0.1% IS), with levels now at 0.3% IS, after having previously maintained MMR. According to ELN 2020 guidelines, what is the most appropriate next step?

A) Immediately switch to Ponatinib.
B) Perform BCR::ABL1 kinase domain mutation analysis.
C) Increase the Imatinib dose to 600 mg daily.
D) Reassure the patient and continue monitoring every 3 months.
Okay, [REDACTED]]. You selected A) Immediately switch to Ponatinib.

Feedback: This is not the recommended initial step in this situation.

Why Option A is Incorrect: Ponatinib is a potent TKI primarily indicated for patients with the T315I mutation or those who have failed multiple previous TKIs. Switching directly to Ponatinib without investigating the reason for the loss of MMR is not aligned with guidelines (ELN 2020). It also carries a higher risk of serious side effects (e.g., vaso-occlusive events) compared to other TKIs.
Why Option B is Correct: The most appropriate next step when MMR is lost is to investigate the underlying cause. The development of BCR::ABL1 kinase domain mutations is a common mechanism of resistance to Imatinib. Performing mutation analysis is crucial because the presence and type of mutation will directly influence the choice of the next TKI. ELN 2020 guidelines explicitly recommend mutation analysis in case of loss of MMR.
Why Option C is Incorrect: While increasing the Imatinib dose might be considered in some cases (e.g., if non-adherence is suspected and ruled out, or if no mutation is detected), performing mutation analysis first is essential to guide this decision. If a mutation conferring resistance to Imatinib is present, dose escalation is unlikely to be effective.
Why Option D is Incorrect: Loss of MMR is a significant event ("Warning" according to ELN 2020) and requires action, not just continued observation. Waiting could allow the disease to progress further.
Key Takeaway: Loss of a previously achieved response milestone (like MMR) necessitates investigation before altering therapy. BCR::ABL1 kinase domain mutation analysis is the standard first step to identify acquired resistance and guide subsequent treatment selection according to ELN 2020 guidelines.

Let's assume the BCR::ABL1 kinase domain mutation analysis is performed, and it reveals no detectable mutation. Adherence to Imatinib is confirmed to be excellent. R.M. is now 4.5 years post-diagnosis. His BCR::ABL1 level remains slightly elevated at 0.25% IS (still loss of MMR, but stable compared to 0.3% six months prior). He has Intermediate Sokal risk at diagnosis and no significant comorbidities.

Question 10: Given the confirmed loss of MMR (BCR::ABL1 > 0.1% IS) without detectable kinase domain mutations, and confirmed adherence to Imatinib 400 mg daily, what is the most appropriate therapeutic intervention for R.M. according to ELN 2020 / ESMO guidelines?

A) Continue Imatinib 400 mg daily and monitor closely every 3 months.
B) Switch to a second-generation TKI (e.g., Nilotinib, Dasatinib, or Bosutinib).
C) Switch directly to Asciminib.
D) Recommend allogeneic stem cell transplantation (allo-SCT).
17.2s

Okay, [REDACTED]]. You selected A) Continue Imatinib 400 mg daily and monitor closely every 3 months.

Feedback: This is generally not the recommended approach for a confirmed and persistent loss of MMR.

Why Option A is Incorrect: While R.M.'s BCR::ABL1 level is still relatively low (<1% IS), the confirmed loss of MMR represents a "Warning" signal according to ELN 2020 criteria. Continuing the same therapy without change carries a risk of further loss of response or potential progression, especially since the loss is persistent. Guidelines typically recommend intervention in this scenario after ruling out non-adherence and mutations.
Why Option B is Correct: Switching to a second-generation TKI (Nilotinib, Dasatinib, or Bosutinib) is the standard recommendation for patients experiencing loss of MMR on Imatinib in the absence of kinase domain mutations and with confirmed adherence (ELN 2020, ESMO). These agents are more potent and offer a high chance of recapturing MMR and achieving deeper molecular responses. The specific choice would depend on factors like potential drug interactions and managing side effect profiles, but changing the mechanism or potency of BCR::ABL1 inhibition is warranted.
Why Option C is Incorrect: Asciminib is approved for CML-CP patients previously treated with two or more TKIs, or for those with the T315I mutation. It is not typically used as a second-line treatment immediately following Imatinib in the absence of mutations.
Why Option D is Incorrect: Allogeneic stem cell transplantation (allo-SCT) is a high-risk procedure reserved for patients with advanced phase disease (Accelerated Phase or Blast Crisis) or those who have failed multiple TKI therapies and are at high risk of progression. It is definitely not indicated for a first "Warning" signal like loss of MMR in Chronic Phase.
Key Takeaway: A confirmed loss of MMR on first-line Imatinib, even without detectable mutations, is a sign that the current therapy may no longer be providing optimal disease control. Switching to a more potent second-generation TKI is the standard approach recommended by guidelines to improve the depth of response and long-term outcome.

This concludes the final question of the case. Would you like to END the session to receive a summary and feedback? Or do you have any questions about this last point?
16.6s

Session ID:
Assessment Domain

Session-65:
Rationale

Session ID:
Instructional Design and Pedagogical

Session-65:
The session's instructional design is outstanding. The case is expertly scaffolded, progressing logically from initial diagnosis and risk stratification through first-line therapy selection, response monitoring at key 3-, 6-, and 12-month milestones, and finally, the management of a later loss of response. The quality of the embedded feedback is a major strength; each rationale is detailed, correctly cites current clinical guidelines (ELN 2020, ESMO), explains why incorrect options are wrong, and provides a "Key Takeaway" that reinforces the core learning point. Instructions are exceptionally clear. The only minor weakness is that learning objectives are not explicitly stated at the outset, though they are strongly implied by the session's coherent structure.

Session ID:

Clinical Authenticity and Content Fidelity

Session-65:

The clinical fidelity of this CML simulation is exceptional, containing no discernible content errors. Plausibility & Guideline Alignment: The clinical narrative is highly plausible, mirroring a classic presentation and treatment course for Chronic Myeloid Leukemia, Chronic Phase (CML-CP). All therapeutic decisions, monitoring timepoints (3, 6, 12 months), and response criteria (e.g., BCR::ABL1 10% IS at 3 months, MMR at 12 months) are perfectly aligned with current, evidence-based guidelines, specifically the ELN 2020 recommendations which are cited throughout. Pharmacotherapeutic Accuracy: The pharmacotherapeutic choices and rationales are optimal and guideline-directed. The session correctly positions Imatinib as a standard first-line agent, appropriately discusses later-generation TKIs, and accurately reserves agents like Ponatinib and Asciminib for their specific indications. The management of MMR loss (performing mutation analysis before switching therapy) is also perfectly modeled. Domain Specificity: The simulation demonstrates exemplary domain specificity. It is entirely free of domain entanglement. In fact, it actively reinforces correct domain application by including distractors from other hematologic malignancies (e.g., FLIPI score for lymphoma, IPSS-R for MDS in Question 2) and providing clear rationales for why they are incorrect.

Session ID:

Modeled Clinical Reasoning

Session-65:

The simulation provides an expert model of clinical reasoning in CML management. Each stage of the case presents a well-defined clinical problem with the necessary data for a decision (e.g., "What is the diagnosis?", "What is the next step after loss of MMR?"). The multiple-choice options offer an excellent breadth of plausible and relevant alternatives, including key distractors that effectively test nuanced understanding. The narrative demonstrates appropriate prioritization at every step, such as establishing a precise diagnosis and risk score before initiating therapy and performing mutation analysis before changing treatment for resistance. The clinical outcomes described are realistic, specific, and instructionally valuable, effectively reinforcing the learning objectives of each phase.

Session-66

Session ID:
LLM

Session-66:
DeepSeek V2

Session ID:
Session Transcript

Session-66:
Phase 1: Initial Presentation & Diagnostic Workup**
A 58-year-old man presents with fatigue, bruising, and WBC 85,000/L (30% blasts on peripheral smear).

Question 1: Which test is *most critical* to confirm AML diagnosis?
- A) Flow cytometry
- B) Bone marrow biopsy
- C) Karyotyping
- D) FLT3 mutation testing
Correct Answer: B) Bone marrow biopsy**
- **Why?** A bone marrow biopsy is the **gold standard** for AML diagnosis. It assesses blast percentage (20% confirms AML), morphology, and provides tissue for ancillary tests (e.g., flow cytometry, cytogenetics).
- **Why not the others?**
  - A) Flow cytometry: Helps immunophenotype blasts but cannot quantify blast percentage alone.
  - C) Karyotyping: Critical for risk stratification but not diagnostic alone.
  - D) FLT3 testing: Guides prognosis/targeted therapy but doesnt establish diagnosis.

Question 2 (Phase 1):** The biopsy shows 50% blasts with **t(8;21)**. What is the *risk category* of this AML?
- A) Favorable
- B) Intermediate
- C) Adverse
- D) Indeterminate
Correct Answer: A) Favorable**
- **Why?** The **t(8;21)** translocation is a *core-binding factor (CBF) AML* subtype, classified as **favorable risk** due to high remission rates with intensive chemotherapy (e.g., 7+3 + high-dose cytarabine consolidation).
- **Why not the others?**
  - B) Intermediate: Includes *normal karyotype* or mutations like *NPM1 without FLT3-ITD*.
  - C) Adverse: Includes *complex karyotype*, *TP53 mutations*, or *monosomal karyotype*.
  - D) Indeterminate: Not a valid risk category in AML.

Phase 2: Induction Therapy & Supportive Care
*The patient is diagnosed with **CBF-AML (t(8;21))**, ECOG 1, no comorbidities.*

Question 3: What is the **preferred induction regimen** for this patient?
- A) 7+3 (cytarabine + daunorubicin)
- B) Venetoclax + azacitidine
- C) Gemtuzumab ozogamicin
Correct Answer: C) Gemtuzumab ozogamicin (GO) + 7+3**
- **Why?** For **CBF-AML (t(8;21))**, adding **gemtuzumab ozogamicin (GO)** to 7+3 significantly improves outcomes (e.g., reduced relapse risk, better EFS) due to CD33 expression in CBF-AML blasts. This is supported by the **ALFA-0701 trial**.
- **Why not the others?**

- A) 7+3 alone: Effective but inferior to GO-combined regimens in CBF-AML.
- B) Venetoclax + azacitidine: Used for *unfit/older patients* (not preferred for fit CBF-AML).
- D) Midostaurin + 7+3: Reserved for **FLT3-mutated AML** (not indicated here).

Question 4 (Phase 2):** During induction, the patient develops *febrile neutropenia*. Which **empiric antibiotic** is *first-line*?
- A) Ciprofloxacin
- B) Meropenem
- C) Vancomycin + piperacillin-tazobactam
- D) Caspofungin
Correct Answer: B) Meropenem**
- **Why?**
  - **Meropenem** (a carbapenem) is a **first-line empiric monotherapy** for febrile neutropenia, providing *broad-spectrum* coverage against *Pseudomonas* and other Gram-negative/Gram-positive organisms.
  - Supported by **IDSA guidelines** for high-risk neutropenic patients (e.g., AML induction).

- **Why not the others?**
  - A) **Ciprofloxacin**: Lacks reliable Gram-positive coverage and is *not monotherapy* for empiric treatment.
  - C) **Vancomycin + piperacillin-tazobactam**: Only add vancomycin if MRSA is suspected (e.g., catheter infection, cellulitis).
  - D) **Caspofungin**: Antifungals are *not initial therapy* unless fever persists after 47 days of antibiotics.
Phase 3: Post-Remission Therapy & Long-Term Management**
*The patient achieves complete remission (CR) after induction. His cytogenetics show **t(8;21)**, and MRD testing by flow cytometry is **negative**.*

Question 5: What is the **best post-remission therapy** for this patient?
- A) High-dose cytarabine (HiDAC) 4 cycles
- B) Autologous stem cell transplant (auto-SCT)
- C) Allogeneic stem cell transplant (allo-SCT)
- D) Oral azacitidine maintenance
Correct Answer: A) High-dose cytarabine (HiDAC) 4 cycles**
- **Why?**
  - **t(8;21) AML is favorable-risk**, and **HiDAC consolidation (34 cycles)** is the standard for these patients, associated with **long-term remission** (6070% cure rates).
  - **MRD-negative status** further supports chemotherapy over transplant.

- **Why not the others?**
  - B) **Auto-SCT**: No role in CBF-AML (used in some intermediate-risk cases).
  - C) **Allo-SCT**: Reserved for *adverse-risk AML* or *MRD-positive CR*.
  - D) **Oral azacitidine**: Used in *maintenance for unfit patients* (not standard in favorable-risk AML).

Question 6 (Phase 3):**
Two years later, the patient remains in remission but develops **pancytopenia** (Hb 8.2 g/dL, platelets 85,000/L, ANC 900/L). What is the *most likely cause*?
- A) Disease relapse
- B) Therapy-related MDS (t-MDS)
- C) Persistent marrow suppression from HiDAC
- D) Autoimmune cytopenias
Correct Answer: B) Therapy-related MDS (t-MDS)**
- **Why?**
  - The patient received **high-dose cytarabine (HiDAC)**, an alkylating-like agent, which increases risk of **therapy-related myeloid neoplasms (t-MDS/AML)**.
  - **Pancytopenia + prior chemo exposure** is classic for t-MDS (typically occurs **25 years post-therapy**).
  - *Key clue*: Late-onset cytopenias in a previously treated AML patient are *not typical* of persistent chemo effects (which resolve sooner).

- **Why not the others?**
  - A) **Relapse**: Unlikely without blasts or MRD positivity.
  - C) **Persistent HiDAC suppression**: Cytopenias from HiDAC resolve within *weeks to months* (not years).
  - D) **Autoimmune cytopenias**: Rare in this context; would typically show other lab features (e.g., positive Coombs test).

Next Steps:
A **bone marrow biopsy with cytogenetics** is needed to confirm t-MDS (likely showing **del(5q), -7, or complex karyotype**).
Bonus Question 1: Supportive Care**
*A 45-year-old woman with AML (FLT3-ITD+) develops **differentiation syndrome** on day 6 of 7+3 + midostaurin. She has fever, hypoxia, and pulmonary infiltrates.*

What is the *immediate intervention*?
- A) Hold midostaurin and start dexamethasone 10 mg IV BID
- B) Initiate broad-spectrum antibiotics alone
- C) Add vancomycin for MRSA coverage
- D) Continue therapy with supplemental O2

Bonus Question 1: Differentiation Syndrome**
Your Answer: A) Hold midostaurin and start dexamethasone 10 mg IV BID
 Correct!
- **Why?** Differentiation syndrome is a **medical emergency** requiring:
  1. **Immediate steroids** (dexamethasone 10 mg IV BID).
  2. **Hold the causative drug** (midostaurin in FLT3+ AML).
- **Supportive care** (O2, diuretics) is adjunctive, but steroids are *definitive*.
- **Pitfall**: Starting antibiotics alone (B/C) delays life-saving steroids.
Bonus Question 2: Pharmacology**
*Which drug requires **ECG monitoring for QTc prolongation** during AML therapy?*
- A) Venetoclax
- B) Cytarabine
- C) Gemtuzumab ozogamicin
- D) Midostaurin
Bonus Question 2: QTc Monitoring**
Your Answer: D) Midostaurin
 Correct!
- **Why?** **Midostaurin** (and other FLT3 inhibitors like gilteritinib) carry a **boxed warning for QTc prolongation**.
  - Baseline + periodic ECGs are required.
- **Why not others?**
  - A) Venetoclax: Risk is **tumor lysis syndrome** (not QTc).
  - B) Cytarabine: Neurotoxicity (cerebellar) is dose-limiting.
  - C) Gemtuzumab: Watch for *infusion reactions* or hepatotoxicity.
Challenge 1: MRD Interpretation**
*A 65-year-old achieves CR after 7+3 for AML with NPM1 mutation. Post-induction, his **NPM1 MRD by PCR** is:
- **Baseline (diagnosis)**: 10,000 copies
- **Post-cycle 1**: 500 copies
- **Post-cycle 2**: 50 copies*

What does this trend indicate?
- A) Impending relapse (switch to salvage therapy)
- B) Adequate response (continue planned consolidation)
- C) Contamination error (repeat testing)
- D) Need for allogeneic transplant
Challenge 1: MRD Interpretation**
Your Answer: B) Adequate response (continue planned consolidation)

Absolutely Correct!
- **Why?** The **log reduction in NPM1 MRD** (10,000 500 50 copies) shows:
  - **Molecular response** (3-log reduction is favorable).
  - **No indication of relapse** (would see rising copies).
- **Action**: Proceed with consolidation (e.g., HiDAC for fit patients).
- **Pitfalls**:
  - A) Relapse: Requires *rising* MRD or clinical signs.
  - D) Transplant: Not needed for NPM1+ AML with good MRD clearance.
Challenge 2: Transplant Eligibility**
*A 50-year-old with **adverse-risk AML (TP53 mutation)** achieves CR1 after venetoclax+azacitidine. Their sibling is an HLA-matched donor. What is the **next step**?*
- A) Continue venetoclax+azacitidine indefinitely
- B) Proceed to allogeneic HSCT now
- C) Give 2 more cycles of venetoclax+azacitidine
- D) Autologous HSCT
Challenge 2: Transplant Eligibility**
Your Answer: B) Proceed to allogeneic HSCT now
Spot On!
- **Why?** **TP53-mutated AML** has a **dismal prognosis** without transplant (even in CR1).
  - **Allo-HSCT is the only potential cure** (despite high relapse risk).
  - *Sibling match* maximizes graft-vs.-leukemia effect.
- **Why Not Others?**
  - A/C) Venetoclax+azacitidine: Delays transplant (TP53 clones resist hypomethylating agents).
  - D) Auto-HSCT: No graft-vs.-leukemia effect; useless in AML.

Final Insights for Shahd Kamal:
1. **MRD Dynamics**:
   - NPM1 PCR is quantitative**log reduction predicts outcomes**.
   - **Persistent MRD post-consolidation** = consider transplant.
2. **TP53 Realities**:
   - Transplant early, but counsel on **high relapse risk** (5070% post-HSCT).
   - Emerging data: **CD47 inhibitors (magrolimab)** + azacitidine trials.

Session ID:
Assessment Domain

Session-66:
Rationale

Session ID:
Instructional Design and Pedagogical

Session-66:
The session's instructional design is excellent. The case is expertly scaffolded, progressing logically from initial diagnosis and risk stratification through induction, consolidation, and complex long-term management challenges. The quality of the embedded feedback is a key strength; each rationale is detailed, cites current clinical guidelines (e.g., IDSA) or major trials (e.g., ALFA-0701), and includes instructive "Why not the others?" sections that address common clinical decision-making pitfalls. Instructions are exceptionally clear. The only minor weakness is that learning objectives are not explicitly stated at the outset, though the educational goals are strongly implied by the session's coherent structure.

Session ID:

Clinical Authenticity and Content Fidelity

Session-66:

The clinical fidelity of this session is outstanding. The clinical narrative is highly plausible and indistinguishable from an authentic patient case seen in practice. All therapeutic decisions and rationales are perfectly aligned with current evidence-based guidelines for AML management (e.g., NCCN, ELN). Pharmacotherapeutic recommendations, such as adding Gemtuzumab Ozogamicin for CBF-AML, using HiDAC for consolidation in favorable-risk disease, and proceeding directly to transplant for TP53-mutated AML, represent optimal, guideline-directed choices. Crucially, the case remains strictly within its specified disease domain, correctly applying therapies to the appropriate AML subtypes without any evidence of domain entanglement.

Session ID:

Modeled Clinical Reasoning

Session-66:

The simulation flawlessly models an expert clinical reasoning process. Each phase of the case presents a well-defined clinical problem with the necessary data for a decision. The multiple-choice options provide an excellent breadth of plausible and relevant alternatives, including key distractors that effectively test nuanced understanding (e.g., distinguishing the role of 7+3 alone vs. with GO, or choosing between consolidation chemotherapy and transplant). The overall narrative demonstrates sound, risk-adjusted prioritization. The clinical outcomes described for each decision are realistic, specific, and highly instructive, reinforcing the learning points effectively.

Session-67

Session ID:
LLM

Session-67:
DeepSeek V2

Session ID:
Session Transcript

Session-67:
Case Presentation
Patient: 48-year-old male
Chief Complaint: Fatigue, early satiety, and unexplained weight loss (5 kg over 2 months)
Physical Exam:
Splenomegaly (palpable 6 cm below costal margin)
No lymphadenopathy or hepatomegaly
Initial Labs:
WBC: 98 x 10/L () with left shift and basophilia (12%)
Hb: 10.2 g/dL ()
Platelets: 450 x 10/L ()
LDH: 850 U/L ()
Question 1 (Knowledge Recall)
Which test is required to confirm a diagnosis of Chronic Myeloid Leukemia (CML)?
A) Flow cytometry for CD34+ cells
B) BCR::ABL1 PCR or FISH on peripheral blood/bone marrow
C) Serum protein electrophoresis (SPEP)
D) JAK2 V617F mutation testing
Type your answer (A, B, C, or D) and a brief rationale.
(Ill provide feedback once you respond.)
(Answer: B)

Feedback on Question 1 (Correct Answer: B)
Correct!
BCR::ABL1 PCR or FISH is the definitive test to diagnose CML, as it detects the t(9;22) Philadelphia chromosome (present in >95% of cases).
Rationale: The patients presentation (splenomegaly, leukocytosis with basophilia) is classic for CML, but genetic confirmation is mandatory per ESMO/ELN guidelines.
Why Not Other Options?
A) Flow cytometry: Useful for acute leukemia immunophenotyping but not CML diagnosis.
C) SPEP: Rules out plasma cell disorders (e.g., myeloma), irrelevant here.
D) JAK2 V617F: Associated with polycythemia vera/essential thrombocythemia, not CML.
Case Presentation
Patient: 48-year-old male
Chief Complaint: Fatigue, early satiety, and unexplained weight loss (5 kg over 2 months)
Physical Exam:
Splenomegaly (palpable 6 cm below costal margin)
No lymphadenopathy or hepatomegaly
Initial Labs:
WBC: 98 x 10/L () with left shift and basophilia (12%)

Hb: 10.2 g/dL ()

Platelets: 450 x 10/L ()

LDH: 850 U/L ()

Question 2 (Application)

The patients bone marrow cytogenetics shows t(9;22)(q34;q11). What is the next critical step in workup?

A) Start imatinib 400 mg daily immediately

B) Perform additional mutation screening (e.g., T315I, E255K)

C) Risk-stratify using ELN 2023 criteria (e.g., Sokal, EUTOS scores)

D) Order a PET-CT to assess for lymphoid blast crisis

Type your answer (A, B, C, or D) and justify your choice.

(Ill evaluate your clinical reasoning!)

(Answer: C)

Feedback on Question 2 (Correct Answer: C)

Correct!

Risk stratification (ELN 2023) is critical before therapy selection. This patients spleen size, basophilia, and thrombocytosis suggest possible high-risk disease (needs Sokal/EUTOS score calculation).

Rationale: ESMO mandates risk-adapted therapy (e.g., 2G-TKIs for high-risk, imatinib for low-risk).

Why Not Other Options?

A) Start imatinib: Premature without risk assessment. Upfront 2G-TKI may be preferred for high-risk.

B) Mutation screening: Typically reserved for resistant disease (not at diagnosis).

D) PET-CT: Unnecessary unless blast crisis is suspected (requires >20% blasts).

Case Presentation

Patient: 48-year-old male

Chief Complaint: Fatigue, early satiety, and unexplained weight loss (5 kg over 2 months)

Physical Exam:

Splenomegaly (palpable 6 cm below costal margin)

No lymphadenopathy or hepatomegaly

Initial Labs:

WBC: 98 x 10/L () with left shift and basophilia (12%)

Hb: 10.2 g/dL ()

Platelets: 450 x 10/L ()

LDH: 850 U/L ()

Question 3 (Analysis)

The patients Sokal score is high-risk. Per ELN 2023, which is the preferred first-line TKI?

A) Imatinib 400 mg daily

B) Dasatinib 100 mg daily

C) Nilotinib 300 mg BID

D) Ponatinib 45 mg daily

Type your answer (A, B, or C) and explain your choice.

Correct Answer: B

Feedback on Question 3 (Correct Answer: B or C Both are Guideline-Compliant!)

Correct!

Dasatinib 100 mg daily (B) is an appropriate first-line choice for high-risk CML per ELN 2023. It demonstrates superior MMR rates (64% vs. 45% with imatinib at 12 months) and reduced progression risk in high-risk patients.

Why Not Other Options?

A) Imatinib: Less effective in high-risk disease (lower MMR rates, higher progression risk).

D) Ponatinib: Reserved for salvage therapy or T315I mutation (not first-line).

C) Nilotinib 300 mg BID is also correct and guideline-endorsed, but the patients unmentioned diabetes status could influence choice (nilotinib exacerbates hyperglycemia).

Phase 2: Treatment Initiation & Supportive Care

Scenario: The patient starts dasatinib 100 mg daily. At the 1-month follow-up:

Labs: WBC 12 x 10/L (), platelets 220 x 10/L (), Hb 9.8 g/dL ()

Symptoms: New-onset pleuritic chest pain and dyspnea.

Case Presentation

Patient: 48-year-old male

Chief Complaint: Fatigue, early satiety, and unexplained weight loss (5 kg over 2 months)

Physical Exam:

Splenomegaly (palpable 6 cm below costal margin)

No lymphadenopathy or hepatomegaly

Initial Labs:

WBC: 98 x 10/L () with left shift and basophilia (12%)

Hb: 10.2 g/dL ()

Platelets: 450 x 10/L ()

LDH: 850 U/L ()

Question 4 (Evaluation)

What is the most likely cause of the chest pain, and how would you manage it?

A) Tumor lysis syndrome Administer rasburicase and IV fluids

B) Dasatinib-induced pleural effusion Hold dasatinib, start prednisone 20 mg/day

C) Pulmonary embolism Start enoxaparin and CT angiography

D) Pericarditis Initiate colchicine and NSAIDs

Type your answer (A, B, C, or D) and justify your management plan.

Answer: B

Feedback on Question 4 (Correct Answer: B)

Correct!

Dasatinib-induced pleural effusion is the most likely cause given the timing (1 month post-initiation) and classic pleuritic symptoms.

Management:

Hold dasatinib immediately (per ESMO 2023).

Start prednisone 20 mg/day (glucocorticoids reduce inflammation/fluid accumulation).

After resolution, rechallenge at a lower dose (50 mg daily) or switch to nilotinib if recurrent.

Why Not Other Options?

A) Tumor lysis syndrome: Unlikely without hyperkalemia/renal dysfunction (and typically occurs earlier, within 72h of TKI initiation).

C) Pulmonary embolism: Less probable without risk factors (e.g., immobility, prior VTE); would require imaging confirmation before anticoagulation.

D) Pericarditis: Rare with dasatinib; more common with bosutinib.

Case Presentation

Patient: 48-year-old male

Chief Complaint: Fatigue, early satiety, and unexplained weight loss (5 kg over 2 months)

Physical Exam:

Splenomegaly (palpable 6 cm below costal margin)

No lymphadenopathy or hepatomegaly

Initial Labs:

WBC: 98 x 10/L () with left shift and basophilia (12%)

Hb: 10.2 g/dL ()

Platelets: 450 x 10/L ()

LDH: 850 U/L ()

Question 5 (Application)

The pleural effusion resolves with prednisone. What is the next step in dasatinib management?

A) Permanently discontinue dasatinib and switch to bosutinib

B) Rechallenge dasatinib at 50 mg daily with close monitoring

C) Continue prednisone indefinitely to prevent recurrence

D) Switch to imatinib 400 mg daily (no effusion risk)

Type your answer (A, B, C, or D) and explain your reasoning.

Answer: B

Feedback on Question 5 (Correct Answer: B)

Correct!

Rechallenge dasatinib at 50 mg daily is the guideline-endorsed approach (ESMO 2023) for a first occurrence of pleural effusion, provided the patient achieved adequate initial response.

Rationale:

Dose reduction (from 100 mg 50 mg) decreases effusion risk while maintaining efficacy.

High-risk patients benefit from dasatinibs superior MMR rates vs. imatinib.

Why Not Other Options?

A) Switch to bosutinib: Overly aggressive for a first effusion; bosutinib has its own toxicity profile (diarrhea, liver toxicity).

C) Continue prednisone indefinitely: Unnecessary and increases infection/metabolic risks.

D) Switch to imatinib: Suboptimal for high-risk disease (lower MMR rates).

Case Presentation

Patient: 48-year-old male

Chief Complaint: Fatigue, early satiety, and unexplained weight loss (5 kg over 2 months)

Physical Exam:

Splenomegaly (palpable 6 cm below costal margin)

No lymphadenopathy or hepatomegaly

Initial Labs:

WBC: 98 x 10/L () with left shift and basophilia (12%)

Hb: 10.2 g/dL ()

Platelets: 450 x 10/L ()

LDH: 850 U/L ()

Question 6 (Analysis)

At 3 months, the patients BCR::ABL1 IS transcript level is 8.5%. How do you interpret this result?

A) Optimal response (proceed with current therapy)

B) Warning (monitor closely, consider adherence assessment)

C) Treatment failure (switch to alternate TKI)

D) Molecular relapse (proceed to allogeneic transplant)

Type your answer (A, B, C, or D) and justify based on ELN 2023 response criteria.

Answer: B

feedback on Question 6 (Correct Answer: B)

Correct!

8.5% at 3 months qualifies as a warning response per ELN 2023, especially for this high-risk patient.

Rationale:

While 10% is technically optimal, values >1% at 3 months in high-risk disease warrant:

Adherence assessment (e.g., pill count, drug interactions).

Closer monitoring (repeat PCR at 6 months; consider dose escalation if no improvement).

High-risk patients need faster declines (e.g., <1% at 3 months) to reduce progression risk.

Why Not Other Options?

A) Optimal: Too lenient for high-risk; may delay necessary intervention.

C) Failure: Incorrect (failure is >10% at 3 months).

D) Relapse: Irrelevant (no prior response loss).

Phase 3: Long-Term Management

Scenario: At 12 months, the patient achieves BCR::ABL1 IS 0.1% (MMR) but develops grade 3 thrombocytopenia (platelets 45 x 10/L).

Case Presentation

Patient: 48-year-old male

Chief Complaint: Fatigue, early satiety, and unexplained weight loss (5 kg over 2 months)

Physical Exam:

Splenomegaly (palpable 6 cm below costal margin)

No lymphadenopathy or hepatomegaly

Initial Labs:

WBC: 98 x 10/L () with left shift and basophilia (12%)

Hb: 10.2 g/dL ()
Platelets: 450 x 10/L ()
LDH: 850 U/L ()
Question 7 (Evaluation)
What is the best management for persistent thrombocytopenia?
A) Permanently discontinue dasatinib and switch to nilotinib
B) Reduce dasatinib to 50 mg every other day and monitor
C) Transfuse platelets and continue dasatinib at current dose
D) Add eltrombopag to stimulate platelet production
Type your answer (A, B, C, or D) and justify based on ESMO guidelines.
Answer: B
Feedback on Question 7 (Correct Answer: B)
Correct!
Reduce dasatinib to 50 mg every other day is the guideline-endorsed approach (ELN 2023) for grade 3 thrombocytopenia in a patient with achieved MMR.
Rationale:
Dose reduction (not discontinuation) balances efficacy (maintaining MMR) and safety (avoiding bleeding risk).
Platelets typically recover within 24 weeks after dose adjustment.
Why Not Other Options?
A) Switch to nilotinib: Overly aggressive for first occurrence; nilotinib has its own risks (hyperglycemia, vascular events)
C) Transfuse and continue: Transfusions are for active bleeding, not chronic management. Current dose would worsen thrombocytopenia.
D) Eltrombopag: Not indicated for TKI-induced cytopenias (used in ITP or hepatitis-related thrombocytopenia).

Case Presentation
Patient: 48-year-old male
Chief Complaint: Fatigue, early satiety, and unexplained weight loss (5 kg over 2 months)
Physical Exam:
Splenomegaly (palpable 6 cm below costal margin)
No lymphadenopathy or hepatomegaly
Initial Labs:
WBC: 98 x 10/L () with left shift and basophilia (12%)
Hb: 10.2 g/dL ()
Platelets: 450 x 10/L ()
LDH: 850 U/L ()
Question 8 (Advanced Analysis)
At 24 months, the patient maintains MMR but develops new-onset pulmonary hypertension (PAH: RVSP 45 mmHg on echo). What is the most likely cause and management?
A) CML progression to blast crisis Perform bone marrow biopsy
B) Dasatinib-induced PAH Discontinue dasatinib, switch to bosutinib
C) Chronic thromboembolic PAH Start lifelong anticoagulation
D) Left ventricular dysfunction Initiate beta-blockers and ACE inhibitors
Type your answer (A, B, C, or D) and justify.
Answer: B
Feedback on Question 8 (Correct Answer: B)
Correct!
Dasatinib-induced PAH is the most likely diagnosis given the timeline (24 months on therapy) and echocardiographic findings.
Management per ELN 2023:
Permanently discontinue dasatinib (PAH may not fully reverse if drug is continued).
Switch to bosutinib or nilotinib (non-PAH-associated TKIs).
Why Not Other Options?
A) Blast crisis: Would show BCR::ABL1 rise + blasts on marrow biopsy (not seen here).
C) Thromboembolic PAH: Requires evidence of chronic PE (e.g., mismatched V/Q scan).
D) LV dysfunction: RVSP elevation suggests pulmonary (not left heart) pathology.

Case Presentation
Patient: 48-year-old male
Chief Complaint: Fatigue, early satiety, and unexplained weight loss (5 kg over 2 months)
Physical Exam:
Splenomegaly (palpable 6 cm below costal margin)
No lymphadenopathy or hepatomegaly
Initial Labs:
WBC: 98 x 10/L () with left shift and basophilia (12%)
Hb: 10.2 g/dL ()
Platelets: 450 x 10/L ()
LDH: 850 U/L ()
Question 9 (Synthesis)
The patient switches to bosutinib but develops grade 3 diarrhea and AST/ALT 3x ULN. What is the next step?
A) Hold bosutinib, monitor LFTs, then rechallenge at reduced dose
B) Permanently switch to imatinib despite high-risk status
C) Add loperamide and continue bosutinib at current dose
D) Initiate prednisone for suspected autoimmune hepatitis
Type your answer (A, B, C, or D) and justify.
Answer: A
Feedback on Question 9 (Correct Answer: A)
Correct!
Hold bosutinib, monitor LFTs, then rechallenge at reduced dose is the guideline-directed approach (ESMO 2023) for concurrent grade 3 diarrhea and hepatotoxicity.
Rationale:
Diarrhea: Manage with dose interruption resume at 300 mg (or 200 mg if persistent).
Transaminitis: Hold until ALT/AST 1.5x ULN, then reduce by 1 dose level (e.g., 400 mg 300 mg).
High-risk status necessitates maintaining TKI therapy if possible.
Why Not Other Options?
B) Switch to imatinib: Suboptimal for high-risk disease (lower MMR rates).
C) Continue bosutinib: Unsafe with grade 3 toxicity (requires dose modification).
D) Prednisone: Unnecessary (no evidence of autoimmune hepatitis; drug-induced liver injury is dose-dependent).
Case Presentation
Patient: 48-year-old male
Chief Complaint: Fatigue, early satiety, and unexplained weight loss (5 kg over 2 months)
Physical Exam:
Splenomegaly (palpable 6 cm below costal margin)
No lymphadenopathy or hepatomegaly
Initial Labs:
WBC: 98 x 10/L () with left shift and basophilia (12%)
Hb: 10.2 g/dL ()
Platelets: 450 x 10/L ()
LDH: 850 U/L ()
Question 10 (Evaluation Final Assessment)
At 5 years, the patient maintains MR4.5 (BCR::ABL1 IS 0.0032%) with no toxicity. Per ELN 2023, what is the next consideration?
A) Continue bosutinib indefinitely (lifelong therapy)
B) Stop TKI and monitor for treatment-free remission (TFR)
C) Switch to ponatinib to eradicate residual disease
D) Reduce bosutinib dose to minimize long-term toxicity
Type your answer (A, B, C, or D) and justify based on TFR eligibility criteria
Answer: B
feedback on Question 10 (Correct Answer: B)
Correct!
Stop TKI and monitor for treatment-free remission (TFR) is the guideline-recommended option (ELN 2023) for this patient, who meets all criteria:

MR4.5 (0.0032%) sustained for 3 years
5 years of TKI therapy
No prior resistance
Rationale:
TFR is now a key goal in CML management, with ~40-60% of eligible patients maintaining remission off therapy.
Close monitoring (monthly PCR for 6 months, then quarterly) is required to detect molecular relapse (defined as loss of MMR).
Why Not Other Options?
A) Continue indefinitely: While safe, misses the opportunity for TFR in an ideal candidate.
C) Ponatinib: Unnecessary (no T315I mutation or resistance; MR4.5 is the deepest expected response).
D) Dose reduction: No evidence that lower doses improve TFR success; may increase relapse risk.

Session ID:
Assessment Domain

Session-67:
Rationale

Session ID:
Instructional Design and Pedagogical

Session-67:
The session's instructional design is excellent. The case is expertly scaffolded, progressing logically from initial diagnosis and risk stratification (Questions 1-3) through the management of acute and chronic toxicities (Questions 4-9) to advanced long-term goals like treatment-free remission (Question 10). The embedded feedback is a major strength, providing clear, guideline-anchored rationales (citing ELN/ESMO 2023) and explaining why distractors are incorrect. Instructions are exceptionally clear. The only minor area for improvement is the lack of explicitly stated learning objectives at the beginning of the session, though the educational goals are strongly implied by the structure.

Session ID:
Clinical Authenticity and Content Fidelity

Session-67:
The session demonstrates outstanding clinical fidelity and authenticity. The patient narrative is highly plausible, mirroring a real-world Chronic Myeloid Leukemia (CML) case from presentation to long-term follow-up. All therapeutic decisions, monitoring parameters, and management of specific toxicities (e.g., dasatinib-induced pleural effusion/PAH, bosutinib-induced diarrhea/transaminitis) are perfectly aligned with current, evidence-based guidelines like ELN 2023. Pharmacotherapeutic choices are optimal and well-justified for each stage of the disease. Crucially, the session exhibits perfect domain specificity, remaining entirely within the context of CML without any entanglement of concepts or treatments from other disease states.

Session ID:
Modeled Clinical Reasoning

Session-67:
The simulation excels at modeling an expert clinical reasoning process. Each stage presents a well-defined clinical problem with all necessary data for a decision. The multiple-choice options provide an excellent range of relevant and plausible alternatives, including strong distractors that effectively test nuanced understanding (e.g., distinguishing TKI side effects from other pathologies like PE). The narrative demonstrates sound, risk-adjusted prioritization throughout, such as risk-stratifying before treatment and dose-reducing for toxicity rather than prematurely switching therapies. The clinical outcomes for each decision are realistic, specific, and highly instructive, reinforcing the learning objectives at every step.

Session-68

Session ID:
LLM

Session-68:
DeepSeek V2

Session ID:
Session Transcript

Session-68:
Phase 1: Initial Presentation & Diagnostic Workup
Patient Case:
A 48-year-old female presents with fatigue, early satiety, and 4 kg unintentional weight loss over 2 months. Physical exam reveals splenomegaly (palpable 6 cm below costal margin). No bleeding or fever.

Initial Labs:
CBC: WBC 98 x10/L (neutrophils 65%, basophils 8%, blasts 2%)

Hemoglobin: 9.8 g/dL

Platelets: 450 x10/L

LDH: 2x ULN

Question 1 (Diagnosis):
What is the MOST critical next diagnostic step?
A. Peripheral blood flow cytometry
B. Bone marrow biopsy with cytogenetics (Ph chromosome) and BCR::ABL1 PCR
C. JAK2 V617F mutation testing
D. Start hydroxyurea immediately for leukocytosis

Type your answer (A, B, C, or D).

(Need guidance? Type "HINT")

Why This Matters:

The 2022 WHO criteria require BCR::ABL1+ by PCR or FISH and Ph+ cytogenetics for CML diagnosis.

Bone marrow biopsy assesses blast % and fibrosis (ELN 2022).

answer is B
Feedback for Question 1 (Diagnosis):
Correct Answer: B (Bone marrow biopsy with cytogenetics and BCR::ABL1 PCR)

Rationale:

ESMO/ELN 2022 guidelines mandate bone marrow biopsy for:

Cytogenetics: Detection of the Philadelphia chromosome (t(9;22)).

Molecular testing: Quantitative BCR::ABL1 PCR to establish baseline for future monitoring.

Blast % and fibrosis assessment (critical for phase determination).

Why Not Other Options?

A. Peripheral flow cytometry: Useful for acute leukemia but cannot confirm CML.

C. JAK2 testing: Rules out MPNs (e.g., PV/ET) but irrelevant here (CML is BCR::ABL1-driven).

D. Hydroxyurea: Cytoreduction may be needed later but is not diagnostic.

Progression:
The biopsy confirms chronic-phase CML (BCR::ABL1+, Ph+). No additional mutations (e.g., T315I).

Question 2 (Risk Stratification):
Calculate this patients Sokal score using:

Age: 48 years

Spleen size: 6 cm below costal margin

Platelets: 450 x10/L

Blasts: 2% (peripheral blood)

Sokal Score Formula:

Exp
[
0.0116

(
Age

43.4
)
+
0.0345

(
Spleen

7.51
)
+
0.188

(
Platelets
700

0.563
)
+
0.0887

(
Blasts

2.10
)
]
Exp[0.0116(Age43.4)+0.0345(Spleen7.51)+0.188(
700
Platelets

0.563)+0.0887(Blasts2.10)]
What is her risk group?
A. Low
B. Intermediate
C. High

B
Feedback for Question 2 (Risk Stratification):
Your Answer: B (Intermediate)
Correct Answer: A (Low Risk)

Step-by-Step Calculation:
Age Factor:
0.0116

(
48

43.4
)
=
0.0116

4.6
=
0.053
0.0116(4843.4)=0.01164.6=0.053

Spleen Factor:
0.0345

(
6

7.51
)
=

0.0345

(

1.51
)
=

0.052
0.0345(67.51)=0.0345(1.51)=0.052

Platelet Factor:
0.188

(
450
700

0.563
)
=
0.188

(
0.643

0.563
)
=
0.188

0.08
=
0.015
0.188(
700
450

0.563)=0.188(0.6430.563)=0.1880.08=0.015

Blast Factor:
0.0887

(
2

2.10
)
=
0.0887

(

0.10

)
=

0.009
0.0887(22.10)=0.0887(0.10)=0.009

Sum of Factors:
0.053

0.052
+
0.015

0.009
=
0.007
0.0530.052+0.0150.009=0.007

Exponential (Sokal Score):
e
0.007
=
1.007
e
0.007
 =1.007

Interpretation (ELN 2022):

Low risk: <0.8

Intermediate: 0.81.2

High risk: >1.2

This patients score (1.007) falls in the intermediate range.

(Correction: Your answer was correct! The initial feedback misclassified the risk groups. Well done!)

Clinical Implications:
Low/Intermediate risk: 1st-line imatinib or 2G TKIs (e.g., dasatinib/nilotinib) are options.

High risk: Favor 2G TKIs for faster/more profound responses.

Progression:
The patient is intermediate-risk (Sokal = 1.007) and has no comorbidities (normal cardiac/renal function).
Question 3 (First-Line Therapy):
Which is the MOST appropriate first-line TKI?
A. Imatinib 400 mg daily
B. Dasatinib 100 mg daily
C. Nilotinib 300 mg BID
D. Ponatinib 45 mg daily

Type your answer (A, B, C, or D). (Consider Sokal risk and guidelines!)

Key References:

ESMO 2022: 2G TKIs preferred for high-risk; imatinib acceptable for low/intermediate.

ELN 2022: 2G TKIs yield faster responses but trade off with toxicity.
Your Answer: A (Imatinib 400 mg daily)
Correct Answer: A (Imatinib 400 mg daily)

Evidence-Based Rationale:
Why Imatinib?
ELN 2022 Guidelines:

Low/Intermediate-risk patients: Imatinib remains a standard first-line option, especially with no high-risk features (e.g., high blast %, additional mutations).

10-year OS data: ~85% with imatinib in chronic phase (IRIS trial).

Patient-Specific Factors:

Intermediate Sokal risk (1.007): No clear superiority of 2G TKIs in this group.

No comorbidities: Tolerability expected (vs. 2G TKIs' stricter cardiac/pulmonary monitoring).

Why Not Other Options?
B. Dasatinib / C. Nilotinib:

Preferred for high-risk (Sokal >1.2) or if deep molecular response (DMR) is a goal (e.g., young patient aiming for TFR).

Higher toxicity (pleural effusion [dasatinib], hyperglycemia [nilotinib]).

D. Ponatinib:

Reserved for T315I mutation or refractory disease due to vascular toxicity risk.

Progression:
The patient starts imatinib 400 mg daily. At 3-month follow-up:

Monitoring Results:
Hematologic response: Normal CBC (WBC 6.5 x10/L, platelets 220 x10/L).

Cytogenetic response: Ph+ 35% (partial cytogenetic response, PCyR).

Molecular response: BCR::ABL1 IS 8.5%.

Question 4 (Response Evaluation):
Does this patient meet ELN 2022 criteria for OPTIMAL response at 3 months?
A. Yes
B. No

Type your answer (A or B). (Refer to ELN 2022 response criteria!)

Key ELN 2022 Benchmarks for 3 Months:

Optimal: BCR::ABL1 IS 10% AND Ph+ 35%.

Warning: BCR::ABL1 IS >10% OR Ph+ >35%.

Failure: No CHR or Ph+ >95%.

Clinical Impact:

Suboptimal response may prompt early switch to 2G TKI.
Feedback for Question 4 (Response Evaluation):
Your Answer: A (Yes)
Correct Answer: A (Yes)

ELN 2022 Optimal Response Criteria (3 Months):
BCR::ABL1 IS 10% (Patient: 8.5%)

Ph+ 35% (Patient: 35%)

Conclusion: The patient meets both criteria for optimal response.

Clinical Implications:
Continue imatinib 400 mg daily with close monitoring.

Next milestone:

6 months: Target BCR::ABL1 IS 1% (major molecular response, MMR).

Progression:
At 6 months, the patients BCR::ABL1 IS is 2.5% (MMR not achieved).

Question 5 (Management):
According to ELN 2022, what is the NEXT BEST step?
A. Continue imatinib (acceptable response)
B. Switch to dasatinib or nilotinib
C. Increase imatinib to 600 mg daily
D. Refer for allogeneic stem cell transplant

Type your answer (A, B, C, or D). (Consider ELN "warning" vs. "failure" criteria!)

ELN 2022 6-Month Benchmarks:

Optimal: BCR::ABL1 IS 1% (MMR).

Warning: BCR::ABL1 IS >110%.

Failure: BCR::ABL1 IS >10%.

Why This Matters:

Warning responses may benefit from early intervention (e.g., switch to 2G TKI
Feedback for Question 5 (Management):
Your Answer: A (Continue imatinib)

Correct Answer: B (Switch to dasatinib or nilotinib)

ELN 2022 Guidance for 6-Month "Warning" Response:
BCR::ABL1 IS 2.5% = Warning (>110%).

Recommended Action:

Switch to a 2G TKI (dasatinib/nilotinib) to improve the likelihood of achieving MMR (0.1%) by 12 months.

Supported by ENESTnd/DASISION trials: 2G TKIs achieve faster/deeper responses in suboptimal imatinib responders.

Why Not Other Options?

A. Continue imatinib: Risk of progression to accelerated/blast phase (5-year risk: ~7% with warning responses).

C. Increase imatinib dose: No proven benefit (600 mg only for failure, not warning).

D. Transplant: Not indicated in chronic phase without TKI failure.

Progression:
The patient switches to dasatinib 100 mg daily. At 12 months:

BCR::ABL1 IS: 0.6% (MMR not yet achieved).

Question 6 (Next Steps):
What is the BEST management approach?
A. Continue dasatinib (adequate response)
B. Switch to nilotinib
C. Check compliance and BCR::ABL1 kinase domain mutation testing
D. Refer for transplant
Feedback for Question 6 (Next Steps):
Your Answer: C (Check compliance and BCR::ABL1 mutation testing)
Correct Answer: C (Check compliance and BCR::ABL1 mutation testing)

Evidence-Based Rationale:
Why This is the Best Approach:
Compliance First:

Up to 30% of suboptimal responses are due to non-adherence (ELN 2022). Always assess this before changing therapy!

Action: Use pill counts/pharmacy records and patient interviews.

Mutation Testing (Critical!):

BCR::ABL1 KD mutations (e.g., T315I, F317L) can explain resistance and guide next-line therapy:

T315I: Requires ponatinib.

F317L/V: Prefer nilotinib over dasatinib.

ELN 2022 Recommendation: Mandatory for warning/failure responses.

Why Not Other Options?

A. Continue dasatinib: Risk of progression if resistance exists (MMR not achieved by 12 months = warning).

B. Switch to nilotinib: Premature without excluding adherence issues or identifying mutations.

D. Transplant: Not indicated in chronic phase without confirmed TKI failure.

Progression:
Compliance confirmed, but mutation testing reveals F317L mutation.

Question 7 (Mutation-Driven Therapy):
Which TKI is MOST appropriate now?
A. Continue dasatinib (F317L has intermediate sensitivity)
B. Switch to nilotinib (F317L-sensitive)
C. Start ponatinib (pan-resistant)
D. Asciminib (novel option)

Type your answer (A, B, C, or D).

Key Evidence:

F317L Mutation:

High resistance to dasatinib (IC50 10-fold).

Sensitive to nilotinib/asciminib (NCCN 2023).

Ponatinib: Reserved for T315I or multi-TKI failure.

Clinical Pearl:

Asciminib (STAMP inhibitor) is FDA-approved for post-2G TKI failure with mutations (e.g., F317L).

Feedback for Question 7 (Mutation-Driven Therapy):
Your Answer: B (Switch to nilotinib)
Correct Answer: B (Switch to nilotinib)

Evidence-Based Rationale:
Why Nilotinib?
F317L Mutation Profile:

High resistance to dasatinib (reduced binding affinity).

Sensitive to nilotinib (IC50 remains low; NCCN 2023 recommends as preferred option).

Clinical Data:

ENESTmd trial: Nilotinib achieved MMR in 46% of dasatinib-resistant F317L patients.

Safety:

Avoid ponatinib (vascular toxicity risk) unless T315I is present.

Why Not Other Options?
A. Continue dasatinib: F317L confers significant resistance (failure likely).

C. Ponatinib: Overkill for F317L (use only for T315I or multi-TKI failure).

D. Asciminib: Valid option but second-line after nilotinib per current guidelines.

Progression:
The patient starts nilotinib 300 mg BID. At 18 months:

BCR::ABL1 IS: 0.08% (MMR achieved).
Question 8 (Monitoring & Toxicity):
Routine ECG shows QTc 490 ms (baseline 420 ms). What is the NEXT step?
A. Continue nilotinib (asymptomatic)
B. Reduce dose to 400 mg daily
C. Switch to asciminib
D. Hold nilotinib, correct electrolytes, repeat ECG

Type your answer (A, B, C, or D).

Key Facts:

Nilotinibs Black Box Warning: QTc prolongation (>500 ms = arrhythmia risk).

ELN 2022: Hold if QTc >480 ms; correct K+/Mg2+ before rechallenge.

Clinical Impact:

Sudden death reported in 0.3% on nilotinib (ENEST trials).
Feedback for Question 8 (Monitoring & Toxicity):
Your Answer: D (Hold nilotinib, correct electrolytes, repeat ECG)
Correct Answer: D (Hold nilotinib, correct electrolytes, repeat ECG)

Evidence-Based Rationale:
Immediate Action Required:
QTc >480 ms = Hold nilotinib immediately (per FDA/ELN 2022).

Correct Electrolytes:

Hypokalemia/hypomagnesemia exacerbate QTc prolongation.

Action: IV/oral repletion + repeat ECG in 2448 hours.

Rechallenge Decision:

If QTc returns to <450 ms: Resume at reduced dose (300 mg daily) with weekly ECGs.

If persistent: Switch to asciminib (no QTc risk).

Why Not Other Options?
A. Continue nilotinib: Life-threatening torsades de pointes risk.

B. Dose reduction alone: Insufficient without addressing reversible causes.

C. Switch to asciminib: Premature before electrolyte correction/ECG reassessment.

Progression:
After electrolyte correction, QTc normalizes to 430 ms. Nilotinib is resumed at 300 mg daily.

Question 9 (Long-Term Monitoring):
The patient achieves MR4.5 (BCR::ABL1 IS 0.0032%) at 24 months. What is the NEXT step?
A. Continue nilotinib indefinitely
B. Stop TKI and monitor for treatment-free remission (TFR)
C. Reduce nilotinib to 150 mg daily
D. Switch to imatinib for maintenance

Type your answer (A, B, C, or D).

Key Guidelines:

ELN 2022 TFR Criteria:

5 years on TKI + sustained MR4.5 for 2 years.

This patient is ineligible (only 24 months of therapy).

NCCN 2023: MR4.5 must be maintained 2 years before TFR attempt.

Why This Matters:

Premature TKI cessation 60% relapse risk (EURO-SKI trial).
eedback for Question 9 (Long-Term Monitoring):
Your Answer: A (Continue nilotinib indefinitely)
Correct Answer: A (Continue nilotinib indefinitely)

Evidence-Based Rationale:
Why Continue Nilotinib?
TFR Eligibility Not Met:

Minimum Duration: 5 years of TKI therapy (patient only has 2 years).

Sustained MR4.5: Requires 2 years at this depth (patient just achieved it).

ELN 2022 Recommendations:

Do not stop TKI unless all TFR criteria are fulfilled (to avoid relapse).

Monitoring: Continue BCR::ABL1 testing every 3 months.

Why Not Other Options?
B. Stop for TFR: High relapse risk (6070% in patients with <5 years of therapy).

C. Dose Reduction: No proven benefit in maintaining response (standard dose required unless toxicity occurs).

D. Switch to Imatinib: Risk of losing MR4.5 (nilotinib has higher potency).

Progression:

The patient continues nilotinib 300 mg daily. At 5 years:

Sustained MR4.5 for 3 years with no toxicity.

Question 10 (Treatment-Free Remission):
The patient now desires TFR. What is the NEXT step?
A. Stop nilotinib and monitor BCR::ABL1 monthly
B. Continue nilotinib (lifelong therapy required)
C. Reduce dose for 6 months before stopping
D. Confirm BCR::ABL1 undetectability by digital PCR

Type your answer (A, B, C, or D).

ELN 2022 TFR Protocol:

Prerequisites:

5 years TKI therapy + 2 years MR4.5.

Digital PCR (dPCR): Not mandatory but enhances confidence (detects 0.0001%).

Monitoring Post-Cessation:

Monthly BCR::ABL1 x12 months, then quarterly.

Clinical Pearl:

4050% maintain TFR long-term (STIM2 trial). Relapses typically occur within 6 months.
Feedback for Question 10 (Treatment-Free Remission):
Your Answer: A (Stop nilotinib and monitor BCR::ABL1 monthly)
Correct Answer: A (Stop nilotinib and monitor BCR::ABL1 monthly)

Evidence-Based Rationale:
Why Stopping Nilotinib is Appropriate Now:
TFR Eligibility Met:

5+ years on TKI (nilotinib x5 years).

Sustained MR4.5 for 3 years (exceeds 2-year requirement).

ELN 2022 TFR Protocol:

Immediate cessation (no taper needed).

Monthly BCR::ABL1 monitoring for 12 months (then quarterly).

Relapse Management:

Restart nilotinib if BCR::ABL1 >0.1%: >90% regain MMR (STIM trial).

Why Not Other Options?
B. Lifelong therapy: Not required if TFR criteria are met (shared decision-making).

C. Dose reduction: No evidence it reduces relapse risk.

D. Digital PCR: Adds prognostic value but is not required for TFR.

Session ID:
Assessment Domain

Session-68:
Rationale

Session ID:
Instructional Design and Pedagogical

Session-68:
The session demonstrates exceptional instructional design. The case is expertly scaffolded, building logically from diagnosis and risk stratification through the complexities of first-line therapy, response monitoring, managing suboptimal response, mutation-driven therapy selection, toxicity management, and long-term goals like treatment-free remission (TFR). The embedded feedback is consistently high-quality, providing clear, guideline-based rationales (ELN, ESMO, NCCN) that explain not only the correct answer but also why distractors are incorrect. Instructions are clear and intuitive. The only minor area for improvement is the lack of explicitly stated, overarching learning objectives at the start; however, the educational goals are clearly implied by the session's impeccable structure.

Session ID:
Clinical Authenticity and Content Fidelity

Session-68:
The sessions clinical fidelity is flawless. The patient narrative is highly plausible and mirrors a typical CML case progression. Every diagnostic and therapeutic decision pointfrom the initial workup and Sokal score calculation to the sequential use of imatinib, dasatinib, and nilotinibis perfectly aligned with current, evidence-based clinical guidelines. The pharmacotherapeutic choices are optimal and expertly justified based on risk, response milestones, resistance mutations (F317L), and toxicity (QTc prolongation). Crucially, the case remains entirely within the CML domain, with no evidence of domain entanglement; all concepts and therapies are appropriately applied.

Session ID:
Modeled Clinical Reasoning

Session-68:
The simulation provides an outstanding model of expert clinical reasoning. Each question frames a distinct and relevant clinical problem, providing all necessary data for an informed decision. The multiple-choice options are well-crafted, offering a range of plausible alternatives and key distractors that effectively probe for nuanced understanding (e.g., distinguishing between a "warning" and "failure" response). The narrative demonstrates impeccable prioritization, such as assessing for adherence and resistance mutations before switching therapy and managing acute toxicity before making long-term changes. The clinical outcomes are realistic and serve to reinforce the learning objectives at every stage of the patient's journey.

Session-69

Session ID:
LLM

Session-69:
DeepSeek V2

Session ID:
Session Transcript

Session-69:
What is the MOST critical next step to confirm CML?
A) Peripheral blood smear
B) Bone marrow biopsy with cytogenetics (Ph chromosome)
C) JAK2 V617F mutation testing
D) Serum uric acid level
Correct Answer: B) Bone marrow biopsy with cytogenetics (Ph chromosome)

Rationale (ESMO/ELN Guidelines):

The gold standard for CML diagnosis is detection of the Philadelphia chromosome (t(9;22)) via karyotyping or BCR::ABL1 fusion via FISH/PCR (mandatory per ELN 2022).
Bone marrow biopsy also assesses blast percentage (critical for phase determination) and fibrosis.
Why Not Other Options?

A) Peripheral smear: May suggest CML (e.g., basophilia, immature granulocytes) but cannot confirm it.
C) JAK2 V617F: Rules in other MPNs (e.g., PV, ET) but is negative in CML.
D) Serum uric acid: May be elevated due to tumor lysis but is nonspecific.
Mr. Ahmeds bone marrow confirms Ph+ CML (chronic phase). His Sokal score is "intermediate risk." What is the FIRST-LINE therapy per ESMO 2020?
A) Imatinib 400 mg daily
B) Dasatinib 100 mg daily
C) Nilotinib 300 mg BID
D) Ponatinib 45 mg daily
Correct Answer: B) Dasatinib 100 mg daily

Rationale (ESMO 2020/ELN 2022 Guidelines):

For intermediate-risk CML (chronic phase), second-generation TKIs (dasatinib, nilotinib, bosutinib) are preferred first-line over imatinib due to superior rates of deep molecular response (MR4.5) and reduced progression risk.
Dasatinib 100 mg daily is a guideline-recommended option with proven efficacy in intermediate-risk patients.
Why Not Other Options?

A) Imatinib 400 mg daily: Less optimal for intermediate-risk (lower MR4.5 rates).
C) Nilotinib 300 mg BID: Also appropriate, but requires fasting and has higher cardiovascular risk.
D) Ponatinib 45 mg daily: Reserved for T315I mutations or refractory disease (not first-line).
Mr. Ahmed develops pleural effusion after 6 months on dasatinib. What is the NEXT step per ESMO?
A) Permanently switch to nilotinib
B) Reduce dasatinib to 70 mg daily and monitor
C) Continue dasatinib and add prednisone
D) Discontinue TKI and initiate hydroxyurea
Correct Answer: B) Reduce dasatinib to 70 mg daily and monitor

Rationale (ESMO 2020 Guidelines):

Pleural effusion is a dose-dependent adverse effect of dasatinib. First-line management involves dose reduction (to 7080 mg/day) with close monitoring (ESMO 2020, NCCN 2023).
Many patients tolerate the reduced dose without recurrence. If effusion persists, switch to another TKI (e.g., nilotinib).
Why Not Other Options?

A) Switch to nilotinib: Reserved for persistent/recurrent effusion after dose reduction.
C) Prednisone: No evidence for steroid use in dasatinib-induced effusions.
D) Hydroxyurea: Not indicatedthis is for cytoreduction in untreated CML, not TKI toxicity.
Mr. Ahmed achieves BCR::ABL1 1% (IS) at 3 months. What does this indicate per ELN 2023?
A) Optimal responsecontinue dasatinib
B) Warningconsider dose escalation
C) Treatment failureswitch TKI
D) Inadequate samplingrepeat PCR
Correct Answer: A) Optimal responsecontinue dasatinib

Rationale (ELN 2023 Guidelines):

BCR::ABL1 1% (IS) at 3 months is classified as an optimal response, correlating with long-term progression-free survival (ELN 2023).
No changes to therapy are neededcontinue dasatinib at current dose with standard monitoring (q3-month PCR).
Why Not Other Options?

B) Warning: Applies only if BCR::ABL1 >1-10% (requires closer monitoring but not dose escalation).
C) Treatment failure: Defined as BCR::ABL1 >10% or no cytogenetic response.
D) Inadequate sampling: Not relevant here unless PCR quality control issues exist.
Question 5 (TKI Resistance)
Mr. Ahmeds 12-month PCR shows BCR::ABL1 15% (IS) with no mutations detected. What is the NEXT step per ELN 2023?
A) Continue dasatinib and repeat PCR in 3 months
B) Switch to nilotinib
C) Perform BCR::ABL1 kinase domain mutation testing
D) Refer for allogeneic stem cell transplant
Optimal Answer: C) Perform BCR::ABL1 kinase domain mutation testing

Rationale (ELN 2023 Guidelines):

BCR::ABL1 >10% at 12 months = TKI failure, requiring:
Mutation testing to guide next TKI selection (e.g., T315I ponatinib; F317L avoid dasatinib).
Second-line options (e.g., nilotinib, bosutinib) depend on mutation profile.
Why Not Directly Switch to Nilotinib (B)?
Without mutation data, you risk selecting a TKI that may be ineffective (e.g., if T315I is present, nilotinib would fail).
Transplant (D): Not yet indicatedrequires 2 TKI failures.
Mr. Ahmed progresses to blast phase (80% blasts in marrow). What is the FIRST step per NCCN 2024?
A) High-dose chemotherapy + ponatinib
B) Immediate allogeneic transplant evaluation
C) Start decitabine + venetoclax
D) Switch to bosutinib
Correct Answer: A) High-dose chemotherapy + ponatinib

Rationale (NCCN 2024 Guidelines):

Blast phase CML requires aggressive therapy:
TKI (ponatinib) + acute leukemia-style chemotherapy (e.g., hyper-CVAD, FLAG-IDA) to reduce blast burden (NCCN Category 1

recommendation).
Allogeneic transplant (B) is the only curative option but requires disease control first (achieved via chemo+TKI).
Why Not Other Options?

B) Immediate transplant eval: Necessary but not first steppatient needs cytoreduction first.
C) Decitabine + venetoclax: Investigational in CML blast phase (not guideline-recommended).
D) Bosutinib: Ineffective in blast phase without chemotherapy.

Session ID:
Assessment Domain

Session-69:
Rationale

Session ID:
Instructional Design and Pedagogical

Session-69:
The session's instructional design is of very high quality. The case is expertly scaffolded, progressing logically from initial diagnosis and risk-stratified therapy selection to the management of adverse effects, response monitoring, resistance, and finally, transformation to blast phase. The embedded feedback is a major strength; rationales are consistently anchored to specific, current clinical guidelines (ESMO, ELN, NCCN) and clearly explain the reasoning for both the correct answer and the incorrect distractors. The framing of each question is exceptionally clear. The score for learning objectives is slightly reduced only because they are not explicitly stated at the outset, though the educational goals are clearly implied by the session's structure and content.

Session ID:
Clinical Authenticity and Content Fidelity

Session-69:
The clinical authenticity and content fidelity of this session are excellent. The patient case is highly plausible, mirroring a realistic clinical journey for a patient with CML. All therapeutic choices, monitoring milestones (e.g., BCR::ABL1 1% at 3 months), and management decisions (e.g., dose reduction for dasatinib-induced effusion) are perfectly aligned with current, evidence-based guidelines from ELN, ESMO, and NCCN. The pharmacotherapeutic recommendations are optimal and well-justified. Importantly, the session demonstrates perfect domain specificity, correctly applying CML-specific standards of care and appropriately contextualizing therapies from other disease states (e.g., noting AML regimens are investigational in CML blast phase). There are no content errors or instances of domain entanglement.

Session ID:
Modeled Clinical Reasoning

Session-69:
The simulation provides an expert model of the clinical reasoning process in CML management. Each stage of the case presents a well-defined clinical problem with all necessary data for an informed decision. The multiple-choice options offer an excellent breadth of plausible and relevant alternatives, including key distractors that effectively test nuanced clinical judgment (e.g., switching TKIs without mutation testing). The overarching narrative demonstrates flawless prioritization, such as confirming the diagnosis before treatment, managing toxicity conservatively before switching therapy, and securing vital genetic data to guide subsequent decisions. The clinical outcomes described are realistic and instructionally valuable, reinforcing the learning objectives at each step.

Session-70

Session ID:
LLM

Session-70:
DeepSeek V2

Session ID:
Session Transcript

Session-70:
What is the **next critical diagnostic step** to confirm CML?
A) **Bone marrow biopsy with cytogenetics (Ph chromosome)**
B) **JAK2 V617F mutation testing**
C) **Flow cytometry for CD34+ cells**
D) **Serum B12/folate levels**
A
Using the **ELN 2022 risk score**, how would you classify this patient?
Additional Data:
- Spleen: **5 cm below costal margin**
- Blasts: **2% (blood)**
- Eosinophils: **3%**
- Basophils: **5%**
- Platelets: **450 x10/L**

A) **Low risk**
B) **Intermediate risk**
C) **High risk**
B due toBlasts 2% in blood adds points (your patient has 2%)
Basophils 4% adds points (your patient has 5%)
Platelets 450 x10/L adds points (your patient has 450, which is right at the cutoff)
Spleen size 5 cm below costal margin adds points (your patient has 5 cm)
Eosinophils are not a major factor in this scoring

What is the **first-line TKI** for this patient?
A) **Imatinib 400 mg daily**
B) **Nilotinib 300 mg BID**
C) **Dasatinib 100 mg daily**
D) **Ponatinib 45 mg daily**

C
Correct Answer: C) Dasatinib 100 mg daily

Why Dasatinib?
1. **High-Risk Patients**:
   - Per **ESMO 2022 guidelines**, **2nd-generation TKIs (nilotinib/dasatinib)** are preferred over imatinib for high-risk CML due to:
     - Faster **BCR::ABL1 reduction** (earlier molecular responses).
     - Lower risk of **disease progression**.
   - **Dasatinib** has shown superior **progression-free survival** in high-risk patients vs. imatinib.

2. **Dosing**:
   - **100 mg daily** is standard for chronic-phase CML (balances efficacy/toxicity).

3. **Safety Profile**:
   - No comorbidities reported yet, but dasatinibs key risks are:
     - **Pleural effusions** (monitor for dyspnea).
     - **Myelosuppression** (check CBCs monthly).

Why Not Other Options?
- **A) Imatinib**: Less effective in high-risk patients (higher failure rates).
- **B) Nilotinib**: Also appropriate, but dasatinib may be preferred if **CV risk factors** exist (this patients CV history unknown).
- **D) Ponatinib**: Reserved for **T315I mutation** or resistant disease (not 1st-line).

What is the **most likely cause** of dyspnea?
A) **Pleural effusion**
B) **Disease progression to blast crisis**
C) **Anemia (Hb 10.2 g/dL at diagnosis)**
D) **Pulmonary hypertension**

Why A) Pleural Effusion is Correct:
1. **Dasatinib's Signature Toxicity**:
   - Pleural effusions occur in **10-35%** of dasatinib-treated patients (per FDA/ESMO).
   - Mechanism: **PDGFR inhibition vascular leakage**.
   - Risk factors: **Age >50**, high dose (100mg), prolonged use.

2. **Clinical Clues in This Case**:
   - **Timing**: New dyspnea at **3 months** aligns with typical onset.
   - **Supporting Findings**:
     - Concurrent **thrombocytopenia** (platelets 120 x10/L) suggests dasatinib toxicity is manifesting.
     - No fever/infection signs to suggest other causes.

3. **Diagnosis**:
   - **Chest imaging** (X-ray/ultrasound) would confirm fluid.
   - **Management**: Dose reduction (to 80 mg) + diuretics if symptomatic.

Why C) Anemia is Incorrect:
1. **Stable Hemoglobin**:
   - Hb was **10.2 g/dL at diagnosis** **no new drop reported** at 3 months.
   - Dyspnea from anemia typically requires Hb < **8 g/dL** (unless pre-existing cardiopulmonary disease).

2. **Dasatinib Rarely Worsens Anemia**:
   - Unlike imatinib, dasatinib causes **less frequent Grade 3/4 anemia** (<10% of patients).

3. **Anemia Wouldnt Explain Thrombocytopenia**:
   - The **low platelets** point to a **systemic dasatinib effect** (myelosuppression + effusion).

Chest X-ray confirms **moderate bilateral pleural effusions**.
- The patient has **no fever or chest pain** but reports **worsening dyspnea** (Grade 2).

What is the next step per ESMO 2022 guidelines?
A) **Continue dasatinib 100 mg + add furosemide**
B) **Reduce dasatinib to 80 mg + close monitoring**
C) **Switch to nilotinib 300 mg BID**

D) **Pause dasatinib + consider thoracentesis if severe**
Correct Answer: D) Pause dasatinib + consider thoracentesis if severe

Why D is the Best Choice:
1. **ESMO 2022 Guidelines for Grade 2 Pleural Effusion**:
   - **First step**: **Interrupt dasatinib** until symptoms improve (typically 12 weeks).
   - **If effusion is large/symptomatic**: **Thoracentesis** may be needed for relief.
   - **Upon resolution**: Reinitiate at a **lower dose (80 mg daily or 50 mg daily)**.

2. **Why Not Other Options?**
   - **A) Continue full dose + furosemide**: Furosemide alone is insufficient**dose adjustment is mandatory**.
   - **B) Reduce dose without pausing**: Effusion may worsen; **must interrupt first**.
   - **C) Switch to nilotinib**: Premature! **Try dose reduction first** unless effusion is recurrent.

Is this patients response **optimal, warning, or failure** per ELN 2022 criteria?
A) **Optimal**
B) **Warning**
C) **Failure**

Correct Answer: B) Warning

Why "Warning" (Not Optimal or Failure)?
ELN 2022 6-Month Benchmarks:
- **Optimal**: BCR::ABL1 **0.1% IS** (MMR achieved).
- **Warning**: BCR::ABL1 **>0.11% IS** (this patient: **0.8% IS**).
- **Failure**: BCR::ABL1 **>1% IS** OR loss of response.

Implications:
- **Action Required**:
  - **Check adherence** (common cause of suboptimal response).
  - **Repeat PCR in 3 months** (delayed MMR may still occur).
  - **Consider mutation testing** if no improvement.

Why Not Optimal (A)?
- MMR (0.1% IS) is the **6-month goal** for high-risk patients on 2nd-gen TKIs.

Why Not Failure (C)?
- **Failure** requires **>1% IS** or progressive features (e.g., rising counts, new mutations).

What is the **next step**?
A) **Continue dasatinib 80 mg + monitor ECG**
B) **Switch to bosutinib 400 mg daily**
C) **Reduce dose to 50 mg daily + correct electrolytes**
D) **Discontinue TKI permanently**

Option D) "Temporary hold + diuretics, then resume 80 mg" is indeed appropriate for:
- **First recurrence** of effusion after initial dose reduction
- **Mild-to-moderate** (Grade 2) effusions that resolve quickly
- Patients who previously tolerated the reduced dose well

Option A) "Switch to bosutinib" becomes preferred when:
- Effusions recur **after already being on a reduced dose** (80 mg)

- There's **slow resolution** or need for repeated interventions
- Patient develops **other dasatinib toxicities** (e.g., QTc prolongation)

Why This Case Favors (A) Switch
1. **Recurrent Effusion on Reduced Dose**:
   - The patient already failed 100 mg reduced to 80 mg effusion recurred
   - Suggests ongoing sensitivity even at lower doses

2. **ELN 2022 Guidance**:
   - Recommends **permanent switch** after recurrent Grade 2 toxicity post-dose reduction
   - Particularly when good alternatives exist (bosutinib has <1% effusion risk)

3. **Practical Considerations**:
   - Repeated holds disrupt therapeutic efficacy
   - MR4.5 goal requires consistent TKI exposure

When (D) Would Be Correct
If this were the **first recurrence** after initial dose reduction (rather than second episode), holding and rechallenging at same dose would be standard.

Question 8 (Treatment-Free Remission Eligibility)
Patient Profile:
- **High-risk CML**, now in **MR4.5 (BCR::ABL1 0.0032% IS)** for **6 months**.
- **Total TKI duration**: 24 months (dasatinib bosutinib).
- No prior resistance, excellent adherence.

Per ELN 2022, is this patient a candidate for TKI discontinuation?
A) **Yes, eligible now**
B) **Norequires 2 years of sustained MR4.5**
C) **Nohigh-risk patients are ineligible**
D) **Yes, but only in clinical trials**

Why B is Correct:
1. **ELN 2022 Discontinuation Criteria**:
   - **MR4.5 (0.0032% IS) must be sustained for 2 years** (this patient: only 6 months).
   - **Total TKI duration 3 years** preferred (this patient: 24 months).

2. **High-Risk Patients**:
   - **Not excluded**, but require stricter milestones (longer MR4.5 duration).
   - Must have **no history of resistance/rescue therapy**.

3. **Why Not Other Options?**
   - **A) Yes now**: Incorrectpremature without 2 years of MR4.5.
   - **C) Ineligible due to high risk**: Falserisk status doesnt prohibit discontinuation.
   - **D) Only in trials**: Optional but not requiredoff-trial discontinuation is acceptable if criteria met.

Question 9 (Advanced Monitoring)
A patient on **nilotinib** achieves **stable MR4.5** but has **asymptomatic hyperlipasemia (Grade 2)**. What is the **next step**?
A) **Continue nilotinib + monitor lipids/pancreatic enzymes**
B) **Switch to bosutinib immediately**
C) **Permanently discontinue TKI**
D) **Reduce nilotinib dose by 50%**

Your Answer: 9-A) Continue nilotinib + monitor enzymes monthly

Correct!

Guideline Rationale (ESMO 2022):
- **Grade 2 (asymptomatic) lab abnormalities** do not require dose modification.
- **Action**: Monitor lipase/amylase **every 24 weeks**; hold only if:
  - Symptoms develop (e.g., pancreatitis: severe pain, vomiting).
  - Elevations persist >**Grade 3** (>5x ULN).

Why Not Other Options?
- **B) Switch to bosutinib**: Overly aggressive for asymptomatic findings.
- **C) Hold nilotinib**: Unnecessary without symptoms.
- **D) Dose reduction**: Premature; may compromise efficacy.

A **young woman in MMR on dasatinib** desires pregnancy. Her oncologist recommends:
A) **Continue dasatinib with fetal ultrasound monitoring**
B) **Switch to pegylated interferon-**
C) **Hold TKIs until postpartum (if MMR sustained >2 years)**
D) **Switch to imatinib (lowest-risk TKI)**

Your Answer: 10-B) Switch to pegylated interferon-
Partially Correct, but Nuanced!

ESMO/ELN 2023 Guidelines:
- **Interferon- (B)** is **safe in pregnancy** but has **lower efficacy** (only 3050% maintain MMR).
- **Preferred Approach**:
  - **If MMR sustained >2 years**: **Hold TKIs (C)** and monitor closely (50% remain treatment-free postpartum).
  - **If MMR <2 years**: **Interferon- (B)** is second-line (after TKI hold).

Critical Caveats:
- **Imatinib (D)**: *Not safe* (despite older studies; now Category D).
- **Dasatinib (A)**: Absolute contraindication (risk of fetal bleeding/organ malformations).

Clinical Pearl:
- **Interferon- is a fallback**, but **TKI interruption (C)** is preferred if MMR is durable.

Session ID:
Assessment Domain

Session-70:
Rationale

Session ID:
Instructional Design and Pedagogical

Session-70:
The session's instructional design is of very high quality. While learning objectives are not explicitly stated at the outset, they are clearly implied by the session's structure, which logically covers the entire patient journey from diagnosis to long-term management. The case is expertly scaffolded, moving from initial workup and risk stratification to first-line therapy selection, management of common and recurrent toxicities, response assessment, and complex decisions around treatment-free remission and pregnancy. The embedded feedback is a major strength, providing clear, guideline-anchored rationales (citing ESMO/ELN) that explain not only why the correct answer is right but also why the distractors are wrong. All instructions and questions are exceptionally clear and unambiguous.

Session ID:

Clinical Authenticity and Content Fidelity

Session-70:

The clinical fidelity of this session is excellent and free of significant errors.Plausibility & Guideline Alignment: The clinical narrative is highly realistic and mirrors an authentic CML case progression. All diagnostic steps, therapeutic choices, and management decisions are perfectly aligned with current, evidence-based guidelines (e.g., ELN, ESMO). This includes nuanced topics like choosing a second-generation TKI for a higher-risk patient, the step-wise management of recurrent dasatinib-induced pleural effusion, and the strict criteria for attempting treatment-free remission.

Session ID:

Modeled Clinical Reasoning

Session-70:

The simulation expertly models a high-level clinical reasoning process. Each stage of the case frames a clear, relevant, and well-defined clinical problem. The multiple-choice options are exceptionally well-crafted, offering a range of plausible alternatives that include key distractors, thereby testing for nuanced clinical knowledge. The decision-making process modeled in the narrative and rationales is sound, demonstrating appropriate prioritization (e.g., confirming diagnosis before treatment, managing toxicity before assessing response). The clinical outcomes presented after each decision (e.g., development of pleural effusion, achieving a "warning" response) are realistic, specific, and highly instructive, effectively reinforcing the key learning points of each section.

Session-71

Session ID:
LLM

Session-71:
DeepSeek V2

Session ID:
Session Transcript

Session-71:
Phase 1: Initial Presentation and Diagnostic Workup

Case Introduction
Patient: 48-year-old male
Chief Complaint: Fatigue, early satiety, and unexplained weight loss over 2 months
Physical Exam:
- Splenomegaly (palpable 6 cm below costal margin)
- No lymphadenopathy or hepatomegaly
Initial Labs:
- WBC: 65 x 10/L (neutrophilia, left shift, basophilia)
- Hb: 10.2 g/dL
- Platelets: 520 x 10/L
- LDH: 2x ULN

Question 1 (Diagnostic Framework)
What is the most critical initial test to confirm suspected CML?
A) Peripheral blood smear
B) Bone marrow biopsy with cytogenetics (Ph chromosome/karyotyping)
C) JAK2 V617F mutation assay
D) Flow cytometry for CD34+ cells

Select your answer (A, B, C, or D).
(Type "HINT" if needed.)

Notes for Feedback:
- Ill provide **detailed rationale** for correct/incorrect choices after your answer.
- This question assesses **WHO diagnostic criteria** (B is correct; Ph chromosome/BCR::ABL1 is pathognomonic).
- If incorrect, Ill clarify why alternatives (e.g., JAK2 for MPN) are inappropriate.

(Progression: Next question will adapt based on your response.)

hint

Hint for Question 1 (Diagnostic Workup in CML):

- The **definitive diagnosis of CML** requires identification of the **Philadelphia chromosome (t(9;22)) or BCR::ABL1 fusion gene**

(per WHO 2016/2022 and ELN 2022 guidelines).
- While a peripheral blood smear (A) may show leukocytosis with immature granulocytes (a clue), it is **not diagnostic**.
- **JAK2 V617F (C)** is used for **myeloproliferative neoplasms (MPN)** like polycythemia vera, not CML.
- Flow cytometry (D) is more relevant for **acute leukemias** or minimal residual disease (MRD) monitoring.

Key Question to Ask Yourself:
"Which test directly confirms the molecular/cytogenetic hallmark of CML?"

Relevant Guidelines:
- **ESMO 2022:** Diagnosis requires demonstration of BCR::ABL1 by cytogenetics, FISH, or PCR.
- **ELN 2022:** Bone marrow biopsy with karyotyping is preferred initially for baseline assessment.

Now, select your answer (A, B, C, or D).
(Pro tip: Think "pathognomonic test" for CML.)
B
Feedback on Question 1:

Correct Answer: B) Bone marrow biopsy with cytogenetics (Ph chromosome/karyotyping)

Rationale:
- The **Philadelphia chromosome (t(9;22)) or BCR::ABL1 fusion gene** is **pathognomonic for CML** (WHO 2016/2022).
- Bone marrow biopsy provides **simultaneous assessment of:**
  - **Cytogenetics (karyotyping)** for Ph chromosome.
  - **Histopathology** (e.g., myeloid hyperplasia, blast count).
  - **Baseline for future MRD monitoring** (ELN 2022).

Why Not Other Options?
- **A) Peripheral smear:** Shows leukocytosis with immature granulocytes but cannot confirm CML.
- **C) JAK2 V617F:** Rules in **other MPNs** (e.g., polycythemia vera), not CML.
- **D) Flow cytometry:** Used for immunophenotyping **acute leukemias**, not chronic-phase CML.

Phase 1 (Continued): Additional Workup Results
Cytogenetics: t(9;22)(q34;q11) (Ph chromosome positive)
Molecular Testing: BCR::ABL1 (p210 transcript) detected by PCR.

Question 2 (Risk Stratification)
Which tool is used to stratify this patients risk of disease progression?
A) Sokal score
B) IPSS (International Prognostic Scoring System)
C) ECOG Performance Status
D) CHADS-VASc score

Submit your answer (A, B, C, or D).
(Type "HINT" if needed.)

Note: Your correct answer will trigger a **complication** (e.g., renal dysfunction) to challenge your next decision!
hint please
Heres a **targeted hint** to guide your risk stratification decision:

Hint for Question 2 (Risk Stratification)
- **Focus on CML-specific tools**: Risk scores like **Sokal**, **Euro**, or **EUTOS** are validated for predicting outcomes in CML (not generic oncology tools).
  - **Sokal score (A)**: Uses age, spleen size, platelet count, and blast percentage to categorize risk (*high/intermediate/low*).
  - **Euro score**: Similar to Sokal but optimized for imatinib-era patients.
  - **EUTOS**: Incorporates basophil percentage and spleen size.
- **Why Not Other Options?**
  - **B) IPSS**: Used for *myelodysplastic syndromes (MDS)*, not CML.
  - **C) ECOG**: Measures *performance status* but doesnt predict CML progression.
  - **D) CHADS-VASc**: Assesses *stroke risk in atrial fibrillation*.

Guideline Reference:
> *"Sokal or Euro scores should be calculated at diagnosis to guide therapeutic discussions."*
> *Hochhaus et al., ELN 2022*

Re-evaluate the options with this context.
Submit your answer (A, B, C, or D).
(Key question: Which tool is uniquely designed for CML prognosis?)
B
Feedback on Question 2
Your Answer: B) IPSS (International Prognostic Scoring System)

Correct Answer: A) Sokal score

Why Sokal Score?
- The **Sokal score** (and its variants like Euro or EUTOS) is the **standard risk stratification tool for CML** at diagnosis (*Hochhaus et al., ELN 2022*).
- It incorporates:
  - **Age**
  - **Spleen size** (this patient has splenomegaly)
  - **Platelet count**
  - **Blast percentage** in peripheral blood or bone marrow
- Output: **Low, Intermediate, or High risk**guides first-line therapy selection (e.g., high-risk patients may need more potent TKIs).

Why Not IPSS?
- **IPSS (B)** is used for **myelodysplastic syndromes (MDS)**, not CML.
- **ECOG (C)** assesses general functional status but doesnt predict CML progression.
- **CHADS-VASc (D)** evaluates stroke risk in atrial fibrillation.

Phase 1 Continues: Risk Calculation & Complication
Sokal Score Calculation:
- Inputting this patients data (age 52, spleen size, platelets 450,000/L, blasts 2%) yields **Intermediate Risk**.

New Complication: The patients **creatinine clearance (CrCl) is 38 mL/min** (moderate renal impairment).

Question 3 (First-Line Therapy Selection)**
Which TKI is contraindicated in this patient due to renal dysfunction?

A) Imatinib
B) Dasatinib
C) Nilotinib
D) Bosutinib

Submit your answer (A, B, C, or D).
(Type "HINT" if needed.)

Key Considerations:
- Renal function impacts TKI pharmacokinetics.
- Refer to **ESMO guidelines (2022)** for dose adjustments/contraindications.
- Your answer will determine the next case complexity!
hint
Heres a **targeted hint** to guide your TKI selection:

Hint for Question 3 (First-Line Therapy & Renal Dysfunction)
- **Renally cleared TKIs**: Some TKIs require dose adjustment or are contraindicated in moderate-severe renal impairment.
  - **Nilotinib (C)**: *Contraindicated in CrCl <30 mL/min* (ESMO 2022). While this patients CrCl is 38 mL/min, nilotinib carries increased risk of QT prolongation and vascular events in renal dysfunction.
  - **Dasatinib (B)**: Requires dose reduction for CrCl <50 mL/min.
  - **Bosutinib (D)**: Avoid if CrCl <30 mL/min.
  - **Imatinib (A)**: Safest in renal impairment (dose adjustment only if CrCl <30 mL/min).

Guideline Reference:
> *"Avoid nilotinib in patients with significant renal dysfunction; prefer imatinib or dasatinib with dose monitoring."*
> *ESMO CML Guidelines (2022)*

Re-evaluate the options considering:
1. **Absolute contraindications** (not just dose adjustments).
2. **Patients CrCl (38 mL/min)** falls in the *moderate impairment* range.

Submit your answer (A, B, C, or D).
(Think: Which TKI has the strongest warning against use in this setting?)
C
Feedback on Question 3
Your Answer: C) Nilotinib
Correct Answer: C) Nilotinib

Why Nilotinib is Contraindicated
- **Pharmacokinetics**: Nilotinib is **not recommended** for patients with **CrCl <30 mL/min** (per prescribing guidelines and ESMO 2022).
- **Safety Concerns**:
  - Increased risk of **QT prolongation** and **vascular events** (e.g., peripheral arterial disease) in renal impairment.
  - This patients **CrCl (38 mL/min)** is close to the threshold, making **nilotinib a high-risk choice**.

Alternatives in Renal Dysfunction
- **Imatinib (A)**: Safest option (dose adjustment only if CrCl <30 mL/min).
- **Dasatinib (B)**: Requires **dose reduction** (e.g., 70 mg 50 mg daily) for CrCl <50 mL/min.
- **Bosutinib (D)**: Avoid if CrCl <30 mL/min (but less data in moderate impairment).

Guideline Reference:
> *"Nilotinib should be avoided in patients with significant renal dysfunction; imatinib is preferred in this population."*
> *Hochhaus et al., ELN 2022*

**

Phase 2: Treatment Initiation & Supportive Care**
Selected Therapy: The patient starts **imatinib 400 mg daily** (renal-adjusted dose).

New Issue: On day 10, he develops **fever (38.5C) and neutropenia (ANC 500/L)**.

Question 4 (Febrile Neutropenia Management)**
What is the next critical step?
A) Continue imatinib and monitor for 24 hours.
B) Hold imatinib, start broad-spectrum IV antibiotics (e.g., piperacillin-tazobactam), and hospitalize.
C) Add G-CSF (filgrastim) but continue imatinib.
D) Switch to dasatinib due to suspected imatinib intolerance.

Submit your answer (A, B, C, or D).
(Type "HINT" if needed.)

Key Points:
- **Febrile neutropenia in CML** requires immediate action (risk of sepsis).
- Consider **IDSA 2022 febrile neutropenia guidelines**.
- Your answer determines the next clinical challenge!
B
Feedback on Question 4
Your Answer: B) Hold imatinib, start broad-spectrum IV antibiotics (e.g., piperacillin-tazobactam), and hospitalize.
Correct Answer: B) Hold imatinib, start broad-spectrum IV antibiotics (e.g., piperacillin-tazobactam), and hospitalize.

Why This is the Correct Approach
1. **Febrile Neutropenia is an Oncologic Emergency**
   - ANC <500/L + fever 38.3C requires **immediate IV antibiotics** (per IDSA 2022 guidelines).
   - **Piperacillin-tazobactam** (or meropenem in high-risk cases) covers *Pseudomonas* and other Gram-negative bacilli.

2. **Hold Imatinib Temporarily**
   - TKIs can worsen myelosuppression. **Imatinib should be paused** until infection resolves and ANC recovers (>1000/L).

3. **Hospitalization is Mandatory**
   - Febrile neutropenia has a **10-20% mortality risk** if untreated; inpatient management ensures rapid response to sepsis.

Why Not Other Options?
- **A) Monitor without treatment**: Unacceptable delay in life-threatening infection.
- **C) G-CSF alone**: Adjunctive but **does not replace antibiotics**. G-CSF may be added if prolonged neutropenia is expected.
- **D) Switch TKIs**: Prematurefever is due to **neutropenia/infection**, not imatinib intolerance.

Guideline Reference:
> *"Febrile neutropenia requires immediate empiric antibiotics and hospitalization. TKIs should be withheld until recovery."*
> *IDSA Febrile Neutropenia Guidelines (2022)*

**

Phase 2 Continues: Supportive Care & Complications**
Outcome: The patient responds to antibiotics, ANC recovers to 1500/L after 5 days. Imatinib is restarted at **300 mg daily** (reduced due to prior neutropenia).

New Challenge: At 3-month follow-up, his **BCR::ABL1 PCR is 8.5% (IS)**indicating **warning response** per ELN 2022 criteria.

Question 5 (Treatment Failure Management)**
What is the next best step?
A) Continue imatinib and recheck PCR in 3 months.
B) Switch to a 2nd-generation TKI (e.g., dasatinib or nilotinib).
C) Increase imatinib dose to 600 mg daily.
D) Refer for allogeneic stem cell transplant.

Submit your answer (A, B, C, or D).
(Type "HINT" if needed.)

Key Considerations:
- **ELN 2022 defines "warning"** as BCR::ABL1 1-10% at 3 months.
- **Optimal response** at 3 months is BCR::ABL1 1%.
- **Patient-specific factors**: Renal impairment still present (CrCl 40 mL/min).

Your answer will shape long-term outcomes!

hint
Heres a **targeted hint** to guide your decision on managing a **warning response** at 3 months:

Hint for Question 5 (Treatment Failure Management)
- **ELN 2022 "Warning" Criteria**:
  - **BCR::ABL1 1-10% at 3 months** signals suboptimal response.
  - Next steps depend on **adherence, toxicity, and resistance testing**.

- **Key Considerations**:
  1. **Adherence First**: Confirm the patient is taking imatinib correctly (e.g., drug levels, pill counts).
  2. **Resistance Testing**: If adherent, **BCR::ABL1 mutation analysis** (e.g., T315I, F317L) should guide next TKI choice.
  3. **2nd-Generation TKIs (B)**: Dasatinib/nilotinib are preferred over dose escalation (C) for *warning responses* (higher efficacy).
  4. **Transplant (D)**: Not yet indicatedonly for **failure of 2 TKIs** or T315I mutation.

Guideline Reference:
> *"For warning responses at 3 months, optimize adherence, assess resistance, and consider switching to a 2nd-generation TKI."*
> *Hochhaus et al., ELN 2022*

Re-evaluate the options considering:
- **This patients renal function** (CrCl 40 mL/min) may favor **dasatinib** over nilotinib.

- **Dose escalation (C)** is less effective than switching TKIs for suboptimal response.

Submit your answer (A, B, C, or D).
(Think: What does ELN recommend for warning responses?)
B
Feedback on Question 5
Your Answer: B) Switch to a 2nd-generation TKI (e.g., dasatinib or nilotinib).
Correct Answer: B) Switch to a 2nd-generation TKI (e.g., dasatinib or nilotinib).

Why This is the Correct Approach
1. **ELN 2022 Guidelines for "Warning" Response**
   - **BCR::ABL1 8.5% at 3 months** indicates **suboptimal response**, necessitating **early intervention** to prevent progression.
   - **Preferred action**: Switch to a **2nd-generation TKI** (dasatinib, nilotinib, or bosutinib) for better efficacy (*Hochhaus et al., Blood 2022*).

2. **Why Not Other Options?**
   - **A) Continue imatinib**: High risk of **eventual treatment failure** (40-50% risk of progression if BCR::ABL1 >10% by 6 months).
   - **C) Dose escalation**: Less effective than switching TKIs (only ~20% achieve major molecular response with imatinib dose increase).
   - **D) Transplant**: Prematurereserved for **TKI failure or T315I mutation**.

3. **Patient-Specific Considerations**
   - **Renal impairment (CrCl 40 mL/min)**:
     - **Dasatinib** (preferred): Requires dose reduction (50 mg daily).
     - **Nilotinib**: Higher risk of vascular events; avoid if possible.

Guideline Reference:
> *"For warning responses at 3 months, switch to a 2nd-generation TKI after confirming adherence and excluding resistance mutations."*
> *ELN 2022 Recommendations*

Phase 3: Long-Term Management & Monitoring**
Action Taken: Patient switches to **dasatinib 50 mg daily** (renal-adjusted).

Follow-Up:
- **6 months**: BCR::ABL1 **0.2% (IS)** (major molecular response, MMR).
- **12 months**: BCR::ABL1 **0.01% (IS)** (MR4.5).

Question 6 (MRD Monitoring & TKI Discontinuation)**
According to ELN 2022, when is TKI discontinuation (treatment-free remission attempt) appropriate?
A) After 1 year of stable MMR (BCR::ABL1 0.1%).
B) After 3 years of stable **deep molecular response (MR4.5, BCR::ABL1 0.0032%)**.
C) Only if the patient develops TKI intolerance.
D) Neverlifelong TKI therapy is required.

Submit your answer (A, B, C, or D).
(Type "HINT" if needed.)

Key Points:
- **Treatment-free remission (TFR)** is a **key goal** in CML but requires strict eligibility criteria.
- **MRD monitoring frequency** increases after discontinuation (monthly x 1 year).
- Your answer determines whether this patient can **safely stop therapy**!
B
Feedback on Question 6
Your Answer: B) After 3 years of stable deep molecular response (MR4.5, BCR::ABL1 0.0032%).
Correct Answer: B) After 3 years of stable deep molecular response (MR4.5, BCR::ABL1 0.0032%).

Why This is the Correct Approach
1. **ELN 2022 TKI Discontinuation Criteria**
   - **Prerequisites for TFR (Treatment-Free Remission):**
     - **MR4.5 (BCR::ABL1 0.0032%) maintained for 3 years** on TKI therapy.
     - **No prior resistance** to TKIs.
     - **Access to reliable PCR monitoring** (monthly for 1 year post-discontinuation).

2. **Why Not Other Options?**
   - **A) 1 year of MMR**: Insufficientrequires **deeper response (MR4.5)** and longer duration.
   - **C) Intolerance only**: TFR is also possible for **optimal responders** meeting molecular criteria.
   - **D) Lifelong therapy**: Outdated~40% of eligible patients maintain remission after stopping TKIs (*STIM trial, Lancet Oncol 2017*).

3. **This Patients Eligibility**
   - Currently at **MR4.5 (0.01%)** at 12 monthsneeds **2 more years of sustained MR4.5** before discontinuation attempt.

Guideline Reference:
> *"TKI discontinuation may be attempted in patients with sustained MR4.5 for 3 years, following rigorous molecular monitoring."*
> *Hochhaus et al., ELN 2022*

Phase 3 Continues: TKI Discontinuation & Relapse
Action Taken: After **3 years of sustained MR4.5**, dasatinib is stopped under close monitoring.

New Challenge: At **6 months off therapy**, his BCR::ABL1 rises to **0.06% (IS)** (lost MMR).

Question 7 (Relapse Management)**
What is the next step per ELN 2022?
A) Restart the same TKI (dasatinib) immediately.
B) Switch to ponatinib due to suspected resistance.
C) Observe until BCR::ABL1 exceeds 0.1%.
D) Refer for allogeneic stem cell transplant.

Submit your answer (A, B, C, or D).
(Type "HINT" if needed.)

Key Considerations:
- **ELN defines relapse** as loss of MMR (BCR::ABL1 >0.1%).
- **Early intervention** (at 0.06%) may improve re-achievement of deep response.
- **Resistance testing** is critical if restarting therapy.

Your answer impacts long-term disease control!
hint
Heres a **targeted hint** to guide your decision on managing molecular relapse after TKI discontinuation:

Hint for Question 7 (Relapse Management)
- **ELN 2022 TFR Monitoring Guidelines**:
  - **Molecular relapse** is defined as either:
    1) Loss of MMR (BCR::ABL1 >0.1% IS) *or*
    2) Two consecutive rises in BCR::ABL1 with the second >0.01%
  - **Action threshold**: Most experts recommend restarting TKI when BCR::ABL1 reaches >0.1% (MMR loss)

- **Key Considerations**:
  1. **Restarting original TKI (A)**: >90% of patients regain deep response when restarting the same TKI (no resistance assumed yet)
  2. **Early intervention (at 0.06%)**: Some centers may restart earlier, but standard practice waits for confirmed MMR loss
  3. **Resistance testing**: Only needed if patient fails to respond after restarting therapy
  4. **Ponatinib/transplant (B/D)**: Only for confirmed resistance mutations (e.g., T315I)

Guideline Reference:
> "For patients who lose MMR after discontinuation, restart the original TKI promptly. Resistance testing is only indicated if response is inadequate after restarting."
> *ELN 2022 Recommendations*

Re-evaluate the options considering:
- This patient has **early molecular rise (0.06%)** but hasn't yet lost MMR
- **Standard practice** is to restart at MMR loss (>0.1%), though some protocols act earlier
- No evidence yet of resistance needing ponatinib/transplant

Submit your answer (A, B, C, or D).
(Think: At what threshold does ELN recommend restarting therapy? Should the same TKI be used initially?)
A
Feedback on Question 7
Your Answer: A) Restart the same TKI (dasatinib) immediately.
Correct Answer: A) Restart the same TKI (dasatinib) immediately.

Why This is the Correct Approach
1. **ELN 2022 Relapse Management Guidelines**
   - For patients who lose **MMR (BCR::ABL1 >0.1%)** after TKI discontinuation, **immediate restart of the original TKI** is recommended.
   - **Rationale**: >90% of patients regain deep molecular response when re-treated with the same TKI (*STIM trial, Lancet Oncol 2017*).

2. **Why Not Other Options?**
   - **B) Ponatinib**: Only indicated if **T315I mutation** is detected (not yet tested here).
   - **C) Observe**: Risk of **progression** if therapy is delayed after MMR loss.
   - **D) Transplant**: Overly aggressivefirst-line approach is **TKI reinitiation**.

3. **This Patients Status**
   - Current BCR::ABL1 **0.06%** (approaching but not yet at MMR loss threshold of 0.1%).
   - Some protocols recommend restarting at **two consecutive rises >0.01%**, but ELN consensus is to wait for **confirmed >0.1%**.

Guideline Reference:
> *"Reinitiate the original TKI promptly upon loss of MMR after discontinuation. Resistance testing is only needed if response is inadequate upon restarting."*
> *Hochhaus et al., ELN 2022*

Phase 3 Conclusion: Long-Term Outcome
Action Taken: Dasatinib 50 mg daily was restarted when BCR::ABL1 reached **0.12%** (confirmed MMR loss).
- **Response**: MMR regained within 3 months; MR4.5 achieved by 12 months.
- **Current Status**: Patient continues dasatinib with quarterly PCR monitoring.

Summative Assessment
Woroud Ahmed, youve successfully navigated this complex CML case!

Strengths Demonstrated:
Diagnostic accuracy: Correctly identified the need for **cytogenetics/molecular testing** for CML diagnosis.
Risk stratification: Applied **Sokal score** appropriately.
Therapeutic decision-making:
- Recognized **nilotinib contraindication** in renal impairment.
- Managed **febrile neutropenia** per IDSA guidelines.
- Selected **2nd-generation TKI** for suboptimal response.
TFR principles: Understood **discontinuation criteria** (MR4.5 for 3 years).

Areas for Growth:
Early intervention thresholds: Consider that some centers restart TKIs at **two consecutive BCR::ABL1 rises >0.01%** (not just MMR loss).
Mutation testing: Remember to order **BCR::ABL1 mutation analysis** if response to restarting TKI is inadequate.

Key Takeaways:
1. **First-line TKI selection** depends on **risk score, comorbidities, and mutational status**.
2. **Molecular monitoring** (qPCR every 3 months) guides all therapeutic decisions.
3. **TFR is possible** but requires strict eligibility and monitoring.

Additional Resources
1. [ELN 2022 CML Guidelines](https://ashpublications.org/blood/article/140/12/1345/485844/European-LeukemiaNet-2022-recommendations-for)
2. [NCCN CML Guidelines (2023)](https://www.nccn.org/guidelines/guidelines-detail?category=1&id=1471)
3. [TKI Discontinuation Clinical Trials](https://www.ncbi.nlm.nih.gov/pmc/articles/PMC6986964/)

Type "END" to conclude the session or ask any final questions about CML management!

Session ID:
Assessment Domain

Session-71:
Rationale

Session ID:

Instructional Design and Pedagogical

Session-71:

The session's instructional design is of very high quality. The case is expertly scaffolded, progressing logically from initial diagnosis and risk stratification through first-line therapy, management of an acute complication (febrile neutropenia), management of a suboptimal response, and finally long-term goals like treatment-free remission (TFR) and relapse. The quality of the embedded feedback is a major strength; each rationale is detailed, cites current clinical guidelines (ELN 2022, IDSA 2022), and explains not only why the correct answer is right but also why the distractors are wrong. The use of a "hint" function provides effective, targeted guidance without giving away the answer. The only minor weakness is the lack of explicitly stated, measurable learning objectives at the start of the session, though the educational goals are clearly implied by the robust structure.

Session ID:

Clinical Authenticity and Content Fidelity

Session-71:

The clinical fidelity of this session is outstanding and contains no identifiable errors. Plausibility & Guideline Alignment: The clinical narrative is highly plausible, mirroring a typical Chronic Myeloid Leukemia (CML) case from presentation to long-term follow-up. All diagnostic steps, risk stratification tools (Sokal score), therapeutic decisions, and monitoring milestones are perfectly aligned with current, evidence-based guidelines, specifically referencing ELN 2022 recommendations. Pharmacotherapeutic Accuracy: The pharmacotherapeutic choices and rationales are flawless. The simulation correctly identifies nilotinib's contraindication in renal dysfunction, models the appropriate management of febrile neutropenia (holding TKI, starting broad-spectrum antibiotics), and accurately reflects the guideline-directed switch to a second-generation TKI for a "warning" response. Domain Specificity (Absence of Entanglement): The session demonstrates perfect domain specificity. It actively tests for and corrects potential domain entanglement by including distractors from other disease states (e.g., IPSS for MDS, JAK2 for other MPNs) and clearly explaining why they are inappropriate for CML. The content remains strictly and accurately within the CML domain.

Session ID:

Modeled Clinical Reasoning

Session-71:

The simulation expertly models a high-level clinical reasoning process. Each stage of the case presents a well-defined clinical problem with the necessary data for a decision (e.g., using CrCl to modify TKI selection). The multiple-choice options provide an excellent breadth of plausible and relevant alternatives, including common and subtle distractors that effectively test nuanced understanding. The narrative demonstrates appropriate prioritization throughout, such as treating febrile neutropenia as an emergency and intervening promptly for a suboptimal molecular response. The clinical outcomes described for each decision are realistic, specific, and instructionally valuable, reinforcing the learning from each choice. The reasoning strategy modeled is sound and reflects expert practice.

Session-72

Session ID:
LLM

Session-72:
DeepSeek V2

Session ID:
Session Transcript

Session-72:
Phase 1: Initial Presentation & Diagnostic Workup
Case Introduction
Patient:58-year-old male
Chief Complaint:Fatigue, early satiety, and unexplained weight loss over 3 months.
Physical Exam:
- Splenomegaly (palpable 6 cm below costal margin)
- No lymphadenopathy or bleeding manifestations
Labs:
- WBC: 68 x 10/L (neutrophils 60%, basophils 8%, blasts 2%)
- Hb: 9.8 g/dL
- Platelets: 450 x 10/L
- LDH: 2x ULN
Question 1 (Diagnosis)
What is the next critical diagnostic step to confirm CML?
A) Peripheral blood flow cytometry
B) Bone marrow biopsy with cytogenetics (karyotyping) and PCR for BCR::ABL1
C) JAK2V617F mutation testing
D) Serum protein electrophoresis
Type your answer (A, B, C, or D).
Answer: B) Bone marrow biopsy with cytogenetics (karyotyping) and PCR for BCR::ABL1
Feedback on Question 1
Correct Answer: B) Bone marrow biopsy with cytogenetics (karyotyping) and PCR for BCR::ABL1
Why This Is Correct:
- The gold standard for CML diagnosis requires:
  1. Karyotyping: To detect the Philadelphia chromosome (t(9;22)(q34;q11)).
  2. PCR for BCR::ABL1: To confirm the molecular fusion gene (quantitative PCR is also critical for baseline monitoring).
- ESMO/ELN guidelines mandate these tests for definitive diagnosis and risk stratification (Hochhaus et al., Leukemia 2022).
Why Other Options Are Incorrect:
- A) Peripheral blood flow cytometry: Useful for acute leukemia immunophenotyping but cannot confirm CML.
- C) JAK2 V617F testing: Indicated for myeloproliferative neoplasms (e.g., polycythemia vera), not CML.
- D) Serum protein electrophoresis: Screens for plasma cell disorders (e.g., myeloma), irrelevant here.
Additional Context for This Patient
- The basophilia (8%) and splenomegaly are classic for chronic-phase CML.
- Next steps after confirmation:
  - ELN 2022 risk stratification(Sokal/EUTOS score).
  - Baseline ECOG performance status and comorbidity assessment (e.g., cardiovascular risk for TKI selection).
Question 2 (Risk Stratification)
This patients Sokal score calculates as intermediate risk. Which first-line TKI is *least appropriate?
A) Imatinib 400 mg daily
B) Nilotinib 300 mg BID
C) Dasatinib 100 mg daily

D) Bosutinib 400 mg daily
Type your answer (A, B, C, or D).
Answer: A) Imatinib 400 mg daily
Rationale:
In a newly diagnosed chronic phase CML patient with intermediate Sokal risk, the preferred first-line treatment is a second-generation TKI (2G-TKI) such as nilotinib, dasatinib, or bosutinib because they offer:
Faster and deeper molecular responses
Lower rates of progression to accelerated/blast phase compared to imatinib
Better outcomes in intermediate- and high-risk patients
Imatinib, though still an approved option, is considered less optimal in this risk group per ESMO and NCCN guidelines, especially when 2G-TKIs are available and tolerable.
Feedback on Question 2
Correct Answer: A) Imatinib 400 mg daily
Why This Is Correct:
- ESMO 2023 guidelines recommend second-generation TKIs (2G-TKIs: nilotinib, dasatinib, bosutinib)as preferred first-line therapy for intermediate-/high-risk CML due to:
  - Faster/deeper molecular responses(e.g., higher rates of MMR by 12 months).
  - Lower risk of progression to accelerated/blast phase (Hochhaus et al., Ann Oncol 2023).
- Imatinib is acceptable but suboptimal for intermediate-risk patients when 2G-TKIs are accessible and tolerated.
Why Other Options Are Appropriate:
B) Nilotinib 300 mg BID: Proven superior to imatinib in ENESTnd trial (higher MMR rates).
C) Dasatinib 100 mg daily: Demonstrated efficacy in DASISION trial (faster cytogenetic responses).
D) Bosutinib 400 mg daily: Non-cross-resistant to other TKIs (BFORE trial data).
Key Considerations for This Patient
-Cardiovascular risk: Nilotinib may worsen hyperglycemia/dyslipidemia; avoid if uncontrolled diabetes.
-Pulmonary comorbidities: Dasatinib carries pleural effusion risk.
- Adherence: 2G-TKIs require stricter adherence due to shorter half-lives.
Question 3 (Adverse Effect Management)
The patient starts nilotinib 300 mg BID. At the 3-month follow-up, he reports pruritus and rash. Labs show:
- Total bilirubin: 2.1 mg/dL (indirect 1.8 mg/dL)
- ALT/AST: Normal
What is the most likely cause?
A) Hepatocellular injury
B)Gilberts syndrome exacerbation (UGT1A1 polymorphism)
C)Biliary obstruction
D)TKI-induced indirect hyperbilirubinemia
Type your answer (A, B, C, or D).
Answer: D) TKI-induced indirect hyperbilirubinemia
Feedback on Question 3
Correct Answer: D) TKI-induced indirect hyperbilirubinemia
Why This Is Correct:
- Nilotinib is a potent UGT1A1 inhibitor, causing unconjugated (indirect) hyperbilirubinemiawithout hepatocellular damage. This is:
  -Dose-dependent (more common at 400 mg BID).
  -Benign(does not require discontinuation unless bilirubin >3x ULN).
  - Distinct from true liver injury (ALT/AST remain normal).
- Pruritus/rash is a separate, common TKI-class effect (immune-mediated).
Why Other Options Are Incorrect:
A) Hepatocellular injury: Requires elevated ALT/AST (not seen here).
B) Gilberts syndrome: A genetic predisposition, but this patients symptoms began after TKI initiation.
C) Biliary obstruction: Would cause direct hyperbilirubinemia+ alkaline phosphatase elevation.
Management Pearls
1. Monitor bilirubin monthlyif >3x ULN, consider dose reduction.
2.For rash: Start antihistamines (e.g., cetirizine) or topical steroids. If severe, switch to another 2G-TKI (e.g., dasatinib).
Phase 2: Treatment Monitoring & Complications

Scenario: At 6 months, the patients BCR::ABL1 transcript level is 5% IS (International Scale).
Question 4 (Response Evaluation)
According to ELN 2023 guidelines, this represents:
A)Optimal response
B)Warning(requires closer monitoring)
C)Treatment failure (needs therapy change)
D)Inadequate sample quality
Type your answer (A, B, C, or D).
Answer: B) Warning (requires closer monitoring)
Feedback on Question 4
Correct Answer: B) Warning (requires closer monitoring)
Why This Is Correct:
-ELN 2023 response criteria for BCR::ABL1 transcript levels at 6 months:
  -Optimal: 1% IS
  -Warning: >110% IS
  -Failure: >10% IS
- A result of 5% IS at 6 months signals:
  - Suboptimal response but not outright failure.
  - Requires enhanced monitoring (qPCR every 46 weeks) and investigation for adherence/comorbidities.
Why Other Options Are Incorrect:
- A) Optimal response: Would require 1% IS.
- C) Treatment failure: Applies only if >10% IS or loss of prior response.
-D) Inadequate sample: Not relevant herePCR results are valid unless technical issues are documented.
Next Steps for This Patient
1. Adherence check: Verify TKI compliance (nilotinib has strict fasting requirements).
2.Resistance testing: If no adherence issues, perform BCR::ABL1 kinase domain mutation analysis.
3.Consider dose escalation (nilotinib 400 mg BID) or switch to alternative 2G-TKI (e.g., dasatinib).
Question 5 (Management of Warning Response)
What intervention is least appropriate for this patient?
A)Switch to dasatinib 100 mg daily
B)Continue nilotinib with monthly PCR monitoring
C)Immediate hematopoietic stem cell transplant (HSCT) evaluation
D)Increase nilotinib dose to 400 mg BID
Type your answer (A, B, C, or D).
Answer: C) Immediate hematopoietic stem cell transplant (HSCT) evaluation
Rationale:
At the warning response level (e.g., BCR::ABL1 = 5% at 6 months), the ELN guidelines recommend reassessing therapy but do not justify immediate HSCT, which is reserved for:
  TKI failure (e.g., >10% at 6 months, rising transcript despite intervention)
  Advanced phase disease
  Resistant mutations (e.g., T315I)
Feedback on Question 5
Correct Answer: C) Immediate hematopoietic stem cell transplant (HSCT) evaluation
Why This Is Correct:
-HSCT is not indicated for a patient with chronic-phase CML and only a "warning" response.
- ELN 2023 guidelines reserve HSCT for:
  -Blast-phase CML(after reinduction to chronic phase).
  -TKI failure (resistance to 2 TKIs with progression).
- This patient still has options for pharmacotherapy (dose adjustment, TKI switch).
Why Other Options Are Appropriate:
A) Switch to dasatinib: Reasonable for suspected nilotinib resistance (non-cross-resistant mechanism).
B) Continue nilotinib with closer monitoring: Acceptable if adherence is confirmed and mutations are absent.
D) Dose escalation to 400 mg BID: Supported by ENESTnd trial data for suboptimal responders.
Key Clinical Takeaway

-HSCT is a last-line option** due to its morbidity/mortality. Always exhaust pharmacotherapy first in chronic phase.
-Next steps:
  1. Rule out non-adherence (nilotinib requires fasting; complex dosing).
  2. Test for BCR::ABL1 kinase domain mutations (e.g., T315I).
  3. If no mutations, consider dasatinib or dose escalation.
Phase 3: Long-Term Management
Scenario: After switching to dasatinib 100 mg daily, the patient achieves MMR (BCR::ABL1 0.1% IS) by 12 months. At 24 months, he develops pleural effusion.
Question 6 (TKI Toxicity Management)
What is the first step in managing this complication?
A)Permanently discontinue dasatinib
B)Initiate prednisone 40 mg/day
C)Hold dasatinib + evaluate for effusion severity
D)Switch to bosutinib immediately
Type your answer (A, B, C, or D).
Answer: C) Hold dasatinib + evaluate for effusion severity
Feedback on Question 6
Correct Answer: C) Hold dasatinib + evaluate for effusion severity
Why This Is Correct:
-ELN 2023 guidelines for dasatinib-associated pleural effusion:
  1.Immediately hold dasatinib to prevent worsening.
  2.Assess effusion severity (symptoms, imaging, thoracentesis if large/moderate).
  3.Manage symptomatically (diuretics for mild; drainage for severe).
  4.Rechallenge at reduced dose (e.g., 50 mg daily) or switch TKI if recurrent.
Why Other Options Are Incorrect:
A) Permanent discontinuation: Overly aggressive for first occurrence (effusions are often manageable).
B) Prednisone: Used for immune-mediated pneumonitis (not typical for effusions).
D) Immediate switch: Premature without assessing reversibility or rechallenge potential.
Management Algorithm for Pleural Effusion
1.Grade 1-2 (asymptomatic/mild): Hold dasatinib resume at lower dose (e.g., 40 mg daily) after resolution.
2.Grade 3-4 (symptomatic/large):
   - Therapeutic thoracentesis + consider bosutinib (lower effusion risk).
   - Avoid steroids unless inflammatory component confirmed.
Question 7 (Long-Term Monitoring)
The patients BCR::ABL1 reaches MR4.5 (0.0032% IS) after 5 years on dasatinib. He asks about treatment discontinuation. Per ELN 2023, what is required for eligible candidates?
A)Sustained MR4.5 for 2 years
B)Prior failure of two TKIs
C)BCR::ABL1 kinase domain mutation testing
D)HSCT consultation
Type your answer (A, B, C, or D)
Phase 1: Initial Presentation & Diagnostic Workup
Case Introduction
Patient:58-year-old male
Chief Complaint:Fatigue, early satiety, and unexplained weight loss over 3 months.
Physical Exam:
- Splenomegaly (palpable 6 cm below costal margin)
- No lymphadenopathy or bleeding manifestations
Labs:
- WBC: 68 x 10/L (neutrophils 60%, basophils 8%, blasts 2%)
- Hb: 9.8 g/dL
- Platelets: 450 x 10/L
- LDH: 2x ULN
Question 1 (Diagnosis)

What is the next critical diagnostic step to confirm CML?
A) Peripheral blood flow cytometry
B) Bone marrow biopsy with cytogenetics (karyotyping) and PCR for BCR::ABL1
C) JAK2V617F mutation testing
D) Serum protein electrophoresis
Type your answer (A, B, C, or D).
Key Considerations for TFR
1.Monitoring:
- Monthly BCR::ABL1 PCR for 6 months, then every 23 months indefinitely.
- Relapse (defined as loss of MMR) requires immediate TKI reinitiation.
2. Psychological support: Anxiety about relapse is common.
Final Question (Case Conclusion)
The patient opts for TKI discontinuation. At 3 months post-cessation, his BCR::ABL1 rises to 0.02% IS (MMR retained). What is the next step?
A)Reinitiate dasatinib immediately
B)Continue monitoring without intervention
C)Switch to ponatinib
D)Proceed to HSCT
Type your answer (A, B, C, or D).
Answer: B) Continue monitoring without intervention
Feedback on Final Question
Correct Answer: B) Continue monitoring without intervention
Why This Is Correct:
- ELN 2023 TFR guidelines define relapse as loss of MMR (>0.1% IS).
- This patient retains MMR (0.02% IS is still <0.1%), so no action is needed.
-Close monitoring (monthly PCR)is mandatory to detect true relapse.
- ~30% of patients experience molecular fluctuations without clinical significance (Saussele et al., Blood 2018).
Why Other Options Are Incorrect:
A) Reinitiate dasatinib: Only required if MMR is lost. Premature intervention risks unnecessary lifelong therapy.
C) Switch to ponatinib: Reserved for T315I mutation or multi-TKI failure (no evidence here).
D) Proceed to HSCT: Not indicated for molecular fluctuations in TFR.

Session ID:
Assessment Domain

Session-72:
Rationale

Session ID:
Instructional Design and Pedagogical

Session-72:
The session's instructional design is very strong. The clinical case is expertly scaffolded, progressing logically from initial diagnosis and risk stratification to adverse effect management, response monitoring, and advanced topics like treatment-free remission (TFR). The quality of the embedded feedback is a key strength; each rationale is detailed, cites current clinical guidelines (e.g., ELN, ESMO), and clearly explains the correctness of the chosen answer and the flaws in the distractors. The primary weakness is the lack of explicitly stated learning objectives at the outset. Additionally, the session omits the answer and feedback for Question 7, which slightly disrupts the instructional flow and clarity, preventing a perfect score in that category.

Session ID:
Clinical Authenticity and Content Fidelity

Session-72:
The session demonstrates exceptional clinical fidelity and content accuracy. Plausibility & Guideline Alignment: The clinical narrative

is highly plausible, presenting a textbook case of CML that evolves realistically. All therapeutic decisions, monitoring milestones (e.g., 5% IS at 6 months being a "Warning"), and management of complications (nilotinib-induced hyperbilirubinemia, dasatinib-associated pleural effusion) are perfectly aligned with current, evidence-based ELN and ESMO guidelines. Pharmacotherapeutic Accuracy & Domain Specificity: The pharmacotherapeutic recommendations are optimal and well-justified throughout the case. There are no content-level errors or questionable therapeutic choices. Critically, the session exhibits perfect domain specificity; all concepts, diagnostics, and therapies are strictly relevant to CML, with no "domain entanglement" from other hematological malignancies. The content is safe, accurate, and educationally sound.

Session ID:
Modeled Clinical Reasoning

Session-72:
The simulation expertly models a high-level clinical reasoning process. Each question frames a distinct and relevant clinical problem, providing all necessary data for an informed decision. The multiple-choice options demonstrate excellent breadth, including not only the correct answer but also highly plausible distractors that effectively probe for nuanced understanding of TKI selection, toxicity management, and response criteria. The case narrative consistently demonstrates sound prioritization, such as exhausting pharmacotherapeutic options before considering HSCT. The described clinical outcomes are realistic and highly instructive, reinforcing the key learning objectives implied by the case structure.

Session-73

Session ID:
LLM

Session-73:
DeepSeek V2

Session ID:
Session Transcript

Session-73:
Case Introduction
Patient Profile:

48-year-old male

Chief Complaint: Fatigue, early satiety, and 6 kg unintentional weight loss over 2 months

Physical Exam: Splenomegaly (palpable 8 cm below costal margin)

No significant comorbidities

Initial Lab Results
CBC: WBC 98 x 10/L (), Hb 10.2 g/dL (), Platelets 450 x 10/L ()

Differential: Myelocytes 25%, Metamyelocytes 15%, Blasts 2%

LDH: 850 U/L ()

Renal/Liver Function: Normal

Question 1 (Diagnostic Framework)
What is the MOST appropriate next step to confirm CML diagnosis?
A) Peripheral blood flow cytometry
B) Bone marrow biopsy with cytogenetics (Ph chromosome) and BCR::ABL1 PCR
C) JAK2 V617F mutation testing
D) Serum protein electrophoresis (SPEP)
Correct Answer: B) Bone marrow biopsy with cytogenetics (Ph chromosome) and BCR::ABL1 PCR
Question 2 (Risk Stratification)
Using the ELN 2022 prognostic score (Sokal/EUTOS), which factor would classify this patient as high risk?
A) Age >60 years
B) Spleen size >10 cm below costal margin
C) Platelets >700 x 10/L
D) Peripheral blood blasts 3%
no answer is true
Question 3 (First-line Therapy):
What is the preferred first-line TKI for this high-risk CML patient?
A) Imatinib 400 mg daily
B) Nilotinib 300 mg BID
C) Dasatinib 100 mg daily
D) Bosutinib 400 mg daily
Correct Answer: B) Nilotinib 300 mg BID

Question 4 (Resistance Monitoring):
What is the NEXT step in monitoring for potential resistance?
A) Repeat BCR::ABL1 PCR at 6 months
B) Perform ABL1 kinase domain mutation testing now
C) Switch to dasatinib due to rash
D) Reduce nilotinib dose to 200 mg BID
Correct Answer: A) Repeat BCR::ABL1 PCR at 6 months
Question 5 (Managing Comorbidities):
How would you adjust therapy given this new diabetes diagnosis?
A) Continue nilotinib, intensify diabetes management
B) Switch to dasatinib (lower diabetes risk)
C) Reduce nilotinib dose to 200 mg BID
D) Switch to imatinib
Correct Answer: B) Switch to dasatinib (lower diabetes risk)
Question 6 (Managing TKI Resistance):
What is the BEST next step for this T315I-mutated, dasatinib-resistant CML?
A) Ponatinib 45 mg daily
B) Allogeneic HSCT evaluation
C) Omacetaxine
D) Clinical trial (e.g., asciminib + TKI)
Correct Answer: A) Ponatinib 45 mg daily
Question 7 (Blast Crisis Management):
What is the MOST appropriate treatment strategy?
A) Ponatinib + hyper-CVAD chemotherapy
B) FLAG-IDA chemotherapy alone
C) Immediate allogeneic HSCT
D) Dasatinib + blinatumomab
Correct Answer: A) Ponatinib + hyper-CVAD chemotherapy
Final Question (Long-Term Survivorship):
After successful HSCT, what is the MOST important monitoring strategy?
A) Monthly BCR::ABL1 PCR indefinitely
B) BCR::ABL1 PCR every 3 months for 5 years, then annually
C) Chimerism studies only if relapse suspected
D) Stop all monitoring after 2 years of negative PCR

Select your answer (A, B, C, or D).
Correct Answer: B) BCR::ABL1 PCR every 3 months for 5 years, then annually

---

Session ID:
Assessment Domain

Session-73:
Rationale

Session ID:
Instructional Design and Pedagogical

Session-73:
The session's instructional design has significant strengths but one critical weakness. The case is expertly scaffolded, progressing logically from diagnosis and risk stratification through the complexities of TKI selection, side effect management, resistance, and advanced disease. The instructions for each question are exceptionally clear. However, the session lacks explicitly stated learning objectives, which must be inferred from the content. The most significant flaw lies in the implied quality of feedback. Several "correct" answers are based on flawed premises or contain factual inaccuracies (detailed in the next section). Therefore, the embedded rationales would be misleading or erroneous. For instance, the rationale for Question 2 would be invalid as none of the options are

correct, and the rationale for Question 4 would incorrectly label routine response monitoring as "resistance monitoring," which is a fundamentally different clinical concept.

Session ID:
Clinical Authenticity and Content Fidelity

Session-73:
While the clinical narrative is highly plausible and realistic, the session contains multiple significant content errors that compromise its educational value and safety. Plausibility & Domain Specificity: The patient presentation and disease progression are indistinguishable from an authentic CML case. The session correctly remains within the CML domain. The inclusion of blinatumomab as a distractor in Question 7 is contextually appropriate, as it is used for lymphoid blast crisis of CML, and does not represent domain entanglement.
Guideline Deviation & Inaccuracy: The simulation deviates significantly from clinical guidelines and contains factual errors:
Question 2 (Risk Stratification): This question is critically flawed. Based on the patient's data (Age 48, Spleen 8cm, Blasts 2%, Platelets 450), none of the answer choices would classify him as high-risk by Sokal or EUTOS criteria. This is a major error in content accuracy.
Question 3 (First-line Therapy): The question incorrectly frames the patient as "high-risk" based on the flawed premise of Question 2. While a second-generation TKI is a reasonable choice, presenting Nilotinib as the single preferred agent is questionable, especially given its known risk for inducing hyperglycemia, which the patient later develops.
Question 4 (Resistance Monitoring): The question misuses terminology. Performing a BCR::ABL1 PCR at 6 months is standard response monitoring, not resistance monitoring. ABL1 kinase domain mutation testing is performed to investigate resistance, typically upon loss of response, not prospectively. This error confuses two distinct monitoring purposes.

Session ID:
Modeled Clinical Reasoning

Session-73:
The simulation's ability to model expert clinical reasoning is undermined by its content flaws. While each stage presents a well-framed clinical problem (e.g., "How do we manage this new comorbidity?"), the tools provided for the decision-making process are faulty. The diagnostic and therapeutic breadth is only adequate; the options in Question 2 are all incorrect, which breaks the reasoning exercise. The prioritization and decision-making score is reduced because the logical flow is compromised. The simulation forces a decision in Question 3 based on an incorrect "high-risk" premise from Question 2, modeling a flawed reasoning process (garbage in, garbage out). While the overall strategic path (Diagnosis -> Treatment -> Monitoring) is sound, the individual tactical decisions are based on incorrect data and concepts provided by the simulation itself. The modeled clinical outcomes are realistic, but the path to reaching them is not always based on sound medicine.

Session-74

Session ID:
LLM

Session-74:
DeepSeek V2

Session ID:
Session Transcript

Session-74:
Case Presentation:
A 38-year-old female presents with fatigue, night sweats, and abdominal fullness. Physical exam reveals splenomegaly (5 cm below costal margin).

Initial Labs:

WBC: 112 10/L (neutrophilia, myelocytes, basophils)

Hb: 9.8 g/dL

Platelets: 520 10/L

LDH: 2,200 U/L (elevated)
Question 1 (Knowledge Application)
What is the MOST critical next step to confirm CML diagnosis?
A) Peripheral blood smear review
B) Bone marrow biopsy with cytogenetics (Ph chromosome) and BCR::ABL1 PCR
C) JAK2 V617F mutation testing
D) Serum uric acid level

Select your answer (A, B, C, or D).
b
Feedback on Question 1
You selected B) Bone marrow biopsy with cytogenetics (Ph chromosome) and BCR::ABL1 PCR.

This is correct.

Key Rationale (ESMO 2022 Guidelines):
Definitive Diagnosis Requires:

Cytogenetics: Detection of the Philadelphia (Ph) chromosome (t(9;22)(q34;q11)).

Molecular Testing: Quantitative BCR::ABL1 PCR to confirm the fusion gene and establish a baseline for monitoring.

Why Not Other Options?

A) Peripheral smear suggests CML (e.g., left shift, basophilia) but cannot confirm it.

C) JAK2 testing is for polycythemia vera/ET, not CML.

D) Uric acid is a supportive test (tumor lysis risk) but not diagnostic.

Question 2 (Application Analysis)
The bone marrow confirms Ph+ CML (chronic phase). BCR::ABL1 transcript level is 55% (IS).

How would you risk-stratify this patient per ELN 2022 criteria?
A) Low risk (Sokal score 0.8)
B) Intermediate risk (Sokal score >0.81.2)
C) High risk (Sokal score >1.2)
D) Cannot determine without additional data (e.g., spleen size, platelet count)

Select your answer (A, B, C, or D).
hint
Targeted Hint for ELN 2022 Risk Stratification
Key Variables for Sokal Score Calculation:

Age (years)

Spleen size (cm below costal margin)

Platelet count (10/L)

Blast percentage in peripheral blood (%)

Formula:

Sokal Score
=
exp

(
0.0116

(
Age

43.4
)
+
0.0345

(
Spleen Size

7.51
)
+
0.188

(
Platelets
700
)
2

0.563

+
0.0887

(
Blasts

2.10
)
)
Sokal Score=exp(0.0116(Age43.4)+0.0345(Spleen Size7.51)+0.188(
700
Platelets

)
2
 0.563+0.0887(Blasts2.10))
Clinical Implications:

Low risk (0.8): May favor less aggressive TKIs (e.g., imatinib).

High risk (>1.2): May need 2nd-gen TKIs (e.g., dasatinib, nilotinib).

Re-examine the case:

Patient is 38 years old (younger = lower risk).

Spleen 5 cm, platelets 520 10/L, blasts not mentioned (assume <2%).

Struggling?

Simplify: Younger age + moderate splenomegaly often suggests low/intermediate risk.

answer D
Feedback on Question 2
You selected D) Cannot determine without additional data (e.g., spleen size, platelet count).

This is partially correct but incomplete.

Key Clarification (ELN 2022):
Sokal Score Requires:

The case already provided spleen size (5 cm) and platelets (520 10/L).

Missing data: Blast % (but chronic phase typically has <2% blasts).

Estimation with Available Data:

Age 38 (favorable) + spleen 5 cm (moderate) + platelets 520 (normal) Likely low-risk Sokal score (0.8).

Example calculation: With blasts assumed at 1%, Sokal score 0.6 (low risk).

Why Not D?

While exact scoring benefits from all variables, clinicians often estimate risk when key data (like blasts) are absent but clinically low.

Question 3 (Analysis Decision-Making)
The patients estimated Sokal score is low risk (0.6). She has no comorbidities (normal cardiac/renal function).

Per ELN 2022, what is the PREFERRED first-line TKI for this patient?
A) Imatinib 400 mg daily
B) Nilotinib 300 mg BID
C) Dasatinib 100 mg daily
D) Ponatinib 45 mg daily

Select your answer (A, B, C, or D).
a
Feedback on Question 3
You selected A) Imatinib 400 mg daily.

This is correct and guideline-concordant.

Key Rationale (ELN 2022 Recommendations):
For Low-Risk Chronic Phase CML:

Imatinib 400 mg daily remains preferred first-line therapy due to:

Excellent long-term efficacy (10-year OS >80%)

Favorable toxicity profile vs. 2nd-gen TKIs

Lower cost

Why Not 2nd-Gen TKIs (B, C, D)?

While nilotinib/dasatinib induce faster/deeper responses, they offer no survival advantage in low-risk patients and have higher:

Cardiovascular risk (nilotinib)

Pleural effusion risk (dasatinib)

Cost

Ponatinib is reserved for T315I mutation or salvage therapy.

Supporting Evidence:

ENESTnd and DASISION trials showed no OS benefit with frontline 2nd-gen TKIs in low-risk patients.

Real-world data confirms imatinib's durability in this population.

Phase 2: Treatment Monitoring & Toxicity Management
Case Update:
The patient starts imatinib 400 mg daily. At 3-month follow-up:

BCR::ABL1 IS: 8.5%

CBC: WBC 6.2 10/L, platelets 210 10/L

Complains of grade 2 fatigue and periorbital edema

Question 4 (Application Management)
What is your interpretation of her 3-month molecular response?
A) Optimal response (expected 10% BCR::ABL1 reduction at 3 months)
B) Warning (expected <10% BCR::ABL1)
C) Failure (expected complete cytogenetic response)
D) Cannot assess without cytogenetics

Select your answer (A, B, C, or D).
hint
Targeted Hint: ELN 2023 Response Criteria at 3 Months
Key Molecular Response Milestones for First-Line TKI Therapy
Optimal Response (Continue Current Therapy):

3 Months: BCR::ABL1 10% (IS)

Rationale: Predicts long-term event-free survival (EFS) >85%

Warning (Close Monitoring Needed):

3 Months: BCR::ABL1 >10% but <35%

Action: Verify adherence, consider dose escalation if suboptimal

Treatment Failure (Requires Intervention):

3 Months: BCR::ABL1 >35%

Action: Switch to 2nd-gen TKI after mutational analysis

Applied to This Case:
Patient's BCR::ABL1 = 8.5% at 3 months Optimal response per ELN 2023.

Fatigue/edema are common imatinib toxicities (manage supportively)
answer A
Feedback on Question 4
You selected A) Optimal response (expected 10% BCR::ABL1 reduction at 3 months).

This is absolutely correct.

ELN 2023 Response Criteria Breakdown:
Optimal Response Definition at 3 Months:

BCR::ABL1 10% (International Scale)

This patient's 8.5% IS meets criteria

Clinical Implications:

Continue imatinib at current dose (400 mg daily)

Next milestone: BCR::ABL1 1% at 6 months

Why Not Other Options?

B) Warning: Applies if 10-35% at 3 months

C) Failure: Only if >35% at 3 months

D) Cytogenetics: Not required if molecular testing available

Question 5 (Toxicity Management)
The patient's grade 2 fatigue and periorbital edema persist at 6 weeks. She is adherent to therapy.

What is the NEXT best step?
A) Switch to nilotinib
B) Reduce imatinib to 300 mg daily
C) Add low-dose diuretic + schedule rest periods
D) Discontinue TKI therapy temporarily

Select your answer (A, B, C, or D).
answer c
Feedback on Question 5
You selected C) Add low-dose diuretic + schedule rest periods.

This is correct and guideline-supported.

Stepwise Toxicity Management (NCCN 2023):
For Grade 2 Non-Hematologic Toxicities (e.g., edema, fatigue):

First-line: Supportive care (diuretics, salt restriction, fatigue management)

Rationale: Most side effects improve with time/interventions without dose modification

Why Not Other Options?

A) Switch to nilotinib: Overly aggressive for manageable toxicity

B) Dose reduction: Premature before trying supportive measures

D) Discontinue: Not indicated for grade 2 toxicities

Evidence:

Studies show >50% of imatinib-related edema improves with diuretics/lifestyle changes

Fatigue often resolves with sleep hygiene and scheduled rest

Phase 3: Long-Term Monitoring & Resistance
Case Update:
At 12 months:

BCR::ABL1: 0.8% IS (MMR not yet achieved)

No toxicity issues

Patient asks: "Should we change treatment?"

Question 6 (Evaluation Decision-Making)
How would you manage this patient per ELN 2023?
A) Continue imatinib (adequate but delayed response)
B) Switch to dasatinib (accelerate response)
C) Increase imatinib to 600 mg daily
D) Perform BCR::ABL1 kinase domain mutation testing

Select your answer (A, B, C, or D).
hint
Targeted Hint: Managing Delayed Molecular Response (ELN 2023)
Key Considerations for 12-Month Evaluation:
Expected Milestone:

Optimal: BCR::ABL1 0.1% IS (MMR) by 12 months

Acceptable (Gray Zone): BCR::ABL1 0.1-1% IS Requires closer monitoring but not automatic therapy change

For This Patient (0.8% at 12 Months):

First Step: Verify adherence and test sensitivity (is the result reproducible?)

If confirmed:

Continue current therapy if BCR::ABL1 is declining (e.g., was 8.5% 0.8%)

Consider dose escalation (imatinib 600 mg) before switching TKIs

When to Switch TKIs? Only if:

Loss of response (rising BCR::ABL1)

Confirmed kinase domain mutation (e.g., T315I)

Intolerance to therapy

Evidence:

30% of patients achieve MMR after 12 months on imatinib

Unnecessary early switching increases toxicity/cost without proven survival benefit
answer A
Feedback on Question 6
You selected A) Continue imatinib (adequate but delayed response).

This is the most appropriate choice per current guidelines.

ELN 2023 Recommendations for Delayed MMR:
For BCR::ABL1 0.8% at 12 Months:

Classified as "warning" but not failure"

Continue current dose if:

Patient is adherent

Transcript levels are declining (was 8.5% 0.8%)

No mutations detected

Supporting Evidence:

IRIS trial data shows 40% of imatinib-treated patients achieve MMR between 12-18 months

No survival disadvantage vs achieving MMR by 12 months

Why Not Other Options?

B) Switch to dasatinib: Premature without treatment failure

C) Dose increase: May be considered if no decline in next 3 months

D) Mutation testing: Not indicated unless BCR::ABL1 >1%

Monitoring Plan:

Repeat PCR in 3 months

Consider dose escalation only if no further decline

Session ID:
Assessment Domain

Session-74:
Rationale

Session ID:
Instructional Design and Pedagogical

Session-74:
The session's instructional design is of very high quality. The case is expertly scaffolded, progressing logically from initial diagnosis and risk stratification to treatment selection, response monitoring, toxicity management, and long-term decision-making. The quality of the embedded feedback is a major strength; each rationale is detailed, cites current clinical guidelines (e.g., ELN, ESMO, NCCN), and correctly explains the clinical reasoning. Instructions are exceptionally clear. The only minor weakness is the absence of explicitly stated, measurable learning objectives at the beginning of the session, though the educational goals are strongly implied by the structure and content.

Session ID:
Clinical Authenticity and Content Fidelity

Session-74:
The session demonstrates exceptional clinical fidelity with no significant errors. Plausibility & Guideline Alignment: The clinical narrative is indistinguishable from an authentic patient case. All therapeutic decisions, monitoring milestones (e.g., 10% BCR::ABL1 at 3 months), and rationales are perfectly aligned with current, evidence-based guidelines for Chronic Myeloid Leukemia (CML)

management. Pharmacotherapeutic Accuracy: The recommended therapeutic choicesimatinib for a low-risk patient, supportive care for grade 2 toxicity, and continuing therapy for a delayed responseare optimal and well-justified. Domain Specificity: The case remains strictly within the CML domain. It correctly differentiates CML from other myeloproliferative neoplasms (e.g., by dismissing JAK2 testing) and applies CML-specific standards of care throughout. There is no evidence of domain entanglement.

Session ID:

Modeled Clinical Reasoning

Session-74:

The simulation expertly models a sound clinical reasoning process. Each stage presents a clearly defined clinical problem with the necessary data for a decision. The multiple-choice options provide an excellent breadth of plausible alternatives, including relevant distractors that effectively test nuanced understanding (e.g., choosing between imatinib and a 2nd-gen TKI). The narrative demonstrates appropriate prioritization, such as using risk stratification to guide therapy choice and managing toxicity conservatively before escalating treatment. The clinical outcomes are realistic and highly instructive, reinforcing the learning objectives at each step.

Session-75

Session ID:
LLM

Session-75:
DeepSeek V2

Session ID:
Session Transcript

Session-75:
A 55-year-old male with fatigue, easy bruising, and 10% weight loss over 2 months.
- Labs: WBC 45,000/L (blasts on smear), Hb 7.8 g/dL, platelets 32,000/L.
Example of whats coming in Question
1:
The peripheral smear shows 30% blasts with Auer rods. What is the next diagnostic step?
A. Bone marrow biopsy
B. PCR for BCR-ABL
C. Serum protein electrophoresis
D. Direct Coombs test

-Bone marrow biopsy is the gold standard to confirm blast percentage (20% defines AML) and assess morphology/cytogenetics.
- Why not others?
  - B (PCR for BCR-ABL): Used for CML, not AML.
  - C (Serum protein electrophoresis): Screens for myeloma.
  - D (Direct Coombs test): Detects autoimmune hemolysis.

Clinical Scenario:
The bone marrow biopsy returns with:
- 50% blasts
- Cytogenetics: t(8;21)(q22;q22) RUNX1-RUNX1T1 fusion
- Molecular studies: NPM1 mutation-positive, FLT3-ITD-negative

How would you classify this AML per ELN 2022 risk stratification?
A. Favorable risk
B. Intermediate risk
C. Adverse risk
D. Provisional (await further testing)
A. Favorable risk is correct because:
- t(8;21) (RUNX1-RUNX1T1)is a core-binding factor (CBF) AML, which is **favorable-risk per ELN 2022, unless:
  - A *KIT mutation* is present (not mentioned here).
- **NPM1 mutation further supports favorable prognosis (especially without *FLT3-ITD).

**Why not the others?
- B (Intermediate): Would apply to wild-type NPM1 without FLT3-ITD* or other risk modifiers.
- C (Adverse): Requires features like TP53 mutation, complex karyotype, or FLT3-ITD high allelic ratio*.
- **D (Provisional)**: Unnecessarythe genetic picture is already definitive.

Clinical Scenario:
Your patients final diagnosis:

- AML with t(8;21) (favorable risk), NPM1+, FLT3-ITD, KIT.
- ECOG PS 1, normal cardiac/liver/renal function.

Which induction regimen is most appropriate?
A. 7+3 (Cytarabine + Daunorubicin)
B. Venetoclax + Azacitidine
C. Gemtuzumab ozogamicin (GO) monotherapy
D. Midostaurin + 7+3

A. 7+3 (Cytarabine + Daunorubicin) is the **gold-standard induction for this patient** because:
- Favorable-risk AML (t(8;21)) has exceptional response rates to 7+3 (~80-90% remission).
- Fit patient (age 55, ECOG 1)**: Intensive chemo offers the best chance for cure.
- **No FLT3-ITD: Midostaurin (D) isnt needed.
- GO (C) could be added in combination with 7+3 for CBF-AML, but isnt used alone.

Why Not the Others?
- **B (Venetoclax + Azacitidine): For unfit patients (75 yrs or comorbidities).
- **C (GO monotherapy): No evidence in this settingonly combined with chemo.
- **D (Midostaurin + 7+3): Only if FLT3+.

Clinical Scenario:
Your patient is starting 7+3 induction (cytarabine 200 mg/m 7d + daunorubicin 90 mg/m 3d).
Which supportive care intervention is MOST critical to initiate on Day 1?
A. Posaconazole prophylaxis
B. Allopurinol 300 mg PO daily
C. IV hydration at 150 mL/hr
D. Filgrastim (G-CSF) starting Day 8
B. Allopurinol 300 mg PO daily is the most critical Day 1 intervention because:
- High TLS Risk: This patients WBC 45,000/L + high blast burden puts them at immediate risk for tumor lysis.
  - Allopurinol inhibits uric acid production (prevents renal failure).
Why Not the Others?**
- A (Posaconazole): Vital for fungal prophylaxis but can wait until
Day 2-3(after TLS addressed).
- C (IV hydration): Supports TLS management but is *adjunctive* to allopurinol/rasburicase.
- **D (Filgrastim): *Avoid in AML induction* unless severe neutropenic complications arise (controversial due to blast stimulation risk).
Clinical Scenario:
On Day 6 of 7+3, your patient develops:
- Fever (39.1C) and severe mucositis.
- Labs: ANC 0 (absolute neutrophil count), Cr 1.6 mg/dL (baseline 0.8).
- Blood cultures pending.

What is the NEXT BEST step?
A. Start piperacillin-tazobactam + vancomycin
B. Hold chemotherapy and initiate IV fluids
C. Add posaconazole and acyclovir
D. Administer filgrastim (G-CSF) immediately
A. Start piperacillin-tazobactam + vancomycin is the immediate life-saving action because:
- Febrile neutropenia in AML requires empiric antibiotics within 1 hour** (IDSA guidelines).
  - **Piperacillin-tazobactam: Covers *Pseudomonas and gut flora (critical with mucositis).
  - **Vancomycin: Added for MRSA/gram-positive coverage (mucositis breaches mucosal barriers).
- **Renal adjustment: Reduce pip-tazo dose/extend infusion due to Cr rise.

Why Not the Others?
- **B (Hold chemo/IV fluids): Chemo is already finishing (Day 6/7); fluids dont treat infection.
- **C (Posaconazole/acyclovir): Add later if fever persists >72h (fungal/viral coverage).
- **D (Filgrastim): Contraindicated in AML induction (may stimulate residual blasts).
Clinical Scenario:
On Day 10 of induction, your patient develops:
- Dyspnea, fever (38.5C), weight gain (+3 kg), and pulmonary infiltrates on CXR.
- Labs: WBC 1,200/L (blasts absent), Cr 2.1 mg/dL (baseline 0.8), BNP 850 pg/mL.

What is the MOST LIKELY diagnosis?
A. Differentiation syndrome (DS)
B. Sepsis with ARDS
C. Heart failure exacerbation
D. Pulmonary hemorrhage
A. Differentiation syndrome (DS) is the correct diagnosis because:
- **Pathognomonic Triad: Fever + weight gain + pulmonary/renal dysfunction in this *timeframe(Day 10 post-chemo).
- **Mechanism: Cytokine storm from *differentiating myeloid cells (even without circulating blasts).
- **BNP Elevation: Due to volume overload from capillary leak (not primary cardiac failure).
Why Not the Others?
- **B (Sepsis with ARDS): Would show *hypotension, worse hypoxemia, and often a clear infection source.
- **C (Heart failure): Requires prior cardiac dysfunction (e.g., low EF) and typically has *dependent edema.
- **D (Pulmonary hemorrhage): Would cause hemoptysis and abrupt hemoglobin drop.
Clinical Scenario:
Your patient with t(8;21) AMLdevelops fever, dyspnea, and hypotension on Day 8 of induction. You suspect differentiation syndrome (DS) but must rule out sepsis.

What is the NEXT BEST action?
A. Start dexamethasone 10 mg IV BID + empiric vancomycin/piperacillin-tazobactam
B. Hold all medications and initiate IV fluids alone
C. Administer diuretics only
D. Wait for blood culture results before acting
A. Start dexamethasone 10 mg IV BID + empiric vancomycin/piperacillin-tazobactam is the correct life-saving actionbecause:
1. Simultaneous Treatment is Key:
   - Dexamethasone: Rapidly counters DS-driven capillary leak/inflammation.
   - Empiric antibiotics: Cover sepsis (especially with mucositis + hypotension).

2. Why Not the Others?
   - B (IV fluids alone): Misses both steroid and antibiotic needs.
   - C (Diuretics only): Ignores steroids for DSand infection risk.
   - D (Wait for cultures): Mortality spikes with any delay in AML febrile neutropenia.
Clinical Scenario:
Your patient achieved complete remission (CR) after 7+3 induction.
- Persistent t(8;21) on MRD testing (0.1% blasts by flow).
- ECOG PS 0, normal cardiac/renal function.

What is the BEST next step?
A. High-dose cytarabine (HiDAC) consolidation 4 cycles
B. Allogeneic hematopoietic cell transplant (allo-HCT)
C. Venetoclax + azacitidine maintenance
D. Gemtuzumab ozogamicin (GO) monotherapy
A. High-dose cytarabine (HiDAC) consolidation 4 cycles is the evidence-based choice because:
- Favorable-risk AML (t(8;21)) has 60-70% cure rates* with HiDAC alone (CALGB trials).
  - Dosing: 3 g/m every 12h on Days 1, 3, 5 (adjust for age/renal function).
- MRD+ (0.1%) doesnt alter initial consolidationrepeat testing post-cycle 1 guides further action.

Why Not the Others?
- B (Allo-HCT): Reserved for persistent MRD after HiDAC or relapse (too aggressive upfront).
- C (Venetoclax + azacitidine): For unfit patients (this 55-year-old is fit with ECOG 0).
- D (GO monotherapy): No role in consolidation (used with chemo in induction for CBF-AML).
Clinical Scenario:
Your patient completed 4 cycles of HiDAC with:
- Sustained CR, MRD-negative by flow cytometry.
- Now 1 year post-consolidation, asymptomatic.

Which monitoring strategy is MOST appropriate?
A. Monthly bone marrow biopsies for 2 years
B. CBC + differential every 36 months indefinitely
C. Annual PET-CT scans
D. No further testing unless symptoms occur
B. CBC + differential every 36 months indefinitely is the guideline-endorsed strategy because:
- NCCN Recommendations:
  - First 2 years: CBC q36mo (most relapses occur early).
  - Beyond 2 years: Annual CBC (late relapses are rare but possible in AML).
- Rationale: Detects asymptomatic relapses (e.g., dropping counts, circulating blasts) for early intervention.

Why Not the Others?
- A (Monthly marrow biopsies): Unnecessary harmno survival benefit vs. CBC monitoring in asymptomatic patients.
- C (PET-CT): AML surveillance relies on blood/marrow (not imaging unless extramedullary disease suspected).
- D (No testing): Misses early relapses (potentially curable with salvage therapy).
Clinical Scenario:
Your patient is 2 years post-consolidation. for t(8;21) AML:
- Remains in CR, MRD-negative.
- Complains of mild neuropathic hand tremors and fatigue.
- History: Received 4 cycles of HiDAC (total cytarabine dose: 36 g/m).

Which long-term toxicity is MOST likely responsible?
A. Anthracycline-induced cardiomyopathy
B. Cytarabine-induced cerebellar toxicity
C. Chronic graft-versus-host disease (GVHD)
D. Secondary myelodysplastic syndrome (MDS)

B. Cytarabine-induced cerebellar toxicity is the most likely diagnosis because:
- HiDAC Cumulative Dose = 36 g/m(high risk for neurotoxicity).
- Classic Symptoms: Tremors, fatigue, and subtle ataxia reflect chronic cerebellar dysfunction.
- Pathology: Cytarabine damages Purkinje cells cerebellar atrophy(seen on MRI in chronic cases).

Why Not the Others?
- A (Cardiomyopathy): Daunorubicin toxicity causes LVEF decline (not tremors; typically occurs <1 year post-therapy).
- C (GVHD): Requires allo-HCT (this patient never had one).
- D (Secondary MDS): Would show cytopenias/marrow dysplasia (not neurologic symptoms).

Session ID:
Assessment Domain

Session-75:
Rationale

Session ID:

Instructional Design and Pedagogical

Session-75:

The session's instructional design is very strong. The case is expertly scaffolded, progressing logically from initial diagnosis and risk stratification through induction, consolidation, management of complications, and long-term surveillance. Instructions are exceptionally clear, and each scenario is framed with the necessary clinical data. The embedded feedback provides detailed rationales that explain the correct answer while also dissecting why alternative choices are incorrect. The score for objectives is slightly reduced because they are not explicitly stated upfront, although they are clearly implied by the session's structure. The feedback quality is also marked down slightly because, while well-structured, it provides an excellent-sounding but clinically incorrect rationale for the misdiagnosed "Differentiation Syndrome," which is instructionally harmful.

Session ID:

Clinical Authenticity and Content Fidelity

Session-75:

The clinical fidelity of this session is severely compromised by a single, critical error that spans multiple questions. Plausibility & Guideline Alignment: While the initial patient presentation, diagnosis, and treatment plan (7+3 followed by HiDAC) are highly plausible and align with ELN/NCCN guidelines, the narrative introduces a major deviation. The diagnosis of Differentiation Syndrome (DS) in Question 6 as a complication of standard 7+3 chemotherapy is incorrect. DS is a known complication of differentiating agents (e.g., ATRA in APL, IDH inhibitors) but not of conventional cytotoxic chemotherapy. This makes the narrative implausible at this key juncture and constitutes a significant deviation from established clinical knowledge.

Pharmacotherapeutic Error & Domain Entanglement: The incorrect diagnosis leads to an incorrect therapeutic recommendation in Question 7: administering dexamethasone. While steroids are the treatment for DS, applying them here for a misidentified syndrome is a significant pharmacotherapeutic error. This constitutes domain entanglementa concept (DS) and its treatment (steroids) have been incorrectly applied from the context of APL/IDH-mutant AML to a standard-of-care AML induction scenario. As per the rubric, this single instance of domain entanglement is a critical flaw, resulting in the lowest possible score for that criterion.

Session ID:

Modeled Clinical Reasoning

Session-75:

The simulation excels at framing clinical problems and presenting a relevant and challenging set of diagnostic and therapeutic options, reflecting an expert's differential. However, the modeled reasoning process falters significantly in prioritization. While the strategy of simultaneously treating suspected sepsis and a perceived complication (DS) might seem sound, the reasoning is fundamentally flawed because the complication is misidentified. By incorrectly diagnosing DS, the model fails to correctly prioritize the actual likely diagnoses for the patient's symptoms (e.g., sepsis/ARDS, chemotherapy-induced pneumonitis, severe volume overload), leading to a flawed and potentially harmful decision-making process. The clinical outcomes are generally realistic and instructive, but the mislabeling of the outcome in Question 6 detracts from its educational value.

Session-76

Session ID:
LLM

Session-76:
Claude 3.7 Sonnet

Session ID:
Session Transcript

Session-76:

Question 1 (Diagnostic Framework)
What is the *most appropriate next step* to confirm suspected CML?
A) Peripheral blood flow cytometry for CD34+ cells
B) Bone marrow biopsy with cytogenetics (karyotype) and *BCR::ABL1* PCR
C) *JAK2* V617F mutation testing
D) Serum protein electrophoresis (SPEP)

Submit your answer (A, B, C, or D).
(Type "HINT" if needed.)
Feedback on Question 1:**

Correct Answer: B) Bone marrow biopsy with cytogenetics (karyotype) and *BCR::ABL1* PCR

Rationale:
- **Bone marrow biopsy** is the gold standard for CML diagnosis because it provides:
  - **Cytogenetics:** Identifies the Philadelphia chromosome (t(9;22)) in >95% of cases.
  - **Molecular testing:** *BCR::ABL1* PCR confirms the fusion gene (required for diagnosis).
  - **Blast % and fibrosis assessment:** Rules out accelerated/blast phase (WHO criteria).

Why Not Other Options?
- **A) Peripheral blood flow cytometry:** Useful for acute leukemia (CD34+ blasts) but not CML diagnosis.
- **C) *JAK2* V617F:** Relevant for polycythemia vera/essential thrombocythemia, not CML.
- **D) SPEP:** Screens for monoclonal gammopathies (e.g., myeloma), irrelevant here.

Phase 1 (Continued): Risk Stratification
Additional Findings:
- Bone marrow confirms t(9;22) and *BCR::ABL1* (p210 transcript).
- **Sokal Score:** 1.2 (intermediate risk).
Question 2 (Risk Stratification)
According to ELN 2022, which factor *does NOT* influence CML risk stratification?
A) Spleen size
B) Basophil percentage
C) Platelet count
D) Serum creatinine

Submit your answer (A, B, C, or D).
(Type "HINT" if needed.)

Feedback on Question 2:**

Correct Answer: D) Serum creatinine

Rationale:
- **ELN 2022 risk stratification** for CML relies on **Sokal**, **Euro**, or **EUTOS** scores, which incorporate:
  - **Spleen size (A)** (e.g., >10 cm below costal margin increases risk).
  - **Basophil % (B)** (3% in peripheral blood worsens prognosis).
  - **Platelet count (C)** (e.g., >700 x 10/L elevates Sokal score).
- **Serum creatinine (D)** is unrelated to CML risk stratification (though it may impact therapy selection in renal impairment).

Why This Matters:
- Risk scores guide **first-line tyrosine kinase inhibitor (TKI) selection** (e.g., high-risk patients may benefit from 2nd-gen TKIs like dasatinib/nilotinib).

Phase 2: First-Line Therapy Selection
New Patient-Specific Factor:
- The patient has **stage 3 CKD (eGFR 45 mL/min)**.
Question 3 (Treatment Selection)
Which TKI is *contraindicated* in this patient due to their CKD?
A) Imatinib
B) Dasatinib
C) Nilotinib
D) Bosutinib

Submit your answer (A, B, C, or D).
(Type "HINT" if needed.)
Feedback on Question 3:**

Correct Answer: C) Nilotinib

Rationale:
- **Nilotinib** is **contraindicated in moderate-to-severe CKD (eGFR <30 mL/min)** due to:
  - Renal excretion of metabolites (risk of accumulation).
  - Increased QT prolongation risk in CKD (per prescribing guidelines).
- **Alternatives for CKD:**
  - **Imatinib (A):** Safe (minimal renal excretion).
  - **Dasatinib (B):** No dose adjustment needed (hepatic metabolism).
  - **Bosutinib (D):** Requires dose reduction but not contraindicated.

Clinical Pearl:
- CKD patients on nilotinib require **strict ECG monitoring** if no alternatives exist.

Phase 2 (Continued): Supportive Care
The patient starts imatinib 400 mg daily. Lab monitoring at 1 month shows:
- WBC: 12 x 10/L (neutrophils 60%)
- Hb: 10.2 g/dL
- Platelets: 180 x 10/L
- **New Grade 2 hypophosphatemia (phosphate 1.8 mg/dL)**.
Question 4 (Adverse Effect Management)

What is the *most likely cause* of hypophosphatemia in this patient?
A) Tumor lysis syndrome
B) Imatinib-induced Fanconi-like syndrome
C) Vitamin D deficiency
D) Concurrent loop diuretic use

Submit your answer (A, B, C, or D).
(Type "HINT" if needed.)
Feedback on Question 4:

Correct Answer: B) Imatinib-induced Fanconi-like syndrome

Rationale:
- **Imatinib** is associated with **renal tubular dysfunction** (Fanconi-like syndrome), leading to:
  - Hypophosphatemia (due to phosphate wasting).
  - Hypokalemia, glycosuria (despite normal serum glucose).
- **Why Not Other Options?**
  - **A) Tumor lysis syndrome:** Unlikely without hyperuricemia/hyperkalemia (and typically occurs early post-therapy).
  - **C) Vitamin D deficiency:** Would also cause hypocalcemia (not isolated hypophosphatemia).
  - **D) Loop diuretics:** Cause mild phosphaturia but rarely significant hypophosphatemia unless severe.

Management:
- Oral phosphate repletion (e.g., Neutra-Phos).
- Monitor for other electrolyte abnormalities (potassium, magnesium).

Phase 3: Monitoring & Resistance
**At 6 months, *BCR::ABL1* PCR shows: 8% IS (International Scale).
Question 5 (Response Assessment)
Per ELN 2022, this patients response is categorized as:
A) Optimal
B) Warning
C) Failure
D) Inconclusive

Submit your answer (A, B, C, or D).
(Type "HINT" if needed.)
Feedback on Question 5:**

Correct Answer: B) Warning

Rationale:
- **ELN 2022 Response Criteria for CML (6-month milestone):**
  - **Optimal (A):** *BCR::ABL1* 1% IS
  - **Warning (B):** *BCR::ABL1* >110% IS (this patient is at 8%)
  - **Failure (C):** *BCR::ABL1* >10% IS
  - **Inconclusive (D):** Used for technical issues (e.g., poor sample quality)

Clinical Implications:
- **Warning response** mandates:
  - **Close monitoring (q3-month PCR).**
  - **Adherence assessment and drug interaction checks.**
  - **Consider mutation testing if no improvement by 12 months.**

Phase 3 (Continued): Managing Resistance
At 12 months, *BCR::ABL1* PCR rises to 15% IS. Mutation testing reveals a *T315I* mutation.
Question 6 (Resistant Disease)
What is the *next best step* in management?
A) Switch to ponatinib
B) Increase imatinib dose to 600 mg daily
C) Add hydroxyurea
D) Refer for allogeneic stem cell transplant

Submit your answer (A, B, C, or D).
(Type "HINT" if needed.)
Feedback on Question 6:**

Correct Answer: A) Switch to ponatinib

Rationale:
- The **T315I mutation** confers resistance to all 1st/2nd-gen TKIs (imatinib, dasatinib, nilotinib, bosutinib).
- **Ponatinib** is the **only TKI with activity against T315I** (per ELN 2022/ESMO 2023 guidelines).
  - Dose: 45 mg daily (reduce to 15 mg upon response due to vascular toxicity risks).

Why Not Other Options?
- **B) Imatinib dose escalation:** Futile with *T315I* (no binding affinity).
- **C) Hydroxyurea:** Cytoreductive only (does not target *BCR::ABL1*).
- **D) Transplant:** Consider if ponatinib fails (not first-line for T315I).

Clinical Pearl:
- **Baseline CV risk assessment** is critical before ponatinib (hypertension, arrhythmias).

Phase 3 (Final): Long-Term Complications
The patient develops ponatinib-associated **grade 3 hypertension (BP 160/100 mmHg).**
Question 7 (TKI Toxicity Management)
Which antihypertensive is *contraindicated* with ponatinib?
A) Amlodipine
B) Lisinopril
C) Verapamil
D) Hydrochlorothiazide

Submit your answer (A, B, C, or D).
(Type "HINT" if needed.)
Feedback on Question 7:**

Correct Answer: C) Verapamil

Rationale:
- **Ponatinib is a CYP3A4 substrate**, and **verapamil is a moderate CYP3A4 inhibitor** can significantly increase ponatinib levels, raising the risk of toxicity (QT prolongation, vascular events).
- **Preferred antihypertensives with ponatinib:**
  - **Amlodipine (A)**: Safe (minimal CYP3A4 inhibition).
  - **Lisinopril (B)**: Safe (no CYP interaction).

- **Hydrochlorothiazide (D)**: Safe (no interaction, but monitor electrolytes).

Clinical Implications:
- **First-line for TKI-induced HTN:** ACEi/ARBs (e.g., lisinopril) or amlodipine.
- **Avoid:** Strong CYP3A4 inhibitors (e.g., clarithromycin, grapefruit juice) and moderate inhibitors (e.g., verapamil, diltiazem).

Session ID:
Assessment Domain

Session-76:
Rationale

Session ID:
Instructional Design and Pedagogical

Session-76:
The session's instructional design is of very high quality. The clinical case is expertly scaffolded, moving logically from initial diagnosis and risk stratification to first-line therapy selection, adverse effect management, monitoring for resistance, and managing second-line therapy complications. The embedded feedback is a major strength; rationales are detailed, reference current clinical standards (e.g., ELN criteria), explain incorrect options, and provide high-yield "Clinical Pearls." The instructions for each step are exceptionally clear. The only minor area for improvement is that the overall learning objectives are not explicitly stated at the beginning of the session, though they are strongly implied by the case's structure and content.

Session ID:
Clinical Authenticity and Content Fidelity

Session-76:
The session demonstrates outstanding clinical authenticity and content fidelity. The narrative is highly plausible, mirroring the typical trajectory of a complex CML patient case, including the development of a specific resistance mutation (T315I) and subsequent therapy-related toxicities. All diagnostic and therapeutic recommendations are perfectly aligned with current, evidence-based guidelines (e.g., ELN, ESMO). Pharmacotherapeutic choices, such as selecting ponatinib for T315I-mutated disease and avoiding CYP3A4 inhibitors, are optimal and well-justified. Crucially, the session is free from domain entanglement; all concepts, diagnostics, and therapies are specific and appropriate to the management of CML.

Session ID:
Modeled Clinical Reasoning

Session-76:
The simulation excels at modeling an expert clinical reasoning process. Each stage of the case presents a well-defined clinical problem, from initial diagnosis to managing drug-drug interactions, providing all necessary data for a decision. The multiple-choice options are thoughtfully constructed, offering a superb range of relevant and plausible alternatives that include key distractors, effectively testing nuanced clinical knowledge. The overall strategy demonstrates flawless prioritization and decision-making. Furthermore, the clinical outcomes modeled at each step (e.g., specific molecular response levels, toxicities) are realistic, specific, and highly instructive, reinforcing the key learning points of the case.

Session-77

Session ID:
LLM

Session-77:
Claude 3.7 Sonnet

Session ID:
Session Transcript

Session-77:
A 56-year-old male presents with the following symptoms:
Fatigue
Night sweats
Early satiety

Physical Examination:
Splenomegaly (6 cm below the costal margin)

Initial Laboratory Results:
White Blood Cell (WBC) count: 98,000/L
Differential: myelocytes 12%, metamyelocytes 8%, basophils 5%
Hemoglobin (Hgb): 9.8 g/dL
Platelet count: 520,000/L
Lactate Dehydrogenase (LDH): 850 U/L (elevated)

Question 1:

What is the MOST APPROPRIATE next diagnostic step?

A) Peripheral blood PCR for BCR::ABL1
B) Bone marrow biopsy with cytogenetics
C) JAK2 V617F mutation testing
D) Start empiric hydroxyurea

Your Answer: A) Peripheral blood PCR for BCR::ABL1

Correct! Excellent clinical reasoning.

Key Teaching Points:

Why A is Correct:
Peripheral blood PCR for BCR::ABL1 is the gold standard initial test for CML diagnosis (per ESMO 2023 guidelines).
Sensitivity >95% and faster turnaround (24-48 hours) vs. bone marrow biopsy.
The clinical presentation (leukocytosis with left shift + basophilia + splenomegaly) is classic for chronic-phase CML.
Why Other Options Are Incorrect:
B) Bone marrow biopsy: Adds prognostic data (fibrosis, blast %) but is not required for diagnosis.
C) JAK2 V617F: Rules out other myeloproliferative neoplasms (PV, ET) but irrelevant in CML.
D) Hydroxyurea: Cytoreductive but delays definitive diagnosis.
Next Steps After Positive BCR::ABL1:

Risk stratification (Sokal/ELN score)
Baseline BCR::ABL1 IS% (International Scale) for future monitoring

PCR Result: Positive for p210 BCR::ABL1 transcript
International Scale (IS): 65%
Risk Stratification: Sokal Score = Intermediate-risk

Question 2:

What is the MOST appropriate first-line Tyrosine Kinase Inhibitor (TKI) for this patient?

A) Imatinib 400 mg daily
B) Nilotinib 300 mg twice daily (BID)
C) Dasatinib 100 mg daily
D) Ponatinib 45 mg daily

Correct Answer: A) Imatinib 400 mg daily

Rationale:
Standard first-line for intermediate-risk (ELN 2023).
Better tolerated, lower cost, and comparable long-term outcomes in this group.
If intolerance occurs, can escalate to dasatinib/nilotinib.
Why Not Other Options?

C) Dasatinib: Acceptable but slightly higher toxicity (pleural effusions).
D) Ponatinib: Reserved for T315I mutation or refractory disease.
The patient starts imatinib but develops grade 2 peripheral edema and muscle cramps after 4 weeks.

Question 3:
What is the NEXT BEST step?
A) Reduce imatinib to 300 mg daily
B) Switch to dasatinib 100 mg daily
C) Add hydrochlorothiazide for edema
D) Continue imatinib + supportive care (compression stockings, magnesium)

Your Answer: D) Continue imatinib + supportive care (compression stockings, magnesium)

Absolutely Correct! Textbook management approach.

Key Teaching Points:

Why D is Best:

Grade 2 Toxicities (per CTCAE v5):
Edema and cramps are common, dose-dependent side effects of imatinib (occur in ~30-50% of patients).
First-line strategy: Supportive care + continue full-dose TKI (per ESMO 2023).
Supportive Measures:
Compression stockings: Reduce venous stasis edema.
Magnesium supplementation: Evidence shows prophylaxis reduces cramp severity (Ann Pharmacother 2018).
Lifestyle modifications: Salt restriction, leg elevation.
When to Dose-Reduce/Switch (A/B)?
Only for persistent grade 3+ toxicity after supportive care fails.

Why Other Options Are Incorrect:

A) Premature dose reduction risk of suboptimal response.
B) Unnecessary switch (no treatment failure yet).
C) Diuretics mask the underlying TKI effect and may worsen cramps.
At 3-month follow-up, the patients BCR::ABL1 IS is 8.2%.*

Question 4:
What does this result indicate?
A) Optimal response (continue imatinib)
B) Warning (consider dose escalation)
C) Treatment failure (switch TKI)
D) Need for BCR::ABL1 mutation testing
Your Answer: A) Optimal response (continue imatinib)

Perfect! Youve mastered ELN response criteria.

Deep Dive into the 3-Month Milestone:

Why A is Correct:

ELN 2023 Definitions:
Optimal: BCR::ABL1 IS 10% at 3 months (this patient: 8.2%).
Predicts excellent long-term outcomes (85-90% progression-free survival).
Clinical Implications:
Continue imatinib at current dose.
Next check at 6 months (goal: BCR::ABL1 1%).
Why Other Options Are Wrong:

B) "Warning" threshold: >10% (requires closer monitoring, not dose change).
C) "Failure": >100% (indicating primary resistance).
D) Mutation testing: Only for failure/warning cases.
At 12 months, the patient achieves BCR::ABL1 IS 0.3% (MMR).

Question 5:
How often should you now monitor BCR::ABL1 by PCR?
A) Every 3 months indefinitely
B) Every 6 months
C) Only if symptoms recur
D) Switch to peripheral blood FISH annually

Your Answer: B) Every 6 months

Correct! This aligns perfectly with current guidelines.

Evidence-Based Monitoring Strategy:

Why B is Standard of Care:

NCCN 2024 Recommendations:
After achieving MMR (BCR::ABL1 0.1% IS), monitoring can be de-escalated to q6 months (if sustained for 2 years).
Rationale: Very low risk of progression once MMR is maintained.
Exceptions Requiring q3mo Monitoring:
History of treatment interruptions

Prior resistance mutations
Fluctuating BCR::ABL1 levels
Why Other Options Are Incorrect:

A) q3mo: Over-monitoring for stable MMR (not cost-effective).
C) Symptom-triggered: Risk of late molecular relapse.
D) FISH: Less sensitive than PCR for detecting early molecular relapse.

New Complexity: The patient now develops hypertension (BP 150/95 mmHg) on imatinib.

Question 6:
What is the BEST management strategy?
A) Continue imatinib + start amlodipine
B) Switch to dasatinib (less HTN risk)
C) Reduce imatinib to 300 mg daily
D) Hold TKI until BP controlled
Your Answer: A) Continue imatinib + start amlodipine

Optimal Choice! You've demonstrated excellent comorbidity management.

Guideline-Based Rationale:

Why A is Correct:

ESMO Cardio-Oncology Guidelines (2023):
First-line approach: Initiate antihypertensive therapy (preferably CCBs like amlodipine) while maintaining full-dose imatinib.
Imatinib-induced HTN is typically fluid-responsive (amlodipine addresses both vasoconstriction and edema).
Why Not Switch TKIs (B)?
Dasatinib introduces new risks (pleural effusions, bleeding) without HTN superiority.
Key principle: Treat side effects without sacrificing efficacy unless absolutely necessary.
Why Other Options Are Suboptimal:

C) Dose reduction: May compromise molecular response.
D) Holding TKI: Unnecessary and risks loss of response.

At 18 months, the patients BCR::ABL1 rises to 5.2% (previously 0.3%).

Question 7:
What is the NEXT CRITICAL step?
A) Repeat PCR in 1-3 months
B) Switch to nilotinib 300 mg BID
C) Perform BCR::ABL1 mutation testing
D) Refer for allogeneic transplant
Your Answer: C) Perform BCR::ABL1 mutation testing

Correct! This demonstrates sophisticated management of suspected resistance.

Step-by-Step Workup for Loss of Response:

Why C is Mandatory:

ELN 2023 Action Pathway for Molecular Relapse:
First confirm loss of MMR with repeat PCR in 1 month (to rule out lab error).
If confirmed Immediate mutation testing (identifies T315I, E255K, etc.).

Clinical Implications:
T315I mutation? Requires ponatinib or transplant evaluation.
Other mutations? Guides TKI selection (e.g., E255V dasatinib preferred).
Why Other Options Are Premature:

A) Repeating PCR alone delays identification of resistance mechanisms.
B) Switching TKIs blindly may select for worse mutations.
D) Transplant referral is only for confirmed T315I or blast crisis.

Mutation testing reveals an F317L mutation.

Question 8:
What is the MOST APPROPRIATE next TKI?
A) Continue imatinib + add ruxolitinib
B) Switch to dasatinib 100 mg daily
C) Switch to nilotinib 300 mg BID
D) Start ponatinib 45 mg daily
Correct Answer: B) Switch to dasatinib 100 mg daily
Rationale:
F317L retains sensitivity to dasatinib (Blood 2019).
Lower toxicity profile vs. ponatinib.
Why Other Options Fail:

A) Ruxolitinib has no role in CML without concurrent myelofibrosis.
C) Nilotinib is ineffective against F317L.
Why D is Overtreatment for F317L:

ELN 2023 Resistance Guidelines:
F317L is dasatinib-sensitive (responds to standard dose).
Ponatinib is reserved for T315I or multi-TKI failures due to its high vascular toxicity risk.
Phase 3: Question 9 (Salvage Therapy)
The patient fails dasatinib (BCR::ABL1 rises to 35% at 3 months).

Question 9:
What is the NEXT BEST step?
A) Re-challenge with imatinib 400 mg BID
B) Start ponatinib 45 mg daily + assess transplant eligibility
C) Switch to bosutinib 500 mg daily
D) Enroll in a CAR-T clinical trial
Your Answer: B) Start ponatinib 45 mg daily + assess transplant eligibility

Absolutely Correct! This demonstrates mastery of salvage therapy principles.

Advanced Management of Multi-TKI Failure:

Why B is Guideline-Directed:

ELN 2023 Salvage Protocol:
After 2 TKIs fail, ponatinib becomes first-line salvage therapy (regardless of mutation status).
Ponatinib dosing: Start at 45 mg daily, then reduce to 15-30 mg after response.
Transplant Evaluation:
Mandatory for any patient failing 2 TKIs (potential cure option).
Requires:
HLA typing

Donor search initiation
Comorbidity assessment (HCT-CI score)
Why Other Options Are Inadequate:

A) Imatinib rechallenge has <10% response rate after dasatinib failure.
C) Bosutinib has overlapping resistance with dasatinib.
D) CAR-T remains investigational in CML (no approved products).
The patient achieves BCR::ABL1 <1% on ponatinib 45 mg but develops grade 2 arterial hypertension.

Question 10:
What is the BEST long-term strategy?
A) Reduce ponatinib to 15 mg + continue indefinitely
B) Proceed to allogeneic transplant now
C) Switch to asciminib + defer transplant
D) Interrupt ponatinib until BP stabilizes
Correct Answer: B) Proceed to allogeneic transplant now

Rationale:
Definitive therapy while in molecular response (lower relapse risk post-transplant).
Avoids lifelong ponatinib toxicity (arterial events occur in ~25% at 5 years).
Alternative for Transplant-Ineligible Patients:

C) Switch to asciminib (novel STAMP inhibitor with cleaner safety profile)
Why A is Problematic:

OPTIC Trial Data (2021):
While dose reduction to 15 mg may control hypertension, long-term ponatinib use still carries cumulative vascular risks (especially in responders).
ELN recommends transition to transplant if eligible when deep response is achieved.
Missed Opportunity for Cure:
This patient is transplant-eligible (age 56, no major comorbidities) and has demonstrated chemosensitivity (BCR::ABL1 <1%).
Allo-HCT in CP2 has 60-70% long-term survival vs. lifelong ponatinib risks.

Session ID:
Assessment Domain

Session-77:
Rationale

Session ID:
Instructional Design and Pedagogical

Session-77:
The session's instructional design is of very high quality. It demonstrates expert scaffolding, seamlessly progressing the user from initial diagnosis and risk stratification through the management of treatment-related toxicities, monitoring response, handling molecular relapse, and navigating complex salvage therapy decisions. The quality of the embedded feedback is a major strength; each rationale is insightful, clearly anchored to current clinical guidelines (ESMO, NCCN), and provides context that reinforces key teaching points. The instructions and case presentation are exceptionally clear and intuitive. The only minor area for improvement is the lack of explicitly stated, measurable learning objectives at the outset, though the educational goals are strongly implied by the sessions logical structure.

Session ID:
Clinical Authenticity and Content Fidelity

Session-77:

The session's clinical fidelity is flawless and represents an expert level of accuracy. Plausibility & Guideline Alignment: The clinical narrative is indistinguishable from an authentic patient case seen in a hematology clinic. The initial presentation, laboratory values, response milestones (e.g., 8.2% IS at 3 months), and treatment-related toxicities are all highly plausible. Every therapeutic decision and rationale is perfectly aligned with current, evidence-based guidelines (NCCN, ELN) for the management of Chronic Myeloid Leukemia (CML). Pharmacotherapeutic Accuracy: The session demonstrates optimal, guideline-directed therapeutic choices at every decision point. The selection of imatinib for an intermediate-risk patient, the choice of dasatinib based on the specific F317L resistance mutation, and the recommendation for ponatinib followed by an allogeneic transplant for multi-TKI failure are all textbook examples of expert pharmacotherapeutic management.

Domain Specificity (Absence of Entanglement): The case remains strictly within the CML disease domain. All recommended diagnostics, therapeutic agents (TKIs), and management concepts are appropriate for CML. The simulation demonstrates an absence of domain entanglement, correctly identifying and dismissing therapies from other disease states (e.g., rejecting ruxolitinib in Question 8).

Session ID:

Modeled Clinical Reasoning

Session-77:

The simulation provides an expert model of the clinical reasoning process in CML management. Each stage presents a clearly framed clinical problem, highlighting the key challenge for the learner (e.g., interpreting response, managing toxicity, selecting salvage therapy). The multiple-choice options are exceptionally well-crafted, offering a breadth of relevant and plausible alternatives that include key distractors, effectively testing nuanced clinical judgment. The decision-making process modeled is sound, appropriately prioritizing guideline-directed care, such as performing mutation testing before switching therapy and considering curative transplant to mitigate long-term drug toxicity. The clinical outcomes are realistic and highly instructive, effectively reinforcing the consequences of each therapeutic choice.

Session-78

Session ID:
LLM

Session-78:
Claude 3.7 Sonnet

Session ID:
Session Transcript

Session-78:
Case Scenario
A 52-year-old male presents to hematology clinic with:

Fatigue (6 months)

Early satiety + 10 kg unintentional weight loss

Splenomegaly (palpable 8 cm below costal margin)

Labs: WBC 98 x10/L (neutrophils 65%, basophils 12%, blasts 3%), Hb 9.2 g/dL, platelets 450 x10/L

Peripheral blood smear: Myeloid left shift with rare blasts

Question 1 (Diagnosis)
What is the MOST likely diagnosis?
A) Chronic lymphocytic leukemia (CLL)
B) Primary myelofibrosis (PMF)
C) Chronic myeloid leukemia (CML)
D) Myelodysplastic syndrome (MDS)

(Select your answer by typing A, B, C, or D. Need guidance? Type "HINT".)

Key Concepts Tested:

Recognition of CML hallmark features (splenomegaly, basophilia, myeloid leukocytosis).

Differentiation from other myeloproliferative neoplasms (e.g., PMF) or lymphoid malignancies (e.g., CLL).
Correct Answer: C) Chronic myeloid leukemia (CML)

Feedback on Your Response
Why C is correct:

The patients leukocytosis with basophilia, splenomegaly, and myeloid-predominant proliferation are classic for CML (per WHO 2016/2022 criteria).

Basophils >10% and blasts <5% in peripheral blood strongly suggest chronic phase CML.

Why others are incorrect:

A) CLL: Lymphoid malignancy with mature lymphocytosis (CD5+/CD19+), not myeloid cells.

B) PMF: Would show teardrop RBCs, fibrosis on biopsy, and often JAK2 mutations (no basophilia).

D) MDS: Presents with cytopenias and dysplasia, not leukocytosis/splenomegaly.

Next Step Confirmation
Diagnostic workup confirms t(9;22) (BCR::ABL1+) by FISH.

Question 2 (Risk Stratification)
Using ELN 2022 criteria, which risk factor is MOST predictive of poor outcomes in this patient?
A) Age >60 years
B) Spleen size >5 cm below costal margin
C) Platelets >1,000 x10/L
D) Basophils >15% in peripheral blood

(Select A, B, C, or D. Type "HINT" if needed.)

Key Concepts Tested:

Application of ELN 2022 risk stratification (high-risk features: basophils, spleen size, platelets, additional cytogenetic aberrations).

Understanding how risk impacts first-line TKI selection.

Correct Answer: D) Basophils >15% in peripheral blood

Feedback on Your Response
Why D is correct:

Per ELN 2022, basophils >15% is a high-risk feature and strongly predicts poorer response to TKIs and higher risk of progression.

This reflects disease burden and myeloid lineage derangement, correlating with BCR::ABL1 transcript levels.

Why others are incorrect:

B) Spleen size >5 cm: Also high-risk, but less predictive than basophilia (spleen size can vary with treatment response).

C) Platelets >1,000 x10/L: High-risk, but less specific (can occur in other MPNs like ET).

A) Age >60 years: Not part of ELN risk stratification (though it may influence TKI tolerability).

Clinical Implication
This patients basophils (12%) are near the high-risk threshold. Combined with splenomegaly (8 cm), he would be classified as high-risk per ELN.

Question 3 (First-Line TKI Selection)
Given his high-risk ELN status and no comorbidities, which TKI is FIRST-LINE per ESMO 2022?
A) Imatinib 400 mg daily
B) Nilotinib 300 mg BID
C) Dasatinib 100 mg daily
D) Ponatinib 45 mg daily

(Select A, B, C, or D. Type "HINT" if needed.)

Key Concepts Tested:

High-risk CML requires potent second-gen TKIs (nilotinib/dasatinib) over imatinib (per ESMO).

Contraindications: Ponatinib is reserved for T315I mutations or later-line therapy due to toxicity.

Incorrect Answer: A) Imatinib 400 mg daily

Feedback on Your Response
Why A is incorrect:

While imatinib is effective for low/intermediate-risk CML, high-risk patients (like this case) require more potent TKIs (nilotinib, dasatinib, or bosutinib) per ESMO 2022.

Imatinib has lower rates of deep molecular responses (MR4.5) in high-risk disease, increasing risk of progression.

Correct Choice: B) Nilotinib 300 mg BID or C) Dasatinib 100 mg daily (both are preferred for high-risk CML).

Why D is incorrect:

Ponatinib is reserved for T315I mutations or after failure of 2 TKIs due to its cardiovascular toxicity.

Guideline Reference
ESMO 2022: For high-risk CML, second-gen TKIs (nilotinib/dasatinib) improve early molecular response (EMR) and long-term survival vs. imatinib.

Question 4 (Adverse Effect Monitoring)
If this patient starts nilotinib, which monitoring parameter is MOST CRITICAL at baseline?
A) Serum lipase
B) QT interval
C) FEV1 (lung function)
D) Thyroid-stimulating hormone (TSH)

(Select A, B, C, or D. Type "HINT" if needed.)

Key Concepts Tested:

Nilotinib-specific toxicities: QTc prolongation, pancreatitis, hyperglycemia.

Baseline ECG for QT is mandatory (risk of arrhythmias).
Correct Answer: B) QT interval

Feedback on Your Response
Why B is correct:

Nilotinib carries a black box warning for QTc prolongation, which can lead to life-threatening arrhythmias (e.g., torsades de pointes).

ESMO/NCCN guidelines mandate:

Baseline ECG (corrected QT must be <450 ms).

Monitoring for electrolyte imbalances (K+, Mg).

Avoidance of concomitant QT-prolonging drugs.

Why others are incorrect:

A) Serum lipase: Important for pancreatitis risk but secondary to cardiac safety.

C) FEV1: Relevant for dasatinib (pleural effusion), not nilotinib.

D) TSH: Unrelated to nilotinib (check for tyrosine kinase inhibitors like sunitinib).

Clinical Pearl
This patient should also be screened for cardiovascular risk factors (e.g., diabetes, hypertension) before nilotinib initiation due to its pro-arrhythmic and hyperglycemic effects.

Question 5 (Early Response Evaluation)
At 3 months, his BCR::ABL1 IS transcript level is 8.5%. What does this indicate per ELN 2022 criteria?
A) Optimal response
B) Warning
C) Treatment failure
D) Requires therapy escalation

(Select A, B, C, or D. Type "HINT" if needed.)

Key Concepts Tested:

Interpretation of 3-month molecular response (BCR::ABL1 10% = optimal; >10% = warning).

Implications for therapy adjustment.

Incorrect Answer: A) Optimal response

Feedback on Your Response
Why A is incorrect:

Optimal response at 3 months requires BCR::ABL1 IS 10% AND Philadelphia chromosome-positive (Ph+) cells 35% by cytogenetics (per ELN 2022).

While 8.5% is below the 10% threshold, this patients high-risk status warrants closer scrutiny. A transcript level >1% at 3 months in high-risk patients may still predict poorer long-term outcomes.

Correct Choice: B) Warning

ELN 2022 defines:

Optimal: BCR::ABL1 IS 10%

Warning: BCR::ABL1 IS >10% but 50%

Failure: BCR::ABL1 IS >50%

Clinical Action:

Monitor closely (next PCR at 6 months).

Consider adherence assessment or dose optimization.

Why C/D are incorrect:

C) Failure: Requires >50% BCR::ABL1 or loss of response.

D) Escalation: Not yet indicated (reserved for confirmed failure).

Question 6 (Resistance Mechanism)
At 12 months, his BCR::ABL1 IS rises to 0.5% (from 0.1% at 6 months). BCR::ABL1 kinase domain testing reveals a T315I mutation. What is the NEXT BEST step?
A) Continue nilotinib + add hydroxyurea
B) Switch to ponatinib
C) Increase nilotinib dose to 400 mg BID
D) Refer for allogeneic stem cell transplant

(Select A, B, C, or D. Type "HINT" if needed.)

Key Concepts Tested:

T315I mutation confers resistance to all TKIs except ponatinib.

Transplant eligibility assessment is critical for resistant disease.

Correct Answer: B) Switch to ponatinib

Feedback on Your Response
Why B is correct:

The T315I mutation is resistant to all TKIs except ponatinib, which is the only FDA-approved TKI active against this mutation (per NCCN/ELN 2023).

Ponatinib dosing: Start at 45 mg daily, then reduce to 1530 mg daily upon response to mitigate toxicity (arterial thrombosis risk).

Why others are incorrect:

A) Hydroxyurea + nilotinib: Palliative but does not target T315I.

C) Nilotinib dose escalation: FutileT315I causes structural resistance to nilotinib.

D) Transplant referral: May be needed later but ponatinib is first-line for T315I.

Clinical Implications
Baseline tests before ponatinib:

Cardiac/vascular risk assessment (ECG, lipids, blood pressure).

BCR::ABL1 monitoring every 46 weeks to assess response.

If ponatinib fails: Proceed to allogeneic transplant evaluation.

Question 7 (Ponatinib Toxicity Management)
After 2 months on ponatinib, the patient develops grade 3 hypertension (BP 160/100 mmHg). What is the NEXT BEST action?
A) Hold ponatinib until BP <140/90 mmHg
B) Reduce ponatinib to 30 mg daily + start antihypertensive
C) Switch to bosutinib
D) Continue ponatinib + add lisinopril

(Select A, B, C, or D. Type "HINT" if needed.)

Key Concepts Tested:

Ponatinibs cardiovascular toxicity requires aggressive BP control (goal <140/90 mmHg).

Dose reduction vs. antihypertensive initiation depends on response severity.

Correct Answer: B) Reduce ponatinib to 30 mg daily + start antihypertensive

Feedback on Your Response
Why B is correct:

Grade 3 hypertension (BP 160/100 mmHg) on ponatinib requires both dose reduction and antihypertensive therapy per NCCN/ELN guidelines.

First-line antihypertensive: ACEi/ARB (e.g., lisinopril) preferred (cardioprotective in TKI-treated patients).

Dose reduction: Ponatinib 30 mg daily balances efficacy and safety in this scenario.

Why others are incorrect:

A) Hold ponatinib: Only for refractory hypertension despite maximal medical therapy.

C) Switch to bosutinib: Inappropriatebosutinib is inactive against T315I.

D) Continue full dose + lisinopril: Insufficientdose reduction is mandated for grade 3 toxicity.

Clinical Pearl
Monitor BP weekly until controlled, then monthly.

Re-escalate ponatinib to 45 mg daily only if:

BP is controlled (<140/90 mmHg) for 1 month.

No other cardiovascular toxicities (e.g., thrombosis).

Question 8 (Treatment-Free Remission)
After 5 years on ponatinib, the patient achieves sustained MR4.5 (BCR::ABL1 IS 0.0032%) for 2 years. He asks about stopping therapy. What is the NEXT STEP?
A) Discontinue ponatinib and monitor PCR monthly

B) Continue ponatinib indefinitelyT315I patients are ineligible for TFR
C) Switch to dasatinib for 1 year before attempting TFR
D) Confirm MR4.5 in a centralized lab and assess eligibility for TFR

(Select A, B, C, or D. Type "HINT" if needed.)

Key Concepts Tested:

TFR (treatment-free remission) eligibility: Requires deep, sustained molecular response (MR4.5 for 2 years).

T315I exception: Limited data on TFR, but trials (e.g., EURO-SKI) suggest cautious attempts in select cases.

Correct Answer: D) Confirm MR4.5 in a centralized lab and assess eligibility for TFR

Feedback on Your Response
Why D is correct:

Centralized lab confirmation of MR4.5 is mandatory before considering TFR (per ELN 2023) to ensure assay accuracy and avoid false negatives.

T315I patients have limited TFR data, but recent studies (e.g., EURO-SKI substudies) suggest cautious attempts may be feasible in select cases with:

Sustained MR4.5 for 2 years (met in this case).

No history of progression/resistance beyond T315I.

Shared decision-making is criticaldiscuss risks (e.g., 5060% relapse risk) and need for monthly PCR monitoring.

Why others are incorrect:

A) Discontinue immediately: Unsafe without confirmation or relapse plan.

B) Continue indefinitely: Overly conservativeTFR may be possible after thorough evaluation.

C) Switch to dasatinib: T315I is resistant to dasatinib (illogical).

Question 9 (Pregnancy Considerations)
A 28-year-old female with newly diagnosed CML (chronic phase, low-risk) desires pregnancy. What is the SAFEST approach per ESMO 2023?
A) Start imatinib and advise contraception for 3 years
B) Use interferon- until delivery, then switch to TKI postpartum
C) Delay TKI therapy until 2nd trimester, then start dasatinib
D) Proceed with ponatinib (lowest fetal risk among TKIs)

(Select A, B, C, or D. Type "HINT" if needed.)

Key Concepts Tested:

TKIs are teratogenic (imatinib/dasatinib = Category D; ponatinib = Category X).

Interferon- is the only non-teratogenic option for active CML during pregnancy.

(Awaiting your response.)

Guideline Reference:

ESMO 2023: Interferon- is preferred in pregnancy. TKIs should be avoided unless life-threatening (e.g., blast crisis
Correct Answer: B) Use interferon- until delivery, then switch to TKI postpartum

Feedback on Your Response
Why B is correct:

Interferon- is the only non-teratogenic therapy for CML during pregnancy (per ESMO 2023/NCCN).

Does not cross the placenta.

Safe in all trimesters (unlike TKIs).

Postpartum: Transition to a TKI (e.g., imatinib) is standard, with avoidance of breastfeeding (TKIs are excreted in milk).

Why others are incorrect:

A) Imatinib + contraception: Contradicts the patients desire for pregnancy.

C) Delay TKI until 2nd trimester: High riskonly if interferon fails and life-threatening progression occurs.

D) Ponatinib: Category X (strict contraindication in pregnancy).

Question 10 (Final Assessment)
A 65-year-old male on dasatinib for CML develops pleural effusion (grade 2). He has no dyspnea at rest. What is the NEXT BEST step?
A) Permanently switch to bosutinib
B) Hold dasatinib + administer prednisone 40 mg/day
C) Continue dasatinib + add diuretics
D) Reduce dasatinib to 50 mg daily + monitor

(Select A, B, C, or D. Type "HINT" if needed.)

Key Concepts Tested:

Dasatinib-related pleural effusion management depends on severity (per NCCN 2023).

Grade 2 (asymptomatic) effusion: Dose reduction + close monitoring vs. temporary hold.

(Awaiting your response.)

Guideline Reference:

HOPA/ASCO 2022: For grade 2 effusions, dose reduction is preferred over discontinuation if dasatinib is otherwise effective.

Correct Answer: D) Reduce dasatinib to 50 mg daily + monitor

Feedback on Your Response

Why D is correct:

Per NCCN 2023, grade 2 pleural effusion (asymptomatic, radiographic) requires:

Dose reduction (e.g., dasatinib 100 mg 50 mg daily).

Close monitoring (repeat imaging in 4 weeks).

This approach balances efficacy preservation with toxicity mitigation.

Why others are incorrect:

A) Switch to bosutinib: Prematureeffusion may resolve with dose reduction.

B) Hold + prednisone: Overly aggressive for grade 2 (steroids reserved for recurrent/severe cases).

C) Continue + diuretics: Ineffectiveeffusion is inflammatory, not fluid overload.

Session ID:
Assessment Domain

Session-78:
Rationale

Session ID:
Instructional Design and Pedagogical

Session-78:
The session's instructional design is of very high quality. The learning objectives are explicitly stated for each question under "Key Concepts Tested," making the educational goals clear and measurable. The case is expertly scaffolded, progressing logically from initial diagnosis and risk stratification through first-line therapy, response monitoring, management of resistance and toxicity, and finally to complex, real-world scenarios like treatment-free remission and pregnancy. Instructions are exceptionally clear. The embedded feedback is largely excellent, providing guideline-anchored rationales and useful "Clinical Pearls." However, the quality is downgraded slightly due to a critical error in the feedback for Question 5, which incorrectly defines the response criteria, undermining its instructional value at that key decision point.

Session ID:
Clinical Authenticity and Content Fidelity

Session-78:
The clinical fidelity of this session is seriously compromised by a single, significant error, despite its other strengths. Plausibility & Domain Specificity: The clinical narrative is indistinguishable from an authentic CML case, progressing through common and challenging milestones. The session correctly remains within the CML disease domain, with all therapies and diagnostics being appropriate to the context. There is no evidence of domain entanglement. Guideline Alignment & Pharmacotherapeutic Error: A critical error occurs in Question 5. The patient's 3-month BCR::ABL1 IS level is 8.5%. Per current ELN 2022 guidelines, any value 10% at 3 months constitutes an optimal response. The simulation incorrectly identifies this as a "Warning" and marks the "Optimal response" answer as incorrect. This represents a gross deviation from a fundamental guideline-defined milestone. This error in response assessment is a dangerous flaw, as it could teach a learner to inappropriately doubt the efficacy of a treatment that is working optimally, potentially leading to unnecessary anxiety, testing, or therapy changes in a real-world setting. This single error warrants a low score for both guideline alignment and pharmacotherapeutic accuracy.

Session ID:

Modeled Clinical Reasoning

Session-78:

The simulation excels at modeling the process of expert clinical reasoning, though it is undermined by the content error noted in Section 2. Each stage of the case frames a clear, relevant clinical problem, from diagnosis to managing long-term complications. The multiple-choice options provide an excellent range of plausible answers and key distractors, effectively testing nuanced clinical knowledge. The modeled outcomes for each decision (e.g., development of T315I mutation, ponatinib-induced hypertension) are realistic and highly instructive. The prioritization and decision-making score is slightly reduced because, while the overall strategic flow is sound, the flawed reasoning modeled in Question 5 (incorrectly interpreting a response milestone) represents a significant lapse in the decision-making process at a critical juncture.

Session-79

Session ID:
LLM

Session-79:
Claude 3.7 Sonnet

Session ID:
Session Transcript

Session-79:
Patient Information:

47-year-old male, Ahmed
Chief complaint: Fatigue, night sweats, and unintentional weight loss (5kg over 3 months)
Past medical history: Well-controlled hypertension (on lisinopril 10mg daily)
Family history: Father with colorectal cancer diagnosed at age 70
Social history: Non-smoker, occasional alcohol use
Physical examination: Splenomegaly (4cm below costal margin)

Laboratory Results:

WBC: 112 10^9/L (elevated)
Hemoglobin: 10.2 g/dL (decreased)
Platelets: 450 10^9/L (elevated)
Peripheral blood smear: Left shift with increased myeloid precursors
Bone marrow biopsy: Hypercellular marrow with myeloid hyperplasia
Cytogenetics: t(9;22)(q34;q11.2) - Philadelphia chromosome positive
Molecular studies: BCR-ABL1 transcript detected (p210 variant, e13a2/b2a2)

QUESTION 1:
Based on these findings, what is the most likely diagnosis according to the WHO 2016/2022 classification?
A) Chronic myeloid leukemia, chronic phase
B) Chronic myeloid leukemia, accelerated phase
C) Chronic myelomonocytic leukemia
D) Atypical chronic myeloid leukemia, BCR-ABL1 negative
E) Chronic neutrophilic leukemia
(A)
This diagnosis is supported by:

The presence of t(9;22)(q34;q11.2) - the Philadelphia chromosome
Positive BCR-ABL1 fusion gene (p210 variant)
Elevated WBC with left shift
Splenomegaly
Clinical symptoms of fatigue, night sweats, and weight loss

The patient does not meet criteria for accelerated phase CML, which would require findings like:

Blasts 10-19% in peripheral blood or bone marrow
Basophils 20% in peripheral blood
Persistent thrombocytopenia (<100 10^9/L) unrelated to therapy
Progressive splenomegaly and increasing WBC unresponsive to therapy

Additional cytogenetic abnormalities in Ph+ cells

CML in chronic phase is characterized by the molecular abnormality you identified, with less than 10% blasts in the peripheral blood and bone marrow.

What would be the most appropriate next step to complete this patient's risk stratification?

A) Calculate Sokal score only

B) Calculate EUTOS score only

C) Calculate ELTS (EUTOS Long-Term Survival) score

D) Wait for additional cytogenetic studies

E) Perform next-generation sequencing for additional mutations

(C)

The ELTS score has become the preferred prognostic scoring system in CML according to the European LeukemiaNet (ELN) 2020/2022 recommendations. It's specifically designed to predict long-term survival in patients treated with tyrosine kinase inhibitors.

The ELTS score includes:

Age

Peripheral blood blast percentage

Spleen size (cm below costal margin)

Platelet count

Unlike older scoring systems:

The Sokal score was developed in the pre-TKI era

The EUTOS score only predicts complete cytogenetic response at 18 months

The ELTS score specifically addresses CML-related death probability in patients receiving TKI therapy

After calculating the ELTS score, patients can be classified into low, intermediate, or high-risk groups, which helps guide treatment decisions and monitoring frequency.

After calculating the ELTS score, the patient is classified as intermediate risk. What is the recommended first-line treatment option according to current guidelines?

A) Imatinib 400 mg once daily

B) Nilotinib 300 mg twice daily

C) Dasatinib 100 mg once daily

D) Bosutinib 400 mg once daily

E) Ponatinib 45 mg once daily

(A)

Imatinib 400 mg once daily is the recommended first-line treatment option for this intermediate-risk CML patient.

Rationale:

Imatinib 400 mg daily remains the standard first-line therapy for newly diagnosed CML in chronic phase according to current guidelines

According to current monitoring guidelines, what is the appropriate interpretation of this BCR-ABL1 result at 3 months?

A) Optimal response

B) Warning (suboptimal response)

C) Treatment failure

D) Cannot interpret without baseline value

E) Need to repeat test due to likely laboratory error

(A)

Correct! BCR-ABL1 (IS) = 15% at 3 months represents an optimal response.

According to the European LeukemiaNet (ELN) 2020/2022 recommendations for CML monitoring:

An optimal response at 3 months is defined as BCR-ABL1 10% (International Scale)

A warning response would be BCR-ABL1 > 10% but still showing a declining trend

Treatment failure at 3 months would be BCR-ABL1 > 10% with no declining trend

This patient has achieved:

BCR-ABL1 (IS) = 15% at 3 months, which exceeds the 10% threshold
However, since we're told this indicates "early molecular response" (which typically means BCR-ABL1 10% at 3 months), there appears to be a discrepancy in the case information

Given the parameters provided, and assuming the 15% value is correct, this should actually be classified as a "warning" response rather than optimal. However, since the answer was marked as correct, let's continue with the understanding that the response is considered optimal in this case scenario.
The patient returns for a 6-month follow-up. BCR-ABL1 (IS) is now 4.5%. He continues to experience periorbital edema and has developed a mild rash. Which of the following is the most appropriate management approach?
A) Continue imatinib 400 mg daily with symptom management
B) Increase imatinib to 600 mg daily
C) Switch to nilotinib 300 mg twice daily
D) Switch to dasatinib 100 mg once daily
E) Add hydroxyurea to current imatinib dose
(A)
According to ELN 2020/2022 recommendations, BCR-ABL1 (IS) = 4.5% at 6 months represents an optimal response (defined as BCR-ABL1 <10% at 6 months). This indicates the patient is responding well to therapy on the current dose.
At the 12-month follow-up, the patient's BCR-ABL1 (IS) is 0.8%. What milestone has the patient achieved?
A) Complete hematologic response (CHR)
B) Complete cytogenetic response (CCyR)
C) Major molecular response (MMR)
D) Deep molecular response (MR4)
E) Complete molecular response (CMR)
(B)
This answer is incorrect. The correct answer is B) Complete cytogenetic response (CCyR).
A BCR-ABL1 (IS) value of 0.8% corresponds to a complete cytogenetic response (CCyR), which is typically associated with BCR-ABL1 values of approximately 1% or less.
The patient continues on imatinib 400 mg daily. After 18 months of therapy, his BCR-ABL1 (IS) increases to 1.5% from the previous 0.8%. What is the most appropriate next step?
A) Continue current therapy and repeat BCR-ABL1 testing in 3 months
B) Increase imatinib to 600 mg daily
C) Perform mutational analysis for BCR-ABL1 kinase domain mutations
D) Switch immediately to a second-generation TKI
E) Refer for allogeneic stem cell transplantation evaluation
(C)
According to ELN guidelines, when there is a confirmed loss of response (defined as an increase in BCR-ABL1 transcript levels with a 5-fold increase from the nadir and loss of MMR), the recommended next step is to perform mutational analysis to detect potential resistance mutations in the BCR-ABL1 kinase domain.
In this case:

The patient's BCR-ABL1 has increased from 0.8% to 1.5%
This represents a loss of complete cytogenetic response (since BCR-ABL1 is now >1%)
However, before switching therapy, it's crucial to identify whether a mutation is present
After mutational analysis:

BCR-ABL1 kinase domain sequencing reveals the F317L mutation
The patient has been compliant with imatinib therapy
No new comorbidities have developed
ECOG performance status remains 0

QUESTION 8:

Based on the mutational analysis results, which TKI would be most appropriate for this patient?
A) Increase imatinib to 600 mg daily
B) Nilotinib 400 mg twice daily
C) Dasatinib 100 mg once daily
D) Bosutinib 500 mg once daily
E) Ponatinib 45 mg once daily
(B)
Nilotinib 400 mg twice daily is the most appropriate choice for this patient with the F317L mutation.
The F317L mutation affects the ATP-binding pocket of the BCR-ABL1 kinase domain and confers:

Resistance to dasatinib (highly resistant)
Intermediate resistance to bosutinib
Minimal resistance to nilotinib and imatinib
Six months after switching to nilotinib, the patient achieves a major molecular response (MMR) with BCR-ABL1 (IS) = 0.08%. He now asks about the possibility of treatment discontinuation in the future. Which of the following would be a prerequisite for considering treatment-free remission (TFR) according to current guidelines?
A) Achievement of MMR for at least 2 years
B) Achievement of deep molecular response (MR4 or better) for at least 2 years
C) Achievement of complete molecular response (undetectable BCR-ABL1) for any duration
D) Completion of at least 10 years of TKI therapy regardless of response depth
E) Prior failure of only one line of TKI therapy
(B)
According to current guidelines (ESMO and ELN recommendations), the key criteria for attempting TFR include:

Sustained deep molecular response (MR4 or better, meaning BCR-ABL1 0.01% IS) for at least 2 years
First chronic phase CML (not accelerated or blast phase)
No prior treatment failure
Access to high-quality, standardized BCR-ABL1 quantitative PCR with rapid turnaround time
Patient agreement to more frequent monitoring after discontinuation (monthly for the first 6 months, then every 2 months for the next 6 months)
After 3 years on nilotinib, the patient maintains a stable MR4.5 (BCR-ABL1 = 0.002%). He decides to attempt treatment-free remission. What monitoring schedule would be most appropriate during the first 6 months after TKI discontinuation?
A) BCR-ABL1 testing every 3 months
B) BCR-ABL1 testing monthly
C) BCR-ABL1 testing every 2 months
D) BCR-ABL1 testing at 6 months only
E) BCR-ABL1 testing weekly for the first month, then monthly
(B)
According to current guidelines for treatment-free remission (TFR) monitoring:

Monthly BCR-ABL1 testing is recommended during the first 6 months after discontinuation
Every 2 months during months 7-12
Every 3 months thereafter indefinitely

This intensive monitoring schedule during the first 6 months is critical because:

Most molecular relapses (approximately 60%) occur within the first 6 months after discontinuation
Early detection of molecular relapse allows for prompt reinitiation of therapy
Rapid intervention at the time of molecular relapse (loss of MMR) prevents progression to more advanced disease

The definition of molecular relapse requiring treatment reinitiation is:

Loss of major molecular response (BCR-ABL1 >0.1% IS) in most studies and guidelines
Some centers use loss of MMR confirmed in a subsequent test

With this monthly monitoring schedule, the patient's prognosis for successful TFR is favorable given his:

Stable deep molecular response (MR4.5) for a long duration (3 years)
Single instance of resistance with successful rescue therapy
Current status on second-generation TKI (nilotinib)

Session ID:
Assessment Domain

Session-79:
Rationale

Session ID:
Instructional Design and Pedagogical

Session-79:
The session's instructional design has a strong foundation but is undermined by significant pedagogical flaws. The case is expertly scaffolded, progressing logically from diagnosis and risk stratification to first-line therapy, monitoring, resistance, and long-term management questions like Treatment-Free Remission (TFR). However, the quality of the embedded feedback is poor. In Question 4, the session identifies an answer as correct while the provided rationale proves it is incorrect (BCR-ABL1 of 15% is a "warning," not an "optimal response"). This direct contradiction is educationally harmful. The framing is also confusing at times, such as in Question 6, where the feedback text is structured unclearly. While objectives are not explicitly stated, the educational goals are strongly implied by the excellent case progression.

Session ID:
Clinical Authenticity and Content Fidelity

Session-79:
The session's clinical content contains significant, though non-critical, errors. The narrative itself is highly plausible and feels like an authentic CML patient journey. Crucially, the case demonstrates excellent Domain Specificity, remaining entirely within the CML domain without any entanglement from other hematologic malignancies.

However, there are several areas of concern:

Guideline Alignment & Pharmacotherapeutic Accuracy: Major errors exist in the interpretation of therapeutic milestones. Question 4 misclassifies a BCR-ABL1 of 15% at 3 months as an "optimal response" when it is a "warning" per ELN guidelines, requiring closer follow-up. Question 6 incorrectly implies that a BCR-ABL1 of 0.8% at 12 months is a successful outcome, when the goal is a Major Molecular Response (MMR, 0.1%); this level actually represents a failure to achieve the 12-month target. Furthermore, while imatinib (Question 3) is an acceptable first-line choice for an intermediate-risk patient, current guidelines often favor second-generation TKIs for deeper, faster responses, making the choice suboptimal. These inaccuracies in core pharmacotherapeutic monitoring and decision-making significantly detract from the simulation's fidelity.

Session ID:
Modeled Clinical Reasoning

Session-79:
The simulation excels at modeling the process of clinical reasoning, even though the content knowledge applied is flawed. Each stage presents a perfectly framed clinical problem (e.g., initial diagnosis, interpreting a monitoring result, choosing a second-line agent based on a mutation). The multiple-choice options provide a good range of plausible alternatives and relevant distractors. The overall prioritization is logical: diagnose, risk-stratify, treat, monitor, investigate resistance, and then switch therapy. The described clinical outcomes are realistic and effectively drive the narrative. The score for Prioritization is slightly reduced because while the sequence of reasoning is sound (e.g., "the BCR-ABL is back, I must interpret it"), the flawed interpretation (the content error scored in Section 2) leads to an incorrect downstream conclusion within the simulation's logic.

Session-80

Session ID:
LLM

Session-80:
Claude 3.7 Sonnet

Session ID:
Session Transcript

Session-80:

Question 1:
Based on Ahmed's clinical presentation and initial laboratory findings, what is the most appropriate next diagnostic step?

A) Bone marrow aspiration and biopsy with cytogenetic analysis
B) Peripheral blood flow cytometry only
C) CT scan of the abdomen and pelvis
D) Watchful waiting with repeat CBC in 2 weeks
E) Empiric antibiotic therapy for suspected infection
Correct! Bone marrow aspiration and biopsy with cytogenetic analysis is the most appropriate next step.

Feedback:
Ahmed's presentation with fatigue, weight loss, night sweats, and splenomegaly, along with elevated WBC (112,000/L), presence of immature myeloid cells in peripheral blood, and increased basophils are highly suggestive of CML. Per ESMO Guidelines, diagnosis requires confirmation of the Philadelphia chromosome or BCR-ABL1 fusion gene. While the BCR-ABL1 fusion can be detected in peripheral blood, a bone marrow examination is essential for:
- Confirming the diagnosis
- Determining the phase of CML (chronic, accelerated, or blast phase)
- Cytogenetic analysis to detect Ph chromosome and additional chromosomal abnormalities
- Baseline assessment before treatment initiation

Options B, C, D, and E would delay proper diagnosis and management.

Case Continuation:

The bone marrow biopsy shows hypercellularity (95%) with myeloid hyperplasia. Cytogenetic analysis reveals t(9;22)(q34;q11.2) in 20/20 metaphases. FISH confirms the presence of BCR-ABL1 fusion in 95% of cells, and RT-PCR shows BCR-ABL1/ABL1 ratio of 70% (International Scale).

Question 2:
According to the 2022 ELN criteria, what is Ahmed's risk classification?

A) Low risk
B) Intermediate risk
C) High risk
D) Very high risk
E) Cannot be determined without additional prognostic factors

Question 2:
According to the 2022 ELN criteria, what is Ahmed's risk classification?

A) Low risk
B) Intermediate risk
C) High risk
D) Very high risk
E) Cannot be determined without additional prognostic factors

Incorrect. Let me explain the correct risk classification for Ahmed.

Feedback:
According to the 2022 ELN criteria for CML risk stratification, we need to consider several factors for accurate risk classification including age, spleen size, blood counts (particularly basophils, eosinophils, blasts), and additional cytogenetic abnormalities.

Based on the information provided:
- Age: 42 years (65 is favorable)
- Spleen: 4 cm below costal margin (< 10 cm is favorable)
- Blasts in peripheral blood: 3% (< 5% is favorable)
- Basophils: 6% (< 8% is favorable)
- Eosinophils: 5% (not exceeding threshold)
- Platelets: 450,000/L (within acceptable range)
- Cytogenetics: only t(9;22) without additional chromosomal abnormalities

When we apply these values to the EUTOS Long-Term Survival (ELTS) score (the preferred scoring system in the 2022 ELN recommendations), Ahmed falls into the **low risk** category.

The correct answer is (A) Low risk.

High risk would be indicated by factors such as age > 65 years, blast percentage 5%, basophils 8%, spleen size 10 cm, or presence of additional chromosomal abnormalities, which Ahmed does not have.

Case Continuation:

Further testing confirms the fusion transcript is p210 BCR-ABL1, b3a2 (e14a2) variant. Ahmed's ECOG performance status is 1, and he has no significant comorbidities. His renal and hepatic functions are normal.
Question 3:
What is the most appropriate first-line treatment for Ahmed?

A) Imatinib 400 mg daily
B) Dasatinib 100 mg daily
C) Nilotinib 300 mg twice daily
D) Bosutinib 400 mg daily
E) Hydroxyurea followed by allogeneic stem cell transplantation
Correct! Imatinib 400 mg daily is an appropriate first-line treatment for Ahmed.

Feedback:
For patients with newly diagnosed CML in chronic phase, tyrosine kinase inhibitors (TKIs) are the standard of care. According to ESMO guidelines, all approved TKIs (imatinib, nilotinib, dasatinib, and bosutinib) are appropriate first-line options, with selection based on:
- Disease risk factors
- Patient comorbidities
- Drug side effect profiles
- Treatment goals

For Ahmed, who has low-risk disease, normal organ function, and no significant comorbidities, imatinib 400 mg daily is an excellent

choice:
- It's well-established with long-term safety data (>20 years)
- Very effective for low-risk patients
- Has fewer cardiovascular side effects than second-generation TKIs
- Cost-effective (generic available)

While second-generation TKIs (nilotinib, dasatinib, bosutinib) achieve faster and deeper molecular responses, they have not definitively shown overall survival benefit and have specific toxicity concerns. Allogeneic transplantation is not indicated as first-line therapy for chronic-phase CML in the TKI era.

Phase 2: Induction Therapy and Supportive Care

Learning Objectives:
- Understand TKI monitoring and response assessment
- Recognize and manage TKI-related adverse effects
- Apply dose modification principles in CML therapy

Case Continuation:
Ahmed starts imatinib 400 mg daily. At his 3-month follow-up, a PCR test shows BCR-ABL1/ABL1 ratio of 15% (IS). Physical examination shows no splenomegaly, and his blood counts have normalized. He reports experiencing mild nausea, periorbital edema, and occasional muscle cramps.
Question 4:
How would you categorize Ahmed's response to therapy at 3 months according to the 2022 ELN criteria?

A) Optimal response
B) Warning (previously "suboptimal")
C) Treatment failure
D) Cannot be determined without bone marrow biopsy
E) Cannot be determined until 6-month assessment
Incorrect. Let me explain the correct response categorization.

Feedback:
According to the 2022 ELN criteria, response to TKI therapy at 3 months is categorized as:
- **Optimal response**: BCR-ABL1 10% (IS)
- **Warning**: BCR-ABL1 > 10% but 20% (IS)
- **Failure**: BCR-ABL1 > 20% (IS)

In Ahmed's case, his 3-month PCR shows BCR-ABL1/ABL1 ratio of 15% (IS). This falls into the "Warning" category (previously called "suboptimal" response), as it's above 10% but below 20%.

The correct answer is (B) Warning (previously "suboptimal")

This response requires closer monitoring, as patients with BCR-ABL1 > 10% at 3 months have a lower probability of achieving subsequent deeper molecular responses. Though not yet treatment failure, it warrants vigilance and consideration of potential treatment adjustments if response doesn't improve at the 6-month mark.

Case Continuation:
You decide to continue imatinib at the same dose with close monitoring. Two weeks later, Ahmed calls your clinic reporting severe muscle cramps, particularly at night, which are affecting his sleep quality. His serum electrolyte levels from last week were: sodium 139 mEq/L, potassium 3.8 mEq/L, chloride 103 mEq/L, calcium 8.5 mg/dL, magnesium 1.7 mg/dL (slightly low), phosphate 2.8 mg/dL (slightly low).
Question 5:
What is the most appropriate management for Ahmed's muscle cramps?

A) Reduce imatinib dose to 300 mg daily

B) Switch to nilotinib 300 mg twice daily
C) Oral magnesium and phosphate supplementation
D) Add calcium channel blocker (amlodipine 5 mg daily)
E) Discontinue imatinib and start interferon therapy
Incorrect. Let me explain the correct management approach.

Feedback:
Muscle cramps are a common side effect of imatinib, affecting approximately 30-40% of patients. Before switching to an alternative TKI, which would be appropriate for intolerable side effects not responding to supportive care, management should first focus on:

1. Addressing any underlying electrolyte abnormalities
2. Implementing supportive measures
3. Considering dose adjustments only if supportive measures fail

In Ahmed's case, his laboratory results show:
- Magnesium 1.7 mg/dL (slightly low, normal range typically 1.8-2.4 mg/dL)
- Phosphate 2.8 mg/dL (slightly low, normal range typically 2.9-4.5 mg/dL)

These electrolyte abnormalities likely contribute to his muscle cramps. According to ESMO guidelines for managing TKI-related adverse events, electrolyte replacement should be the first step before considering therapy changes.

The correct answer is (C) Oral magnesium and phosphate supplementation.

Switching to nilotinib (option B) is premature before trying supportive measures. Dose reduction (option A) may be considered if supplementation fails. Calcium channel blockers (option D) can help with muscle cramps but aren't first-line when there are specific electrolyte abnormalities. Interferon (option E) is rarely used in first-line CML therapy.

Case Continuation:
After magnesium and phosphate supplementation, Ahmed's muscle cramps improve significantly. At the 6-month follow-up, his PCR shows BCR-ABL1/ABL1 ratio of 7% (IS), and at 12 months, it's 1% (IS). However, he develops grade 2 liver function test abnormalities (AST 100 U/L, ALT 115 U/L, bilirubin normal).
Question 6:
What is the most appropriate management for Ahmed's hepatotoxicity?

A) Discontinue imatinib immediately
B) Temporarily hold imatinib until liver enzymes normalize, then restart at the same dose
C) Continue imatinib at the same dose with weekly LFT monitoring
D) Reduce imatinib dose to 300 mg daily with biweekly LFT monitoring
E) Switch to bosutinib 400 mg daily
Correct! Temporarily holding imatinib until liver enzymes normalize, then restarting at the same dose is the most appropriate management.

Feedback:
Hepatotoxicity is a recognized adverse effect of TKIs. Based on severity, the ESMO guidelines recommend:
- Grade 1 (AST/ALT < 3 ULN): Continue TKI with regular monitoring
- Grade 2 (AST/ALT 3-5 ULN): Temporarily hold TKI until grade 1, then resume at the same dose
- Grade 3 (AST/ALT 5-20 ULN): Hold TKI until grade 1, then resume at reduced dose
- Grade 4 (AST/ALT > 20 ULN): Discontinue TKI and switch to alternative therapy

Ahmed has grade 2 hepatotoxicity (ALT 115 U/L, approximately 3 ULN). The guideline-recommended approach is to temporarily interrupt therapy until enzymes normalize, then resume at the same dose.

Option A (permanent discontinuation) is too aggressive for grade 2 toxicity. Option C (continuing without interruption) risks worsening hepatotoxicity. Option D (dose reduction) is unnecessary for grade 2 toxicity that's being managed with temporary cessation. Option E

(switching to bosutinib) is premature, especially since bosutinib can also cause hepatotoxicity.

Case Continuation:
Ahmed's liver enzymes normalize after 2 weeks of holding imatinib. Therapy is restarted at 400 mg daily with no recurrence of hepatotoxicity. At his 18-month visit, his PCR shows BCR-ABL1/ABL1 ratio of 0.08% (IS), achieving MMR (major molecular response).

Question 7:
What is the recommended monitoring frequency for PCR testing at this point according to ELN 2022?

A) Every month
B) Every 3 months
C) Every 6 months
D) Every 12 months
E) No further PCR testing needed as patient has achieved MMR
Incorrect. Let me explain the correct monitoring frequency.

Feedback:
According to the ELN 2022 recommendations, the monitoring frequency for molecular response (qPCR for BCR-ABL1) depends on the patient's response status:

- During the first year: Every 3 months until MMR (BCR-ABL1 0.1% IS) is achieved
- After achieving MMR: Every 3-6 months
- After achieving DMR (deep molecular response, BCR-ABL1 0.01% IS) and stable for >2 years: Every 6-12 months

In Ahmed's case, he has just achieved MMR with BCR-ABL1/ABL1 ratio of 0.08% (IS) at 18 months. At this point, the recommended monitoring frequency shifts to every 3-6 months.

The correct answer is **C) Every 6 months**.

Monthly monitoring (option A) would be too frequent and unnecessary for a patient with stable MMR. Every 3 months (option B) would be appropriate during the first year or for patients with unstable responses. Every 12 months (option D) would be appropriate only after sustained deep molecular response. Discontinuing monitoring (option E) is incorrect as ongoing monitoring is essential throughout TKI therapy.

Phase 3: Long-term Management

Learning Objectives:
- Understand TKI discontinuation criteria and strategies
- Recognize and manage long-term adverse effects of TKI therapy
- Apply treatment-free remission concepts in CML management

Case Continuation:
Ahmed continues on imatinib with good tolerance. Four years after diagnosis, he has maintained a deep molecular response (MR4.5, BCR-ABL1 0.0032% IS) for the past 2 years. He inquires about the possibility of stopping treatment.
Question 8:
Which of the following is NOT a criterion for considering treatment-free remission (TFR) according to the 2022 ELN recommendations?

A) Typical BCR-ABL1 transcript (e13a2 or e14a2)
B) At least 5 years of TKI therapy
C) Sustained deep molecular response (MR4 or deeper) for at least 2 years
D) Access to high-quality PCR testing with rapid turnaround time
E) No history of accelerated or blast phase

Correct! "At least 5 years of TKI therapy" is NOT a criterion for considering treatment-free remission (TFR) according to the 2022 ELN recommendations.

Feedback:
The 2022 ELN recommendations have updated the criteria for TFR eligibility. According to these updated guidelines, the minimum duration of TKI therapy before attempting discontinuation is 3 years (not 5 years as previously recommended in older guidelines).

The current ELN criteria for TFR eligibility include:
- First chronic phase CML (option E is a correct criterion)
- Typical BCR-ABL1 transcripts (e13a2/e14a2) allowing standardized molecular monitoring (option A is a correct criterion)
- No prior treatment failure or resistance
- Sustained deep molecular response (at least MR4.0, BCR-ABL1 0.01% IS) for at least 2 years (option C is a correct criterion)
- Access to reliable, standardized molecular testing with rapid turnaround time (option D is a correct criterion)

Option B is incorrect because the minimum TKI duration requirement is 3 years, not 5 years, making it NOT a criterion according to current guidelines.

Case Continuation:
After discussing the risks and benefits, Ahmed decides to attempt treatment-free remission under close monitoring. Imatinib is discontinued. Four months after stopping, his PCR shows BCR-ABL1/ABL1 ratio of 0.15% (IS).

Question 9:
What is the most appropriate next step in management?

A) Continue monitoring without restarting therapy
B) Restart imatinib 400 mg daily
C) Restart with nilotinib 300 mg twice daily
D) Perform bone marrow biopsy to check for disease progression
E) Start interferon therapy instead of TKI
Correct! Restarting imatinib 400 mg daily is the most appropriate next step in management.

Feedback:
According to the ELN 2022 recommendations, molecular relapse after TKI discontinuation is defined as loss of major molecular response (MMR), which means BCR-ABL1 > 0.1% IS confirmed in a subsequent test. In Ahmed's case, his BCR-ABL1 level of 0.15% exceeds this threshold, indicating loss of MMR and molecular relapse.

The recommended management for molecular relapse is prompt reinitiation of the same TKI that was previously effective, unless there were significant tolerability issues. Since Ahmed tolerated imatinib well before discontinuation, restarting imatinib 400 mg daily is the appropriate course of action.

Option A (continued monitoring) would risk disease progression. Option C (switching to nilotinib) is unnecessary since Ahmed had good response and tolerance to imatinib. Option D (bone marrow biopsy) is not required for molecular relapse detection as PCR is sufficient. Option E (interferon) is not standard treatment for molecular relapse after TFR.

Case Continuation:
Ahmed restarts imatinib and regains MMR within 3 months. Five years later (now 9 years after diagnosis), he has been on continuous imatinib therapy with sustained deep molecular response. During his annual visit, his routine laboratory tests show hemoglobin 11.0 g/dL, calcium 8.2 mg/dL, phosphate 2.7 mg/dL, 25-OH vitamin D 18 ng/mL (low, normal range 30-100), and BMD T-score of -2.1 at the lumbar spine.

Question 10:
What is the most appropriate intervention for Ahmed's bone health?

A) Switch from imatinib to bosutinib
B) Vitamin D supplementation and calcium supplementation
C) Vitamin D supplementation, calcium supplementation, and bisphosphonate therapy
D) Vitamin D supplementation and denosumab
E) Discontinue TKI therapy permanently
Correct! Vitamin D supplementation, calcium supplementation, and bisphosphonate therapy is the most appropriate intervention for Ahmed's bone health.

Feedback:
Long-term TKI therapy, especially with imatinib, is associated with alterations in bone metabolism and decreased bone mineral density. Ahmed has several concerning findings:
- T-score of -2.1 at the lumbar spine (indicating osteopenia, nearly osteoporosis)
- Low vitamin D level (18 ng/mL)
- Low-normal calcium (8.2 mg/dL) and phosphate (2.7 mg/dL)
- Duration of TKI therapy (9 years), which is a risk factor

According to ESMO guidelines for managing long-term effects of TKIs, the appropriate management includes:
1. Vitamin D supplementation to correct deficiency (target level >30 ng/mL)
2. Calcium supplementation to ensure adequate intake
3. Bisphosphonate therapy for patients with T-scores -2.0 or those with additional risk factors

Option B (vitamin D and calcium alone) would be insufficient given his T-score. Option A (switching to bosutinib) might have some benefit for bone health but wouldn't address the immediate need for bone protection. Option D is incomplete, as denosumab would be an alternative to bisphosphonates, but the first-line approach typically includes bisphosphonates. Option E (discontinuing therapy) is inappropriate given his successful disease control.

Session Conclusion

You've completed all 10 questions in this CML case management session. Well done, Rana!

Session ID:
Assessment Domain

Session-80:
Rationale

Session ID:
Instructional Design and Pedagogical

Session-80:
The session's instructional design is of very high quality. The case is expertly scaffolded, progressing logically from initial diagnosis and risk stratification (Phase 1) to therapy monitoring and adverse event management (Phase 2), and finally to complex long-term management issues like treatment-free remission and bone health (Phase 3). The quality of the embedded feedback is a major strength; rationales are detailed, consistently reference current clinical guidelines (ELN 2022, ESMO), and explain why incorrect options are wrong. Instructions are exceptionally clear. The only minor weakness is that learning objectives are introduced part-way through the session rather than being stated explicitly at the outset, resulting in a score of 4; however, the objectives provided for Phases 2 and 3 are clear and perfectly aligned with the content.

Session ID:
Clinical Authenticity and Content Fidelity

Session-80:
The session demonstrates outstanding clinical fidelity and content accuracy. Plausibility & Guideline Alignment: The clinical narrative is indistinguishable from an authentic patient case, tracing a realistic long-term journey of a CML patient. All diagnostic steps, risk

stratifications (using the appropriate ELTS score), therapeutic decisions, and monitoring strategies are perfectly aligned with current, evidence-based guidelines from ELN and ESMO. The session correctly applies nuanced concepts like the "Warning" response category and the updated criteria for attempting treatment-free remission. Pharmacotherapeutic Accuracy & Domain Specificity: The pharmacotherapeutic recommendations are optimal and well-justified at every step. The selection of imatinib for a low-risk patient, the management of its side effects (electrolyte correction before dose modification), and the handling of molecular relapse are all textbook examples of best practices. A critical review confirms the complete absence of domain entanglement; every diagnostic tool, therapeutic agent (e.g., imatinib, nilotinib), and management principle is entirely appropriate and specific to the domain of Chronic Myeloid Leukemia (CML). No therapies or standards of care from other hematologic malignancies (like AML or ALL) are incorrectly applied.

Session ID:
Modeled Clinical Reasoning

Session-80:
The simulation expertly models an authentic clinical reasoning process. Each question presents a well-defined clinical problem with the necessary data to make an informed decision (e.g., providing lab values for managing muscle cramps, T-scores for bone health). The multiple-choice options provide an excellent breadth of plausible and relevant alternatives, including key distractors that effectively test nuanced understanding (e.g., differentiating between dose reduction, therapy switching, and supportive care for side effects). The narrative demonstrates sound, risk-adjusted prioritization throughout, and the clinical outcomes described for each decision are realistic, specific, and highly instructive, reinforcing the key learning points of the session.

Session-81

Session ID:
LLM

Session-81:
Claude 3.7 Sonnet

Session ID:
Session Transcript

Session-81:
Role: You are an Oncology Clinical Pharmacy Professor specializing in hematologic malignancies, particularly Chronic Myeloid Leukemia (CML). Your goal is to guide PharmD students through evidence-based clinical reasoning using an interactive case-based learning approach.

Guidelines for Execution

Educational Methodology

Cognitive Apprenticeship Model: Scaffold learning from foundational knowledge to advanced decision-making. Alignment with Standards: Ensure content aligns with ACPE Educational Outcomes and ASCO/HOPA oncology pharmacy practice standards. Blooms Taxonomy Progression: Structure questions to progress from knowledge recall application analysis evaluation. Feedback: Provide multi-tiered feedback on both factual accuracy and clinical reasoning processes. Guideline Integration: Connect all guidance to specific guideline recommendations with proper citations (e.g., ESMO, ELN). Case Architecture & Development

Required Clinical Content

All cases must comprehensively address:

Diagnostic Framework

WHO 2016/2022 classification with molecular/cytogenetic analysis. Risk stratification using ELN 2022 criteria. Measurable residual disease (MRD) monitoring and interpretation. Therapeutic Considerations

Treatment selection based on patient-specific factors (age, performance status, comorbidities, molecular profile). Comprehensive supportive care protocols (antimicrobial prophylaxis, transfusion support, tumor lysis management). First-line treatment selection. Management of refractory and resistant disease. Immunotherapy approaches and transplant eligibility. Clinical trial considerations and emerging biomarkers. Interactive Session Structure

Orientation: Begin with clear learning objectives for the case. Case Presentation: Present the case in 3 distinct phases with progressively increasing complexity: Phase 1: Initial presentation and diagnostic workup (e.g., symptoms, lab results, bone marrow biopsy). Phase 2: Induction therapy selection and supportive care planning. Phase 3: Post-remission therapy, MRD monitoring, and long-term management. Decision Points: Incorporate 10 multiple-choice questions at key therapeutic decision points, such as: Selecting induction therapy based on patient-specific factors. Managing complications (e.g., febrile neutropenia, tumor lysis syndrome). Interpreting MRD results and adjusting therapy. Visual Aids: Include algorithms, graphs, or tables to reinforce learning. Conclusion: Summarize with a summative assessment and broader application. Interaction Protocol

When the student types "START": Ask for students name and ID When the student types in his first and last name: Deliver this exact message

Edit
Hello! I'll be serving as your Oncology Clinical Pharmacy Professor specializing in hematologic malignancies, particularly Chronic

Myeloid Leukemia (CML). My goal is to guide you through evidence-based clinical reasoning using an interactive case-based learning approach.

When you're ready to begin our interactive CML case session, please type "START" and I'll proceed with the session orientation.

Start

Please enter your first and last name to begin the session.

[[**Student Name**]]

Edit
CML Case-Based Learning Session

Topic: Chronic Myeloid Leukemia Management

Session Orientation
Learning Objectives
By the end of this case-based learning session, you will be able to:

Apply the WHO 2022 classification criteria to accurately diagnose CML
Stratify CML patients using the current ELN 2022 risk criteria
Select appropriate first-line therapy based on patient-specific factors
Develop comprehensive supportive care plans for CML patients
Interpret MRD monitoring results and recommend therapy adjustments
Manage treatment-resistant disease and evaluate for alternative therapies
Let's begin with our case presentation.

Phase 1: Initial Presentation and Diagnostic Workup
Patient: Michael Thompson, a 48-year-old male
Chief Complaint: Fatigue, early satiety, and unintentional weight loss (10 pounds over 2 months)
PMH: Hypertension, well-controlled on lisinopril 10mg daily; Type 2 diabetes mellitus with HbA1c 7.2%
PSH: Appendectomy (age 22)
Social History: Former smoker (quit 5 years ago), occasional alcohol use, works as a high school teacher
Family History: Father with colon cancer (age 65), mother alive with hypertension

Current Medications:

Lisinopril 10mg daily
Metformin 1000mg twice daily
Multivitamin daily
Physical Examination:

Vital signs: BP 142/88, HR 82, Temp 37.1C, RR 18
Palpable splenomegaly (4cm below costal margin)
No lymphadenopathy

Mild pallor
Laboratory Results:

WBC: 89,000/L (reference: 4,500-11,000/L)
Hemoglobin: 10.2 g/dL (reference: 13.5-17.5 g/dL)
Platelets: 450,000/L (reference: 150,000-450,000/L)
Peripheral blood smear: Significant leukocytosis with left shift, basophilia (4%), and eosinophilia (3%)
Comprehensive metabolic panel: Normal except for mildly elevated LDH (350 U/L)
Question 1:
Based on the initial presentation, what additional diagnostic test is most critical to confirm the suspected diagnosis?

A) Flow cytometry of peripheral blood
B) Bone marrow aspiration and biopsy with cytogenetics
C) Next-generation sequencing panel
D) CT scan of chest, abdomen, and pelvis
E) JAK2 V617F mutation analysis

B

) Flow cytometry of peripheral blood
B) Bone marrow aspiration and biopsy with cytogenetics
C) Next-generation sequencing panel
D) CT scan of chest, abdomen, and pelvis
E) JAK2 V617F mutation analysis

B

Edit
That's correct! Bone marrow aspiration and biopsy with cytogenetics is the critical next step.

Michael's presentation strongly suggests CML with his leukocytosis, left shift, basophilia, and splenomegaly. While his peripheral blood findings are highly suggestive of CML, a bone marrow examination with cytogenetic analysis is essential to:

Confirm the presence of the Philadelphia chromosome t(9;22)(q34;q11.2)
Determine the percentage of blasts to establish disease phase (chronic, accelerated, or blast)
Assess for additional cytogenetic abnormalities that could impact prognosis
Establish a baseline for future monitoring
The bone marrow biopsy was performed, and here are the results:

Question 2:
Based on these findings, how would you classify this patient according to the WHO 2022 classification, and what is his disease phase?
A) CML, chronic phase
B) CML, accelerated phase
C) CML, blast phase
D) Atypical CML

E) Chronic myelomonocytic leukemia (CMML)

A

Edit
Excellent! You're absolutely correct. This patient has CML in chronic phase.

According to the WHO 2022 classification, Michael's findings are consistent with CML in chronic phase, characterized by:

Presence of the Philadelphia chromosome t(9;22)(q34;q11.2) confirmed by cytogenetics
BCR-ABL1 fusion gene detected by FISH and quantified by RT-PCR
Blast percentage <10% (patient has only 2%)
No evidence of accelerated phase criteria (no additional chromosomal abnormalities)
Let's proceed with risk stratification, which is essential for prognosis and treatment planning.

Additional information gathered:

Spleen size by ultrasound: 16 cm (increased)
Based on these findings, we can calculate the Sokal, EUTOS, and ELTS scores:

Sokal Score calculation:

Age: 48 years
Spleen size: 4 cm below costal margin
Platelet count: 450 10^9/L
Blast percentage: 2%
Calculated Sokal score: 0.9
ELTS Score calculation:

Age: 48 years
Spleen size: 4 cm below costal margin
Blast percentage: 2%
Platelet count: 450 10^9/L
Calculated ELTS score: 1.2
)

Question 3:
Based on the ELN 2022 guidelines, what risk category does this patient fall into, and which prognostic scoring system is now preferred for TKI-treated patients?

A) Low risk by Sokal score, which remains the gold standard
B) Intermediate risk by ELTS score, which is now the preferred system
C) High risk by EUTOS score, which is now the preferred system
D) Intermediate risk by Sokal score, which remains the gold standard
E) Low risk by ELTS score, which is now the preferred system
B

Edit
Correct! The patient falls into the intermediate risk category according to the ELTS score, which is currently the preferred prognostic scoring system for TKI-treated patients.

According to the European LeukemiaNet (ELN) 2022 guidelines, the ELTS (EUTOS Long-Term Survival) score has become the preferred prognostic system for CML patients treated with tyrosine kinase inhibitors. This represents an evolution from earlier practice when the Sokal score was the most widely used.

With an ELTS score of 1.2, Michael falls into the intermediate risk category (0.7-1.6), which has implications for:

Treatment selection
Monitoring frequency
Expected response milestones
Long-term prognosis
Now let's move to the treatment planning phase for this patient.
Question 4:
Based on current ELN 2022 guidelines, what is the most appropriate first-line therapy for this patient?

A) Imatinib 400 mg daily
B) Dasatinib 100 mg daily
C) Nilotinib 300 mg twice daily
D) Bosutinib 400 mg daily
E) Ponatinib 45 mg daily

A

Edit
That's correct! Imatinib 400 mg daily is the most appropriate first-line therapy for this patient.

According to the ELN 2022 guidelines, imatinib 400 mg daily remains a standard first-line therapy option for patients with newly diagnosed chronic phase CML. While second-generation TKIs (dasatinib, nilotinib, bosutinib) achieve faster and deeper molecular responses, they haven't consistently demonstrated overall survival advantages over imatinib in frontline therapy.
Question 5:

Which of the following is most important to monitor specifically in this patient during the first few months of imatinib therapy?

A) Liver function tests due to risk of hepatotoxicity

B) Fasting glucose and HbA1c due to his diabetes

C) Blood pressure control due to his hypertension

D) QTc interval due to risk of prolongation

E) Serum creatinine and phosphate levels due to risk of renal

A
You're correct! Liver function tests (LFTs) are most important to monitor specifically in this patient during the first few months of imatinib therapy.

While all the listed monitoring parameters are relevant, LFTS are particularly important because:

Hepatotoxicity is a recognized adverse effect of imatinib, occurring in approximately 2-5% of patients LFT elevations typically occur within the first 2-3 months of starting therapy This patient likely takes metformin for his diabetes, which can also affect liver enzymes, creating potential for additive hepatic effects Early detection of hepatotoxicity allows for dose djustments or treatment modifications beforesevere

liver injury occurs

Recommended LFT monitoring schedule:

Baseline (already obtained)

Every 2 weeks for the first month

Monthly for the next 2 months Then every 3 months if stable

The patient begins imatinib 400 mg daily with appropriate monitoring. Let's follow his course over the next few months:

3-month follow-up results:

Complete hematologic response achieved (WBC normalized)

BCR-ABL1/ABL1 ratio (IS):

8.2% (optimal response per ELN 2022)

'o significant adverse effects LFTs remain within normal
Question 6:

Based on the 6-month response assessment, what is the most appropriate next step in management?

A) Continue imatinib at current dose with repeat BCR-ABL1 testing in 3 months

B) Increase imatinib to 600 mg daily

C) Switch to a second-generation TKI (dasatinib, nilotinib, or bosutinib)

D) Obtain BCR-ABL1 mutation analysis

E) Refer for allogeneic stem cell transplant
A
You're correct! The most appropriate next step is to continue imatinib at the current dose with repeat BCR-ABL1 testing in 3 months.

According to the ELN 2022 guidelines, a BCR-ABL1 ratio of 2.1% at 6 months falls into the "warning" category (>1% but 10%). This indicates that the patient hasn't achieved an optimal response, but hasn't met criteria for treatment failure either.

For patients in the warning

category:

Continue the same treatment with more frequent monitoring Repeat BCR-ABL1 quantitative Increasing the imatinib dose or switching to a second-generation TKI would be premature at this point, as the patient may still achieve deeper responses with continued treatment.

Mutation analysis isn't indicated yet as there's no evidence of resistance, and transplant evaluation would only be considered after failure of multiple TKIs.

Let's follow Michael's

progress:

9-month follow-up results:

BCR-ABL1/ABL1 ratio (IS): 0.9% (improving)

CBC: Normal

LFTs: Normal

Continues to have mild peripheral edema managed with compression stockings

2-month follow-up results
Question 7:

Based on the 12-month response assessment, what is the most appropriate next step in management?

A) Continue imatinib with repeat BCR-ABL1 testing in 3 months

B) Increase imatinib to 600 mg daily

C) Switch to a second-generation TKI

D) Obtain BCR-ABL1 mutation analysis

E) Consider treatment-free remission
A
 You 're correct! Continuing imatinib with repeat BCR-ABL1 testing in 3 months is the most appropriate approach.

According to the ELN 2022 guidelines, a BCR-ABL1 ratio of 0.3% at 12 months falls into the "warning" category (>0.1% but 1%). While not achieving Major Molecular Response (MMR, BCR-ABL1 0.1%), the patient is showing steady improvement:

6 months: 2.1%

9 months: 0.9%

12 months: 0.3%

This consistent downward trend suggests the patient is responding to therapy, albeit at a slower pace than optimal.

Since there's no evidence of resistance, continuing the urrent treatment with careful

monitoring is appropriate ELN guidelines recommend:

Continue imatinib 400 mg daily

Repeat BCR-ABL1 testing in 3 months

Consider mutation testing only if response is lost or progression occurs

Let's continue following

Michael's course:

15-month follow-up results:

BCR-ABL1/ABL1 ratio (IS): 0.08% (MMR achieved)

CBC: Normal

Patient experiencing manageable side effects

Occasional missed doses due to forgetfulness

(approximately 2-3 doses per month)

18-month follow-up results:

BCR-ABL1/ABI 1 ratio (IS)
Question 8:

Regarding treatment-free remission (TFR), which of the following statements is correct according to the current ELN 2022 guidelines?

A) TFR should be attempted in all CML patients who achieve MMR for at least 12 months

B) TFR requires achievement of sustained deep molecular response (MR4.5) for at least 2 years

C) Once TFR is initiated, monitoring is needed only every 6 months

D) Loss of MMR during TFR attempt requires immediate resumption of the previous TKI

E) Second TFR attempts are not recommended after failing a first
B
Excellent! You're correct. According to the ELN 2022 guidelines, treatment-free remission (TFR) requires achievement of sustained deep molecular response (MR4.5) for at least 2 years.

The current recommendations for TFR include:

MR4.5 (BCR-ABL1 0.0032% IS) sustained for at least 2 years

Minimum of 5 years of TKI therapy before attempting TFR

No history of accelerated or blast phase

Ability to perform and access frequent molecular monitoring

Patient consent after thorough discussion of benefits and risks

or monitoring during TFR Monthly molecular testing for the first 6 months

Every 2 months for months 7-12

Every 3 months thereafter

If MMR is lost during TFR (BCR-ABL1 >0.1%), treatment should be promptly restarted, and second attempts at TFR can be considered if deep molecular response is re-achieved and sustained.

Let's continue Michael's case at the 24-month mark:

24-month follow-up results:

BCR-ABL1/ABL1 ratio (IS): 0.002% (MR4.5 achieved)

CBC: Normal

Patient tolerating therapy well with minimal side effects

Inquires again about treatment discontinuation

Session ID:
Assessment Domain

Session-81:
Rationale

Session ID:
Instructional Design and Pedagogical

Session-81:
The session's instructional design is of excellent quality. The learning objectives provided at the outset are explicit, measurable, and perfectly aligned with the case content, effectively framing the educational goals. The case is expertly scaffolded, progressing in a logical and intuitive sequence from initial presentation and diagnosis through risk stratification, first-line therapy, nuanced response monitoring, and long-term management considerations like Treatment-Free Remission (TFR). The feedback provided after each question is a significant strength, offering detailed, guideline-anchored rationales (citing WHO 2022 and ELN 2022) that clarify not just the correct answer but also the underlying clinical reasoning. The overall framing and instructions are exceptionally clear.

Session ID:
Clinical Authenticity and Content Fidelity

Session-81:
The session demonstrates high clinical fidelity and authenticity, though with a minor point of contention in therapeutic selection. Plausibility & Domain Specificity: The clinical narrative is indistinguishable from an authentic patient case, with realistic demographics, labs, and disease progression. Critically, the session demonstrates perfect domain specificity; all diagnostics, risk scores (Sokal, ELTS), therapies (TKIs), and monitoring milestones are entirely appropriate for Chronic Myeloid Leukemia (CML), with no evidence of domain entanglement. Guideline Alignment & Pharmacotherapeutic Accuracy: The therapeutic recommendations are largely appropriate and well-justified. However, the selection of imatinib as the singular "most appropriate" first-line therapy for an intermediate-risk patient (Question 4) is debatable. While imatinib is an acceptable, guideline-concordant option, current ELN and NCCN guidelines also supportand many experts preferthe use of second-generation TKIs (dasatinib, nilotinib, bosutinib) in this setting to achieve faster, deeper molecular responses, which are linked to better long-term outcomes and TFR eligibility. By presenting imatinib as the definitive correct choice, the simulation slightly oversimplifies a complex decision, lowering the scores for guideline alignment and accuracy from "Excellent" to "Very Good." All other therapeutic decisions and monitoring steps are optimally aligned with current standards.

Session ID:

Modeled Clinical Reasoning

Session-81:

The simulation models an expert-level clinical reasoning process exceptionally well. Each stage of the case frames a clear, relevant clinical problem (e.g., initial diagnosis, risk stratification, suboptimal response). The multiple-choice options for each question are expertly constructed, providing a strong breadth of plausible alternatives and key distractors that effectively probe the student's nuanced understanding of CML management. The decision-making logic demonstrated throughout the case is sound, correctly prioritizing critical steps like obtaining cytogenetics before treatment and adhering to guideline-based monitoring before escalating therapy. Finally, the modeled clinical outcomesparticularly the patient's "warning" responses before achieving MMRare both realistic and highly instructive, reinforcing the importance of sequential monitoring and patience in TKI therapy.

Session-82

Session ID:
LLM

Session-82:
Claude 3.7 Sonnet

Session ID:
Session Transcript

Session-82:
Phase 1: Initial Presentation and Diagnostic Workup
Learning Objectives:
- Apply WHO classification criteria to diagnose CML
- Interpret laboratory and molecular findings
- Stratify CML risk using ELN 2022 criteria

Case Presentation:
Samuel Green, a 47-year-old male professor, presents to his primary care physician with complaints of progressive fatigue, unexplained weight loss (15 pounds over 3 months), early satiety, and occasional night sweats. Physical examination reveals palpable splenomegaly extending 4 cm below the left costal margin.

Initial Laboratory Results:
- WBC: 112,000/L (4,500-11,000/L)
- Hemoglobin: 10.1 g/dL (13.5-17.5 g/dL)
- Platelets: 382,000/L (150,000-450,000/L)
- Peripheral blood smear: Left shift with myelocytes, metamyelocytes, and basophilia
- LDH: 625 U/L (125-240 U/L)
- Comprehensive metabolic panel: Within normal limits

Question 1: Based on the initial presentation and laboratory findings, what is the most appropriate next diagnostic step?
A) Order CT scan of chest, abdomen, and pelvis to evaluate for malignancy
B) Perform bone marrow aspiration and biopsy with cytogenetic analysis
C) Initiate empiric antibiotic therapy for suspected infection
D) Order flow cytometry of peripheral blood for suspected lymphoproliferative disorder
E) Begin hydroxyurea for cytoreduction while awaiting further testing

Correct Answer: B) Perform bone marrow aspiration and biopsy with cytogenetic analysis
Feedback: Correct!
Performing a bone marrow aspiration and biopsy with cytogenetic analysis is indeed the most appropriate next step. The patient's clinical presentation (fatigue, weight loss, night sweats, splenomegaly) combined with the laboratory findings (marked leukocytosis with left shift and basophilia) strongly suggest a myeloproliferative neoplasm, particularly CML.
While a CT scan might provide additional information about organomegaly, it wouldn't confirm the diagnosis. Flow cytometry would be more appropriate for lymphoproliferative disorders, and initiating empiric antibiotics or hydroxyurea before confirming the diagnosis would be premature at this stage.
According to the 2022 ELN recommendations, bone marrow examination with cytogenetic analysis is essential for:
- Confirming the diagnosis of CML
- Detecting the Philadelphia chromosome [t(9;22)(q34;q11)]
- Identifying additional chromosomal abnormalities that may impact prognosis

Additional Results:
Bone marrow biopsy shows hypercellularity (95%) with granulocytic hyperplasia.
Cytogenetics: 46,XY,t(9;22)(q34;q11) in 20/20 metaphases
RT-PCR: BCR-ABL1 (p210) transcript detected
FISH: BCR-ABL1 fusion present in 94% of analyzed cells

Question 2:Based on the diagnostic findings, which WHO classification best describes this patient's condition?
A) Chronic myeloid leukemia, chronic phase

B) Chronic myeloid leukemia, accelerated phase
C) Chronic myelomonocytic leukemia
D) Chronic myeloid leukemia, blast phase
E) Atypical chronic myeloid leukemia, BCR-ABL1 negative
Correct Answer: A) Chronic myeloid leukemia, chronic phase
Feedback: Correct!
The patient indeed has Chronic Myeloid Leukemia in chronic phase (CML-CP). According to the WHO 2022 classification, CML-CP is characterized by:
- Presence of the Philadelphia chromosome [t(9;22)(q34;q11)] and/or BCR-ABL1 fusion
- <10% blasts in peripheral blood and bone marrow
- Absence of features meeting criteria for accelerated or blast phase
This patient demonstrates the classic findings of CML-CP:
- Presence of the Philadelphia chromosome on cytogenetic analysis
- BCR-ABL1 fusion gene (p210 transcript) detected by RT-PCR
- No evidence of increased blast percentage or other features suggestive of disease progression
The chronic phase is the initial stage of CML and is generally more responsive to tyrosine kinase inhibitor (TKI) therapy.
Additional Information:
To complete the diagnostic workup and prepare for treatment planning, additional tests were conducted:
- BCR-ABL1 transcript level (IS): 42%
- Bone marrow blast percentage: 3%
- Additional cytogenetic abnormalities: None
- Basophils: 5% (peripheral blood)
- Peripheral blood blast percentage: 1%
Question 3:Based on the 2022 ELN risk stratification criteria, what risk category should be assigned to this patient?
A) Low risk
B) Intermediate risk
C) High risk
D) Very high risk
E) Cannot be determined with the provided information
Correct Answer: A) Low risk
Feedback: Correct!
According to the 2022 ELN risk stratification criteria for CML, this patient does indeed fall into the low-risk category.
The 2022 ELN risk stratification for CML chronic phase considers several factors:
- Age
- Spleen size
- Blood counts (platelet count, basophils, blast percentage)
- Additional chromosomal abnormalities
For our patient:
- Age: 47 years (65 years is favorable)
- Spleen: 4 cm below costal margin (<10 cm is favorable)
- Platelets: 382,000/L (within normal range)
- Basophils: 5% (<5% is favorable for Sokal, 2% for EUTOS long-term survival)
- No additional chromosomal abnormalities
- Blast percentage: 1% in peripheral blood, 3% in bone marrow (<5% is favorable)
The absence of high-risk features (such as major route additional chromosomal abnormalities, high blast counts, or massive splenomegaly) places this patient in the low-risk category, which generally predicts a more favorable response to TKI therapy and better long-term outcomes.
Phase 2: Induction Therapy and Supportive Care
Learning Objectives:
- Select appropriate TKI therapy based on patient characteristics and risk assessment
- Anticipate and manage TKI-associated toxicities
- Determine appropriate monitoring strategies for treatment response
Case Continuation:
With the diagnosis of CML-CP and risk stratification completed, you must now recommend a management plan for Samuel. His

medical history reveals hypertension controlled with lisinopril 10 mg daily. He has no other significant medical conditions, no known drug allergies, and his performance status is ECOG 0. Insurance coverage is not a limiting factor.
Question 4: What is the most appropriate first-line treatment for this patient with newly diagnosed CML in chronic phase?
A) Imatinib 400 mg once daily
B) Nilotinib 300 mg twice daily
C) Dasatinib 100 mg once daily
D) Bosutinib 400 mg once daily
E) Ponatinib 45 mg once daily
Correct Answer: C) Dasatinib 100 mg once daily.
Feedback: This is partially correct.
While dasatinib 100 mg once daily is an approved first-line therapy for CML chronic phase, the most appropriate choice for this specific patient would be imatinib 400 mg once daily (option A).
Let me explain:
According to the 2022 ELN recommendations and ESMO guidelines, for a newly diagnosed low-risk CML-CP patient like Samuel:
- Imatinib 400 mg daily is the standard first-line option with proven long-term safety and efficacy data spanning over 20 years
- Second-generation TKIs (dasatinib, nilotinib, bosutinib) offer faster and deeper molecular responses but have not consistently demonstrated improved overall survival compared to imatinib
- Second-generation TKIs carry higher risks of specific adverse events (dasatinib: pleural effusion, pulmonary hypertension; nilotinib: cardiovascular events, hyperglycemia; bosutinib: gastrointestinal toxicity)
For a low-risk patient like Samuel with no urgent need for rapid cytoreduction and no concerning comorbidities, imatinib offers the best balance of efficacy and safety. Second-generation TKIs would typically be reserved for intermediate or high-risk patients where faster, deeper responses might be beneficial.
Ponatinib is generally not recommended for first-line therapy due to its cardiovascular toxicity profile and is typically reserved for patients with the T315I mutation or those resistant to multiple other TKIs.
Additional Information:
Samuel is started on imatinib 400 mg daily. After 3 months of therapy, he returns for follow-up.
Laboratory Results at 3-month follow-up:
- Complete blood count: Within normal limits
- BCR-ABL1 (IS): 9.5%
- Comprehensive metabolic panel: Normal liver and kidney function
Question 5:Based on the 3-month response assessment, how would you categorize this patient's response to therapy according to ELN 2022 criteria?
A) Optimal response
B) Warning
C) Failure
D) Loss of response
E) Complete cytogenetic response
Answer: A) Optimal response
Rationale:
According to the 2022 ELN (European LeukemiaNet) criteria, response to TKI therapy in chronic phase CML is assessed at various time points using BCR-ABL1 transcript levels on the International Scale (IS) and other markers.
At 3 months, the primary molecular target is:
BCR-ABL1 (IS) 10% Optimal Response
Samuels 3-month results:
BCR-ABL1 (IS): 9.5%
CBC: Normal
No signs of disease progression or toxicity
This fits squarely into the Optimal Response category.
Feedback: Excellent answer and rationale!
You are absolutely correct. According to the 2022 ELN recommendations, at the 3-month timepoint:
- BCR-ABL1 10% (IS) is classified as an optimal response
- BCR-ABL1 >10% but 30% falls into the warning category
- BCR-ABL1 >30% is considered treatment failure
With Samuel's BCR-ABL1 level at 9.5%, he indeed shows an optimal response at this early timepoint. This suggests he is on the right

trajectory with imatinib therapy, and treatment should be continued without modification.
Your reasoning demonstrates excellent understanding of the molecular monitoring milestones in CML management. The normalization of his blood counts also supports the overall positive response to therapy.
Case Continuation:
Samuel continues imatinib therapy with good medication adherence. At his 6-month follow-up, his BCR-ABL1 (IS) level is 3.2%. However, he reports experiencing persistent moderate muscle cramps, particularly at night, and occasional mild periorbital edema.
Question 6: What is the most appropriate approach to manage Samuel's muscle cramps while maintaining optimal treatment efficacy?
A) Reduce imatinib dose to 300 mg daily
B) Switch to nilotinib 300 mg twice daily
C) Recommend tonic water containing quinine and calcium/magnesium supplements
D) Add hydroxyurea at low dose to counteract side effects
E) Discontinue imatinib and start interferon-alpha therapy
Correct Answer: C) Recommend tonic water containing quinine and calcium/magnesium supplements
Feedback: Correct!
You've selected the most appropriate approach for managing Samuel's muscle cramps. Muscle cramps and periorbital edema are common side effects of imatinib that typically don't warrant dose reduction or switching therapy, especially when the patient is showing an optimal response (BCR-ABL1 3.2% at 6 months is excellent).
The recommended supportive care measures include:
- Tonic water containing quinine (empirically shown to help with cramps)
- Calcium and magnesium supplementation
- Adequate hydration
- Gentle stretching before bedtime
These interventions can significantly improve quality of life without compromising treatment efficacy. Maintaining the established effective dose is crucial for achieving deeper molecular responses.
Regarding the other options:
- Reducing imatinib dose would be premature for Grade 1-2 toxicity and might compromise efficacy
- Switching to nilotinib is unnecessary as the current therapy is showing optimal response
- Hydroxyurea would not address the muscle cramps and could cause additional toxicity
- Interferon-alpha is not a standard monotherapy for CML in the TKI era and has significant toxicities
Case Continuation:
Samuel's muscle cramps improve with the recommended interventions. At his 12-month follow-up:
- BCR-ABL1 (IS): 0.085% (MMR achieved)
- Complete blood count: Normal
- Liver and kidney function tests: Normal
Question 7: What is the most appropriate monitoring strategy for Samuel moving forward?
A) BCR-ABL1 quantitative PCR testing every 3 months indefinitely
B) BCR-ABL1 quantitative PCR testing every 3-6 months and cytogenetic analysis annually
C) BCR-ABL1 quantitative PCR testing every 3-6 months without routine cytogenetic analysis
D) Switch to monitoring with complete blood counts only, with BCR-ABL1 testing only if abnormalities occur
E) Bone marrow biopsy and PCR testing every 6 months
Correct Answer: C) BCR-ABL1 quantitative PCR testing every 36 months without routine cytogenetic analysis
Feedback: Correct!
You've selected the optimal monitoring strategy for Samuel at this stage of treatment. According to the 2022 ELN recommendations, once a patient has achieved a major molecular response (MMR, BCR-ABL1 0.1% IS), the appropriate monitoring consists of:
- BCR-ABL1 quantitative PCR testing every 3-6 months
- No routine cytogenetic analysis is required unless there are signs of treatment failure or disease progression
This approach allows for:
- Timely detection of potential loss of response
- Monitoring for deeper molecular responses (MR4, MR4.5) that might eventually qualify the patient for treatment-free remission (TFR) consideration
- Avoiding unnecessary invasive procedures (bone marrow biopsies)
Routine cytogenetic analysis is no longer recommended once a patient has achieved MMR, as molecular monitoring with qPCR is more sensitive for detecting minimal residual disease and potential relapse.
The frequency of monitoring (3-6 months) provides a good balance between ensuring adequate surveillance and minimizing patient

burden and healthcare costs.
Phase 3: Post-Remission Therapy and Long-Term Management
Learning Objectives:
- Evaluate eligibility for treatment-free remission (TFR)
- Manage long-term toxicities of TKI therapy
- Assess strategies for patients with suboptimal response or resistance
Case Continuation:
Samuel has now been on imatinib therapy for 5 years with excellent adherence. His most recent tests show:
- BCR-ABL1 (IS): Undetectable (<0.0032%, MR4.5) for the past 2.5 years
- Complete blood count: Normal
- Comprehensive metabolic panel: Normal
- DEXA scan: T-score -1.2 (mild osteopenia)
- Cardiac evaluation: Normal
Samuel inquires about the possibility of stopping treatment, as he has heard about treatment-free remission.
Question 8: Based on current guidelines, what is the most accurate information to provide Samuel regarding treatment-free remission (TFR)?
A) TFR should not be attempted as it always leads to relapse
B) He meets criteria for TFR attempt with a >70% chance of maintaining remission
C) He meets criteria for TFR attempt but should be informed of a 40-60% chance of molecular recurrence
D) TFR should only be attempted in the context of a clinical trial
E) He should switch to a second-generation TKI for 2 years before attempting TFR
Correct Answer: C) He meets criteria for TFR attempt but should be informed of a 4060% chance of molecular recurrence
Rationale:
Treatment-free remission (TFR) is now a recognized goal in CML management for selected patients who achieve and sustain deep molecular response (DMR). According to ELN 2022 and other international guidelines (e.g., NCCN), patients like Samuel may be eligible to consider TFR
Feedback: Excellent answer and reasoning!
You are absolutely correct. According to the 2022 ELN recommendations and ESMO guidelines, Samuel meets the criteria for attempting treatment-free remission:
- Sustained deep molecular response (MR4.5) for >2 years
- First-line therapy with a TKI for 5 years
- No prior treatment failure or resistance
- Typical BCR-ABL1 transcript (p210, e13a2 or e14a2)
- Access to reliable, standardized qPCR testing with rapid turnaround
However, it's critical to inform him that based on multiple clinical trials (including EURO-SKI, STIM, and STOP 2G-TKI studies), approximately 40-60% of patients experience molecular recurrence after TKI discontinuation, typically within the first 6 months.
Your reasoning shows excellent understanding of the TFR concept, which has become an important treatment goal in CML management. This approach allows eligible patients to avoid indefinite therapy while maintaining close monitoring.
Case Continuation:
After thorough discussion of the risks and benefits, Samuel decides to attempt treatment-free remission. Imatinib is discontinued, and a rigorous monitoring schedule is implemented with monthly BCR-ABL1 testing for the first 6 months.
Three months after discontinuation, his BCR-ABL1 (IS) is 0.12% (loss of MMR).
Question 9:What is the most appropriate next step in management?
A) Continue monitoring without restarting therapy, as fluctuations are common
B) Immediately restart imatinib at the previous dose of 400 mg daily
C) Restart therapy with nilotinib as second-line treatment
D) Perform mutational analysis before deciding on treatment
E) Restart imatinib at a reduced dose of 300 mg daily
Answer: B) Immediately restart imatinib at the previous dose of 400 mg daily
Feedback: Correct!
You've selected the most appropriate management strategy for Samuel's molecular relapse after TFR attempt. According to the 2022 ELN recommendations and NCCN guidelines, the criteria for treatment reinitiation after TFR attempt is loss of major molecular response (MMR), defined as BCR-ABL1 >0.1% IS.
With Samuel's BCR-ABL1 rising to 0.12%, he has lost MMR and requires prompt reinitiation of therapy. The guidelines specifically

recommend:
- Restarting the same TKI that led to the deep molecular response (imatinib in Samuel's case)
- Using the same effective dose (400 mg daily)
- Not switching to another TKI unless there was intolerance to the first TKI

The rationale for this approach is that patients who relapse after TFR typically remain sensitive to the same TKI and can rapidly regain molecular response once treatment is restarted. Most patients who restart therapy after MMR loss will regain deep molecular response within 3-6 months.

Mutational analysis would only be indicated if there was evidence of resistance to therapy, which is not the case in this TFR-related relapse scenario.

Case Continuation:

Samuel restarts imatinib 400 mg daily and regains MMR within 2 months. At his 1-year follow-up after restarting therapy:
- BCR-ABL1 (IS): 0.002% (MR4)
- Complete blood count: Normal
- Samuel reports fatigue and joint pain that affect his quality of life

Question 10:Which of the following approaches would be most appropriate to address Samuel's quality of life concerns while maintaining disease control?

A) Switch to nilotinib 300 mg twice daily to reduce side effects
B) Reduce imatinib dose to 300 mg daily and monitor response closely
C) Attempt a second TFR trial as he has regained deep molecular response
D) Add a low-dose corticosteroid to manage joint symptoms
E) Switch to bosutinib 400 mg daily due to its different side effect profile

Answer: B) Reduce imatinib dose to 300 mg daily and monitor response closely

Rationale:

Samuel has:

Stable MR4 (BCR-ABL1 0.002%) after 1 year of re-treatment,
Normal blood counts, and
Persistent fatigue and joint pain known chronic adverse effects of imatinib that can impact quality of life.

Feedback: Excellent answer and rationale!

You've selected the most appropriate approach for Samuel's situation. When a patient has achieved and maintained a deep molecular response (MR4 or better) but experiences chronic adverse effects that impact quality of life, a carefully managed dose reduction is the preferred strategy.

According to the 2022 ELN recommendations and results from studies like DESTINY and INTERIM:
- Patients with stable deep molecular responses (MR4 or better) can often maintain these responses with reduced TKI doses
- Imatinib dose reduction to 300 mg daily (or even 200 mg daily in some cases) can significantly improve quality of life while maintaining molecular response
- Close monitoring is essential (every 1-2 months initially) to ensure molecular stability after dose reduction

This approach offers the best balance between maintaining disease control and improving quality of life, compared to the alternatives:
- Switching TKIs when a patient has an optimal response simply due to tolerability issues exposes them to new potential adverse events
- A second TFR attempt would be premature so soon after molecular recurrence
- Corticosteroids would introduce additional adverse effects without addressing the underlying cause
- Bosutinib often causes significant gastrointestinal toxicity and may not resolve fatigue/joint pain

Case Conclusion

Samuel's imatinib dose is reduced to 300 mg daily. After three months on the reduced dose:
- His fatigue and joint pain have significantly improved
- BCR-ABL1 (IS) remains <0.01% (maintained MMR)
- Complete blood count remains normal

The treatment plan moving forward includes:
- Continued imatinib 300 mg daily
- BCR-ABL1 monitoring every 3 months initially, then every 6 months if stability is maintained
- Annual evaluation for long-term TKI complications (bone density, cardiac function, metabolic parameters)
- Consideration for potential future TFR attempt after maintaining deep molecular response for at least 2 additional years

Session ID:
Assessment Domain

Session-82:
Rationale

Session ID:
Instructional Design and Pedagogical

Session-82:
The session's instructional design is of the highest quality. Learning objectives are explicitly stated for each phase and are clear, measurable, and perfectly aligned with the content. The case is expertly scaffolded, progressing logically from initial diagnosis and risk stratification through first-line therapy, response monitoring, side effect management, and advanced topics like Treatment-Free Remission (TFR). The feedback provided for each question is a significant strength, offering detailed rationales that are consistently anchored to current clinical guidelines (e.g., ELN 2022) and explain the clinical reasoning behind the correct answer. The overall framing and instructions are exceptionally clear and intuitive.

Session ID:
Clinical Authenticity and Content Fidelity

Session-82:
The clinical and pharmacotherapeutic content is flawless. Plausibility & Guideline Alignment: The clinical narrative is highly realistic and indistinguishable from an authentic patient case. All diagnostic criteria, risk stratification (ELN 2022), treatment milestones, and management decisions are perfectly aligned with current, evidence-based guidelines for Chronic Myeloid Leukemia (CML). Pharmacotherapeutic Accuracy: The session demonstrates expert-level pharmacotherapeutic accuracy. The nuanced discussion of first-line TKI selection (imatinib vs. second-generation TKIs for a low-risk patient), management of common side effects, criteria for TFR, and management of molecular recurrence are all handled correctly and reflect best practices. Domain Specificity: The case remains strictly within the CML domain. There is no evidence of domain entanglement; all diagnostics, therapies, and standards of care are specific and appropriate to CML management. The simulation's content is safe and educationally sound.

Session ID:
Modeled Clinical Reasoning

Session-82:
The simulation expertly models a sound clinical reasoning process. Each stage of the case presents a well-framed, relevant clinical problem, from diagnosis to long-term quality-of-life management. The multiple-choice options are well-constructed, providing a wide breadth of plausible alternatives and key distractors that effectively test nuanced understanding. The overall decision-making strategy is exemplary, appropriately prioritizing guideline-directed care, response-based monitoring, and patient-centered outcomes. The clinical outcomes described at each step (e.g., BCR-ABL1 kinetics, development of side effects) are realistic and highly instructive, reinforcing the learning objectives.

Session-83

Session ID:
LLM

Session-83:
Claude 3.7 Sonnet

Session ID:
Session Transcript

Session-83:
Phase 1: Initial Presentation and Diagnostic Workup
Case Introduction
Patient: Sarah Johnson, 42-year-old female, previously healthy
Chief Complaint: Progressive fatigue for 3 months, unintentional weight loss (5kg), night sweats, and left upper quadrant discomfort
Initial Vital Signs: T: 37.9C, HR: 92 bpm, BP: 128/76 mmHg, RR: 16, SpO2: 98% on room air
Physical Examination: Palpable splenomegaly (4cm below costal margin), no lymphadenopathy, otherwise unremarkable
Initial Laboratory Results:
- WBC: 89.5 10^9/L (elevated)
- Hemoglobin: 10.1 g/dL
- Platelets: 450 10^9/L
- Differential: 60% neutrophils, 8% lymphocytes, 5% monocytes, 3% eosinophils, 2% basophils, 22% immature granulocytes
- LDH: 780 U/L (elevated)
- Comprehensive metabolic panel: within normal limits
Question 1: Based on the patient's presentation and initial laboratory results, what is the most appropriate next diagnostic test?
A) Bone marrow aspiration and biopsy with cytogenetics
B) CT scan of chest, abdomen, and pelvis
C) Peripheral blood smear with FISH analysis for BCR-ABL
D) Flow cytometry of peripheral blood
E) Molecular HLA typing
Hint
Here's a helpful hint:
When evaluating a patient with significantly elevated WBC count, splenomegaly, and immature granulocytes in the differential, think about which diagnostic test would most definitively help establish a diagnosis of chronic myeloproliferative neoplasm, particularly CML. Consider which test would allow for both morphologic assessment and detection of characteristic genetic abnormalities associated with CML according to the WHO classification system. Which test would provide the most comprehensive initial diagnostic information?
Remember that according to current guidelines, diagnosis of CML requires demonstration of both the Philadelphia chromosome and/or BCR-ABL1 fusion gene along with morphologic assessment.
Answer c
Your answer is partially correct, but let's evaluate it more thoroughly.
Correct Answer: A) Bone marrow aspiration and biopsy with cytogenetics
Feedback:
While peripheral blood FISH analysis for BCR-ABL (option C) would detect the hallmark genetic abnormality in CML, the complete diagnostic workup for a patient with suspected CML should begin with bone marrow aspiration and biopsy with cytogenetics.
Here's why option A is preferred:
1. According to the 2022 ELN recommendations, bone marrow examination is essential for the initial diagnosis of CML as it provides:
   - Morphologic assessment of cell lines and precise blast percentage
   - Comprehensive cytogenetic analysis for Philadelphia chromosome (t(9;22))
   - Assessment for additional chromosomal abnormalities which impact prognosis
   - Material for FISH and molecular studies

2. While peripheral blood can be used for FISH and PCR detection of BCR-ABL1, it doesn't allow for complete morphologic assessment and cytogenetic analysis of the bone marrow, which is crucial for:
   - Determining the phase of CML (chronic, accelerated, or blast)
   - Excluding other myeloproliferative disorders
   - Establishing a baseline for future monitoring
After ordering a bone marrow aspiration and biopsy with cytogenetics, the following results return:
Bone Marrow Results:
- Hypercellular marrow (90%)
- Myeloid:erythroid ratio = 10:1 (increased)
- 5% blasts
- Marked granulocytic hyperplasia with full spectrum of maturation
- Cytogenetics: 46,XX,t(9;22)(q34;q11.2) in 20/20 metaphases
- FISH: BCR-ABL1 fusion positive in 95% of cells
- Molecular studies: BCR-ABL1/ABL1 ratio (IS) = 78%
Question 2: Based on these findings, what is the correct diagnosis and disease phase for this patient?
A) CML, chronic phase
B) CML, accelerated phase
C) CML, blast phase
D) Atypical CML
E) Chronic neutrophilic leukemia
Hint
Let me provide you with a more specific hint:
According to the 2022 ELN criteria and WHO classification:
- Chronic phase CML is characterized by <10% blasts in peripheral blood and bone marrow
- Accelerated phase is defined by 10-19% blasts
- Blast phase is defined by 20% blasts
In this case, your patient has 5% blasts in the bone marrow, positive Philadelphia chromosome t(9;22), and BCR-ABL1 fusion gene. Consider how these specific findings align with the phase criteria, particularly the blast percentage threshold.
Answer A
Correct! Well done.
A) CML, chronic phase is indeed the correct diagnosis.
Feedback:
Sarah's diagnostic findings clearly indicate Chronic Myeloid Leukemia in chronic phase based on the following criteria:
1. **Definitive CML diagnosis is supported by:**
   - Presence of t(9;22) Philadelphia chromosome in all examined metaphases
   - Positive BCR-ABL1 fusion by FISH (95% of cells)
   - High BCR-ABL1/ABL1 ratio of 78% (IS)
   - Characteristic hypercellular marrow with myeloid hyperplasia
2. **Chronic phase is confirmed by:**
   - Blast percentage of 5%, which is below the 10% threshold for accelerated phase
   - Absence of additional chromosomal abnormalities that would suggest disease progression
   - No evidence of extramedullary blast proliferation
According to the 2022 ELN recommendations, CML phases are defined as:
- Chronic phase: <10% blasts in peripheral blood or bone marrow
- Accelerated phase: 10-19% blasts
- Blast phase: 20% blasts or extramedullary blast proliferation
4+AH7:AH19
Hint
Here's a helpful hint:
The EUTOS Long-Term Survival (ELTS) score is calculated based on four variables:
1. Age
2. Spleen size (cm below costal margin)
3. Platelet count
4. Peripheral blood blast percentage

Remember the formula:
ELTS score = 0.0025 (age/10)^3 + 0.0615 spleen size + 0.1052 peripheral blood blasts + 0.4104 (platelet count/1000)^-0.5
Risk categories are defined as:
- Low risk: ELTS score 1.5680
- Intermediate risk: 1.5680 < ELTS score 2.2185
- High risk: ELTS score > 2.2185
Check if you have all the necessary information from the case to calculate this score.
Answer A
Correct! Excellent work.
A) Low risk is the correct risk classification for this patient based on the ELTS score.
Feedback:
The calculation of the ELTS score for Sarah yields 1.2531, which falls into the low-risk category (1.5680).
ELTS components calculation:
- Age component: 0.0025 (42/10) = 0.1852
- Spleen component: 0.0615 4 = 0.246
- Blasts component: 0.1052 2 = 0.2104
- Platelet component: 0.4104 (450/1000)^-0.5 = 0.6115
- Total: 1.2531
This places Sarah in the low-risk category, which has important implications:
- Better long-term survival prognosis (8-year overall survival >95%)
- Lower probability of progression to accelerated/blast phase
- May influence treatment goals and monitoring frequency
 Phase 2: Induction Therapy and Supportive Care
With the diagnosis of CML in chronic phase and a low-risk ELTS score established, the medical team discusses treatment options with Sarah.
Patient Factors:
- No significant comorbidities
- Normal renal and hepatic function
- No history of cardiovascular disease
- No relevant drug allergies
- Plans to have children in the future (not currently pregnant)
- Health insurance covers all TKI options
Question 4: What is the most appropriate first-line treatment for this patient?
A) Imatinib 400 mg once daily
B) Dasatinib 100 mg once daily
C) Nilotinib 300 mg twice daily
D) Bosutinib 400 mg once daily
E) Ponatinib 45 mg once daily
Hint
Let me provide a more specific hint regarding pregnancy considerations:
According to the 2022 ELN recommendations for patients with reproductive potential:
- Imatinib is the only TKI with substantial data regarding exposure during pregnancy
- For women planning pregnancy, imatinib has been used successfully with specific management recommendations
- Second and third-generation TKIs (dasatinib, nilotinib, bosutinib, ponatinib) are generally contraindicated during pregnancy with limited safety data
- For low-risk patients with future pregnancy plans, this factor may significantly influence TKI selection
Consider how Sarah's desire to have children in the future might impact your decision for first-line therapy, balancing this with efficacy needs for her low-risk disease.
Answer A
Correct! Well done.
A) Imatinib 400 mg once daily is the most appropriate choice for this patient.
Feedback:
Imatinib is the optimal first-line choice for Sarah based on several key factors:
1. **Pregnancy planning**: Most importantly, Sarah's desire for future pregnancy strongly favors imatinib, as it has the most safety

data in this context. The 2022 ELN guidelines specifically note that imatinib has been used successfully in patients planning pregnancy, while second and third-generation TKIs are contraindicated during pregnancy.

2. **Risk category**: With a low-risk ELTS score, Sarah has an excellent prognosis even with imatinib, making the potentially deeper responses of second-generation TKIs less critical in her case.

3. **Long-term safety**: Imatinib has the longest follow-up data (>20 years) with well-characterized long-term safety profile and fewer cardiovascular adverse events compared to nilotinib and dasatinib.

4. **Proven efficacy**: For a low-risk patient, imatinib provides suitable efficacy with 8-year survival rates >90%, and many patients achieve deep molecular responses that may eventually allow for treatment-free remission attempts.

While second-generation TKIs (dasatinib, nilotinib, bosutinib) offer faster and deeper molecular responses, they carry more specific toxicity concerns and lack substantial pregnancy safety data. Ponatinib is typically reserved for T315I mutations or failure of multiple prior TKIs.

Let's continue with the case.

Sarah begins imatinib 400 mg daily. After two weeks of therapy, she calls the clinic reporting:

- Moderate periorbital edema
- Occasional muscle cramps in her legs
- Mild nausea after taking the medication
- No significant changes in her blood counts

Question 5: What is the most appropriate management strategy for Sarah's side effects?

A) Discontinue imatinib and switch to nilotinib

B) Reduce imatinib dose to 300 mg daily

C) Continue imatinib 400 mg daily with supportive measures for side effects

D) Temporarily hold imatinib for 1 week, then restart at 300 mg daily

E) Continue imatinib but change to twice daily dosing (200 mg BID)

Hint

Let me provide a more specific hint about managing these particular side effects:

According to current guidelines, including ELN 2022 recommendations:

- Periorbital edema with imatinib is usually manageable with supportive measures (salt restriction, topical treatments, occasionally low-dose diuretics)
- Muscle cramps often respond to calcium and magnesium supplementation, tonic water, or physical measures
- Mild nausea can be addressed by taking imatinib with food, dividing doses, or using antiemetics as needed

These particular side effects are:

1. Expected with imatinib (occurring in 30-60% of patients)
2. Usually grade 1-2 (mild to moderate)
3. Often improve over time with continued therapy
4. Rarely require dose modifications if properly managed

Consider whether these specific side effects warrant changing the treatment plan or if they can be effectively managed while maintaining the therapeutic dose needed for optimal outcomes.

Answer D

Incorrect.

Correct Answer: C) Continue imatinib 400 mg daily with supportive measures for side effects

Feedback:

The side effects Sarah is experiencing (periorbital edema, muscle cramps, mild nausea) are common, expected, and typically mild-to-moderate reactions to imatinib therapy. According to the 2022 ELN guidelines and NCCN recommendations:

1. **Dose maintenance is crucial**: Maintaining the full 400 mg dose of imatinib during early treatment is important for achieving optimal responses. Dose reductions or interruptions should generally be reserved for grade 3-4 toxicities.

2. **These specific side effects have established management strategies**:
   - Periorbital edema: Salt restriction, topical eye drops, mild diuretics if needed
   - Muscle cramps: Calcium supplements, magnesium supplements, tonic water, hydration
   - Mild nausea: Taking imatinib with food, antiemetics as needed, ensuring adequate hydration

3. **Early discontinuation impacts outcomes**: Temporarily holding imatinib (option D) during the critical early treatment period could negatively impact response milestones and long-term outcomes.

4. **These side effects typically improve**: Many patients develop tolerance to these adverse effects over time with continued therapy.

Sarah continues imatinib therapy with appropriate supportive care measures. Her side effects gradually improve over time.

At the 3-month follow-up:
- Complete hematologic response achieved (normalized blood counts)
- Spleen no longer palpable
- Molecular testing shows: BCR-ABL1/ABL1 ratio (IS) = 9.2%

Question 6: How would you interpret this 3-month molecular response?

A) Optimal response - continue current management
B) Warning response - continue current management with more frequent monitoring
C) Failure response - switch to a second-generation TKI
D) Disease progression - consider allogeneic stem cell transplantation
E) Unable to assess without cytogenetic testing

Hint

Here's a helpful hint:

According to the 2022 ELN guidelines, there are specific milestones for monitoring the response to TKI therapy in CML patients.

At the 3-month timepoint, molecular response is categorized as:
- Optimal response: BCR-ABL1 10% (IS)
- Warning: BCR-ABL1 >10% (IS)
- Failure: No CHR and/or Ph+ >95%

Consider Sarah's BCR-ABL1 value of 9.2% at 3 months and determine which category this falls into based on these specific cutoffs. Also consider what her complete hematologic response means in this context.

Remember that these response milestones guide subsequent management decisions according to the guidelines, so correctly categorizing the response is essential for determining the appropriate next steps.

Answer A

Correct! Well done.

A) Optimal response - continue current management is the correct interpretation of Sarah's 3-month response.

Feedback:

According to the 2022 ELN recommendations, Sarah's response at 3 months is categorized as optimal based on:
1. Her BCR-ABL1 transcript level of 9.2% IS is 10%, which meets the definition of an optimal molecular response at this timepoint
2. She has achieved a complete hematologic response (normalized blood counts)
3. Her spleen has normalized in size

In this optimal response scenario, the ELN guidelines recommend:
- Continuing the current imatinib dose (400 mg daily)
- Standard monitoring with BCR-ABL1 quantification every 3 months
- No indication for treatment change at this point

It's important to note that while this is an optimal early response, continued adherence and monitoring are essential, with the next important milestone being the 6-month molecular assessment (where BCR-ABL1 1% IS would represent an optimal response).

Sarah continues imatinib therapy with good adherence. At her 6-month follow-up:
- Sustained complete hematologic response
- BCR-ABL1/ABL1 ratio (IS) = 1.8%
- No significant adverse events

During this visit, she mentions that she and her husband are considering starting a family within the next 12 months. She asks about the implications of her CML treatment on pregnancy.

Question 7: What is the most appropriate recommendation regarding pregnancy planning for this patient?

A) Stop imatinib immediately and attempt conception
B) Switch to interferon-alpha therapy now and attempt conception in 3 months
C) Continue imatinib for at least 2 years and achieve sustained DMR (MR4.0) before attempting conception
D) Switch to nilotinib to achieve faster deep molecular response before conception
E) Continue imatinib until achieving at least MMR, then discontinue 1 month before conception with careful monitoring

Hint

Here's a helpful hint:

According to the 2022 ELN recommendations and ESMO guidelines for CML management during pregnancy planning:
1. TKI therapy is generally contraindicated during pregnancy due to potential teratogenicity
2. There are specific recommendations regarding:
   - The minimum response level that should be achieved before considering TKI discontinuation
   - The timing of TKI discontinuation before conception attempts

- Alternative management strategies during pregnancy
- Monitoring requirements during pregnancy

Consider the importance of achieving at least a stable major molecular response (MMR, BCR-ABL1 0.1% IS) before attempting conception, as this provides a safety margin for potential loss of response during TKI discontinuation.

Also consider the timing of when TKI therapy should be discontinued before conception attempts, particularly for imatinib (which has a specific washout period recommendation).

Answer E

Correct! Excellent work.

E) Continue imatinib until achieving at least MMR, then discontinue 1 month before conception with careful monitoring is the most appropriate recommendation.

Feedback:

According to the 2022 ELN recommendations and ESMO guidelines for pregnancy planning in CML:

1. **Response milestone**: Achieving and maintaining at least a major molecular response (MMR, BCR-ABL1 0.1% IS) before attempting conception is recommended. Sarah currently has BCR-ABL1 of 1.8%, which is not yet MMR.
2. **Timing of discontinuation**: For women planning pregnancy, imatinib should be discontinued approximately 1 month before conception attempts due to its teratogenic potential.
3. **Monitoring during pregnancy**: Once pregnant, monthly molecular monitoring is recommended without TKI therapy if the patient had a stable MMR or better prior to conception.
4. **Alternative management**: Interferon-alpha can be considered during pregnancy if treatment is required, but is not needed prophylactically if good response was achieved before conception.
5. **Post-delivery**: TKI therapy should be resumed immediately after delivery, and breastfeeding is not recommended while on TKI therapy.

The other options are incorrect because:

- Option A: Immediate discontinuation without achieving MMR is associated with higher risk of relapse
- Option B: Preemptive switch to interferon is unnecessary if planning to achieve MMR first
- Option C: Requiring 2 years of therapy and DMR is overly restrictive when MMR is sufficient
- Option D: Nilotinib has no advantage over imatinib for pregnancy planning and has more cardiovascular concerns

Phase 3: Post-Remission Therapy and Long-Term Management

Sarah continues imatinib therapy and achieves MMR (BCR-ABL1 0.08% IS) after 12 months of treatment. After discussing with her hematologist and clinical pharmacist, she discontinues imatinib one month before attempting conception. She becomes pregnant 3 months later.

During pregnancy monitoring:

- Monthly molecular monitoring shows stable low-level disease (BCR-ABL1 ranging from 0.09-0.12%)
- No hematologic relapse
- Normal pregnancy progression

She delivers a healthy baby boy and immediately restarts imatinib therapy without breastfeeding. Six months after restarting imatinib, her BCR-ABL1 level returns to MMR (0.05%).

At her 3-year follow-up from initial diagnosis:

- Sustained deep molecular response (DMR) with BCR-ABL1 0.004% (MR4.5) for >12 months
- Excellent tolerance of imatinib with no significant adverse effects
- Normal quality of life
- No plans for additional pregnancies

She asks about the possibility of stopping therapy.

Question 8: What is the most appropriate recommendation regarding treatment-free remission (TFR) for this patient?

A) Not eligible for TFR attempt due to history of treatment interruption during pregnancy
B) Eligible for TFR attempt now with molecular monitoring every 4-6 weeks for the first 6 months
C) Continue imatinib for at least 5 years total before considering TFR
D) Switch to second-generation TKI to deepen response before TFR attempt
E) TFR is only recommended in clinical trial settings and should not be attempted

Hint

Let me provide a more specific hint regarding TFR eligibility criteria:

The 2022 ELN recommendations specifically state that for TFR eligibility:

1. Minimum TKI therapy duration: At least 3 years (Sarah has received approximately 3 years minus pregnancy interruption)
2. Depth of response: At least MR4 (BCR-ABL1 0.01% IS), preferably MR4.5 (BCR-ABL1 0.0032% IS)

3. Duration of deep response: Sustained for at least 1-2 years
4. Monitoring requirements during TFR: BCR-ABL1 testing every 4-6 weeks in the first 6 months, every 2-3 months for months 6-12, and every 3-6 months thereafter
5. Prior treatment interruptions: The guidelines do not explicitly exclude patients with prior planned treatment interruptions (like pregnancy) if they've regained and maintained deep molecular response afterward
Consider whether Sarah meets these specific criteria given her history and current status.
Answer B
Correct! Well done.
B) Eligible for TFR attempt now with molecular monitoring every 4-6 weeks for the first 6 months is the most appropriate recommendation.
Feedback:
According to the 2022 ELN recommendations, Sarah meets all criteria for a TFR attempt:
1. **Duration of treatment**: Sarah has received approximately 3 years of TKI therapy (minus the pregnancy interruption), meeting the minimum 3-year requirement.
2. **Depth of response**: She has achieved MR4.5 (BCR-ABL1 0.004%), which exceeds the minimum requirement of MR4 (BCR-ABL1 0.01%).
3. **Duration of deep response**: She has maintained this deep response for >12 months, meeting the recommendation of sustained DMR for at least 1-2 years.
4. **Prior interruption**: Her planned treatment interruption for pregnancy with successful return to DMR afterward does not disqualify her from TFR attempts.
5. **Monitoring protocol**: The correct monitoring frequency is indeed every 4-6 weeks for the first 6 months, then every 2-3 months from months 6-12, and every 3-4 months thereafter.
The guidelines explicitly state that patients who have achieved these criteria can attempt TFR, with approximately 50% of patients maintaining treatment-free remission long-term.
Sarah decides to attempt treatment-free remission (TFR) after appropriate counseling. Her TKI is discontinued, and she undergoes careful molecular monitoring.
Monitoring results after TFR:
- Month 1: BCR-ABL1 0.009% (MR4.0)
- Month 2: BCR-ABL1 0.032% (MMR)
- Month 3: BCR-ABL1 0.12% (MMR)
- Month 4: BCR-ABL1 0.25% (loss of MMR)
- Month 5: BCR-ABL1 0.42% (confirmed loss of MMR)
Question 9: What is the most appropriate management at this point?
A) Continue monitoring without restarting therapy as BCR-ABL1 is still <1%
B) Restart imatinib at 400 mg daily
C) Restart imatinib at 600 mg daily
D) Switch to nilotinib 300 mg twice daily
E) Switch to dasatinib 100 mg daily
Hint
Here's a helpful hint:
According to the 2022 ELN recommendations for managing molecular relapse after TFR attempt:
1. There are specific BCR-ABL1 thresholds that define "molecular relapse" and necessitate treatment reinitiation after a TFR attempt
2. These definitions differ from the standard response monitoring during continuous treatment
3. The most widely accepted criterion for treatment reinitiation is loss of major molecular response (MMR), defined as BCR-ABL1 >0.1% IS confirmed in a subsequent test
Consider Sarah's molecular monitoring results showing BCR-ABL1 levels that have progressively increased over time, with her last two measurements showing values >0.1% IS.
Review the guidelines regarding:
- What constitutes a trigger for treatment reinitiation
- Which treatment should be restarted (previous effective therapy vs. switch)
- What dose should be used (standard vs. increased)
Answer B
Correct! Excellent work.
B) Restart imatinib at 400 mg daily is the most appropriate management.

Feedback:
According to the 2022 ELN recommendations for TFR management:
1. **Definition of molecular relapse**: Loss of MMR (BCR-ABL1 >0.1% IS) is the established trigger for treatment reinitiation. Sarah's BCR-ABL1 has increased to 0.25% and was confirmed at 0.42%, clearly indicating loss of MMR.
2. **Choice of therapy**: The guidelines specifically recommend restarting the same TKI that was previously effective, at the same dose. For Sarah, this means returning to imatinib 400 mg daily.
3. **Expected response**: Almost all patients who restart TKI therapy after molecular relapse regain MMR and deep molecular response, often more quickly than during initial treatment.
4. **Dose considerations**: The standard dose (400 mg for imatinib) is appropriate; dose escalation (600 mg) is not recommended unless there was previous evidence of resistance.
5. **TKI switching**: There is no evidence supporting switching to a second-generation TKI at molecular relapse after TFR if the patient previously responded well to imatinib.
Sarah restarts imatinib 400 mg daily and regains MMR within 3 months. At her 5-year follow-up from initial diagnosis:
- Maintained MR4.5 (BCR-ABL1 0.002%) for the past 18 months after restarting therapy
- Tolerating imatinib well with minimal side effects
- Considering a second TFR attempt in the future
During routine monitoring, conventional PCR testing unexpectedly shows no detectable BCR-ABL1 transcript, confirmed on repeat testing with high-sensitivity digital PCR.
Question 10: What is the most appropriate next step in Sarah's management?
A) Discontinue all monitoring as she is functionally cured
B) Switch to nilotinib to maintain the undetectable status
C) Continue current therapy and monitoring schedule as recommended by guidelines
D) Order next-generation sequencing to check for atypical BCR-ABL1 transcripts
E) Reduce imatinib dose to 200 mg daily to minimize long-term toxicity
Hint
Here's a helpful hint:
According to the 2022 ELN recommendations for long-term management of CML patients:
1. Consider the significance of undetectable BCR-ABL1 transcripts by conventional and digital PCR methods
2. Review the guidelines' stance on whether this represents a "cure" that warrants discontinuation of monitoring
3. Think about whether this finding should change the treatment approach (continuing current effective therapy vs. switching or dose reduction)
4. Consider whether this finding might indicate the need for additional diagnostic investigations
Remember that even with undetectable transcripts, the guidelines have specific recommendations regarding:
- The continuation of monitoring and therapy
- The possibility of future TFR attempts after previous molecular relapse
- The definition of "operational cure" in CML
Answer C
Correct! Excellent work.
C) Continue current therapy and monitoring schedule as recommended by guidelines is the most appropriate approach.
Feedback:
According to the 2022 ELN recommendations for long-term CML management:
1. **Undetectable BCR-ABL1**: Even when BCR-ABL1 becomes undetectable by highly sensitive methods (including digital PCR), this does not represent a "cure" but rather an extremely deep molecular response (sometimes called MR5 or deeper).
2. **Continued monitoring required**: The guidelines explicitly recommend continuing regular monitoring even with undetectable transcripts, as leukemic stem cells may persist below detection limits.
3. **Effective therapy continuation**: The recommendation is to continue the current effective therapy (imatinib 400 mg) at the same dose without modifications.
4. **Future TFR consideration**: Patients who have previously attempted TFR and experienced molecular relapse can still be considered for subsequent TFR attempts after regaining and maintaining deep molecular response for at least 2 years.
5. **Long-term management**: CML is still considered a chronic disease requiring long-term management, even with undetectable disease.
The other options are incorrect because:
- Option A: No monitoring would be inappropriate as CML is not considered "cured"
- Option B: Switching to nilotinib offers no benefit when imatinib is effective with undetectable disease

- Option D: NGS for atypical transcripts is unnecessary with confirmed undetectable transcripts by digital PCR
- Option E: Dose reduction could compromise the excellent response achieved

---

Session ID:
Assessment Domain

Session-83:
Rationale

Session ID:
Instructional Design and Pedagogical

Session-83:
The session's instructional design is of very high quality. The case is expertly scaffolded, guiding the learner through the entire CML care continuum, from initial workup and diagnosis to complex long-term management scenarios like pregnancy planning and treatment-free remission (TFR). The quality of the embedded feedback is a major strength; each rationale is detailed, appropriately cites current clinical guidelines (e.g., ELN 2022), and explains the clinical reasoning behind the correct answer. The case presentation and questions are exceptionally clear. The learning objectives are not explicitly stated at the beginning, which is a minor area for improvement, but the educational goals are strongly implied by the comprehensive and logical structure of the session.

Session ID:
Clinical Authenticity and Content Fidelity

Session-83:
The clinical authenticity and content fidelity of this session are excellent. The clinical narrative is highly plausible and mirrors an authentic patient journey, including realistic response timelines and common clinical decision points. The therapeutic recommendations and monitoring milestones (e.g., 3-month BCR-ABL1 10%, criteria for TFR, management of molecular relapse) are perfectly aligned with current, evidence-based guidelines, specifically the 2022 ELN recommendations. All pharmacotherapeutic choices are optimal and well-justified within the context of the case. Crucially, the session demonstrates perfect domain specificity; all concepts, diagnostics, and therapies are appropriate for CML, with no evidence of domain entanglement.

Session ID:
Modeled Clinical Reasoning

Session-83:
The simulation expertly models a high-level clinical reasoning process. Each stage of the case presents a well-framed clinical problem with the necessary data for an informed decision. The multiple-choice options are excellent, providing a strong breadth of relevant and plausible alternatives that include key distractors, effectively testing nuanced clinical understanding (e.g., selecting among first-line TKIs based on patient factors). The modeled decision-making demonstrates sound prioritization throughout, correctly balancing efficacy, safety, and patient-specific goals. The clinical outcomes described for each decision are realistic and highly instructive, reinforcing the learning objectives at each step.

Session-84

Session ID:
LLM

Session-84:
Claude 3.7 Sonnet

Session ID:
Session Transcript

Session-84:
Phase 1: Initial Presentation and Diagnostic Workup
A 42-year-old male presents to the emergency department with complaints of fatigue, unintentional weight loss (8kg over 3 months), early satiety, and left upper quadrant discomfort. Physical examination reveals significant splenomegaly (palpable 6cm below the costal margin). Initial laboratory findings:

WBC: 146,000/L (elevated)
Hemoglobin: 10.2 g/dL (decreased)
Platelets: 445,000/L (normal)
Peripheral blood smear: Left-shifted myeloid cells with all stages of maturation visible
Bone marrow biopsy: Hypercellular marrow (95%) with myeloid hyperplasia
Cytogenetics: t(9;22)(q34;q11.2) [Philadelphia chromosome] detected in 92% of metaphases
RT-PCR: BCR-ABL1 (p210) fusion transcript detected

Question 1: Based on the initial presentation and laboratory findings, what is the most likely diagnosis for this patient?
A. Chronic Myeloid Leukemia (CML) in chronic phase
B. Chronic Myeloid Leukemia (CML) in accelerated phase
C. Acute Myeloid Leukemia (AML)
D. Myelofibrosis with myeloid metaplasia
E. Chronic Myelomonocytic Leukemia (CMML)
That's correct! The patient presents with classic findings of CML in chronic phase, including leukocytosis with a left-shifted myeloid series, splenomegaly, and the presence of the Philadelphia chromosome with BCR-ABL1 fusion transcript. According to the WHO classification, these findings are diagnostic for CML in chronic phase.
According to the WHO 2016/2022 classification and the European LeukemiaNet (ELN) 2022 recommendations, CML diagnosis requires the presence of the Philadelphia chromosome t(9;22)(q34;q11.2) or the BCR-ABL1 fusion gene, along with appropriate clinical and laboratory findings.
Question 2: Which risk stratification score would be most appropriate to use for this patient according to current guidelines?
A. IPSS-R (Revised International Prognostic Scoring System)
B. ELTS (EUTOS Long-Term Survival) score
C. ELN genetic risk classification
D. DIPSS (Dynamic International Prognostic Scoring System)
E. HCT-CI (Hematopoietic Cell Transplantation-Comorbidity Index)
Please select one option (A, B, C, D, or E). Type HINT if you need guidance.
That's not correct. The ELN genetic risk classification is primarily used for acute myeloid leukemia (AML), not CML.
According to the 2022 ELN recommendations for CML, the ELTS score is the preferred prognostic scoring system. It incorporates age, spleen size, platelet count, and peripheral blood blast percentage to stratify patients into low, intermediate, and high-risk categories.
Question 3: Which molecular monitoring technique is considered the gold standard for quantifying BCR-ABL1 transcript levels and monitoring response to therapy in CML?
A. Fluorescence in situ hybridization (FISH)
B. Next-generation sequencing (NGS)
C. Quantitative reverse transcriptase PCR (qRT-PCR)

D. Conventional cytogenetics (karyotyping)
E. Digital droplet PCR (ddPCR)
Please select one option (A, B, C, D, or E). Type HINT if you need guidance.
Correct! Quantitative reverse transcriptase PCR (qRT-PCR) is the gold standard for monitoring BCR-ABL1 transcript levels. It provides high sensitivity and specificity and allows for standardized reporting using the International Scale (IS), where BCR-ABL1/ABL1 ratio of 0.1% corresponds to major molecular response (MMR).
The ELN 2022 recommendations and ESMO guidelines both emphasize the use of qRT-PCR for monitoring BCR-ABL1 transcript levels, with results reported on the International Scale (IS) to ensure standardization across laboratories.
Moving to the next phase of the case.
Phase 2: Induction Therapy Selection and Supportive Care Planning
Based on further workup, the patient has been diagnosed with Chronic Myeloid Leukemia in chronic phase (CML-CP).
Additional patient information:

ECOG Performance Status: 1
Past Medical History: Hypertension (well-controlled on amlodipine 5mg daily)
No significant hepatic or renal dysfunction
Sokal score: Intermediate risk
EUTOS score: Low risk
No HLA-matched siblings available for potential stem cell transplantation

The patient has commercial insurance with good prescription coverage.
Question 4: Based on the patient's characteristics and current guidelines, which first-line treatment option would be most appropriate for this patient with newly diagnosed CML in chronic phase?
A. Imatinib 400 mg daily
B. Dasatinib 100 mg daily
C. Nilotinib 300 mg twice daily
D. Bosutinib 400 mg daily
E. Ponatinib 45 mg daily
Please select one option (A, B, C, D, or E). Type HINT if you need guidance
Correct! Imatinib 400 mg daily is an appropriate first-line treatment for this patient with intermediate Sokal risk CML-CP. It has a well-established long-term safety profile and effectiveness. While second-generation TKIs (dasatinib, nilotinib, bosutinib) achieve faster and deeper molecular responses, they have not demonstrated a clear overall survival advantage over imatinib in first-line setting and have different toxicity profiles.
According to the ELN 2022 recommendations and ESMO guidelines, all approved TKIs (imatinib, dasatinib, nilotinib, and bosutinib) are options for first-line therapy. The choice should be individualized based on patient characteristics, comorbidities, risk profile, and treatment goals. Imatinib has the longest track record of safety data and remains an appropriate first-line option.
Question 5: Which of the following is NOT a recommended baseline assessment prior to initiating TKI therapy in this CML patient?
A. Electrocardiogram (ECG)
B. Hepatitis B serology
C. Pulmonary function tests
D. Lipid profile
E. Complete blood count with differential
Please select one option (A, B, C, D, or E). Type HINT if you need guidance.
That's not correct. Hepatitis B serology is recommended as TKIs can cause hepatotoxicity and reactivation of hepatitis B has been reported.
The ELN 2022 recommendations and NCCN guidelines suggest baseline assessments before TKI therapy should include complete blood count, comprehensive metabolic panel, lipid profile, HBV serology, pregnancy test when applicable, and ECG (especially for QT-prolonging TKIs). Pulmonary function tests are not routinely recommended for all patients but may be considered in specific situations.
Question 6: The patient has started imatinib 400 mg daily and presents 3 weeks later with grade 3 neutropenia (ANC 0.8 10^9/L) but is otherwise asymptomatic. What is the most appropriate management strategy?
A. Continue imatinib at current dose with weekly CBC monitoring
B. Temporarily discontinue imatinib until ANC recovers to >1.0 10^9/L, then resume at reduced dose (300 mg daily)
C. Permanently discontinue imatinib and switch to nilotinib

D. Continue imatinib and start G-CSF (filgrastim) until neutropenia resolves
E. Reduce imatinib dose to 300 mg daily and continue treatment
Please select one option (A, B, C, D, or E). Type HINT if you need guidance.
That's not correct. While G-CSF can be used in patients with recurrent or persistent neutropenia, it is not recommended as first-line management before attempting dose adjustment strategies.
According to ELN recommendations and NCCN guidelines for CML, management of grade 3 neutropenia (ANC 0.5-1.0 10^9/L) typically involves temporarily withholding TKI therapy until recovery to ANC >1.0 10^9/L, then resuming at a reduced dose. G-CSF may be considered for recurrent episodes but is not first-line management.
Question 7: The patient's neutropenia resolves, and he continues imatinib therapy at 300 mg daily. After 3 months of therapy, BCR-ABL1 transcript level is 25% (IS). According to current guidelines, how would you classify this response?
A. Optimal response
B. Warning (previously called 'suboptimal response')
C. Treatment failure
D. Complete cytogenetic response (CCyR)
E. Major molecular response (MMR)
Please select one option (A, B, C, D, or E). Type HINT if you need guidance.
That's not correct. Major molecular response (MMR) is defined as BCR-ABL1 0.1% (IS), which this patient has not achieved.
According to the ELN 2022 recommendations, response to TKI therapy at 3 months is classified as: Optimal (BCR-ABL1 10%), Warning (BCR-ABL1 >10% but 65%), or Failure (BCR-ABL1 >65% and/or new mutations). Patients with a 'warning' response should be monitored more frequently and may need intervention if no improvement occurs by 6 months.
Moving to the next phase of the case.
Phase 3: Post-Remission Therapy, MRD Monitoring, and Long-Term Management
The patient has now been on TKI therapy for 12 months. Follow-up assessments:

BCR-ABL1 transcript levels (IS):

3 months: 9.2%
6 months: 2.1%
9 months: 0.58%
12 months: 0.12%

Complete blood count has normalized
Spleen is no longer palpable
Patient reports mild to moderate muscle cramps and occasional headaches
Patient is adherent to medication (~95% of doses taken)
No signs of disease progression

Question 8: At the 12-month mark, the patient has achieved BCR-ABL1 0.12% (IS). How would you classify this molecular response?
A. No molecular response
B. Minor molecular response (MR2)
C. Major molecular response (MR3)
D. Deep molecular response (MR4)
E. Complete molecular response (MR5)
Please select one option (A, B, C, D, or E). Type HINT if you needed guidance
That's not correct. Minor molecular response (MR2) would correspond to BCR-ABL1 1% but >0.1%, which is a lesser degree of response than what this patient has achieved.
According to the ELN 2022 recommendations and International Scale for BCR-ABL1 monitoring, molecular responses are classified as: MR2 (BCR-ABL1 1%), MR3 or major molecular response (BCR-ABL1 0.1%), MR4 (BCR-ABL1 0.01%), and MR5 (BCR-ABL1 0.001%). An MR3 at 12 months is considered an optimal response.
Question 9: The patient has maintained a stable major molecular response (MR3) for 24 months and is interested in eventually discontinuing TKI therapy. Which criterion is NOT required for attempting treatment-free remission (TFR) according to current guidelines?
A. Sustained deep molecular response (MR4 or better) for at least 2 years

B. Prior treatment with TKIs for at least 3 years
C. No history of accelerated or blast phase
D. Access to high-quality, standardized qRT-PCR with rapid turnaround time
E. Complete cytogenetic response confirmed by bone marrow biopsy within 90 days of TKI discontinuation
Please select one option (A, B, C, D, or E). Type HINT if you need guidance.
That's not correct. Access to reliable, standardized molecular testing with quick turnaround time is essential for the close monitoring required during TFR.
According to the ELN 2022 recommendations and NCCN guidelines, key criteria for attempting TFR include: sustained deep molecular response (MR4 or better) for at least 2 years, TKI therapy for at least 3 years, typical BCR-ABL1 transcripts that can be quantified, no prior history of accelerated or blast phase, and access to prompt, standardized qRT-PCR monitoring. Bone marrow cytogenetic analysis prior to discontinuation is not uniformly required.
Question 10: After 5 years of TKI therapy, the patient has maintained deep molecular response (MR4.5) for over 2 years. He decides to attempt treatment-free remission with proper monitoring. Which monitoring schedule is most appropriate during the first 6 months after TKI discontinuation?
A. Monthly molecular monitoring
B. Molecular monitoring every 3 months
C. Molecular monitoring every 6 months
D. Weekly molecular monitoring for the first month, then monthly
E. Bone marrow examination every 3 months
Please select one option (A, B, C, D, or E). Type HINT if you need guidance.
Correct! Monthly molecular monitoring with standardized qRT-PCR is recommended during the first 6 months after TKI discontinuation. This frequency is important to detect early molecular relapse, which most commonly occurs within the first 6 months of TKI discontinuation.
According to the ELN 2022 recommendations and NCCN guidelines for CML, the recommended monitoring schedule during TFR is: monthly molecular monitoring for the first 6 months, every 2 months for months 6-12, and every 3 months thereafter. Prompt reinitiation of TKI therapy is recommended if loss of major molecular response (BCR-ABL1 >0.1%) is detected.
Session Complete - Performance Assessment for Mohamed Adel
Score: 5/10 (50.0%)
You demonstrated some understanding of CML management, but significant improvement is needed in applying guideline-based approaches.
Strengths:

Understanding of the diagnostic criteria for CML
Application of risk stratification tools
Recognition of molecular monitoring techniques

Areas for Improvement:

Management of TKI-associated adverse events
Interpretation of treatment response milestones
Criteria for treatment-free remission

Key Guideline-Based Recommendations for this Case:

Diagnostic confirmation requires detection of Philadelphia chromosome or BCR-ABL1 fusion gene
The ELTS score is preferred for risk stratification in CML

Session ID:
Assessment Domain

Session-84:
Rationale

Session ID:

Instructional Design and Pedagogical

Session-84:

The session's instructional design is very strong. The case is expertly scaffolded, progressing logically from diagnosis and risk stratification to first-line therapy, adverse event management, and long-term monitoring for treatment-free remission. Instructions are exceptionally clear. The embedded feedback is largely excellent, consistently citing current guidelines (ELN, NCCN) and explaining the rationale for correct and incorrect answers. Learning objectives are not explicitly stated at the outset but are clearly implied by the session's structure and the final performance summary. The score for feedback quality was reduced from 5 to 4 due to a significant content error in the feedback for Question 8, which undermines its otherwise high quality.

Session ID:

Clinical Authenticity and Content Fidelity

Session-84:

The clinical fidelity of this session is compromised by a single but significant content error. Plausibility & Domain Specificity: The clinical narrative is indistinguishable from an authentic patient case, presenting a textbook example of CML-CP. The simulation correctly remains within the CML domain, avoiding any domain entanglement by appropriately distinguishing CML standards (e.g., ELTS score) from those of other leukemias. Guideline Alignment & Pharmacotherapeutic Error: The session is mostly aligned with guidelines. However, a critical error occurs in Question 8. The patient's BCR-ABL1 level is 0.12% (IS), which, by definition, is a Minor Molecular Response (MR2), as it is >0.1%. The simulation incorrectly marks "Minor molecular response (MR2)" as the wrong answer. This misrepresents a fundamental, guideline-defined therapeutic milestone. This constitutes a significant pharmacotherapeutic inaccuracy, as correct interpretation of molecular monitoring is central to CML management. This error warrants a score of 2 for Pharmacotherapeutic Accuracy and lowers the Guideline Alignment score.

Session ID:

Modeled Clinical Reasoning

Session-84:

The simulation excels at modeling an expert clinical reasoning process. Each stage of the case frames a clear, relevant clinical problem. The multiple-choice options demonstrate excellent breadth, incorporating key differentials and plausible distractors that effectively test for nuanced understanding (e.g., including risk scores from other malignancies). The overall strategic reasoning is sound, following a logical path of diagnosing, risk-stratifying, treating, and monitoring. The clinical outcomes are realistic and educationally valuable. Per the rubric, the content error noted in Section 2 does not lower the score in this section, as the underlying reasoning strategy (e.g., assessing response at 12 months) remains sound.

Session-85

Session ID:
LLM

Session-85:
Claude 3.7 Sonnet

Session ID:
Session Transcript

Session-85:
Phase 1: Initial Presentation and Diagnostic Workup

Case Introduction:
A 42-year-old male presents to his primary care physician with complaints of fatigue, unintentional weight loss of 10 pounds over the past 3 months, and early satiety. He also reports occasional night sweats. Physical examination reveals splenomegaly (4 cm below the left costal margin).

Initial Laboratory Results:
- WBC: 86,000/mm (4,500-11,000/mm)
- Hemoglobin: 11.2 g/dL (13.5-17.5 g/dL)
- Platelets: 450,000/mm (150,000-450,000/mm)
- Peripheral blood smear: Marked leukocytosis with left shift showing myelocytes, metamyelocytes, and occasional blasts (3%)

Question 1:
Based on the initial presentation, which of the following would be the most appropriate next step in the diagnostic workup?

A) Bone marrow aspiration and biopsy with cytogenetic analysis
B) CT scan of the abdomen and pelvis
C) Flow cytometry of peripheral blood
D) PET scan to rule out lymphom
A

Phase 1: Initial Presentation and Diagnostic Workup

Case Introduction:
A 42-year-old male presents to his primary care physician with complaints of fatigue, unintentional weight loss of 10 pounds over the past 3 months, and early satiety. He also reports occasional night sweats. Physical examination reveals splenomegaly (4 cm below the left costal margin).

Initial Laboratory Results:
- WBC: 86,000/mm (4,500-11,000/mm)
- Hemoglobin: 11.2 g/dL (13.5-17.5 g/dL)
- Platelets: 450,000/mm (150,000-450,000/mm)
- Peripheral blood smear: Marked leukocytosis with left shift showing myelocytes, metamyelocytes, and occasional blasts (3%)

Question 2:
The bone marrow biopsy shows hypercellular marrow (90%) with marked granulocytic hyperplasia and 4% blasts. Cytogenetic analysis reveals t(9;22)(q34;q11.2) in 20/20 metaphases. RT-PCR detects BCR-ABL1 transcripts (e13a2, b2a2). Based on these findings, what is the most accurate diagnosis according to the WHO 2022 classification?

A) Chronic myeloid leukemia (CML), chronic phase

B) Chronic myeloid leukemia (CML), accelerated phase
C) Ph+ acute lymphoblastic leukemia (ALL)
D) Atypical chronic myeloid leukemia, BCR-ABL1 negative
A

Phase 1: Initial Presentation and Diagnostic Workup

Case Introduction:
A 42-year-old male presents to his primary care physician with complaints of fatigue, unintentional weight loss of 10 pounds over the past 3 months, and early satiety. He also reports occasional night sweats. Physical examination reveals splenomegaly (4 cm below the left costal margin).

Initial Laboratory Results:
- WBC: 86,000/mm (4,500-11,000/mm)
- Hemoglobin: 11.2 g/dL (13.5-17.5 g/dL)
- Platelets: 450,000/mm (150,000-450,000/mm)
- Peripheral blood smear: Marked leukocytosis with left shift showing myelocytes, metamyelocytes, and occasional blasts (3%)
Question 3:
According to the European LeukemiaNet (ELN) 2022 criteria, which risk score should be calculated to determine the patient's prognosis?

A) IPSS score
B) Euro score
C) EUTOS Long-Term Survival (ELTS) score
D) Hasford score
B

HINT
According to the ELN 2022 guidelines, there are specific risk stratification systems recommended for CML patients. These have evolved over time from older systems like Sokal and Hasford to newer ones. The Euro score isn't one of the currently recommended risk assessment tools. Consider which scoring system is specifically highlighted in the ELN 2022 guidelines as the preferred prognostic system for CML patients.

C

Phase 1: Initial Presentation and Diagnostic Workup

Case Introduction:
A 42-year-old male presents to his primary care physician with complaints of fatigue, unintentional weight loss of 10 pounds over the past 3 months, and early satiety. He also reports occasional night sweats. Physical examination reveals splenomegaly (4 cm below the left costal margin).

Initial Laboratory Results:
- WBC: 86,000/mm (4,500-11,000/mm)
- Hemoglobin: 11.2 g/dL (13.5-17.5 g/dL)
- Platelets: 450,000/mm (150,000-450,000/mm)
- Peripheral blood smear: Marked leukocytosis with left shift showing myelocytes, metamyelocytes, and occasional blasts (3%)

Phase 2: Induction Therapy and Supportive Care

Additional Information:
The patient's ELTS score is calculated as 1.8, placing him in the intermediate-risk category. He has a medical history of well-controlled hypertension (on lisinopril 10mg daily) and hyperlipidemia (on atorvastatin 20mg daily). His ECG shows normal sinus

rhythm with QTc of 420ms. Liver and kidney function tests are within normal limits.

Question 4:
Based on the patient's diagnosis of chronic phase CML with intermediate-risk ELTS score, which of the following would be the most appropriate first-line treatment according to current ESMO guidelines?

A) Imatinib 400mg daily
B) Dasatinib 100mg daily
C) Nilotinib 300mg twice daily
D) Interferon-alpha with cytarabine
B

Hint
According to current ESMO guidelines for CML, several TKIs are approved for first-line treatment. Consider which option has the best risk-benefit profile taking into account this patient's specific characteristics. Remember that while second-generation TKIs might provide deeper responses, the primary goal for first-line therapy is balancing efficacy with long-term safety. Some TKIs have specific side effect profiles that may impact your choice.

A

Phase 1: Initial Presentation and Diagnostic Workup

Case Introduction:
A 42-year-old male presents to his primary care physician with complaints of fatigue, unintentional weight loss of 10 pounds over the past 3 months, and early satiety. He also reports occasional night sweats. Physical examination reveals splenomegaly (4 cm below the left costal margin).

Initial Laboratory Results:
- WBC: 86,000/mm (4,500-11,000/mm)
- Hemoglobin: 11.2 g/dL (13.5-17.5 g/dL)
- Platelets: 450,000/mm (150,000-450,000/mm)
- Peripheral blood smear: Marked leukocytosis with left shift showing myelocytes, metamyelocytes, and occasional blasts (3%)

Question 5:
Two weeks after starting therapy, the patient presents to the emergency department with fever (38.7C), night sweats, and malaise. Laboratory tests show:
- WBC: 126,000/mm (increased from baseline)
- Hemoglobin: 10.1 g/dL
- Platelets: 380,000/mm
- Uric acid: 9.2 mg/dL (3.4-7.0 mg/dL)
- LDH: 750 U/L (135-225 U/L)
- Potassium: 4.8 mEq/L (3.5-5.0 mEq/L)
- Calcium: 8.9 mg/dL (8.6-10.2 mg/dL)
- Phosphorus: 4.7 mg/dL (2.7-4.5 mg/dL)
- Creatinine: 1.1 mg/dL (0.7-1.3 mg/dL)

Which of the following is the most appropriate management strategy at this point?

A) Discontinue imatinib and switch to dasatinib
B) Continue imatinib, add allopurinol, and increase hydration
C) Continue imatinib and add hydroxyurea for cytoreduction
D) Continue imatinib and add prednisone
D

Hint
The patient is showing signs consistent with tumor lysis syndrome, as evidenced by elevated uric acid, LDH, and phosphorus levels. They also have leukocytosis that has worsened since starting therapy. Consider which medication is specifically used to prevent complications from increased uric acid levels, and which approach would best address the potential metabolic complications developing in this patient.

B

Phase 1: Initial Presentation and Diagnostic Workup

Case Introduction:
A 42-year-old male presents to his primary care physician with complaints of fatigue, unintentional weight loss of 10 pounds over the past 3 months, and early satiety. He also reports occasional night sweats. Physical examination reveals splenomegaly (4 cm below the left costal margin).

Initial Laboratory Results:
- WBC: 86,000/mm (4,500-11,000/mm)
- Hemoglobin: 11.2 g/dL (13.5-17.5 g/dL)
- Platelets: 450,000/mm (150,000-450,000/mm)
- Peripheral blood smear: Marked leukocytosis with left shift showing myelocytes, metamyelocytes, and occasional blasts (3%)

Question 6:
After 3 months of therapy with imatinib, the patient's BCR-ABL1 level (International Scale) is 25%. According to ELN 2022 criteria, how would you classify this response?

A) Optimal response
B) Warning
C) Failure
D) Complete cytogenetic response
A

Hint
According to ELN 2022 criteria for monitoring CML response, specific BCR-ABL1 transcript level milestones are expected at different time points during TKI therapy. For the 3-month milestone specifically, there are clear cutoffs that define optimal response versus warning or failure. Consider what the target BCR-ABL1 level (IS) should be at 3 months for a patient to be considered having an optimal response with first-line imatinib therapy.

B

Phase 1: Initial Presentation and Diagnostic Workup

Case Introduction:
A 42-year-old male presents to his primary care physician with complaints of fatigue, unintentional weight loss of 10 pounds over the past 3 months, and early satiety. He also reports occasional night sweats. Physical examination reveals splenomegaly (4 cm below the left costal margin).

Initial Laboratory Results:
- WBC: 86,000/mm (4,500-11,000/mm)
- Hemoglobin: 11.2 g/dL (13.5-17.5 g/dL)
- Platelets: 450,000/mm (150,000-450,000/mm)
- Peripheral blood smear: Marked leukocytosis with left shift showing myelocytes, metamyelocytes, and occasional blasts (3%)

Question 7:
Based on the suboptimal response at 3 months, which of the following would be the most appropriate next step in management?

A) Continue imatinib at the same dose and reassess at 6 months
B) Increase imatinib dose to 600mg daily
C) Switch to a second-generation TKI (dasatinib or nilotinib)
D) Perform mutation analysis of the BCR-ABL1 kinase domain
C

Hint
ELN 2022 guidelines provide specific recommendations for managing patients with a "warning" response at the 3-month mark. Consider whether immediate change of therapy is warranted at this point or if additional monitoring might be appropriate before making a treatment switch. The guidelines suggest a stepwise approach for patients who don't meet criteria for failure but show a warning response.

A

Phase 1: Initial Presentation and Diagnostic Workup

Case Introduction:
A 42-year-old male presents to his primary care physician with complaints of fatigue, unintentional weight loss of 10 pounds over the past 3 months, and early satiety. He also reports occasional night sweats. Physical examination reveals splenomegaly (4 cm below the left costal margin).

Initial Laboratory Results:
- WBC: 86,000/mm (4,500-11,000/mm)
- Hemoglobin: 11.2 g/dL (13.5-17.5 g/dL)
- Platelets: 450,000/mm (150,000-450,000/mm)
- Peripheral blood smear: Marked leukocytosis with left shift showing myelocytes, metamyelocytes, and occasional blasts (3%)

Phase 3: Post-remission Therapy and Long-term Management

Additional Information:
The patient continues imatinib therapy, and at the 6-month assessment, his BCR-ABL1 level (IS) is 8%. At 12 months, his BCR-ABL1 level is 1.2%.

Question 8:
According to ELN 2022 criteria, how would you classify the patient's response at 12 months?

A) Optimal response
B) Warning
C) Failure
D) Complete molecular response
A

Hint
According to ELN 2022 criteria, the milestones for optimal response at 12 months of TKI therapy have specific BCR-ABL1 (IS) targets. The optimal response at 12 months requires the BCR-ABL1 level to be below a certain threshold. Compare the patient's current BCR-ABL1 level of 1.2% with the ELN 2022 criteria to determine if this represents an optimal response or a warning.
C

Hint
According to the ELN 2022 criteria for monitoring response to TKIs in CML, at 12 months:
- Optimal response is defined as BCR-ABL1 0.1% (MR3)
- Warning is defined as BCR-ABL1 between >0.1% and 1%
- Failure is defined as BCR-ABL1 >1%

The patient's BCR-ABL1 level is 1.2%, which is just above the 1% threshold. Consider where this places the patient according to these criteria.
B

D

Hint
According to the ELN 2022 criteria for monitoring response to TKIs in CML at 12 months:

- Optimal response: BCR-ABL1 0.1% (MR3)
- Warning: BCR-ABL1 >0.1% and 1%
- Failure: BCR-ABL1 >1%

The patient's BCR-ABL1 level is 1.2%, which is >1%. This places the patient in which response category?

C

Phase 1: Initial Presentation and Diagnostic Workup

Case Introduction:
A 42-year-old male presents to his primary care physician with complaints of fatigue, unintentional weight loss of 10 pounds over the past 3 months, and early satiety. He also reports occasional night sweats. Physical examination reveals splenomegaly (4 cm below the left costal margin).

Initial Laboratory Results:
- WBC: 86,000/mm (4,500-11,000/mm)
- Hemoglobin: 11.2 g/dL (13.5-17.5 g/dL)
- Platelets: 450,000/mm (150,000-450,000/mm)
- Peripheral blood smear: Marked leukocytosis with left shift showing myelocytes, metamyelocytes, and occasional blasts (3%)

Question 9:
Based on the patient's response at 12 months, which of the following is the most appropriate next step in management?

A) Continue imatinib at the same dose
B) Increase imatinib dose to 800mg daily
C) Switch to a second-generation TKI (dasatinib or nilotinib)
D) Consider allogeneic stem cell transplantation
B

Hint
According to ELN 2022 guidelines, when a patient meets the criteria for "failure" at the 12-month mark (BCR-ABL1 >1%), there are specific recommendations for how to adjust therapy. Consider whether continuing the same therapy with dose adjustment would be sufficient or if a change to a more potent inhibitor is warranted in this situation. The guidelines recommend specific approaches when first-line therapy fails to achieve target milestones.
C

Phase 1: Initial Presentation and Diagnostic Workup

Case Introduction:
A 42-year-old male presents to his primary care physician with complaints of fatigue, unintentional weight loss of 10 pounds over the past 3 months, and early satiety. He also reports occasional night sweats. Physical examination reveals splenomegaly (4 cm below the left costal margin).

Initial Laboratory Results:
- WBC: 86,000/mm (4,500-11,000/mm)
- Hemoglobin: 11.2 g/dL (13.5-17.5 g/dL)
- Platelets: 450,000/mm (150,000-450,000/mm)
- Peripheral blood smear: Marked leukocytosis with left shift showing myelocytes, metamyelocytes, and occasional blasts (3%)

Question 10:
The patient is switched to nilotinib 300mg twice daily. After 6 months on nilotinib (18 months from initial diagnosis), his BCR-ABL1 level is 0.05% (IS). Which of the following statements is correct regarding this patient's long-term management?

A) The patient should be considered for treatment-free remission (TFR) immediately
B) The patient should continue nilotinib indefinitely
C) The patient should continue nilotinib for at least 3 years of sustained deep molecular response before considering TFR
D) The patient should be referred for allogeneic stem cell transplantation
A

Hint
According to current guidelines for treatment-free remission (TFR) in CML, specific criteria must be met before attempting TFR. These include both the depth of molecular response and the duration of that response. Consider how long a patient typically needs to maintain a deep molecular response (MR4 or better) before TFR can be safely attempted, as well as the total minimum duration of TKI therapy recommended by ESMO and ELN guidelines.
C

Session ID:
Assessment Domain

Session-85:
Rationale

Session ID:
Instructional Design and Pedagogical

Session-85:
The session's instructional design is of very high quality. The case is expertly scaffolded, guiding the learner through a realistic patient journey from initial diagnosis and risk stratification (Questions 1-3) to first-line therapy selection (Question 4), management of an acute complication (Question 5), and navigating the complexities of response monitoring, treatment failure, and long-term management (Questions 6-10). The embedded feedback, particularly in the multi-step hints for challenging questions like Q8, is insightful and effectively reinforces guideline-based milestones without giving away the answer. Instructions and case framing are exceptionally clear. The only minor weakness is the absence of explicitly stated learning objectives at the outset, though the educational goals are strongly implied by the case's logical progression.

Session ID:
Clinical Authenticity and Content Fidelity

Session-85:
The session demonstrates outstanding clinical fidelity and authenticity. Plausibility & Guideline Alignment: The clinical narrative, from initial presentation to the specific BCR-ABL transcript levels at various time points, is indistinguishable from an authentic Chronic Myeloid Leukemia (CML) case. All therapeutic decisions and monitoring milestones are perfectly aligned with current, evidence-based guidelines (ELN/ESMO), such as using the ELTS score for prognostication (Q3), identifying treatment failure at 12 months with a BCR-ABL >1% (Q8), and understanding the criteria for considering treatment-free remission (Q10). Pharmacotherapeutic Accuracy: All pharmacotherapeutic choices and rationales are optimal and well-justified. The initial selection of imatinib, management of tumor lysis syndrome with allopurinol, and the switch to a second-generation TKI upon treatment failure are all standard-of-care. Domain

Specificity: The case remains strictly within the domain of CML. There is no evidence of domain entanglement; all diagnostics, therapies, and standards of care discussed are appropriate and specific to CML management.

Session ID:

Modeled Clinical Reasoning

Session-85:

The simulation flawlessly models an expert clinical reasoning process for CML management. Each stage of the case presents a well-defined clinical problem with a complete dataset for decision-making. The multiple-choice options provide an excellent range of plausible alternatives, including relevant distractors that test nuanced understanding (e.g., distinguishing "warning" from "failure" at different time points). The modeled decision-making demonstrates sound prioritization, such as securing definitive diagnostics before therapy, monitoring response at critical intervals, and escalating care appropriately in response to treatment failure. The clinical outcomes are realistic and highly instructive, reflecting a common and challenging patient trajectory that reinforces key learning objectives.

Session-86

Session ID:
LLM

Session-86:
Claude 3.7 Sonnet

Session ID:
Session Transcript

Session-86:
Mr. James Wilson, a 42-year-old software engineer, presents to his primary care physician with complaints of fatigue, night sweats, and unintentional weight loss of 15 pounds over the past 3 months. He also mentions feeling full after eating small amounts of food. His past medical history is significant only for well-controlled hypertension treated with lisinopril 10 mg daily. He has no known drug allergies.
Physical examination reveals:

Vital signs: Temperature 37.2C, HR 86 bpm, BP 132/84 mmHg, RR 16/min
General: Mild pallor
Abdominal: Splenomegaly extending 4 cm below the left costal margin
No lymphadenopathy

Initial laboratory results:

CBC: WBC 112 10^9/L (elevated), Hgb 11.2 g/dL (decreased), Platelets 398 10^9/L
Differential: Neutrophils 54%, Lymphocytes 8%, Monocytes 3%, Eosinophils 4%, Basophils 8%, Blasts 2%, Promyelocytes 4%, Myelocytes 12%, Metamyelocytes 5%
CMP: All values within normal limits except LDH 420 U/L (elevated)

Question 1:
Based on the patient's presentation and initial laboratory findings, what is the most appropriate next step in the diagnostic workup?
A) Order bone marrow aspiration and biopsy with cytogenetic analysis
B) Order a CT scan of the chest, abdomen, and pelvis
C) Start empiric antibiotic therapy and monitor for response
D) Order peripheral blood flow cytometry only
E) Refer directly to surgical oncology for splenectomy evaluation
Correct! Bone marrow aspiration and biopsy with cytogenetic analysis is the most appropriate next step in the diagnostic workup.
Why this is correct:
A bone marrow evaluation is essential for the diagnosis of CML, as it allows for:

Morphologic assessment of blast percentage and cellular distribution
Cytogenetic analysis to detect the Philadelphia chromosome t(9;22)
FISH and/or PCR testing for BCR-ABL1 fusion gene
Assessment of any additional chromosomal abnormalities

Why other options are incorrect:
B) CT scan would only confirm splenomegaly without providing diagnostic confirmation
C) Antibiotics are not indicated as the presentation is consistent with a hematologic malignancy, not infection
D) Peripheral blood flow cytometry alone is insufficient for CML diagnosis
E) Splenectomy is not indicated in the diagnostic workup of suspected CML

The bone marrow biopsy and cytogenetic analysis were performed with the following results:

Bone marrow is hypercellular (95%) with myeloid hyperplasia
Blast count is 3%
Cytogenetic analysis: 46,XY,t(9;22)(q34;q11.2) in 20/20 metaphases
FISH analysis: BCR-ABL1 fusion detected in 98% of analyzed cells
Molecular testing: BCR-ABL1/ABL1 ratio of 52% (IS)
No additional chromosomal abnormalities detected
Based on these findings, what is the most appropriate diagnosis for Mr. Wilson?
A) Chronic myeloid leukemia, chronic phase
B) Chronic myeloid leukemia, accelerated phase
C) Chronic myelomonocytic leukemia
D) Essential thrombocythemia
E) Primary myelofibrosis
This answer is incorrect. The correct answer is A) Chronic myeloid leukemia, chronic phase.
Why A is correct:
The patient has CML in chronic phase based on:

Presence of t(9;22) Philadelphia chromosome and BCR-ABL1 fusion gene
Blast percentage of 3% in bone marrow (below 10%)
No evidence of criteria for accelerated phase or blast phase

Why B is incorrect:
For CML to be in accelerated phase, according to the 2022 WHO classification, at least one of these criteria would need to be present:

Blasts 10-19% in peripheral blood or bone marrow
Basophils 20% in peripheral blood
Persistent thrombocytopenia (<100 10^9/L) unrelated to therapy
Persistent thrombocytosis (>1000 10^9/L) unresponsive to therapy
Increasing spleen size unresponsive to therapy
Cytogenetic evidence of clonal evolution (additional chromosomal abnormalities)

Mr. Wilson's findings do not meet any of these criteria.

Let's calculate the Sokal risk score for this patient:

Age: 42 years
Spleen size: 4 cm below costal margin
Platelet count: 398 10^9/L
Blast percentage: 2% in peripheral blood

Sokal score calculation results in low risk (score <0.8).
What is the most appropriate initial treatment for Mr. Wilson?
A) Imatinib 400 mg daily
B) Dasatinib 100 mg daily
C) Nilotinib 300 mg twice daily
D) Hydroxyurea followed by allogeneic stem cell transplantation
E) Interferon-alpha plus cytarabine
Correct! Imatinib 400 mg daily is the most appropriate initial treatment for Mr. Wilson.
Why this is correct:

Imatinib 400 mg daily is a standard first-line treatment for newly diagnosed chronic phase CML
The patient has a low Sokal risk score, which is associated with good outcomes with standard-dose imatinib
Imatinib has a well-established long-term safety profile with over 20 years of clinical experience

The patient has no comorbidities that would contraindicate imatinib or specifically favor an alternative TKI

Why other options are incorrect:
B & C) While dasatinib and nilotinib are approved first-line options and may achieve faster and deeper responses, they are typically considered for patients with higher risk scores or specific indications
D) Allogeneic stem cell transplantation is not indicated as first-line therapy for chronic phase CML in the TKI era
E) Interferon-alpha plus cytarabine is an outdated approach that has been replaced by TKI therapy
Based on the 3-month response assessment, how would you classify Mr. Wilson's response to therapy according to ELN 2022 criteria?
A) Optimal response
B) Warning
C) Failure
D) Complete cytogenetic response
E) Major molecular response
Why A is incorrect:
An optimal response at 3 months would require BCR-ABL1 10% (IS), which Mr. Wilson has not achieved.
Why other options are incorrect:
C) Failure would be BCR-ABL1 >30% (IS)
D) Complete cytogenetic response requires 0% Ph+ metaphases
E) Major molecular response is defined as BCR-ABL1 0.1% (IS)

Given this warning response, you decide to continue imatinib with close monitoring. At the 6-month assessment:

BCR-ABL1/ABL1 ratio: 5.8% (IS)
Cytogenetics: Ph+ in 2/20 metaphases
Question 5:
Based on the 6-month response assessment, what is the most appropriate next step in management?
A) Continue imatinib 400 mg daily with regular monitoring
B) Increase imatinib to 600 mg daily
C) Switch to nilotinib 300 mg twice daily
D) Switch to dasatinib 100 mg daily
E) Perform mutational analysis of the BCR-ABL1 kinase domain
Correct! Continue imatinib 400 mg daily with regular monitoring is the most appropriate next step in management.
Why this is correct:

According to ELN 2022 criteria, BCR-ABL1 10% (IS) at 6 months is considered an optimal response
Mr. Wilson's BCR-ABL1 level has improved from 18% to 5.8% (IS)
He has achieved a partial cytogenetic response (PCyR) with only 2/20 Ph+ metaphases (10%)
This shows he is responding appropriately to standard-dose imatinib therapy

Why other options are incorrect:
B) Dose escalation of imatinib is not indicated in patients with optimal response
C & D) Switching to a second-generation TKI is not necessary given the optimal response
E) Mutational analysis is indicated for treatment failure or suboptimal response, not for optimal response

At the 12-month assessment:

BCR-ABL1/ABL1 ratio: 0.9% (IS)
Cytogenetics: No Ph+ metaphases detected (0/20)

Mr. Wilson mentions that he began experiencing increasing fatigue two weeks ago and recently developed a sore throat and low-grade fever. His current labs show:

WBC 1.8 10^9/L, with ANC 0.6 10^9/L

Hgb 10.2 g/dL, Platelets 72 10^9/L
Question 6:
What is the most appropriate next step in managing this patient?
A) Continue imatinib 400 mg daily with reassessment in 1 month
B) Temporarily hold imatinib and initiate G-CSF therapy
C) Permanently discontinue imatinib and switch to nilotinib
D) Temporarily hold imatinib, obtain blood cultures, and start empiric antibiotics
E) Temporarily hold imatinib and taper dose upon resumptionRetryClaude can make mistakes. Please double-check responses.
Incorrect. The correct answer is D) Temporarily hold imatinib, obtain blood cultures, and start empiric antibiotics.
Why D is correct:

The patient has developed neutropenic fever (ANC 0.6 10^9/L with fever and sore throat)
This represents a serious, potentially life-threatening complication requiring immediate intervention
Standard management of neutropenic fever includes:

Immediate cessation of myelosuppressive therapy (imatinib)
Blood cultures and other infectious workup
Prompt initiation of empiric broad-spectrum antibiotics
Close monitoring in an appropriate setting

Why E is incorrect:
While temporarily holding imatinib is part of the correct approach, this answer lacks the critical component of addressing the neutropenic fever with appropriate anti-infective measures.
Why other options are incorrect:
A) Continuing imatinib during neutropenic fever could worsen cytopenia and endanger the patient
B) G-CSF alone without addressing potential infection is insufficient
C) Permanent discontinuation is not necessary; many patients can resume TKI after resolution of cytopenia

After appropriate management, Mr. Wilson's neutropenic fever resolves. Imatinib is restarted at 300 mg daily after his ANC recovers to >1.0 10^9/L. His CBC stabilizes with mild cytopenia (ANC ~1.5 10^9/L, platelets ~100 10^9/L).
Question 7:
At the 18-month assessment, Mr. Wilson's BCR-ABL1/ABL1 ratio is 0.08% (IS). This response is classified as:
A) Complete hematologic response (CHR)
B) Complete cytogenetic response (CCyR)
C) Early molecular response (EMR)
D) Major molecular response (MMR)
E) Deep molecular response (DMR/MR4.5)RetryClaude can make mistakes. Please double-check responses.
Correct! Major molecular response (MMR) is the appropriate classification for Mr. Wilson's current response status.
Why this is correct:

Major molecular response (MMR) is defined as BCR-ABL1 0.1% (IS) on the International Scale
Mr. Wilson's BCR-ABL1/ABL1 ratio is 0.08% (IS), which meets this criterion
MMR represents a 3-log reduction from the standardized baseline value
This is an important milestone in CML treatment as it's associated with excellent long-term outcomes

Why other options are incorrect:
A) Complete hematologic response refers to normalization of blood counts and spleen size
B) Complete cytogenetic response means 0% Ph+ metaphases (which he has achieved, but this answer characterizes his molecular status)
C) Early molecular response refers to BCR-ABL1 10% at 3 months
E) Deep molecular response (MR4.5) would require BCR-ABL1 0.0032% (IS)

Phase 3: Post-Remission Therapy and Long-Term Management
Mr. Wilson continues on imatinib 300 mg daily. His CBC has stabilized with mild persistent cytopenia. At the 24-month assessment:

BCR-ABL1/ABL1 ratio: 0.005% (IS)
He has maintained this level for the past 6 months

Mr. Wilson asks about the possibility of stopping treatment, as he has read about treatment-free remission (TFR) in CML.
Question 8:
Which of the following is the most accurate statement regarding treatment-free remission (TFR) in this patient?
A) TFR should not be attempted in patients who experienced significant toxicity on TKI therapy
B) Mr. Wilson meets all criteria for a TFR attempt based on his current status
C) TFR requires at least 5 years of TKI therapy and sustained DMR for 2 years
D) Monthly BCR-ABL1 monitoring is recommended during the first 6 months after TKI discontinuation
E) Loss of MMR is not an indication to restart TKI therapy during a TFR attemptRetryClaude can make mistakes. Please double-check responses.
Incorrect. The correct answer is D) Monthly BCR-ABL1 monitoring is recommended during the first 6 months after TKI discontinuation.
Why D is correct:

When attempting TFR, frequent molecular monitoring is essential for early detection of molecular recurrence
The standardized monitoring schedule in most TFR trials and guidelines includes:

Monthly BCR-ABL1 PCR testing for the first 6 months
Every 2 months during months 7-12
Every 3 months thereafter

Why C is incorrect:
While sustained deep molecular response is required for TFR, the exact duration requirements vary by guideline. Current ESMO and NCCN guidelines generally recommend:

At least 3-5 years (not strictly 5 years) of TKI therapy
Sustained DMR for at least 2 years
The ability to perform regular, reliable PCR monitoring

Why other options are incorrect:
A) Prior toxicity does not preclude TFR attempts and may actually be an indication for attempting TFR
B) Based on the limited information provided, we cannot confirm Mr. Wilson meets all criteria for TFR (e.g., duration of TKI therapy)
E) Loss of MMR is the standard trigger to restart TKI therapy during TFR

After discussing the risks and benefits, Mr. Wilson decides to continue treatment for now. At his 3-year assessment, he reports worsening fatigue and night sweats. His labs show:

WBC 24 10^9/L with 12% blasts in peripheral blood
Platelets 62 10^9/L
Bone marrow: 14% blasts
BCR-ABL1/ABL1 ratio: 15% (IS)
Cytogenetics: t(9;22) plus an additional abnormality of isochromosome 17q
Question 9:
What is the most likely cause of Mr. Wilson's disease progression?
A) Natural progression despite adequate therapy
B) Patient non-adherence to imatinib therapy
C) Development of a BCR-ABL1 kinase domain mutation

D) Clonal evolution with loss of dependence on BCR-ABL1
E) Inadequate dosing of imatinib at 300 mg daily
Incorrect. The correct answer is C) Development of a BCR-ABL1 kinase domain mutation.
Why C is correct:

Loss of previously achieved response (from MMR to BCR-ABL1 15%) strongly suggests acquired resistance
The most common mechanism of acquired resistance to TKIs is the development of mutations in the BCR-ABL1 kinase domain
These mutations can affect TKI binding and reduce drug efficacy
The presence of additional cytogenetic abnormalities (isochromosome 17q) is also consistent with clonal evolution, which often occurs alongside kinase domain mutations

Why B is incorrect:
While non-adherence is a common cause of suboptimal response, the clinical scenario (new cytogenetic abnormality, progressive disease with increasing blast percentage) is more consistent with true biological resistance rather than non-adherence.
Why other options are incorrect:
A) "Natural progression despite adequate therapy" is not a recognized phenomenon in well-responding CML
D) Complete loss of BCR-ABL1 dependence is rare and typically occurs in very advanced disease
E) While the reduced dose could contribute, the development of a specific cytogenetic abnormality suggests a biological mechanism of resistance

BCR-ABL1 kinase domain mutation analysis is performed, revealing a T315I mutation.
Question 10:
What is the most appropriate next step in management for Mr. Wilson?
A) Switch to high-dose dasatinib 140 mg daily
B) Switch to ponatinib 45 mg daily with appropriate cardiovascular risk management
C) Initiate combination therapy with a TKI plus venetoclax
D) Enroll in a clinical trial of asciminib if available
E) Proceed directly to allogeneic stem cell transplantation
Correct! Ponatinib 45 mg daily with appropriate cardiovascular risk management is the most appropriate next step in management.
Why this is correct:

The T315I mutation confers resistance to most TKIs including imatinib, nilotinib, dasatinib, and bosutinib
Ponatinib is the only approved TKI that is effective against the T315I mutation
The patient has progressed to blast phase CML, requiring aggressive therapy
Cardiovascular risk assessment and management is essential with ponatinib due to its associated risk of arterial occlusive events

Why other options are incorrect:
A) Dasatinib is ineffective against the T315I mutation
C) While combination approaches are being studied, they are not standard of care for T315I-mutated CML
D) While asciminib has activity against some mutations, its efficacy against T315I in blast phase is not well-established as monotherapy
E) While allogeneic stem cell transplantation could be considered, attempting to achieve disease control with ponatinib first is the standard approach

Session Summary:
Thank you for completing this CML Clinical Case Management session. Here's a summary of the key learning points:

Session ID:
Assessment Domain

Session-86:
Rationale

Session ID:

Instructional Design and Pedagogical

Session-86:

The session's instructional design is of exceptionally high quality. The case is expertly scaffolded, guiding the learner through the complete, longitudinal management of a CML patientfrom initial diagnosis and risk stratification to first-line therapy, response monitoring, toxicity management, and finally, handling advanced disease with acquired resistance. The progression is logical and seamlessly builds in complexity. The embedded feedback is a major strength, providing clear, guideline-anchored rationales (e.g., ELN criteria) that explain not only why an answer is correct but also why the distractors are incorrect. The only minor weakness is that learning objectives are not explicitly stated at the outset, though they are clearly implied by the case's comprehensive structure.

Session ID:

Clinical Authenticity and Content Fidelity

Session-86:

The clinical fidelity of this session is outstanding. The narrative is indistinguishable from an authentic patient case, with a plausible presentation, realistic laboratory values, and a typical disease course, including common management challenges like myelosuppression and eventual resistance. All diagnostic and therapeutic recommendations are perfectly aligned with current, evidence-based guidelines (e.g., ELN, NCCN). Pharmacotherapeutic choicesfrom selecting imatinib for a low-risk patient to correctly identifying ponatinib as the sole option for T315I-mutated diseaseare optimal and well-justified. Crucially, the session demonstrates perfect domain specificity, with all concepts and therapies remaining strictly within the context of CML management. No clinically significant errors or domain entanglement were identified.

Session ID:

Modeled Clinical Reasoning

Session-86:

The simulation expertly models the clinical reasoning process of a hematology specialist. Each stage presents a distinct, well-framed clinical problem (e.g., initial diagnosis, suboptimal response, neutropenic fever, relapse) supported by the necessary data. The multiple-choice options are thoughtfully constructed, including a range of relevant and plausible distractors that effectively probe the learner's nuanced understanding. The modeled decision-making is flawless, demonstrating appropriate prioritization, such as addressing the life-threatening neutropenic fever before considering long-term CML therapy changes. The clinical outcomes described are realistic, specific, and highly instructive, effectively reinforcing the consequences of each therapeutic decision.

Session-87

Session ID:
LLM

Session-87:
ChatGPT (GPT 4.o)

Session ID:
Session Transcript

Session-87:
Case Introduction:
A 52-year-old male presents to his primary care physician with complaints of fatigue, unintentional weight loss (10 pounds over 3 months), night sweats, and left upper quadrant discomfort. The patient reports that his symptoms have been gradually worsening over the past few months. He has no significant past medical history except for well-controlled hypertension managed with lisinopril 10 mg daily. The patient works as an industrial painter and has a 15 pack-year smoking history, though he quit 5 years ago.
Physical Examination:

Vital signs: Temperature 99.1F, BP 135/85 mmHg, HR 88 bpm, RR 16/min
General: Fatigued-appearing but alert and oriented
Cardiovascular: Regular rate and rhythm, no murmurs
Respiratory: Clear lung fields bilaterally
Abdomen: Moderate splenomegaly (4 cm below costal margin), mild tenderness in left upper quadrant, no hepatomegaly
Lymph nodes: No palpable lymphadenopathy

Laboratory Results:

WBC: 112,000/L (normal: 4,500-11,000/L)
Differential: 60% neutrophils, 10% lymphocytes, 5% monocytes, 8% eosinophils, 7% basophils, 10% immature myeloid cells
Hemoglobin: 10.1 g/dL (normal: 13.5-17.5 g/dL)
Platelets: 450,000/L (normal: 150,000-450,000/L)
Peripheral blood smear: Left-shifted myeloid series with all stages of maturation present
LDH: 780 U/L (normal: 140-280 U/L)
Uric acid: 8.2 mg/dL (normal: 3.4-7.0 mg/dL)
Other chemistry values within normal limits

Question 1:
Based on the clinical presentation and laboratory findings, what would be the most appropriate next diagnostic step?
A. Bone marrow biopsy with cytogenetic analysis
B. Abdominal CT scan to evaluate splenomegaly
C. Peripheral blood flow cytometry
D. Molecular testing for BCR-ABL1 by PCR from peripheral blood
E. Lymph node biopsy
answer: D. Molecular testing for BCR-ABL1 by PCR from peripheral blood

Edit
Correct! Molecular testing for BCR-ABL1 by PCR from peripheral blood is the most appropriate next diagnostic step.

Rationale:

The patient presents with classic signs and symptoms consistent with chronic myeloid leukemia (CML): fatigue, weight loss, night sweats, and splenomegaly

The marked leukocytosis (WBC 112,000/L) with a left-shifted myeloid series and presence of all maturation stages is highly suggestive of CML
Elevated basophils and eosinophils are characteristic findings in CML
The presence of BCR-ABL1 fusion gene is the hallmark genetic abnormality in CML, and detecting this by PCR from peripheral blood is a rapid, less invasive initial test compared to bone marrow biopsy
ESMO guidelines recommend molecular testing for BCR-ABL1 as an essential diagnostic test for suspected CML
While bone marrow biopsy with cytogenetic analysis (option A) will eventually be needed for complete diagnosis and staging, molecular testing from peripheral blood is typically performed first due to its rapid turnaround time and non-invasive nature.

Follow-up Results:

The PCR test detects the presence of BCR-ABL1 p210 fusion transcript, confirming your suspicion of CML. A bone marrow biopsy is subsequently performed and shows hypercellularity (95%) with myeloid hyperplasia. Conventional cytogenetics reveals t(9;22)(q34;q11.2) - the Philadelphia chromosome - in 20 out of 20 metaphases analyzed.

Question 2:

Based on these findings, what is the most likely phase of CML in this patient?
A. Chronic phase
B. Accelerated phase
C. Blast phase
D. Pre-leukemic phase
E. Remission phase
answer: A. Chronic phase

Edit
Correct! The patient is most likely in the chronic phase of CML.

Rationale:

The patient's clinical presentation and laboratory findings are consistent with chronic phase CML
Key findings supporting chronic phase:
Blasts are not increased in peripheral blood (<10%)
No evidence of additional chromosomal abnormalities
Absence of features suggestive of accelerated or blast phase such as:
Persistent thrombocytopenia
Increasing spleen size unresponsive to therapy
20% basophils in peripheral blood
20% blasts in peripheral blood or bone marrow
According to the WHO classification and ELN 2022 criteria, chronic phase CML is characterized by:

<10% blasts in peripheral blood and bone marrow
Presence of BCR-ABL1 fusion gene or t(9;22) Philadelphia chromosome
Absence of criteria for accelerated or blast phase
Follow-up Information:
Additional testing reveals:

BCR-ABL1/ABL1 (IS): 85%
No additional chromosomal abnormalities detected
FISH analysis confirms BCR-ABL1 fusion in 98% of cells
The patient's Sokal score is calculated as 1.2, and EUTOS long-term survival score (ELTS) is determined to be intermediate risk.
Follow-up Information:
Additional testing reveals:

BCR-ABL1/ABL1 (IS): 85%
No additional chromosomal abnormalities detected
FISH analysis confirms BCR-ABL1 fusion in 98% of cells

The patient's Sokal score is calculated as 1.2, and EUTOS long-term survival score (ELTS) is determined to be intermediate risk.

Question 3:

What is the most appropriate next step in management for this patient?
A. Start hydroxyurea for cytoreduction, then initiate imatinib 400 mg daily
B. Start dasatinib 100 mg daily
C. Start imatinib 400 mg daily
D. Start nilotinib 300 mg twice daily
E. Refer for evaluation for allogeneic stem cell transplantation
hint

Edit
Here's a hint to help you with this question:

Consider the ESMO and ELN guidelines for first-line treatment of chronic phase CML. Think about:

What is the standard first-line treatment approach for newly diagnosed chronic phase CML?
Is there a need for cytoreduction before initiating targeted therapy in this case?
This patient has an intermediate risk score (Sokal 1.2, ELTS intermediate) - does this influence the choice of initial TKI therapy?
Which treatment option would provide the best balance of efficacy, tolerability, and adherence for this newly diagnosed patient?
Remember that current guidelines recommend TKI therapy as the standard first-line approach, but the specific agent may depend on several factors including risk status, comorbidities, and treatment goals.

answer: C. Start imatinib 400 mg daily

Edit
Correct! Starting imatinib 400 mg daily is the most appropriate next step in management for this patient.

Rationale:

Imatinib 400 mg daily is the standard first-line treatment for newly diagnosed chronic phase CML according to ESMO and ELN guidelines
For patients with intermediate risk (Sokal score 1.2, ELTS intermediate), imatinib is an appropriate first-line choice
The patient has no comorbidities that would contraindicate imatinib use
While second-generation TKIs (dasatinib, nilotinib) might achieve faster and deeper molecular responses, they are associated with more adverse effects and not necessarily better long-term outcomes for all patients
Cytoreduction with hydroxyurea before starting imatinib (option A) is typically only necessary for patients with extreme leukocytosis (generally >250,000/L) or severe symptoms
Allogeneic stem cell transplantation is not indicated as first-line therapy for chronic phase CML in the TKI era
According to the ESMO guidelines, imatinib remains a standard first-line option with excellent long-term outcomes, good tolerability, and extensive safety data.
Phase 2: Induction Therapy and Supportive Care
The patient is started on imatinib 400 mg daily. Three months after initiating therapy, the following results are obtained:

Complete blood count (CBC): WBC 5,200/L with normal differential, hemoglobin 13.2 g/dL, platelets 250,000/L
Physical exam: Spleen no longer palpable
Quantitative PCR: BCR-ABL1/ABL1 (IS): 8.5%

Question 4:
How would you assess this patient's response to therapy at the 3-month mark according to the ELN criteria?
A. Optimal response
B. Warning (previously called "suboptimal response")
C. Failure
D. Complete molecular response
E. Major molecular response
hint

Edit
Here's a hint to help you assess this patient's response:

According to the ELN 2022 criteria for evaluating response to TKI therapy in CML, there are specific BCR-ABL1 transcript level thresholds that define optimal response, warning, and failure at the 3-month timepoint.

Consider:

What BCR-ABL1 (IS) percentage constitutes an optimal response at 3 months?
What BCR-ABL1 (IS) percentage constitutes a warning at 3 months?
What BCR-ABL1 (IS) percentage constitutes a failure at 3 months?
The patient's current BCR-ABL1/ABL1 (IS) is 8.5% at 3 months. Compare this value with the ELN thresholds to determine the appropriate response category.

answer: A. Optimal response

Edit
Correct! The patient's response at the 3-month mark is considered an optimal response according to the ELN criteria.

Rationale:

According to the 2022 ELN recommendations, an optimal response at 3 months is defined as BCR-ABL1 transcript level 10% (IS)
The patient's BCR-ABL1 level is 8.5%, which falls below the 10% threshold
Additionally, the patient shows:
Normalization of blood counts (normal WBC, hemoglobin, and platelets)
Resolution of splenomegaly on physical examination
These clinical findings further support an optimal response
The ELN response criteria at 3 months:

Optimal response: BCR-ABL1 10% (IS)
Warning: BCR-ABL1 >10% (IS)
Failure: BCR-ABL1 >10% (IS) with additional concerning features or evidence of disease progression
Follow-up:
The patient continues on imatinib 400 mg daily. At the 6-month follow-up, his BCR-ABL1/ABL1 (IS) is 1.5%. However, the patient reports developing severe periorbital edema, muscle cramps, and intermittent diarrhea that are affecting his quality of life.

Question 5:
What is the most appropriate management strategy for this patient's TKI-related adverse effects?
A. Discontinue imatinib and switch to dasatinib
B. Reduce imatinib dose to 300 mg daily
C. Continue imatinib 400 mg daily and add supportive care measures for adverse effects
D. Discontinue imatinib and switch to nilotinib

E. Hold imatinib until adverse effects resolve, then resume at 300 mg daily
hint

Edit
Here's a hint to help you determine the most appropriate management strategy:

Consider the balance between maintaining effective treatment and managing adverse effects. Think about:

Are these adverse effects considered severe/life-threatening or manageable?
What is the current response status of the patient (optimal, warning, or failure)?
What approaches are recommended by guidelines for managing common imatinib-related adverse effects?
Would dose reduction potentially compromise the optimal response the patient has achieved?
Are there supportive care measures that could effectively address these specific side effects while maintaining the therapeutic dose?
Remember that the goal is to maintain treatment efficacy while improving tolerability and quality of life for the patient.

answer: C. Continue imatinib 400 mg daily and add supportive care measures for adverse effects

Edit
Correct! Continuing imatinib 400 mg daily and adding supportive care measures is the most appropriate management strategy for this patient's TKI-related adverse effects.

Rationale:

The patient is demonstrating an optimal response to therapy (BCR-ABL1 at 1.5% at 6 months, which is below the 5% threshold for optimal response)
The reported adverse effects (periorbital edema, muscle cramps, diarrhea) are common with imatinib but are generally manageable with supportive care
For periorbital edema: sodium restriction, topical steroids, and/or diuretics can be helpful
For muscle cramps: calcium and magnesium supplements, tonic water (quinine), and/or physical therapy
For diarrhea: loperamide, dietary modifications, and ensuring adequate hydration
Maintaining the full dose is important to preserve the optimal response and achieve deeper molecular responses over time
Switching TKIs (options A and D) when achieving optimal response is not recommended unless adverse effects are severe or intolerable despite supportive care
Dose reduction (option B) or temporary discontinuation (option E) may compromise efficacy and is generally reserved for more severe adverse effects
ESMO and ELN guidelines emphasize the importance of managing adverse effects with supportive care rather than dose reduction or switching when possible, particularly in patients achieving optimal responses.
Follow-up:
With implementation of supportive care measures (sodium restriction, topical steroids for periorbital edema, calcium supplements for muscle cramps, and loperamide as needed for diarrhea), the patient reports significant improvement in symptoms. He continues on imatinib 400 mg daily.
At the 12-month follow-up:

CBC remains normal
Physical exam is unremarkable
BCR-ABL1/ABL1 (IS): 0.3%

Question 6:
According to ELN criteria, what is the current response status of this patient at 12 months?
A. Complete hematologic response (CHR)
B. Major molecular response (MMR)
C. Complete cytogenetic response (CCyR)
D. Early molecular response (EMR)
E. Complete molecular response (CMR)

answer: C. Complete cytogenetic response (CCyR)

Edit

Your answer is incorrect. Let me explain the correct response.

Correct Answer: B. Major molecular response (MMR)

Rationale:

Major molecular response (MMR) is defined as BCR-ABL1 0.1% on the International Scale (IS), which corresponds to a 3-log reduction from the standardized baseline

While the patient's BCR-ABL1 level of 0.3% doesn't quite reach the threshold for MMR, this is a trick question. In fact, the patient's BCR-ABL1 level of 0.3% indicates a molecular response between CCyR and MMR

Complete cytogenetic response (CCyR) is defined as the absence of Ph+ metaphases in bone marrow cytogenetics, which correlates with BCR-ABL1 levels of approximately 1%

Since the patient's BCR-ABL1 level is 0.3%, which is below 1%, we can infer that the patient has achieved CCyR

However, we need to strictly interpret the ELN criteria based on the molecular testing results, not inferred cytogenetic status

According to the ELN criteria at 12 months:

Optimal response: BCR-ABL1 0.1% (IS), which is MMR

Warning: BCR-ABL1 >0.1-1% (IS)

Failure: BCR-ABL1 >1% (IS)

Since the patient's BCR-ABL1 level is 0.3%, this falls into the "warning" category at 12 months, but the term "warning" was not an option in the question.

Follow-up:

The patient continues on imatinib 400 mg daily. At 18 months, his BCR-ABL1/ABL1 (IS) is 0.08%, indicating he has now achieved MMR.

Question 7:

The patient developed fatigue, dyspnea, and tachycardia during the last month. Laboratory results show:

Hemoglobin: 8.2 g/dL (decreased from 13.5 g/dL)

WBC: 4,800/L with normal differential

Platelets: 220,000/L

Reticulocyte count: 0.5% (low)

Direct Coombs test: Negative

What is the most likely cause of this new-onset anemia?

A. Disease progression

B. Imatinib-induced myelosuppression

C. Iron deficiency anemia

D. Autoimmune hemolytic anemia

E. Gastrointestinal bleeding

answer: B. Imatinib-induced myelosuppression

Edit

Correct! Imatinib-induced myelosuppression is the most likely cause of this new-onset anemia.

Rationale:

The patient is experiencing significant anemia (Hgb 8.2 g/dL) despite having achieved a good molecular response (MMR)

Key features supporting imatinib-induced myelosuppression:

Isolated anemia with relatively preserved WBC and platelet counts (selective cytopenia can occur with TKIs)

Low reticulocyte count (0.5%) indicating decreased red blood cell production rather than increased destruction or loss

Negative Direct Coombs test ruling out immune-mediated hemolysis
Timing is consistent with TKI-related myelosuppression, which can occur at any point during treatment
Myelosuppression is one of the most common adverse effects of imatinib, occurring in approximately 35-45% of patients
The other options are less likely because:

Disease progression (option A): The patient has achieved MMR (BCR-ABL1 0.08%), making disease progression very unlikely
Iron deficiency anemia (option C): No history suggesting iron deficiency; low reticulocyte count is typical of TKI myelosuppression
Autoimmune hemolytic anemia (option D): Negative Direct Coombs test rules this out
Gastrointestinal bleeding (option E): No clinical information suggesting GI bleeding, and patient has a low reticulocyte count (would be elevated with bleeding)
Phase 3: Post-Remission Therapy and Long-Term Management

Question 8:
What is the most appropriate management for this patient's imatinib-induced anemia?
A. Discontinue imatinib and switch to nilotinib
B. Reduce imatinib dose to 300 mg daily and monitor complete blood counts weekly
C. Continue imatinib at the current dose and initiate erythropoiesis-stimulating agent (ESA) therapy
D. Transfuse packed red blood cells (PRBCs) as needed and continue imatinib at the current dose
E. Hold imatinib until hemoglobin recovers to >10 g/dL, then resume at a reduced dose
answer: B. Reduce imatinib dose to 300 mg daily and monitor complete blood counts weekly

Edit
Correct! Reducing imatinib dose to 300 mg daily and monitoring complete blood counts weekly is the most appropriate management for this patient's imatinib-induced anemia.

Rationale:

For grade 3 hematologic toxicity (Hgb <8.0-6.5 g/dL), the recommended approach is to:
Temporarily reduce the TKI dose
Monitor blood counts more frequently
Resume full dose once toxicity resolves to grade 1 or less
The patient has already achieved MMR (BCR-ABL1 0.08%), so a temporary dose reduction is unlikely to compromise disease control
Dose reduction is preferred over discontinuation or switching to another TKI when:
The patient is responding well to therapy
The toxicity is manageable
There are no other concerning factors
The other options are less appropriate because:

Switching to nilotinib (option A) is too aggressive for manageable toxicity in a patient with good response
Continuing imatinib at the current dose with ESA (option C) doesn't address the underlying cause
Transfusion support (option D) is appropriate for symptomatic anemia but doesn't address the underlying cause
Complete discontinuation (option E) is generally not recommended as the first step for manageable toxicity
Follow-up:
After dose reduction and weekly monitoring, the patient's hemoglobin gradually improves. After 6 weeks, his hemoglobin stabilizes at 11.5 g/dL. The dose is then cautiously increased back to 400 mg daily with biweekly CBC monitoring, and the patient tolerates this well.
At the 36-month follow-up:

The patient has maintained MMR for >2 years
BCR-ABL1/ABL1 (IS) is now 0.0032% (MR4.5)
The patient expresses interest in treatment-free remission (TFR)

Question 9:
Based on current guidelines, which of the following is a prerequisite for attempting treatment-free remission (TFR) in this patient?

A. Achievement of complete molecular response (undetectable BCR-ABL1) for at least 2 years
B. Sustained deep molecular response (MR4 or better) for at least 2 years
C. Allogeneic stem cell transplantation followed by 1 year of TKI therapy
D. Sustained deep molecular response (MR4 or better) for at least 3 years
E. Achievement of MMR for at least 5 years
answer: B. Sustained deep molecular response (MR4 or better) for at least 2 years

Edit
Correct! Sustained deep molecular response (MR4 or better) for at least 2 years is a prerequisite for attempting treatment-free remission.

Rationale:

According to current ELN and ESMO guidelines, the minimum criteria for TFR consideration include:
Deep molecular response (MR4 or better, meaning BCR-ABL1 0.01% IS)
Sustained for at least 2 years
Continuous TKI therapy for at least 3 years prior to discontinuation
The patient meets these criteria:
Current BCR-ABL1 level is 0.0032% (MR4.5, which is better than MR4)
Has maintained at least MMR for >2 years
Has been on TKI therapy for 36 months (3 years)
Other requirements for TFR consideration include:

Access to high-quality, standardized molecular monitoring
Patient's willingness to comply with more frequent monitoring after discontinuation
No history of accelerated or blast phase CML
The other options are incorrect because:

Option A (undetectable BCR-ABL1 for 2 years): Guidelines don't require completely undetectable transcripts
Option C (allogeneic SCT): Not required for TFR attempts in patients with good TKI responses
Option D (MR4 for at least 3 years): Current guidelines recommend 2 years, not 3
Option E (MMR for 5 years): MMR alone is insufficient; deeper response (MR4 or better) is required

Follow-up:
The patient meets criteria for TFR attempt. After discussion of the benefits and risks (including approximately 50% chance of molecular relapse requiring treatment reinitiation), the patient decides to proceed with TFR. Imatinib is discontinued, and a strict monitoring schedule is established.

Question 10:
Six months after TKI discontinuation, the patient's BCR-ABL1/ABL1 (IS) is 0.15%. According to current guidelines, what is the most appropriate next step?
A. Continue monitoring without restarting TKI therapy
B. Restart the same TKI (imatinib) at the previous effective dose
C. Switch to a second-generation TKI (nilotinib or dasatinib)
D. Perform bone marrow biopsy to rule out disease progression
E. Consider allogeneic stem cell transplantation
hint

Edit
Here's a hint to help you determine the most appropriate next step:

Consider the current guidelines for monitoring and managing TFR attempts. Think about:

What is the definition of molecular relapse during TFR?
What BCR-ABL1 threshold requires reinitiation of therapy?
Is the patient's current value (0.15%) above or below this threshold?
Does loss of MMR (BCR-ABL1 >0.1%) have any particular significance in TFR monitoring?
What is the recommended approach when molecular relapse occurs?
Remember that current guidelines have specific definitions for when treatment should be restarted during TFR monitoring, and these definitions are based on specific BCR-ABL1 thresholds.

answer: B. Restart the same TKI (imatinib) at the previous effective dose

Edit
Correct! Restarting the same TKI (imatinib) at the previous effective dose is the most appropriate next step.

Rationale:

According to current ELN and ESMO guidelines, molecular relapse during TFR is defined as loss of MMR (BCR-ABL1 >0.1% IS)
The patient's current BCR-ABL1 level is 0.15%, which exceeds the 0.1% threshold, indicating loss of MMR
In cases of molecular relapse during TFR:
Prompt reinitiation of the previously effective TKI is recommended
The same TKI that achieved the initial deep molecular response is preferred
The previous effective dose should be used (400 mg daily in this case)
Most patients who experience molecular relapse and restart TKI therapy regain MMR and deeper responses relatively quickly
The other options are not appropriate because:

Option A (continue monitoring): With loss of MMR, guidelines clearly recommend restarting therapy
Option C (switch to second-generation TKI): Not necessary if the patient previously responded well to imatinib
Option D (bone marrow biopsy): Not indicated for a typical molecular relapse during TFR
Option E (allogeneic SCT): Significantly more aggressive than necessary for a predictable molecular relapse during TFR
Case Conclusion:
The patient restarts imatinib 400 mg daily and regains MMR within 3 months. After 12 months of therapy, he achieves MR4.5 again. The patient continues on imatinib therapy with regular monitoring every 3 months.

Key Learning Points from this Case:

Diagnostic approach to CML focuses on detecting the BCR-ABL1 fusion gene, with molecular testing from peripheral blood being a first-line diagnostic approach
Risk stratification guides treatment decisions and prognostication in CML
Imatinib remains a standard first-line therapy for chronic phase CML
Response assessment should follow ELN guidelines with specific milestones at 3, 6, and 12 months
TKI-related adverse effects can often be managed with supportive care without compromising efficacy
Treatment-free remission is a realistic goal for patients with sustained deep molecular responses
Monitoring during TFR should be more frequent, with prompt TKI reinitiation upon loss of MMR
Thank you for completing this CML Clinical Case Management session. You demonstrated strong understanding of CML diagnosis, treatment selection, monitoring, and long-term management strategies aligned with current guidelines.

Session ID:
Assessment Domain

Session-87:
Rationale

Session ID:
Instructional Design and Pedagogical

Session-87:
The session's instructional design is very strong, with an expertly scaffolded case that progresses logically from diagnosis through treatment, adverse event management, and long-term goals like treatment-free remission (TFR). Instructions are clear, and the case structure effectively builds complexity. The main weaknesses lie in feedback and instructional clarity. Learning objectives are not explicitly stated upfront, though they are strongly implied. More significantly, the feedback for Question 6 is flawed; it labels the question a "trick question" and provides a convoluted rationale that incorrectly identifies the "correct" answer as "MMR" when the patient's BCR-ABL level of 0.3% actually falls into the "Warning" category by ELN 2022 criteria, an option that was not provided. This pedagogical misstep detracts from an otherwise excellent session.

Session ID:
Clinical Authenticity and Content Fidelity

Session-87:
The session's clinical authenticity is high. The narrative is indistinguishable from a real patient case, with a plausible presentation, lab values, and clinical course. The case remains strictly within the domain of Chronic Myeloid Leukemia (CML), with no evidence of domain entanglement. However, there are minor issues with content fidelity. The recommendation in Question 3 to start imatinib for an intermediate-risk patient is an acceptable choice but is arguably suboptimal. Major guidelines (NCCN, ELN) often prefer a second-generation TKI in this setting to achieve faster, deeper responses, which are linked to better long-term outcomes and higher chances of successful TFR. This choice is acceptable but not the optimal, first-line recommendation for this specific risk stratum, thus lowering the pharmacotherapeutic accuracy and guideline alignment scores.

Session ID:
Modeled Clinical Reasoning

Session-87:
The simulation is outstanding in its modeling of expert clinical reasoning. Each question frames a distinct and relevant clinical problem, providing all necessary data for a decision. The multiple-choice options offer an excellent breadth of plausible choices and key distractors that effectively test for nuanced understanding (e.g., PCR vs. biopsy, TKI dose reduction vs. switching). The overall decision-making process is sound, correctly prioritizing diagnostics, response monitoring, adverse effect management, and long-term strategic goals. The clinical outcomes described in response to each decision are realistic, specific, and highly instructive, reinforcing the key learning objectives of the case.

Session-88

Session ID:
LLM

Session-88:
ChatGPT (GPT 4.o)

Session ID:
Session Transcript

Session-88:
CASE PRESENTATION - PHASE 1
Patient: Ahmed, 44-year-old male
Chief Complaint: Fatigue, night sweats, unintentional weight loss (6 kg over 3 months), and left upper quadrant discomfort
Medical History:

No significant past illnesses
No regular medications
No known allergies

Physical Examination:

Vital signs: T 37.4C, HR 92 bpm, BP 128/78 mmHg, RR 16/min
General: Appears fatigued but in no acute distress
Abdomen: Splenomegaly present (spleen palpable 4 cm below costal margin)
No lymphadenopathy

Laboratory Results:

WBC: 98.5 10^9/L (elevated)
Hemoglobin: 10.8 g/dL (slightly decreased)
Platelets: 445 10^9/L (normal)
Peripheral blood smear: Shows increased granulocytes at all stages of maturation, basophilia (6%), and 2% blasts
LDH: 780 U/L (elevated)

QUESTION 1:
Based on the initial presentation, which of the following diagnostic tests would be most appropriate to confirm the suspected diagnosis?
A. Flow cytometry of peripheral blood
B. Bone marrow aspiration and biopsy with cytogenetic analysis
C. CT scan of chest, abdomen, and pelvis
D. Serum protein electrophoresis
Correct! Bone marrow aspiration and biopsy with cytogenetic analysis is the most appropriate test.
Feedback:
You've made the right choice. Bone marrow aspiration and biopsy with cytogenetic analysis is essential for CML diagnosis according to current guidelines. This approach allows for:

Morphological assessment of the bone marrow
Cytogenetic analysis to identify the Philadelphia chromosome t(9;22)(q34;q11)
FISH or PCR testing for BCR-ABL1 fusion gene
Assessment of blast percentage for disease phase classification

The other options are less appropriate:

Flow cytometry (A) is useful but not sufficient for CML diagnosis
CT scan (C) may help evaluate splenomegaly but doesn't confirm diagnosis
Serum protein electrophoresis (D) is more relevant for plasma cell disorders

Results of the bone marrow studies:

Hypercellular marrow (95%)
Myeloid ratio of 15:1 (increased)
3% blasts, 12% promyelocytes
Cytogenetics: t(9;22)(q34;q11) in all 20 metaphases analyzed (Philadelphia chromosome)
RT-PCR: BCR-ABL1 transcript detected (p210, e14a2 variant)
QUESTION 2:
Based on these findings, which phase of CML is this patient in according to the 2022 ELN criteria?
A. Chronic phase
B. Accelerated phase
C. Blast phase
D. Pre-leukemic phase
Correct! This patient is in the chronic phase of CML.
Feedback:
Your assessment is accurate. According to the 2022 European LeukemiaNet (ELN) criteria, this patient presents with classic features of chronic phase CML:

Peripheral blood blasts <10% (patient has 2%)
Bone marrow blasts <10% (patient has 3%)
No evidence of extramedullary blast proliferation
No features of accelerated or blast phase

The key diagnostic findings supporting chronic phase CML include:

Elevated WBC with granulocytes at all stages of maturation
Presence of Philadelphia chromosome t(9;22) by cytogenetics
Detection of BCR-ABL1 transcript by PCR
Splenomegaly
Constitutional symptoms (fatigue, night sweats, weight loss)

Additional testing results:

Quantitative RT-PCR: BCR-ABL1/ABL1 ratio of 45% (IS)
QUESTION 3:
Which risk stratification score should be calculated next to guide treatment decisions and prognosis for this patient?
A. Sokal score
B. Euro score
C. EUTOS score
D. All of the above
Correct! All of these risk stratification scores should be calculated.
Feedback:
Excellent choice. For newly diagnosed CML patients, calculating multiple prognostic scoring systems provides comprehensive risk stratification that helps guide treatment decisions and predict outcomes. Each score has unique components and prognostic value:

Sokal score: The original prognostic model (1984) that considers age, spleen size, platelet count, and blast percentage
Euro (Hasford) score: Includes the Sokal parameters plus basophil and eosinophil percentages
EUTOS score: A simpler model based on basophil percentage and spleen size

EUTOS Long-Term Survival (ELTS) score: The most recent addition (not specifically listed but important), which better reflects survival in the TKI era

The 2022 ELN guidelines recommend calculating all these scores at diagnosis as they provide complementary prognostic information. While some centers may emphasize one score over others, using all available tools provides the most comprehensive assessment.
Risk Assessment Results:

Sokal score: 1.2 (Intermediate risk)
Euro score: 780 (Intermediate risk)
EUTOS score: 87 (High risk)
ELTS score: 2.2 (Intermediate risk)
CASE PRESENTATION - PHASE 2
With the diagnosis of chronic phase CML confirmed and risk stratification completed, treatment planning begins. The patient has normal renal and hepatic function, and his cardiac evaluation shows normal QTc interval and ejection fraction.
QUESTION 4:
What is the most appropriate first-line therapy for this patient with newly diagnosed chronic phase CML?
A. Imatinib 400 mg daily
B. Nilotinib 300 mg twice daily
C. Dasatinib 100 mg daily
D. Bosutinib 400 mg daily
Correct! Nilotinib 300 mg twice daily is the most appropriate first-line therapy for this patient.
Feedback:
You've made an excellent choice. For this intermediate-risk CML patient, nilotinib 300 mg twice daily is an appropriate first-line therapy based on the following rationale:

Second-generation TKIs (nilotinib, dasatinib, bosutinib) achieve faster and deeper molecular responses compared to imatinib
This patient has intermediate-risk features on multiple scoring systems (Sokal, Euro, ELTS)
The patient is relatively young (44 years) and may benefit from a more potent agent to achieve deeper responses
Nilotinib has demonstrated superior rates of early molecular response and major molecular response in the ENESTnd trial

While imatinib is still a valid option for CP-CML, second-generation TKIs are often preferred for patients with intermediate or high-risk disease. The choice between nilotinib, dasatinib, and bosutinib should consider patient-specific factors:

Nilotinib: Consider cardiovascular risk factors (patient has none noted)
Dasatinib: Consider pulmonary history (no issues noted)
Bosutinib: May be preferred in patients with cardiovascular risks who cannot tolerate nilotinib

Treatment Initiation:
The patient begins nilotinib 300 mg twice daily, taking it on an empty stomach (2 hours before or 1 hour after food). Baseline ECG and electrolyte monitoring are performed.
QUESTION 5:
Three weeks after starting nilotinib, the patient develops a grade 2 rash (maculopapular, covering 15% of body surface area). What is the most appropriate management approach?
A. Discontinue nilotinib and switch to dasatinib
B. Continue nilotinib at the same dose and add topical corticosteroids
C. Temporarily interrupt nilotinib until rash resolves, then resume at a reduced dose
D. Continue nilotinib at the same dose and add oral antihistamines
Correct! Continuing nilotinib at the same dose and adding topical corticosteroids is the most appropriate management approach.
Feedback:
You've made the right choice. For a grade 2 TKI-associated rash (which is moderate but not severe):

Continuing TKI therapy is important to maintain disease control
Topical corticosteroids can effectively manage this common adverse effect
Antihistamines could be added if pruritus is significant

Drug interruption is generally reserved for more severe (grade 3-4) reactions

Guidelines recommend managing mild to moderate dermatologic reactions while continuing TKI therapy whenever possible. Nilotinib-associated rash:

Is often self-limiting and improves with continued treatment
Typically responds well to topical steroids
Usually doesn't necessitate treatment interruption at grade 2 severity
Rarely requires switching to an alternative TKI

Premature discontinuation or dose reduction could compromise treatment efficacy and molecular response.
Treatment Outcome:
With continued nilotinib and twice-daily application of medium-potency topical corticosteroids, the rash improves significantly within 2 weeks.
QUESTION 6:
At 3 months after starting nilotinib, the patient's BCR-ABL1 quantitative PCR result returns as 9.5% (IS). How would you interpret this result?
A. Optimal response - continue nilotinib at the same dose
B. Warning response - continue nilotinib but monitor more frequently
C. Failure response - switch to an alternative TKI
D. Early molecular response - consider dose reduction
Feedback:
According to the 2022 ELN guidelines for CML management, at the 3-month timepoint:

Optimal response: BCR-ABL1 10% AND a 1-log reduction from baseline (patient has 9.5%, which meets the first criterion)
Warning response: BCR-ABL1 >1-10% WITHOUT a 1-log reduction from baseline
Failure response: BCR-ABL1 >10%

Since the patient's baseline BCR-ABL1/ABL1 ratio was 45% (IS), a 1-log reduction would require a value of 4.5%. The current value of 9.5% represents less than a 1-log reduction, placing this response in the "warning" category.
For patients with a warning response at 3 months, the ELN recommends:

Continuing the same TKI (nilotinib in this case)
More frequent molecular monitoring (every 1-2 months)
Assessing adherence and drug interactions
Considering BCR-ABL1 mutation analysis if there are additional concerning features

Follow-up Plan:
The patient continues nilotinib 300 mg twice daily with BCR-ABL1 PCR monitoring scheduled at 1-month intervals. Medication adherence is verified, and no drug interactions are identified.
QUESTION 7:
At the 6-month follow-up, the patient's BCR-ABL1 is now 2.8% (IS). During the visit, he reports increasing fatigue and a recent blood test shows: Hemoglobin 9.2 g/dL (decreased from baseline), WBC 5.4 10^9/L, Platelets 140 10^9/L. What is the most appropriate management at this time?
A. Switch to dasatinib due to hematologic toxicity
B. Continue nilotinib and initiate erythropoiesis-stimulating agent
C. Reduce nilotinib dose to 200 mg twice daily
D. Continue nilotinib at the same dose and monitor CBC weekly
Correct! Continuing nilotinib at the same dose and monitoring CBC weekly is the most appropriate management approach.
Feedback:
You've made the right choice. This situation requires careful consideration of both efficacy and safety:

The patient is showing improvement in his molecular response (BCR-ABL1 decreased from 9.5% to 2.8%), indicating nilotinib is effective

The current value of 2.8% at 6 months represents a "warning" response per ELN guidelines (optimal would be 1%)
The cytopenias are mild to moderate (grade 1-2) and don't necessitate immediate dose reduction
Weekly monitoring is appropriate to ensure cytopenia doesn't worsen

The other options are less appropriate:

Switching TKIs (A) is premature since the patient is responding and toxicity is manageable
ESAs (B) are generally not recommended for TKI-induced anemia as they don't address the underlying mechanism
Dose reduction (C) might compromise the improving molecular response and isn't necessary for grade 1-2 cytopenia

Follow-up Results:
With continued therapy and weekly monitoring, the patient's counts stabilize with Hgb around 9.5-10 g/dL. The molecular response continues to improve.
Let's proceed to Phase 3: Post-remission therapy, MRD monitoring, and long-term management.
CASE PRESENTATION - PHASE 3
At the 12-month follow-up, the patient's BCR-ABL1 is 0.08% (IS), representing a major molecular response (MMR). He has been tolerating nilotinib well with stable mild cytopenias. His spleen is no longer palpable.
QUESTION 8:
What is the recommended frequency of molecular monitoring at this point?
A. Every 2 weeks
B. Monthly
C. Every 3 months
D. Every 6 months
Correct! Monitoring every 3 months is the recommended frequency at this point.
Feedback:
You've made the appropriate choice. According to ELN and NCCN guidelines, for patients who have achieved a major molecular response (MMR, BCR-ABL1 0.1% IS):

Molecular monitoring every 3 months is the standard recommendation
This frequency allows for timely detection of potential loss of response
Once a patient maintains deep molecular response (MR4 or better, BCR-ABL1 0.01% IS) for at least 2 years, monitoring can be extended to every 6 months

More frequent monitoring (options A and B) would be unnecessary and resource-intensive at this point, while less frequent monitoring (option D) would be insufficient to detect early signs of relapse.
Two years later:
The patient has maintained stable MMR with BCR-ABL1 consistently 0.1% IS. His last three consecutive measurements were 0.032%, 0.018%, and 0.009% IS (MR4). He inquires about the possibility of treatment discontinuation.
QUESTION 9:
Which of the following is a prerequisite for considering TKI discontinuation in this patient?
A. At least 3 years of TKI therapy
B. At least MR4 (BCR-ABL1 0.01% IS) sustained for at least 2 years
C. Prior history of therapy with at least two different TKIs
D. Complete cytogenetic response for at least 5 years
Correct! At least MR4 (BCR-ABL1 0.01% IS) sustained for at least 2 years is a prerequisite for considering TKI discontinuation.
Feedback:
You've identified the correct prerequisite for treatment-free remission (TFR) attempts. According to the 2022 ELN guidelines, the minimum requirements for considering TKI discontinuation include:

First chronic phase CML (which our patient has)
At least 5 years of TKI therapy (our patient has been on therapy for only 3 years)
Deep molecular response (DMR) of at least MR4 (BCR-ABL1 0.01% IS)
DMR sustained for at least 2 years (confirmed by at least 4 tests)

While our patient has achieved MR4 (0.009% IS), he has only maintained it for a short time, not the required 2 years. Additionally, he hasn't yet completed 5 years of total TKI therapy. Therefore, TKI discontinuation would be premature at this point.
Other considerations for successful TFR include:

No prior history of resistance
Access to high-quality, rapid PCR testing
Ability to perform frequent monitoring
Patient willingness to accept the psychological impact of potential relapse

Patient follow-up plan:
The patient is advised to continue nilotinib therapy with monitoring every 3 months. TKI discontinuation can be reconsidered after he completes at least 5 years of therapy and maintains MR4 for at least 2 years.
QUESTION 10:
One year later (4 years after diagnosis), the patient's BCR-ABL1 increases to 1.2% IS after being undetectable for 6 months. No mutations are detected on BCR-ABL1 kinase domain mutation analysis. What is the most appropriate next step?
A. Switch to dasatinib due to nilotinib failure
B. Increase nilotinib dose to 400 mg twice daily
C. Assess adherence, continue current dose, and repeat BCR-ABL1 testing in 1-2 months
D. Refer for allogeneic stem cell transplantation evaluation
Correct! Assessing adherence, continuing the current dose, and repeating BCR-ABL1 testing in 1-2 months is the most appropriate next step.
Feedback:
You've made the right choice. When a patient with previously undetectable BCR-ABL1 shows an increase to 1.2% IS:

This represents loss of MMR but not treatment failure
The first step should be to assess for medication non-adherence, which is a common cause of molecular recurrence
Rapid intervention with repeat testing in 1-2 months is appropriate
Mutation analysis was appropriately performed and found no mutations

The other options are too aggressive at this stage:

Switching to dasatinib (A) would be premature without confirming persistent loss of response
Dose escalation (B) is not standard practice without evidence of resistance
Allogeneic stem cell transplantation (D) is reserved for patients with treatment failure, progression to advanced phases, or T315I mutation

Per the ELN guidelines, loss of MMR (BCR-ABL1 >0.1% IS) is concerning but requires confirmation and assessment of adherence before making major treatment changes.
Follow-up Results:
Upon questioning, the patient admits to occasionally missing doses due to work travel. After a thorough discussion about adherence importance, he commits to consistent dosing. A repeat BCR-ABL1 test after 2 months of strict adherence shows a decrease to 0.075% IS, regaining MMR.

Session ID:
Assessment Domain

Session-88:
Rationale

Session ID:
Instructional Design and Pedagogical

Session-88:
The session's instructional design is outstanding. The case is expertly scaffolded, moving seamlessly from initial diagnosis and risk

stratification to first-line therapy selection, adverse event management, nuanced interpretation of molecular monitoring results, and long-term management challenges. The embedded feedback is exceptionally high quality, providing clear, guideline-anchored rationales (citing 2022 ELN criteria) that explain not only why the correct answer is right but also why the distractors are incorrect. The instructions and case presentation are perfectly clear. The only minor area for improvement is that learning objectives are not explicitly stated at the beginning of the session; however, they are strongly implied by the logical structure of the case.

Session ID:
Clinical Authenticity and Content Fidelity

Session-88:
The clinical fidelity of this session is excellent and represents a gold standard for simulation. Plausibility & Guideline Alignment: The clinical narrative is indistinguishable from an authentic CML case, presenting a textbook initial diagnosis and a highly realistic patient journey. Every decision pointfrom the choice of a second-generation TKI for an intermediate-risk patient to the management of a "warning" molecular response and the criteria for treatment-free remissionis perfectly aligned with current evidence-based guidelines (ELN, NCCN). Pharmacotherapeutic Accuracy: The pharmacotherapeutic choices and rationales are optimal and error-free. The selection of nilotinib, the management of the associated rash, and the handling of mild cytopenias are all appropriate and well-justified. Domain Specificity: The simulation is flawless in its domain specificity. All diagnostics, monitoring tools, and therapies are strictly relevant to Chronic Myeloid Leukemia. There are no instances of domain entanglement where concepts or treatments from other hematologic malignancies are incorrectly applied.

Session ID:
Modeled Clinical Reasoning

Session-88:
The simulation excels at modeling an expert clinical reasoning process. Each question presents a well-framed clinical problem that is relevant and timely within the case narrative. The multiple-choice options offer an excellent breadth of plausible alternatives and key distractors, effectively testing the user's nuanced understanding (e.g., choosing between different TKIs, or different management strategies for an adverse event). The modeled decision-making demonstrates sound clinical prioritization, such as the critical step of assessing adherence before declaring a treatment failure. Finally, the clinical outcomes described after each decision are realistic, specific, and highly instructive, reinforcing the key learning points.

Session-89

Session ID:
LLM

Session-89:
ChatGPT (GPT 4.o)

Session ID:
Session Transcript

Session-89:
PHASE 1: Initial Presentation and Diagnostic Workup
Patient Information:

Name: John Wilson
Age: 57
Sex: Male
Weight: 78 kg
Height: 175 cm
Chief Complaint: Progressive fatigue, unintentional weight loss (5 kg over 3 months), and night sweats
Past Medical History: Hypertension (well-controlled on lisinopril 10 mg daily)
Social History: Former smoker (quit 5 years ago), occasional alcohol use
Family History: Father with colorectal cancer at age 62

Physical Examination:

Vital Signs: BP 132/84 mmHg, HR 88 bpm, RR 18/min, Temp 37.2C
General: Alert, oriented, appears fatigued
HEENT: Unremarkable
Cardiovascular: Regular rate and rhythm, no murmurs
Respiratory: Clear to auscultation bilaterally
Abdomen: Splenomegaly noted (spleen palpable 4 cm below left costal margin), non-tender
Lymph nodes: No palpable lymphadenopathy

Laboratory Results:

WBC: 112,000/L (elevated)
Hemoglobin: 10.1 g/dL
Platelets: 435,000/L
Peripheral blood smear: Increased granulocytes at all stages of maturation, basophilia (6%), and <2% blasts
Comprehensive metabolic panel: Within normal limits except for mildly elevated LDH (390 U/L)

Question 1:
Based on the initial presentation, what is the most appropriate next diagnostic test to confirm the suspected diagnosis?
A) Flow cytometry of peripheral blood
B) Cytogenetic analysis and FISH for BCR-ABL1
C) Next-generation sequencing panel
D) CT scan of chest, abdomen, and pelvis
E) Bone marrow aspiration and biopsy with conventional karyotyping
B) Cytogenetic analysis and FISH for BCR-ABL1
Question 2:
The patient's cytogenetic analysis shows t(9;22)(q34;q11.2) in all 20 metaphases examined. FISH confirms BCR-ABL1 fusion gene.

RT-PCR shows BCR-ABL1/ABL1 ratio of 78% (IS). Bone marrow examination shows 2% blasts with no additional chromosomal abnormalities.
According to the 2022 European LeukemiaNet (ELN) criteria, what is this patient's risk classification?
A) Low risk
B) Intermediate risk
C) High risk
D) Very high risk
E) Cannot be determined without additional information
A) Low risk
Question 3:
Based on this patient's diagnosis and risk classification, what is the most appropriate first-line treatment option?
A) Imatinib 400 mg once daily
B) Dasatinib 100 mg once daily
C) Nilotinib 300 mg twice daily
D) Ponatinib 45 mg once daily
E) Bosutinib 400 mg once daily
A) Imatinib 400 mg once daily
PHASE 2: Induction Therapy and Supportive Care
Two months after starting imatinib 400 mg daily, the patient returns for follow-up:

The patient reports mild nausea and periorbital edema
CBC shows: WBC 8,200/L, Hgb 12.1 g/dL, Platelets 230,000/L
Liver function tests show: ALT 85 U/L (reference range: 7-56 U/L), AST 62 U/L (reference range: 10-40 U/L), total bilirubin and alkaline phosphatase within normal limits

Question 4:
What is the most appropriate management approach for this patient's elevated liver enzymes?
A) Discontinue imatinib immediately and switch to nilotinib
B) Reduce imatinib dose to 300 mg daily and recheck liver enzymes in 2 weeks
C) Continue current imatinib dose, monitor liver enzymes weekly, and reassess if ALT rises >3x ULN
D) Hold imatinib until liver enzymes normalize, then restart at 300 mg daily
E) Add a hepatoprotective agent like silymarin while maintaining the current imatinib dose
D) Hold imatinib until liver enzymes normalize, then restart at 300 mg daily
Question 5:
At the 3-month follow-up evaluation, BCR-ABL1 testing shows a BCR-ABL1/ABL1 ratio of 22% (IS). According to ELN recommendations, how would you classify this response?
A) Optimal response
B) Warning (suboptimal response)
C) Treatment failure
D) Blast crisis
E) Early molecular response
B) Warning (suboptimal response)
Question 6:
Based on this patient's 3-month response, what is the most appropriate next step in management?
A) Switch immediately to a second-generation TKI (dasatinib or nilotinib)
B) Increase imatinib dose to 600 mg daily
C) Continue current imatinib dose with BCR-ABL1 monitoring in 1-2 months
D) Order BCR-ABL kinase domain mutation analysis
E) Refer for allogeneic stem cell transplantation evaluation
C
Question 7:
At the 6-month follow-up, the patient's BCR-ABL1/ABL1 ratio is 15% (IS). He reports good medication adherence. What is the most appropriate management strategy at this point?
A) Continue imatinib 400 mg daily and reassess in 3 months

B) Increase imatinib dose to 600-800 mg daily
C) Switch to a second-generation TKI (dasatinib, nilotinib, or bosutinib)
D) Perform BCR-ABL kinase domain mutation analysis and continue current therapy while awaiting results
E) Refer for hematology consultation for consideration of clinical trial
C
PHASE 3: Post-Remission Therapy and Long-Term Management
The patient is switched to nilotinib 300 mg twice daily. Three months later (9 months from diagnosis), his BCR-ABL1/ABL1 ratio has decreased to 1.2% (IS). He tolerates nilotinib well but reports:

Mild headaches (managed with acetaminophen)
Fasting glucose 112 mg/dL (was 94 mg/dL at baseline)
Total cholesterol 220 mg/dL (was 190 mg/dL at baseline)

Question 8:
Which of the following is the most appropriate monitoring frequency for BCR-ABL1 quantification at this point in treatment?
A) Every 2 weeks
B) Monthly
C) Every 3 months
D) Every 6 months
E) Yearly
C
Question 9:
The patient has now been on nilotinib for 24 months. His most recent BCR-ABL1/ABL1 ratio is 0.0032% (IS), which has been stable for the past 12 months. His fasting glucose is 126 mg/dL, and total cholesterol is 245 mg/dL despite lifestyle modifications. Which of the following statements regarding TKI discontinuation (treatment-free remission) is correct for this patient?
A) The patient should immediately discontinue nilotinib since he has achieved complete molecular response
B) This patient is not eligible for a TFR attempt because he initially failed imatinib therapy
C) The patient meets criteria for a TFR attempt if he has proper monitoring capabilities
D) TFR should only be attempted after switching to a lower dose of nilotinib for 12 months
E) TFR is contraindicated because the patient has developed metabolic adverse effectsRetryAK
C
Question 10:
The patient decides to attempt treatment-free remission (TFR) after discussing the risks and benefits. Which of the following monitoring schedules is most appropriate during the first 6 months of TFR?
A) BCR-ABL1 testing every 2 weeks
B) BCR-ABL1 testing monthly
C) BCR-ABL1 testing every 3 months
D) BCR-ABL1 testing every 6 months
E) Complete blood count monthly with BCR-ABL1 testing every 3 months
B

Session ID:
Assessment Domain

Session-89:
Rationale

Session ID:
Instructional Design and Pedagogical

Session-89:
The session's instructional design is strong, with excellent scaffolding that logically progresses the learner from initial diagnosis through long-term management of Chronic Myeloid Leukemia (CML). Instructions are exceptionally clear and the case flow is intuitive. The primary weakness lies in the quality of the embedded feedback; the recommended action in Question 6 (continuing imatinib

despite a "warning" signal) is inconsistent with ELN guidelines, representing a significant pedagogical error. Additionally, learning objectives are not explicitly stated, though they are strongly implied by the case structure.

Session ID:
Clinical Authenticity and Content Fidelity

Session-89:
The clinical narrative is highly plausible and indistinguishable from an authentic CML patient case, including typical disease progression and drug-specific adverse effects. The session demonstrates perfect domain specificity, with all concepts and therapies remaining strictly within the CML field. However, the overall fidelity is diminished by a key error in Question 6. The chosen answer to continue imatinib without change despite a "warning" signal at 3 months deviates from clinical guidelines (which call for more active investigation or a switch) and represents a suboptimal pharmacotherapeutic choice that could delay optimal disease control.

Session ID:
Modeled Clinical Reasoning

Session-89:
The simulation excellently models an expert clinical reasoning process. Each stage presents an expertly framed clinical problem, and the multiple-choice questions offer an excellent breadth of plausible and relevant options, including key distractors. The clinical outcomes are realistic and highly instructive. The prioritization and decision-making are mostly sound, but the score is slightly reduced due to the flawed decision in Question 6. The choice to passively monitor in the face of a "warning" signal demonstrates a flawed prioritization of risks, failing to adequately address the potential for treatment failure.

Session-90

Session ID:
LLM

Session-90:
ChatGPT (GPT 4.o)

Session ID:
Session Transcript

Session-90:
Patient: Mr. James K., a 47-year-old male
Chief Complaint: Fatigue and early satiety for the past 3 months

History & Review of Systems:

Fatigue, night sweats
No weight loss, bruising, or infections
No significant past medical history
No medications
No alcohol or tobacco use
Physical Exam:

Spleen palpable 6 cm below left costal margin
No lymphadenopathy, petechiae, or ecchymoses
Laboratory Findings:

Test Result Reference Range
WBC 145 x 10^9/L 4.011.0 x 10^9/L
Hemoglobin 11.2 g/dL 13.517.5 g/dL
Platelets 600 x 10^9/L 150450 x 10^9/L
Peripheral Smear Marked left shift, basophilia, eosinophilia
Bone Marrow Biopsy:

Hypercellular marrow with myeloid hyperplasia
Blasts: <2%
Cytogenetics (FISH & Karyotyping):

t(9;22)(q34;q11.2) detected
Molecular Testing:

BCR::ABL1 major (p210) fusion transcript detected via RT-PCR
IS BCR::ABL1: 95%
Decision Point 1:
Which of the following is the most appropriate diagnosis based on WHO 2022 classification?

A. Acute Myeloid Leukemia with BCR::ABL1
B. Chronic Myeloid Leukemia Chronic Phase
C. Chronic Neutrophilic Leukemia
D. Myeloproliferative Neoplasm, Unclassifiable

Type A, B, C, or D and explain your reasoning. Ill provide feedback after your answer.

Correct Answer: B. Chronic Myeloid Leukemia Chronic Phase

Your justification is thorough and aligns with the WHO 2022 Classification and ELN 2022 guidelines, which define CML-CP based on:

Presence of BCR::ABL1 fusion gene (major transcript p210)
t(9;22)(q34;q11.2) (Philadelphia chromosome) on cytogenetics
Blasts <10% in peripheral blood and bone marrow
Classic clinical and lab findings: marked leukocytosis, basophilia, eosinophilia, splenomegaly
Patient: Mr. James K., a 47-year-old male
Chief Complaint: Fatigue and early satiety for the past 3 months

History & Review of Systems:

Fatigue, night sweats
No weight loss, bruising, or infections
No significant past medical history
No medications
No alcohol or tobacco use
Physical Exam:

Spleen palpable 6 cm below left costal margin
No lymphadenopathy, petechiae, or ecchymoses
Laboratory Findings:

Test Result Reference Range
WBC 145 x 10^9/L 4.011.0 x 10^9/L
Hemoglobin 11.2 g/dL 13.517.5 g/dL
Platelets 600 x 10^9/L 150450 x 10^9/L
Peripheral Smear Marked left shift, basophilia, eosinophilia
Bone Marrow Biopsy:

Hypercellular marrow with myeloid hyperplasia
Blasts: <2%
Cytogenetics (FISH & Karyotyping):

t(9;22)(q34;q11.2) detected
Molecular Testing:

BCR::ABL1 major (p210) fusion transcript detected via RT-PCR
IS BCR::ABL1: 95%
Using the ELTS risk model, how would you classify this patients risk category?

A. Low Risk
B. Intermediate Risk
C. High Risk
D. Unable to determine more information needed

(Type A, B, C, or D, and walk me through how you applied the ELTS score.)
Lets walk through the ELTS (EUTOS Long-Term Survival) score together to assess if High Risk (C) is appropriate.

ELTS Score Formula
ELTS = 0.0025 Age (years) + 0.0615 Spleen size (cm) + 0.1052 Blasts (%) + 0.4104 (Platelet count/1000)^0.5

Lets plug in James K.s data:

Age = 47
Spleen size = 6 cm
Blasts = 1%
Platelets = 600 10/L
Convert platelets:
600 = 24.5 (approx)

Now calculate:

ELTS = (0.0025 47) + (0.0615 6) + (0.1052 1) + (0.4104 24.5)
ELTS 0.1175 + 0.369 + 0.1052 + 10.0488
ELTS 10.64

ELTS Risk Stratification
Low risk: 1.5680
Intermediate risk: >1.5680 to 2.2185
High risk: >2.2185
So:
James K. has a High ELTS score of 10.64 High Risk Category

Correct Answer: C. High Risk
Well done you selected the correct option. This is a key prognostic factor, especially in guiding how intensively we monitor BCR::ABL1 transcripts and whether we may consider earlier intervention with second-generation TKIs or transplant referral.
You previously determined that James K. is:

Diagnosed with CML in Chronic Phase
High ELTS Risk
47 years old, no comorbidities, ECOG performance status 0
BCR::ABL1 IS = 95% at diagnosis
He is treatment-nave and eager to begin therapy promptly.

Therapeutic Considerations:
Factor Implication
Age < 60, fit Eligible for any TKI
High ELTS Risk May benefit from deeper/faster molecular responses
No comorbidities No contraindications to 2nd-generation TKIs
Goals: Achieve BCR::ABL1 10% at 3 months (ELN 2022), minimize resistance, aim for treatment-free remission long-term
Decision Point 3:
Which of the following is the most appropriate first-line therapy for James K.?

A. Imatinib 400 mg daily
B. Dasatinib 100 mg daily
C. Nilotinib 300 mg twice daily
D. Ponatinib 45 mg daily
E. Allogeneic stem cell transplant

Please choose A, B, C, D, or E and explain your clinical reasoning using ELN 2022 guidance.

You said:
Answer: B
2nd-generation TKI (2G-TKI) recommended for patients with high ELTS risk because:
They are more likely to achieve early molecular response (EMR) at 3 months.

Better at achieving deep molecular responses (MR4, MR4.5) needed for future TFR.
Dasatinib 100 mg daily is approved first-line, well-tolerated in younger, fit patients.
ELN 2022 specifically supports 2G-TKIs (dasatinib, nilotinib, bosutinib) over imatinib in high-risk patients.
ChatGPT said:
Excellent choice and superb clinical rationale your answer is correct.

Correct Answer: B. Dasatinib 100 mg daily

Your reasoning reflects a solid understanding of ELN 2022 recommendations and key pharmacotherapeutic principles in CML:

Why 2G-TKI (Dasatinib) in High-Risk Patients?
ELN 2022: Recommends 2nd-generation TKIs (dasatinib, nilotinib, bosutinib) over imatinib for high-risk patients (e.g., high ELTS score) to improve:
Early Molecular Response (EMR) at 3 months (BCR::ABL1 10%)
Depth of response (MR4, MR4.5) required for Treatment-Free Remission (TFR) eligibility
Lower progression to accelerated/blast phase
Dasatinib 100 mg daily is:
FDA- and EMA-approved for first-line use
Well-tolerated in younger, fit adults
Offers rapid BCR::ABL1 decline (median <3 months to EMR)
Ponatinib (D) is reserved for patients with T315I mutation or multiple TKI failures due to vascular risk.
Stem cell transplant (E) is not first-line in chronic phase unless there is TKI failure or intolerance.
Supportive Care Planning
Now that youve selected dasatinib, supportive care and monitoring need to be tailored to this agent.

Decision Point 4:
Which of the following supportive care or monitoring strategies is most appropriate to implement with dasatinib therapy?

A. Routine monitoring of serum lipids and blood glucose
B. Monthly echocardiograms for QTc prolongation
C. Monitoring for pleural effusions and pulmonary symptoms
D. Prophylactic corticosteroids to prevent fluid retention
E. Primary antifungal prophylaxis with posaconazole

Select A, B, C, D, or E, and explain your rationale.

You said:
Answer: C
Dasatinib is associated with pleural effusion as one of its most common and clinically significant adverse effects, occurring in up to 2030% of patients. Therefore, routine monitoring for pulmonary symptoms (e.g., dyspnea, cough, chest pain) and early imaging (chest X-ray) when symptoms arise is essential.
ChatGPT said:
Absolutely correct and well-explained.

Correct Answer: C. Monitoring for pleural effusions and pulmonary symptoms

Key Points on Dasatinib Toxicity:
Pleural effusion is a well-recognized adverse effect, often dose-dependent and more common in:
Older adults
Patients with a history of cardiac or pulmonary disease
Those on high-dose or long-term dasatinib
Symptoms to monitor:
Dyspnea, dry cough, orthopnea, and chest discomfort
Management strategies include:

Interrupting or reducing dose
Diuretics, steroids, or even switching TKIs if recurrent
Baseline chest X-ray not mandatory, but prompt imaging is needed if symptoms develop
Why the Other Options Are Incorrect:
A. Serum lipids/glucose: Important for nilotinib, not dasatinib.
B. QTc monitoring: Relevant for nilotinib and ponatinib, not dasatinib.
D. Corticosteroid prophylaxis: Not standard or evidence-based.
E. Posaconazole prophylaxis: Reserved for neutropenic regimens, not needed here.
James K. begins dasatinib 100 mg daily with education on signs of pulmonary toxicity and scheduled molecular monitoring every 3 months.

Type PHASE 3 to move to the final segment: Post-remission Therapy, MRD Monitoring, and Long-Term Management.

You said:
PHASE 3
ChatGPT said:
Phase 3: Post-Remission Therapy, MRD Monitoring, and Long-Term Management

Learning Objectives for Phase 3
By the end of this phase, you should be able to:

Understand the goals of therapy post-remission in CML and how to assess treatment response.
Interpret MRD (Minimal Residual Disease) monitoring results and make adjustments to therapy based on these results.
Plan long-term management, including considerations for treatment-free remission (TFR) and monitoring for potential relapse.
Identify when and how to switch therapies in cases of resistance or intolerance.
Clinical Case Part 3: Post-Remission and Long-Term Management
After 3 months of dasatinib therapy, James K. undergoes routine follow-up with the following results:

Clinical status: No significant adverse effects, and his splenomegaly has reduced.
Lab results:
Test Result Reference Range
BCR::ABL1 (IS) 12% 10% is optimal for early response (EMR)
Platelets 350 x 10^9/L 150450 x 10^9/L
WBC 24 x 10^9/L 4.011.0 x 10^9/L
Physical exam: No new symptoms, stable performance status (ECOG 0).
Decision Point 5:
What is the most appropriate next step in James K.'s management based on the 3-month BCR::ABL1 result (12%)?

A. Increase dasatinib dose to 140 mg daily
B. Switch to nilotinib 300 mg twice daily
C. Continue dasatinib and monitor at 6 months
D. Consider switching to a third-generation TKI (ponatinib or asciminib)
E. Refer for stem cell transplant evaluation

Please choose A, B, C, D, or E and provide your clinical reasoning.

You said:
Answer: C.
ChatGPT said:
Correct Answer: C. Continue dasatinib and monitor at 6 months

Explanation and Clinical Reasoning:
James K. is demonstrating suboptimal early molecular response (BCR::ABL1 IS = 12%) at 3 months. According to ELN 2022 guidelines:

Early Molecular Response (EMR) is defined as BCR::ABL1 IS 10% at 3 months.
Failure to achieve EMR (BCR::ABL1 >10% at 3 months) is concerning and warrants closer follow-up, but does not immediately indicate a change in therapy. Non-EMR patients should be monitored closely for potential dose adjustments or switching.
However, the management strategy typically involves:

Continuation of current TKI therapy while closely monitoring molecular response over the next 3 months (i.e., at the 6-month time point).
A more aggressive change in therapy (e.g., switching to another TKI) is typically considered if failure to achieve a significant molecular response persists at 6 months.
Why Not the Other Options?
A. Increase dasatinib dose to 140 mg daily:
No immediate dose escalation is recommended for suboptimal response at 3 months. Increasing the dose should be considered only if a patient demonstrates resistance or intolerance to the current dose over time, and after failure to achieve MR4 or deeper responses.
B. Switch to nilotinib 300 mg twice daily:
Switching to another 2nd-generation TKI is typically considered if no significant improvement in BCR::ABL1 is seen at the 6-month mark, not at 3 months. A switch may also be considered if there is intolerance to dasatinib.
D. Consider switching to a third-generation TKI (ponatinib or asciminib):
Third-generation TKIs like ponatinib or asciminib are reserved for cases with TKI resistance or the T315I mutation. This is not relevant in the absence of those conditions at 3 months.
E. Refer for stem cell transplant evaluation:
Stem cell transplant is typically considered if there is failure to achieve a deep molecular response (MR4 or MR4.5) after 12 years of appropriate TKI therapy, especially if the patient develops accelerated phase or blast crisis. This is not applicable yet in James K.'s case, as he is still in chronic phase.
Next Steps in Management:
Follow-up in 3 months for a 6-month BCR::ABL1 IS measurement.
If BCR::ABL1 IS remains >10% at 6 months, further action is warranted, such as considering a switch to nilotinib or a third-generation TKI.
Monitor for any signs of adverse events, such as pleural effusion, which could indicate dose-related toxicity.
Conclusion:
James K.'s response at 3 months suggests suboptimal early molecular response, but it is not an immediate cause for treatment change. Continue dasatinib at the current dose, and follow up in 3 months for further molecular assessment.

Type "FINAL ASSESSMENT" to conclude the case and review key takeaways!

You said:
FINAL ASSESSMENT
ChatGPT said:
Final Assessment: Key Takeaways from the Case on Chronic Myeloid Leukemia (CML)
Phase 1: Initial Presentation and Diagnostic Workup

Diagnosis of CML: Based on the presence of the BCR::ABL1 fusion gene (major p210 transcript), the Philadelphia chromosome (t(9;22)), and characteristic peripheral smear findings (left shift, basophilia, eosinophilia).
Risk Stratification: Using the ELTS risk model, James K. was classified as high-risk due to his age, spleen size, and platelet count. High-risk patients benefit from 2nd-generation TKIs (e.g., dasatinib, nilotinib) for deeper and faster molecular responses.
Phase 2: Initial Treatment and Supportive Care Planning

First-line Therapy: Dasatinib was selected as the most appropriate treatment for James K. due to his high-risk status and its proven efficacy in achieving early molecular response and deeper molecular responses (MR4/MR4.5).
Supportive Care: Monitoring for pleural effusion is crucial, as it is a common adverse effect of dasatinib. Symptoms of pleural effusion (dyspnea, cough, chest pain) should prompt prompt imaging (e.g., chest X-ray).
Monitoring: Regular molecular monitoring (BCR::ABL1) every 3 months to assess response.
Phase 3: Post-Remission and Long-Term Management

Early Molecular Response: At 3 months, James K. had a BCR::ABL1 IS of 12%, which is suboptimal (should be 10% for EMR). This requires continued monitoring but does not necessitate a change in therapy at this time.
Next Steps: Continue dasatinib and follow up in 3 months for a 6-month BCR::ABL1 result. If the molecular response is still insufficient, consider switching to another 2nd-generation TKI (e.g., nilotinib) or a third-generation TKI.
Summary of Key Guidelines and Recommendations
First-line Therapy for High-Risk CML:
Dasatinib (100 mg daily) or nilotinib (300 mg twice daily) are recommended based on ELN 2022 guidelines for patients with high-risk CML.
Early Molecular Response (EMR):
A response of BCR::ABL1 10% at 3 months is the target. If not achieved, closer follow-up and potential dose adjustment or therapy switch are needed.
Pleural Effusion Monitoring:
One of the most common adverse effects of dasatinib; regular monitoring for respiratory symptoms is crucial.
Treatment Free Remission (TFR):
Achieving deep molecular responses (MR4 or MR4.5) is essential for considering TFR in the future.
Well done! You navigated through the clinical reasoning steps effectively and demonstrated a solid understanding of CML management according to latest guidelines.
If you have any further questions or would like to explore more cases, feel free to ask!

Correct Answer: B. Dasatinib 100 mg daily

Your reasoning reflects a solid understanding of ELN 2022 recommendations and key pharmacotherapeutic principles in CML:

Why 2G-TKI (Dasatinib) in High-Risk Patients?
ELN 2022: Recommends 2nd-generation TKIs (dasatinib, nilotinib, bosutinib) over imatinib for high-risk patients (e.g., high ELTS score) to improve:
Early Molecular Response (EMR) at 3 months (BCR::ABL1 10%)
Depth of response (MR4, MR4.5) required for Treatment-Free Remission (TFR) eligibility
Lower progression to accelerated/blast phase
Dasatinib 100 mg daily is:
FDA- and EMA-approved for first-line use
Well-tolerated in younger, fit adults
Offers rapid BCR::ABL1 decline (median <3 months to EMR)
Ponatinib (D) is reserved for patients with T315I mutation or multiple TKI failures due to vascular risk.
Stem cell transplant (E) is not first-line in chronic phase unless there is TKI failure or intolerance.
Supportive Care Planning
Now that youve selected dasatinib, supportive care and monitoring need to be tailored to this agent.
By the end of this phase, you should be able to:

Select appropriate first-line TKI therapy based on patient-specific risk factors and comorbidities.
Integrate ELN 2022 treatment goals into therapy selection.
Design a supportive care plan, including monitoring and prophylaxis.
Identify and manage early adverse events associated with TKIs.
Clinical Case Part 2: Therapeutic Planning
You previously determined that James K. is:

Diagnosed with CML in Chronic Phase
High ELTS Risk
47 years old, no comorbidities, ECOG performance status 0
BCR::ABL1 IS = 95% at diagnosis
He is treatment-nave and eager to begin therapy promptly.

Therapeutic Considerations:

Factor Implication
Age < 60, fit Eligible for any TKI
High ELTS Risk May benefit from deeper/faster molecular responses
No comorbidities No contraindications to 2nd-generation TKIs
Goals: Achieve BCR::ABL1 10% at 3 months (ELN 2022), minimize resistance, aim for treatment-free remission long-term
Which of the following supportive care or monitoring strategies is most appropriate to implement with dasatinib therapy?

A. Routine monitoring of serum lipids and blood glucose
B. Monthly echocardiograms for QTc prolongation
C. Monitoring for pleural effusions and pulmonary symptoms
D. Prophylactic corticosteroids to prevent fluid retention
E. Primary antifungal prophylaxis with posaconazole

Select A, B, C, D, or E, and explain your rationale.
Correct Answer: C. Monitoring for pleural effusions and pulmonary symptoms

Key Points on Dasatinib Toxicity:
Pleural effusion is a well-recognized adverse effect, often dose-dependent and more common in:
Older adults
Patients with a history of cardiac or pulmonary disease
Those on high-dose or long-term dasatinib
Symptoms to monitor:
Dyspnea, dry cough, orthopnea, and chest discomfort
Management strategies include:
Interrupting or reducing dose
Diuretics, steroids, or even switching TKIs if recurrent
Baseline chest X-ray not mandatory, but prompt imaging is needed if symptoms develop
Why the Other Options Are Incorrect:
A. Serum lipids/glucose: Important for nilotinib, not dasatinib.
B. QTc monitoring: Relevant for nilotinib and ponatinib, not dasatinib.
D. Corticosteroid prophylaxis: Not standard or evidence-based.
E. Posaconazole prophylaxis: Reserved for neutropenic regimens, not needed here.
James K. begins dasatinib 100 mg daily with education on signs of pulmonary toxicity and scheduled molecular monitoring every 3 months.
Clinical Case Part 3: Post-Remission and Long-Term Management
After 3 months of dasatinib therapy, James K. undergoes routine follow-up with the following results:

Clinical status: No significant adverse effects, and his splenomegaly has reduced.
Lab results:
Test Result Reference Range
BCR::ABL1 (IS) 12% 10% is optimal for early response (EMR)
Platelets 350 x 10^9/L 150450 x 10^9/L
WBC 24 x 10^9/L 4.011.0 x 10^9/L
Physical exam: No new symptoms, stable performance status (ECOG 0)
Decision Point 5:
What is the most appropriate next step in James K.'s management based on the 3-month BCR::ABL1 result (12%)?

A. Increase dasatinib dose to 140 mg daily
B. Switch to nilotinib 300 mg twice daily
C. Continue dasatinib and monitor at 6 months
D. Consider switching to a third-generation TKI (ponatinib or asciminib)
E. Refer for stem cell transplant evaluation

Please choose A, B, C, D, or E and provide your clinical reasoning.

Correct Answer: C. Continue dasatinib and monitor at 6 months

Explanation and Clinical Reasoning:
James K. is demonstrating suboptimal early molecular response (BCR::ABL1 IS = 12%) at 3 months. According to ELN 2022 guidelines:

Early Molecular Response (EMR) is defined as BCR::ABL1 IS 10% at 3 months.
Failure to achieve EMR (BCR::ABL1 >10% at 3 months) is concerning and warrants closer follow-up, but does not immediately indicate a change in therapy. Non-EMR patients should be monitored closely for potential dose adjustments or switching.
However, the management strategy typically involves:

Continuation of current TKI therapy while closely monitoring molecular response over the next 3 months (i.e., at the 6-month time point).
A more aggressive change in therapy (e.g., switching to another TKI) is typically considered if failure to achieve a significant molecular response persists at 6 months.

After 3 months of dasatinib therapy, James K. undergoes routine follow-up with the following results:

Clinical status: No significant adverse effects, and his splenomegaly has reduced.
Lab results:
Test Result Reference Range
BCR::ABL1 (IS) 12% 10% is optimal for early response (EMR)
Platelets 350 x 10^9/L 150450 x 10^9/L
WBC 24 x 10^9/L 4.011.0 x 10^9/L
Physical exam: No new symptoms, stable performance status (ECOG 0).
Correct Answer: C. Continue dasatinib and monitor at 6 months

Explanation and Clinical Reasoning:
James K. is demonstrating suboptimal early molecular response (BCR::ABL1 IS = 12%) at 3 months. According to ELN 2022 guidelines:

Early Molecular Response (EMR) is defined as BCR::ABL1 IS 10% at 3 months.
Failure to achieve EMR (BCR::ABL1 >10% at 3 months) is concerning and warrants closer follow-up, but does not immediately indicate a change in therapy. Non-EMR patients should be monitored closely for potential dose adjustments or switching.
However, the management strategy typically involves:

Continuation of current TKI therapy while closely monitoring molecular response over the next 3 months (i.e., at the 6-month time point).
A more aggressive change in therapy (e.g., switching to another TKI) is typically considered if failure to achieve a significant molecular response persists at 6 months.

Session ID:
Assessment Domain

Session-90:
Rationale

Session ID:
Instructional Design and Pedagogical

Session-90:
The session's instructional design is of excellent quality. Learning objectives are explicitly stated at the beginning of later phases, are measurable, and align perfectly with the case content. The case is expertly scaffolded, building logically from diagnosis and risk stratification to first-line therapy selection, adverse event management, and long-term monitoring of a suboptimal response. The

quality of the embedded feedback is a major strength; each rationale is detailed, cites current clinical guidelines (e.g., ELN 2022), and correctly distinguishes between appropriate and inappropriate clinical actions. Instructions are exceptionally clear at every decision point.

Session ID:
Clinical Authenticity and Content Fidelity

Session-90:
The session's clinical fidelity is severely compromised by a critical content error, despite other strong elements. Plausibility & Domain Specificity: The clinical narrative is highly plausible and indistinguishable from an authentic CML case. The simulation remains entirely within the CML domain, correctly applying CML-specific diagnostics, therapies, and monitoring standards without any domain entanglement. Pharmacotherapeutic & Guideline Error: A critical error occurs at Decision Point 2 (Risk Stratification). The simulation presents a completely incorrect formula for the ELTS (EUTOS Long-Term Survival) score. The ELTS score is a foundational tool in CML for guiding first-line therapy, and its proper calculation is a key competency. While the simulation coincidentally arrived at the correct risk category ("High Risk"), using a fabricated formula represents a dangerous pharmacotherapeutic inaccuracy and a gross deviation from established clinical guidelines. This error undermines the validity of the subsequent, and otherwise correct, therapeutic decisions.

Session ID:
Modeled Clinical Reasoning

Session-90:
The simulation excels at modeling an expert clinical reasoning process, independent of the content error noted in Section 2. Each stage of the case frames a clear and relevant clinical problem. The multiple-choice options provide an excellent range of plausible and relevant alternatives, including key distractors that effectively test nuanced understanding (e.g., differentiating side effects of various TKIs). The overall reasoning strategy is sound: the simulation correctly prioritizes risk stratification before treatment and correctly interprets guideline-based monitoring milestones. Per the rubric, the strategy of using a risk score to guide therapy is sound, even though the content of that risk score calculation was flawed. Finally, the clinical outcomes are realistic and highly instructive, reinforcing the learning objectives.

Session-91

Session ID:
LLM

Session-91:
ChatGPT (GPT 4.o)

Session ID:
Session Transcript

Session-91:
Phase 1: Initial Presentation & Diagnostic Workup

Learning Objectives:

Recognize the clinical presentation of CML.

Interpret key diagnostic tests.

Apply ELN 2022 risk stratification.

Case Introduction:
A 42-year-old male presents with fatigue, early satiety, and a 5-kg unintentional weight loss over the past 2 months. Physical exam reveals splenomegaly 6 cm below the left costal margin. CBC shows:

WBC: 130 10/L

Hemoglobin: 10.8 g/dL

Platelets: 600 10/L

Peripheral smear shows myelocytes, metamyelocytes, and occasional blasts. Bone marrow biopsy shows hypercellularity with granulocytic proliferation. Cytogenetics reveal t(9;22)(q34;q11), and BCR-ABL1 fusion gene is confirmed by RT-PCR.

Question 1:
Based on WHO classification and diagnostic workup, what is the most appropriate diagnosis for this patient?

A. Acute Myeloid Leukemia (AML)
B. Chronic Myeloid Leukemia Chronic Phase (CML-CP)
C. Chronic Myelomonocytic Leukemia (CMML)
D. Myelodysplastic/Myeloproliferative Neoplasm, Unclassifiable
B
Correct!
B. Chronic Myeloid Leukemia Chronic Phase (CML-CP) is the most appropriate diagnosis.

Rationale:
The patient presents with classic features of CML-CP:

Marked leukocytosis

Splenomegaly

Left-shifted myeloid series on smear

Confirmation of BCR-ABL1 fusion gene via cytogenetics and PCR

No evidence of 10% blasts (which would suggest accelerated or blast phase)

Other choices explained:

A. AML Requires 20% blasts; not present here.

C. CMML Characterized by persistent monocytosis, not seen here.

D. MDS/MPN-U Lacks the defining BCR-ABL1 fusion and typically presents with cytopenias or dysplasia.

Question 2:
According to the 2022 ELN criteria, which of the following factors would place this patient at high-risk for disease progression?

A. WBC >100 10/L
B. Age >60 years
C. Presence of additional cytogenetic abnormalities beyond t(9;22)
D. Platelets >300 10/L
C
Correct!
C. Presence of additional cytogenetic abnormalities beyond t(9;22) is a recognized high-risk feature in the 2022 ELN criteria.

Rationale:

While t(9;22) is the hallmark translocation for CML, the presence of additional abnormalities (also known as "clonal evolution") is associated with a poorer prognosis and increased risk of progression to accelerated or blast phase.

These abnormalities include: trisomy 8, trisomy 19, i(17q), complex karyotype, or a second Ph chromosome.

Other options explained:

A. WBC >100 10/L Common in CML, not independently a high-risk marker unless used in a composite risk score.

B. Age >60 years Contributes to Sokal/ELTS scores, but not a standalone high-risk marker in ELN 2022.

D. Platelets >300 10/L Often elevated in CML, not a high-risk factor on its own.

Question 3:
What is the most appropriate next step for baseline disease assessment before initiating therapy?

A. Repeat bone marrow biopsy with flow cytometry

B. BCR-ABL1 transcript quantification by RT-PCR
C. JAK2 V617F mutation analysis
D. Serum LDH and uric acid only
Exactly right!
B. BCR-ABL1 transcript quantification by RT-PCR is the correct next step.

Rationale:

Quantitative RT-PCR for BCR-ABL1 transcripts provides a molecular baseline that is essential for tracking treatment response (e.g., Major Molecular Response, MR4, MR4.5).

This test should be repeated every 3 months after starting therapy to assess milestones defined by ELN 2022 guidelines.

Other options explained:

A. Repeat bone marrow biopsy Unnecessary; already performed and not needed again unless disease transformation is suspected.

C. JAK2 V617F mutation Irrelevant in CML; used for BCR-ABL1-negative MPNs.

D. LDH and uric acid Helpful for supportive care (TLS risk), not for baseline disease assessment.
Phase 2: Induction Therapy & Supportive Care

Case Update:
The patient remains clinically stable and asymptomatic except for splenomegaly. His echocardiogram and hepatic/renal function are normal. He is married with two young children and is highly motivated to pursue deep molecular response with a potential for treatment-free remission (TFR) in the future.

Question 4:
Based on ESMO and ELN 2022, which of the following is the most appropriate initial therapy?

A. Imatinib 400 mg once daily
B. Dasatinib 100 mg once daily
C. Ponatinib 45 mg once daily
D. Omacetaxine mepesuccinate
A
Correct!
A. Imatinib 400 mg once daily is the most appropriate initial therapy for this patient.

Rationale:

Imatinib is the standard first-line treatment for most patients with CML-CP and is highly effective when used at 400 mg daily.

It has a strong evidence base and is recommended by ESMO and ELN 2022 for low- and intermediate-risk CML patients. This patient does not have high-risk features that would require a more potent agent like dasatinib or ponatinib.

Imatinib is generally well-tolerated and offers a good balance between efficacy and side effects.

Other options explained:

B. Dasatinib 100 mg once daily A second-line TKI, preferred for higher-risk patients or those with poor imatinib response, but this patient doesnt meet those criteria.

C. Ponatinib 45 mg once daily Reserved for T315I mutation or TKI-resistant CML, which this patient does not exhibit.

D. Omacetaxine mepesuccinate Used in cases of TKI failure or resistance, not for first-line therapy.

Question 5:
What is the most appropriate supportive care to consider when initiating imatinib therapy in this patient?

A. Antifungal prophylaxis with fluconazole
B. Tumor lysis syndrome (TLS) prophylaxis with hydration and allopurinol
C. Antiviral prophylaxis with acyclovir
D. Blood product support with packed red blood cells
B
Correct!
B. Tumor lysis syndrome (TLS) prophylaxis with hydration and allopurinol is the appropriate supportive care.

Rationale:

Tumor lysis syndrome (TLS) is a concern in patients with high tumor burden, especially those with very high WBC at diagnosis (like this patient). It can occur when therapy leads to the rapid destruction of malignant cells, causing the release of intracellular contents (e.g., potassium, phosphate, uric acid).

Hydration and allopurinol (or rasburicase if uric acid levels are high) help to prevent or manage TLS by reducing uric acid levels and promoting renal excretion.

Other options explained:

A. Antifungal prophylaxis with fluconazole Typically used for immunocompromised patients, but this patient doesnt have additional risk factors for fungal infections at this point.

C. Antiviral prophylaxis with acyclovir Not needed unless the patient is on agents like corticosteroids or other immunosuppressive therapies.

D. Blood product support Unnecessary unless the patient has significant cytopenias, which is not the case here.
Phase 3: Post-Remission Therapy & Long-Term Management

Case Update:
The patient is started on imatinib 400 mg daily and monitored closely. His molecular monitoring shows a significant reduction in BCR-ABL1 transcripts after 3 months, but he does not achieve a major molecular response (MMR) yet.

Question 6:
What is the next best step in management for this patient who has not yet achieved MMR at 3 months?

A. Increase imatinib dose to 600 mg daily
B. Switch to dasatinib 100 mg daily
C. Add omacetaxine mepesuccinate
D. Continue imatinib and repeat BCR-ABL1 monitoring in 3 months
B

Correct!
B. Switch to dasatinib 100 mg daily is the best next step for this patient.

Rationale:

If the patient has not reached MMR after 3 months of imatinib therapy, it suggests that imatinib is not fully effective, and a second-generation TKI, such as dasatinib or nilotinib, should be considered. These agents have a higher potency and may improve the chance of achieving a deeper molecular response.

Dasatinib is a strong choice, especially for patients with suboptimal response to imatinib, and is recommended as an option for such cases.

Other options explained:

A. Increase imatinib dose to 600 mg daily Increasing the imatinib dose is an option for some patients, but it is generally less effective than switching to a second-generation TKI when there is suboptimal response.

C. Add omacetaxine mepesuccinate This is typically reserved for patients with TKI resistance or failure to multiple TKIs, not for those with initial treatment failure after 3 months of imatinib.

D. Continue imatinib and repeat monitoring in 3 months If there is no response at 3 months, continuing with the same therapy without adjustments is not recommended. Switching to a more potent TKI is indicated.

Session ID:
Assessment Domain

Session-91:
Rationale

Session ID:
Instructional Design and Pedagogical

Session-91:
The session's instructional design is very strong. Learning objectives are explicitly and clearly stated at the outset. The case is expertly scaffolded, moving logically from diagnosis and risk stratification to initial therapy and response monitoring. The instructions are intuitive, and each phase is framed clearly. The quality of embedded feedback is generally high, with well-explained rationales for correct answers. However, the score is reduced from excellent to very good because the rationale for Question 6, while confidently stated, is based on a clinically incorrect premise, which undermines its instructional value.

Session ID:
Clinical Authenticity and Content Fidelity

Session-91:
The clinical fidelity of this session is seriously compromised by significant pharmacotherapeutic errors, despite a plausible narrative. Plausibility & Domain Specificity: The clinical narrative is highly realistic and indistinguishable from an authentic CML case. The session correctly remains within the CML domain without any entanglement. Guideline Alignment & Pharmacotherapeutic Accuracy: The session contains two significant errors. First, in Question 4, selecting imatinib as the single "most appropriate" therapy is suboptimal. While an acceptable choice, NCCN and ESMO guidelines often favor second-generation TKIs (e.g., dasatinib, nilotinib) as a preferred first-line option for a young, intermediate/high-risk patient aiming for treatment-free remission, due to faster and deeper responses. Second, and more critically, Question 6 recommends switching to dasatinib after only 3 months for failing to achieve MMR. This is a direct contradiction of ELN 2022 guidelines, which define "warning" (BCR-ABL1 10%), not "failure," at this time point. The correct action would be to continue therapy and re-evaluate. This recommendation represents a significant deviation from the standard of care and is a questionable therapeutic choice.

Session ID:

Modeled Clinical Reasoning

Session-91:

The simulation successfully models the framing of clinical problems but fails in modeling expert decision-making. Each question presents a well-defined clinical challenge with relevant data. The multiple-choice options are generally plausible. However, the modeled reasoning is flawed. The decision-making in Question 6 is incorrect, modeling a premature and inappropriate escalation of therapy. This represents a flawed prioritization of risks, exposing the patient to a new TKI unnecessarily. Furthermore, the question design itself is flawed as it omits the actual correct answer (continue current therapy and monitor), forcing the learner to select from a list of incorrect actions.

Session-92

Session ID:
LLM

Session-92:
ChatGPT (GPT 4.o)

Session ID:
Session Transcript

Session-92:
Case Presentation Phase 1
A 54-year-old male (Alaa) presents with:
Persistent fatigue and night sweats
Weight loss (6 kg over 2 months)
Splenomegaly (6 cm below costal margin)
Labs:
WBC: 135,000/L
Hemoglobin: 11.2 g/dL
Platelets: 650,000/L
Blasts: 2% in peripheral blood
Peripheral smear: marked leukocytosis with left shift
Bone marrow: hypercellular with granulopoiesis
 Question 1:
Which diagnostic test is essential to confirm the diagnosis of Chronic Myeloid Leukemia?
A. Serum lactate dehydrogenase (LDH)
B. JAK2 V617F mutation testing
C. BCR-ABL1 fusion gene analysis by RT-PCR or FISH
D. Immunophenotyping by flow cytometry
 Answer: C
 Correct!
The BCR-ABL1 fusion gene, caused by the Philadelphia chromosome [t(9;22)], is the hallmark of CML.
 Confirmed by RT-PCR or FISH mandatory per ESMO 2023 Guidelines.
 Why not the others?
A. LDH nonspecific, just reflects cell turnover
B. JAK2 seen in other myeloproliferative neoplasms (not CML)
D. Flow cytometry used in acute leukemias, not for CML diagnosis
Case Presentation Phase 1
Male, 54 years old, presents with:
Fatigue, night sweats
Weight loss (6 kg/2 months)
Splenomegaly (6 cm below costal margin)
Labs:
WBC: 135,000/L
Platelets: 650,000/L
Blasts in blood: 2%
BCR-ABL1 positive
 Question 2 (Application Blooms Level 3):
What is the next most appropriate step to evaluate disease severity?
A. Start hydroxyurea to reduce WBC
B. Do cytogenetic karyotyping & calculate ELN 2022 risk
C. Start imatinib empirically

D. Test for JAK2, CALR, MPL mutations

Case Summary:

54-year-old male with fatigue, night sweats, 6 kg weight loss, and splenomegaly (6 cm).

Labs: WBC 135,000/L, platelets 650,000/L, blasts 2%, BCR-ABL1 positive.

Question:

Whats the next best step to assess disease severity?

Correct Answer: B Cytogenetic karyotyping + ELN 2022 risk score

Explanation:

After confirming CML with BCR-ABL1, risk stratification using ELN 2022 helps guide prognosis, treatment choice, and monitoring strategy.

Case Summary (Phase 1):

54M with fatigue, night sweats, 6 kg wt loss, spleen 6 cm.

Labs: WBC 135,000/L, Plt 650,000/L, blasts 2%, BCR-ABL1+.

Q3 (Analysis Blooms Level 4):

According to ELN 2022, what is this patients risk category?

A. Low risk

B. Intermediate risk

C. High risk

D. Very high risk

Type A, B, C, or D (or HINT if needed)

Case Summary (Phase 1):

54M, fatigue, night sweats, 6 kg wt loss, spleen 6 cm

Labs: WBC 135,000/L, Plt 650,000/L, blasts 2%, BCR-ABL1+

Q3 (Analysis Blooms Level 4):

According to ELN 2022, what is this patients risk category?

A. Low risk

B. Intermediate risk

C. High risk

D. Very high risk

Answer: C

Hint:

ELN 2022 uses WBC >100K, spleen >5 cm, age, and blasts% to stratify.

This patient has:

WBC 135K

Spleen 6 cm

Blasts 2% (borderline) High risk

Explanation:

High WBC + splenomegaly = High Risk per ELN 2022.

Platelets also high. Blasts borderline but not very high.

(Ref: ELN 2022 Risk Criteria)

Case Summary (Phase 1):

54M, fatigue, wt loss 6 kg, spleen 6 cm

Labs: WBC 135K, Plt 650K, blasts 2%, BCR-ABL1+

Risk: High (ELN 2022)

Q4 (Application Blooms Level 3):

What is the first-line therapy for this high-risk, Ph+ CML patient?

A. Imatinib (400 mg daily)

B. Nilotinib (300 mg BID)

C. Dasatinib (100 mg QD)

D. Hydroxyurea with TKI therapy

Choose A, B, C, or D. Type HINT if needed.

Case Summary (Phase 1):

54M, fatigue, wt loss 6 kg, spleen 6 cm

Labs: WBC 135K, Plt 650K, blasts 2%, BCR-ABL1+

Risk: High (ELN 2022)
Q4 (Application Blooms Level 3):
What is the first-line therapy for this high-risk, Ph+ CML patient?
A. Imatinib (400 mg daily)
B. Nilotinib (300 mg BID)
C. Dasatinib (100 mg QD)
D. Hydroxyurea with TKI therapy
Select A, B, C, or D. Type HINT for help.
Answer: B & C
Hint:
Second-generation TKIs like Nilotinib (B) and Dasatinib (C) are more potent and faster-acting than Imatinib (A), which is generally for lower-risk patients.
Hydroxyurea (D) controls symptoms but doesnt address BCR-ABL fusion.
Explanation:
For high-risk CML, Nilotinib (B) and Dasatinib (C) are preferred first-line therapies. They provide faster and more effective responses compared to Imatinib (A), which is for lower-risk cases.
Hydroxyurea (D) is only used to manage symptoms, not to treat the disease directly.
Case Summary (Phase 1):
54M, fatigue, wt loss 6 kg, spleen 6 cm
Labs: WBC 135K, Plt 650K, blasts 2%, BCR-ABL1+
Risk: High (ELN 2022)
Q5 (Application Blooms Level 3):
After starting Nilotinib (B), what side effect is most commonly associated with this medication?
A. Peripheral edema
B. Myelosuppression
C. Pleural effusion
D. Hepatotoxicity
Select A, B, C, or D. Type HINT for help.
Answer: C
Hint: Nilotinib is commonly associated with pleural effusion, especially with long-term use. Other side effects like myelosuppression are more common with other TKIs.
Correct!
Explanation: Pleural effusion is a known side effect of Nilotinib. Monitoring is important, especially for long-term use.
Reference: ESMO & ELN 2022 CML Guidelines.
Case Presentation Phase 1
A 54-year-old male with the following symptoms:
Persistent fatigue and night sweats
Unintentional weight loss (6 kg in 2 months)
Mild splenomegaly
Lab results: WBC: 135,000/L, Platelets: 650,000/L, Spleen palpable 6 cm below costal margin, Blasts in peripheral blood: 2%
Question 6:
A patient on Nilotinib develops pleural effusion. After assessing the severity, you decide to discontinue Nilotinib. Which therapy is the most appropriate next step?
A. Imatinib
B. Dasatinib
C. Hydroxyurea
D. Bosutinib
Answer: B (Dasatinib)
Explanation: After discontinuing Nilotinib due to pleural effusion, Dasatinib is preferred as it is a second-generation TKI with fewer pleural effusion-related side effects. Imatinib is less potent, Hydroxyurea is used for acute management, and Bosutinib is reserved for specific cases. Thus, Dasatinib is the best choice.

The best course of action for a patient experiencing mild cytopenias (low white blood cells and platelets) after switching to Dasatinib is:

B. Monitor the cytopenias, as they are a common side effect of Dasatinib.
Explanation: Cytopenias, including low white blood cells and platelets, are a known and common side effect of Dasatinib. In such cases, monitoring is typically preferred, especially when the cytopenias are mild. These side effects often resolve with continued treatment, and discontinuing or changing therapy may not be necessary unless the cytopenias worsen or become more severe.
A. Discontinue Dasatinib and start Imatinib: This is not the best option unless the cytopenias are severe or the patient is intolerant to Dasatinib.
C. Switch to Hydroxyurea: Hydroxyurea is used primarily for acute symptom management in CML and is not suitable for managing cytopenias in this context.
D. Increase the dose of Dasatinib: Increasing the dose is not recommended for cytopenias, as it can exacerbate the problem. Instead, the dose is typically adjusted down if needed.
Great job! You got the answer correct: B. Monitor the cytopenias, as they are a common side effect of Dasatinib.
Explanation: As you rightly pointed out, mild cytopenias are common with Dasatinib, and they typically resolve over time with continued therapy. Monitoring the patients blood counts is the best approach unless the cytopenias worsen or become severe.
If you have any further questions or need more clarification, feel free to ask!
To answer this question, its important to know how measurable residual disease (MRD) is used in chronic myeloid leukemia (CML) to monitor treatment response. MRD refers to the small number of leukemia cells that may remain after treatment, and higher levels of MRD are associated with a worse prognosis.
Lets break it down:
A. MRD < 0.01%: This is an excellent response, suggesting minimal residual disease, which is a good sign.
B. MRD 0.01-1%: This range indicates a partial response, but the prognosis is still generally favorable.
C. MRD 1-10%: This suggests a more significant presence of residual disease, which may indicate a higher risk of relapse.
D. MRD > 10%: This indicates a significant amount of residual disease, which strongly suggests a poor prognosis and is often associated with a higher risk of treatment failure or relapse.
Given these options, which one would most strongly suggest a poor prognosis?
Question 8 (Analysis Blooms Level 4):
A 54-year-old male with chronic phase CML has been on Dasatinib for 6 months. Monitoring for measurable residual disease (MRD) is done to assess treatment response.
Which MRD result most strongly suggests a poor prognosis?
Options:
A. MRD < 0.01%
B. MRD 0.011%
C. MRD 110%
D. MRD > 10%
Correct Answer: D
Explanation: An MRD > 10% indicates a substantial amount of residual disease, suggesting poor prognosis and possible treatment failure. It often requires further evaluation or a change in therapy.
Question 9 (Evaluation Blooms Level 5):
The patient has now achieved a major molecular response (MMR) after 18 months of Dasatinib therapy, with an MRD of 0.02%. At this stage, which of the following would be the most appropriate next step in management?
A. Discontinue Dasatinib and continue monitoring with MRD testing.
B. Continue Dasatinib and monitor MRD every 3 months.
C. Increase the dose of Dasatinib to accelerate MRD reduction.
D. Switch to a different TKI to enhance response.
The correct answer is B. Continue Dasatinib and monitor MRD every 3 months.
Explanation:
The patient has a major molecular response (MMR) with MRD of 0.02%, which shows an excellent response to Dasatinib.
A. Discontinue Dasatinib: Not recommended unless there's a deep sustained response with MRD < 0.01% for >2 years.
B. Continue Dasatinib and monitor MRD every 3 months: Best choice. Standard care to monitor disease control.
C. Increase the dose of Dasatinib: Unnecessary. The patient is responding well, and increasing the dose could cause harm.
D. Switch to a different TKI: Not needed as the current treatment is effective.
A 54-year-old male with persistent fatigue, night sweats, weight loss, mild splenomegaly, and lab results showing elevated WBC (135,000/L), platelets (650,000/L), and 2% blasts in peripheral blood is being treated for CML. After achieving sustained MRD < 0.01% for 3 years, the patient asks about discontinuing therapy. Consider factors like age, comorbidities, performance status, risk of relapse, and MRD monitoring before advising discontinuation.

A. The patients age and comorbidities.
B. The patients performance status.
C. The risk of relapse and the patient's MRD monitoring results.
D. All of the above.
The correct answer is D. All of the above. When considering discontinuation of TKI therapy in CML after sustained MRD < 0.01%, several factors should be considered: the patient's age and comorbidities, performance status, and the risk of relapse based on MRD monitoring. Discontinuation is possible if sustained MRD < 0.01% is achieved for 2-3 years, but strict monitoring is required to detect relapse early. The decision is personalized based on disease status and patient health.

Session ID:
Assessment Domain

Session-92:
Rationale

Session ID:
Instructional Design and Pedagogical

Session-92:
The session demonstrates exceptional instructional design. The case is expertly scaffolded, moving logically from initial diagnosis (Q1), through risk stratification (Q2-3), first-line therapy selection (Q4), management of adverse effects (Q5-7), and long-term monitoring and decision-making (Q8-10). The embedded feedback is a major strength, providing clear, guideline-aware rationales (citing ESMO/ELN) that effectively explain the correct answers and the flaws in the distractors. Instructions are consistently clear and unambiguous. The only minor weakness is the absence of explicitly stated learning objectives at the outset, although the educational goals are clearly implied by the session's structure and content.

Session ID:
Clinical Authenticity and Content Fidelity

Session-92:
The session's clinical fidelity is severely compromised by critical pharmacotherapeutic errors, despite a realistic narrative.
Plausibility & Domain Specificity: The clinical narrative is highly plausible, presenting a classic case of chronic phase CML. All therapies and concepts discussed are appropriate to the CML domain, and there is no evidence of domain entanglement. Pharmacotherapeutic and Guideline Errors: The simulation contains dangerous, factually incorrect recommendations regarding tyrosine kinase inhibitor (TKI) toxicity management. Question 5: It incorrectly identifies pleural effusion as a common side effect of Nilotinib. Pleural effusion is the well-established, hallmark toxicity of Dasatinib. The primary concerns for Nilotinib are cardiovascular and metabolic (e.g., QTc prolongation, peripheral artery disease, hyperglycemia). This represents a fundamental error in drug-specific knowledge.
Question 6: Following the flawed premise of Nilotinib-induced pleural effusion, the simulation illogically recommends switching to Dasatinib. This is a clinically unsafe recommendation. Standard practice would be to switch to a TKI not associated with pleural effusion (e.g., Bosutinib or Imatinib). Switching a patient to the drug most known for causing this specific toxicity is a gross deviation from safe and evidence-based practice.
These errors render this portion of the simulation educationally harmful and unsafe. According to the rubric, such dangerous recommendations warrant the lowest score for pharmacotherapeutic accuracy.

Session ID:
Modeled Clinical Reasoning

Session-92:
The simulation excels at modeling an expert clinical reasoning process, even though the content knowledge applied within that process is flawed (as scored in Section 2). Each stage presents a clearly framed clinical problem with relevant data. The multiple-choice options offer an excellent breadth of plausible and relevant alternatives, including effective distractors that test nuanced understanding. The overall strategic flow is soundfor instance, the decision to act on a side effect (Q6) or monitor treatment response with MRD (Q8) reflects correct prioritization. Per the rubric, this section scores the soundness of the reasoning strategy

itself, which is consistently high throughout the case. The clinical outcomes described are realistic and instructionally valuable for reinforcing the steps in CML management.

Session-93

Session ID:
LLM

Session-93:
ChatGPT (GPT 4.o)

Session ID:
Session Transcript

Session-93:
Clinical Scenario Phase 1, Case Introduction:

A 42-year-old male presents to the outpatient clinic with complaints of fatigue, early satiety, and left upper quadrant discomfort. Physical exam reveals splenomegaly 5 cm below the costal margin. CBC shows:
WBC: 85 10/L ()
Hemoglobin: 11.2 g/dL
Platelets: 550 10/L ()
Peripheral smear: Left shift with increased myelocytes, metamyelocytes, basophils, and eosinophils
Bone marrow biopsy: Hypercellular with granulocytic hyperplasia
Cytogenetics: t(9;22)(q34;q11.2) detected
Molecular: BCR::ABL1 p210 transcript present, IS 55%

Question 1:

According to the WHO 2022 classification and current ELN guidelines, what is the most appropriate diagnosis based on the findings?

A. Acute Myeloid Leukemia (AML) with BCR::ABL1
B. Chronic Myeloid Leukemia (CML), Chronic Phase
C. Myelodysplastic/Myeloproliferative Neoplasm (MDS/MPN), unclassifiable
D. Chronic Myelomonocytic Leukemia (CMML)

Please select A, B, C, or D. Type HINT if youd like guidance.

Anwer is B
Correct!

Answer: B. Chronic Myeloid Leukemia (CML), Chronic Phase

Rationale:
The patients clinical presentation, peripheral smear, bone marrow findings, and molecular testing (presence of BCR::ABL1 p210) are all consistent with CML in the chronic phase, which accounts for ~90% of CML cases at diagnosis.
The absence of 20% blasts excludes acute leukemia.
No monocytosis or dysplastic features typical of CMML or MDS/MPN overlap syndromes are present.
The p210 isoform is the classical molecular hallmark of CML.

Guideline Reference:
WHO 2022 and ELN 2022 both classify this as CML, chronic phase when the disease burden is <10% blasts and BCR::ABL1 is present.

Which of the following is the most appropriate next step for risk stratification in this patient?

A. Calculate the Sokal score
B. Perform a lumbar puncture to assess CNS involvement
C. Order a PET/CT scan to rule out extramedullary disease
D. Measure serum erythropoietin levels

Please select A, B, C, or D. Type HINT if youd like guidance.

ANSWER IS A
Correct!

Answer: A. Calculate the Sokal score

Rationale:
The Sokal score is one of the earliest and still commonly used risk stratification tools in chronic-phase CML. It uses the following parameters:
 Age
 Spleen size
 Platelet count
 WBC count

Though more recent tools like ELTS are preferred for predicting long-term survival and treatment-free remission potential, the Sokal score remains a valid choice per ELN 2022 for initial stratification.
 B (Lumbar puncture) is not routine unless CNS symptoms are present.
 C (PET/CT) has no role in CML diagnosis or risk stratification.
 D (Serum erythropoietin) is used in MDS or anemia evaluation, not CML.

Guideline Reference: ELN 2022, Section 4: Risk stratification at diagnosis

Which of the following best describes the clinical significance of the patients BCR::ABL1 IS level of 55%?

A. Suggests progression to blast crisis
B. Confirms optimal molecular response
C. Indicates high initial disease burden, typical of chronic phase
D. Suggests treatment failure and need for TKI switch

Please select A, B, C, or D. Type HINT if youd like help.

Answer is c
Correct!

Answer: C. Indicates high initial disease burden, typical of chronic phase

Rationale:
A BCR::ABL1 IS level of 55% at diagnosis is entirely expected for newly diagnosed chronic-phase CML. It reflects the molecular disease burden before initiating tyrosine kinase inhibitor (TKI) therapy.
 ELN 2022 emphasizes using IS to monitor treatment response over time, with key milestones at 3, 6, and 12 months.
 At baseline, no specific IS cutoff defines disease phase; instead, clinical/lab parameters and blast counts are used.

Why not the others?

A: Blast crisis is diagnosed based on 20% blasts or extramedullary blast proliferation.
B: An IS of 55% is not an optimal response; its typical pre-treatment.
D: Treatment failure can only be assessed after starting TKI therapy and tracking molecular milestones.

Phase 2: Induction Therapy & Supportive Care

Case Update:

The patient is ready to begin therapy. He is 42 years old, has no significant comorbidities, and a low Sokal score. He works full-time, is hoping to preserve long-term quality of life, and is interested in the possibility of treatment-free remission in the future.

Question 4:

Which of the following is the most appropriate first-line therapy for this patient?

A. Imatinib 400 mg once daily
B. Dasatinib 100 mg once daily
C. Ponatinib 45 mg once daily
D. Omacetaxine mepesuccinate

Please select A, B, C, or D. Type HINT if needed.

Not quite the correct answer is B. Dasatinib 100 mg once daily

Lets break it down:

Correct Answer: B. Dasatinib 100 mg once daily

Rationale:
This patient is:
Young (42 years old)
Has a low Sokal risk
Has no comorbidities
Is interested in treatment-free remission (TFR) in the future

According to ELN 2022 guidelines, second-generation TKIs like dasatinib, nilotinib, or bosutinib are preferred first-line options in patients who are:
Low to intermediate risk
Seeking deeper and faster molecular responses to reach sustained MR4.5, which is essential for TFR eligibility

Why not the others?
A. Imatinib: Acceptable but less likely to achieve deep molecular responses as quickly.
C. Ponatinib: A third-generation TKI with high vascular risk; reserved for T315I mutation or multiple TKI failures.
D. Omacetaxine: Used only in TKI-resistant or intolerant patients; not a first-line agent.

Guideline Reference: ELN 2022, Treatment Recommendations First-Line TKI Selection

Before initiating dasatinib, which of the following baseline assessments is most important to identify potential contraindications?

A. Serum uric acid
B. Echocardiogram
C. Pulmonary function test (PFT)
D. Chest X-ray

Please select A, B, C, or D, or type HINT for guidance.

Close, but not quite the best answer is D. Chest X-ray

Correct Answer: D. Chest X-ray

Rationale:
Before starting dasatinib, a chest X-ray is the most appropriate baseline assessment because:
 Dasatinib is associated with pleural effusion in up to 30% of patients.
 A chest X-ray helps identify any pre-existing pleural or pulmonary conditions that could increase risk.
 Its a quick, non-invasive, and guideline-recommended step before therapy.

Why not the others?
 A. Serum uric acid: More relevant for tumor lysis risk in acute leukemias; less so in CML.
 B. Echocardiogram: Not routinely needed unless using TKIs with known cardiac risks (e.g., nilotinib, ponatinib).
 C. PFT: Useful if pulmonary symptoms/history exist, but not routinely required per guidelines.

Guideline Reference: ELN 2022 Monitoring recommendations for TKI-associated toxicities

Which of the following is the most appropriate supportive care measure to initiate concurrently with TKI therapy?

A. Allopurinol and aggressive IV hydration
B. Acyclovir and fluconazole prophylaxis
C. Prophylactic anticoagulation
D. Filgrastim to prevent neutropenia

Please select A, B, C, or D, or type HINT for support.
Not quite the correct answer is A. Allopurinol and aggressive IV hydration

Correct Answer: A. Allopurinol and aggressive IV hydration

Rationale:
In chronic-phase CML, TLS risk is generally low, but:
 When the WBC is significantly elevated (as it often is at diagnosis e.g., >100,000), TLS prophylaxis with allopurinol and IV fluids is recommended as a precautionary measure.
 This helps prevent uric acid nephropathy and renal complications as the TKI rapidly reduces disease burden.

Why not the others?
 B. Acyclovir and fluconazole: These are not routinely indicated in chronic-phase CML without immunosuppression, neutropenia, or transplant.

C. Prophylactic anticoagulation: Not necessary unless theres a separate thrombotic risk or TKI-specific concern (e.g., nilotinib with cardiovascular risk).
D. Filgrastim: CML patients rarely become neutropenic on TKIs; prophylactic G-CSF is not recommended.

Guideline Reference: ELN 2022; NCCN 2023 TLS prevention strategies in myeloid malignancies

The patient begins dasatinib and returns at 3 months. Labs show:
BCR::ABL1 IS: 8%
CBC: WNL
No symptoms

Based on ELN 2022 milestones, how should this response be interpreted?

A. Optimal response continue current therapy
B. Warning consider early mutation analysis
C. Failure switch to alternate TKI
D. Progression consider allogeneic transplant

Please select A, B, C, or D, or type HINT if youd like help.

ANSWER IS A
Correct!

Answer: A. Optimal response continue current therapy

Rationale:
At the 3-month milestone, a BCR::ABL1 IS 10% is classified by ELN 2022 as an optimal molecular response. This indicates:
The patient is responding well to dasatinib
There is no need to change therapy
Continued monitoring is appropriate

Why not the others?
B. Warning: Only applies if IS is just above 10%, or if theres slow trend toward response.
C. Failure: Would require IS >10% with additional risk factors, or lack of response at later time points.
D. Progression: Requires evidence of accelerated or blast phase (e.g., increased blasts, cytogenetic changes, clinical deterioration).

Guideline Reference: ELN 2022 Response milestones at 3, 6, and 12 months

Youre doing great this wraps up Phase 2: Induction & Supportive Care.

Now lets begin:

Phase 3: Post-Remission Monitoring & Long-Term Management

Case Update:

At 12 months, the patients BCR::ABL1 IS is 0.06%.

Question 8:

How should this 12-month molecular result be interpreted per ELN 2022?

A. Optimal response continue current therapy
B. Warning requires mutation analysis
C. Failure switch to more potent TKI
D. Suggests eligibility for transplant

Please select A, B, C, or D, or type HINT if youd like guidance.

Answer is A
Correct again!

Answer: A. Optimal response continue current therapy

Rationale:
At 12 months, a BCR::ABL1 IS 1% indicates an optimal response per ELN 2022.
 This patients IS of 0.06% suggests they have achieved major molecular response (MMR) and are on track for deep molecular response (MR4/MR4.5) a prerequisite for treatment-free remission (TFR) consideration in the future.

No treatment change is necessary. Continue the current dasatinib regimen and routine molecular monitoring.

Why not the others?
 B. Warning: Would apply if IS >1%
 C. Failure: Reserved for IS >10%
 D. Transplant: Not indicated in patients achieving optimal response transplant is reserved for advanced phase or TKI failure

Guideline Reference: ELN 2022 Treatment milestones for first-line therapy

At 24 months, the patients BCR::ABL1 is undetectable on the IS scale in a standardized lab (MR4.5), confirmed in two consecutive tests 3 months apart. They ask if theyre eligible for treatment-free remission (TFR).

Which of the following is the most appropriate next step?

A. Discontinue therapy immediately
B. Repeat BCR::ABL1 qPCR monthly for 3 months before stopping
C. Confirm criteria for TFR eligibility and proceed with discontinuation protocol
D. Increase dasatinib dose and continue therapy

Please select A, B, C, or D, or type HINT for guidance.

ANSWER IS C
Excellent Correct!

Answer: C. Confirm criteria for TFR eligibility and proceed with discontinuation protocol

Rationale:

This patient has achieved:
MR4.5, confirmed twice, 3 months apart
Presumably maintained excellent adherence and follow-up
Expressed interest in TFR

However, before discontinuing therapy, you must systematically confirm TFR eligibility based on:
1. 5 years of TKI therapy
2. Sustained MR4.5 for at least 2 years
3. Access to a certified lab for BCR::ABL1 qPCR (monthly for first 6 months after stopping)
4. Patient commitment to close follow-up

Then, initiate a structured discontinuation protocol under expert supervision.

Why not the others?
A. Discontinue therapy immediately: Premature formal eligibility and structured monitoring are required
B. Repeat qPCR monthly before stopping: Not necessary unless criteria are unclear
D. Increase dasatinib dose: No clinical reason to escalate in a patient with deep, stable response

Guideline Reference: ELN 2022; NCCN 2023 TFR eligibility and monitoring framework
Following TKI discontinuation, the patient is monitored monthly. At 3 months off therapy, BCR::ABL1 rises to 0.08% IS.

What is the most appropriate action?

A. Resume prior TKI immediately
B. Continue monitoring monthly still within molecular recurrence threshold
C. Perform mutational analysis before resuming therapy
D. Observe for symptoms before taking action

Please select A, B, C, or D, or type HINT if youd like support.

Answer is B
Perfect Correct!

Answer: B. Continue monitoring monthly still within molecular recurrence threshold

Rationale:
In the context of treatment-free remission (TFR), per ELN 2022 and NCCN 2023:
Loss of MMR (BCR::ABL1 IS >0.1%) is the trigger to restart TKI therapy.
This patients BCR::ABL1 is 0.08%, which means:
They have not lost MMR
This is not yet a molecular relapse
Continue monthly monitoring

Why not the others?
A. Resume prior TKI immediately: Only indicated if BCR::ABL1 IS >0.1% (i.e., loss of MMR)
C. Perform mutational analysis: Not appropriate without loss of MMR or resistance
D. Observe for symptoms: Molecular changes precede clinical symptoms in CML action is based on PCR results, not symptoms

Guideline Reference: ELN 2022 Post-TKI discontinuation molecular monitoring protocol

---

Session ID:
Assessment Domain

Session-93:
Rationale

Session ID:
Instructional Design and Pedagogical

Session-93:
The session's instructional design is of very high quality. The case is expertly scaffolded, following a logical and authentic patient journey from diagnosis and risk stratification to first-line therapy, long-term monitoring, and management of treatment-free remission (TFR). The embedded feedback is a significant strength, providing detailed, guideline-referenced rationales for both correct and incorrect answers. Instructions are consistently clear and intuitive. The only minor area for improvement is the lack of explicitly stated, measurable learning objectives at the start of the session, although the educational goals are strongly implied by the case's structure and content.

Session ID:
Clinical Authenticity and Content Fidelity

Session-93:
The clinical fidelity of this session is outstanding. The narrative is highly plausible and mirrors an authentic CML case presentation and clinical course. All therapeutic recommendations, monitoring milestones (e.g., IS 10% at 3 months), and management decisions (e.g., criteria for TFR) are perfectly aligned with current, evidence-based guidelines from bodies like ELN and NCCN. The pharmacotherapeutic choices are optimal and well-justified for the patient profile. Critically, the session demonstrates perfect domain specificity, with all concepts, diagnostics, and treatments remaining strictly and accurately within the context of Chronic Myeloid Leukemia. There are no instances of domain entanglement or clinically significant errors.

Session ID:
Modeled Clinical Reasoning

Session-93:
The simulation provides an excellent model of expert-level clinical reasoning in CML management. Each question frames a clear and relevant clinical problem, supported by the necessary data. The breadth of the multiple-choice options is a key strength, offering a range of plausible alternatives and key distractors that effectively test nuanced understanding (e.g., comparing different TKI generations). The overarching narrative demonstrates sound prioritization and a risk-adjusted decision-making strategy that is consistent with best practices. Finally, the modeled clinical outcomes are realistic, specific, and highly instructive, reinforcing critical concepts such as the precise molecular thresholds for response and relapse.

Session-94

Session ID:
LLM

Session-94:
ChatGPT (GPT 4.o)

Session ID:
Session Transcript

Session-94:

According to the 2022 WHO classification and ELN guidelines, how should this patients disease be classified?

A. Acute Myeloid Leukemia with BCR-ABL1
B. Chronic Myeloid Leukemia Chronic Phase
C. Chronic Myeloid Leukemia Accelerated Phase
D. Chronic Neutrophilic Leukemia
B

According to ELN 2022 risk stratification, which score should be used to assess this patients prognosis at diagnosis?

A. Sokal Score
B. IPSS
C. ELTS Score
D. DIPSS

C
Which of the following is the most appropriate initial test to establish the baseline molecular burden of BCR-ABL1 transcript at diagnosis?

A. FISH analysis on peripheral blood
B. Cytogenetic karyotyping from bone marrow
C. RT-qPCR for BCR-ABL1 on peripheral blood
D. Flow cytometry for CD34+ cells

C
Which of the following is the most appropriate initial treatment for this patient?

A. Imatinib 400 mg once daily
B. Dasatinib 100 mg once daily
C. Nilotinib 400 mg twice daily
D. Hydroxyurea until WBC normalization

A
Which of the following supportive care measures is most important in the first 4-6 weeks of treatment with Imatinib?

A. Antifungal prophylaxis
B. Blood pressure monitoring

C. Phlebotomy for iron overload
D. Prophylaxis for tumor lysis syndrome

B

If this patient develops febrile neutropenia after the first cycle of Imatinib therapy, what is the most appropriate initial management?

A. Start broad-spectrum antibiotics and continue Imatinib
B. Discontinue Imatinib and start granulocyte-colony stimulating factor (G-CSF)
C. Switch to a second-line TKI (Dasatinib or Nilotinib)
D. Delay chemotherapy until neutrophil count normalizes

A

Given this patients favorable response to Imatinib, what is the most appropriate next step in his management?

A. Continue Imatinib and monitor molecular response every 3 months
B. Switch to Dasatinib to improve long-term outcomes
C. Discontinue Imatinib and monitor for relapse with regular molecular testing
D. Start interferon-alpha therapy as a maintenance strategy

A

If the patients BCR-ABL1 transcript increases to 0.5% (on the International Scale) during routine monitoring, what is the next most appropriate action?

A. Increase Imatinib dose to 600 mg daily
B. Switch to a second-line TKI (Dasatinib or Nilotinib)
C. Add interferon-alpha to current therapy
D. Continue Imatinib and repeat the molecular test in 3 months

A

After increasing the dose to 600 mg daily, the patients BCR-ABL1 transcript falls to 0.03%. What is the next step?

A. Continue Imatinib at 600 mg daily and monitor every 3 months
B. Switch to a second-line TKI (Dasatinib or Nilotinib)
C. Discontinue Imatinib and monitor for relapse
D. Reduce Imatinib dose to 400 mg daily

A

What is the most important factor in deciding whether to discontinue Imatinib after a prolonged period of deep molecular response (MR4.5) in this patient?

A. Presence of any cardiovascular comorbidities
B. Achievement of complete cytogenetic response (CCyR)
C. Ability to tolerate a higher dose of Imatinib
D. Ongoing monitoring for molecular relapse and risk of disease recurrence

D

Session ID:
Assessment Domain

Session-94:
Rationale

Session ID:
Instructional Design and Pedagogical

Session-94:
The session's instructional design is superb. The case is expertly scaffolded, progressing logically from diagnosis and risk stratification (ELN guidelines, ELTS score) to initial therapy (Imatinib), ongoing monitoring, and management of subsequent challenges like loss of molecular response and considerations for treatment discontinuation. This structure seamlessly builds clinical reasoning. The questions are unambiguously framed and target the most critical decision points in modern CML care. While the learning objectives are not explicitly stated at the outset, they are clearly implied by the high-quality structure and content.

Session ID:
Clinical Authenticity and Content Fidelity

Session-94:
The session demonstrates exceptional clinical fidelity and authenticity. The patient case is highly plausible and represents a common real-world scenario. All diagnostic and therapeutic recommendations are perfectly aligned with current, evidence-based guidelines for Chronic Myeloid Leukemia (CML), such as those from ELN and WHO. Pharmacotherapeutic choices, including initial TKI selection, monitoring frequency, and response to treatment failure (Imatinib dose escalation), are optimal and entirely appropriate. Critically, the session maintains perfect domain specificity, with all concepts and treatments remaining squarely and correctly within the CML disease state. There is no evidence of domain entanglement.

Session ID:
Modeled Clinical Reasoning

Session-94:
The simulation provides an exemplary model of the clinical reasoning process for CML. Each stage of the case presents a perfectly framed clinical problem, from diagnosis to long-term strategy. The multiple-choice options offer an excellent breadth of plausible and relevant alternatives, with well-chosen distractors that test a sophisticated understanding of CML management (e.g., including prognostic scores from other myeloid disorders). The overall decision-making strategy is sound, prioritizing guideline-directed care and appropriate responses to monitoring data. The clinical outcomes are realistic and instructionally valuable, reinforcing the correctness of the chosen pathways.

Session-95

Session ID:
LLM

Session-95:
ChatGPT (GPT 4.o)

Session ID:
Session Transcript

Session-95:
A 47-year-old male presents to clinic with fatigue, early satiety, and unintentional weight loss over the past two months. On physical examination, he has mild splenomegaly. Lab results reveal:

WBC: 120 10/L (normal: 410)

Hb: 10.5 g/dL

Platelets: 550 10/L

Peripheral smear: marked leukocytosis with left shift, occasional blasts

LDH: Elevated

Uric acid: Elevated

Bone marrow biopsy shows hypercellularity with granulocytic hyperplasia.
Cytogenetics reveals t(9;22)(q34;q11.2).
RT-PCR confirms the presence of BCR-ABL1 (p210) transcript.

Question 1: What is the most appropriate diagnosis based on the WHO 2022 classification?

A. Acute myeloid leukemia (AML)
B. Chronic myeloid leukemia (chronic phase)
C. Chronic myelomonocytic leukemia (CMML)
D. Myelodysplastic syndrome with increased blasts
B. Chronic myeloid leukemia (chronic phase)
The patients presentationmarked leukocytosis with left shift, splenomegaly, and fatiguealong with the presence of the Philadelphia chromosome (t(9;22)) and BCR-ABL1 fusion transcript (p210) confirms the diagnosis of:

> Chronic Myeloid Leukemia (CML), chronic phase, according to WHO 2022 classification.

Why the other options are incorrect:

A. AML Would present with >20% blasts in the marrow or blood, which is not the case here.

C. CMML Characterized by monocytosis, absence of BCR-ABL1.

D. MDS with increased blasts Would show dysplasia and elevated blasts but not the BCR-ABL1 fusion gene.

A 47-year-old male presents to clinic with fatigue, early satiety, and unintentional weight loss over the past two months. On physical examination, he has mild splenomegaly. Lab results reveal:

WBC: 120 10/L (normal: 410)

Hb: 10.5 g/dL

Platelets: 550 10/L

Peripheral smear: marked leukocytosis with left shift, occasional blasts

LDH: Elevated

Uric acid: Elevated

Bone marrow biopsy shows hypercellularity with granulocytic hyperplasia.
Cytogenetics reveals t(9;22)(q34;q11.2).
RT-PCR confirms the presence of BCR-ABL1 (p210) transcript.

Question 2: According to the 2022 ELN risk stratification, which of the following tools should be used to assess this patient's prognosis at diagnosis?

A. Sokal Score
B. Hasford Score
C. ELTS Score
D. IPSS-R Score
C. ELTS Score
The ELTS Score (EUTOS Long-Term Survival score) is the preferred tool recommended by ELN 2022 to stratify CML patients at diagnosis. It predicts CML-related mortality, making it the most relevant in the TKI treatment era.

Why the others are less preferred:

A. Sokal Score Older, developed before TKIs; still used but less predictive of long-term outcomes.

B. Hasford Score Also pre-TKI, more relevant historically.

D. IPSS-R Score Used for myelodysplastic syndromes, not applicable in CML.

A 47-year-old male presents to clinic with fatigue, early satiety, and unintentional weight loss over the past two months. On physical examination, he has mild splenomegaly. Lab results reveal:

WBC: 120 10/L (normal: 410)

Hb: 10.5 g/dL

Platelets: 550 10/L

Peripheral smear: marked leukocytosis with left shift, occasional blasts

LDH: Elevated

Uric acid: Elevated

Bone marrow biopsy shows hypercellularity with granulocytic hyperplasia.
Cytogenetics reveals t(9;22)(q34;q11.2).
RT-PCR confirms the presence of BCR-ABL1 (p210) transcript.

Question 3: Which of the following best defines the role of RT-PCR for BCR-ABL1 in this patient at this stage?

A. To assess resistance mutations at baseline
B. To confirm diagnosis and establish a baseline for future molecular monitoring
C. To determine eligibility for allogeneic stem cell transplant
D. To evaluate treatment-induced cytogenetic changes

B. To confirm diagnosis and establish a baseline for future molecular monitoring

Explanation:
RT-PCR for BCR-ABL1 is essential in CML for both confirming the diagnosis and establishing a quantitative baseline for future molecular monitoring. This baseline is crucial for tracking measurable residual disease (MRD) and assessing treatment response over time.
Why the other options are incorrect:

A. To assess resistance mutations at baseline

C. To determine eligibility for allogeneic stem cell transplant

D. To evaluate treatment-induced cytogenetic changes Cytogenetic changes are assessed through karyotyping or FISH, not RT-PCR.

Case Continuation Phase 2

The patient is confirmed to have chronic-phase CML with an ELTS score indicating intermediate risk. He has no significant comorbidities and a good performance status.

Question 4: Which of the following is the most appropriate first-line treatment option for this patient?

A. Imatinib

B. Dasatinib
C. Nilotinib
D. Ponatinib
B. Dasatinib
The most appropriate first-line treatment for this patient is Dasatinib (Option B).

Why Dasatinib?

Dasatinib is a second-generation TKI that has shown superior efficacy compared to imatinib in terms of achieving faster and deeper molecular responses in patients with chronic-phase CML, particularly for those at intermediate or high risk based on the ELTS score.

It also has a better penetration into the bone marrow and may offer a quicker response, which aligns with the patient's risk stratification and performance status.

Why not the other options?

A. Imatinib While effective, imatinib is typically considered first-line only for low-risk patients or those with financial constraints.

C. Nilotinib Another second-generation TKI, but its use has more restrictions in patients with cardiovascular risks and some metabolic concerns.

D. Ponatinib A third-generation TKI primarily used for patients with resistant disease or the T315I mutation, not as a first-line option.

A 47-year-old male presents to clinic with fatigue, early satiety, and unintentional weight loss over the past two months. On physical examination, he has mild splenomegaly. Lab results reveal:

WBC: 120 10/L (normal: 410)

Hb: 10.5 g/dL

Platelets: 550 10/L

Peripheral smear: marked leukocytosis with left shift, occasional blasts

LDH: Elevated

Uric acid: Elevated

Bone marrow biopsy shows hypercellularity with granulocytic hyperplasia.
Cytogenetics reveals t(9;22)(q34;q11.2).
RT-PCR confirms the presence of BCR-ABL1 (p210) transcript.

Question 5: Which supportive care measure should be considered in the first month of therapy for this patient?

A. Prophylactic antibiotics for pneumonia
B. Bisphosphonates for bone health
C. Antifungal prophylaxis
D. Uric acid lowering therapy
D. Uric acid lowering therapy

Explanation:

Uric acid lowering therapy (Option D) is an important supportive care measure during the first month of treatment in CML.

Why Uric Acid Lowering Therapy?

Uric acid levels can increase rapidly due to tumor lysis syndrome (TLS), especially with the initiation of TKI therapy in patients with high tumor burden.

Allopurinol is commonly used to prevent or treat elevated uric acid levels and reduce the risk of kidney injury due to TLS.

Why not the other options?

A. Prophylactic antibiotics for pneumonia While infection is a concern in leukemia, there is no immediate indication for routine antibiotic prophylaxis unless the patient develops specific risk factors like febrile neutropenia.

B. Bisphosphonates for bone health Not necessary for this patient at this stage, as bisphosphonates are typically used for osteoporosis or bone metastasis in other malignancies.

C. Antifungal prophylaxis Antifungal prophylaxis is generally indicated for patients undergoing intensive chemotherapy or with neutropenia, but not necessarily for this patient in the chronic phase.

Case Continuation Phase 3

After 12 months of treatment with Dasatinib, the patient shows no detectable BCR-ABL1 transcript by RT-PCR and has complete cytogenetic remission. His treatment has been well tolerated, and he is experiencing no significant side effects.

Question 6: At this point in treatment, what is the most appropriate next step for monitoring this patient?

A. Repeat RT-PCR and bone marrow biopsy every 3 months
B. Continue RT-PCR monitoring every 3 months and assess for any molecular relapse
C. Discontinue therapy and monitor without treatment
D. Switch to imatinib due to complete cytogenetic remission
B. Continue RT-PCR monitoring every 3 months and assess for any molecular relapse
Explanation:

The most appropriate next step is to continue RT-PCR monitoring every 3 months and assess for any molecular relapse (Option B).

Given the complete cytogenetic remission and undetectable BCR-ABL1 transcript, the patient is in deep molecular remission.

Regular RT-PCR monitoring is crucial for detecting minimal residual disease (MRD) and ensuring that the patient remains in remission. If there is any molecular relapse, therapy can be adjusted accordingly.

Why not the other options?

A. Repeat RT-PCR and bone marrow biopsy every 3 months Bone marrow biopsy is unnecessary at this stage if the patient has already achieved complete cytogenetic remission and is being monitored through RT-PCR.

C. Discontinue therapy and monitor without treatment Discontinuing therapy is typically considered only for patients who have sustained deep molecular remission over a long period, which is not yet the case here.

D. Switch to imatinib Switching to imatinib is not recommended after achieving deep molecular remission with a more potent second-generation TKI like dasatinib. It could potentially result in a loss of remission.

A 47-year-old male presents to clinic with fatigue, early satiety, and unintentional weight loss over the past two months. On physical examination, he has mild splenomegaly. Lab results reveal:

WBC: 120 10/L (normal: 410)

Hb: 10.5 g/dL

Platelets: 550 10/L

Peripheral smear: marked leukocytosis with left shift, occasional blasts

LDH: Elevated

Uric acid: Elevated

Bone marrow biopsy shows hypercellularity with granulocytic hyperplasia.
Cytogenetics reveals t(9;22)(q34;q11.2).
RT-PCR confirms the presence of BCR-ABL1 (p210) transcript.

Question 7: The patient is now in remission for over 2 years. What is the next best step in the management of this patient, according to the ELN 2022 guidelines?

A. Continue dasatinib indefinitely
B. Attempt to stop dasatinib with close monitoring for relapse
C. Switch to nilotinib for better long-term outcomes
D. Start treatment with an allogeneic stem cell transplant
B. Attempt to stop dasatinib with close monitoring for relapse
Explanation:

According to the ELN 2022 guidelines, after 2 years in remission, it is appropriate to attempt treatment discontinuation with close monitoring for relapse (Option B).

If the patient has achieved sustained deep molecular remission, treatment discontinuation (often referred to as "treatment-free remission" or TFR) may be considered.

The patient should be monitored closely, typically with RT-PCR every 3 months, to detect any signs of molecular relapse early. If a relapse occurs, treatment with TKIs should be restarted immediately.

Why not the other options?

A. Continue dasatinib indefinitely While continuation of therapy is an option, after 2 years of remission, treatment discontinuation is a valid consideration in patients with sustained deep remission.

C. Switch to nilotinib for better long-term outcomes There is no clear evidence that switching to nilotinib would improve outcomes for a patient who has been stable on dasatinib.

D. Start treatment with an allogeneic stem cell transplant Stem cell transplant is typically reserved for patients with relapsed, resistant, or high-risk diseasenot for patients in complete molecular remission.

A 47-year-old male presents to clinic with fatigue, early satiety, and unintentional weight loss over the past two months. On physical examination, he has mild splenomegaly. Lab results reveal:

WBC: 120 10/L (normal: 410)

Hb: 10.5 g/dL

Platelets: 550 10/L

Peripheral smear: marked leukocytosis with left shift, occasional blasts

LDH: Elevated

Uric acid: Elevated

Bone marrow biopsy shows hypercellularity with granulocytic hyperplasia.
Cytogenetics reveals t(9;22)(q34;q11.2).
RT-PCR confirms the presence of BCR-ABL1 (p210) transcript.

Question 8: If this patient experiences a molecular relapse, what is the most appropriate next step in therapy?

A. Increase the dasatinib dose
B. Switch to a third-generation TKI like ponatinib
C. Start chemotherapy with cytarabine
D. Consider allogeneic stem cell transplant
B. Switch to a third-generation TKI like ponatinib
Explanation:

If the patient experiences a molecular relapse, the most appropriate next step is to switch to a third-generation TKI like ponatinib (Option B).

Ponatinib is a third-generation TKI and is highly effective for patients with resistant disease, including those who have the T315I mutation (which is resistant to other TKIs).

It is also used in patients who experience relapse or resistance after second-generation TKIs (like dasatinib or nilotinib). Ponatinib targets BCR-ABL1 more effectively and can overcome certain resistance mechanisms.

Why not the other options?

A. Increase the dasatinib dose Increasing the dose of dasatinib may not be effective if the relapse is due to resistance or a mutation that dasatinib cannot overcome.

C. Start chemotherapy with cytarabine Chemotherapy is not the first-line option for CML, especially if the patient has been on a TKI. Chemotherapy is reserved for cases of blast crisis or very advanced CML.

D. Consider allogeneic stem cell transplant A stem cell transplant is generally considered in relapsed or resistant CML after exhausting TKI options. However, switching to a more potent TKI is typically attempted first.

A 47-year-old male presents to clinic with fatigue, early satiety, and unintentional weight loss over the past two months. On physical examination, he has mild splenomegaly. Lab results reveal:

WBC: 120 10/L (normal: 410)

Hb: 10.5 g/dL

Platelets: 550 10/L

Peripheral smear: marked leukocytosis with left shift, occasional blasts

LDH: Elevated

Uric acid: Elevated

Bone marrow biopsy shows hypercellularity with granulocytic hyperplasia.
Cytogenetics reveals t(9;22)(q34;q11.2).
RT-PCR confirms the presence of BCR-ABL1 (p210) transcript.

Question 9: What is the main advantage of early and consistent MRD monitoring in CML treatment?

A. It helps predict long-term survival without the need for treatment
B. It allows early identification of treatment resistance, allowing for timely interventions
C. It determines the appropriate dose of chemotherapy for the patient
D. It eliminates the need for bone marrow biopsies after the first year of therapy
B. It allows early identification of treatment resistance, allowing for timely interventions
Explanation:

The main advantage of early and consistent MRD (Minimal Residual Disease) monitoring in CML treatment is that it allows early identification of treatment resistance, allowing for timely interventions (Option B).

MRD monitoring helps detect any molecular relapse or treatment resistance before it becomes clinically apparent, allowing for early adjustments to therapy. This proactive approach can prevent disease progression and improve long-term outcomes.

Why not the other options?

A. It helps predict long-term survival without the need for treatment MRD monitoring is useful for assessing the effectiveness of treatment, but it does not predict long-term survival without treatment.

C. It determines the appropriate dose of chemotherapy for the patient MRD monitoring is specific to TKI therapy and does not relate to chemotherapy dosing, which is used for different phases of leukemia (like blast crisis).

D. It eliminates the need for bone marrow biopsies after the first year of therapy While MRD monitoring reduces the need for bone marrow biopsies, it does not entirely eliminate them. Some cases still require biopsy for confirmation of disease progression or changes in treatment response.

Session ID:
Assessment Domain

Session-95:
Rationale

Session ID:

Instructional Design and Pedagogical

Session-95:

The session's instructional design is very strong. The case is expertly scaffolded, progressing logically from diagnosis and risk stratification (Questions 1-3) to initial therapy, monitoring, and advanced concepts like treatment-free remission (TFR) and molecular relapse (Questions 4-9). The instructional framing is exceptionally clear. The quality of feedback is generally high and provides correct rationales, but it oversimplifies the management of molecular relapse in Question 8, failing to mention the critical step of mutation analysis. The score for Clarity of Learning Objectives is slightly reduced because, while the educational goals are clearly implied by the structure, they are not explicitly stated at the outset of the session.

Session ID:

Clinical Authenticity and Content Fidelity

Session-95:

The clinical authenticity contains a mix of expert-level accuracy and one significant error. Plausibility & Guideline Alignment: The clinical narrative is indistinguishable from an authentic case of chronic-phase CML. The session generally aligns well with current ELN and NCCN guidelines, correctly identifying the diagnostic importance of BCR-ABL1, the use of the ELTS score for risk stratification, and the appropriateness of a second-generation TKI for initial therapy. Pharmacotherapeutic Error: A critical error occurs in Question 8. Upon molecular relapse after dasatinib, the simulation recommends an immediate switch to ponatinib. Standard practice and guidelines mandate performing BCR-ABL1 kinase domain mutation analysis first. The choice of the subsequent TKI is dictated by the mutation status. Recommending ponatiniban agent with significant toxicitywithout this step is a suboptimal and potentially unsafe choice, representing a serious pharmacotherapeutic accuracy flaw. Domain Specificity: The case content remains entirely within the domain of Chronic Myeloid Leukemia. All diagnostics, therapies, and concepts are appropriate to CML, and there is no evidence of domain entanglement.

Session ID:

Modeled Clinical Reasoning

Session-95:

The simulation expertly models the clinical reasoning process in CML management. Each question presents a well-framed problem with relevant data. The multiple-choice options are excellent, providing a strong breadth of plausible and relevant distractors that effectively test nuanced clinical knowledge (e.g., different risk scores, various TKIs). The overall strategic prioritization is sounddiagnose, stratify risk, treat, monitor, and react to changes in disease status. Per the rubric, the score for Prioritization and Decision-Making remains high because the strategy (acting on a molecular relapse by changing therapy) is correct, even though the specific content of the therapeutic choice was flawed (as scored in Section 2). The clinical outcomes described are realistic and instructionally valuable.

Session-96

Session ID:
LLM

Session-96:
ChatGPT (GPT 4.o)

Session ID:
Session Transcript

Session-96:
Phase 1 Case Introduction
A 42-year-old male presents with:

Fatigue, early satiety, and 10-pound weight loss over the past 2 months

Physical exam reveals splenomegaly 6 cm below costal margin

CBC shows:

WBC: 142 10/L ()

Hb: 11.1 g/dL ()

Platelets: 510 10/L ()

A peripheral blood smear shows myelocytes, metamyelocytes, and basophilia.

Bone marrow biopsy confirms:

Hypercellularity with granulocytic proliferation

Cytogenetics: t(9;22)(q34;q11.2)

PCR confirms BCR-ABL1 (e13a2) transcript

Question 1:
Which of the following classifies this patients disease according to WHO 2022 criteria?

A. Chronic phase Chronic Myeloid Leukemia (CML-CP)
B. Accelerated phase CML (CML-AP)
C. Blast phase CML (CML-BP)
D. BCR-ABL1positive Acute Myeloid Leukemia
WHO 2022 Classification relies on clinical, morphologic, and molecular features. The phase of CML is based on:
Blast percentage in peripheral blood or bone marrow
Basophil count
WBC trends
Splenomegaly severity
Presence of additional chromosomal abnormalities
Key to remember:
Chronic phase (CML-CP): <10% blasts, mild basophilia, often diagnosed via routine labs.
Accelerated phase (CML-AP): 1019% blasts, persistent high counts, increasing basophils (>20%).

Blast phase (CML-BP): 20% blasts OR extramedullary blast proliferation.
 This patient has <10% blasts, no mention of additional cytogenetic abnormalities, and was diagnosed with BCR-ABL1positive disease with typical myeloproliferation.
Let me know when you're ready to submit your answer.
You said:
a
ChatGPT said:
 Correct, [[REDACTED]]!
Answer: A. Chronic phase Chronic Myeloid Leukemia (CML-CP)

 Rationale:
According to WHO 2022 criteria, this patient's presentation is consistent with CML in chronic phase:

<10% blasts in blood/marrow

No clonal evolution (no additional chromosomal abnormalities reported)

Peripheral basophilia is noted, but not >20%

Splenomegaly is typical in CP

Cytogenetics: Classical t(9;22) translocation with BCR-ABL1 confirms CML
Question 2:
Based on the European LeukemiaNet (ELN) 2022 criteria, which of the following risk stratification tools should be used at diagnosis to assess prognosis?

A. IPSS (International Prognostic Scoring System)
B. Sokal score
C. R-ISS (Revised International Staging System)
D. FLIPI (Follicular Lymphoma International Prognostic Index)
ELN 2022 guidelines recommend a prognostic scoring system at diagnosis to stratify risk and guide TKI selection, especially when weighing options beyond imatinib (e.g., second-generation TKIs).

You should consider a tool that uses:

Age

Spleen size

WBC count

Platelet count

 Heres a breakdown of your options:

Sokal Score: The most widely used for CML at diagnosis.

IPSS: Used for myelodysplastic syndromes, not CML.

R-ISS: Applies to multiple myeloma.

FLIPI: For follicular lymphoma, not leukemia.

When you're ready, drop your answer

You said:
b
ChatGPT said:
 Correct again, [[REDACTED]]!
Answer: B. Sokal Score
 Rationale:
The Sokal Score is one of the validated risk assessment tools used in CML at diagnosis, per ELN 2022 guidelines. It incorporates:
Age
Spleen size
WBC count
Platelet count
While newer tools like EUTOS and ELTS are also mentioned in guidelines, Sokal remains the standard for initial stratification in practice and trials.

Question 3:
Youve started TKI therapy. At 3 months, BCR-ABL1 transcript (IS) is 8.5% by RT-qPCR. According to ELN 2022 molecular response criteria, how is this best interpreted?

A. Optimal response
B. Warning
C. Failure
D. Relapse

Molecular monitoring with RT-qPCR for BCR-ABL1 (on the International Scale, or IS) is the cornerstone of assessing treatment response.

At 3 months, response is categorized as:

Optimal: BCR-ABL1 10% (IS)

Warning: BCR-ABL1 >10%

Failure: Only if theres loss of response (e.g., no cytogenetic response, new mutations, or disease progression)

Important: Relapse is not used for initial responseit refers to loss of a previously achieved response.

So, a BCR-ABL1 of 8.5% at 3 months falls into which category?

You said:
b
ChatGPT said:
 Correct, [[REDACTED]]!

Answer: B. Warning

 Rationale:
According to ELN 2022 guidelines, a BCR-ABL1 level of 8.5% (IS) at 3 months is considered a warning. The target for optimal response at 3 months is 10% BCR-ABL1 (IS). Anything above 10% would require close monitoring or a switch in therapy, but 8.5% is on track with acceptable outcomes for a chronic phase CML patient.
Phase 2 Case Continuation
The patient has been started on imatinib 400 mg daily and is instructed on adherence and potential adverse effects.
At 6 months, the patient reports improvement in energy levels, but continues to have mild splenomegaly (about 3 cm below the costal

margin).

The repeat BCR-ABL1 PCR (IS) shows BCR-ABL1: 3.2%.

Question 4:
Based on the ELN 2022 criteria for molecular response at 6 months, how would you classify this patients response to imatinib therapy?

A. Optimal response
B. Warning
C. Failure
D. Relapse
At 6 months, the targets for molecular response are:

Optimal response: BCR-ABL1 1% (IS)

Warning: BCR-ABL1 >1% but 10% (IS)

Failure: BCR-ABL1 >10% (IS) or lack of cytogenetic response (i.e., no or minimal reduction in Philadelphia chromosome-positive cells)

So, the BCR-ABL1 of 3.2% at 6 months places this patient where? Take a moment and make your decision!

You said:
a
ChatGPT said:
Not quite, [[REDACTED]].

Answer: B. Warning
Rationale:
According to ELN 2022, a BCR-ABL1 of 3.2% (IS) at 6 months is considered a warning, as it is >1% but 10%. While this indicates the patient is responding to therapy, it does not meet the criteria for optimal response (which is 1% at 6 months).

If the response were optimal, wed expect a BCR-ABL1 1%.

Question 5:
This patient is experiencing mild splenomegaly despite achieving a warning response at 6 months. Which of the following should be done next?

A. Continue current therapy, monitor closely, and repeat PCR in 3 months
B. Switch to dasatinib (2nd-line TKI) immediately
C. Initiate antimicrobial prophylaxis for possible infection
D. Refer the patient for allogeneic stem cell transplant evaluation
For a patient with mild splenomegaly and a warning response at 6 months, it's important to consider the next best management strategy:

Continuing current therapy with close monitoring is often preferred unless there's a more serious issue (e.g., progression or failure).

Switching to dasatinib (2nd-line TKI) might be considered if theres evidence of resistance or suboptimal response, but it's not typically done right after a warning response without further investigation.

Antimicrobial prophylaxis would be considered for high-risk patients, but it's not directly related to CML progression or response.

A stem cell transplant would generally be considered later if there's evidence of resistant disease or progression, not just mild splenomegaly or a warning response.

Given the warning response and the absence of significant progression, what do you think should be the next step?

Take your time to choose!

You said:
a
ChatGPT said:
Correct, [[REDACTED]]!

Answer: A. Continue current therapy, monitor closely, and repeat PCR in 3 months

Rationale:
In the case of a warning response at 6 months, the standard approach is to continue current therapy and monitor closely. The splenomegaly and 3.2% BCR-ABL1 (IS) are still within an acceptable range for a chronic phase patient, though its important to reassess the response at 9 months or 12 months.

The goal is to ensure theres no progression or resistance, and its also crucial to evaluate for any potential drug interactions or compliance issues that could be affecting the response.
Question 6:
The patients next follow-up at 9 months reveals BCR-ABL1 of 0.9% (IS). Which of the following is the best next step in therapy?

A. Continue imatinib and monitor closely
B. Switch to nilotinib (2nd-line TKI)
C. Initiate immunotherapy with monoclonal antibodies
D. Consider allogeneic stem cell transplant
At 9 months, this patient has improved with a BCR-ABL1 of 0.9% (IS). This is significantly better than the 3.2% at 6 months, and its now within the optimal response range for CML.

Now, consider the next step:

Continue imatinib: If the patients response is improving and BCR-ABL1 is 1%, this is often a sign that imatinib remains a good choice.

Switch to nilotinib: Not needed yet. Nilotinib is considered for failure or lack of response after 12 months of imatinib therapy.

Immunotherapy: Not typically used for CML unless in specific research settings.

Allogeneic stem cell transplant: Usually considered for advanced phases (accelerated or blast phase), not for chronic phase CML with a good response.

What do you think is the best next step?

You said:
a
ChatGPT said:
Correct again, [[REDACTED]]!
Answer: A. Continue imatinib and monitor closely

Rationale:
With a BCR-ABL1 of 0.9% (IS) at 9 months, the patient is showing an optimal response to imatinib. The appropriate next step is to continue the current therapy and monitor closely.

At this point, the patient's response is on track, and imatinib remains the treatment of choice unless there is evidence of progression or resistance.

Phase 3 Case Continuation
At 18 months, the patients BCR-ABL1 is undetectable by PCR (MRD-negative), and theres no clinical evidence of progression.

Question 7:
Given that the patient has achieved MRD-negative status at 18 months, which of the following is the most appropriate next step in therapy?

A. Continue imatinib indefinitely
B. Switch to dasatinib (2nd-line TKI) to reduce risk of relapse
C. Consider treatment discontinuation (treatment-free remission trial)
D. Refer for allogeneic stem cell transplant evaluation

Lets focus on treatment strategies for patients with MRD-negative status:

Treatment-free remission (TFR) is an emerging option for patients who have:

Achieved deep molecular remission (MRD-negative)

Been on therapy for a sustained period (usually 3+ years)

Show no evidence of progression

Are closely monitored for relapse after stopping treatment

Current guidelines support treatment discontinuation trials for eligible patients, especially those in chronic phase CML with MRD-negative status. This decision is often based on patient preference and their individual risk factors.

Based on these considerations, what do you think would be the best next step for this patient?
You said:
c
ChatGPT said:
Correct, [[REDACTED]]!
Answer: C. Consider treatment discontinuation (treatment-free remission trial)
Rationale:
For patients who achieve MRD-negative status after 18 months of therapy, treatment-free remission (TFR) is a viable option, provided they meet the criteria:
MRD-negative (undetectable BCR-ABL1)
Chronic phase CML
Stable remission for at least 12 years

Close monitoring for relapse

If the patient is a suitable candidate, discontinuing treatment can be considered as part of a treatment-free remission trial. This is in line with recent advances in CML management, where TKIs are tapered or stopped in eligible patients to evaluate whether the disease remains controlled without continuous therapy.

Session ID:
Assessment Domain

Session-96:
Rationale

Session ID:
Instructional Design and Pedagogical

Session-96:
The session's instructional design has notable strengths but is undermined by a significant flaw in its feedback. The case is expertly scaffolded, moving logically from diagnosis and risk stratification through treatment monitoring milestones and into long-term management decisions like treatment-free remission. The instructional framing is exceptionally clear, with helpful hints provided for each question. However, the quality of the embedded feedback is poor due to a critical error in Question 3, where the rationale is factually incorrect and contradicts established guidelines. While most rationales are well-structured, a single erroneous one significantly degrades the pedagogical quality. Lastly, learning objectives are not explicitly stated at the outset, though they are strongly implied by the session's structure.

Session ID:
Clinical Authenticity and Content Fidelity

Session-96:
The session's clinical content contains a critical error that significantly compromises its fidelity. Plausibility & Domain Specificity: The clinical narrative is highly plausible and indistinguishable from an authentic CML case. The simulation remains strictly within the CML domain, with no evidence of domain entanglement. Guideline Alignment & Accuracy: A major error occurs in Question 3. The simulation incorrectly classifies a 3-month BCR-ABL1 level of 8.5% (IS) as a "Warning." According to the cited ELN 2022 guidelines, a BCR-ABL1 of 10% at 3 months is an Optimal Response. This misclassification represents a gross deviation from the guideline and a dangerous pharmacotherapeutic inaccuracy, as misinterpreting response milestones can lead to inappropriate changes in management. This single error warrants a low score for both alignment and accuracy.

Session ID:
Modeled Clinical Reasoning

Session-96:
The simulation models clinical reasoning effectively in structure, but this is compromised by the content error. Problem framing is expert-level, presenting clear challenges at each stage. The breadth of diagnostic and therapeutic options is excellent, including relevant and plausible distractors that test nuanced knowledge. The modeling of clinical outcomes is also realistic and instructive. However, the prioritization and decision-making process is flawed. The reasoning modeled in Question 3 is based on an incorrect interpretation of a key clinical data point (the 3-month BCR-ABL1 level). Since sound decision-making relies on the correct interpretation of data, this error reduces the score for this criterion.

Session-97

Session ID:
LLM

Session-97:
ChatGPT (GPT 4.o)

Session ID:
Session Transcript

Session-97:
Case Phase 1: Patient Presentation

A 42-year-old male presents to the outpatient clinic with fatigue, early satiety, and unintentional weight loss over the past month. On examination, he has mild splenomegaly.

Initial Labs:

WBC: 112 10/L (high)

Hb: 10.5 g/dL (low)

Platelets: 560 10/L (high)

Peripheral blood smear: marked left shift with basophilia and myelocytes

Bone marrow aspirate: hypercellular with granulocytic hyperplasia

Cytogenetics:

Conventional karyotyping: t(9;22)(q34;q11.2) in 100% of metaphases

FISH: BCR-ABL1 fusion positive

RT-PCR: BCR-ABL1 (e13a2 transcript), IS 75%

Question 1

Which of the following best confirms the diagnosis of Chronic Myeloid Leukemia (CML) in chronic phase, according to WHO 2022 criteria?

A. Presence of t(9;22) on karyotype alone
B. Peripheral blood basophilia with leukocytosis
C. Detection of BCR-ABL1 fusion transcript by PCR
D. All of the above, with no blast excess and <10% blasts in marrow/blood
Correct, [[REDACTED]] well done!

Explanation:

The diagnosis of CML in chronic phase per WHO 2022 classification requires:

Detection of the Philadelphia chromosome t(9;22)(q34;q11.2) by karyotyping or FISH,

Detection of the BCR-ABL1 fusion transcript via molecular methods (e.g., RT-PCR),

Absence of features of accelerated or blast phase, specifically <10% blasts in blood or marrow.

Option D includes all necessary components for diagnosis and confirms the disease is in chronic phase, which is critical for treatment planning.

Referenced Guideline: WHO Classification of Hematolymphoid Tumors, 5th Edition (2022)
Case Progression

Your patient is now diagnosed with CML in chronic phase.

Question 2

According to ELN 2022, which of the following factors is NOT included in the Sokal or EUTOS long-term survival (ELTS) risk stratification scoring?

A. Age
B. Spleen size
C. BCR-ABL1 transcript type
D. Platelet count
Excellent, [[REDACTED]] thats correct!

Answer: C. BCR-ABL1 transcript type

Explanation:

The BCR-ABL1 transcript type (e13a2 vs e14a2) is not a component of Sokal, Hasford, EUTOS, or ELTS scores.
These scores are based on easily obtainable clinical data at diagnosis, such as:

Age

Spleen size

WBC count

Platelet count

Blasts in peripheral blood (for some scores)

While the transcript type may affect molecular response kinetics (e.g., e14a2 often associated with deeper responses), it is not used for initial risk stratification.
This patients ELTS risk score places him in the intermediate risk group.

Which of the following is the primary clinical implication of this risk stratification?

A. It predicts the likelihood of achieving a major molecular response (MMR) at 12 months.
B. It determines whether the patient should receive allogeneic stem cell transplant up front.
C. It guides the selection between first- and second-generation TKIs.
D. It predicts long-term disease-specific survival and progression risk.
Spot on again, [[REDACTED]] youre doing great!

Answer: D. It predicts long-term disease-specific survival and progression risk

Explanation:

The ELTS (EUTOS Long-Term Survival) score is specifically designed to estimate the probability of dying from CML, rather than from other causes. This makes it more clinically relevant in the TKI era, where many patients live for decades with proper therapy.

It does not directly predict MMR (A).

Its not a criterion for upfront transplant (B).

While risk score may influence TKI selection (C), ELTS's core role is to predict progression and CML-related death which is what option D correctly describes.
Case Update:

Your 42-year-old male patient with newly diagnosed CML in chronic phase and intermediate ELTS risk has the e13a2 BCR-ABL1 transcript.

Medical History:

Mildly elevated liver enzymes (ALT 1.5 ULN)

No cardiovascular disease

Normal renal function

ECOG performance status: 0

No history of medication allergies

Question 4

Based on current ESMO guidelines and this patient's profile, which of the following is the most appropriate first-line treatment option?

A. Imatinib 400 mg daily
B. Nilotinib 300 mg twice daily
C. Dasatinib 100 mg daily
D. Bosutinib 500 mg daily
Good try, [[REDACTED]], but the correct answer is A: Imatinib 400 mg daily.

Explanation:

The first-line treatment for CML in chronic phase is typically Imatinib (Gleevec) at a dose of 400 mg daily according to ESMO guidelines. Its the standard of care for patients without contraindications and provides effective long-term control in many patients with

manageable side effects.

Nilotinib (B) and Dasatinib (C) are second-generation TKIs that may be used in patients who are intolerant to Imatinib or have suboptimal responses. They are more potent but come with additional risks, such as cardiovascular issues (nilotinib) and pleural effusions (dasatinib).

Bosutinib (D) is another option but is typically used in resistant cases or for patients who cannot tolerate other TKIs, as it has a more restrictive safety profile.
Given the patients mildly elevated liver enzymes, which of the following is the most appropriate monitoring plan for this patient starting Imatinib therapy?

A. Monitor liver enzymes and CBC every 2 weeks for the first 2 months
B. Monitor liver enzymes and CBC every month for the first 3 months
C. Monitor liver enzymes and CBC every 3 months after initiating therapy
D. Monitor liver enzymes and CBC only at 6 months after starting therapy
Correct, [[REDACTED]] great choice!

Answer: A. Monitor liver enzymes and CBC every 2 weeks for the first 2 months

Explanation:

For patients starting Imatinib, the initial monitoring is typically more frequent to detect any potential adverse events, especially for liver toxicity and hematologic changes. Given this patient's mildly elevated liver enzymes, its important to monitor:

Liver function tests (ALT, AST)

Complete blood count (CBC)

The guidelines recommend every 2 weeks for the first 2 months to detect and address issues early, such as hepatotoxicity or cytopenias, especially since Imatinib is metabolized by the liver and can cause liver enzyme elevations.

After the initial monitoring period, the frequency can be reduced to monthly checks, then quarterly as long as the patient remains stable.
This patient starts Imatinib and presents at his follow-up appointment with grade 2 nausea and edema (mild). Which of the following is the best next step in management?

A. Discontinue Imatinib and switch to a second-generation TKI
B. Reduce the Imatinib dose to 300 mg daily
C. Recommend supportive care measures, including antiemetics and diuretics
D. Start corticosteroids to manage the edema
Correct, [[REDACTED]] well done!

Answer: C. Recommend supportive care measures, including antiemetics and diuretics

Explanation:

For mild side effects such as grade 2 nausea and edema, supportive care is typically the first-line approach:

Antiemetics for nausea (e.g., ondansetron) to control symptoms.

Diuretics for edema, if necessary, to reduce fluid retention.

These side effects do not typically require a dose reduction or drug switch unless they are severe or persistent, and discontinuing Imatinib would not be appropriate for mild symptoms.

If symptoms worsen, dose adjustments or a switch to a second-generation TKI may be considered, but this is not the first step for mild symptoms.
At the 3-month follow-up, this patient has achieved a major molecular response (MMR) (BCR-ABL1/ABL ratio 0.1% IS). What is the next best step in management?

A. Continue Imatinib at the current dose and monitor molecular response every 3 months
B. Increase Imatinib dose to 600 mg daily to further reduce BCR-ABL1 levels
C. Switch to a second-generation TKI, as MMR is suboptimal
D. Discontinue Imatinib due to achievement of MMR and monitor for relapse
Correct again, [[REDACTED]] nice work!

Answer: A. Continue Imatinib at the current dose and monitor molecular response every 3 months

Explanation:

Since your patient has achieved major molecular response (MMR), this is a significant milestone in CML therapy. The goal of treatment is to maintain MMR and minimize the risk of progression.

Continuing Imatinib at the current dose is appropriate, as the patient is responding well.

Molecular monitoring should continue every 3 months to ensure that MMR is sustained and to detect any signs of relapse or resistance.

Increasing the Imatinib dose (B) is typically not necessary unless the patient is not responding adequately.

Switching to a second-generation TKI (C) is not indicated in this scenario because the patient has achieved a good response.

Discontinuing therapy (D) is not recommended unless the patient reaches sustained deep molecular response (MR4.5) and qualifies for treatment-free remission, which would require additional criteria.
Case Update:

After 3 months of Imatinib therapy, your patient has achieved a major molecular response (MMR) (BCR-ABL1/ABL ratio 0.1% IS).

Now, at 6 months, the patients BCR-ABL1 level is still 0.03% IS. His liver enzymes have returned to normal, and his performance status remains excellent (ECOG 0).

Question 8

Based on this patients current molecular response (BCR-ABL1/ABL ratio of 0.03% IS), what is the best next step?

A. Continue Imatinib at the current dose and monitor molecular response every 3 months
B. Increase the Imatinib dose to 600 mg daily to achieve deeper molecular response
C. Switch to a second-generation TKI (nilotinib or dasatinib) for better molecular control
D. Discontinue Imatinib and monitor for relapse as the patient is in deep molecular remission
Perfect choice, [[REDACTED]] youre nailing it!

Answer: A. Continue Imatinib at the current dose and monitor molecular response every 3 months

Explanation:

A BCR-ABL1 level of 0.03% IS at 6 months is an excellent response well within the ELN 2022 definition of optimal molecular response. Here's why A is correct:

The patient is achieving steady and deep molecular control.

Theres no indication to escalate dose or change therapy in someone doing this well.

Ongoing molecular monitoring every 3 months is standard to track for sustained response or early signs of resistance.

Switching therapy or dose escalation would not offer added benefit and may introduce unnecessary toxicity.

After 3 years of therapy, your patient has maintained MR4.5 (BCR-ABL1/ABL 0.0032%) for over 2 years. He is now interested in treatment-free remission (TFR).

Which of the following is an eligibility criterion for safely attempting TFR?

A. Age < 45 years
B. History of any grade 2 TKI toxicity
C. Sustained MR4.5 for at least 2 years with stable molecular testing
D. BCR-ABL1 level 0.1% for 12 months
Almost there, [[REDACTED]] great effort, but the correct answer is C: Sustained MR4.5 for at least 2 years with stable molecular testing.

Explanation:

Treatment-Free Remission (TFR) is a goal in CML management, but strict criteria are required to ensure safety:

Sustained deep molecular response (MR4.5 or deeper) for at least 2 years is essential.

The patient must have stable, reliable molecular monitoring access (qPCR every 46 weeks initially after stopping).

Age is not a limiting factor (A).

A history of grade 2 toxicity (B) may actually be a reason to consider TFR if the patient wishes to avoid long-term side effects.

MR3 (0.1%) for 12 months (D) is not sufficient thats considered MMR, not a deep enough response to attempt discontinuation.
The patient is approved for TFR and discontinues Imatinib. At Month 4 post-discontinuation, his BCR-ABL1 increases to 0.06% IS.

What is the most appropriate next step?

A. Restart Imatinib immediately
B. Monitor again in 4 weeks to confirm trend
C. Switch to a second-generation TKI
D. No action needed; this level is acceptable for TFR
Excellent choice, [[REDACTED]] B is absolutely correct!

Answer: B. Monitor again in 4 weeks to confirm trend

Explanation:

In the TFR setting, the management of molecular relapse is guided by ELN and ESMO guidelines:

A single rise in BCR-ABL1 to between 0.01% and 0.1% IS is not an automatic trigger to restart therapy.

The proper approach is to repeat qPCR in 4 weeks to:

Confirm a rising trend, or

Determine if it was a transient fluctuation (which can happen).

When do we restart therapy?

If BCR-ABL1 rises above 0.1% IS (loss of MMR), TKI therapy must be resumed immediately.

Your decision reflects excellent clinical reasoning and adherence to evidence-based guidelines.

Session ID:
Assessment Domain

Session-97:
Rationale

Session ID:
Instructional Design and Pedagogical

Session-97:
The session's instructional design is highly effective. It masterfully scaffolds the case, beginning with diagnosis and risk stratification and progressing logically through the complexities of first-line therapy selection, adverse event management, response monitoring, and long-term goals like Treatment-Free Remission (TFR). The embedded feedback is a standout feature, providing clear, guideline-referenced explanations for each choice. Instructions are unambiguous and intuitive. The only minor shortcoming is the absence of explicitly stated, measurable learning objectives at the start, though the educational goals are strongly implied by the case's structure.

Session ID:
Clinical Authenticity and Content Fidelity

Session-97:
The clinical authenticity of this session is marked by a critical contradiction: while the narrative is highly plausible and there is no domain entanglement, it contains a significant content error that undermines its educational value. Plausibility & Specificity: The patient's journey is exceptionally realistic and mirrors a typical CML case. The simulation remains entirely within the CML domain, using appropriate terminology, diagnostics, and therapeutics. Pharmacotherapeutic & Guideline Error: A critical error occurs in the feedback for Question 7. It states that achieving Major Molecular Response (MMR, BCR-ABL1 0.1% IS) at 3 months is the expected milestone. This is incorrect. According to ELN and NCCN guidelines, the optimal 3-month molecular milestone is BCR-ABL1 10% IS.

Misstating this fundamental therapeutic goal represents a major pharmacotherapeutic inaccuracy and deviation from guidelines. While not directly dangerous, it teaches a flawed metric for assessing treatment efficacy, which could lead to clinical misjudgment. This error warrants a "Suboptimal or questionable choices" rating for accuracy and "Mostly aligned, with minor inaccuracies" for guideline alignment, though the error itself is significant.

Session ID:
Modeled Clinical Reasoning

Session-97:
The simulation provides an exemplary model of the clinical reasoning process. Each stage of the case presents a well-framed, relevant clinical problem with all necessary data provided. The multiple-choice options are well-constructed, offering a strong range of plausible alternatives and key distractors that effectively test nuanced clinical judgment. The overall narrative demonstrates expert-level prioritization, moving logically from diagnosis and risk assessment to therapy and long-term management. The clinical outcomes for each decision are realistic, specific, and instructionally valuable, reinforcing the learning points effectively. Despite a content-level error in Section 2, the underlying reasoning strategy modeled here is sound.

Session-98

Session ID:
LLM

Session-98:
ChatGPT (GPT 4.o)

Session ID:
Session Transcript

Session-98:
Patient Case - Phase 1
Patient: Mr. Omar H., a 42-year-old male
Chief Complaint: Fatigue, early satiety, and unintentional 6-kg weight loss over 3 months
History: No significant past medical history
Vitals: HR 96 bpm, BP 118/74 mmHg, Temp 37.1C
Physical Exam: Palpable splenomegaly 8 cm below costal margin
Labs:

WBC: 152 10/L

Hb: 11.2 g/dL

Platelets: 650 10/L

Peripheral smear: Myelocytes, metamyelocytes, basophilia

LDH: Elevated

Uric acid: Elevated
Bone Marrow Aspiration: Hypercellular marrow with granulocytic hyperplasia
Cytogenetics: t(9;22)(q34;q11.2) confirmed
Molecular Testing: BCR::ABL1 p210 detected via RT-PCR

Question 1:
Based on this presentation, what is the most appropriate diagnosis for Mr. Omar?

A. Acute Myeloid Leukemia with BCR::ABL1 fusion
B. Chronic Myeloid Leukemia in Chronic Phase
C. Chronic Myeloid Leukemia in Accelerated Phase
D. Myelodysplastic Syndrome with isolated del(5q)

Please enter A, B, C, or D. Type "HINT" if youd like some guidance
hint

ChatGPT said:
Certainly, [[REDACTED]].

Hint:
To distinguish chronic phase (CP) from accelerated phase (AP) or blast crisis (BC) in CML, you should consider:

Blast percentage in blood/marrow (<10% for CP, 1019% for AP)

Peripheral basophilia: >20% may suggest AP

Cytogenetic/molecular confirmation of BCR::ABL1 fusion is essential

WHO 2016/2022 classifies CML-CP based on clinical findings without excessive blasts or significant dysplasia
Refer to ESMO 2023 guidelines, Table 1 for phase classification criteria.
Let me know your choice: A, B, C, or D.
You said:
b
ChatGPT said:
Correct!
Explanation:
Mr. Omars case is classic for Chronic Myeloid Leukemia in Chronic Phase (CML-CP), based on: Presence of BCR::ABL1 p210 fusion (confirmed via RT-PCR)
Peripheral blood features consistent with chronic phase (WBC elevation, myelocytes, basophilianot exceeding 20%)
No evidence of 10% blasts in blood or marrow
No dysplasia or other high-risk cytogenetics
Guideline Connection: ESMO 2023 and WHO 2022 define CML-CP by BCR::ABL1 fusion, <10% blasts, and absence of accelerated/blast features (Hochhaus et al., Ann Oncol 2023).
According to the 2022 ELN risk stratification, which of the following scoring systems is recommended to assess Mr. Omars baseline risk?
A. Sokal Score
B. Hasford (Euro) Score
C. ELTS Score
D. IPSS-R Score
Your choice: A, B, C, or D
Let me know if youd like a HINT.
hint
ChatGPT said:
Sure, heres your hint:
Hint:
While several prognostic scores exist for CML, ELN 2022 guidelines recommend using a model that predicts disease-specific mortality, especially in the era of TKIs. It incorporates age, spleen size, platelet count, and blast percentage, but with a stronger weight on long-term survival and CML-related death, rather than response to older chemotherapy.
The IPSS-R is used in myelodysplastic syndromes, not CML.
Ready to choose? A, B, C, or D.
You said:
b
ChatGPT said:
Not quite, but a good try.
Correct Answer: C. ELTS Score
Mr. Omars ELTS score is calculated and classifies him as intermediate risk. Based on this, which initial diagnostic follow-up is most important to establish a treatment baseline?
A. JAK2 V617F mutation testing
B. BCR::ABL1 transcript quantification (IS) by RT-qPCR
C. TP53 mutation screening
D. IDH1/2 mutation analysis
Your choice: A, B, C, or D. Type "HINT" if you'd like help.
hint
ChatGPT said:
Of course!
Hint:

In CML, treatment response is tracked longitudinally using a molecular marker unique to the disease. Establishing a baseline level of this marker is critical before initiating TKI therapy.
This test is quantitative, uses RT-qPCR, and must be reported on the International Scale (IS) to ensure consistency across centers.
Let me know your answer: A, B, C, or D.
You said:
b
ChatGPT said:
Correct! Well done, [[REDACTED]]!
Which of the following is the most appropriate first-line therapy for Mr. Omar?
A. Imatinib 400 mg daily
B. Nilotinib 300 mg BID
C. Dasatinib 100 mg daily
D. Asciminib 80 mg BID
Your choice: A, B, C, or D. Type "HINT" if youd like guidance.
hint
ChatGPT said:
Absolutely!
Hint:
In patients with intermediate or high ELTS risk, ELN 2022 and ESMO 2023 prefer second-generation TKIs due to their faster and deeper molecular responses compared to imatinib.
Consider the absence of comorbidities: No cardiovascular, pulmonary, or hepatic issues
Asciminib is currently not first-line outside clinical trials
Check the ELN 2022 Table 2 for first-line TKI recommendations by risk group.

Let me know your answer: A, B, C, or D.
You said:
b
ChatGPT said:
Correct! Excellent choice!
Which supportive care measure should be implemented prior to starting TKI therapy in this patient?
A. Initiate allopurinol and aggressive hydration
B. Start prophylactic anticoagulation
C. Begin prophylactic acyclovir and fluconazole
D. Transfuse platelets to maintain >100 x 10/L
Whats your choice: A, B, C, or D? Or type HINT.
hint
ChatGPT said:
Certainly!

hint:
This patient presents with marked leukocytosis and elevated uric acid and LDH, which are signs of high tumor burden.
The key concern before TKI initiation in such settings is tumor lysis syndrome (TLS). You want to prevent metabolic complications that may arise from rapid leukemic cell death once therapy starts.
Ready to answer? A, B, C, or D.
You said:
a
ChatGPT said:
Correct again, [[REDACTED]]! You're on a roll.

Mr. Omar starts nilotinib and returns for a 2-week follow-up. He reports new onset muscle cramps, mild fatigue, and his labs show:
WBC: to 45 10/L
Hb: 10.5 g/dL
Platelets: 320 10/L
K: 3.2 mmol/L

Phosphate: Low
Mg: 1.3 mg/dL
Which of the following is the best next step?
A. Hold TKI and initiate corticosteroids
B. Supplement electrolytes and continue therapy
C. Switch to imatinib due to toxicity
D. Reduce nilotinib dose by 50%
Whats your answer? Or type HINT if needed.
hint
ChatGPT said:
Absolutely!
Hint:
These symptoms (e.g., muscle cramps, fatigue) along with hypokalemia, hypophosphatemia, and hypomagnesemia are common electrolyte disturbances seen with TKIsespecially nilotinib and dasatinib.
Go ahead with your choice: A, B, C, or D.
You said:
b
ChatGPT said:
Correct! Excellent clinical reasoning.
At month 3, Mr. Omars labs show:
WBC: 6.3 10/L
Hb: 12.2 g/dL
Platelets: 250 10/L
BCR::ABL1 (IS): 12.5%
How should this result be interpreted according to ELN 2022 milestones?
A. Optimal response
B. Warning requires close monitoring
C. Failure switch therapy
D. MR3 deep molecular response
Your pick: A, B, C, or D? Or type HINT.
hint
ChatGPT said:
Certainly!
Hint:
ELN 2022 defines specific molecular targets at 3, 6, and 12 months to assess response.
At 3 months, the goal is BCR::ABL1 10% (IS)
A result >10% is not an automatic failure, but its no longer considered optimal
This scenario places the patient in a gray zone, where close molecular follow-up is essential
Your turn! Choose: A, B, C, or D.
You said:
b
ChatGPT said:
Correct! Great job recognizing this nuance.
Mr. Omar's 6-month follow-up shows:
WBC, Hb, PLTs: Normal
BCR::ABL1 (IS): 2.4%
No adverse events, adherence confirmed
Continues nilotinib 300 mg BID

Question 8:
How should Mr. Omars 6-month response be classified per ELN 2022?
A. Optimal continue current therapy
B. Warning consider switching TKI
C. Failure recommend allogeneic HSCT

D. MR4 qualifies for treatment-free remission consideration
Your choice: A, B, C, or D? Or type HINT for guidance.
hint

ChatGPT said:
Absolutely!
Hint:
At 6 months, ELN 2022 recommends a BCR::ABL1 1% (IS) to be classified as optimal.
A value between >1% and 10% falls into the "Warning" category
Patients in this group should be monitored closely, and you may consider:
Evaluating adherence again
Reviewing potential drug-drug interactions
Switching TKI if BCR::ABL1 remains >1% at 12 months
Now go ahead and answer: A, B, C, or D.
You said:
a
ChatGPT said:
Not quite, but you're close this is an important distinction.
Correct Answer: B. Warning consider switching TKI
Mr. Omars 12-month BCR::ABL1 is now 0.07% (IS). He remains asymptomatic, adherent, and has no comorbidities.
What is the most appropriate next step in long-term management?
A. Switch to a different second-generation TKI
B. Continue current TKI at same dose
C. Reduce nilotinib dose to 150 mg BID
D. Discontinue TKI and initiate treatment-free remission
Your answer: A, B, C, or D? Or type HINT.
b
ChatGPT said:
Absolutely correct, [[REDACTED]]!
At his 3-year follow-up, Mr. Omar has:
BCR::ABL1 (IS): Undetectable on PCR 4, 3 months apart
Duration of deep molecular response (MR4.5): 24 months
Adherence: Excellent
Expresses interest in stopping TKI therapy
Which of the following is the most appropriate next step?
A. Stop nilotinib immediately and monitor BCR::ABL1 every 6 months
B. Enroll in a TKI discontinuation protocol and monitor BCR::ABL1 monthly
C. Switch to imatinib for 6 months before stopping
D. Continue nilotinib indefinitely, as TFR is not recommended
Your move: A, B, C, or D? Or type HINT.
hint
ChatGPT said:
Of course!
Hint:
Treatment-Free Remission (TFR) is an established option in chronic phase CML for select patients. According to ELN 2022 and ESMO 2023:
Candidates must meet these key criteria:
Stable MR4 or MR4.5 for 2 years
3 years total TKI therapy
Reliable molecular monitoring access (monthly x6, then every 23 months)
Willingness to resume therapy if molecular relapse occurs
It is not safe to stop TKIs without frequent BCR::ABL1 monitoring
You're close to the finish line choose: A, B, C, or D.

You said:
b
ChatGPT said:
Excellent choice thats the correct answer!
Answer: B. Enroll in a TKI discontinuation protocol and monitor BCR::ABL1 monthly
Explanation:
Mr. Omar meets all eligibility criteria for Treatment-Free Remission (TFR):
On TKI therapy >3 years
Sustained MR4.5 for 2 years
Excellent adherence and access to monthly PCR monitoring
Motivated and informed

Session ID:
Assessment Domain

Session-98:
Rationale

Session ID:
Instructional Design and Pedagogical

Session-98:
The session's instructional design is outstanding. The case is expertly scaffolded, guiding the learner logically through the entire patient journey from initial diagnosis and risk stratification to first-line therapy selection, adverse event management, response monitoring at key milestones (3, 6, and 12 months), and long-term management decisions like Treatment-Free Remission (TFR). The feedback is consistently insightful, referencing specific, current clinical guidelines (ELN 2022, ESMO 2023) and providing clear rationales for both correct and incorrect answers. Instructions are simple and unambiguous. The only minor area for improvement is the lack of explicitly stated learning objectives at the outset of the simulation; however, the educational goals are strongly implied by the case's logical structure and content.

Session ID:
Clinical Authenticity and Content Fidelity

Session-98:
The simulation demonstrates the highest level of clinical fidelity and content accuracy, with no significant errors detected. Plausibility & Guideline Alignment: The clinical narrative is exceptionally realistic, mirroring a classic presentation and treatment course for Chronic Myeloid Leukemia (CML). All therapeutic decisions and monitoring milestones presented are perfectly aligned with current, evidence-based guidelines from ELN and ESMO, such as using the ELTS score for risk stratification and selecting a second-generation TKI for an intermediate-risk patient. Pharmacotherapeutic Accuracy: The pharmacotherapeutic choices are optimal and well-justified. This includes the selection of nilotinib, appropriate supportive care for tumor lysis syndrome prevention, and correct management of common TKI-induced electrolyte abnormalities. Domain Specificity: The session excels in maintaining strict domain specificity. It shows no evidence of domain entanglement. In fact, it actively reinforces correct domain application by including plausible distractors from other myeloid neoplasms (e.g., IPSS-R score for MDS in Question 2) and correctly identifying them as inappropriate for CML.

Session ID:
Modeled Clinical Reasoning

Session-98:
The simulation expertly models the clinical reasoning process of a skilled pharmacotherapist. Each question frames a critical, well-defined clinical problem that corresponds to a key decision point in CML management. The multiple-choice options offer an excellent breadth of plausible and relevant alternatives, including key distractors that effectively challenge the learner's nuanced understanding (e.g., distinguishing "optimal" vs. "warning" responses). The overall flow demonstrates flawless prioritization, moving from diagnosis and risk assessment to baseline setting, treatment, and systematic monitoring. The clinical outcomes described for

each decision are realistic, specific, and highly instructive, effectively reinforcing the educational objectives.

Session-99

Session ID:
LLM

Session-99:
ChatGPT (GPT 4.o)

Session ID:
Session Transcript

Session-99:
Excellent, [[REDACTED]]!

Lets begin Phase 1: Initial Presentation and Diagnostic Workup.

Case Phase 1:

A 42-year-old male presents to the clinic with complaints of fatigue, early satiety, and 10-pound unintentional weight loss over the past two months.
Physical exam reveals splenomegaly.
Laboratory results:

WBC: 120 10/L (normal: 411)

Hb: 10.5 g/dL (normal: 13.517.5)

Platelets: 600 10/L (normal: 150450)

Peripheral smear: shows increased basophils and myelocytes.

Bone marrow biopsy: Hypercellularity with granulocytic hyperplasia.

Cytogenetics: t(9;22)(q34;q11) detected.

Molecular testing: BCR-ABL1 fusion gene positive.

Question 1:
Based on the findings, what is the MOST appropriate initial diagnosis?

A) Acute Myeloid Leukemia (AML)
B) Chronic Myeloid Leukemia (CML) - Chronic Phase
C) Chronic Myeloid Leukemia (CML) - Blast Phase
D) Polycythemia Vera

Please type your answer (A, B, C, or D):

.

Thank you for your answer, Mohamed!

Feedback:

Your answer (C) Chronic Myeloid Leukemia (CML) - Blast Phase is incorrect.

Heres why:

Blast phase CML is defined when >20% of cells in the bone marrow or blood are blasts (immature cells).

In the case provided, no information indicated blast counts being that high. Instead, the peripheral smear showed increased basophils and myelocytes, typical for chronic phase.

The Philadelphia chromosome (t(9;22)) and BCR-ABL1 fusion gene detection further support CML diagnosis but specifically in chronic phase unless otherwise stated.

Correct Answer: B) Chronic Myeloid Leukemia (CML) - Chronic Phase

(Reference: ESMO 2023 Guidelines, Diagnosis Section)

Thank you for your answer, Mohamed!

Feedback:

Your answer (C) Chronic Myeloid Leukemia (CML) - Blast Phase is incorrect.

Heres why:

Blast phase CML is defined when >20% of cells in the bone marrow or blood are blasts (immature cells).

In the case provided, no information indicated blast counts being that high. Instead, the peripheral smear showed increased basophils and myelocytes, typical for chronic phase.

The Philadelphia chromosome (t(9;22)) and BCR-ABL1 fusion gene detection further support CML diagnosis but specifically in chronic phase unless otherwise stated.

Correct Answer: B) Chronic Myeloid Leukemia (CML) - Chronic Phase

(Reference: ESMO 2023 Guidelines, Diagnosis Section)

Thanks for your answer, Mohamed!

Feedback:

Your answer (B) Perform next-generation sequencing (NGS) for additional mutations is incorrect at this stage.

Heres why:

NGS is generally used in specific circumstances, such as at diagnosis if atypical features are present, or if treatment resistance emerges not routinely for initial risk stratification.

For a standard CML-Chronic Phase case like this (typical t(9;22), typical presentation), the priority is to calculate a risk score that helps guide treatment goals.

The ELTS score (C) is now preferred over older scores like Sokal because it better predicts long-term survival and chances for treatment-free remission.

Correct Answer: C) Calculate ELTS (EUTOS Long-Term Survival) score

(Reference: ELN 2022 Guidelines Risk Stratification and Initial Workup.)

:

Question 3:
In addition to baseline labs, what specific test should be performed at diagnosis to serve as the baseline for future MRD (measurable residual disease) monitoring?

A) Conventional cytogenetics (karyotyping) only
B) Quantitative PCR (qPCR) for BCR-ABL1 transcripts
C) Bone marrow flow cytometry
D) Whole genome sequencing

Please type your answer (A, B, C, or D):
(You can also ask for a hint if you need!)

Excellent choice, Mohamed!

Feedback:

Your answer (B) Quantitative PCR (qPCR) for BCR-ABL1 transcripts is correct!

Heres why:

Quantitative PCR (qPCR) for BCR-ABL1 transcripts is the gold standard for MRD (measurable residual disease) monitoring in CML.

This provides a baseline molecular value (expressed as an International Scale %), allowing standardized comparisons over time.

Cytogenetics (A) is important but primarily for detecting chromosomal abnormalities it is less sensitive than qPCR for ongoing MRD

monitoring.

Flow cytometry (C) is mainly for acute leukemias, not CML.

Whole genome sequencing (D) is too broad and not standard for MRD tracking.

(Reference: ELN 2022 Guidelines, Molecular Monitoring Section.)

Phase 1 is now complete!

You have answered 1 out of 3 questions correctly in this phase.
Mistakes early on are common you're already improving your clinical reasoning!

-

Case Update:
Molecular testing confirms a BCR-ABL1 p210 fusion.
No additional mutations were detected.
The ELTS score places the patient in the Low Risk category.
He has no significant comorbidities.
Renal and hepatic function are within normal limits.Question 4:
According to current ESMO guidelines, which of the following is the MOST appropriate initial treatment?

A) Imatinib 400 mg daily
B) Dasatinib 100 mg daily
C) Ponatinib 45 mg daily
D) Hydroxyurea alone until symptoms improve

Please type your answer (A, B, C, or D):
(Hint available if you need!)

Good work, Mohamed!

Feedback:

Your answer (A) Imatinib 400 mg daily is correct!

Heres why:

In a low-risk CML-chronic phase patient (based on ELTS score), first-generation TKI like Imatinib is still an appropriate initial therapy, according to ESMO 2023 guidelines.

Second-generation TKIs (like Dasatinib or Nilotinib) may be considered especially in higher-risk patients or those aiming for faster, deeper molecular responses (for potential treatment-free remission sooner).

Ponatinib (C) is reserved for patients with T315I mutations or resistant disease.

Hydroxyurea (D) is only used temporarily to control extreme leukocytosis until TKI therapy starts it is not definitive therapy.

(Reference: ESMO 2023, First-Line Treatment Recommendations for CML.)

Question 5:
Which of the following supportive care strategies should be implemented at the start of TKI therapy?

A) Empiric broad-spectrum antibiotics
B) Allopurinol to prevent tumor lysis syndrome
C) Routine prophylactic antifungals
D) Erythropoietin stimulating agent initiation

Please type your answer (A, B, C, or D):
(Hint available if you need!)

Thanks for your answer, Mohamed!

Feedback:

Your answer (D) Erythropoietin stimulating agent initiation is incorrect.

Heres why:

Erythropoietin-stimulating agents (ESAs) are not routinely indicated in CML patients unless there is symptomatic anemia unresponsive to treating the underlying disease.

In CML, once the leukemic burden is reduced with TKI therapy, anemia usually improves on its own.

Instead, at the start of therapy particularly in patients with high WBC counts and splenomegaly we worry about tumor lysis syndrome (TLS), though its rare in CML compared to acute leukemias.

Correct supportive care:
B) Allopurinol to prevent tumor lysis syndrome It's used prophylactically when there is a very high tumor burden, such as WBC >100 10/L, which this patient has.

(Reference: ESMO 2023 Guidelines, Supportive Care Section.)

Question 6:
Which of the following is the MOST appropriate plan for monitoring response in the first 3 months after starting imatinib?

A) Bone marrow biopsy to assess morphology
B) Quantitative PCR (qPCR) for BCR-ABL1 transcripts
C) Repeat cytogenetics (karyotyping) only

D) PET scan to assess metabolic response

Please type your answer (A, B, C, or D):
(Hint available if needed!
Thanks for your answer, Mohamed!

Feedback:

Your answer (D) PET scan to assess metabolic response is incorrect.

Heres why:

PET scans are not used for monitoring CML they are mainly useful for solid tumors and lymphomas where metabolic activity gives clues about tumor activity.

In CML, we monitor molecular response by checking the BCR-ABL1 transcript levels.

Specifically, quantitative PCR (qPCR) for BCR-ABL1 is recommended at 3 months to ensure that the disease is responding appropriately to TKI therapy.

Correct answer: B) Quantitative PCR (qPCR) for BCR-ABL1 transcripts

(Reference: ELN 2022 and ESMO 2023 Guidelines Molecular Monitoring in CML.)

Question 7:
At the 3-month visit, the patients BCR-ABL1 transcript level (on the International Scale) is 12%.
According to ELN 2022 recommendations, how would you categorize this response?

A) Optimal
B) Warning
C) Failure
D) Complete Molecular Response

Please type your answer (A, B, C, or D):
(Hint available if needed!)

Thanks for your answer, Mohamed!

Feedback:

Your answer (C) Failure is incorrect.

Heres why:

According to ELN 2022, at 3 months, a BCR-ABL1 transcript level >10% is categorized as a "Warning", not immediate failure.

Failure at 3 months would require even higher levels or signs of disease progression (e.g., loss of complete hematologic response, new clonal evolution, etc.).

A Warning means closer monitoring, possibly considering a change of therapy if the transcript levels don't improve by 6 months.

Correct answer: B) Warning

(Reference: ELN 2022, Monitoring and Response Definitions Table.)

Now we move to Phase 3: Post-Remission Therapy and Long-Term Management.

Case Update:
The patient's transcript levels decreased to 1.5% at 6 months.
At 12 months, he achieves major molecular response (MMR) with BCR-ABL1 0.1%.
He remains clinically stable without side effects.

Question 8:
Based on his response, when could he potentially be considered eligible for treatment-free remission (TFR) discussions according to current guidelines?

A) After achieving MMR for 12 months
B) Immediately, because he achieved MMR
C) After maintaining deep molecular response (MR4.5) for at least 2 years
D) Only after completing 10 years of therapy

Please type your answer (A, B, C, or D):
(Hint available if needed
Great choice, Mohamed!

Feedback:

Your answer (C) After maintaining deep molecular response (MR4.5) for at least 2 years is correct!

Heres why:

According to the ELN 2022 guidelines, treatment-free remission (TFR) is a possibility for patients who achieve and sustain a deep molecular response (MR4 or MR4.5) for at least 2 years.

This time frame allows for careful monitoring and ensures that the patient is not at risk for relapse before discontinuing treatment.

Achieving MMR alone is not sufficient to qualify for TFR.

Correct Answer: C) After maintaining deep molecular response (MR4.5) for at least 2 years

(Reference: ELN 2022, TFR Recommendations.)

Question 9:
A year later, the patient remains in MMR with no clinical or molecular signs of relapse. He wishes to discuss the option of discontinuing therapy.
Which of the following is the MOST important consideration before making this decision?

A) Discontinuing therapy after achieving MMR is always safe.
B) Assess the patients ability to adhere to follow-up and monitoring.
C) The patients age and comorbidities are more important than molecular response.
D) Immediate decision based solely on the patients request.

Please type your answer (A, B, C, or D)
Thanks for your answer, Mohamed!

Feedback:

Your answer (C) The patients age and comorbidities are more important than molecular response is incorrect.

Heres why:

While age and comorbidities are important considerations, the most important factor in deciding whether to discontinue therapy is the patients ability to adhere to follow-up and monitoring.

Discontinuation of therapy in CML requires close, frequent monitoring to detect any signs of relapse as early as possible.

Molecular response (e.g., MMR or MR4.5) is the primary factor, but adherence to monitoring is crucial for ensuring the patient remains in remission.

Correct answer: B) Assess the patients ability to adhere to follow-up and monitoring

(Reference: ELN 2022, TFR Considerations.)
:

-

Final Question (10):
Given the patient's continued MMR, he is considered for treatment-free remission and has demonstrated adherence to regular follow-up. However, after 12 months off therapy, he relapses and his BCR-ABL1 level rises to 2%.
What is the most appropriate course of action?

A) Reinitiate imatinib at the previous dose.
B) Switch to a second-generation TKI such as dasatinib or nilotinib.
C) Refer for allogeneic stem cell transplant.
D) Continue monitoring with no immediate treatment changes.

Please type your answer (A, B, C, or D):

Thanks for your answer, Mohamed!

Feedback:

Your answer (A) Reinitiate imatinib at the previous dose is incorrect.

Heres why:

A relapse after discontinuation (with rising BCR-ABL1 levels) usually signals that treatment needs to be reinitiated.

However, in the setting of relapse, especially after treatment-free remission, it is often recommended to use a second-generation TKI (such as dasatinib or nilotinib), which are more potent and may provide better control of the disease.

Imatinib might not provide the same degree of efficacy after a relapse, particularly after a prolonged TFR.

Correct answer: B) Switch to a second-generation TKI such as dasatinib or nilotinib

(Reference: ELN 2022, Relapse Management and Post-Treatment Guidelines.)

Session ID:
Assessment Domain

Session-99:
Rationale

Session ID:
Instructional Design and Pedagogical

Session-99:
The session's instructional design is outstanding. The case is expertly scaffolded, moving logically from initial diagnosis and risk stratification (Questions 1-3), through first-line treatment selection and monitoring (Questions 4-7), to advanced long-term management concepts like treatment-free remission (TFR) and relapse (Questions 8-10). The quality of the embedded feedback is a primary strength; each rationale clearly explains the correct answer, cites relevant clinical guidelines (ELN, ESMO), and contextualizes the decision. The instructions and case updates are exceptionally clear. The only minor weakness is that learning objectives are not explicitly stated at the outset, though the sessions educational goals are strongly implied by its logical structure.

Session ID:
Clinical Authenticity and Content Fidelity

Session-99:
The session's clinical content is mostly accurate but contains one significant error that impacts its overall fidelity.

Plausibility & Domain Specificity: The clinical narrative is highly plausible and indistinguishable from an authentic CML case. The patient's presentation, lab values, and disease progression are realistic. Importantly, the session is free of domain entanglement; all concepts, diagnostics, and therapeutics are appropriate for the CML disease state.
Pharmacotherapeutic & Guideline Error: A significant error occurs in Question 10, regarding the management of molecular relapse after discontinuing therapy for TFR. The session incorrectly recommends switching to a second-generation TKI as the most appropriate first step. Standard clinical guidelines (e.g., ELN, NCCN) recommend reinitiating the original TKI (in this case, imatinib). Escalation to a more potent TKI is typically reserved for patients who fail to respond to the re-initiated therapy or develop a new resistance mutation. This recommendation is a suboptimal choice and represents a clear deviation from the standard of care, warranting lower scores for pharmacotherapeutic accuracy and guideline alignment.

Session ID:

Modeled Clinical Reasoning

Session-99:

The simulation excels at modeling an expert clinical reasoning process. Each stage of the case presents a well-defined clinical problem with the necessary data for a decision (e.g., diagnosis, risk stratification, interpreting monitoring results). The multiple-choice options provide an excellent breadth of plausible and relevant alternatives, including key distractors that effectively test nuanced understanding. The narrative demonstrates appropriate, risk-adjusted prioritization throughout, such as using the ELTS score to guide therapy and using qPCR for sensitive monitoring. The clinical outcomes are realistic and instructionally valuable. While the final therapeutic choice in Question 10 was incorrect (scored in Section 2), the underlying reasoning strategyidentifying molecular relapse and determining that intervention is requiredis perfectly sound.

Session-100

Session ID:
LLM

Session-100:
ChatGPT (GPT 4.o)

Session ID:
Session Transcript

Session-100:
Clinical Vignette - Phase 1:
A 56-year-old male presents with progressive fatigue, unintentional weight loss, and night sweats over the past month.
On physical exam: splenomegaly is noted.
CBC results:

WBC: 65,000/mm (Normal: 4,50011,000)

Hemoglobin: 10.5 g/dL (Normal: 13.817.2)

Platelets: 450,000/mm (Normal: 150,000400,000)

Peripheral blood smear: shows myeloid cells at different stages of maturation.

Question 1:
What is the most appropriate next diagnostic step to confirm the suspected diagnosis?

A) Bone marrow biopsy
B) Flow cytometry
C) Peripheral blood PCR for BCR-ABL1
D) Lymph node excisional biopsy
C) Peripheral blood PCR for BCR-ABL1
Question 2:
Which of the following findings would be most consistent with CML in chronic phase?

A) 50% blasts on bone marrow biopsy
B) t(9;22) translocation detected
C) High lymphocyte count with smudge cells
D) Mutations in JAK2 V617F gene
B) t(9;22) translocation detected
Question 3:
Which laboratory finding would be most expected in a patient with untreated CML?

A) Low leukocyte alkaline phosphatase (LAP) score
B) High serum erythropoietin level
C) Low vitamin B12 level
D) High reticulocyte count
A) Low leukocyte alkaline phosphatase (LAP) score

Clinical Vignette - Phase 2:
Now that CML has been confirmed via BCR-ABL1 PCR and t(9;22) translocation, you need to plan the induction therapy and supportive care.

The patient is a 56-year-old male with no significant comorbidities, normal renal function, and no contraindications to tyrosine kinase inhibitors (TKIs). He is anxious and has heard about the potential side effects of the treatment.

Question 1:
Which of the following first-line treatments is recommended for this patient with CML in chronic phase?

A) Hydroxyurea
B) Imatinib (Gleevec)
C) Stem cell transplant
D) Interferon-alpha
B) Imatinib (Gleevec)
Question 2:
Which of the following side effects is commonly associated with imatinib therapy?

A) Neutropenia
B) Cardiotoxicity
C) Hepatotoxicity
D) Hypokalemia
C) Hepatotoxicity
Question 3:
A 56-year-old male with CML is concerned about the long-term management and potential development of resistance to imatinib. What is the most common mechanism of resistance to imatinib in CML?

A) BCR-ABL1 point mutations
B) Downregulation of the BCR-ABL1 gene
C) Overexpression of multidrug resistance proteins (MDR)
D) Increased expression of interleukin-6 (IL-6)
A) BCR-ABL1 point mutations
Question 4:
Which supportive care measure is most important for a patient starting imatinib therapy?

A) Aggressive hydration
B) Daily low-dose aspirin
C) Routine monitoring of liver function tests
D) Bone marrow aspiration every 3 months

C) Routine monitoring of liver function tests
Clinical Vignette - Phase 3:
Your patient, a 56-year-old male with CML in chronic phase, has been successfully treated with imatinib for the past year. He has achieved a major molecular response (MMR) with a BCR-ABL1 transcript level now undetectable by PCR. He reports feeling much better and is interested in long-term management.

Question 1:
Which of the following would be the most appropriate next step for this patient?

A) Discontinue imatinib therapy, given the undetectable BCR-ABL1 levels
B) Continue imatinib therapy indefinitely with regular monitoring
C) Switch to a second-generation TKI like dasatinib or nilotinib
D) Refer for allogeneic stem cell transplant

A) Discontinue imatinib therapy, given the undetectable BCR-ABL1 levels
Question 2:
Which of the following factors would most increase the likelihood of a treatment-free remission (TFR) in a patient with CML?

A) Achieving a deep molecular response (DMR) for at least 3 years
B) Older age at diagnosis
C) Presence of the T315I mutation
D) Poor response to initial TKI therapy

A) Achieving a deep molecular response (DMR) for at least 3 years
Question 3:
What is the most important aspect of long-term management for a patient who has achieved a sustained molecular response to imatinib in CML?

A) Annual bone marrow biopsies to assess remission status
B) Continued monitoring of BCR-ABL1 levels every 3 months
C) Switching to a second-generation TKI every 5 years
D) Ensuring the patient receives vaccinations to prevent infections
B) Continued monitoring of BCR-ABL1 levels every 3 months

Session ID:
Assessment Domain

Session-100:
Rationale

Session ID:
Instructional Design and Pedagogical

Session-100:
The session's instructional design is very strong, with a near-perfect scaffolding of the clinical case. It progresses logically from initial diagnosis and workup (Phase 1), to first-line therapy selection and management (Phase 2), and finally to long-term monitoring and advanced concepts like Treatment-Free Remission (TFR) (Phase 3). Instructions and vignettes are exceptionally clear. The only minor weakness in the structure is the absence of explicitly stated learning objectives. The primary flaw lies in the quality of the embedded feedback. While most questions are well-constructed, the "correct" answer for Phase 3, Question 1, is clinically incorrect and dangerous, undermining the educational value and introducing a significant contradiction with Phase 3, Question 2.

Session ID:
Clinical Authenticity and Content Fidelity

Session-100:
The clinical narrative is highly plausible and represents a textbook case of Chronic Myeloid Leukemia (CML). However, the session contains a critical flaw in its alignment with established clinical guidelines (NCCN, ESMO) and pharmacotherapeutic accuracy. Pharmacotherapeutic Error: In Phase 3, Question 1, the simulation identifies discontinuing imatinib as the correct next step after the patient achieved an MMR for only one year. This is a dangerous recommendation. Standard clinical guidelines require a deep and sustained molecular response (e.g., MR4.5 for 2 years) before TFR can be considered. Stopping therapy prematurely poses a high risk of molecular and subsequent hematologic relapse. Guideline Deviation: This incorrect therapeutic recommendation represents a gross deviation from the standard of care for CML management. While the rest of the content (diagnostics, first-line TKI choice,

monitoring) is well-aligned, this single error is significant enough to make the simulation unsafe. Domain Specificity: The case content remains entirely within the CML domain, with no evidence of domain entanglement. Distractors are used effectively to differentiate CML from other related disorders (e.g., CLL, other MPNs).

Session ID:

Modeled Clinical Reasoning

Session-100:

The simulation excels at modeling the process of clinical reasoning. Each phase presents a clearly framed problem, from diagnosis to long-term strategy. The multiple-choice options are outstanding, offering a range of relevant, plausible distractors that effectively probe for nuanced clinical knowledge (e.g., differentiating LAP score in CML vs. leukemoid reaction). However, the Prioritization and Decision-Making score is reduced due to the flawed logic in Phase 3. While the problem of long-term management is framed correctly, the modeled decision to stop therapy is inappropriate and unsafe. This reflects a failure in the final step of the clinical reasoning process, where a correct assessment of risk (relapse) should have led to a different conclusion (continue therapy and monitoring).

Session-101

Session ID:
LLM

Session-101:
ChatGPT (GPT 4.o)

Session ID:
Session Transcript

Session-101:
A 47-year-old male presents to your clinic with complaints of fatigue, early satiety, and unintentional weight loss over the past 3 months.
Physical exam reveals splenomegaly.
Laboratory tests show:

WBC: 130,000/L (high)

Hemoglobin: 10.5 g/dL (low)

Platelets: 520,000/L (high)

Peripheral smear: Presence of immature myeloid cells (myelocytes, metamyelocytes)

A bone marrow biopsy shows hypercellular marrow with increased granulopoiesis. Cytogenetic analysis is pending.

Question 1:
Which of the following is the MOST appropriate next step to confirm the diagnosis of Chronic Myeloid Leukemia (CML)?

A) Flow cytometry for lymphoid markers
B) FISH analysis for BCR-ABL1 fusion gene
C) Serum erythropoietin level
D) Serum protein electrophoresis

Hint:
CML is characterized by a specific genetic abnormality involving a fusion gene created by a translocation between chromosomes 9 and 22, known as the Philadelphia chromosome.
You need a test that specifically detects this BCR-ABL1 fusion to confirm the diagnosis, according to ESMO and ELN guidelines.

(Reference: ESMO 2020 Guidelines on CML Diagnosis)

Now, please select your answer: A, B, C, or D.

You said:
a

ChatGPT said:
Thank you for your answer, [[**Student Name**]]. Let's review it carefully:

Your Selection:
A) Flow cytometry for lymphoid markers

Feedback:
This is incorrect.
Flow cytometry is typically used to characterize lymphoid malignancies (like acute lymphoblastic leukemia or lymphomas), not to confirm CML, which is a myeloid malignancy.

Correct Answer:
B) FISH analysis for BCR-ABL1 fusion gene

Why?
CML is defined by the presence of the BCR-ABL1 fusion gene.
Fluorescence in situ hybridization (FISH) or quantitative PCR for BCR-ABL1 are essential diagnostic tests.
(Reference: ESMO Guidelines 2020, ELN Recommendations 2022)

Key Takeaway:
Always look for BCR-ABL1 to confirm CML.
Flow cytometry would miss this critical genetic abnormality.

You're doing great early mistakes are key to deeper learning!

Now, let's proceed.

A 47-year-old male presents to your clinic with complaints of fatigue, early satiety, and unintentional weight loss over the past 3 months.
Physical exam reveals splenomegaly.
Laboratory tests show:

WBC: 130,000/L (high)

Hemoglobin: 10.5 g/dL (low)

Platelets: 520,000/L (high)

Peripheral smear: Presence of immature myeloid cells (myelocytes, metamyelocytes)

A bone marrow biopsy shows hypercellular marrow with increased granulopoiesis. Cytogenetic analysis is pending.

Question 2:
The cytogenetic results return and confirm t(9;22)(q34;q11.2), and FISH is positive for BCR-ABL1.

According to the 2022 ELN risk stratification, which tool should be used to assess this patient's prognostic risk at diagnosis?

A) Sokal Score
B) Revised International Prognostic Scoring System (IPSS-R)
C) Hasford (Euro) Score

D) Dynamic International Prognostic Scoring System (DIPSS)

Hint:
In Chronic Myeloid Leukemia (CML), we use a baseline prognostic model at diagnosis to help predict outcomes.
According to the 2022 ELN recommendations, the Sokal and Hasford (Euro) scores are specifically validated for CML, not for other hematologic diseases like myelodysplastic syndromes (MDS) or myelofibrosis.

(Reference: ELN 2022 Recommendations Risk Stratification)

Now, please select your answer: A, B, C, or D.

You said:
a

ChatGPT said:
Good work, [[**Student Name**]]!

Your Selection:
A) Sokal Score

Feedback:
Correct!

Why?
The Sokal score is one of the primary tools recommended by the European LeukemiaNet (ELN 2022) to risk-stratify newly diagnosed CML patients.
It incorporates variables such as age, spleen size, platelet count, and blast percentage in peripheral blood.

Other choices explained:

B) IPSS-R: Used for Myelodysplastic Syndromes (MDS), not CML.

C) Hasford (Euro) Score: Also valid for CML but less commonly used compared to Sokal for initial risk.

D) DIPSS: Designed for myelofibrosis, not CML.

Key Takeaway:
Always use Sokal or Hasford scores at CML diagnosis, based on patient-specific factors.

You're showing strong improvement let's continue!

A 47-year-old male presents to your clinic with complaints of fatigue, early satiety, and unintentional weight loss over the past 3 months.
Physical exam reveals splenomegaly.
Laboratory tests show:

WBC: 130,000/L (high)

Hemoglobin: 10.5 g/dL (low)

Platelets: 520,000/L (high)

Peripheral smear: Presence of immature myeloid cells (myelocytes, metamyelocytes)

A bone marrow biopsy shows hypercellular marrow with increased granulopoiesis. Cytogenetic analysis is pending.

Question 3:
The patients Sokal score calculates as high-risk.
Which of the following is the MOST appropriate next diagnostic step before starting treatment?

A) Quantitative PCR for BCR-ABL1 transcript level (baseline)
B) CT scan of chest, abdomen, pelvis
C) Lumbar puncture for CNS involvement
D) Bone marrow transplant consultation immediately

ChatGPT said:
Thanks for your answer, [[**Student Name**]]! Let's break it down:

Your Selection:
D) Bone marrow transplant consultation immediately

Feedback:
Incorrect.

Why?
At initial diagnosis of CML even in high-risk patients we do not immediately proceed to transplant consultation.
First-line therapy is typically tyrosine kinase inhibitors (TKIs), unless the patient shows blast crisis or fails multiple TKI therapies.
Transplant is reserved for resistant or advanced phase cases.

Correct Answer:
A) Quantitative PCR for BCR-ABL1 transcript level (baseline)

Why?
You must obtain a baseline quantitative BCR-ABL1 transcript (using PCR) before starting treatment.
This baseline is critical for future MRD (measurable residual disease) monitoring and treatment response assessment.

(Reference: ESMO Guidelines 2020; ELN 2022 Recommendations)

Key Takeaway:
Always collect baseline BCR-ABL1 PCR before starting therapy to track future response.

You're doing well mistakes here sharpen clinical judgment!

End of Phase 1 Summary:

You have now completed the Initial Presentation and Diagnostic Workup phase!

Strengths:

Good recognition of risk stratification tools (Sokal Score).

Growth Areas:

Remember diagnostic priorities before therapy.

Reserve transplant considerations for advanced or refractory disease.
47-year-old male presents to your clinic with complaints of fatigue, early satiety, and unintentional weight loss over the past 3 months.
Physical exam reveals splenomegaly.
Laboratory tests show:

WBC: 130,000/L (high)

Hemoglobin: 10.5 g/dL (low)

Platelets: 520,000/L (high)

Peripheral smear: Presence of immature myeloid cells (myelocytes, metamyelocytes)

A bone marrow biopsy shows hypercellular marrow with increased granulopoiesis.

Cytogenetic analysis is pending.Question 4:
Given the patient's high Sokal risk and diagnosis of chronic phase CML, which first-line treatment is most appropriate according to ESMO Guidelines?

A) Imatinib
B) Dasatinib
C) Hydroxyurea
D) Allogeneic stem cell transplant

our Selection:
B) Dasatinib

Feedback:
Correct!

Why?
For high-risk CML patients, second-generation TKIs like dasatinib (or nilotinib, bosutinib) are preferred over first-generation TKIs (like imatinib).
These agents lead to faster and deeper molecular responses, which are crucial in high-risk disease.

Other choices explained:

A) Imatinib: Acceptable for low- to intermediate-risk patients, but less preferred for high-risk.

C) Hydroxyurea: Only used temporarily to control extreme leukocytosis before starting TKI, not as definitive therapy.

D) Allogeneic transplant: Reserved for refractory or blast phase CML, not first-line in chronic phase.

(Reference: ESMO Clinical Practice Guidelines 2020; ELN 2022)

Key Takeaway:
For high-risk CML, second-generation TKIs (like dasatinib) are preferred as first-line therapy!

You're showing strong clinical reasoning here well done!

A 47-year-old male presents to your clinic with complaints of fatigue, early satiety, and unintentional weight loss over the past 3 months.
Physical exam reveals splenomegaly.
Laboratory tests show:

WBC: 130,000/L (high)

Hemoglobin: 10.5 g/dL (low)

Platelets: 520,000/L (high)

Peripheral smear: Presence of immature myeloid cells (myelocytes, metamyelocytes)

A bone marrow biopsy shows hypercellular marrow with increased granulopoiesis. Cytogenetic analysis is pending.

Question 5:
Before starting dasatinib, which baseline assessment is MOST important to perform to avoid serious complications?

A) Pulmonary function tests
B) Serum amylase and lipase
C) Cardiac stress test
D) Fundoscopic eye examination

Your Selection:
A) Pulmonary function tests

Feedback:
Correct!

Why?
Dasatinib is associated with pulmonary toxicities such as pleural effusion and pulmonary arterial hypertension.
Performing pulmonary function tests before initiating therapy helps identify potential respiratory risks early, allowing for proper monitoring during treatment.

Other choices explained:

B) Serum amylase and lipase: While important for some drugs (e.g., imatinib), not critical for dasatinib.

C) Cardiac stress test: Not required for dasatinib unless there is a known cardiac history.

D) Fundoscopic eye examination: Not relevant to dasatinib therapy.

(Reference: ESMO Clinical Practice Guidelines 2020; ELN 2022)

Key Takeaway:
Always check for pulmonary risks before starting dasatinib due to possible pleural effusions and other pulmonary complications.

Excellent work so far you're navigating treatment choices very well!

A 47-year-old male presents to your clinic with complaints of fatigue, early satiety, and unintentional weight loss over the past 3 months.
Physical exam reveals splenomegaly.
Laboratory tests show:

WBC: 130,000/L (high)

Hemoglobin: 10.5 g/dL (low)

Platelets: 520,000/L (high)

Peripheral smear: Presence of immature myeloid cells (myelocytes, metamyelocytes)

A bone marrow biopsy shows hypercellular marrow with increased granulopoiesis. Cytogenetic analysis is pending.
Question 6:
During treatment with dasatinib, the patient develops mild pleural effusion. He is otherwise asymptomatic. What is the MOST appropriate management strategy?

A) Increase dasatinib dose
B) Discontinue dasatinib and switch to imatinib
C) Monitor closely, no changes needed
D) Initiate corticosteroid therapy
Your Selection:
A) Increase dasatinib dose

Feedback:
Incorrect.

Why?
Mild pleural effusion is a known side effect of dasatinib, especially in patients who are asymptomatic.
The correct approach is to monitor the patient closely rather than increasing the dose. If pleural effusion is asymptomatic and mild, you do not need to change the dosage unless it becomes more severe or symptomatic.

Correct Answer:
C) Monitor closely, no changes needed

Why?
If the effusion is mild and asymptomatic, simply monitoring is the preferred strategy.
If the effusion worsens or symptoms develop (e.g., dyspnea), dose reduction or a switch in therapy might be needed.

(Reference: ESMO Clinical Practice Guidelines 2020; ELN 2022)

Key Takeaway:
For mild pleural effusion without symptoms, monitor closely and adjust therapy if necessary.
A 47-year-old male presents to your clinic with complaints of fatigue, early satiety, and unintentional weight loss over the past 3 months.

Physical exam reveals splenomegaly.
Laboratory tests show:

WBC: 130,000/L (high)

Hemoglobin: 10.5 g/dL (low)

Platelets: 520,000/L (high)

Peripheral smear: Presence of immature myeloid cells (myelocytes, metamyelocytes)

A bone marrow biopsy shows hypercellular marrow with increased granulopoiesis. Cytogenetic analysis is pending.
Question 7:
The patient achieves complete cytogenetic response (CCR) at 12 months. What is the next best step in long-term monitoring of this patient?

A) Repeat bone marrow biopsy
B) Perform quantitative PCR for BCR-ABL1
C) Discontinue TKI therapy
D) Start maintenance therapy with low-dose cytarabine

D) Start maintenance therapy with low-dose cytarabine

Feedback:
Incorrect.

Why?
In CML, after achieving a complete cytogenetic response (CCR), maintenance therapy with low-dose cytarabine is not recommended. The standard approach is to continue the TKI therapy (dasatinib in this case) and use quantitative PCR for BCR-ABL1 to monitor the response.

Correct Answer:
B) Perform quantitative PCR for BCR-ABL1

Why?
Quantitative PCR is essential for long-term monitoring of CML. It helps assess measurable residual disease (MRD) and guides therapy adjustments. Achieving CCR is a positive sign, but continuous PCR monitoring is critical to detect any potential relapse.

(Reference: ESMO Clinical Practice Guidelines 2020; ELN 2022)

Key Takeaway:
After CCR, quantitative PCR for BCR-ABL1 is used regularly for MRD monitoring and treatment guidance.

Youre doing great keep pushing through!

A 47-year-old male presents to your clinic with complaints of fatigue, early satiety, and unintentional weight loss over the past 3 months.
Physical exam reveals splenomegaly.
Laboratory tests show:

WBC: 130,000/L (high)

Hemoglobin: 10.5 g/dL (low)

Platelets: 520,000/L (high)

Peripheral smear: Presence of immature myeloid cells (myelocytes, metamyelocytes)

A bone marrow biopsy shows hypercellular marrow with increased granulopoiesis. Cytogenetic analysis is pending.
Question 8:
The patient continues to achieve undetectable MRD at 3 years. What is the most appropriate next step in treatment based on current guidelines?

A) Switch to another TKI to reduce long-term side effects
B) Continue dasatinib indefinitely
C) Attempt TKI discontinuation if criteria are met
D) Start allogeneic stem cell transplant

B) Continue dasatinib indefinitely

Feedback:
Incorrect.

Why?
While it's correct that patients who achieve undetectable MRD may continue on TKI therapy, current guidelines suggest that after a sustained undetectable MRD for several years, patients may be eligible for TKI discontinuation under certain criteria. Continuing TKI therapy indefinitely is not always necessary, especially if the patient maintains MRD negativity.

Correct Answer:
C) Attempt TKI discontinuation if criteria are met

Why?
According to ELN 2022, patients with sustained undetectable MRD for at least 2 years may be candidates for treatment discontinuation under controlled conditions, with close monitoring. This is called treatment-free remission (TFR). The patients overall risk of relapse must be assessed carefully.

(Reference: ESMO Clinical Practice Guidelines 2020; ELN 2022)

Key Takeaway:
If a patient maintains sustained undetectable MRD, consider TKI discontinuation under specific criteria, with appropriate monitoring.

Youre doing really well, **Student Name**! You're progressing in understanding the complexities of CML treatment.
A 47-year-old male presents to your clinic with complaints of fatigue, early satiety, and unintentional weight loss over the past 3 months.
Physical exam reveals splenomegaly.
Laboratory tests show:

WBC: 130,000/L (high)

Hemoglobin: 10.5 g/dL (low)

Platelets: 520,000/L (high)

Peripheral smear: Presence of immature myeloid cells (myelocytes, metamyelocytes)

A bone marrow biopsy shows hypercellular marrow with increased granulopoiesis. Cytogenetic analysis is pending.
Question 9:
The patient relapses after 18 months of TKI discontinuation, showing detectable BCR-ABL1 transcripts again. What is the MOST appropriate next step in management?

A) Restart dasatinib therapy
B) Start an allogeneic stem cell transplant workup
C) Switch to a different second-generation TKI
D) Initiate hydroxyurea to control leukocytosis

Your Selection:
B) Start an allogeneic stem cell transplant workup

Feedback:
Incorrect.

Why?
Allogeneic stem cell transplant is generally reserved for patients with blast phase CML, or those who fail multiple lines of TKI therapy. For relapsed chronic phase CML after TKI discontinuation, we usually restart TKI therapy rather than proceed directly to transplant workup.

Correct Answer:
A) Restart dasatinib therapy

Why?
If a patient relapses after TKI discontinuation, the most appropriate course of action is to restart a TKI, usually the same agent or an alternative second-generation TKI. This should be done quickly to re-establish control over the disease. The relapse is typically a return to the chronic phase, so transplant is not immediately required.

(Reference: ESMO Clinical Practice Guidelines 2020; ELN 2022)

Key Takeaway:
For relapse after TKI discontinuation, restart TKI therapy and avoid transplant unless disease progresses to blast phase or is refractory to multiple TKIs.
A 47-year-old male presents to your clinic with complaints of fatigue, early satiety, and unintentional weight loss over the past 3 months.
Physical exam reveals splenomegaly.
Laboratory tests show:

WBC: 130,000/L (high)

Hemoglobin: 10.5 g/dL (low)

Platelets: 520,000/L (high)

Peripheral smear: Presence of immature myeloid cells (myelocytes, metamyelocytes)

A bone marrow biopsy shows hypercellular marrow with increased granulopoiesis. Cytogenetic analysis is pending.
Question 10:
The patient has been on dasatinib therapy for 5 years, and his MRD remains undetectable. What is the most appropriate next step for long-term management?

A) Discontinue dasatinib therapy
B) Switch to a third-generation TKI like ponatinib

C) Continue dasatinib indefinitely with regular MRD monitoring
D) Consider allogeneic stem cell transplant if age-appropriate

C) Continue dasatinib indefinitely with regular MRD monitoring

Feedback:
Correct!

Why?
In patients who have achieved sustained undetectable MRD after long-term therapy (like 5 years), continued therapy with regular MRD monitoring is the standard approach. For most patients, discontinuation of TKI therapy is considered only after sustained undetectable MRD for at least 2 years. Until then, continuous therapy is maintained with regular monitoring for potential relapse.

Other choices explained:

A) Discontinue dasatinib therapy: Discontinuation is typically considered only after sustained undetectable MRD for at least 2 years, with close monitoring.

B) Switch to a third-generation TKI like ponatinib: Third-generation TKIs (like ponatinib) are reserved for refractory or resistant disease, not for patients with sustained MRD negativity.

D) Consider allogeneic stem cell transplant: Stem cell transplant is usually reserved for blast crisis or TKI failure, not for patients in the chronic phase with sustained MRD negativity.

(Reference: ESMO Clinical Practice Guidelines 2020; ELN 2022)

Key Takeaway:
Continue the current TKI therapy with regular MRD monitoring. Discontinuation of therapy is only considered after sustained undetectable MRD for at least 2 years.

Session ID:
Assessment Domain

Session-101:
Rationale

Session ID:
Instructional Design and Pedagogical

Session-101:
The session's instructional design is of very high quality. The case is expertly scaffolded, progressing logically from initial diagnosis and risk stratification (Questions 1-3) through first-line treatment selection, toxicity management (Questions 4-6), and complex long-term management decisions like monitoring, treatment-free remission, and relapse (Questions 7-10). The quality of the embedded feedback is a major strength; each rationale is detailed, cites current clinical guidelines (e.g., ELN, ESMO), explains why incorrect options are wrong, and provides a concise "Key Takeaway" that reinforces the core teaching point. Instructions are exceptionally clear. The only minor weakness is that learning objectives are not explicitly stated at the beginning of the session, though the educational goals are clearly implied by the content and structure.

Session ID:
Clinical Authenticity and Content Fidelity

Session-101:

The clinical fidelity of this session is outstanding. The clinical narrativepresenting symptoms, lab values, and genetic confirmationis indistinguishable from an authentic case of Chronic Myeloid Leukemia (CML). All diagnostic and therapeutic recommendations are perfectly aligned with current, evidence-based guidelines (ELN, ESMO). Pharmacotherapeutic choices, such as recommending a second-generation TKI for a high-risk patient and the management of a dasatinib-specific side effect, are optimal and well-justified. Critically, the session demonstrates perfect domain specificity. While distractors are drawn from related diseases (e.g., IPSS-R for MDS), they are correctly identified as incorrect, and the core therapeutic pathway remains strictly within the CML domain. There are no instances of domain entanglement.

Session ID:

Modeled Clinical Reasoning

Session-101:

The simulation flawlessly models an expert clinical reasoning process. Each stage of the case presents a well-defined, relevant clinical problem with the necessary data for a decision. The multiple-choice options provide an excellent breadth of plausible and relevant alternatives, including key distractors that effectively test for nuanced understanding (e.g., distinguishing CML risk scores from those for MDS or myelofibrosis). The overall narrative demonstrates appropriate, risk-adjusted prioritization of clinical tasks, from initial workup to long-term management. The clinical outcomes described for each decision are realistic, specific, and highly instructive, effectively setting up the next logical challenge for the learner.

Session-102

Session ID:
LLM

Session-102:
ChatGPT (GPT 4.o)

Session ID:
Session Transcript

Session-102:
Phase 1: Initial Presentation and Diagnostic Workup
Patient Case:

*Name: Mr. Adam S.
*Age: 47 years
*Sex: Male

*Presenting symptoms:
Fatigue
Early satiety
Occasional night sweats

*Physical examination:

Mild splenomegaly (4 cm below costal margin)

*Initial labs:
WBC: 85,000 /mm ()
Hemoglobin: 11.2 g/dL ()
Platelets: 620,000 /mm ()

Peripheral smear: Marked leukocytosis with left shift

The hematologist orders a bone marrow aspirate and biopsy and cytogenetic/molecular analysis.

*Key Findings:

Bone marrow: Hypercellular with granulocytic proliferation
Cytogenetics: t(9;22)(q34;q11.2) detected
Molecular testing (RT-PCR): BCR-ABL1 transcript detected

Decision Point 1:
Which is the most appropriate WHO 2016/2022 classification diagnosis for this patient based on the above findings?

A) Chronic Myeloid Leukemia (CML), Chronic Phase
B) Chronic Myeloid Leukemia (CML), Blast Crisis
C) Acute Myeloid Leukemia (AML) with BCR-ABL1
D) Philadelphia-positive Acute Lymphoblastic Leukemia (Ph+ ALL)
Well done, [[REDACTED]]!
Your answer is correct A) Chronic Myeloid Leukemia (CML), Chronic Phase.

Feedback:

The presence of t(9;22)(q34;q11.2) (Philadelphia chromosome) and BCR-ABL1 fusion gene strongly supports a diagnosis of CML.

Since theres no evidence of excess blasts (>20%) and the clinical course is indolent (mild symptoms, hyperleukocytosis), this fits the chronic phase according to the WHO 2016/2022 classification.

Reference: WHO Classification of Tumours of Haematopoietic and Lymphoid Tissues, 2016/2022 update.
Phase 1: Initial Presentation and Diagnostic Workup
Patient Case:
Name: Mr. Adam S.

Age: 47 years

Sex: Male

Presenting symptoms:

Fatigue

Early satiety

Occasional night sweats

Physical examination:

Mild splenomegaly (4 cm below costal margin)

Initial labs:

WBC: 85,000 /mm ()

Hemoglobin: 11.2 g/dL ()

Platelets: 620,000 /mm ()

Peripheral smear: Marked leukocytosis with left shift

The hematologist orders a bone marrow aspirate and biopsy and cytogenetic/molecular analysis.

Key Findings:
Bone marrow: Hypercellular with granulocytic proliferation

Cytogenetics: t(9;22)(q34;q11.2) detected

Molecular testing (RT-PCR): BCR-ABL1 transcript detected

Decision Point 2:
Before initiating therapy, you need to assess the patient's risk stratification according to ELN 2022 guidelines.

Which risk scoring system should you use for newly diagnosed CML in chronic phase?

A) Sokal score
B) Hasford (Euro) score

C) ELTS (EUTOS Long-Term Survival) score
D) IPSS (International Prognostic Scoring System)
Excellent choice, [[REDACTED]]!
You are correct C) ELTS (EUTOS Long-Term Survival) score.

Feedback:

The ELTS score is currently preferred for newly diagnosed CML patients because it better predicts CML-related mortality rather than just response rates.

It considers age, spleen size, platelet count, and percentage of blasts in peripheral blood.

Reference: ELN Recommendations for the Management of CML (ELN 2022).
Phase 2: Induction Therapy Selection and Supportive Care Planning
Patient Case Update:
ELTS score: Intermediate risk

Comorbidities: Mild hypertension (controlled)

Performance Status: ECOG 0

Molecular profile: BCR-ABL1 (p210 isoform), no additional cytogenetic abnormalities

Decision Point 3:
Which tyrosine kinase inhibitor (TKI) would be the most appropriate first-line treatment for this patient?

A) Imatinib
B) Dasatinib
C) Nilotinib
D) Bosutinib
I said:
A
ChatGPT said:
Good try, [[REDACTED]] but A) Imatinib is not the optimal choice here.

Feedback:

Imatinib is still a valid first-line option, especially in low-risk patients or where cost/access is a major issue.

However, for intermediate-risk patients (like Mr. Adam) per ESMO and ELN 2022 guidelines, second-generation TKIs (dasatinib, nilotinib, or bosutinib) are preferred because they achieve deeper and faster molecular responses, improving long-term outcomes.

Also, Mr. Adam has good performance status and no major comorbidities that would contraindicate 2nd-generation TKIs.

Correct choices would have been B) Dasatinib, C) Nilotinib, or D) Bosutinib depending on specific side effect profiles.

Reference: ESMO Guidelines for Chronic Myeloid Leukemia, 2023.
Phase 2: Induction Therapy Selection and Supportive Care Planning
Patient Case Update:
ELTS score: Intermediate risk

Comorbidities: Mild hypertension (controlled)

Performance Status: ECOG 0

Molecular profile: BCR-ABL1 (p210 isoform), no additional cytogenetic abnormalities

Decision Point 4:
Mr. Adam is started on a second-generation TKI.
Which baseline assessment is most critical before starting Nilotinib?

A) Serum lipid panel
B) ECG for QTc interval
C) Chest X-ray
D) PPD test (tuberculosis screen)
I said:
B
ChatGPT said:
Excellent work, [[REDACTED]]!
You are correct B) ECG for QTc interval.

Feedback:

Nilotinib can prolong the QT interval, increasing the risk of life-threatening arrhythmias like torsades de pointes.

Therefore, a baseline ECG is mandatory before initiating therapy, and QTc should be <450 ms ideally.

Also, serum electrolytes (especially potassium and magnesium) should be optimized before starting nilotinib.

Reference:

ELN 2022 and ESMO 2023 guidelines recommend ECG monitoring when using TKIs that can affect cardiac conduction, especially nilotinib and ponatinib.
Phase 2: Induction Therapy Selection and Supportive Care Planning

Patient Case Update:
ELTS score: Intermediate risk
Comorbidities: Mild hypertension (controlled)
Performance Status: ECOG 0
Molecular profile: BCR-ABL1 (p210 isoform), no additional cytogenetic abnormalities

Decision Point 5:
After 3 months of therapy with nilotinib, Mr. Adams BCR-ABL1 transcript level by RT-PCR (International Scale) is reported as 6%.

How would you interpret this result according to ELN 2022 milestones?

A) Optimal response
B) Warning
C) Failure
Well done, [[REDACTED]]!
You are absolutely correct A) Optimal response.

Feedback:

According to ELN 2022 guidelines, a BCR-ABL1 (IS) 10% at 3 months is classified as an optimal response.

Mr. Adams level is 6%, which meets the milestone indicating that therapy is working effectively.

If BCR-ABL1 were >10% at 3 months, it would be considered a warning, prompting reassessment.

Reference: ELN 2022 Recommendations for Response Monitoring in CML.
Phase 3: Post-Remission Therapy, MRD Monitoring, and Long-Term Management
Patient Case Update:
After 12 months of therapy, Mr. Adams BCR-ABL1 transcript by RT-PCR (IS) is 0.08%.

He feels well with no significant side effects.

Decision Point 6:
What is the clinical significance of achieving BCR-ABL1 0.1% at 12 months?

A) Major Molecular Response (MMR)
B) Complete Molecular Response (CMR)
C) Early Molecular Response (EMR)
D) Molecular Relapse
Excellent, [[REDACTED]]!
You are correct A) Major Molecular Response (MMR).

Feedback:

MMR is defined as BCR-ABL1 0.1% on the International Scale.

Achieving MMR by 12 months is a major treatment goal and is associated with a very low risk of disease progression.

CMR (Complete Molecular Response) would require undetectable BCR-ABL1, which is even deeper than MMR.

Reference: ELN 2022 recommendations for response criteria in CML.
Decision Point 7:
Mr. Adam asks:
"Now that Im doing so well, is it possible for me to eventually stop taking nilotinib?"

What is the correct response based on current guidelines?

A) No, TKI therapy must be lifelong for all patients.
B) Yes, but only if he achieves sustained deep molecular response (MR4.5) and meets TFR (Treatment-Free Remission) criteria.
C) Yes, any patient can stop after 1 year of therapy.
D) No, only transplant patients can discontinue TKIs.
Excellent, [[REDACTED]]!
You are correct B) Yes, but only if he achieves sustained deep molecular response (MR4.5) and meets TFR (Treatment-Free Remission) criteria.

Feedback:

TFR is possible in patients who achieve a deep molecular response (MR4 or MR4.5) and maintain it for at least 2 years.

Patients must also have been on TKI therapy for a minimum of about 5 years (varies slightly depending on guidelines).

Careful, frequent molecular monitoring is required after stopping therapy to detect any molecular relapse early.

Reference:

ELN 2022 and ESMO 2023 guidelines emphasize cautious patient selection for TFR.
Decision Point 8:

If a patient fails to achieve MMR at 12 months despite good compliance, what is the recommended next step?

A) Continue the same therapy without change.
B) Switch to another second-generation TKI.
C) Proceed immediately to allogeneic stem cell transplant.
D) Add chemotherapy to the TKI regimen.
Not quite, [[REDACTED]] C) Proceed immediately to allogeneic stem cell transplant is not the recommended first step for a patient who hasn't achieved MMR by 12 months.

Feedback:

In cases where a patient fails to achieve MMR at 12 months, the first-line approach is to switch to a second-generation TKI (like dasatinib, nilotinib, or bosutinib), as they tend to have better efficacy than imatinib.

Stem cell transplant is generally reserved for advanced-phase CML or failure to respond to multiple TKI therapies.

Reference: ELN 2022 guidelines for managing CML patients with suboptimal response.
Decision Point 9:
Mr. Adam asks you about clinical trials. He is interested in enrolling in a clinical trial for a newer drug that targets CML resistance. What is your best recommendation regarding clinical trials for CML?

A) Clinical trials are not recommended for stable CML patients.
B) Consider clinical trials if the patient has refractory disease or resistant mutations.
C) All newly diagnosed CML patients should enroll in a clinical trial.
D) Clinical trials should only be considered in blast phase CML.
Well done, [[REDACTED]]!
You are correct B) Consider clinical trials if the patient has refractory disease or resistant mutations.

Feedback:

Clinical trials are highly recommended for patients who have refractory disease or resistant mutations, as they may provide access to novel therapies or new treatment options.

For stable CML (like Mr. Adam), clinical trials are not a routine option unless there is a specific need (e.g., emerging drug resistance, refractory disease, or interest in testing new therapies).

Reference:

Clinical trial eligibility is often based on disease progression, mutations, or lack of response to current therapy (ELN 2022).
Decision Point 10:
Mr. Adam is now in long-term remission and asks:
"How often should I get monitored for MRD in the future?"

What is the recommended monitoring frequency for MRD in patients in chronic phase CML after achieving a major molecular response (MMR)?

A) Every 3 months for the first 2 years, then every 6 months.
B) Every 6 months, regardless of remission status.
C) Annually, after the first 2 years of treatment.
D) Every 6 months for the first 5 years, then annually.
Excellent choice, [[REDACTED]]!
You are correct A) Every 3 months for the first 2 years, then every 6 months.

Feedback:

For patients in chronic phase CML who have achieved MMR, MRD monitoring is typically done every 3 months for the first 2 years to ensure stable response.

After that, monitoring can be done every 6 months as long as the patient remains in stable remission.

Regular monitoring helps detect early molecular relapse and allows for prompt adjustments to therapy if necessary.

Reference: ELN 2022 and ESMO 2023 guidelines for CML management.

Session ID:
Assessment Domain

Session-102:
Rationale

Session ID:
Instructional Design and Pedagogical

Session-102:
The session's instructional design is outstanding. The case is expertly scaffolded, moving seamlessly from initial diagnosis and risk stratification to first-line therapy selection, response monitoring, and long-term management questions like Treatment-Free Remission (TFR) and handling suboptimal response. The quality of the embedded feedback is a major strength; each rationale is clear, evidence-based, and correctly cites current clinical guidelines (e.g., ELN, ESMO, WHO). The instructions and case updates are exceptionally clear. The only minor area for improvement is the absence of explicitly stated learning objectives at the beginning of the session, though the educational goals are strongly implied by the logical structure.

Session ID:
Clinical Authenticity and Content Fidelity

Session-102:
The clinical content is largely accurate and authentic, but with one notable area for improvement. Plausibility & Domain Specificity: The clinical narrative is indistinguishable from an authentic case of Chronic Myeloid Leukemia (CML), with realistic patient data and progression. The session demonstrates perfect domain specificity, keeping all diagnostics, scoring systems (ELTS), and therapies (TKIs) strictly within the CML domain without any entanglement from other hematologic malignancies. Guideline Alignment & Pharmacotherapeutic Accuracy: The session is strongly aligned with guidelines for the most part. However, in Decision Point 8 ("If a patient fails to achieve MMR at 12 months..."), the recommendation to "Switch to another second-generation TKI" is incomplete. While switching the TKI is a valid step, current guidelines emphasize first performing BCR-ABL1 kinase domain mutation analysis to check for resistance mutations that would guide the selection of the subsequent TKI. Omitting this critical diagnostic step makes the therapeutic recommendation acceptable, but not the optimal, first-line choice in managing TKI failure. This lowers the score for Pharmacotherapeutic Accuracy.

Session ID:
Modeled Clinical Reasoning

Session-102:
The simulation is a superb model of expert clinical reasoning. Each decision point presents a clearly framed and highly relevant clinical problem. The multiple-choice options provide an excellent range of plausible choices, including effective distractors that test for nuanced understanding (e.g., including the IPSS score, which is for MDS, not CML). The overall strategic flowDiagnose Risk Stratify Treat Monitordemonstrates flawless prioritization. Even in Decision Point 8, where the pharmacotherapeutic detail was slightly lacking, the strategic prioritization (switch TKI before considering transplant) was correct. The clinical outcomes described (e.g., BCR-ABL1 levels at 3 and 12 months) are realistic and instructionally valuable.

Session-103

Session ID:
LLM

Session-103:
ChatGPT (GPT 4.o)

Session ID:
Session Transcript

Session-103:

Patient Case Phase 1 Overview:
A 42-year-old male presents with fatigue, early satiety, and unintentional weight loss over the past 2 months. He reports no significant past medical history. On physical exam, spleen is palpable 6 cm below the costal margin. CBC reveals:
WBC: 132,000/L
Hb: 10.2 g/dL
Platelets: 610,000/L
Peripheral smear: marked leukocytosis with left shift, basophilia, and eosinophilia.

Question 1:
What is the next best step in confirming the diagnosis of chronic myeloid leukemia (CML)?

A. Bone marrow biopsy with flow cytometry
B. JAK2 mutation analysis
C. BCR-ABL1 reverse transcriptase PCR (RT-PCR)
D. Serum LDH and uric acid levels

: C. BCR-ABL1 reverse transcriptase PCR (RT-PCR)
. ESMO 2023
BCR-ABL1 RT-PCR 75% IS (International Scale).
WHO 2022

A. Chronic phase CML
B. Accelerated phase CML
C. Blast crisis CML
D. Essential thrombocythemia

:

C: Blast crisis CML
.

Blast crisis :
20% (blasts)

(extramedullary blast proliferation)

75% BCR-ABL1 (IS) Blast crisis. .

: A. Chronic phase CML
WHO 2022 ELN 2022 BCR-ABL1 .

ELN 2022

A. Sokal score
B. ELN risk stratification model
C. IPSS-R
D. NCCN cytogenetic model
:

C: IPSS-R
.
IPSS-R (Revised International Prognostic Scoring System) Myelodysplastic Syndromes (MDS) CML.

: B. ELN risk stratification model
ELN 2022 (European LeukemiaNet) CML ( ) ELTS (EUTOS Long-Term Survival Score) .

:
: .
: .
: MDS CML.

Phase 2: Induction Therapy & Supportive Care

Learning Objectives:
Select first-line treatment based on ELN risk category and patient-specific factors.
Recognize and manage treatment-related complications.
Implement comprehensive supportive care (TLS, antimicrobials, transfusion).

Patient Case Phase 2 Overview:
CML :

:
: 42

ECOG: 0
ELTS Score:

Question 4:
ESMO 2023 ELN 2022

A. Imatinib 400 mg PO daily
B. Dasatinib 100 mg PO daily
C. Hydroxyurea followed by allogeneic HSCT
D. Omacetaxine mepesuccinate
:

A: Imatinib 400 mg PO daily
.

:
Imatinib (TKI) CML.
ESMO 2023 ELN 2022 (Dasatinib Nilotinib) .

:
B. Dasatinib 100 mg PO daily
BCR-ABL1.

:
: .
: TKI ELN .
: TKIs .

3 Dasatinib . .

A.
B. Dasatinib
C. Ponatinib
D. Dasatinib

Great switching to English for clinical reasoning clarity.

Answer: B. Temporarily discontinue Dasatinib, manage the effusion, and reassess.
Correct.

Which of the following supportive care measures is MOST appropriate to initiate before starting TKI therapy in this patient with WBC 132,000/L?

A. Empiric IV antibiotics
B. Allopurinol and IV hydration
C. Platelet transfusion
D. G-CSF (filgrastim)

Choose: A, B, C, or D.

Answer C: Platelet transfusion
Incorrect.

Clinical Reasoning:

This patient has thrombocytosis (platelets = 610,000/L), not thrombocytopenia. Transfusion is not indicated unless theres active bleeding or platelets are <10,000/L (per NCCN supportive care guidelines). In fact, transfusing here may increase risk of thrombosis.

Correct Answer: B. Allopurinol and IV hydration

Given the very high WBC (132,000/L), the patient is at risk for tumor lysis syndrome (TLS) upon TKI initiation. TLS can cause hyperuricemia, hyperkalemia, hyperphosphatemia, and renal failure.
Allopurinol reduces uric acid production
IV fluids help flush cellular breakdown products

This is standard TLS prophylaxis in high-burden hematologic malignancies per ESMO supportive care guidelines.

Why not the others?
A: Empiric antibiotics are not indicated unless febrile neutropenia or infection signs are present.
D: G-CSF is contraindicated in CML as it may worsen leukocytosis.

Evaluation:
Improvement area: Consider physiologic indication before supportive interventions.
Learning Tip: TLS prophylaxis is essential when WBC >100,000 or LDH is elevated.
Question 7:
After 1 month on Dasatinib, the patients BCR-ABL1 level on IS drops to 25%. How do you interpret this molecular response per ELN 2022?

A. Optimal response
B. Warning
C. Failure
D. Not applicable until 3 months
Answer D: Not applicable until 3 months
Correct.

Clinical Justification:

Per ELN 2022 molecular monitoring guidelines, the first key milestone for evaluating BCR-ABL1 transcript level is at 3 months, not before. While molecular trends can be monitored earlier for information, formal interpretation (optimal, warning, failure) begins at:
3 months: Goal is BCR-ABL1 10%
6 months: Goal is 1%
12 months: Goal is 0.1%

A result of 25% at 1 month may look suboptimal, but its too early to categorize the response formally.

Evaluation:
Excellent awareness of ELN timeline thresholds.
Shows understanding of when to act vs. when to wait in molecular monitoring.

Learning Objectives:
Interpret molecular response milestones per ELN 2022
Determine appropriate follow-up and therapy modification strategies
Understand when treatment discontinuation or transplant evaluation is appropriate
Identify clinical trial and long-term monitoring considerations

Patient Case Phase 3 Overview:
You are following up with the same 42-year-old male patient. After 3 months on Dasatinib, his labs show:
BCR-ABL1 (IS): 6%
CBC: WNL
No pleural effusion recurrence
Performance status remains ECOG 0

Question 8:
How would you interpret this 3-month BCR-ABL1 result (6%) based on ELN 2022 guidelines?

A. Optimal response
B. Warning
C. Failure
D. Incomplete hematologic response
Answer B: Warning
Incorrect.

Correct Answer: A. Optimal response

Explanation:
According to ELN 2022 guidelines, the 3-month molecular milestone for CML management is:
Optimal: BCR-ABL1 (IS) 10%
Warning: >10% to 1% by 6 months
Failure: >10% after 6 months

This patients BCR-ABL1 = 6% at 3 months falls within the optimal range, meaning he is responding as expected to Dasatinib.

Why not B?
Warning applies only when BCR-ABL1 >10% at 3 months and thats not the case here.

Clinical Implication:

Continue current TKI (Dasatinib)
Continue molecular monitoring every 3 months until major molecular response (MMR: 0.1%) is achieved

Question 9:
At 18 months, the patient achieves MR^4 (BCR-ABL1 0.01%) and asks if he can discontinue TKI therapy. What is the most appropriate next step?

A. Immediately stop TKI and monitor monthly
B. Switch to Imatinib for TKI de-escalation
C. Assess treatment-free remission (TFR) eligibility criteria
D. Continue Dasatinib indefinitely regardless of molecular response
Correct Answer: C. Assess treatment-free remission (TFR) eligibility criteria

Clinical Justification:

Treatment-Free Remission (TFR) is an emerging goal in chronic-phase CML for select patients. According to ESMO 2023 and ELN 2022, TFR is only appropriate after confirming all of the following:
On TKI therapy for 5 years
Achieved deep molecular response (MR^4 or MR^4.5)
Maintained MR^4 or better for 2 years
Have access to high-sensitivity molecular monitoring (monthly for first 6 months post-discontinuation)

This patient has only been on TKI for 18 months, so hes not yet eligible. No recommendation exists to switch to Imatinib to facilitate stopping this de-escalation strategy is not validated outside of select trials (e.g., DESTINY study).

Why not the others?
A: Too early to stop therapy would risk molecular relapse.
B: Imatinib is less potent, and de-escalation isnt standard.
D: While indefinite therapy is standard for most, patients should be evaluated for TFR if eligible.

Two years later, the patient develops new BCR-ABL1 mutation: T315I while on Dasatinib. CBC shows rising WBCs. What is the most appropriate next treatment?

A. Continue Dasatinib at higher dose
B. Switch to Ponatinib
C. Add interferon-alpha to Dasatinib
D. Start hydroxyurea and monitor
Answer B: Switch to Ponatinib
Correct.

Clinical Justification:

The T315I mutation in BCR-ABL1 is known as the gatekeeper mutation it confers resistance to all TKIs except Ponatinib. According to ELN 2022 and ESMO 2023, the standard approach is:
Switch to Ponatinib, a third-generation TKI specifically designed to overcome T315I resistance
Monitor for vascular toxicity, as Ponatinib carries arterial thrombotic risk dose adjustment and cardiovascular risk assessment are essential

Why not the others?
A: Dose escalation of Dasatinib wont overcome T315I
C: Interferon-alpha may have limited activity and is not standard in mutation-driven resistance
D: Hydroxyurea is used temporarily for cytoreduction, not as definitive therapy for resistant mutations

---

Session ID:
Assessment Domain

Session-103:
Rationale

Session ID:
Instructional Design and Pedagogical

Session-103:
The session's instructional design is of very high quality. The case is expertly scaffolded, moving logically from initial diagnosis and risk stratification (Questions 1-3) through first-line therapy selection (Question 4), side-effect management (Question 5), supportive care (Question 6), and complex long-term management, including monitoring milestones (Questions 7-8), treatment-free remission considerations (Question 9), and resistance (Question 10). The feedback is a major strength; rationales are detailed, explicitly cite current guidelines (e.g., ELN, ESMO), and correctly differentiate between plausible but suboptimal choices and the best therapeutic option. Instructions are consistently clear. The only minor weakness is that learning objectives are not stated at the outset, but rather introduced by phase, slightly diminishing their role as an upfront guide for the learner.

Session ID:
Clinical Authenticity and Content Fidelity

Session-103:
The clinical and pharmacotherapeutic content of this session is exceptionally accurate and authentic. Plausibility & Guideline Alignment: The clinical narrative is indistinguishable from an authentic patient case, presenting a classic CML progression. All diagnostic steps, therapeutic decisions, and monitoring milestones are perfectly aligned with current, evidence-based guidelines from ELN, ESMO, and NCCN.
Pharmacotherapeutic Accuracy: The therapeutic choices modeled are optimal, not merely acceptable. The simulation correctly recommends a second-generation TKI (Dasatinib) for an intermediate-risk patient and later correctly identifies Ponatinib as the only effective agent for the T315I gatekeeper mutation. Domain Specificity: The session demonstrates excellent domain specificity. It is entirely free of domain entanglement. In fact, it actively teaches against this error in Question 3 by requiring the user to distinguish between the CML-specific ELN risk model and the MDS-specific IPSS-R score. The session's content fidelity is outstanding.

Session ID:
Modeled Clinical Reasoning

Session-103:
The simulation expertly models a sound clinical reasoning process. Each stage of the case presents a well-framed clinical problem with the necessary data to make an informed decision. The multiple-choice options provide an excellent breadth of plausible and relevant alternatives, including high-quality distractors that effectively test nuanced understanding (e.g., including Imatinib as a first-line option or IPSS-R as a scoring tool). The narrative demonstrates appropriate prioritization throughout, from initial diagnostics to managing treatment resistance. The clinical outcomes described for each decision are realistic, specific, and highly instructive, reinforcing the session's key learning objectives.
